# Cobalt‐Catalyzed Radical Ligand Transfer (RLT) Enables *Remote*‐Markovnikov Hydroamination of Alkenes

**DOI:** 10.1002/anie.8746225

**Published:** 2026-06-23

**Authors:** Arman Khosravi, Ho Martin Yuen, Seth Fremin, Nuwan A. Pannilawithana, Nol Supasueb, Yu Zhang, Peng Liu, Ming‐Yu Ngai

**Affiliations:** ^1^ James Tarpo Jr. and Margaret Tarpo Department of Chemistry Purdue University West Lafayette Indiana USA; ^2^ Department of Chemistry University of Pittsburgh Pittsburgh Pennsylvania USA

**Keywords:** 1,2‐radical acyloxy migration, hydroamination, metal‐hydride hydrogen atom transfer, radical ligand transfer, *remote*‐Markovnikov

## Abstract

Amino alcohols are prevalent in pharmaceuticals, agrochemicals, and functional materials, yet regioselective remote C─N bond formation from simple alkenes remains limited. We report a *remote*‐Markovnikov formal 1,3‐hydroamination of allyl carboxylates enabled by cooperative photoredox, cobalt, and Brønsted acid catalysis. The reaction proceeds via metal–hydride hydrogen atom transfer (MHAT) followed by 1,2‐radical acyloxy migration (1,2‐RAM) to generate a tertiary carbon‐centered radical at a site remote from the original alkene, affording protected tertiary alkyl amino alcohols across a broad substrate scope, with secondary variants also accessible. Experimental and computational studies support a C─N bond‐forming step best described as radical ligand transfer (RLT) at a Co(III)─N intermediate, rather than Co(IV)/S_N_2 or carbocationic pathways. This rare nitrogen‐transfer manifold accounts for the observed regioselectivity and catalyst‐influenced stereochemical outcomes. By enabling migration‐controlled 1,3‐hydroamination, this strategy complements conventional 1,2‐hydroamination and expands accessible amino alcohol chemical space.

## Graphical abstract

A cobalt‐catalyzed remote‐Markovnikov 1,3‐hydroamination of allyl carboxylates proceeds through MHAT, 1,2‐radical acyloxy migration (1,2‐RAM), and radical ligand transfer (RLT) from a Co‐N intermediate, providing protected amino alcohols.

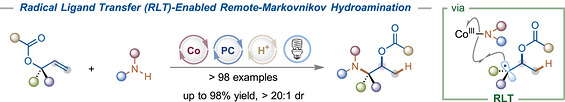

## 1 Introduction

**FIGURE 1:**
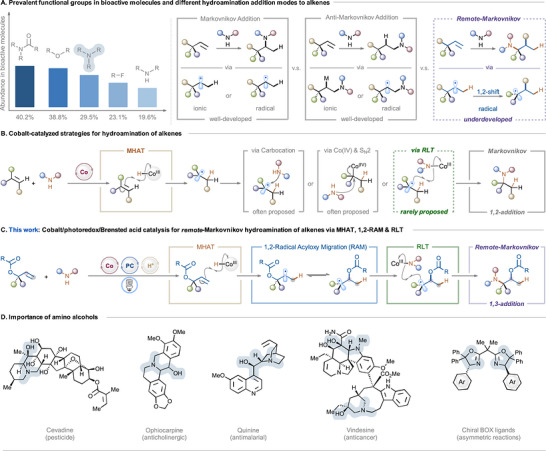
Hydroamination of alkenes and importance of amino alcohols.

Nitrogen‐containing functional groups are ubiquitous across pharmaceuticals [1, 2, 3, 4, 5, 6, 7], agrochemicals [8, 9], and functional materials [10, 11, 12, 13, 14, 15]. Analyses of bioactive molecules consistently identify alkyl amines among the most frequently occurring functionalities [2, 3, 16], highlighting the ongoing demand for efficient and selective strategies to construct C─N bonds while maintaining broad functional‐group compatibility and structural flexibility (Figure 1A). Alkene hydroamination represents one of the most direct approaches to alkyl amines [17, 18], although achieving broad scope and high regioselectivity remains challenging. Brønsted acid catalysis enables Markovnikov‐selective addition through cationic intermediates [19, 20, 21, 22, 23] but protonation can reduce amine nucleophilicity [17]. Alkali‐metal‐mediated methods rely on strongly basic amide nucleophiles and are most effective with electronically activated alkenes [24, 25]. Transition‐metal and lanthanide catalysis have expanded the scope of hydroamination by enabling milder conditions [17, 26, 27, 28, 29, 30], anti‐Markovnikov selectivity [18, 31, 32, 33, 34, 35], and asymmetric variants [36, 37, 38, 39, 40, 41, 42, 43]. Despite these advances, most established methods proceed through direct 1,2‐addition, and migration‐enabled or remote C─N bond formation remains less explored (Figure 1A) [44, 45, 46].

Cobalt‐catalyzed hydrofunctionalization via metal‐hydride hydrogen‐atom transfer (MHAT) has emerged as a powerful alternative paradigm for alkene functionalization [47, 48, 49, 50, 51, 52, 53, 54, 55, 56, 57, 58, 59, 60, 61, 62, 63]. In this manifold, MHAT [64, 65, 66, 67, 68] converts an alkene into a carbon‐centered radical under mild conditions, enabling bond constructions that are difficult to access through ionic pathways. Despite increasing application of MHAT to hydroamination [53, 69, 70, 71, 72, 73], the nature of the C─N bond‐forming step remains mechanistically unsettled. Prevailing proposals invoke either ionization to a carbocation and subsequent capture [53] or the formation of a Co(IV)–alkyl intermediate followed by nucleophilic substitution (Figure 1B) [69, 70, 71, 72, 73, 74, 75, 76, 77, 78]. While formation of Co(IV)–alkyl species at primary and secondary carbon centers is well established, their relevance to sterically congested tertiary radicals is far less certain.

Here we report a *remote*‐Markovnikov‐selective formal 1,3‐hydroamination of allyl carboxylates enabled by photoredox, cobalt, and Brønsted acid catalysis. MHAT‐initiated radical generation followed by 1,2‐radical acyloxy migration (1,2‐RAM) furnishes a tertiary radical at a site remote from the original alkene (Figure 1C). Supported by experimental and computational evidence, we propose that C─N bond formation proceeds through radical ligand transfer (RLT) [79] at a Co(III)─N intermediate. This pathway represents a rare and previously weakly supported mode of nitrogen transfer in Co‐Salen‐catalyzed MHAT hydroamination and accounts for both the regioselectivity and stereochemical outcomes observed. Notably, this process directly furnishes tertiary alkyl amines, a class of nitrogen motifs that remains challenging to access selectively through catalytic hydroamination, particularly at sterically congested centers [80, 81, 82, 83, 84]. From a synthetic perspective, this migration‐enabled hydroamination provides access to amino alcohol architectures complementary to those obtained through conventional 1,2‐hydroamination, expanding accessible amino alcohol chemical space useful for medicinal and functional molecule design (Figure 1D) [85, 86, 87, 88, 89, 90, 91, 92, 93].

We commenced our studies using 2‐phenylbut‐3‐en‐2‐yl benzoate (**1a**) and *N*‐methylaniline (**2a**) as a model substrate (Table 1). A systematic evaluation of reaction parameters revealed that combining **1a** (1.00 equiv) and **2a** (1.50 equiv), **HX‐1** (10.0 mol%) with **Co‐1** (2.00 mol%) and Ir(ppy)_3_ (1.00 mol%) in THF (0.100 M), under blue LED irradiation at 40 °C for 18 h, afforded the desired product **3a** in 84% yield (entry 1). No product formation was observed without cobalt (entry 2). Substituting **Co‐2** gave only 41% yield (entry 3), while **Co‐3** afforded 63% (entry 4), suggests that ligand structure can significantly influence the efficiency of cobalt‐mediated steps in the catalytic cycle (e.g., MHAT and RLT). Ir(ppy)_3_ was found to be irreplaceable: without it (entry 5), or when substituted with PTH or 4CzIPN (entries 6–7), no product was observed, signifying that its distinct redox and excited‐state properties are required. In the absence of **HX‐1**, the reaction did not proceed, highlighting its essential function in the formation of Co^III^─H intermediate (entry 8). Additionally, substituting **HX‐1** with other **HX** surrogates (entries 9–11) suggested the essential roles of the counter ion for optimal reactivity [94]. Control experiments confirmed the photochemical nature of the process, as no conversion was observed in the absence of light (entry 12). While heating was not strictly required, omitting external heating resulted in a diminished yield of 50% (entry 13). Finally, exposure to air completely inhibited the reaction (entry 14), consistent with the involvement of oxygen‐sensitive catalytic intermediates.

**TABLE 1:** Selected optimization experiments^a^.

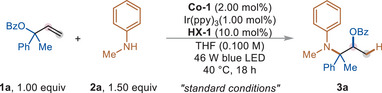		
Entry	Deviation from the standard conditions	Yield%
1	none	84
2	without **Co‐1**	<2
3	**Co‐2** instead of **Co‐1**	41
4	**Co‐3** instead of **Co‐1**	63
5	without Ir(ppy)_3_	<2
6	PTH instead of Ir(ppy)_3_	<2
7	4CzIPN instead of Ir(ppy)_3_	<2
8	without **HX‐1**	<2
9	**HX‐2** instead of **HX‐1**	75
10	**HX‐3** instead of **HX‐1**	73
11	**HX‐4** instead of **HX‐1**	77
12	without light	<2
13	without heat	50
14	air	<2
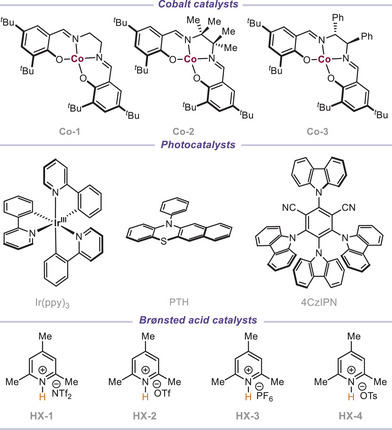		

^a^ See Supporting Information (SI) for experimental details. Reaction yields were determined by ^1^H‐NMR using CH_2_Br_2_ as an internal standard. Bz, benzoyl.

With optimized conditions in hand, the scope of anilines was evaluated (Table 2). A wide range of anilines underwent efficient migration‐enabled functionalization to afford β‐acyloxy tertiary alkyl amines. *N*‐methylaniline delivered **3a** in 84% yield. Ortho‐, meta‐, and para‐methyl‐substituted anilines (**3b–3d**) were readily converted (63%–84% yield). Para‐substituted anilines bearing electron‐donating and electron‐withdrawing groups, including tert‐butyl (**3e**), methoxy (**3f**), halogens (**3g–3i**), trifluoromethyl (**3j**), trifluoromethoxy (**3k**), and pentafluoroaniline (**3l**), all furnished the corresponding products. Broad functional‐group tolerance was observed, accommodating nitrile (**3m**), ester (**3n**), ketone (**3o**), phenyl (**3p**), biphenyl (**3q**), dibenzothiophene (**3r**), dibenzofuran (**3s**), and boronic ester (**3t**) substituents. Disubstituted anilines, including 2,4‐dimethyl (**3u**) and 3,5‐dimethyl (**3v**) derivatives, were effective, as were other highly substituted systems (**3w–3ad**). Naphthyl (**3ae**) and heteroaryl anilines (**3af**, **3ag**) participated efficiently. Beyond *N*‐methylanilines, tetrahydroquinoline (**3ah**), *N*‐cyclohexylaniline (**3ai**), *N*‐cyclobutylaniline (**3aj**), diphenylamines (**3ak**, **3al**), and dihydrodibenzazepine (**3am**) afforded the corresponding aminated products in moderate to excellent yields. Overall, anilines spanning a broad electronic and steric range furnished the corresponding β‐acyloxy alkyl amines in generally good to excellent yields (typically 60%–90%). Diastereoselectivity varied depending on substrate structure, ranging from modest to high levels (up to >20:1), consistent with substrate‐dependence in the radical C─N bond‐forming step. Gram‐scale synthesis of **3k** proceeded smoothly, delivering the product in 78% isolated yield.

**TABLE 2:** 1,3‐Hydroamination of allyl carboxylates with anilines via photoredox/cobalt/Brønsted acid catalysis^a^.

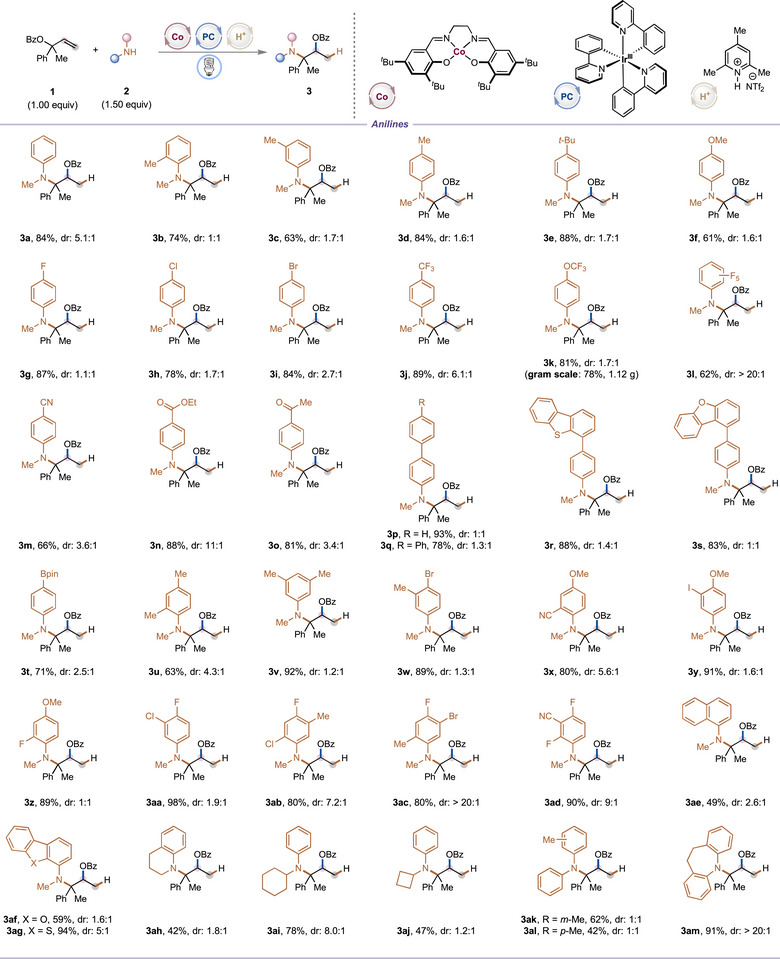

^a^ See Supporting Information for experimental details. Standard reaction conditions: **1** (0.200 mmol, 1.00 equiv.), **2** (0.300 mmol, 1.50 equiv.), **Co‐1** (2.00 mol%), Ir(ppy)_3_ (1.00 mol%), **HX‐1** (10.0 mol%), THF (0.100 M), blue LED, 40 °C, 18 h.

The scope of allyl carboxylates was next examined (Table 3). Aryl‐substituted substrates bearing electron‐withdrawing groups at the ortho‐, meta‐, or para‐positions (**3an–3ar**) underwent efficient 1,3‐hydroamination, as did biphenyl (**3as**) and ester‐substituted aryl (**3at**) derivatives. Heteroaromatic ester‐substituted aryl allyl carboxylates (**3au–3aw**) and a meta‐methoxy‐substituted substrate (**3ax**) were also competent. Secondary allyl carboxylates required elevated temperatures but afforded the desired products in synthetically useful yields (**3ay–3aae**). Notably, modification of the migrating group to a 4‐methoxybenzoate (**3aaf**, **3aag**) improved reaction efficiency. Evaluation of the migrating group revealed that both electron‐rich and electron‐deficient benzoates (**3aah–3aaq**), as well as naphthyl (**3aar**), 4‐methoxybenzoate (**3aas**), 3‐trifluoromethylbenzoate (**3aat**), cyclohexanecarboxylate (**3aau**), and thiophenyl carboxylate (**3aav**) substituents were viable. Across aryl and aliphatic allyl carboxylates, the reaction delivered the desired products in moderate to excellent yields (50%–96%). Additional substrate examples are provided in Figure S2. Single‐crystal x‐ray diffraction of **3aag** unambiguously confirmed the product structure and its relative stereochemistry.

**TABLE 3:** 1,3‐Hydroamination of allyl carboxylates with anilines via photoredox/cobalt/Brønsted acid catalysis^a^.

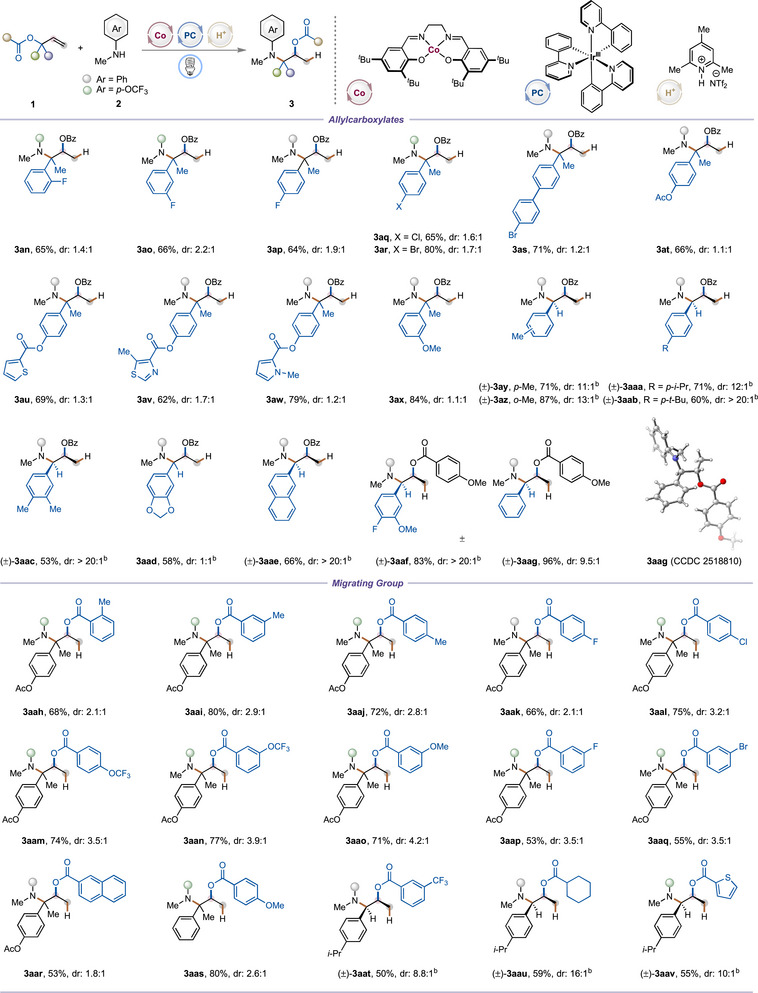

^a^ See Supporting Information for experimental details. Standard reaction conditions: **1** (0.200 mmol, 1.00 equiv.), **2** (0.300 mmol, 1.50 equiv.), **Co‐1** (2.00 mol%), Ir(ppy)_3_ (1.00 mol%), **HX‐1** (10.0 mol%), THF (0.100 M), blue LED, 40 °C, 18 h.

^b^ 80 °C instead of 40 °C.

To demonstrate practicality, complex substrates were evaluated (Table 4). Allyl carboxylates or anilines derived from adapalene (**4a**), probenecid (**4b**), ciprofibrate (**4c**), ibuprofen (**4d**), gemfibrozil (**4e**), D‐valine (**4f**), febuxostat (**4g**), and oxaprozin (**4h**) underwent selective 1,3‐hydroamination. Natural‐product‐derived substrates bearing vitamin E (**4i**), cholestanol (**4j**), tigogenin (**4k**), and estrone (**4l**) motifs also furnished the corresponding products. Late‐stage functionalization of complex molecules proceeded with good efficiency, highlighting the functional‐group tolerance and synthetic practicality of the method.

**TABLE 4:** 1,3‐Hydroamination of complex molecules via photoredox/cobalt/Brønsted acid catalysis^a^.

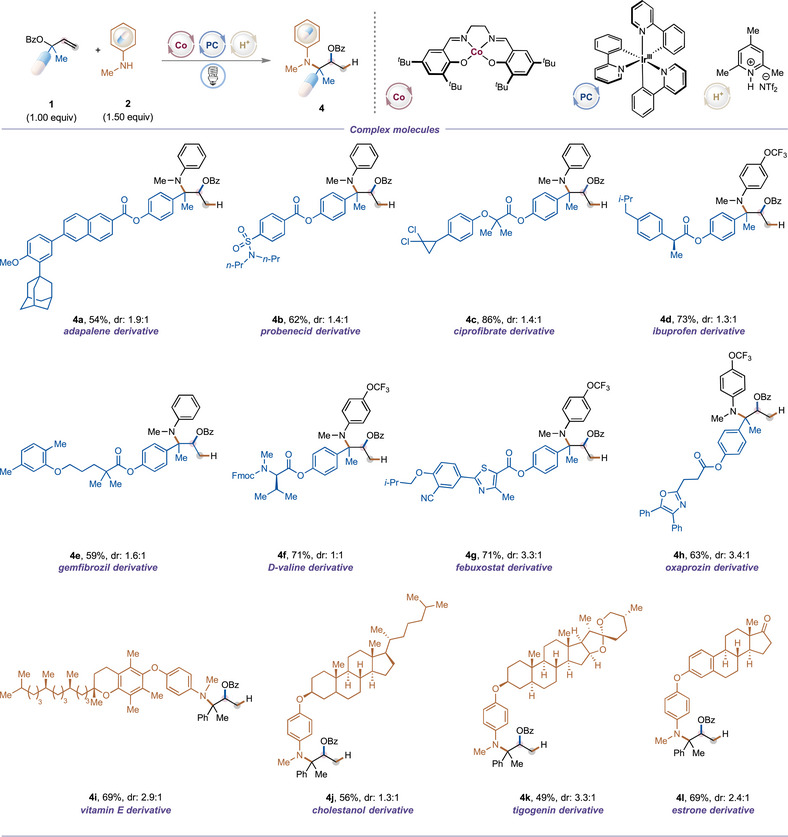

^a^ See Supporting Information for experimental details. Standard reaction conditions: **1** (0.200 mmol, 1.00 equiv.), **2** (0.300 mmol, 1.50 equiv.), **Co‐1** (2.00 mol%), Ir(ppy)_3_ (1.00 mol%), **HX‐1** (10.0 mol%), THF (0.100 M), blue LED, 40 °C, 18 h.

**FIGURE 2:**
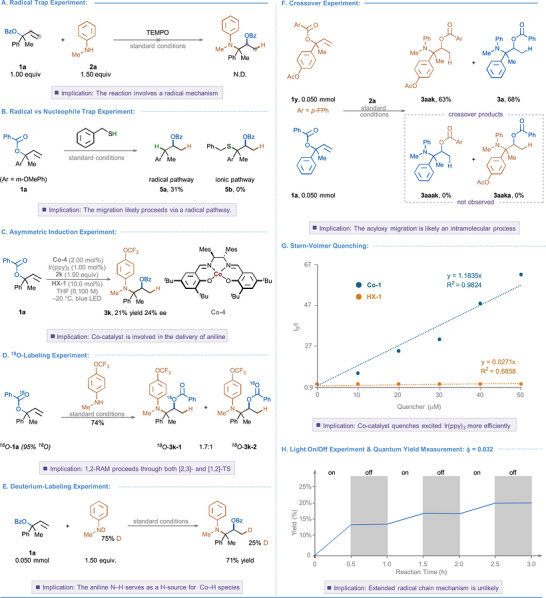
Mechanistic studies, see Supporting Information for details.

Mechanistic studies were performed to gain insight into the reaction mechanism (Figure 2). Addition of TEMPO to reaction mixture completely inhibited the product formation implying a radical mechanism (Figure 1A). To further probe the nature of 1,2‐acyloxy migration, benzyl thiol, a known radical H‐atom donor and nucleophile [95], was employed. The formation of reduced **5a**, rather than thioether **5b**, supported a radical‐mediated 1,2‐acyloxy migration (Figure 2B). Using a enantioenriched catalyst at −20°C, product **3k** was obtained with 24% ee, consistent with involvement of the cobalt catalyst in the C─N bond‐forming step and its ability to impart stereochemical bias (Figure 2C and Figure S4). To probe the topology of the migration, ^18^O‐labeling experiments with ^18^O‐**1a** generated both ^18^O**‐3k‐1** and ^18^O**‐3k‐2** in a 1.7:1 ratio (Figure 2D), indicating that 1,2‐radical acyloxy migration proceeds through both [2,3]‐ and [1,2]‐transition states, with the former favored. Measured O^18^/O^16^ ratio reflects the rearrangement pathway rather than isotopic scrambling (see Supporting Information for more details). Deuterium‐labeling experiments resulted in 25% deuterium incorporation at the terminal position of the product (Figure 2E), indicating that aniline N─H bonds contribute to formation of the Co─H species in the catalytic process. To distinguish between intra‐ and intermolecular acyloxy migration pathways, crossover experiments using **1y** and **1a** were conducted under optimized conditions (Figure 2F). The exclusive formation of non‐crossover products, **3aak** and **3a**, supports an intramolecular acyloxy migration mechanism. Stern–Volmer quenching studies revealed that the excited photocatalyst Ir(ppy)_3_* is quenched more efficiently by **Co‐1** than by **HX‐1**, **1a** or *N*‐methylaniline (Figure 2G), consistent with initial reduction of Co(II) to Co(I) by photoexcited Ir(ppy)_3_*, followed by protonation of Co(I) by **HX‐1** to generate Co(III)–H species [96]. Finally, both light on/off experiments and quantum yield measurements indicated that an extended radical chain mechanism is unlikely under standard reaction conditions (Figure 2H) [97].

**FIGURE 3:**
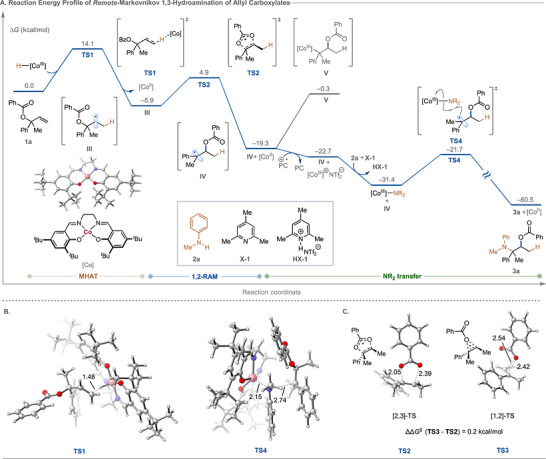
(A). Computed reaction energy profile for the photoredox/cobalt/Brønsted acid–catalyzed formal 1,3‐hydroamination of allyl carboxylate **1a** (M06/def2‐TZVP/SMD(THF)//B3LYP‐D3/6‐31G(d)−SDD). (B). 3D transition‐state structures for MHAT and NR_2_ transfer generated using CYLview [100]. (C). Activation barriers for the [2,3]‐ and [1,2]‐RAM pathways, with Gibbs free energies referenced to secondary radical **III** (M06‐2X/6‐311+G(d,p)/SMD(THF)//B3LYP‐D3/6–31G(d)).

DFT calculations provide energetic support for a radical relay pathway consistent with the experimental observations (Figure 3A) [98, 99]. MHAT from [Co^III^]–H to **1b** proceeds via **TS1** with Markovnikov selectivity (Δ*G* ^‡^ = 14.1 kcal/mol), generating secondary radical **III** and [Co^II^] (Figure 3B). Radical **III** undergoes 1,2‐RAM to form a more stable tertiary radical **IV** via a [2,3]‐shift ([2,3]‐**TS2**), favored over the [1,2]‐pathway ([1,2]‐**TS3**) by 0.2 kcal/mol (Figure 3, see also Table S9), consistent with ^18^O‐labeling studies. Oxidation of [Co^II^] by [Ir^IV^(ppy)_3_]^•+^(NTf_2_^−^) followed by coordination of aniline and collidine (**X‐1**) assisted deprotonation to form [Co^III^]–NR_2_ is exergonic (Δ*G* = –12.1 kcal/mol). Competing pathways involving solvent cage collapse of radical pair [68] to **V** (19.0 kcal/mol) is computed to be less favorable under these conditions. Final NR_2_ transfer from [Co^III^]–NR_2_ to **IV** proceeds through a RLT process (**TS4**, Δ*G* ^‡^ = 9.7 kcal/mol), affording the desired coupling product **3a** in a highly exergonic step.

**FIGURE 4:**
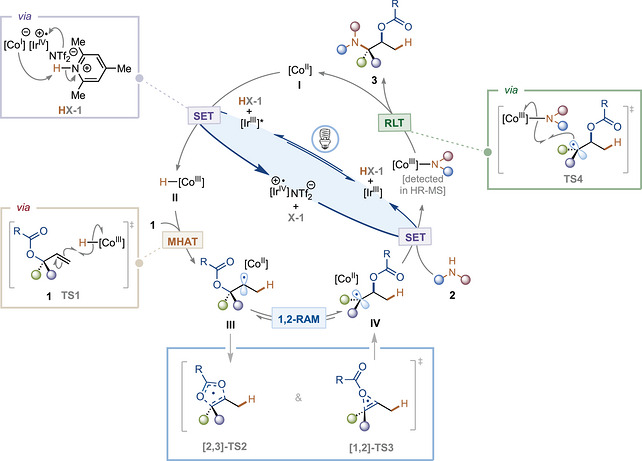
Proposed reaction mechanism of the photoredox/cobalt/Brønsted acid‐catalyzed 1,3‐hydroamination of allyl carboxylate.

A proposed catalytic cycle consistent with experimental observations and literature precedents [96, 101, 102] is shown in Figure 4. Upon the irradiation by blue light, the excited‐state photocatalyst [Ir^III^]* reduces [Co^II^] to [Co^I^], which is protonated by **HX‐1** to furnish [Co^III^]–H along with [Ir^IV^]NTf_2_ and collidine (**X‐1**) [96]. The resulting cobalt hydride engages allyl carboxylate **1** via MHAT through **TS1**, affording the β‐acyloxyalkyl radical **III** and [Co^II^] species. Radical **III** subsequently undergoes 1,2‐RAM through both **TS2** and **TS3** to generate the more stable radical **IV** [99]. Concurrently, oxidation of [Co^II^] by [Ir^IV^]NTf_2_ forms [Co^III^] intermediate, closing the photoredox cycle. Coordination of aniline to the resulting [Co^III^] catalyst followed deprotonation regenerates **HX‐1** and delivers the [Co^III^]–NR_2_ species, which was detected in HR‐MS (Figure S22). The key C─N bond‐forming event is proposed to proceed via radical ligand transfer, wherein radical **IV** undergoes an RLT reaction with the [Co^III^]–NR_2_ species through **TS4 [** 79, 103, 104]. This step furnishes the remote 1,3‐hydroamination product **3** and regenerates [Co^II^] catalyst, completing the catalytic cycle.

In summary, we establish a migration‐enabled strategy for remote C─N bond formation that expands both the reactivity and mechanistic understanding of cobalt‐salen‐catalyzed MHAT hydroamination. By merging MHAT with 1,2‐RAM, the method enables functionalization at positions inaccessible to conventional 1,2‐hydroamination, affording protected β‐acyloxy alkyl amines with broad functional‐group tolerance. Combined experimental and computational studies support a C─N bond‐forming step proceeding via RLT at a Co(III)─N intermediate, in contrast to commonly invoked Co(IV)/S_N_2 or carbocationic pathways. More broadly, this work highlights the viability of RLT processes at cobalt─N bonds in MHAT catalysis and provides a conceptual foundation for future radical hydrofunctionalization design.

## Author Contributions

**Arman Khosravi**: conceptualization, investigation, writing – original draft, methodology, validation, visualization, writing – review and editing, data curation, supervision. **Ho Martin Yuen**: methodology, writing – review and editing, data curation, visualization, investigation, formal analysis, software. **Seth Fremin**: investigation, data curation, methodology, writing – review and editing, software. **Nuwan A. Pannilawithana**: methodology, data curation, investigation, validation, visualization. **Nol Supasueb**: methodology, investigation, formal analysis, data curation, visualization. **Yu Zhang**: investigation, methodology, visualization, formal analysis, software. **Peng Liu**: investigation, methodology, software, data curation, project administration, writing – original draft, validation, writing – review and editing, resources, supervision, funding acquisition. **Ming‐Yu Ngai**: validation, methodology, conceptualization, investigation, funding acquisition, writing – original draft, data curation, supervision, resources, project administration, writing – review and editing.

## Conflicts of Interest

The authors declare no conflicts of interest.

## Supporting information




**Supporting File**: anie73134‐sup‐0001‐SuppMat.pdf.

## Data Availability

The authors have nothing to report.
