# Supplementary material for: Cobalt‐Catalyzed Radical Ligand Transfer (RLT) Enables Remote‐Markovnikov Hydroamination of Alkenes
Source: Angew Chem Int Ed Engl. 2026 Jun 23;65(34):e8746225. doi: 10.1002/anie.8746225 (PMC13480516; doi:10.1002/anie.8746225)
Supplement: Supplementary file 1 [file ANIE-65-e8746225-s001.pdf]

Table of Contents

General Information 6

Photoredox Reaction Setup 7

Experimental Data 8

General Procedure A (for the synthesis of allylic benzoate): 8

2-phenylbut-3-en-2-yl benzoate (1a) 8
2-(2-fluorophenyl)but-3-en-2-yl benzoate (1b) 8
2-(3-fluorophenyl)but-3-en-2-yl benzoate (1c) 9
2-(4-chlorophenyl)but-3-en-2-yl benzoate (1d) 9
2-(4-fluorophenyl)but-3-en-2-yl benzoate (1e) 9
2-(4-bromophenyl)but-3-en-2-yl benzoate (1f) 9
2-(4-acetoxyphenyl)but-3-en-2-yl benzoate (1h) 10
4-(2-(benzoyloxy)but-3-en-2-yl)phenyl thiophene-2-carboxylate (1i) 10
2-(3-methoxyphenyl)but-3-en-2-yl benzoate (1j) 11
4-(2-(benzoyloxy)but-3-en-2-yl)phenyl 1-methyl-1H-pyrrole-2-carboxylate (1k) 11
2-(3-methoxyphenyl)but-3-en-2-yl benzoate (1l) 11
1-(*p*-tolyl)allyl benzoate (1m) 11
1-(*o*-tolyl)allyl benzoate (1n) 12
1-(4-isopropylphenyl)allyl benzoate (1o) 12
1-(4-(tert-butyl)phenyl)allyl benzoate (1p) 12
1-(3,4-dimethylphenyl)allyl benzoate (1q) 13
1-(4-fluoro-3-methoxyphenyl)allyl 4-methoxybenzoate (1s) 13
1-(naphthalen-2-yl)allyl benzoate (1t) 13
1-phenylallyl 4-methoxybenzoate (1u) 14
2-(4-acetoxyphenyl)but-3-en-2-yl 2-methylbenzoate (1v) 14
2-(4-acetoxyphenyl)but-3-en-2-yl 3-methylbenzoate (1w) 14
2-(4-acetoxyphenyl)but-3-en-2-yl 4-methylbenzoate (1x) 15
2-(4-acetoxyphenyl)but-3-en-2-yl 4-fluorobenzoate (1y) 15
2-(4-acetoxyphenyl)but-3-en-2-yl 4-chlorobenzoate (1z) 15
2-(4-acetoxyphenyl)but-3-en-2-yl 3-methoxybenzoate (1aa) 16
2-(4-acetoxyphenyl)but-3-en-2-yl 3-(trifluoromethoxy)benzoate (1ab) 16
2-(4-acetoxyphenyl)but-3-en-2-yl 4-(trifluoromethoxy)benzoate (1ac) 16
2-(4-acetoxyphenyl)but-3-en-2-yl 4-bromobenzoate (1ad) 17
2-(4-acetoxyphenyl)but-3-en-2-yl 2-naphthoate (1ae) 17
1-(4-isopropylphenyl)allyl cyclohexanecarboxylate (1af) 17
1-(4-isopropylphenyl)allyl thiophene-2-carboxylate (1ag) 18
4-(2-(benzoyloxy)but-3-en-2-yl)phenyl 6-(3-((1s,3s)-adamantan-1-yl)-4-methoxyphenyl)-2-naphthoate (1ah) 18

4-(2-(benzoyloxy)but-3-en-2-yl)phenyl 4-(N,N-dipropylsulfamoyl)benzoate (1ai) 19
2-(4-((2-(4-(2,2-dichlorocyclopropyl)phenoxy)-2-methylpropanoyl)oxy)phenyl)but-3-en-2-yl benzoate (1aj) 19
2-(4-((5-(2,5-dimethylphenoxy)-2,2-dimethylpentanoyl)oxy)phenyl)but-3-en-2-yl benzoate (1ak) 20
2-(4-((N-(2-(9H-fluoren-9-yl)acetyl)-N-methyl-D-valyl)oxy)phenyl)but-3-en-2-yl benzoate (1al) 20
Optimization Studies: 20
Screening for cobalt catalyst 21
Screening for solvent 22
Screening for temperature 22
Screening for salt 23
Screening for photocatalyst 23
General Procedure B (for photoredox/cobalt/Brønsted acid-catalyzed hydroamination of ally carboxylates): 24
Additional Substrates 24
Unsuccessful Substrates 24
3-(methyl(phenyl)amino)-3-phenylbutan-2-yl benzoate (3a) 25
3-(methyl(*o*-tolyl)amino)-3-phenylbutan-2-yl benzoate (3b) 25
3-(methyl(m-tolyl)amino)-3-phenylbutan-2-yl benzoate (3c) 25
3-(methyl(p-tolyl)amino)-3-phenylbutan-2-yl benzoate (3d) 26
3-((4-(tert-butyl)phenyl)(methyl)amino)-3-phenylbutan-2-yl benzoate (3e) 26
3-((4-methoxyphenyl)(methyl)amino)-3-phenylbutan-2-yl benzoate (3f) 27
3-((4-fluorophenyl)(methyl)amino)-3-phenylbutan-2-yl benzoate (3g) 27
3-((4-chlorophenyl)(methyl)amino)-3-phenylbutan-2-yl benzoate (3h) 28
3-((4-bromophenyl)(methyl)amino)-3-phenylbutan-2-yl benzoate (3i) 28
3-(methyl(4-(trifluoromethyl)phenyl)amino)-3-phenylbutan-2-yl benzoate (3j) 29
3-(methyl(4-(trifluoromethoxy)phenyl)amino)-3-phenylbutan-2-yl benzoate (3k) 29
3-(methyl(perfluorophenyl)amino)-3-phenylbutan-2-yl benzoate (3l) 30
3-((4-cyanophenyl)(methyl)amino)-3-phenylbutan-2-yl benzoate (3m) 30
ethyl 4-((3-(benzoyloxy)-2-phenylbutan-2-yl)(methyl)amino)benzoate (3n) 31
3-((4-acetylphenyl)(methyl)amino)-3-phenylbutan-2-yl benzoate (3o) 31
3-([1,1'-biphenyl]-4-yl(methyl)amino)-3-phenylbutan-2-yl benzoate (3p) 32
3-([1,1':4',1''-terphenyl]-4-yl(methyl)amino)-3-phenylbutan-2-yl benzoate (3q) 32
3-((4-(dibenzo[*b,d*]thiophen-4-yl)phenyl)(methyl)amino)-3-phenylbutan-2-yl benzoate (3r) 33
3-((4-(dibenzo[*b,d*]furan-1-yl)phenyl)(methyl)amino)-3-phenylbutan-2-yl benzoate (3s) 33
3-(methyl(4-(4,4,5,5-tetramethyl-1,3,2-dioxaborolan-2-yl)phenyl)amino)-3-phenylbutan-2-yl benzoate (3t) 34
3-((2,4-dimethylphenyl)(methyl)amino)-3-phenylbutan-2-yl benzoate (3u) 35
3-((3,5-dimethylphenyl)(methyl)amino)-3-phenylbutan-2-yl benzoate (3v) 35
3-((4-bromo-3-methylphenyl)(methyl)amino)-3-phenylbutan-2-yl benzoate (3w) 35

3-((2-cyano-4-methoxyphenyl)(methyl)amino)-3-phenylbutan-2-yl benzoate (3x) 36
3-((3-iodo-4-methoxyphenyl)(methyl)amino)-3-phenylbutan-2-yl benzoate (3y)......................... 36
3-((2-fluoro-4-methoxyphenyl)(methyl)amino)-3-phenylbutan-2-yl benzoate (3z)...................... 37
3-((3-chloro-4-fluorophenyl)(methyl)amino)-3-phenylbutan-2-yl benzoate (3aa) 38
3-((2-chloro-4-fluoro-5-methylphenyl)(methyl)amino)-3-phenylbutan-2-yl benzoate (3ab)........ 38
3-((5-bromo-4-fluoro-2-methylphenyl)(methyl)amino)-3-phenylbutan-2-yl benzoate (3ac)........ 38
3-((3-cyano-2,4-difluorophenyl)(methyl)amino)-3-phenylbutan-2-yl benzoate (3ad) 39
3-(methyl(naphthalen-1-yl)amino)-3-phenylbutan-2-yl benzoate (3ae) 39
3-(dibenzo[*b,d*]furan-4-ylamino)-3-phenylbutan-2-yl benzoate (3af)... 40
3-(dibenzo[*b,d*]thiophen-4-yl(methyl)amino)-3-phenylbutan-2-yl benzoate (3ag) 40
3-(3,4-dihydroquinolin-1(2H)-yl)-3-phenylbutan-2-yl benzoate (3ah) 41
3-(cyclohexyl(phenyl)amino)-3-phenylbutan-2-yl benzoate (3ai) 41
3-(cyclobutyl(phenyl)amino)-3-phenylbutan-2-yl benzoate (3aj) 42
3-phenyl-3-(phenyl(*m*-tolyl)amino)butan-2-yl benzoate (3ak)... 42
3-phenyl-3-(phenyl(*p*-tolyl)amino)butan-2-yl benzoate (3al).. 43
3-(10,11-dihydro-5*H*-dibenzo[*b,f*]azepin-5-yl)-3-phenylbutan-2-yl benzoate (3am) 43
3-(2-fluorophenyl)-3-(methyl(4-(trifluoromethoxy)phenyl)amino)butan-2-yl benzoate (3an) 44
3-(3-fluorophenyl)-3-(methyl(4-(trifluoromethoxy)phenyl)amino)butan-2-yl benzoate (3ao)..... 44
3-(4-fluorophenyl)-3-(methyl(phenyl)amino)butan-2-yl benzoate (3ap) 45
3-(4-chlorophenyl)-3-(methyl(4-(trifluoromethoxy)phenyl)amino)butan-2-yl benzoate (3aq).... 45
3-(4-bromophenyl)-3-(methyl(4-(trifluoromethoxy)phenyl)amino)butan-2-yl benzoate (3ar) 46
3-(4'-bromo-[1,1'-biphenyl]-4-yl)-3-(methyl(phenyl)amino)butan-2-yl benzoate (3as) 46
3-(4-acetoxyphenyl)-3-(methyl(phenyl)amino)butan-2-yl benzoate (3at).................................... 47
4-(3-(benzoyloxy)-2-(methyl(phenyl)amino)butan-2-yl)phenyl thiophene-2-carboxylate (3au) .. 47
4-(3-(benzoyloxy)-2-(methyl(phenyl)amino)butan-2-yl)phenyl 5-methylthiazole-4-carboxylate (3av)... 48
4-(3-(benzoyloxy)-2-(methyl(phenyl)amino)butan-2-yl)phenyl 1-methyl-1H-pyrrole-2-carboxylate (3aw).. 48
3-(3-methoxyphenyl)-3-(methyl(phenyl)amino)butan-2-yl benzoate (3ax) 49
1-(methyl(phenyl)amino)-1-(p-tolyl)propan-2-yl benzoate (3ay).. 49
1-(methyl(phenyl)amino)-1-(o-tolyl)propan-2-yl benzoate (3az) 50
1-(4-isopropylphenyl)-1-(methyl(phenyl)amino)propan-2-yl benzoate (3aaa)............................. 50
1-(4-(tert-butyl)phenyl)-1-(methyl(phenyl)amino)propan-2-yl benzoate (3aab).......................... 51
1-(3,4-dimethylphenyl)-1-(methyl(phenyl)amino)propan-2-yl benzoate (3aac) 51
1-(benzo[*d*][1,3]dioxol-5-yl)-1-(methyl(phenyl)amino)propan-2-yl benzoate (3aad) 52
1-(methyl(phenyl)amino)-1-(naphthalen-2-yl)propan-2-yl benzoate (3aae)................................ 52
1-(4-fluoro-3-methoxyphenyl)-1-(methyl(phenyl)amino)propan-2-yl 4-methoxybenzoate (3aaf)53
1-(methyl(phenyl)amino)-1-phenylpropan-2-yl 4-methoxybenzoate (3aag)................................ 53
3-(4-acetoxyphenyl)-3-(methyl(4-(trifluoromethoxy)phenyl)amino)butan-2-yl 2-methylbenzoate (3aah) 54

3-(4-acetoxyphenyl)-3-(methyl(4-(trifluoromethoxy)phenyl)amino)butan-2-yl 3-methylbenzoate (3aai)....... 54
3-(4-acetoxyphenyl)-3-(methyl(4-(trifluoromethoxy)phenyl)amino)butan-2-yl 4-methylbenzoate (3aaj) 55
3-(4-acetoxyphenyl)-3-(methyl(phenyl)amino)butan-2-yl 4-fluorobenzoate (3aak)....... 55
3-(4-acetoxyphenyl)-3-(methyl(4-(trifluoromethoxy)phenyl)amino)butan-2-yl 4-chlorobenzoate (3aal)....... 56
3-(4-acetoxyphenyl)-3-(methyl(4-(trifluoromethoxy)phenyl)amino)butan-2-yl 4-(trifluoromethoxy)benzoate (3aam)....... 56
3-(4-acetoxyphenyl)-3-(methyl(4-(trifluoromethoxy)phenyl)amino)butan-2-yl 3-(trifluoromethoxy)benzoate (3aan)....... 57
3-(4-acetoxyphenyl)-3-(methyl(4-(trifluoromethoxy)phenyl)amino)butan-2-yl 3-methoxybenzoate (3aao) 57
3-(4-acetoxyphenyl)-3-(methyl(4-(trifluoromethoxy)phenyl)amino)butan-2-yl 3-fluorobenzoate (3aap) 58
3-(4-acetoxyphenyl)-3-(methyl(4-(trifluoromethoxy)phenyl)amino)butan-2-yl 3-bromobenzoate (3aaq) 58
3-(4-acetoxyphenyl)-3-(methyl(phenyl)amino)butan-2-yl 2-naphthoate (3aar) 59
3-(methyl(4-(trifluoromethoxy)phenyl)amino)-3-phenylbutan-2-yl 4-methoxybenzoate (3aas).. 59
1-(4-isopropylphenyl)-1-(methyl(phenyl)amino)propan-2-yl 3-(trifluoromethyl)benzoate (3aat) 60
1-(4-isopropylphenyl)-1-(methyl(phenyl)amino)propan-2-yl cyclohexanecarboxylate (3aau) 60
1-(4-isopropylphenyl)-1-(methyl(4-(trifluoromethoxy)phenyl)amino)propan-2-yl thiophene-2-carboxylate (3aav)....... 61
4-(3-(benzoyloxy)-2-(methyl(phenyl)amino)butan-2-yl)phenyl 6-(3-((1s,3s)-adamantan-1-yl)-4-methoxyphenyl)-2-naphthoate (4a) 61
4-(3-(benzoyloxy)-2-(methyl(phenyl)amino)butan-2-yl)phenyl 4-(N,N-dipropylsulfamoyl)benzoate (4b)....... 62
3-(4-((2-(4-(2,2-dichlorocyclopropyl)phenoxy)-2-methylpropanoyl)oxy)phenyl)-3-(methyl(phenyl)amino)butan-2-yl benzoate (4c)....... 63
3-(4-(((S)-2-(4-isobutylphenyl)propanoyl)oxy)phenyl)-3-(methyl(4-(trifluoromethoxy)phenyl)amino)butan-2-yl benzoate (4d)....... 63
3-(4-((5-(2,5-dimethylphenoxy)-2,2-dimethylpentanoyl)oxy)phenyl)-3-(methyl(phenyl)amino)butan-2-yl benzoate (4e)....... 64
3-(4-((N-(2-(9H-fluoren-9-yl)acetyl)-N-methyl-D-valyl)oxy)phenyl)-3-(methyl(4-(trifluoromethoxy)phenyl)amino)butan-2-yl benzoate (4f)....... 65
4-(3-(benzoyloxy)-2-(methyl(4-(trifluoromethoxy)phenyl)amino)butan-2-yl)phenyl 2-(3-cyano-4-isobutoxyphenyl)-4-methylthiazole-5-carboxylate (4g)....... 65
3-(4-((3-(4,5-diphenyloxazol-2-yl)propanoyl)oxy)phenyl)-3-(methyl(4-(trifluoromethoxy)phenyl)amino)butan-2-yl benzoate (4h)....... 66

3-(methyl(4-(((R)-2,5,7,8-tetramethyl-2-((4R,8R)-4,8,12-trimethyltridecyl)chroman-6-yl)oxy)phenyl)amino)-3-phenylbutan-2-yl benzoate (4i)....... 67
3-((4-(((3S,5S,8R,9S,10S,13R,14S,17R)-10,13-dimethyl-17-((R)-6-methylheptan-2-yl)hexadecahydro-1H-cyclopenta[a]phenanthren-3-yl)oxy)phenyl)(methyl)amino)-3-phenylbutan-2-yl benzoate (4j)....... 67
3-(methyl(4-(((2aS,4S,5'R,6aS,6bS,8aS,8bR,10R,11aS,12aS,12bR)-5',6a,8a-trimethyldocosahydrospiro[naphtho[2',1':4,5]indeno[2,1-b]furan-10,2'-pyran]-4-yl)oxy)phenyl)amino)-3-phenylbutan-2-yl benzoate (4k) 68
3-(methyl(4-(((8R,9S,13S,14S)-13-methyl-17-oxo-7,8,9,11,12,13,14,15,16,17-decahydro-6H-cyclopenta[a]phenanthren-3-yl)oxy)phenyl)amino)-3-phenylbutan-2-yl benzoate (4l) 69
3-((2-methoxyphenyl)(methyl)amino)-3-phenylbutan-2-yl benzoate (3aaw)....... 69
3-((3-methoxyphenyl)(methyl)amino)-3-phenylbutan-2-yl benzoate (3aax) 70
3-((2-fluorophenyl)(methyl)amino)-3-phenylbutan-2-yl benzoate (3aay) 70
3-((2-chlorophenyl)(methyl)amino)-3-phenylbutan-2-yl benzoate (3aaz) 71
3-((2-bromophenyl)(methyl)amino)-3-phenylbutan-2-yl benzoate (3aaaa)....... 71
3-((3-fluorophenyl)(methyl)amino)-3-phenylbutan-2-yl benzoate (3aaab) 72
3-((3-chlorophenyl)(methyl)amino)-3-phenylbutan-2-yl benzoate (3aaac) 72
3-((3-bromophenyl)(methyl)amino)-3-phenylbutan-2-yl benzoate (3aaad)....... 73
3-(ethyl(phenyl)amino)-3-phenylbutan-2-yl benzoate (3aaae)....... 73
3-(3-chlorophenyl)-3-(methyl(phenyl)amino)butan-2-yl benzoate (3aaaf)....... 73
1-(methyl(phenyl)amino)-1-phenylpropan-2-yl benzoate (3aaag) 74
Gram-Scale Reaction 74
Mechanistic Studies....... 76
Radical trap using TEMPO....... 76
Radical trap using benzenemethanethiol 76
Asymmetric Induction Probing....... 77
^{18}O-Labeling Experiment....... 78
Deuterium-Labeling Experiment 81
Cross-over Experiment....... 83
Stern Volmer Quenching Studies:....... 83
Light on and off experiment....... 84
Quantum Yield Experiment:....... 85
Cobalt-NR_2 complex detection:....... 88
Crystal Data....... 90

DFT Calculations....... 92

Computational Details 92
Spin state of Co species....... 92
Benchmark of the 1,2-radical acyloxy migration (RAM) selectivity 94

Spectroscopic Data 96

Cartesian Coordinates **274**

References **328**

General Information

All air- and moisture-insensitive reactions were carried out under an ambient atmosphere, magnetically stirred, and monitored by thin-layer chromatography (TLC) using Agela Technologies TLC plates pre-coated with 250 μm thickness silica gel 60 F254 plates and visualized by fluorescence quenching under UV light. Flash column chromatography was performed on SiliaFlash® Silica Gel 40-63μm 60 Å particle size using a forced flow of eluent at 0.3–0.5 bar pressure.[1] Preparative TLC was performed on Uniplate® UV254 (20 x 20 cm) with 1000 μm thickness and visualized fluorescence quenching under UV light.

All air and moisture-sensitive manipulations were performed using oven-dried glassware, including standard Schlenk and glovebox techniques under an atmosphere of nitrogen. All reaction vials were capped using green caps with F-217 PTFE liners. Isopropyl acetate was distilled from calcium chloride $CaCl_2$. Diethyl ether and THF were distilled from deep purple sodium benzophenone ketyl. Acetonitrile was dried ver CaH_2 and distilled. Isopropyl acetate and acetonitrile were degassed *via* three freeze-pump-thaw cycles. All other chemicals were used as received.

All deuterated solvents were purchased from Cambridge Isotope Laboratories. NMR spectra were recorded on either a Bruker 800 spectrometer operating at 800 MHz for ^{1}H acquisitions and 201 MHz for ^{13}C acquisitions, a Bruker 500 Advance spectrometer operating at 500 MHz for ^{1}H acquisitions and 125 MHz for ^{13}C acquisitions. A Bruker 400 Nanobay spectrometer was operating at 400 MHz, 100 MHz, and 376 MHz for ^{1}H, ^{13}C, and ^{19}F acquisitions, respectively. Chemical shifts were referenced to the residual proton solvent peaks (^{1}H: $CDCl_3$, δ7.26; CD_3CN, δ1.94) and ^{13}C solvent signals ($CDCl_3$, δ 77.16; CD_3CN, δ118.26).[2] Signals are listed in ppm, and multiplicity identified as s = singlet, br = broad, d = doublet, t = triplet, q = quartet, m = multiplet; coupling constants in Hz; integration.

UV-Vis Absorptions were measured on a Cary 100 UV-Vis spectrophotometer from Agilent Technologies. Emission intensities were recorded using a Perkin Elmer LS50B Luminescence spectrometer. High-resolution mass spectra were performed at University of Illinois Urbana Champaign and were obtained using an Waters LC-UV-TOF mass spectrometer. Concentration under reduced pressure was performed by rotary evaporation at 25–30 °C at the appropriate pressure. Purified compounds were further dried under a high vacuum (0.01–0.05 Torr). Yields refer to purified and spectroscopically pure compounds.

The blue light-emitting diodes used for the quantum yield measurements: 40 W Blue LEDs (Kessil).

Abbreviations: DCM = dichloromethane; THF = tetrahydrofuran

Photoredox Reaction Setup

LED Light: 40 W blue LED (440 nm) Kessil lamp from Kessil.com

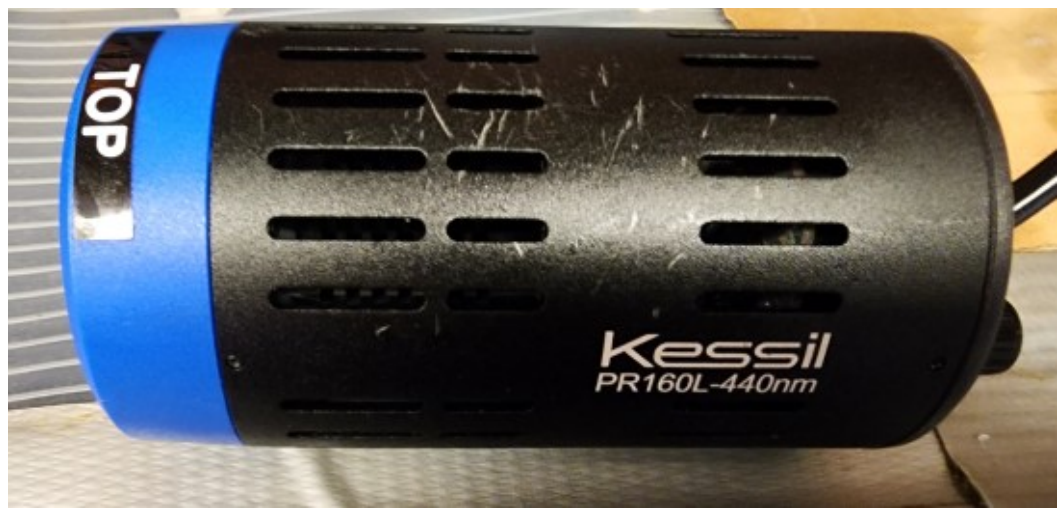

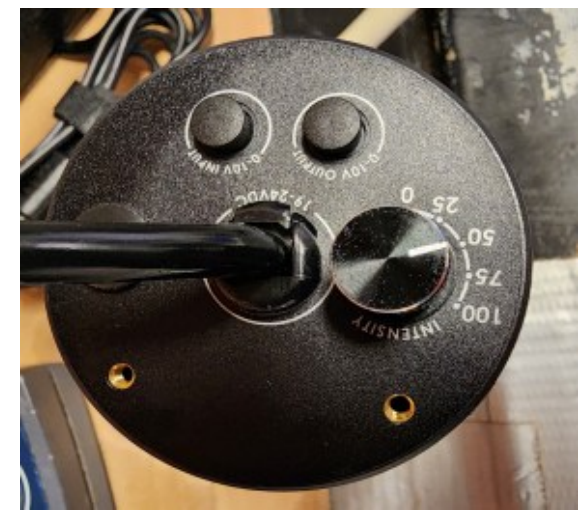

The reaction set up: An oil bath was preheated to 40–80 °C, the 4.00 mL reaction vial was caped and sealed with electrical tape, and then the vial was placed into the oil bath. A 40 W Blue LED lamp (100% intensity) was placed perpendicular to the reaction vial (shown in the picture below). The distance between the blue LED lamp and the vial was 2.00 cm.

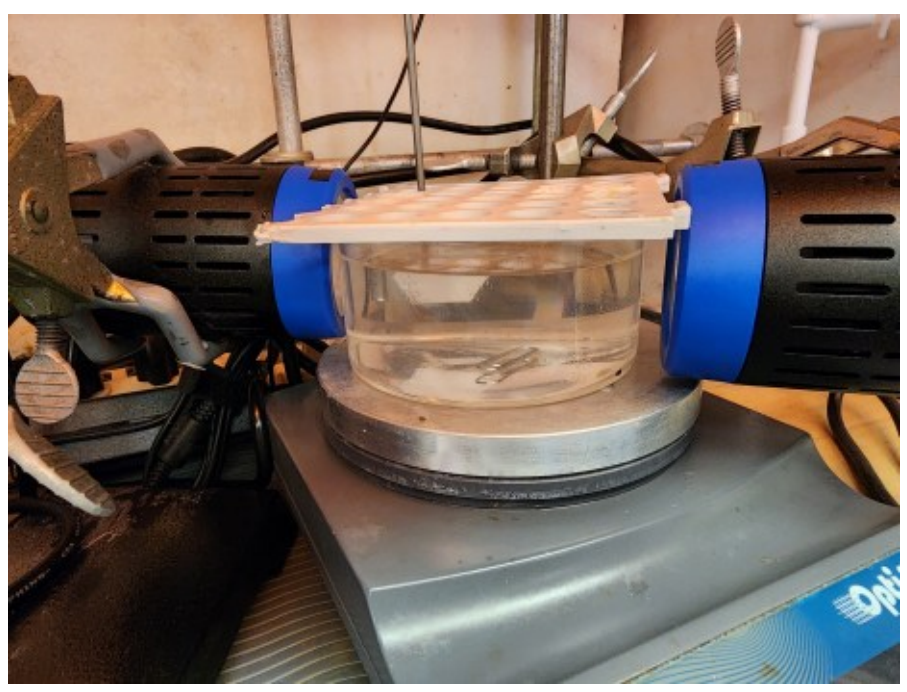

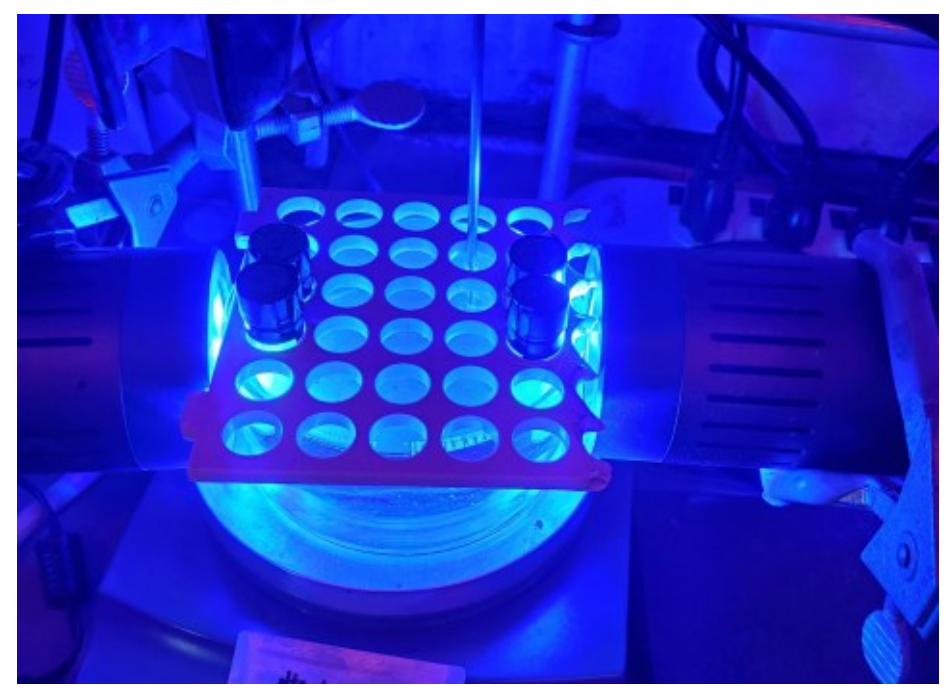

Experimental Data

General Procedure A (for the synthesis of allylic benzoate):

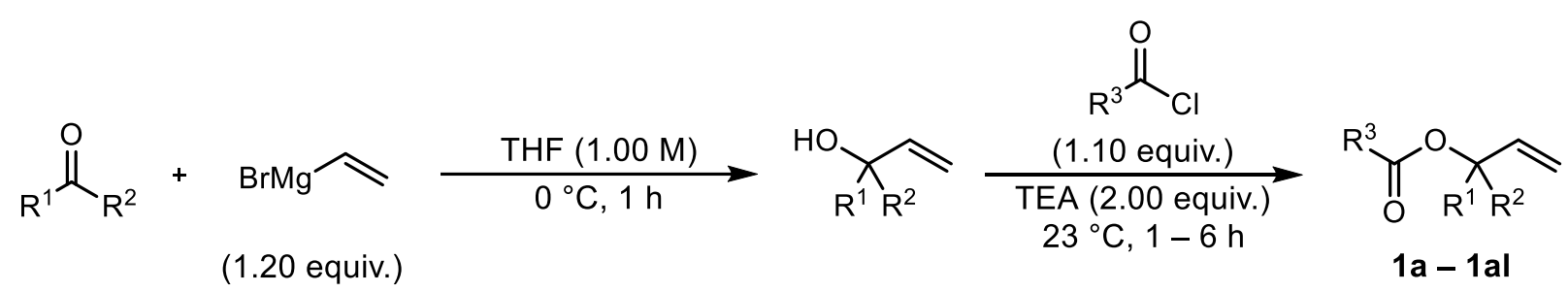

All allylic benzoates were synthesized following a reported literature procedure.[3] The corresponding ketone (1.00 equiv.) and anhydrous THF (1.00 M) were added to a round-bottom flask and cooled to 0 °C under a positive pressure of nitrogen. Vinylmagnesium bromide (1.20 equiv., 1.00 M in THF) was then added dropwise. The mixture was stirred at 0 °C for 1 hour, followed by the addition of triethyl amine (2.00 equiv.) and then corresponding acid chloride (1.10 equiv.). It was warmed to room temperature and stirred for an additional 1 to 6 hours (completion was confirmed by TLC). The reaction was quenched with sat. NH_4Cl, and the organic layer was separated. The aqueous layer was extracted with EtOAc, and the combined organic layers were washed with brine, dried over Na_2SO_4, filtered, and concentrated *in vacuo*. The crude product was purified by flash column silica gel chromatography (eluting with Hex:EtOAc) to afford the allylic benzoates.

2-phenylbut-3-en-2-yl benzoate (1a)

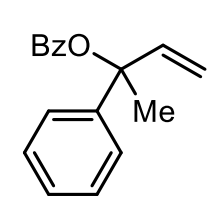

The reaction was performed according to General Procedure A to afford desired product (2.90 g, 69%) as a yellow sticky oil. **^{1}H NMR** (400 MHz, $CDCl_3$) δ 8.17 – 8.09 (m, 2H), 7.63 – 7.54 (m, 1H), 7.54 – 7.43 (m, 4H), 7.45 – 7.32 (m, 2H), 7.35 – 7.24 (m, 1H), 6.44 (dd, *J* = 17.4, 10.8 Hz, 1H), 5.44 (dd, *J* = 17.4, 0.9 Hz, 1H), 5.35 (dd, *J* = 10.8, 0.9 Hz, 1H), 2.08 (s, 3H). **^{13}C NMR** (101 MHz, $CDCl_3$) δ 164.92, 143.82, 141.67, 132.90, 131.48, 129.69, 128.44, 127.41, 125.33, 114.59, 83.81, 25.65.

2-(2-fluorophenyl)but-3-en-2-yl benzoate (1b)

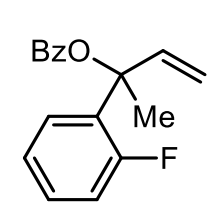

The reaction was performed according to General Procedure A to afford the title compound (986 mg, 51%) as a yellow sicky oil. **^{1}H NMR** (400 MHz, $CDCl_3$) δ 8.14 – 8.03 (m, 2H), 7.66 – 7.39 (m, 4H), 7.39 – 7.21 (m, 1H), 7.16 (td, *J* = 7.7, 1.3 Hz, 1H), 7.04 (ddd, *J* = 12.2, 8.2, 1.4 Hz, 1H), 6.51 (dd, *J* = 17.4, 10.8 Hz, 1H), 5.49 – 5.24 (m, 2H), 2.11 (s, 3H). **^{13}C NMR** (101 MHz, $CDCl_3$) δ 164.75, 160.98, 158.50, 140.40, 132.73, 131.10, 130.39, 130.28, 129.56, 129.33, 129.24, 128.25, 127.31, 127.27, 123.82, 123.78, 116.58, 116.35, 114.50, 81.92, 24.70.

2-(3-fluorophenyl)but-3-en-2-yl benzoate (1c)

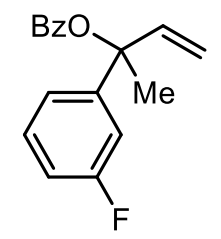

The reaction was performed according to General Procedure A to afford the title compound (0.86 g, 44%) as a yellow sicky oil. **^{1}H NMR** (400 MHz, $CDCl_3$) δ 8.16 – 8.09 (m, 2H), 7.63 (t, *J* = 7.4 Hz, 1H), 7.51 (t, *J* = 7.7 Hz, 2H), 7.43 – 7.33 (m, 1H), 7.33 – 7.25 (m, 1H), 7.26 – 7.18 (m, 1H), 7.08 – 6.98 (m, 1H), 6.43 (dd, *J* = 17.4, 10.8 Hz, 1H), 5.47 (dd, *J* = 17.4, 0.8 Hz, 1H), 5.39 (dd, *J* = 10.8, 0.8 Hz, 1H), 2.08 (s, 3H). **^{13}C NMR** (101 MHz, $CDCl_3$) δ 164.87, 162.91 (d, *J* = 245.7 Hz), 146.58 (d, *J* = 6.8 Hz), 141.08, 133.10, 131.17, 129.99 (d, *J* = 8.1 Hz), 129.71, 128.53, 121.00, 115.15, 114.34 (d, *J* = 21.2 Hz), 112.72 (d, *J* = 23.0 Hz), 83.20, 25.63. **^{19}F NMR** (282 MHz, $CDCl_3$) δ -112.64 – -112.87 (m).

2-(4-chlorophenyl)but-3-en-2-yl benzoate (1d)

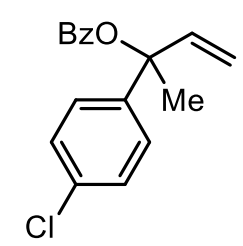

The reaction was performed according to General Procedure A to afford the title compound (962 mg, 52%) as a yellow sicky oil. **1H NMR** (400 MHz, $CDCl_3$) δ 8.09 – 8.05 (m, 2H), 7.61 – 7.55 (m, 1H), 7.49 – 7.44 (m, 2H), 7.42 – 7.38 (m, 2H), 7.37 – 7.31 (m, 2H), 6.37 (dd, *J* = 17.4, 10.8 Hz, 1H), 5.41 (dd, *J* = 17.4, 0.8 Hz, 1H), 5.33 (dd, *J* = 10.8, 0.8 Hz, 1H), 2.02 (s, 3H). **^{13}C NMR** (101 MHz, $CDCl_3$) δ 164.90, 142.35, 141.24, 133.31, 133.10, 131.19, 129.70, 128.86, 128.63, 128.52, 127.60, 127.29, 126.91, 115.04, 83.25, 25.60.

2-(4-fluorophenyl)but-3-en-2-yl benzoate (1e)

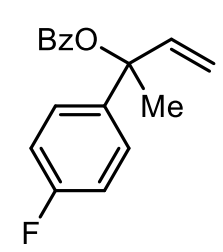

The reaction was performed according to General Procedure A to afford the title compound (2.00 g, 68%) as a yellow sticky oil. **^{1}H NMR** (400 MHz, $CDCl_3$) δ 8.10 – 8.04 (m, 2H), 7.61 – 7.53 (m, 1H), 7.50 – 7.40 (m, 4H), 7.08 – 6.98 (m, 2H), 6.37 (dd, *J* = 17.4, 10.8 Hz, 1H), 5.40 (dd, *J* = 17.3, 0.8 Hz, 1H), 5.32 (dd, *J* = 10.8, 0.8 Hz, 1H), 2.03 (s, 3H). **^{13}C NMR** (101 MHz, $CDCl_3$) δ 164.95, 163.29, 160.84, 141.55, 139.56, 133.06, 131.32, 129.70, 128.52, 127.31, 127.23, 115.39, 115.17, 114.79, 83.38, 25.71. **^{19}F NMR** (282 MHz, $CDCl_3$) δ -115.37 – -115.57 (m).

2-(4-bromophenyl)but-3-en-2-yl benzoate (1f)

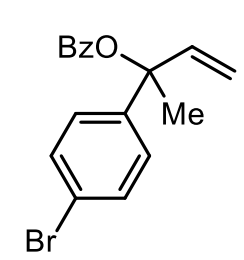

The reaction was performed according to General Procedure A to afford the title compound (1.50 g, 60%) as a yellow sicky oil. ^{1}**H NMR** (400 MHz, $CDCl_3$) δ 8.11 – 8.02 (m, 2H), 7.61 – 7.53 (m, 1H), 7.50 – 7.41 (m, 4H), 7.39 – 7.29 (m, 2H), 6.36 (dd, *J* = 17.4, 10.8 Hz, 1H), 5.41 (dd, *J* = 17.4, 0.8 Hz, 1H), 5.33 (dd, *J* = 10.8, 0.8 Hz, 1H), 2.01 (s, 3H). ^{13}**C NMR** (101 MHz, $CDCl_3$) δ 164.88, 142.90, 141.15, 133.10, 131.81, 131.57, 131.16, 129.69, 128.52, 127.92, 127.24, 121.50, 115.09, 83.27, 25.57.

2-(4'-bromo-[1,1'-biphenyl]-4-yl)but-3-en-2-yl benzoate (1g)

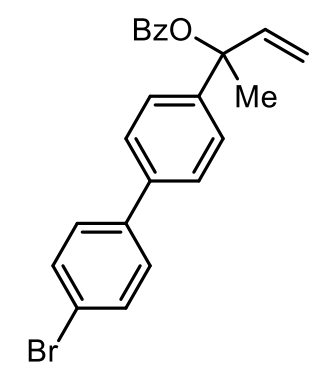

The reaction was performed according to General Procedure A to afford the title compound (500 mg, 34%) as a yellow solid. ^{1}**H NMR** (400 MHz, $CDCl_3$) δ 8.13 – 8.05 (m, 3H), 7.66 – 7.42 (m, 10H), 6.39 (dd, *J* = 17.4, 10.8 Hz, 1H), 5.43 (dd, *J* = 17.4, 0.7 Hz, 1H), 5.36 (dd, *J* = 10.8, 0.7 Hz, 1H), 2.04 (s, 3H). ^{13}**C NMR** (101 MHz, $CDCl_3$) δ 164.68, 147.63, 140.66, 133.01, 130.80, 129.52, 128.37, 126.17, 125.52, 125.32, 115.30, 82.99, 25.49.

2-(4-acetoxyphenyl)but-3-en-2-yl benzoate (1h)

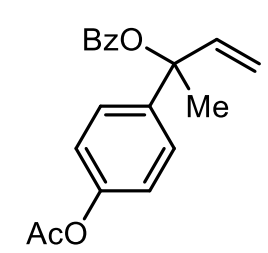

The reaction was performed according to General Procedure A to afford the title compound (500 mg, 29%) as a yellow sicky oil. ^{1}**H NMR** (400 MHz, CD_3Cl) δ 8.11 – 8.04 (m, 2H), 7.60 – 7.53 (m, 1H), 7.52 – 7.42 (m, 4H), 7.13 – 7.06 (m, 2H), 6.38 (dd, *J* = 17.4, 10.8 Hz, 1H), 5.42 (dd, *J* = 17.5, 0.8 Hz, 1H), 5.33 (dd, *J* = 10.8, 0.8 Hz, 1H), 2.30 (s, 3H), 2.04 (s, 3H). ^{13}**C NMR** (101 MHz, $CDCl_3$) δ 169.48, 164.85, 149.83, 141.32, 132.97, 131.28, 129.65, 128.45, 126.59, 121.41, 114.84, 83.44, 25.66, 21.25.

4-(2-(benzoyloxy)but-3-en-2-yl)phenyl thiophene-2-carboxylate (1i)

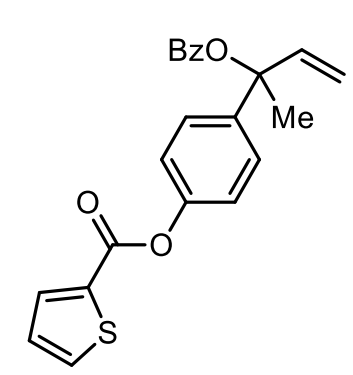

The reaction was performed according to General Procedure A to afford the title compound (493 mg, 32%) as a yellow sticky oil. ^{1}**H NMR** (400 MHz, $CDCl_3$) δ 8.13 – 8.05 (m, 2H), 8.01 – 7.94 (m, 1H), 7.69 – 7.63 (m, 1H), 7.61 – 7.48 (m, 3H), 7.46 (t, *J* = 7.8 Hz, 2H), 7.28 – 7.19 (m, 2H), 7.22 – 7.14 (m, 1H), 6.40 (dd, *J* = 17.4, 10.8 Hz, 1H), 5.48 – 5.39 (m, 1H), 5.38 – 5.31 (m, 1H), 2.06 (s, 3H). ^{13}**C NMR** (101 MHz, $CDCl_3$) δ 164.73, 160.40, 149.58, 141.35, 141.18, 134.66, 133.52, 132.83, 132.75, 131.14, 129.52, 128.84, 128.32, 127.98, 126.53, 121.34, 114.77, 83.31, 25.53.

2-(3-methoxyphenyl)but-3-en-2-yl benzoate (1j)

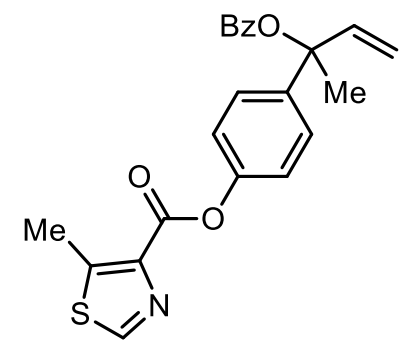

The reaction was performed according to General Procedure A to afford the title compound (768 mg, 34%) as a orange sticky oil. **^{1}H NMR** (400 MHz, $CDCl_3$) δ 8.86 (s, 1H), 8.10 – 8.03 (m, 2H), 7.61 – 7.49 (m, 3H), 7.49 – 7.40 (m, 2H), 7.23 – 7.16 (m, 2H), 6.39 (dd, J = 17.4, 10.8 Hz, 1H), 5.47 – 5.37 (m, 1H), 5.37 – 5.29 (m, 1H), 2.83 (s, 3H), 2.04 (s, 3H). **^{13}C NMR** (101 MHz, $CDCl_3$) δ 164.70, 162.35, 160.37, 156.17, 149.16, 141.67, 141.11, 132.85, 131.08, 129.51, 128.31, 126.59, 121.31, 121.10, 114.83, 83.23, 25.53, 17.49.

4-(2-(benzoyloxy)but-3-en-2-yl)phenyl 1-methyl-1H-pyrrole-2-carboxylate (1k)

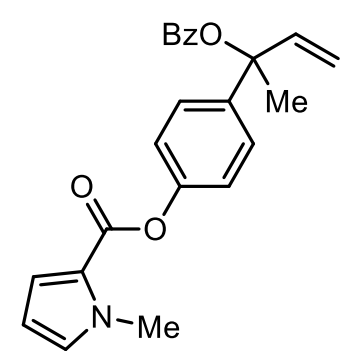

The reaction was performed according to General Procedure A to afford the title compound (600 mg, 39%) as a white solid. **^{1}H NMR** (400 MHz, $CDCl_3$) δ 8.11 – 8.04 (m, 2H), 7.60 – 7.41 (m, 5H), 7.21 – 7.13 (m, 3H), 6.89 (t, J = 2.2 Hz, 1H), 6.39 (dd, J = 17.4, 10.8 Hz, 1H), 6.20 (dd, J = 4.0, 2.5 Hz, 1H), 5.42 (dd, J = 17.4, 0.8 Hz, 1H), 5.33 (dd, J = 10.8, 0.8 Hz, 1H), 3.96 (s, 3H), 2.05 (s, 3H). **^{13}C NMR** (101 MHz, $CDCl_3$) δ 159.70, 149.84, 141.46, 141.14, 132.99, 131.40, 130.78, 129.73, 128.50, 126.65, 121.86, 121.49, 119.38, 114.91, 108.48, 83.60, 37.05, 25.72.

2-(3-methoxyphenyl)but-3-en-2-yl benzoate (1l)

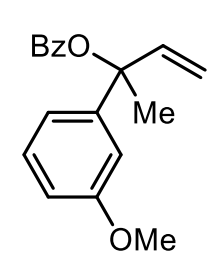

The reaction was performed according to General Procedure A to afford the title compound (1.10 g, 59%) as a yellow sicky oil. **^{1}H NMR** (300 MHz, $CDCl_3$) δ 8.14 – 8.05 (m, 2H), 7.62 – 7.52 (m, 1H), 7.50 – 7.38 (m, 2H), 7.35 – 7.22 (m, 1H), 7.10 – 7.00 (m, 2H), 6.88 – 6.80 (m, 1H), 6.40 (dd, J = 17.5, 10.8 Hz, 1H), 5.43 (dd, J = 17.4, 0.9 Hz, 1H), 5.32 (dd, J = 10.8, 0.9 Hz, 1H), 3.80 (s, 3H), 2.04 (s, 3H). **^{13}C NMR** (75 MHz, $CDCl_3$) δ 164.91, 159.65, 145.61, 141.52, 132.91, 131.46, 129.70, 129.48, 128.45, 117.72, 114.65, 112.17, 111.88, 83.67, 55.28, 25.65.

1-(*p*-tolyl)allyl benzoate (1m)

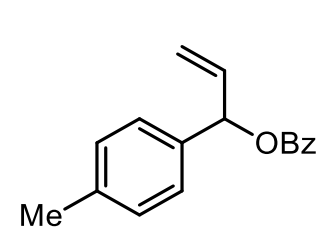

The reaction was performed according to General Procedure A to afford the title compound (1.60 g, 76%) as a yellow sticky oil. **^{1}H NMR** (300 MHz, $CDCl_3$) δ 8.26 – 8.02 (m, 2H), 7.64 – 7.54 (m, 1H), 7.52 – 7.43 (m, 2H), 7.43 – 7.34 (m, 2H), 7.23 (d, *J* = 7.9 Hz, 2H), 6.54 (dt, *J* = 5.9, 1.5 Hz, 1H), 6.17 (ddd, *J* = 17.2, 10.4, 5.7 Hz, 1H), 5.68 – 5.05 (m, 2H), 2.39 (s, 3H). **^{13}C NMR** (75 MHz, $CDCl_3$) δ 165.56, 138.04, 136.48, 136.06, 133.03, 130.42, 129.74, 129.32, 128.39, 127.19, 116.82, 21.21.

1-(*o*-tolyl)allyl benzoate (1n)

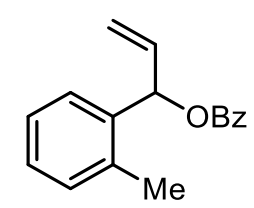

The reaction was performed according to General Procedure A to afford the title compound (1.66 g, 79%) as a yellow sicky oil. **^{1}H NMR** (300 MHz, $CDCl_3$) δ 8.22 – 8.15 (m, 2H), 7.69 – 7.59 (m, 1H), 7.58 – 7.47 (m, 3H), 7.38 – 7.21 (m, 3H), 6.77 (dt, *J* = 5.5, 1.5 Hz, 1H), 6.20 (ddd, *J* = 17.2, 10.5, 5.5 Hz, 1H), 5.51 – 5.31 (m, 2H), 2.53 (s, 3H). **^{13}C NMR** (75 MHz, $CDCl_3$) δ 165.54, 137.10, 135.85, 135.76, 133.07, 130.64, 130.44, 130.32, 130.20, 129.74, 129.29, 128.59, 128.43, 128.11, 126.99, 126.29, 126.08, 117.07, 74.08, 65.25, 19.34.

1-(4-isopropylphenyl)allyl benzoate (1o)

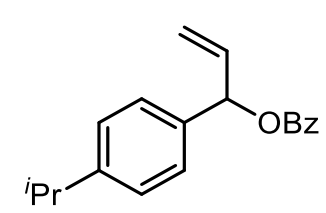

The reaction was performed according to General Procedure A to afford the title compound (1.50 g, 79%) as a yellow sticky oil. **^{1}H NMR** (300 MHz, $CDCl_3$) δ 8.09 – 7.93 (m, 2H), 7.50 – 7.39 (m, 1H), 7.38 – 7.22 (m, 4H), 7.19 – 7.04 (m, 2H), 6.42 (dt, *J* = 5.8, 1.5 Hz, 1H), 6.04 (ddd, *J* = 17.2, 10.4, 5.8 Hz, 1H), 5.49 – 5.06 (m, 2H), 2.81 (hept, *J* = 6.9 Hz, 1H), 1.15 (d, *J* = 7.0 Hz, 7H). **^{13}C NMR** (75 MHz, $CDCl_3$) δ 165.57, 148.96, 136.48, 136.36, 133.02, 130.44, 129.75, 128.40, 127.25, 126.71, 116.80, 33.90, 23.98.

1-(4-(tert-butyl)phenyl)allyl benzoate (1p)

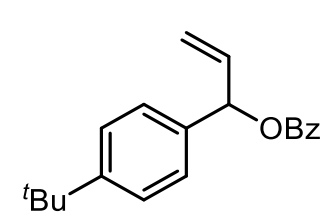

The reaction was performed according to General Procedure A to afford the title compound (1.50 mg, 55%) as a yellow sicky oil. **^{1}H NMR** (300 MHz, $CDCl_3$) δ 8.22 – 8.01 (m, 2H), 7.87 – 7.50 (m, 1H), 7.50 – 7.34 (m, 6H), 6.52 (dt, *J* = 5.9, 1.4 Hz, 1H), 6.14 (ddd, *J* = 17.2, 10.4, 5.9 Hz, 1H), 5.59 – 5.17 (m, 2H), 1.33 (s, 9H). **^{13}C NMR** (75 MHz, $CDCl_3$) δ 165.56, 151.19, 136.43, 135.96, 133.01, 130.42, 129.74, 128.38, 126.93, 125.55, 116.80, 60.39, 34.60, 31.33, 14.22.

1-(3,4-dimethylphenyl)allyl benzoate (1q)

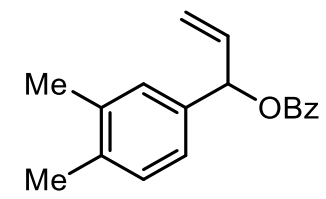

The reaction was performed according to General Procedure A to afford the title compound (1.34 g, 68%) as a yellow sicky oil. **^{1}H NMR** (300 MHz, $CDCl_3$) δ 8.22 – 8.10 (m, 2H), 7.67 – 7.53 (m, 1H), 7.53 – 7.39 (m, 2H), 7.31 – 7.13 (m, 3H), 6.51 (dt, *J* = 5.8, 1.5 Hz, 1H), 6.17 (ddd, *J* = 17.2, 10.4, 5.8 Hz, 1H), 5.55 – 5.18 (m, 2H), 2.38 – 2.20 (m, 6H). **^{13}C NMR** (75 MHz, $CDCl_3$) δ 165.64, 136.93, 136.79, 136.63, 136.56, 133.06, 130.54, 129.96, 129.82, 128.59, 128.45, 124.73, 116.71, 19.94, 19.61.

1-(benzo[*d*][1,3]dioxol-5-yl)allyl benzoate (1r)

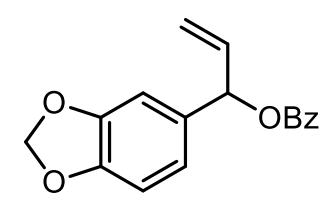

The reaction was performed according to General Procedure A to afford the title compound (1.32 G, 70%) as a yellow solid. **^{1}H NMR** (400 MHz, $CDCl_3$) δ 8.13 – 8.03 (m, 2H), 7.56 (ddt, *J* = 8.0, 6.8, 1.3 Hz, 1H), 7.49 – 7.39 (m, 2H), 7.00 – 6.91 (m, 2H), 6.80 (dt, *J* = 8.1, 0.7 Hz, 1H), 6.43 (d, *J* = 5.6 Hz, 1H), 6.10 (ddd, *J* = 17.2, 10.5, 5.6 Hz, 1H), 5.97 – 5.91 (m, 2H), 5.39 (dt, *J* = 17.1, 1.4 Hz, 1H), 5.29 (dt, *J* = 10.5, 1.3 Hz, 1H). **^{13}C NMR** (101 MHz, $CDCl_3$) δ 165.60, 148.00, 147.68, 136.44, 133.19, 132.98, 130.41, 129.83, 128.52, 121.19, 116.91, 108.39, 107.88, 101.30, 76.56.

1-(4-fluoro-3-methoxyphenyl)allyl 4-methoxybenzoate (1s)

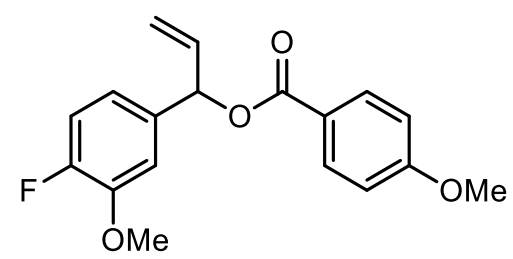

The reaction was performed according to General Procedure A to afford the title compound (1.40 g, 75%) as a orange sticky oil. **^{1}H NMR** (400 MHz, $CDCl_3$) δ 8.04 (d, *J* = 8.9 Hz, 2H), 7.12 – 6.96 (m, 3H), 6.97 – 6.89 (m, 2H), 6.44 (d, *J* = 5.6 Hz, 1H), 6.19 – 6.00 (m, 1H), 5.52 – 5.18 (m, 2H), 3.87 (d, *J* = 14.8 Hz, 6H). **^{13}C NMR** (101 MHz, $CDCl_3$) δ 165.11, 163.46, 136.11, 135.39, 131.64, 122.39, 119.60, 119.53, 116.93, 116.02, 115.84, 113.60, 112.47, 75.66, 56.18, 55.35. **^{19}F NMR** (376 MHz, $CDCl_3$) δ -134.72 – -139.81 (m).

1-(naphthalen-2-yl)allyl benzoate (1t)

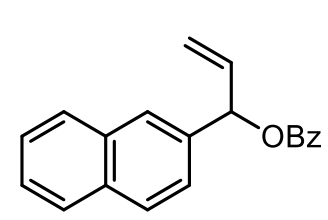

The reaction was performed according to General Procedure A to afford desired product (1.40 g, 76%) as a yellow sticky oil. **^{1}H NMR** (300 MHz, $CDCl_3$) δ 8.24 – 8.09 (m, 2H), 8.00 – 7.77 (m, 4H), 7.67 – 7.37 (m,

6H), 6.72 (dd, J = 5.7, 1.6 Hz, 1H), 6.25 (ddd, J = 17.2, 10.5, 5.7 Hz, 1H), 5.57 – 5.32 (m, 2H). **^{13}C NMR** (75 MHz, $CDCl_3$) δ 165.59, 136.33, 136.30, 133.25, 133.22, 133.13, 130.34, 129.79, 128.53, 128.46, 128.17, 127.74, 126.37, 126.33, 124.94, 117.32.

1-phenylallyl 4-methoxybenzoate (1u)

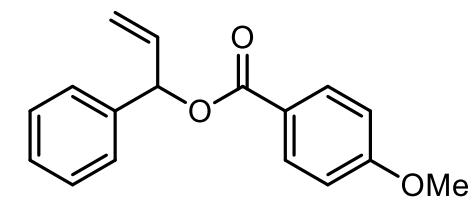

The reaction was performed according to General Procedure A to afford the title compound (1.62 g, 82%) as a yellow sticky oil. **^{1}H NMR** (300 MHz, $CDCl_3$) δ 8.12 – 7.99 (m, 2H), 7.50 – 7.28 (m, 5H), 6.97 – 6.88 (m, 2H), 6.51 (dt, J = 5.8, 1.5 Hz, 1H), 6.13 (ddd, J = 17.1, 10.4, 5.8 Hz, 1H), 5.55 – 5.24 (m, 2H), 3.86 (s, 3H). **^{13}C NMR** (75 MHz, $CDCl_3$) δ 165.27, 163.50, 139.20, 136.55, 131.78, 128.60, 128.13, 127.12, 122.72, 116.89, 113.67, 66.41, 55.45.

2-(4-acetoxyphenyl)but-3-en-2-yl 2-methylbenzoate (1v)

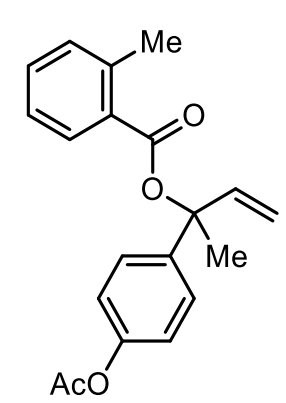

The reaction was performed according to General Procedure A to afford the title compound (380 mg, 21%) as a yellow sticky oil. **^{1}H NMR** (400 MHz, Chloroform-*d*) δ 7.95 (dd, J = 7.7, 1.5 Hz, 1H), 7.50 – 7.42 (m, 2H), 7.45 – 7.35 (m, 1H), 7.30 – 7.20 (m, 2H), 7.12 – 7.03 (m, 2H), 6.39 (dd, J = 17.4, 10.8 Hz, 1H), 5.38 (dd, J = 17.4, 0.8 Hz, 1H), 5.31 (dd, J = 10.8, 0.8 Hz, 1H), 2.55 (s, 3H), 2.29 (s, 3H), 2.01 (s, 3H). **^{13}C NMR** (201 MHz, $CDCl_3$) δ 155.74, 152.18, 136.06, 127.69, 126.47, 118.12, 118.05, 117.02, 116.71, 115.00, 112.83, 112.06, 108.24, 107.63, 101.13, 69.71, 12.16, 8.03, 7.51.

2-(4-acetoxyphenyl)but-3-en-2-yl 3-methylbenzoate (1w)

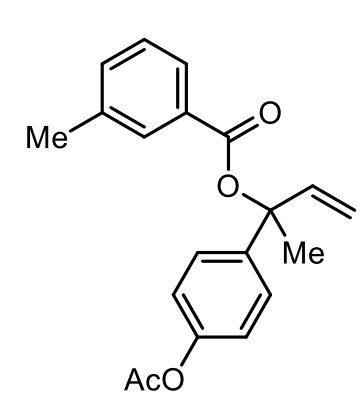

The reaction was performed according to General Procedure A to afford the title compound (500 mg, 28%) as a yellow sticky oil. **^{1}H NMR** (400 MHz, $CDCl_3$) δ 7.90 – 7.86 (m, 2H), 7.51 – 7.46 (m, 2H), 7.40 – 7.31 (m, 2H), 7.12 – 7.06 (m, 2H), 6.39 (dd, J = 17.4, 10.8 Hz, 1H), 5.41 (dd, J = 17.4, 0.8 Hz, 1H), 5.33 (dd, J = 10.8, 0.8 Hz, 1H), 2.42 (s, 3H), 2.30 (s, 3H), 2.04 (s, 3H). **^{13}C NMR** (101 MHz, $CDCl_3$) δ 169.48, 165.07, 149.86, 141.44, 138.24, 133.73, 131.28, 130.22, 128.37, 126.82, 126.61, 121.40, 114.82, 83.39, 25.74, 21.40, 21.24.

2-(4-acetoxyphenyl)but-3-en-2-yl 4-methylbenzoate (1x)

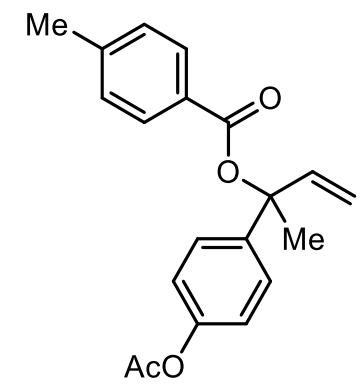

The reaction was performed according to General Procedure A to afford the title compound (673 mg, 37%) as a yellow solid. **^{1}H NMR** (400 MHz, $CDCl_3$) δ 7.95 (d, *J* = 8.0 Hz, 2H), 7.47 (d, *J* = 8.7 Hz, 2H), 7.28 – 7.20 (m, 2H), 7.08 (d, *J* = 8.7 Hz, 2H), 6.36 (dd, *J* = 17.4, 10.8 Hz, 1H), 5.40 (d, *J* = 17.4 Hz, 1H), 5.31 (d, *J* = 10.8 Hz, 1H), 2.41 (s, 3H), 2.29 (s, 3H), 2.02 (s, 3H). **^{13}C NMR** (101 MHz, $CDCl_3$) δ 169.32, 164.80, 149.65, 143.45, 141.32, 141.29, 130.13, 129.53, 129.10, 128.99, 128.44, 127.17, 126.42, 121.20, 114.55, 83.07, 25.55, 21.56, 21.06.

2-(4-acetoxyphenyl)but-3-en-2-yl 4-fluorobenzoate (1y)

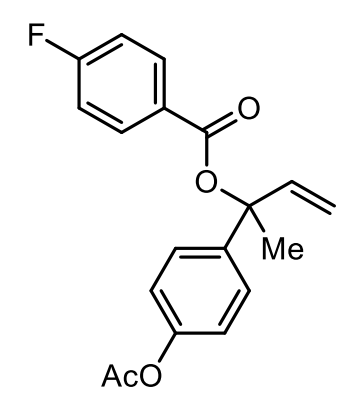

The reaction was performed according to General Procedure A to afford the title compound (1.00 g, 54%) as a yellow sticky oil. **^{1}H NMR** (400 MHz, $CDCl_3$) δ 8.09 – 8.03 (m, 2H), 7.49 – 7.42 (m, 2H), 7.15 – 7.06 (m, 4H), 6.36 (dd, *J* = 17.4, 10.8 Hz, 1H), 5.39 (d, *J* = 17.4 Hz, 1H), 5.33 (d, *J* = 10.8 Hz, 1H), 2.29 (s, 3H), 2.02 (s, 3H). **^{13}C NMR** (101 MHz, $CDCl_3$) δ 169.47, 165.84 (d, *J* = 253.5 Hz), 163.95, 149.95, 141.24, 141.19, 132.21 (d, *J* = 9.1 Hz), 127.60, 126.61, 121.46, 115.60 (d, *J* = 21.9 Hz), 115.01, 83.72, 25.68, 21.25. **^{19}F NMR** (282 MHz, $CDCl_3$) δ -105.81 – -105.98 (m).

2-(4-acetoxyphenyl)but-3-en-2-yl 4-chlorobenzoate (1z)

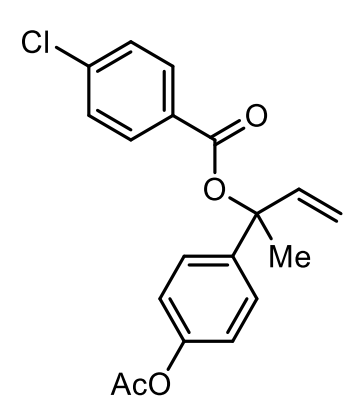

The reaction was performed according to General Procedure A to afford the title compound (500 mg, 26%) as a yellow sticky oil. **^{1}H NMR** (400 MHz, $CDCl_3$) δ 8.03 – 7.94 (m, 2H), 7.49 – 7.38 (m, 4H), 7.12 – 7.03 (m, 2H), 6.36 (dd, *J* = 17.4, 10.8 Hz, 1H), 5.39 (d, *J* = 17.4 Hz, 1H), 5.33 (d, *J* = 10.8 Hz, 1H), 2.29 (s, 3H), 2.02 (s, 3H). **^{13}C NMR** (101 MHz, $CDCl_3$) δ 169.50, 164.08, 149.99, 141.17, 139.46, 131.11, 129.82, 128.84, 126.64, 121.50, 115.14, 83.91, 25.69, 21.29.

2-(4-acetoxyphenyl)but-3-en-2-yl 3-methoxybenzoate (1aa)

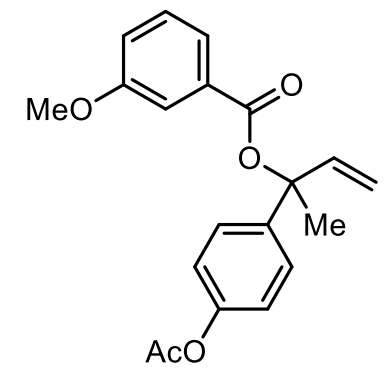

The reaction was performed according to General Procedure A to afford the title compound (700 mg, 37%) as a yellow sticky oil. *Major diastereomer*: **^{1}H NMR** (300 MHz, $CDCl_3$) δ 7.73 – 7.65 (m, 1H), 7.63 – 7.31 (m, 4H), 7.16 – 7.05 (m, 3H), 6.47 – 6.21 (m, 1H), 5.49 – 5.23 (m, 2H), 3.85 (s, 3H), 2.30 (s, 3H), 2.04 (s, 3H). *Mixture of diastereomer*: **^{13}C NMR** (75 MHz, $CDCl_3$) δ 169.43, 164.75, 159.78, 159.66, 149.89, 141.34, 141.26, 132.66, 129.70, 129.48, 127.41, 126.70, 126.60, 126.53, 122.66, 122.01, 121.74, 121.55, 121.41, 121.28, 120.25, 119.40, 114.84, 114.70, 114.61, 114.23, 83.56, 82.85, 55.57, 55.47, 25.66, 25.55, 22.27, 21.22.

2-(4-acetoxyphenyl)but-3-en-2-yl 3-(trifluoromethoxy)benzoate (1ab)

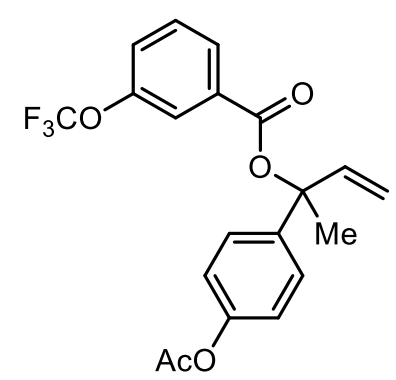

The reaction was performed according to General Procedure A to afford the title compound (700 mg, 32%) as a yellow sticky oil. **^{1}H NMR** (400 MHz, $CDCl_3$) δ 8.00 – 7.96 (m, 1H), 7.91 – 7.87 (m, 1H), 7.52 – 7.39 (m, 4H), 7.12 – 7.06 (m, 2H), 6.36 (dd, J = 17.4, 10.8 Hz, 1H), 5.40 (d, J = 17.4 Hz, 1H), 5.34 (d, J = 10.8 Hz, 1H), 2.29 (s, 3H), 2.03 (s, 3H). **^{13}C NMR** (101 MHz, $CDCl_3$) δ 169.50, 163.53, 150.05, 149.39, 141.00, 140.91, 133.44, 130.04, 127.99, 126.67, 125.38, 122.12, 121.54, 120.55 (d, J = 257.3 Hz), 115.28, 84.25, 25.62, 21.27. **^{19}F NMR** (376 MHz, $CDCl_3$) δ -59.38

2-(4-acetoxyphenyl)but-3-en-2-yl 4-(trifluoromethoxy)benzoate (1ac)

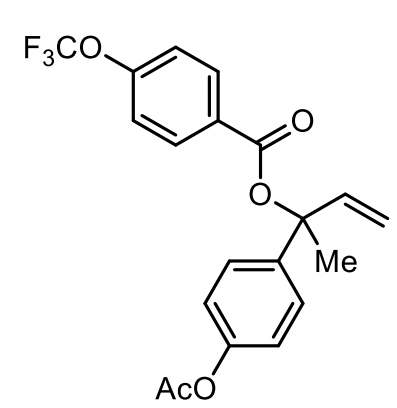

The reaction was performed according to General Procedure A to afford the title compound (521 mg, 23%) as a yellow sticky oil. **^{1}H NMR** (800 MHz, $CDCl_3$) δ 8.10 (d, J = 8.8 Hz, 2H), 7.48 – 7.45 (m, 2H), 7.28 (d, J = 8.4 Hz, 2H), 7.09 (d, J = 8.8 Hz, 2H), 6.37 (dd, J = 17.4, 10.8 Hz, 1H), 5.40 (d, J = 17.4 Hz, 1H), 5.34 (d, J = 10.8 Hz, 1H), 2.29 (s, 3H), 2.03 (s, 3H). **^{13}C NMR** (201 MHz, $CDCl_3$) δ 169.38, 163.62, 152.59, 149.90, 141.00, 140.94, 131.66, 131.58, 129.67, 126.52, 121.41, 120.31, 115.05, 83.89, 25.55, 21.14.

2-(4-acetoxyphenyl)but-3-en-2-yl 4-bromobenzoate (1ad)

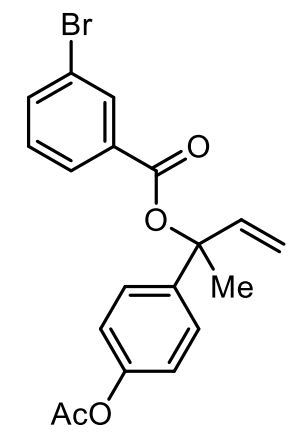

The reaction was performed according to General Procedure A to afford the title compound (706 mg, 32%) as a yellow sticky oil. **^{1}H NMR** (800 MHz, $CDCl_3$) δ 8.17 (s, 1H), 7.97 (dd, J = 7.8, 1.4 Hz, 1H), 7.70 – 7.67 (m, 1H), 7.46 – 7.42 (m, 2H), 7.32 (t, J = 7.9 Hz, 1H), 7.10 – 7.07 (m, 2H), 6.40 – 6.34 (m, 1H), 5.41 – 5.37 (m, 1H), 5.33 (dd, J = 10.8, 1.1 Hz, 1H), 2.29 (s, 3H), 2.02 (s, 3H). **^{13}C NMR** (201 MHz, $CDCl_3$) δ 169.40, 163.45, 149.89, 140.90, 140.86, 135.84, 133.19, 132.57, 129.98, 128.19, 126.53, 122.49, 121.40, 115.18, 84.03, 25.60, 21.18.

2-(4-acetoxyphenyl)but-3-en-2-yl 2-naphthoate (1ae)

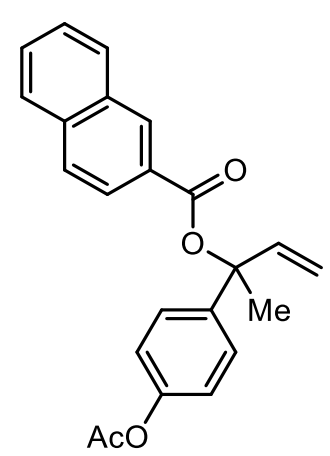

The reaction was performed according to General Procedure A to afford the title compound (202 mg, 10%) as a white solid. **^{1}H NMR** (400 MHz, Chloroform-*d*) δ 8.67 – 8.62 (m, 1H), 8.16 – 8.08 (m, 1H), 8.01 – 7.95 (m, 1H), 7.90 – 7.87 (m, 2H), 7.65 – 7.51 (m, 4H), 7.17 – 7.05 (m, 2H), 6.46 (dd, J = 17.4, 10.8 Hz, 1H), 5.47 (dd, J = 17.4, 0.8 Hz, 1H), 5.37 (dd, J = 10.8, 0.8 Hz, 1H), 2.30 (s, 3H), 2.10 (s, 3H). **^{13}C NMR** (75 MHz, $CDCl_3$) δ 169.52, 165.11, 149.92, 141.41, 135.62, 132.60, 131.11, 129.61, 129.47, 128.59, 128.33, 128.26, 127.87, 127.46, 127.05, 126.75, 126.67, 125.38, 121.80, 121.69, 121.47, 121.31, 115.00, 83.68, 25.82, 21.26.

1-(4-isopropylphenyl)allyl cyclohexanecarboxylate (1af)

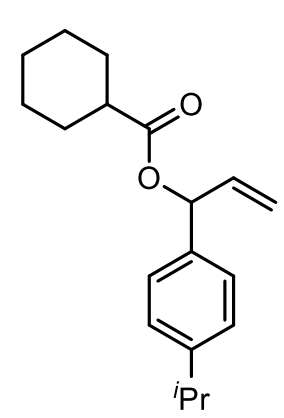

The reaction was performed according to General Procedure A to afford the title compound (1.20 g, 73%) as a yellow sicky oil. **^{1}H NMR** (400 MHz, $CDCl_3$) δ 7.34 – 7.18 (m, 4H), 6.27 (d, J = 5.8 Hz, 1H), 6.02 (ddd, J = 16.6, 10.4, 5.8 Hz, 1H), 5.39 – 5.20 (m, 2H), 2.93 (hept, J = 6.9 Hz, 1H), 2.52 – 2.27 (m, 1H), 2.05 – 1.89 (m, 2H), 1.84 – 1.72 (m, 2H), 1.73 – 1.59 (m, 2H), 1.58 – 1.39 (m, 2H), 1.27 (d, J = 6.9 Hz, 8H). **^{13}C**

NMR (101 MHz, $CDCl_3$) δ 174.86, 148.59, 136.55, 136.44, 128.05, 126.90, 126.49, 116.26, 75.42, 43.26, 33.74, 28.93, 28.84, 25.67, 25.35, 23.85.

1-(4-isopropylphenyl)allyl thiophene-2-carboxylate (1ag)

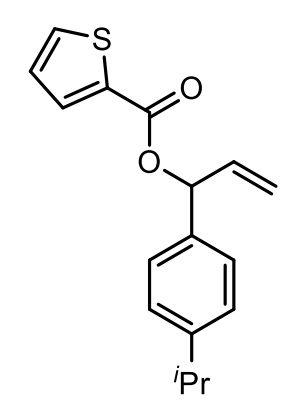

The reaction was performed according to General Procedure A to afford the title compound (1.32 g, 79%) as a yellow sicky oil. **^{1}H NMR** (400 MHz, $CDCl_3$) δ 7.76 (dd, J = 3.8, 1.2 Hz, 1H), 7.47 (dd, J = 5.0, 1.2 Hz, 1H), 7.29 (d, J = 8.1 Hz, 2H), 7.15 (d, J = 8.1 Hz, 2H), 7.01 (dd, J = 4.9, 3.7 Hz, 1H), 6.37 (d, J = 5.9 Hz, 1H), 6.02 (ddd, J = 16.6, 10.5, 5.8 Hz, 1H), 5.46 – 5.09 (m, 2H), 2.82 (hept, J = 6.8 Hz, 1H), 1.16 (d, J = 6.9 Hz, 6H). **^{13}C NMR** (101 MHz, $CDCl_3$) δ 161.14, 148.89, 136.13, 136.00, 133.86, 133.48, 132.38, 128.33, 127.65, 127.10, 126.58, 116.81, 66.62, 33.78, 23.85.

4-(2-(benzoyloxy)but-3-en-2-yl)phenyl 6-(3-((1s,3s)-adamantan-1-yl)-4-methoxyphenyl)-2-naphthoate (1ah)

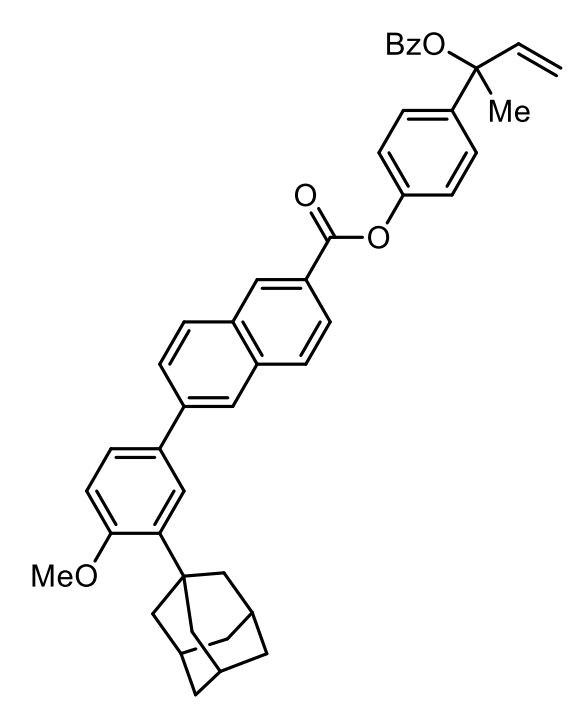

The reaction was performed according to General Procedure A to afford the title compound (111 mg, 59%) as a white foam . **^{1}H NMR** (400 MHz, $CDCl_3$) δ 8.76 (s, 1H), 8.27 – 8.15 (m, 1H), 8.12 – 7.88 (m, 5H), 7.81 (dd, J = 8.5, 1.8 Hz, 1H), 7.61 (d, J = 2.4 Hz, 1H), 7.58 – 7.51 (m, 3H), 7.44 (t, J = 7.6 Hz, 2H), 7.32 – 7.17 (m, 3H), 6.99 (d, J = 8.5 Hz, 1H), 6.40 (dd, J = 17.4, 10.8 Hz, 1H), 5.61 – 5.17 (m, 2H), 3.89 (s, 3H), 2.18 (s, 6H), 2.13 – 2.00 (m, 6H), 1.80 (s, 6H). **^{13}C NMR** (101 MHz, $CDCl_3$) δ 164.75, 158.93, 150.13, 141.70, 141.24, 138.96, 136.20, 132.79, 132.35, 131.64, 131.22, 131.15, 129.77, 129.53, 128.37, 128.30, 126.56, 126.11, 125.91, 125.69, 124.67, 121.46, 114.73, 112.05, 83.39, 55.09, 40.54, 37.14, 37.05, 29.03, 25.56.

4-(2-(benzoyloxy)but-3-en-2-yl)phenyl 4-(N,N-dipropylsulfamoyl)benzoate (1ai)

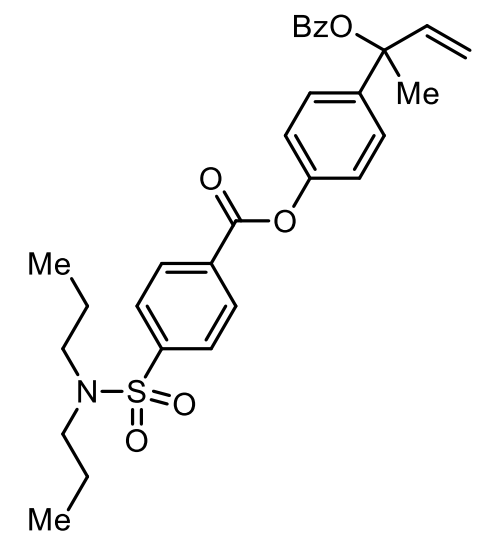

The reaction was performed according to General Procedure A to afford the title compound (250 mg, 28%) as a white solid. **^{1}H NMR** (400 MHz, $CDCl_3$) δ 8.31 (d, J = 8.4 Hz, 2H), 8.10 – 8.05 (m, 2H), 7.95 (d, J = 8.3 Hz, 2H), 7.61 – 7.43 (m, 5H), 7.25 – 7.20 (m, 2H), 6.40 (dd, J = 17.4, 10.8 Hz, 1H), 5.44 (d, J = 17.4 Hz, 1H), 5.35 (d, J = 10.8 Hz, 1H), 3.16 – 3.09 (m, 4H), 2.06 (s, 3H), 1.57 (h, J = 7.4 Hz, 4H), 0.89 (t, J = 7.4 Hz, 6H). **^{13}C NMR** (101 MHz, $CDCl_3$) δ 164.92, 163.87, 149.84, 145.06, 141.88, 141.36, 133.05, 132.93, 131.33, 130.91, 129.71, 128.52, 128.33, 127.30, 126.85, 125.78, 121.39, 115.01, 83.45, 50.09, 25.76, 22.08, 11.29.

2-(4-((2-(4-(2,2-dichlorocyclopropyl)phenoxy)-2-methylpropanoyl)oxy)phenyl)but-3-en-2-yl benzoate (1aj)

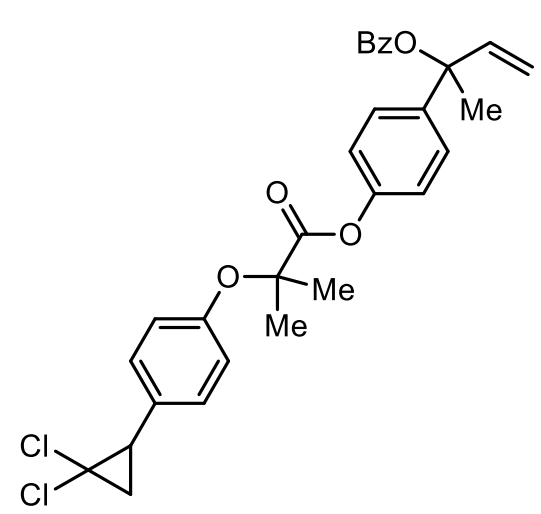

The reaction was performed according to General Procedure A to afford the title compound (331 mg, 25%) as a yellow sticky oil. **^{1}H NMR** (300 MHz, $CDCl_3$) δ 8.14 – 8.02 (m, 2H), 7.69 – 7.38 (m, 5H), 7.26 – 7.08 (m, 3H), 7.04 – 6.89 (m, 3H), 6.48 – 6.31 (m, 1H), 5.52 – 5.25 (m, 2H), 2.94 – 2.78 (m, 1H), 2.03 (s, 3H), 1.99 – 1.90 (m, 1H), 1.77 (s, 7H). **^{13}C NMR** (75 MHz, $CDCl_3$) δ 172.83, 164.89, 155.14, 149.75, 141.71, 141.45, 141.35, 133.74, 133.02, 131.33, 130.30, 129.94, 129.70, 128.71, 128.50, 126.74, 126.69, 121.60, 121.14, 118.71, 114.96, 83.43, 79.46, 60.98, 34.94, 25.96, 25.71, 25.63.

2-(4-((5-(2,5-dimethylphenoxy)-2,2-dimethylpentanoyl)oxy)phenyl)but-3-en-2-yl benzoate (1ak)

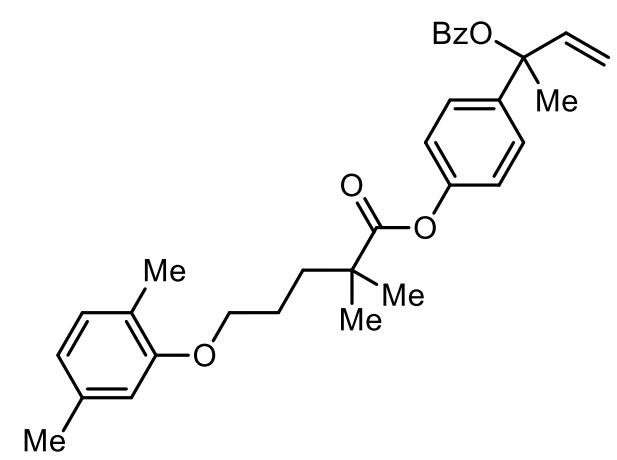

The reaction was performed according to General Procedure A to afford the title compound (502 mg, 61%) as a yellow sticky oil. **^{1}H NMR** (400 MHz, $CDCl_3$) δ 8.13 – 8.04 (m, 2H), 7.64 – 7.54 (m, 1H), 7.52 – 7.38 (m, 4H), 7.12 – 6.97 (m, 3H), 6.73 – 6.58 (m, 2H), 6.39 (dd, *J* = 17.4, 10.8 Hz, 1H), 5.50 – 5.28 (m, 2H), 4.05 – 3.94 (m, 2H), 2.32 (s, 3H), 2.19 (s, 3H), 2.05 (s, 3H), 1.95 – 1.83 (m, 4H), 1.38 (s, 6H). **^{13}C NMR** (101 MHz, $CDCl_3$) δ 176.34, 164.91, 157.00, 150.24, 141.45, 141.20, 136.59, 132.98, 131.41, 130.46, 129.70, 128.48, 126.62, 123.75, 121.37, 120.87, 114.90, 112.08, 83.53, 77.36, 67.91, 42.58, 37.29, 25.70, 25.39, 25.27, 21.52, 15.93.

2-(4-((N-(2-(9H-fluoren-9-yl)acetyl)-N-methyl-D-valyl)oxy)phenyl)but-3-en-2-yl benzoate (1al)

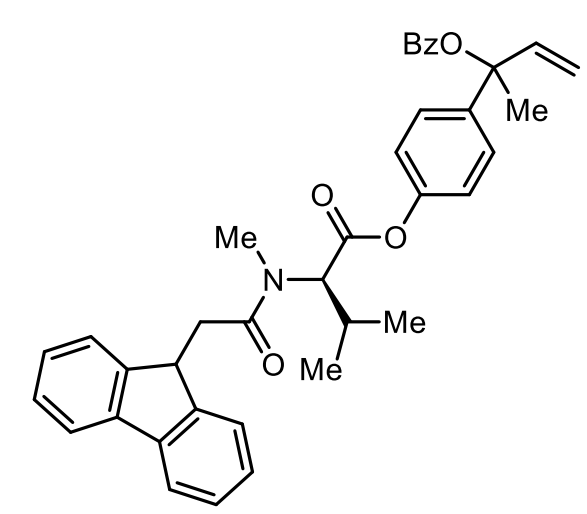

The reaction was performed according to General Procedure A to afford the title compound (200 mg, 26%) as a white foam. **^{1}H NMR** (400 MHz, $CDCl_3$) δ 8.11 – 8.03 (m, 2H), 7.82 – 7.71 (m, 2H), 7.69 – 7.52 (m, 3H), 7.51 – 7.27 (m, 8H), 7.13 – 6.98 (m, 2H), 6.44 – 6.32 (m, 1H), 5.41 (dd, *J* = 17.4, 5.6 Hz, 1H), 5.33 (dd, *J* = 10.8, 4.6 Hz, 1H), 4.77 – 4.21 (m, 4H), 2.97 (d, *J* = 3.6 Hz, 3H), 2.44 – 2.14 (m, 1H), 2.08 – 1.97 (m, 3H), 1.07 (dd, *J* = 49.8, 6.5 Hz, 3H), 0.88 (dd, *J* = 64.5, 6.7 Hz, 3H). **^{13}C NMR** (101 MHz, $CDCl_3$) δ 169.85, 169.11, 164.90, 157.57, 149.63, 144.01, 141.50, 141.38, 133.02, 131.36, 129.70, 128.50, 127.83, 127.20, 126.68, 125.08, 124.97, 121.32, 120.10, 114.98, 83.46, 67.83, 64.30, 47.45, 30.68, 30.30, 28.08, 27.76, 25.71, 19.89, 19.14, 18.88.

Optimization Studies:

In a glovebox, to an oven-dried 4 mL screw cap vial was added **Co-X** (1.00 μmol, 2.00 mol%), photocatalyst (0.500 μmol, 1.00 mol%), **HX-X** (5.00 μmol, 10.0 mol%), **1a** (12.6 mg, 0.0500 mmol, 1.00 equiv.), **2a** (8.04 mg, 0.0750 mmol, 1.50 equiv.) in varied solvent (2.00 ml, 0.100 M). The vial was equipped with a stir bar,

capped and sealed with electrical tape, then taken out of the glovebox. The reaction mixture was stirred at varied temperatures and irradiated with 40 W Blue LED for 18 h. The reaction mixture was then concentrated *in vacuo* and their yields were determined using ^{1}H NMR and dibromomethane as the internal standard.

Screening for cobalt catalyst

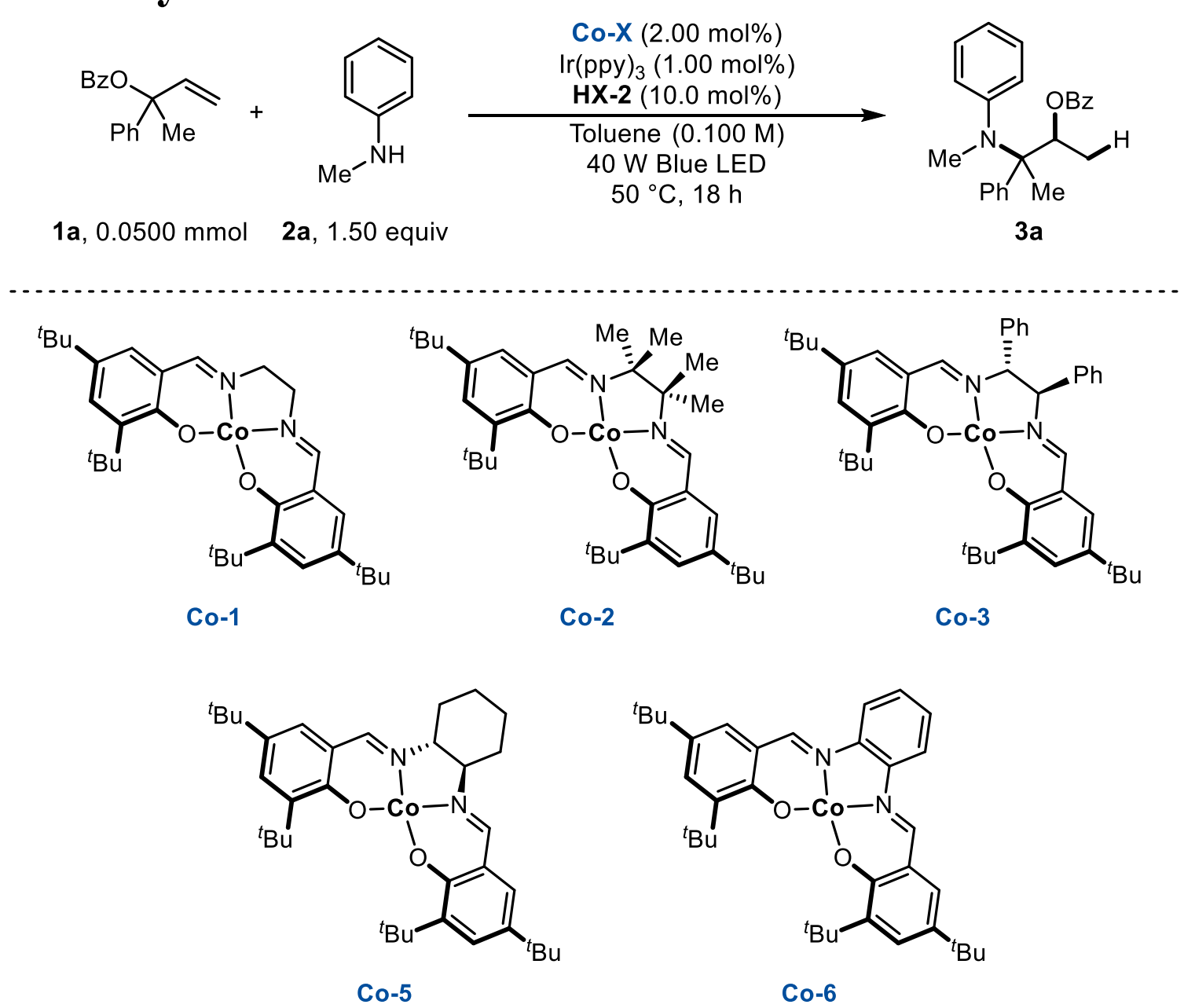

Entry	Co-X	Yield (%)
1	Co-1	46
2	Co-2	12
3	Co-3	18
4	Co-5	30
5	Co-6	<5

Table S1. Screening for cobalt catalyst

Screening for solvent

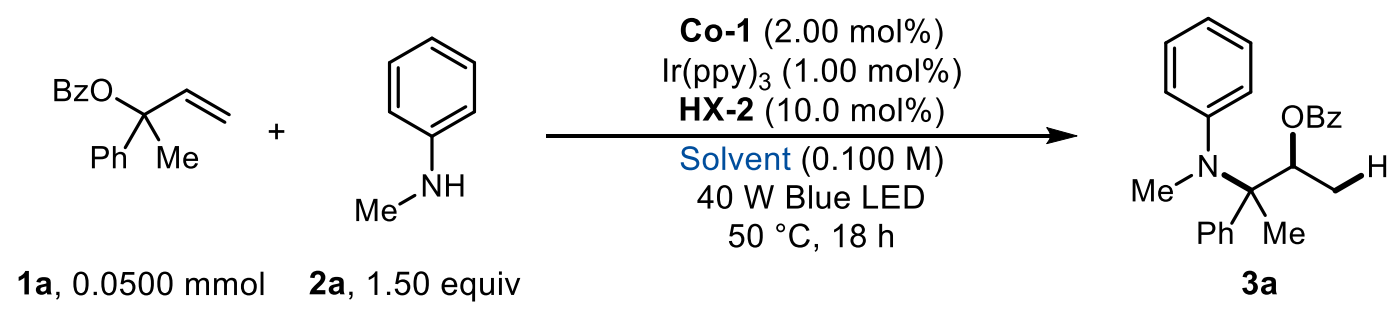

Entry	Solvent	Yield (%)
1	Toluene	46
2	THF	74
3	DME	33
4	EtOAc	47
5	DCE	51
6	Benzene	50
7	MeCN	23
8	Dioxane	23

Table S2. Screening for solvent

Screening for temperature

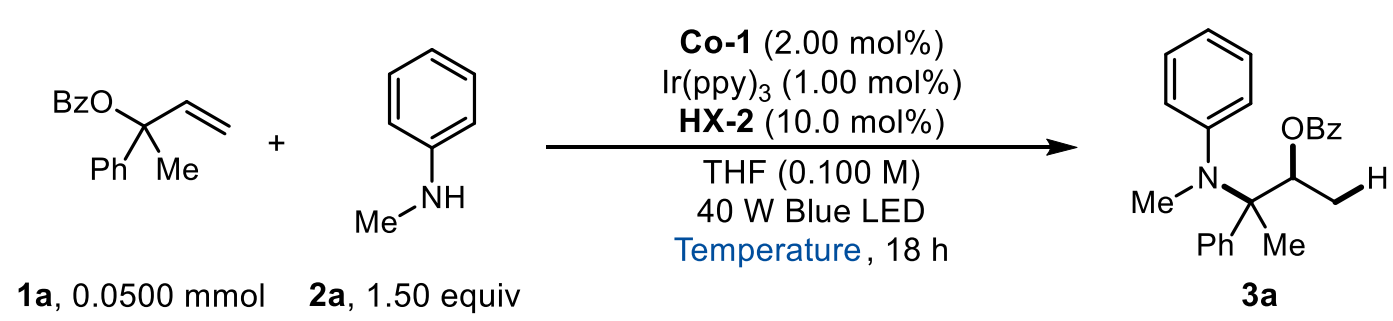

Entry	Temperature	Yield (%)
1	RT	42
2	40	75
3	50	74
4	60	78
5	80	78

Table S3. Screening for temperature

Screening for salt

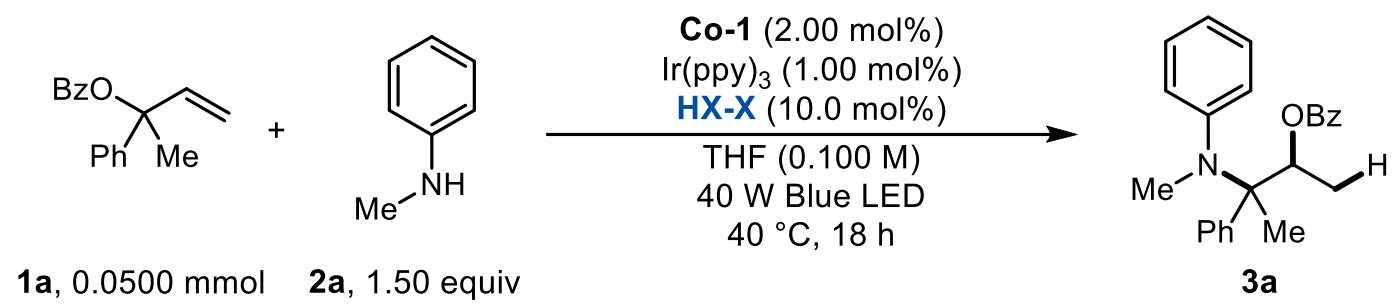

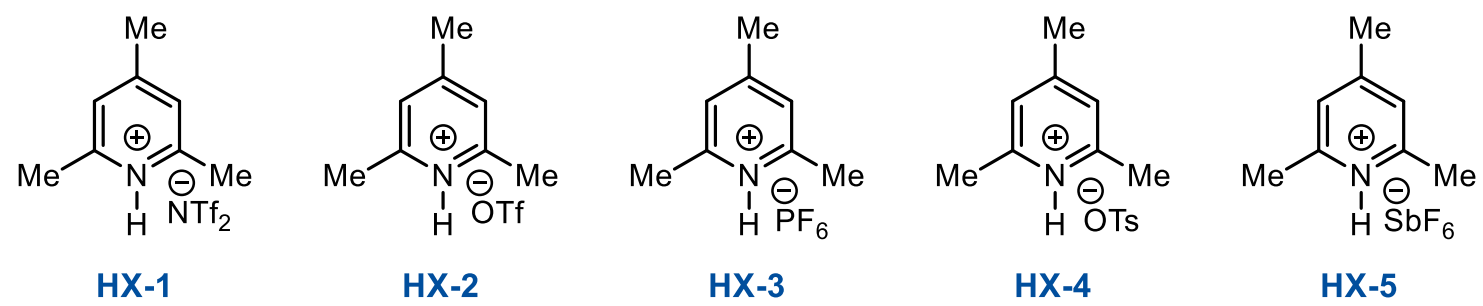

Entry	HX-X	Yield (%)
1	HX-1	84
2	HX-2	75
3	HX-3	73
4	HX-4	77
5	HX-5	79

Table S4. Screening for salt

Screening for photocatalyst

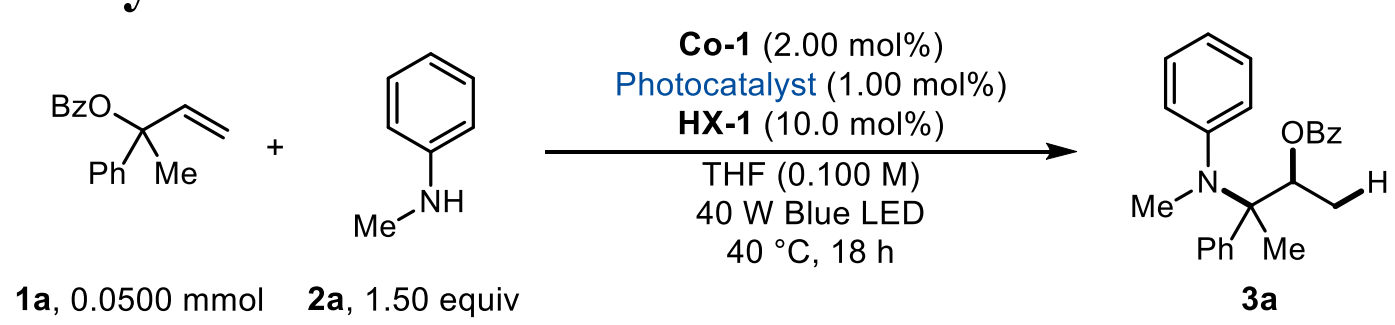

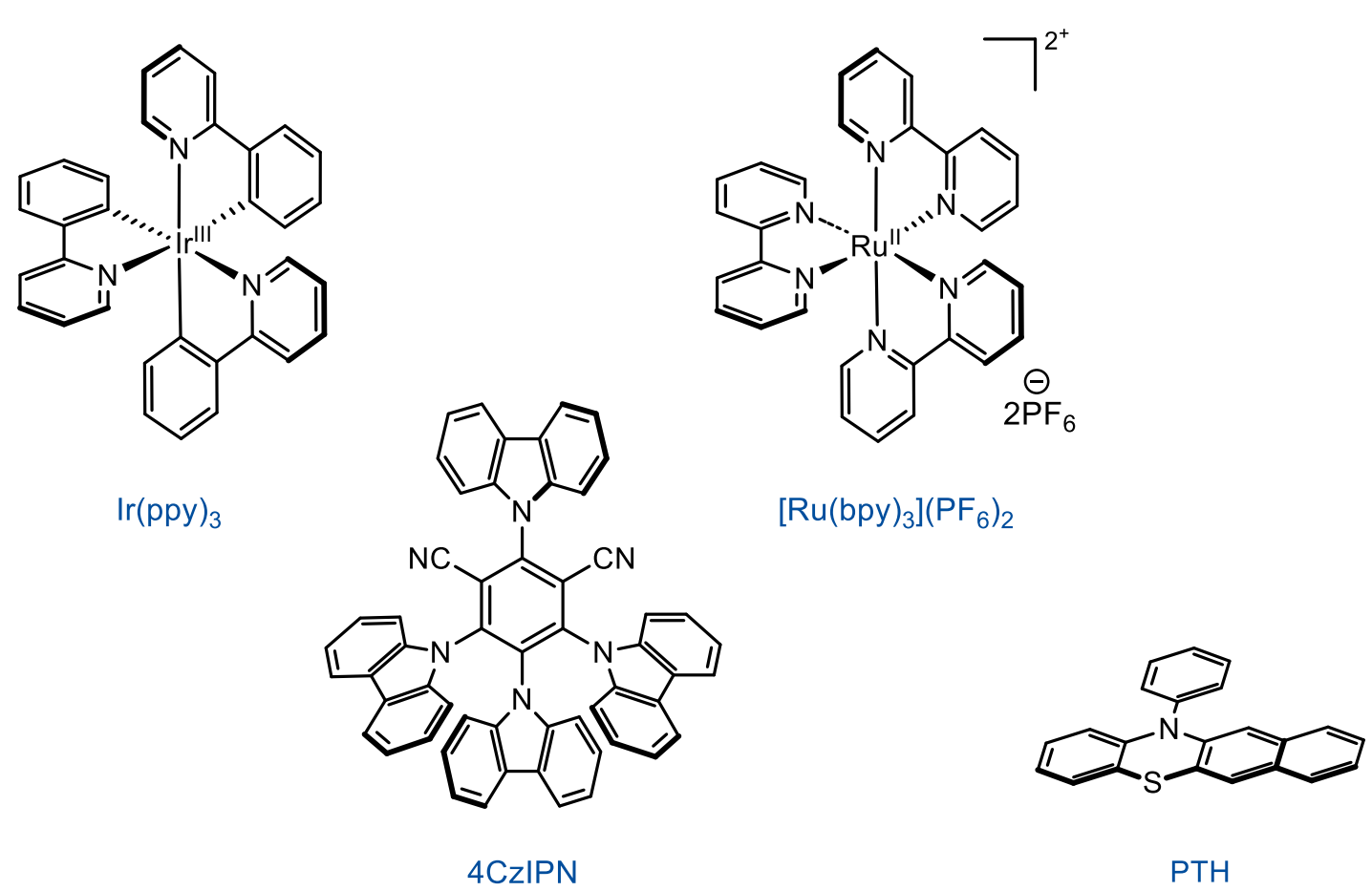

Entry	Photocatalyst	Yield (%)
1	$Ir(ppy)_3$	84
2	$[Ru(bpy)_3](PF_6)_2$	<2
3	4CzIPN	<2
4	PTH	<2

Table S5. Screening for photocatalyst

General Procedure B (for photoredox/cobalt/Brønsted acid-catalyzed hydroamination of ally carboxylates):

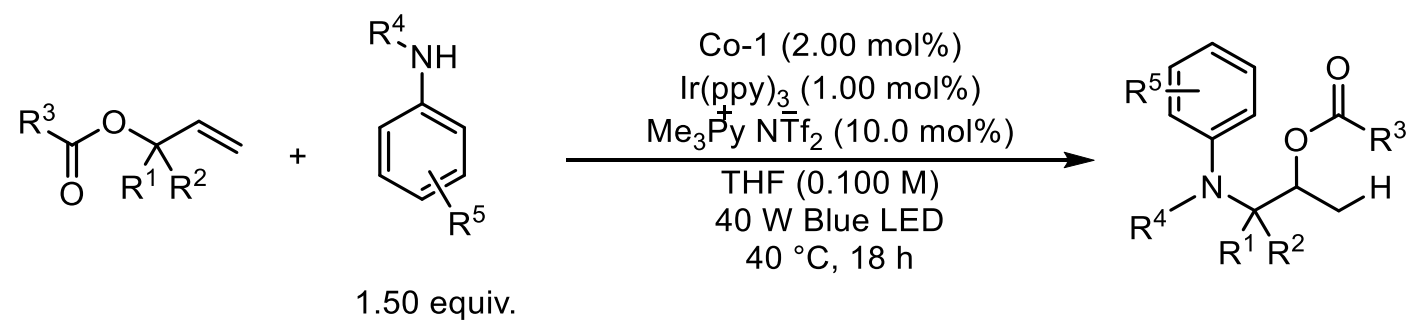

In a glovebox, to an oven-dried 4 mL screw cap vial was added **Co-1** (2.20 mg, 4.00 μmol, 2.00 mol%), $Ir(ppy)_3$ (1.31 mg, 2.00 μmol, 1.00 mol%), 2,4,6-trimethylpyridin-1-ium bis((trifluoromethyl)sulfonyl)amide (8.04 mg, 20.0 μmol, 10.0 mol%), corresponding allyl ester (0.200 mmol) and aniline (0.300 mmol, 1.50 equiv.) in THF (2.00 ml, 0.100 M). The vial was equipped with a stir bar, capped and sealed with electrical tape, then taken out of the glovebox. The reaction mixture was stirred at 40–80 °C and irradiated with 40 W Blue LED for 18 h. The reaction mixture was then concentrated *in vacuo* and residue was purified by flash column chromatography on silica gel to afford the desired product.

Additional Substrates

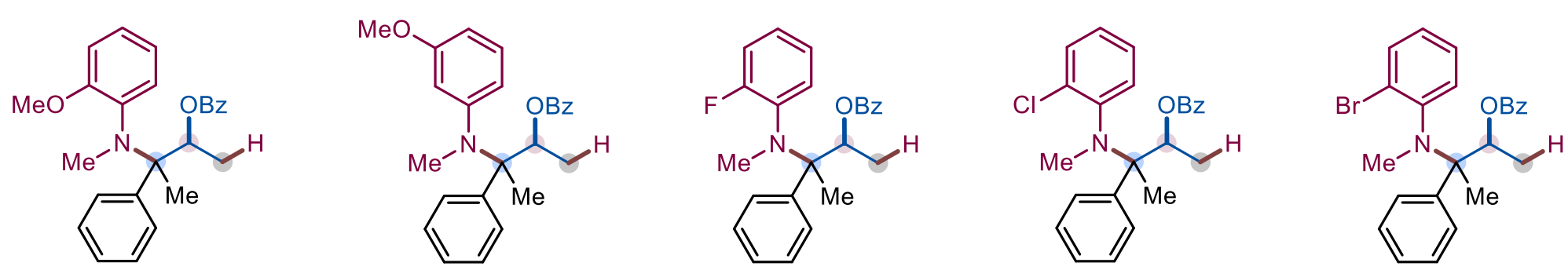

3aaw, 40% **3aax**, 43% **3aay**, 69% **3aaz**, 31% **3aaaa**, 41%

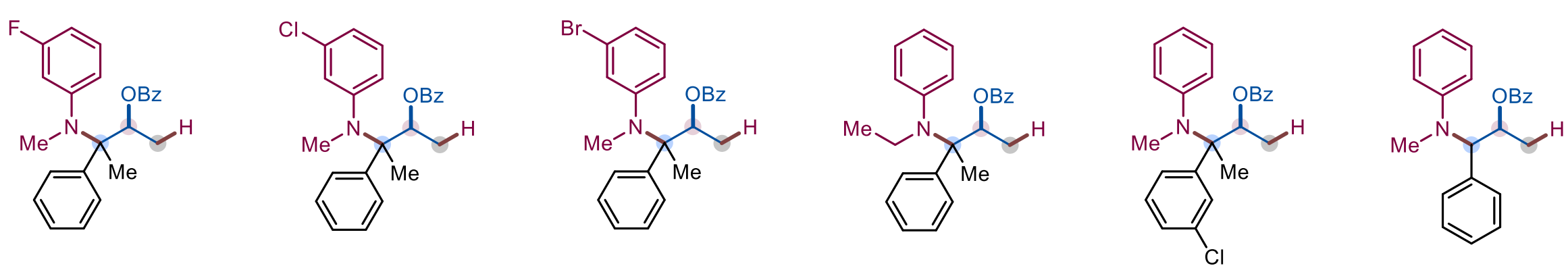

3aaab, 68% **3aaac**, 56% **3aaad**, 77% **3aaae**, 36% **3aaaf**, 50% **3aaag**, 72%

Figure S1. Additional substrates

Unsuccessful Substrates

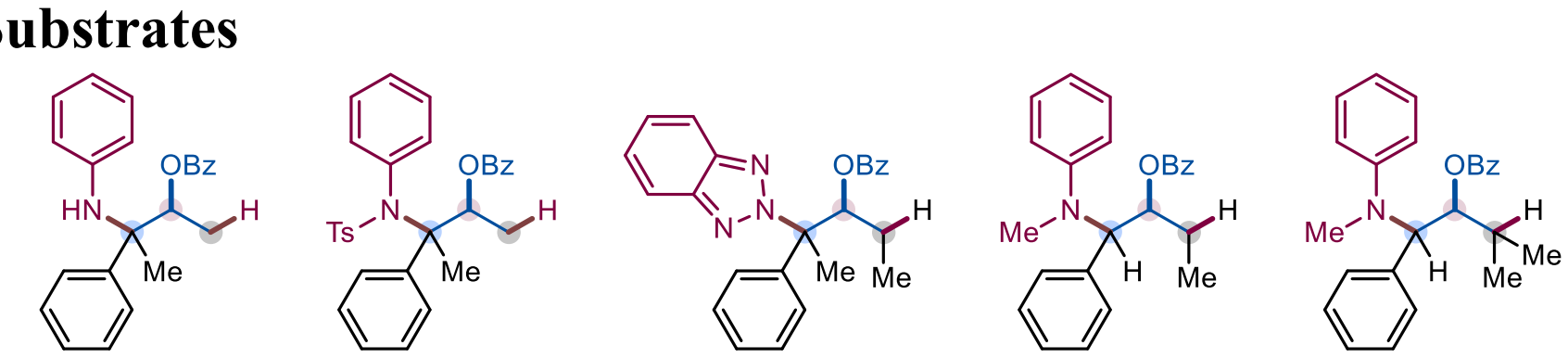

N.D N.D. N.D. 20% N.D.

Figure S2. Unsuccessful substrates

3-(methyl(phenyl)amino)-3-phenylbutan-2-yl benzoate (3a)

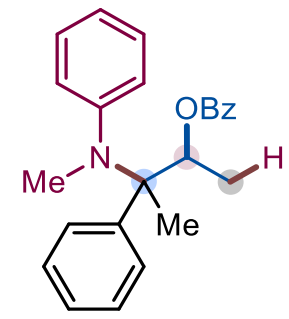

The reaction was performed according to General Procedure B at 40°C to afford the title compound (68.3 mg, 95% yield) as a colorless oil. **R*f*** = 0.5 [Hex: EtOAc 5:1 (v/v)]. *Major diastereomer*: **^{1}H NMR** (500 MHz, Chloroform-*d*) δ 7.89 – 7.84 (m, 2H), 7.51 – 7.43 (m, 1H), 7.43 – 7.38 (m, 2H), 7.38 – 7.29 (m, 3H), 7.28 – 7.22 (m, 2H), 7.19 – 7.14 (m, 1H), 7.13 – 7.06 (m, 1H), 7.05 – 6.89 (m, 1H), 6.87 – 6.82 (m, 2H), 5.75 – 5.66 (m, 1H), 2.68 (s, 3H), 1.63 (s, 3H), 1.30 (d, *J* = 6.3 Hz, 3H). *Mixture of diastereomers*: **^{13}C NMR** (201 MHz, $CDCl_3$) δ 165.97, 150.94, 150.06, 143.23, 142.13, 132.82, 132.67, 130.71, 129.54, 129.49, 128.31, 128.19, 128.15, 128.07, 128.02, 127.79, 127.71, 126.94, 126.72, 126.58, 125.81, 123.18, 122.79, 74.89, 73.43, 66.56, 65.84, 38.60, 37.96, 19.37, 16.79, 15.70. **HRMS** (ESI-TOF) *m/z* calcd for $C_{24}H_{26}NO_2$ [(M + H)$^+$] 360.1964, found 360.1972.

3-(methyl(*o*-tolyl)amino)-3-phenylbutan-2-yl benzoate (3b)

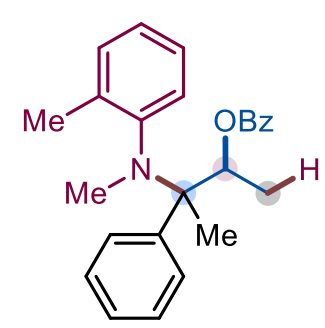

The reaction was performed according to General Procedure B at 40°C to afford the title compound (55.4 mg, 74% yield) as a colorless sticky oil. **R*f*** = 0.4 [Hex: EtOAc 20:1 (v/v)]. *Major diastereomer*: **^{1}H NMR** (300 MHz, $CDCl_3$) δ 7.92 – 7.80 (m, 2H), 7.50 – 7.38 (m, 1H), 7.39 – 7.22 (m, 2H), 7.23 – 7.11 (m, 5H), 7.13 – 7.03 (m, 2H), 6.99 – 6.90 (m, 1H), 6.51 – 6.40 (m, 1H), 5.96 – 5.81 (m, 1H), 2.80 (s, 3H), 2.00 (s, 3H), 1.69 (s, 3H), 1.13 (d, *J* = 6.3 Hz, 3H). *Mixture of diastereomers*: **^{13}C NMR** (201 MHz, $CDCl_3$) δ 166.28, 149.64, 147.50, 145.33, 133.96, 132.72, 130.83, 130.51, 129.59, 129.54, 128.95, 128.25, 128.06, 127.96, 127.91, 127.85, 127.79, 127.11, 126.84, 126.23, 125.80, 125.75, 125.69, 125.64, 121.28, 108.48, 62.95, 50.11, 49.73, 44.25, 40.17, 30.83, 28.25, 25.17, 23.63, 17.67, 17.65, 16.15, 16.09. **HRMS** (ESI-TOF) *m/z* calcd for $C_{25}H_{28}NO_2$ [(M + H)$^+$] 374.2120, found 374.2110.

3-(methyl(m-tolyl)amino)-3-phenylbutan-2-yl benzoate (3c)

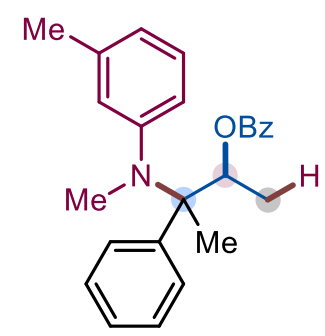

The reaction was performed according to General Procedure B at 40°C to afford the title compound (47.2 mg, 63% yield) as a colorless sticky oil. $\mathbf{R_f}$ = 0.4 [Hex: EtOAc 20:1 (v/v)]. *Major diastereomer*: **^{1}H NMR** (300 MHz, $CDCl_3$) δ 7.89 – 7.82 (m, 2H), 7.53 – 7.38 (m, 3H), 7.38 – 7.10 (m, 5H), 7.02 – 6.91 (m, 1H), 6.90 – 6.59 (m, 3H), 5.57 (q, *J* = 6.3 Hz, 1H), 2.60 (s, 3H), 2.11 (s, 3H), 1.59 (s, 3H), 1.24 (d, *J* = 6.3 Hz, 3H). *Mixture of diastereomers*: **^{13}C NMR** (126 MHz, $CDCl_3$) δ 165.97, 165.50, 150.89, 149.97, 143.71, 142.19, 137.88, 132.79, 132.58, 130.79, 130.56, 129.57, 129.41, 128.31, 128.06, 127.99, 127.95, 127.92, 127.71, 127.65, 127.30, 126.96, 126.73, 124.40, 124.00, 123.81, 123.18, 75.21, 73.52, 66.74, 65.97, 38.59, 38.10, 21.42, 21.26, 19.03, 16.96, 15.80. **HRMS** (ESI-TOF) *m/z* calcd for $C_{25}H_{28}NO_2$ [$(M + H)^+$] 374.2120, found 374.2113.

3-(methyl(p-tolyl)amino)-3-phenylbutan-2-yl benzoate (3d)

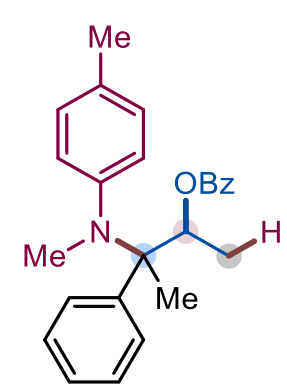

The reaction was performed according to General Procedure B at 40°C to afford the title compound (62.8 mg, 84% yield) as a colorless sticky oil. $\mathbf{R_f}$ = 0.4 [Hex: EtOAc 20:1 (v/v)]. *Major diastereomer*: **^{1}H NMR** (400 MHz, $CDCl_3$) δ 7.94 – 7.87 (m, 2H), 7.56 – 7.44 (m, 2H), 7.43 – 7.18 (m, 6H), 6.99 (t, *J* = 8.1 Hz, 2H), 6.84 (t, *J* = 8.6 Hz, 2H), 5.69 (q, *J* = 6.3 Hz, 1H), 2.64 (s, 3H), 2.28 (s, 3H), 1.64 (s, 3H), 1.36 (d, *J* = 6.2 Hz, 3H). *Mixture of diastereomers*: **^{13}C NMR** (201 MHz, $CDCl_3$) δ 166.01, 165.56, 148.39, 147.47, 143.54, 141.81, 133.60, 133.05, 132.76, 132.54, 130.79, 130.62, 129.54, 129.43, 128.95, 128.83, 128.29, 128.04, 127.95, 127.75, 127.62, 127.21, 126.96, 126.73, 126.70, 75.26, 73.32, 66.68, 65.77, 38.75, 38.16, 20.79, 20.52, 18.86, 16.96, 15.67, 15.30, 1.05. **HRMS** (ESI-TOF) *m/z* calcd for $C_{25}H_{28}NO_2$ [$(M + H)^+$] 374.2120, found 374.2113.

3-((4-(tert-butyl)phenyl)(methyl)amino)-3-phenylbutan-2-yl benzoate (3e)

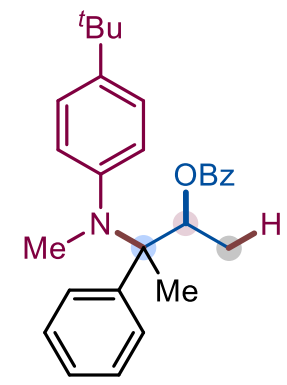

The reaction was performed according to General Procedure B at 40°C to afford the title compound (73.2 mg, 88% yield) as a yellow sticky oil. **R*f*** = 0.4 [Hex: EtOAc 20:1 (v/v)]. *Major diastereomers*: **^{1}H NMR** (300 MHz, $CDCl_3$) δ 7.97 – 7.88 (m, 2H), 7.55 – 7.44 (m, 3H), 7.43 – 7.13 (m, 5H), 7.08 (s, 2H), 6.90 – 6.80 (m, 2H), 5.79 – 5.64 (m, 1H), 2.68 (s, 3H), 1.66 (s, 3H), 1.35 (d, *J* = 6.2 Hz, 3H), 1.28 (s, 9H). *Mixture of diastereomers*: **^{13}C NMR** (126 MHz, $CDCl_3$) δ 166.10, 165.50, 148.35, 147.43, 146.49, 145.86, 144.15, 142.52, 132.88, 132.69, 130.94, 130.70, 129.67, 129.57, 128.41, 128.22, 128.14, 127.98, 127.83, 127.79, 127.07, 126.76, 125.75, 125.23, 125.10, 75.58, 73.59, 66.93, 65.90, 38.78, 38.18, 34.29, 34.08, 31.58, 31.27, 19.43, 17.17, 15.85, 15.26. **HRMS** (ESI-TOF) *m/z* calcd for $C_{28}H_{34}NO_2$ [$(M + H)^+$] 416.2590, found 416.2582.

3-((4-methoxyphenyl)(methyl)amino)-3-phenylbutan-2-yl benzoate (3f)

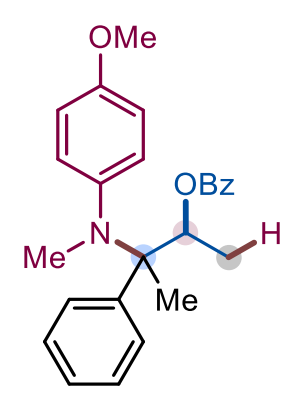

The reaction was performed according to General Procedure B at 40°C to afford the title compound (47.6 mg, 61% yield) as a yellow sticky oil. **R*f*** = 0.4 [Hex: EtOAc 20:1 (v/v)]. *Major diastereomer*: **^{1}H NMR** (500 MHz, $CDCl_3$) δ 7.81 (d, *J* = 6.9 Hz, 2H), 7.47 – 7.40 (m, 3H), 7.34 – 7.28 (m, 3H), 7.27 – 7.20 (m, 2H), 6.82 (d, *J* = 8.9 Hz, 2H), 6.65 (d, *J* = 8.9 Hz, 2H), 5.57 (q, *J* = 6.3 Hz, 1H), 3.70 (s, 3H), 2.49 (s, 3H), 1.53 (s, 3H), 1.29 (d, *J* = 6.3 Hz, 3H). *Mixture of diastereomers*: **^{13}C NMR** (126 MHz, $CDCl_3$) δ 166.12, 165.67, 156.67, 156.60, 144.04, 143.44, 143.13, 141.32, 132.83, 132.67, 130.96, 130.79, 129.63, 129.54, 129.22, 129.09, 128.38, 128.21, 128.15, 128.09, 127.89, 127.66, 127.12, 126.87, 113.71, 113.52, 75.27, 73.22, 66.78, 65.89, 55.48, 55.24, 39.12, 38.63, 18.49, 17.17, 15.72, 14.97. **HRMS** (ESI-TOF) *m/z* calcd for $C_{25}H_{28}NO_3$ [$(M + H)^+$] 390.2069, found 390.2069.

3-((4-fluorophenyl)(methyl)amino)-3-phenylbutan-2-yl benzoate (3g)

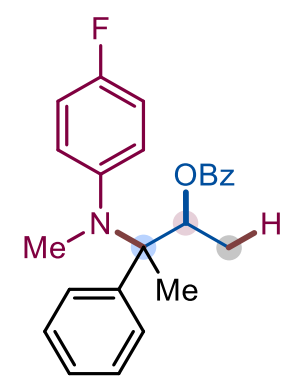

The reaction was performed according to General Procedure B at 40°C to afford the title compound (65.6 mg, 87% yield) as a colorless sticky oil. **R*f*** = 0.4 [Hex: EtOAc 20:1 (v/v)]. *Major diastereomer*: **^{1}H NMR** (500 MHz, $CDCl_3$) δ 7.92 – 7.86 (m, 2H), 7.56 – 7.48 (m, 3H), 7.43 – 7.35 (m, 5H), 6.92 – 6.81 (m, 4H), 5.67 (q, *J* = 6.2 Hz, 1H), 2.61 (s, 3H), 1.62 (s, 3H), 1.36 (d, *J* = 6.2 Hz, 3H). *Mixture of diastereomers*: **^{13}C NMR** (126 MHz, $CDCl_3$) δ 165.96, 165.59, 160.55, 158.59, 146.93, 146.05, 142.87, 141.08, 132.83, 132.78, 130.70, 130.53, 129.51, 129.40, 129.01, 128.94, 128.83, 128.77, 128.32, 128.12, 127.97, 127.68, 127.14, 126.91, 114.94, 114.90, 114.76, 114.73, 74.72, 73.02, 66.61, 65.79, 38.90, 38.41, 18.67, 16.97, 15.63. *Major diastereomer*: **^{19}F NMR** (470 MHz, Chloroform-*d*) δ -118.89 – -119.04 (m). **HRMS** (ESI-TOF) *m/z* calcd for $C_{24}H_{25}NO_2F$ [$(M + H)^+$] 378.1869, found 378.1865.

3-((4-chlorophenyl)(methyl)amino)-3-phenylbutan-2-yl benzoate (3h)

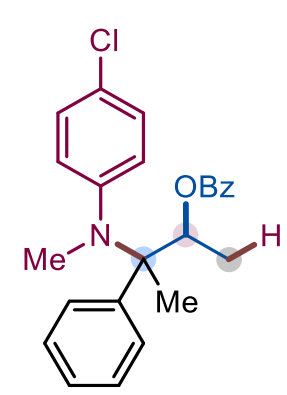

The reaction was performed according to General Procedure B at 40°C to afford the title compound (61.4 mg, 78% yield) as a colorless sticky oil. **R*f*** = 0.4 [Hex: EtOAc 20:1 (v/v)]. *Major diastereomer*: **^{1}H NMR** (400 MHz, $CDCl_3$) δ 7.84 (d, *J* = 7.0 Hz, 2H), 7.58 – 7.46 (m, 2H), 7.45 – 7.33 (m, 4H), 7.34 – 7.19 (m, 2H), 6.99 (d, *J* = 8.8 Hz, 2H), 6.93 (d, *J* = 8.9 Hz, 2H), 5.80 – 5.69 (m, 1H), 2.66 (s, 3H), 1.73 (s, 3H), 1.06 (d, *J* = 6.4 Hz, 3H). *Mixture of diastereomers*: **^{13}C NMR** (201 MHz, $CDCl_3$) δ 165.94, 165.62, 149.54, 148.63, 142.95, 141.69, 132.93, 132.85, 130.57, 130.40, 129.54, 129.39, 128.57, 128.37, 128.28, 128.23, 128.17, 128.15, 127.85, 127.70, 127.67, 127.57, 127.15, 126.92, 126.54, 74.84, 73.32, 66.69, 65.91, 38.50, 37.88, 19.40, 16.86, 16.25, 15.70. **HRMS** (ESI-TOF) *m/z* calcd for $C_{24}H_{25}NO_2Cl$ [$(M + H)^+$] 394.1574, found 394.1569.

3-((4-bromophenyl)(methyl)amino)-3-phenylbutan-2-yl benzoate (3i)

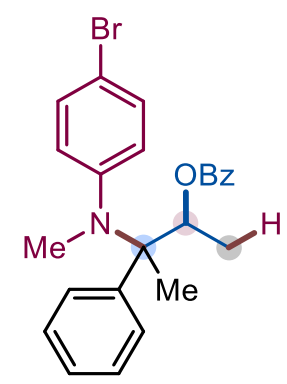

The reaction was performed according to General Procedure B at 40°C to afford the title compound (73.6 mg, 84% yield) as a yellow sticky oil. **R*f*** = 0.4 [Hex: EtOAc 20:1 (v/v)]. *Major diastereomer*: **^{1}H NMR** (500 MHz, $CDCl_3$) δ 7.87 – 7.80 (m, 2H), 7.57 – 7.50 (m, 1H), 7.52 – 7.46 (m, 2H), 7.44 – 7.34 (m, 4H), 7.33 – 7.26 (m, 1H), 7.17 – 7.10 (m, 2H), 6.90 – 6.83 (m, 2H), 5.80 – 5.70 (m, 1H), 2.67 (s, 3H), 1.73 (s, 3H), 1.06 (d, *J* = 6.3 Hz, 3H). *Mixture of diastereomers*: **^{13}C NMR** (126 MHz, $CDCl_3$) δ 165.95, 165.63, 150.05, 149.13, 142.99, 141.83, 132.96, 132.89, 131.20, 131.09, 130.57, 130.40, 129.56, 129.42, 128.39, 128.22, 128.20, 127.89, 127.66, 127.57, 127.17, 126.94, 126.56, 116.20, 115.11, 74.88, 73.41, 66.70, 65.94, 38.43, 37.78, 19.56, 16.85, 16.41, 15.74. **HRMS** (ESI-TOF) *m/z* calcd for $C_{24}H_{25}NO_2Br$ [$(M + H)^+$] 438.1069, found 438.1053.

3-(methyl(4-(trifluoromethyl)phenyl)amino)-3-phenylbutan-2-yl benzoate (3j)

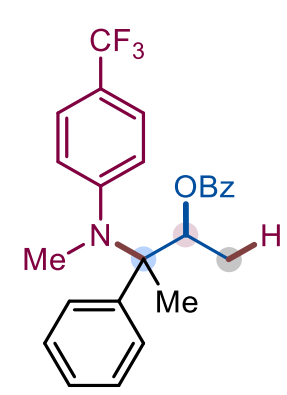

The reaction was performed according to General Procedure B at 40°C to afford the title compound (76.0 mg, 89% yield) as a colorless sticky oil. **R*f*** = 0.4 [Hex: EtOAc 20:1 (v/v)]. *Major diastereomer*: **^{1}H NMR** (500 MHz, $CDCl_3$) δ 7.88 – 7.82 (m, 2H), 7.60 – 7.50 (m, 1H), 7.49 – 7.42 (m, 2H), 7.43 – 7.33 (m, 4H), 7.34 – 7.21 (m, 3H), 6.94 (d, *J* = 8.5 Hz, 2H), 5.80 (q, *J* = 6.3 Hz, 1H), 2.93 (s, 3H), 1.81 (s, 3H), 1.15 (d, *J* = 6.3 Hz, 3H). *Mixture of diastereomers*: **^{13}C NMR** (126 MHz, $CDCl_3$) δ 165.88, 165.64, 153.64, 152.54, 143.19, 142.89, 133.15, 133.04, 130.42, 130.21, 129.61, 129.45, 128.49, 128.39, 128.29, 128.24, 127.34, 127.30, 127.25, 127.04, 125.56, 125.24, 125.21, 125.18, 125.15, 123.41, 122.97, 122.69, 122.43, 120.41, 111.45, 75.13, 74.08, 66.96, 66.42, 37.98, 37.22, 20.70, 17.95, 16.71, 15.96. *Major diastereomer*: **^{19}F NMR** (282 MHz, $CDCl_3$) δ -61.78. **HRMS** (ESI-TOF) *m/z* calcd for $C_{25}H_{25}NO_2F_3$ [$(M + H)^+$] 428.1837, found 428.1840.

3-(methyl(4-(trifluoromethoxy)phenyl)amino)-3-phenylbutan-2-yl benzoate (3k)

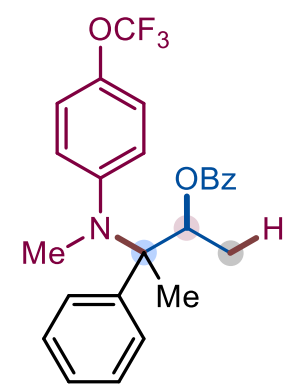

The reaction was performed according to General Procedure B at 40°C to afford the title compound (71.8 mg, 81% yield) as a colorless sticky oil. **R$_f$** = 0.4 [Hex: EtOAc 20:1 (v/v)]. *Major diastereomer*: **^{1}H NMR** (400 MHz, $CDCl_3$) δ 7.88 – 7.81 (m, 2H), 7.56 – 7.46 (m, 2H), 7.46 – 7.34 (m, 4H), 7.34 – 7.20 (m, 2H), 7.03 – 6.94 (m, 2H), 6.92 – 6.81 (m, 2H), 5.77 (p, *J* = 6.5 Hz, 1H), 2.68 (s, 3H), 1.74 (s, 3H), 1.06 (d, *J* = 6.3 Hz, 3H). *Mixture of diastereomers*: **^{13}C NMR** (201 MHz, $CDCl_3$) δ 165.92, 165.57, 149.53, 148.69, 144.70, 144.20, 142.99, 141.74, 132.94, 132.87, 130.55, 130.34, 129.53, 129.37, 128.37, 128.20, 127.88, 127.69, 127.53, 127.26, 127.18, 126.95, 126.12, 121.17, 120.99, 120.72, 120.64, 119.90, 119.71, 74.74, 73.30, 66.69, 65.92, 38.56, 37.99, 19.47, 16.87, 16.51, 15.73. *Major diastereomer*: **^{19}F NMR** (376 MHz, $CDCl_3$) δ -59.69. **HRMS** (ESI-TOF) *m/z* calcd for $C_{25}H_{25}NO_3F_3$ $[(M + H)^+]$ 444.1787, found 444.1779.

3-(methyl(perfluorophenyl)amino)-3-phenylbutan-2-yl benzoate (3l)

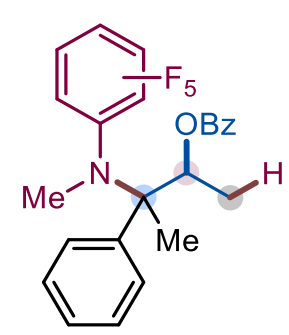

The reaction was performed according to General Procedure B at 40°C to afford the title compound (55.7 mg, 62% yield) as a yellow sticky oil. **R$_f$** = 0.4 [Hex: EtOAc 20:1 (v/v)]. *Major diastereomer*: **^{1}H NMR** (400 MHz, $CDCl_3$) δ 7.83 – 7.77 (m, 2H), 7.76 – 7.70 (m, 2H), 7.56 (t, *J* = 7.3 Hz, 1H), 7.42 (t, *J* = 7.5 Hz, 4H), 7.33 (t, *J* = 7.2 Hz, 1H), 5.47 (q, *J* = 6.4 Hz, 1H), 2.50 (s, 3H), 1.91 (s, 3H), 0.86 (d, *J* = 6.3 Hz, 3H). *Major diastereomer*: **^{13}C NMR** (126 MHz, $CDCl_3$) δ 165.08, 143.73, 133.27, 129.67, 128.98, 128.72, 128.51, 127.81, 127.65, 75.93, 68.58, 37.27, 17.39, 14.60. *Major diastereomer*: **^{19}F NMR** (376 MHz, $CDCl_3$) δ -142.47 (d, *J* = 22.3 Hz), -144.92 (dd, *J* = 24.7, 6.4 Hz), -160.53 (t, *J* = 21.5 Hz), -163.96 – -165.29 (m), -165.55 – -166.39 (m). **HRMS** (ESI-TOF) *m/z* calcd for $C_{24}H_{20}NO_2F_5Na$ $[(M + Na)^+]$ 472.1312, found 472.1312.

3-((4-cyanophenyl)(methyl)amino)-3-phenylbutan-2-yl benzoate (3m)

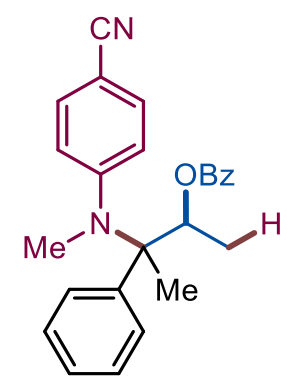

The reaction was performed according to General Procedure B at 40°C to afford the title compound (50.7 mg, 66% yield) as a white solid. **R***f* = 0.3 [Hex: EtOAc 5:1 (v/v)]. *Major diastereomer*: **^{1}H NMR** (400 MHz, $CDCl_3$) δ 7.94 – 7.87 (m, 2H), 7.61 – 7.53 (m, 1H), 7.43 (t, *J* = 7.6 Hz, 2H), 7.41 – 7.30 (m, 4H), 7.32 – 7.20 (m, 3H), 6.74 (d, *J* = 8.8 Hz, 2H), 5.79 (q, *J* = 6.3 Hz, 1H), 3.11 (s, 3H), 1.84 (s, 3H), 1.21 (d, *J* = 6.3 Hz, 3H). *Major diastereomers*: **^{13}C NMR** (201 MHz, $CDCl_3$) δ 165.64, 153.75, 142.92, 133.27, 132.08, 130.07, 129.54, 128.58, 128.45, 127.41, 127.04, 120.42, 119.89, 101.15, 75.09, 67.16, 37.46, 19.36, 16.62. **HRMS** (ESI-TOF) *m/z* calcd for $C_{25}H_{24}N_2O_2Na$ [$(M + Na)^+$] 407.1735, found 407.1732.

ethyl 4-((3-(benzoyloxy)-2-phenylbutan-2-yl)(methyl)amino)benzoate (3n)

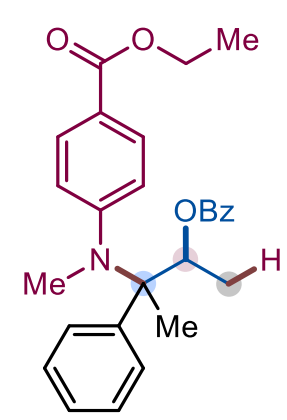

The reaction was performed according to General Procedure B at 40°C to afford the title compound (76.0 mg, 88% yield) as a white foam. **R***f* = 0.3 [Hex: EtOAc 20:1 (v/v)]. *Major diastereomer*: **^{1}H NMR** (300 MHz, $CDCl_3$) δ 7.94 – 7.85 (m, 2H), 7.76 – 7.65 (m, 2H), 7.61 – 7.48 (m, 1H), 7.47 – 7.19 (m, 7H), 6.86 – 6.75 (m, 2H), 5.80 (q, *J* = 6.3 Hz, 1H), 4.26 (q, *J* = 7.2 Hz, 2H), 3.04 (s, 3H), 1.83 (s, 3H), 1.32 (t, *J* = 7.1 Hz, 3H), 1.20 (d, *J* = 6.3 Hz, 3H). *Major diastereomer*: **^{13}C NMR** (126 MHz, $CDCl_3$) δ 166.68, 165.82, 154.66, 143.27, 133.14, 130.37, 129.85, 129.74, 129.66, 128.62, 128.46, 128.43, 128.36, 127.42, 127.28, 121.57, 121.16, 75.33, 67.09, 60.40, 37.87, 18.87, 16.71, 14.51. **HRMS** (ESI-TOF) *m/z* calcd for $C_{27}H_{29}NO_4Na$ [$(M + Na)^+$] 454.1994, found 454.1991.

3-((4-acetylphenyl)(methyl)amino)-3-phenylbutan-2-yl benzoate (3o)

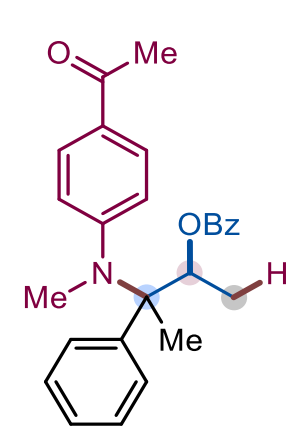

The reaction was performed according to General Procedure B at 40°C to afford the title compound (65.0 mg, 81% yield) as a colorless sticky oil. **R*f*** = 0.4 [Hex: EtOAc 20:1 (v/v)]. *Major diastereomer*: **^{1}H NMR** (400 MHz, $CDCl_3$) δ 7.92 – 7.85 (m, 2H), 7.67 – 7.59 (m, 2H), 7.54 (ddt, *J* = 7.9, 7.0, 1.3 Hz, 1H), 7.47 – 7.32 (m, 6H), 7.31 – 7.26 (m, 1H), 6.84 – 6.76 (m, 2H), 5.79 (q, *J* = 6.3 Hz, 1H), 3.08 (s, 3H), 2.39 (s, 3H), 1.85 (s, 3H), 1.20 (d, *J* = 6.3 Hz, 3H). *Major diastereomer*: **^{13}C NMR** (126 MHz, $CDCl_3$) δ 196.72, 165.67, 154.70, 143.22, 133.12, 130.22, 129.55, 128.86, 128.60, 128.45, 128.36, 127.27, 127.22, 120.50, 75.30, 67.17, 37.67, 26.09, 18.82, 16.65. **HRMS** (ESI-TOF) *m/z* calcd for $C_{26}H_{27}NO_3Na$ [$(M + Na)^+$] 424.1889, found 424.1881.

3-([1,1'-biphenyl]-4-yl(methyl)amino)-3-phenylbutan-2-yl benzoate (3p)

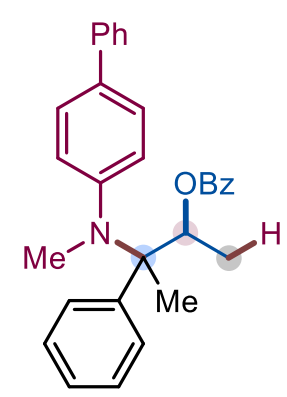

The reaction was performed according to General Procedure B at 40°C to afford the title compound (81.1 mg, 93% yield) as a white solid. **R*f*** = 0.4 [Hex: EtOAc 20:1 (v/v)]. *Mixture of diastereomers*: **^{1}H NMR** (800 MHz, $CDCl_3$) δ 7.96 (d, *J* = 8.1 Hz, 2H), 7.83 (d, *J* = 7.4 Hz, 2H), 7.60 – 7.56 (m, 2H), 7.57 – 7.51 (m, 3H), 7.50 – 7.47 (m, 2H), 7.48 – 7.43 (m, 1H), 7.44 – 7.37 (m, 8H), 7.36 – 7.27 (m, 12H), 7.26 – 7.22 (m, 2H), 7.12 (d, *J* = 8.1 Hz, 2H), 6.93 (d, *J* = 8.3 Hz, 2H), 5.84 (q, *J* = 6.3 Hz, 1H), 5.79 (q, *J* = 6.4 Hz, 1H), 2.84 (s, 3H), 2.75 (s, 3H), 1.81 (s, 3H), 1.74 (s, 3H), 1.39 (d, *J* = 6.3 Hz, 3H), 1.08 (d, *J* = 6.4 Hz, 3H). *Mixture of diastereomers*: **^{13}C NMR** (201 MHz, $CDCl_3$) δ 166.00, 165.62, 150.32, 149.38, 143.55, 142.44, 140.81, 135.89, 134.68, 132.91, 132.71, 130.68, 130.50, 129.59, 129.42, 128.67, 128.42, 128.37, 128.17, 128.10, 127.86, 127.70, 127.67, 127.06, 126.91, 126.80, 126.77, 126.72, 126.69, 126.66, 126.60, 126.45, 124.97, 75.27, 73.63, 66.86, 66.03, 38.53, 37.82, 19.77, 16.93, 16.23, 15.80. **HRMS** (ESI-TOF) *m/z* calcd for $C_{30}H_{30}NO_2$ [$(M + H)^+$] 436.2252, found 436.2273.

3-([1,1':4',1''-terphenyl]-4-yl(methyl)amino)-3-phenylbutan-2-yl benzoate (3q)

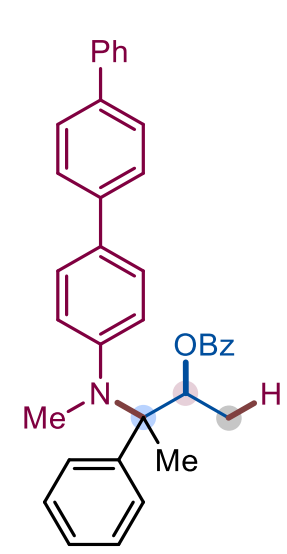

The reaction was performed according to General Procedure B at 40°C to afford the title compound (79.8 mg, 78% yield) as a yellow solid. **R*f*** = 0.5 [Hex: EtOAc 20:1 (v/v)]. *Major diastereomer*: **^{1}H NMR** (500 MHz, $CDCl_3$) δ 7.87 – 7.82 (m, 2H), 7.68 – 7.61 (m, 4H), 7.61 – 7.52 (m, 3H), 7.52 – 7.29 (m, 11H), 7.25 (t, *J* = 7.1 Hz, 1H), 7.17 – 7.11 (m, 2H), 5.81 (q, *J* = 6.3 Hz, 1H), 2.77 (s, 3H), 1.83 (s, 3H), 1.10 (d, *J* = 6.3 Hz, 3H). *Mixture of diastereomers*: **^{13}C NMR** (126 MHz, $CDCl_3$) δ 166.11, 165.74, 150.54, 149.60, 143.67, 142.61, 140.93, 139.85, 139.54, 139.48, 135.36, 134.11, 133.03, 132.84, 130.80, 130.63, 129.71, 129.55, 128.91, 128.49, 128.29, 128.23, 127.99, 127.81, 127.77, 127.50, 127.33, 127.26, 127.19, 127.09, 126.92, 126.72, 126.51, 124.96, 75.40, 73.78, 67.01, 66.18, 38.63, 37.91, 19.95, 17.04, 16.43, 15.93. **HRMS** (ESI-TOF) *m/z* calcd for $C_{36}H_{34}NO_2$ $[(M + H)^+]$ 512.2590, found 512.2588.

3-((4-(dibenzo[*b,d*]thiophen-4-yl)phenyl)(methyl)amino)-3-phenylbutan-2-yl benzoate (3r)

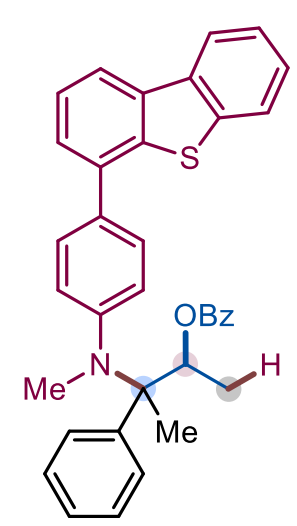

The reaction was performed according to General Procedure B at 40°C to afford the title compound (95.4 mg, 88% yield) as a white solid. **R*f*** = 0.6 [Hex: EtOAc 5:1 (v/v)]. *Major diastereomer*: **^{1}H NMR** (500 MHz, $CDCl_3$) δ 8.21 – 8.14 (m, 1H), 8.08 (dd, *J* = 7.9, 1.1 Hz, 1H), 7.93 – 7.88 (m, 1H), 7.87 – 7.79 (m, 2H), 7.62 – 7.57 (m, 2H), 7.59 – 7.29 (m, 11H), 7.18 (d, *J* = 8.5 Hz, 2H), 7.10 (dd, *J* = 7.4, 1.2 Hz, 1H), 5.90 – 5.83 (m, 1H), 2.85 (s, 3H), 1.86 (s, 3H), 1.14 (d, *J* = 6.3 Hz, 3H). *Mixture of diastereomers*: **^{13}C NMR** (126 MHz, $CDCl_3$) δ 166.10, 165.76, 150.91, 149.95, 143.68, 142.75, 139.78, 138.51, 138.45, 136.95, 136.83, 136.28, 136.13, 135.97, 134.83, 133.82, 133.04, 132.90, 130.79, 130.69, 129.72, 129.65, 128.50, 128.34, 128.33, 128.13, 128.08, 128.05, 127.79, 127.74, 127.19, 126.96, 126.81, 126.77, 126.00, 125.17, 124.97, 124.44, 124.41, 124.38, 122.70, 122.66, 121.82, 121.78, 120.05, 120.02, 75.32, 73.83, 67.00, 66.24, 38.57, 37.88, 20.12, 17.01, 15.98. **HRMS** (ESI-TOF) *m/z* calcd for $C_{36}H_{32}NO_2S$ $[(M + H)^+]$ 542.2154, found 542.2155.

3-((4-(dibenzo[*b,d*]furan-1-yl)phenyl)(methyl)amino)-3-phenylbutan-2-yl benzoate (3s)

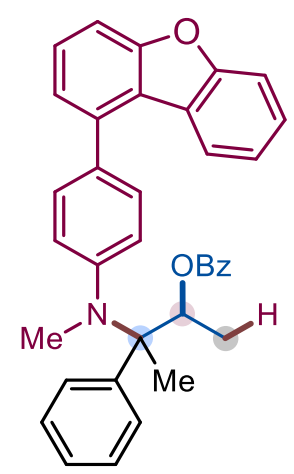

The reaction was performed according to General Procedure B at 40°C to afford the title compound (87.3 mg, 83% yield) as a white solid. **R$_f$** = 0.6 [Hex: EtOAc 5:1 (v/v)]. *Mixture of diastereomers*: **^{1}H NMR** (500 MHz, $CDCl_3$) δ 8.01 (d, *J* = 6.9 Hz, 2H), 7.93 (d, *J* = 6.9 Hz, 2H), 7.72 – 7.26 (m, 30H), 7.25 – 7.10 (m, 5H), 7.01 (d, *J* = 8.5 Hz, 2H), 6.84 (dd, *J* = 7.5, 1.0 Hz, 1H), 5.93 – 5.82 (m, 2H), 2.94 (s, 3H), 2.90 (s, 3H), 1.88 (s, 3H), 1.83 (s, 3H), 1.41 (d, *J* = 6.2 Hz, 3H), 1.17 (d, *J* = 6.3 Hz, 3H). *Mixture of diastereomers*: **^{13}C NMR** (126 MHz, $CDCl_3$) δ 166.14, 165.77, 156.57, 156.47, 156.43, 156.39, 150.84, 150.02, 143.35, 142.39, 138.00, 137.86, 134.15, 133.41, 133.04, 132.95, 130.83, 130.68, 129.71, 129.68, 128.87, 128.52, 128.38, 128.21, 128.12, 127.94, 127.17, 127.04, 126.99, 126.96, 126.86, 125.88, 124.74, 124.12, 124.07, 123.98, 123.95, 122.75, 122.66, 122.34, 122.30, 121.86, 121.73, 111.51, 110.15, 110.09, 75.18, 73.92, 66.90, 66.36, 38.63, 38.03, 19.63, 17.09, 16.98, 16.06. **HRMS** (ESI-TOF) *m/z* calcd for $C_{36}H_{32}NO_3$ [$(M + H)^+$] 526.2382, found 526.2386.

3-(methyl(4-(4,4,5,5-tetramethyl-1,3,2-dioxaborolan-2-yl)phenyl)amino)-3-phenylbutan-2-yl benzoate (3t)

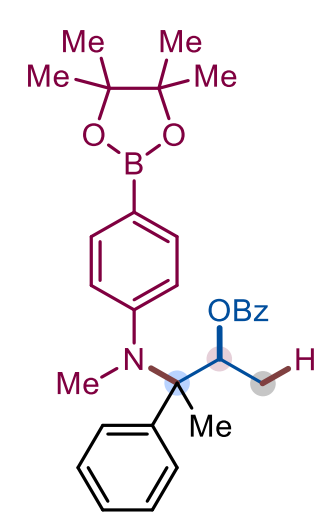

The reaction was performed according to General Procedure B at 40°C to afford the title compound (68.9 mg, 71% yield) as a white solid. **R$_f$** = 0.6 [Hex: EtOAc 5:1 (v/v)]. *Major diastereomer*: **^{1}H NMR** (300 MHz, $CDCl_3$) δ 7.93 – 7.74 (m, 2H), 7.59 – 7.13 (m, 10H), 6.89 (d, *J* = 8.2 Hz, 2H), 5.79 (q, *J* = 6.5 Hz, 1H), 2.88 (s, 3H), 1.77 (s, 3H), 1.39 – 1.19 (m, 12H), 1.15 (d, *J* = 6.3 Hz, 3H). *Mixture of diastereomers*: **^{13}C NMR** (201 MHz, $CDCl_3$) δ 166.07, 165.85, 153.72, 143.37, 143.16, 135.00, 133.11, 132.94, 130.71, 130.58, 129.73, 129.67, 129.19, 128.53, 128.38, 128.32, 128.24, 128.11, 127.69, 127.52, 127.07, 126.86, 123.40, 121.51, 83.52, 83.48, 75.22, 74.17, 66.81, 66.29, 38.21, 37.35, 25.02, 24.96, 22.84, 20.52, 18.12, 16.72, 15.96, 14.27. **HRMS** (ESI-TOF) *m/z* calcd for $C_{30}H_{37}NO_4B$ [$(M + H)^+$] 486.2816, found 486.2819.

3-((2,4-dimethylphenyl)(methyl)amino)-3-phenylbutan-2-yl benzoate (3u)

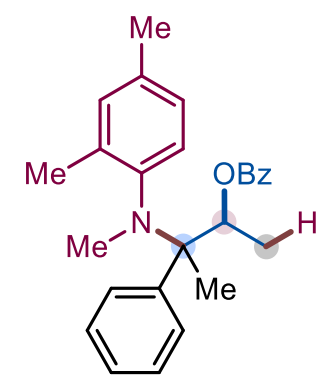

The reaction was performed according to General Procedure B at 40°C to afford the title compound (48.9 mg, 63% yield) as a colorless sticky liquid. **R*f*** = 0.4 [Hex: EtOAc 20:1 (v/v)]. *Major diastereomer*: **^{1}H NMR** (500 MHz, $CDCl_3$) δ 7.93 – 7.87 (m, 2H), 7.67 – 7.57 (m, 2H), 7.56 – 7.49 (m, 1H), 7.48 – 7.28 (m, 5H), 6.88 – 6.84 (m, 1H), 6.80 (d, *J* = 8.1 Hz, 1H), 6.66 (dd, *J* = 8.1, 2.2 Hz, 1H), 5.88 (q, *J* = 6.3 Hz, 1H), 2.46 (s, 3H), 2.36 (s, 3H), 2.01 (s, 3H), 1.73 (s, 3H), 0.99 – 0.81 (m, 3H). *Major diastereomer*: **^{13}C NMR** (201 MHz, $CDCl_3$) δ 165.58, 147.20, 143.08, 137.57, 134.75, 132.55, 131.45, 130.61, 129.66, 129.57, 128.56, 128.35, 127.96, 127.83, 127.01, 126.70, 126.06, 73.71, 66.59, 39.22, 20.63, 18.78, 16.74, 15.12. **HRMS** (ESI-TOF) *m/z* calcd for $C_{26}H_{30}NO_2$ $[(M + H)^+]$ 388.2270, found 388.2268.

3-((3,5-dimethylphenyl)(methyl)amino)-3-phenylbutan-2-yl benzoate (3v)

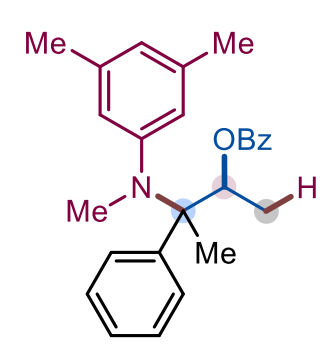

The reaction was performed according to General Procedure B at 40°C to afford the title compound (71.4 mg, 92% yield) as a yellow sticky oil. **R*f*** = 0.4 [Hex: EtOAc 20:1 (v/v)]. *Major diastereomer*: **^{1}H NMR** (800 MHz, $CDCl_3$) δ 7.98 (d, *J* = 7.6 Hz, 2H), 7.58 (d, *J* = 7.8 Hz, 2H), 7.56 (t, *J* = 7.4 Hz, 1H), 7.46 – 7.39 (m, 2H), 7.37 (t, *J* = 7.7 Hz, 3H), 6.79 (s, 1H), 6.62 (s, 2H), 5.61 – 5.55 (m, 1H), 2.66 (s, 3H), 2.19 (s, 6H), 1.69 (s, 3H), 1.33 (dd, *J* = 6.3, 1.7 Hz, 3H). *Mixture of diastereomers*: **^{13}C NMR** (201 MHz, $CDCl_3$) δ 165.95, 165.28, 150.81, 149.85, 144.16, 142.20, 137.58, 132.76, 132.46, 130.83, 130.50, 129.57, 129.26, 128.30, 128.16, 128.09, 127.79, 127.67, 127.59, 126.96, 126.71, 125.77, 125.32, 125.13, 124.58, 75.51, 73.55, 66.89, 66.06, 38.50, 38.17, 21.33, 21.12, 18.69, 17.10, 15.87, 14.75. **HRMS** (ESI-TOF) *m/z* calcd for $C_{26}H_{30}NO_2$ $[(M + H)^+]$ 388.2277, found 388.2267.

3-((4-bromo-3-methylphenyl)(methyl)amino)-3-phenylbutan-2-yl benzoate (3w)

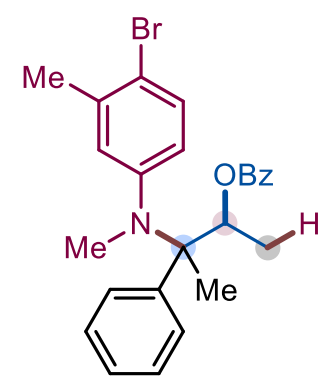

The reaction was performed according to General Procedure B at 40°C to afford the title compound (80.5 mg, 89% yield) as a yellow sticky oil. $\mathbf{R_f}$ = 0.4 [Hex: EtOAc 20:1 (v/v)]. *Major diastereomer*: **^{1}H NMR** (300 MHz, Chloroform-*d*) δ 7.78 (dt, *J* = 7.1, 1.4 Hz, 2H), 7.60 – 7.20 (m, 8H), 7.16 (d, *J* = 8.5 Hz, 1H), 6.95 (d, *J* = 2.7 Hz, 1H), 6.82 – 6.71 (m, 1H), 5.75 – 5.62 (m, 1H), 2.59 (s, 3H), 2.14 (s, 3H), 1.76 (s, 3H), 1.00 (d, *J* = 6.4 Hz, 3H). *Mixture of diastereomers*: **^{13}C NMR** (201 MHz, $CDCl_3$) δ 166.05, 165.53, 150.37, 149.38, 143.64, 141.99, 137.59, 133.03, 132.91, 131.96, 131.82, 130.74, 130.38, 129.68, 129.36, 128.49, 128.34, 128.28, 128.22, 127.97, 127.92, 127.67, 127.28, 127.03, 125.76, 124.59, 119.61, 118.37, 75.39, 73.55, 67.01, 66.13, 38.56, 38.04, 23.07, 22.86, 19.33, 17.17, 15.93, 15.23. **HRMS** (ESI-TOF) *m/z* calcd for $C_{25}H_{27}NO_2Br$ $[(M + H)^+]$ 452.1225, found 452.1219.

3-((2-cyano-4-methoxyphenyl)(methyl)amino)-3-phenylbutan-2-yl benzoate (3x)

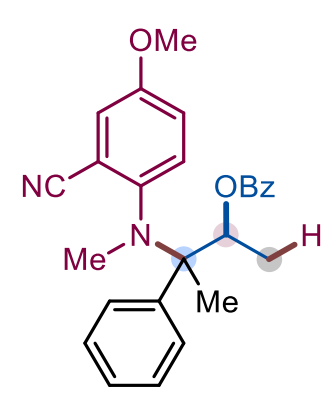

The reaction was performed according to General Procedure B at 40°C to afford the title compound (66.3 mg, 80% yield) as a yellow solid. $\mathbf{R_f}$ = 0.3 [Hex: EtOAc 5:1 (v/v)]. *Major diastereomer*: **^{1}H NMR** (500 MHz, $CDCl_3$) δ 7.80 – 7.75 (m, 4H), 7.53 – 7.48 (m, 1H), 7.43 (t, *J* = 7.6 Hz, 2H), 7.38 – 7.33 (m, 3H), 7.22 (d, *J* = 9.0 Hz, 1H), 6.87 (d, *J* = 3.1 Hz, 1H), 6.71 (dd, *J* = 9.0, 3.1 Hz, 1H), 5.79 (q, *J* = 6.3 Hz, 1H), 3.48 (s, 3H), 2.55 (s, 3H), 1.86 (s, 3H), 0.91 (d, *J* = 6.3 Hz, 3H). *Major diastereomer*: **^{13}C NMR** (126 MHz, $CDCl_3$) δ 165.15, 156.69, 147.78, 143.16, 132.88, 132.79, 130.27, 129.99, 129.64, 129.59, 128.51, 128.27, 128.07, 128.03, 127.93, 127.52, 127.32, 119.77, 119.49, 118.86, 117.12, 117.03, 115.41, 75.59, 72.66, 68.16, 66.00, 55.79, 55.46, 39.66, 39.37, 18.96, 17.16, 15.63, 14.15. **HRMS** (ESI-TOF) *m/z* calcd for $C_{26}H_{26}N_2O_3Na$ $[(M + Na)^+]$ 437.1841, found 437.1838.

3-((3-iodo-4-methoxyphenyl)(methyl)amino)-3-phenylbutan-2-yl benzoate (3y)

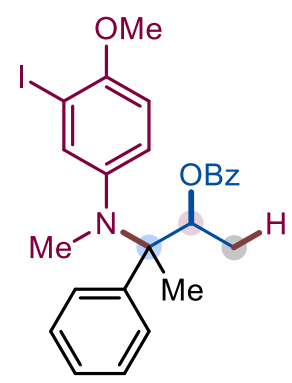

The reaction was performed according to General Procedure B at 40°C to afford the title compound (93.7 mg, 91% yield) as a yellow sticky oil. **R*f*** = 0.3 [Hex: EtOAc 20:1 (v/v)]. *Major diastereomer*: **^{1}H NMR** (800 MHz, $CDCl_3$) δ 7.93 – 7.87 (m, 2H), 7.63 – 7.56 (m, 2H), 7.54 – 7.47 (m, 2H), 7.44 – 7.29 (m, 5H), 6.92 (dd, *J* = 8.8, 2.6 Hz, 1H), 6.63 (d, *J* = 8.7 Hz, 1H), 5.58 (q, *J* = 6.2 Hz, 1H), 3.82 (s, 3H), 2.55 (s, 3H), 1.60 (s, 3H), 1.34 (d, *J* = 6.3 Hz, 3H). *Mixture of diastereomers*: **^{13}C NMR** (201 MHz, $CDCl_3$) δ 166.03, 165.37, 155.34, 145.46, 144.40, 143.57, 140.93, 139.15, 138.78, 132.91, 132.72, 130.76, 130.42, 129.69, 129.34, 129.22, 128.88, 128.41, 128.35, 128.19, 128.15, 127.75, 127.66, 127.33, 127.08, 110.32, 110.03, 85.62, 85.03, 75.66, 73.09, 67.09, 66.08, 56.60, 56.26, 38.99, 38.62, 18.39, 17.30, 15.78, 13.69. **HRMS** (ESI-TOF) *m/z* calcd for $C_{25}H_{27}NO_3I$ [$(M + H)^+$] 516.1036, found 516.1035.

3-((2-fluoro-4-methoxyphenyl)(methyl)amino)-3-phenylbutan-2-yl benzoate (3z)

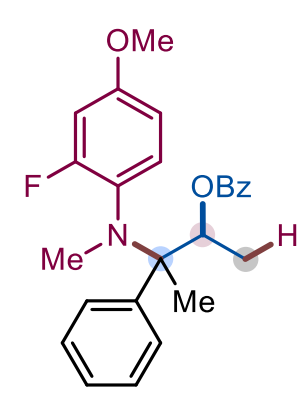

The reaction was performed according to General Procedure B at 40°C to afford the title compound (72.4 mg, 89% yield) as a yellow sticky oil. **R*f*** = 0.4 [Hex: EtOAc 20:1 (v/v)]. *Mixture of diastereomers*: **^{1}H NMR** (300 MHz, Chloroform-*d*) δ 7.89 – 7.76 (m, 4H), 7.69 – 7.60 (m, 2H), 7.60 – 7.54 (m, 2H), 7.53 – 7.44 (m, 2H), 7.43 – 7.28 (m, 9H), 7.25 – 7.19 (m, 1H), 7.11 (t, *J* = 9.2 Hz, 1H), 6.85 (t, *J* = 9.0 Hz, 1H), 6.60 – 6.45 (m, 2H), 6.41 – 6.27 (m, 2H), 5.78 – 5.56 (m, 2H), 3.75 (s, 3H), 3.46 (s, 3H), 2.54 (s, 3H), 2.50 (s, 3H), 1.79 (s, 3H), 1.63 (s, 3H), 1.38 (d, *J* = 6.3 Hz, 3H), 0.91 (d, *J* = 6.4 Hz, 3H). *Mixture of diastereomers*: **^{13}C NMR** (201 MHz, $CDCl_3$) δ 165.90, 165.32, 161.62, 161.53, 160.39, 160.30, 158.03, 157.97, 157.73, 157.68, 143.82, 141.14, 132.64, 132.49, 131.38, 131.29, 130.76, 130.65, 130.50, 129.99, 129.93, 129.48, 129.40, 128.17, 128.15, 128.09, 127.87, 127.72, 127.58, 127.08, 126.82, 109.40, 109.22, 102.38, 102.26, 102.21, 102.08, 75.35, 72.97, 67.09, 65.92, 55.56, 55.28, 38.23, 38.14, 18.34, 16.99, 15.60, 14.79. *Mixture of diastereomers*: **^{19}F NMR** (282 MHz, $CDCl_3$) δ -114.21 (t, *J* = 10.8 Hz), -114.61. **HRMS** (ESI-TOF) *m/z* calcd for $C_{25}H_{26}NO_3NaF$ [$(M + Na)^+$] 430.1794, found 430.1792.

3-((3-chloro-4-fluorophenyl)(methyl)amino)-3-phenylbutan-2-yl benzoate (3aa)

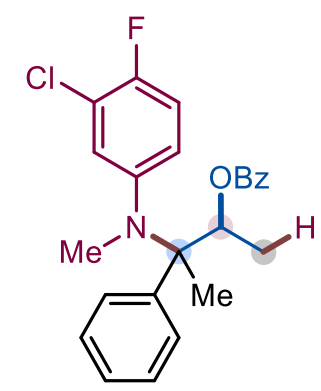

The reaction was performed according to General Procedure B at 40°C to afford the title compound (80.8 mg, 98% yield) as a yellow sticky oil. $\mathbf{R_f}$ = 0.4 [Hex: EtOAc 20:1 (v/v)]. *Major diastereomer*: **^{1}H NMR** (500 MHz, $CDCl_3$) δ 7.82 (d, *J* = 6.9 Hz, 2H), 7.56 – 7.50 (m, 3H), 7.43 – 7.37 (m, 3H), 7.36 – 7.29 (m, 2H), 7.10 (dd, *J* = 6.7, 2.7 Hz, 1H), 6.95 – 6.88 (m, 1H), 6.76 (t, *J* = 8.8 Hz, 1H), 5.76 – 5.62 (m, 1H), 2.56 (s, 3H), 1.74 (s, 3H), 1.00 (d, *J* = 6.4 Hz, 3H). *Mixture of diastereomers*: **^{13}C NMR** (126 MHz, $CDCl_3$) δ 166.04, 165.52, 155.98, 155.67, 154.02, 153.71, 147.86, 147.83, 146.91, 143.05, 141.11, 133.06, 133.00, 130.64, 130.32, 129.65, 129.36, 129.21, 128.62, 128.49, 128.41, 128.29, 127.99, 127.97, 127.59, 127.45, 127.23, 127.21, 127.16, 126.32, 126.26, 120.27, 120.12, 115.91, 115.84, 115.74, 115.67, 75.07, 73.16, 67.04, 66.14, 38.83, 38.36, 19.03, 17.14, 15.85, 15.21. *Major diastereomer*: **^{19}F NMR** (470 MHz, $CDCl_3$) δ -121.55 – -121.77 (m). **HRMS** (ESI-TOF) *m/z* calcd for $C_{24}H_{24}NO_2ClF$ [$(M + H)^+$] 412.1480, found 412.1476.

3-((2-chloro-4-fluoro-5-methylphenyl)(methyl)amino)-3-phenylbutan-2-yl benzoate (3ab)

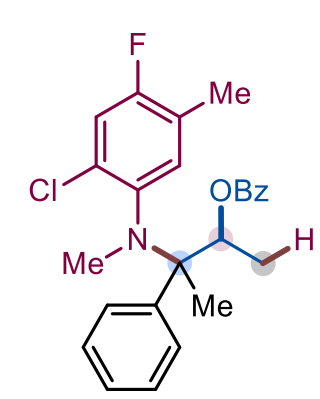

The reaction was performed according to General Procedure B at 40°C to afford the title compound (68.1 mg, 80% yield) as a colorless sticky oil. $\mathbf{R_f}$ = 0.4 [Hex: EtOAc 20:1 (v/v)]. *Major diastereomer*: **^{1}H NMR** (400 MHz, $CDCl_3$) δ 7.88 – 7.68 (m, 4H), 7.57 – 7.47 (m, 1H), 7.46 – 7.28 (m, 5H), 7.07 – 6.89 (m, 2H), 5.89 – 5.63 (m, 1H), 2.43 (s, 3H), 1.89 (s, 3H), 1.83 (s, 3H), 0.85 (s, 3H). *Major diastereomer*: **^{13}C NMR** (201 MHz, $CDCl_3$) δ 165.22, 158.88, 157.66, 143.83, 143.69, 133.16, 132.71, 132.43, 130.61, 129.46, 128.30, 128.15, 127.37, 123.76, 116.74, 116.61, 74.65, 67.74, 39.23, 29.84, 17.15, 14.35, 14.24. *Major diastereomer*: **^{19}F NMR** (376 MHz, $CDCl_3$) δ -120.19. **HRMS** (ESI-TOF) *m/z* calcd for $C_{25}H_{25}NO_2FNaCl$ [$(M + Na)^+$] 448.1456, found 448.1452.

3-((5-bromo-4-fluoro-2-methylphenyl)(methyl)amino)-3-phenylbutan-2-yl benzoate (3ac)

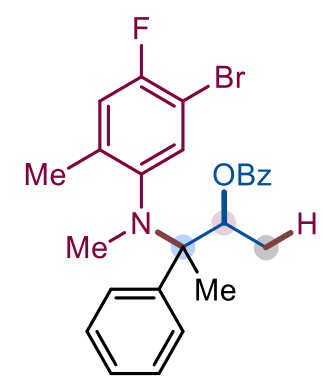

The reaction was performed according to General Procedure B at 40°C to afford the title compound (75.3 mg, 80% yield) as a white solid. $\mathbf{R}_f$ = 0.4 [Hex: EtOAc 20:1 (v/v)]. *Major diastereomer*: **^{1}H NMR** (400 MHz, $CDCl_3$) δ 7.86 (d, *J* = 7.6 Hz, 2H), 7.64 – 7.47 (m, 3H), 7.50 – 7.29 (m, 5H), 7.00 (d, *J* = 6.8 Hz, 1H), 6.76 (d, *J* = 9.4 Hz, 1H), 5.84 (q, *J* = 6.4 Hz, 1H), 2.37 (d, *J* = 8.1 Hz, 6H), 1.72 (s, 3H), 0.89 (s, 3H). *Major diastereomer*: **^{13}C NMR** (201 MHz, $CDCl_3$) δ 165.58, 156.96, 155.73, 146.93, 142.56, 139.92, 133.33, 133.05, 132.94, 130.32, 129.79, 129.42, 128.70, 128.47, 128.34, 128.03, 127.70, 127.62, 126.20, 117.68, 117.57, 105.13, 105.03, 74.05, 73.06, 67.14, 39.57, 18.75, 17.14, 13.92. *Major diastereomer*: **^{19}F NMR** (376 MHz, $CDCl_3$) δ -113.92. **HRMS** (ESI-TOF) *m/z* calcd for $C_{25}H_{26}NO_2FBr$ [$(M + H)^+$] 470.1131, found 470.1140.

3-((3-cyano-2,4-difluorophenyl)(methyl)amino)-3-phenylbutan-2-yl benzoate (3ad)

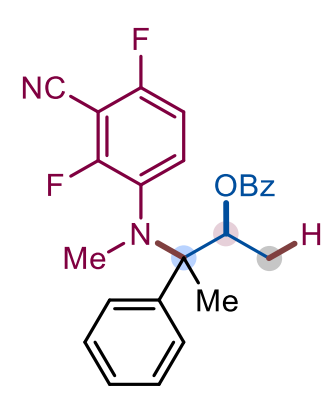

The reaction was performed according to General Procedure B at 40°C to afford the title compound (75.6 mg, 90% yield) as a colorless sticky oil. $\mathbf{R}_f$ = 0.2 [Hex: EtOAc 10:1 (v/v)]. *Major diastereomer*: **^{1}H NMR** (400 MHz, $CDCl_3$) δ 7.83 – 7.76 (m, 2H), 7.61 – 7.53 (m, 3H), 7.47 – 7.37 (m, 5H), 7.36 – 7.29 (m, 1H), 6.66 (ddd, *J* = 9.3, 7.9, 1.6 Hz, 1H), 5.59 (q, *J* = 6.4 Hz, 1H), 2.52 (d, *J* = 1.3 Hz, 3H), 1.82 (d, *J* = 1.6 Hz, 3H), 0.94 (d, *J* = 6.4 Hz, 3H). *Major diastereomer*: **^{13}C NMR** (201 MHz, $CDCl_3$) δ 165.28, 162.02, 160.73, 160.62, 159.32, 142.71, 137.36, 135.44, 135.39, 133.40, 129.83, 129.21, 128.64, 128.54, 127.78, 127.54, 111.60, 111.50, 109.17, 93.14, 93.05, 75.12, 67.67, 37.81, 17.03, 15.11. *Major diastereomer*: **^{19}F NMR** (376 MHz, Chloroform-*d*) δ -107.09 (d, *J* = 8.8 Hz), -109.61 (t, *J* = 7.0 Hz). **HRMS** (ESI-TOF) *m/z* calcd for $C_{25}H_{22}N_2O_2F_2Na$ [$(M + Na)^+$] 443.1547, found 443.1542.

3-(methyl(naphthalen-1-yl)amino)-3-phenylbutan-2-yl benzoate (3ae)

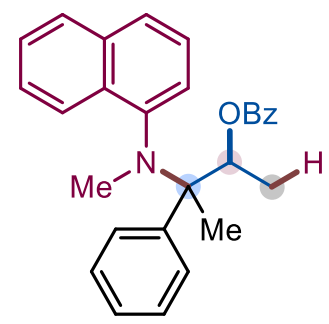

The reaction was performed according to General Procedure B at 40°C to afford the title compound (40.2 mg, 49% yield) as a white solid. **R$_f$** = 0.4 [Hex: EtOAc 20:1 (v/v)]. *Major diastereomer*: **^{1}H NMR** (500 MHz, $CDCl_3$) δ 8.58 (d, *J* = 8.5 Hz, 1H), 8.15 – 8.07 (m, 2H), 7.71 (d, *J* = 8.1 Hz, 1H), 7.65 – 7.56 (m, 3H), 7.55 – 7.41 (m, 5H), 7.42 – 7.33 (m, 2H), 7.19 (t, *J* = 7.7 Hz, 2H), 6.79 (d, *J* = 1.1 Hz, 1H), 6.29 (q, *J* = 6.4 Hz, 1H), 2.70 (s, 3H), 1.57 (s, 3H), 1.05 (d, *J* = 6.4 Hz, 3H). *Major diastereomer*: **^{13}C NMR** (126 MHz, $CDCl_3$) δ 165.91, 148.09, 141.04, 134.64, 134.12, 133.00, 130.86, 129.97, 128.40, 128.15, 128.07, 127.89, 127.37, 125.82, 125.65, 125.47, 125.42, 124.92, 124.76, 72.23, 66.31, 39.50, 17.09, 16.63. **HRMS** (ESI-TOF) *m/z* calcd for $C_{28}H_{27}NO_2Na$ [$(M + Na)^+$] 432.1939, found 432.1937.

3-(dibenzo[*b,d*]furan-4-ylamino)-3-phenylbutan-2-yl benzoate (3af)

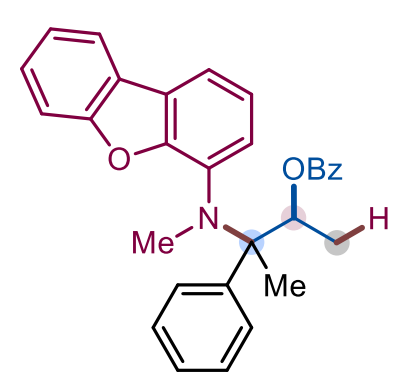

The reaction was performed according to General Procedure B at 40°C to afford the title compound (53.1 mg, 59% yield) as a yellow sticky oil. **R$_f$** = 0.4 [Hex: EtOAc 20:1 (v/v)]. *Diastereomer 1*: **^{1}H NMR** (300 MHz, $CDCl_3$) δ 7.82 – 7.65 (m, 3H), 7.62 – 7.47 (m, 3H), 7.50 – 7.38 (m, 3H), 7.40 – 7.25 (m, 5H), 7.18 – 6.93 (m, 3H), 5.80 (q, *J* = 6.3 Hz, 1H), 2.79 (s, 3H), 1.96 (s, 3H), 0.98 (d, *J* = 6.4 Hz, 3H). *Diastereomer 1*: **^{13}C NMR** (201 MHz, $CDCl_3$) δ 165.20, 155.88, 153.84, 143.99, 135.95, 132.38, 130.22, 129.20, 128.28, 128.02, 127.74, 127.64, 127.25, 126.94, 125.55, 124.69, 122.88, 122.54, 120.58, 117.11, 111.80, 75.34, 67.62, 38.11, 16.99, 16.03. *Diastereomer 2*: **^{1}H NMR** (300 MHz, $CDCl_3$) δ 7.90 – 7.77 (m, 3H), 7.76 – 7.62 (m, 3H), 7.58 – 7.21 (m, 9H), 7.20 – 7.04 (m, 2H), 5.72 (q, *J* = 6.3 Hz, 1H), 2.81 (s, 3H), 1.81 (s, 3H), 1.36 (d, *J* = 6.3 Hz, 3H). *Diastereomer 2*: **^{13}C NMR** (201 MHz, $CDCl_3$) δ 165.94, 155.80, 153.54, 141.84, 135.52, 132.62, 130.70, 129.63, 129.56, 128.57, 128.32, 128.17, 127.75, 127.02, 126.92, 125.53, 124.65, 122.79, 122.55, 120.59, 117.12, 111.78, 73.43, 66.69, 38.27, 19.08, 16.15. **HRMS** (ESI-TOF) *m/z* calcd for $C_{30}H_{27}NO_3Na$ [$(M + Na)^+$] 472.1889, found 472.1884.

3-(dibenzo[*b,d*]thiophen-4-yl(methyl)amino)-3-phenylbutan-2-yl benzoate (3ag)

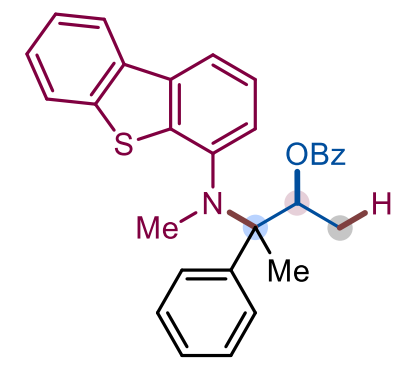

The reaction was performed according to General Procedure B at 40°C to afford the title compound (87.6 mg, 94% yield) as a white solid. **R*f*** = 0.4 [Hex: EtOAc 20:1 (v/v)]. *Major diastereomer*: **^{1}H NMR** (300 MHz, $CDCl_3$) δ 8.09 – 7.95 (m, 1H), 7.93 – 7.84 (m, 2H), 7.85 – 7.78 (m, 1H), 7.79 – 7.69 (m, 3H), 7.59 – 7.31 (m, 8H), 7.23 – 7.13 (m, 2H), 5.99 (q, *J* = 6.3 Hz, 1H), 2.74 (s, 3H), 1.89 (s, 3H), 1.01 (d, *J* = 6.3 Hz, 3H). *Major diastereomers*: **^{13}C NMR** (201 MHz, $CDCl_3$) δ 165.60, 146.21, 142.32, 139.82, 136.84, 136.46, 132.73, 130.62, 129.81, 128.30, 128.15, 128.06, 127.34, 126.66, 125.26, 125.23, 124.20, 122.94, 121.67, 118.58, 74.10, 67.34, 38.72, 16.69, 16.59. **HRMS** (ESI-TOF) *m/z* calcd for $C_{30}H_{27}NO_2SNa$ [(M + Na)$^+$] 488.1660, found 488.1654.

3-(3,4-dihydroquinolin-1(2H)-yl)-3-phenylbutan-2-yl benzoate (3ah)

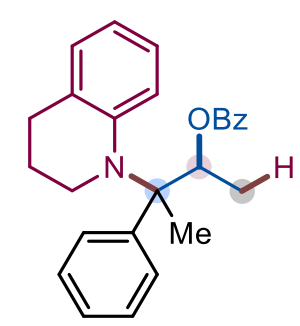

The reaction was performed according to General Procedure B at 40°C to afford the title compound (32.4 mg, 42% yield) as a colorless sticky oil. **R*f*** = 0.4 [Hex: EtOAc 20:1 (v/v)]. *Major diastereomer*: **^{1}H NMR** (400 MHz, $CDCl_3$) δ 8.00 – 7.92 (m, 2H), 7.62 – 7.51 (m, 1H), 7.51 – 7.37 (m, 4H), 7.36 – 7.26 (m, 3H), 7.01 – 6.90 (m, 1H), 6.69 – 6.47 (m, 2H), 6.22 (d, *J* = 8.3 Hz, 1H), 5.90 – 5.76 (m, 1H), 3.50 – 3.40 (m, 1H), 3.39 – 3.26 (m, 1H), 2.84 – 2.60 (m, 2H), 2.18 – 1.99 (m, 1H), 1.99 – 1.84 (m, 4H), 1.25 – 1.18 (m, 3H). *Mixture of diastereomer*: **^{13}C NMR** (201 MHz, $CDCl_3$) δ 165.96, 165.88, 145.72, 145.43, 143.86, 142.75, 133.08, 130.48, 130.30, 129.70, 129.64, 128.81, 128.61, 128.47, 128.41, 128.10, 127.87, 127.83, 127.34, 126.78, 126.66, 125.01, 124.83, 119.07, 118.14, 117.57, 117.05, 75.21, 74.95, 66.12, 65.79, 44.49, 44.27, 28.20, 27.96, 24.83, 24.53, 19.07, 17.65, 16.00, 15.85. **HRMS** (ESI-TOF) *m/z* calcd for $C_{26}H_{28}NO_2$ [(M + H)$^+$] 386.2120, found 386.2113.

3-(cyclohexyl(phenyl)amino)-3-phenylbutan-2-yl benzoate (3ai)

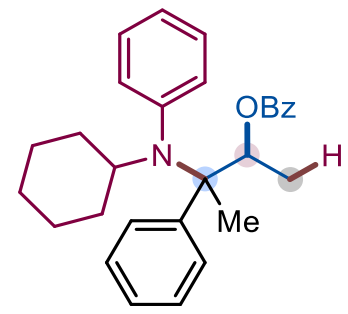

The reaction was performed according to General Procedure B at 40°C to afford the title compound (66.8 mg, 78% yield) as a yellow sticky oil. **R*f*** = 0.4 [Hex: EtOAc 20:1 (v/v)]. *Major diastereomer*: **^{1}H NMR** (500 MHz, $CDCl_3$) δ 7.93 (d, *J* = 6.9 Hz, 2H), 7.57 – 7.46 (m, 1H), 7.45 – 7.29 (m, 3H), 7.29 – 7.18 (m, 4H), 7.19 – 7.07 (m, 3H), 6.56 – 6.47 (m, 2H), 6.01 – 5.89 (m, 1H), 3.27 – 3.16 (m, 1H), 2.07 – 2.01 (m, 2H), 1.76 – 1.70 (m, 5H), 1.43 – 1.29 (m, 2H), 1.29 – 1.06 (m, 7H). *Mixture of diastereomers*: **^{13}C NMR** (126 MHz, $CDCl_3$) δ 166.41, 147.58, 145.64, 134.08, 132.83, 130.94, 129.71, 129.67, 129.37, 128.75, 128.38, 128.21, 128.17, 128.03, 127.94, 127.90, 127.24, 125.95, 112.91, 112.79, 112.62, 75.71, 75.53, 51.94, 50.21, 33.74, 33.72, 26.06, 25.17, 25.11, 16.25. **HRMS** (ESI-TOF) *m/z* calcd for $C_{29}H_{34}NO_2$ [$(M + H)^+$] 428.2590, found 428.2583.

3-(cyclobutyl(phenyl)amino)-3-phenylbutan-2-yl benzoate (3aj)

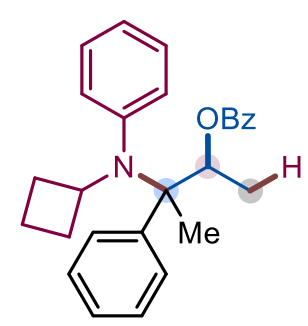

The reaction was performed according to General Procedure B at 40°C to afford the title compound (37.6 mg, 47% yield) as a yellow sticky oil. **R*f*** = 0.4 [Hex: EtOAc 20:1 (v/v)]. *Major diastereomer*: **^{1}H NMR** (500 MHz, $CDCl_3$) δ 7.95 – 7.89 (m, 2H), 7.54 – 7.47 (m, 1H), 7.42 – 7.31 (m, 3H), 7.29 – 7.20 (m, 4H), 7.18 – 7.10 (m, 3H), 6.51 – 6.43 (m, 2H), 5.93 (q, *J* = 6.2 Hz, 1H), 3.93 – 3.82 (m, 1H), 2.44 – 2.34 (m, 2H), 1.85 – 1.73 (m, 7H), 1.20 (d, *J* = 6.3 Hz, 3H). *Major diastereomer*: **^{13}C NMR** (126 MHz, $CDCl_3$) δ 166.43, 147.54, 145.47, 134.62, 132.86, 130.94, 129.72, 129.37, 128.74, 128.40, 128.21, 128.07, 127.94, 125.98, 112.68, 112.50, 75.68, 50.25, 49.23, 31.49, 25.10, 16.26, 15.39. **HRMS** (ESI-TOF) *m/z* calcd for $C_{25}H_{28}NO_2$ [$(M + H)^+$] 400.2277, found 400.2267.

3-phenyl-3-(phenyl(*m*-tolyl)amino)butan-2-yl benzoate (3ak)

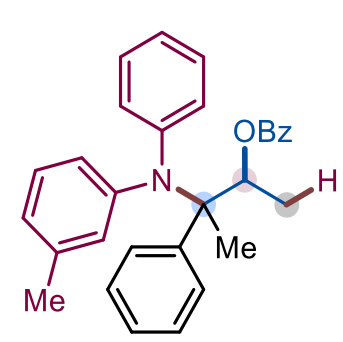

The reaction was performed according to General Procedure B at 40°C to afford the title compound (56.0 mg, 62% yield) as a colorless sticky oil. **R_f** = 0.3 [Hex: EtOAc 20:1 (v/v)]. *Mixture of diastereomers*: **^{1}H NMR** (300 MHz, $CDCl_3$) δ 8.03 – 7.87 (m, 4H), 7.61 – 7.46 (m, 2H), 7.47 – 7.32 (m, 4H), 7.35 – 7.07 (m, 18H), 7.07 – 6.94 (m, 4H), 6.95 – 6.81 (m, 4H), 6.80 – 6.69 (m, 2H), 6.05 – 5.89 (m, 2H), 2.29 (s, 3H), 1.89 (s, 3H), 1.81 (s, 3H), 1.77 (s, 3H), 1.25 – 1.15 (m, 6H). *Mixture of diastereomers*: **^{13}C NMR** (201 MHz, $CDCl_3$) δ 166.36, 166.27, 147.16, 145.76, 143.19, 143.15, 141.39, 141.30, 139.32, 138.67, 138.34, 137.75, 133.02, 132.95, 130.83, 129.79, 129.70, 129.45, 129.42, 129.35, 129.27, 128.45, 128.21, 128.17, 128.00, 127.86, 127.82, 126.22, 126.17, 122.22, 121.90, 120.93, 118.56, 117.93, 117.16, 114.96, 114.33, 76.14, 75.47, 50.40, 24.72, 22.51, 21.80, 21.67, 16.76, 16.27. **HRMS** (ESI-TOF) *m/z* calcd for $C_{30}H_{30}NO_2$ [$(M + H)^+$] 436.2277, found 436.2274.

3-phenyl-3-(phenyl(*p*-tolyl)amino)butan-2-yl benzoate (3al)

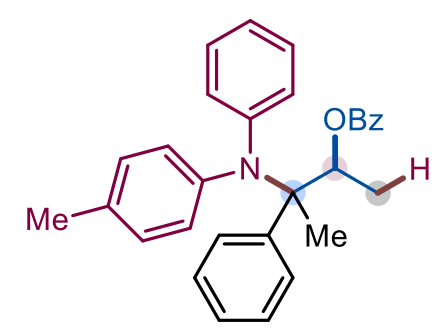

The reaction was performed according to General Procedure B at 40°C to afford the title compound (36.6 mg, 42% yield) as a colorless sticky oil. **R_f** = 0.4 [Hex: EtOAc 20:1 (v/v)]. *Major diastereomer*: **^{1}H NMR** (500 MHz, $CDCl_3$) δ 7.85 – 7.79 (m, 2H), 7.45 – 7.38 (m, 1H), 7.29 (t, *J* = 7.7 Hz, 2H), 7.22 – 7.14 (m, 5H), 7.14 – 7.09 (m, 2H), 7.10 – 7.03 (m, 1H), 6.96 (d, *J* = 8.2 Hz, 2H), 6.90 – 6.80 (m, 4H), 5.87 (q, *J* = 6.3 Hz, 1H), 2.19 (s, 3H), 1.70 (s, 3H), 1.13 (d, *J* = 6.3 Hz, 3H). *Major diastereomer*: **^{13}C NMR** (126 MHz, $CDCl_3$) δ 166.36, 147.22, 142.09, 140.45, 137.78, 132.92, 130.88, 130.85, 129.95, 129.70, 129.35, 128.43, 128.18, 127.87, 126.14, 118.87, 116.32, 75.51, 50.38, 24.79, 20.81, 16.26. **HRMS** (ESI-TOF) *m/z* calcd for $C_{30}H_{30}NO_2$ [$(M + H)^+$] 436.2277, found 436.2275.

3-(10,11-dihydro-5*H*-dibenzo[*b,f*]azepin-5-yl)-3-phenylbutan-2-yl benzoate (3am)

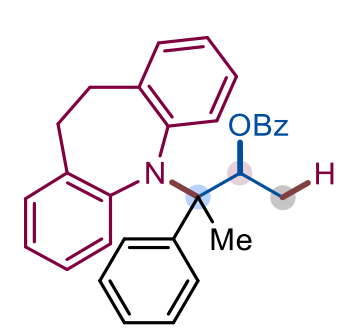

The reaction was performed according to General Procedure B at 40°C to afford the title compound (81.5 mg, 91% yield) as a white solid. **R_f** = 0.5 [Hex: EtOAc 20:1 (v/v)]. *Major diastereomer*: **^{1}H NMR** (400 MHz,

$CDCl_3$) δ 7.95 (d, *J* = 7.0 Hz, 2H), 7.61 – 7.48 (m, 1H), 7.41 (t, *J* = 7.6 Hz, 2H), 7.33 – 7.25 (m, 4H), 7.20 (q, *J* = 4.2 Hz, 1H), 7.12 – 6.92 (m, 4H), 6.83 – 6.47 (m, 3H), 6.06 – 5.84 (m, 2H), 3.14 – 2.92 (m, 4H), 1.80 (s, 3H), 1.24 (d, *J* = 6.2 Hz, 3H). *Major diastereomer*: ^{13}**C NMR** (126 MHz, $CDCl_3$) δ 166.34, 147.18, 142.71, 140.73, 132.93, 130.87, 129.69, 128.90, 128.42, 128.17, 127.90, 126.94, 126.12, 119.60, 118.00, 117.55, 75.40, 50.26, 35.30, 34.98, 24.82, 16.28. **HRMS** (ESI-TOF) *m/z* calcd for $C_{31}H_{30}NO_2$ [(M + H)$^+$] 448.2277, found 448.2276.

3-(2-fluorophenyl)-3-(methyl(4-(trifluoromethoxy)phenyl)amino)butan-2-yl benzoate (3an)

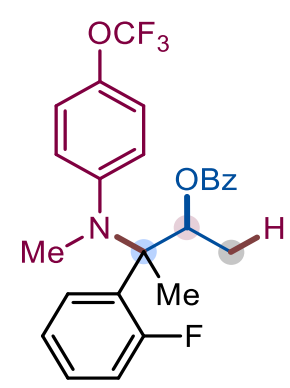

The reaction was performed according to General Procedure B at 40°C to afford the title compound (60.0 mg, 65% yield) as a colorless sticky oil. $\mathbf{R}_f$ = 0.4 [Hex: EtOAc 20:1 (v/v)]. *Major diastereomer*: ^{1}**H NMR** (300 MHz, Chloroform-*d*) δ 7.97 – 7.82 (m, 2H), 7.59 – 7.48 (m, 1H), 7.44 – 7.35 (m, 3H), 7.23 – 7.13 (m, 1H), 7.10 – 6.99 (m, 1H), 6.99 – 6.86 (m, 5H), 6.16 – 6.06 (m, 1H), 2.80 (s, 3H), 1.78 (d, *J* = 1.7 Hz, 3H), 1.23 (d, *J* = 0.9 Hz, 3H). *Mixture of diastereomers*: ^{13}**C NMR** (201 MHz, $CDCl_3$) δ 165.99, 165.66, 161.76, 160.52, 148.29, 144.44, 143.21, 133.17, 133.00, 130.58, 130.55, 129.70, 129.56, 129.27, 129.10, 129.06, 128.53, 128.39, 126.50, 124.08, 123.88, 123.34, 120.89, 120.75, 117.07, 117.00, 116.94, 116.87, 74.06, 73.98, 66.98, 65.77, 38.39, 37.63, 20.21, 18.71, 16.96, 15.50. *Major diastereomer*: ^{19}**F NMR** (282 MHz, $CDCl_3$) δ -58.18. **HRMS** (ESI-TOF) *m/z* calcd for $C_{25}H_{24}NO_3F_4$ [(M + H)$^+$] 462.1692, found 462.1691.

3-(3-fluorophenyl)-3-(methyl(4-(trifluoromethoxy)phenyl)amino)butan-2-yl benzoate (3ao)

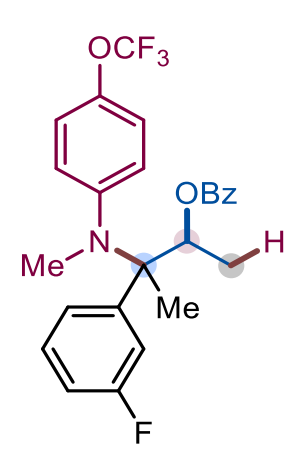

The reaction was performed according to General Procedure B at 40°C to afford the title compound (60.9 mg, 66% yield) as a colorless sticky oil. $\mathbf{R}_f$ = 0.4 [Hex: EtOAc 20:1 (v/v)]. *Major diastereomer*: ^{1}**H NMR** (400 MHz, $CDCl_3$) δ 7.99 – 7.88 (m, 2H), 7.60 – 7.49 (m, 1H), 7.47 – 7.37 (m, 2H), 7.32 – 7.18 (m, 3H), 7.10 – 6.92 (m, 3H), 6.91 – 6.84 (m, 2H), 5.65 (q, *J* = 6.3 Hz, 1H), 2.73 (s, 3H), 1.66 (s, 3H), 1.31 (d, *J* = 6.3

Hz, 3H). *Major diastereomer*: **^{13}C NMR** (126 MHz, $CDCl_3$) δ 165.91, 163.88, 148.47, 144.80, 133.17, 130.55, 129.64, 129.33, 129.27, 128.57, 126.77, 123.45, 121.00, 115.47, 115.28, 114.19, 114.02, 73.27, 66.07, 38.26, 19.26, 15.92. *Major diastereomer*: **^{19}F NMR** (376 MHz, $CDCl_3$) δ -59.57, -113.14 – -116.25 (m). **HRMS** (ESI-TOF) *m/z* calcd for $C_{25}H_{24}NO_3F_4$ [$(M + H)^+$] 462.1692, found 462.1689.

3-(4-fluorophenyl)-3-(methyl(phenyl)amino)butan-2-yl benzoate (3ap)

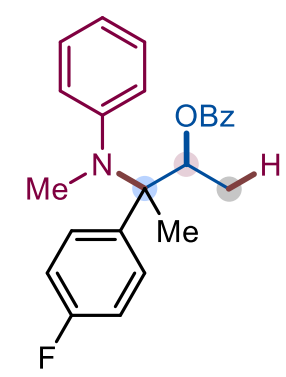

The reaction was performed according to General Procedure B at 40°C to afford the title compound (48.6 mg, 64% yield) as a colorless sticky oil. **R_f** = 0.4 [Hex: EtOAc 20:1 (v/v)]. *Major diastereomer*: **^{1}H NMR** (500 MHz, $CDCl_3$) δ 7.91 (d, J = 6.9 Hz, 2H), 7.46 – 7.34 (m, 5H), 7.16 (t, J = 7.7 Hz, 2H), 7.11 – 6.95 (m, 3H), 6.89 (d, J = 7.8 Hz, 2H), 5.74 – 5.66 (m, 1H), 2.70 (s, 3H), 1.66 (s, 3H), 1.33 (d, J = 6.3 Hz, 3H). *Mixture of diastereomers*: **^{13}C NMR** (126 MHz, $CDCl_3$) δ 166.02, 162.79, 160.84, 150.89, 150.04, 139.07, 137.75, 133.04, 132.88, 130.74, 130.60, 129.69, 129.62, 128.50, 128.43, 128.37, 128.24, 126.79, 126.34, 123.55, 123.38, 114.98, 114.81, 114.63, 114.46, 74.95, 73.44, 66.37, 65.67, 38.75, 38.08, 30.88, 19.27, 16.84, 15.84. *Major diastereomer*: **^{19}F NMR** (470 MHz, $CDCl_3$) δ -116.34 – -116.55 (m). **HRMS** (ESI-TOF) *m/z* calcd for $C_{24}H_{24}NO_2FNa$ [$(M + Na)^+$] 400.1689, found 400.1685.

3-(4-chlorophenyl)-3-(methyl(4-(trifluoromethoxy)phenyl)amino)butan-2-yl benzoate (3aq)

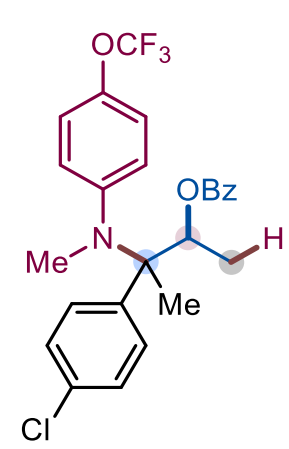

The reaction was performed according to General Procedure B at 40°C to afford the title compound (62.1 mg, 65% yield) as a colorless sticky oil. **R_f** = 0.4 [Hex: EtOAc 20:1 (v/v)]. *Major diastereomer*: **^{1}H NMR** (300 MHz, $CDCl_3$) δ 7.85 – 7.77 (m, 2H), 7.57 – 7.48 (m, 1H), 7.47 – 7.31 (m, 5H), 7.30 – 7.23 (m, 1H), 7.05 – 6.94 (m, 2H), 6.94 – 6.79 (m, 2H), 5.76 – 5.63 (m, 1H), 2.66 (s, 3H), 1.71 (s, 3H), 1.04 (d, J = 6.3 Hz, 3H). *Mixture of diastereomers*: **^{13}C NMR** (201 MHz, $CDCl_3$) δ 165.93, 165.61, 149.33, 148.50, 145.06, 144.78, 141.67, 140.22, 133.20, 133.10, 133.01, 130.51, 130.28, 129.62, 129.50, 129.37, 129.20, 128.58,

128.48, 128.37, 128.12, 127.54, 126.83, 120.97, 120.89, 74.61, 73.08, 66.59, 65.81, 38.77, 38.14, 19.29, 16.91, 16.60, 15.83. *Major diastereomer*: **^{19}F NMR** (282 MHz, $CDCl_3$) δ -58.18. **HRMS** (ESI-TOF) *m/z* calcd for $C_{25}H_{24}NO_3F_3Cl$ [$(M + H)^+$] 478.1397, found 478.1392.

3-(4-bromophenyl)-3-(methyl(4-(trifluoromethoxy)phenyl)amino)butan-2-yl benzoate (3ar)

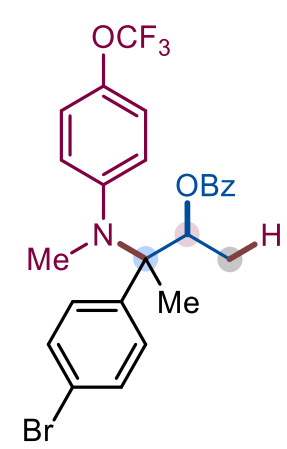

The reaction was performed according to General Procedure B at 40°C to afford the title compound (83.6 mg, 80% yield) as a colorless oil. **R_f** = 0.4 [Hex: EtOAc 20:1 (v/v)]. *Diastereomer 1*: **^{1}H NMR** (300 MHz, $CDCl_3$) δ 7.89 – 7.76 (m, 2H), 7.58 – 7.45 (m, 3H), 7.42 – 7.33 (m, 4H), 7.06 – 6.96 (m, 2H), 6.94 – 6.81 (m, 2H), 5.70 (q, *J* = 6.3 Hz, 1H), 2.67 (s, 3H), 1.71 (s, 3H), 1.05 (d, *J* = 6.3 Hz, 3H). **^{13}C NMR** (201 MHz, $CDCl_3$) δ 165.60, 145.09, 142.21, 133.11, 131.45, 130.27, 129.58, 129.50, 128.38, 127.55, 121.40, 120.91, 74.52, 66.67, 38.79, 16.92, 16.59. **^{19}F NMR** (282 MHz, $CDCl_3$) δ -58.18. *Diastereomer 2*: **^{1}H NMR** (300 MHz, $CDCl_3$) δ 7.97 – 7.84 (m, 2H), 7.60 – 7.51 (m, 1H), 7.48 – 7.37 (m, 4H), 7.35 – 7.28 (m, 2H), 7.04 – 6.93 (m, 2H), 6.90 – 6.81 (m, 2H), 5.68 (q, *J* = 6.2 Hz, 1H), 2.70 (s, 3H), 1.64 (s, 3H), 1.32 (d, *J* = 6.2 Hz, 3H). **^{13}C NMR** (201 MHz, $CDCl_3$) δ 165.92, 144.84, 140.73, 133.22, 131.10, 130.49, 129.76, 129.63, 128.59, 126.85, 121.28, 120.99, 120.01, 73.01, 65.93, 38.17, 19.26, 15.84. **^{19}F NMR** (282 MHz, $CDCl_3$) δ -58.04.

3-(4'-bromo-[1,1'-biphenyl]-4-yl)-3-(methyl(phenyl)amino)butan-2-yl benzoate (3as)

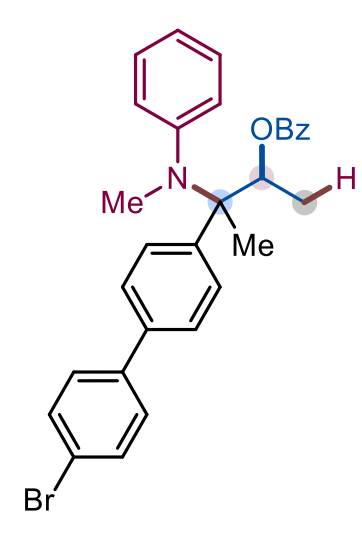

The reaction was performed according to General Procedure B at 40°C to afford the title compound (73.8 mg, 71% yield) as a colorless sticky oil. **R_f** = 0.4 [Hex: EtOAc 20:1 (v/v)]. *Major diastereomer*: **^{1}H NMR** (500 MHz, $CDCl_3$) δ 7.97 – 7.92 (m, 2H), 7.62 – 7.48 (m, 11H), 7.20 – 7.14 (m, 2H), 7.02 – 6.97 (m, 1H), 6.96 – 6.91 (m, 2H), 5.82 – 5.75 (m, 1H), 2.75 (s, 3H), 1.70 (s, 3H), 1.39 (d, *J* = 6.2 Hz, 3H). *Mixture of diastereomers*: **^{13}C NMR** (126 MHz, $CDCl_3$) δ 165.97, 165.68, 150.86, 149.98, 142.80, 141.77, 139.59,

138.39, 138.09, 132.90, 132.75, 131.91, 131.84, 130.69, 130.56, 129.56, 129.53, 128.62, 128.57, 128.40, 128.36, 128.28, 128.25, 128.12, 126.69, 126.42, 126.11, 126.01, 123.36, 123.04, 121.55, 121.44, 74.73, 73.35, 66.47, 65.75, 38.71, 38.03, 19.43, 17.00, 16.82, 15.72. **HRMS** (ESI-TOF) *m/z* calcd for $C_{30}H_{29}NO_2Br$ [(M + H)$^+$] 514.1382, found 514.1376.

3-(4-acetoxyphenyl)-3-(methyl(phenyl)amino)butan-2-yl benzoate (3at)

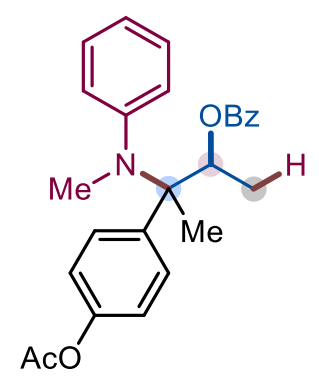

The reaction was performed according to General Procedure B at 40°C to afford the title compound (55.4 mg, 66% yield) as a colorless sticky oil. **R*f*** = 0.4 [Hex: EtOAc 20:1 (v/v)]. *Mixture of diastereomers*: **^{1}H NMR** (500 MHz, $CDCl_3$) δ 7.91 (d, *J* = 6.9 Hz, 2H), 7.83 (d, *J* = 6.8 Hz, 2H), 7.56 – 7.49 (m, 4H), 7.49 – 7.44 (m, 2H), 7.43 – 7.34 (m, 4H), 7.20 – 7.02 (m, 10H), 7.01 – 6.97 (m, 1H), 6.91 (d, *J* = 7.2 Hz, 2H), 6.87 – 6.78 (m, 1H), 5.79 – 5.67 (m, 2H), 2.70 (s, 3H), 2.69 (s, 3H), 2.31 (s, 3H), 2.27 (s, 3H), 1.73 (s, 3H), 1.66 (s, 3H), 1.34 (d, *J* = 6.2 Hz, 3H), 1.08 (d, *J* = 6.3 Hz, 3H). *Mixture of diastereomers*: **^{13}C NMR** (126 MHz, $CDCl_3$) δ 169.44, 169.41, 166.07, 165.74, 150.02, 149.72, 149.57, 139.75, 133.01, 132.84, 130.77, 130.65, 129.66, 129.63, 128.99, 128.88, 128.49, 128.41, 128.38, 128.22, 126.96, 126.28, 123.62, 123.30, 121.06, 120.72, 74.84, 73.47, 66.49, 65.79, 38.83, 38.15, 21.36, 21.33, 19.53, 17.05, 16.92, 15.87. **HRMS** (ESI-TOF) *m/z* calcd for $C_{26}H_{27}NO_4Na$ [(M + H)$^+$] 440.1838, found 440.1833.

4-(3-(benzoyloxy)-2-(methyl(phenyl)amino)butan-2-yl)phenyl thiophene-2-carboxylate (3au)

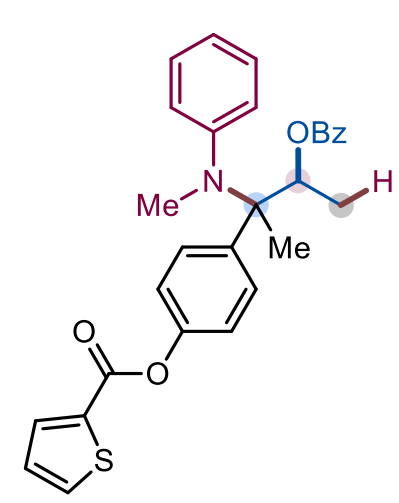

The reaction was performed according to General Procedure B at 40°C to afford the title compound (66.2 mg, 69% yield) as a white solid. **R*f*** = 0.4 [Hex: EtOAc 20:1 (v/v)]. *Major diastereomer*: **^{1}H NMR** (800 MHz, $CDCl_3$) δ 7.99 (s, 1H), 7.95 (d, *J* = 7.5 Hz, 2H), 7.68 (t, *J* = 4.3 Hz, 1H), 7.58 – 7.51 (m, 4H), 7.46 – 7.37 (m, 2H), 7.24 – 7.16 (m, 4H), 7.03 (t, *J* = 7.6 Hz, 1H), 6.97 – 6.93 (m, 2H), 5.76 – 5.72 (m, 1H), 2.75 (s, 3H), 1.71 (t, *J* = 2.2 Hz, 3H), 1.38 (dd, *J* = 6.3, 2.6 Hz, 3H). *Mixture of diastereomers*: **^{13}C NMR** (201 MHz,

$CDCl_3$) δ 165.96, 165.62, 160.51, 160.48, 150.76, 149.87, 149.48, 149.33, 140.85, 139.76, 134.70, 134.63, 133.53, 133.46, 133.00, 132.97, 132.90, 132.73, 130.62, 130.50, 129.54, 129.51, 128.95, 128.85, 128.38, 128.30, 128.27, 128.11, 128.07, 128.04, 126.81, 126.18, 123.49, 123.20, 121.04, 120.69, 74.69, 73.31, 66.37, 65.69, 38.70, 38.02, 19.35, 16.89, 16.78, 15.74. **HRMS** (ESI-TOF) *m/z* calcd for $C_{29}H_{27}NO_4SNa$ [$(M + Na)^+$] 508.1558, found 508.1562.

4-(3-(benzoyloxy)-2-(methyl(phenyl)amino)butan-2-yl)phenyl 5-methylthiazole-4-carboxylate (3av)

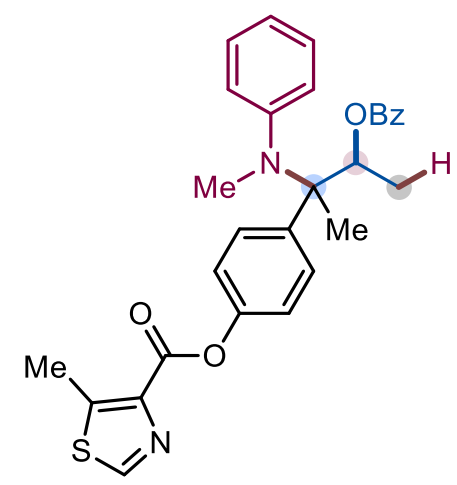

The reaction was performed according to General Procedure B at 40°C to afford the title compound (62.0 mg, 62% yield) as a white solid. R_f = 0.3 [Hex: EtOAc 5:1 (v/v)]. *Major diastereomer*: **^{1}H NMR** (800 MHz, $CDCl_3$) δ 8.85 (s, 1H), 7.92 (d, *J* = 7.8 Hz, 2H), 7.55 – 7.48 (m, 3H), 7.43 – 7.35 (m, 2H), 7.19 – 7.13 (m, 4H), 7.00 (t, *J* = 7.6 Hz, 1H), 6.92 (d, *J* = 7.9 Hz, 2H), 5.73 – 5.68 (m, 1H), 2.83 (s, 3H), 2.71 (s, 3H), 1.68 (s, 3H), 1.35 (d, *J* = 4.5 Hz, 3H). *Mixture of diastereomers*: **^{13}C NMR** (201 MHz, $CDCl_3$) δ 165.93, 165.60, 162.49, 162.39, 160.48, 160.44, 156.20, 156.14, 150.70, 149.82, 149.08, 148.94, 141.26, 140.14, 133.03, 132.92, 132.75, 130.59, 130.46, 129.72, 129.66, 129.54, 129.50, 129.01, 128.93, 128.48, 128.39, 128.32, 128.29, 128.11, 127.41, 126.81, 126.16, 123.54, 123.23, 121.73, 121.31, 121.26, 121.03, 120.67, 74.67, 73.27, 72.29, 66.39, 65.67, 38.70, 38.01, 22.38, 19.37, 17.57, 17.54, 16.88, 16.79, 15.82, 15.74. **HRMS** (ESI-TOF) *m/z* calcd for $C_{29}H_{28}N_2O_4NaS$ [$(M + Na)^+$] 523.1667, found 523.1662.

4-(3-(benzoyloxy)-2-(methyl(phenyl)amino)butan-2-yl)phenyl 1-methyl-1H-pyrrole-2-carboxylate (3aw)

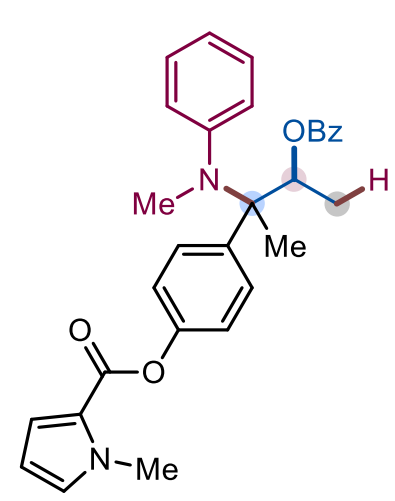

The reaction was performed according to General Procedure B at 40°C to afford the title compound (75.8 mg, 79% yield) as a white solid. **R$_f$** = 0.4 [Hex: EtOAc 20:1 (v/v)]. *Major diastereomer*: **^{1}H NMR** (800 MHz, $CDCl_3$) δ 7.84 (d, *J* = 7.5 Hz, 2H), 7.57 (d, *J* = 8.2 Hz, 2H), 7.55 – 7.48 (m, 2H), 7.38 (t, *J* = 7.6 Hz, 2H), 7.22 – 7.12 (m, 5H), 6.91 – 6.86 (m, 2H), 6.82 (t, *J* = 7.2 Hz, 1H), 6.21 (d, *J* = 3.4 Hz, 1H), 5.76 (q, *J* = 6.8, 6.4 Hz, 1H), 3.95 (s, 3H), 2.69 (s, 3H), 1.74 (s, 3H), 1.09 (d, *J* = 5.0 Hz, 3H). *Mixture of diastereomers*: **^{13}C NMR** (201 MHz, $CDCl_3$) δ 165.97, 165.63, 159.60, 159.58, 150.81, 149.92, 149.52, 149.36, 140.44, 139.41, 132.96, 132.88, 132.70, 130.68, 130.66, 130.60, 130.54, 129.66, 129.57, 129.51, 128.87, 128.75, 128.37, 128.29, 128.26, 128.09, 127.29, 126.90, 126.15, 123.51, 123.12, 122.08, 121.61, 121.58, 121.39, 121.03, 119.28, 119.24, 119.19, 108.38, 108.34, 74.69, 73.37, 72.42, 66.37, 65.69, 38.68, 38.04, 36.94, 36.91, 22.36, 19.44, 16.83, 15.76. **HRMS** (ESI-TOF) *m/z* calcd for $C_{30}H_{30}N_2O_4Na$ [(M + H)$^+$] 505.2103, found 505.2105.

3-(3-methoxyphenyl)-3-(methyl(phenyl)amino)butan-2-yl benzoate (3ax)

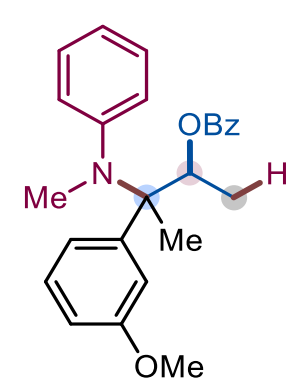

The reaction was performed according to General Procedure B at 40°C to afford the title compound (65.4 mg, 84% yield) as a colorless sticky oil. **R$_f$** = 0.4 [Hex: EtOAc 20:1 (v/v)]. *Mixture of diastereomer*: **^{1}H NMR** (500 MHz, $CDCl_3$) δ 7.96 – 7.91 (m, 2H), 7.86 – 7.80 (m, 2H), 7.56 – 7.48 (m, 2H), 7.43 – 7.34 (m, 4H), 7.29 (t, *J* = 7.9 Hz, 1H), 7.21 (t, *J* = 7.9 Hz, 1H), 7.18 – 7.09 (m, 4H), 7.09 – 7.01 (m, 6H), 7.00 – 6.95 (m, 1H), 6.95 – 6.88 (m, 2H), 6.83 (dd, *J* = 8.1, 2.5 Hz, 1H), 6.81 – 6.74 (m, 2H), 5.81 – 5.69 (m, 2H), 3.82 (s, 3H), 3.72 (s, 3H), 2.74 (s, 3H), 2.70 (s, 3H), 1.72 (s, 3H), 1.66 (s, 3H), 1.36 (d, *J* = 6.2 Hz, 3H), 1.08 (d, *J* = 6.3 Hz, 3H). *Mixture of diastereomers*: **^{13}C NMR** (126 MHz, $CDCl_3$) δ 165.98, 165.64, 159.54, 159.21, 150.95, 150.09, 145.23, 144.00, 132.84, 132.67, 130.76, 130.60, 129.57, 129.50, 128.92, 128.54, 128.32, 128.22, 128.17, 128.06, 126.64, 125.83, 123.27, 122.85, 120.27, 120.24, 114.04, 113.99, 112.11, 111.93, 75.00, 73.44, 66.65, 65.89, 55.26, 55.14, 38.63, 37.99, 19.54, 16.85, 16.67, 15.76. **HRMS** (ESI-TOF) *m/z* calcd for $C_{25}H_{28}NO_3$ [(M + H)$^+$] 390.2069, found 390.2062.

1-(methyl(phenyl)amino)-1-(p-tolyl)propan-2-yl benzoate (3ay)

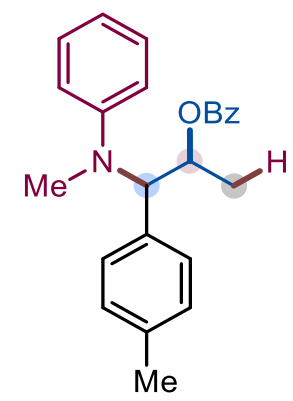

The reaction was performed according to General Procedure B at 80°C to afford the title compound (51.1 mg, 71% yield) as a yellow sticky oil. $\mathbf{R_f}$ = 0.4 [Hex: EtOAc 20:1 (v/v)]. *Major diastereomer*: **^{1}H NMR** (800 MHz, $CDCl_3$) δ 7.92 (d, J = 7.6 Hz, 2H), 7.55 (t, J = 7.6 Hz, 1H), 7.44 – 7.38 (m, 2H), 7.35 – 7.30 (m, 2H), 7.20 (d, J = 7.2 Hz, 2H), 7.09 (d, J = 7.7 Hz, 2H), 6.99 (d, J = 8.0 Hz, 2H), 6.82 (t, J = 7.2 Hz, 1H), 5.97 – 5.92 (m, 1H), 5.24 (d, J = 9.6 Hz, 1H), 2.68 (s, 3H), 2.30 (s, 3H), 1.57 – 1.52 (m, 3H). *Major diastereomer*: **^{13}C NMR** (201 MHz, $CDCl_3$) δ 166.13, 150.47, 137.05, 134.82, 133.01, 130.50, 129.71, 129.42, 129.39, 129.34, 129.15, 128.41, 127.78, 117.57, 117.44, 113.84, 112.59, 70.53, 65.61, 32.65, 30.90, 21.16, 18.54. **HRMS** (ESI-TOF) *m/z* calcd for $C_{24}H_{26}NO_2$ [$(M + H)^+$] 360.1964, found 360.1965.

1-(methyl(phenyl)amino)-1-(o-tolyl)propan-2-yl benzoate (3az)

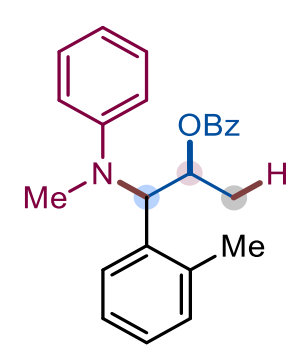

The reaction was performed according to General Procedure B at 80°C to afford the title compound (62.5 mg, 87% yield) as a colorless sticky oil. $\mathbf{R_f}$ = 0.4 [Hex: EtOAc 20:1 (v/v)]. *Major diastereomer*: **^{1}H NMR** (400 MHz, $CDCl_3$) δ 7.88 – 7.81 (m, 2H), 7.55 – 7.43 (m, 2H), 7.36 (t, J = 7.6 Hz, 2H), 7.32 – 7.27 (m, 2H), 7.14 (t, J = 4.2 Hz, 3H), 6.95 (d, J = 8.2 Hz, 2H), 6.76 (t, J = 7.3 Hz, 1H), 5.96 (dq, J = 8.6, 6.4 Hz, 1H), 5.33 (d, J = 8.8 Hz, 1H), 2.62 (s, 3H), 2.13 (s, 3H), 1.51 (d, J = 6.3 Hz, 3H). *Major diastereomer*: **^{13}C NMR** (126 MHz, $CDCl_3$) δ 166.15, 149.97, 138.51, 136.03, 133.01, 131.13, 130.55, 129.67, 129.44, 128.43, 127.48, 127.36, 125.66, 117.18, 112.91, 70.69, 61.53, 32.65, 20.78, 18.45. **HRMS** (ESI-TOF) *m/z* calcd for $C_{24}H_{26}NO_2$ [$(M + H)^+$] 360.1964, found 360.1963.

1-(4-isopropylphenyl)-1-(methyl(phenyl)amino)propan-2-yl benzoate (3aaa)

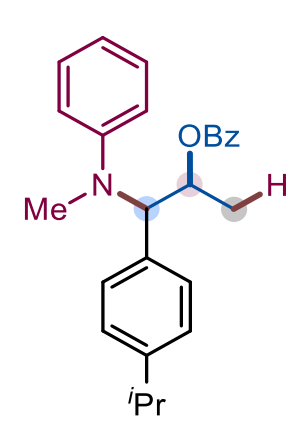

The reaction was performed according to General Procedure B at 80°C to afford the title compound (53.9 mg, 71% yield) as a yellow sticky oil. $\mathbf{R_f}$ = 0.4 [Hex: EtOAc 20:1 (v/v)]. *Major diastereomer*: ^{1}**H NMR** (800 MHz, $CDCl_3$) δ 7.90 (d, J = 7.8 Hz, 2H), 7.54 (t, J = 7.5 Hz, 1H), 7.40 (t, J = 7.8 Hz, 2H), 7.34 – 7.31 (m, 2H), 7.25 – 7.21 (m, 2H), 7.15 – 7.12 (m, 2H), 6.99 (d, J = 8.1 Hz, 2H), 6.82 (t, J = 7.4 Hz, 1H), 5.97 – 5.90 (m, 1H), 5.25 (d, J = 9.3 Hz, 1H), 2.90 – 2.80 (m, 1H), 2.69 (s, 3H), 1.55 – 1.52 (m, 3H), 1.21 (dd, J = 6.9, 3.3 Hz, 6H). *Major diastereomer*: ^{13}**C NMR** (201 MHz, $CDCl_3$) δ 166.19, 150.48, 147.95, 135.28, 132.99, 130.57, 129.81, 129.72, 129.43, 129.34, 129.25, 128.38, 128.31, 127.82, 126.48, 117.46, 113.67, 112.58, 70.79, 65.45, 33.76, 32.75, 24.01, 23.98, 18.55. **HRMS** (ESI-TOF) *m/z* calcd for $C_{26}H_{30}NO_2$ [(M + H)$^+$] 388.2277, found 388.2281.

1-(4-(tert-butyl)phenyl)-1-(methyl(phenyl)amino)propan-2-yl benzoate (3aab)

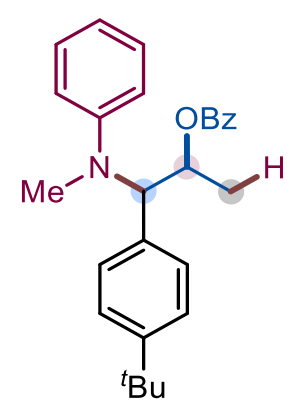

The reaction was performed according to General Procedure B at 80°C to afford the title compound (48.0 mg, 60% yield) as a colorless sticky oil. $\mathbf{R_f}$ = 0.4 [Hex: EtOAc 20:1 (v/v)]. *Major diastereomer*: ^{1}**H NMR** (500 MHz, $CDCl_3$) δ 7.91 – 7.82 (m, 2H), 7.53 – 7.48 (m, 1H), 7.36 (t, J = 7.8 Hz, 2H), 7.31 – 7.23 (m, 4H), 7.20 (d, J = 8.5 Hz, 2H), 6.95 (d, J = 8.2 Hz, 2H), 6.78 (t, J = 7.2 Hz, 1H), 5.89 (dq, J = 9.1, 6.2 Hz, 1H), 5.21 (d, J = 9.2 Hz, 1H), 2.66 (s, 3H), 1.49 (d, J = 6.2 Hz, 3H), 1.24 (s, 9H). *Major diastereomer*: ^{13}**C NMR** (126 MHz, $CDCl_3$) δ 166.21, 150.49, 150.25, 134.95, 132.99, 130.61, 129.74, 129.44, 128.38, 127.58, 125.35, 117.44, 113.63, 70.89, 65.33, 34.55, 32.82, 31.40, 18.56. **HRMS** (ESI-TOF) *m/z* calcd for $C_{27}H_{32}NO_2$ [(M + H)$^+$] 402.2433, found 402.2429.

1-(3,4-dimethylphenyl)-1-(methyl(phenyl)amino)propan-2-yl benzoate (3aac)

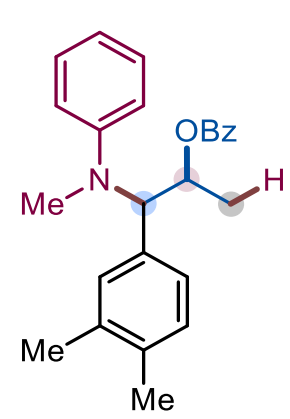

The reaction was performed according to General Procedure B at 80°C to afford the title compound (40.3 mg, 53% yield) as a colorless sticky oil. $\mathbf{R_f}$ = 0.4 [Hex: EtOAc 20:1 (v/v)]. *Major diastereomer*: ^{1}**H NMR**

(500 MHz, $CDCl_3$) δ 7.91 – 7.85 (m, 2H), 7.54 – 7.47 (m, 1H), 7.37 (t, *J* = 7.8 Hz, 2H), 7.28 (dd, *J* = 7.0, 1.7 Hz, 2H), 7.06 – 6.96 (m, 3H), 6.94 (d, *J* = 8.2 Hz, 2H), 6.82 – 6.74 (m, 1H), 5.88 (dq, *J* = 9.2, 6.2 Hz, 1H), 5.17 (d, *J* = 9.2 Hz, 1H), 2.66 (s, 3H), 2.17 (s, 3H), 2.15 (s, 3H), 1.47 (d, *J* = 6.2 Hz, 3H). *Major diastereomer*: **^{13}C NMR** (126 MHz, $CDCl_3$) δ 166.13, 150.46, 136.56, 135.71, 135.41, 132.98, 130.59, 129.73, 129.61, 129.42, 128.40, 125.16, 117.38, 113.61, 70.75, 65.33, 32.77, 19.97, 19.50, 18.55. **HRMS** (ESI-TOF) *m/z* calcd for $C_{25}H_{28}NO_2$ $[(M + H)^+]$ 374.2120, found 374.2115.

1-(benzo[*d*][1,3]dioxol-5-yl)-1-(methyl(phenyl)amino)propan-2-yl benzoate (3aad)

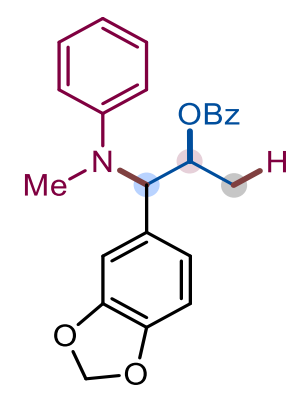

The reaction was performed according to General Procedure B at 80°C to afford the title compound (44.8 mg, 58% yield) as a yellow sticky oil. **R_f** = 0.6 [Hex: EtOAc 5:1 (v/v)]. *Mixture of diastereomers*: **^{1}H NMR** (500 MHz, $CDCl_3$) δ 7.93 – 7.87 (m, 2H), 7.83 – 7.76 (m, 2H), 7.56 – 7.45 (m, 2H), 7.42 – 7.36 (m, 2H), 7.35 – 7.30 (m, 2H), 7.30 – 7.26 (m, 2H), 7.25 – 7.17 (m, 2H), 6.98 – 6.90 (m, 2H), 6.91 – 6.86 (m, 2H), 6.82 – 6.71 (m, 7H), 6.67 (d, *J* = 8.0 Hz, 1H), 5.94 (s, 2H), 5.93 – 5.79 (m, 4H), 5.12 (d, *J* = 9.4 Hz, 1H), 5.05 (d, *J* = 8.8 Hz, 1H), 2.73 (s, 3H), 2.66 (s, 3H), 1.49 (d, *J* = 6.2 Hz, 3H), 1.38 (d, *J* = 6.2 Hz, 3H). *Mixture of diastereomers*: **^{13}C NMR** (126 MHz, $CDCl_3$) δ 166.28, 166.07, 151.15, 150.37, 148.03, 147.77, 147.09, 146.86, 133.08, 132.93, 131.77, 131.65, 130.46, 130.38, 129.81, 129.72, 129.46, 129.28, 128.46, 128.35, 121.53, 121.02, 117.83, 117.45, 114.10, 114.04, 108.78, 108.47, 108.37, 108.11, 101.24, 101.06, 70.55, 70.04, 66.94, 65.95, 32.66, 32.55, 18.81, 18.57. **HRMS** (ESI-TOF) *m/z* calcd for $C_{24}H_{24}NO_4$ $[(M + H)^+]$ 390.1705, found 390.1698.

1-(methyl(phenyl)amino)-1-(naphthalen-2-yl)propan-2-yl benzoate (3aae)

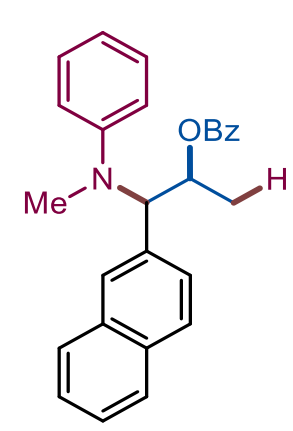

The reaction was performed according to General Procedure B at 80°C to afford the title compound (52.8 mg, 66% yield) as a colorless sticky oil. **R_f** = 0.4 [Hex: EtOAc 20:1 (v/v)]. *Major diastereomer*: **^{1}H NMR** (800 MHz, $CDCl_3$) δ 7.94 (d, *J* = 7.7 Hz, 2H), 7.84 – 7.75 (m, 4H), 7.53 (t, *J* = 7.7 Hz, 1H), 7.50 – 7.47 (m,

2H), 7.44 (d, *J* = 8.7 Hz, 1H), 7.41 – 7.35 (m, 4H), 7.06 (d, *J* = 8.0 Hz, 2H), 6.88 (t, *J* = 7.6 Hz, 1H), 6.16 – 6.06 (m, 1H), 5.47 (d, *J* = 9.4 Hz, 1H), 2.73 (s, 3H), 1.62 (d, *J* = 5.6 Hz, 3H). *Major diastereomer*: ^{13}**C NMR** (201 MHz, $CDCl_3$) δ 166.18, 150.43, 135.55, 133.29, 133.06, 132.83, 130.37, 129.83, 129.68, 129.52, 129.35, 128.43, 128.34, 128.26, 128.17, 127.61, 126.56, 126.28, 126.14, 126.01, 117.76, 113.84, 70.50, 65.83, 32.78, 18.57. **HRMS** (ESI-TOF) *m/z* calcd for $C_{27}H_{26}NO_2$ [$(M + H)^+$] 396.1964, found 396.1964.

1-(4-fluoro-3-methoxyphenyl)-1-(methyl(phenyl)amino)propan-2-yl 4-methoxybenzoate (3aaf)

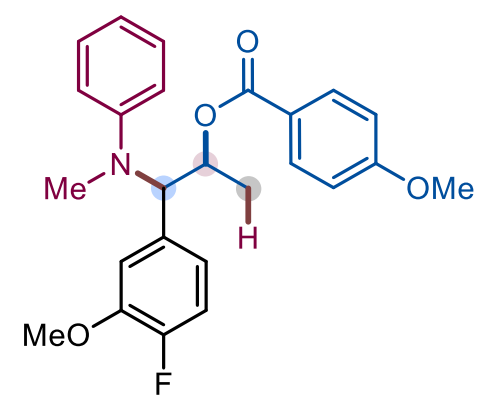

The reaction was performed according to General Procedure B at 80°C to afford the title compound (70.2 mg, 83% yield) as a yellow sticky oil. $\mathbf{R_f}$ = 0.3 [Hex: EtOAc 20:1 (v/v)]. *Major diastereomer*: ^{1}**H NMR** (500 MHz, $CDCl_3$) δ 7.84 (d, *J* = 8.9 Hz, 2H), 7.33 – 7.25 (m, 2H), 6.99 – 6.89 (m, 3H), 6.89 – 6.71 (m, 5H), 5.87 (dq, *J* = 9.6, 6.2 Hz, 1H), 5.13 (d, *J* = 9.5 Hz, 1H), 3.83 (s, 3H), 3.70 (s, 3H), 2.63 (s, 3H), 1.50 (d, *J* = 6.2 Hz, 3H). *Major diastereomer*: ^{13}**C NMR** (126 MHz, $CDCl_3$) δ 165.83, 163.63, 152.67, 150.45, 147.33, 134.29, 131.71, 129.49, 122.67, 120.27, 120.21, 118.10, 115.89, 115.75, 114.32, 113.78, 113.12, 69.84, 66.11, 56.23, 55.57, 32.68, 18.69. *Major diastereomer*: ^{19}**F NMR** (470 MHz, $CDCl_3$) δ -136.61 – -138.04 (m). **HRMS** (ESI-TOF) *m/z* calcd for $C_{25}H_{27}NO_4F$ [$(M + H)^+$] 424.1924, found 424.1923.

1-(methyl(phenyl)amino)-1-phenylpropan-2-yl 4-methoxybenzoate (3aag)

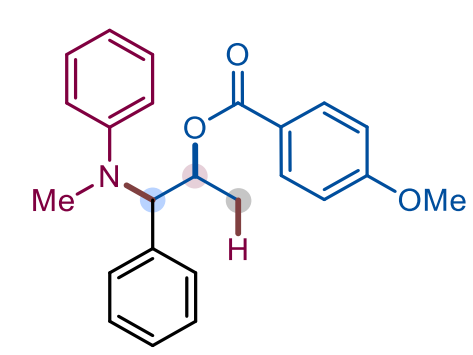

The reaction was performed according to General Procedure B at 80°C to afford the title compound (72.1 mg, 96% yield) as a colorless sticky oil. $\mathbf{R_f}$ = 0.3 [Hex: EtOAc 20:1 (v/v)]. *Major diastereomer*: ^{1}**H NMR** (500 MHz, $CDCl_3$) δ 7.82 (d, *J* = 8.8 Hz, 2H), 7.31 – 7.16 (m, 7H), 6.95 (d, *J* = 8.3 Hz, 2H), 6.86 – 6.82 (m, 2H), 6.82 – 6.76 (m, 1H), 5.89 (dq, *J* = 9.4, 6.2 Hz, 1H), 5.22 (d, *J* = 9.4 Hz, 1H), 3.82 (s, 3H), 2.65 (s, 3H), 1.50 (d, *J* = 6.2 Hz, 3H). *Major diastereomer*: ^{13}**C NMR** (126 MHz, $CDCl_3$) δ 165.74, 163.38, 150.36, 137.92, 131.61, 129.32, 129.16, 128.59, 128.32, 128.23, 127.80, 127.33, 122.75, 117.54, 113.76, 113.56, 113.48, 69.96, 65.88, 55.42, 32.59, 18.49.

3-(4-acetoxyphenyl)-3-(methyl(4-(trifluoromethoxy)phenyl)amino)butan-2-yl 2-methylbenzoate (3aah)

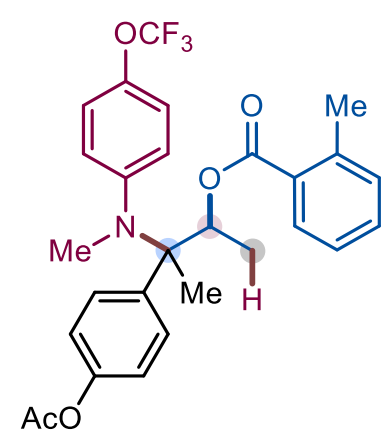

The reaction was performed according to General Procedure B at 40°C to afford the title compound (70.1 mg, 68% yield) as a colorless sticky oil. **R_f** = 0.4 [Hex: EtOAc 10:1 (v/v)]. *Major diastereomer*: **^{1}H NMR** (300 MHz, $CDCl_3$) δ 7.64 (dd, *J* = 7.8, 1.5 Hz, 1H), 7.50 – 7.43 (m, 2H), 7.42 – 7.31 (m, 2H), 7.24 – 6.80 (m, 7H), 5.82 – 5.70 (m, 1H), 2.68 (s, 3H), 2.54 (s, 3H), 2.31 (s, 3H), 1.69 (s, 3H), 1.11 (d, *J* = 6.3 Hz, 3H). *Mixture of diastereomers*: **^{13}C NMR** (201 MHz, $CDCl_3$) δ 169.43, 169.38, 167.06, 166.59, 149.82, 149.71, 149.44, 148.66, 144.98, 144.87, 140.35, 140.09, 139.04, 132.03, 131.97, 131.79, 131.71, 130.41, 130.28, 130.17, 129.71, 128.83, 128.80, 128.65, 127.63, 127.12, 125.85, 125.67, 122.04, 121.25, 121.16, 120.89, 120.75, 119.89, 74.21, 72.78, 66.42, 65.61, 38.73, 38.14, 21.81, 21.48, 21.33, 19.60, 17.66, 16.98, 15.75. *Major diastereomer*: **^{19}F NMR** (282 MHz, $CDCl_3$) δ -58.10. **HRMS** (ESI-TOF) *m/z* calcd for $C_{28}H_{29}NO_5F_3$ $[(M + H)^+]$ 516.1998, found 516.1993.

3-(4-acetoxyphenyl)-3-(methyl(4-(trifluoromethoxy)phenyl)amino)butan-2-yl 3-methylbenzoate (3aai)

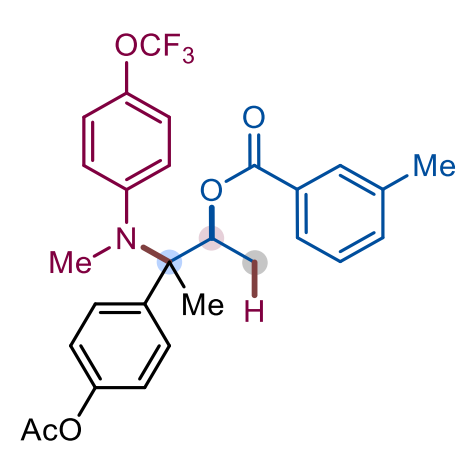

The reaction was performed according to General Procedure B at 40°C to afford the title compound (82.5 mg, 80% yield) as a white solid. **R_f** = 0.4 [Hex: EtOAc 10:1 (v/v)]. *Major diastereomer*: **^{1}H NMR** (400 MHz, $CDCl_3$) δ 7.65 – 7.58 (m, 2H), 7.53 – 7.47 (m, 2H), 7.37 – 7.30 (m, 1H), 7.30 – 7.23 (m, 1H), 7.16 – 7.09 (m, 2H), 7.03 – 6.95 (m, 2H), 6.94 – 6.84 (m, 2H), 5.78 – 5.65 (m, 1H), 2.67 (s, 3H), 2.38 (s, 3H), 2.31 (s, 3H), 1.72 (s, 3H), 1.06 (d, *J* = 6.3 Hz, 3H). *Mixture of diastereomers*: **^{13}C NMR** (201 MHz, $CDCl_3$) δ 169.41, 165.85, 149.84, 149.70, 149.48, 148.61, 145.00, 140.55, 138.32, 138.12, 133.90, 133.78, 130.51, 130.31, 130.26, 130.10, 128.95, 128.72, 128.43, 128.22, 127.59, 126.76, 126.62, 126.60, 121.25, 121.11, 120.93, 120.88, 120.79, 119.83, 74.58, 73.20, 66.58, 65.90, 38.76, 38.16, 21.43, 21.36, 21.34, 19.56, 16.98, 16.80,

15.90. *Major diastereomer*: **^{19}F NMR** (376 MHz, $CDCl_3$) δ -59.71. **HRMS** (ESI-TOF) *m/z* calcd for $C_{28}H_{29}NO_5F_3$ [(M + H)$^+$] 516.1998, found 516.1999.

3-(4-acetoxyphenyl)-3-(methyl(4-(trifluoromethoxy)phenyl)amino)butan-2-yl 4-methylbenzoate (3aaj)

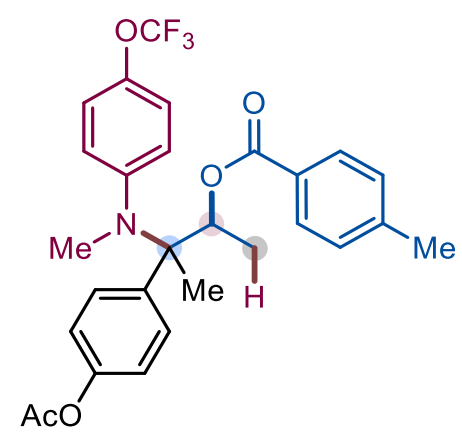

The reaction was performed according to General Procedure B at 40°C to afford the title compound (74.2 mg, 72% yield) as a white solid. **R_f** = 0.4 [Hex: EtOAc 10:1 (v/v)]. *Major diastereomer*: **^{1}H NMR** (400 MHz, $CDCl_3$) δ 7.77 (d, *J* = 7.9 Hz, 2H), 7.58 – 7.51 (m, 2H), 7.27 – 7.20 (m, 2H), 7.20 – 7.12 (m, 2H), 7.07 – 6.99 (m, 2H), 6.95 (d, *J* = 8.6 Hz, 2H), 5.77 (q, *J* = 6.2 Hz, 1H), 2.71 (s, 3H), 2.44 (s, 3H), 2.36 (s, 3H), 1.76 (s, 3H), 1.11 (d, *J* = 6.3 Hz, 3H). *Mixture of diastereomers*: **^{13}C NMR** (126 MHz, $CDCl_3$) δ 169.39, 166.06, 165.73, 149.83, 149.49, 144.96, 143.82, 143.71, 140.57, 139.45, 129.69, 129.55, 129.25, 129.05, 128.90, 128.71, 127.69, 127.53, 126.56, 121.23, 120.89, 120.79, 74.40, 73.11, 66.57, 65.89, 38.78, 38.16, 21.75, 21.32, 19.68, 16.98, 15.91. *Major diastereomer*: **^{19}F NMR** (470 MHz, $CDCl_3$) δ -58.18. **HRMS** (ESI-TOF) *m/z* calcd for $C_{28}H_{29}NO_5F_3$ [(M + H)$^+$] 516.1998, found 516.2004.

3-(4-acetoxyphenyl)-3-(methyl(phenyl)amino)butan-2-yl 4-fluorobenzoate (3aak)

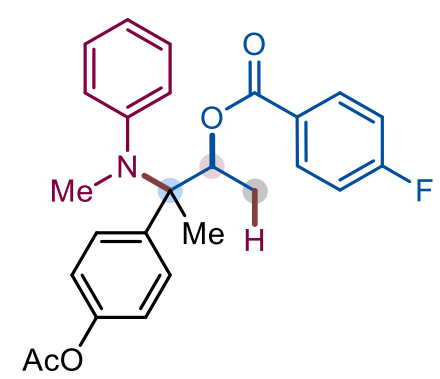

The reaction was performed according to General Procedure B at 40°C to afford the title compound (57.5 mg, 66% yield) as a yellow sticky oil. **R_f** = 0.4 [Hex: EtOAc 10:1 (v/v)]. *Major diastereomer*: **^{1}H NMR** (500 MHz, $CDCl_3$) δ 7.83 – 7.77 (m, 2H), 7.57 – 7.50 (m, 2H), 7.22 – 6.97 (m, 7H), 6.95 – 6.89 (m, 1H), 6.83 – 6.75 (m, 1H), 5.72 – 5.63 (m, 1H), 2.65 (s, 3H), 2.31 (s, 3H), 1.72 (s, 3H), 1.05 (d, *J* = 6.3 Hz, 3H). *Mixture of diastereomers*: **^{13}C NMR** (126 MHz, $CDCl_3$) δ 169.43, 166.77, 165.10, 164.73, 150.94, 149.99, 149.77, 149.60, 140.84, 139.57, 132.20, 132.11, 132.04, 128.97, 128.83, 128.45, 128.41, 127.46, 127.11, 126.86, 126.51, 123.78, 123.53, 121.84, 121.13, 120.74, 115.72, 115.54, 115.38, 115.20, 75.19, 73.60, 66.53, 65.76,

38.83, 38.20, 21.34, 19.40, 16.98, 16.54, 15.86. *Major diastereomer*: ^{19}**F NMR** (470 MHz, $CDCl_3$) δ -106.17 (p, J = 6.9 Hz). **HRMS** (ESI-TOF) *m/z* calcd for $C_{26}H_{26}NO_4FNa$ [$(M + Na)^+$] 458.1744, found 458.1740.

3-(4-acetoxyphenyl)-3-(methyl(4-(trifluoromethoxy)phenyl)amino)butan-2-yl 4-chlorobenzoate (3aal)

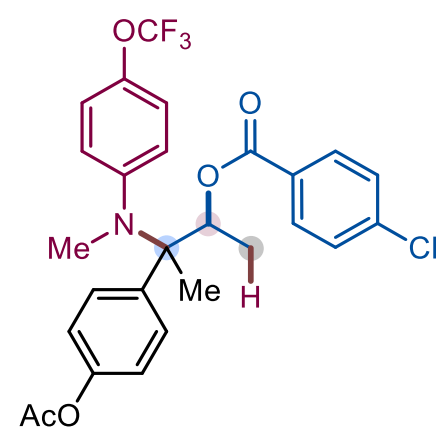

The reaction was performed according to General Procedure B at 40°C to afford the title compound (80.4 mg, 75% yield) as a yellow sticky oil. $\mathbf{R}_f$ = 0.4 [Hex: EtOAc 10:1 (v/v)]. *Major diastereomer*: ^{1}**H NMR** (300 MHz, $CDCl_3$) δ 7.76 – 7.69 (m, 2H), 7.56 – 7.46 (m, 2H), 7.37 – 7.30 (m, 2H), 7.15 – 7.08 (m, 2H), 7.08 – 6.96 (m, 2H), 6.94 – 6.80 (m, 2H), 5.75 – 5.63 (m, 1H), 2.63 (s, 3H), 2.31 (s, 3H), 1.71 (s, 3H), 1.04 (d, J = 6.3 Hz, 3H). *Mixture of diastereomers*: ^{13}**C NMR** (201 MHz, $CDCl_3$) δ 169.28, 164.60, 149.78, 149.34, 145.08, 144.73, 140.33, 139.47, 139.36, 138.93, 130.87, 130.69, 128.77, 128.73, 128.65, 128.54, 127.78, 126.91, 121.21, 120.84, 120.80, 120.75, 74.99, 73.40, 66.49, 65.67, 38.68, 38.13, 21.20, 19.33, 16.90, 16.24, 15.73. *Major diastereomer*: ^{19}**F NMR** (282 MHz, $CDCl_3$) δ -58.25. **HRMS** (ESI-TOF) *m/z* calcd for $C_{27}H_{26}NO_5F_3Cl$ [$(M + H)^+$] 536.1452, found 536.1455.

3-(4-acetoxyphenyl)-3-(methyl(4-(trifluoromethoxy)phenyl)amino)butan-2-yl 4-(trifluoromethoxy)benzoate (3aam)

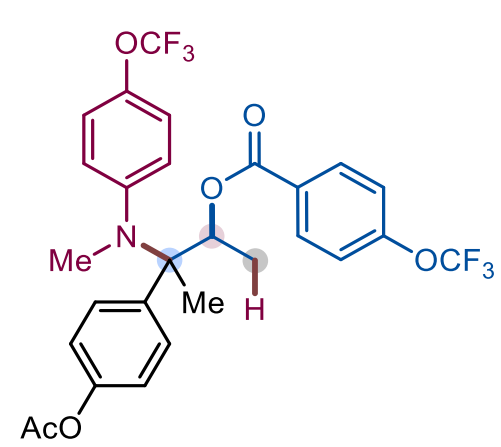

The reaction was performed according to General Procedure B at 40°C to afford the title compound (86.7 mg, 74% yield) as a colorless sticky oil. $\mathbf{R}_f$ = 0.4 [Hex: EtOAc 10:1 (v/v)]. *Major diastereomer*: ^{1}**H NMR** (400 MHz, $CDCl_3$) δ 7.83 – 7.78 (m, 2H), 7.56 – 7.51 (m, 2H), 7.21 – 7.18 (m, 2H), 7.15 – 7.11 (m, 2H), 7.09 – 7.04 (m, 2H), 6.92 – 6.87 (m, 2H), 5.69 (q, J = 6.3 Hz, 1H), 2.61 (s, 3H), 2.32 (s, 3H), 1.73 (s, 3H), 1.02 (d, J = 6.4 Hz, 3H). *Mixture of diastereomers*: ^{13}**C NMR** (201 MHz, $CDCl_3$) δ 169.28, 164.68, 164.22, 152.58, 149.81, 149.63, 149.40, 145.22, 140.47, 131.47, 131.23, 128.73, 128.56, 128.51, 127.96, 126.97,

121.27, 120.83, 120.77, 120.31, 120.14, 75.34, 73.46, 66.59, 65.66, 38.71, 38.13, 21.20, 21.18, 19.31, 16.98, 15.72, 15.61. *Major diastereomer*: **^{19}F NMR** (376 MHz, $CDCl_3$) δ -59.26, -59.92. **HRMS** (ESI-TOF) *m/z* calcd for $C_{28}H_{26}NO_6F_6$ $[(M + H)^+]$ 586.1664, found 586.1666.

3-(4-acetoxyphenyl)-3-(methyl(4-(trifluoromethoxy)phenyl)amino)butan-2-yl 3-(trifluoromethoxy)benzoate (3aan)

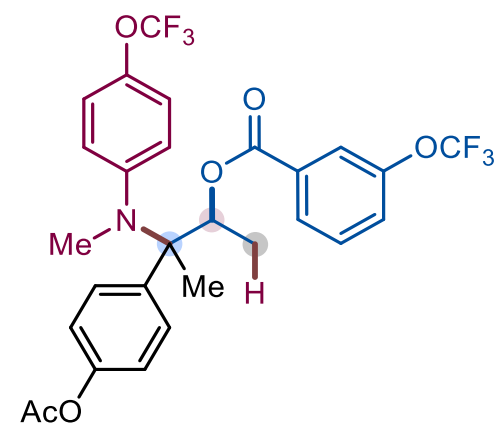

The reaction was performed according to General Procedure B at 40°C to afford the title compound (90.2 mg, 77% yield) as a yellow sticky oil. **R*f*** = 0.4 [Hex: EtOAc 10:1 (v/v)]. *Major diastereomer*: **^{1}H NMR** (400 MHz, $CDCl_3$) δ 7.74 – 7.68 (m, 1H), 7.63 – 7.59 (m, 1H), 7.57 – 7.50 (m, 2H), 7.42 – 7.39 (m, 1H), 7.39 – 7.37 (m, 1H), 7.16 – 7.11 (m, 2H), 7.10 – 7.03 (m, 2H), 6.92 – 6.84 (m, 2H), 5.73 – 5.61 (m, 1H), 2.59 (s, 3H), 2.32 (s, 3H), 1.74 (s, 3H), 1.01 (d, *J* = 6.4 Hz, 3H). *Mixture of diastereomers*: **^{13}C NMR** (201 MHz, $CDCl_3$) δ 169.27, 164.47, 163.95, 149.84, 149.69, 149.35, 149.02, 145.32, 140.43, 132.46, 132.13, 129.98, 129.72, 128.81, 128.51, 128.11, 127.76, 127.63, 127.06, 125.39, 125.31, 121.82, 121.63, 121.28, 121.07, 120.87, 120.83, 120.78, 119.78, 119.60, 75.49, 73.81, 66.59, 65.73, 38.66, 38.24, 21.20, 19.09, 16.96, 15.77, 15.44. *Major diastereomer*: **^{19}F NMR** (376 MHz, $CDCl_3$) δ -59.44, -59.87. **HRMS** (ESI-TOF) *m/z* calcd for $C_{28}H_{26}NO_6F_6$ $[(M + H)^+]$ 586.1664, found 586.1666.

3-(4-acetoxyphenyl)-3-(methyl(4-(trifluoromethoxy)phenyl)amino)butan-2-yl 3-methoxybenzoate (3aao)

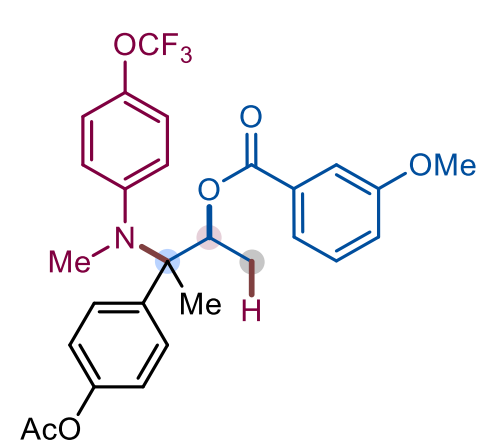

The reaction was performed according to General Procedure B at 40°C to afford the title compound (75.5 mg, 71% yield) as a yellow sticky oil. **R*f*** = 0.4 [Hex: EtOAc 10:1 (v/v)]. *Major diastereomer*: **^{1}H NMR** (400 MHz, $CDCl_3$) δ 7.54 – 7.48 (m, 2H), 7.36 (dd, *J* = 2.7, 1.5 Hz, 1H), 7.33 – 7.28 (m, 1H), 7.14 – 7.10 (m, 2H), 7.09 – 7.04 (m, 2H), 7.03 – 6.99 (m, 2H), 6.95 – 6.89 (m, 2H), 5.73 (p, *J* = 6.2 Hz, 1H), 3.83 (s, 3H),

2.66 (s, 3H), 2.31 (s, 3H), 1.72 (s, 3H), 1.08 (t, J = 6.3 Hz, 4H). *Mixture of diastereomers*: **^{13}C NMR** (201 MHz, $CDCl_3$) δ 169.41, 165.51, 159.88, 159.57, 149.86, 149.47, 145.07, 140.48, 131.69, 129.80, 129.58, 129.36, 128.98, 128.84, 128.73, 127.69, 126.78, 122.74, 121.92, 121.44, 121.27, 121.06, 120.95, 120.89, 120.86, 119.68, 119.44, 114.67, 114.13, 74.78, 73.44, 66.58, 55.53, 38.80, 38.23, 21.34, 19.46, 16.97, 16.76, 15.92. *Major diastereomer*: **^{19}F NMR** (282 MHz, $CDCl_3$) δ -58.18. **HRMS** (ESI-TOF) *m/z* calcd for $C_{28}H_{29}NO_6F_3$ [(M + H)$^+$] 532.1947, found 532.1942.

3-(4-acetoxyphenyl)-3-(methyl(4-(trifluoromethoxy)phenyl)amino)butan-2-yl 3-fluorobenzoate (3aap)

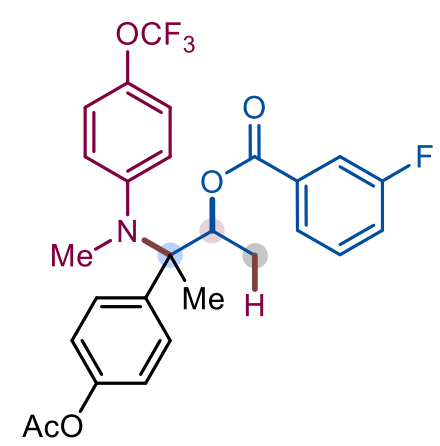

The reaction was performed according to General Procedure B at 40°C to afford the title compound (55.1 mg, 53% yield) as a yellow sticky oil. **R_f** = 0.4 [Hex: EtOAc 10:1 (v/v)]. *Major diastereomer*: **^{1}H NMR** (300 MHz, $CDCl_3$) δ 7.62 – 7.58 (m, 1H), 7.55 – 7.49 (m, 2H), 7.48 – 7.41 (m, 1H), 7.40 – 7.29 (m, 1H), 7.25 – 7.16 (m, 1H), 7.16 – 7.09 (m, 2H), 7.09 – 6.96 (m, 2H), 6.95 – 6.83 (m, 2H), 5.76 – 5.64 (m, 1H), 2.61 (s, 3H), 2.32 (s, 3H), 1.73 (s, 3H), 1.03 (d, J = 6.3 Hz, 3H). *Mixture of diastereomers*: **^{13}C NMR** (201 MHz, $CDCl_3$) δ 169.28, 164.30, 163.01, 161.78, 149.80, 149.35, 145.21, 143.82, 140.37, 137.63, 132.32, 130.09, 129.85, 129.81, 128.75, 128.52, 127.94, 126.93, 125.23, 125.05, 121.24, 120.92, 120.84, 120.82, 120.76, 119.98, 119.88, 119.64, 116.45, 116.21, 116.09, 75.22, 73.57, 66.53, 65.69, 38.68, 38.15, 21.20, 19.26, 16.93, 15.88, 15.71. *Major diastereomer*: **^{19}F NMR** (282 MHz, $CDCl_3$) δ -58.26, -112.50 – -112.96 (m). **HRMS** (ESI-TOF) *m/z* calcd for $C_{27}H_{26}NO_5F_4$ [(M + H)$^+$] 520.1747, found 520.1743.

3-(4-acetoxyphenyl)-3-(methyl(4-(trifluoromethoxy)phenyl)amino)butan-2-yl 3-bromobenzoate (3aaq)

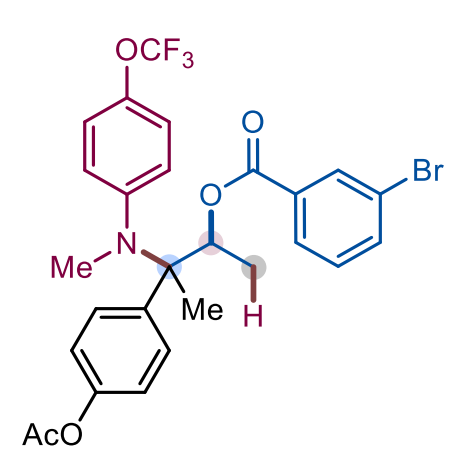

The reaction was performed according to General Procedure B at 40°C to afford the title compound (63.8 mg, 55% yield) as a yellow sticky oil. **R$_f$** = 0.4 [Hex: EtOAc 10:1 (v/v)]. *Major diastereomer*: **^{1}H NMR** (400 MHz, $CDCl_3$) δ 7.96 (d, *J* = 1.9 Hz, 1H), 7.79 (d, *J* = 7.8 Hz, 1H), 7.73 – 7.68 (m, 1H), 7.61 – 7.55 (m, 2H), 7.34 – 7.31 (m, 1H), 7.24 – 7.17 (m, 2H), 7.15 – 7.08 (m, 2H), 7.02 – 6.95 (m, 2H), 5.76 (q, *J* = 6.3 Hz, 1H), 2.68 (s, 3H), 2.38 (s, 3H), 1.80 (s, 3H), 1.09 (d, *J* = 6.3 Hz, 3H). *Mixture of diastereomers*: **^{13}C NMR** (201 MHz, $CDCl_3$) δ 169.28, 164.12, 149.81, 149.35, 145.22, 140.33, 135.95, 135.81, 132.57, 132.33, 132.07, 130.00, 129.77, 128.82, 128.53, 128.04, 127.92, 127.86, 126.98, 122.25, 121.25, 120.87, 120.82, 120.76, 75.31, 73.63, 66.53, 65.75, 38.68, 38.17, 21.21, 19.11, 16.91, 15.86, 15.75. *Mixture of diastereomers*: **^{19}F NMR** (376 MHz, $CDCl_3$) δ -59.73. **HRMS** (ESI-TOF) *m/z* calcd for $C_{27}H_{26}NO_5F_3Br$ $[(M + H)^+]$ 580.0946, found 580.0941.

3-(4-acetoxyphenyl)-3-(methyl(phenyl)amino)butan-2-yl 2-naphthoate (3aar)

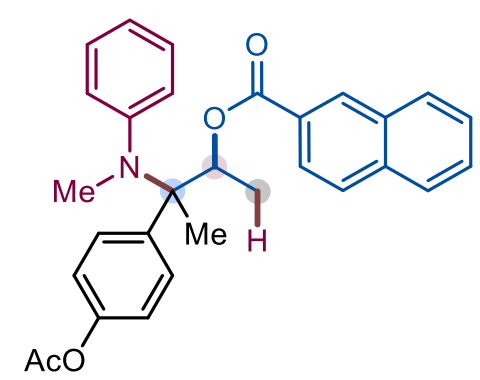

The reaction was performed according to General Procedure B at 40°C to afford the title compound (35.3 mg, 43% yield) as a yellow sticky oil. **R$_f$** = 0.4 [Hex: EtOAc 10:1 (v/v)]. *Major diastereomer*: **^{1}H NMR** (500 MHz, $CDCl_3$) δ 8.35 (s, 1H), 7.89 – 7.80 (m, 4H), 7.63 – 7.50 (m, 3H), 7.11 – 7.03 (m, 6H), 6.97 – 6.92 (m, 1H), 6.78 – 6.72 (m, 1H), 5.79 (q, *J* = 6.3 Hz, 1H), 2.71 (s, 3H), 2.32 (s, 3H), 1.79 (s, 3H), 1.12 (d, *J* = 6.3 Hz, 3H). *Mixture of diastereomers*: **^{13}C NMR** (126 MHz, $CDCl_3$) δ 169.32, 166.08, 165.77, 150.83, 149.92, 149.64, 140.76, 135.47, 132.36, 131.06, 130.95, 129.40, 129.03, 128.82, 128.31, 128.23, 128.14, 127.83, 127.75, 126.80, 126.63, 126.54, 126.28, 125.24, 125.19, 123.49, 123.27, 120.97, 120.60, 74.94, 73.52, 66.44, 65.83, 38.74, 38.13, 21.24, 19.30, 16.88, 16.80, 15.87. **HRMS** (ESI-TOF) *m/z* calcd for $C_{30}H_{29}NO_4Na$ $[(M + Na)^+]$ 490.1994, found 490.1995.

3-(methyl(4-(trifluoromethoxy)phenyl)amino)-3-phenylbutan-2-yl 4-methoxybenzoate (3aas)

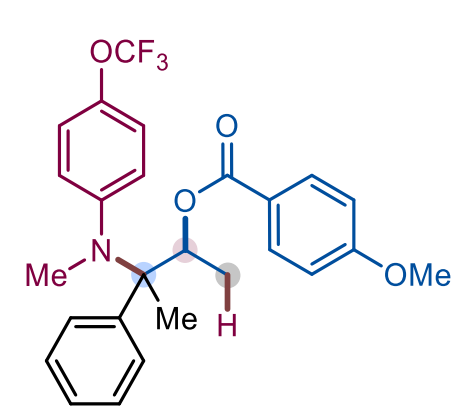

The reaction was performed according to General Procedure B at 40°C to afford the title compound (75.8 mg, 80% yield) as a yellow sticky oil. $\mathbf{R}_f$ = 0.4 [Hex: EtOAc 10:1 (v/v)]. *Major diastereomer*: **^{1}H NMR** (300 MHz, $CDCl_3$) δ 7.97 – 7.82 (m, 2H), 7.45 – 7.36 (m, 2H), 7.34 – 7.17 (m, 3H), 7.02 – 6.93 (m, 2H), 6.91 – 6.79 (m, 4H), 5.72 (q, J = 6.2 Hz, 1H), 3.84 (s, 3H), 2.75 (s, 3H), 1.66 (s, 3H), 1.33 (d, J = 6.2 Hz, 3H). *Major diastereomer*: **^{13}C NMR** (201 MHz, $CDCl_3$) δ 165.78, 163.51, 131.71, 128.00, 127.84, 127.05, 126.05, 123.09, 120.83, 113.76, 73.08, 55.58, 38.09, 19.70, 15.94. *Major diastereomer*: **^{19}F NMR** (282 MHz, $CDCl_3$) δ -58.06. **HRMS** (ESI-TOF) *m/z* calcd for $C_{26}H_{27}NO_4F_3$ [(M + H)$^+$] 474.1892, found 474.1888.

1-(4-isopropylphenyl)-1-(methyl(phenyl)amino)propan-2-yl 3-(trifluoromethyl)benzoate (3aat)

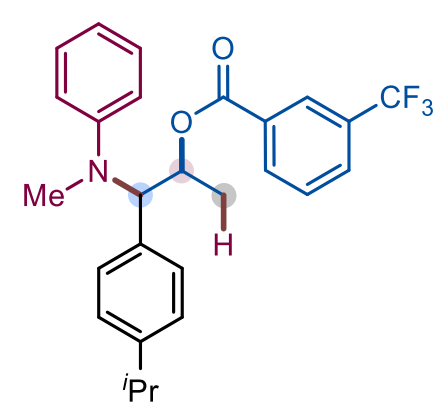

The reaction was performed according to General Procedure B at 80°C to afford the title compound (41.3 mg, 50% yield) as a colorless sticky oil. $\mathbf{R}_f$ = 0.4 [Hex: EtOAc 20:1 (v/v)]. *Major diastereomer*: **^{1}H NMR** (500 MHz, $CDCl_3$) δ 8.06 (s, 1H), 8.01 (d, J = 7.8 Hz, 1H), 7.75 (dd, J = 7.9, 1.6 Hz, 1H), 7.49 (t, J = 7.8 Hz, 1H), 7.28 (dd, J = 8.8, 7.2 Hz, 2H), 7.18 (d, J = 8.1 Hz, 2H), 7.10 (d, J = 8.2 Hz, 2H), 6.95 (d, J = 8.2 Hz, 2H), 6.82 – 6.75 (m, 1H), 5.91 (dq, J = 9.4, 6.2 Hz, 1H), 5.20 (d, J = 9.3 Hz, 1H), 2.82 (p, J = 6.9 Hz, 1H), 2.68 (s, 3H), 1.52 (d, J = 6.2 Hz, 3H), 1.16 (dd, J = 6.9, 2.2 Hz, 6H). *Major diastereomer*: **^{13}C NMR** (126 MHz, $CDCl_3$) δ 164.94, 148.19, 135.02, 132.88, 131.40, 129.45, 129.06, 127.80, 126.55, 117.74, 113.94, 71.46, 65.78, 33.77, 32.84, 23.97, 23.93, 18.55. *Major diastereomer*: **^{19}F NMR** (470 MHz, $CDCl_3$) δ -62.83. **HRMS** (ESI-TOF) *m/z* calcd for $C_{27}H_{29}NO_2F_3$ [(M + H)$^+$] 456.2150, found 456.2145.

1-(4-isopropylphenyl)-1-(methyl(phenyl)amino)propan-2-yl cyclohexanecarboxylate (3aau)

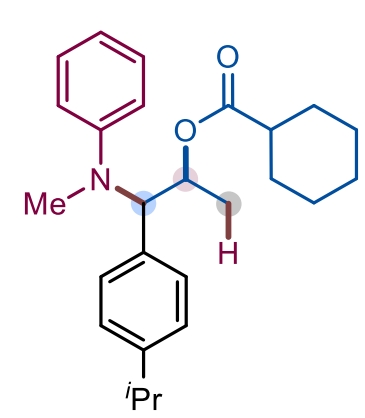

The reaction was performed according to General Procedure B at 80°C to afford the title compound (48.1 mg, 59% yield) as a white solid. $\mathbf{R}_f$ = 0.6 [Hex: EtOAc 20:1 (v/v)]. *Major diastereomer*: **^{1}H NMR** (400 MHz, $CDCl_3$) δ 7.30 – 7.22 (m, 2H), 7.11 (s, 4H), 6.91 (d, J = 8.1 Hz, 2H), 6.80 – 6.72 (m, 1H), 5.69 (dq, J = 10.4,

6.1 Hz, 1H), 5.00 (d, *J* = 9.9 Hz, 1H), 2.91 – 2.81 (m, 1H), 2.62 (s, 3H), 2.21 – 2.05 (m, 1H), 1.79 – 1.59 (m, 2H), 1.59 – 1.44 (m, 2H), 1.35 (d, *J* = 6.2 Hz, 3H), 1.31 – 1.07 (m, 12H). *Major diastereomer*: **^{13}C NMR** (201 MHz, $CDCl_3$) δ 175.50, 150.49, 148.04, 135.16, 129.36, 128.01, 126.32, 117.45, 113.82, 112.61, 68.99, 65.78, 43.31, 33.84, 32.40, 28.75, 28.70, 25.83, 25.40, 25.33, 24.09, 24.07, 18.47. **HRMS** (ESI-TOF) *m/z* calcd for $C_{26}H_{36}NO_2$ [$(M + H)^+$] 394.2746, found 394.2738.

1-(4-isopropylphenyl)-1-(methyl(4-(trifluoromethoxy)phenyl)amino)propan-2-yl thiophene-2-carboxylate (3aav)

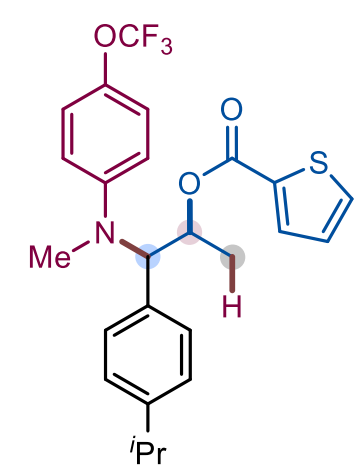

The reaction was performed according to General Procedure B at 80°C to afford the title compound (52.5 mg, 55% yield) as a yellow sticky oil. **R_f** = 0.5 [Hex: EtOAc 20:1 (v/v)]. *Major diastereomer*: **^{1}H NMR** (400 MHz, $CDCl_3$) δ 7.66 (dd, *J* = 3.8, 1.3 Hz, 1H), 7.51 (dd, *J* = 5.0, 1.3 Hz, 1H), 7.23 – 7.17 (m, 2H), 7.15 – 7.09 (m, 4H), 7.04 (dd, *J* = 5.0, 3.8 Hz, 1H), 6.89 – 6.84 (m, 2H), 5.82 (dq, *J* = 8.8, 6.2 Hz, 1H), 5.08 (d, *J* = 8.8 Hz, 1H), 2.93 – 2.77 (m, 1H), 2.67 (s, 3H), 1.48 (d, *J* = 6.2 Hz, 3H), 1.19 (dd, *J* = 6.9, 1.0 Hz, 6H). *Major diastereomer*: **^{13}C NMR** (126 MHz, $CDCl_3$) δ 161.68, 149.12, 148.24, 140.56, 134.74, 133.97, 133.64, 132.64, 127.91, 127.82, 126.60, 122.39, 114.01, 71.13, 65.78, 33.79, 33.32, 24.01, 23.97, 18.54. *Major diastereomer*: **^{19}F NMR** (376 MHz, $CDCl_3$) δ -59.88. **HRMS** (ESI-TOF) *m/z* calcd for $C_{25}H_{27}NO_3SF_3$ [$(M + H)^+$] 478.1664, found 478.1664.

4-(3-(benzoyloxy)-2-(methyl(phenyl)amino)butan-2-yl)phenyl 6-(3-((1s,3s)-adamantan-1-yl)-4-methoxyphenyl)-2-naphthoate (4a)

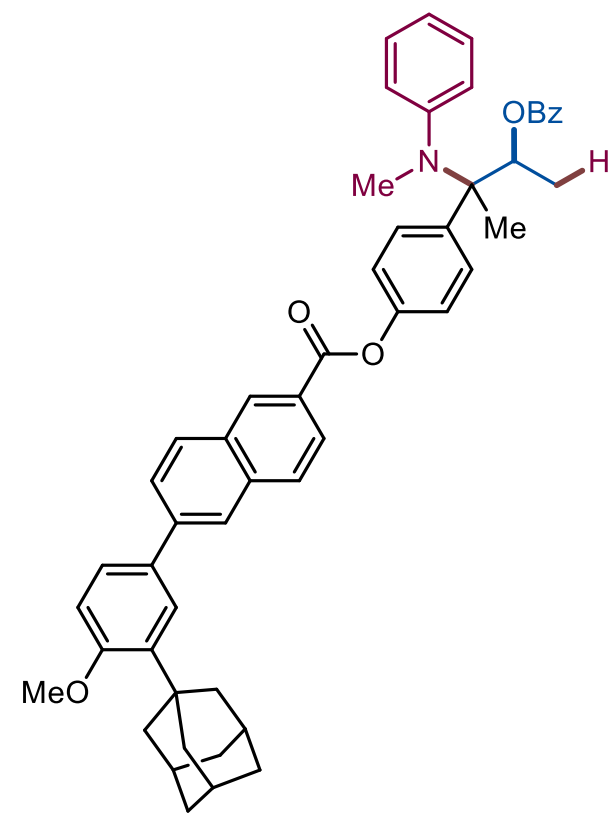

The reaction was performed according to General Procedure B at 40°C to afford the title compound (83.2 mg, 54% yield) as a yellow sticky oil. **R$_f$** = 0.3 [Hex: EtOAc 20:1 (v/v)]. *Major diastereomer*: **^{1}H NMR** (500 MHz, $CDCl_3$) δ 8.81 (s, 1H), 8.43 – 8.15 (m, 1H), 8.08 – 7.92 (m, 4H), 7.89 – 7.79 (m, 2H), 7.66 – 7.59 (m, 2H), 7.60 – 7.49 (m, 3H), 7.45 – 7.37 (m, 2H), 7.34 – 7.29 (m, 2H), 7.22 – 7.17 (m, 1H), 7.14 – 7.04 (m, 2H), 7.05 – 6.99 (m, 2H), 6.86 – 6.82 (m, 1H), 5.79 (q, *J* = 6.3 Hz, 1H), 3.92 (s, 3H), 2.74 (s, 3H), 2.21 (d, *J* = 2.9 Hz, 9H), 1.82 (s, 6H), 1.78 (s, 3H), 1.14 (d, *J* = 6.3 Hz, 3H). *Mixture of diastereomers*: **^{13}C NMR** (126 MHz, $CDCl_3$) δ 166.12, 165.77, 165.44, 159.17, 150.94, 150.13, 141.97, 140.85, 139.22, 136.45, 133.03, 132.86, 132.61, 131.85, 131.41, 130.80, 130.68, 130.00, 129.70, 129.66, 129.14, 129.02, 128.63, 128.59, 128.52, 128.44, 128.41, 128.25, 126.99, 126.83, 126.43, 126.34, 126.16, 125.95, 125.92, 124.93, 123.63, 123.33, 121.31, 120.96, 112.30, 74.87, 73.51, 66.55, 65.87, 55.33, 40.78, 38.86, 38.20, 37.39, 37.29, 29.27, 19.53, 17.07, 16.96, 15.91. **HRMS** (ESI-TOF) *m/z* calcd for $C_{52}H_{52}NO_5$ [$(M + H)^+$] 770.3845, found 770.3852.

4-(3-(benzoyloxy)-2-(methyl(phenyl)amino)butan-2-yl)phenyl 4-(N,N-dipropylsulfamoyl)benzoate (4b)

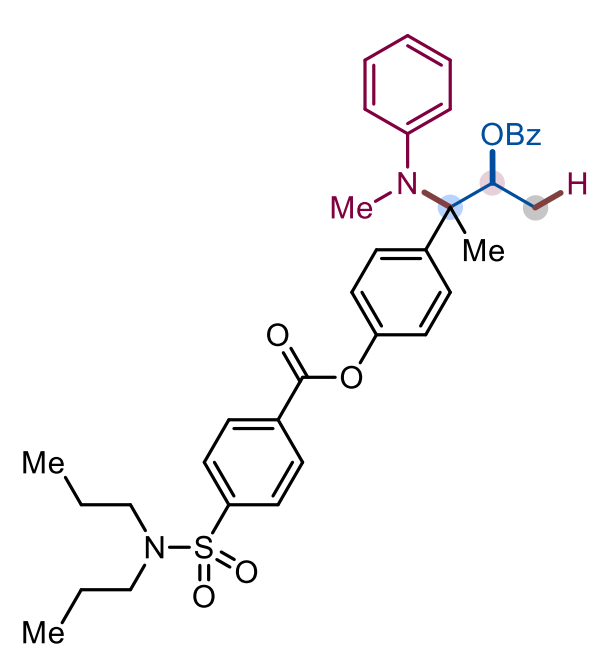

The reaction was performed according to General Procedure B at 40°C to afford the title compound (79.7 mg, 62% yield) as a white solid. **R$_f$** = 0.5 [Hex: EtOAc 5:1 (v/v)]. *Major diastereomer*: **^{1}H NMR** (800 MHz, $CDCl_3$) δ 8.40 – 8.34 (m, 2H), 8.01 – 7.93 (m, 2H), 7.87 (d, *J* = 7.6 Hz, 2H), 7.64 (d, *J* = 8.1 Hz, 2H), 7.58

– 7.51 (m, 1H), 7.41 (t, *J* = 7.6 Hz, 2H), 7.28 (t, *J* = 4.4 Hz, 2H), 7.15 – 7.06 (m, 3H), 6.96 (d, *J* = 7.9 Hz, 1H), 6.85 (t, *J* = 7.2 Hz, 1H), 5.78 (q, *J* = 6.6 Hz, 1H), 3.19 – 3.13 (m, 4H), 2.74 (s, 3H), 1.79 (s, 3H), 1.60 (p, *J* = 7.9 Hz, 4H), 1.13 (d, *J* = 6.0 Hz, 3H), 0.91 (q, *J* = 7.5 Hz, 6H). *Mixture of diastereomers*: ^{13}**C NMR** (201 MHz, $CDCl_3$) δ 165.93, 165.60, 163.78, 163.74, 150.71, 149.83, 149.53, 149.38, 144.96, 144.91, 141.27, 140.14, 132.93, 132.91, 132.76, 130.83, 130.80, 130.76, 130.60, 130.46, 129.67, 129.54, 129.50, 129.07, 128.98, 128.48, 128.39, 128.33, 128.29, 128.20, 128.12, 127.47, 127.22, 127.19, 126.80, 126.15, 123.54, 123.24, 121.63, 121.21, 120.90, 120.54, 74.72, 73.30, 66.41, 65.70, 49.97, 38.72, 38.03, 21.97, 19.36, 16.88, 16.81, 15.75, 11.19. **HRMS** (ESI-TOF) *m/z* calcd for $C_{37}H_{43}N_2O_6S$ $[(M + H)^+]$ 643.2842, found 643.2839.

3-(4-((2-(4-(2,2-dichlorocyclopropyl)phenoxy)-2-methylpropanoyl)oxy)phenyl)-3-(methyl(phenyl)amino)butan-2-yl benzoate (4c)

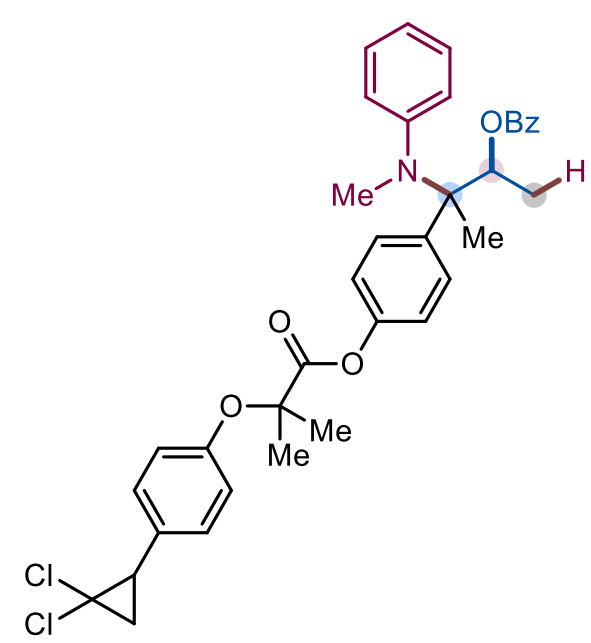

The reaction was performed according to General Procedure B at 40°C to afford the title compound (92.7 mg, 86% yield) as a yellow sticky oil. $\mathbf{R_f}$ = 0.4 [Hex: EtOAc 10:1 (v/v)]. *Major diastereomer*: ^{1}**H NMR** (500 MHz, $CDCl_3$) δ 7.86 – 7.80 (m, 2H), 7.56 – 7.49 (m, 4H), 7.48 – 7.34 (m, 2H), 7.21 – 7.12 (m, 2H), 7.12 – 7.05 (m, 2H), 7.05 – 6.88 (m, 5H), 6.84 – 6.79 (m, 1H), 5.80 – 5.66 (m, 1H), 2.91 – 2.81 (m, 1H), 2.68 (s, 3H), 1.98 – 1.91 (m, 1H), 1.84 – 1.77 (m, 7H), 1.72 (s, 3H), 1.07 (d, *J* = 6.3 Hz, 3H). *Mixture of diastereomers*: ^{13}**C NMR** (126 MHz, $CDCl_3$) δ 172.84, 172.79, 165.93, 165.60, 155.04, 155.00, 150.71, 149.83, 149.46, 149.30, 141.13, 140.03, 132.93, 132.75, 130.61, 130.49, 129.87, 129.84, 129.71, 129.54, 129.51, 128.91, 128.85, 128.49, 128.40, 128.32, 128.28, 128.23, 128.12, 126.80, 126.11, 123.52, 123.19, 121.02, 120.73, 120.39, 118.64, 79.36, 79.32, 74.68, 73.28, 66.37, 65.63, 60.89, 38.69, 37.99, 34.87, 25.88, 25.56, 25.52, 19.44, 16.91, 16.77, 15.72. **HRMS** (ESI-TOF) *m/z* calcd for $C_{37}H_{38}NO_5Cl_2$ $[(M + H)^+]$ 646.2127, found 646.2125.

3-(4-(((S)-2-(4-isobutylphenyl)propanoyl)oxy)phenyl)-3-(methyl(4-(trifluoromethoxy)phenyl)amino)butan-2-yl benzoate (4d)

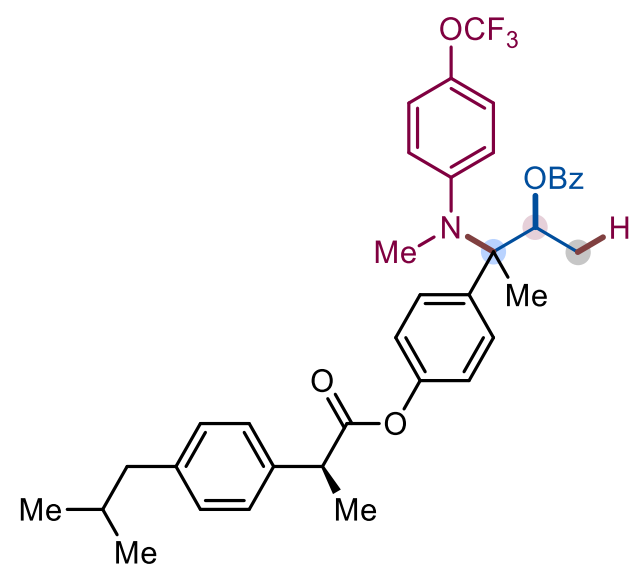

The reaction was performed according to General Procedure B at 40°C to afford the title compound (66.6 mg, 59% yield) as a yellow sticky oil. **R*f*** = 0.3 [Hex: EtOAc 20:1 (v/v)]. *Major diastereomer*: **^{1}H NMR** (800 MHz, $CDCl_3$) δ 7.81 (d, *J* = 7.7 Hz, 2H), 7.51 (t, *J* = 7.5 Hz, 1H), 7.45 (d, *J* = 8.3 Hz, 2H), 7.42 – 7.34 (m, 2H), 7.31 (d, *J* = 7.8 Hz, 2H), 7.15 (d, *J* = 7.7 Hz, 2H), 7.05 – 7.00 (m, 2H), 7.00 – 6.94 (m, 2H), 6.88 (d, *J* = 8.4 Hz, 2H), 5.74 – 5.65 (m, 1H), 3.98 – 3.88 (m, 1H), 2.63 (s, 3H), 2.49 – 2.44 (m, 2H), 1.89 – 1.83 (m, 1H), 1.69 (s, 3H), 1.63 – 1.60 (m, 3H), 1.03 (d, *J* = 6.3 Hz, 3H), 0.96 – 0.87 (m, 6H). *Mixture of diastereomers*: **^{13}C NMR** (201 MHz, $CDCl_3$) δ 173.18, 165.87, 165.50, 149.92, 149.76, 149.32, 148.47, 144.92, 144.43, 140.89, 140.85, 140.31, 139.06, 137.22, 132.98, 132.89, 130.43, 130.25, 129.56, 129.52, 129.36, 128.64, 128.47, 128.41, 128.21, 127.55, 127.24, 127.22, 126.56, 126.53, 121.05, 120.96, 120.78, 120.69, 119.69, 74.48, 73.08, 66.43, 65.67, 45.32, 45.29, 45.07, 38.59, 38.00, 30.21, 22.41, 19.52, 18.53, 16.84, 16.59, 15.71. *Major diastereomer*: **^{19}F NMR** (470 MHz, $CDCl_3$) δ -58.16.

3-(4-((5-(2,5-dimethylphenoxy)-2,2-dimethylpentanoyl)oxy)phenyl)-3-(methyl(phenyl)amino)butan-2-yl benzoate (4e)

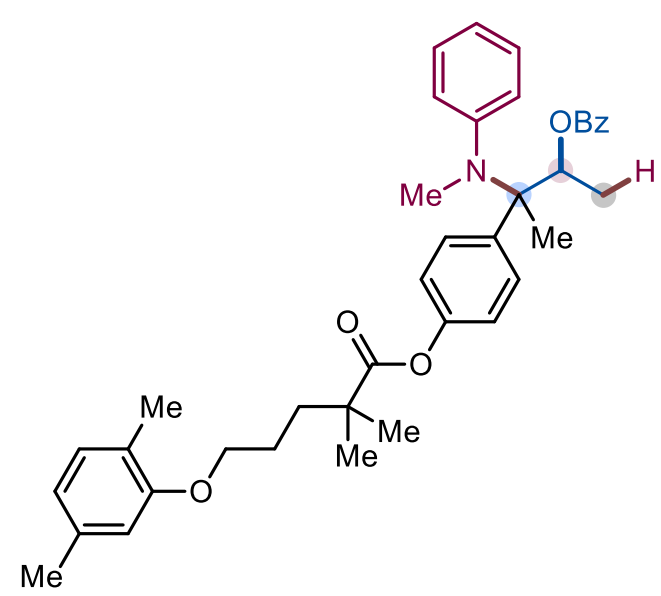

The reaction was performed according to General Procedure B at 40°C to afford the title compound (71.7 mg, 59% yield) as a white solid. **R*f*** = 0.3 [Hex: EtOAc 20:1 (v/v)]. *Major diastereomer*: **^{1}H NMR** (500 MHz, $CDCl_3$) δ 7.88 – 7.81 (m, 2H), 7.56 – 7.50 (m, 3H), 7.44 – 7.35 (m, 2H), 7.12 – 6.97 (m, 7H), 6.85 – 6.79 (m, 1H), 6.70 – 6.61 (m, 2H), 5.78 – 5.69 (m, 1H), 4.04 – 3.95 (m, 2H), 2.69 (s, 3H), 2.32 (s, 3H), 2.19 (s, 3H), 1.93 – 1.89 (m, 4H), 1.73 (s, 3H), 1.40 (s, 6H), 1.09 (d, *J* = 6.3 Hz, 3H). *Mixture of diastereomers*: **^{13}C NMR** (126 MHz, $CDCl_3$) δ 176.34, 176.30, 165.96, 165.63, 156.92, 150.80, 149.92, 149.76, 140.53, 139.47, 136.51, 132.89, 132.73, 130.66, 130.55, 130.39, 129.56, 129.52, 128.82, 128.75, 128.39, 128.30, 128.26,

128.12, 126.87, 126.14, 123.66, 123.52, 123.14, 120.97, 120.81, 120.78, 120.63, 112.01, 74.67, 73.31, 67.85, 66.36, 65.63, 65.22, 42.52, 42.48, 38.68, 38.00, 37.21, 25.34, 25.31, 25.21, 25.18, 21.44, 19.49, 16.90, 16.80, 15.84, 15.81, 15.73. **HRMS** (ESI-TOF) *m/z* calcd for $C_{39}H_{45}NO_5Na$ [$(M + Na)^+$] 630.3195, found 630.3193.

3-(4-((N-(2-(9H-fluoren-9-yl)acetyl)-N-methyl-D-valyl)oxy)phenyl)-3-(methyl(4-(trifluoromethoxy)phenyl)amino)butan-2-yl benzoate (4f)

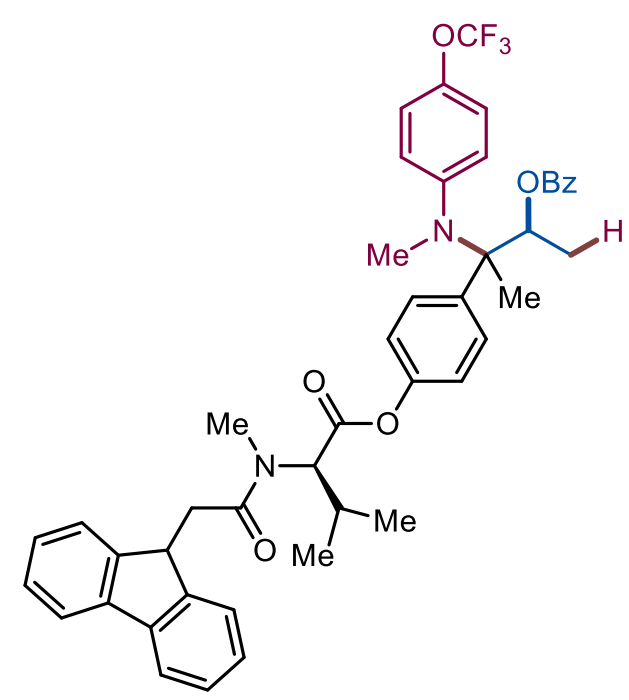

The reaction was performed according to General Procedure B at 40°C to afford the title compound (110.6 mg, 71% yield) as a white foam. $\mathbf{R}_f$ = 0.5 [Hex: EtOAc 5:1 (v/v)]. *Major diastereomer* : **^{1}H NMR** (400 MHz, CDCl$_3$) δ 7.85 (d, *J* = 7.7 Hz, 2H), 7.80 (d, *J* = 7.9 Hz, 2H), 7.71 – 7.61 (m, 2H), 7.59 – 7.49 (m, 3H), 7.45 – 7.35 (m, 4H), 7.37 – 7.28 (m, 2H), 7.13 (d, *J* = 8.5 Hz, 1H), 7.11 – 7.00 (m, 3H), 6.94 (t, *J* = 6.4 Hz, 2H), 5.81 – 5.70 (m, 1H), 4.60 – 4.44 (m, 1H), 4.38 – 4.26 (m, 1H), 3.05 – 2.97 (m, 3H), 2.74 – 2.63 (m, 3H), 2.39 – 2.19 (m, 1H), 1.78 – 1.70 (m, 3H), 1.61 (s, 2H), 1.20 – 0.94 (m, 9H). *Major diastereomer*: **^{13}C NMR** (201 MHz, CDCl$_3$) δ 169.93, 169.33, 165.63, 157.12, 156.33, 149.57, 149.43, 145.10, 144.08, 141.53, 133.05, 130.34, 129.49, 128.74, 128.35, 127.87, 127.70, 127.21, 125.10, 125.00, 121.24, 121.12, 120.86, 120.15, 74.63, 67.90, 66.61, 64.48, 64.36, 47.48, 47.42, 38.78, 30.74, 30.34, 28.10, 27.79, 19.96, 19.85, 19.17, 18.93, 16.99, 16.74. *Major diastereomer* : **^{19}F NMR** (376 MHz, CDCl$_3$) δ -59.68.

4-(3-(benzoyloxy)-2-(methyl(4-(trifluoromethoxy)phenyl)amino)butan-2-yl)phenyl 2-(3-cyano-4-isobutoxyphenyl)-4-methylthiazole-5-carboxylate (4g)

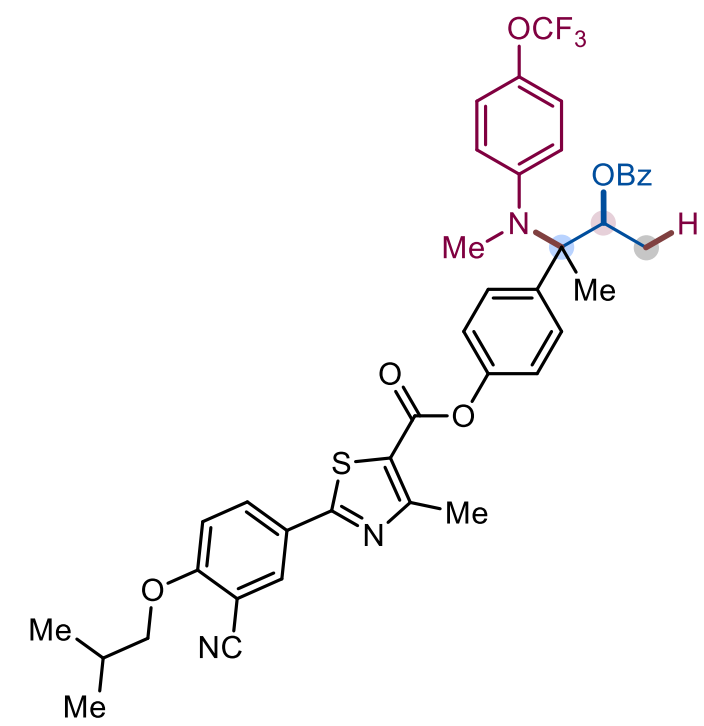

The reaction was performed according to General Procedure B at 40°C to afford the title compound (107.6 mg, 71% yield) as a yellow sticky oil. **R*f*** = 0.3 [Hex: EtOAc 5:1 (v/v)]. *Major diastereomer*: **^{1}H NMR** (400 MHz, $CDCl_3$) δ 8.24 (d, *J* = 2.3 Hz, 1H), 8.16 – 8.11 (m, 1H), 7.87 – 7.80 (m, 2H), 7.62 – 7.52 (m, 2H), 7.56 – 7.47 (m, 1H), 7.47 – 7.34 (m, 3H), 7.24 (s, 1H), 7.08 – 6.97 (m, 3H), 6.96 – 6.85 (m, 2H), 5.81 – 5.68 (m, 1H), 3.95 – 3.88 (m, 2H), 2.85 (s, 3H), 2.68 (s, 3H), 2.27 – 2.15 (m, 1H), 1.75 (s, 3H), 1.13 – 1.04 (m, 9H). *Mixture of diastereomers*: **^{13}C NMR** (126 MHz, $CDCl_3$) δ 168.25, 165.87, 165.51, 163.19, 162.74, 160.37, 149.31, 145.01, 141.00, 133.04, 132.94, 132.70, 132.29, 130.45, 130.24, 129.54, 129.39, 128.94, 128.75, 128.45, 128.24, 127.60, 126.64, 125.89, 121.26, 120.86, 120.75, 120.59, 115.36, 112.73, 103.17, 75.79, 74.55, 73.15, 66.55, 65.79, 38.70, 38.10, 28.19, 19.51, 19.07, 17.75, 17.72, 16.91, 16.66, 15.79. *Major diastereomer*: **^{19}F NMR** (376 MHz, $CDCl_3$) δ -59.68. **HRMS** (ESI-TOF) *m/z* calcd for $C_{41}H_{39}N_3O_6SF_3$ [$(M + H)^+$] 758.2512, found 758.2502.

3-(4-((3-(4,5-diphenyloxazol-2-yl)propanoyl)oxy)phenyl)-3-(methyl(4-(trifluoromethoxy)phenyl)amino)butan-2-yl benzoate (4h)

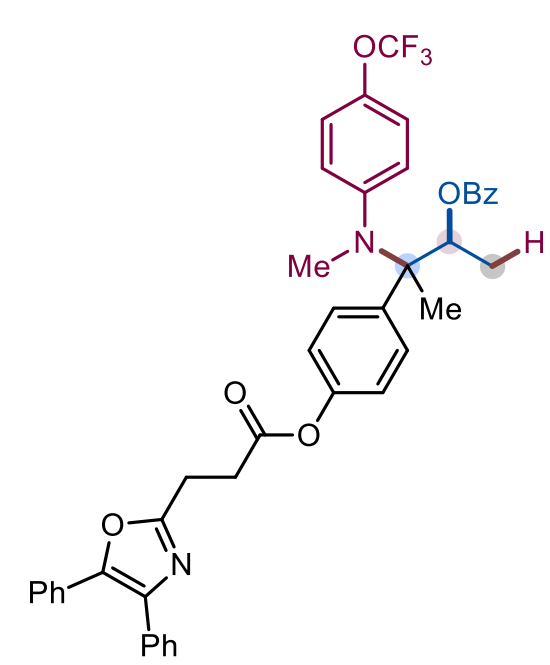

The reaction was performed according to General Procedure B at 40°C to afford the title compound (92.6 mg, 63% yield) as a white solid. **R*f*** = 0.5 [Hex: EtOAc 5:1 (v/v)]. *Major diastereomer*: **^{1}H NMR** (400 MHz, $CDCl_3$) δ 7.86 – 7.79 (m, 2H), 7.69 – 7.63 (m, 2H), 7.63 – 7.55 (m, 2H), 7.55 – 7.47 (m, 3H), 7.46 – 7.29 (m, 8H), 7.17 – 7.12 (m, 2H), 7.04 – 6.97 (m, 2H), 6.93 – 6.84 (m, 2H), 5.79 – 5.66 (m, 1H), 3.40 – 3.24 (m, 2H), 3.24 – 3.04 (m, 2H), 2.65 (d, *J* = 1.7 Hz, 3H), 1.72 (s, 3H), 1.06 (dd, *J* = 6.4, 1.9 Hz, 3H). *Mixture of*

diastereomers: ^{13}C NMR (201 MHz, $CDCl_3$) δ 170.56, 165.88, 165.51, 161.46, 149.68, 149.33, 148.48, 145.62, 144.96, 140.55, 139.33, 135.22, 133.01, 132.90, 132.43, 130.44, 130.24, 129.52, 129.37, 128.96, 128.78, 128.69, 128.67, 128.60, 128.56, 128.42, 128.22, 128.14, 128.12, 127.91, 127.60, 126.60, 126.56, 121.13, 120.97, 120.81, 120.76, 120.71, 119.69, 74.52, 73.16, 66.46, 65.72, 38.64, 38.05, 31.32, 23.54, 19.49, 16.88, 16.59, 15.76. *Major diastereomer*: **^{19}F NMR** (376 MHz, $CDCl_3$) δ -59.67. **HRMS** (ESI-TOF) *m/z* calcd for $C_{43}H_{38}N_2O_6F_3$ [(M + H)$^+$] 735.2682, found 735.2679.

3-(methyl(4-(((R)-2,5,7,8-tetramethyl-2-((4R,8R)-4,8,12-trimethyltridecyl)chroman-6-yl)oxy)phenyl)amino)-3-phenylbutan-2-yl benzoate (4i)

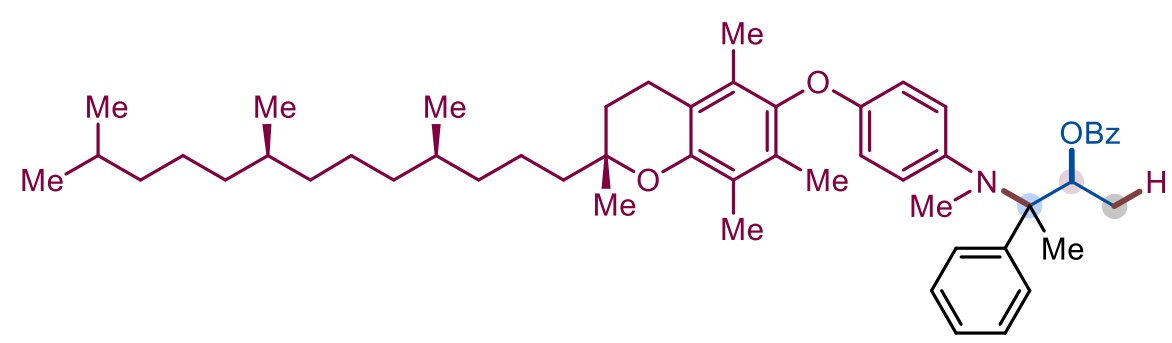

The reaction was performed according to General Procedure B at 40°C to afford the title compound (110.7 mg, 69% yield) as a yellow sticky oil. **R_f** = 0.3 [Hex: EtOAc 20:1 (v/v)]. *Major diastereomer*: **^{1}H NMR** (500 MHz, $CDCl_3$) δ 7.89 – 7.81 (m, 2H), 7.57 – 7.45 (m, 3H), 7.44 – 7.27 (m, 4H), 7.24 – 7.16 (m, 1H), 6.89 – 6.81 (m, 2H), 6.58 – 6.52 (m, 2H), 5.65 – 5.58 (m, 1H), 2.61 (t, *J* = 6.8 Hz, 2H), 2.54 (s, 3H), 2.12 (s, 3H), 2.02 (s, 3H), 1.98 (s, 3H), 1.90 – 1.74 (m, 2H), 1.66 – 1.47 (m, 7H), 1.46 – 1.35 (m, 4H), 1.35 – 1.19 (m, 13H), 1.18 – 1.04 (m, 6H), 0.91 – 0.82 (m, 12H). *Mixture of diastereomers*: **^{13}C NMR** (126 MHz, $CDCl_3$) δ 165.90, 155.75, 148.63, 143.75, 143.10, 141.60, 132.66, 130.87, 129.53, 129.47, 129.00, 128.94, 128.32, 128.23, 128.08, 127.93, 127.79, 127.53, 126.90, 126.70, 126.38, 123.16, 117.78, 117.67, 114.27, 75.00, 74.55, 73.17, 66.49, 65.78, 40.07, 39.41, 39.04, 38.61, 37.63, 37.51, 37.44, 37.40, 37.34, 32.83, 32.73, 31.33, 28.02, 24.84, 24.48, 23.89, 23.86, 22.75, 22.66, 21.09, 20.67, 19.79, 19.72, 19.65, 18.49, 16.95, 16.19, 15.69, 12.96, 12.81, 12.09, 11.94, 11.83, 11.79. **HRMS** (ESI-TOF) *m/z* calcd for $C_{53}H_{74}NO_4$ [(M + H)$^+$] 788.5618, found 788.5611.

3-((4-(((3S,5S,8R,9S,10S,13R,14S,17R)-10,13-dimethyl-17-((R)-6-methylheptan-2-yl)hexadecahydro-1H-cyclopenta[a]phenanthren-3-yl)oxy)phenyl)(methyl)amino)-3-phenylbutan-2-yl benzoate (4j)

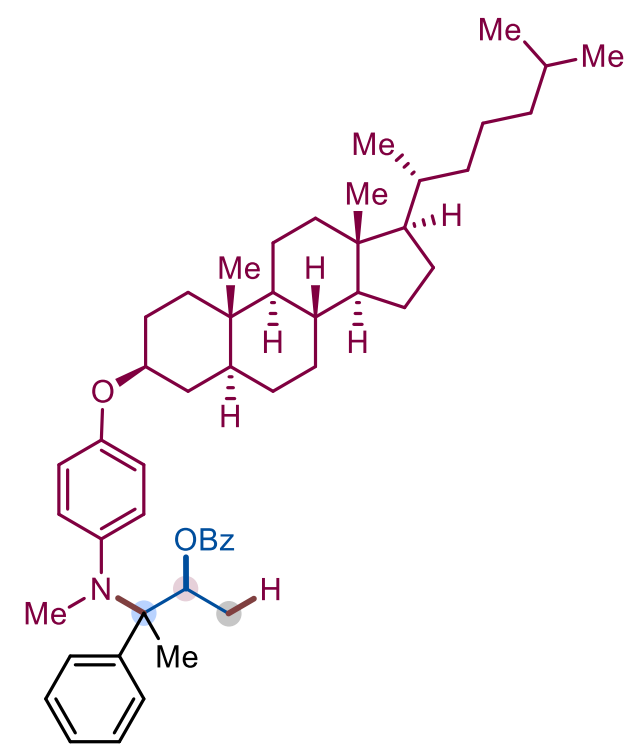

The reaction was performed according to General Procedure B at 40°C to afford the title compound (83.6 mg, 56% yield) as a white solid. **R*f*** = 0.3 [Hex: EtOAc 5:1 (v/v)]. *Major diastereomer*: **^{1}H NMR** (400 MHz, $CDCl_3$) δ 7.94 – 7.87 (m, 2H), 7.56 – 7.47 (m, 4H), 7.45 – 7.31 (m, 4H), 6.88 (d, *J* = 8.4 Hz, 2H), 6.73 (d, *J* = 8.7 Hz, 2H), 5.75 – 5.62 (m, 1H), 4.19 – 4.05 (m, 1H), 2.57 (s, 3H), 1.92 – 0.77 (m, 46H), 0.69 (s, 6H). *Mixture of diastereomers*: **^{13}C NMR** (126 MHz, $CDCl_3$) δ 166.09, 165.64, 155.00, 154.95, 143.75, 143.56, 142.96, 141.43, 132.81, 132.63, 130.98, 130.81, 129.63, 129.55, 129.12, 129.05, 128.37, 128.19, 128.14, 127.97, 127.89, 127.64, 127.32, 127.08, 126.82, 115.69, 115.54, 115.49, 75.30, 73.23, 66.77, 65.86, 56.66, 56.46, 54.59, 53.91, 45.06, 45.00, 42.77, 40.21, 39.68, 39.12, 38.60, 37.11, 36.34, 35.95, 35.89, 35.81, 35.67, 34.67, 34.47, 32.27, 28.90, 28.40, 28.25, 28.17, 28.07, 24.38, 23.99, 22.97, 22.72, 21.41, 18.84, 18.56, 17.35, 17.20, 15.73, 14.97, 12.52, 12.48, 12.24. **HRMS** (ESI-TOF) *m/z* calcd for $C_{50}H_{72}NO_3$ [$(M + H)^+$] 746.5512, found 746.5518.

3-(methyl(4-(((2aS,4S,5'R,6aS,6bS,8aS,8bR,10R,11aS,12aS,12bR)-5',6a,8a-trimethyldocosahydrospiro[naphtho[2',1':4,5]indeno[2,1-b]furan-10,2'-pyran]-4-yl)oxy)phenyl)amino)-3-phenylbutan-2-yl benzoate (4k)

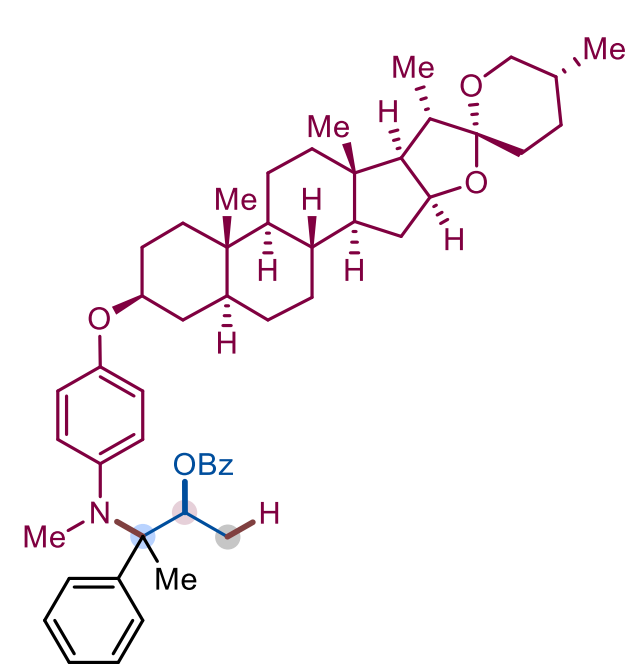

The reaction was performed according to General Procedure B at 40°C to afford the title compound (74.5 mg, 49% yield) as a white solid. **R*f*** = 0.3 [Hex: EtOAc 5:1 (v/v)]. *Major diastereomer*: **^{1}H NMR** (800 MHz, $CDCl_3$) δ 7.89 – 7.84 (m, 2H), 7.52 – 7.44 (m, 3H), 7.40 – 7.33 (m, 3H), 7.33 – 7.28 (m, 2H), 6.88 – 6.82 (m, 2H), 6.72 – 6.68 (m, 2H), 4.46 – 4.33 (m, 2H), 4.12 – 4.06 (m, 1H), 3.50 – 3.44 (m, 2H), 3.41 – 3.30 (m,

1H), 2.54 (s, 3H), 1.81 – 0.59 (m, 42H). *Mixture of diastereomers*: **^{13}C NMR** (201 MHz, $CDCl_3$) δ 165.96, 154.76, 142.85, 141.28, 132.68, 132.49, 130.82, 129.49, 129.41, 128.98, 128.24, 128.06, 128.01, 127.75, 127.50, 126.95, 126.69, 115.55, 115.39, 115.33, 109.27, 80.86, 73.08, 66.86, 66.64, 65.71, 62.22, 56.32, 54.41, 44.87, 41.63, 40.60, 40.07, 38.98, 38.46, 36.92, 35.86, 35.79, 35.13, 34.49, 32.27, 31.94, 31.79, 31.39, 30.32, 29.37, 28.81, 28.63, 28.09, 22.70, 21.07, 18.44, 18.39, 17.14, 17.06, 16.51, 15.59, 14.51, 14.13, 12.41, 12.37. **HRMS** (ESI-TOF) *m/z* calcd for $C_{51}H_{68}NO_5$ [(M + H)$^+$] 774.5097, found 774.5103.

3-(methyl(4-(((8R,9S,13S,14S)-13-methyl-17-oxo-7,8,9,11,12,13,14,15,16,17-decahydro-6H-cyclopenta[a]phenanthren-3-yl)oxy)phenyl)amino)-3-phenylbutan-2-yl benzoate (4l)

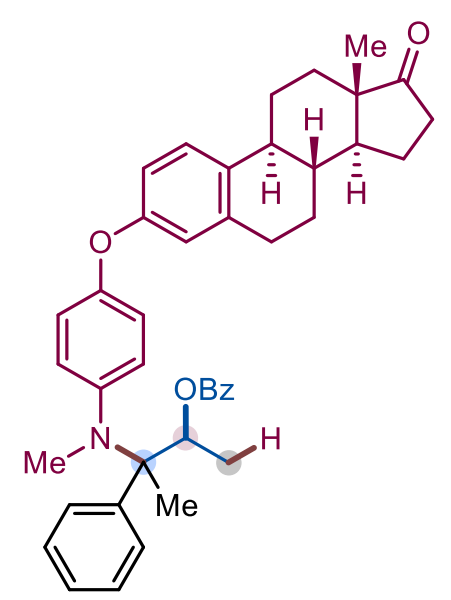

The reaction was performed according to General Procedure B at 40°C to afford the title compound (86.7 mg, 69% yield) as a white foam. **R_f** = 0.5 [Hex: EtOAc 5:1 (v/v)]. *Major diastereomer*: **^{1}H NMR** (800 MHz, $CDCl_3$) δ 7.87 (d, *J* = 7.5 Hz, 2H), 7.48 (d, *J* = 7.8 Hz, 3H), 7.36 (t, *J* = 7.8 Hz, 2H), 7.29 (d, *J* = 8.1 Hz, 2H), 7.25 – 7.19 (m, 2H), 7.00 (d, *J* = 8.2 Hz, 1H), 6.88 (d, *J* = 8.0 Hz, 2H), 6.79 (d, *J* = 7.7 Hz, 2H), 6.71 (s, 1H), 5.64 (q, *J* = 6.3, 5.4 Hz, 1H), 2.93 – 2.77 (m, 2H), 2.60 (s, 3H), 2.53 – 2.45 (m, 1H), 2.41 – 2.34 (m, 1H), 2.26 (t, *J* = 12.4 Hz, 1H), 2.17 – 2.09 (m, 1H), 2.08 – 1.92 (m, 3H), 1.67 – 1.37 (m, 9H), 1.35 – 1.30 (m, 3H), 0.91 (d, *J* = 3.2 Hz, 3H). *Mixture of diastereomers*: **^{13}C NMR** (201 MHz, $CDCl_3$) δ 220.88, 165.95, 165.54, 155.52, 154.82, 153.92, 153.53, 153.50, 145.90, 145.36, 143.16, 141.46, 138.15, 138.05, 134.56, 134.38, 132.77, 132.73, 130.76, 130.68, 129.50, 128.62, 128.52, 128.30, 128.19, 128.05, 128.03, 127.70, 127.62, 127.03, 126.81, 126.53, 126.47, 119.15, 118.61, 118.59, 117.90, 116.35, 115.97, 115.95, 74.90, 73.16, 66.63, 65.87, 50.47, 48.01, 44.12, 38.90, 38.45, 38.27, 38.23, 35.89, 31.61, 29.53, 29.48, 26.49, 25.89, 21.62, 18.70, 17.00, 15.72, 15.62, 13.88. **HRMS** (ESI-TOF) *m/z* calcd for $C_{42}H_{46}NO_4$ [(M + H)$^+$] 628.3427, found 628.3431.

3-((2-methoxyphenyl)(methyl)amino)-3-phenylbutan-2-yl benzoate (3aaw)

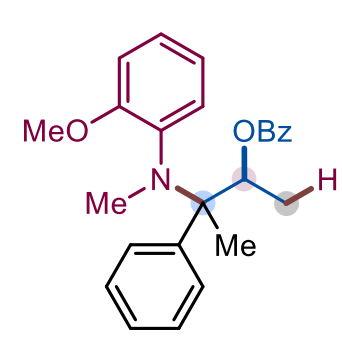

The reaction was performed according to General Procedure B at 40°C to afford the title compound (31.2 mg, 40% yield) as a colorless sticky liquid. **R_f** = 0.5 [Hex: EtOAc 5:1 (v/v)]. *Mixture of diastereomer*: **^{1}H NMR** (500 MHz, $CDCl_3$) δ 7.83 (d, *J* = 7.0 Hz, 2H), 7.76 – 7.70 (m, 4H), 7.66 (d, *J* = 7.1 Hz, 2H), 7.51 – 7.44 (m, 2H), 7.42 – 7.19 (m, 11H), 7.11 – 7.05 (m, 1H), 6.99 (dd, *J* = 7.8, 1.7 Hz, 1H), 6.81 – 6.70 (m, 3H), 6.67 – 6.59 (m, 2H), 5.78 (q, *J* = 6.4 Hz, 1H), 5.72 (q, *J* = 6.3 Hz, 1H), 3.78 (s, 3H), 3.59 (s, 3H), 2.55 (s, 3H), 2.52 (s, 3H), 1.81 (s, 3H), 1.68 (s, 3H), 1.31 (d, *J* = 6.3 Hz, 3H), 0.87 (d, *J* = 6.4 Hz, 3H). *Mixture of diastereomers*: **^{13}C NMR** (126 MHz, $CDCl_3$) δ 165.69, 165.32, 157.46, 157.28, 144.88, 142.31, 139.60, 138.84, 132.47, 132.35, 130.90, 130.68, 130.00, 129.51, 129.44, 128.46, 128.07, 128.02, 127.92, 127.76, 127.40, 126.86, 126.57, 126.00, 125.71, 120.21, 120.14, 111.58, 111.55, 75.29, 73.14, 67.40, 66.07, 55.16, 54.97, 38.36, 38.01, 18.71, 16.99, 15.87. **HRMS** (ESI-TOF) *m/z* calcd for $C_{25}H_{28}NO_3$ [$(M + H)^+$] 390.2069, found 390.2064.

3-((3-methoxyphenyl)(methyl)amino)-3-phenylbutan-2-yl benzoate (3aax)

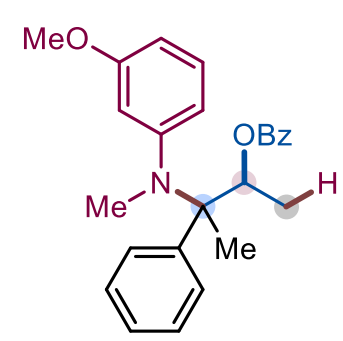

The reaction was performed according to General Procedure B at 40°C to afford the title compound (38.2 mg, 49% yield) as a colorless sticky liquid. **R_f** = 0.5 [Hex: EtOAc 5:1 (v/v)]. *Major diastereomer*: **^{1}H NMR** (800 MHz, $CDCl_3$) δ 7.97 (d, *J* = 7.7 Hz, 2H), 7.58 – 7.52 (m, 3H), 7.45 – 7.38 (m, 3H), 7.35 – 7.29 (m, 2H), 7.25 (t, *J* = 7.4 Hz, 1H), 6.53 (d, *J* = 5.7 Hz, 1H), 6.41 (s, 1H), 6.33 (d, *J* = 8.4 Hz, 1H), 5.82 – 5.74 (m, 1H), 3.62 (s, 3H), 2.80 (s, 3H), 1.72 (s, 3H), 1.35 (d, *J* = 5.9 Hz, 3H). *Mixture of diastereomers*: **^{13}C NMR** (201 MHz, $CDCl_3$) δ 165.96, 165.62, 159.49, 159.40, 152.30, 151.36, 143.54, 142.61, 132.87, 132.66, 130.69, 130.55, 129.56, 129.48, 128.66, 128.62, 128.34, 128.09, 128.04, 127.80, 127.74, 127.65, 126.99, 126.75, 118.79, 117.43, 112.31, 110.82, 108.58, 107.93, 75.09, 73.64, 66.73, 65.99, 54.99, 54.91, 38.49, 37.87, 19.60, 16.86, 16.24, 15.79. **HRMS** (ESI-TOF) *m/z* calcd for $C_{25}H_{28}NO_3$ [$(M + H)^+$] 390.2069, found 390.2061.

3-((2-fluorophenyl)(methyl)amino)-3-phenylbutan-2-yl benzoate (3aay)

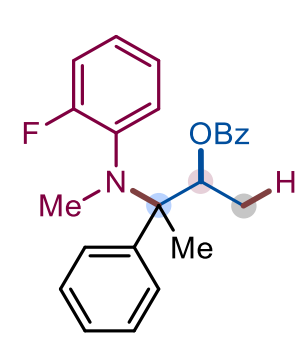

The reaction was performed according to General Procedure B at 40°C to afford the title compound (52.0 mg, 69% yield) as a colorless sticky oil. **R*f*** = 0.4 [Hex: EtOAc 20:1 (v/v)]. *Major diastereomer*: **^{1}H NMR** (400 MHz, $CDCl_3$) δ 7.79 (d, *J* = 6.7 Hz, 2H), 7.63 (d, *J* = 7.0 Hz, 2H), 7.54 – 7.45 (m, 1H), 7.43 – 7.25 (m, 5H), 7.18 (td, *J* = 7.8, 1.6 Hz, 1H), 6.87 – 6.71 (m, 3H), 5.71 (q, *J* = 6.4 Hz, 1H), 2.58 (s, 3H), 1.80 (s, 3H), 0.97 (d, *J* = 6.3 Hz, 3H). *Major diastereomer*: **^{13}C NMR** (201 MHz, $CDCl_3$) δ 165.41, 161.13, 159.90, 143.49, 138.17, 138.12, 132.57, 130.86, 130.41, 129.45, 128.11, 127.92, 127.75, 127.09, 125.96, 125.91, 123.76, 123.74, 116.36, 116.25, 75.01, 67.09, 38.13, 16.77, 15.87. *Major diastereomer*: **^{19}F NMR** (376 MHz, $CDCl_3$) δ -118.53. **HRMS** (ESI-TOF) *m/z* calcd for $C_{24}H_{24}NO_2NaF$ [$(M + Na)^+$] 400.1689, found 400.1685.

3-((2-chlorophenyl)(methyl)amino)-3-phenylbutan-2-yl benzoate (3aaz)

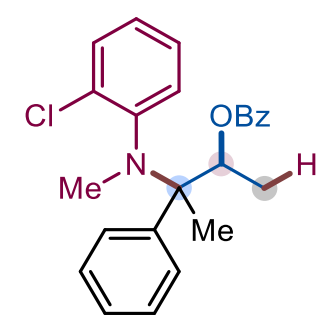

The reaction was performed according to General Procedure B at 40°C to afford the title compound (24.4 mg, 31% yield) as a colorless sticky liquid. **R*f*** = 0.4 [Hex: EtOAc 20:1 (v/v)]. *Major diastereomer*: **^{1}H NMR** (500 MHz, $CDCl_3$) δ 7.94 – 7.91 (m, 2H), 7.55 – 7.49 (m, 1H), 7.39 (t, *J* = 7.8 Hz, 2H), 7.28 – 7.13 (m, 8H), 6.59 (d, *J* = 8.6 Hz, 1H), 5.93 (q, *J* = 6.3 Hz, 1H), 2.89 (s, 3H), 1.74 (s, 3H), 1.21 (d, *J* = 6.2 Hz, 3H). *Major diastereomer*: **^{13}C NMR** (126 MHz, $CDCl_3$) δ 166.19, 147.02, 143.26, 134.35, 132.82, 130.64, 129.59, 129.56, 128.34, 128.12, 127.74, 127.60, 126.07, 118.67, 109.88, 75.16, 50.19, 30.45, 25.19, 16.08. **HRMS** (ESI-TOF) *m/z* calcd for $C_{24}H_{25}NO_2Cl$ [$(M + H)^+$] 394.1574, found 394.1563.

3-((2-bromophenyl)(methyl)amino)-3-phenylbutan-2-yl benzoate (3aaaa)

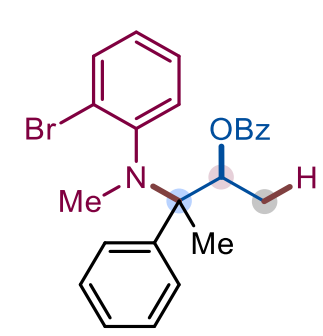

The reaction was performed according to General Procedure B at 40°C to afford the title compound (40.2 mg, 46% yield) as a colorless sticky oil. **R*f*** = 0.4 [Hex: EtOAc 20:1 (v/v)]. *Major diastereomer*: **^{1}H NMR** (500 MHz, $CDCl_3$) δ 7.93 (d, *J* = 6.9 Hz, 2H), 7.56 – 7.48 (m, 1H), 7.44 (d, *J* = 2.2 Hz, 1H), 7.39 (t, *J* = 7.8 Hz, 2H), 7.29 – 7.20 (m, 6H), 7.16 (ddt, *J* = 8.4, 5.7, 2.1 Hz, 1H), 6.57 (d, *J* = 8.6 Hz, 1H), 5.93 (q, *J* = 6.2 Hz, 1H), 2.88 (s, 3H), 1.73 (s, 3H), 1.21 (d, *J* = 6.2 Hz, 3H). *Major diastereomer*: **^{13}C NMR** (126 MHz, $CDCl_3$) δ 166.18, 147.05, 144.24, 134.78, 132.84, 130.63, 129.60, 128.48, 128.35, 128.13, 127.59, 126.08,

109.88, 109.30, 75.11, 50.19, 30.65, 25.31, 16.08. **HRMS** (ESI-TOF) *m/z* calcd for $C_{24}H_{25}NO_2Br$ [$(M + H)^+$] 438.1069, found 438.1062.

3-((3-fluorophenyl)(methyl)amino)-3-phenylbutan-2-yl benzoate (3aaab)

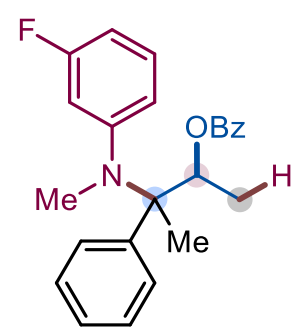

The reaction was performed according to General Procedure B at 40°C to afford the title compound (51.2 mg, 68% yield) as a colorless sticky oil. **R*f*** = 0.4 [Hex: EtOAc 20:1 (v/v)]. *Major diastereomer*: ^{1}**H NMR** (400 MHz, $CDCl_3$) δ 7.90 – 7.83 (m, 2H), 7.58 – 7.45 (m, 3H), 7.45 – 7.34 (m, 3H), 7.33 – 7.22 (m, 2H), 7.07 – 6.88 (m, 1H), 6.76 – 6.70 (m, 1H), 6.66 (dt, *J* = 11.8, 2.3 Hz, 1H), 6.48 – 6.40 (m, 1H), 5.86 – 5.67 (m, 1H), 2.77 (s, 3H), 1.76 (s, 3H), 1.10 (d, *J* = 6.3 Hz, 3H). *Mixture of diastereomers*: ^{13}**C NMR** (201 MHz, $CDCl_3$) δ 165.93, 165.62, 163.36, 163.25, 162.14, 162.04, 152.71, 152.67, 151.79, 143.17, 142.48, 132.99, 132.86, 130.54, 130.38, 129.65, 129.57, 129.46, 128.91, 128.86, 128.81, 128.76, 128.40, 128.22, 128.19, 127.98, 127.51, 127.49, 127.14, 126.92, 120.94, 119.19, 112.07, 111.96, 110.50, 110.38, 109.08, 108.97, 108.02, 107.92, 74.97, 73.69, 66.80, 66.15, 38.31, 37.63, 30.71, 20.00, 17.14, 16.76, 15.81. *Major diastereomer*: ^{19}**F NMR** (376 MHz, $CDCl_3$) δ -114.96 – -115.11 (m). **HRMS** (ESI-TOF) *m/z* calcd for $C_{24}H_{25}NO_2F$ [$(M + H)^+$] 378.1869, found 378.1866.

3-((3-chlorophenyl)(methyl)amino)-3-phenylbutan-2-yl benzoate (3aaac)

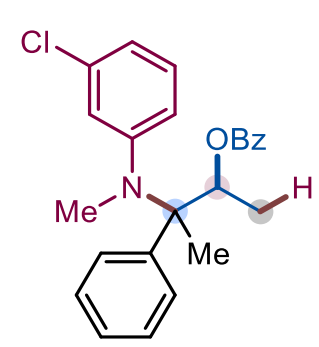

The reaction was performed according to General Procedure B at 40°C to afford the title compound (44.1 mg, 56% yield) as a colorless sticky oil. **R*f*** = 0.4 [Hex: EtOAc 20:1 (v/v)]. *Major diastereomer*: ^{1}**H NMR** (500 MHz, $CDCl_3$) δ 7.86 – 7.81 (m, 2H), 7.58 – 7.47 (m, 3H), 7.46 – 7.34 (m, 3H), 7.35 – 7.20 (m, 2H), 7.04 – 6.97 (m, 1H), 6.93 (t, *J* = 8.0 Hz, 1H), 6.90 – 6.82 (m, 1H), 6.74 – 6.63 (m, 1H), 5.79 – 5.70 (m, 1H), 2.71 (s, 3H), 1.76 (s, 3H), 1.07 (d, *J* = 6.3 Hz, 3H). *Mixture of diastereomers*: ^{13}**C NMR** (126 MHz, $CDCl_3$) δ 165.93, 165.54, 152.28, 151.30, 143.22, 142.13, 133.76, 133.72, 132.97, 132.82, 130.55, 130.34, 129.59, 129.43, 129.00, 128.89, 128.40, 128.26, 128.16, 127.96, 127.64, 127.50, 127.20, 126.97, 125.85, 124.31,

124.14, 122.84, 122.46, 121.82, 75.01, 73.56, 66.90, 66.16, 38.36, 37.73, 19.81, 16.85, 16.60, 15.84. **HRMS** (ESI-TOF) *m/z* calcd for $C_{24}H_{25}NO_2Cl$ $[(M + H)^+]$ 394.1574, found 394.1565.

3-((3-bromophenyl)(methyl)amino)-3-phenylbutan-2-yl benzoate (3aaad)

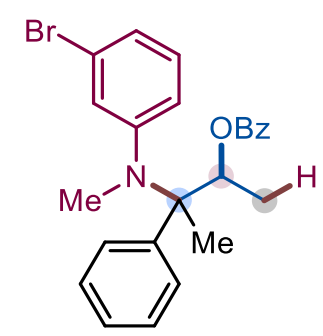

The reaction was performed according to General Procedure B at 40°C to afford the title compound (67.5 mg, 77% yield) as a colorless sticky oil. $\mathbf{R_f}$ = 0.4 [Hex: EtOAc 20:1 (v/v)]. *Major diastereomers*: **^{1}H NMR** (500 MHz, $CDCl_3$) δ 7.83 (d, *J* = 6.8 Hz, 2H), 7.58 – 7.47 (m, 2H), 7.47 – 7.35 (m, 4H), 7.35 – 7.21 (m, 2H), 7.18 (t, *J* = 1.9 Hz, 1H), 6.98 – 6.81 (m, 3H), 5.78 – 5.68 (m, 1H), 2.69 (s, 3H), 1.76 (s, 3H), 1.07 (d, *J* = 6.3 Hz, 3H). *Mixture of diastereomers*: **^{13}C NMR** (126 MHz, $CDCl_3$) δ 165.92, 165.52, 152.48, 151.48, 143.22, 142.05, 132.98, 132.81, 130.54, 130.32, 129.60, 129.43, 129.32, 129.21, 128.89, 128.83, 128.40, 128.27, 128.17, 127.95, 127.66, 127.49, 127.34, 127.21, 126.99, 125.84, 124.81, 124.72, 123.02, 121.96, 75.01, 73.53, 66.94, 66.18, 38.38, 37.76, 19.76, 16.87, 16.44, 15.85. **HRMS** (ESI-TOF) *m/z* calcd for $C_{24}H_{25}NO_2Br$ $[(M + H)^+]$ 438.1069, found 438.1061.

3-(ethyl(phenyl)amino)-3-phenylbutan-2-yl benzoate (3aaae)

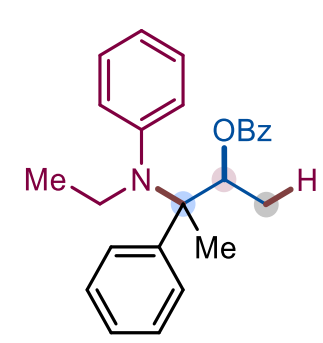

The reaction was performed according to General Procedure B at 40°C to afford the title compound (26.9 mg, 36% yield) as a colorless sticky oil. $\mathbf{R_f}$ = 0.4 [Hex: EtOAc 20:1 (v/v)]. *Major diastereomer*: **^{1}H NMR** (300 MHz, $CDCl_3$) δ 7.81 – 7.72 (m, 2H), 7.69 – 7.61 (m, 2H), 7.56 – 7.27 (m, 6H), 7.23 – 7.16 (m, 2H), 7.17 – 7.02 (m, 2H), 6.85 – 6.74 (m, 1H), 5.60 (q, *J* = 6.3 Hz, 1H), 3.02 – 2.86 (m, 1H), 2.86 – 2.70 (m, 1H), 1.73 (s, 3H), 0.92 (d, *J* = 6.3 Hz, 3H), 0.63 (t, *J* = 7.0 Hz, 3H). *Major diastereomer*: **^{13}C NMR** (126 MHz, $CDCl_3$) δ 165.47, 147.91, 144.48, 132.60, 130.70, 130.22, 129.58, 128.51, 128.41, 128.17, 127.99, 127.75, 127.08, 125.14, 75.07, 67.68, 44.01, 17.19, 15.98, 14.29. **HRMS** (ESI-TOF) *m/z* calcd for $C_{25}H_{28}NO_2$ $[(M + H)^+]$ 374.2120, found 374.2118.

3-(3-chlorophenyl)-3-(methyl(phenyl)amino)butan-2-yl benzoate (3aaaf)

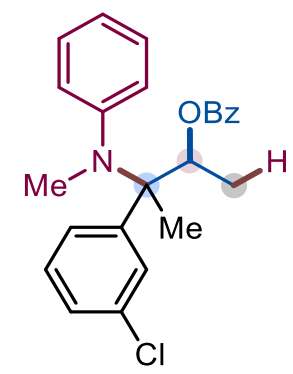

The reaction was performed according to General Procedure B at 40°C to afford the title compound (40.0 mg, 50% yield) as a colorless sticky oil. $\mathbf{R_f}$ = 0.4 [Hex: EtOAc 20:1 (v/v)]. *Major diastereomer*: **^{1}H NMR** (800 MHz, $CDCl_3$) δ 7.94 (d, *J* = 7.8 Hz, 2H), 7.61 (s, 1H), 7.55 (t, *J* = 7.5 Hz, 1H), 7.43 (t, *J* = 7.6 Hz, 2H), 7.36 (d, *J* = 7.2 Hz, 1H), 7.25 – 7.22 (m, 2H), 7.17 (t, *J* = 7.7 Hz, 2H), 7.02 (t, *J* = 7.3 Hz, 1H), 6.94 (d, *J* = 7.8 Hz, 2H), 5.54 (q, *J* = 6.3 Hz, 1H), 2.69 (s, 3H), 1.65 (s, 3H), 1.27 (d, *J* = 7.1 Hz, 3H). *Major diastereomer*: **^{13}C NMR** (201 MHz, $CDCl_3$) δ 165.91, 144.58, 134.04, 133.07, 130.70, 129.69, 128.97, 128.54, 128.52, 127.17, 126.63, 126.16, 123.81, 73.37, 66.20, 38.29, 18.55, 15.96. **HRMS** (ESI-TOF) *m/z* calcd for $C_{24}H_{25}NO_2Cl$ [$(M + H)^+$] 394.1574, found 394.1569.

1-(methyl(phenyl)amino)-1-phenylpropan-2-yl benzoate (3aaag)

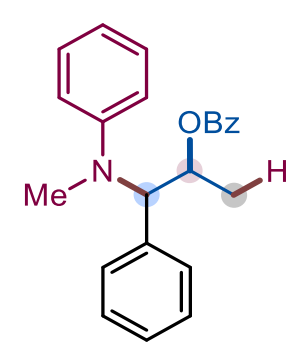

The reaction was performed according to General Procedure B at 80°C to afford the title compound (49.2 mg, 72% yield) as a colorless oil. $\mathbf{R_f}$ = 0.4 [Hex: EtOAc 20:1 (v/v)]. *Major diastereomer*: **^{1}H NMR** (500 MHz, $CDCl_3$) δ 7.85 (dd, *J* = 8.3, 1.4 Hz, 2H), 7.55 – 7.44 (m, 1H), 7.36 (t, *J* = 7.8 Hz, 2H), 7.31 – 7.16 (m, 7H), 6.95 (d, *J* = 8.2 Hz, 2H), 6.79 (t, *J* = 7.2 Hz, 1H), 5.92 (dq, *J* = 9.5, 6.2 Hz, 1H), 5.22 (d, *J* = 9.5 Hz, 1H), 2.65 (s, 3H), 1.51 (d, *J* = 6.2 Hz, 3H). *Major diastereomer*: **^{13}C NMR** (201 MHz, $CDCl_3$) δ 166.00, 150.31, 137.75, 132.91, 130.31, 129.55, 129.31, 128.33, 128.28, 127.76, 127.37, 117.59, 113.80, 70.28, 65.86, 32.57, 18.41. **HRMS** (ESI-TOF) *m/z* calcd for $C_{23}H_{24}NO_2$ [$(M + H)^+$] 346.1807, found 346.1808.

Gram-Scale Reaction

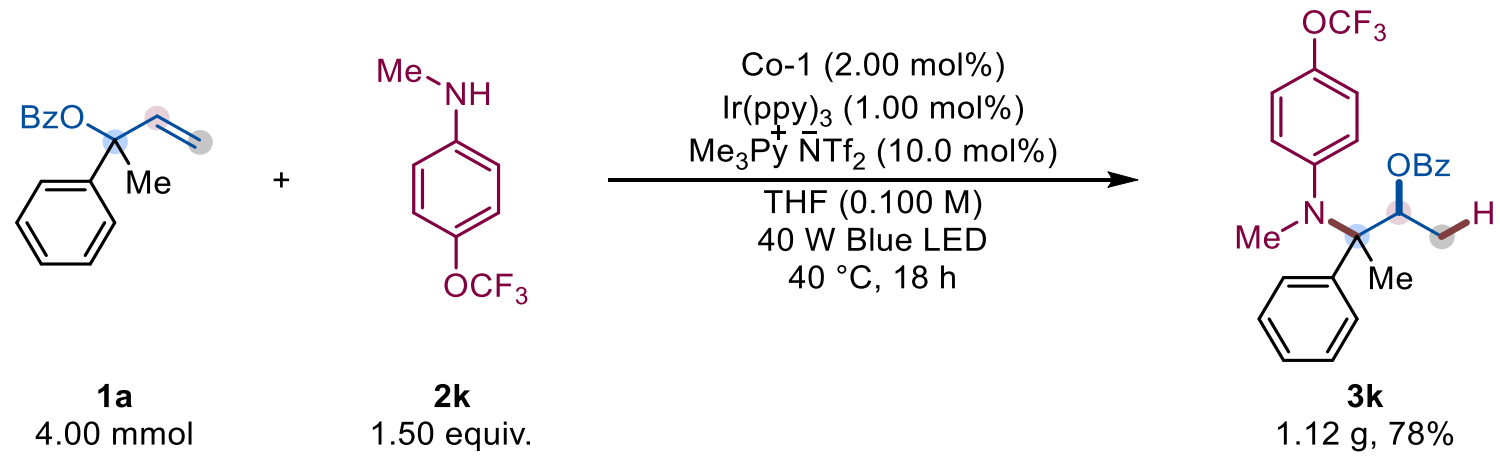

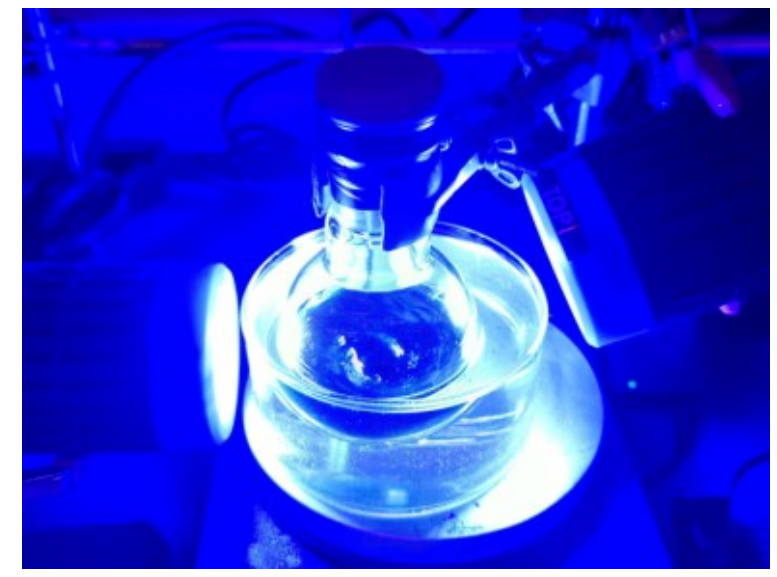

In a glovebox, to an oven-dried 100 mL round bottom flask was added **Co-1** (56.1 mg, 80.0 µmol, 2.00 mol%), Ir(ppy)$_3$ (26.2 mg, 40.0 µmol, 1.00 mol%), 2,4,6-trimethylpyridin-1-ium bis((trifluoromethyl)sulfonyl)amide (161 mg, 400 µmol, 10.0 mol%), **1a** (1.01 g, 4.00 mmol, 1.00 equiv.), **2k** (1.15 g, 896 µL, 6.00 mmol, 1.50 equiv.) in THF (40.0 mL). The round bottom flask was equipped with a stir bar, capped with rubber stopper, and sealed with electrical tape, then taken out of the glovebox. The reaction mixture was stirred at 40 °C and irradiated with 40W blue LEDs for 18 hours. The reaction mixture was then concentrated *in vacuo* and residue was purified by flash column chromatography on silica gel to afford the desired product compound **3k** (1.12 g, 3.12 mmol, 78% yield).

Mechanistic Studies

Radical trap using TEMPO

The procedure is based on General Procedure B: In a glovebox, to an oven-dried 4 mL screw cap vial was added **Co-1** (0.55 mg, 1.00 μmol, 2.00 mol%), Ir(ppy)$_3$ (0.33 mg, 0.500 μmol, 1.00 mol%), 2,4,6-trimethylpyridin-1-ium bis((trifluoromethyl)sulfonyl)amide (2.01 mg, 5.00 μmol, 10.0 mol%), **1a** (12.6 mg, 0.0500 mmol, 1.00 equiv.), **2a** (8.04 mg, 0.0750 mmol, 1.50 equiv.), TEMPO (11.7 mg, 0.0750 mmol, 1.50 equiv.) and THF (0.500 mL, 0.100 M). The vial was equipped with a stir bar, capped and sealed with electrical tape, then taken out of the glovebox. The reaction mixture was stirred at 40 °C and irradiated with 40 W Blue LED for 18 hours. No product formation was observed in crude NMR.

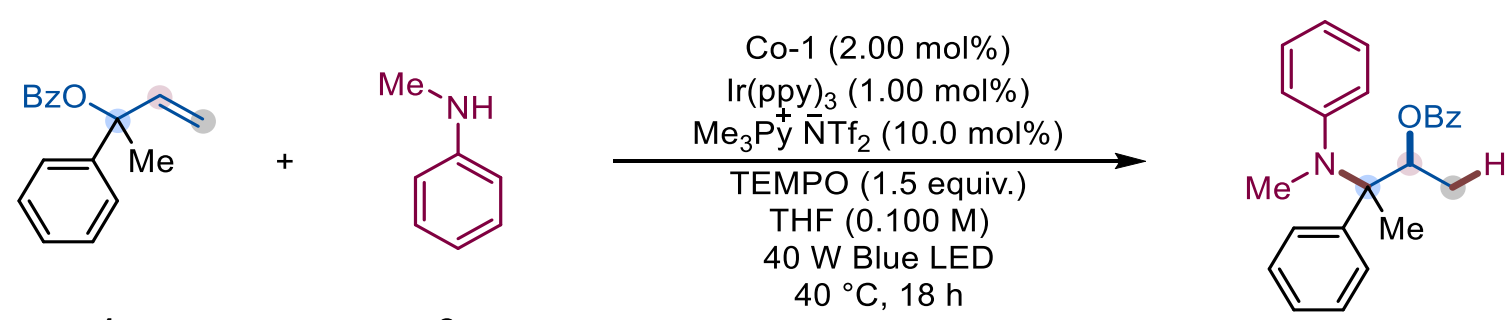

Figure S3. Radical trap using TEMPO

Results and Conclusion: No desired product was observed. The reaction is likely to involve radical intermediates.

Radical trap using benzenemethanethiol

The procedure is based on General Procedure B: In a glovebox, to an oven-dried 4 mL screw cap vial was added **Co-1** (0.55 mg, 1.00 μmol, 2.00 mol%), Ir(ppy)$_3$ (0.33 mg 0.500 μmol, 1.00 mol%), 2,4,6-trimethylpyridin-1-ium bis((trifluoromethyl)sulfonyl)amide (2.01 mg, 5.00 μmol, 10.0 mol%), **1l** (14.1 mg, 0.0500 mmol, 1.00 equiv.), **2a** (8.04 mg, 0.0750 mmol, 1.50 equiv.), benzenemethanethiol (9.32 mg, 0.0750 mmol, 1.50 equiv.) and THF (0.500 mL, 0.100 M). The vial was equipped with a stir bar, capped and sealed with electrical tape, then taken out of the glovebox. The reaction mixture was stirred at 40 °C and irradiated with 40 W Blue LED for 18 h. The reaction mixture was then concentrated in vacuo and residue was purified on silica hexanes: EtOAc [40:1 (v/v)] to afford the desired compound **5a** as a colorless oil (4.41 mg, 15.5 μmol) in 31% yield. ^{1}**H NMR** (800 MHz, $CDCl_3$) δ 8.10 – 8.04 (m, 2H), 7.58 – 7.53 (m, 1H), 7.47 – 7.40 (m, 2H), 7.23 (t, J = 7.8 Hz, 1H), 6.85 (s, 1H), 6.81 – 6.72 (m, 2H), 5.28 (dt, J = 7.7, 6.2 Hz, 1H), 3.78 (s, 3H), 3.00 (p, J = 7.2 Hz, 1H), 1.36 (d, J = 7.0 Hz, 3H), 1.20 (d, J = 6.3 Hz, 3H). ^{13}**C NMR** (201 MHz, $CDCl_3$) δ 166.29, 159.81, 145.09, 132.98, 130.90, 129.72, 129.57, 128.51, 120.52, 113.92, 112.05, 75.53, 55.29, 45.64, 18.42, 17.98. **HRMS** (EI+) *m/z* calcd for $C_{18}H_{20}O_3Na$, 307.1310, found 307.1314.

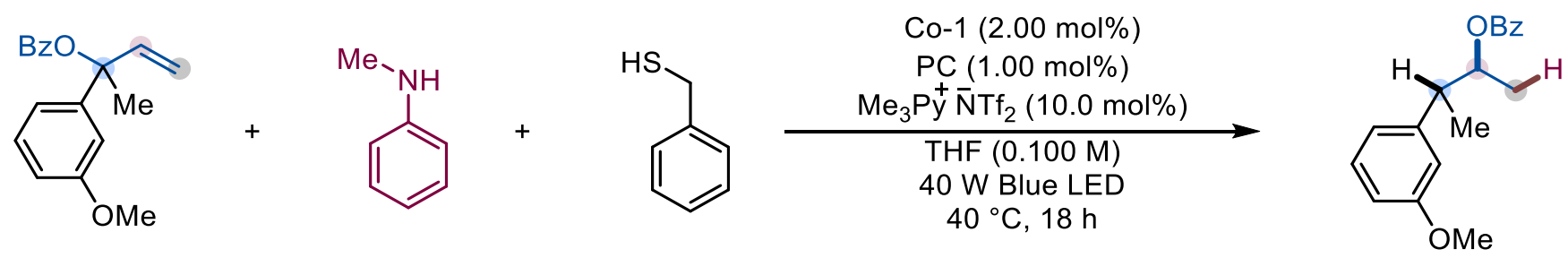

1l 0.0500 mmol | **2a** 1.50 equiv. | 1.50 equiv. | **5a** 31%

Figure S4. Radical trap using benzenemethanethiol

Results and Conclusion: Reduced product **5a** was observed. Migration likely proceeds via a radical pathway.

Asymmetric Induction Probing

In a glovebox, to an oven-dried 4 mL screw cap vial was added **Co-4** (0.79 mg, 1.00 µmol, 2.00 mol%), Ir(ppy)3 (0.33 mg, 0.500 µmol, 1.00 mol%), 2,4,6-trimethylpyridin-1-ium bis((trifluoromethyl)sulfonyl)amide (2.01 mg, 5.00 µmol, 10.0 mol%), **1a** (12.6 mg, 0.0500 mmol, 1.00 equiv.), **2k** (14.3 mg, 0.0750 mmol, 1.50 equiv.), and THF (0.500 mL, 0.100 M). The vial was equipped with a stir bar, capped and sealed with electrical tape, then taken out of the glovebox. The reaction mixture was stirred at -20 °C and irradiated with 40 W Blue LED for 48 hours. HPLC analysis (Dicel CHIRALPAK OX 5 mic, hexane/ iPrOH, 50:1 v/v, flow rate 1.0 mL/min, λ = 220 nm, 25 ℃), tR (major) = 5.312 min, tR (minor) = 4.489 min.

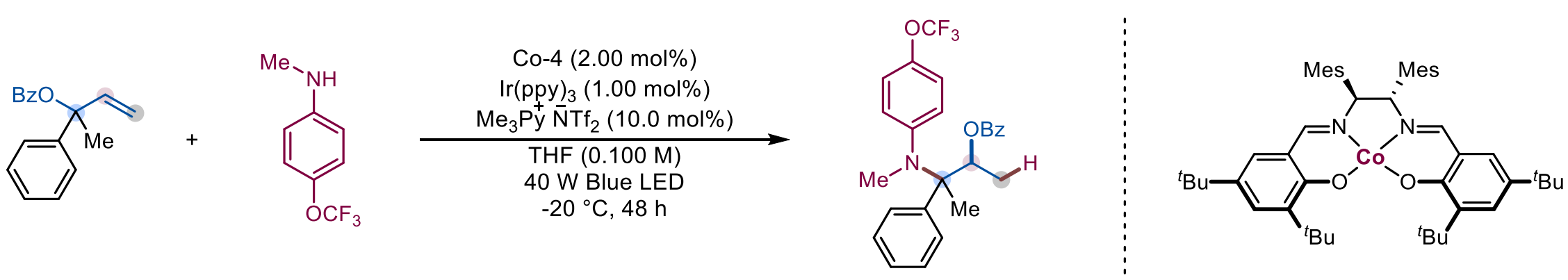

1a 0.0500 mmol | **2k** 1.50 equiv. | **3k** 21% yield, 24% ee | **Co-4**

Figure S5. Asymmetric induction probing with chiral catalyst

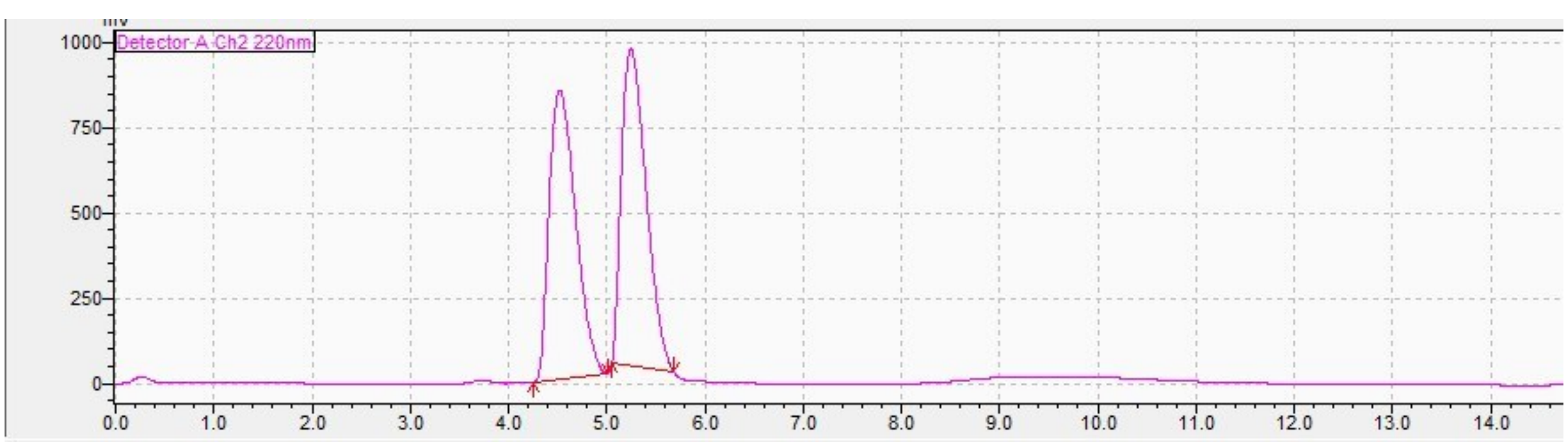

Results View - Peak Table

Peak Table | Compound | Group | Calibration Curve

Peak#	Ret. Time	Area	Height	Mark	Conc.	Unit	ID#	Name	Area%
1	4.522	15931169	848375	M	49.557				49.557
2	5.248	16215894	928029	M	50.443				50.443
Total		32147063	1776404		100.000				100.000

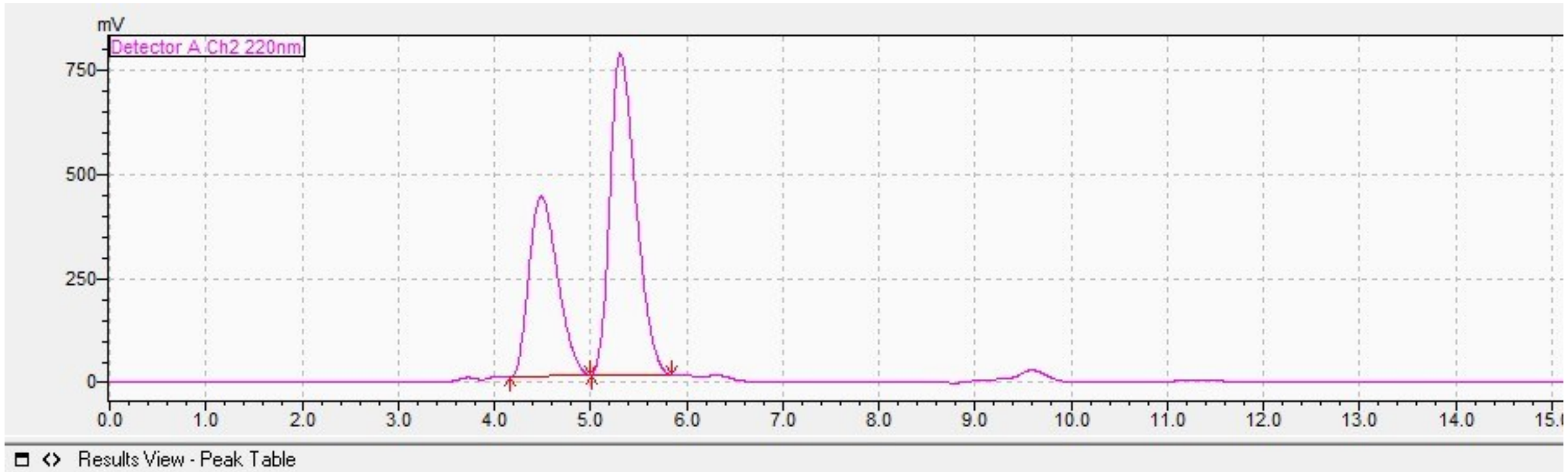

Peak#	Ret. Time	Area	Height	Mark	Conc.	Unit	ID#	Name	Area%
1	4.489	8954438	432293	M	38.063				38.063
2	5.312	14570707	772781	M	61.937				61.937
Total		23525145	1205074		100.000				100.000

Figure S6. HPLC results for asymmetric induction probing

Results and Conclusion: Enantiomerically enriched product was observed. Co-catalyst is involved in the delivery of aniline.

^{18}O-Labeling Experiment

Prepared according to General Procedure B: In a glovebox, to an oven-dried 4 mL screw cap vial was added **Co-1** (0.55 mg, 1.00 μmol, 2.00 mol%), Ir(ppy)$_3$ (0.33 mg, 0.500 μmol, 1.00 mol%), 2,4,6-trimethylpyridin-1-ium bis((trifluoromethyl)sulfonyl)amide (2.01 mg, 5.00 μmol, 10.0 mol%), *^{18}O*-**1a** which was prepared following general procedure 1 using ^{18}O-labeled benzoylchoride[4] (12.6 mg, 0.0500 mmol, 1.00 equiv.), **2k** (14.3 mg, 0.0750 mmol, 1.50 equiv.), and THF (0.500 mL, 0.100 M). The vial was equipped with a stir bar, capped and sealed with electrical tape, then taken out of the glovebox. The reaction mixture was stirred at 40 °C and irradiated with 40 W Blue LED for 18 hours. Product was obtained as a yellow sticky oil (16.5 mg, 0.0370 mmol, 74% yield, *^{18}O*-**3k-1**:*^{18}O*-**3k-2** = 1.7:1). The ratio was determined after the deprotection of benzoyl group.

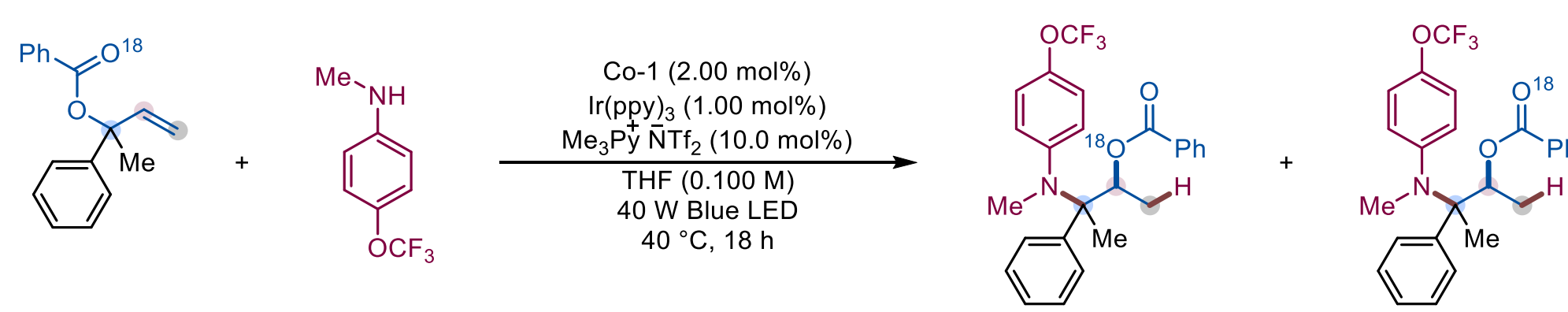

^{18}O-**1a** (95% ^{18}O) 0.0500 mmol

2k 1.50 equiv.

74%, ^{18}O-**3k-1**:^{18}O-**3k-2** = 1.7:1

Figure S7. ^{18}O-Labeling experiment

Deprotection of *^{18}O*-3k-1 and *^{18}O*-3k-2

To the resulting mixture of *^{18}O*-**3k-1** and *^{18}O*-**3k-2** in MeOH/H_2O (0.500 mL, 1:1, v/v) was added excess KOH. The reaction mixture was stirred at room temperature for 20 minutes. Then the reaction mixture was diluted with MeCN and injected to LC-MS, *^{18}O*-**3k'** and *^{16}O*-**3k'** ratio was determined to be 1.7:1.

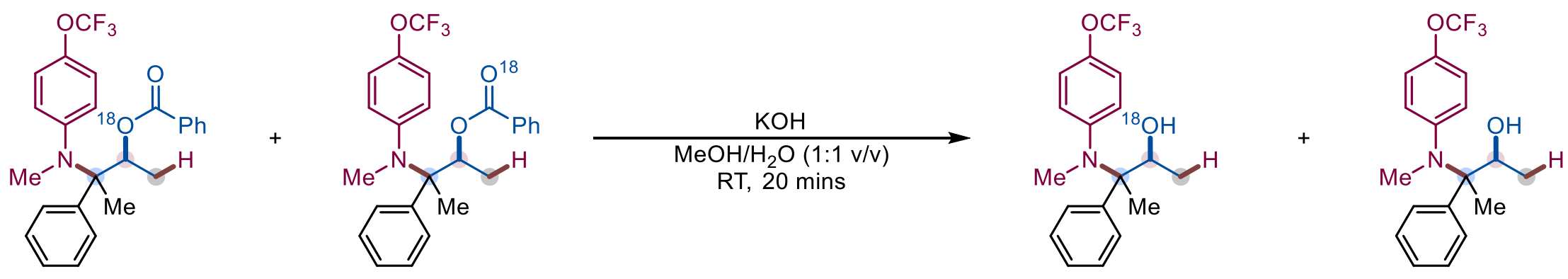

^{18}O-**3k-1** ^{18}O-**3k-2** ^{18}O-**3k'**:^{16}O-**3k'** = 1.7:1

Figure S8. Deprotection of benzoyl group for ^{18}O-Labeling experiment

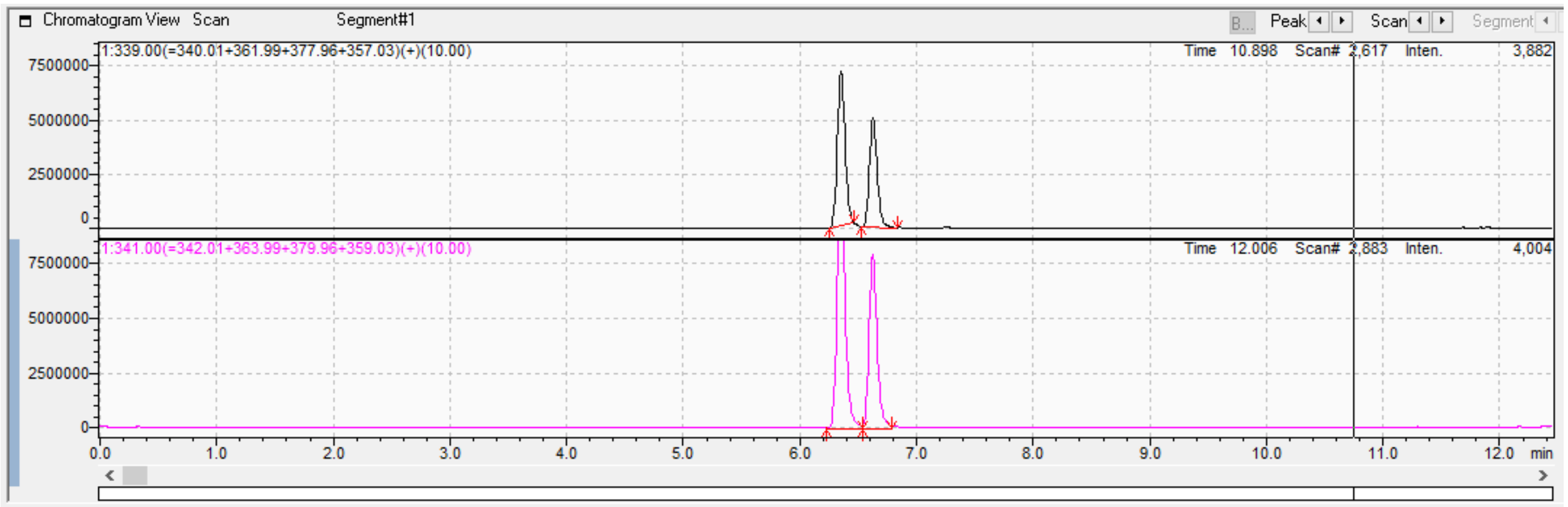

Spectrum | Peak Table | Compound | Group | Calibration Curve

Mass, Compound Name, PeakTable

339.00(=340.01+361.99+37
341.00(=342.01+363.99+37

Peak#	Ret. Time	Area	S/N	Mark	Conc.	Unit	ID#	Name	m.
1	6.351	3223519	108.52		0.000				
2	6.627	2325585	76.82	M	0.000				
Total		5549105			0.000				

Mass, Compound Name, PeakTable

339.00(=340.01+361.99+37
341.00(=342.01+363.99+37

Peak#	Area	S/N	Mark	Conc.	Unit	ID#	Name
1	5372083	78.15	M	0.000			
2	3874274	56.97	V M	0.000			
Total	9246357			0.000			

Figure S9. LCMS spectrum

Control for ^{18}O-labeling experiment

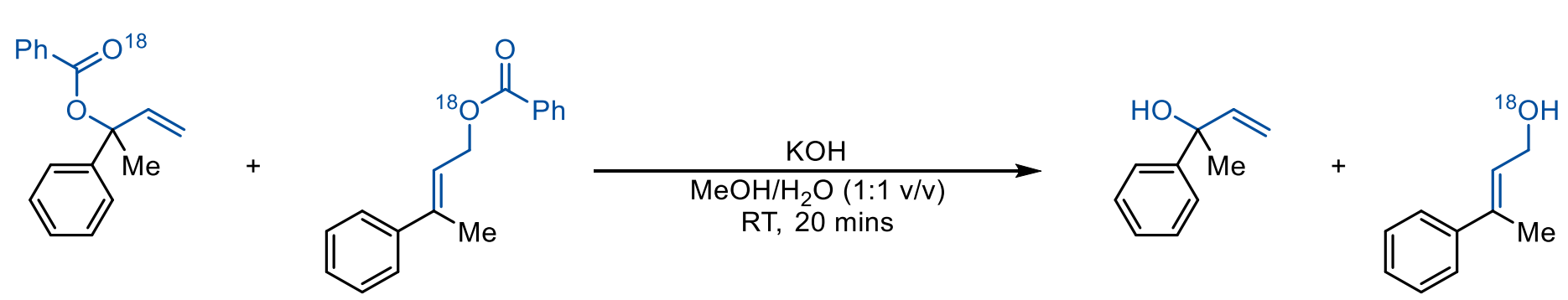

^{18}O-**1a** 9:1 ^{18}O-**1a'** ^{16}O-**1a''** 9:1 ^{18}O-**1a'''**

Figure S10. First control for ^{18}O-labeling experiment

To ^{18}*O*-**1a** with 10% sigmatropic rearraged material ^{18}*O*-**1a'** in MeOH/H_2O (0.500 mL, 1:1, v/v) was added excess KOH. The reaction mixture was stirred at room temperature for 20 minutes. Then the reaction mixture was diluted with MeCN and injected to GC-MS, ^{16}*O*-**1a"** and ^{18}*O*-**1a'''** ratio was determined to be 9:1.

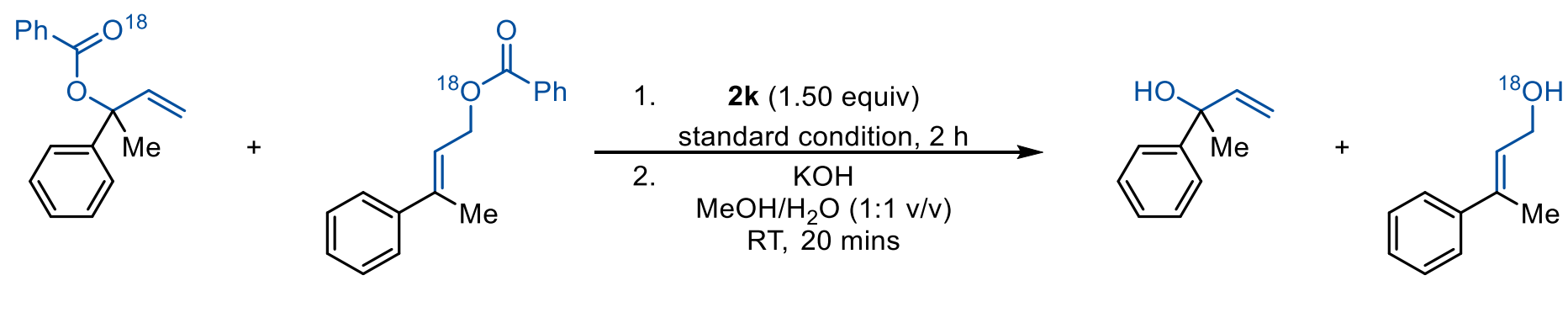

^{18}O-**1a** 9:1 ^{18}O-**1a'** ^{16}O-**1a"** 9:1 ^{18}O-**1a'''**

Figure S11. Second control for ^{18}O-labeling experiment

In a glovebox, to an oven-dried 4 mL screw cap vial was added **Co-1** (0.55 mg, 1.00 μmol, 2.00 mol%), Ir(ppy)$_3$ (0.33 mg, 0.500 μmol, 1.00 mol%), 2,4,6-trimethylpyridin-1-ium bis((trifluoromethyl)sulfonyl)amide (2.01 mg, 5.00 μmol, 10.0 mol%), ^{18}*O*-**1a** with 10% sigmatropic rearraged material ^{18}*O*-**1a'** (12.6 mg, 0.0500 mmol, 1.00 equiv.), **2k** (14.3 mg, 0.0750 mmol, 1.50 equiv.), and THF (0.500 mL, 0.100 M). The vial was equipped with a stir bar, capped and sealed with electrical tape, then taken out of the glovebox. The reaction mixture was stirred at 40 °C and irradiated with 40 W Blue LED for 2 hours. The reaction mixture was then concentrated in vacuo and residue was purified on silica to retrieve ^{18}*O*-**1a** and ^{18}*O*-**1a'**. To the resulting mixture of ^{18}*O*-**1a** and ^{18}*O*-**1a'** was added excess KOH. The reaction mixture was stirred at room temperature for 20 minutes. Then the reaction mixture was diluted with MeCN and injected to GC-MS, ^{16}*O*-**1a"** and ^{18}*O*-**1a'''** ratio was determined to be 9:1.

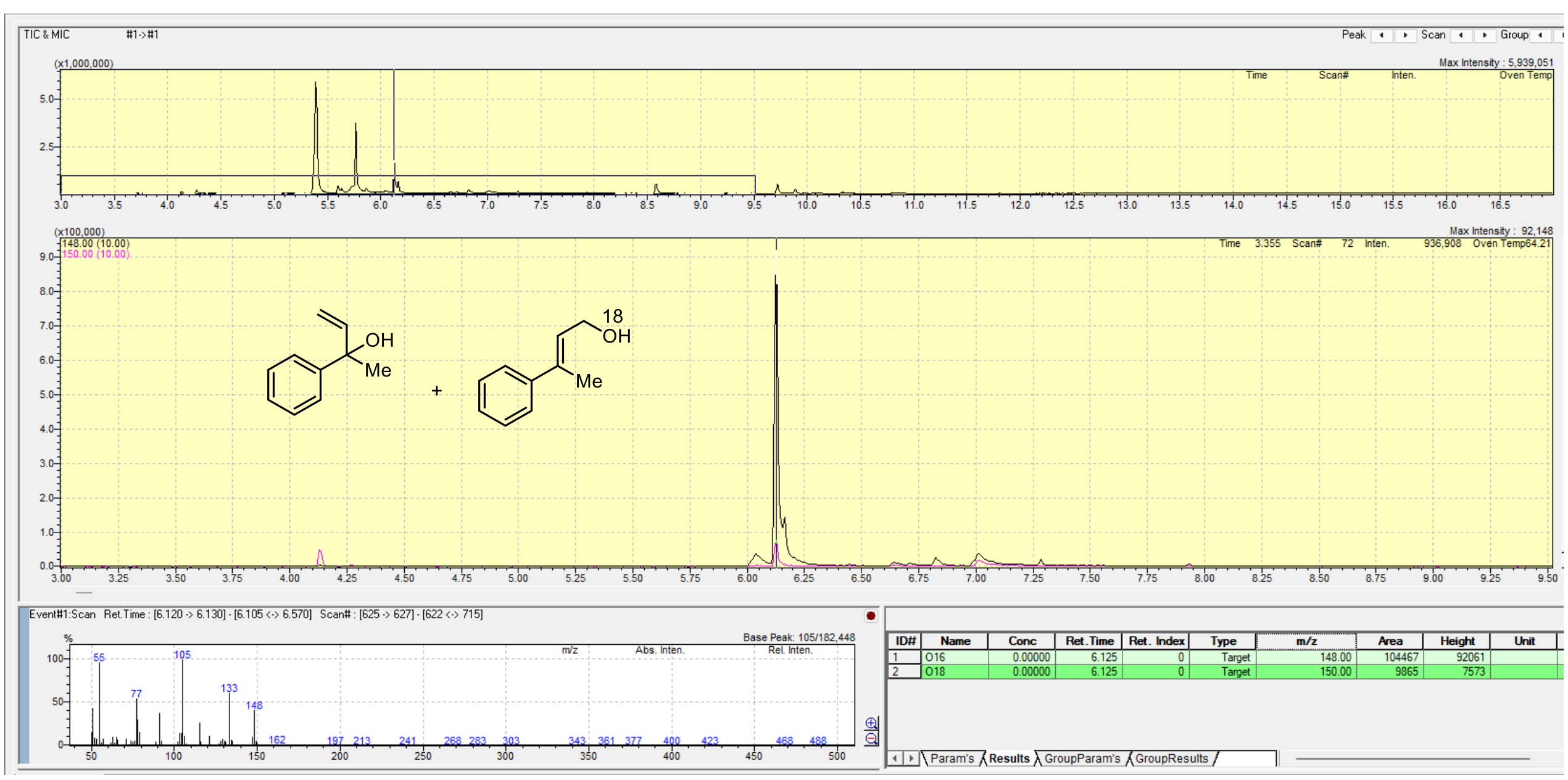

Figure S 12. GC-MS analysis of deprotected O^{18} labeled starting material

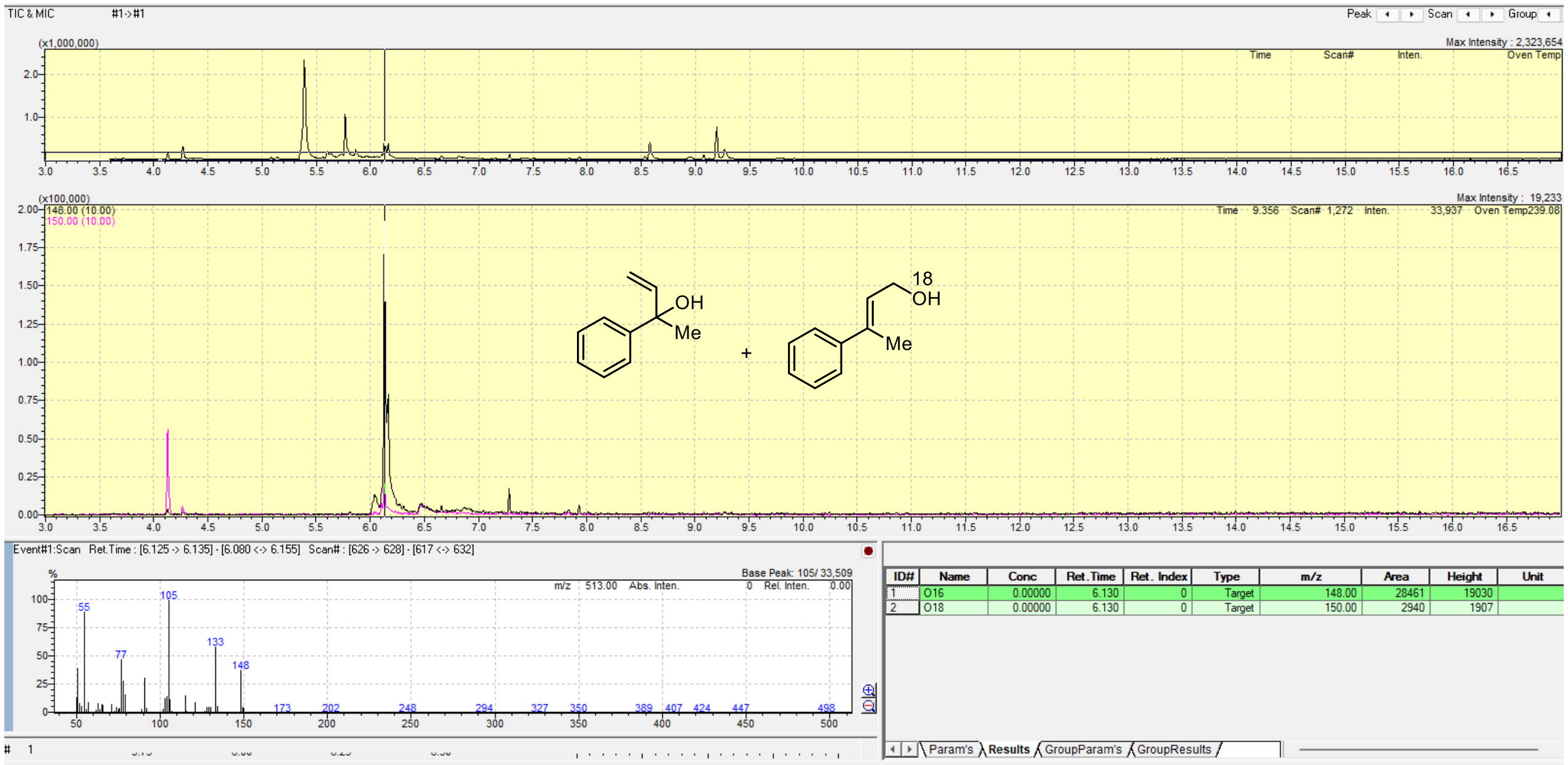

Figure S 13. GC-MS analysis of deprotected O[18] labeled recovered starting material from reaction mixture

Results and Conclusion: A mixture of ^{18}O- and ^{16}O-labeled products were observed with unlikely isotope exchange under standard reaction conditions. Migration may proceed through both [2,3]- and [1,2]-TS.

Deuterium-Labeling Experiment

Deuterium labeling experiment with *N*-methylaniline-d_1

The procedure is based on General Procedure B: In a glovebox, to an oven-dried 4 mL screw cap vial was added **Co-1** (0.55 mg, 1.00 µmol, 2.00 mol%), Ir(ppy)$_3$ (0.33 mg, 0.500 µmol, 1.00 mol%), 2,4,6-trimethylpyridin-1-ium bis((trifluoromethyl)sulfonyl)amide (2.01 mg, 5.00 µmol, 10.0 mol%), **1a** (12.6 mg, 0.0500 mmol, 1.00 equiv.), **2a**-d_1 (8.11 mg, 0.0750 mmol, 1.50 equiv.) and THF (0.500 mL, 0.100 M). The vial was equipped with a stir bar, capped and sealed with electrical tape, then taken out of the glovebox. The reaction mixture was stirred at 40 °C and irradiated with 40 W Blue LED for 18 hours. Deuterium incorporation was determined using ^{1}H NMR and dibromomethane as the internal standard.

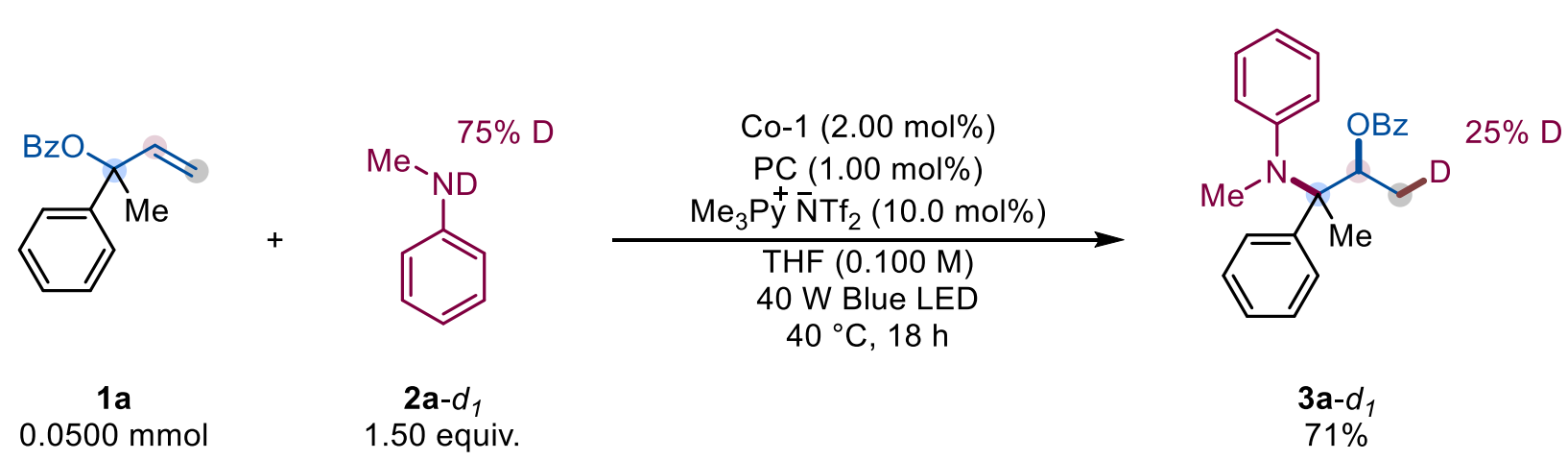

Figure S14. Deuterium-labeling experiment with *N*-methylaniline-d_1

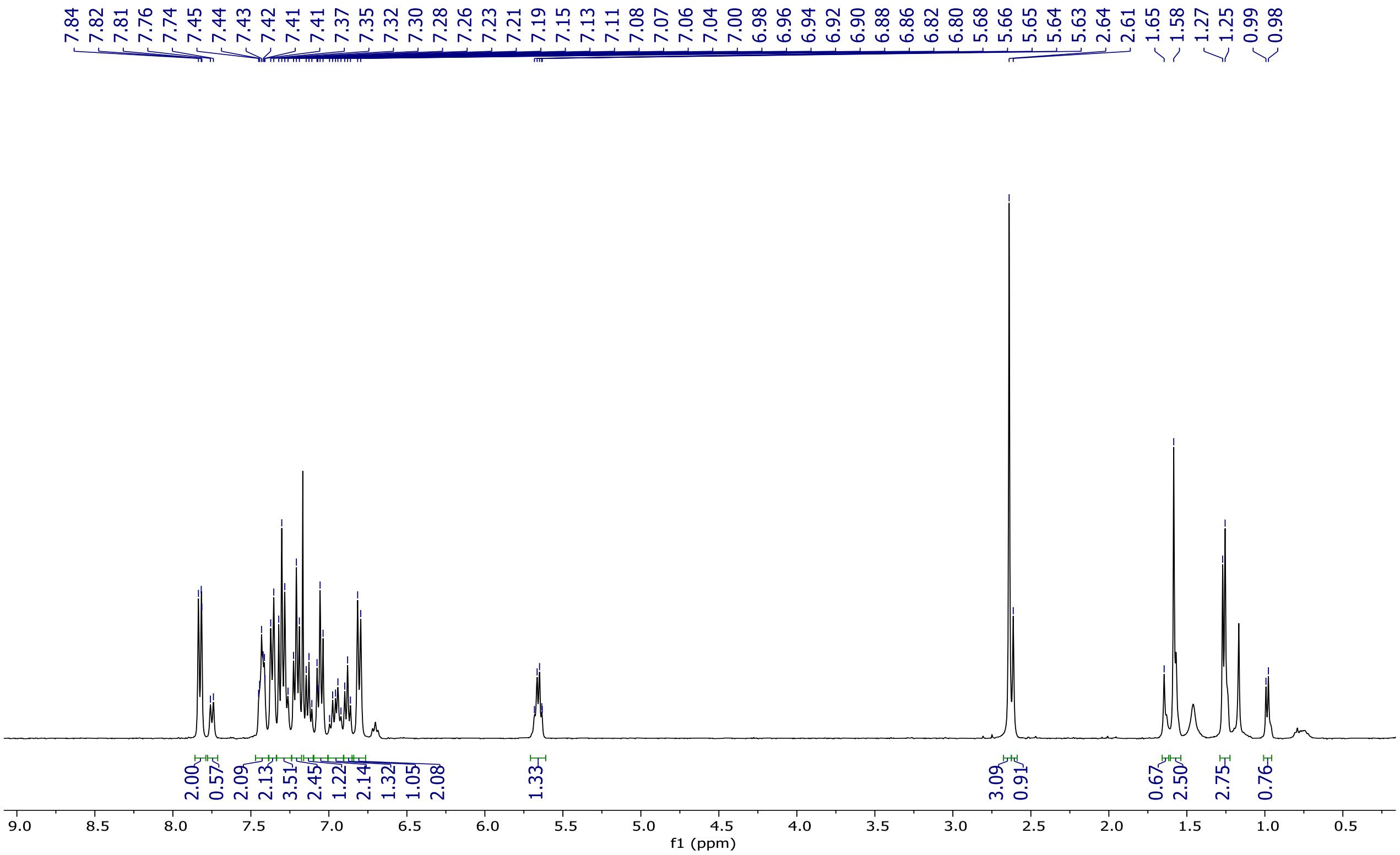

Figure S15. ^{1}H NMR for deuterium-labeling experiment with *N*-methylaniline-d_1

Deuterium labeling experiment with THF-d_8

The procedure is based on General Procedure B: In a glovebox, to an oven-dried 4 mL screw cap vial was added **Co-1** (0.55 mg, 1.00 µmol, 2.00 mol%), Ir(ppy)$_3$ (0.33 mg, 0.500 µmol, 1.00 mol%), 2,4,6-trimethylpyridin-1-ium bis((trifluoromethyl)sulfonyl)amide (2.01 mg, 5.00 µmol, 10.0 mol%), **1a** (12.6 mg, 0.0500 mmol, 1.00 equiv.), **2a** (8.04 mg, 0.0750 mmol, 1.50 equiv.) and THF-d_8 (0.500 mL, 0.100 M). The vial was equipped with a stir bar, capped and sealed with electrical tape, then taken out of the glovebox. The reaction mixture was stirred at 40 °C and irradiated with 40 W Blue LED for 18 hours. Deuterium incorporation was determined using ^{1}H NMR and dibromomethane as the internal standard.

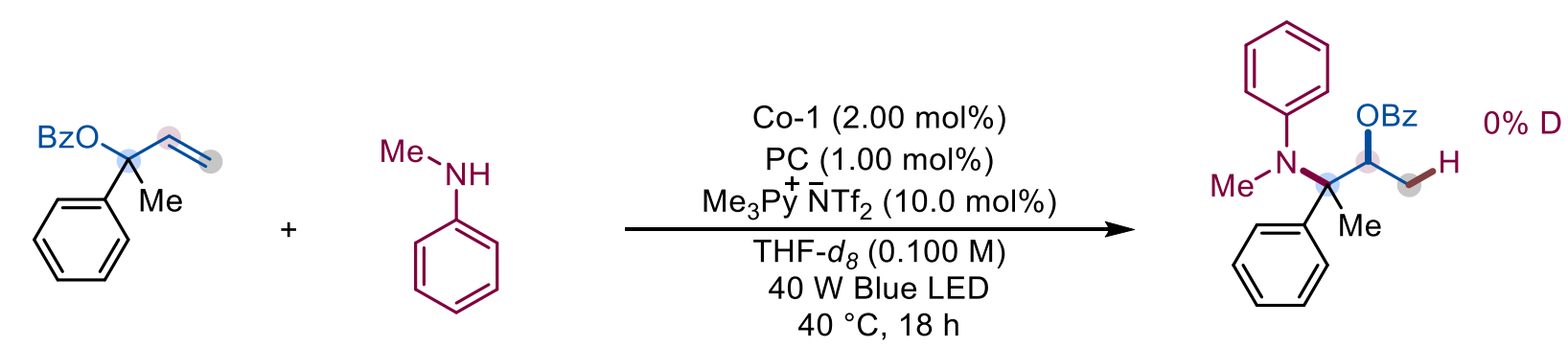

1a 0.0500 mmol

2a 1.50 equiv.

3a 68%

Figure S16. Deuterium-Labeling experiment with THF-d_8

Results and Conclusion: Product bearing deuterium was observed when **2a**-d$_1$ was used as the substrate. Aniline is likely a hydrogen atom source for the reaction.

Cross-over Experiment

The procedure is based on General Procedure B: In a glovebox, to an oven-dried 4 mL screw cap vial was added **Co-1** (1.10 mg, 2.00 μmol, 2.00 mol%), Ir(ppy)$_3$ (0.655 mg, 1.00 μmol, 1.00 mol%), 2,4,6-trimethylpyridin-1-ium trifluoromethanesulfonate (2.73 mg, 10.0 μmol, 10.0 mol%), **1y** (16.4 mg, 0.0500 mmol, 0.500 equiv.), **1a** (12.6 mg, 0.0500 mmol, 0.500 equiv.), **2a** (16.1 mg, 0.150 mmol, 1.50 equiv.) in THF (1.00 ml, 0.100 M). The vial was equipped with a stir bar, capped and sealed with electrical tape, then taken out of the glovebox. The reaction mixture was stirred at 40 °C and irradiated with 40 W Blue LED for 18 hours. At the end of the reaction, **3aak** and **3aaka** were not observed. The yield of **3aak** and **3a** were determined based on crude ^{1}H NMR and dibromomethane as the internal standard.

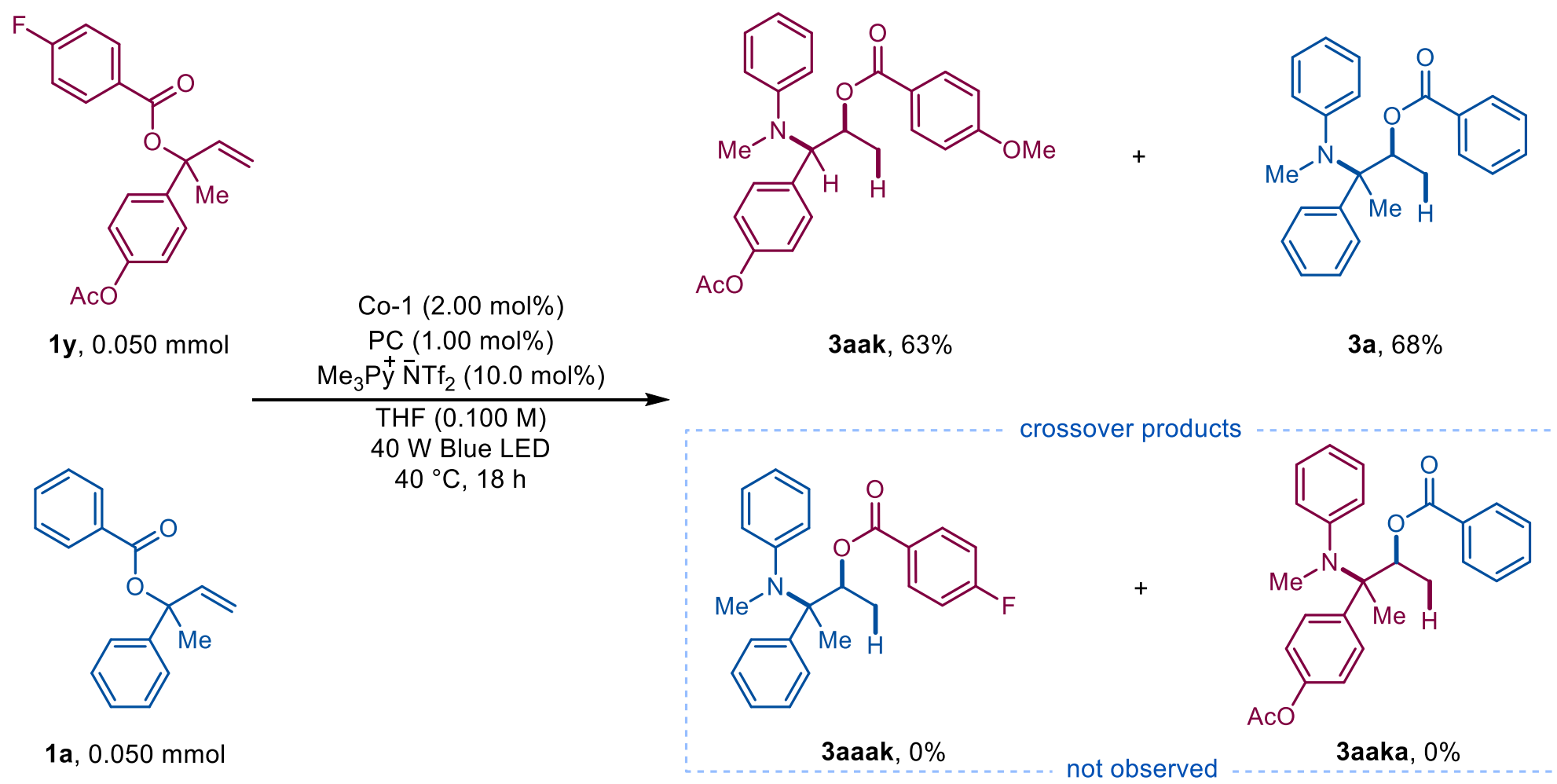

Figure S17. Cross-over experiment

Results and Conclusion: No cross-over products (**3aak** and **3aaka**) was observed. The acyloxyl migration likely proceeds in an intramolecular manner.

Stern Volmer Quenching Studies:

Inside a glovebox, a screw-top 1.0 cm quartz cuvette was loaded with a 10 μM solution of Ir(ppy)$_3$ in THF, along with quencher (**Co-1**, allyl carboxylate, aniline and **HX-1**) at varying concentrations (10 μM, 20 μM, 30 μM, 40 μM, 50 μM) in THF. The fluorescence intensity was recorded at λ = 425 nm following excitation at λ = 400 nm in the quartz cuvette.

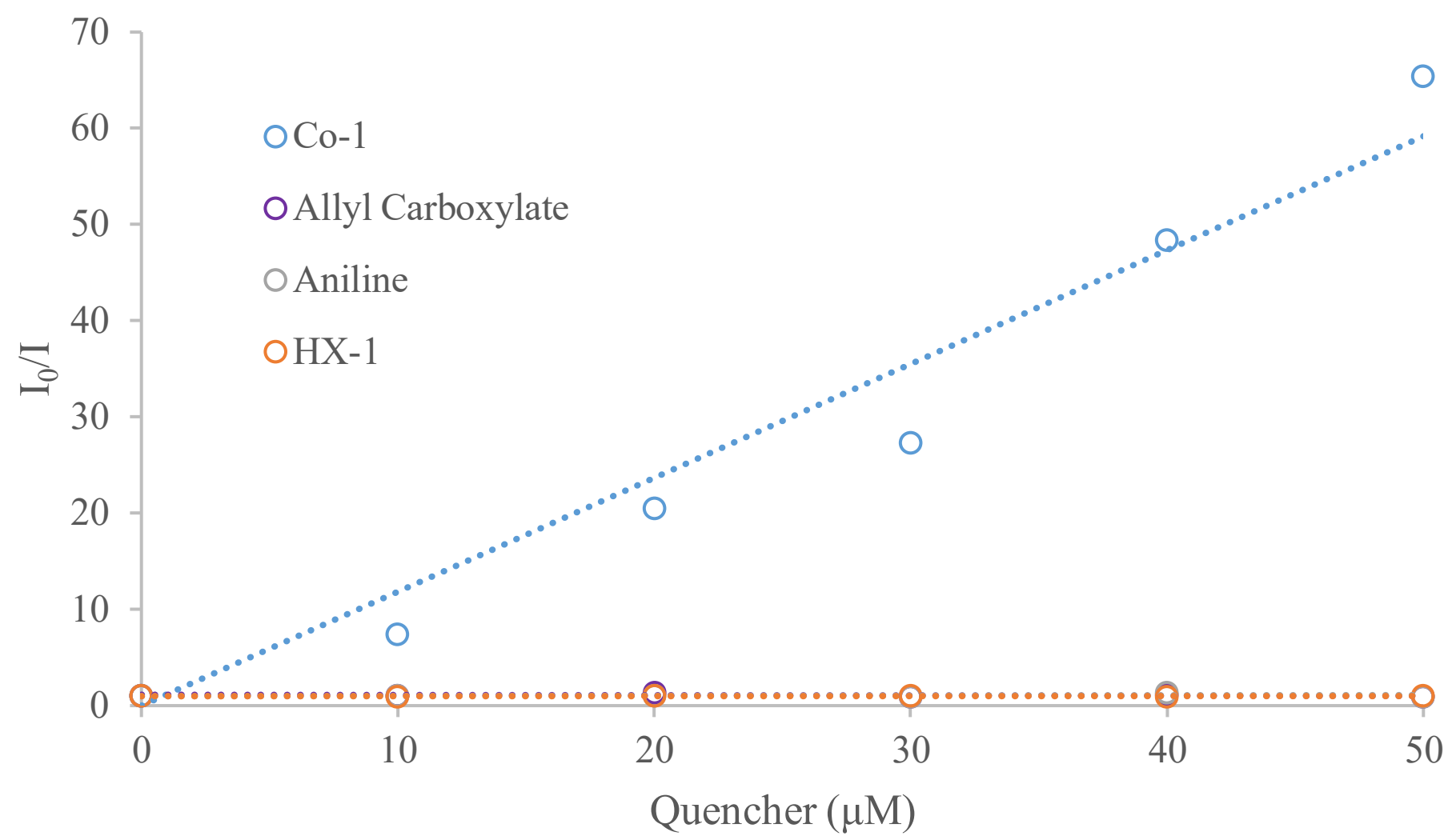

Figure S18. Stern Volmer quenching

Results and Conclusion: **Co-1** quenched efficiently excited state Ir(ppy$_3$) more efficiently than allyl carboxylate, aniline and **HX-1**.

Light on and off experiment

The procedure is based on General Procedure B: In a glovebox, to an oven-dried 4 mL screw cap vial was added **Co-1** (1.10 mg, 2.00 μmol, 2.00 mol%), Ir(ppy)$_3$ (0.66 mg 1.00 μmol, 1.00 mol%), 2,4,6-trimethylpyridin-1-ium bis((trifluoromethyl)sulfonyl)amide (4.02 mg, 10.0 μmol, 10.0 mol%), **1a** (25.2 mg, 0.100 mmol, 1.00 equiv.), **2a** (16.07 mg, 0.150 mmol, 1.50 equiv.) and THF (1.00 mL, 0.100 M). The vial was equipped with a stir bar, capped and sealed with electrical tape, then taken out of the glovebox. The reaction mixture was stirred at 40 °C. 100 μL of reaction mixture was removed after 30 minutes, and the lights were turned off for 30 minutes before another aliquot of the reaction was removed. This sequence (30 minutes lights on, 30 minutes lights off) was continued until all vials were removed. Crude ^{1}H NMR yield was obtained using dibromomethane as an internal standard.

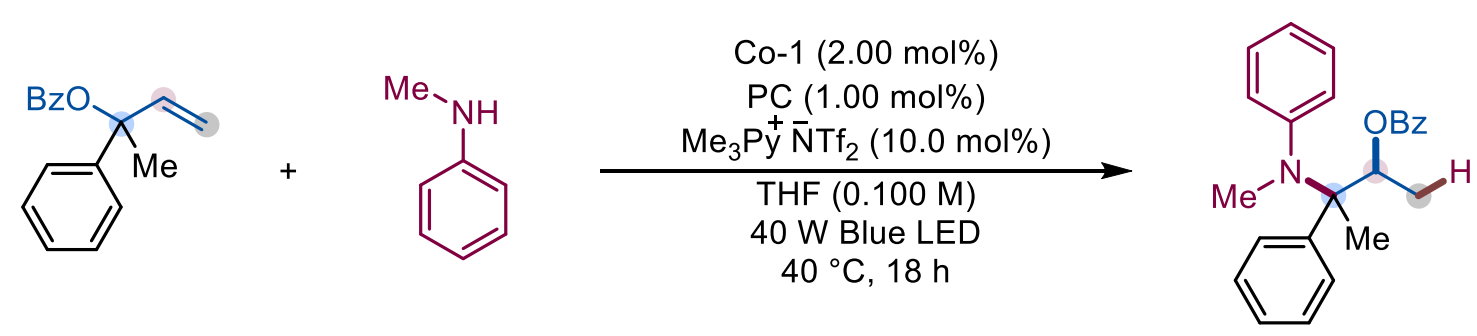

1a
0.0500 mmol

2a
1.50 equiv.

3a

Figure S19. Light on and off experiment reaction scheme

	Time (min)	Yield (%)
	0	0
on	30	13
off	60	13
on	90	17
off	120	17
on	150	21
off	180	21

Table S6. Yields from light on/off experiment

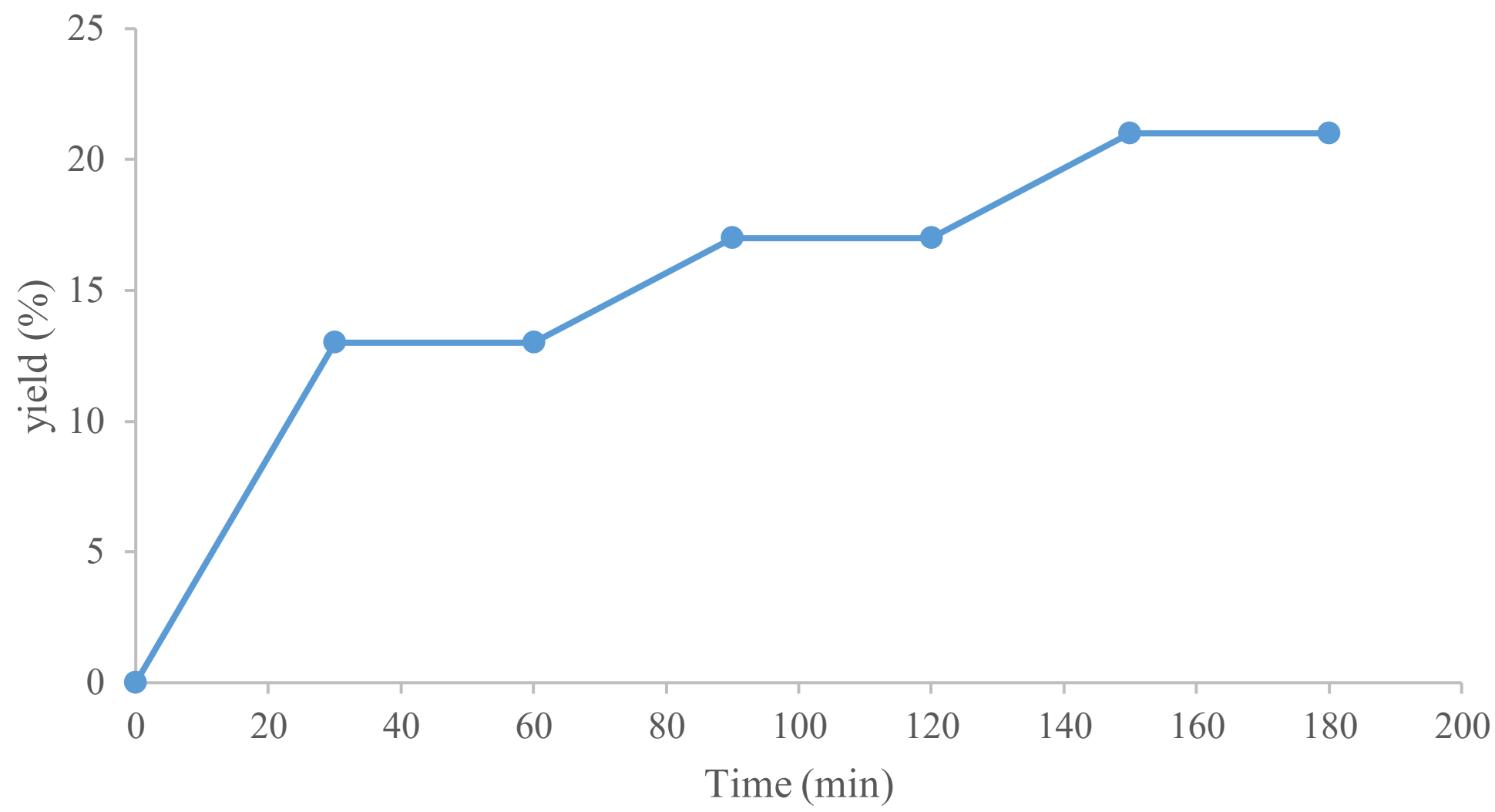

Figure S20. Light on/off experiment

Results and Conclusion: No increase in yield was observed in light off conditions. Extended radical chain mechanism is unlikely under standard condition.

Quantum Yield Experiment:

Quantum yield experiments suggest that an extended radical chain propagation is unlikely under our reaction conditions. The following quantum yield measurements are adapted from the procedure developed by Yoon et al.[5]

Determination of Fraction of Light Absorbed at 440 nm:

The fraction of light absorbed (f) by this solution was calculated as shown below. Where the absorbance of the ferrioxalate solution at 440 nm was measured to be 0.7255 (A), based on the equation ($f = 1\text{-}10^{-A}$), indicating f = 0.81186.

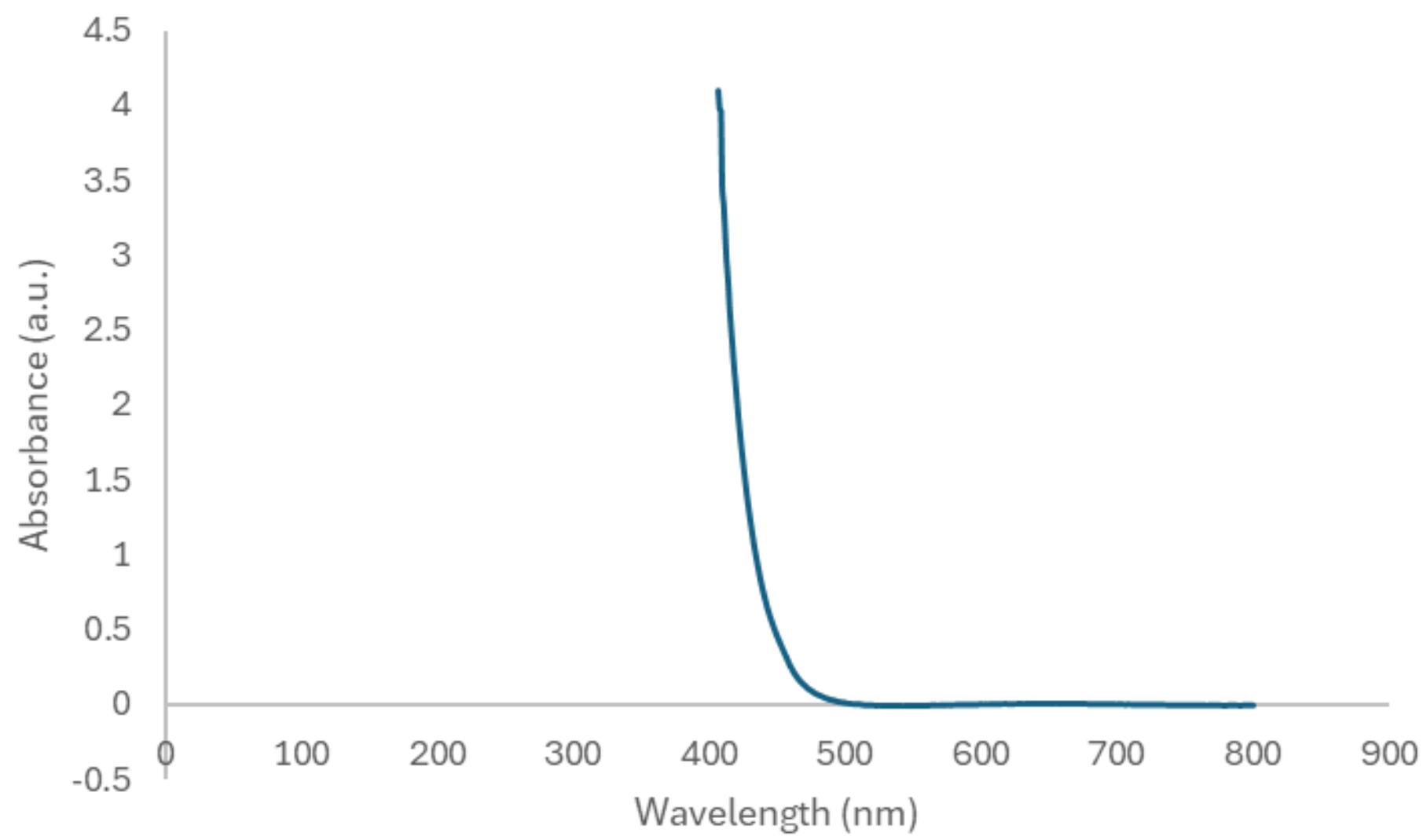

Figure S21. Absorbance of the ferrioxalate solution at 440 nm

The photon flux of the 40 W Blue LEDs (λmax = 440 nm) was determined by standard ferrioxalate actinometry. A 0.150 M solution of ferrioxalate was prepared by dissolving 2.21 g of potassium ferrioxalate hydrate ($K_3[Fe(C_2O_4)_3]\cdot 3\ H_2O$) in 30.0 mL of 0.0500 M H_2SO_4 (aq). Next, a buffered solution of phenanthroline was prepared by dissolving 50.0 mg of phenanthroline and 11.25 g of sodium acetate in 50.0 mL of 0.500 M H_2SO_4 (aq). Both solutions were stored in an amber vial in the dark. To determine the photon flux of the 46 W blue LEDs, 2.00 mL of the ferrioxalate solution was placed in a 4.00 ml vial and irradiated for 5.00 seconds at λ = 440 nm. After irradiation, 0.500 mL of the phenanthroline solution was added to the vial. The solution was then rested for 1 h in the dark to allow the ferrous ions to completely coordinate to the phenanthroline. A non-irradiated sample was also prepared and developed in the dark (*note: after developing the non-irradiated samples they were diluted with a dilution factor of 6 to prevent deviation from the Beer-Lambert law at high concentrations A = >2. Thus, to obtain the actual mol of Fe^{2+} they were multiplied by six. The values of the optical difference are the average of three trials*).

1 Ferrioxalate Actinometry

$$\text{mol of Fe}^{2+} = 6 \times \left[\frac{V \times \Delta A_{510}}{l \times \varepsilon_{510}} \right]$$

$$\text{mol of Fe}^{2+} = 6 \times \left[\frac{0.0030 \times 0.071587}{1 \times 11{,}100} \right] \text{mol}$$

$$= 1.16 \times 10^{-7}\ \text{mol}$$

V= 0.0030L *(total volume)*
ΔA_{510} = 0.071587 *(difference in absorption at 510 nm)*
l = 1.00 cm *(path length)*
ε_{510} = 11,100 L mol^{-1}cm^{-1} *(molar absorptivity at 510 nm)*

2 Determination of photon flux of 40 W blue LED light

$$\text{photon flux} = \left[\frac{\text{mol of Fe}^{2+}}{\phi \times t \times f} \right]$$

$$\text{photon flux} = \left[\frac{1.16 \times 10^{-7}}{1.01 \times 5.00 \times 0.81186} \right] \text{einstein s}^{-1}$$

$$= 2.83 \times 10^{-8}\ \text{einstein s}^{-1}$$

ϕ = 1.01 *(quantum yield of ferrioxalate actiometer)*
t = 5.00s *(time)*
f = 0.81186 *(Fraction of light absorbed)*

Afterward, the absorbance of both solutions was measured at 510 nm and with mol of Fe^{2+} known. Next the photon flux was determined to be 2.83 × 10^{-8} einstein s^{-1}. We can obtain the quantum yield of our reaction provided if it is irradiated using the same geometry.

Determination of Quantum Yield of 1,3-Hydroamination reaction:

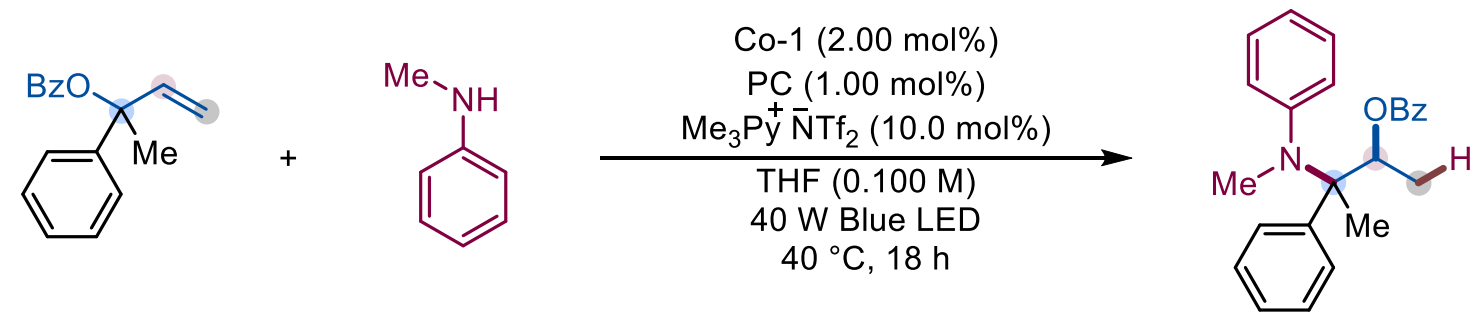

1a 0.0500 mmol | **2a** 1.50 equiv. | **3a**

The procedure is based on General Procedure B: In a glovebox, to an oven-dried 4 mL screw cap vial was added **Co-1** (1.10 mg, 2.00 μmol, 2.00 mol%), Ir(ppy)$_3$ (0.66 mg 1.00 μmol, 1.00 mol%), 2,4,6-trimethylpyridin-1-ium bis((trifluoromethyl)sulfonyl)amide (4.02 mg, 10.0 μmol, 10.0 mol%), **1a** (25.2 mg, 0.100 mmol, 1.00 equiv.), **2a** (16.07 mg, 0.150 mmol, 1.50 equiv.) and THF (1.00 mL, 0.100 M). The vial was equipped with a stir bar, capped and sealed with electrical tape, then taken out of the glovebox. The reaction mixture was stirred at 40 °C. Three batches of 100 μL of reaction mixture were removed after 30 minutes, and the crude ^{1}H NMR yield was obtained using dibromomethane as an internal standard. The yields were determined to be 13.1%, 13.4%, and 13.2%.

1 Quantum Yield

$$\phi = \left[\frac{\text{mol x yield \%}}{\text{photon flux x t x f}} \right]$$

$$\phi = \left[\frac{0.0000100 \times 13.2\%}{2.83 \times 10^{-8} \times 1800 \times 0.81186} \right]$$

$$= 0.032$$

ϕ = quantum yield of the reaction
t = 1800s (time)
f = (Fraction of light absorbed)
yield% = averaged yield of three trials

Cobalt-NR$_2$ complex detection:

In a glovebox, to an oven-dried 4 mL screw cap vial was added **Co-1** (13.8 mg, 25.0 μmol, 0.500 equiv), Ir(ppy)$_3$ (16.5 mg, 25.0 μmol, 0.500 equiv.), 2,4,6-trimethylpyridin-1-ium bis((trifluoromethyl)sulfonyl)amide (20.0 mg, 50.0 μmol, 1.00 equiv.), **1a** (12.6 mg, 0.0500 mmol, 1.00 equiv.), **2a** (8.04 mg, 0.0750 mmol, 1.50 equiv) and THF (0.500 mL, 0.100 M). The vial was equipped with a stir bar, capped and sealed with electrical tape, then taken out of the glovebox. The reaction mixture was stirred at 40 °C and irradiated with 40 W Blue LED for 30 minutes. 5 μL of reaction mixture was removed, diluted with MeCN, and injected into HRMS. **HRMS** (ESI-TOF) *m/z* calcd for $C_{39}H_{53}CoN_3O_2$ [(M - H)$^-$] 654.3475 (100%), 655.3508 (42.2%), 656.3542 (8.7%) found 654.3468 (100%), 655.3497 (36.6%), 656.3531 (8.0%).

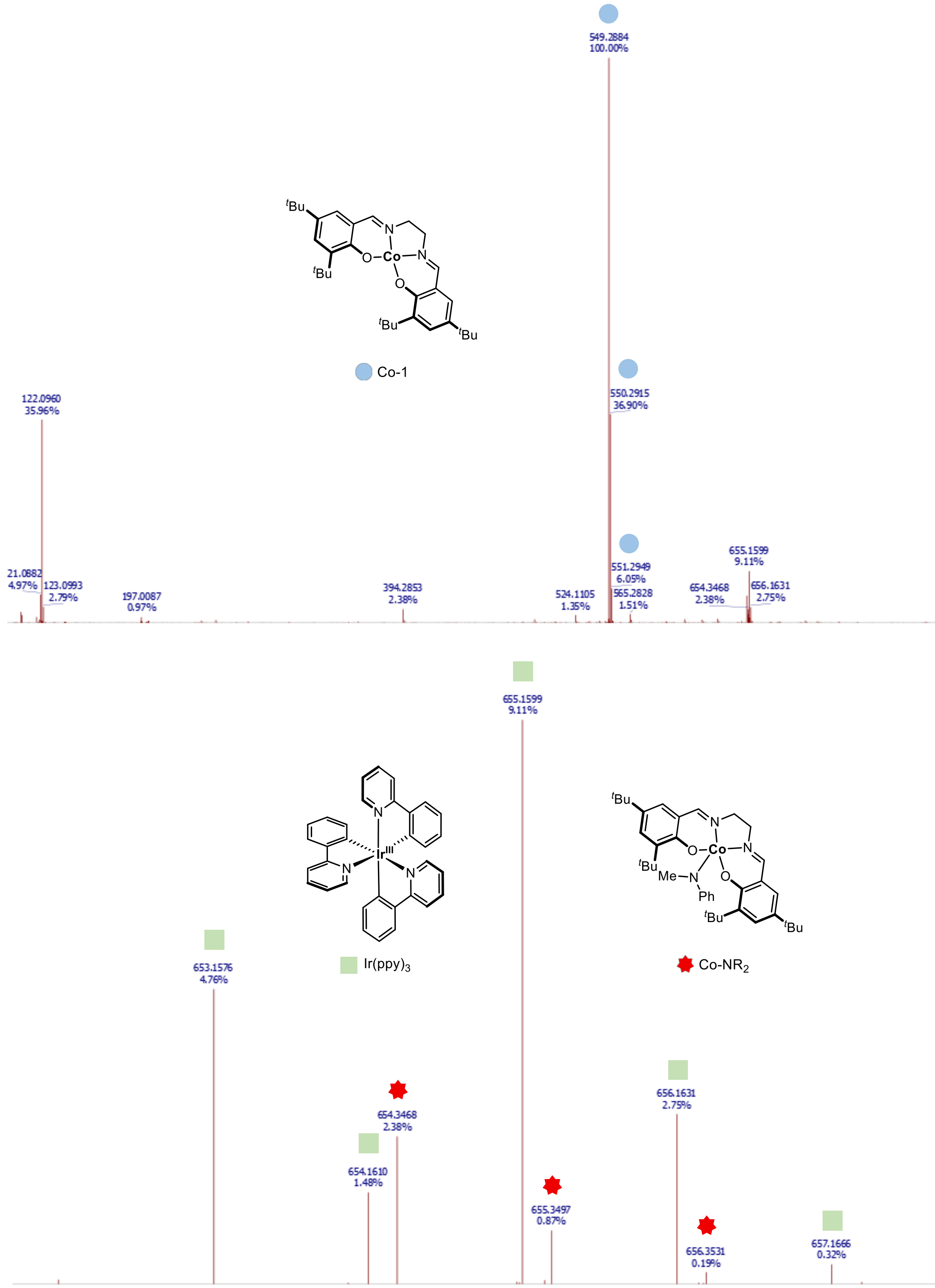

Figure S22. HRMS detection of Co-NR_2 intermediate

Crystal Data

For **3aag**: White needle-shaped crystals were grown under slow evaporation of dichloromethane at room temperature. A suitable crystal was selected and mounted on a Bruker AXS D8 Quest four-circle diffractometer equipped with I-mu-S 3.0 microsource X-ray tube, HELIOS multilayer Montel optics, and PhotonIII_C14 charge-integrating and photon counting detector. A total of 3689 reflection data were collected by using Mo Kα (λ = 0.71073) radiation while the crystal sample was cooled at 150 (2) K during the data collection. Reflections were merged by SHELXL according to the crystal class for the calculation of statistics and refinement. The molecular structure and crystal data of **3aag** are shown in **(**Figure S23**)**.

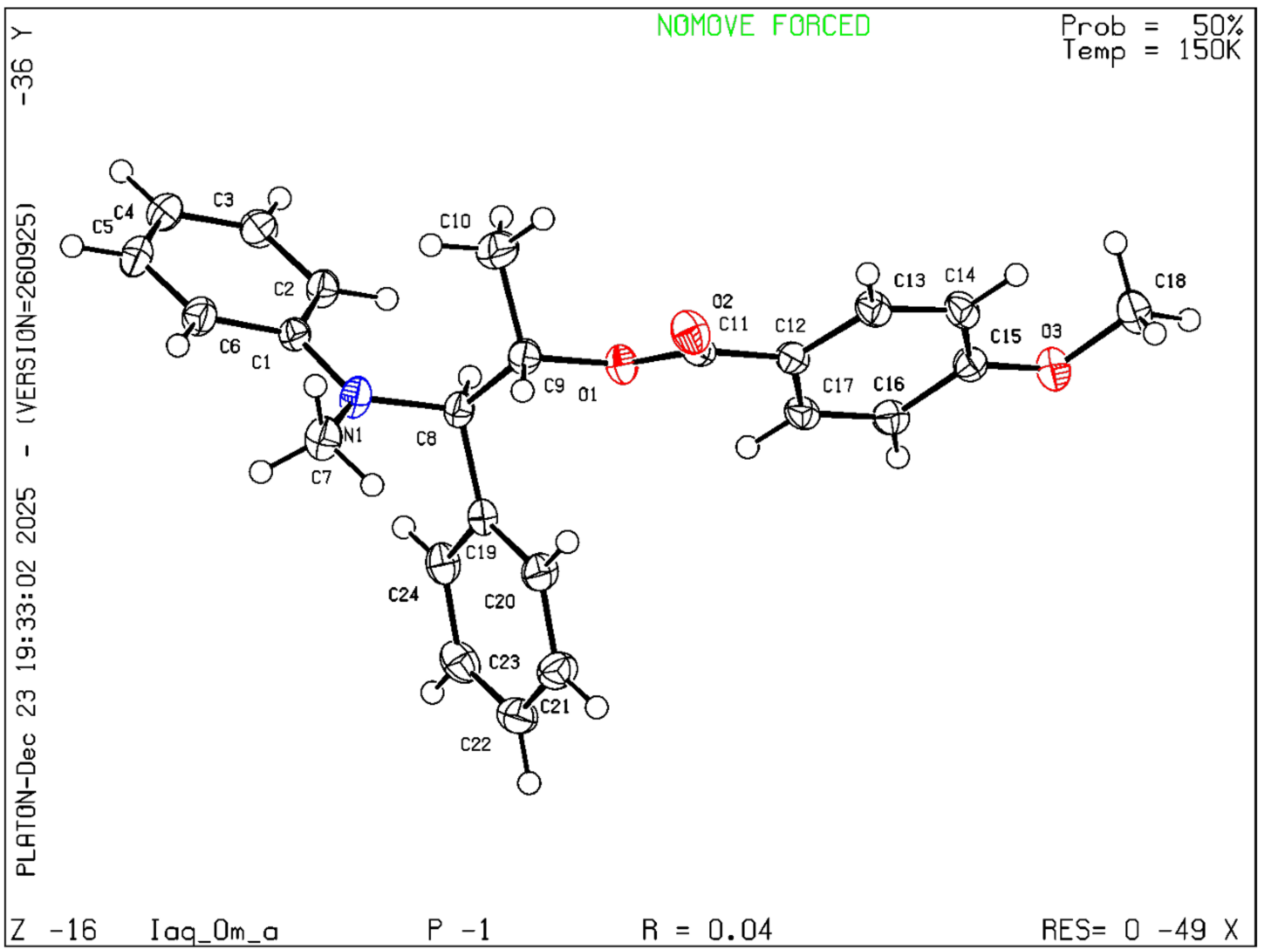

Figure S23. Molecular Structure of **3aag**. (CCDC 2518810)

Identification code	Iaq_0m_a
Empirical formula	$C_{24}H_{25}NO_3$
Formula weight	375.45
Temperature/K	150 K
Crystal system	triclinic
Space group	P -1
a/Å	9.8045(5)
b/Å	9.8060(4)
c/Å	10.9504(6)
α/°	85.201(2)
β/°	71.692(2)
γ/°	79.962(2)
Volume/Å^3	983.74(8)
Z	2
ρcalcg/cm^3	1.268
μ/mm^{-1}	0.083
F(000)	400.0
Radiation	Mo Kα (λ = 0.71073)
Diffrn_reflns_theta	θ_{min} 2.110, θ_{max} 28.366, θ_{full} 25.242
Index ranges	-13 ≤ h ≤ 13, -13 ≤ k ≤ 13, -14 ≤ l ≤ 14
Reflns_number_total	4900
Reflns_number_gt	4388
Reflns_threshold_expression	I > 2\s(I)

Table S7. Crystal Data and Structure Refinement for **3aag**.

DFT Calculations

Computational Details

All density functional theory (DFT) calculations were carried out using the Gaussian 16 program.[6] Geometries of intermediates and transition states were optimized in the gas phase using the dispersion-corrected B3LYP[7]-D3[8] functional with a mixed basis set of SDD[9] (*i.e.*, the Stuttgart/Dresden effective core potentials and the associated valence electron basis set) for Co and Ir, and 6-31G(d) for other atoms. Vibrational frequency calculations were performed to confirm whether each structure is a local minimum or a transition state. Thermal corrections to Gibbs free energies were calculated at 298 K. Single-point energy calculations were carried out using the M06 functional[10] with the def2-TZVP basis set[11] for all atoms. Solvation energy corrections were calculated in single point energy calculations using the SMD solvation model[12] in 1,4-dioxane. CYLView[13] was used to generate all 3D structures.

Spin state of Co species

The spin states of Co species used in our DFT calculations are summarized in Figure S1. The spin states of $[Co^{III}]$–H, $[Co^{II}]$, and $[Co^{III}]^+$ were taken from our recent computational study.[14] The spin state energies of $[Co^{III}]$–NR_2 and **TS4** (ΔG) are provided with respect to the lowest-energy spin state of each species. **TS4** was optimized in the quartet spin state, and single-point calculations were performed in alternative spin states to evaluate their relative stability. The geometry of $[Co^{III}]$–NR_2 was optimized at the same spin state as the single point energy calculations.

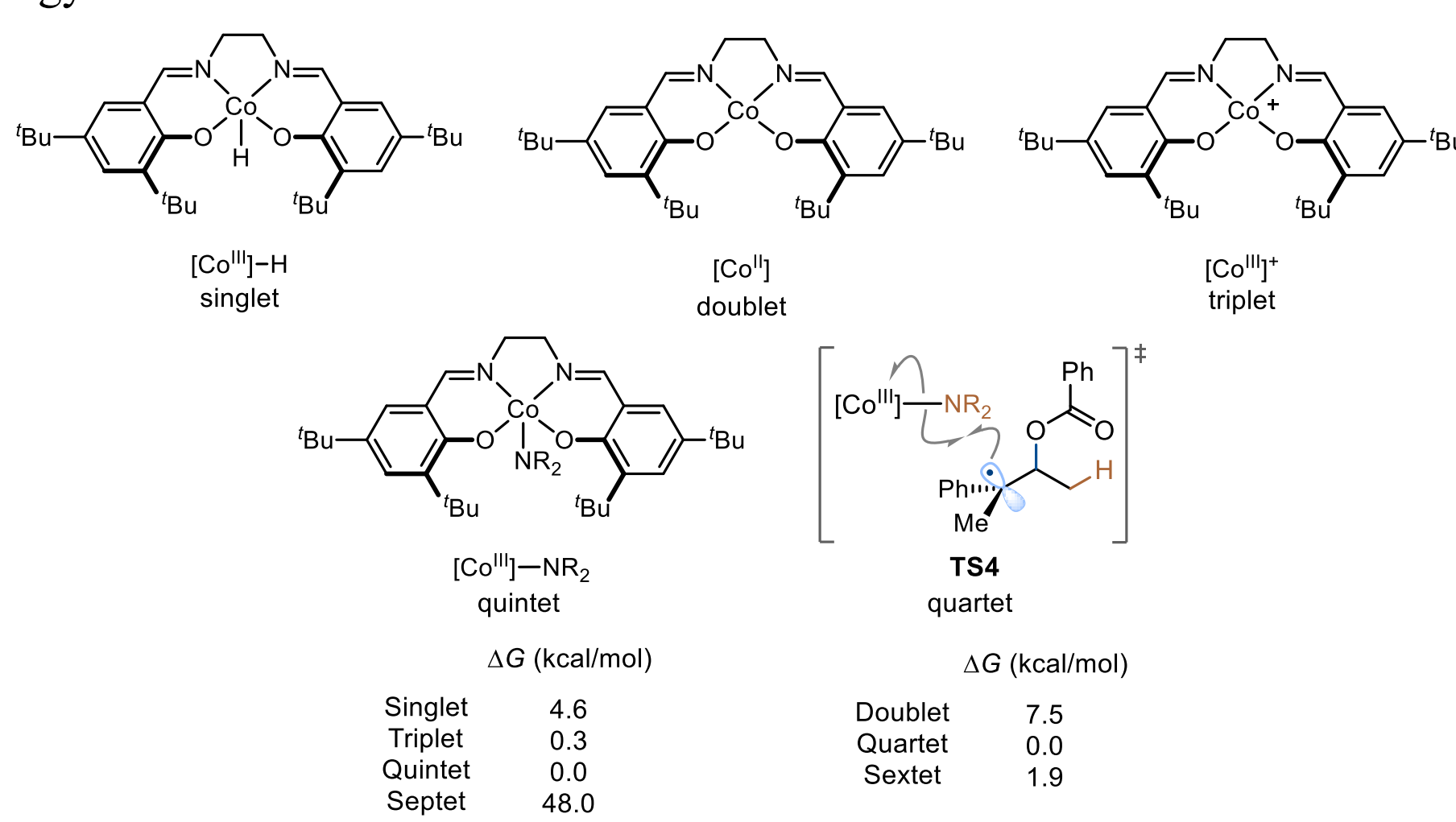

Figure S24. Spin states of $[Co^{III}]$–H, $[Co^{II}]$, $[Co^{III}]^+$, $[Co^{III}]$–NR_2, and **TS4.**

Calculated energies of intermediates and transition states

Table S8. Calculated total (SCF) energies, enthalpies, and Gibbs free energies of intermediates and transition states.

	Level of theory used to compute the reaction energy profile: M06/def2-TZVP/SMD(THF)//B3LYP-D3/SDD-6-31G(d)			
	SCF energy (a.u.)	Enthalpy (a.u.)	Gibbs free energy (a.u)	Imaginary Frequency (cm^{-1})
**1a**	-807.6080	-807.3040	-807.3672	-
**III**	-808.1787	-807.8649	-807.9312	-
**IV**	-808.2024	-807.8868	-807.9526	-
**V**	-3697.5731	-3696.4858	-3696.6312	-
**VI**	-3697.3621	-	-	-
**TS1**	-3697.5370	-3696.4590	-3696.6082	1611.3i
**TS2**	-808.1608	-807.8480	-807.9141	109.8i
**Co-III-H-singlet**	-2889.9320	-2889.1566	-2889.2635	-
**Co-III-triplet**	-2889.1850	-2888.4176	-2888.5256	-
**Co-II-doublet**	-2889.3679	-2888.6015	-2888.7089	-
**Co-III-NR_2-singlet**	-3215.5182	-3214.6076	-3214.7304	-
**Co-III-NR_2-triplet**	-3215.5200	-3214.6110	-3214.7373	-
**Co-III-NR2-quintet**	-3215.5172	-3214.6095	-3214.7377	-
**Co-III-NR_2-septet**	-3215.4382	-3214.5318	-3214.6611	-
**$Ir(ppy)_3$**	-1540.1272	-1539.6159	-1539.7061	-
**$[Ir(ppy)_3]^+$**	-1539.9376	-1539.4253	-1539.5173	-
**X-1**	-366.0945	-365.9120	-365.9591	-
**2a**	-326.7830	-326.6289	-326.6683	-
**3a**	-1134.4117	-1133.9494	-1134.0279	-
**3a'**	-1134.4160	-1133.9539	-1134.0325	-
**TS4**	-4023.7370	-4022.5102	-4022.6748	108.9i
**HX-1**	-2194.0282	-2193.7611	-2193.8455	-
**NTf_2^-**	-1827.4551	-1827.3863	-1827.4442	-
	Level of theory used for the 2,3- and 1,2-radical migration transition state calculations:			

	M06-2X/6-311+G(d,p)/SMD(THF)//B3LYP-D3/SDD-6-31G(d)			
	SCF energy (a.u.)	Enthalpy (a.u.)	Gibbs free energy (a.u)	Imaginary Frequency (cm^{-1})
**III**	-808.3441428	-808.0303104	-808.0966204	-
**TS2**	-808.3201509	-808.0733754	-808.0073084	109.8i
**TS3**	-808.3200708	-808.0735012	-808.0069842	54.5i

Benchmark of the 1,2-radical acyloxy migration (RAM) selectivity

We evaluated the kinetic barriers of the competing 2,3- and 1,2-radical acyloxy migration pathways using different DFT methods and local coupled-cluster theory. Among the DFT methods tested, the relative Gibbs free energies ($\Delta\Delta G^{\ddagger}$) from M06-2X/def2-TZVP, M06-2X/6-311+G(d,p), and ωB97X-D/def2-TZVP are all within 1.5 kcal/mol from the experimentally derived $\Delta\Delta G^{\ddagger}$ value. However, only M06-2X/6-311+G(d,p) exactly reproduced the experimental selectivity that slightly favored the 2,3-migration (TS2). DLPNO-CCSD(T) calculations also favor the 2,3-RAM pathway over the 1,2 pathway, whereas the selectivity is overestimated compared with experiment. The M06 functional, which is recommended for metal-containing species, gave larger error compared with M06-2X and ωB97X-D for this non-metal system. Based on these benchmark studies, we reported the kinetic barriers of the 2,3- and 1,2-RAM pathways using M06-2X/6-311+G(d,p) (Table S 9) and used M06/def2-TZVP for the overall catalytic cycle of the Co/photoredox-catalyzed reaction.

Table S 9. Barriers to the 2,3- (TS2) and 1,2-radical migration (TS3) pathways computed at different levels of theories. All structures were optimized at the level of theory specified in the Computational Details section. Single-point energy calculations were performed using the SMD solvation model in tetrahydrofuran. All Gibbs free energies are in kcal/mol.

**Method**	**TS2 ($\Delta G^{\ddagger}$)**	**TS3 ($\Delta G^{\ddagger}$)**	**$\Delta\Delta G^{\ddagger}$ (TS2 – TS3)**
M06/def2-TZVP	10.7	8.8	1.9
M06-2X/def2-TZVP	14.7	14.6	0.1
M06-2X/6-311+G(d,p)	14.4	14.6	-0.2
ωB97X-D/def2-TZVP	12.0	11.1	0.9
DLPNO-CCSD(T)/NormalPNO/def2-TZVP	15.9	19.5	-3.6
DLPNO-CCSD(T)/TightPNO/def2-TZVP	14.9	17.7	-2.8
UKS-DLPNO-CCSD(T)/NormalPNO/def2-TZVP [a]	14.6	16.6	-2.0
Experimental selectivity[b]	-	-	-0.3

[a] M06 reference wavefunction was used.

[b] Derived from the product ratio (1.7:1) from the ^{18}O-labeling experiments.

Alternate Co-catalyzed pathways for hydroamination of alkenes

We considered other possible mechanisms for the alkene hydroamination involving the formation of a high-valent cobalt species followed by backside S_N2 attack. We sought to evaluate these two-electron pathways using density functional theory calculations. Attempts to freely optimize the high-valent Co^{IV}–alkyl species (**VI**) were unsuccessful. The C–Co bond in **VI** dissociates upon geometry optimization, indicating the association of the sterically congested tertiary benzylic radical **IV** to the $[Co^{III}]^+$ complex is unfavorable. Consequently, a constrained geometry optimization was performed on **VI** with the C–Co bond restricted (**Figure S25**). These estimates suggest that the formation of the Co^{IV}–alkyl species (VI) is electronically disfavored by 15.8 kcal/mol.

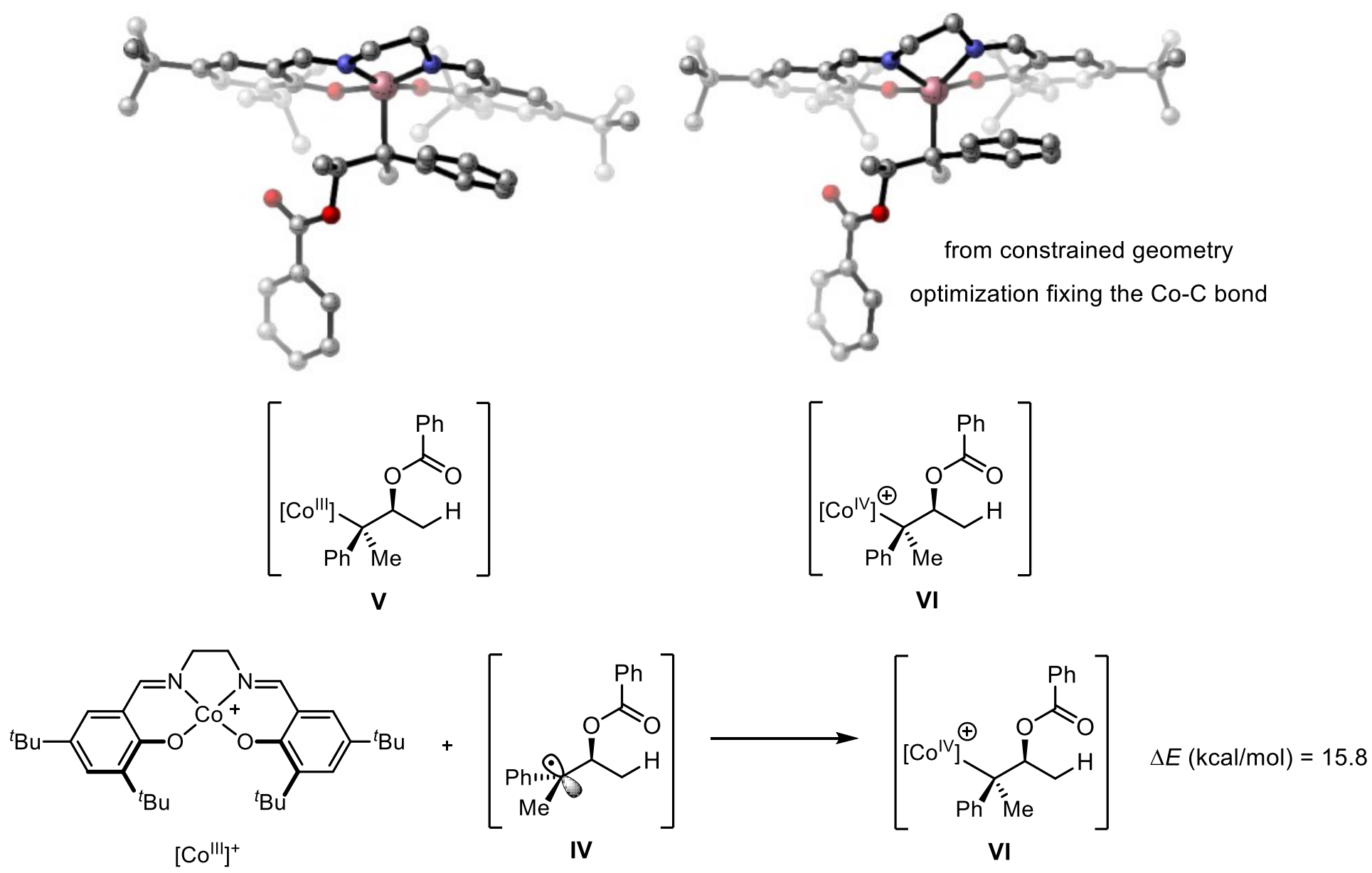

Figure S25. Computed electronic energy for the formation of the proposed Co^{IV}–alkyl intermediate (**IV**).

Spectroscopic Data

^{1}H NMR (400 MHz, $CDCl_3$, 25 ºC) of 1a

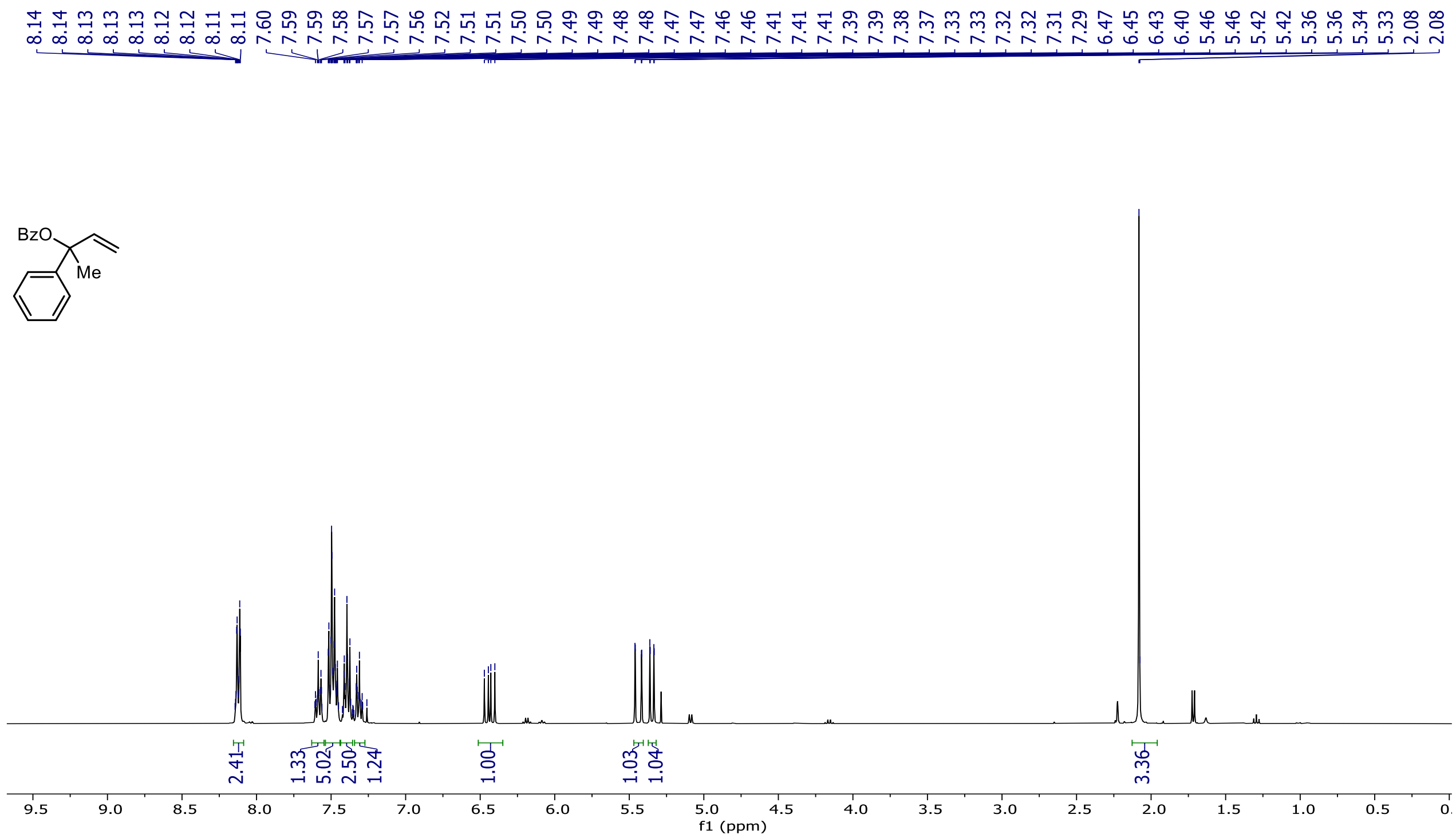

^{13}C NMR (101 MHz, $CDCl_3$, 25 ºC) of 1a

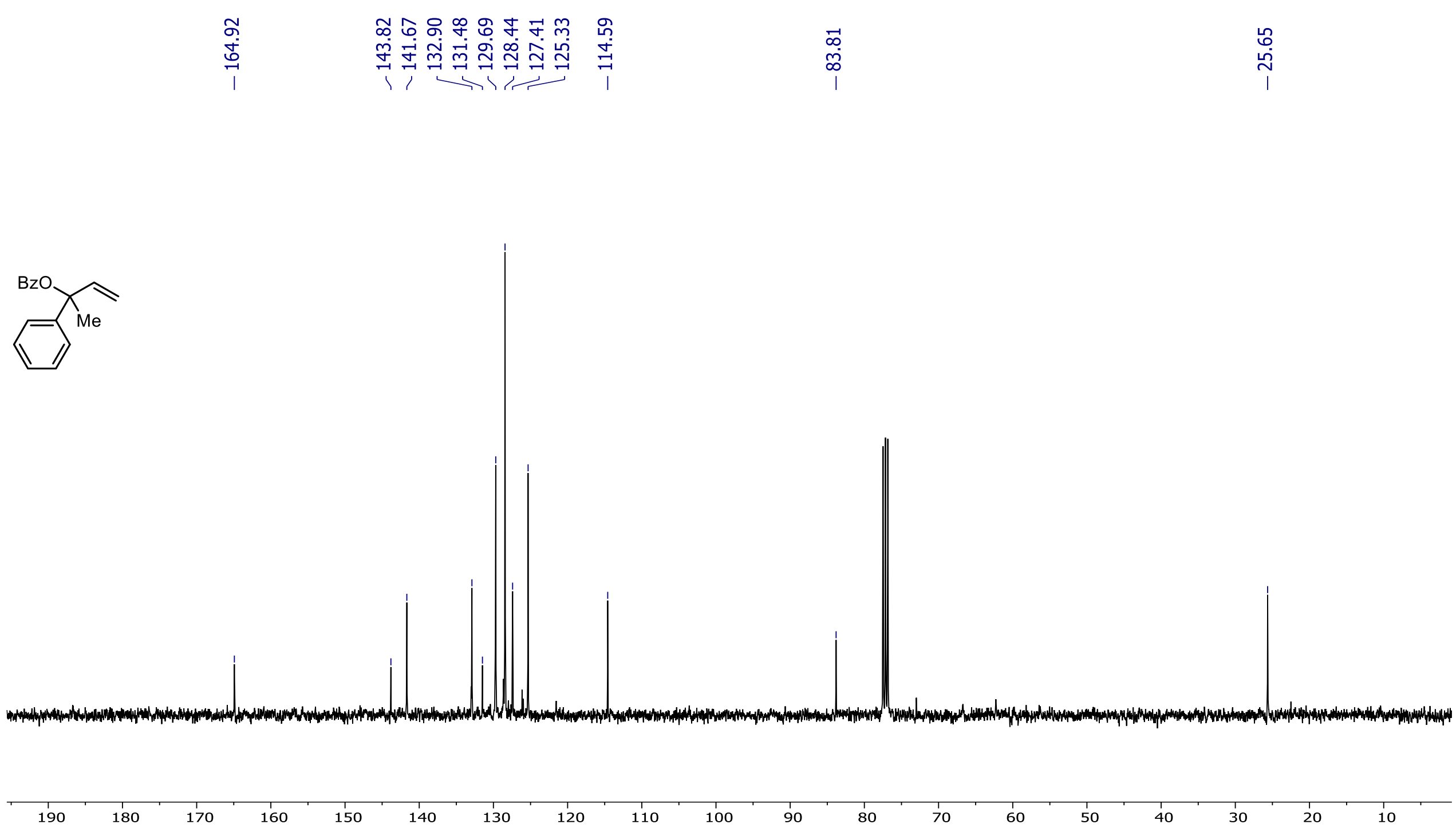

^{1}H NMR (400 MHz, $CDCl_3$, 25 ºC) of 1b

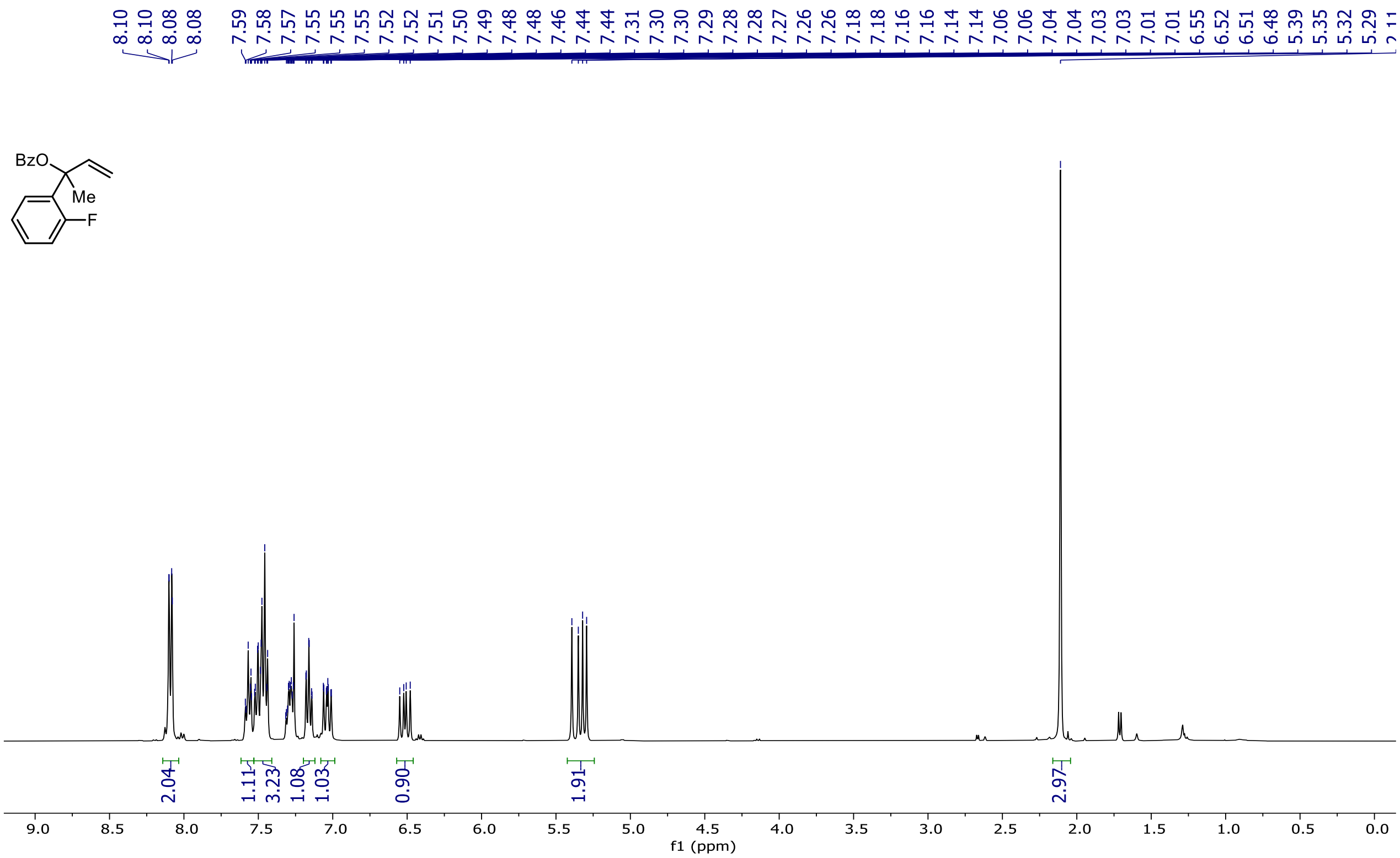

^{13}C NMR (101 MHz, $CDCl_3$, 25 ºC) of 1b

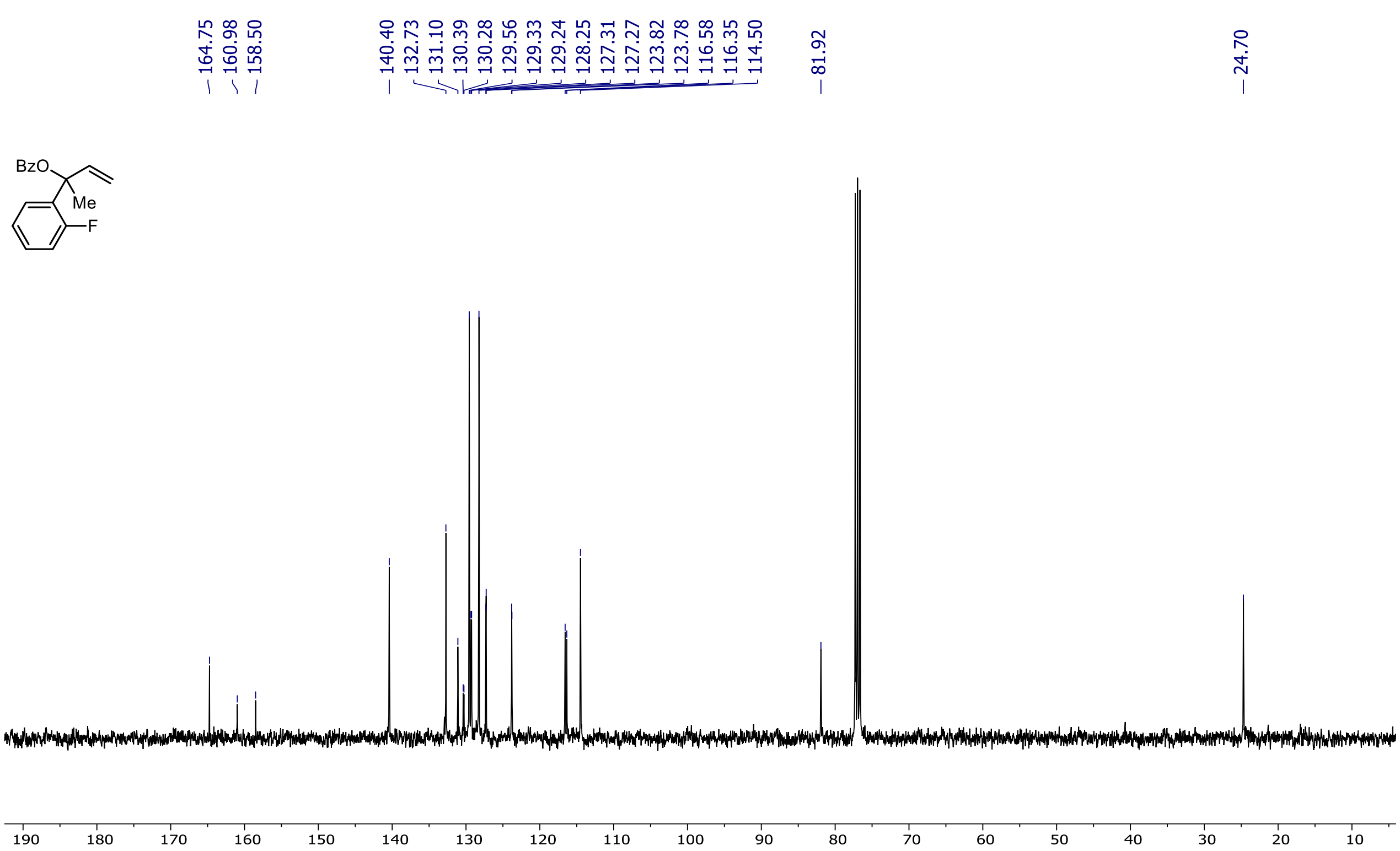

^{1}H NMR (400 MHz, CDCl$_3$, 25 ºC) of 1c

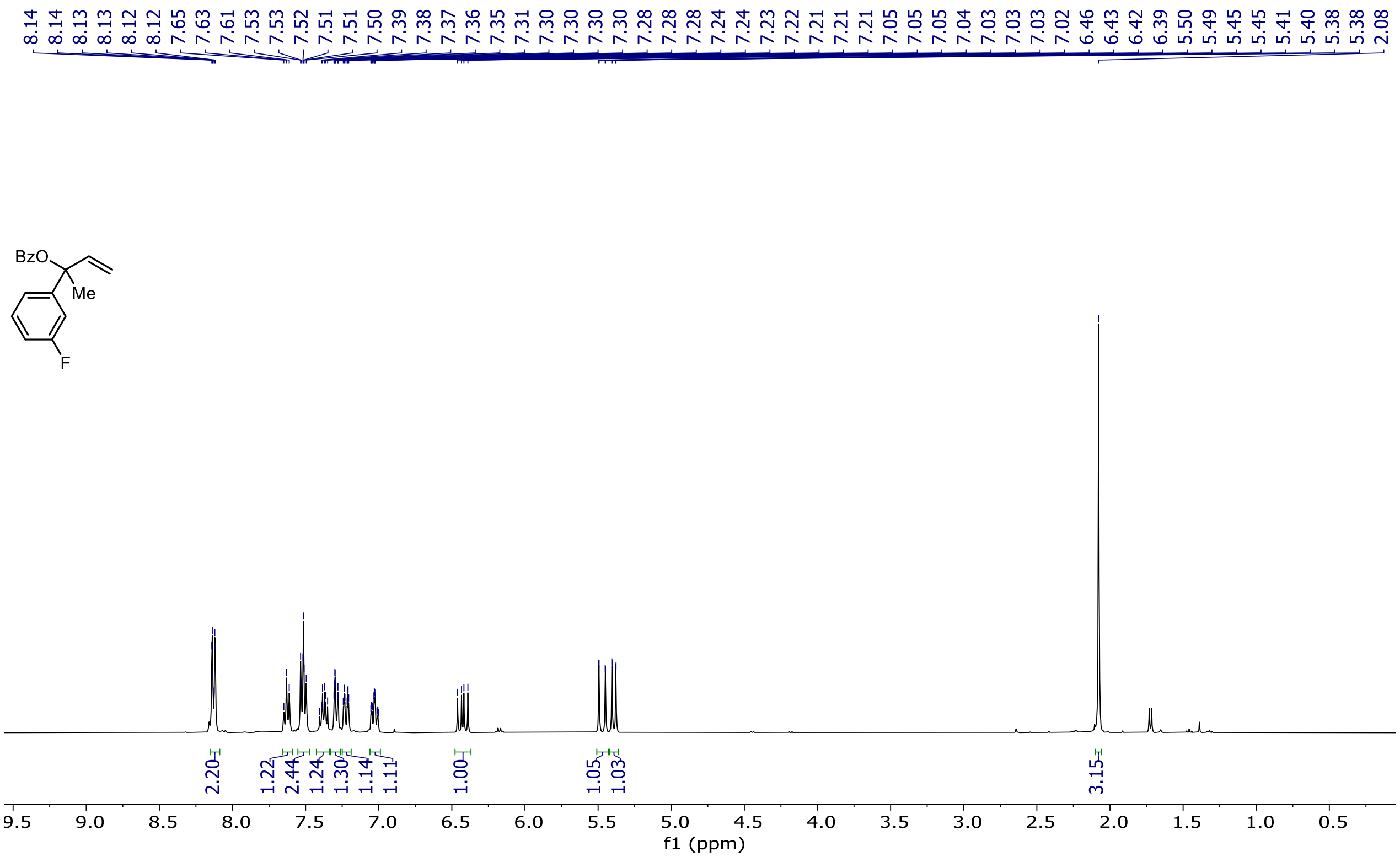

^{13}C NMR (101 MHz, CDCl$_3$, 25 ºC) of 1c

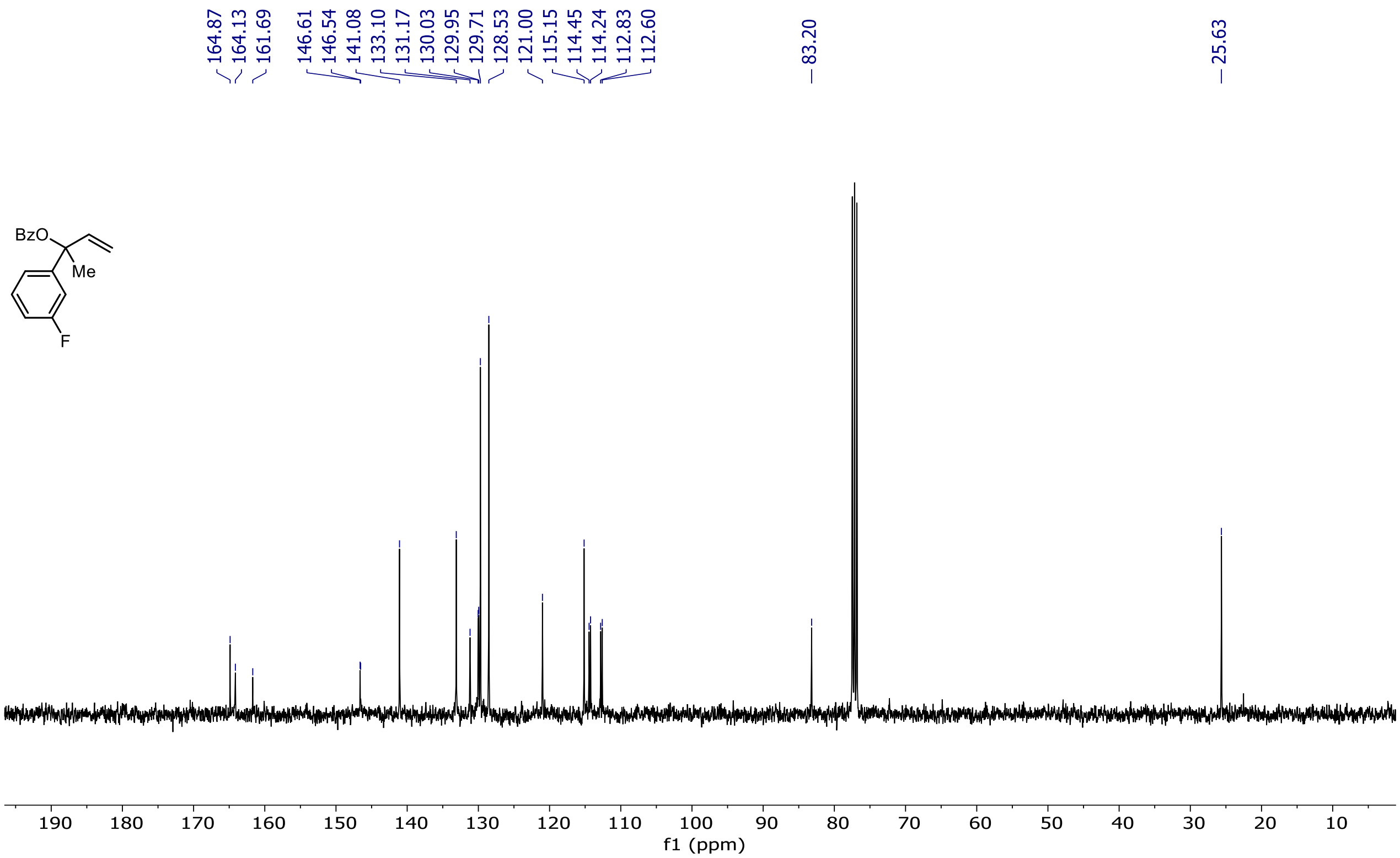

^{19}F NMR (282 MHz, $CDCl_3$, 25 ºC) of 1c

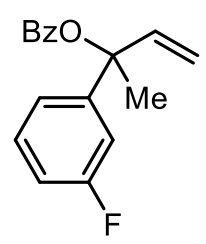

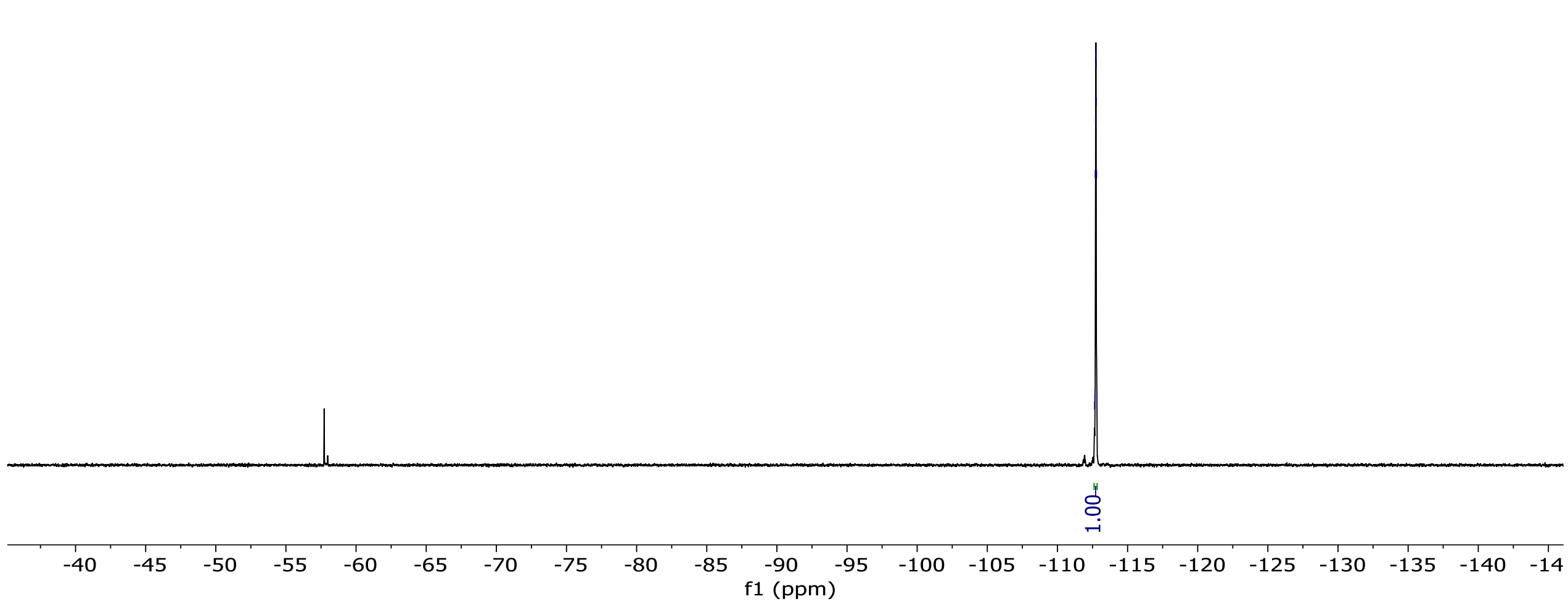

^{1}H NMR (400 MHz, $CDCl_3$, 25 ºC) of 1d

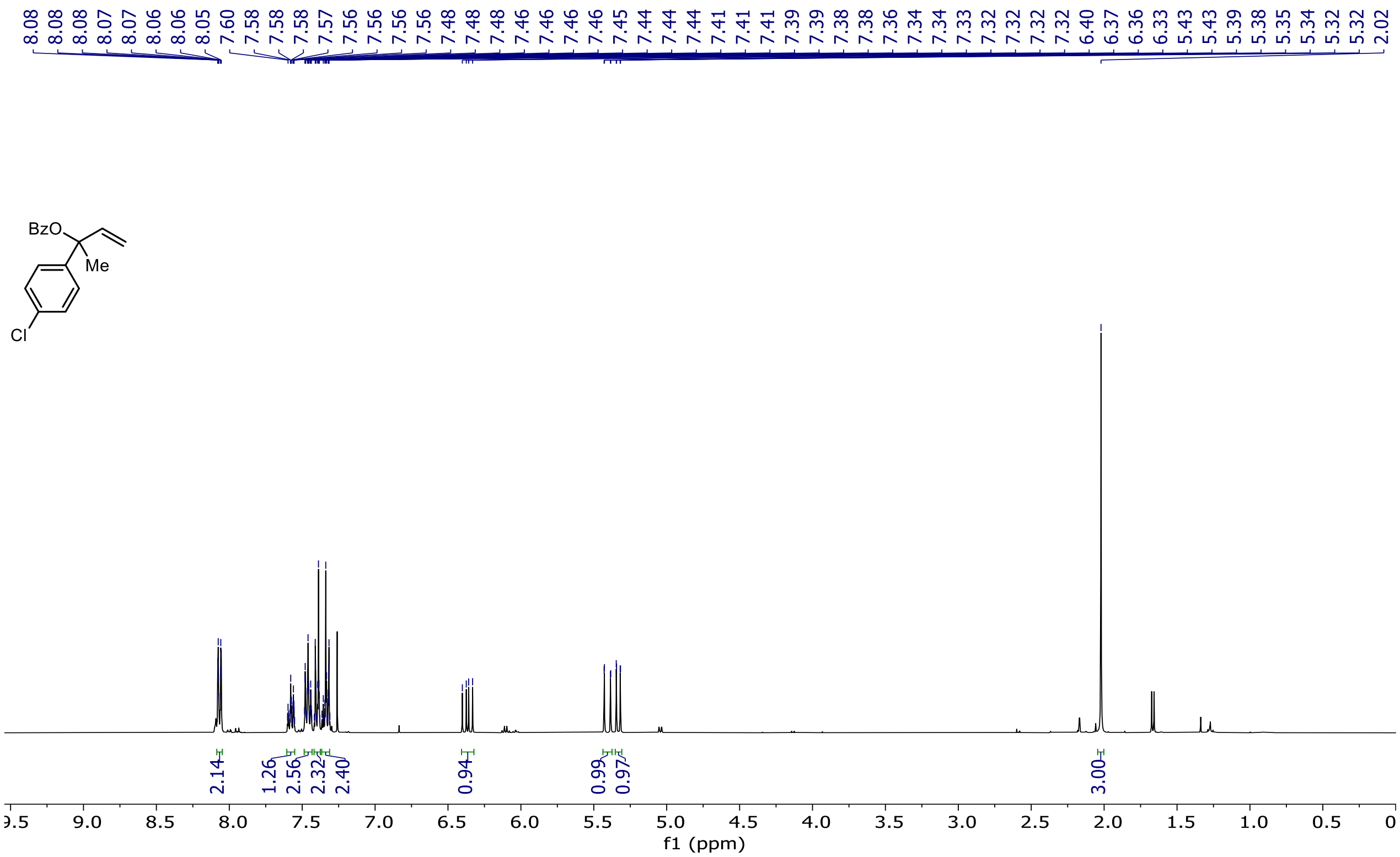

^{13}C NMR (101 MHz, $CDCl_3$, 25 ºC) of 1d

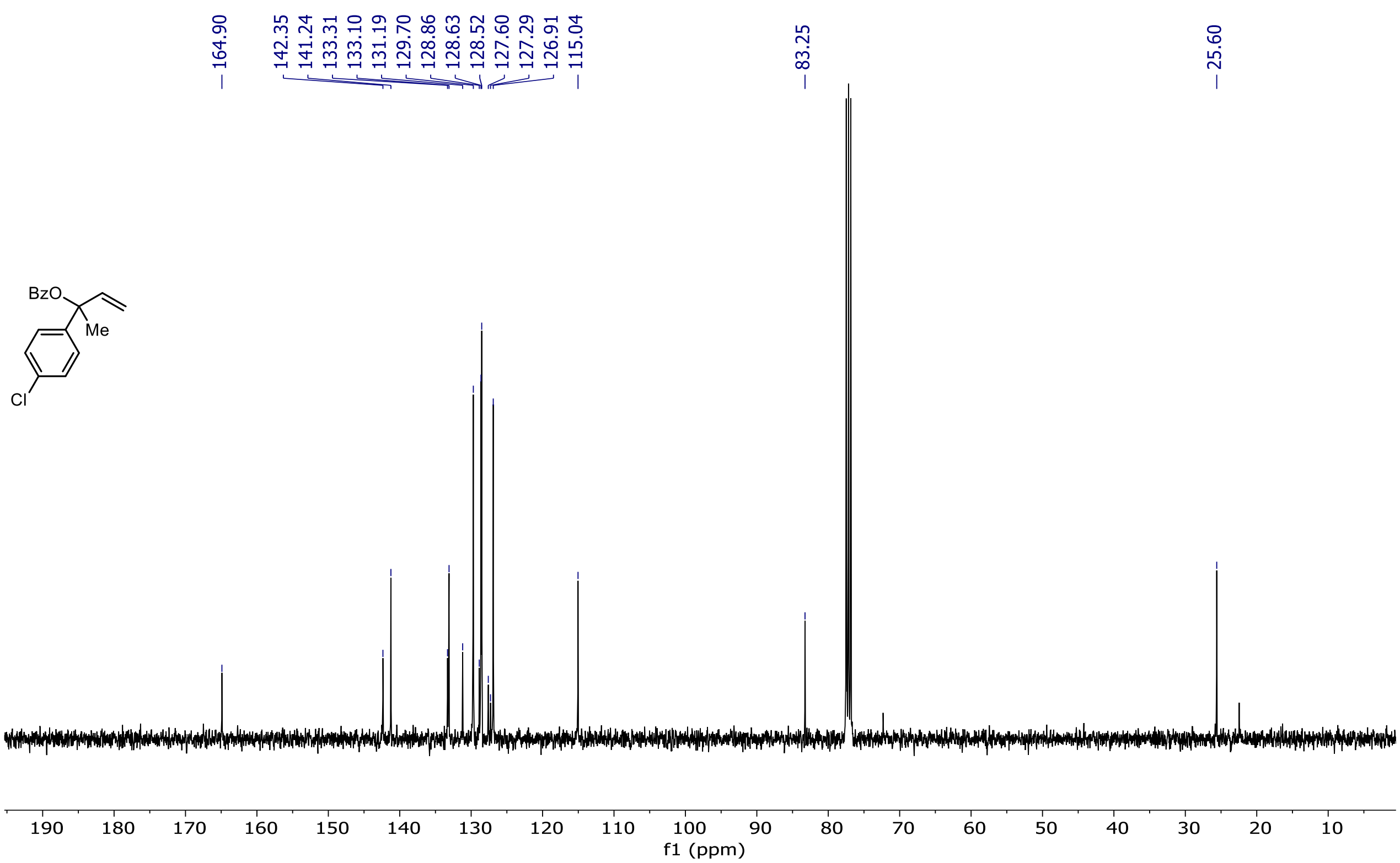

^{1}H NMR (400 MHz, CDCl$_3$, 25 ºC) of 1e

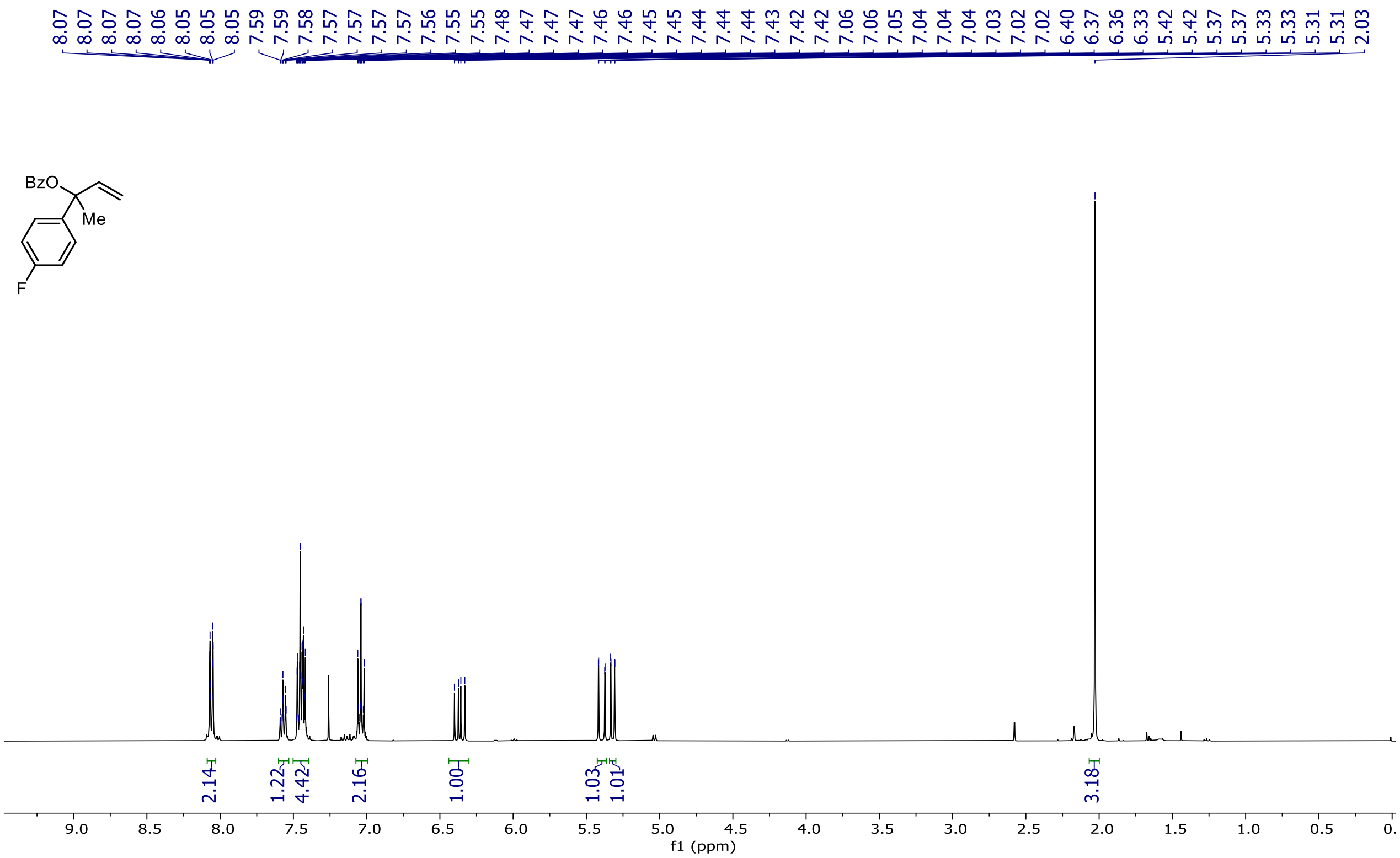

^{13}C NMR (101 MHz, CDCl$_3$, 25 ºC) of 1e

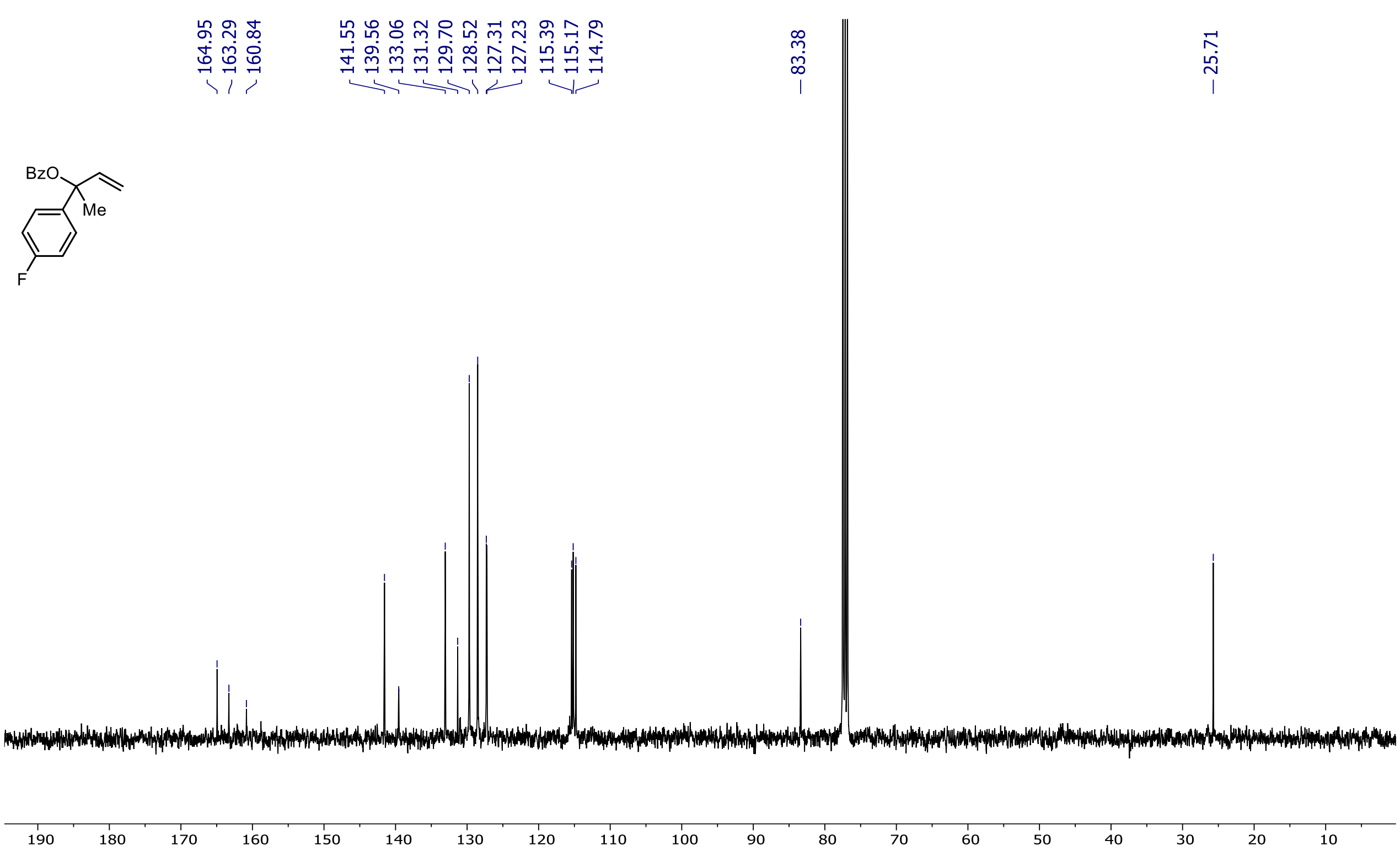

^{19}F NMR (282 MHz, $CDCl_3$, 25 °C) of 1e

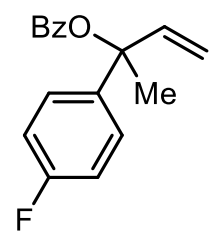

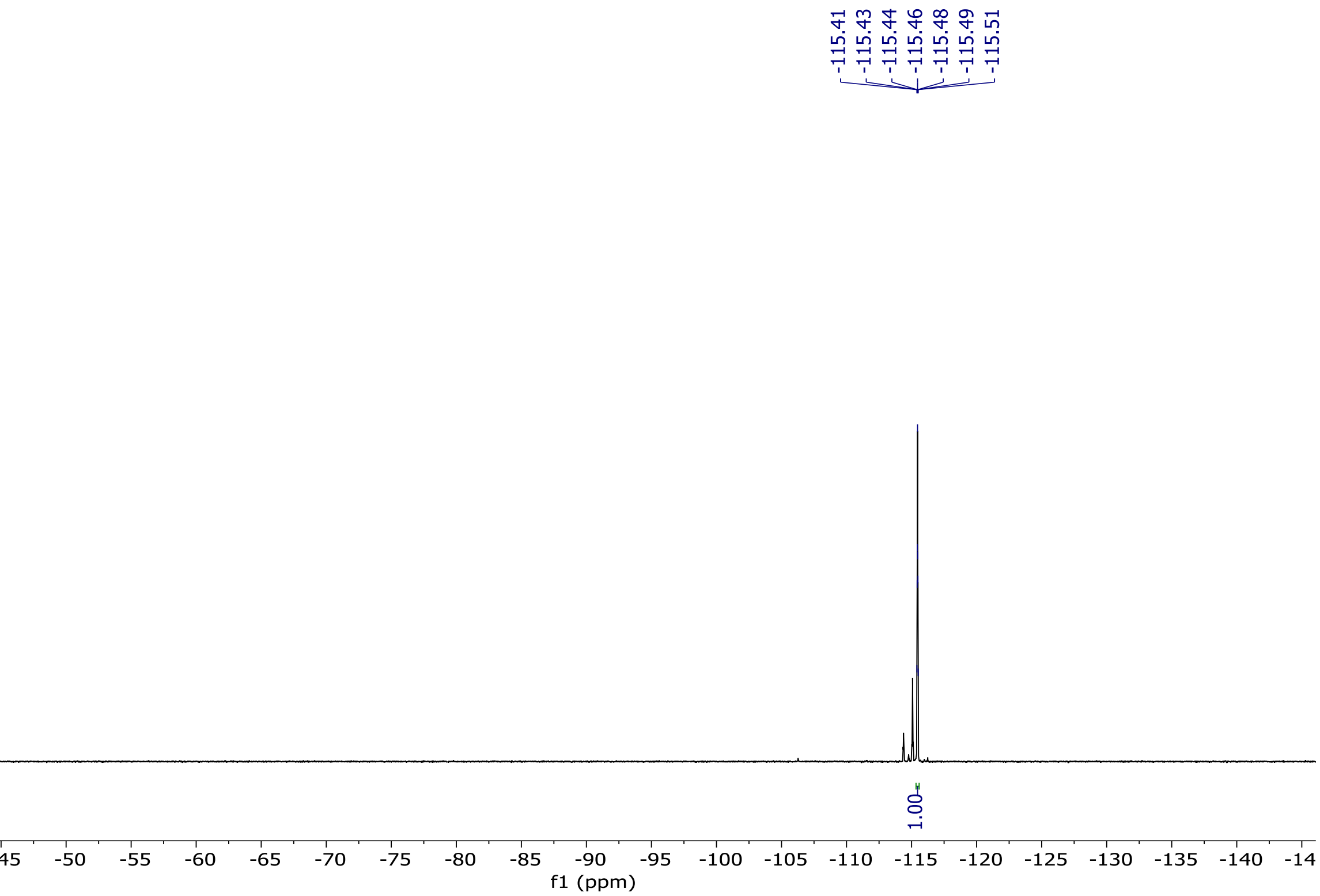

^{1}H NMR (400 MHz, CDCl$_3$, 25 ºC) of 1f

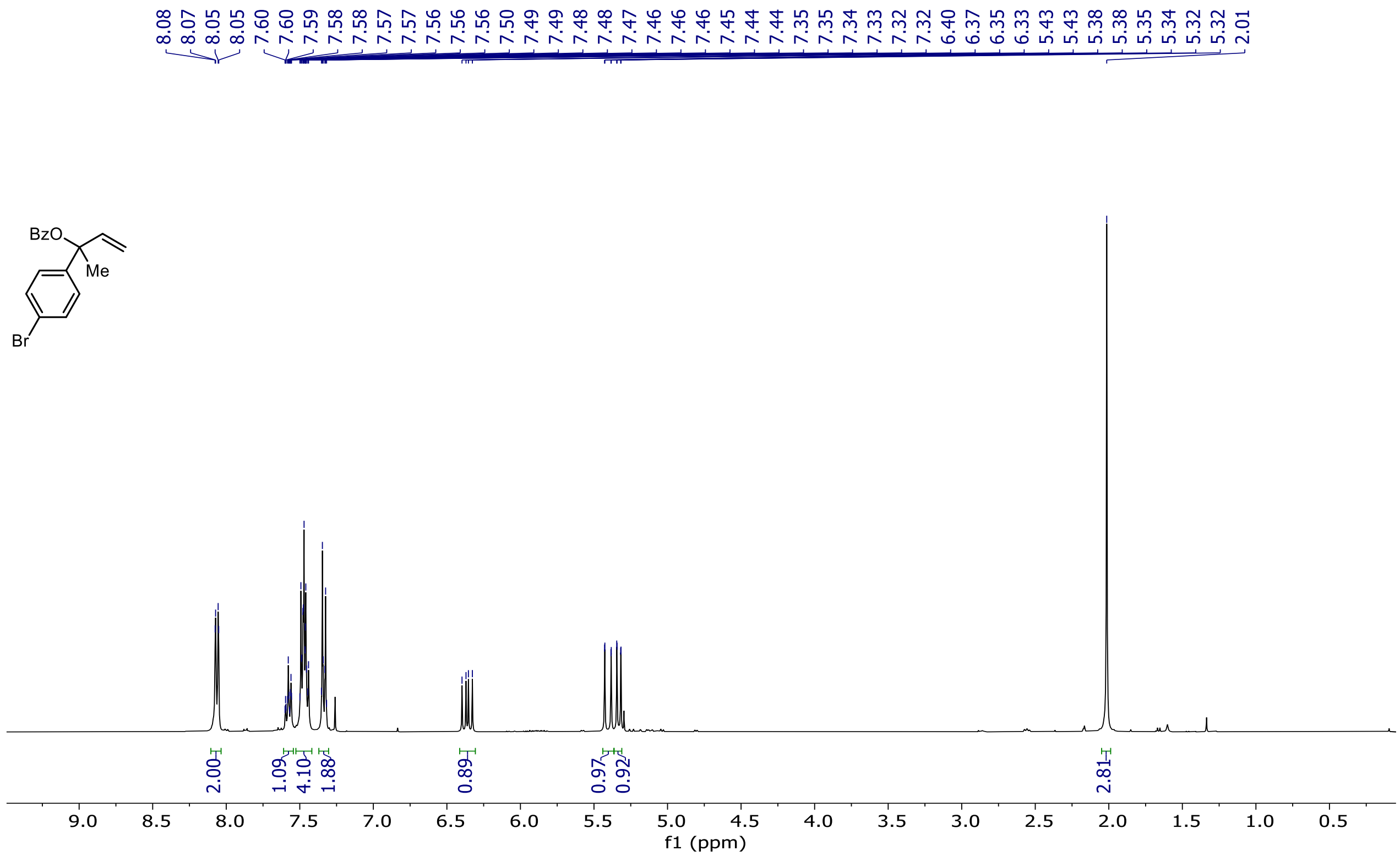

^{13}C NMR (101 MHz, CDCl$_3$, 25 ºC) of 1f

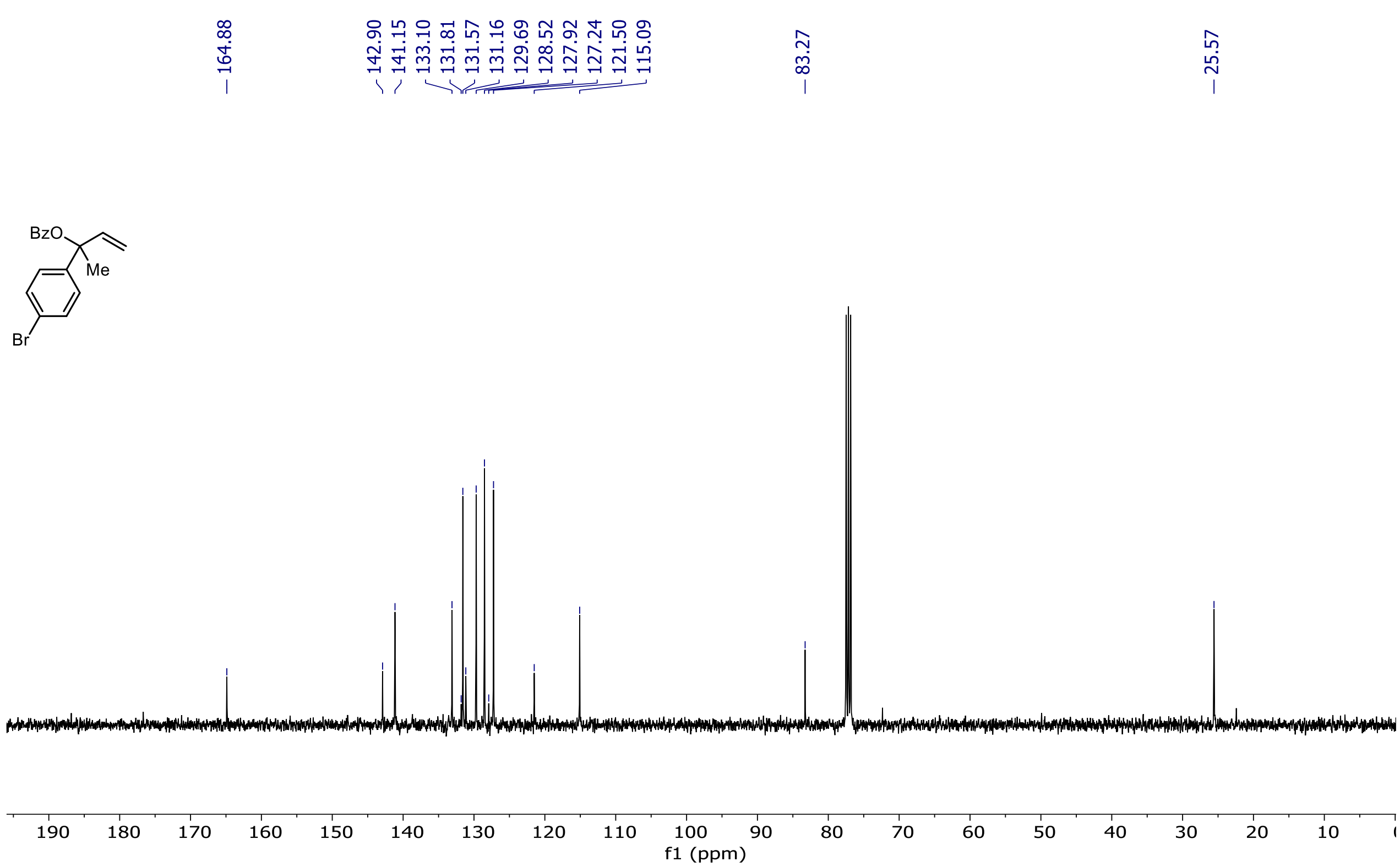

^{1}H NMR (400 MHz, CDCl$_3$, 25 ºC) of 1g

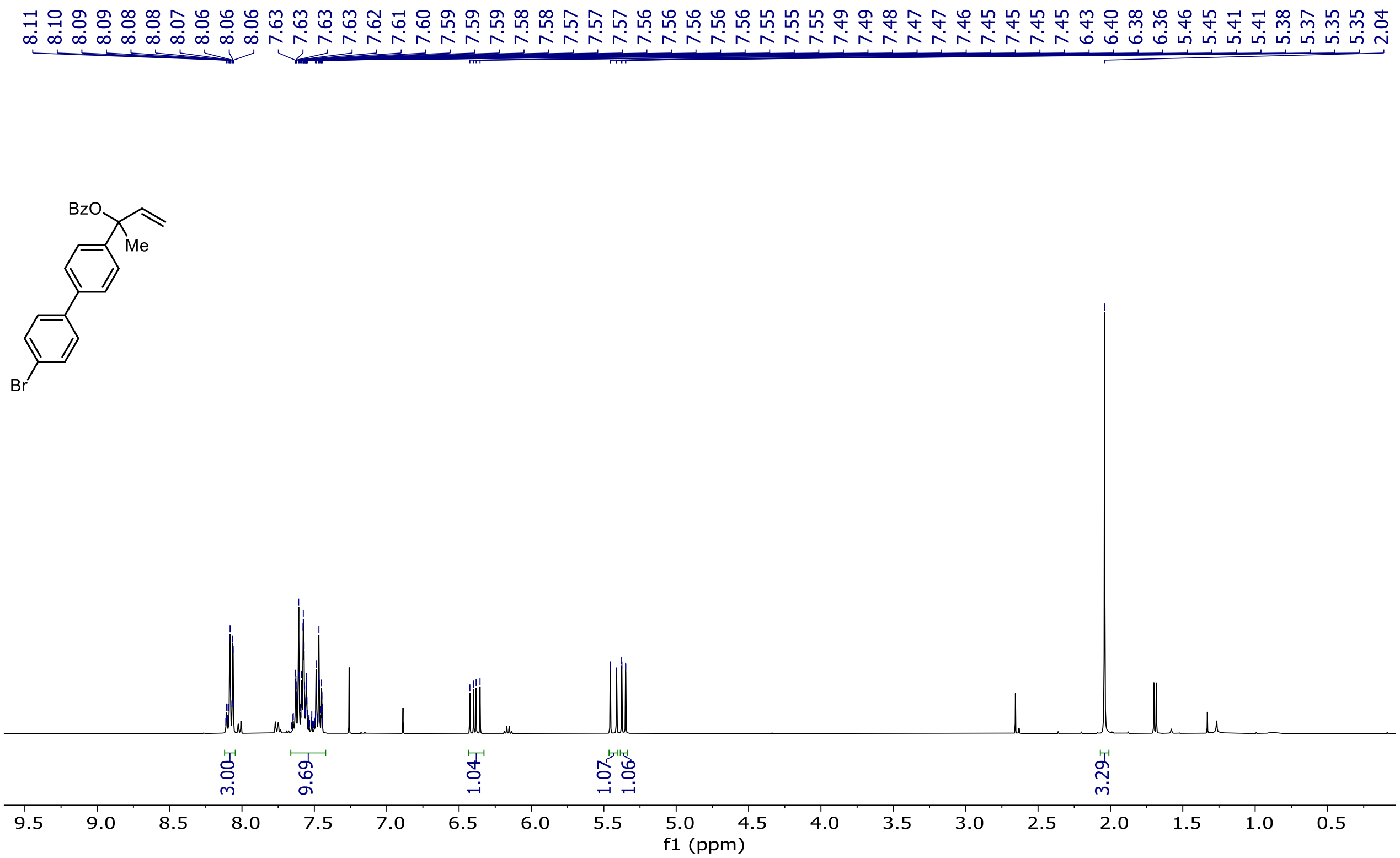

^{13}C NMR (101 MHz, CDCl$_3$, 25 ºC) of 1g

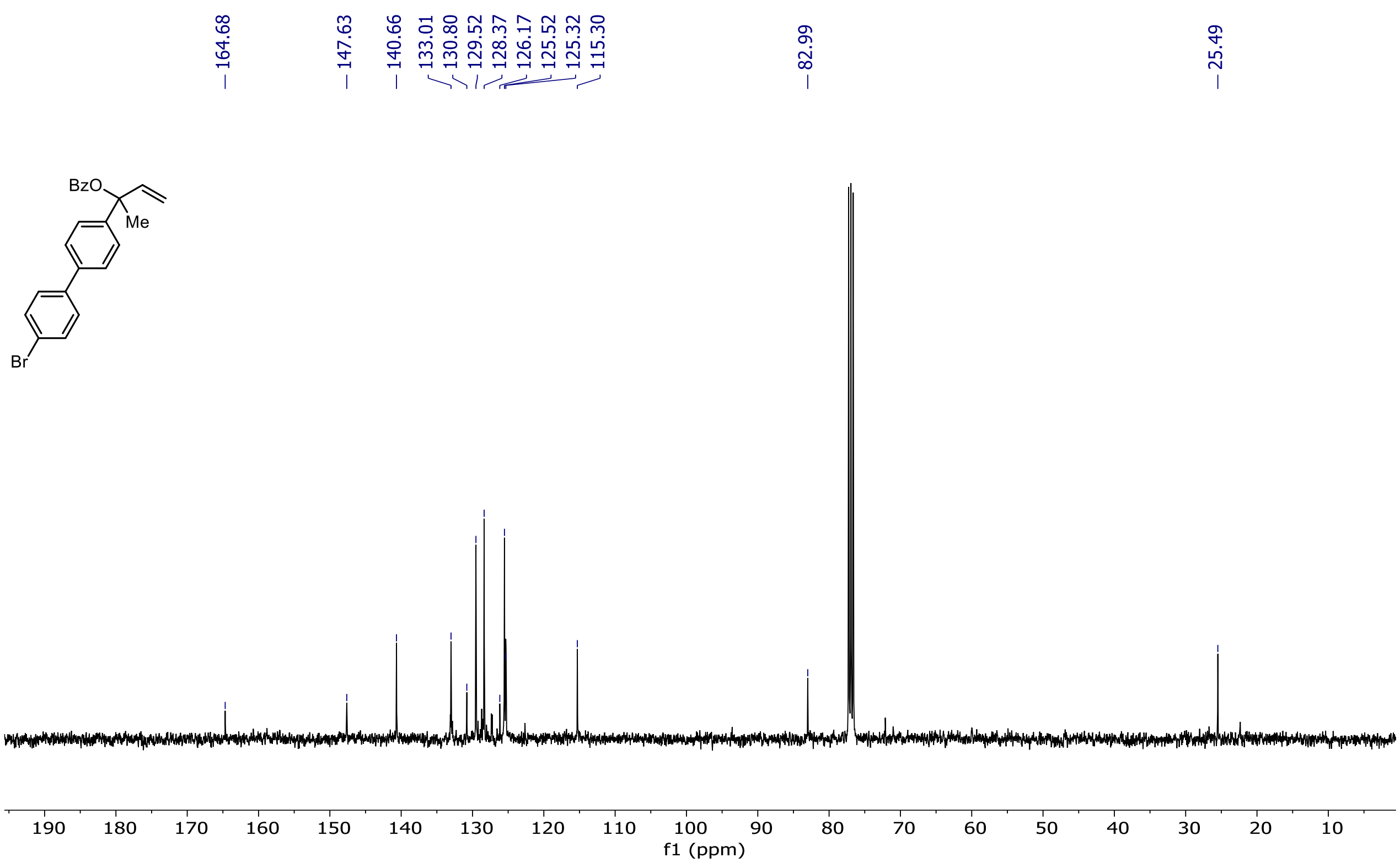

¹H NMR (400 MHz, $CDCl_3$, 25 ºC) of 1h

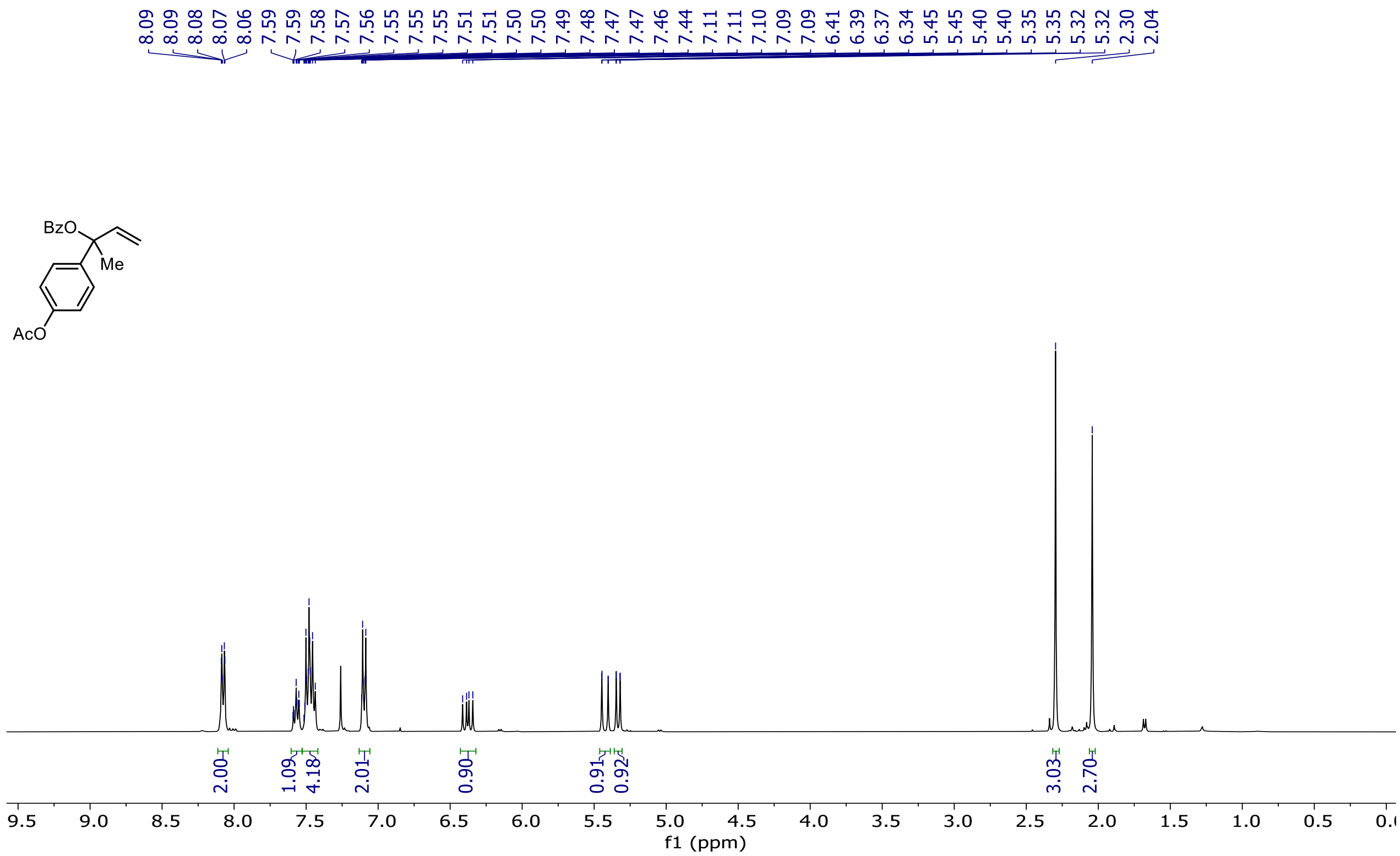

¹³C NMR (101 MHz, $CDCl_3$, 25 ºC) of 1h

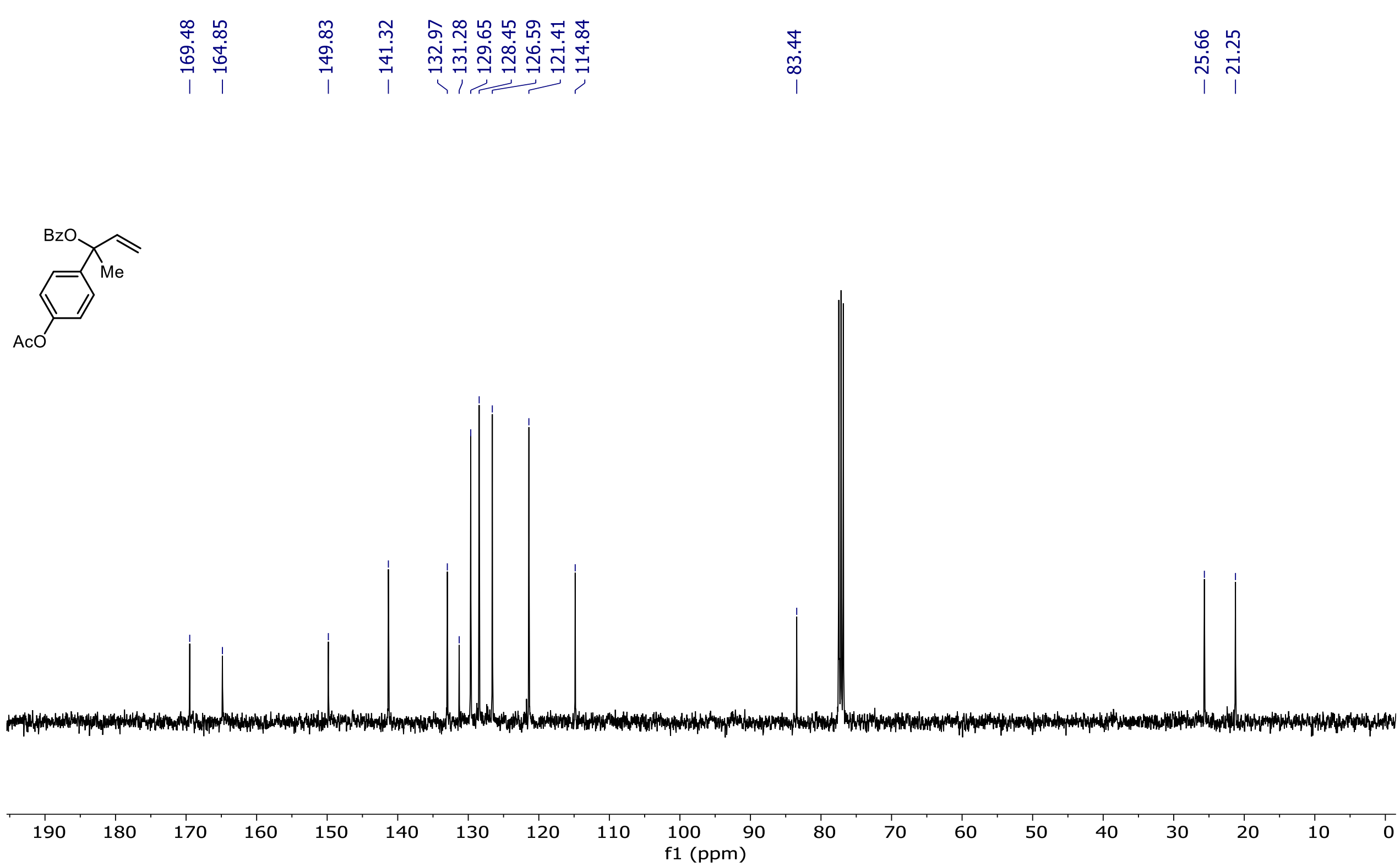

^{1}H NMR (400 MHz, $CDCl_3$, 25 ºC) of 1i

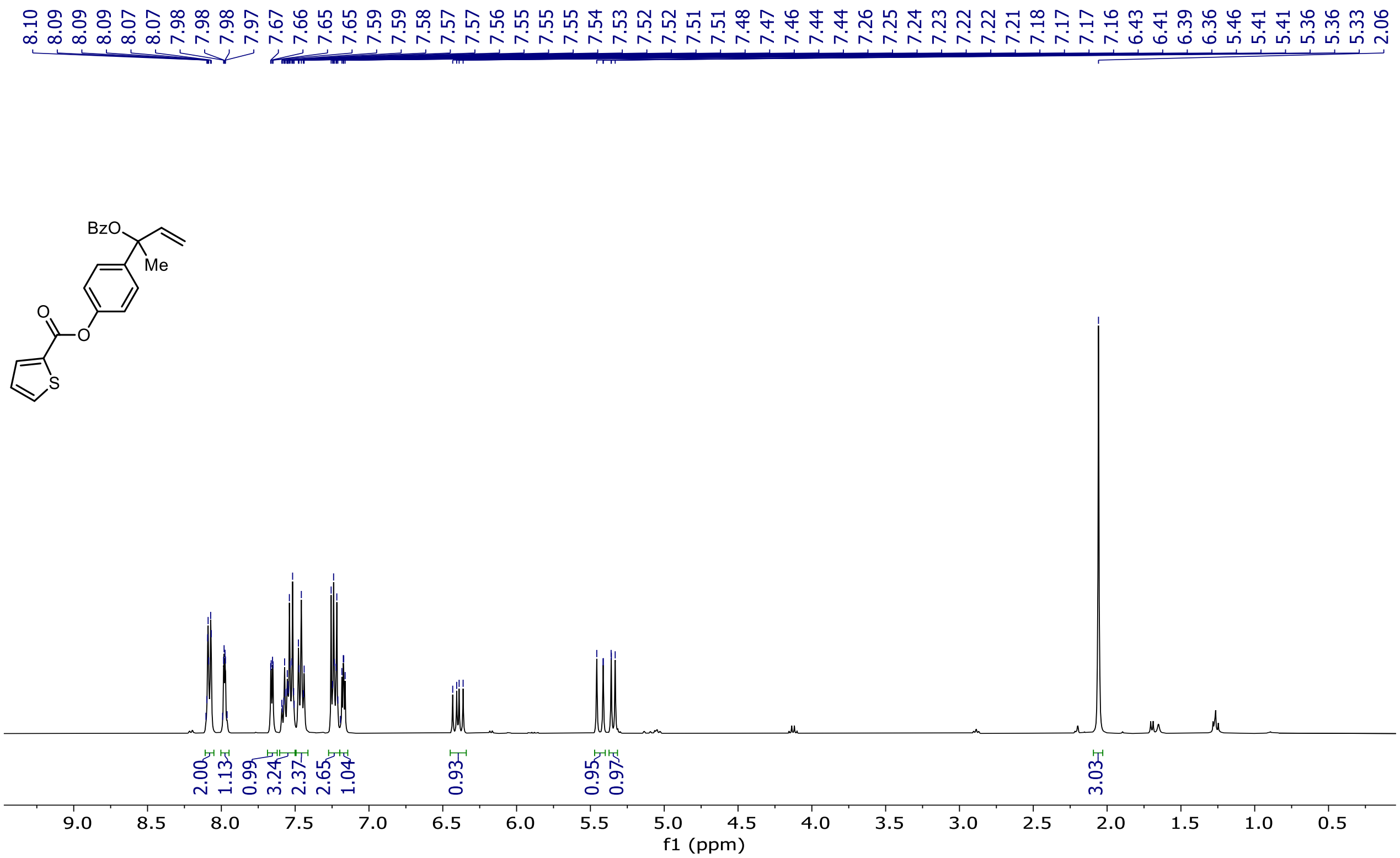

^{13}C NMR (101 MHz, $CDCl_3$, 25 ºC) of 1i

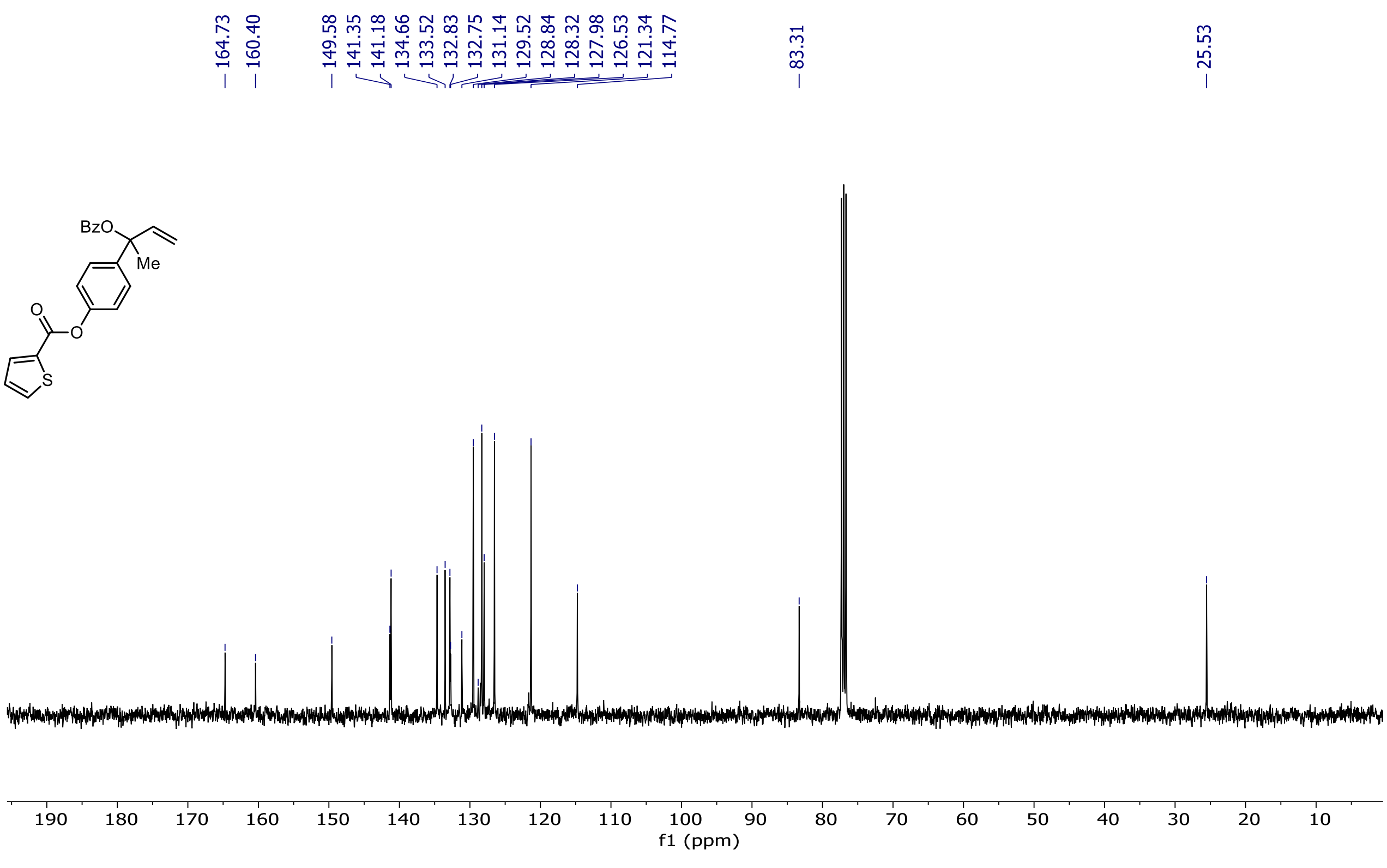

^{1}H NMR (400 MHz, $CDCl_3$, 25 ºC) of 1j

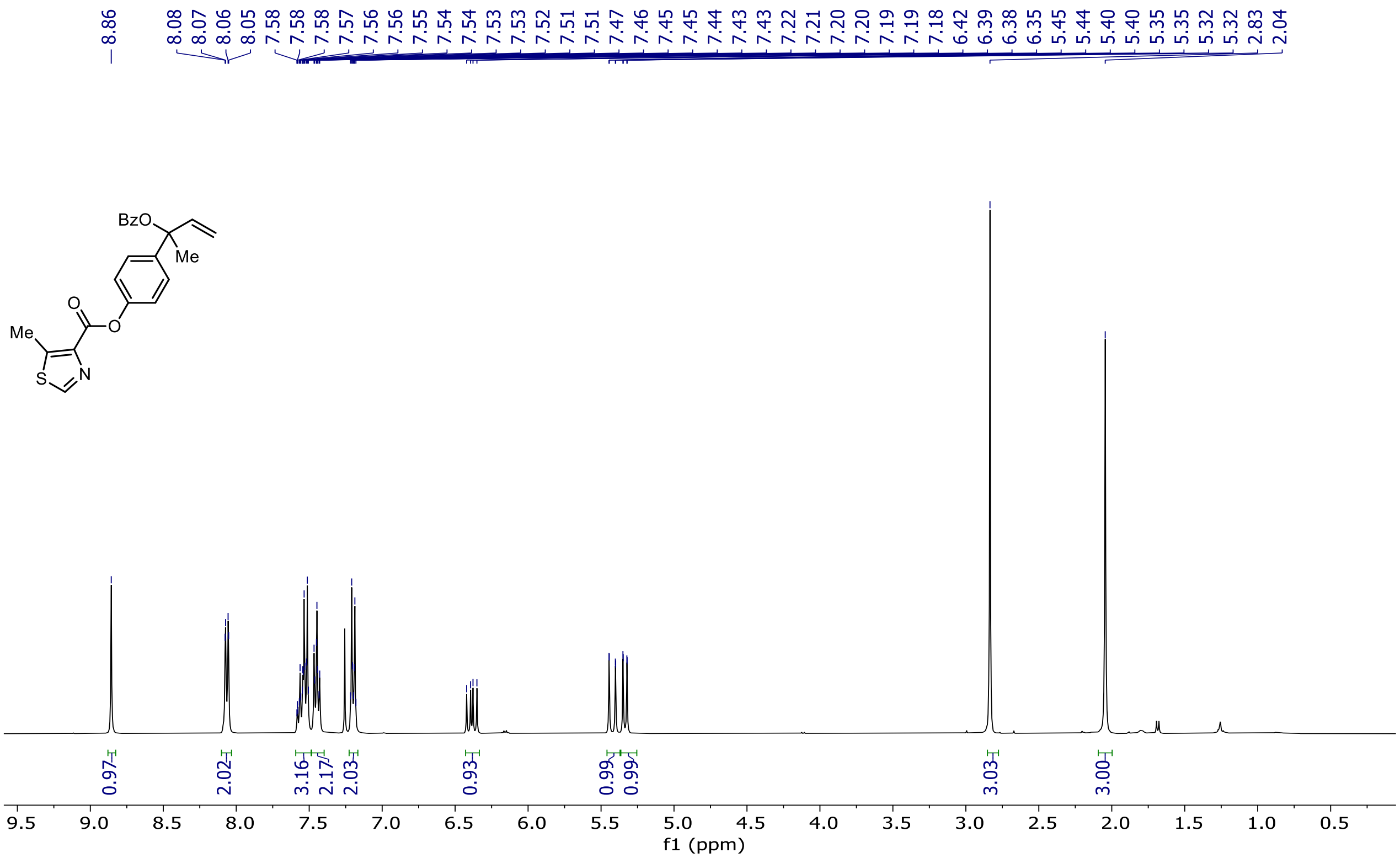

^{13}C NMR (101 MHz, $CDCl_3$, 25 ºC) of 1j

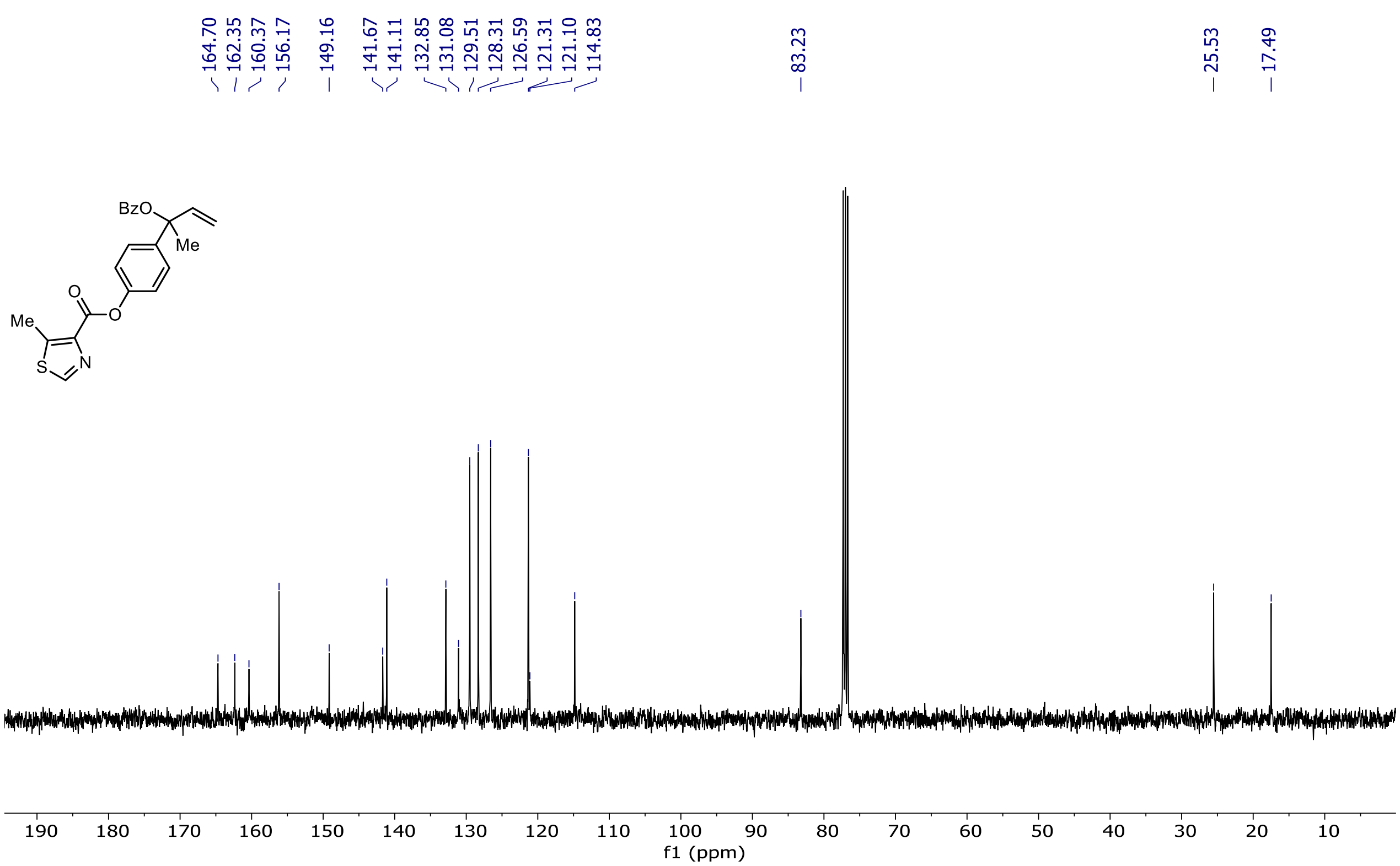

^{1}H NMR (400 MHz, CDCl$_3$, 25 ºC) of 1k

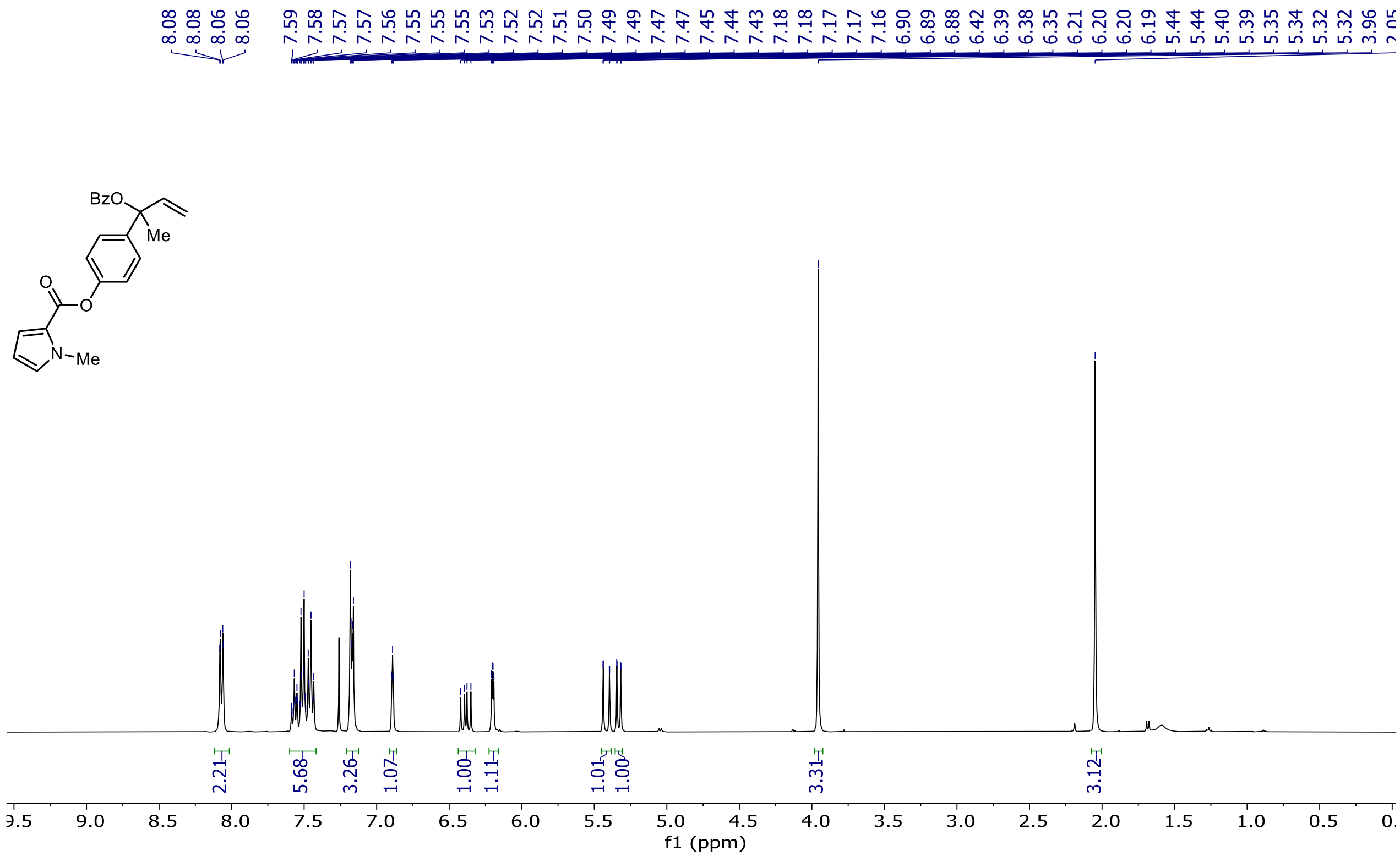

^{13}C NMR (101 MHz, CDCl$_3$, 25 ºC) of 1k

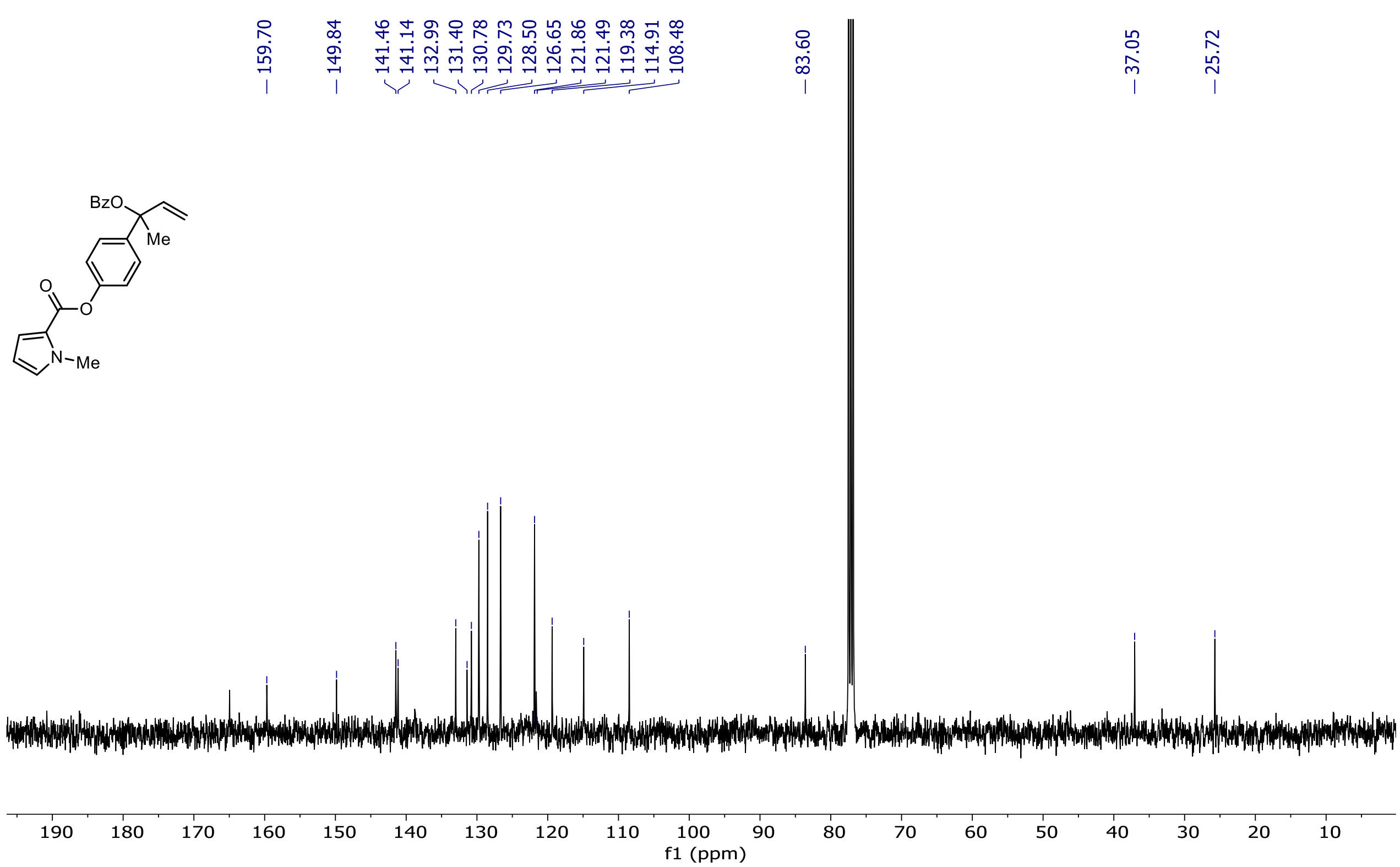

^{1}H NMR (300 MHz, $CDCl_3$, 25 ºC) of 1l

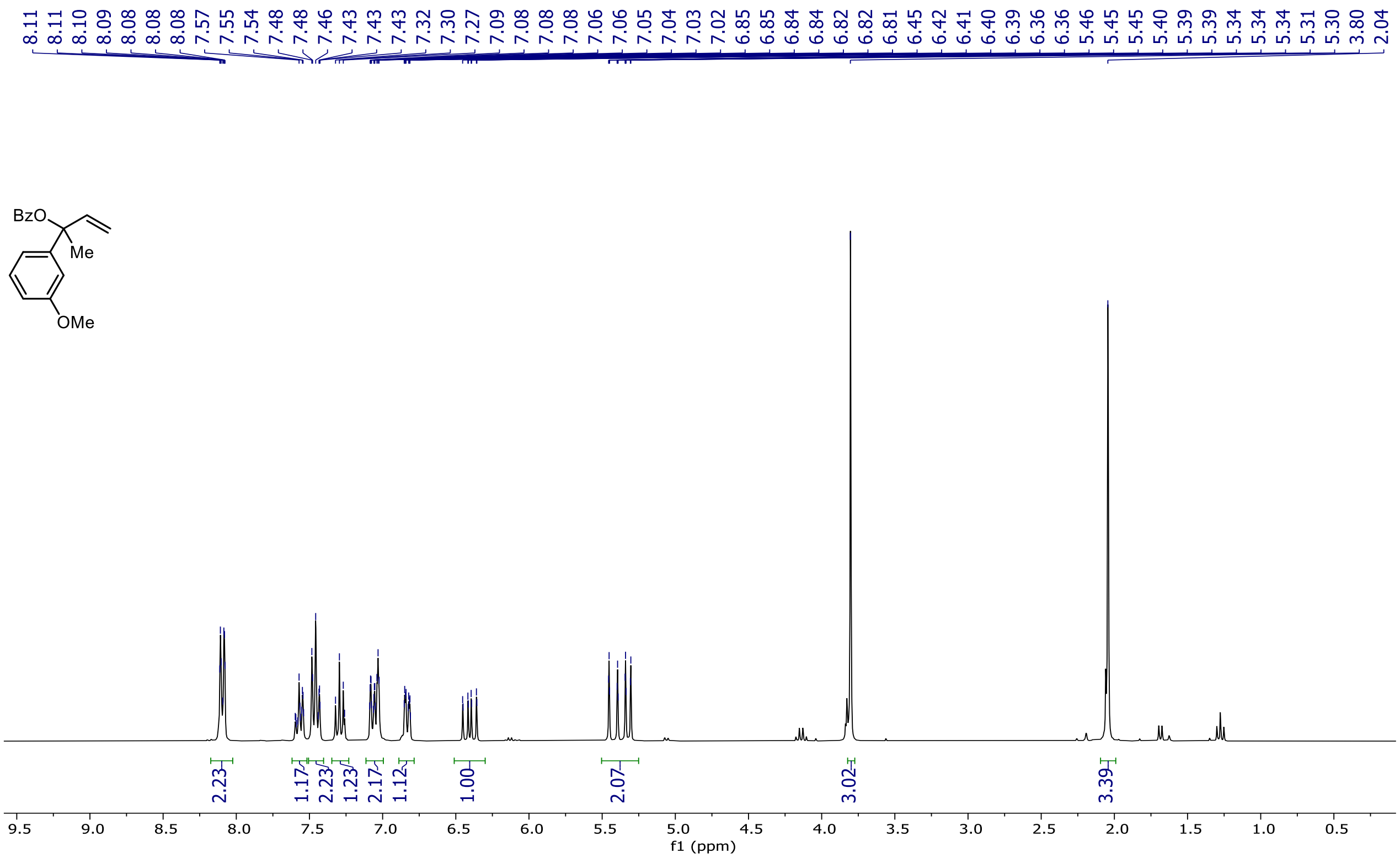

^{13}C NMR (75 MHz, $CDCl_3$, 25 ºC) of 1l

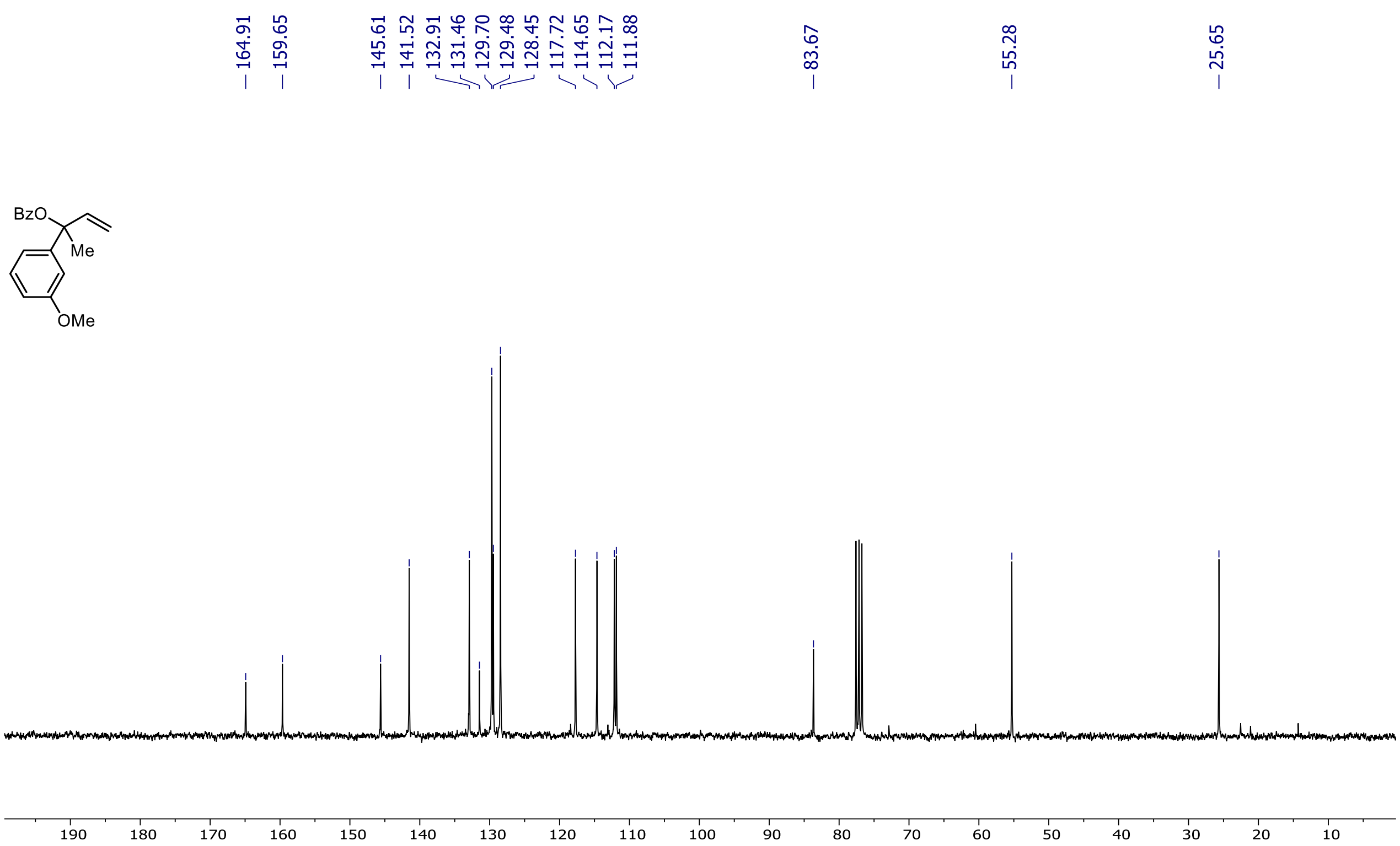

^{1}H NMR (300 MHz, CDCl$_3$, 25 ºC) of 1m

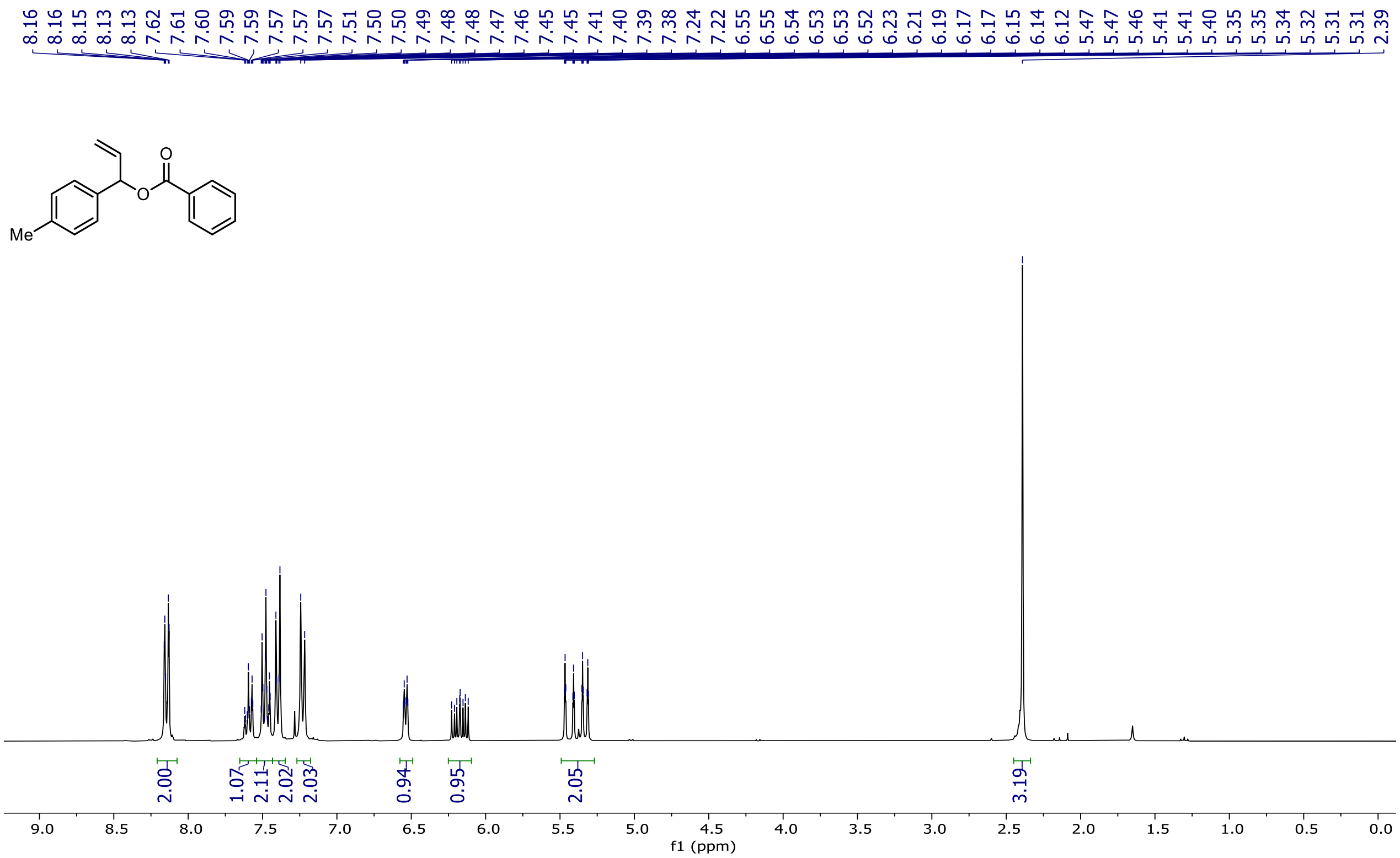

^{13}C NMR (75 MHz, CDCl$_3$, 25 ºC) of 1m

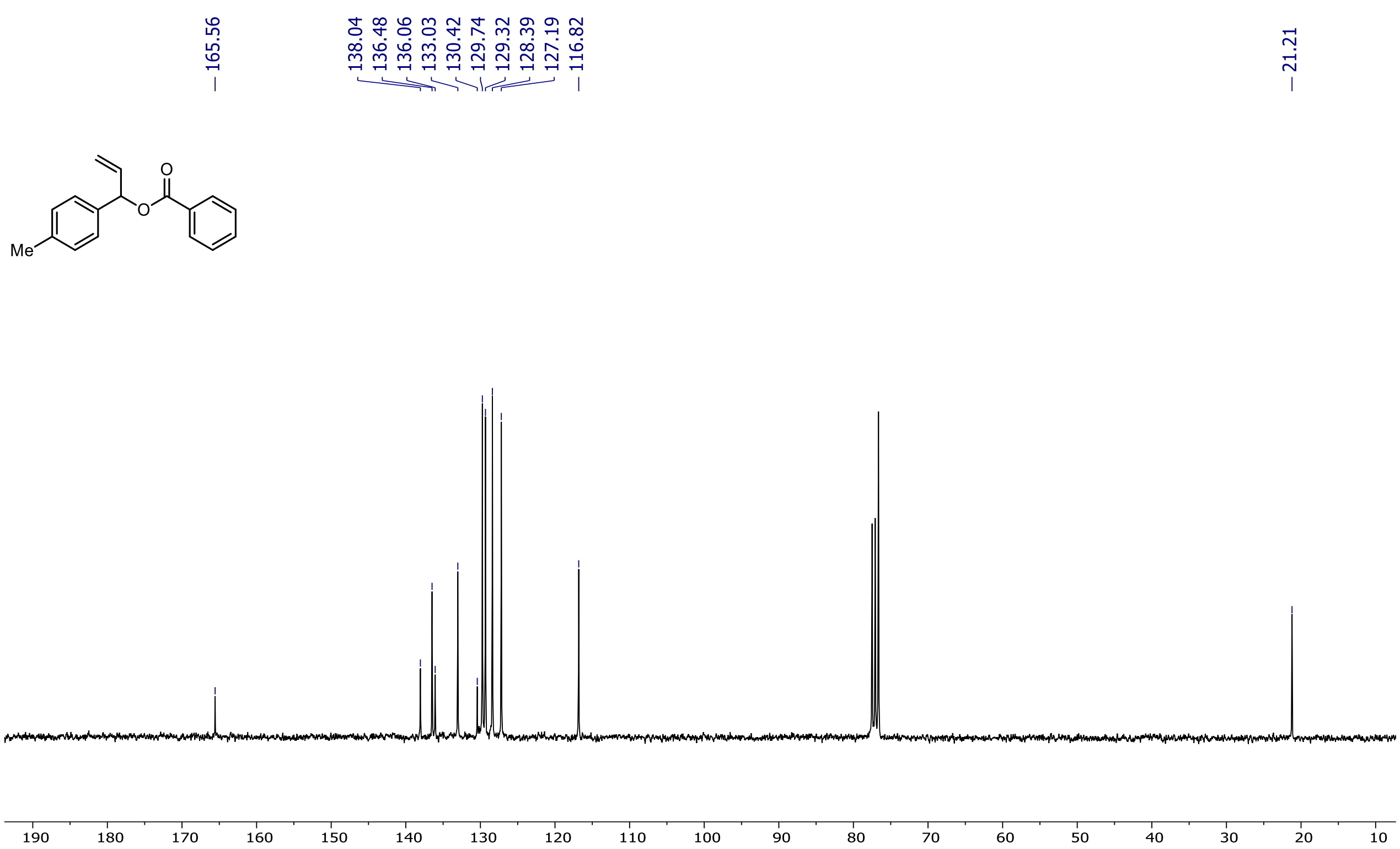

^{1}H NMR (300 MHz, $CDCl_3$, 25 ºC) of 1n

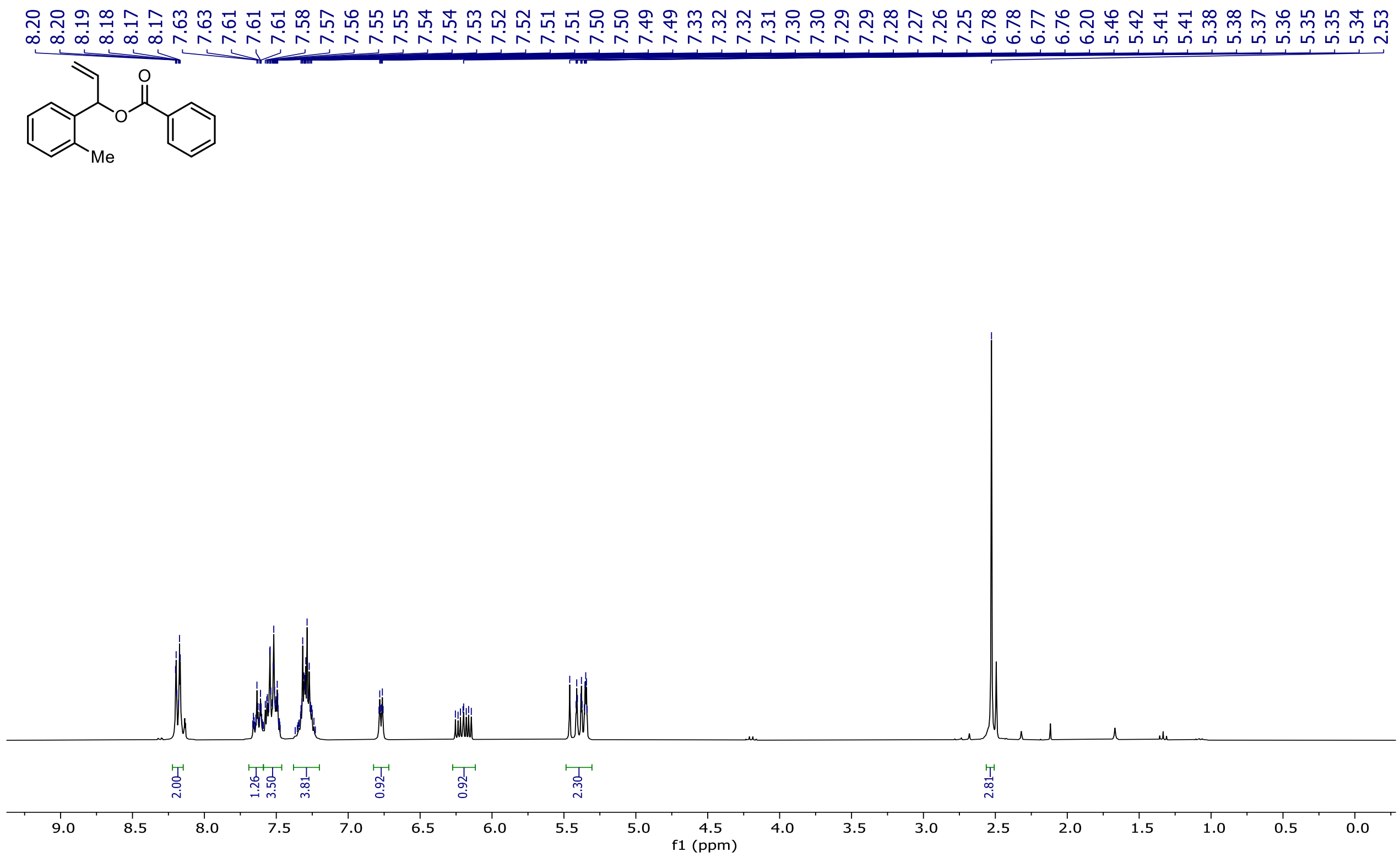

^{13}C NMR (75 MHz, $CDCl_3$, 25 ºC) of 1n

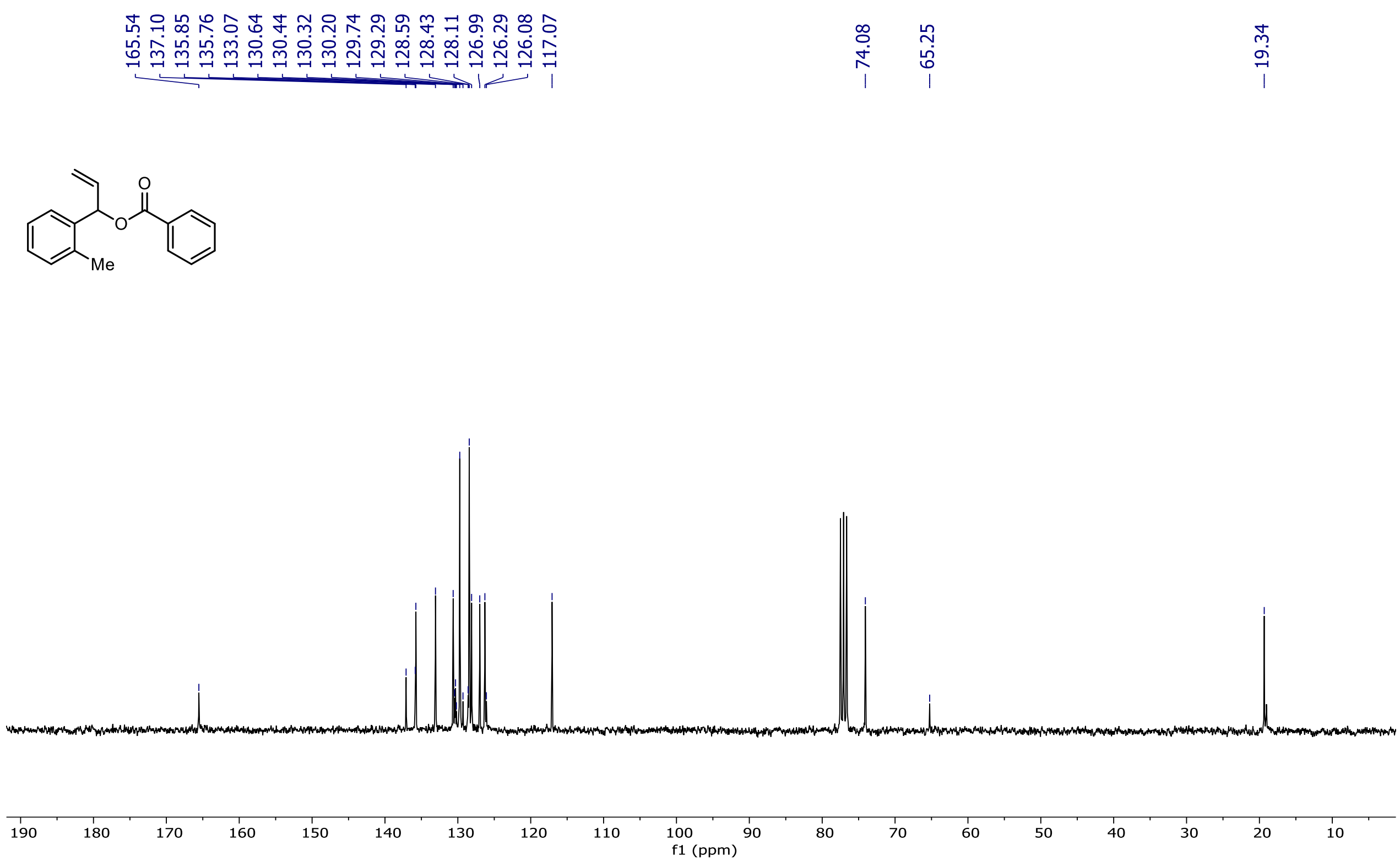

^{1}H NMR (300 MHz, CDCl$_3$, 25 ºC) of 1o

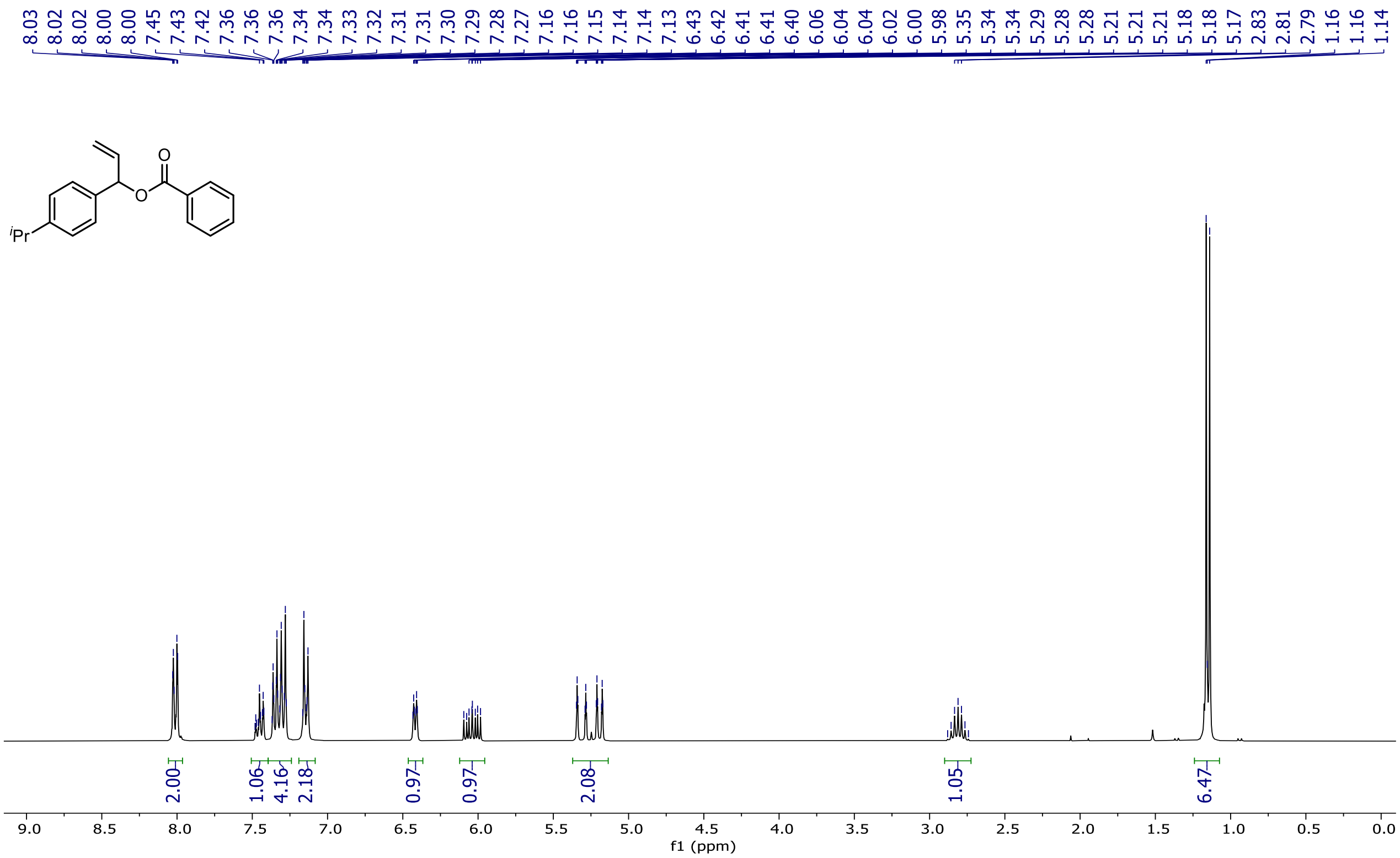

^{13}C NMR (75 MHz, CDCl$_3$, 25 ºC) of 1o

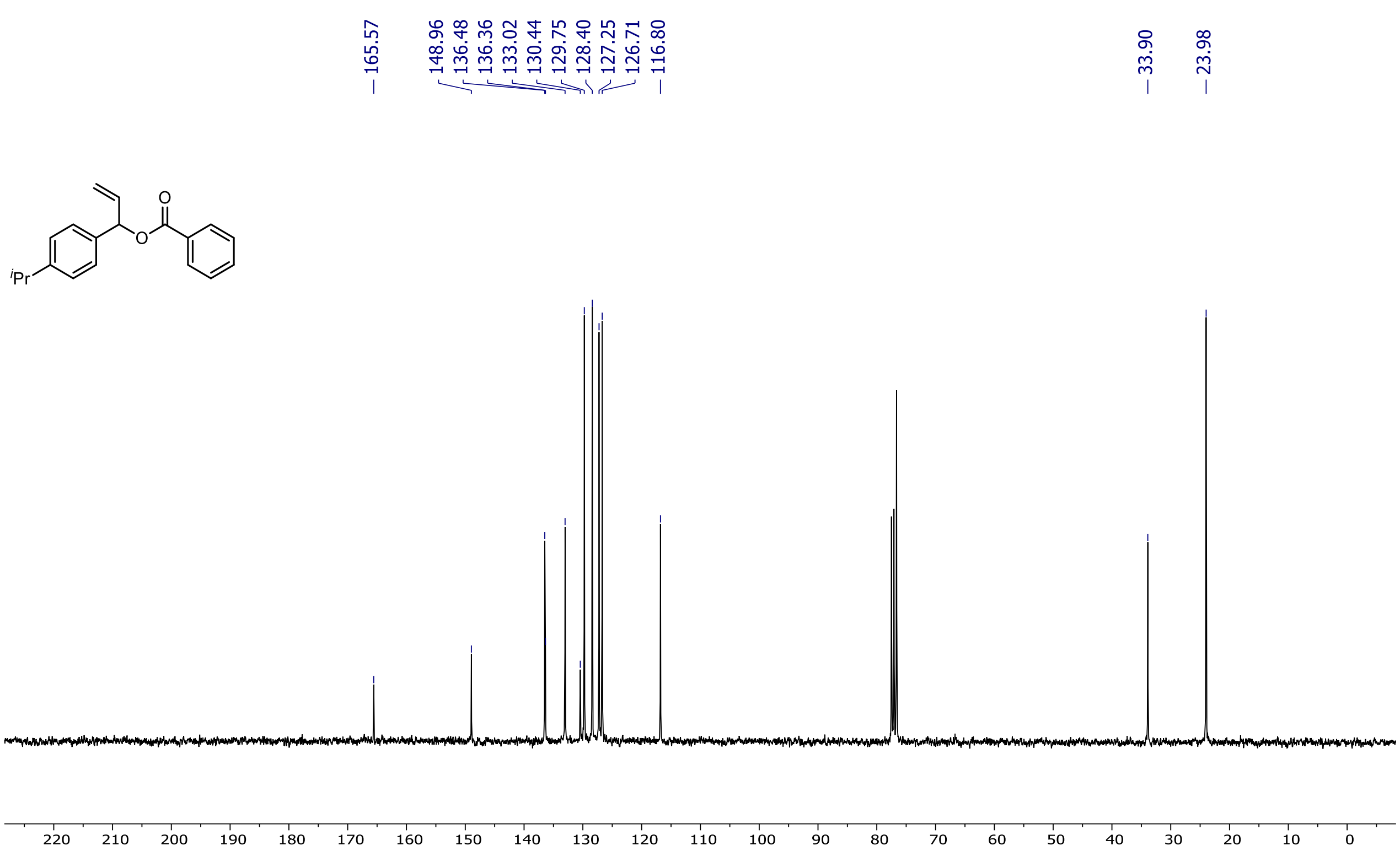

^{1}H NMR (300 MHz, $CDCl_3$, 25 ºC) of 1p

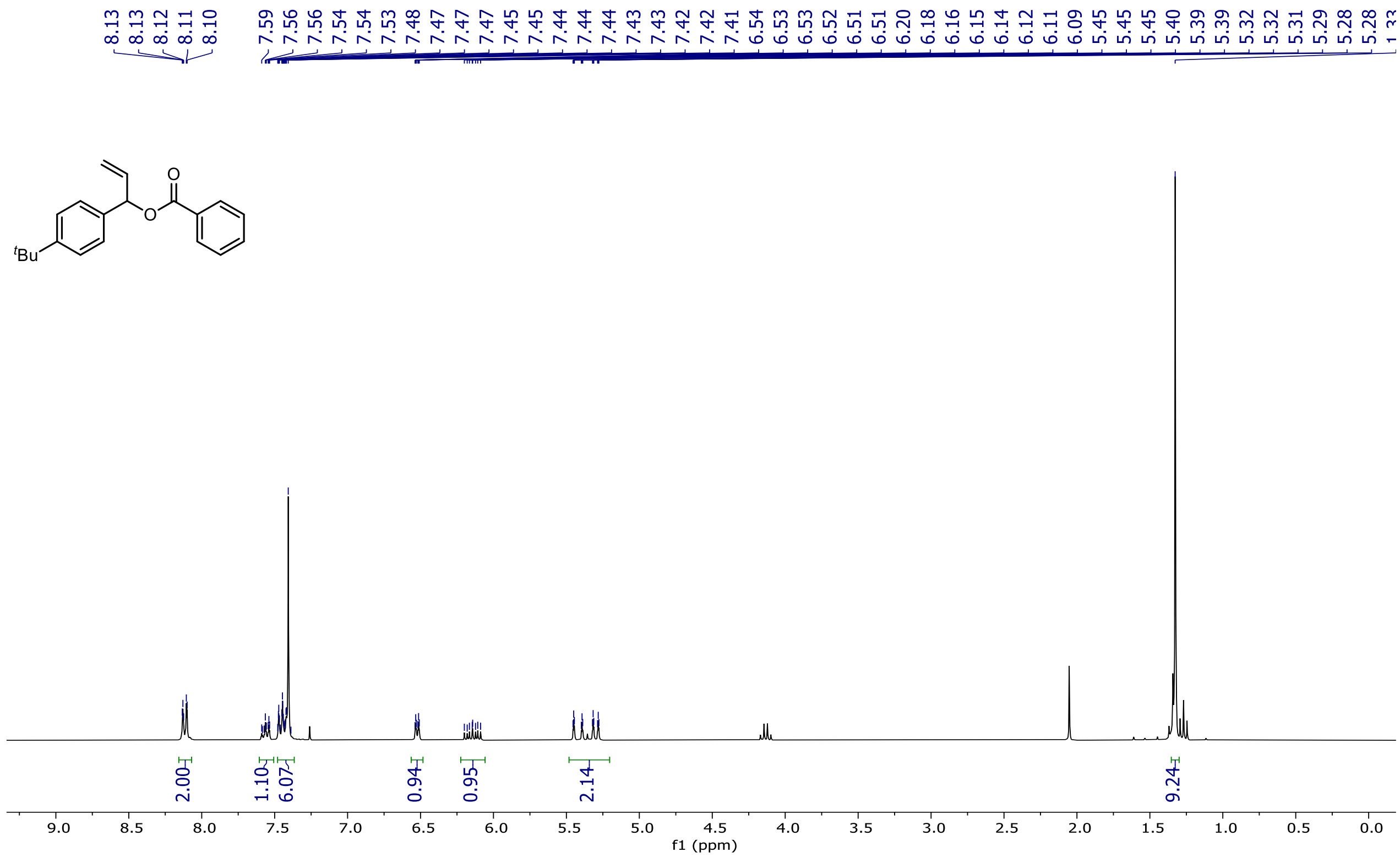

^{13}C NMR (75 MHz, $CDCl_3$, 25 ºC) of 1p

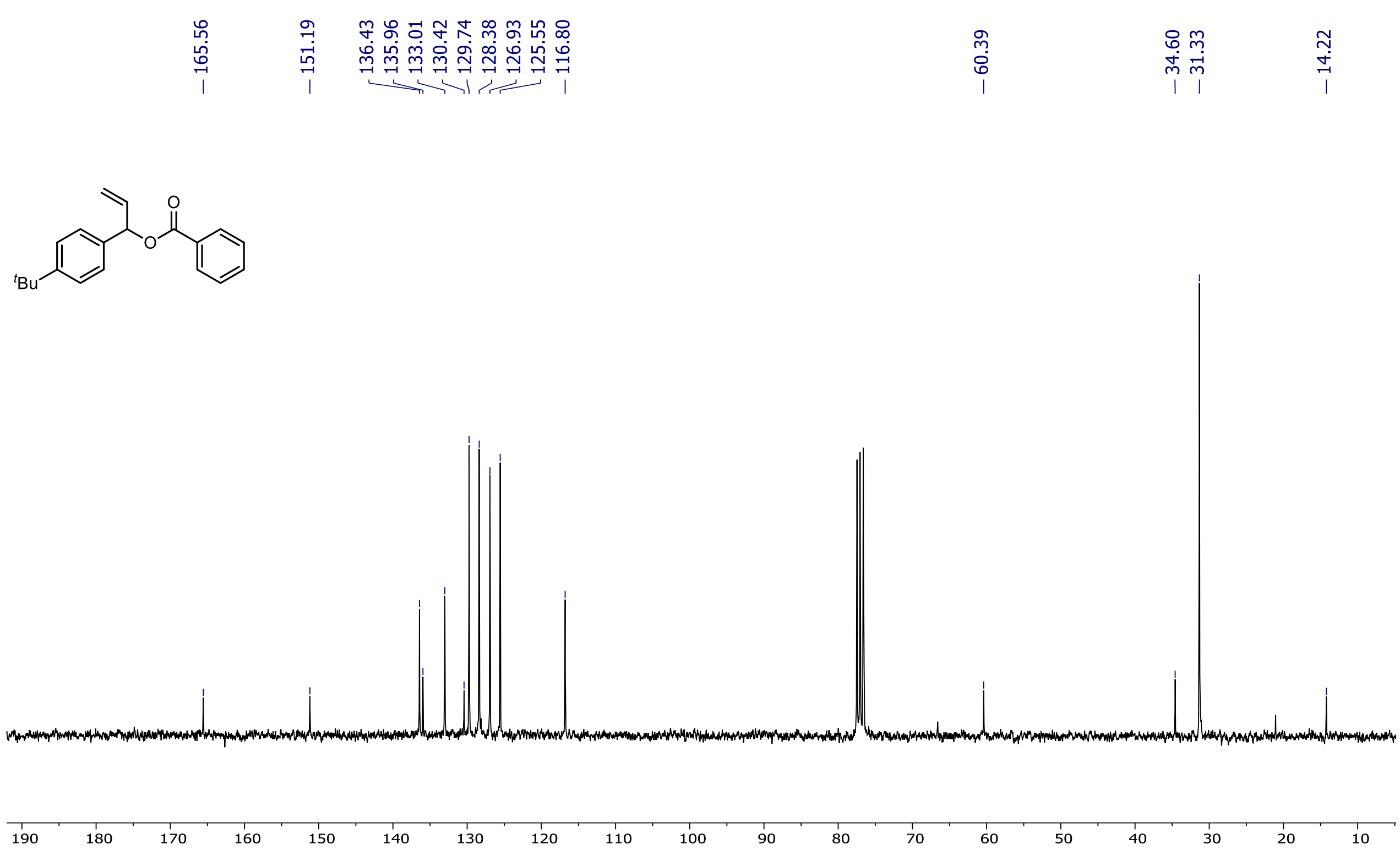

^{1}H NMR (300 MHz, $CDCl_3$, 25 ºC) of 1q

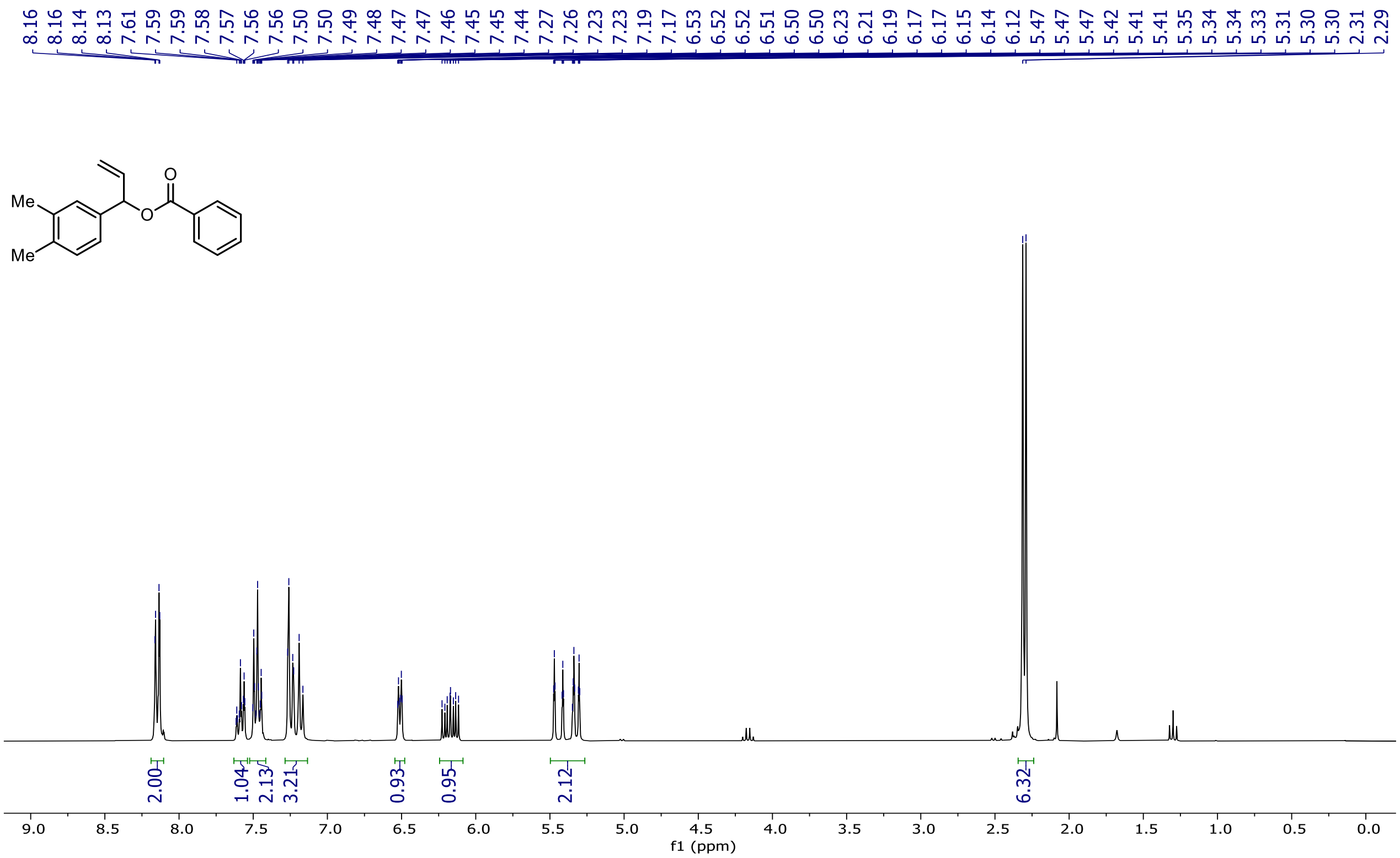

^{13}C NMR (75 MHz, $CDCl_3$, 25 ºC) of 1q

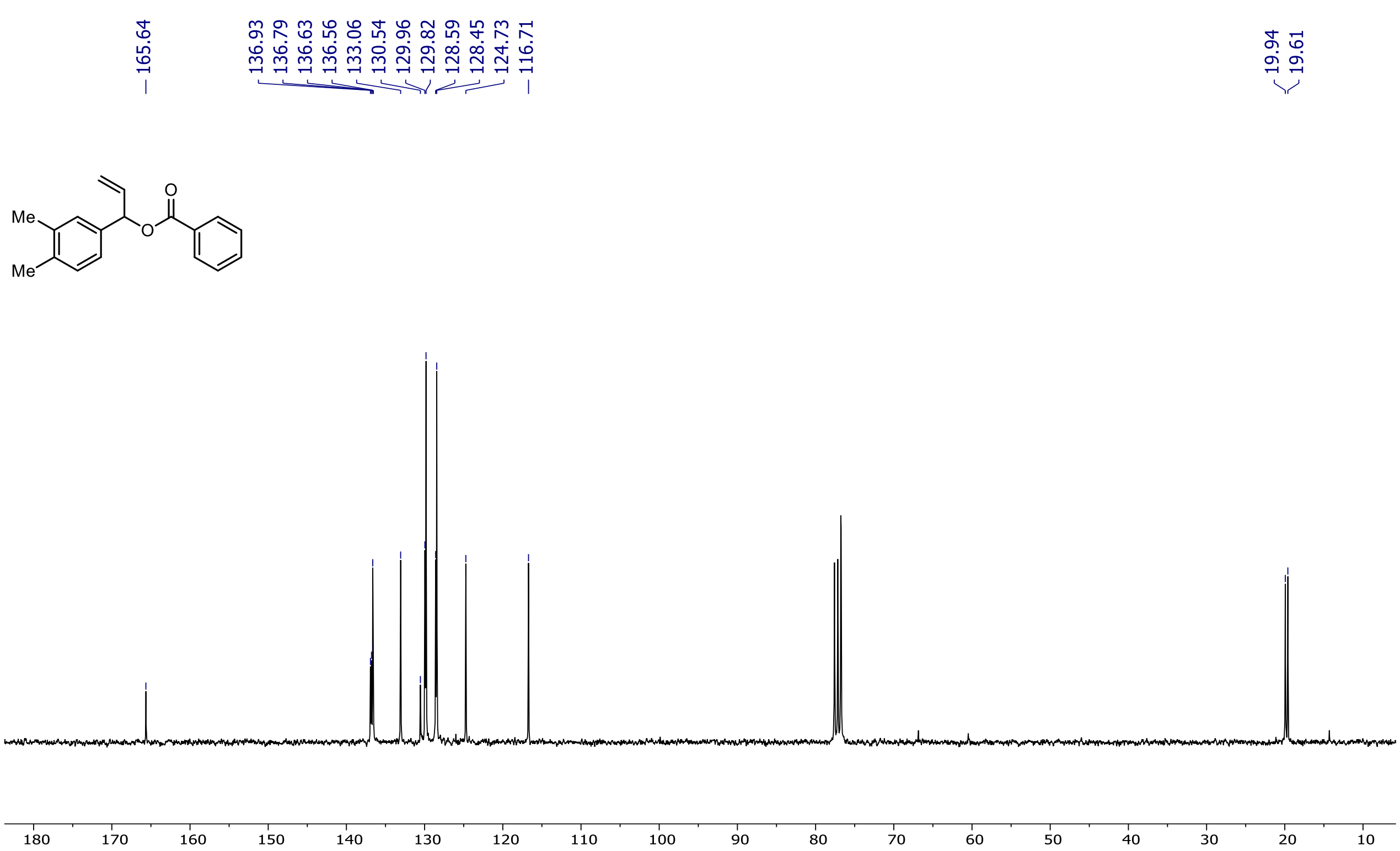

^{1}H NMR (400 MHz, $CDCl_3$, 25 ºC) of 1r

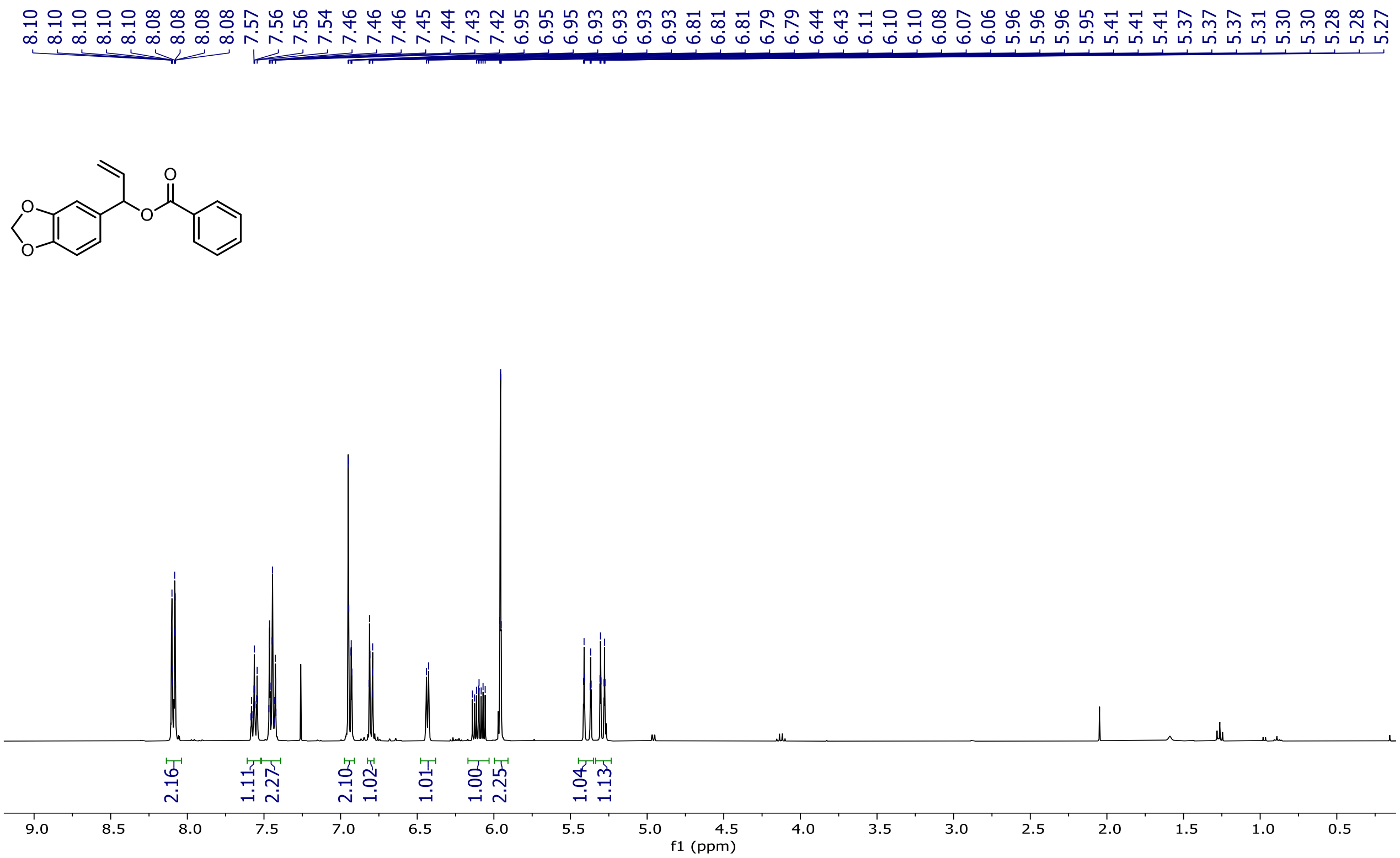

^{13}C NMR (101 MHz, $CDCl_3$, 25 ºC) of 1r

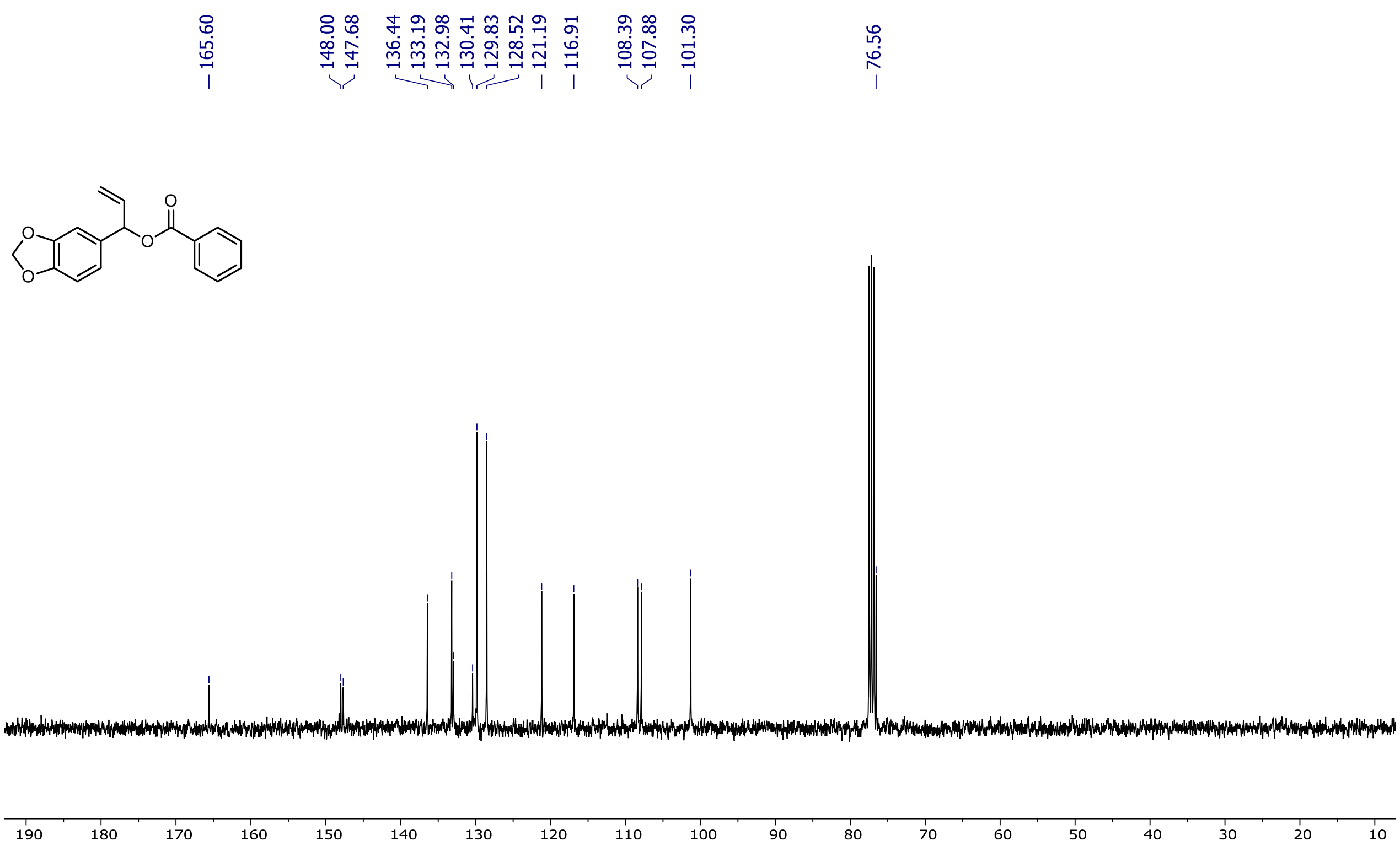

^{1}H NMR (400 MHz, CDCl$_3$, 25 ºC) of 1s

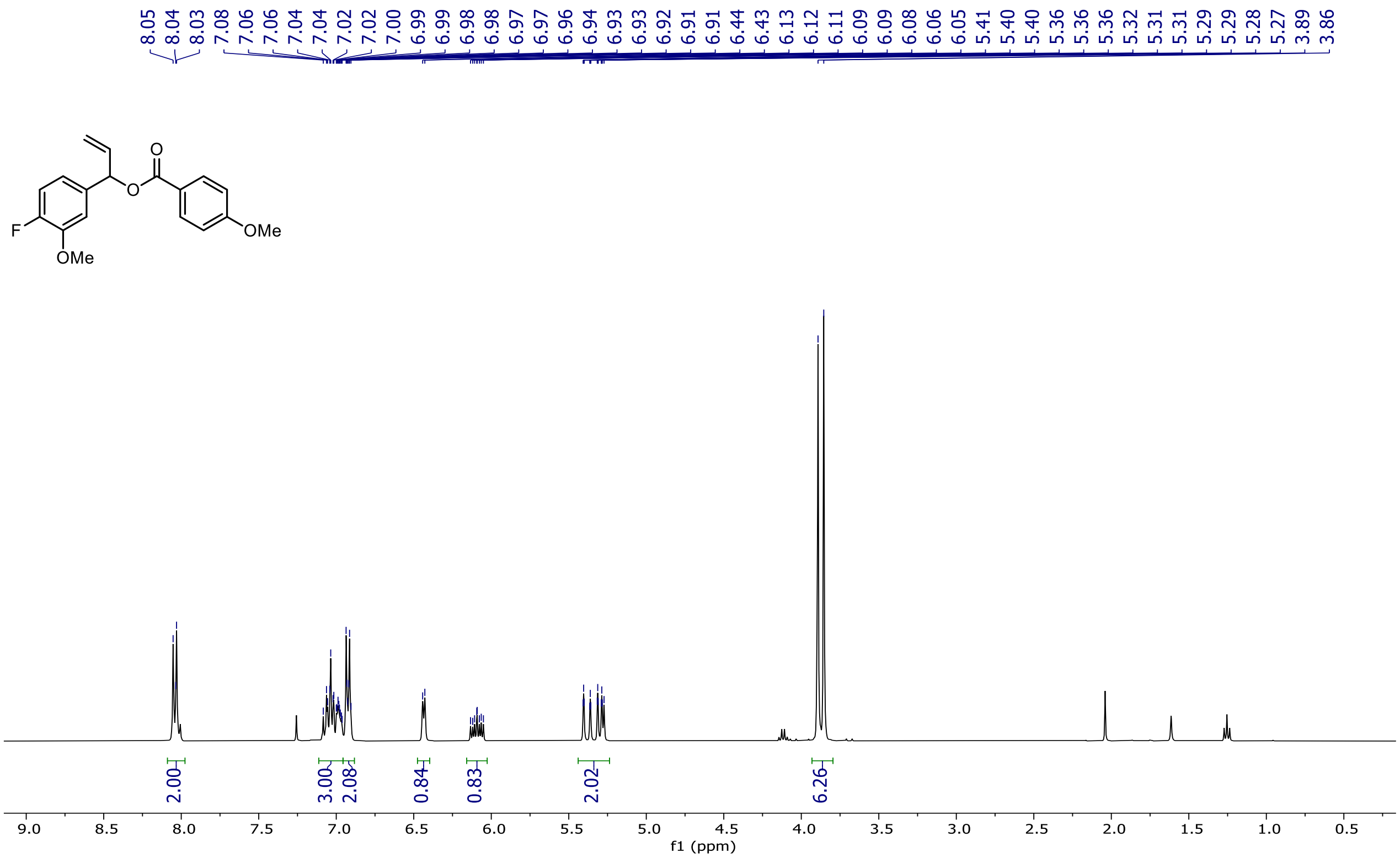

^{13}C NMR (101 MHz, CDCl$_3$, 25 ºC) of 1s

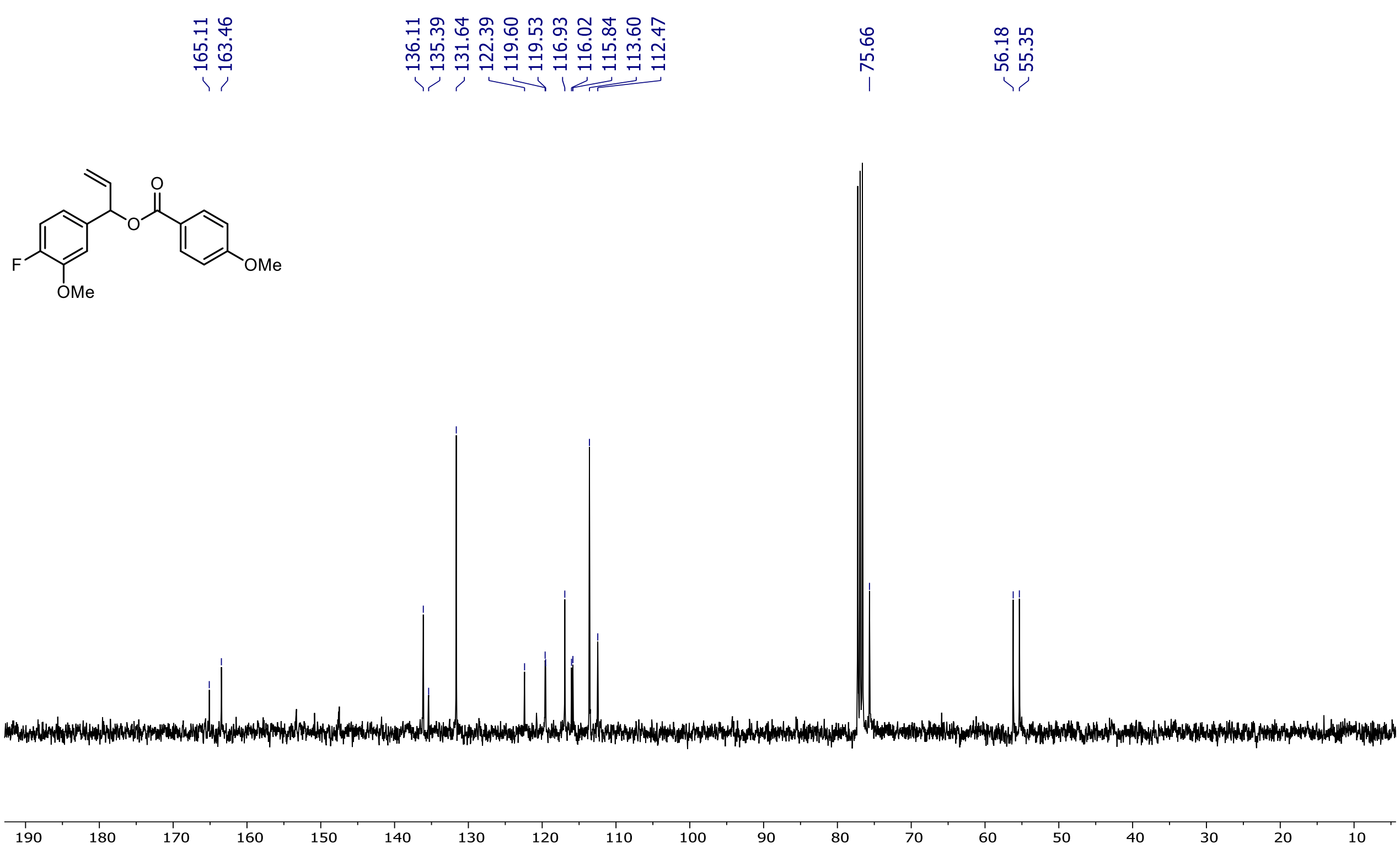

^{19}F NMR (376 MHz, $CDCl_3$, 25 ºC) of 1s

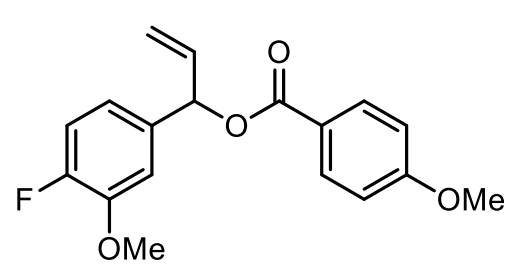

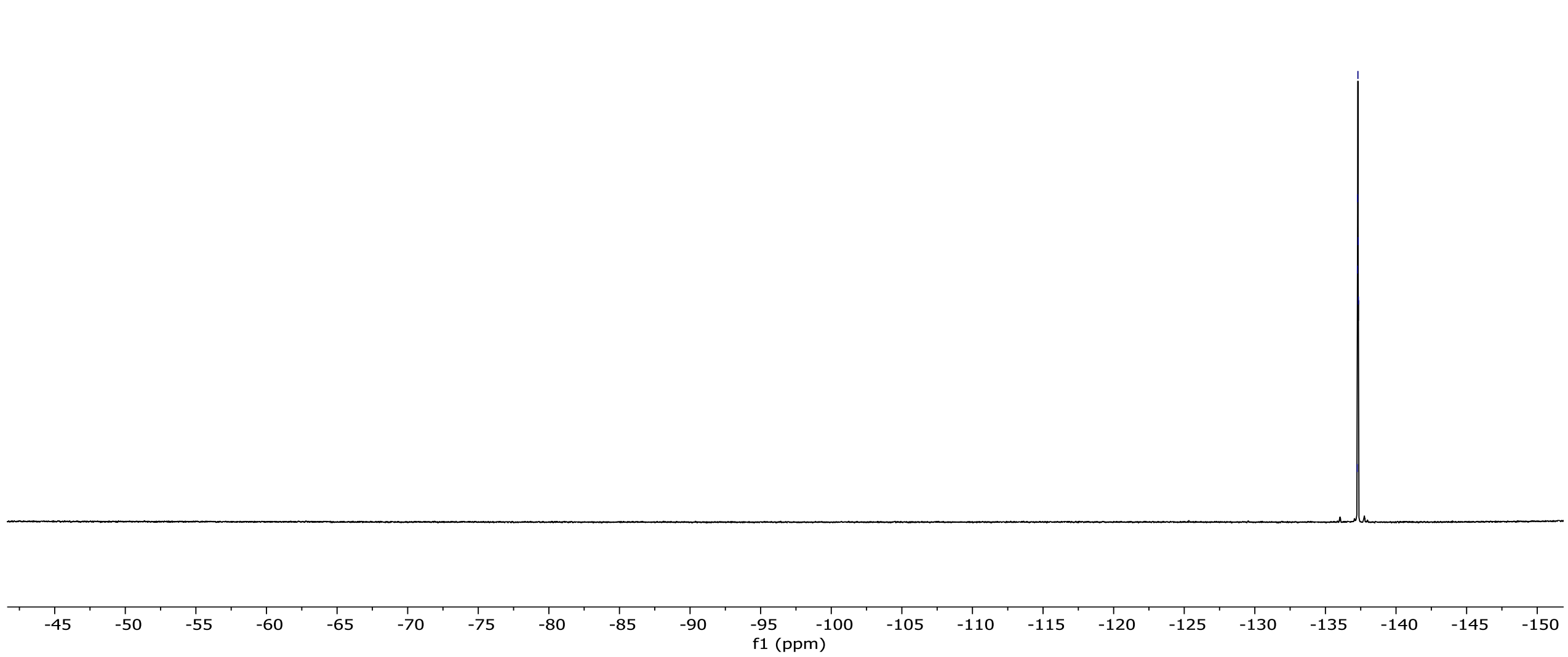

^{1}H NMR (300 MHz, $CDCl_3$, 25 ºC) of 1t

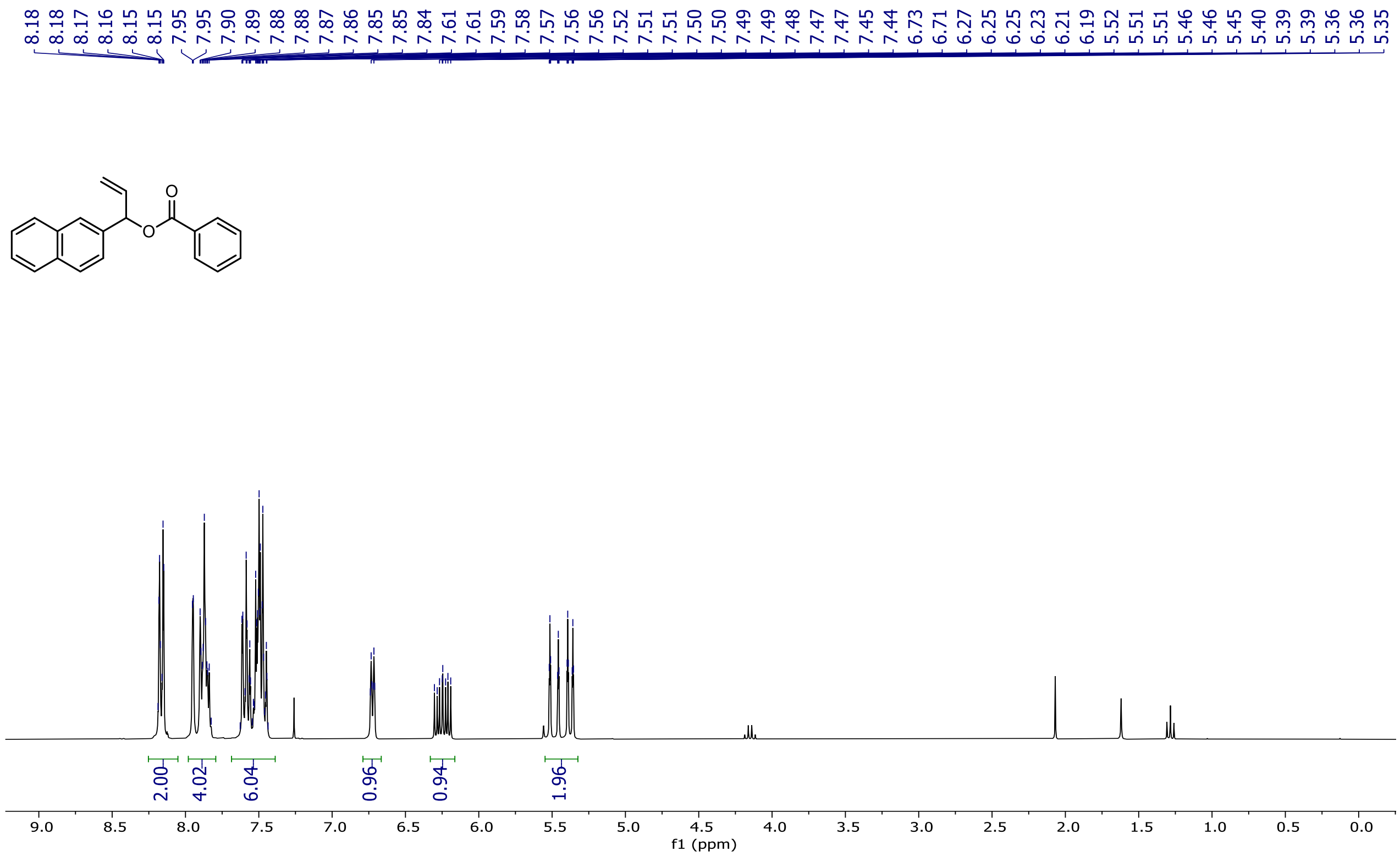

^{13}C NMR (75 MHz, $CDCl_3$, 25 ºC) of 1t

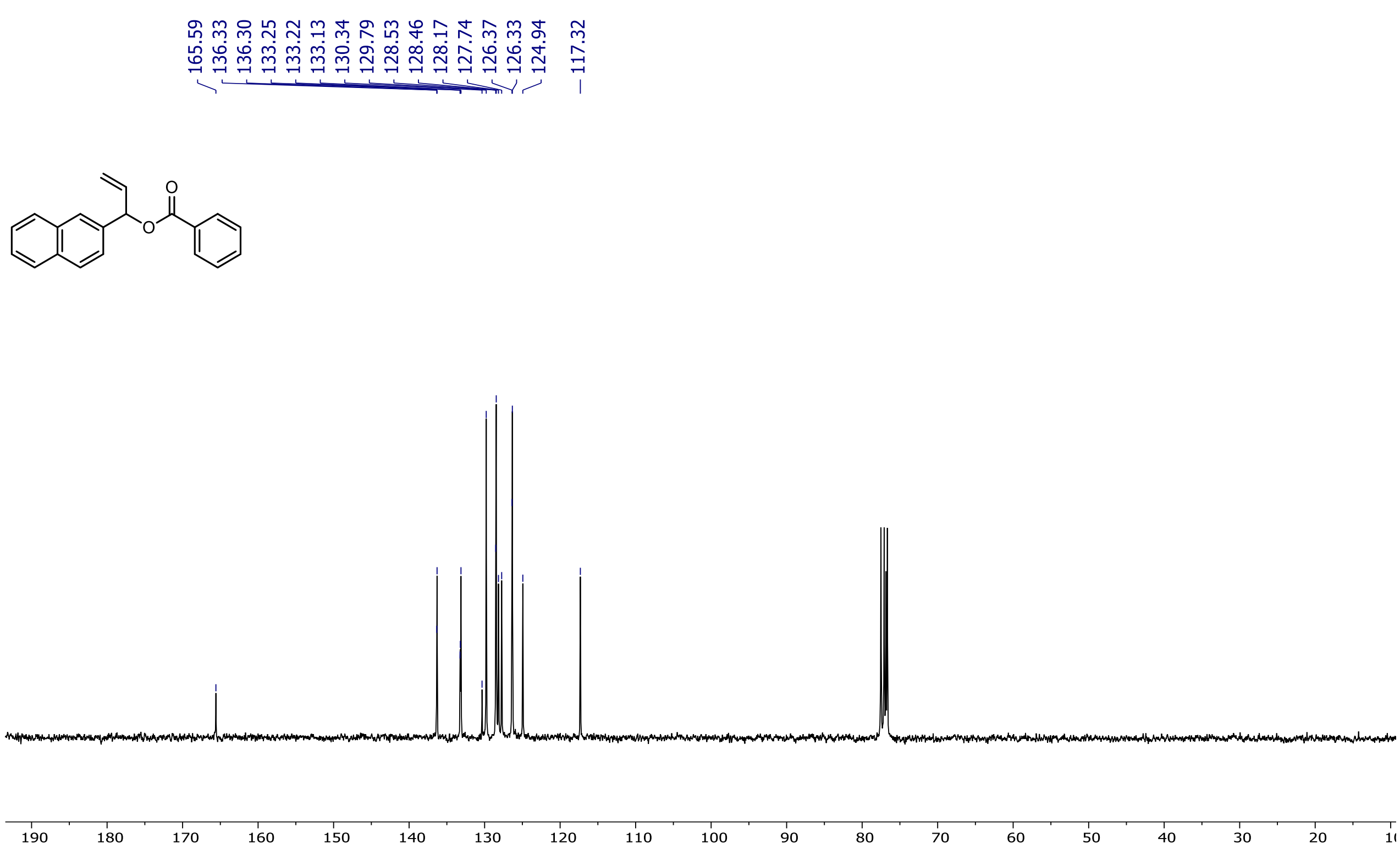

^{1}H NMR (300 MHz, $CDCl_3$, 25 ºC) of 1u

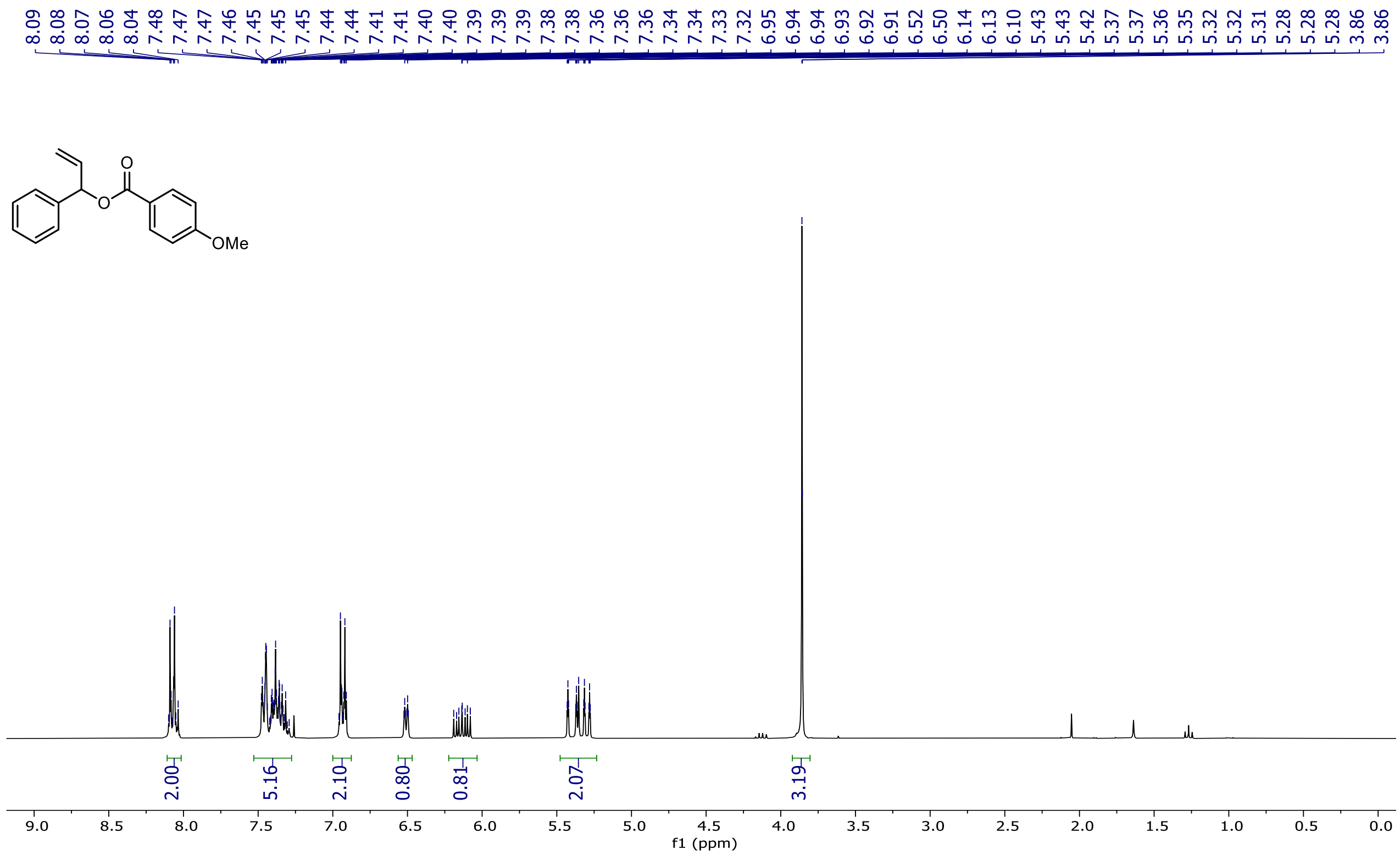

^{13}C NMR (75 MHz, $CDCl_3$, 25 ºC) of 1u

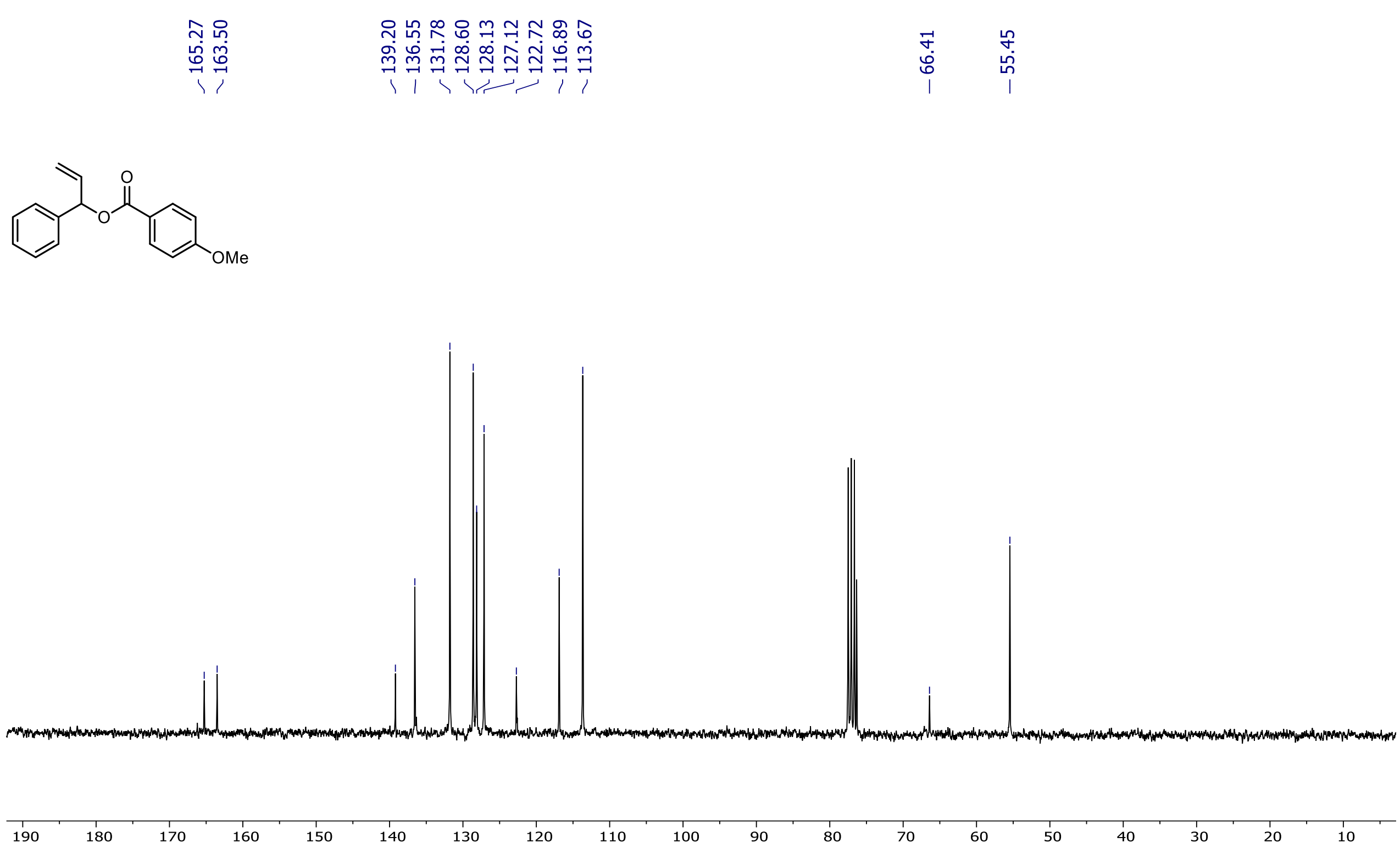

^{1}H NMR (400 MHz, $CDCl_3$, 25 ºC) of 1v

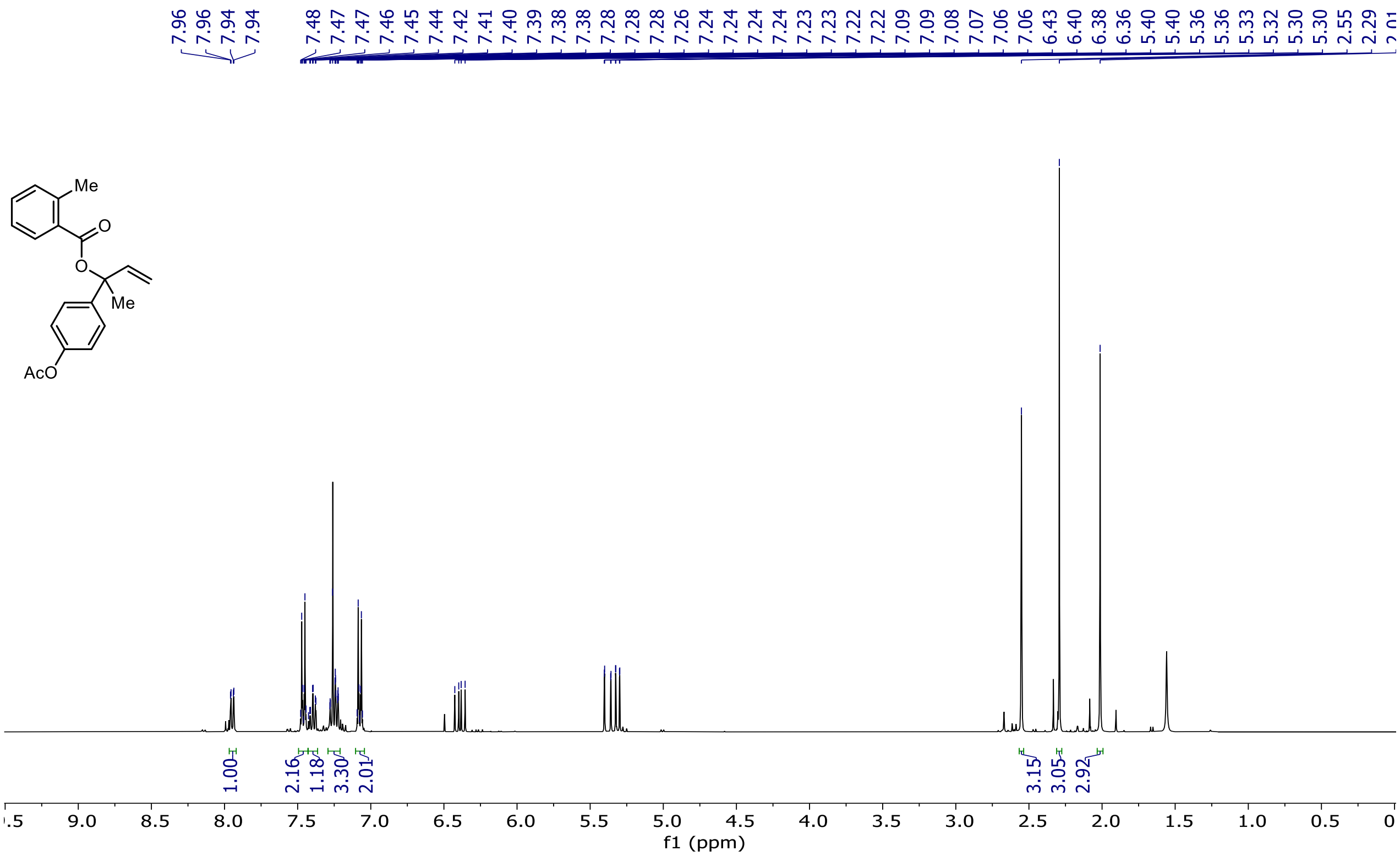

^{13}C NMR (201 MHz, $CDCl_3$, 25 ºC) of 1v

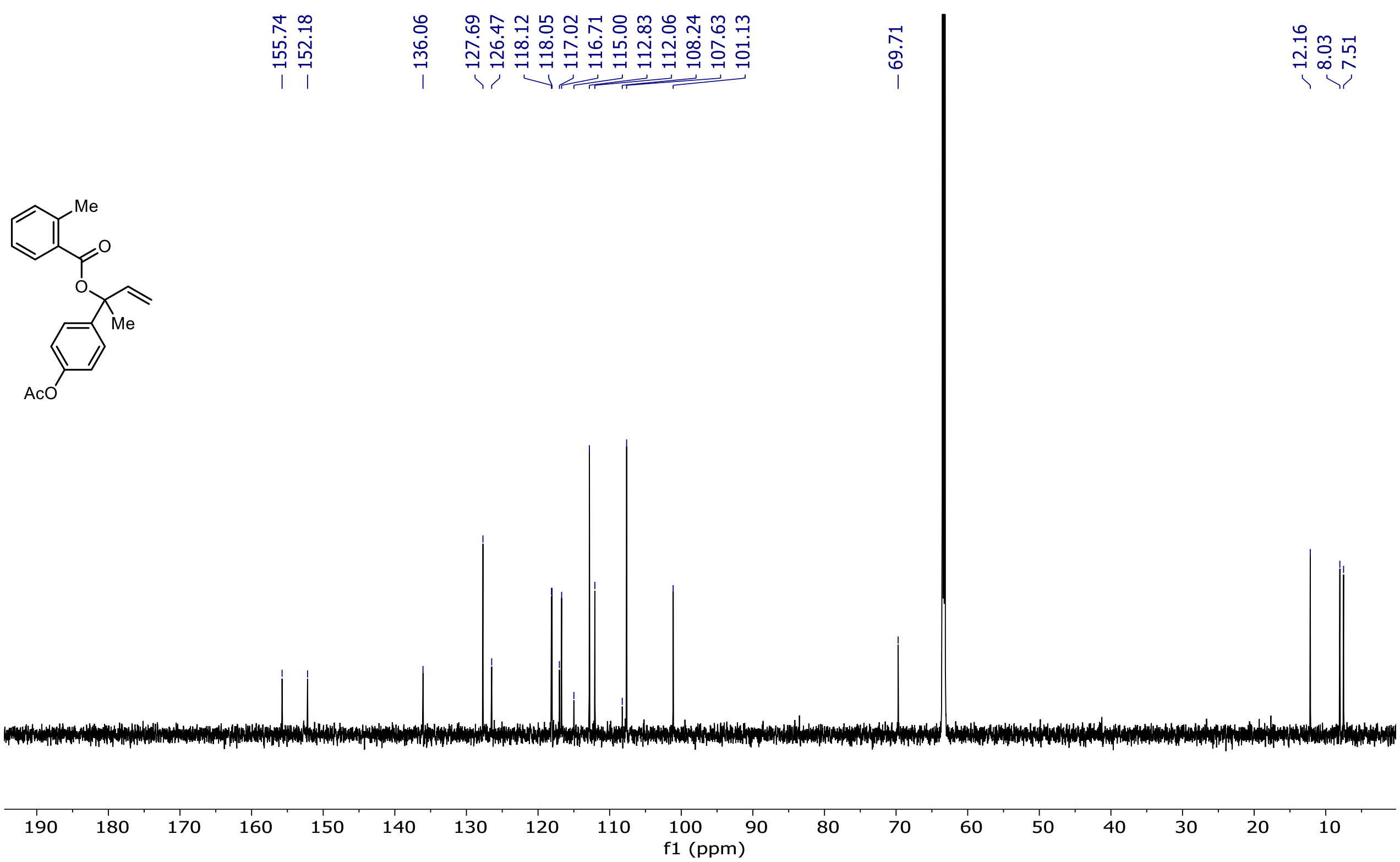

^{1}H NMR (400 MHz, $CDCl_3$, 25 ºC) of 1w

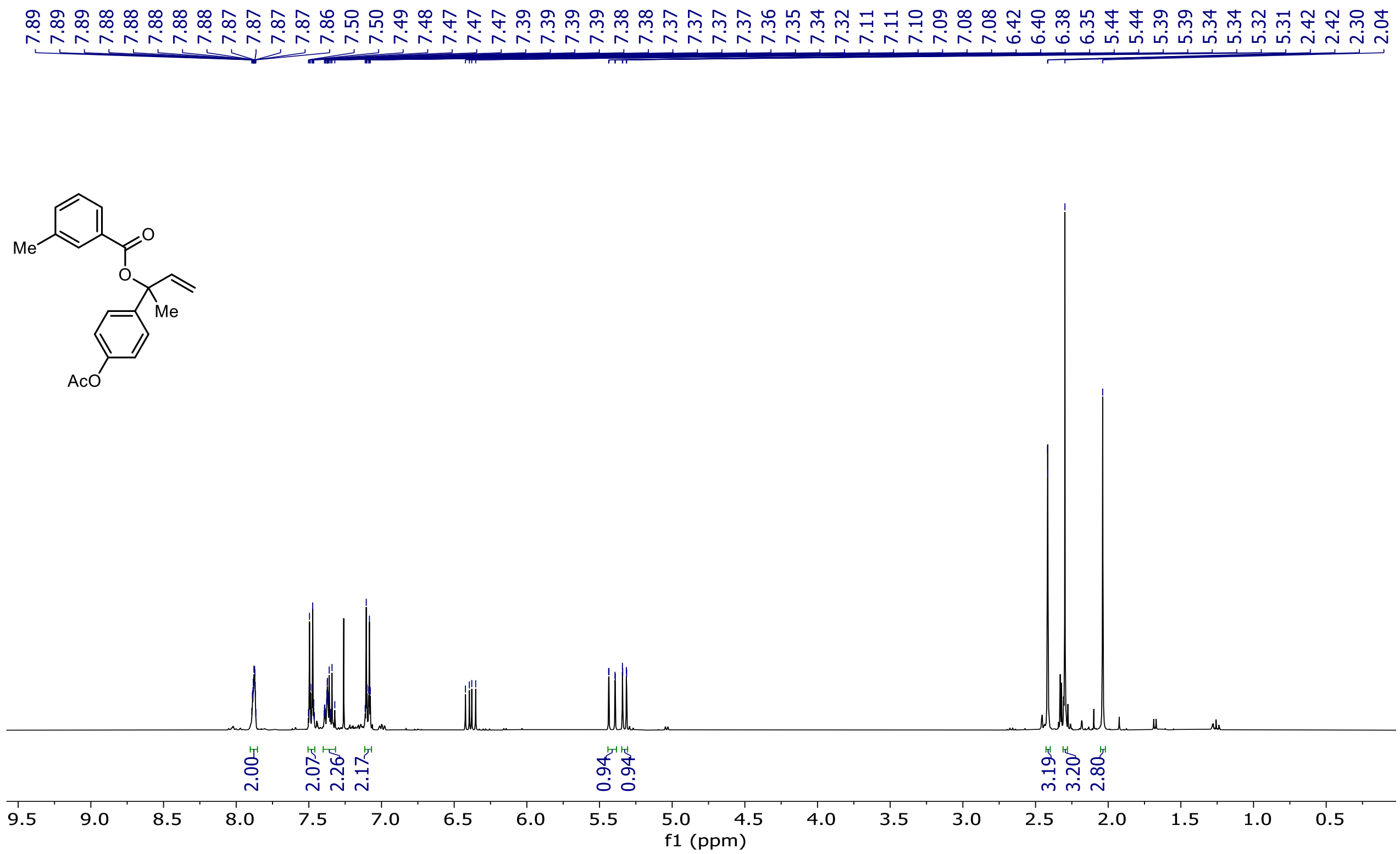

^{13}C NMR (101 MHz, $CDCl_3$, 25 ºC) of 1w

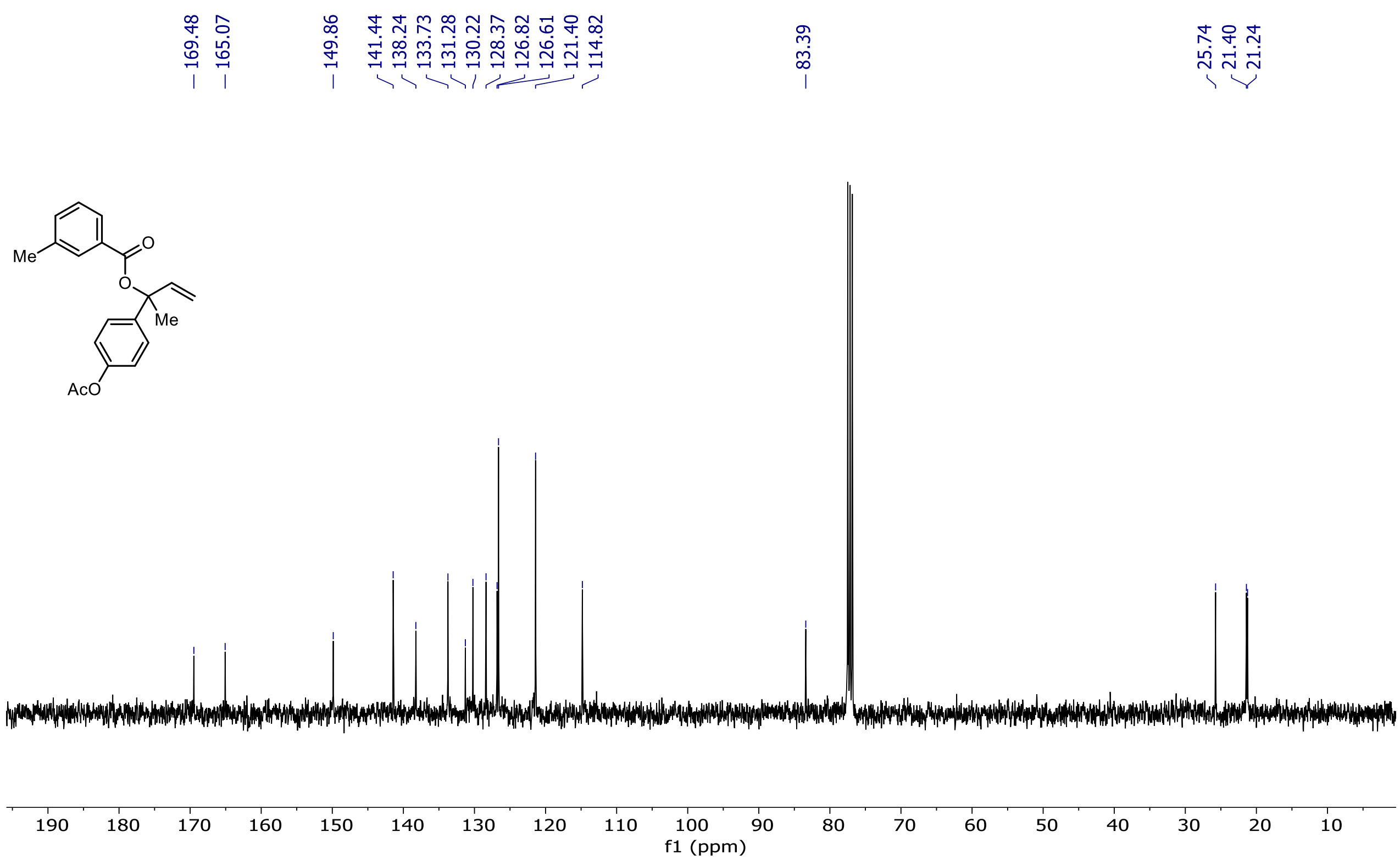

^{1}H NMR (400 MHz, CDCl$_3$, 25 ºC) of 1x

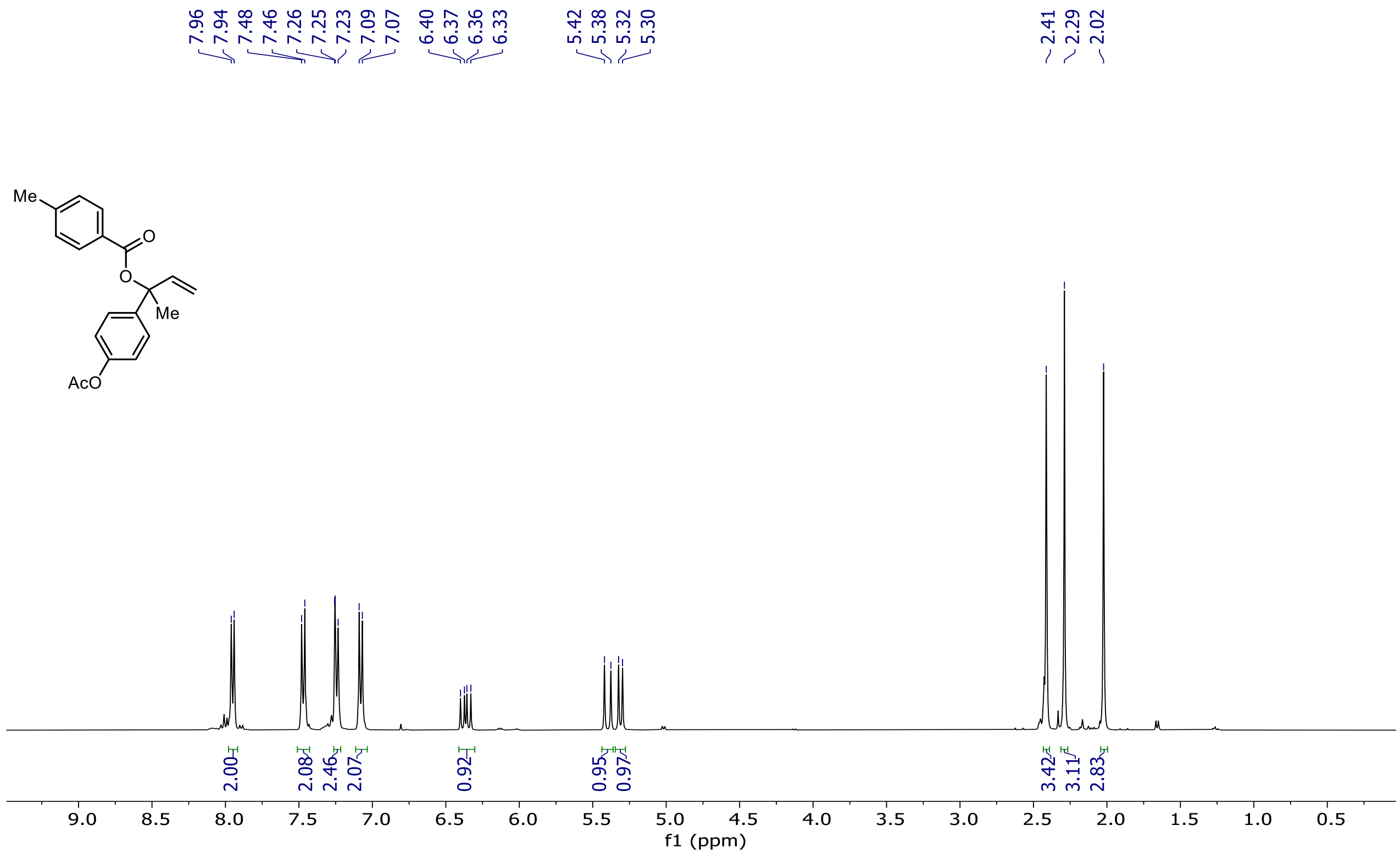

^{13}C NMR (101 MHz, CDCl$_3$, 25 ºC) of 1x

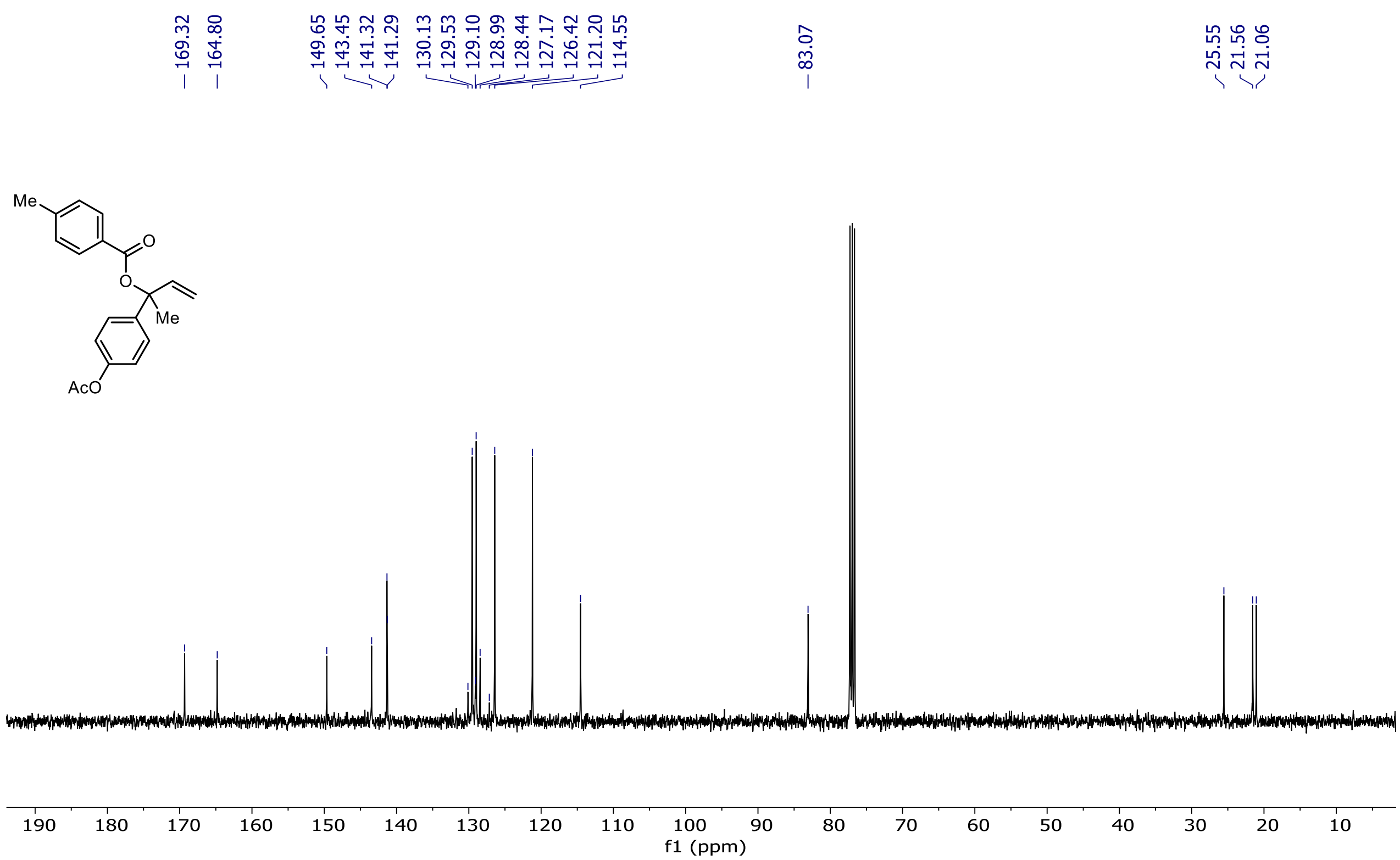

^{1}H NMR (400 MHz, $CDCl_3$, 25 ºC) of 1y

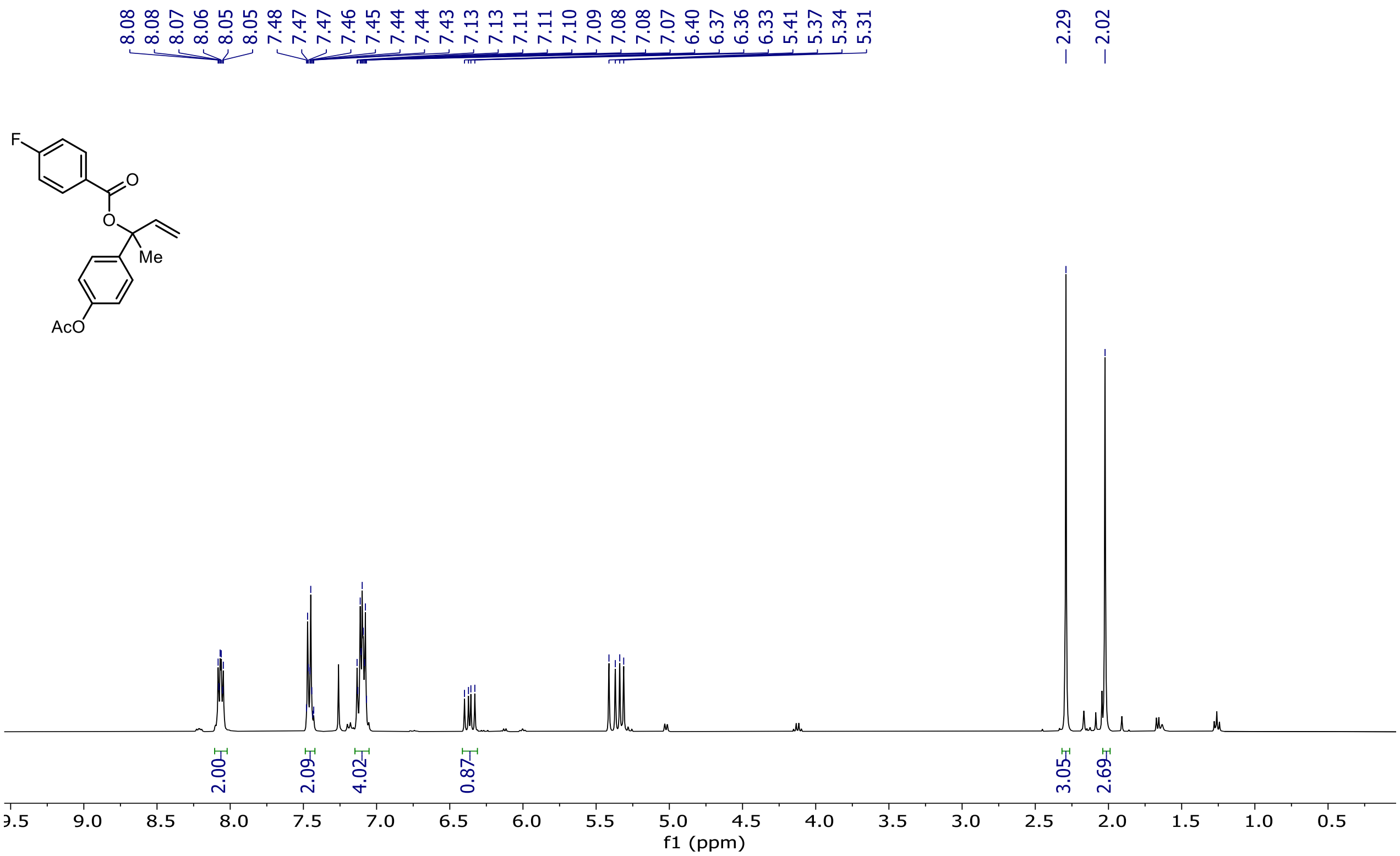

^{13}C NMR (101 MHz, $CDCl_3$, 25 ºC) of 1y

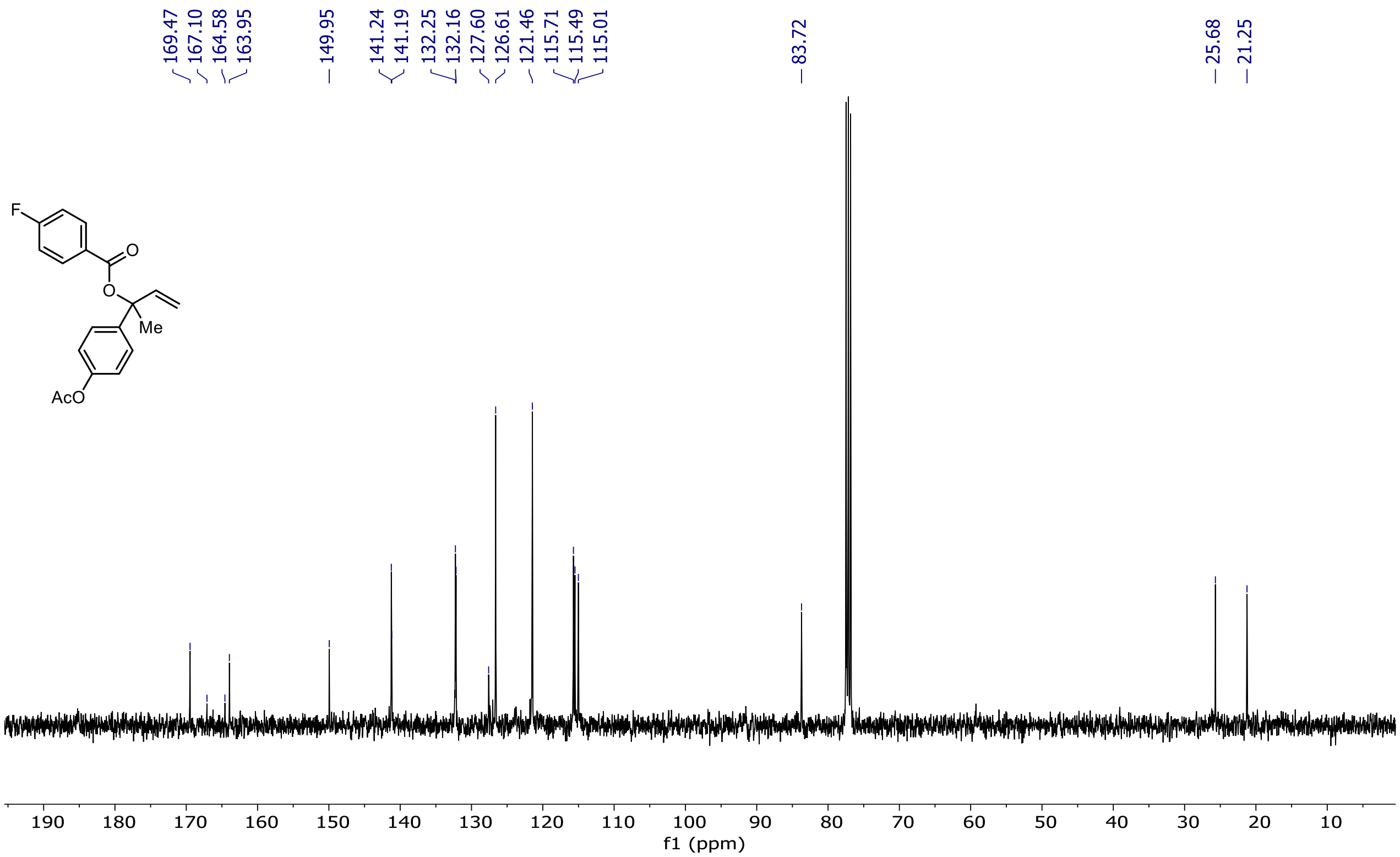

^{19}F NMR (282 MHz, $CDCl_3$, 25 °C) of 1y

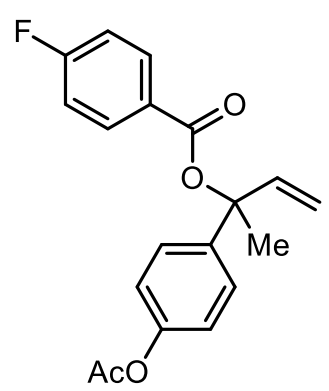

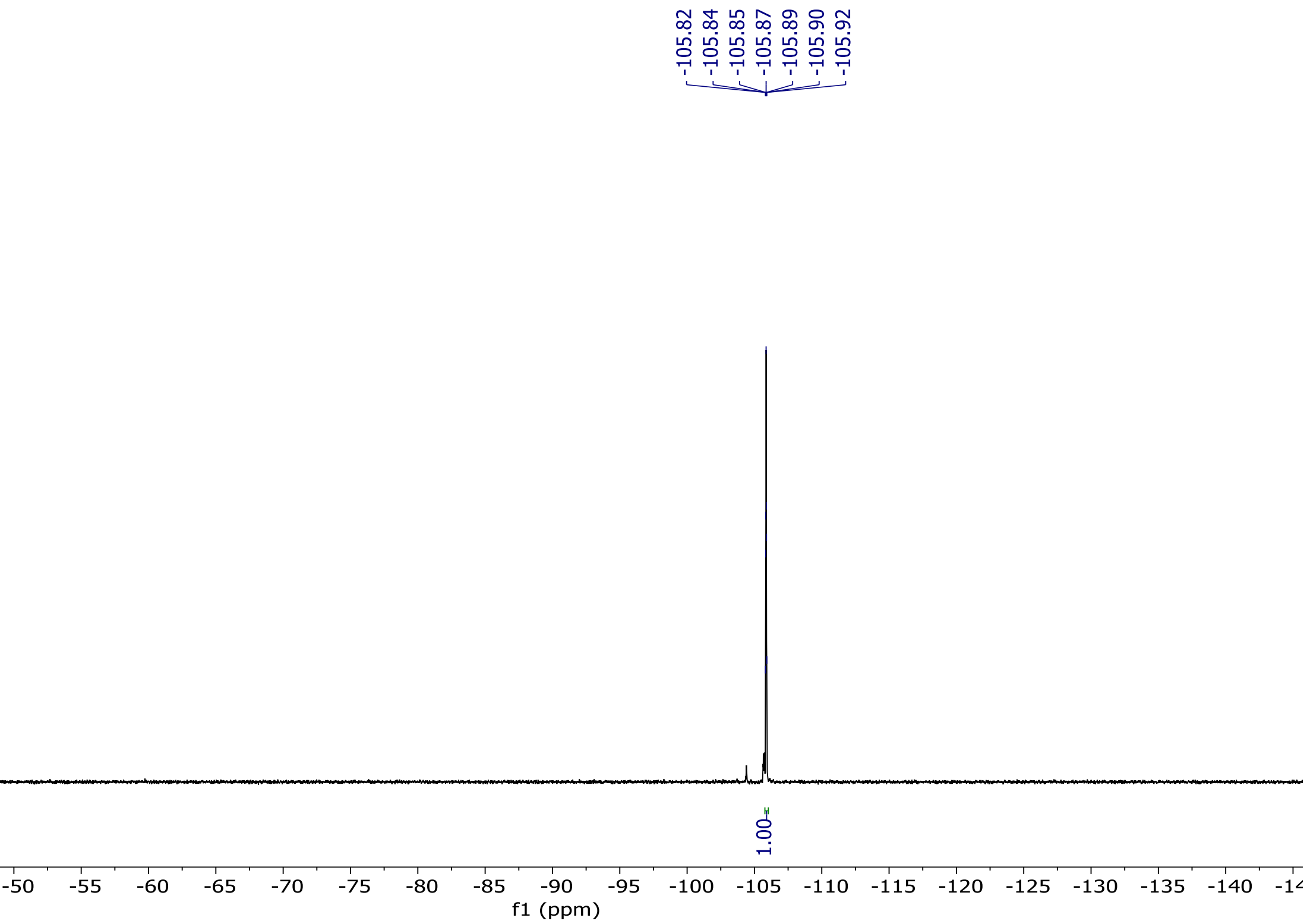

^{1}H NMR (400 MHz, CDCl$_3$, 25 ºC) of 1z

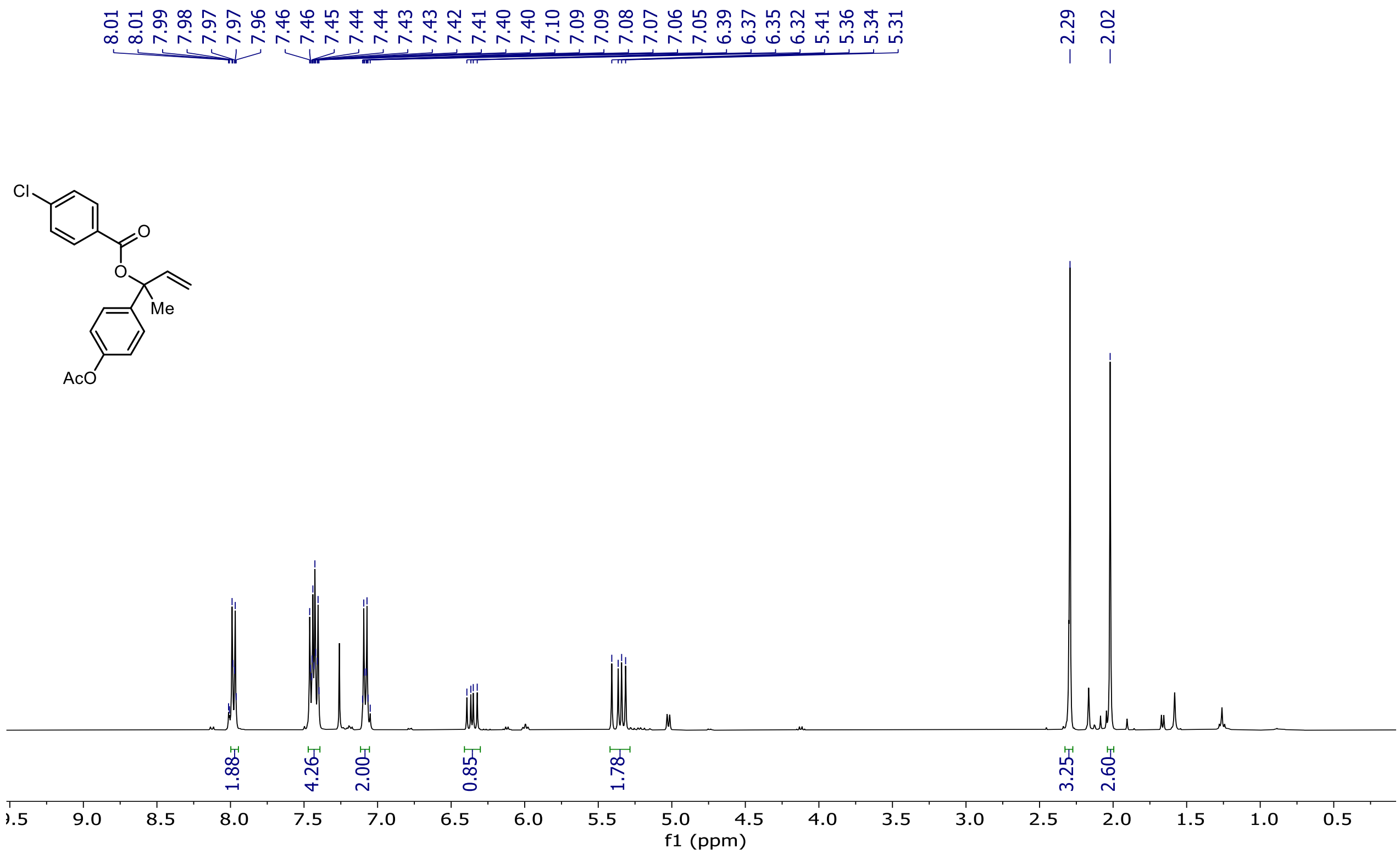

^{13}C NMR (101 MHz, CDCl$_3$, 25 ºC) of 1z

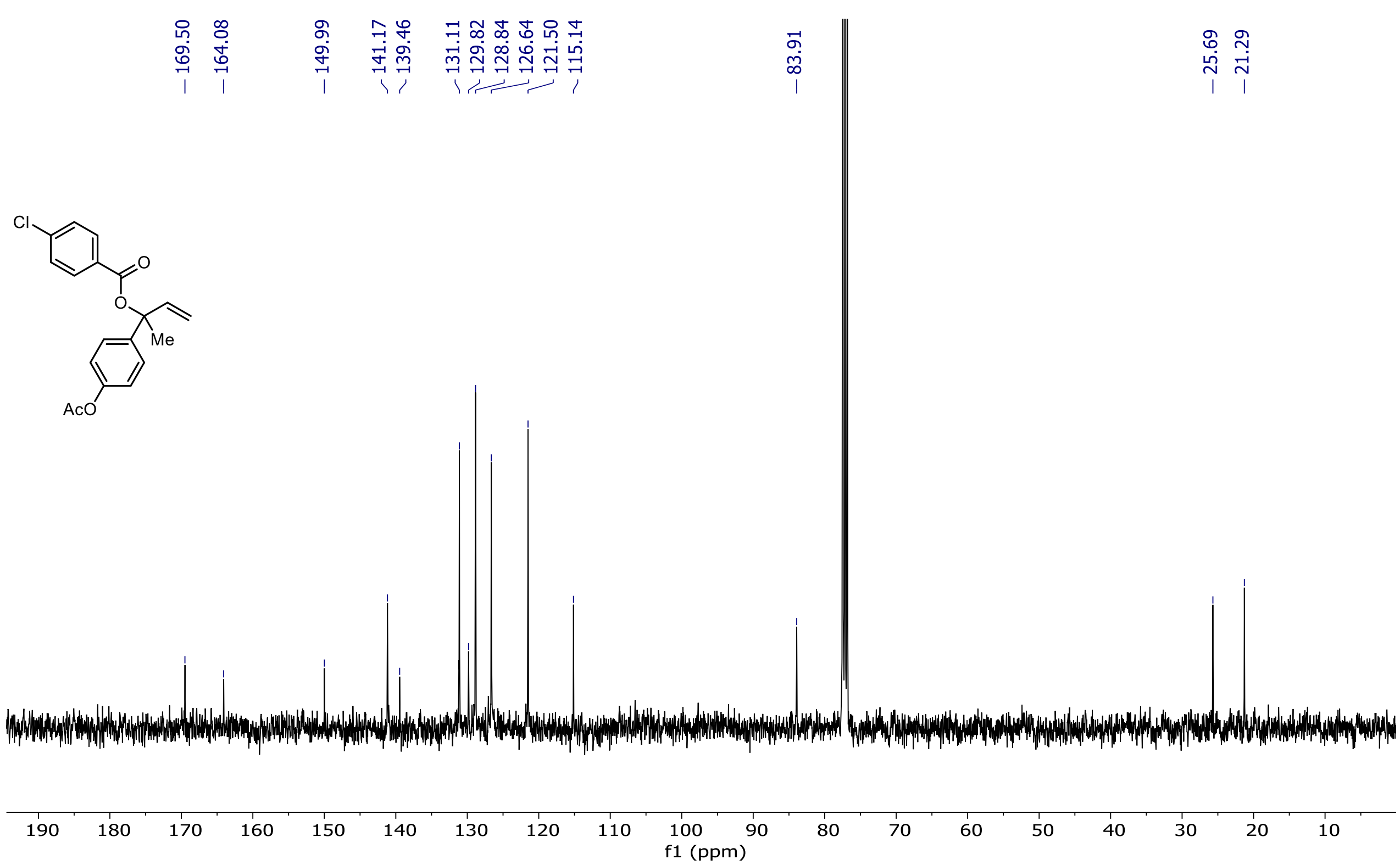

^{1}H NMR (300 MHz, $CDCl_3$, 25 ºC) of 1aa

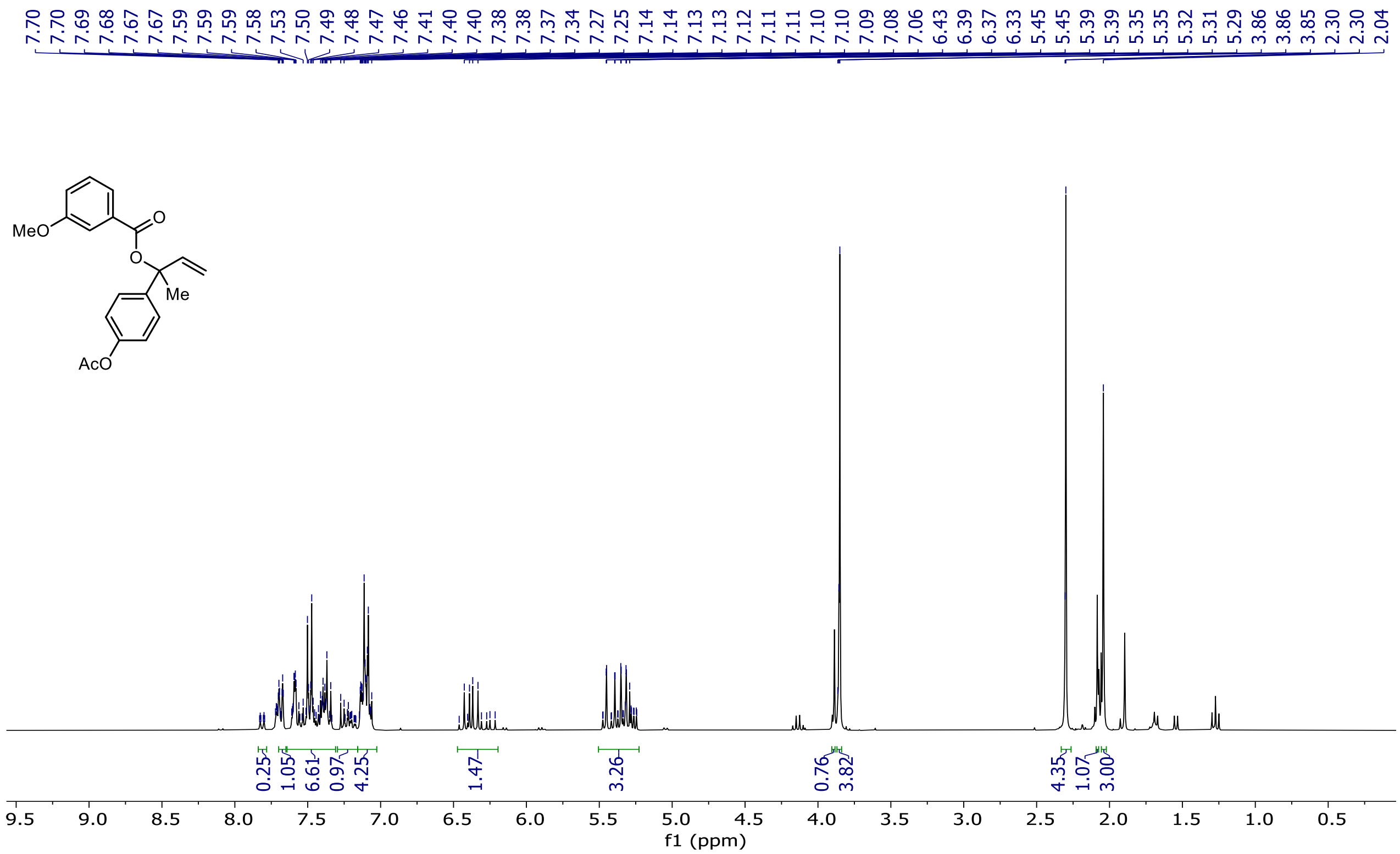

^{13}C NMR (75 MHz, $CDCl_3$, 25 ºC) of 1aa

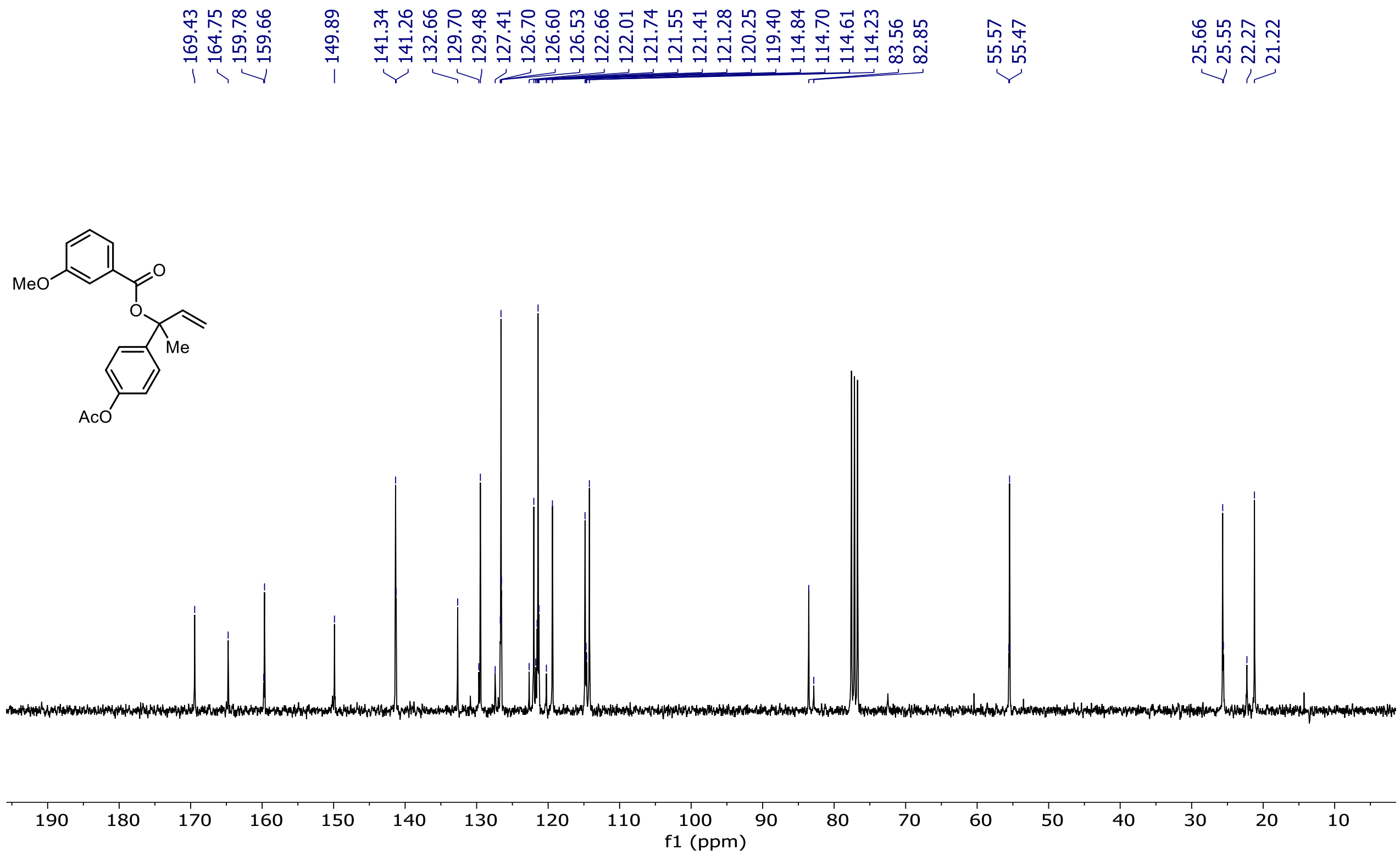

^{1}H NMR (400 MHz, $CDCl_3$, 25 °C) of 1ab

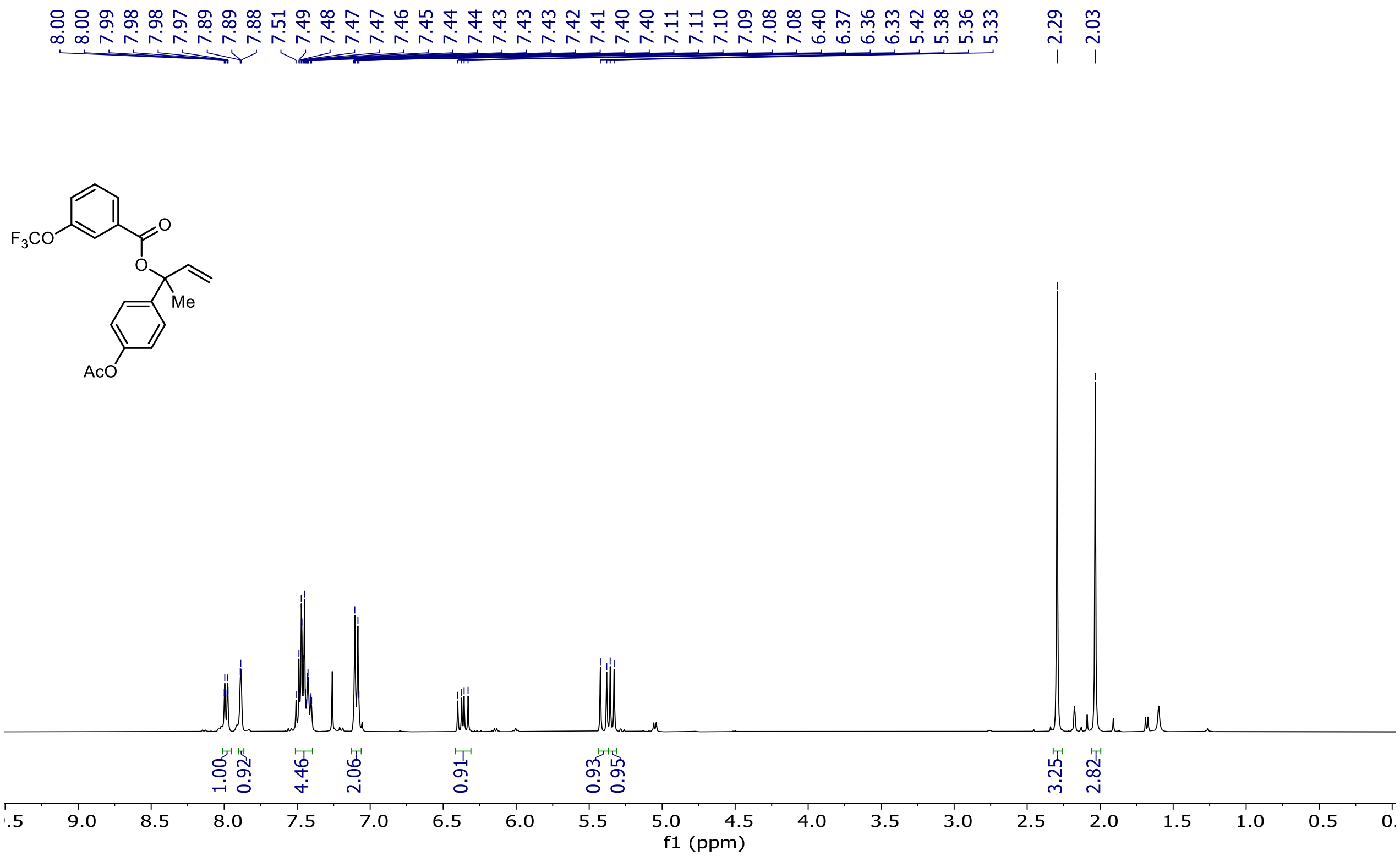

^{13}C NMR (101 MHz, $CDCl_3$, 25 °C) of 1ab

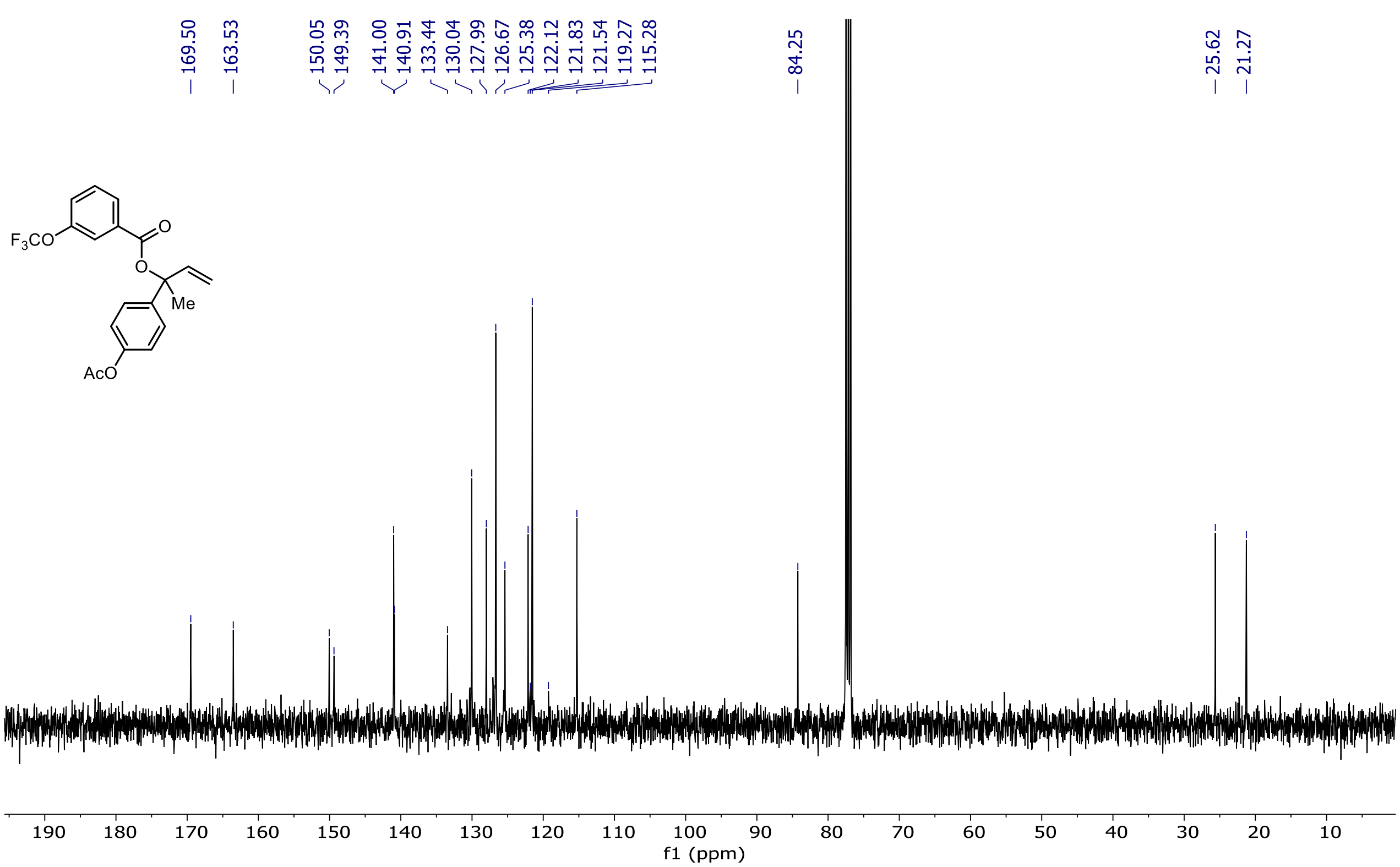

^{19}F NMR (376 MHz, $CDCl_3$, 25 ºC) of 1ab

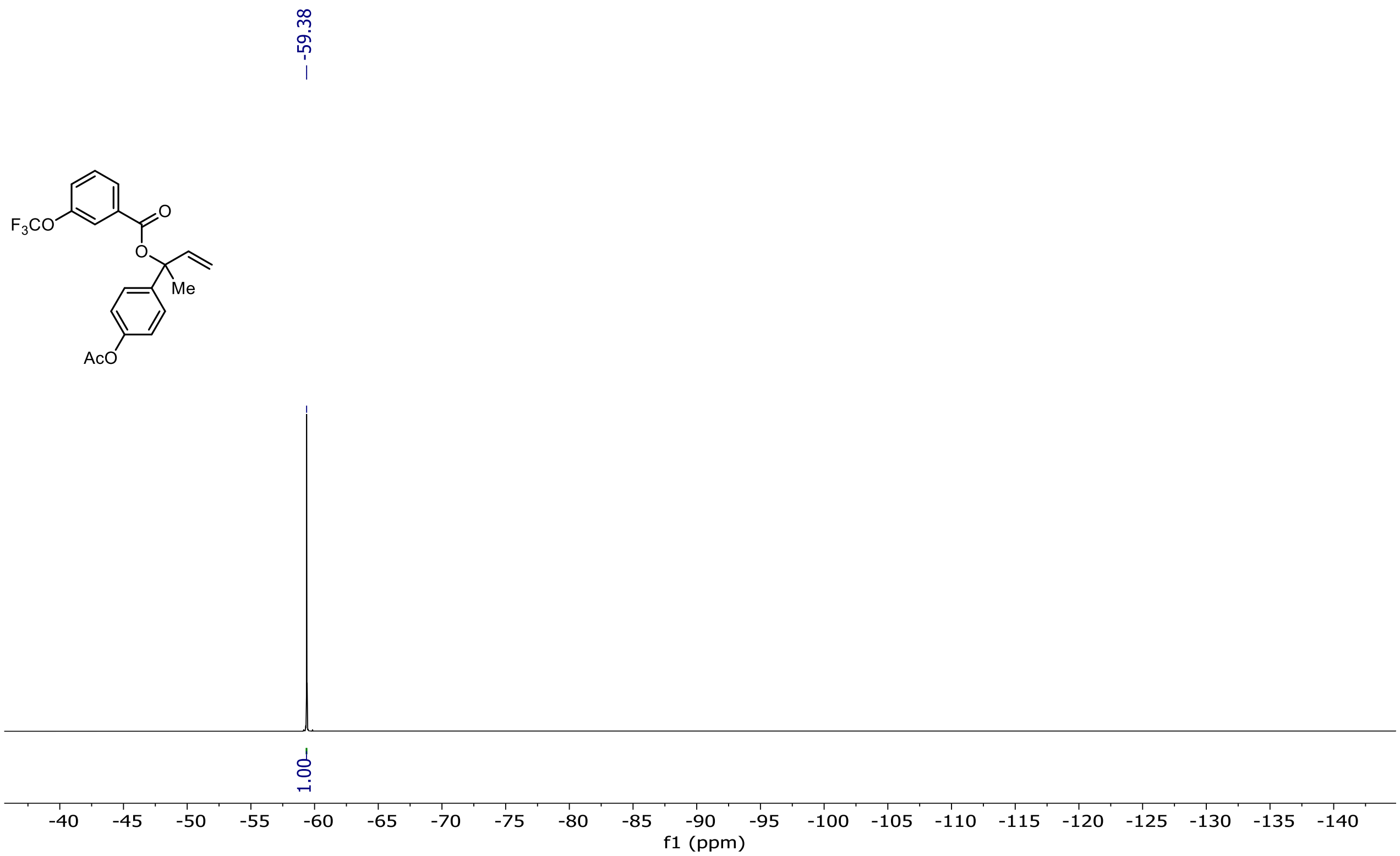

^{1}H NMR (800 MHz, $CDCl_3$, 25 ºC) of 1ac

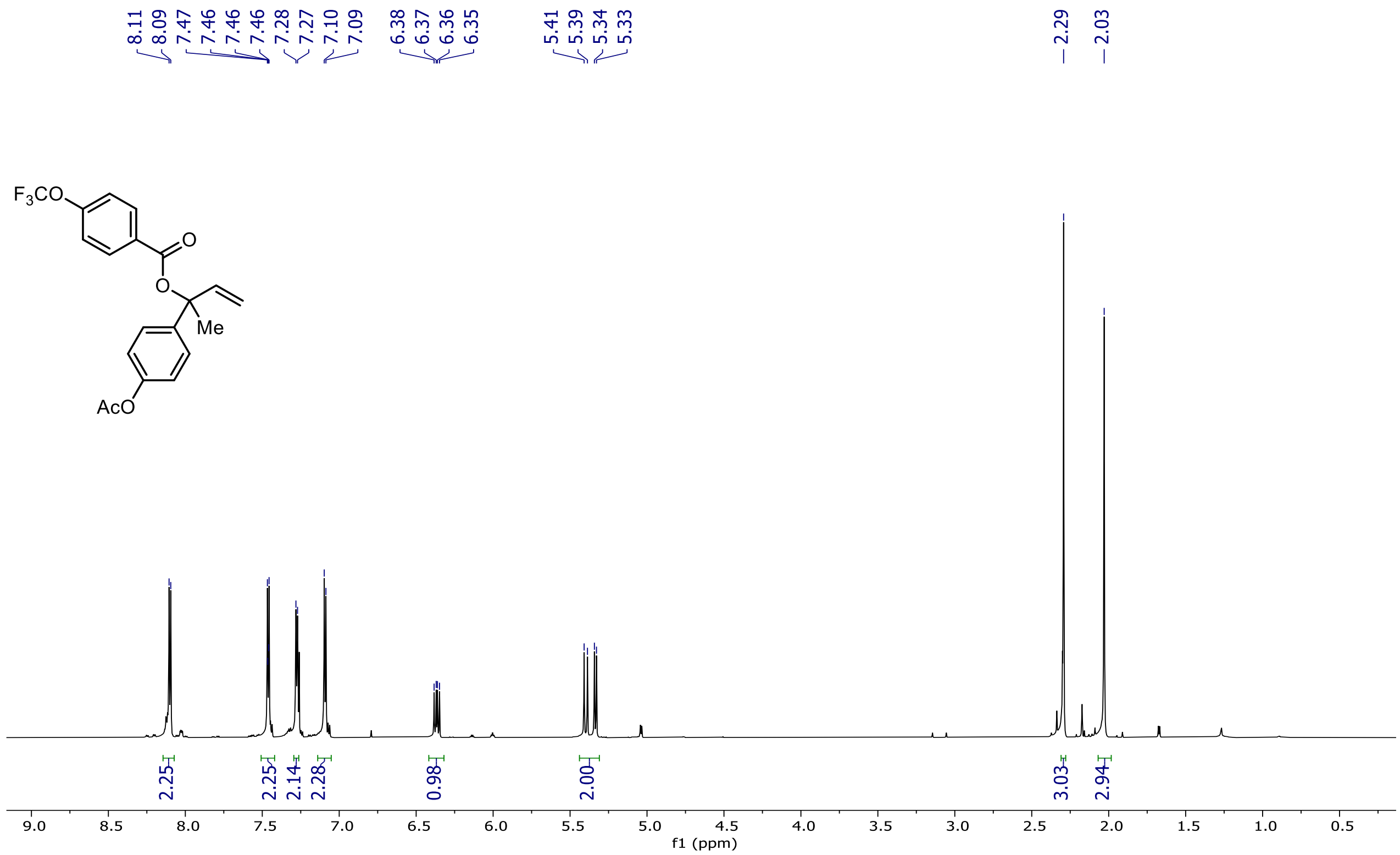

^{13}C NMR (201 MHz, $CDCl_3$, 25 ºC) of 1ac

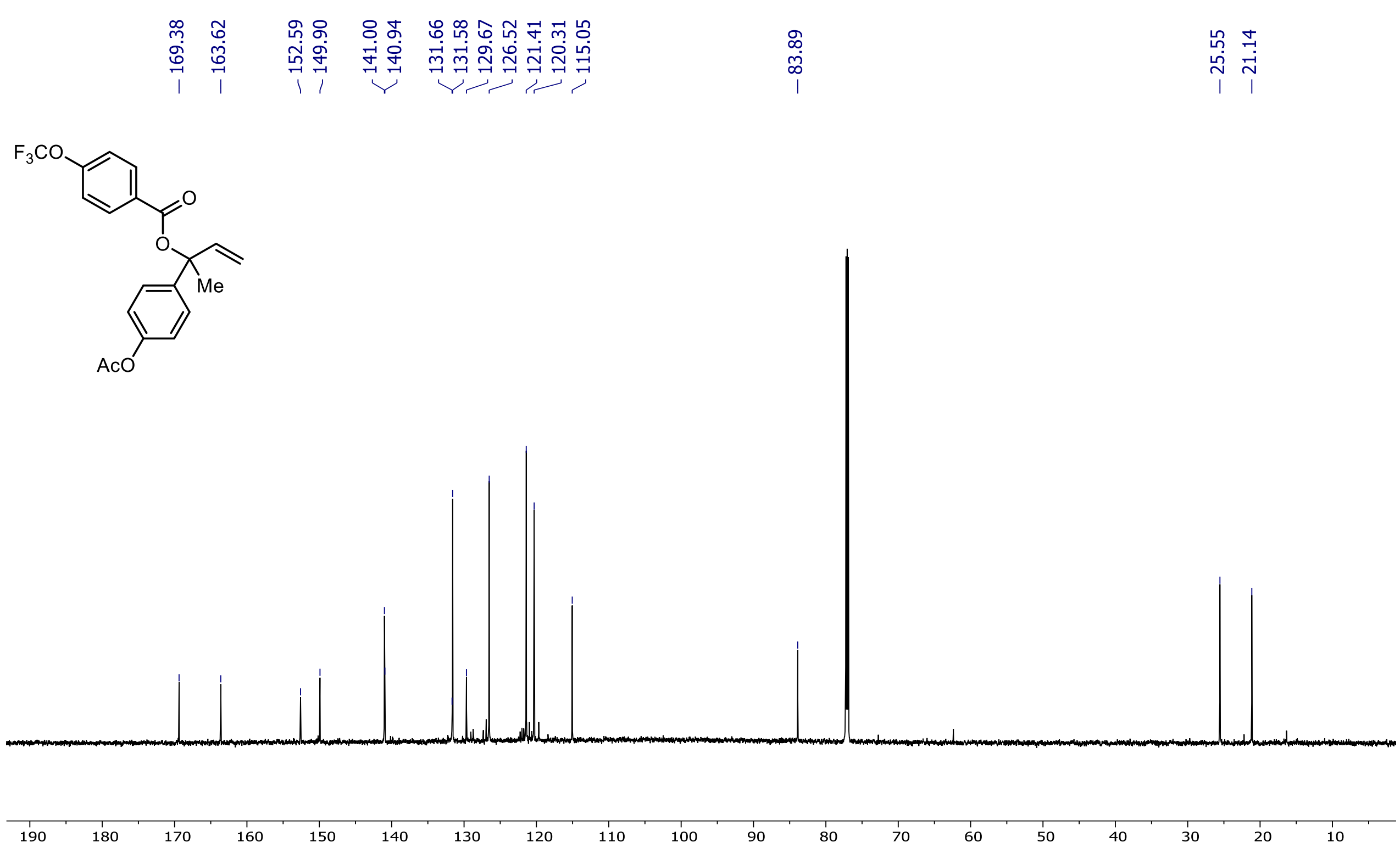

^{1}H NMR (800 MHz, CDCl$_3$, 25 ºC) of 1ad

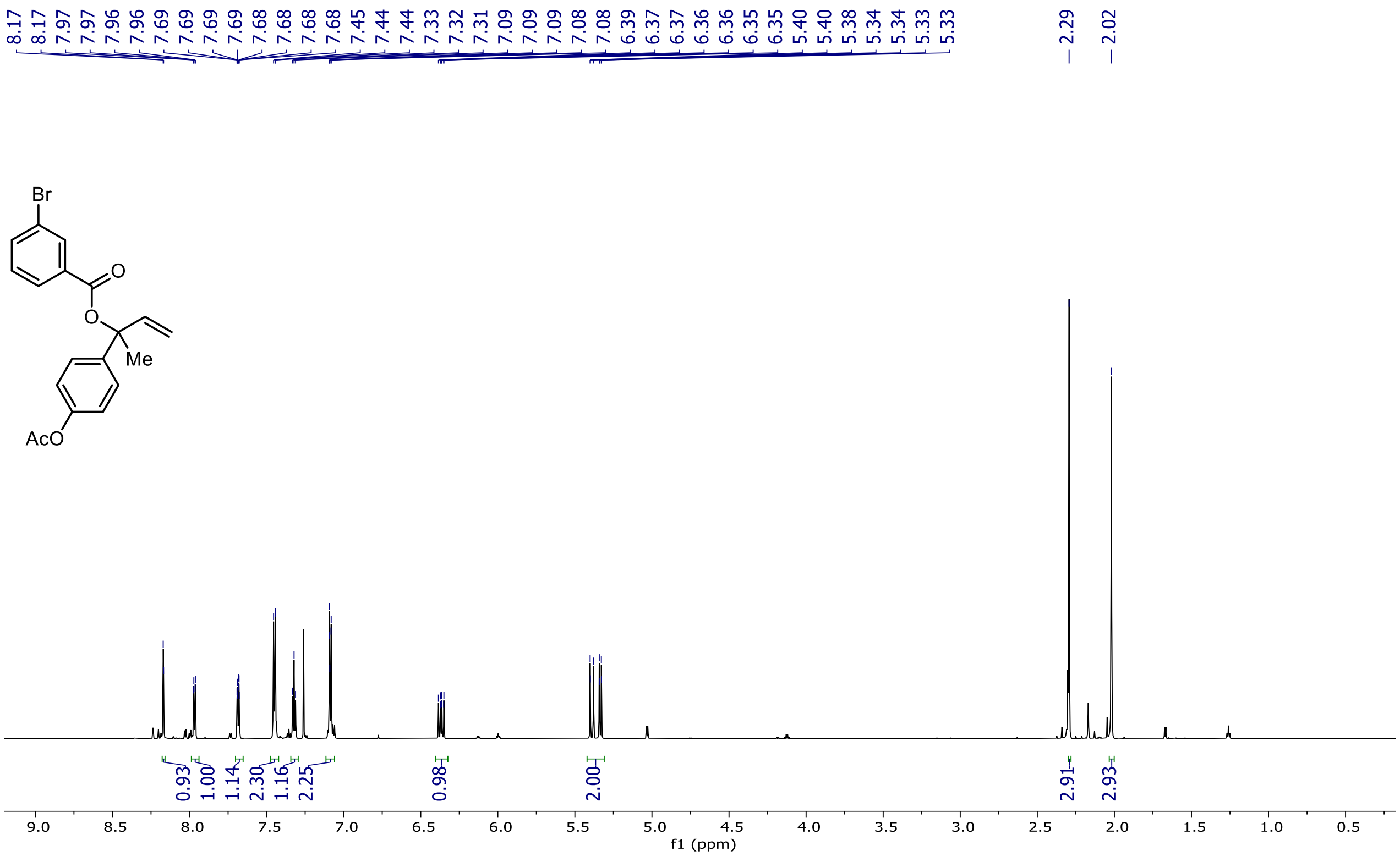

^{13}C NMR (201 MHz, CDCl$_3$, 25 ºC) of 1ad

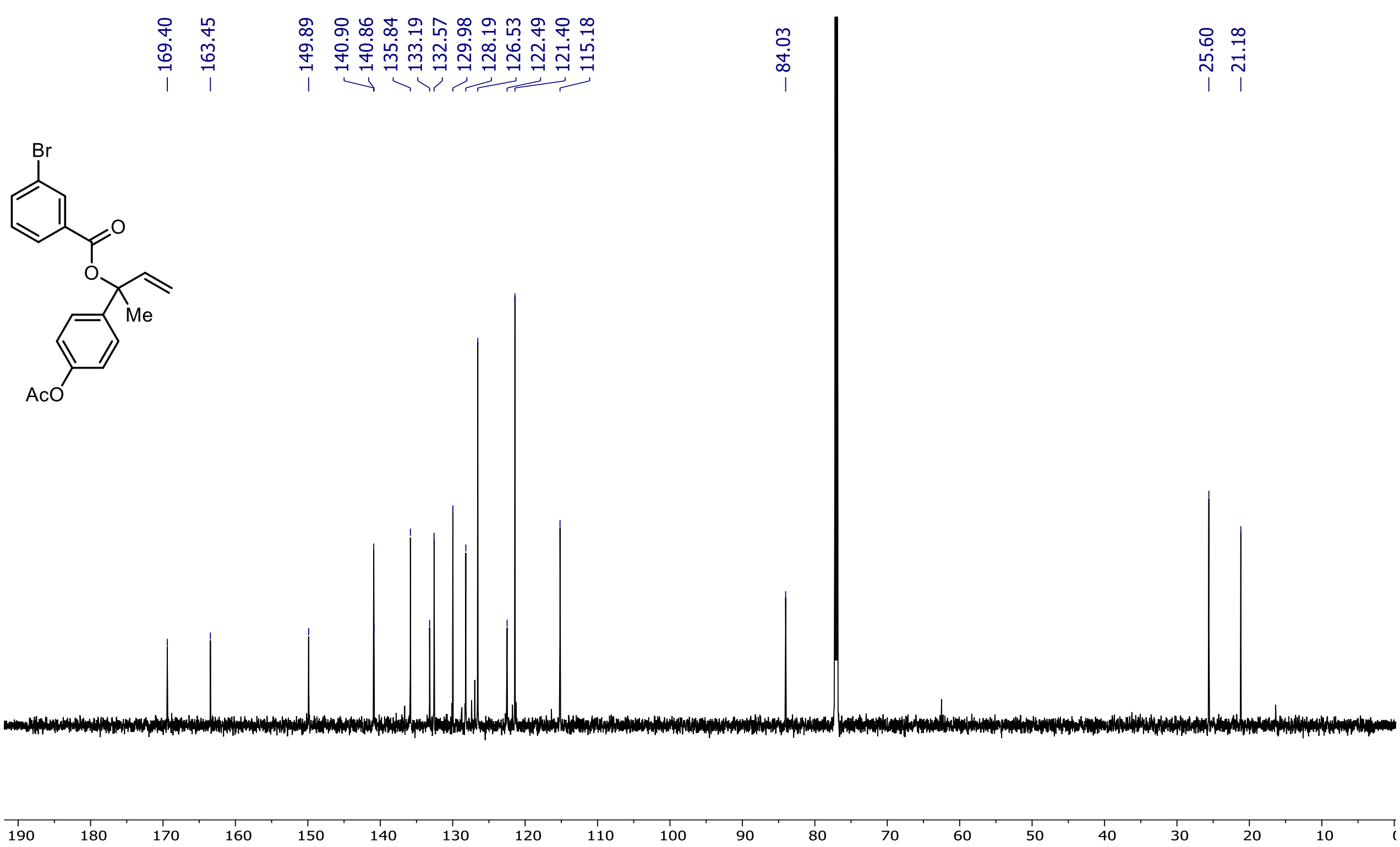

^{1}H NMR (400 MHz, CDCl$_3$, 25 ºC) of 1ae

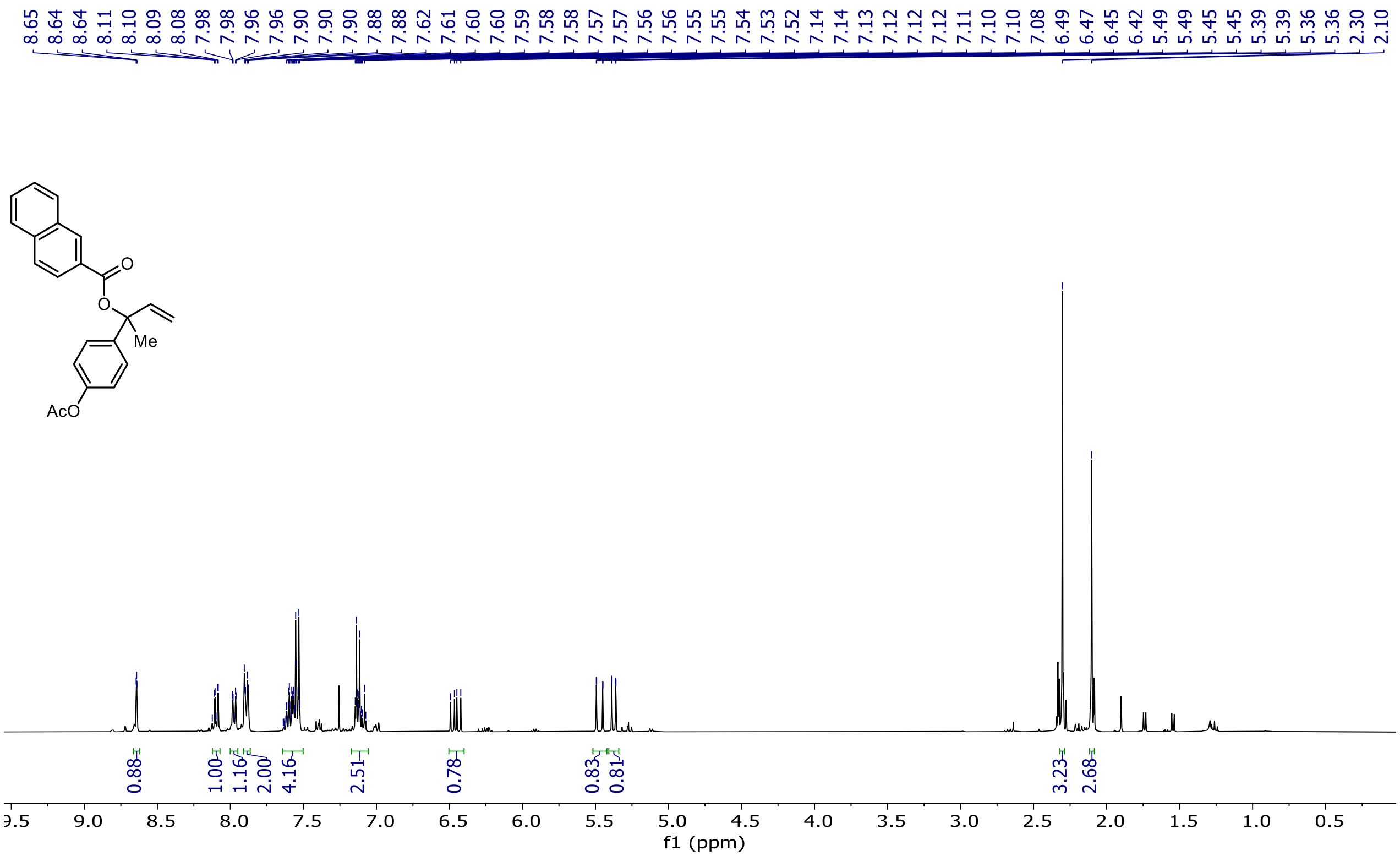

^{13}C NMR (75 MHz, CDCl$_3$, 25 ºC) of 1ae

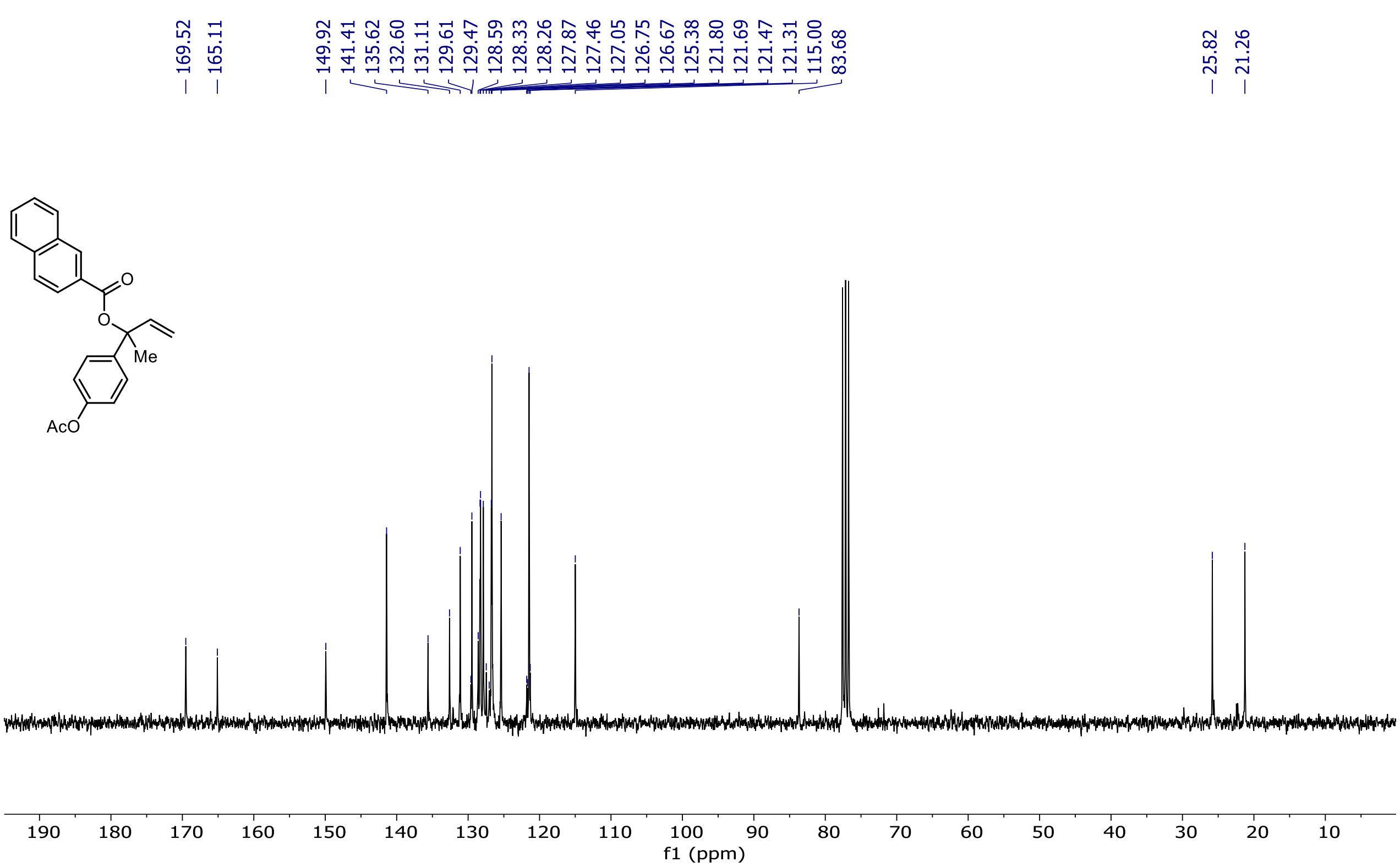

^{1}H NMR (400 MHz, $CDCl_3$, 25 ºC) of 1af

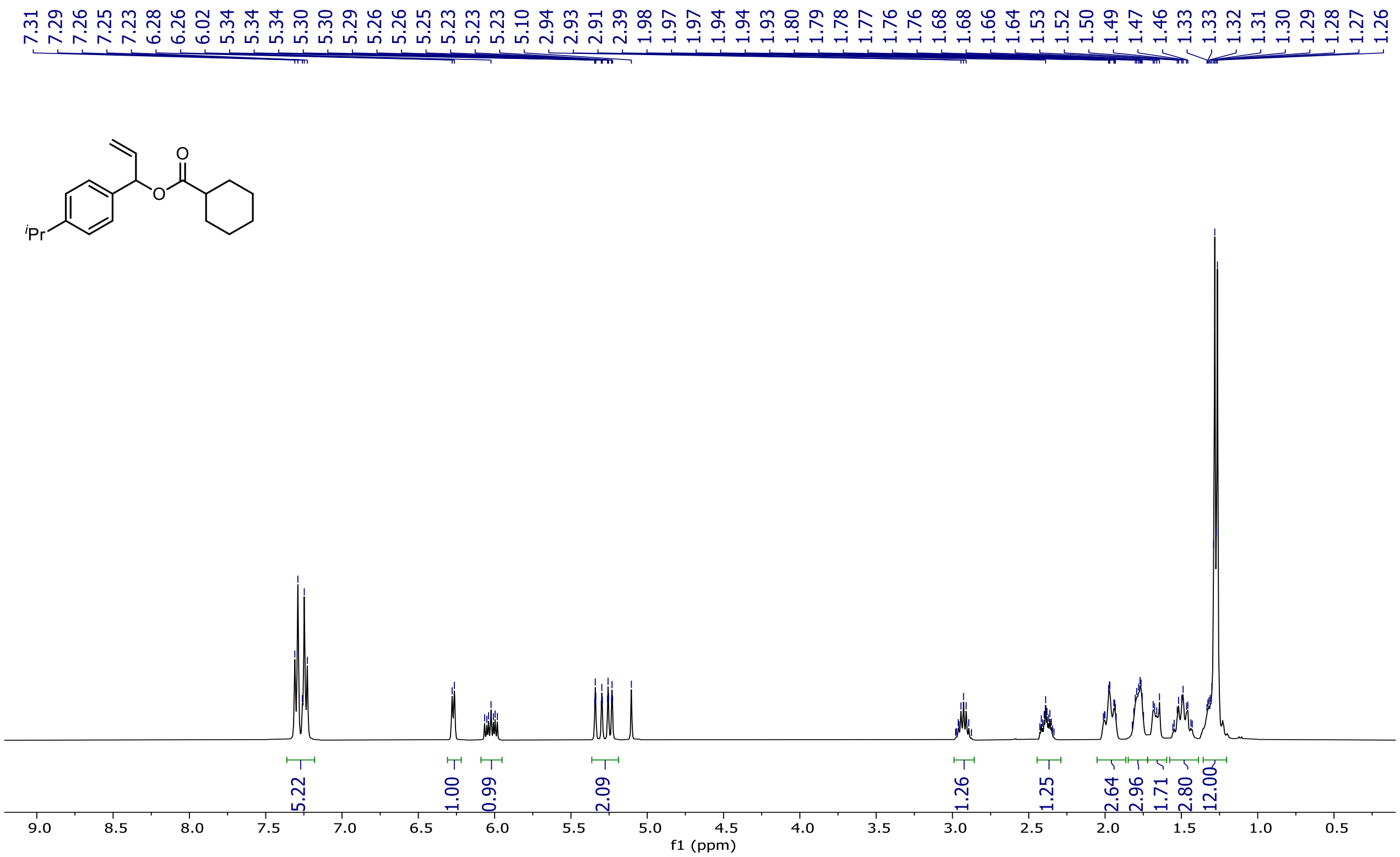

^{13}C NMR (101 MHz, $CDCl_3$, 25 ºC) of 1af

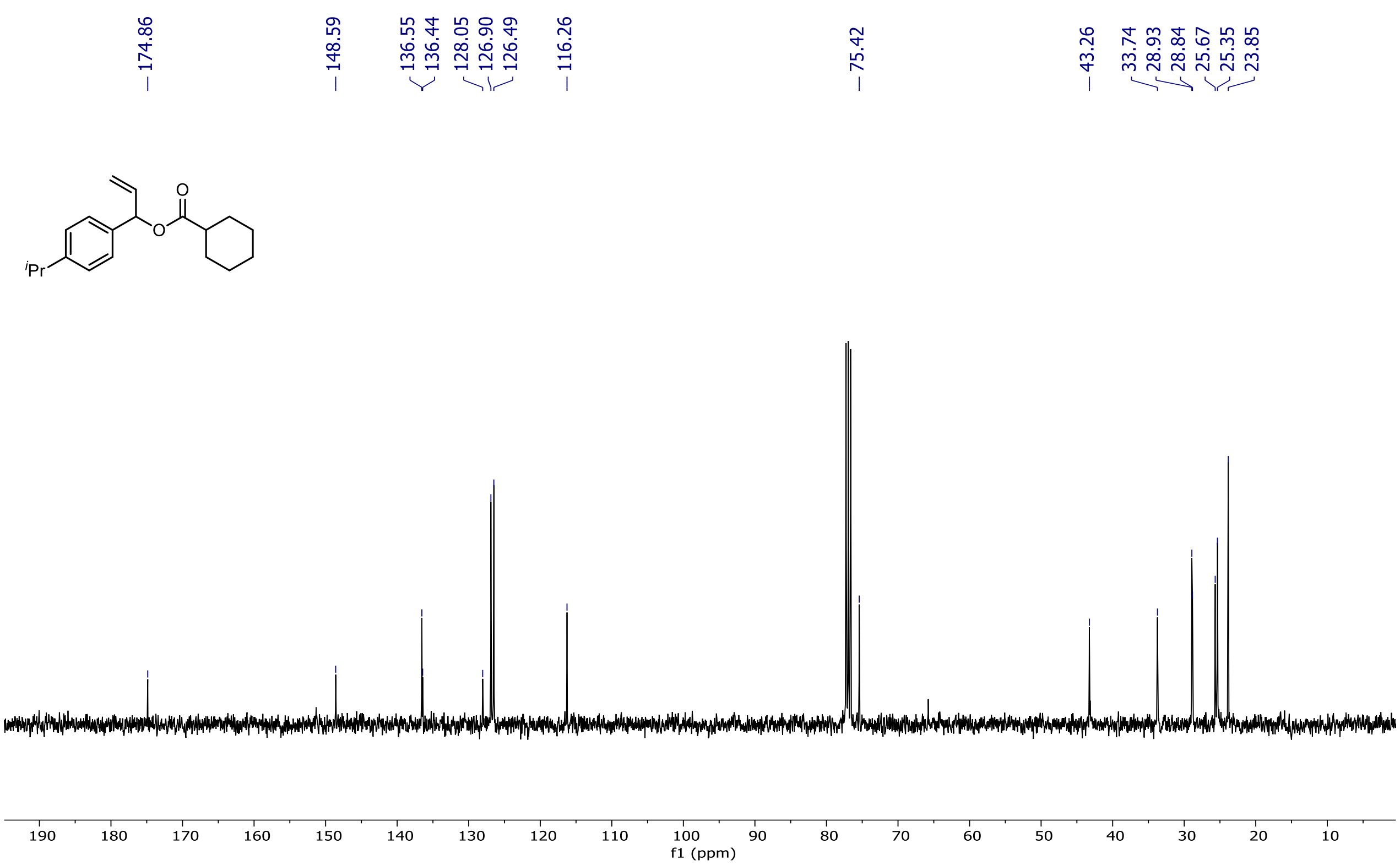

^{1}H NMR (400 MHz, $CDCl_3$, 25 ºC) of 1ag

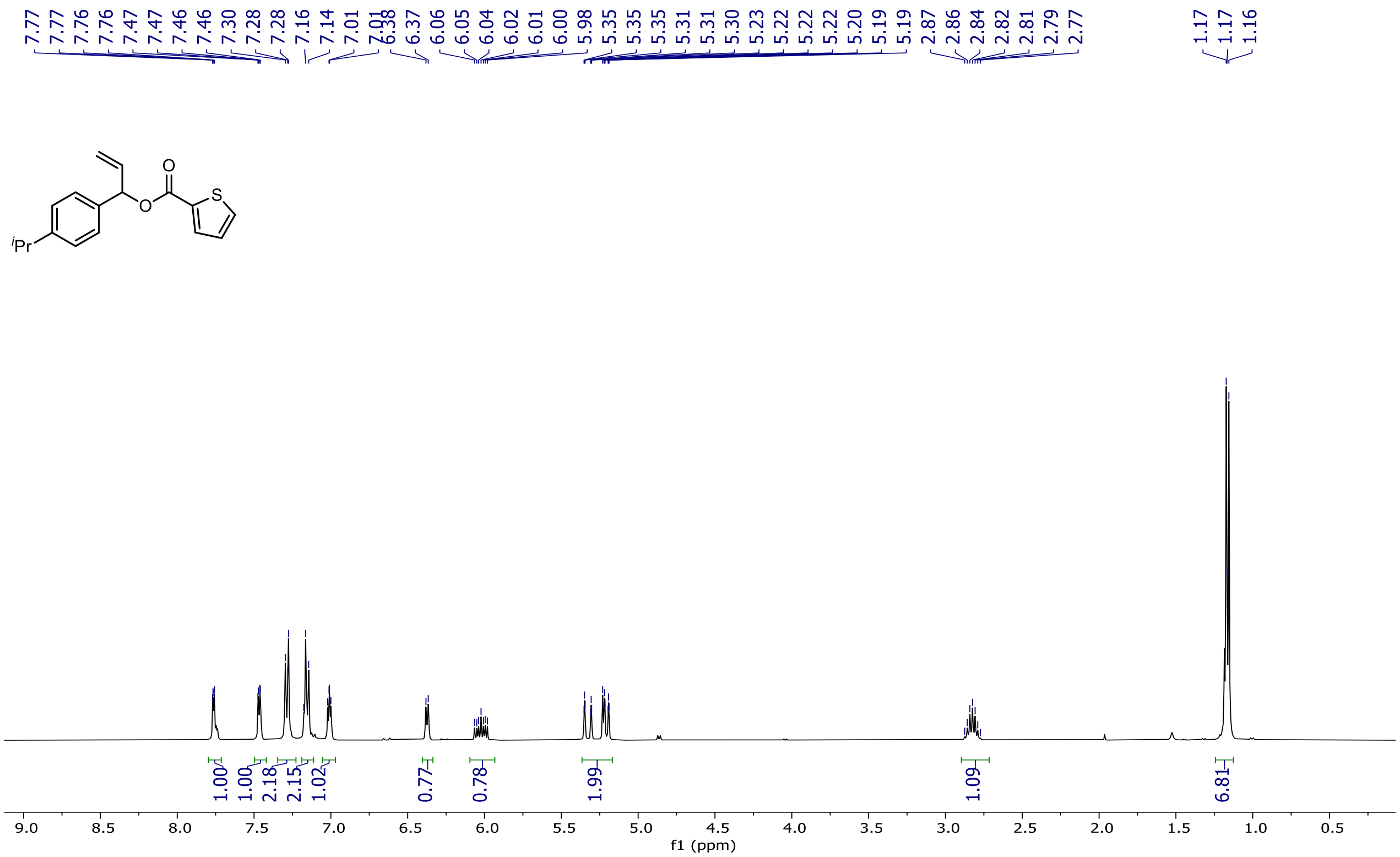

^{13}C NMR (101 MHz, $CDCl_3$, 25 ºC) of 1ag

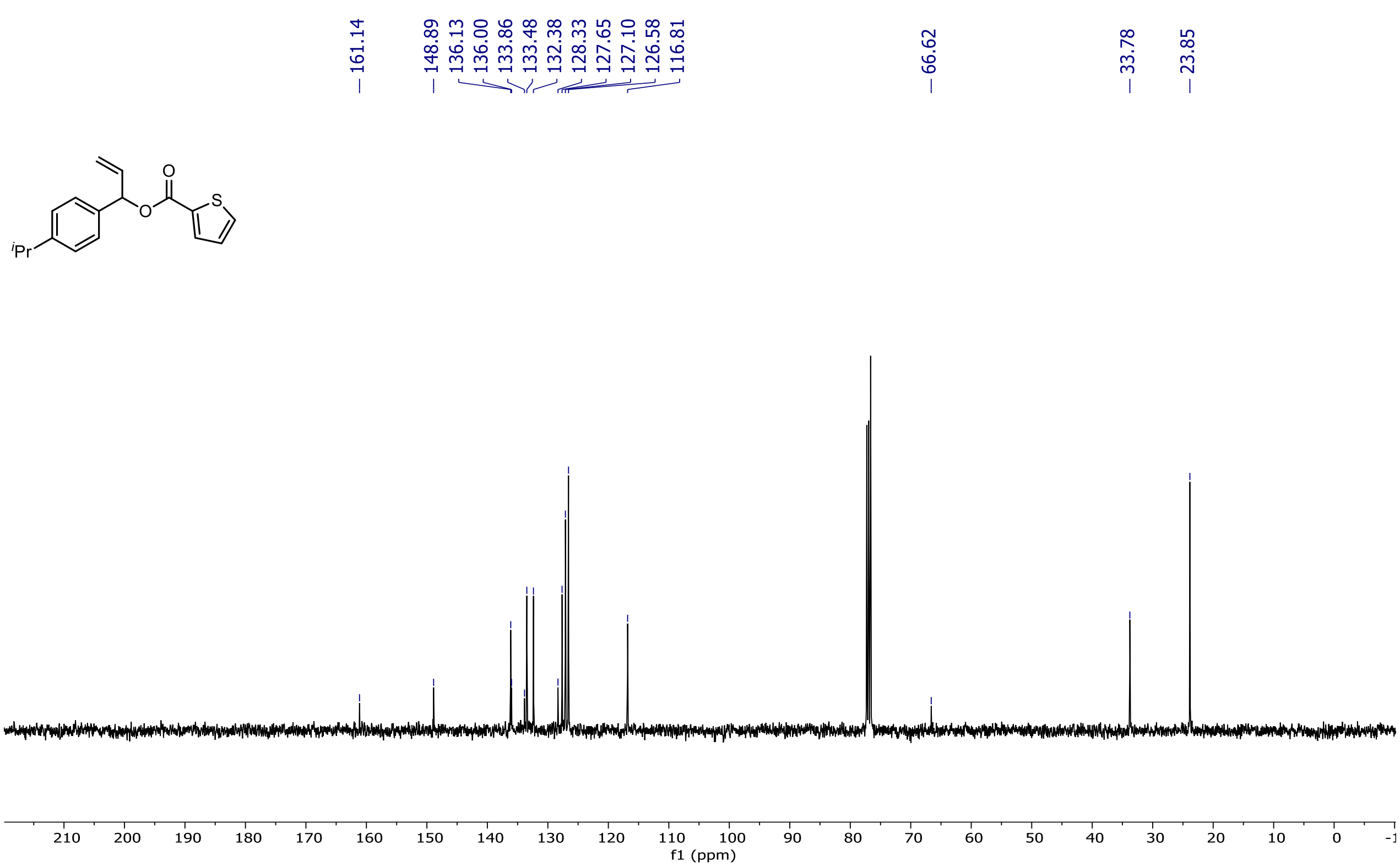

^{1}H NMR (400 MHz, $CDCl_3$, 25 ºC) of 1ah

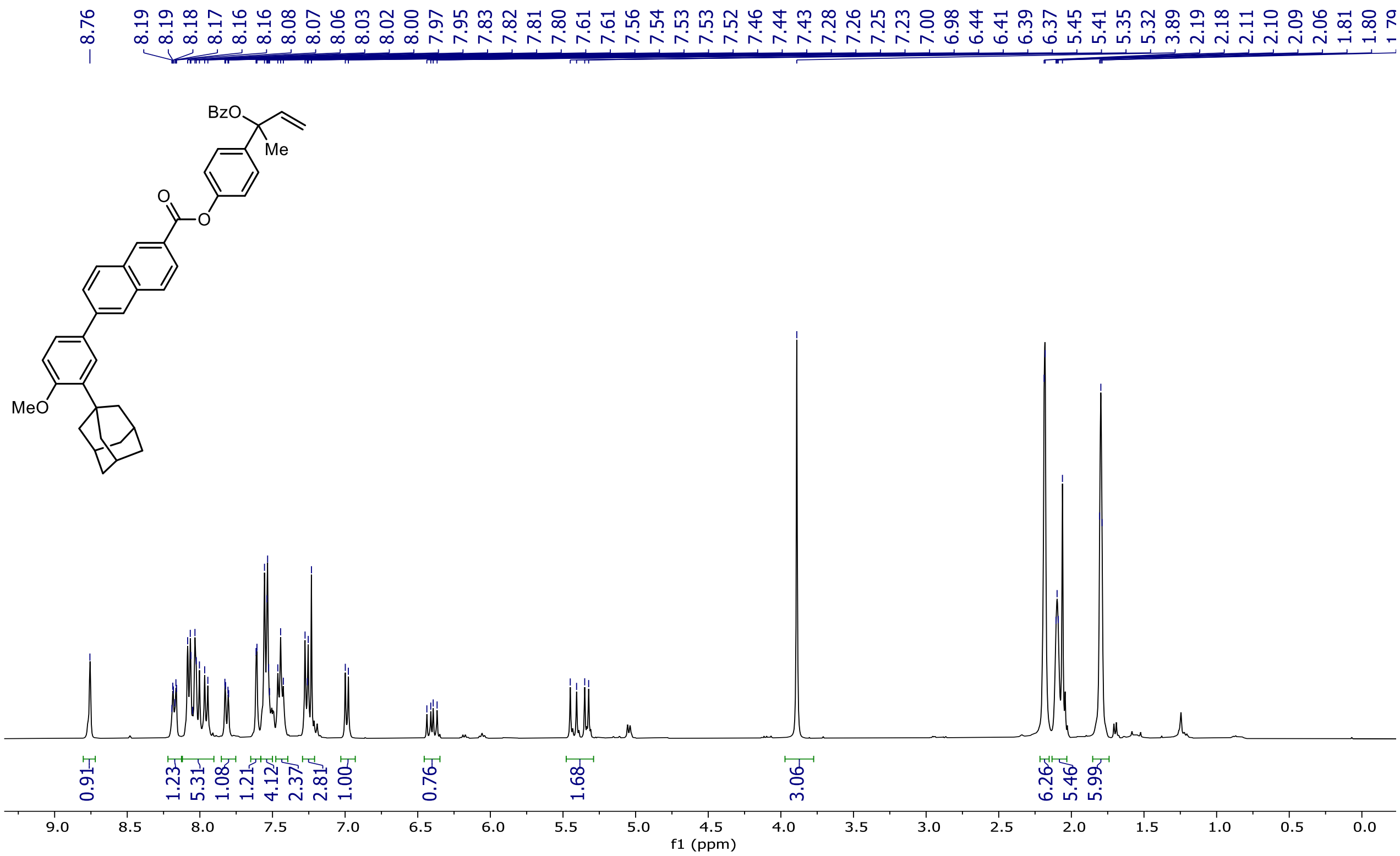

^{13}C NMR (101 MHz, $CDCl_3$, 25 ºC) of 1ah

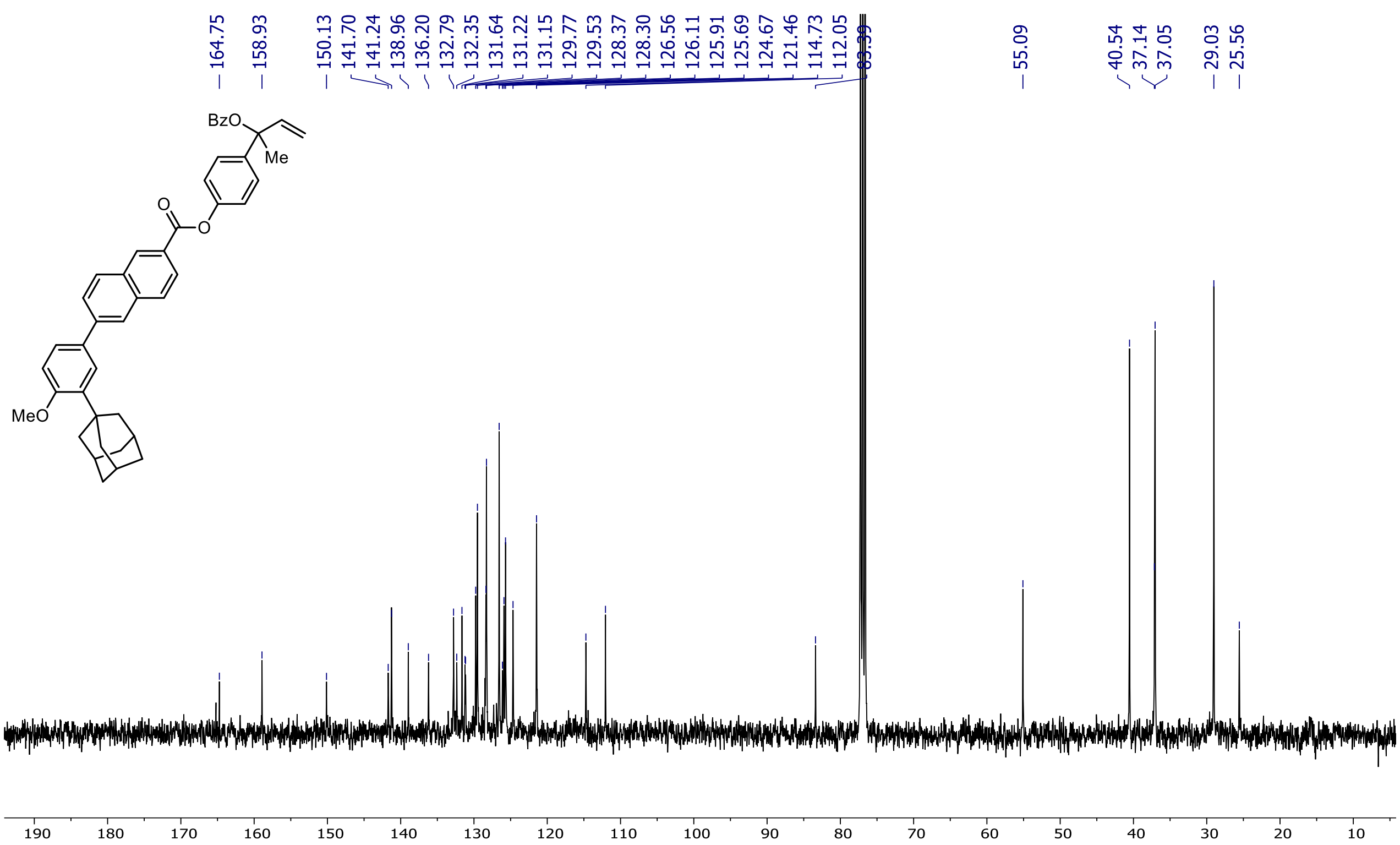

^{1}H NMR (400 MHz, CDCl$_3$, 25 ºC) of 1ai

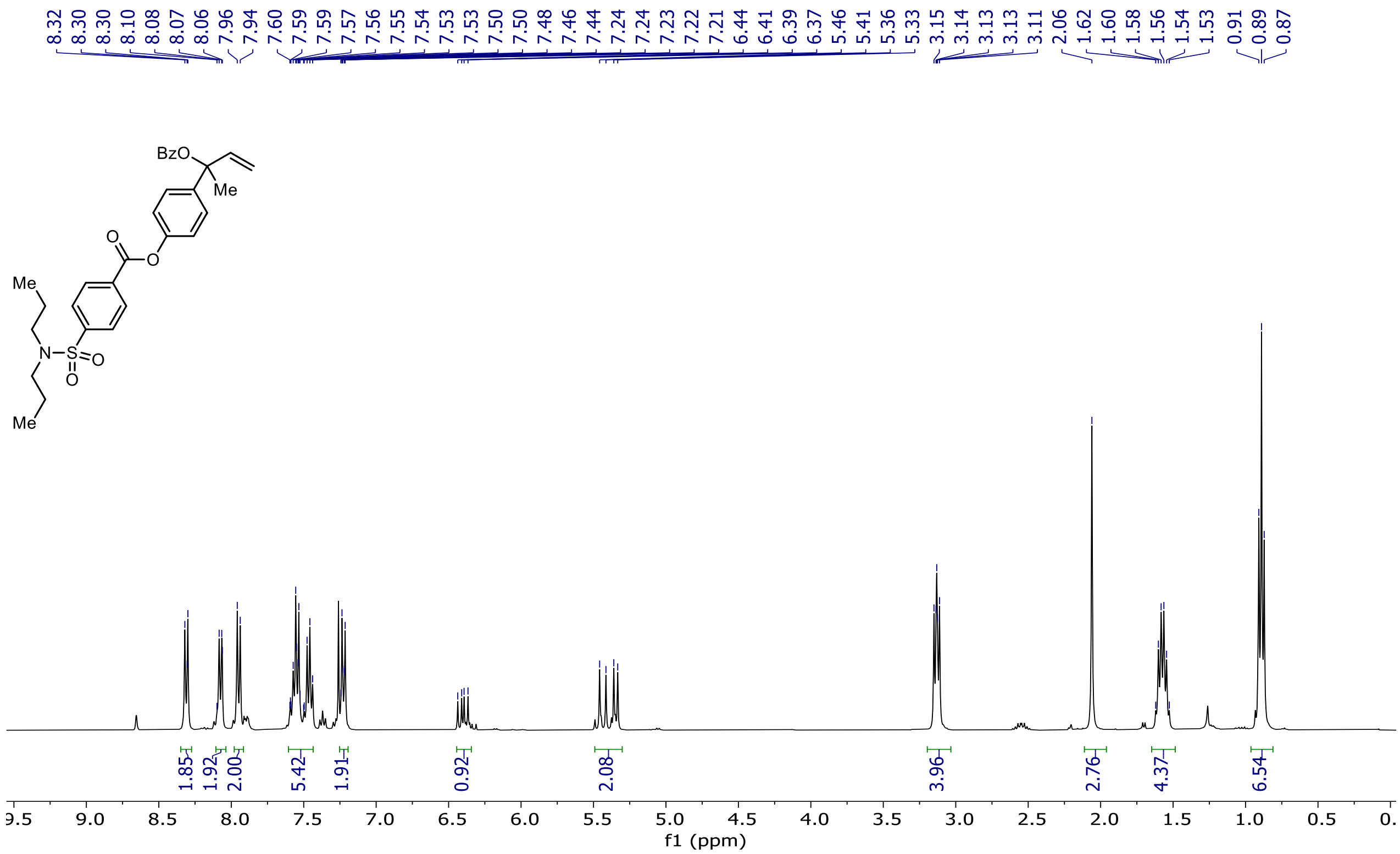

^{13}C NMR (101 MHz, CDCl$_3$, 25 ºC) of 1ai

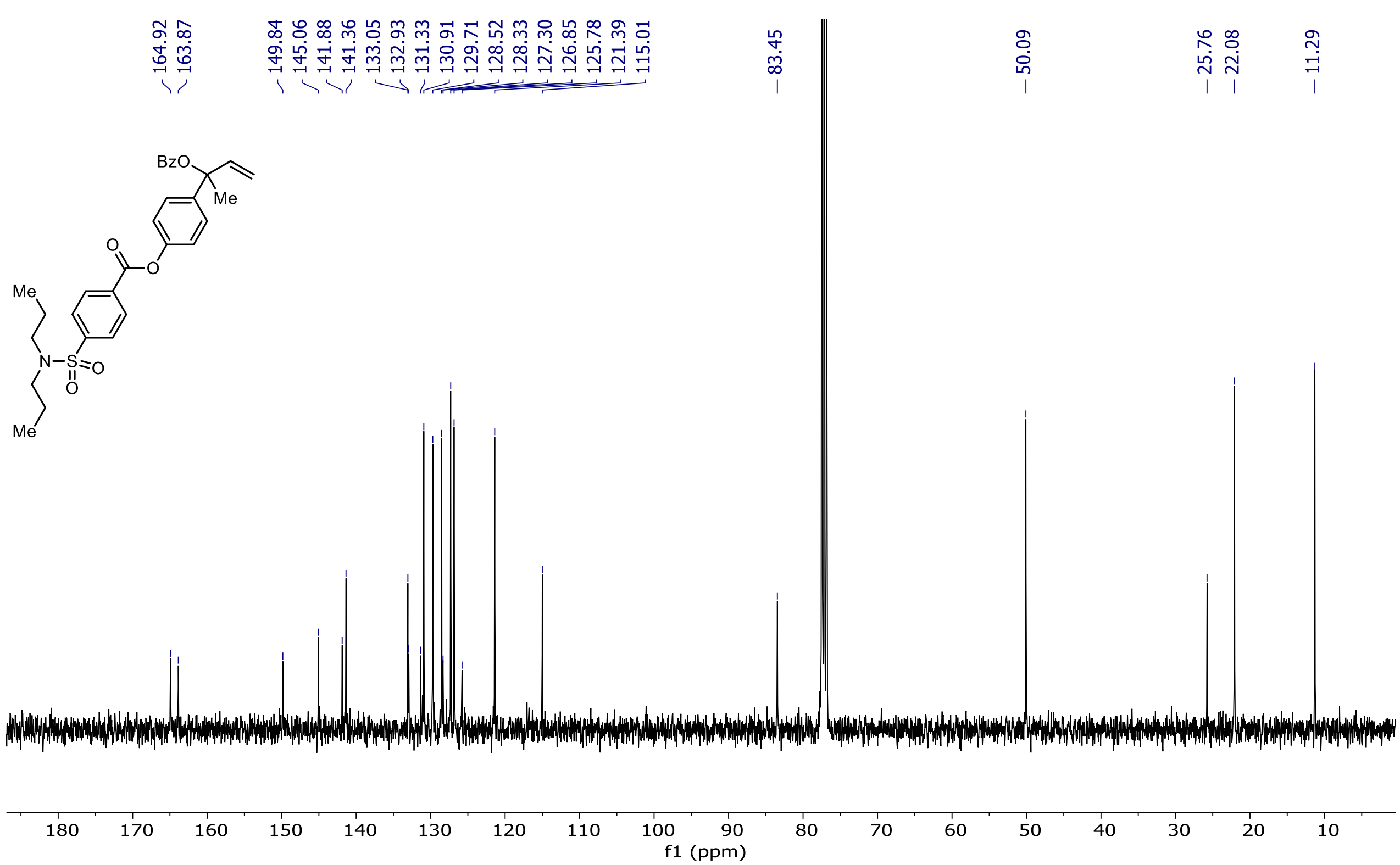

^{1}H NMR (300 MHz, $CDCl_3$, 25 ºC) of 1aj

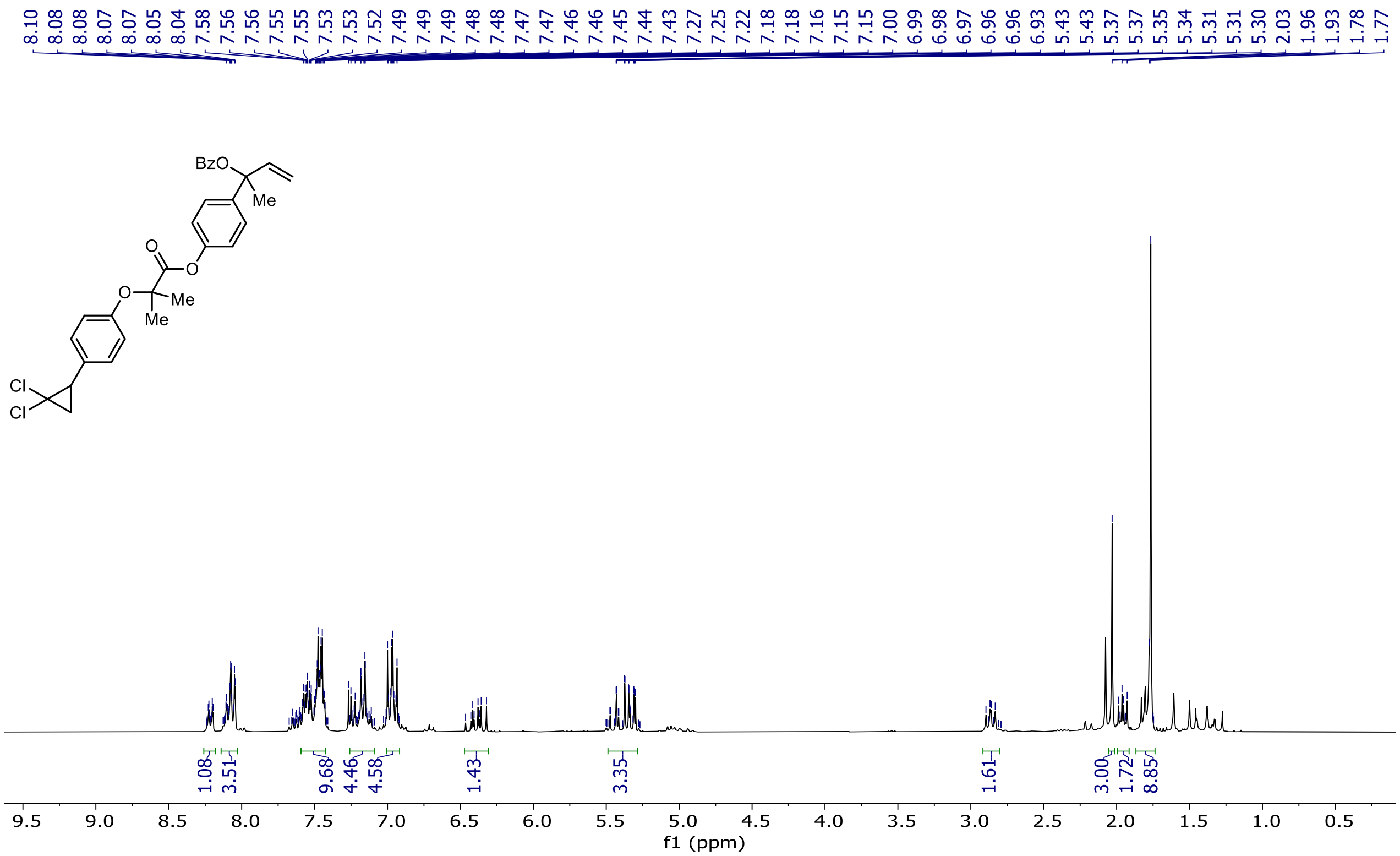

^{13}C NMR (75 MHz, $CDCl_3$, 25 ºC) of 1aj

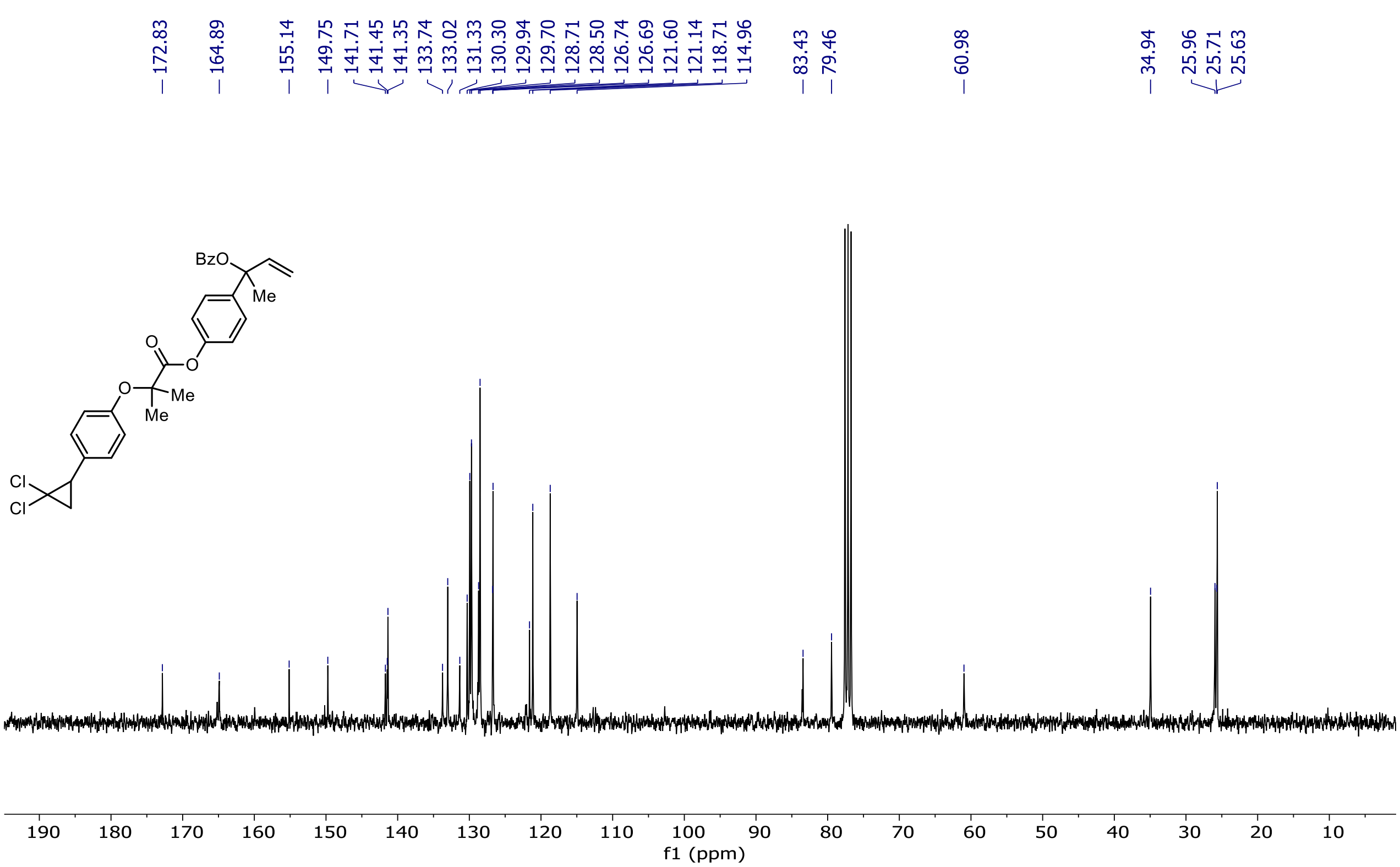

^{1}H NMR (400 MHz, $CDCl_3$, 25 ºC) of 1ak

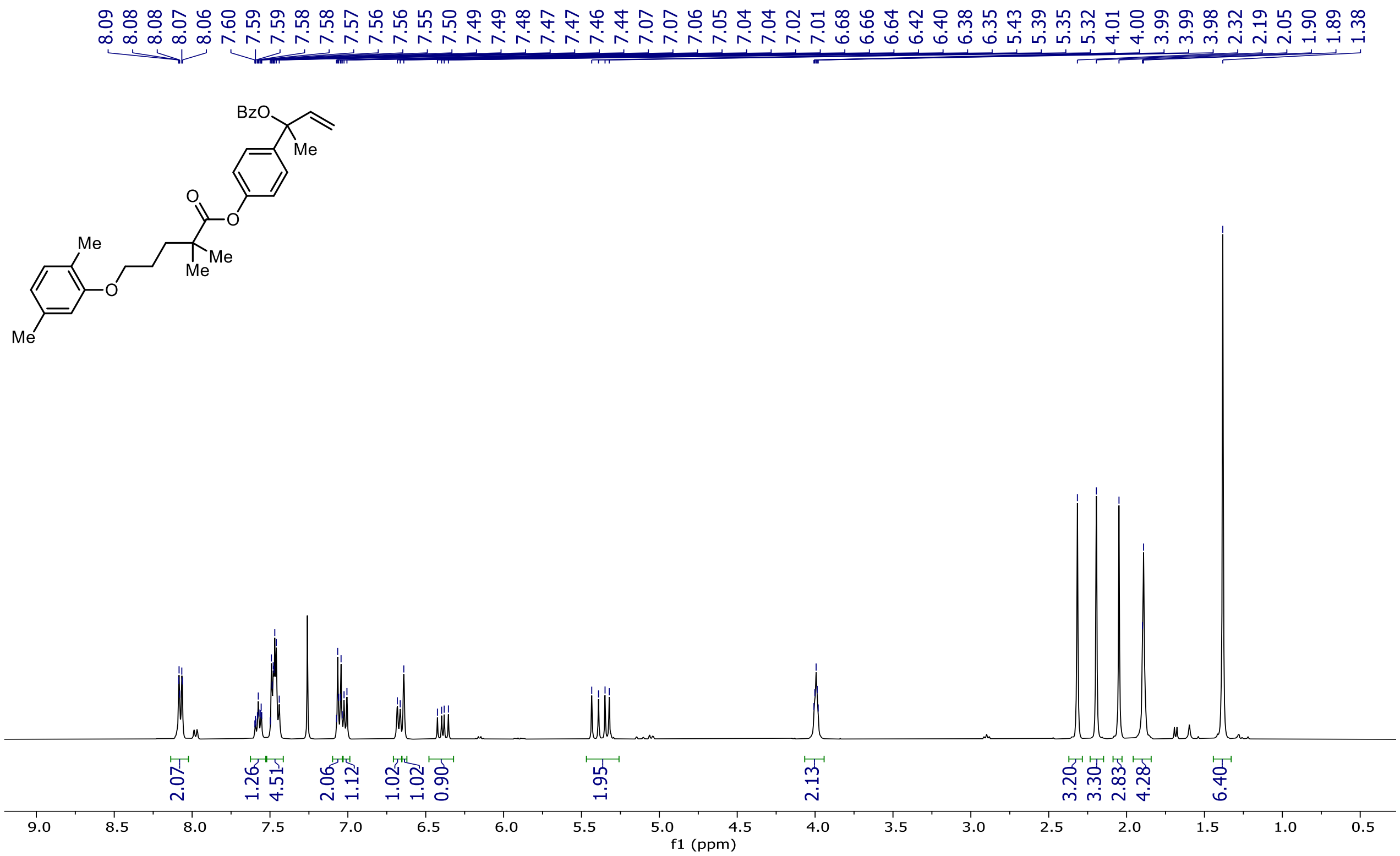

^{13}C NMR (101 MHz, $CDCl_3$, 25 ºC) of 1ak

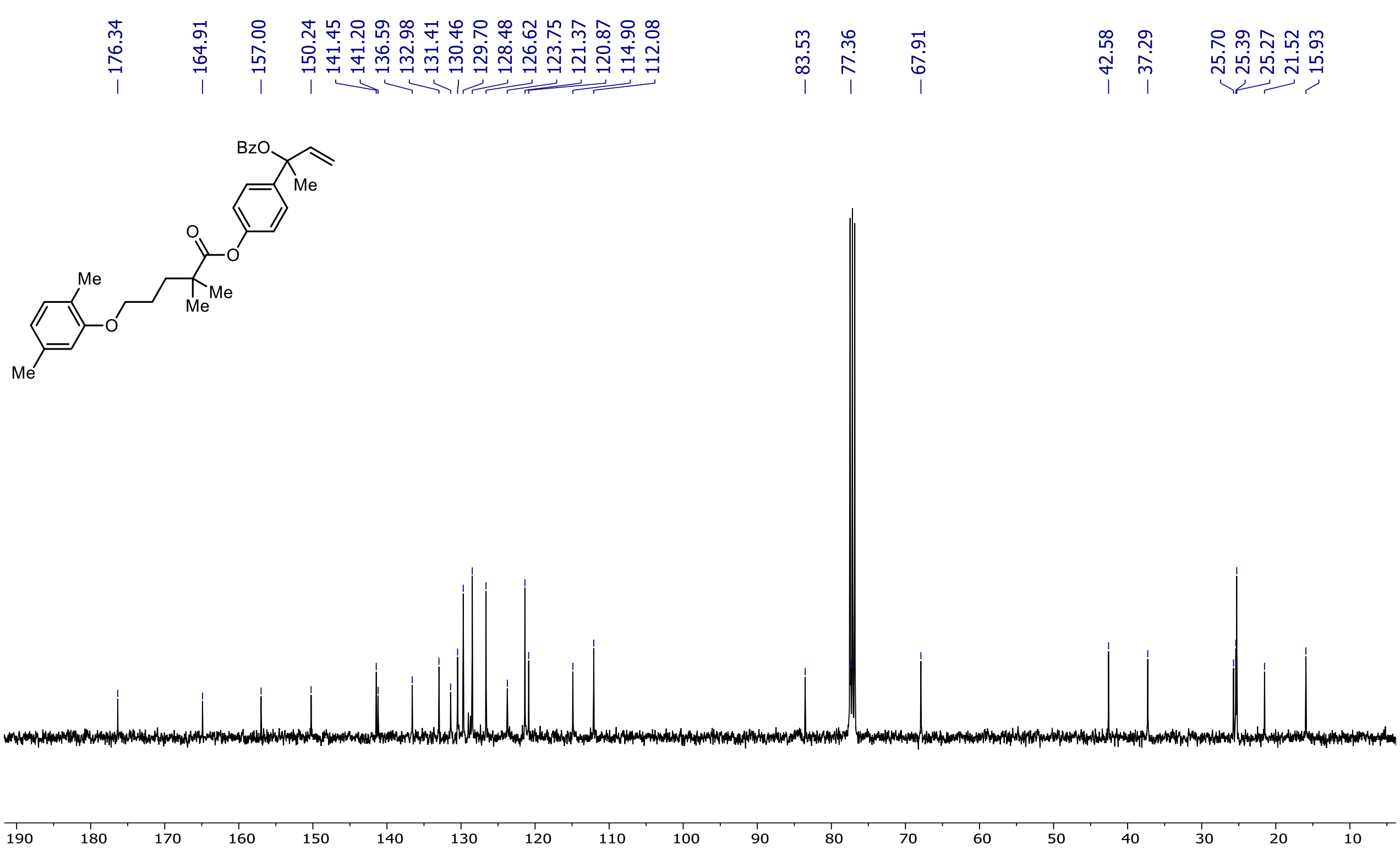

^{1}H NMR (400 MHz, CDCl$_3$, 25 ºC) of 1al

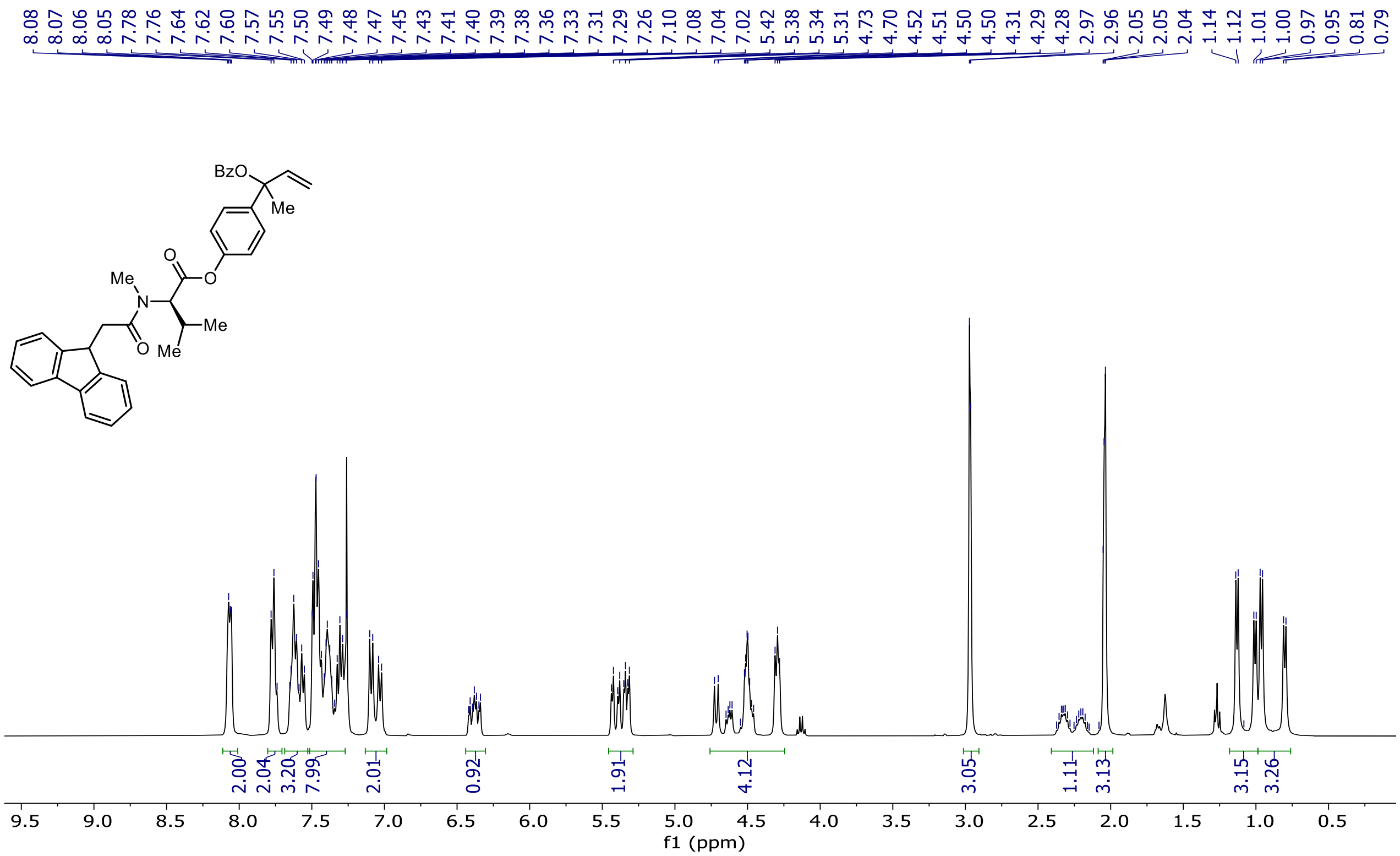

^{13}C NMR (101 MHz, CDCl$_3$, 25 ºC) of 1al

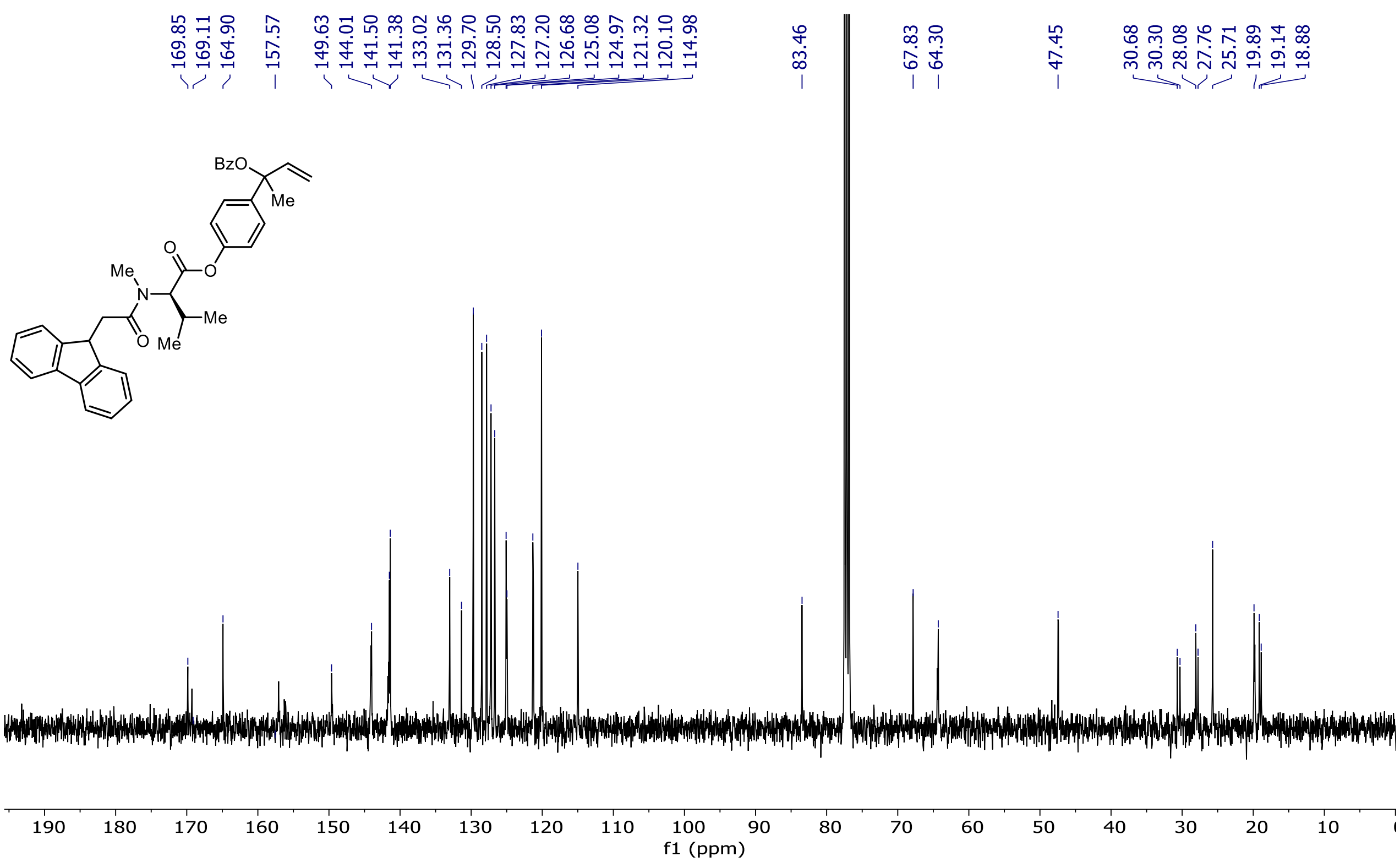

^{1}H NMR (500 MHz, $CDCl_3$, 25 ºC) of 3a

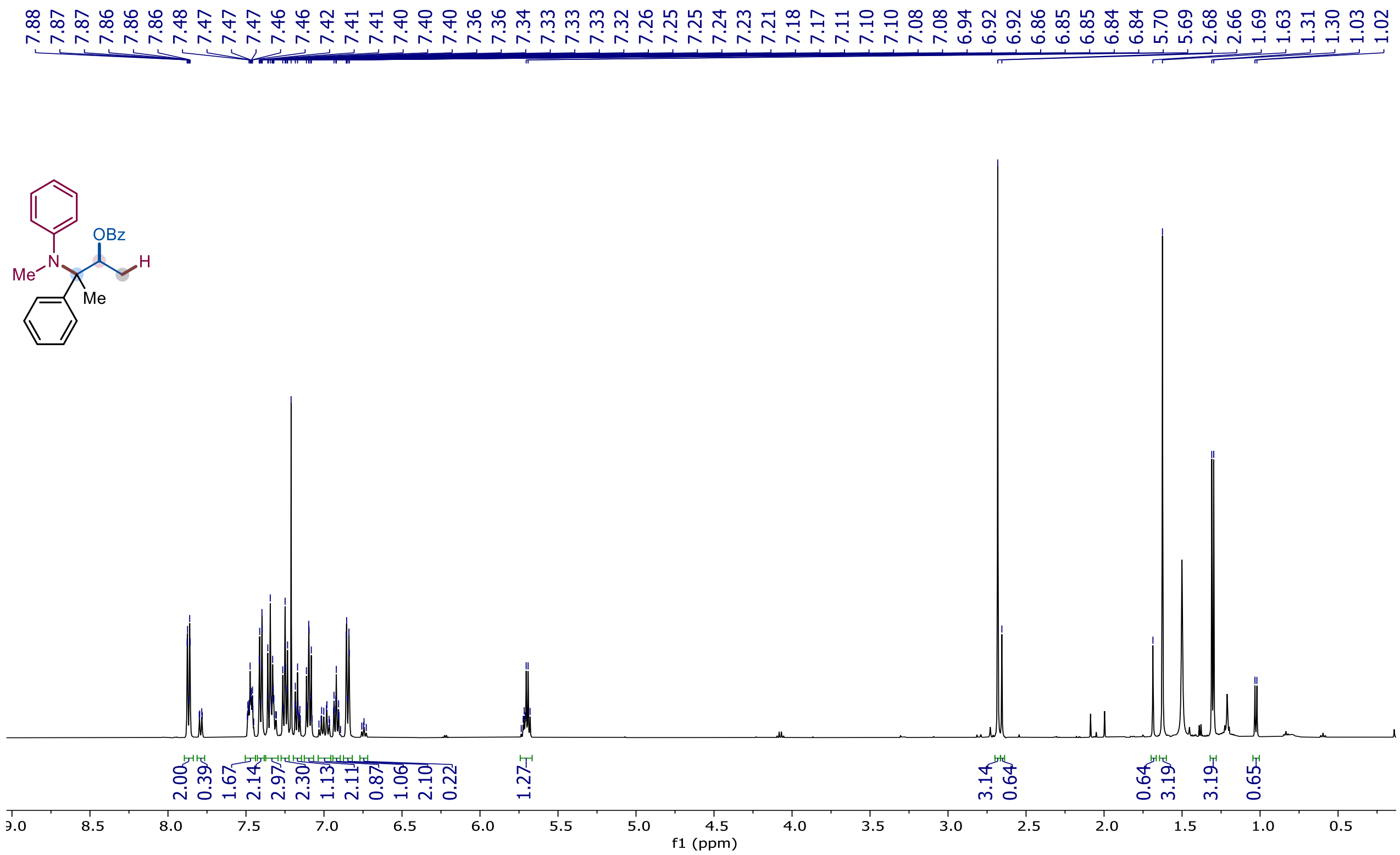

^{13}C NMR (201 MHz, $CDCl_3$, 25 ºC) of 3a

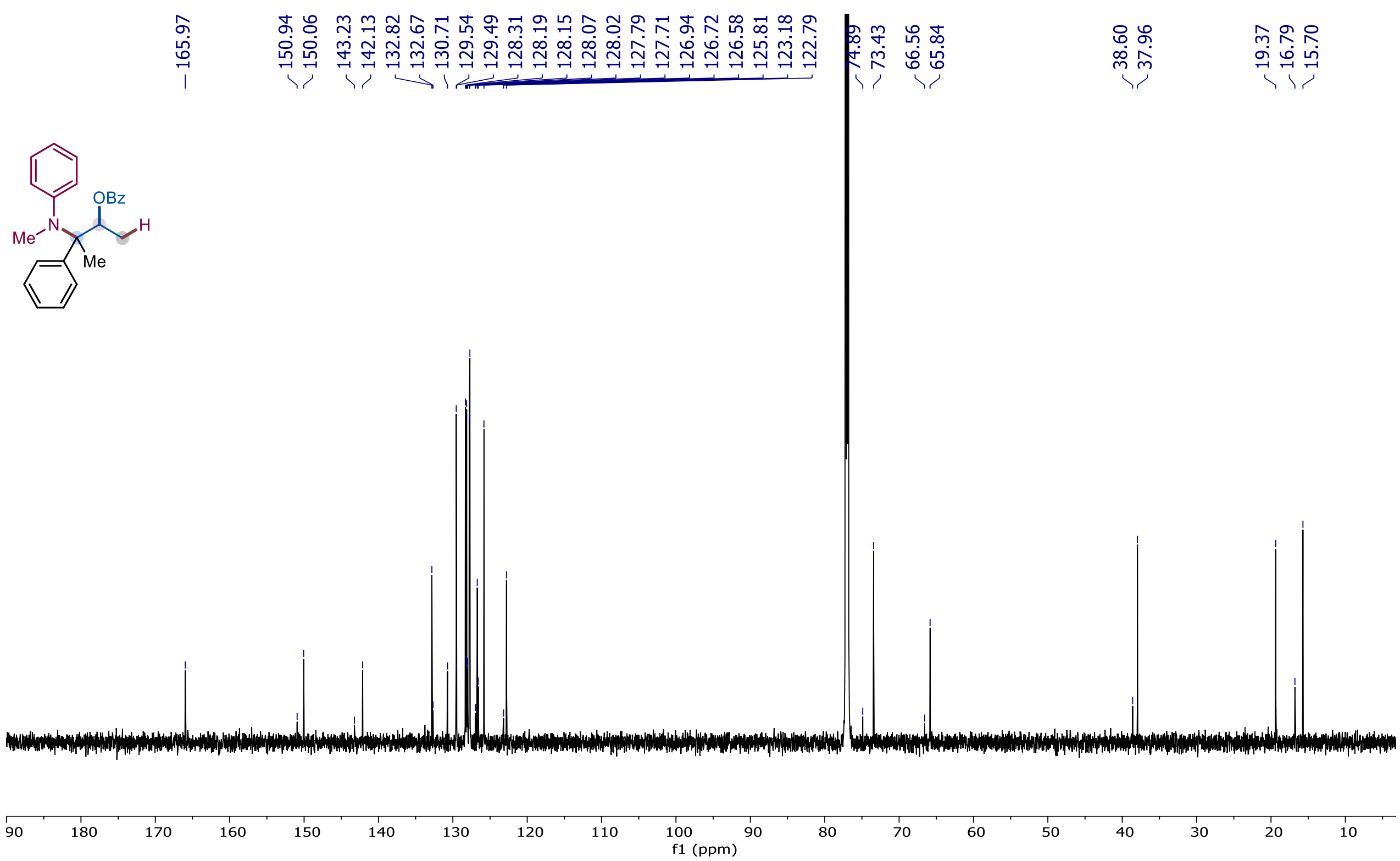

^{1}H NMR (300 MHz, CDCl$_3$, 25 ºC) of 3b

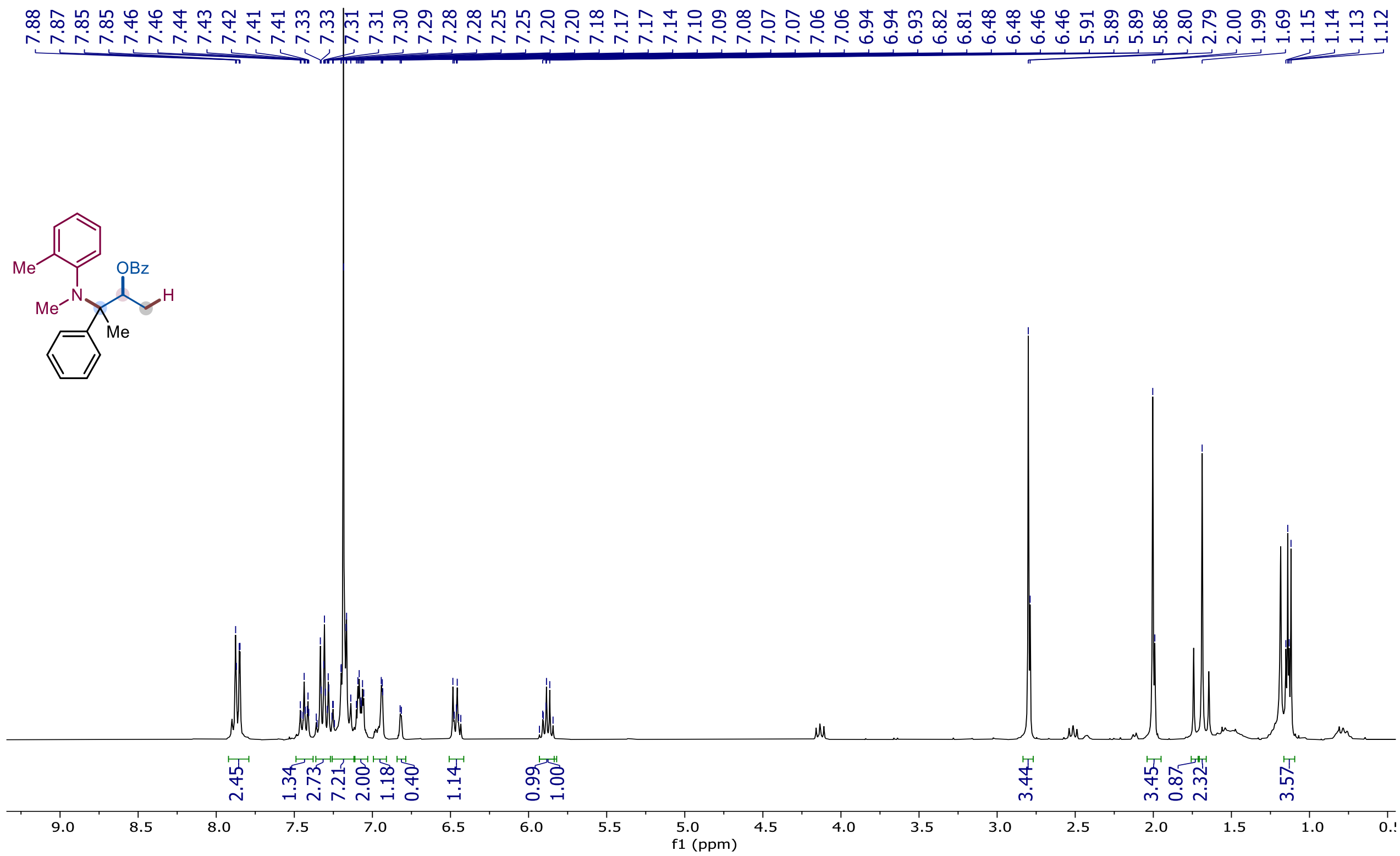

^{13}C NMR (201 MHz, CDCl$_3$, 25 ºC) of 3b

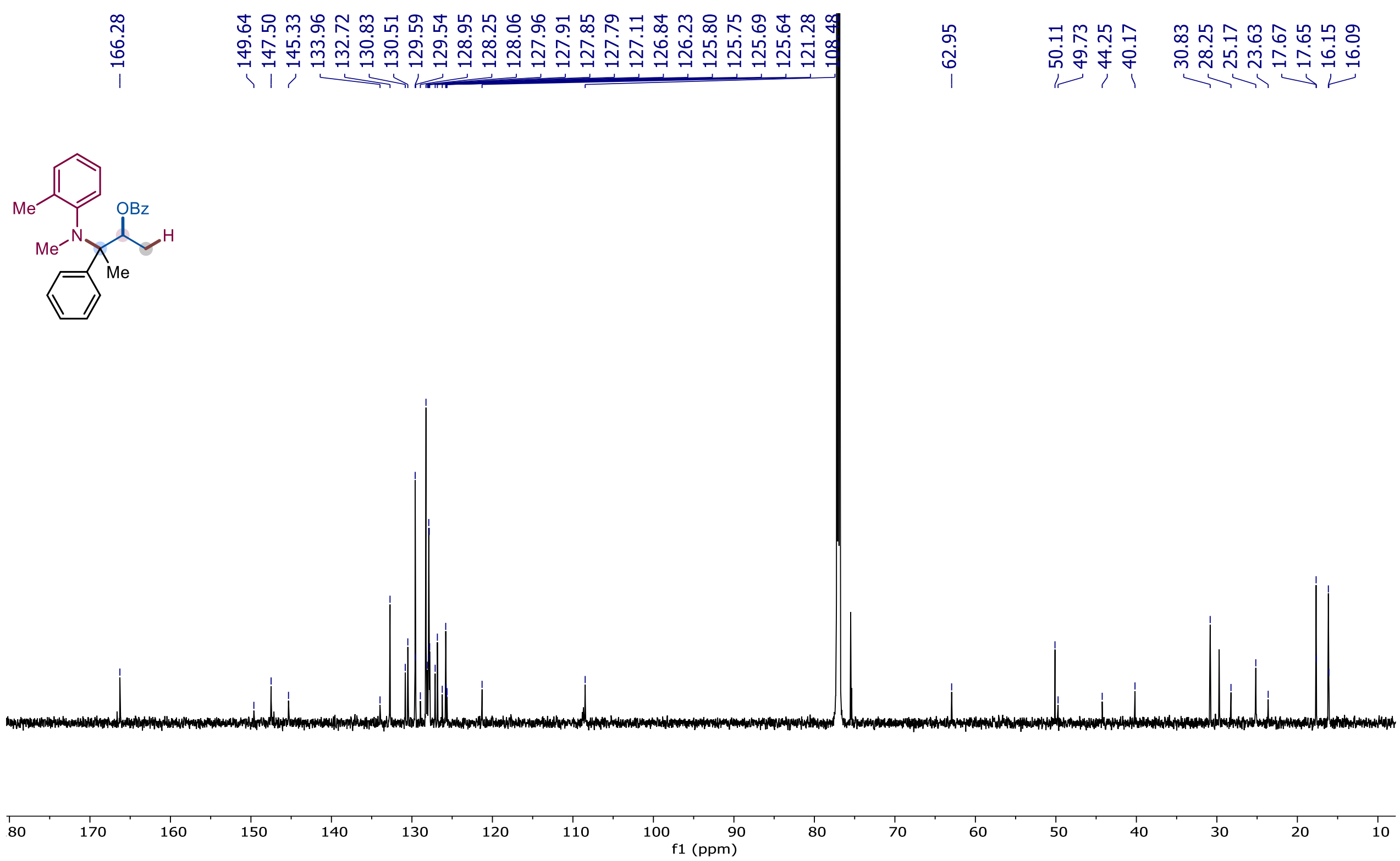

^{1}H NMR (300 MHz, $CDCl_3$, 25 ºC) of 3c

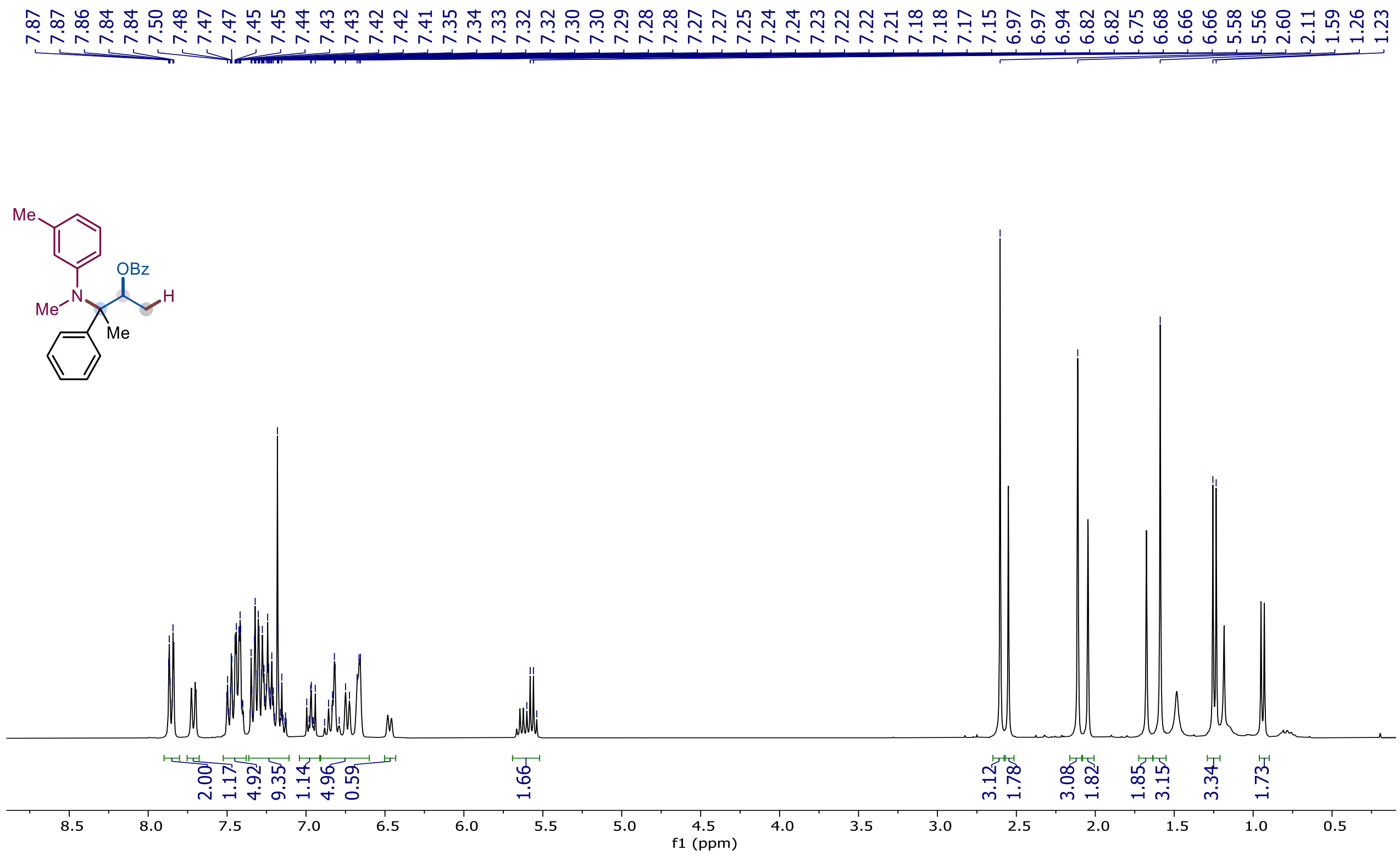

^{13}C NMR (126 MHz, $CDCl_3$, 25 ºC) of 3c

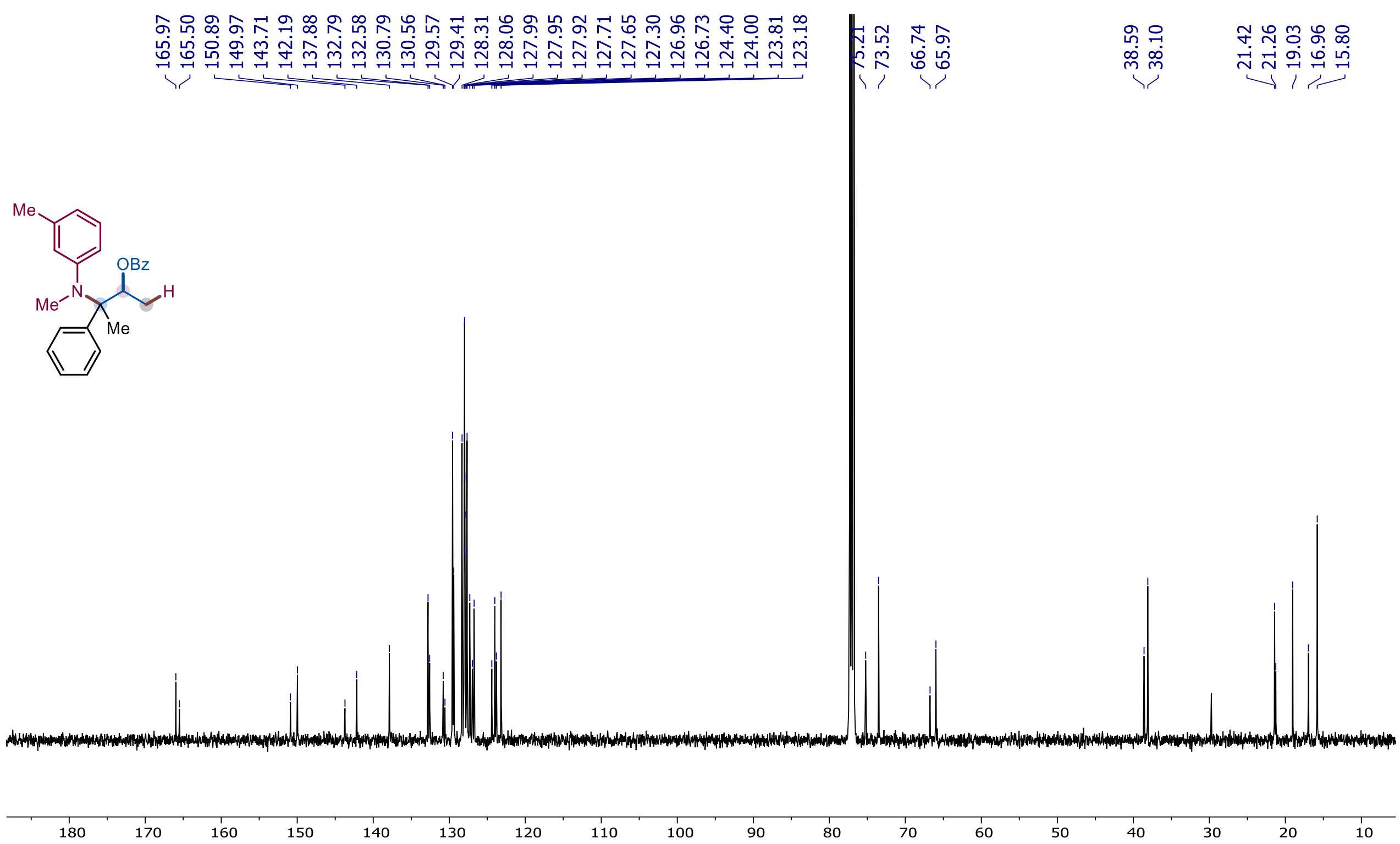

^{1}H NMR (400 MHz, $CDCl_3$, 25 ºC) of 3d

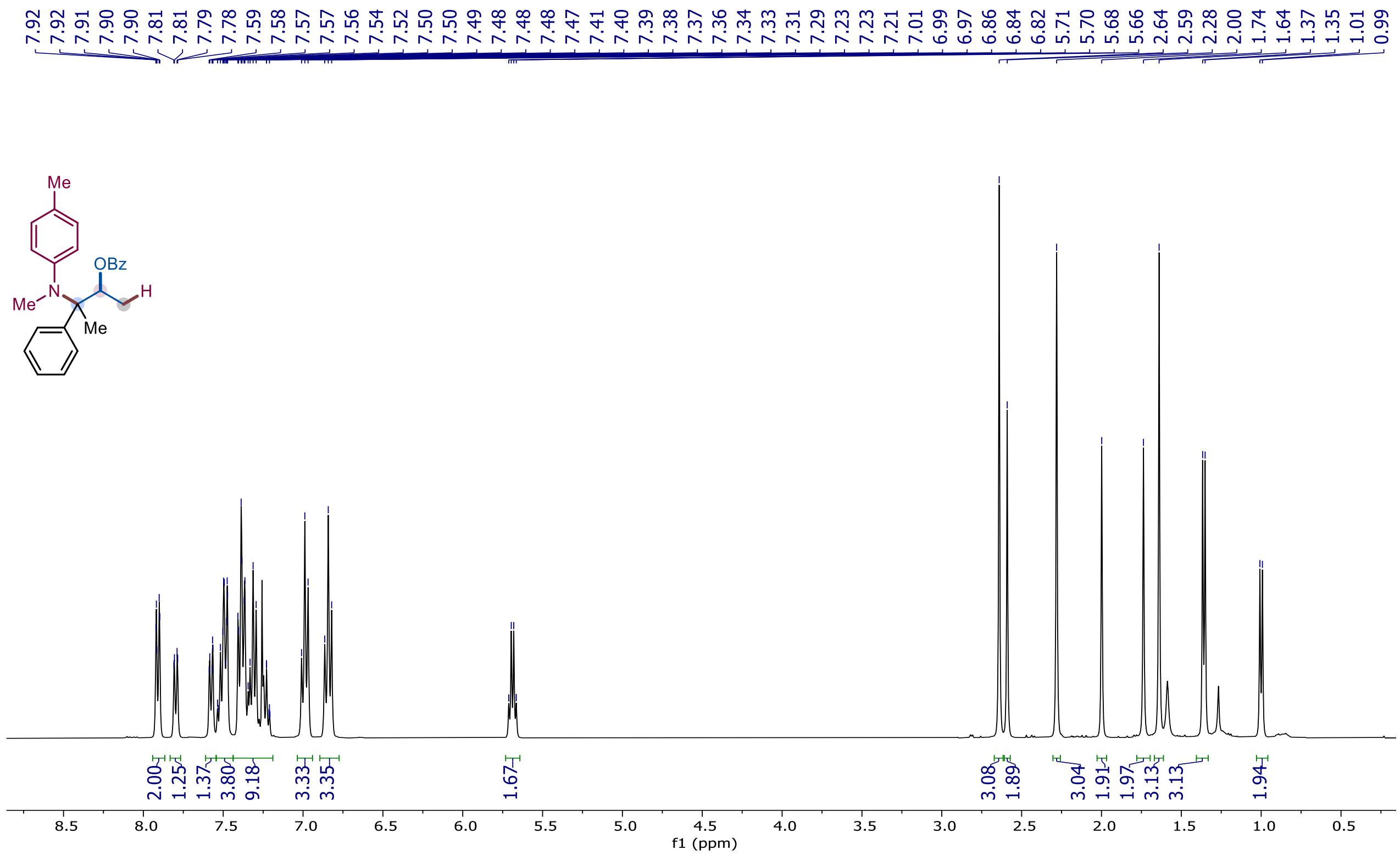

^{13}C NMR (201 MHz, $CDCl_3$, 25 ºC) of 3d

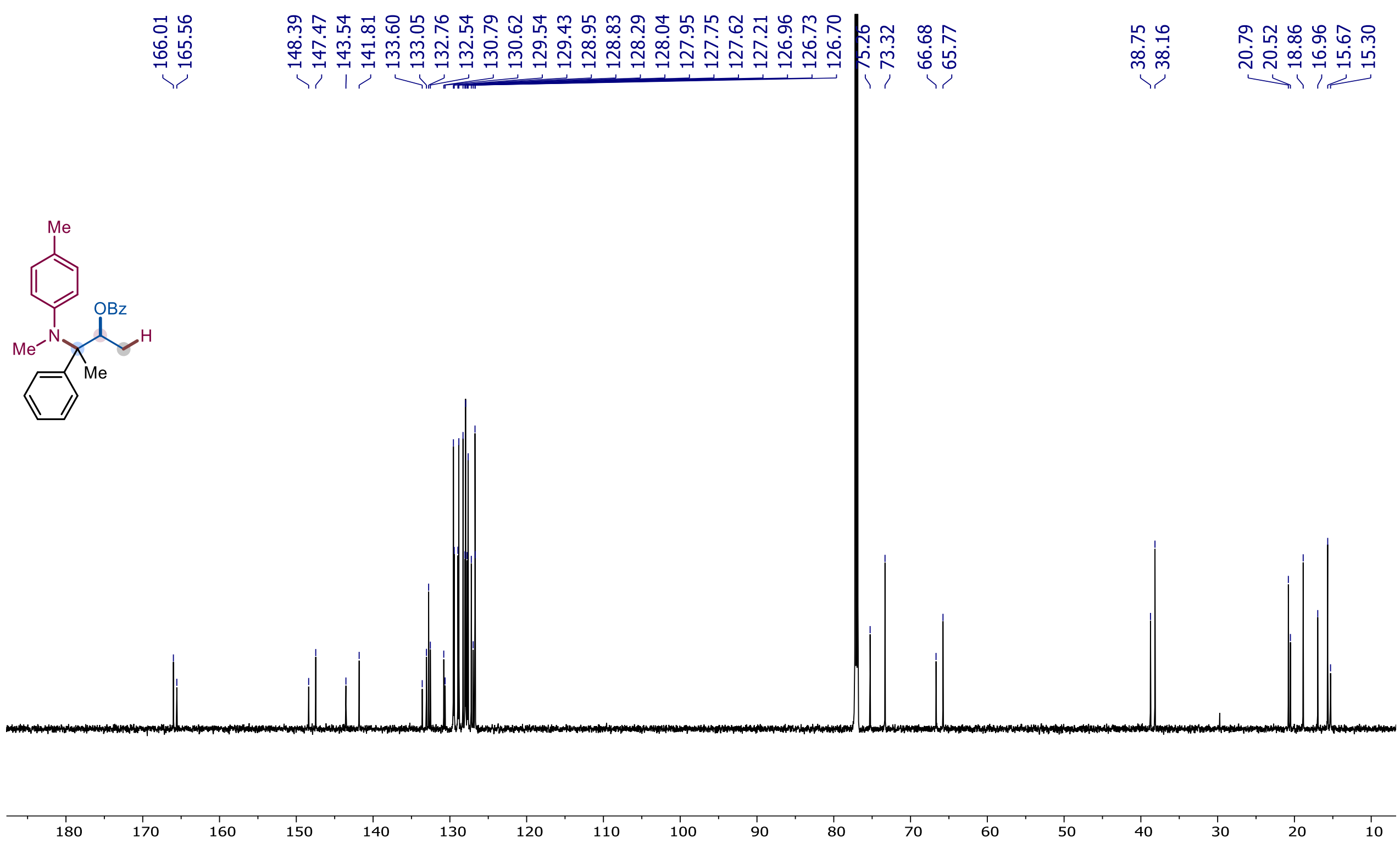

^{1}H NMR (300 MHz, $CDCl_3$, 25 ºC) of 3e

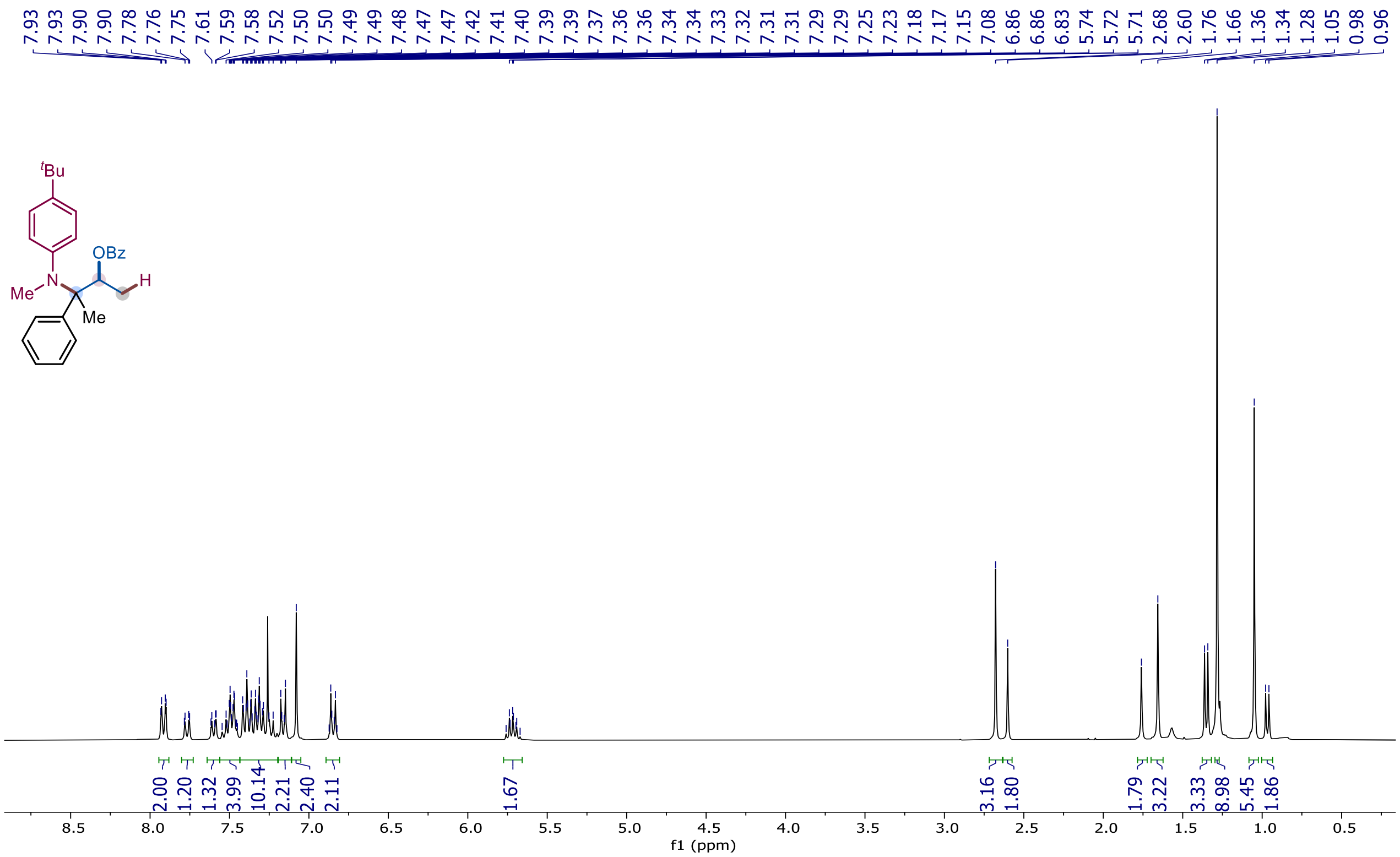

^{13}C NMR (126 MHz, $CDCl_3$, 25 ºC) of 3e

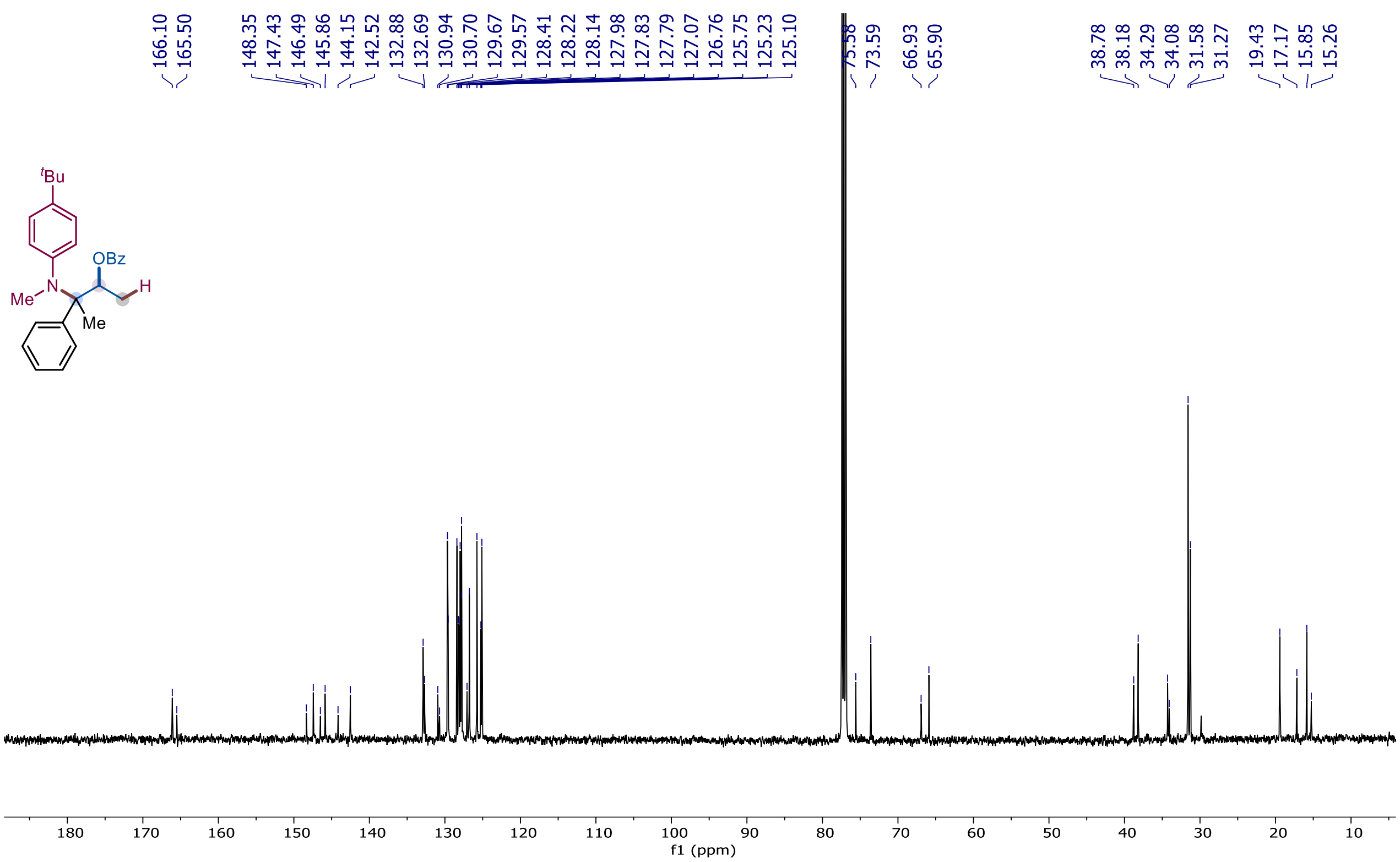

^{1}H NMR (500 MHz, CDCl$_3$, 25 °C) of 3f

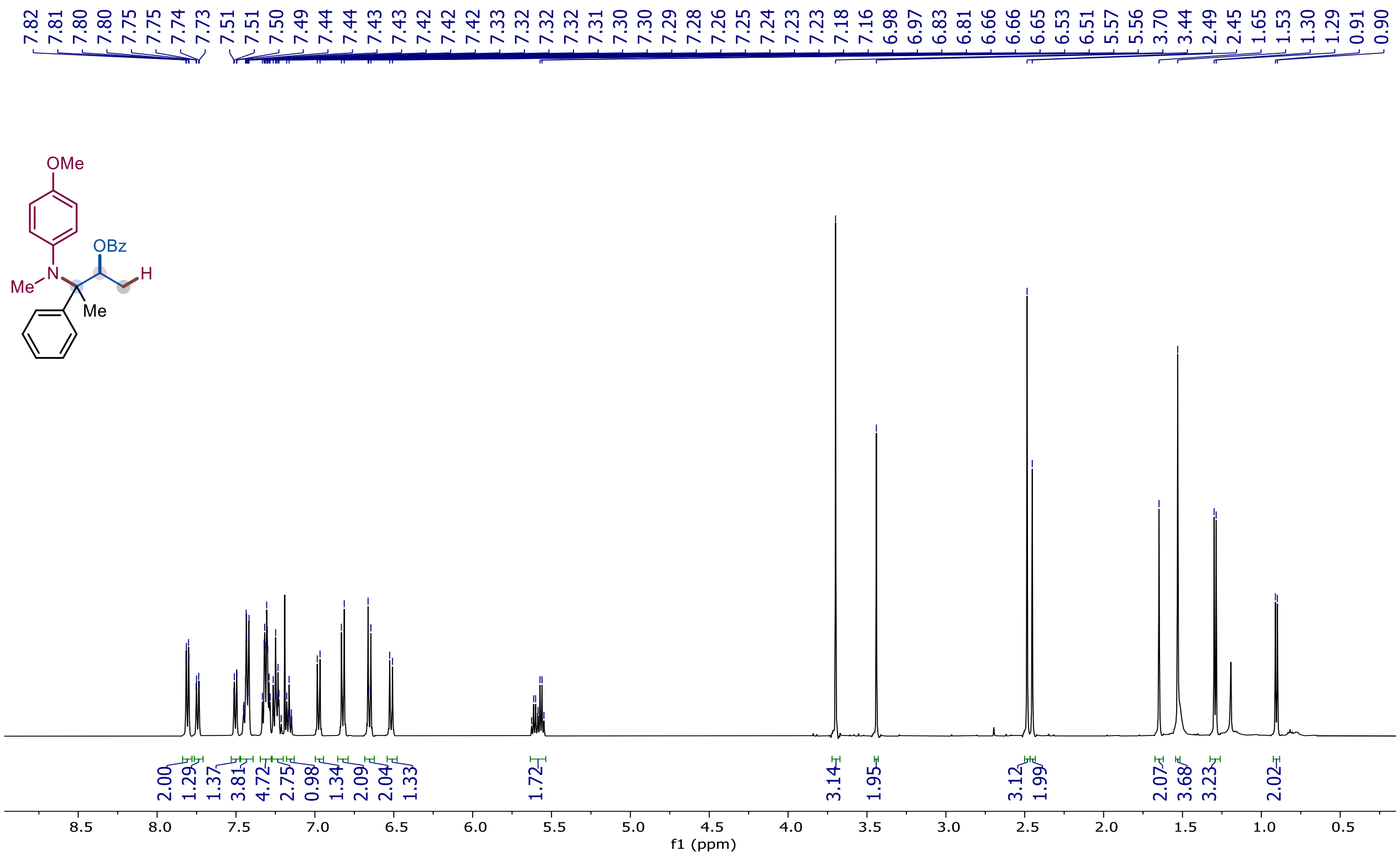

^{13}C NMR (126 MHz, CDCl$_3$, 25 °C) of 3f

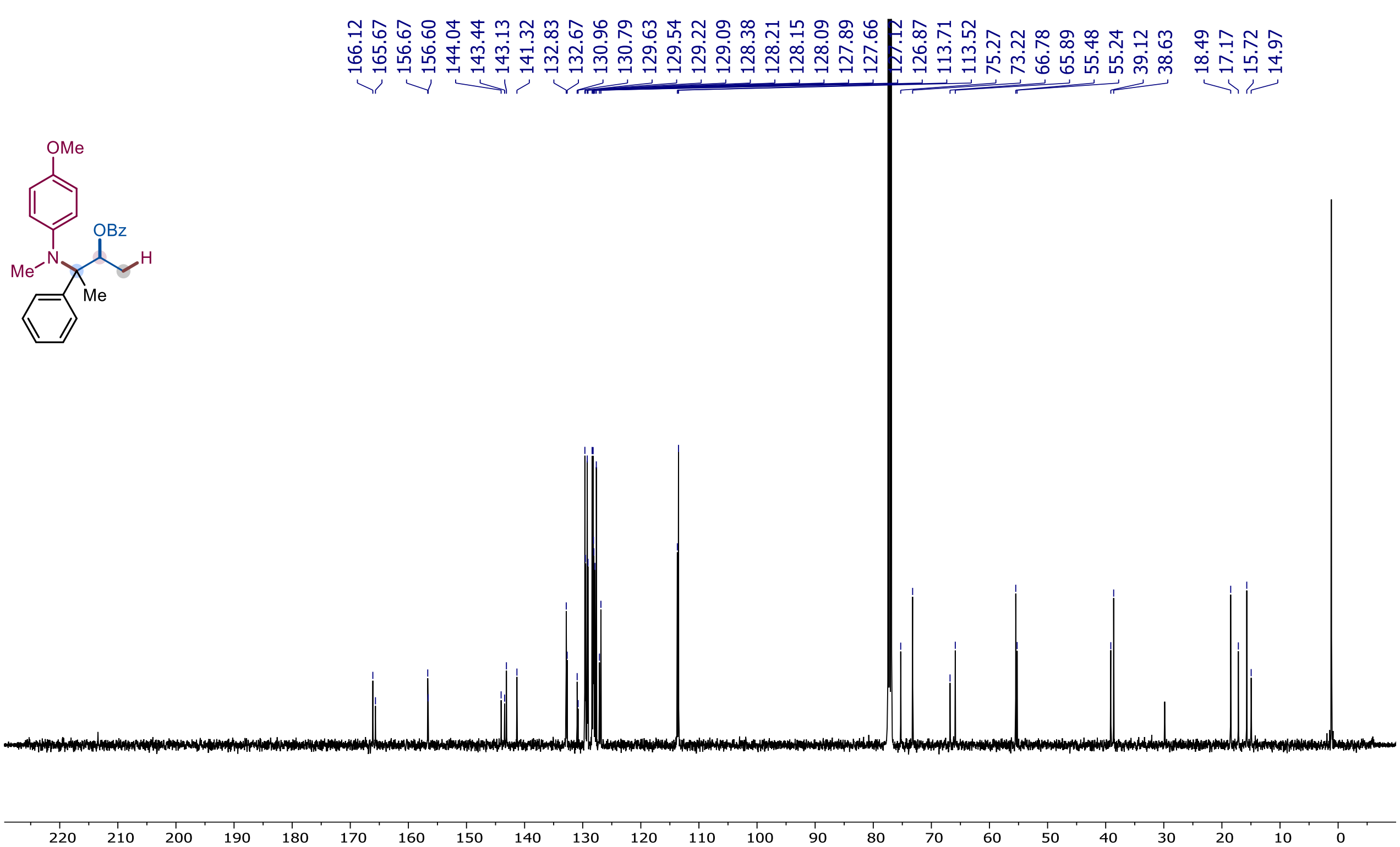

^{1}H NMR (500 MHz, $CDCl_3$, 25 ºC) of 3g

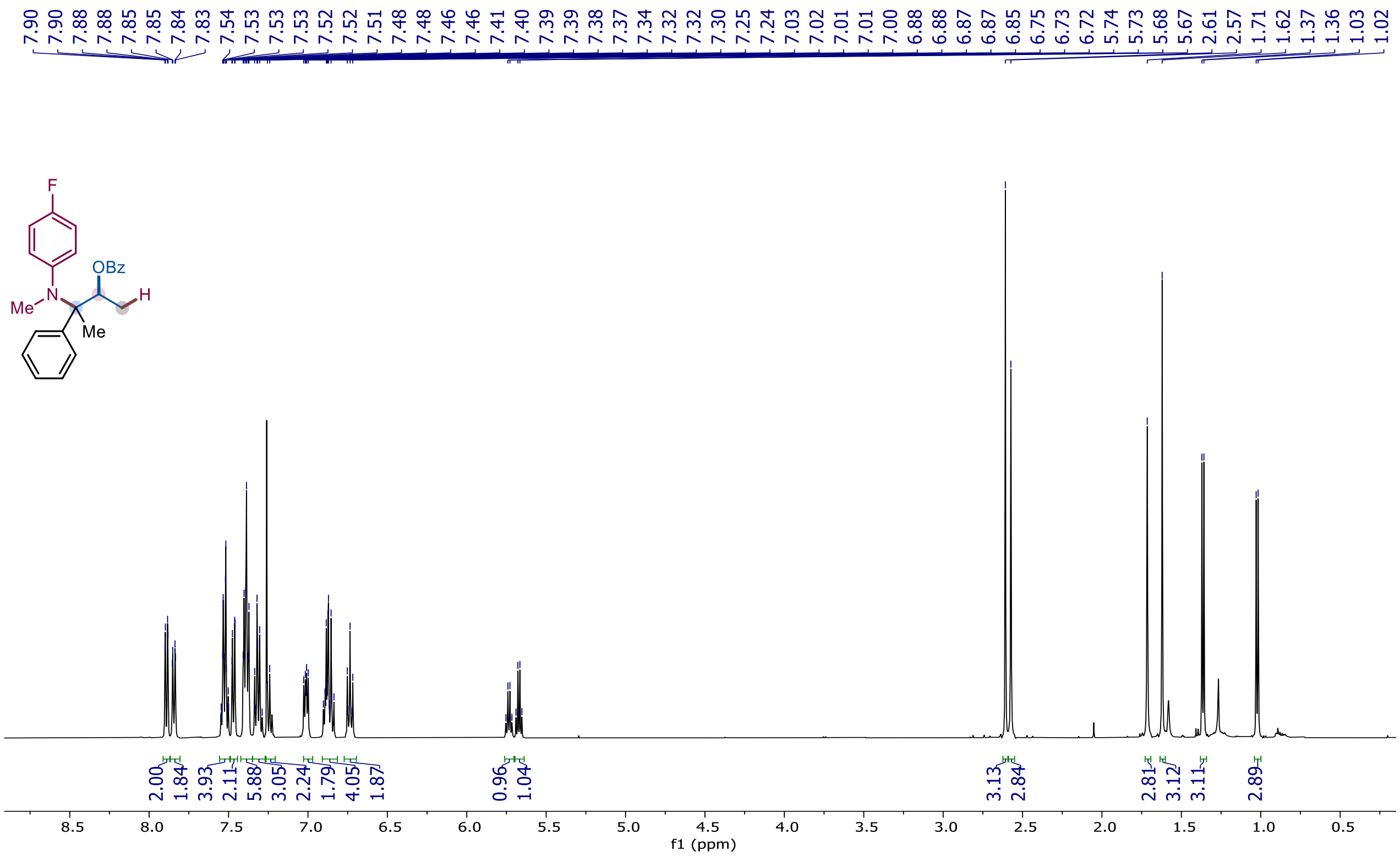

^{13}C NMR (126 MHz, $CDCl_3$, 25 ºC) of 3g

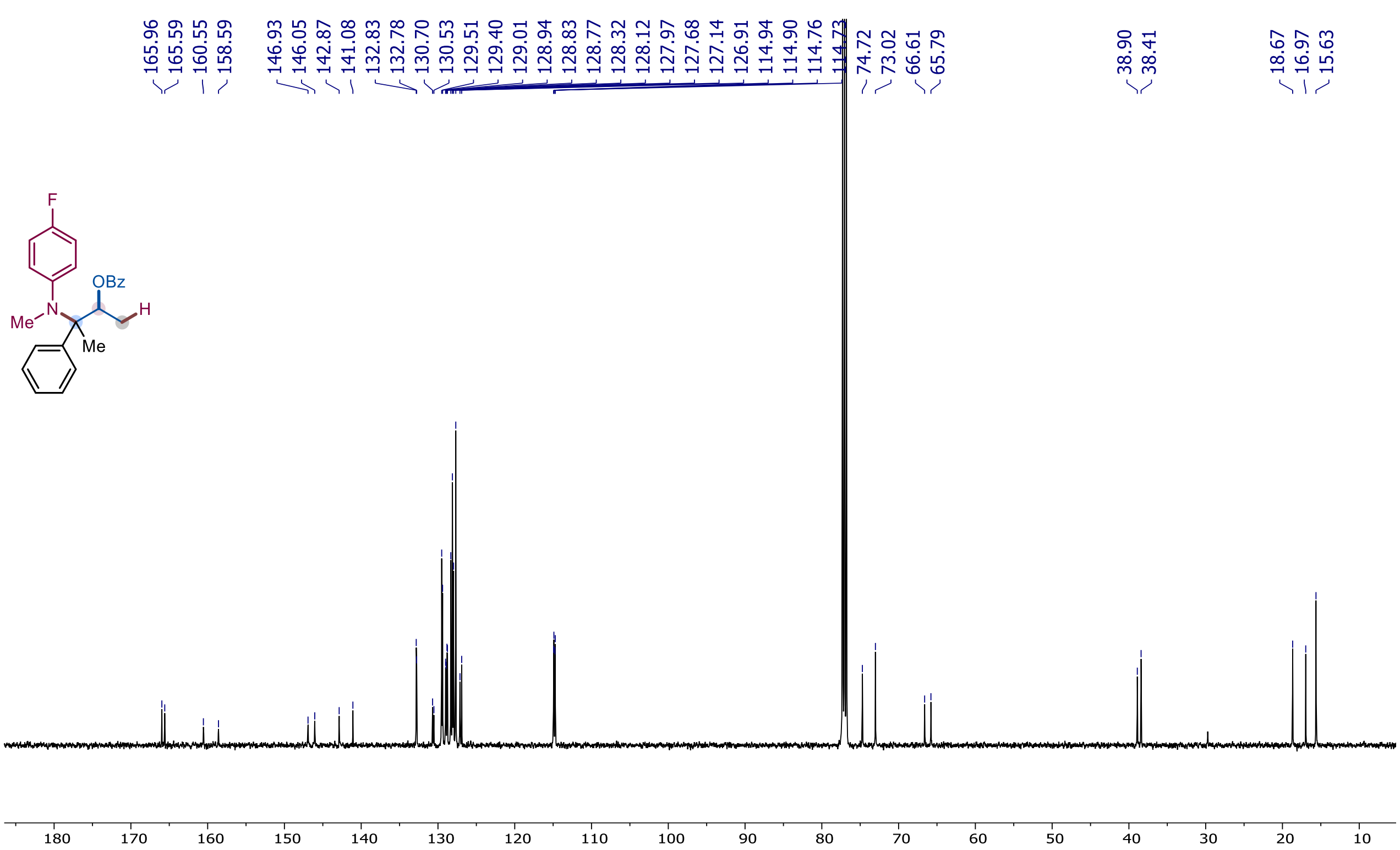

^{19}F NMR (470 MHz, $CDCl_3$, 25 ^{o}C) of 3g

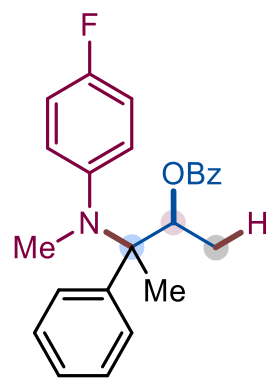

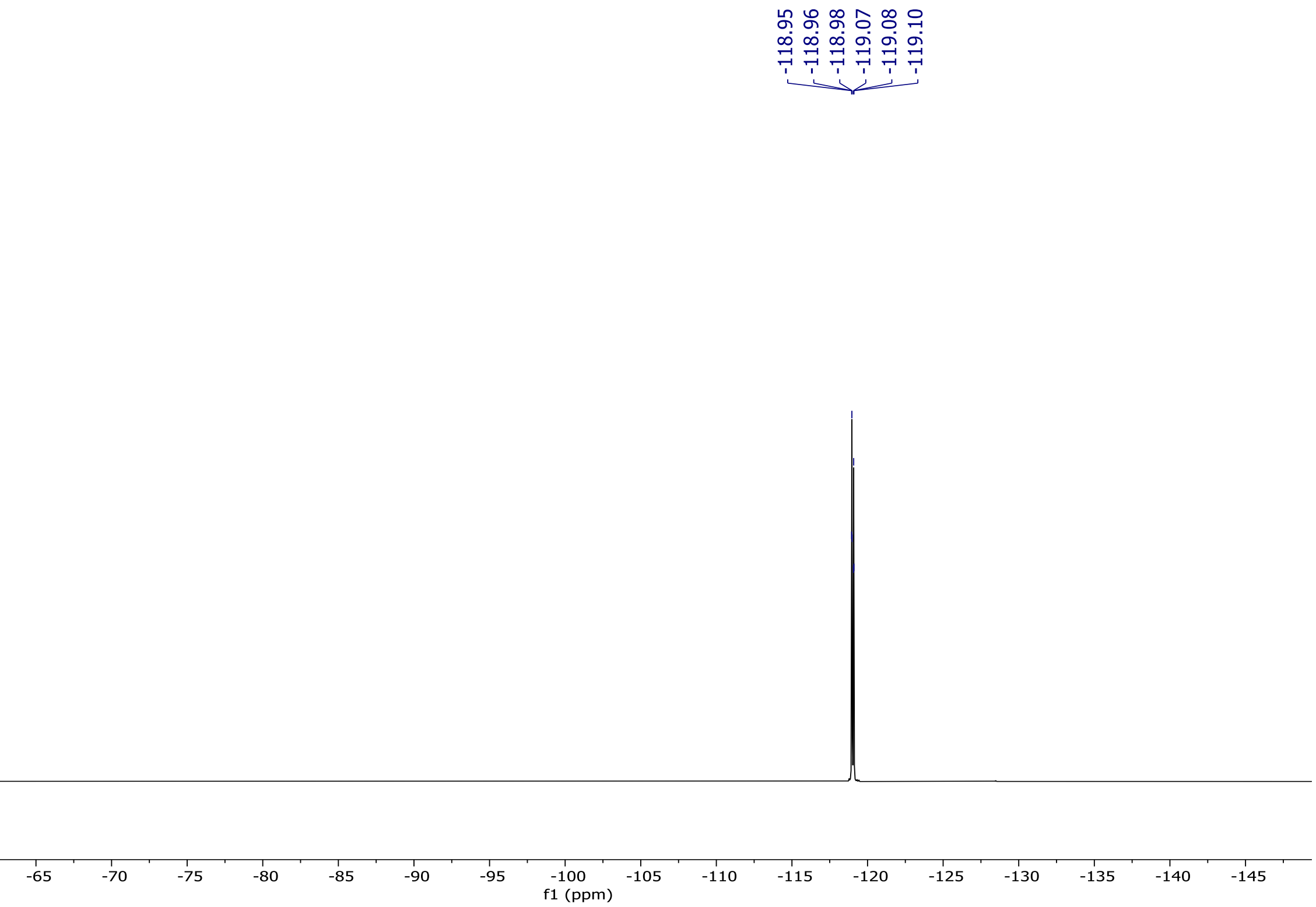

^{1}H NMR (400 MHz, $CDCl_3$, 25 ºC) of 3h

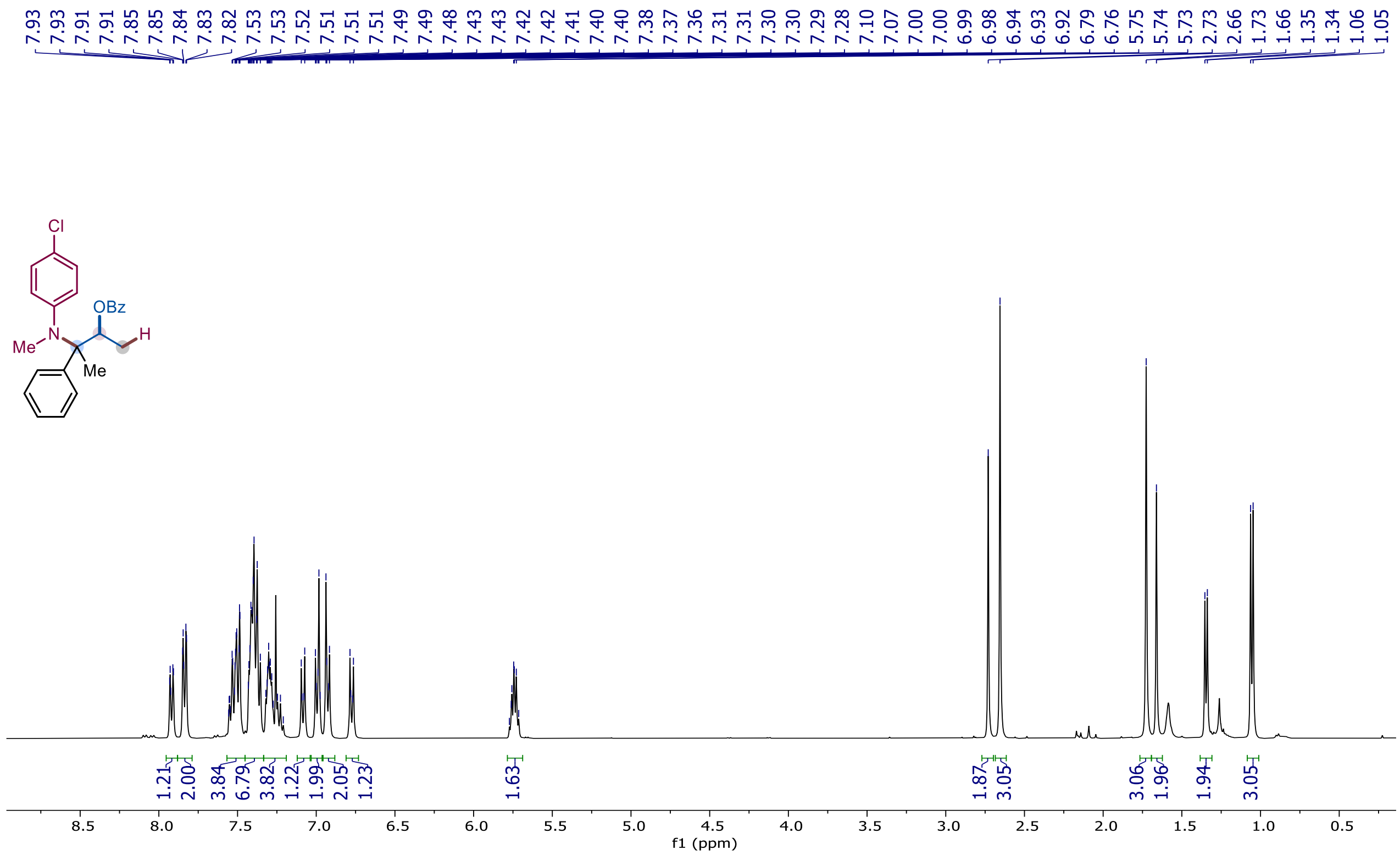

^{13}C NMR (201 MHz, $CDCl_3$, 25 ºC) of 3h

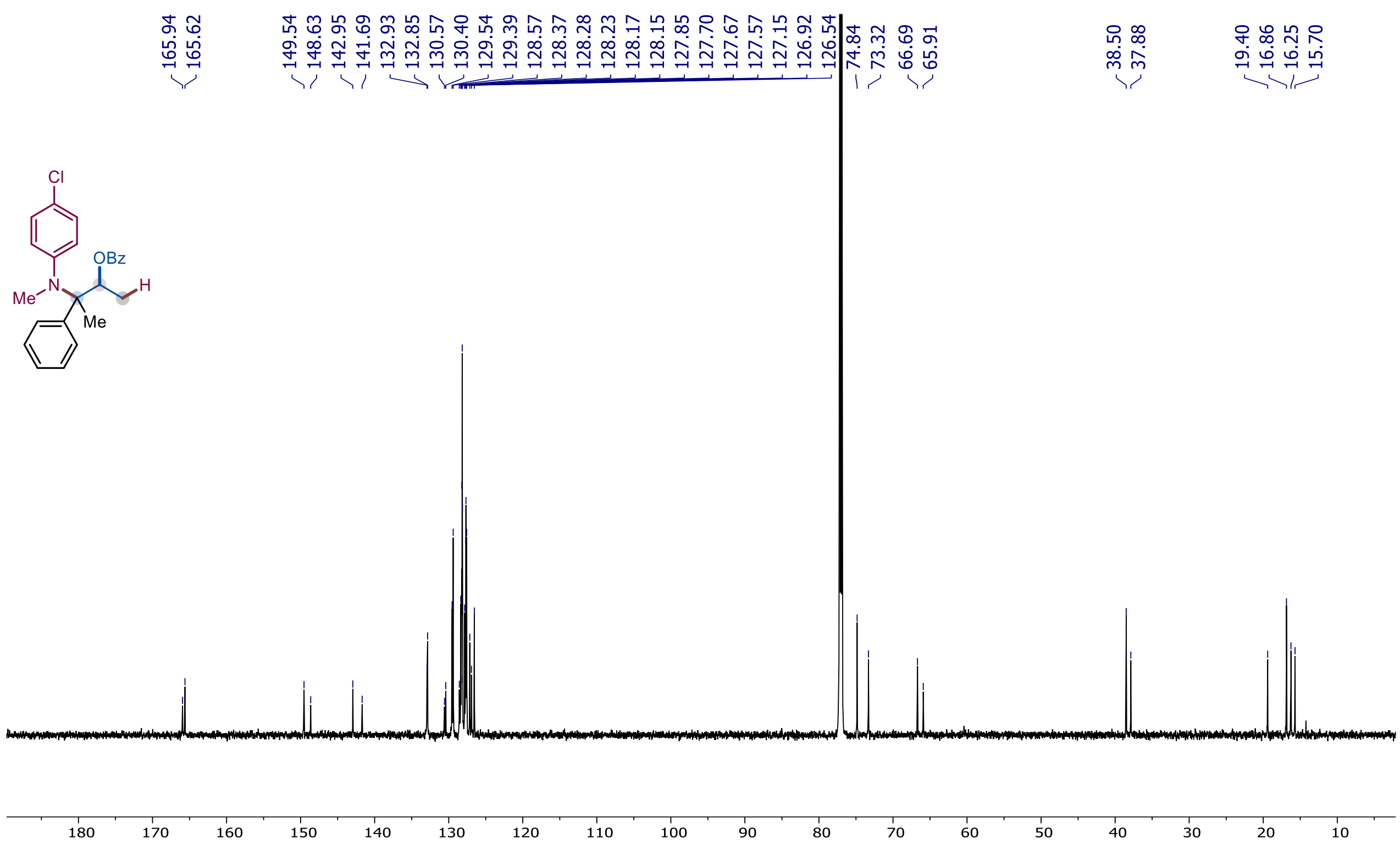

^{1}H NMR (500 MHz, $CDCl_3$, 25 ºC) of 3i

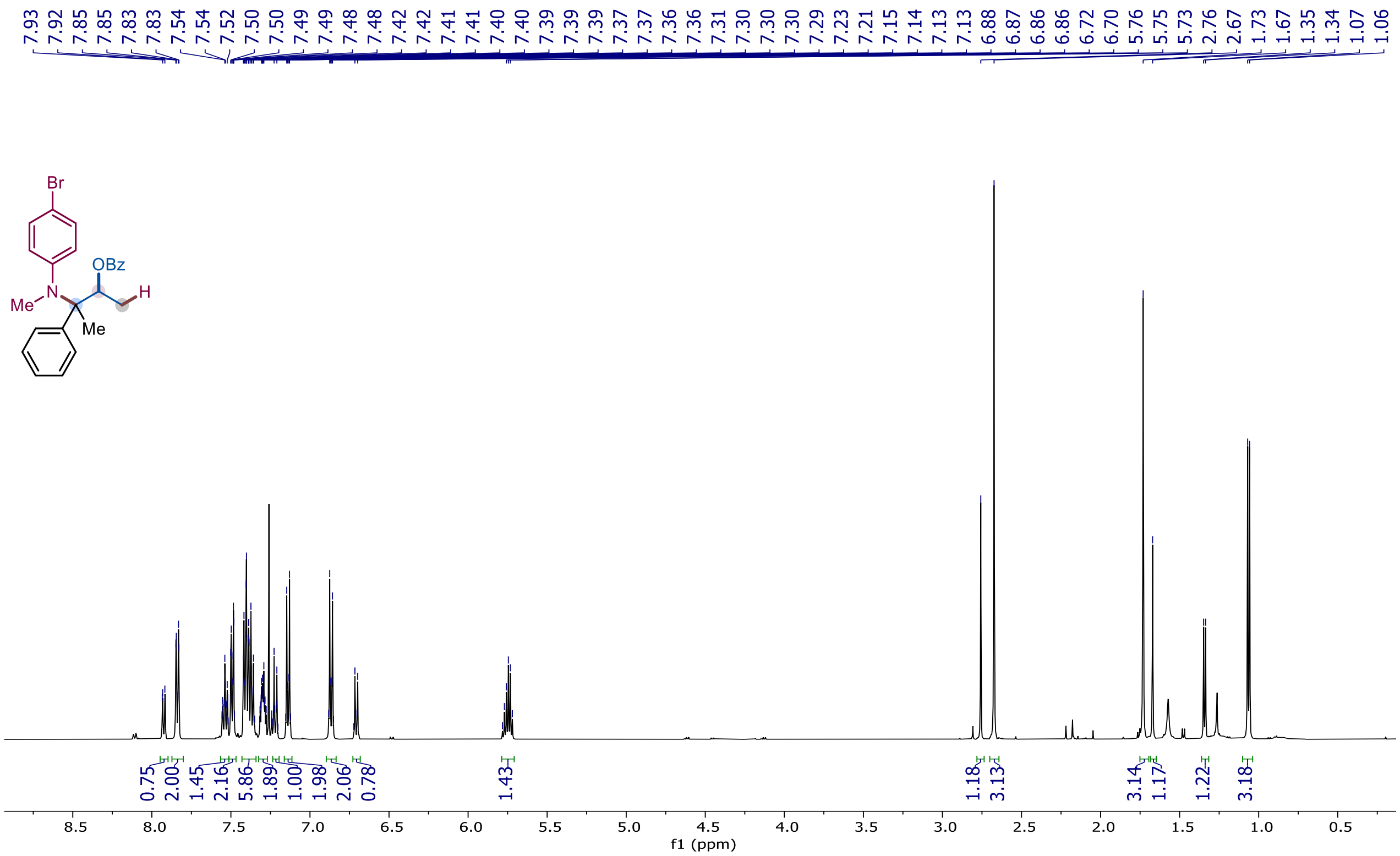

^{13}C NMR (126 MHz, $CDCl_3$, 25 ºC) of 3i

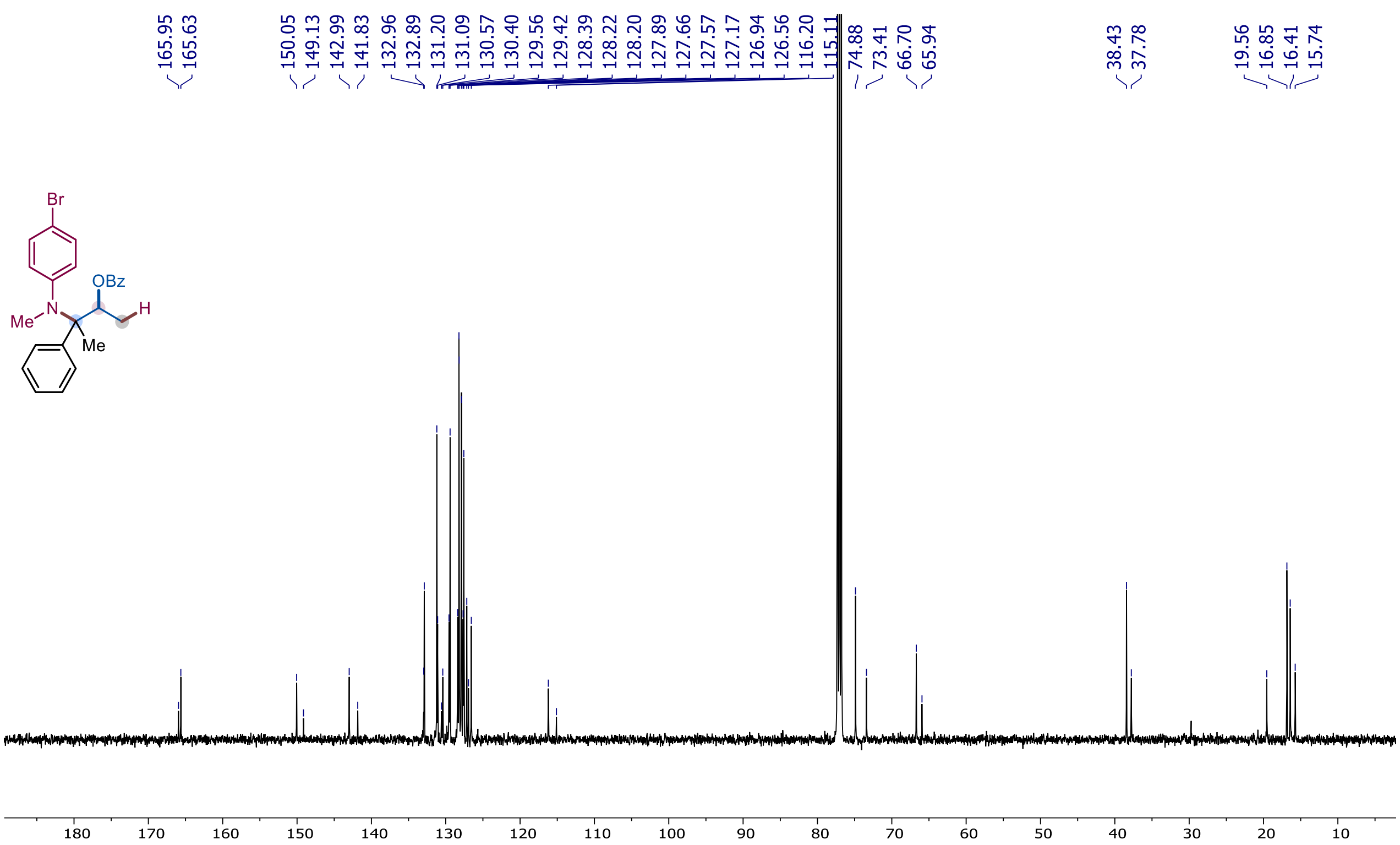

^{1}H NMR (500 MHz, $CDCl_3$, 25 ºC) of 3j

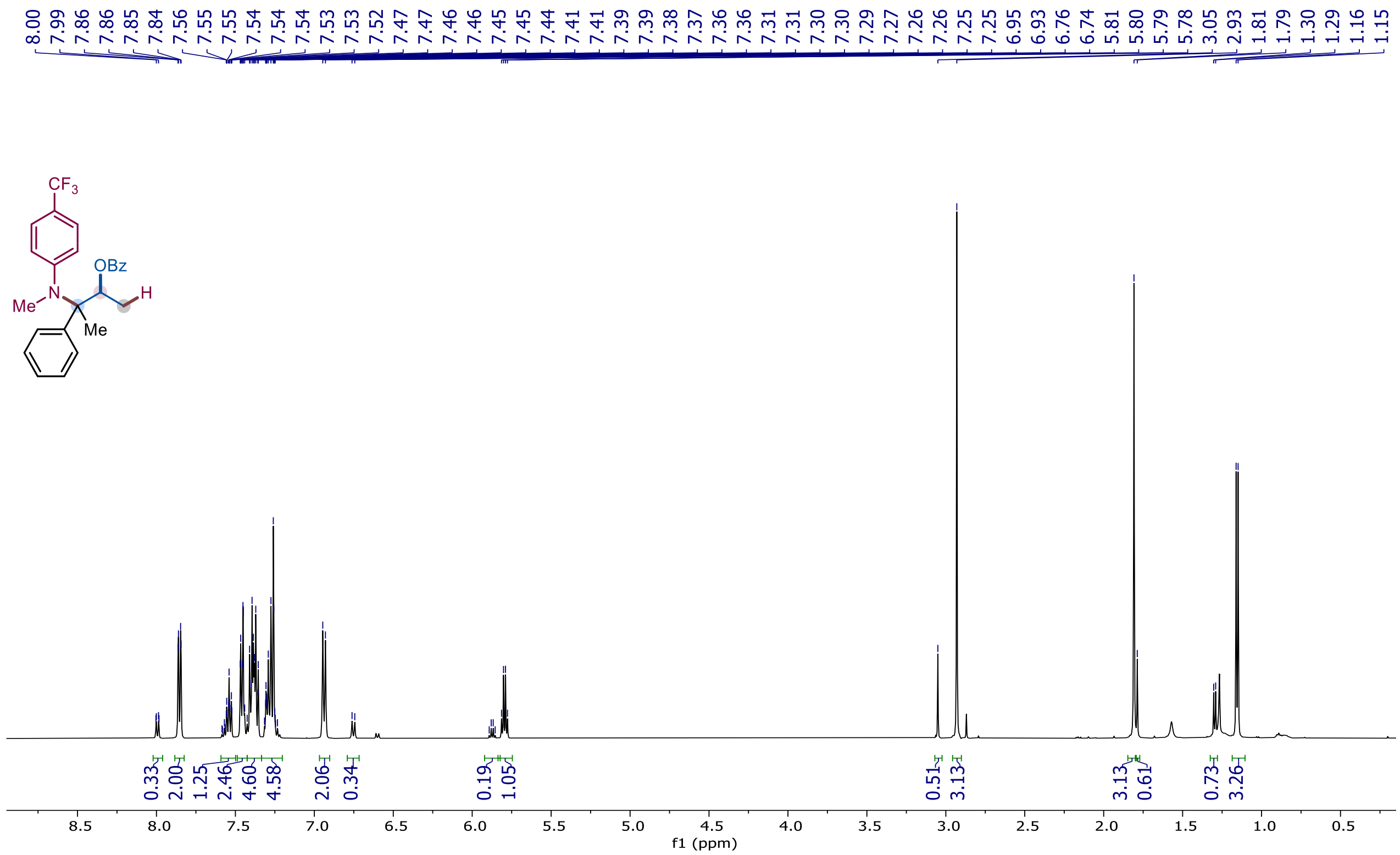

^{13}C NMR (126 MHz, $CDCl_3$, 25 ºC) of 3j

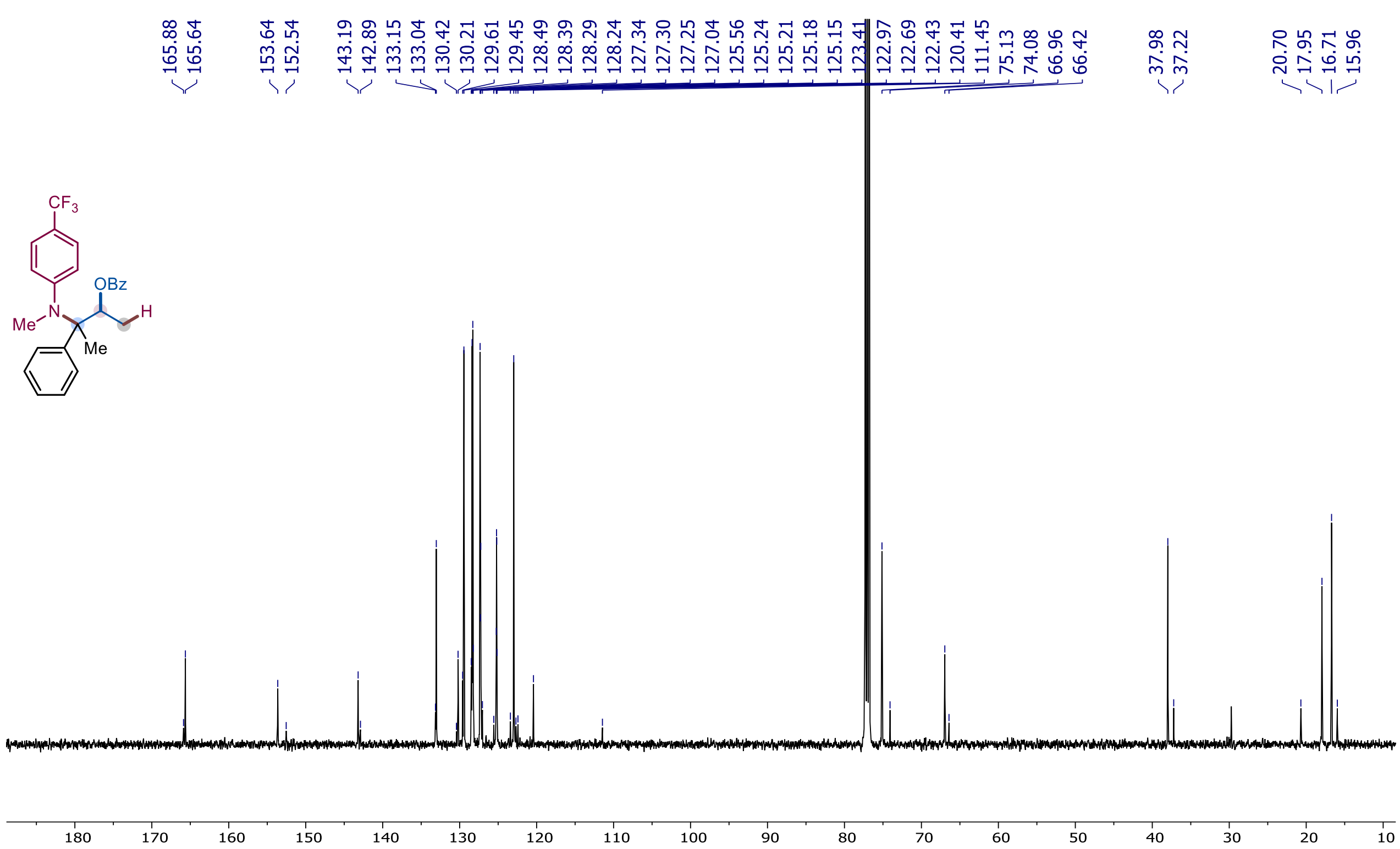

^{19}F NMR (282 MHz, $CDCl_3$, 25 °C) of 3j

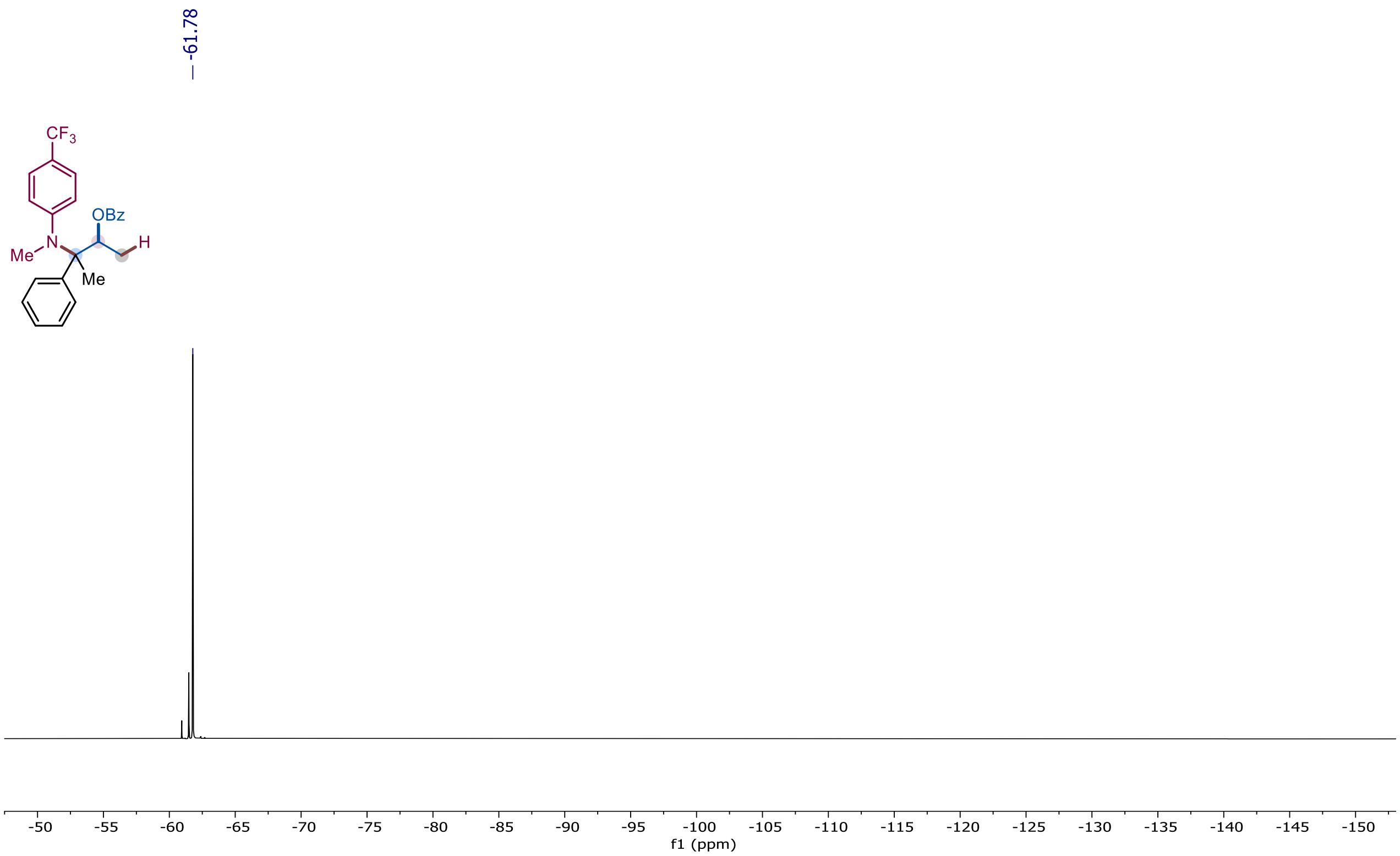

^{1}H NMR (400 MHz, $CDCl_3$, 25 ºC) of 3k

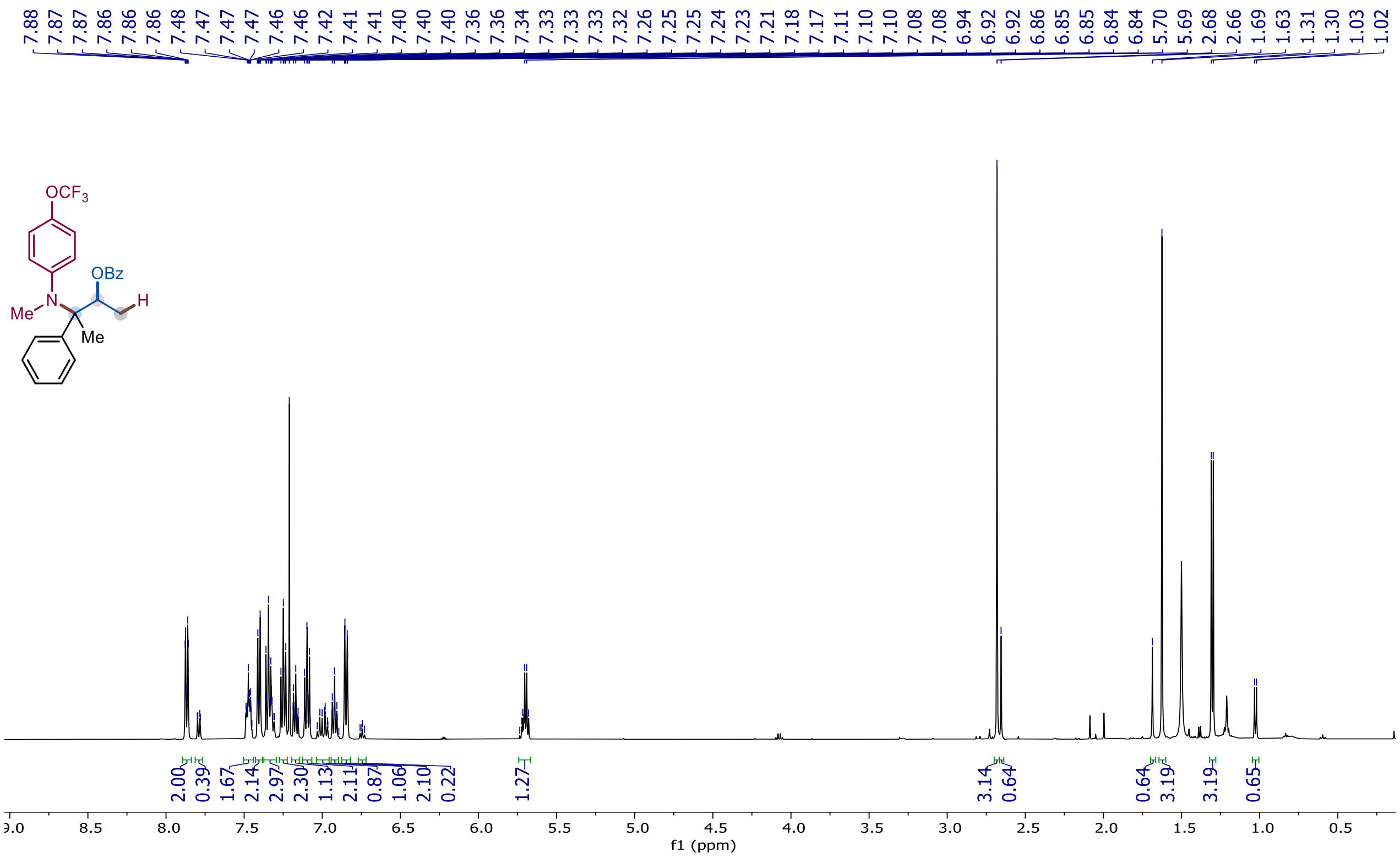

^{13}C NMR (201 MHz, CD_3CN, 25 ºC) of 3k

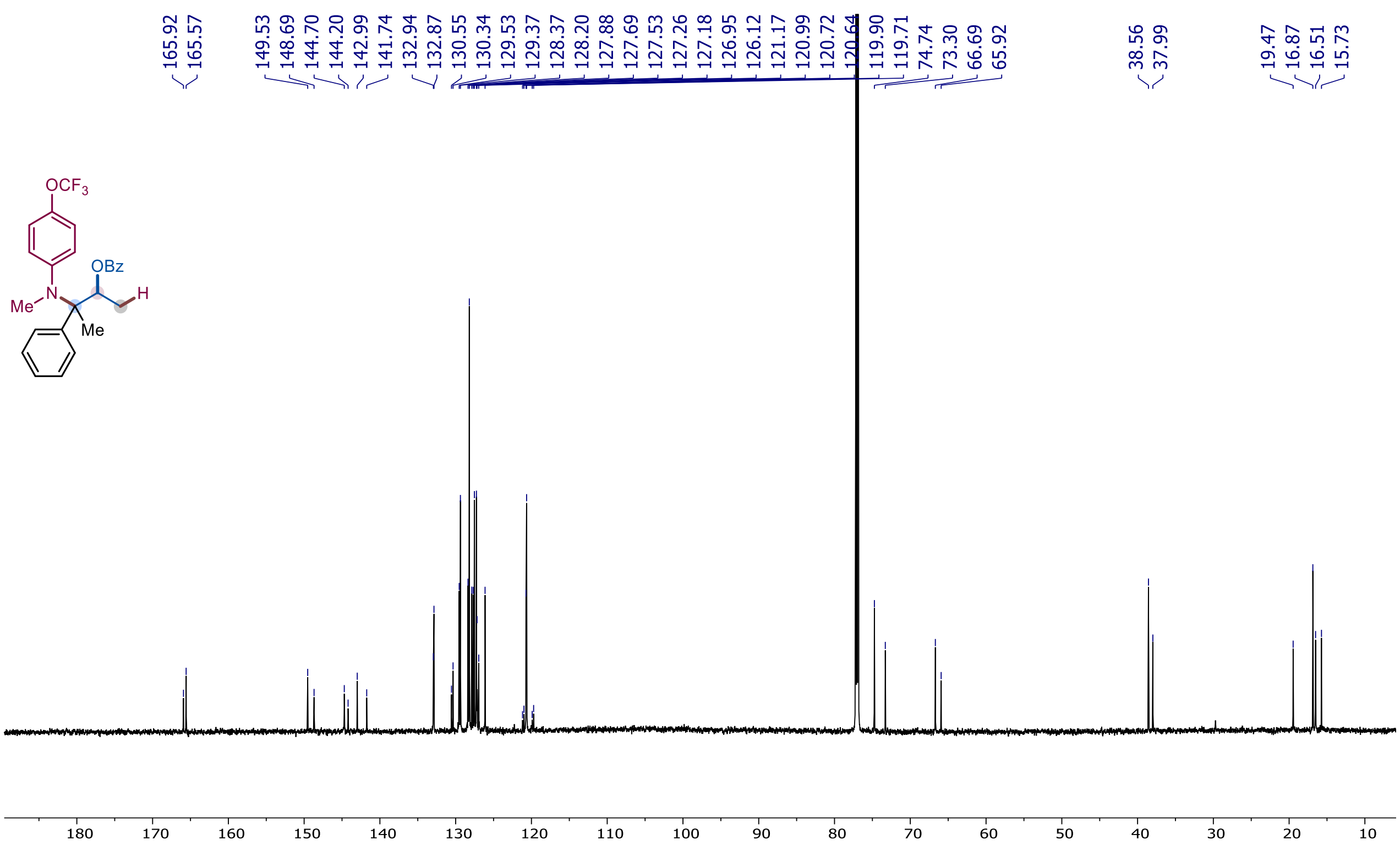

^{19}F NMR (376 MHz, $CDCl_3$, 25 °C) of 3k

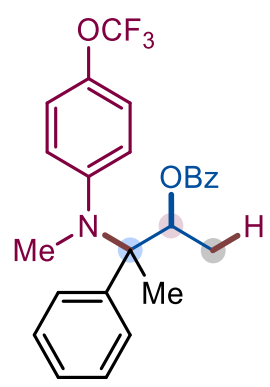

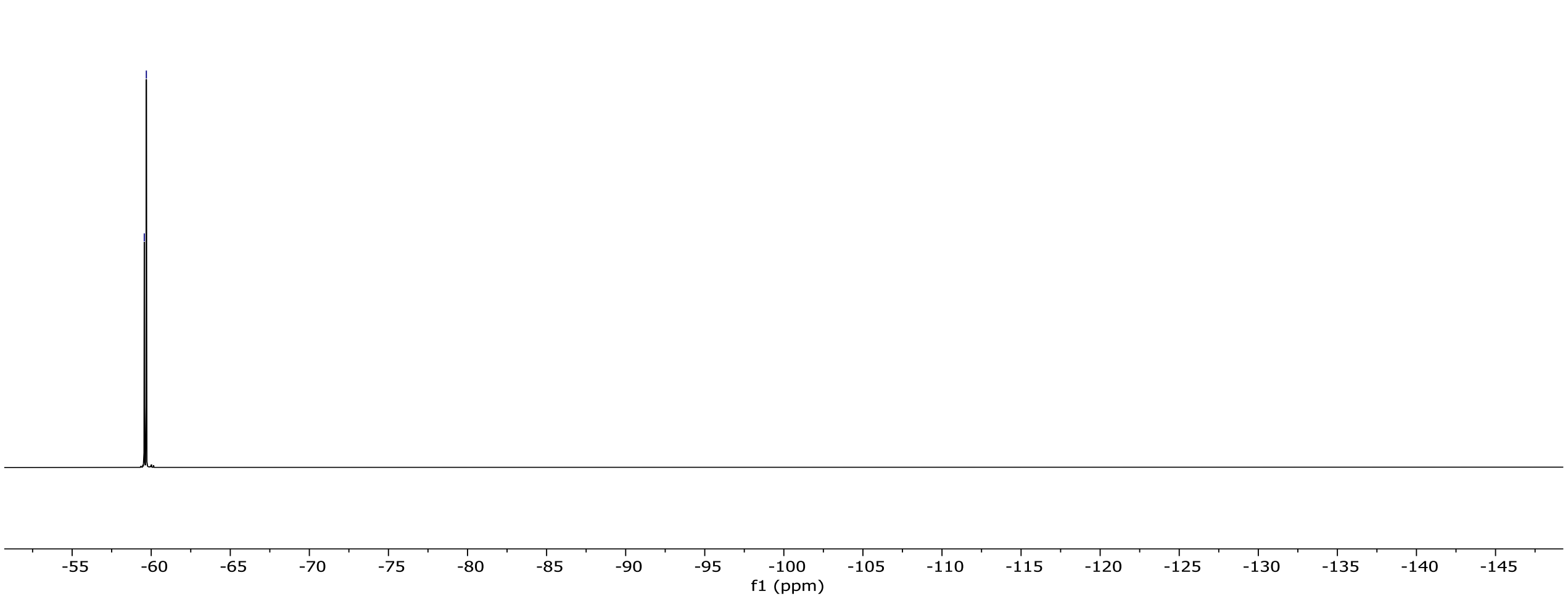

^{1}H NMR (400 MHz, CDCl$_3$, 25 ºC) of 3l

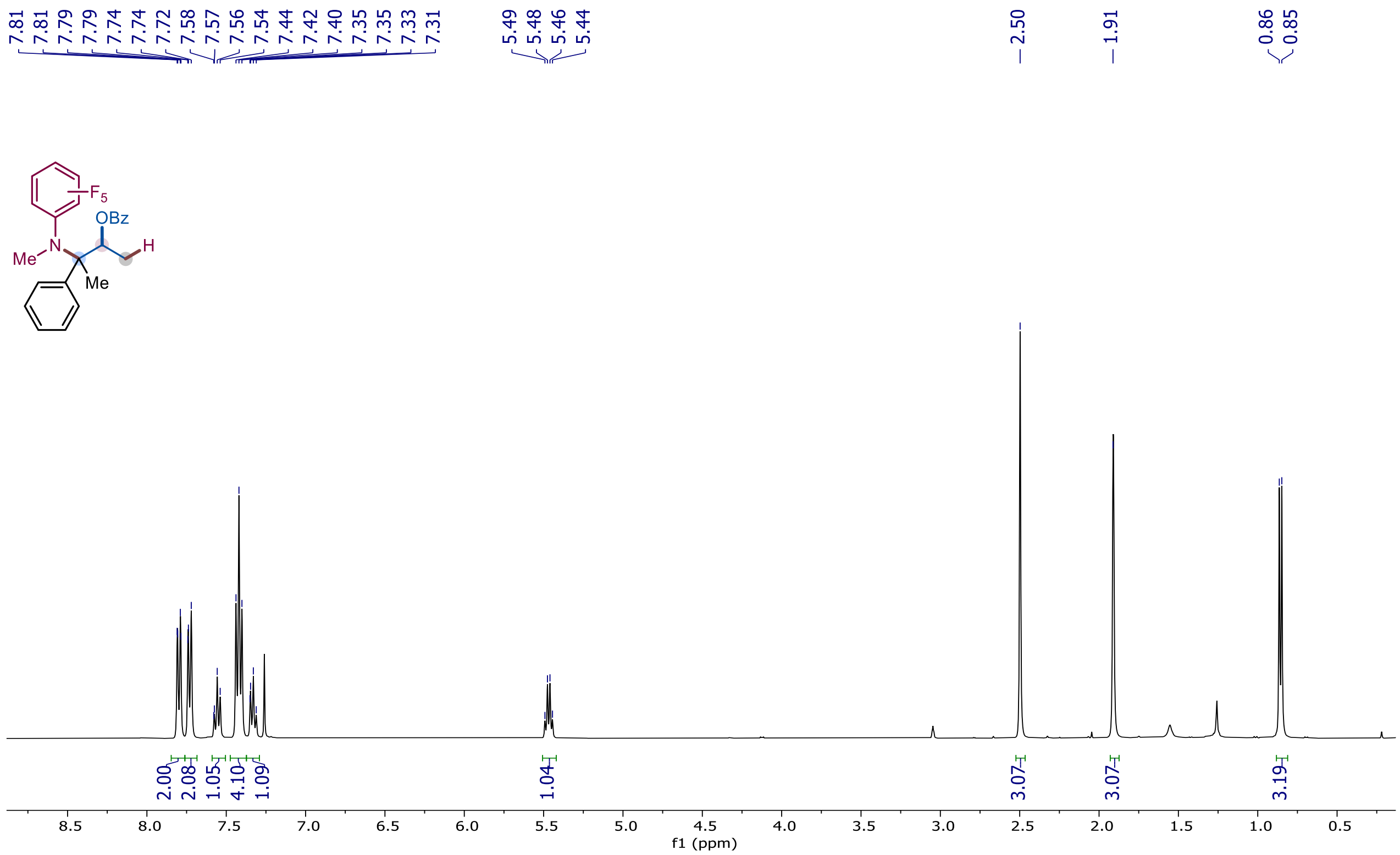

^{13}C NMR (126 MHz, CDCl$_3$, 25 ºC) of 3l

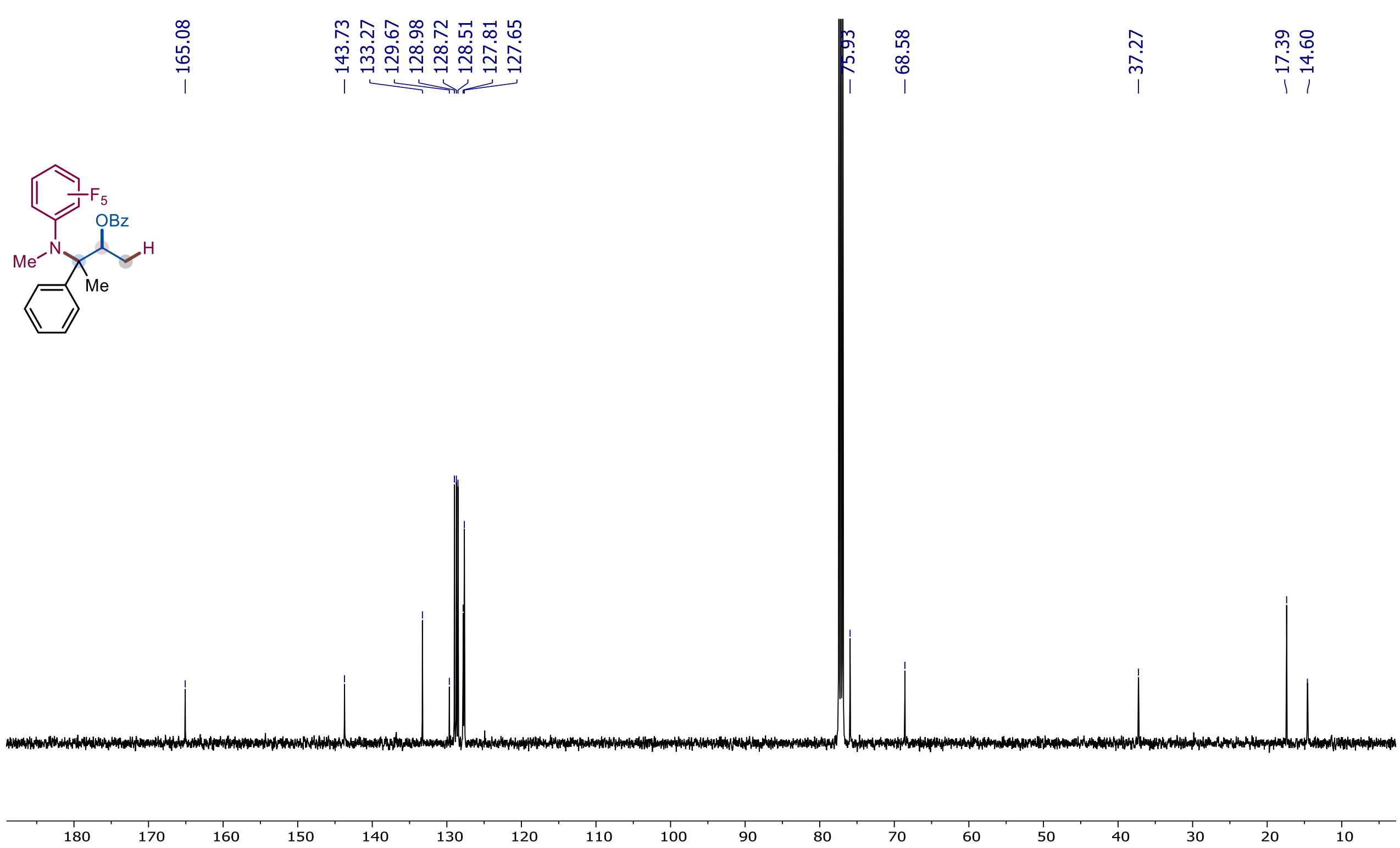

^{19}F NMR (376 MHz, CDCl$_3$, 25 ºC) of 3l

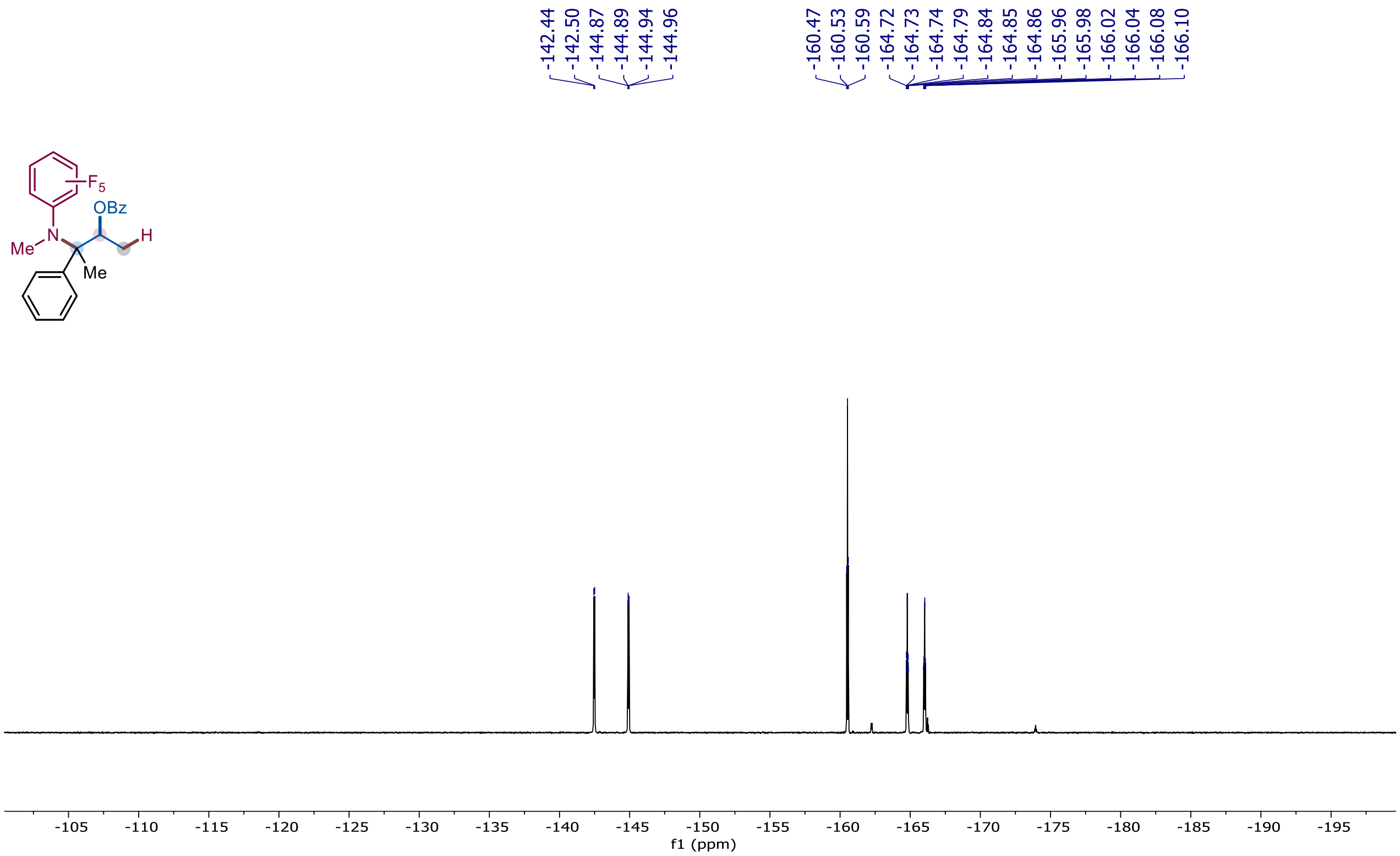

^{1}H NMR (400 MHz, $CDCl_3$, 25 ºC) of 3m

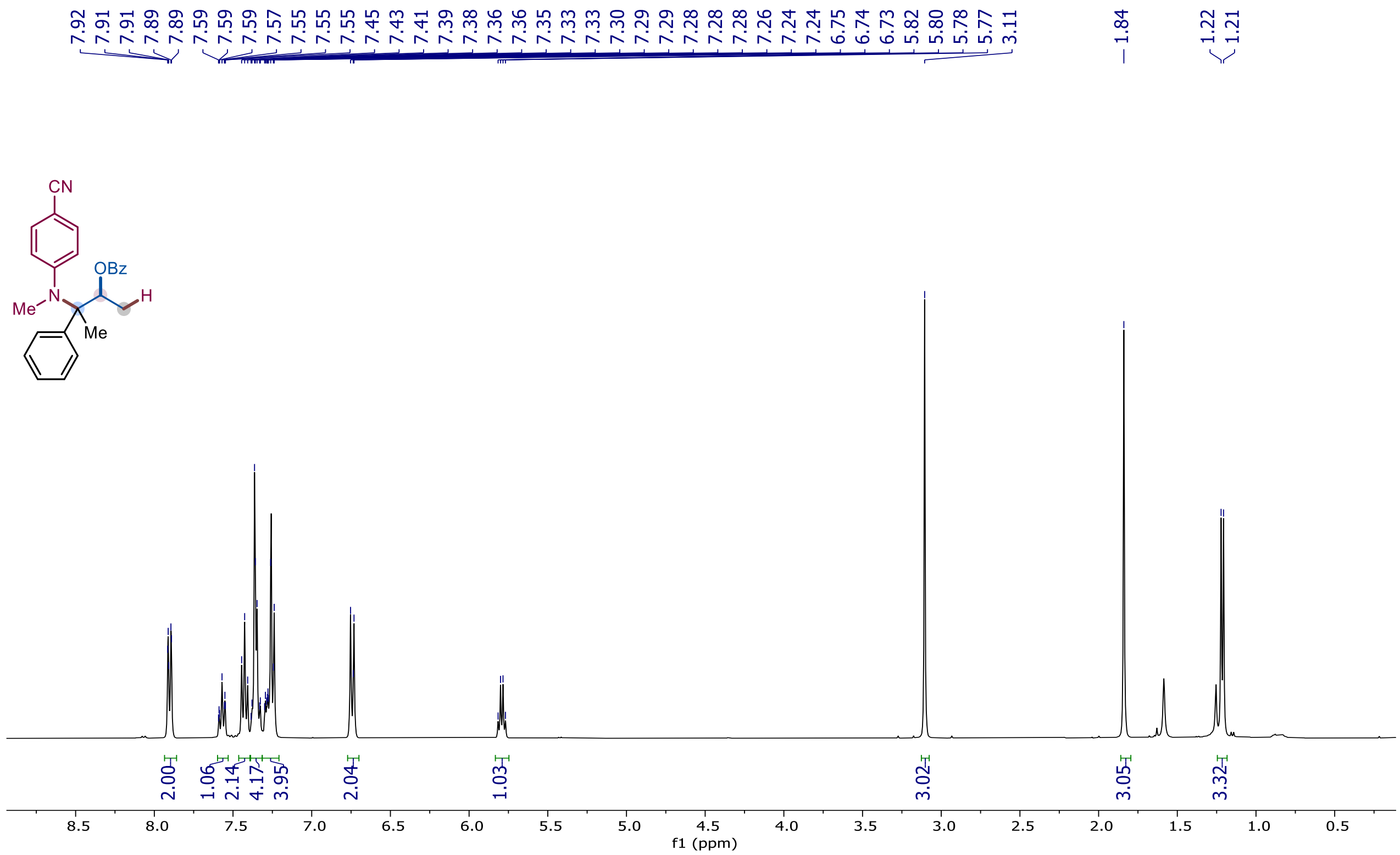

^{13}C NMR (201 MHz, $CDCl_3$, 25 ºC) of 3m

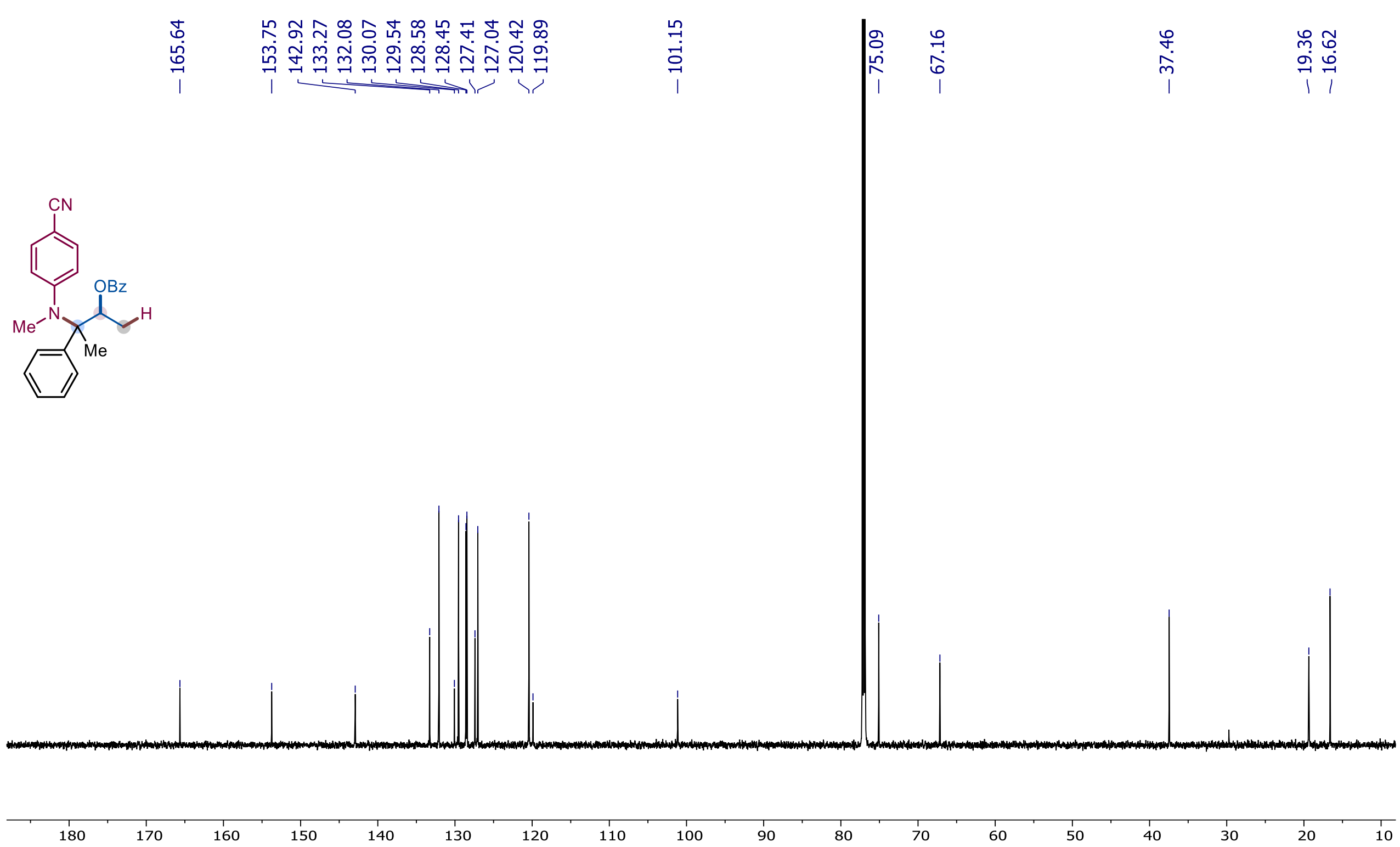

^{1}H NMR (300 MHz, $CDCl_3$, 25 ºC) of 3n

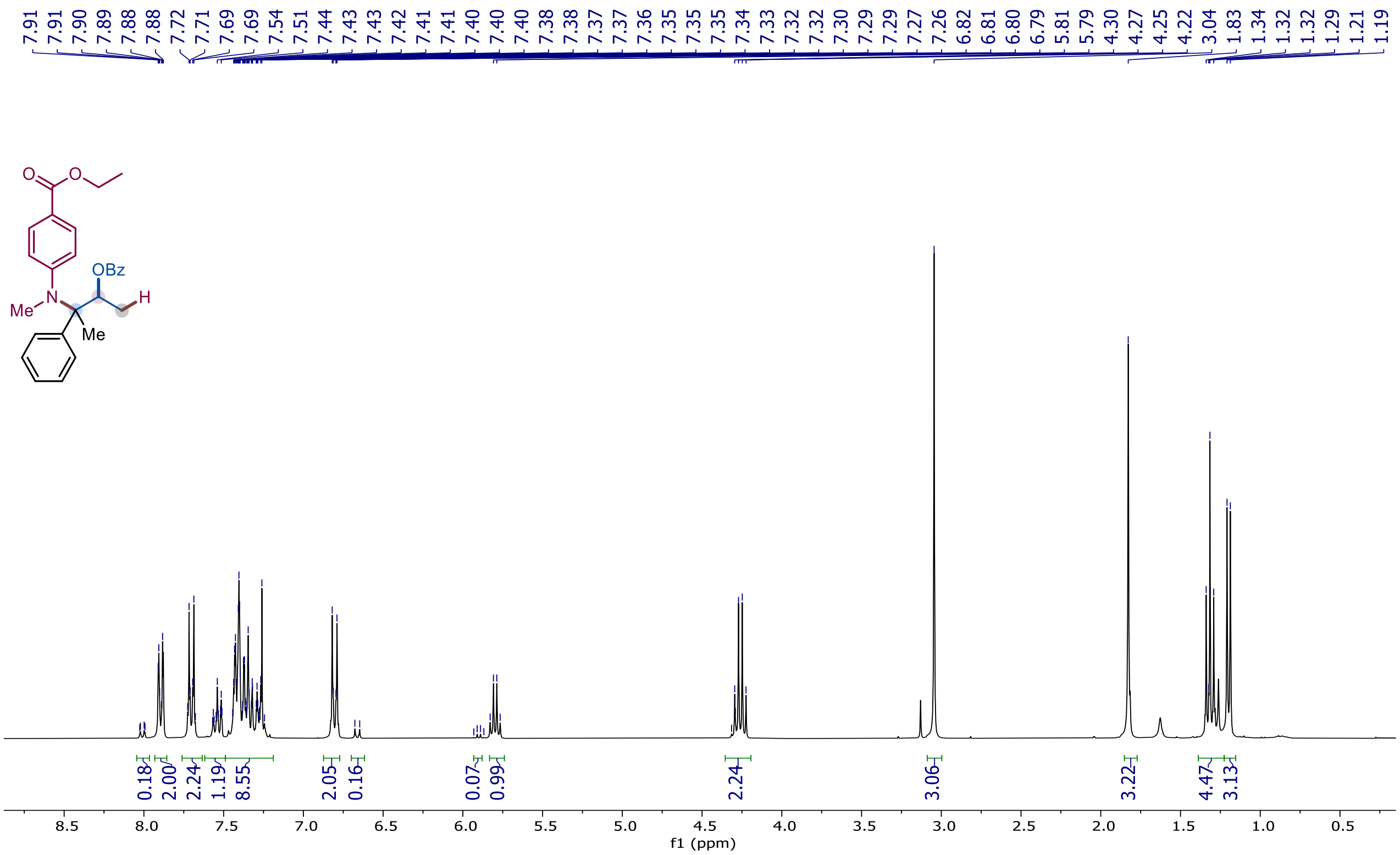

^{13}C NMR (126 MHz, CD_3CN, 25 ºC) of 3n

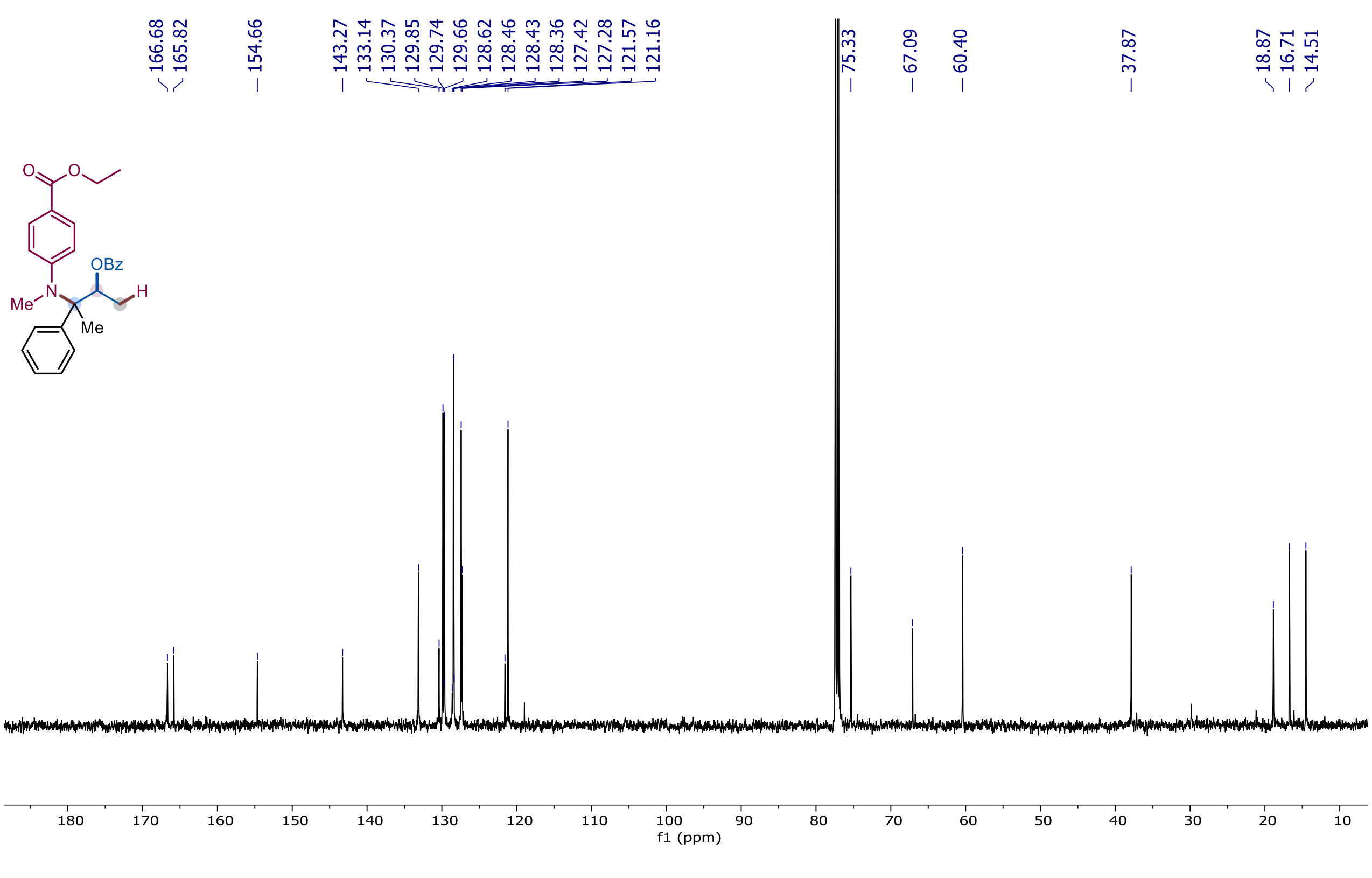

^{1}H NMR (400 MHz, CDCl$_3$, 25 °C) of 3o

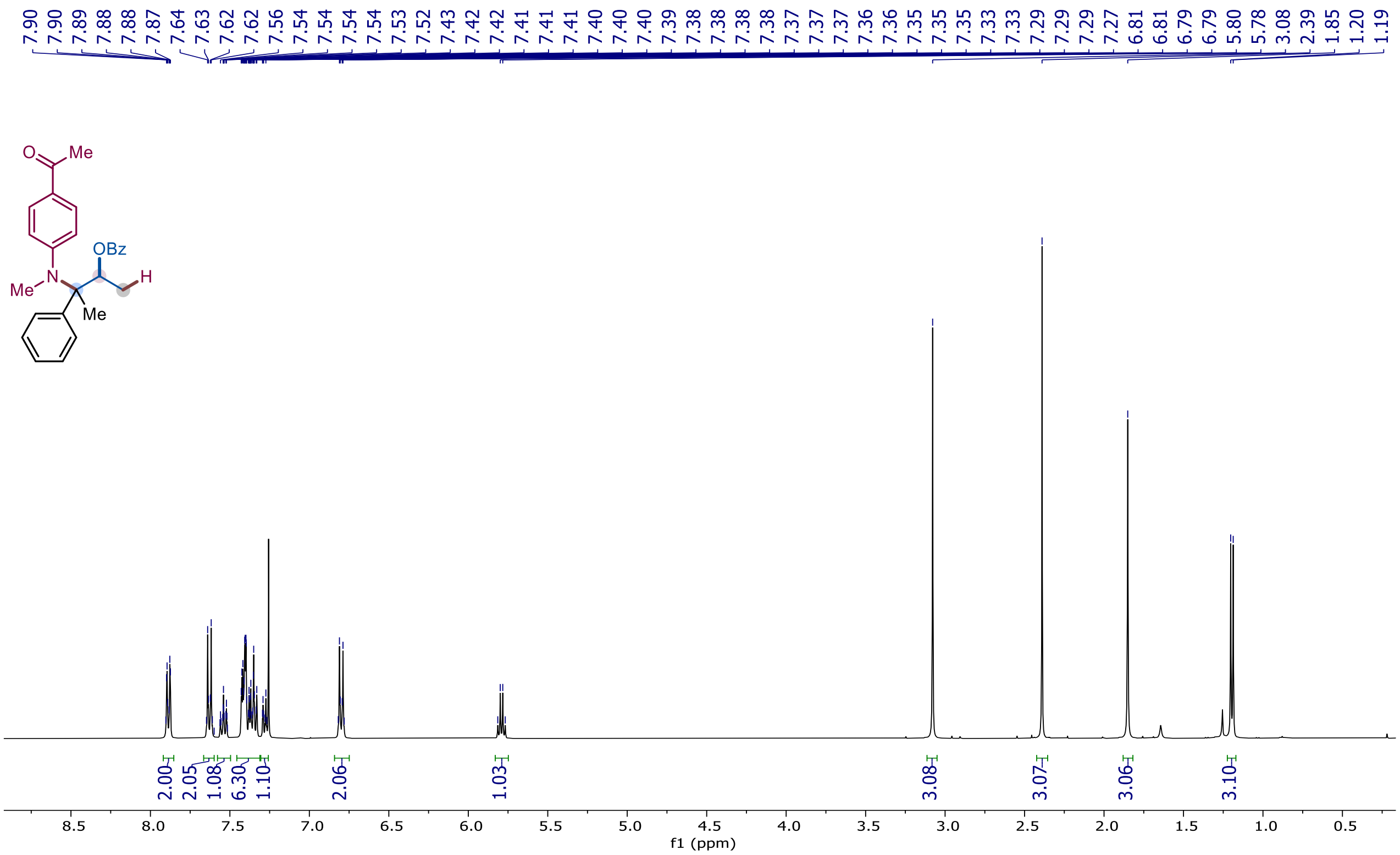

^{13}C NMR (126 MHz, CDCl$_3$, 25 °C) of 3o

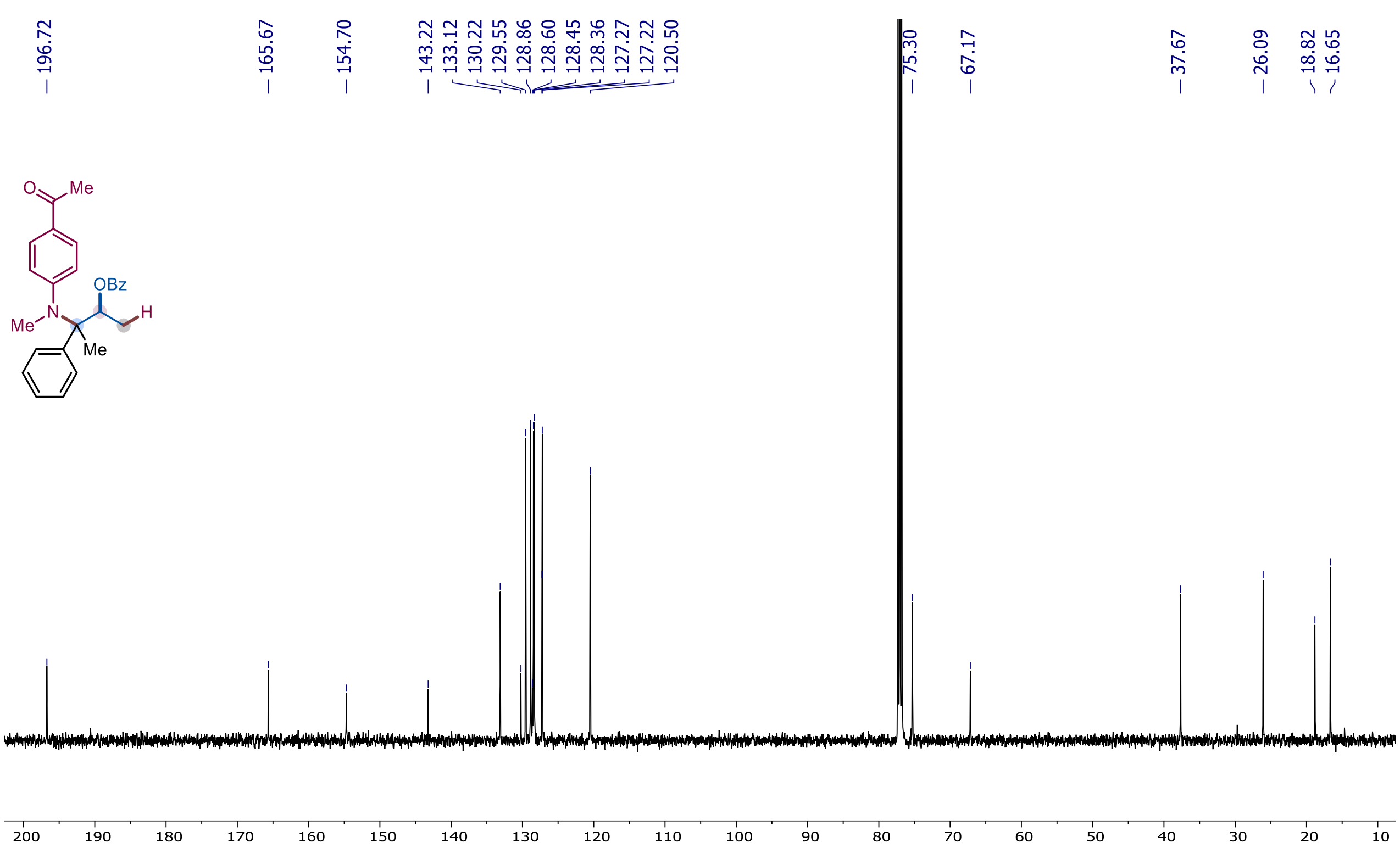

^{1}H NMR (800 MHz, $CDCl_3$, 25 ºC) of 3p

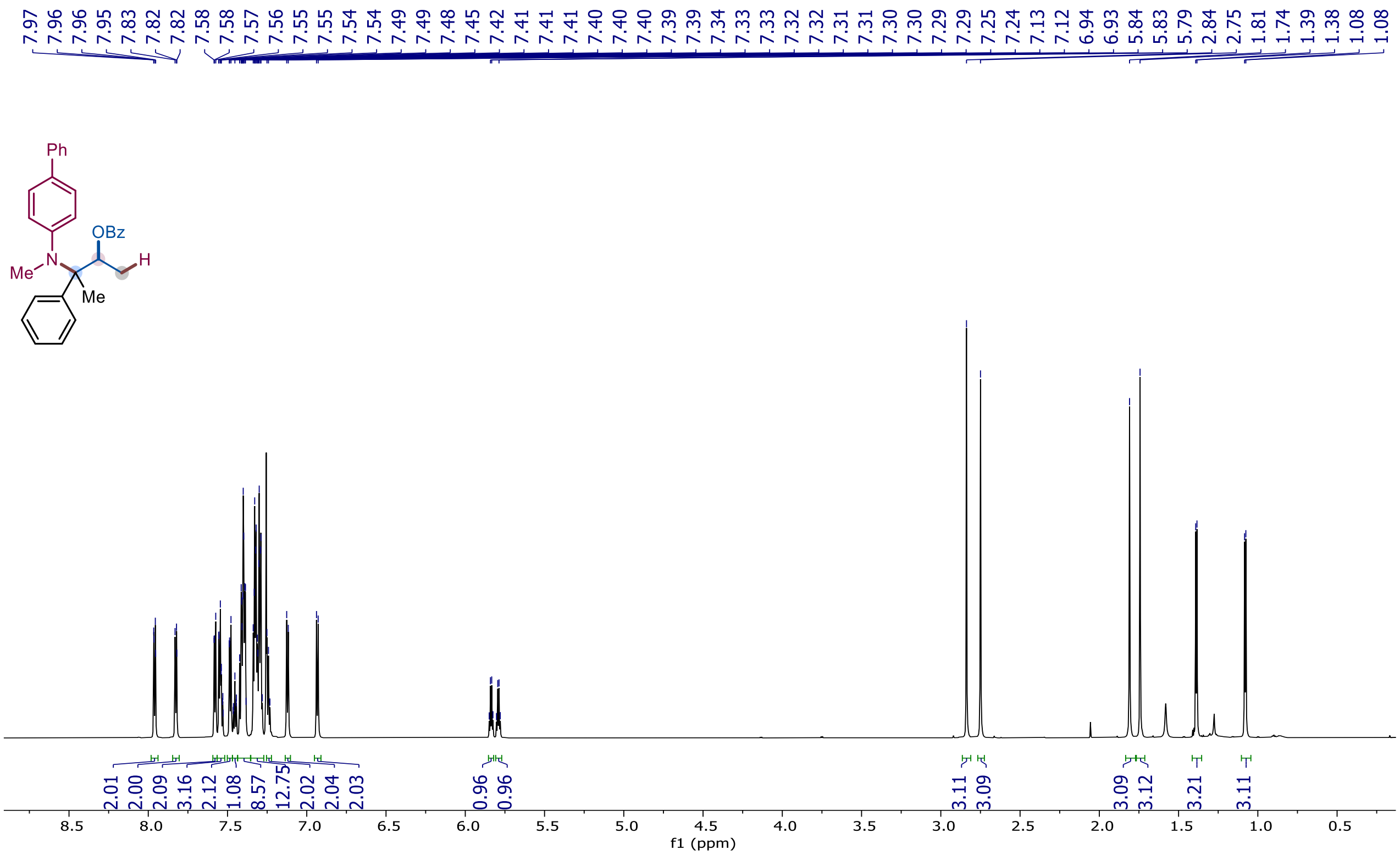

^{13}C NMR (201 MHz, $CDCl_3$, 25 ºC) of 3p

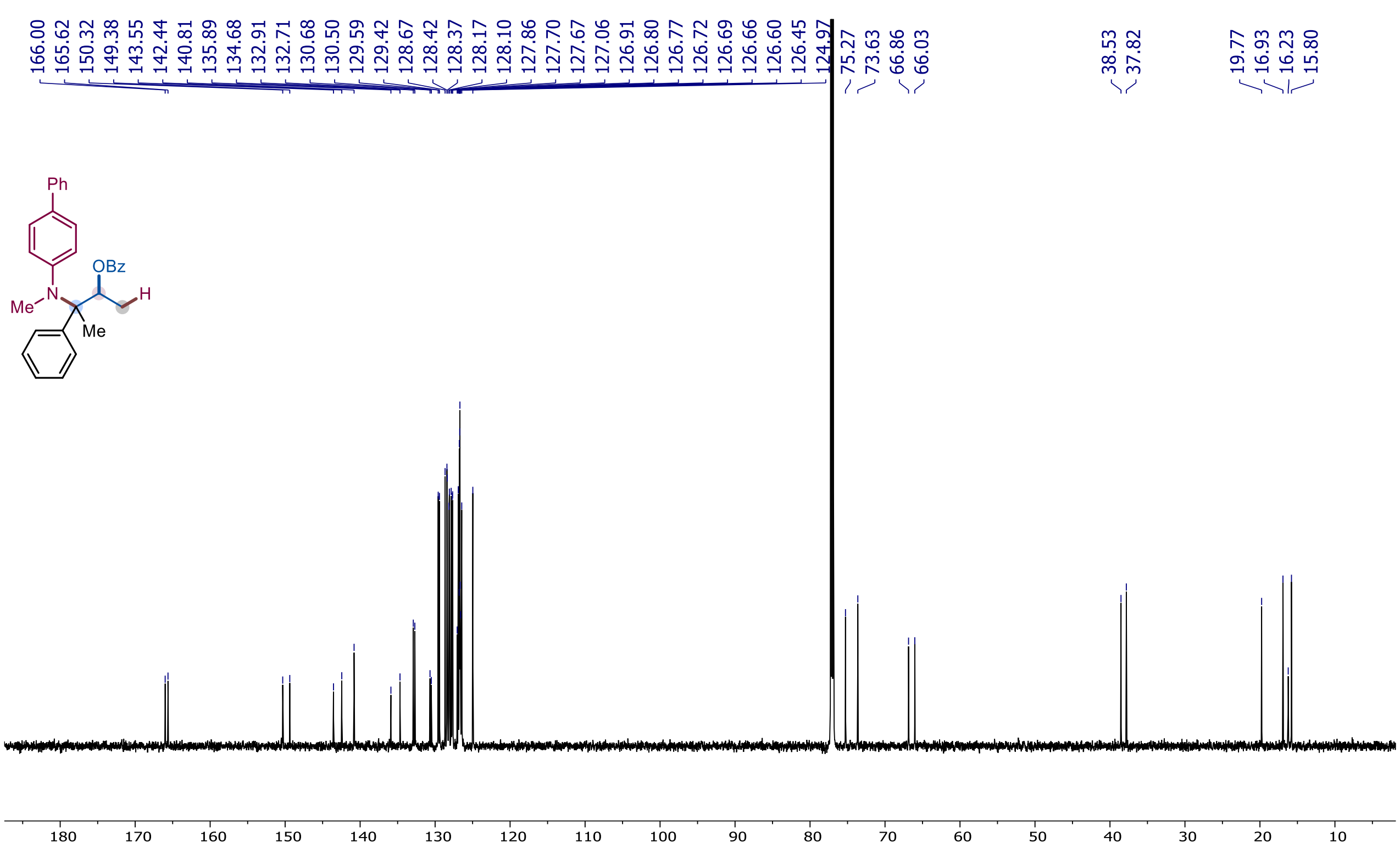

^{1}H NMR (500 MHz, $CDCl_3$, 25 ºC) of 3q

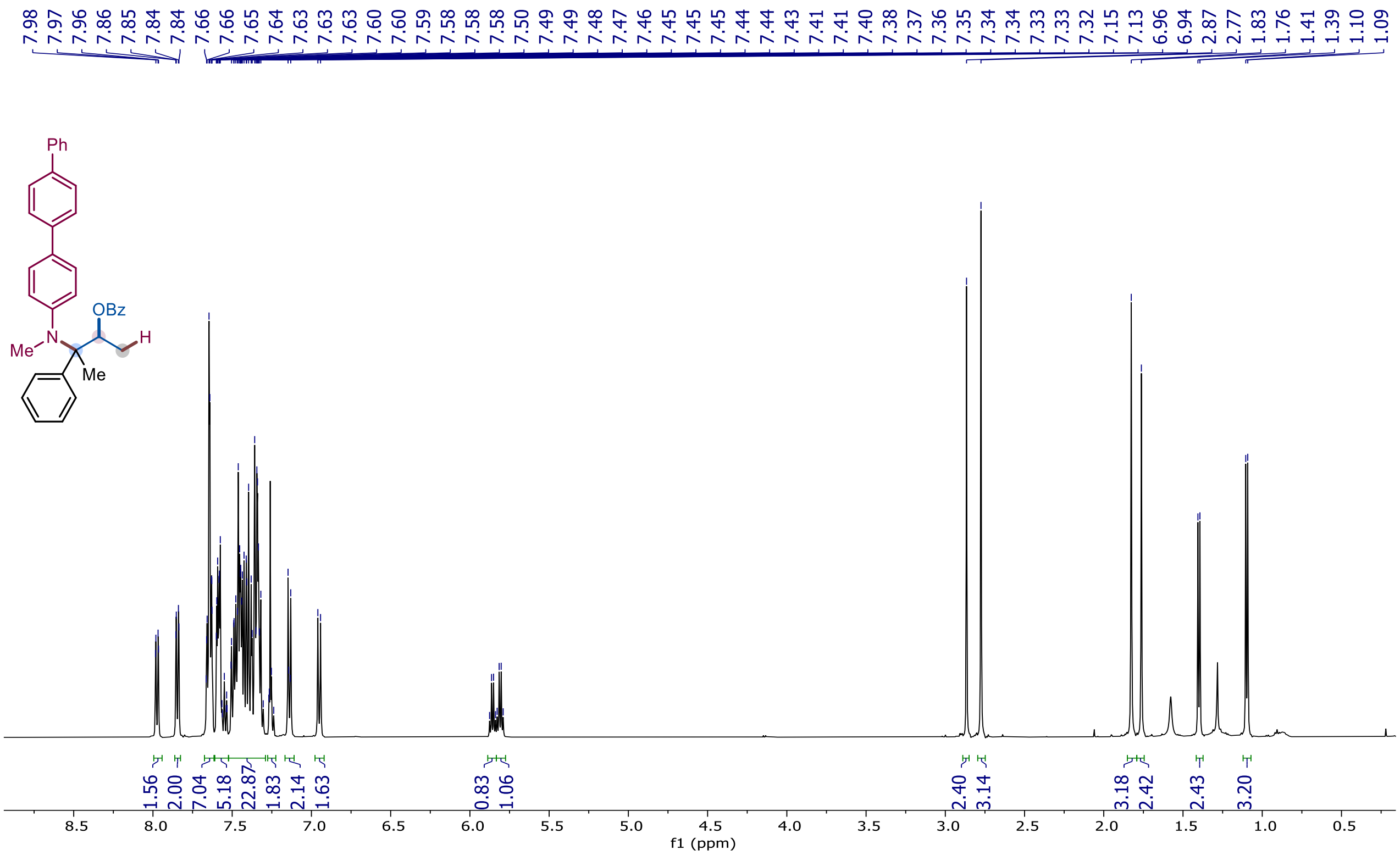

^{13}C NMR (126 MHz, $CDCl_3$, 25 ºC) of 3q

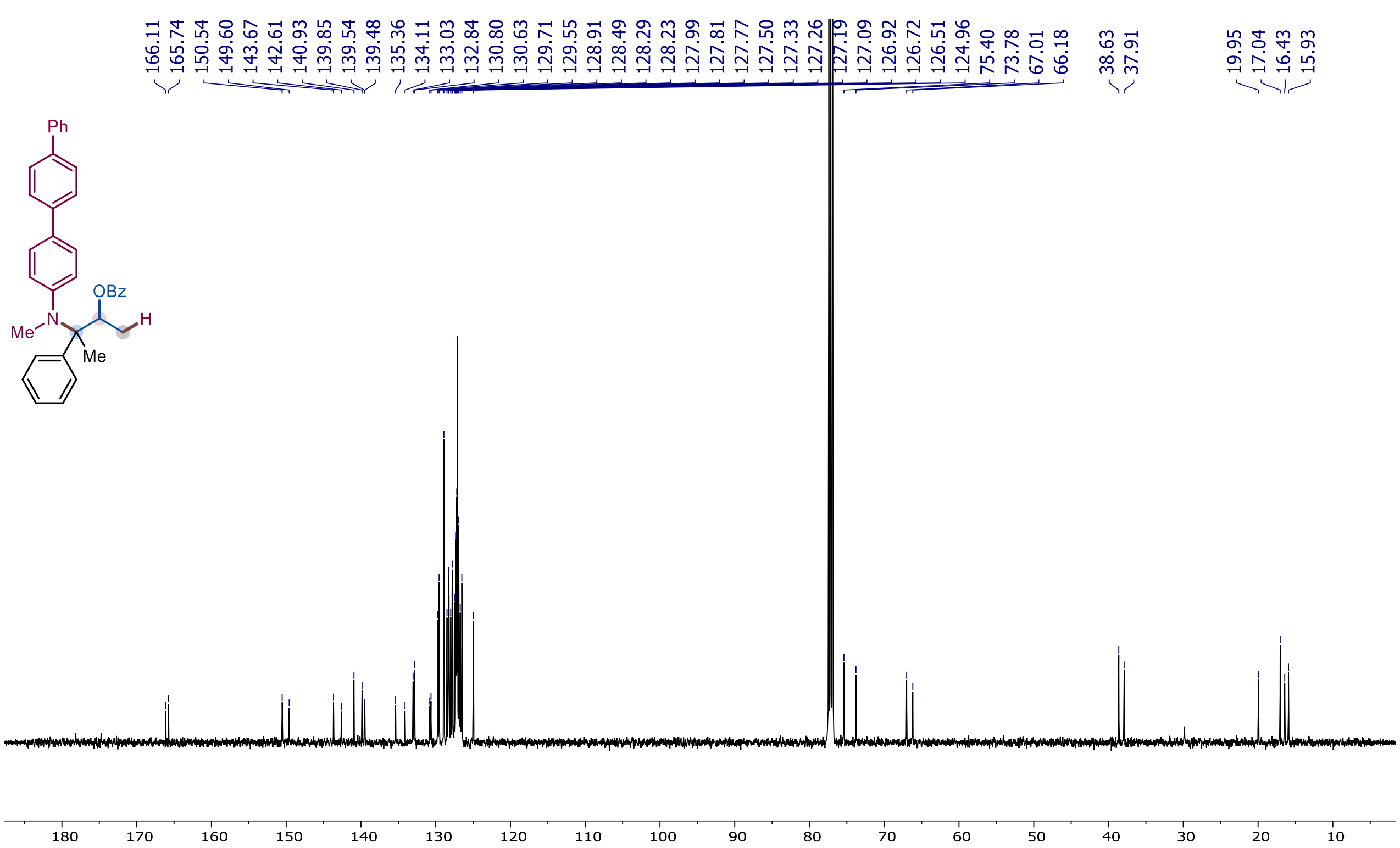

^{1}H NMR (500 MHz, $CDCl_3$, 25 °C) of 3r

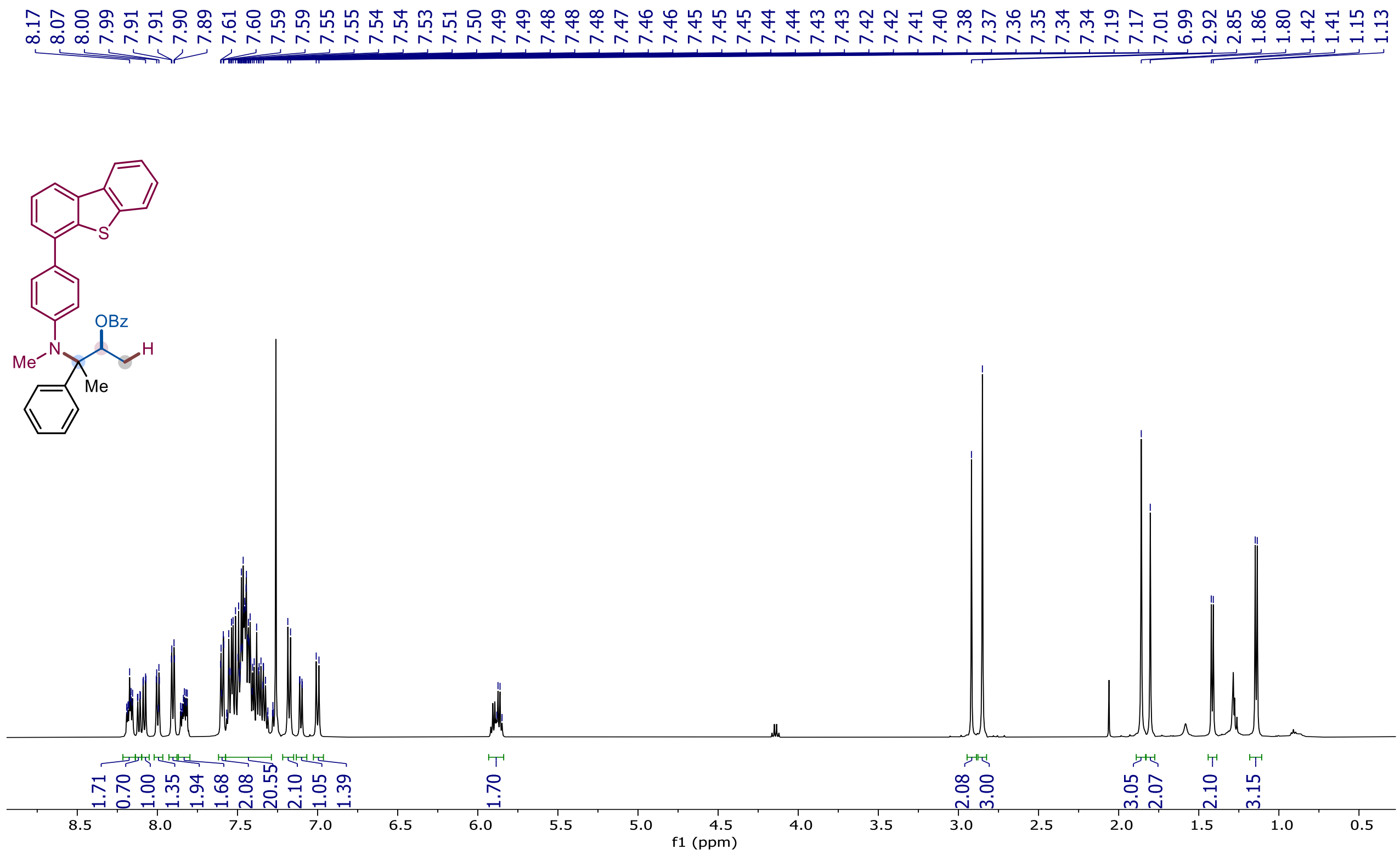

^{13}C NMR (126 MHz, $CDCl_3$, 25 °C) of 3r

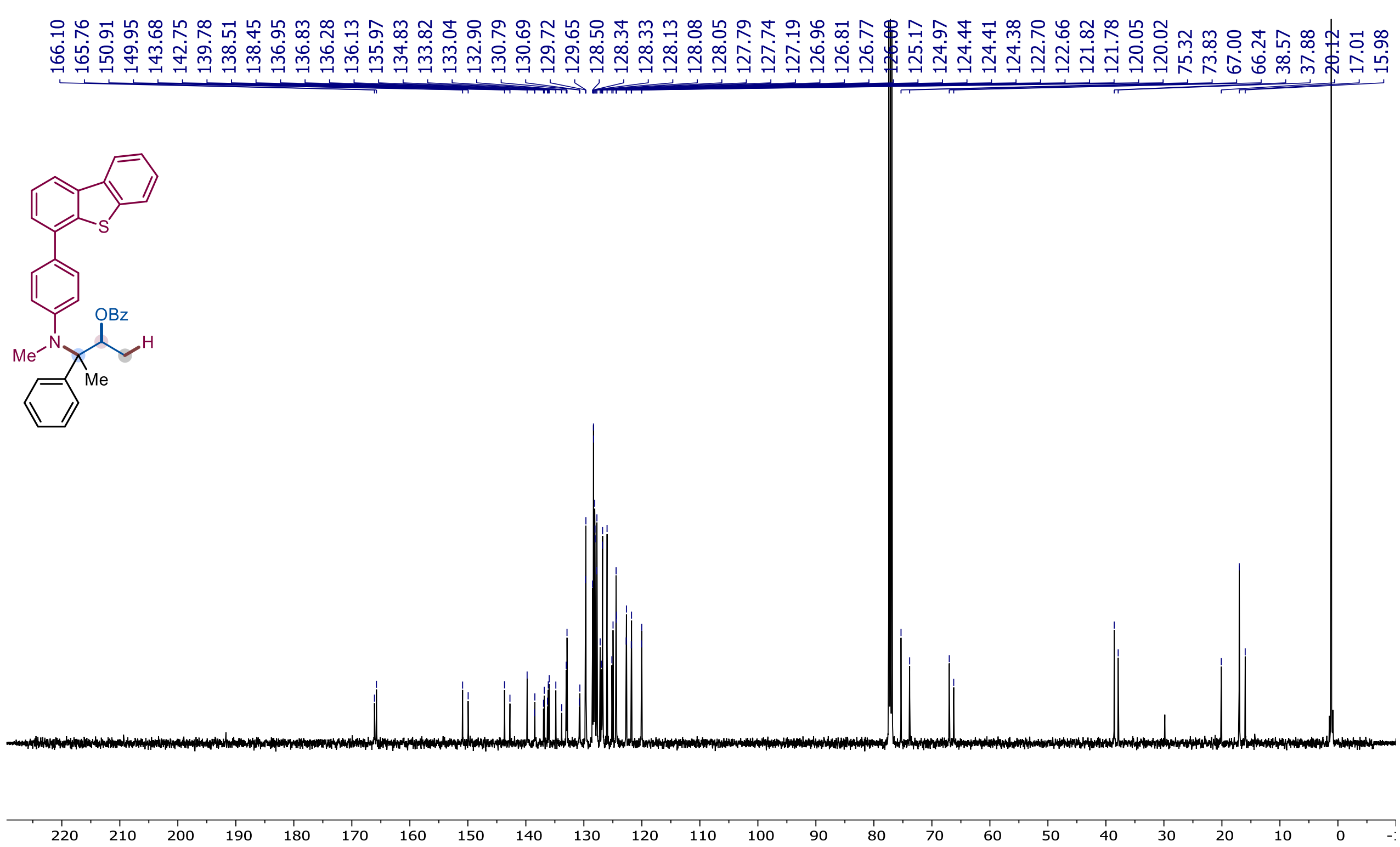

^{1}H NMR (500 MHz, $CDCl_3$, 25 ºC) of 3s

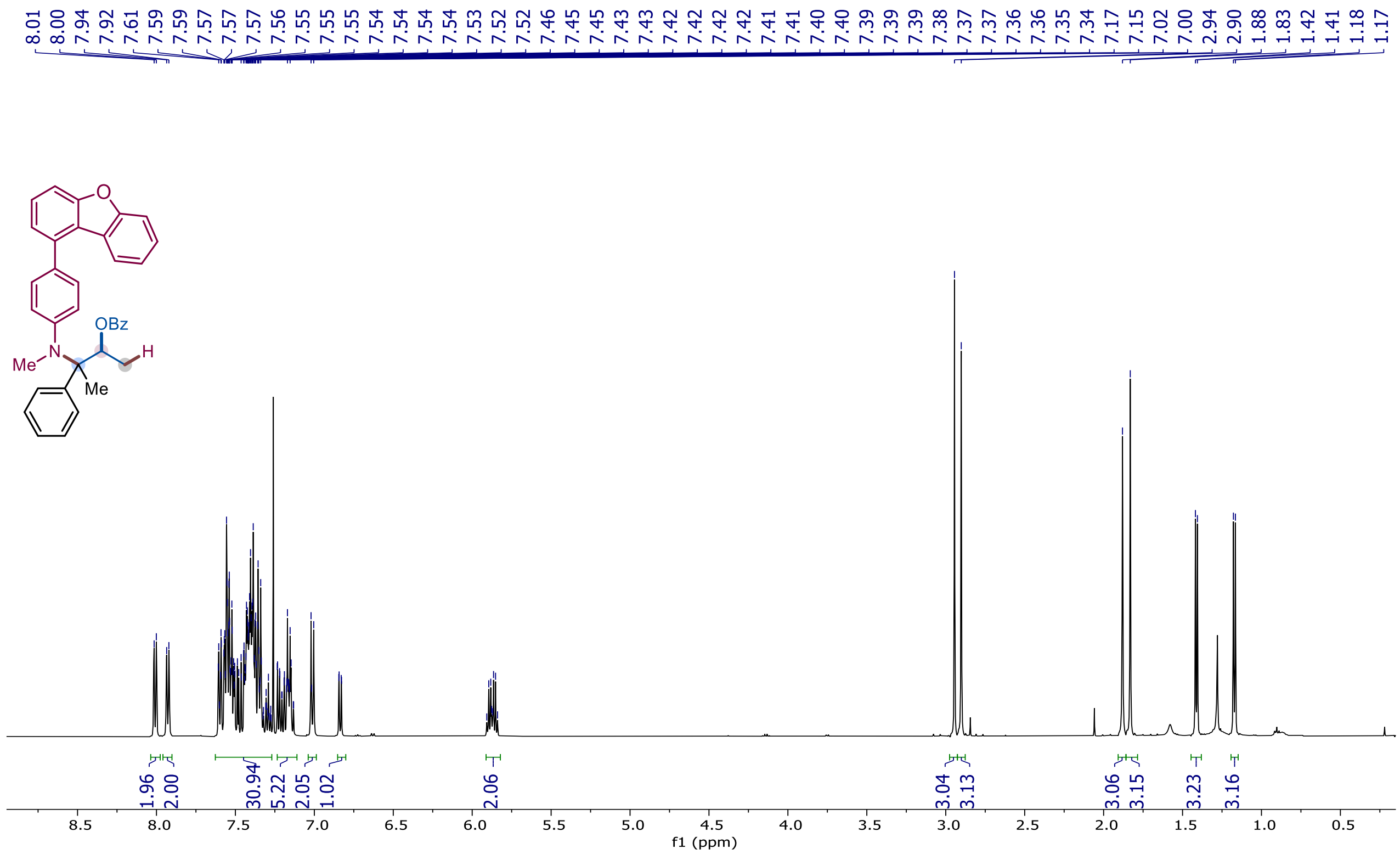

^{13}C NMR (126 MHz, $CDCl_3$, 25 ºC) of 3s

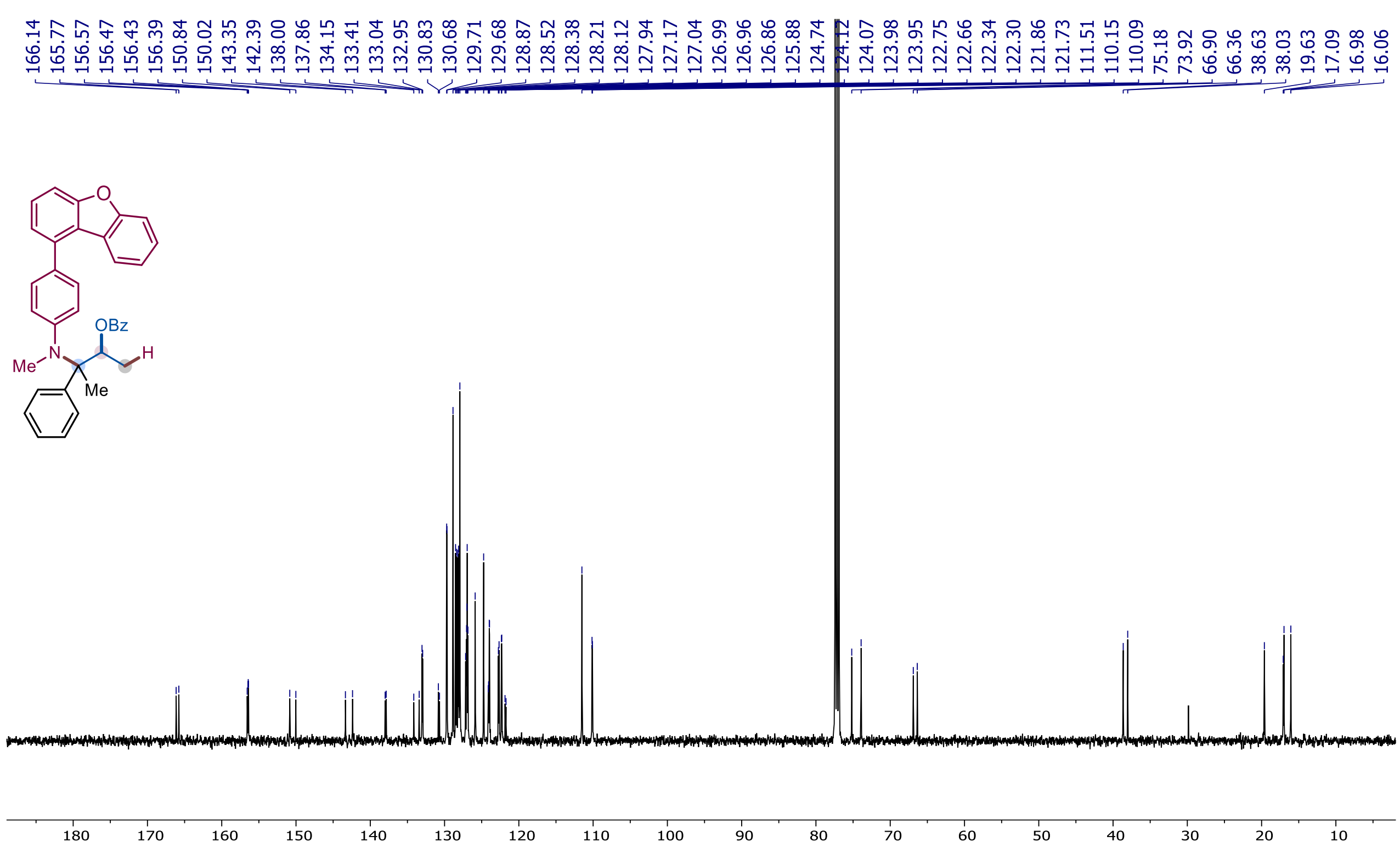

^{1}H NMR (300 MHz, $CDCl_3$, 25 ºC) of 3t

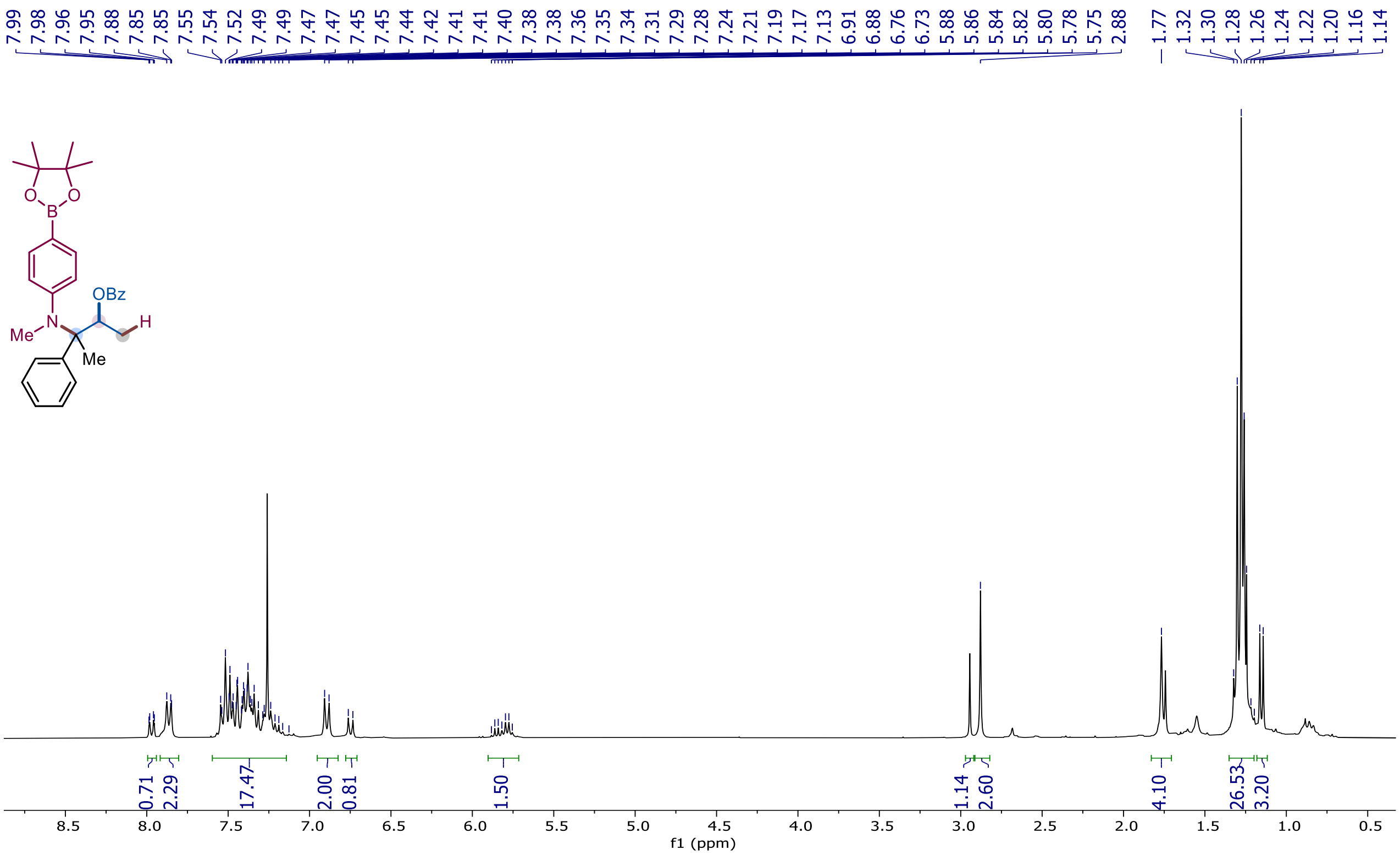

^{13}C NMR (201 MHz, $CDCl_3$, 25 ºC) of 3t

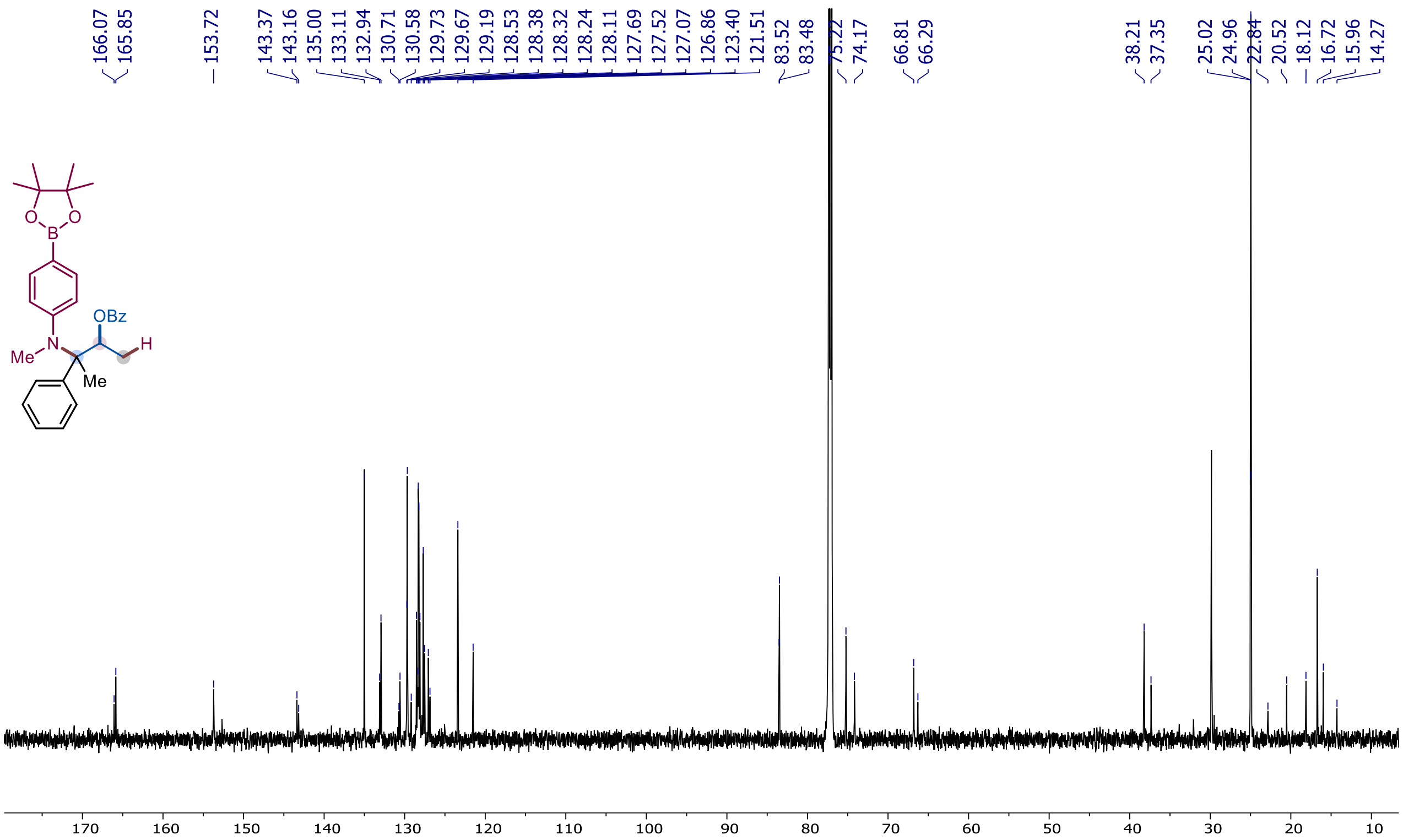

^{1}H NMR (500 MHz, $CDCl_3$, 25 °C) of 3u

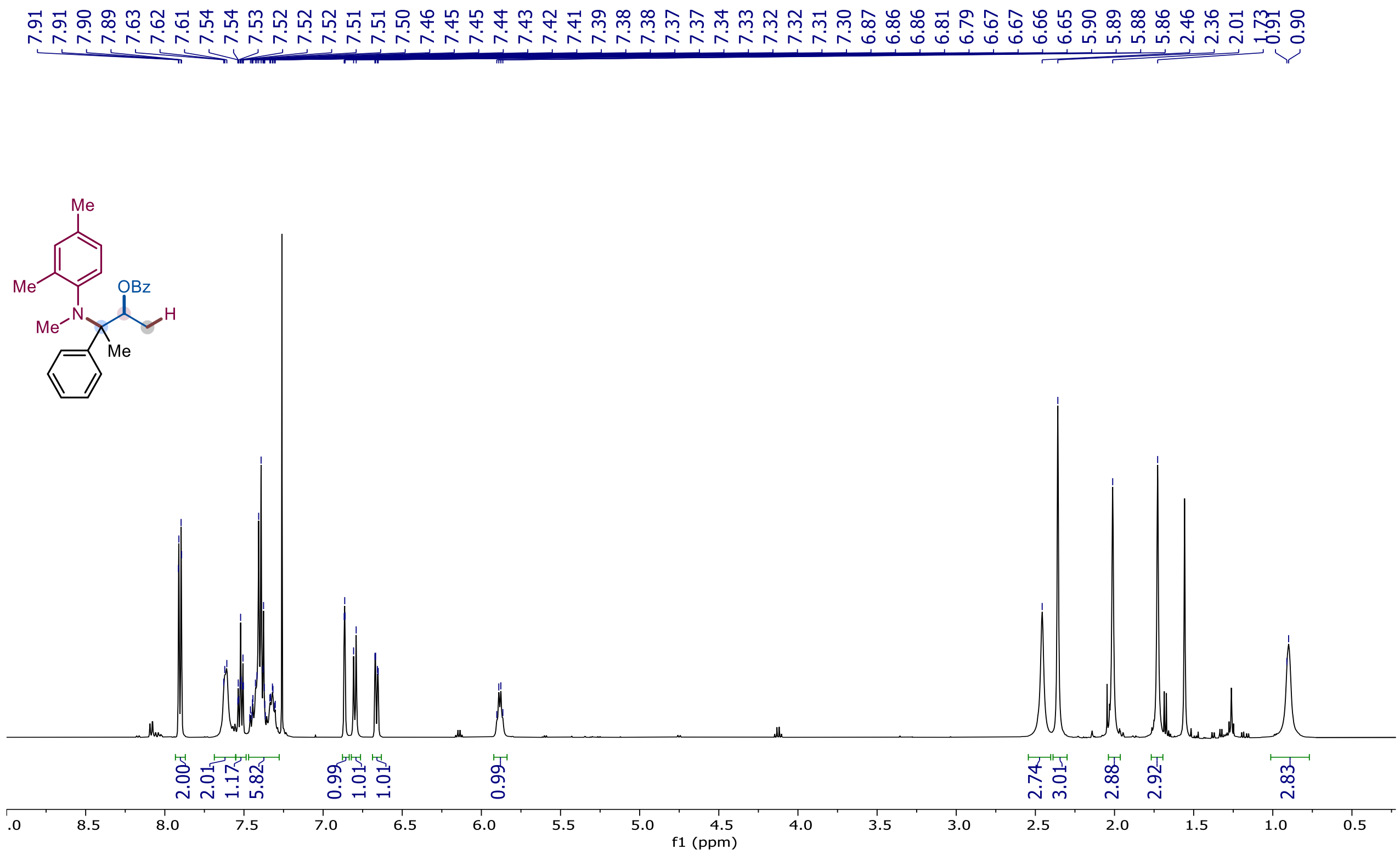

^{13}C NMR (201 MHz, $CDCl_3$, 25 °C) of 3u

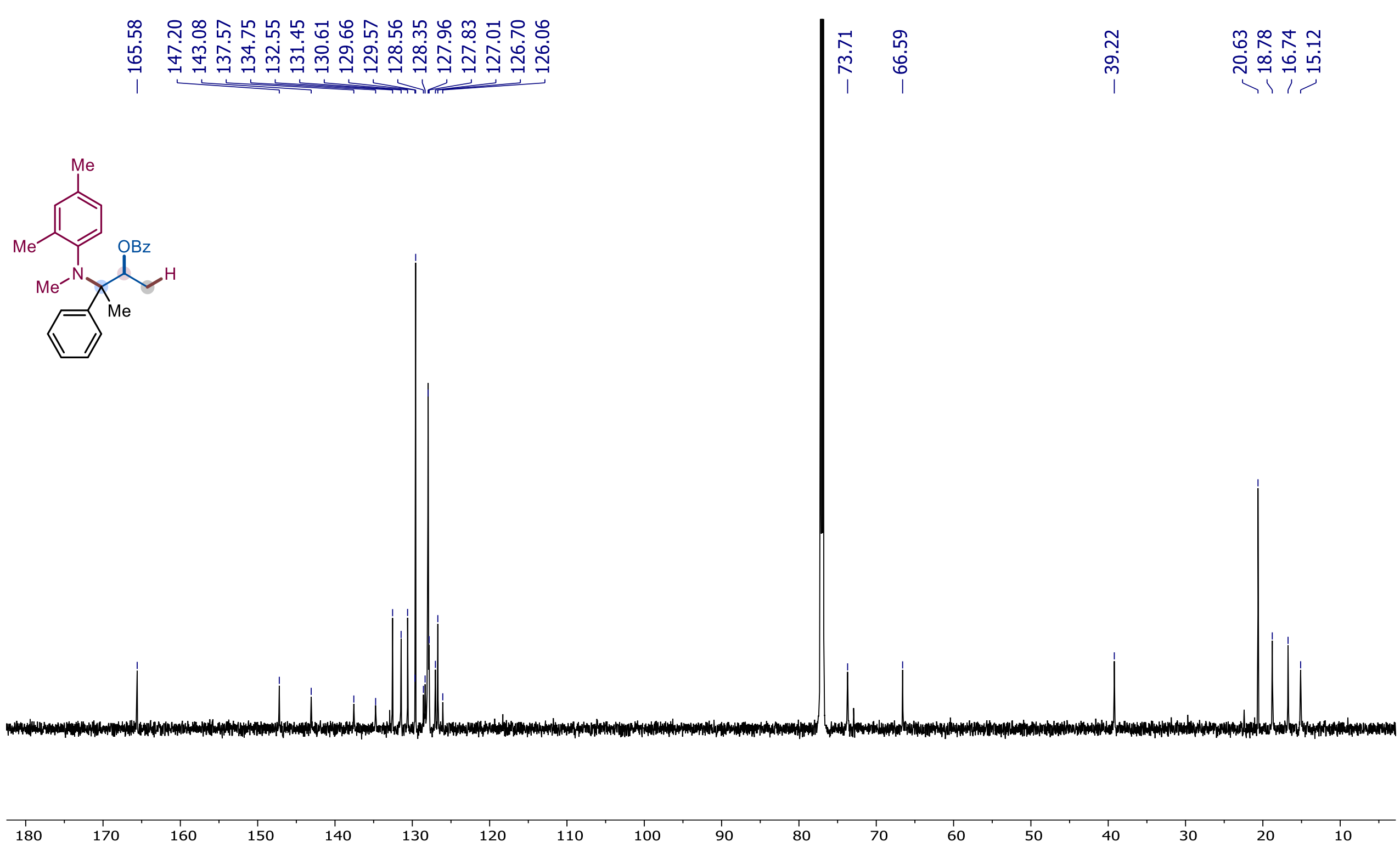

^{1}H NMR (800 MHz, $CDCl_3$, 25 ºC) of 3v

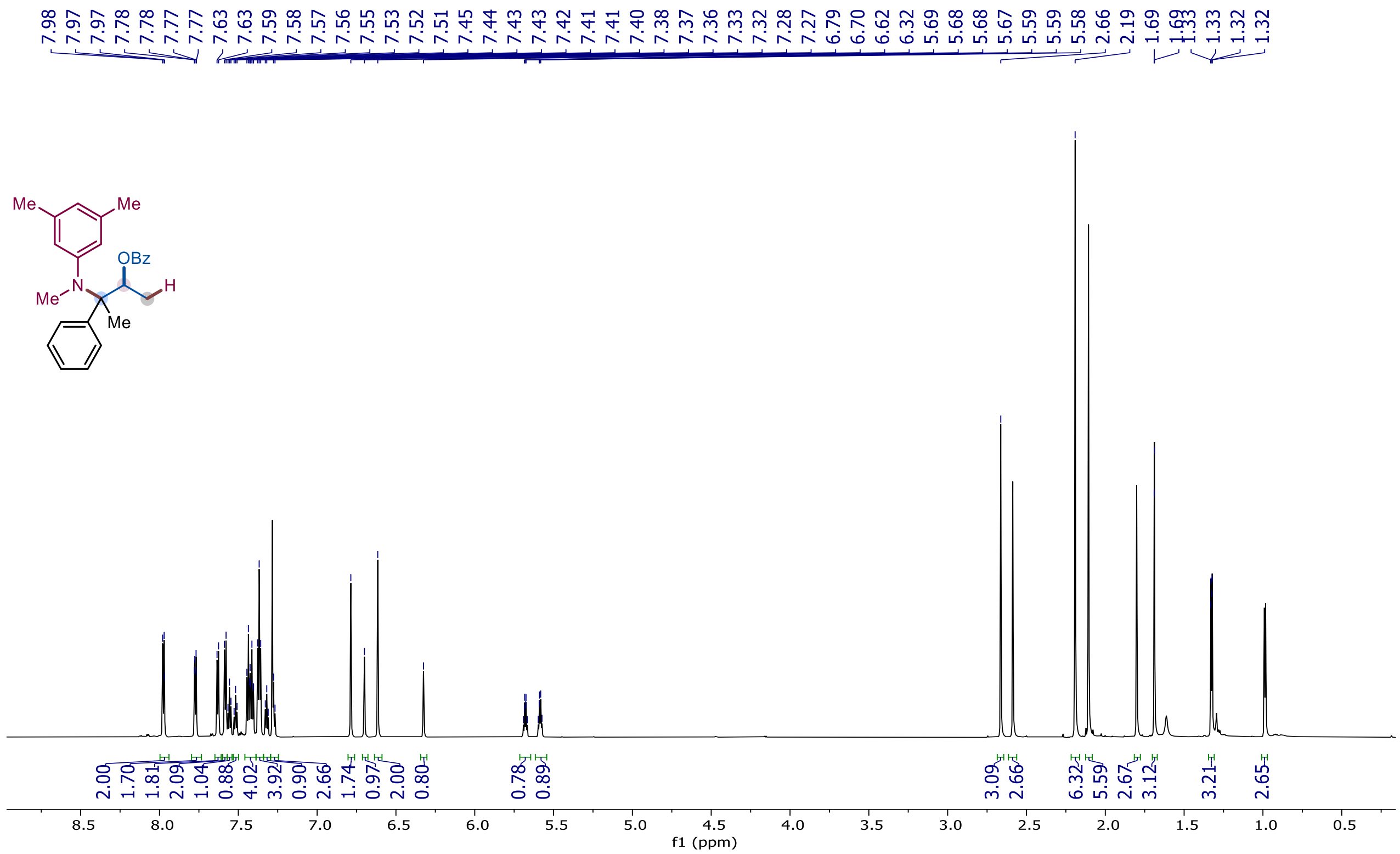

^{13}C NMR (201 MHz, $CDCl_3$, 25 ºC) of 3v

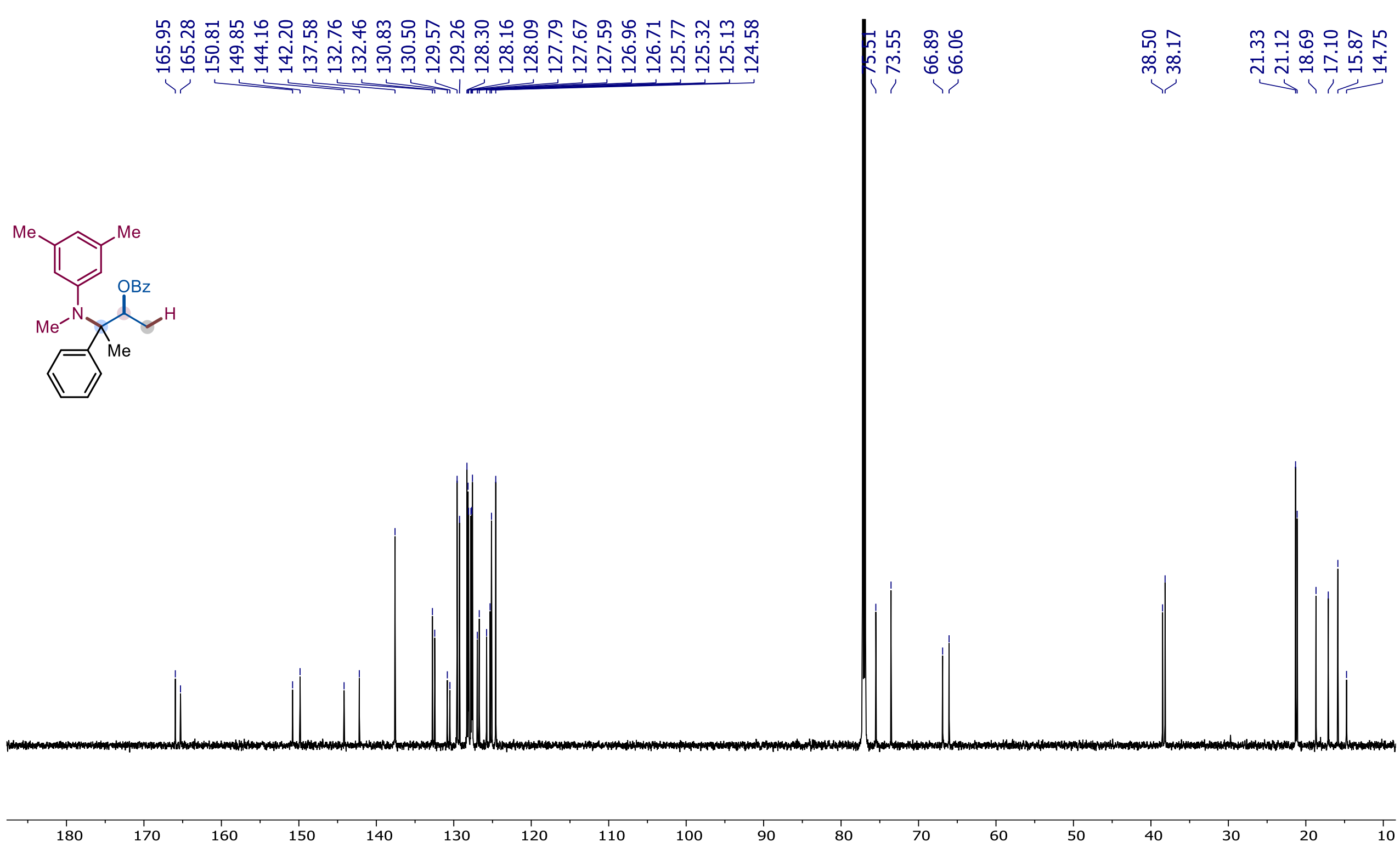

^{1}H NMR (300 MHz, $CDCl_3$, 25 °C) of 3w

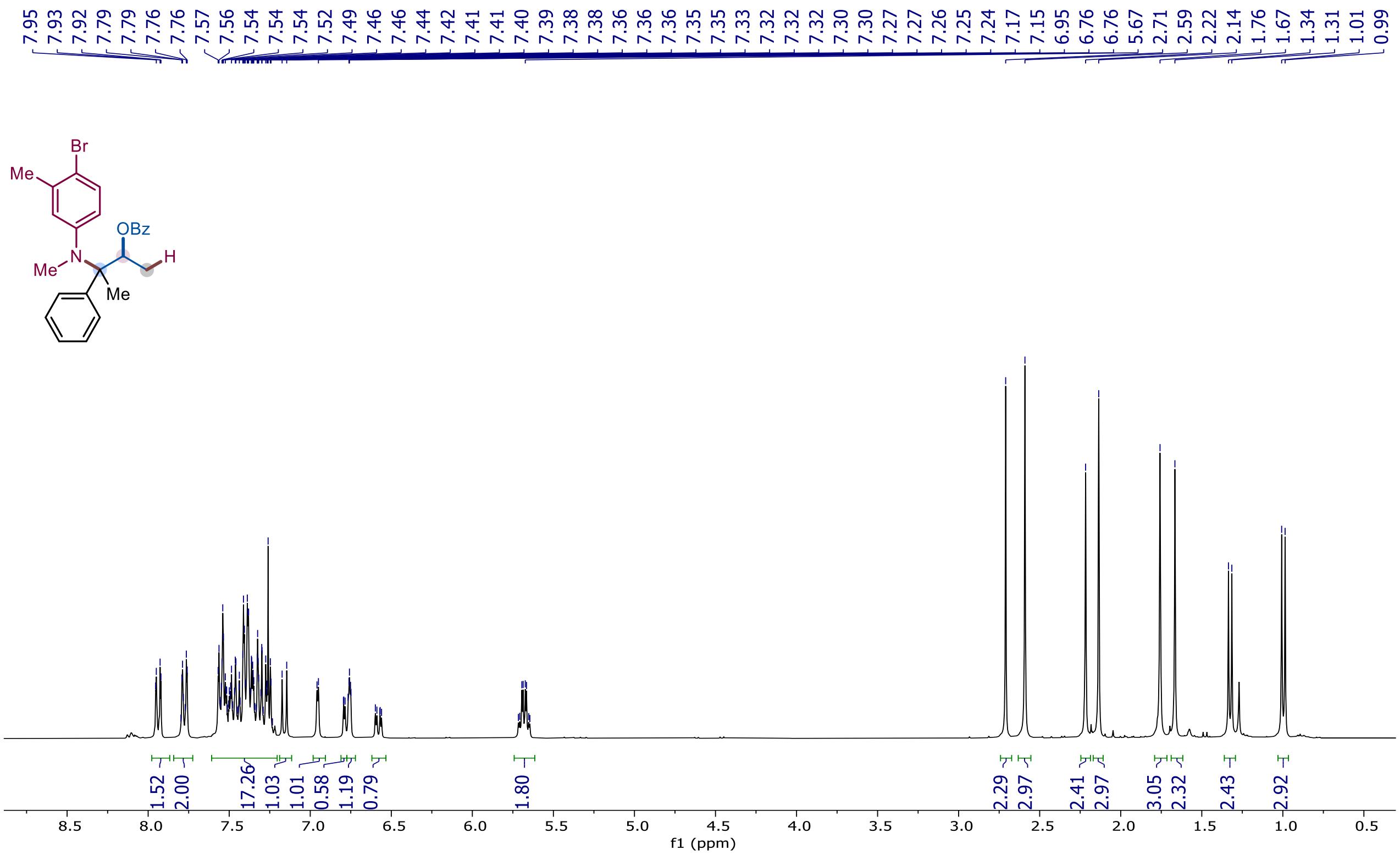

^{13}C NMR (201 MHz, $CDCl_3$, 25 °C) of 3w

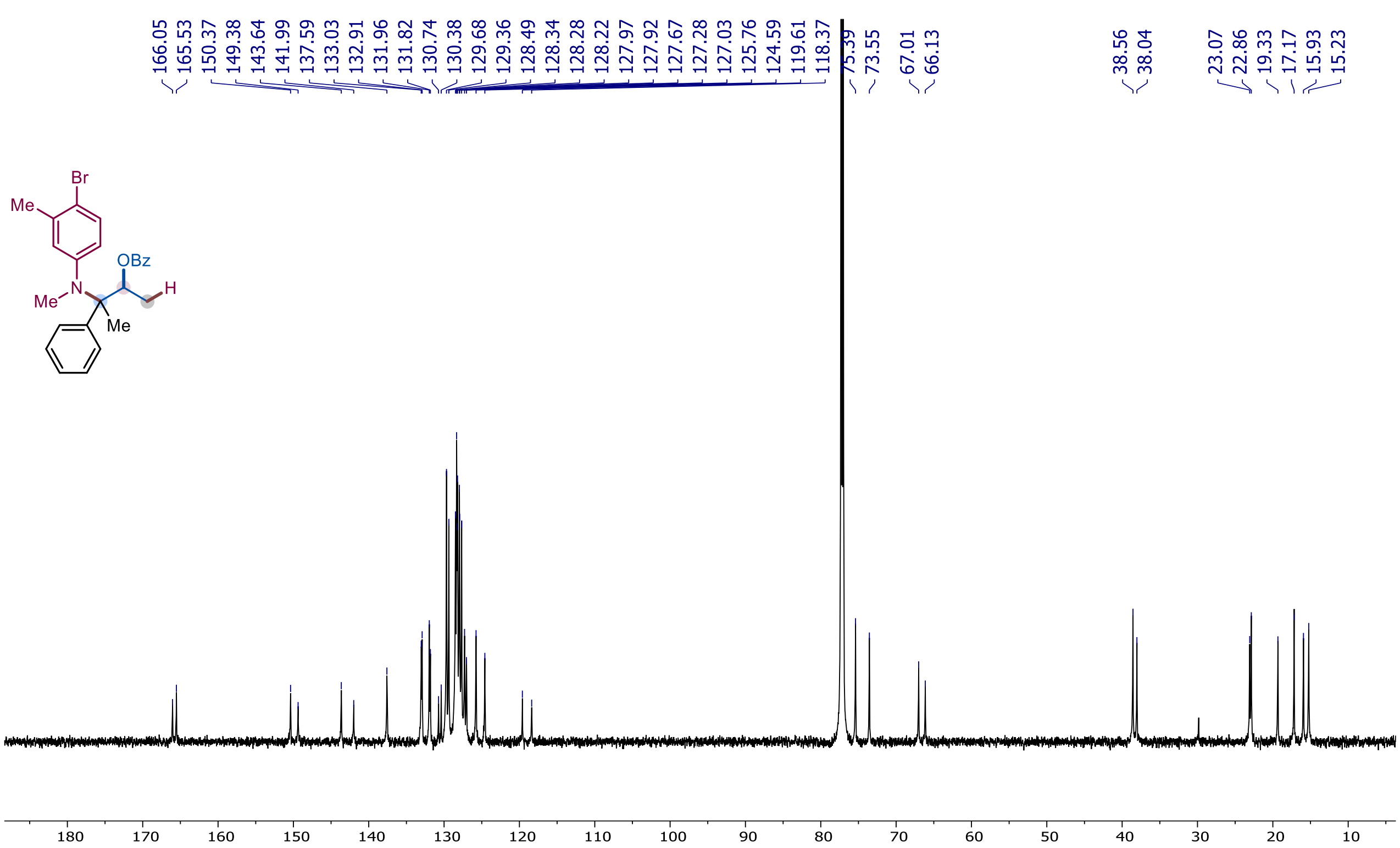

^{1}H NMR (500 MHz, $CDCl_3$, 25 ºC) of 3x

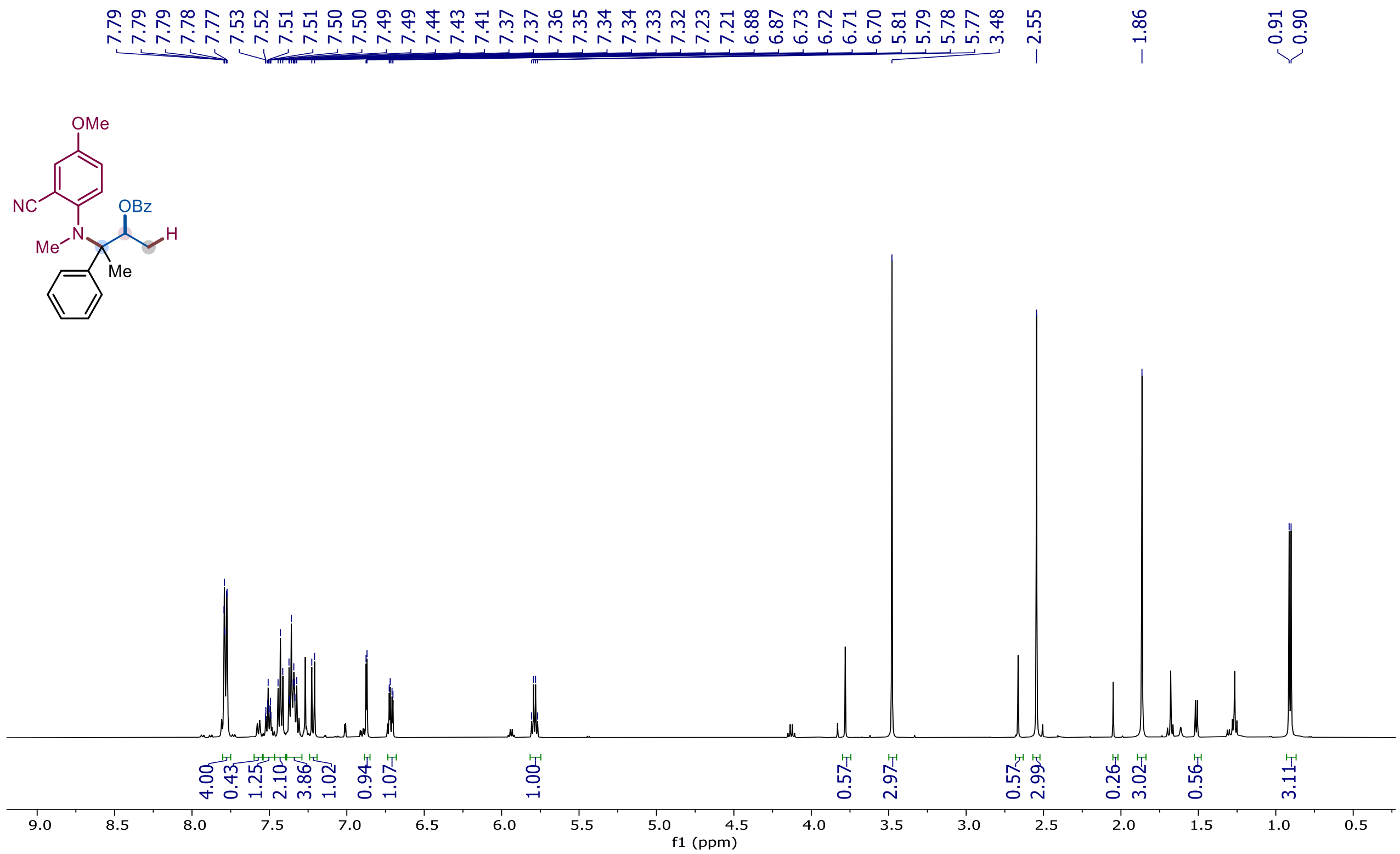

^{13}C NMR (126 MHz, $CDCl_3$, 25 ºC) of 3x

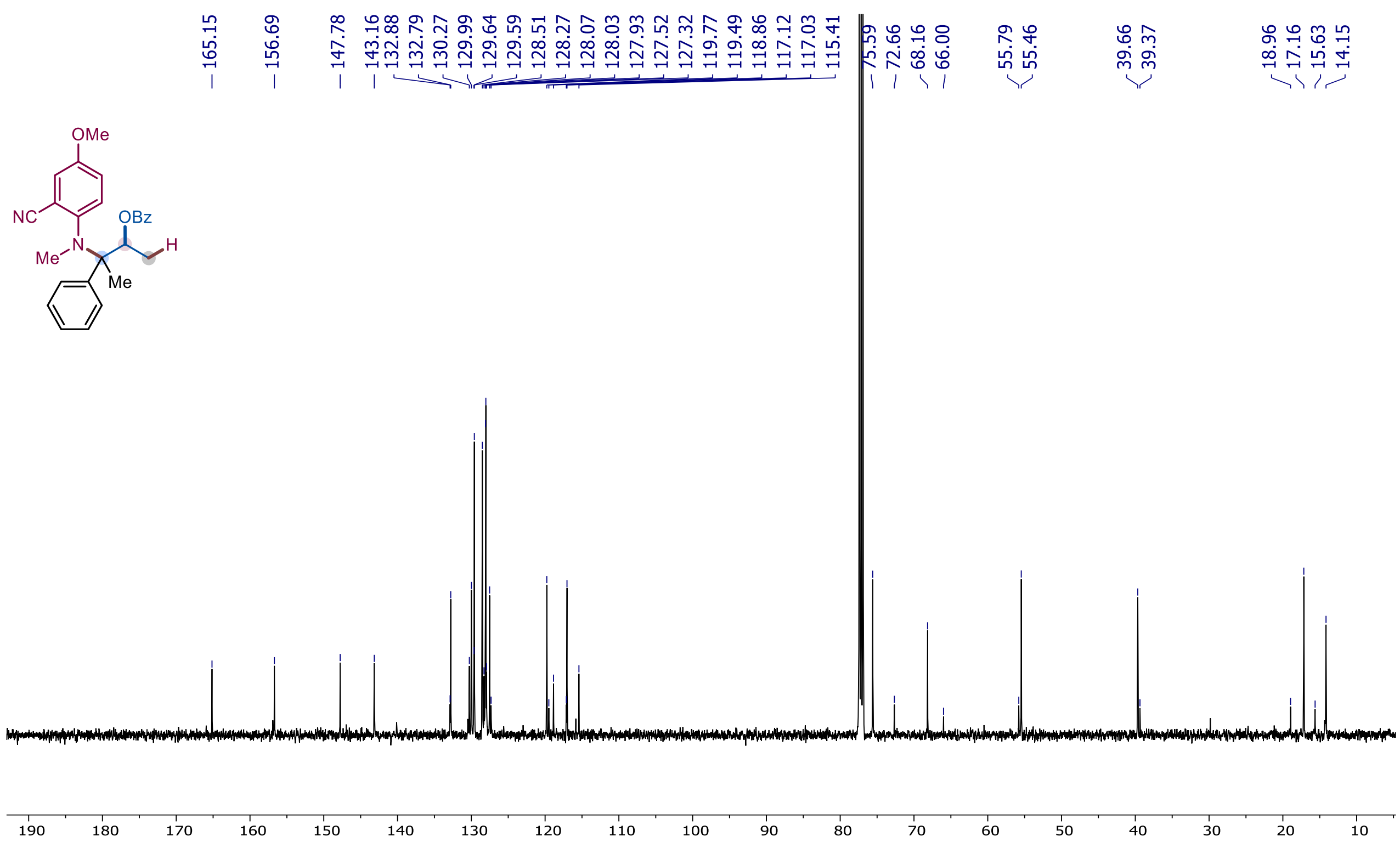

^{1}H NMR (800 MHz, CDCl₃, 25 ºC) of 3y

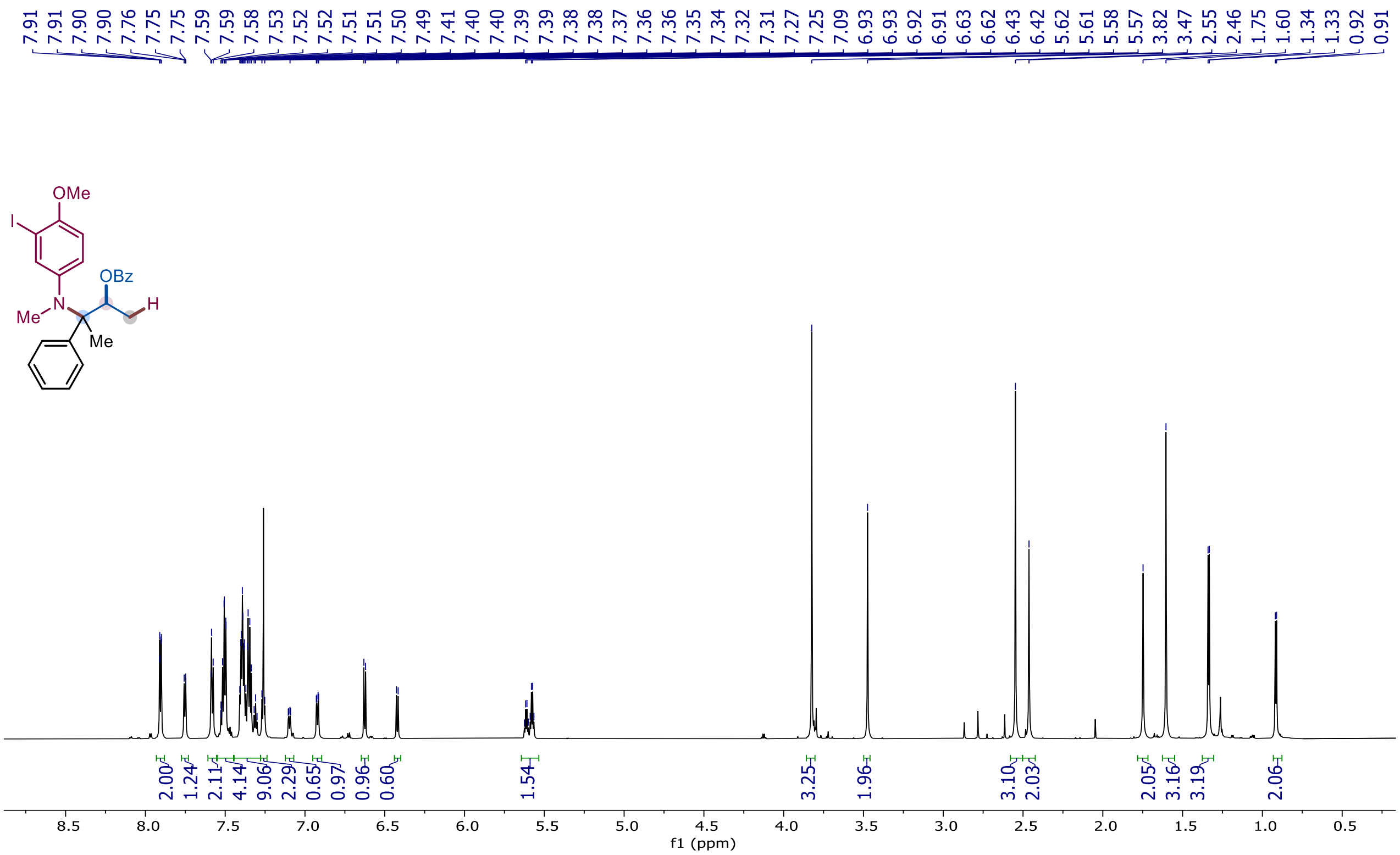

^{13}C NMR (201 MHz, CDCl₃, 25 ºC) of 3y

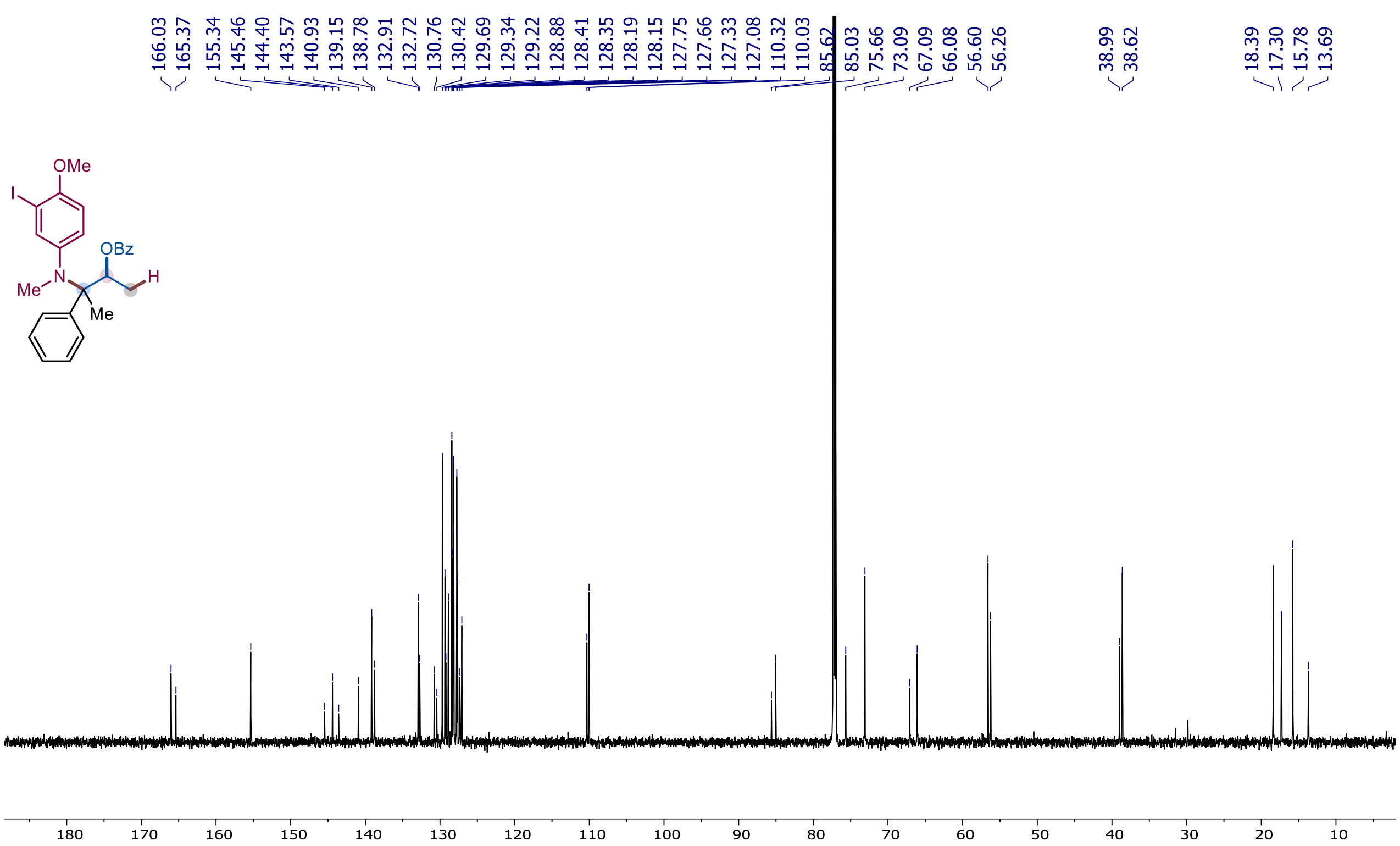

^{1}H NMR (300 MHz, $CDCl_3$, 25 ºC) of 3z

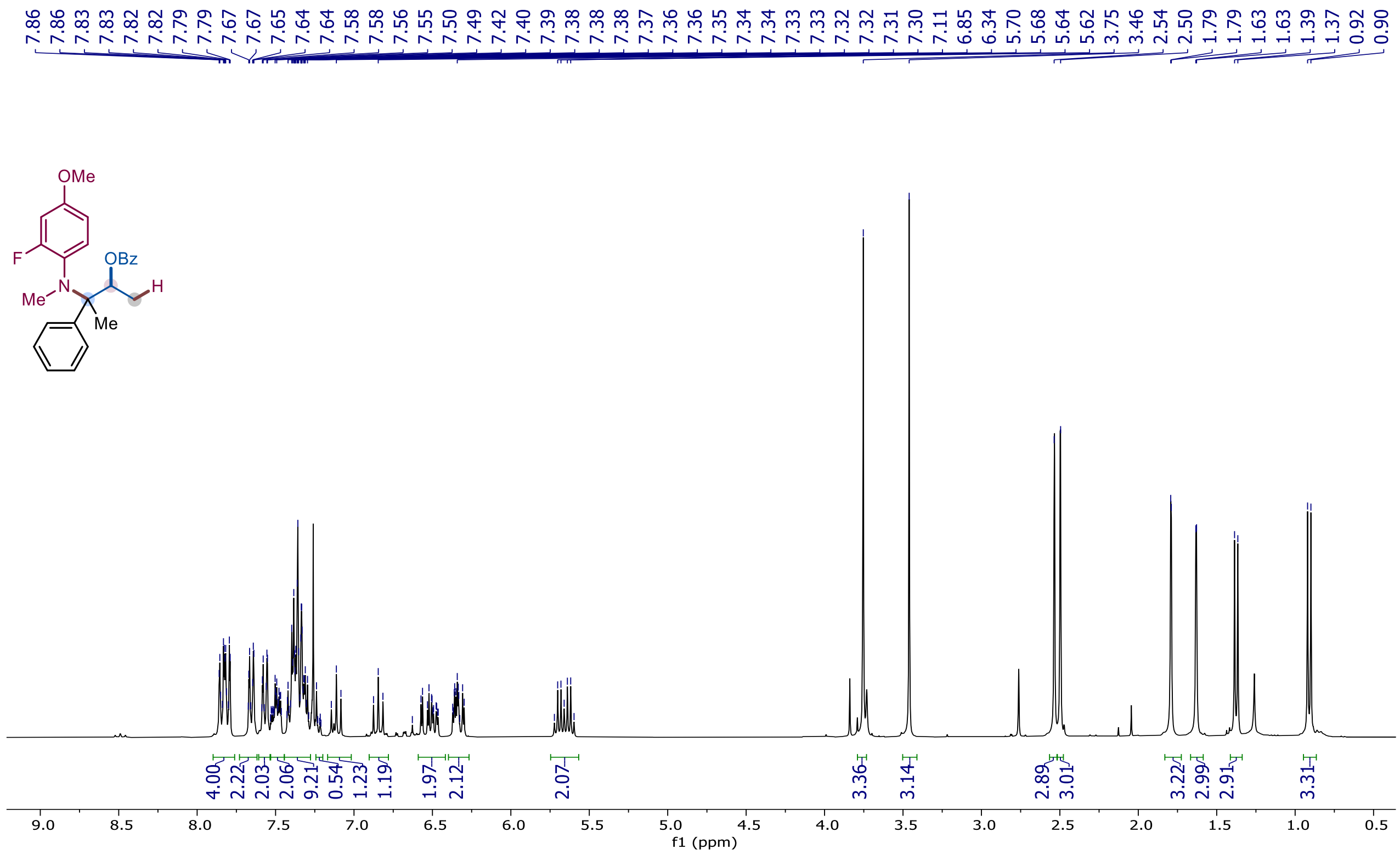

^{13}C NMR (201 MHz, $CDCl_3$, 25 ºC) of 3z

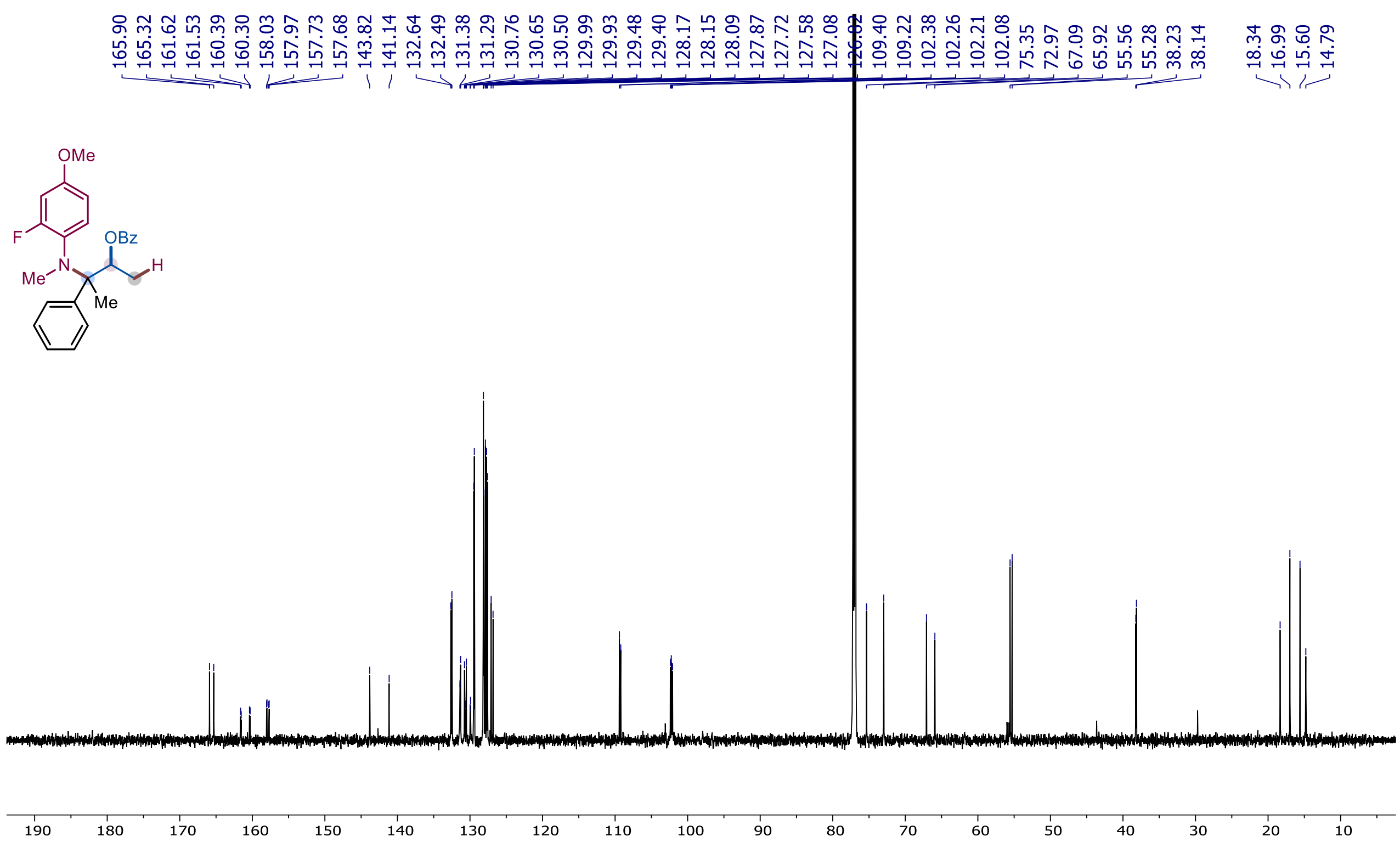

^{19}F NMR (282 MHz, $CDCl_3$, 25 °C) of 3z

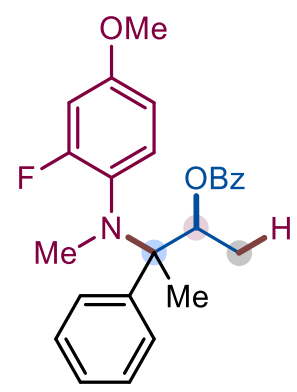

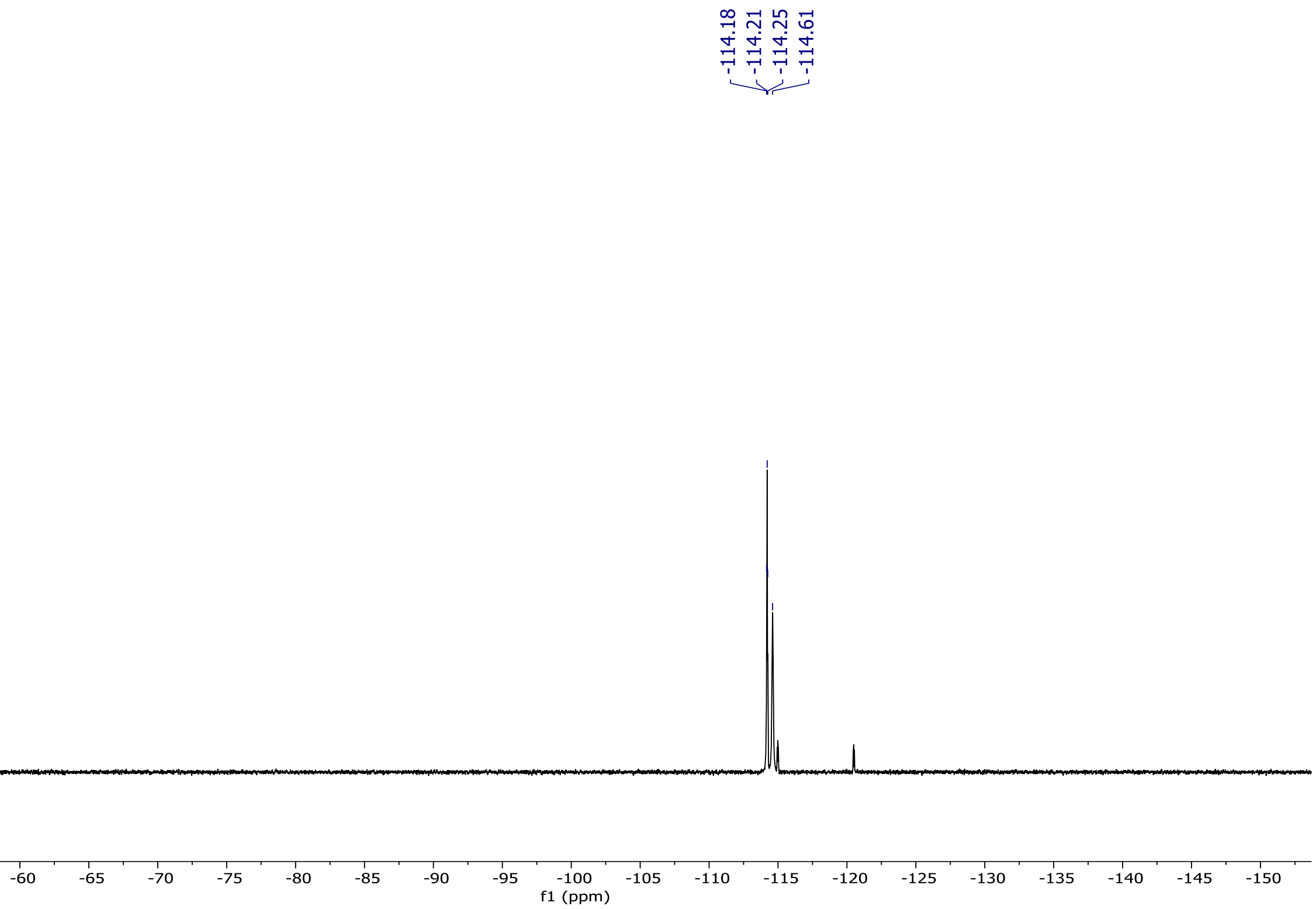

^{1}H NMR (500 MHz, $CDCl_3$, 25 °C) of 3aa

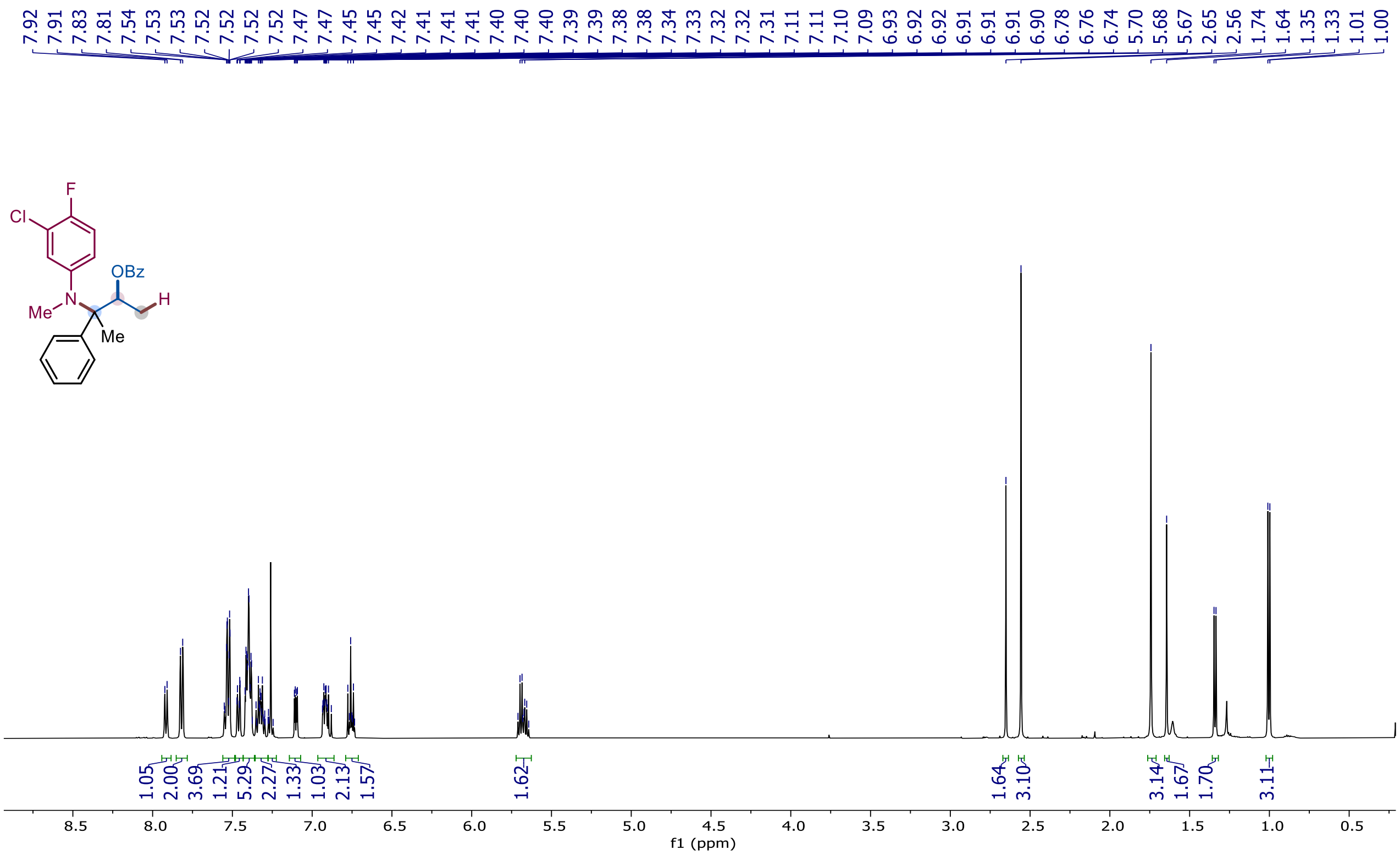

^{13}C NMR (126 MHz, $CDCl_3$, 25 °C) of 3aa

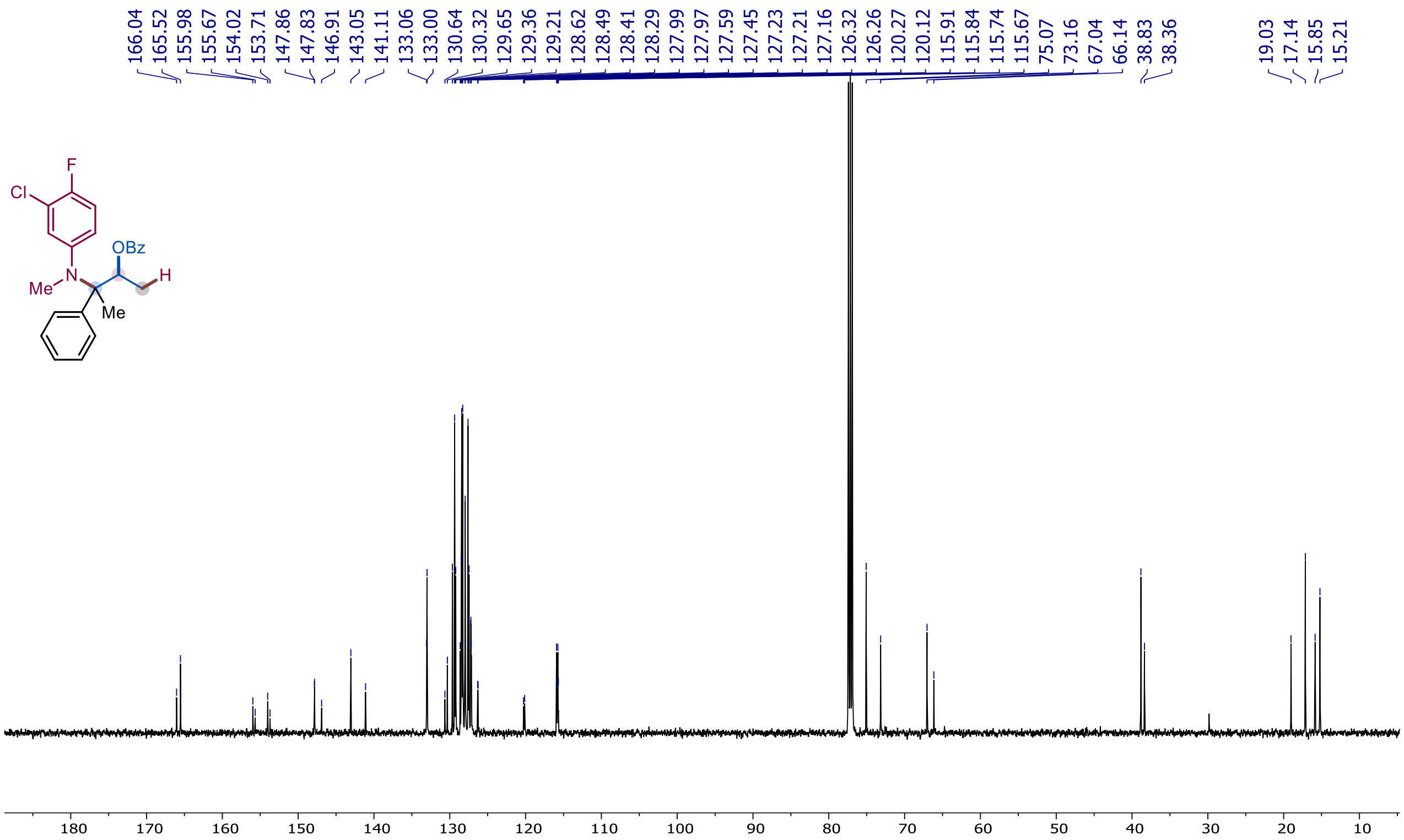

^{19}F NMR (470 MHz, $CDCl_3$, 25 ºC) of 3aa

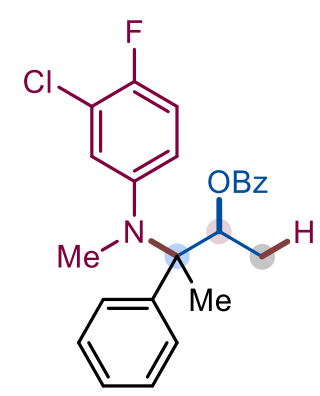

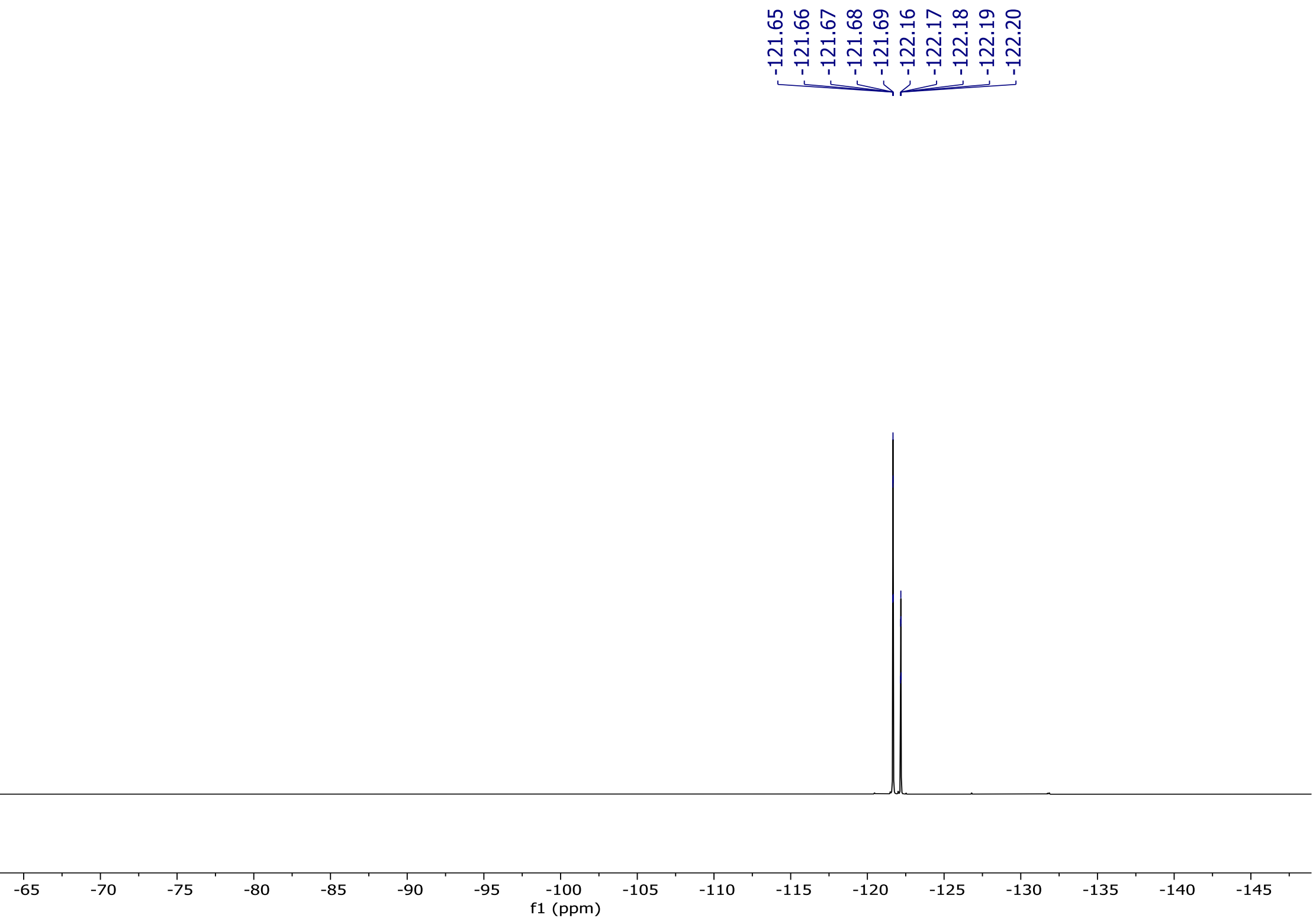

^{1}H NMR (400 MHz, $CDCl_3$, 25 ºC) of 3ab

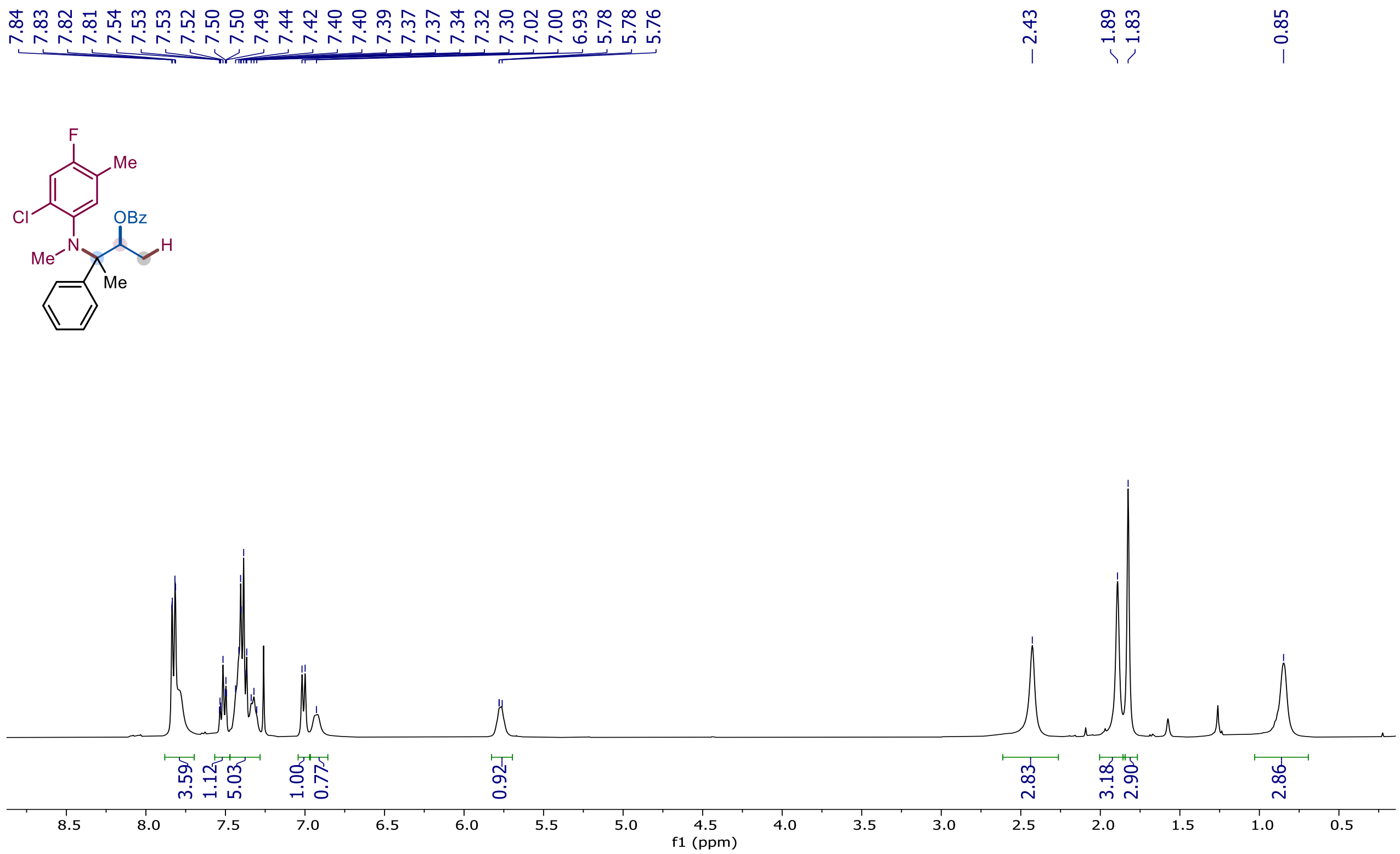

^{13}C NMR (201 MHz, $CDCl_3$, 25 ºC) of 3ab

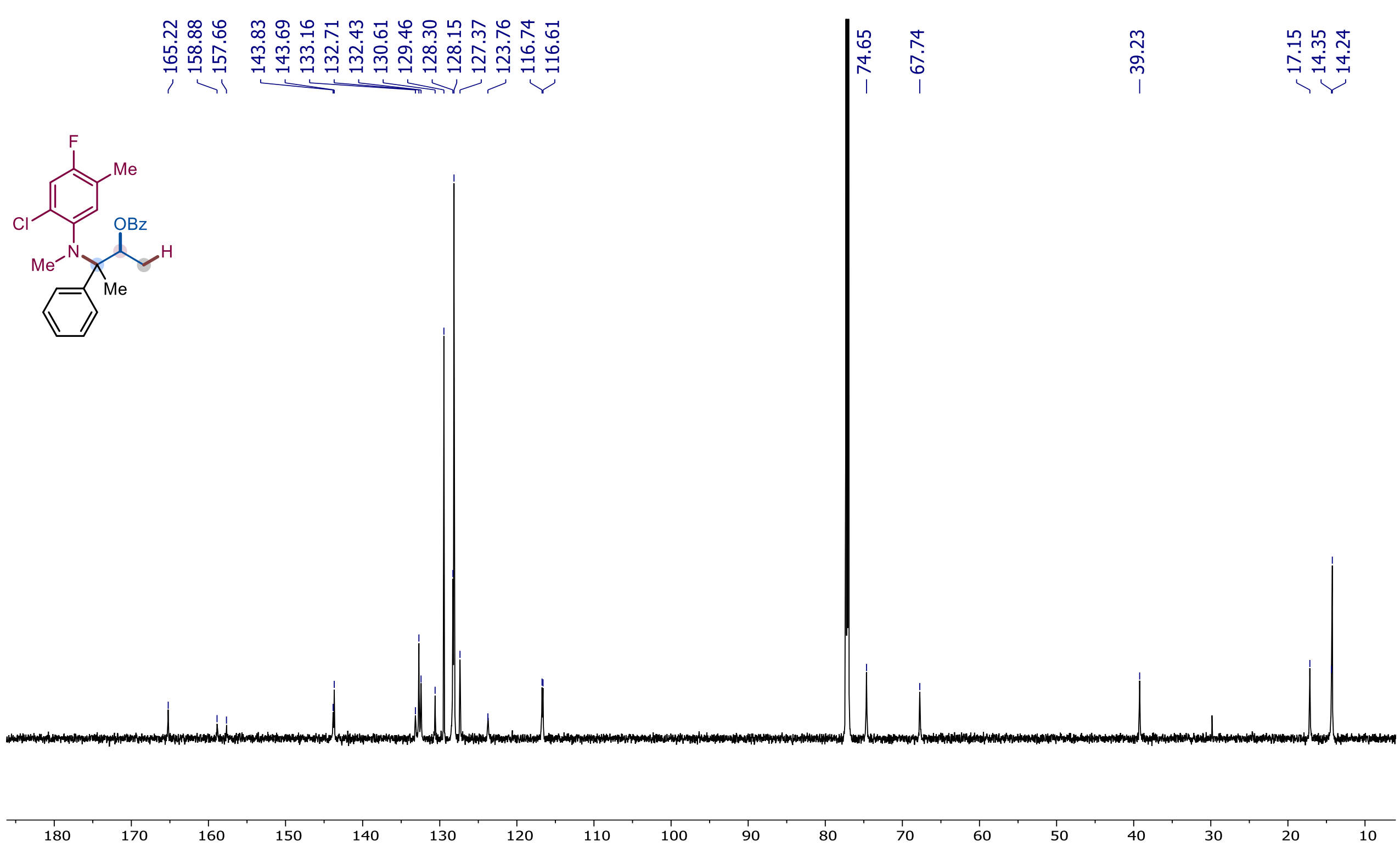

^{19}F NMR (376 MHz, $CDCl_3$, 25 °C) of 3ab

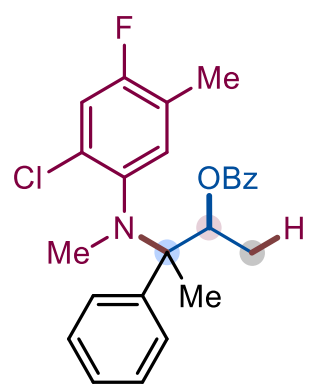

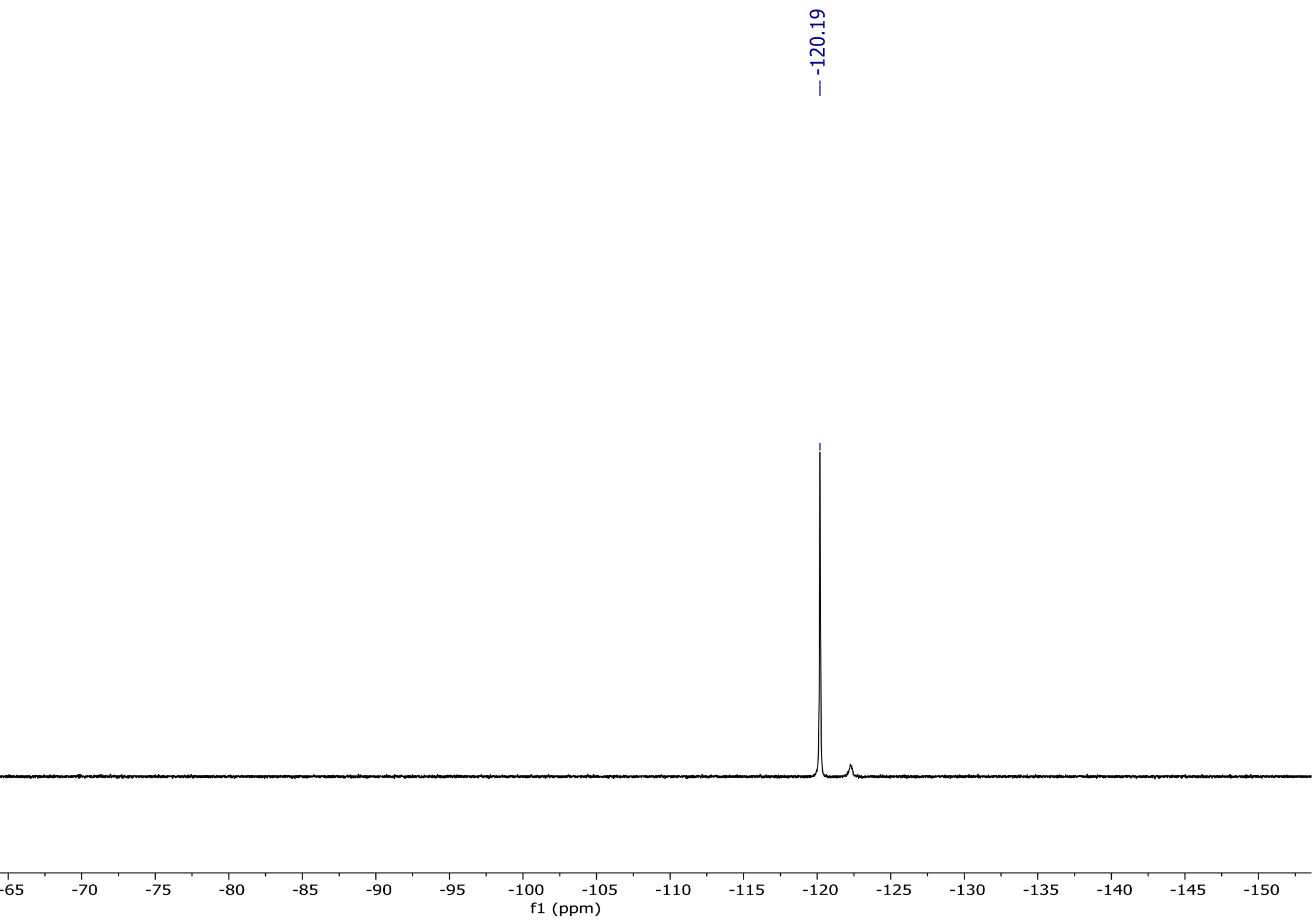

^{1}H NMR (400 MHz, $CDCl_3$, 25 °C) of 3ac

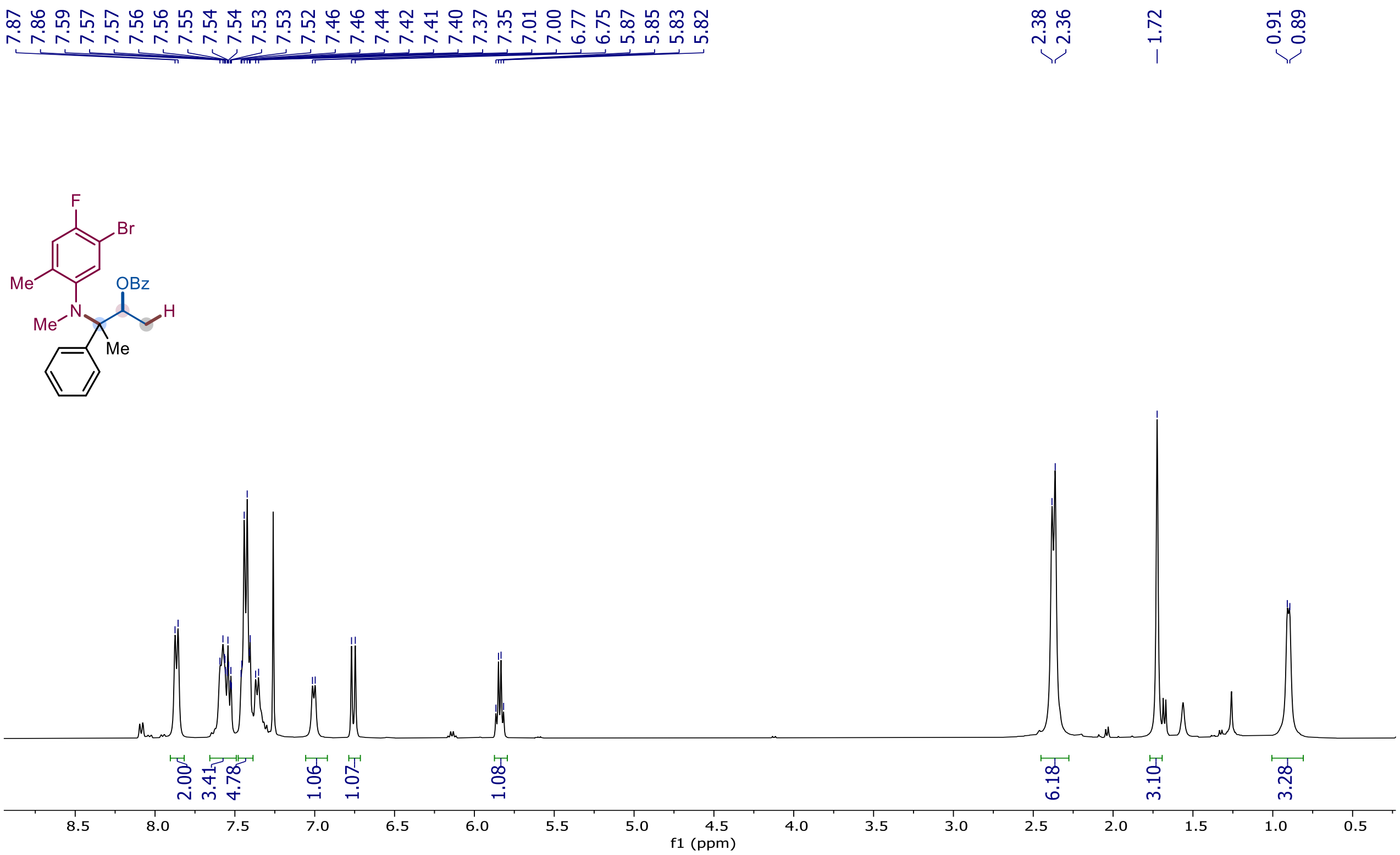

^{13}C NMR (201 MHz, $CDCl_3$, 25 °C) of 3ac

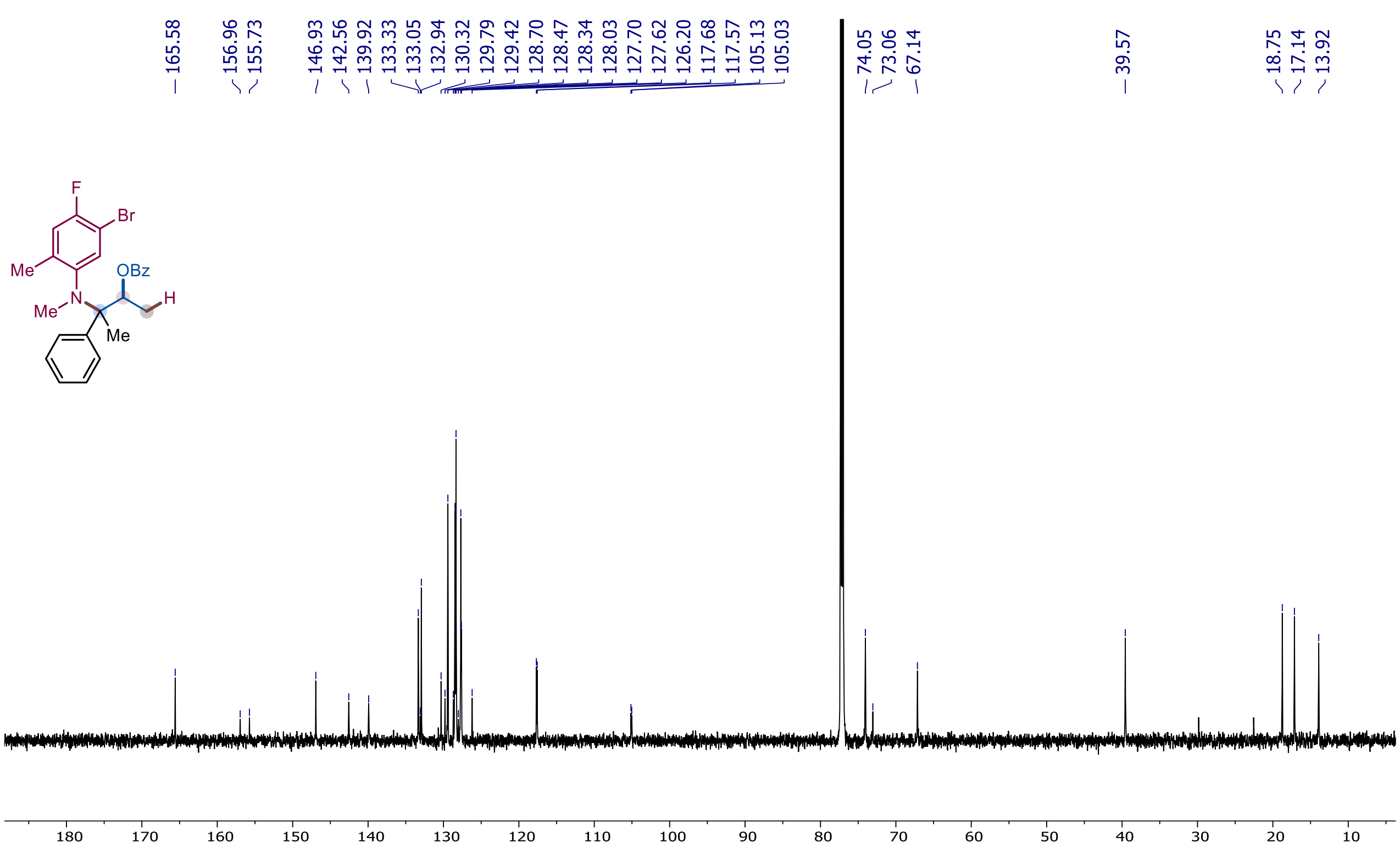

^{19}F NMR (376 MHz, $CDCl_3$, 25 °C) of 3ac

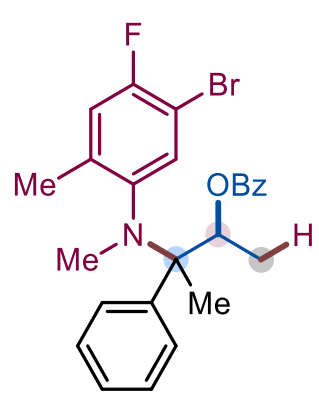

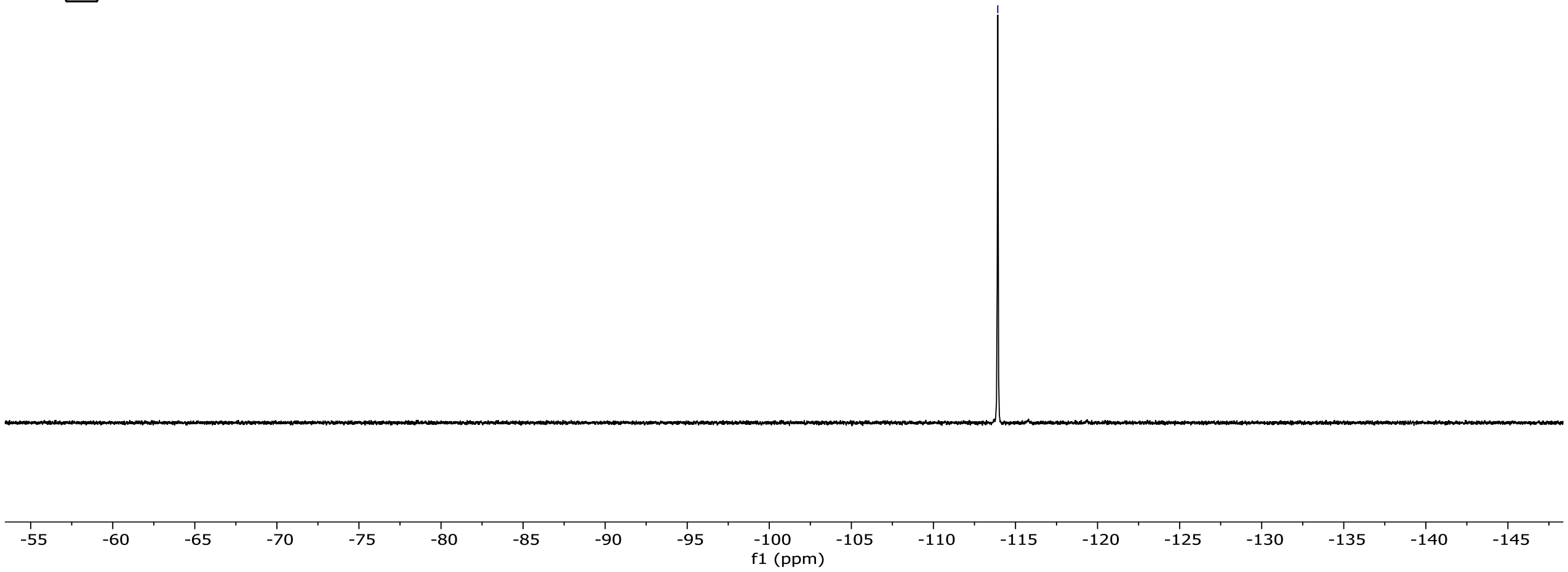

^{1}H NMR (400 MHz, CDCl₃, 25 ºC) of 3ad

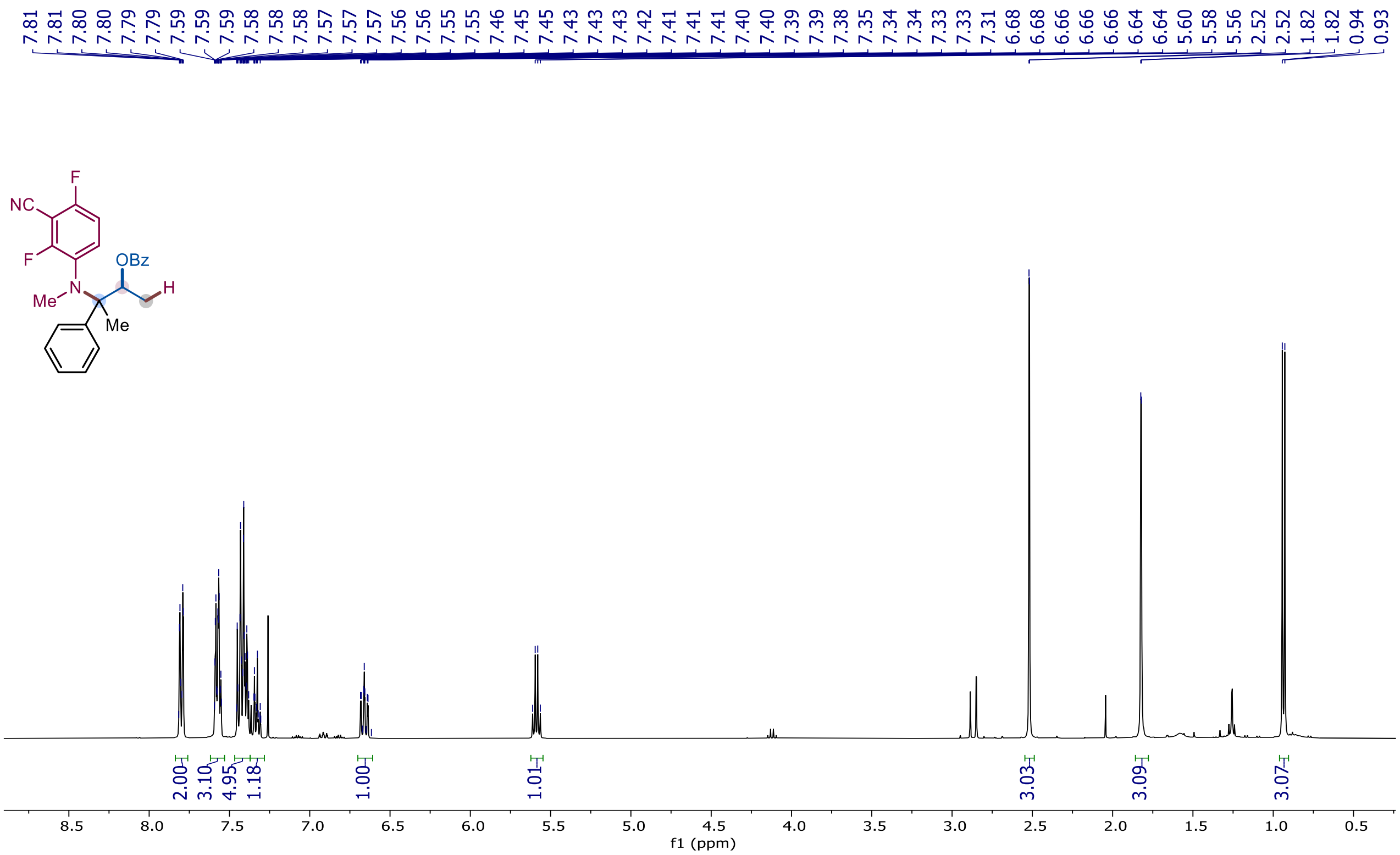

^{13}C NMR (201 MHz, CDCl₃, 25 ºC) of 3ad

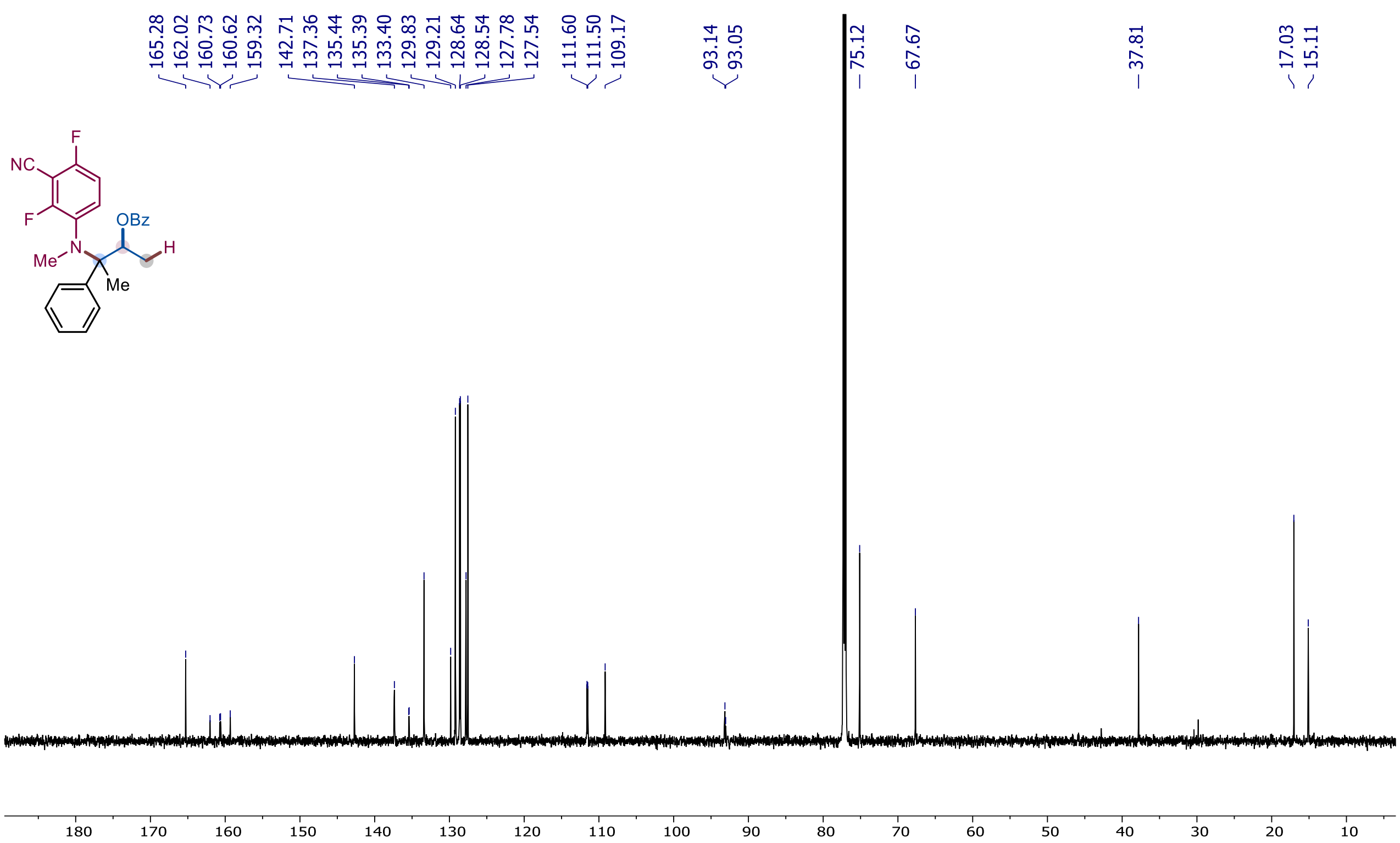

^{19}F NMR (376 MHz, $CDCl_3$, 25 °C) of 3ad

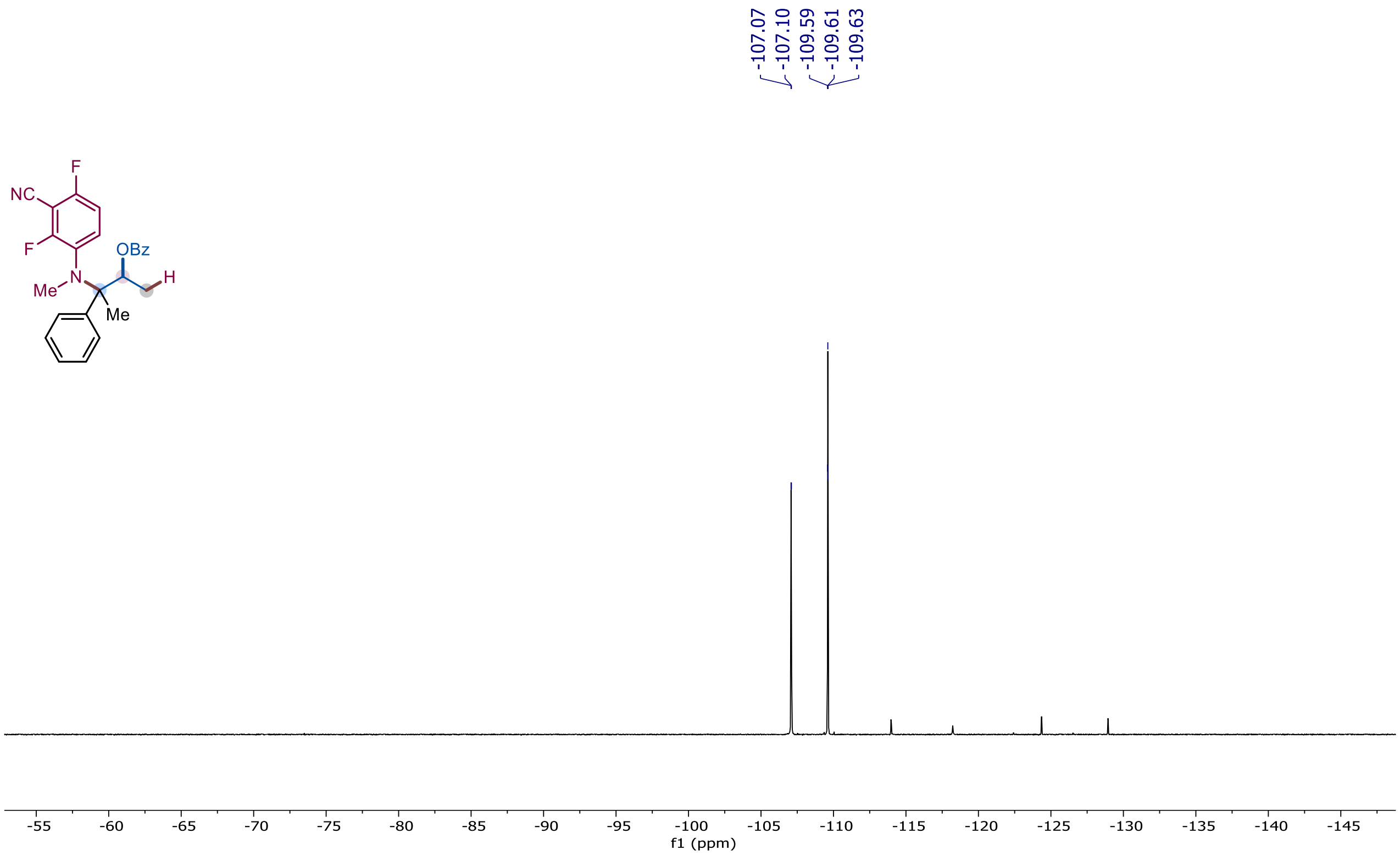

^{1}H NMR (500 MHz, CDCl$_3$, 25 °C) of 3ae

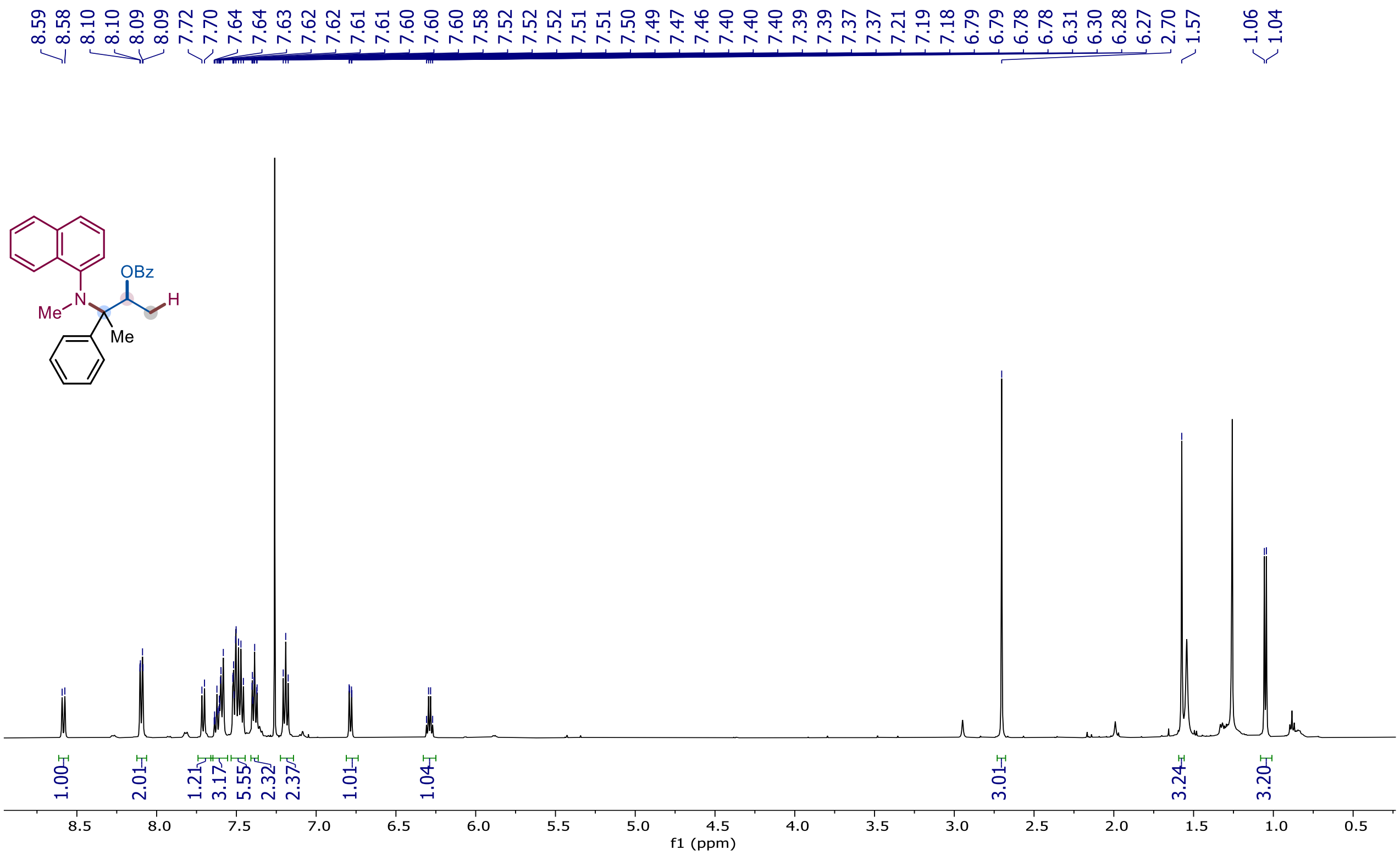

^{13}C NMR (126 MHz, CDCl$_3$, 25 °C) of 3ae

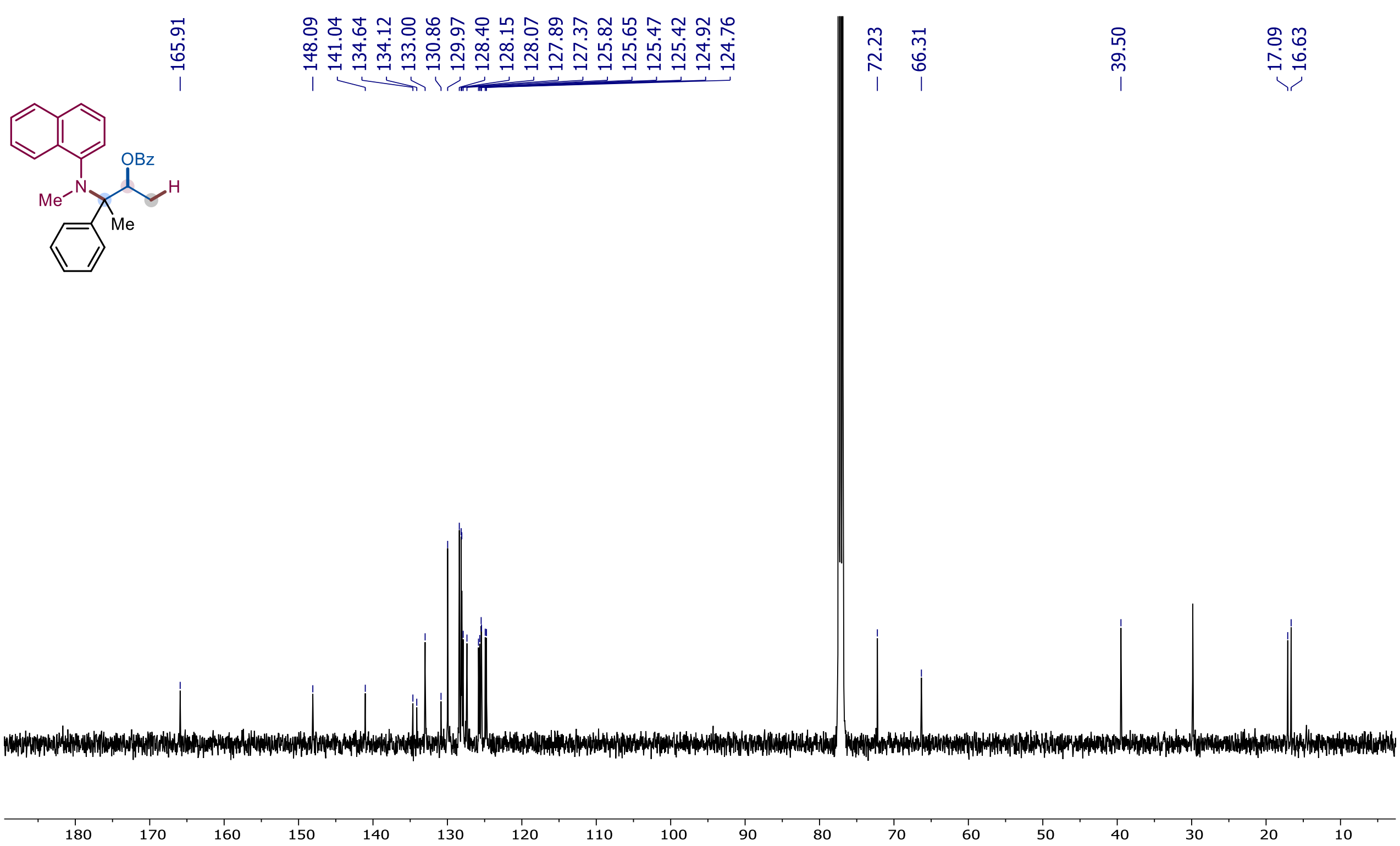

^{1}H NMR (300 MHz, $CDCl_3$, 25 ºC) of 3af (Diastereomer 1)

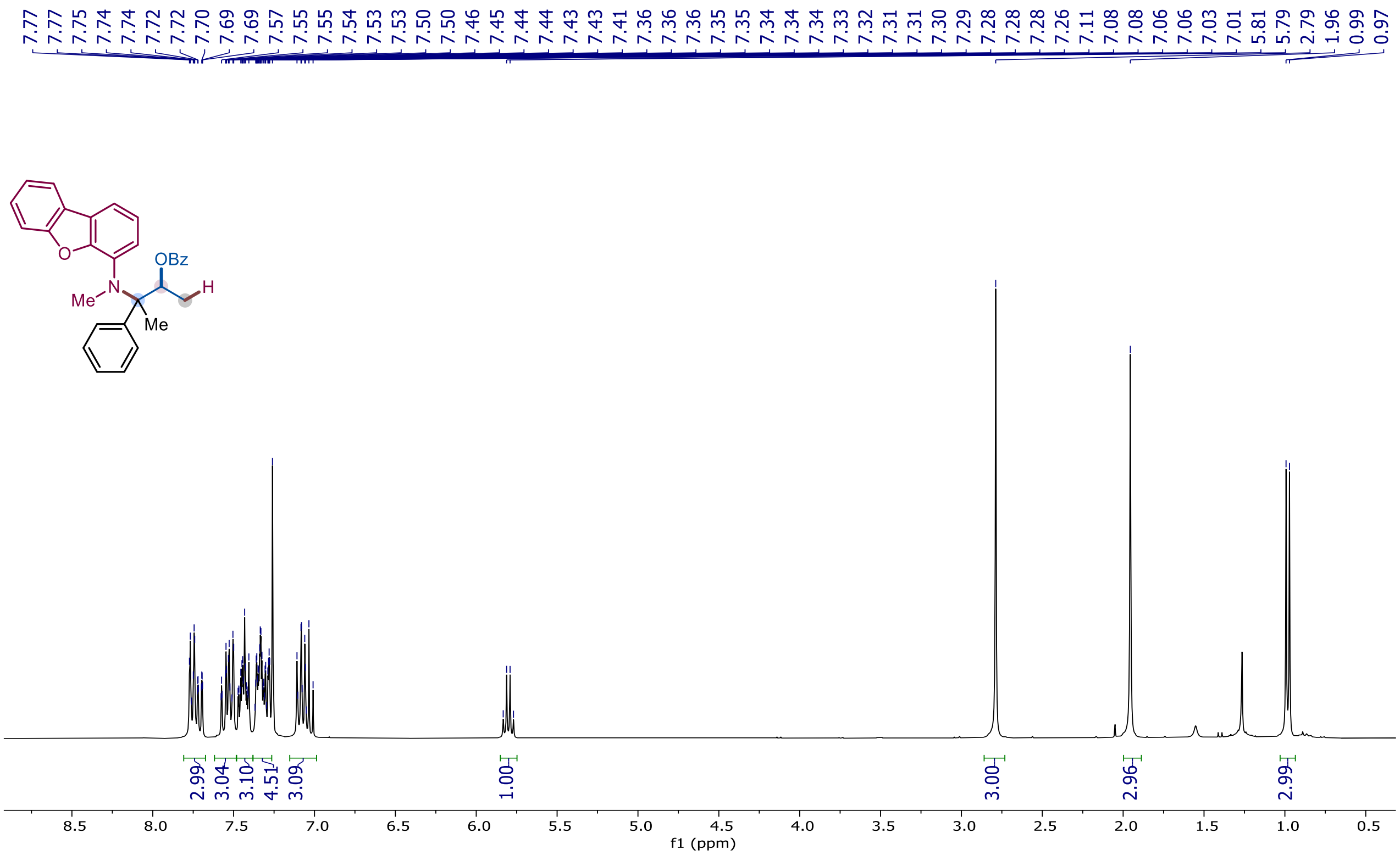

^{13}C NMR (201 MHz, $CDCl_3$, 25 ºC) of 3af (Diastereomer 1)

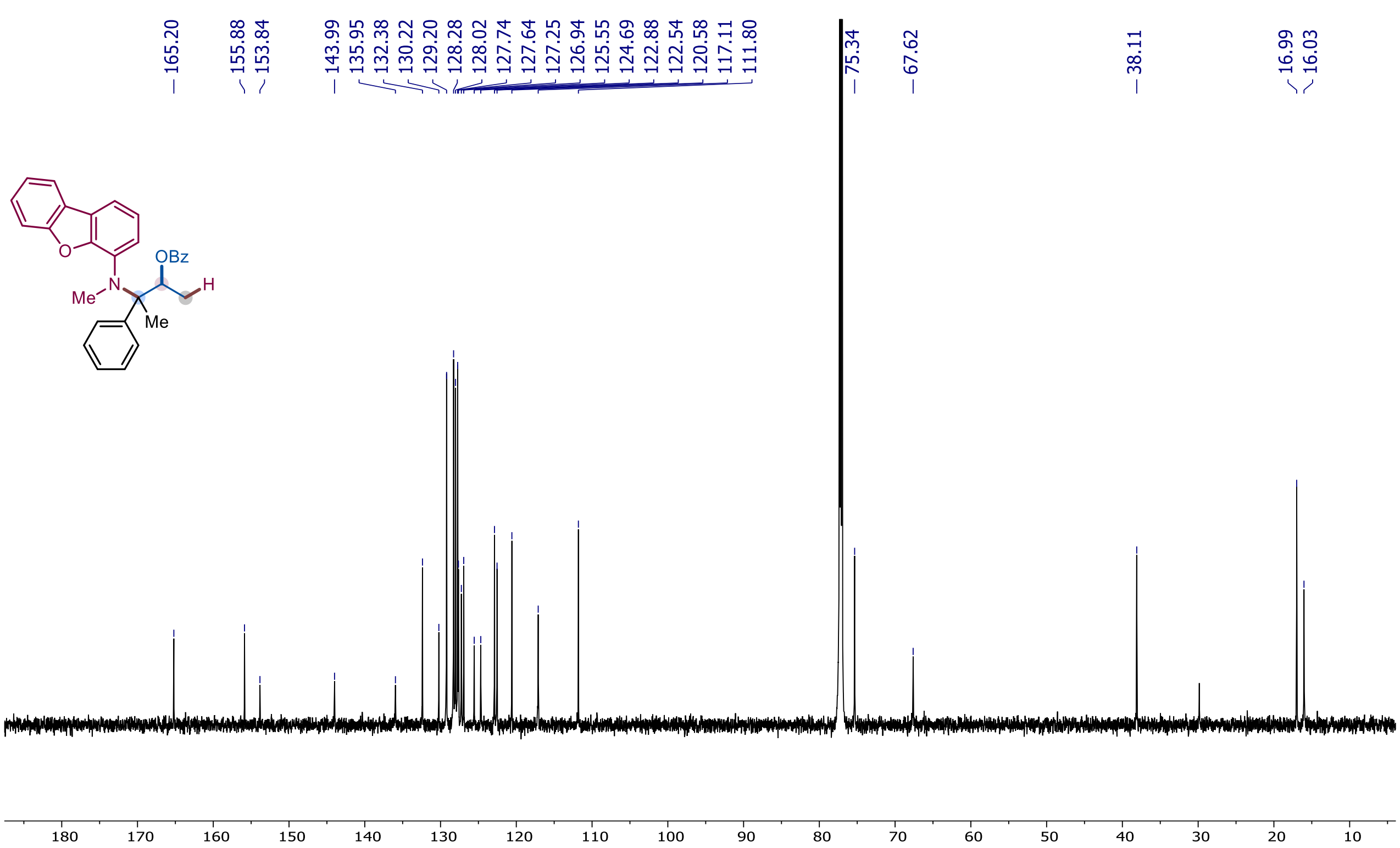

^{1}H NMR (300 MHz, CDCl$_3$, 25 ºC) of 3af (Diastereomer 2)

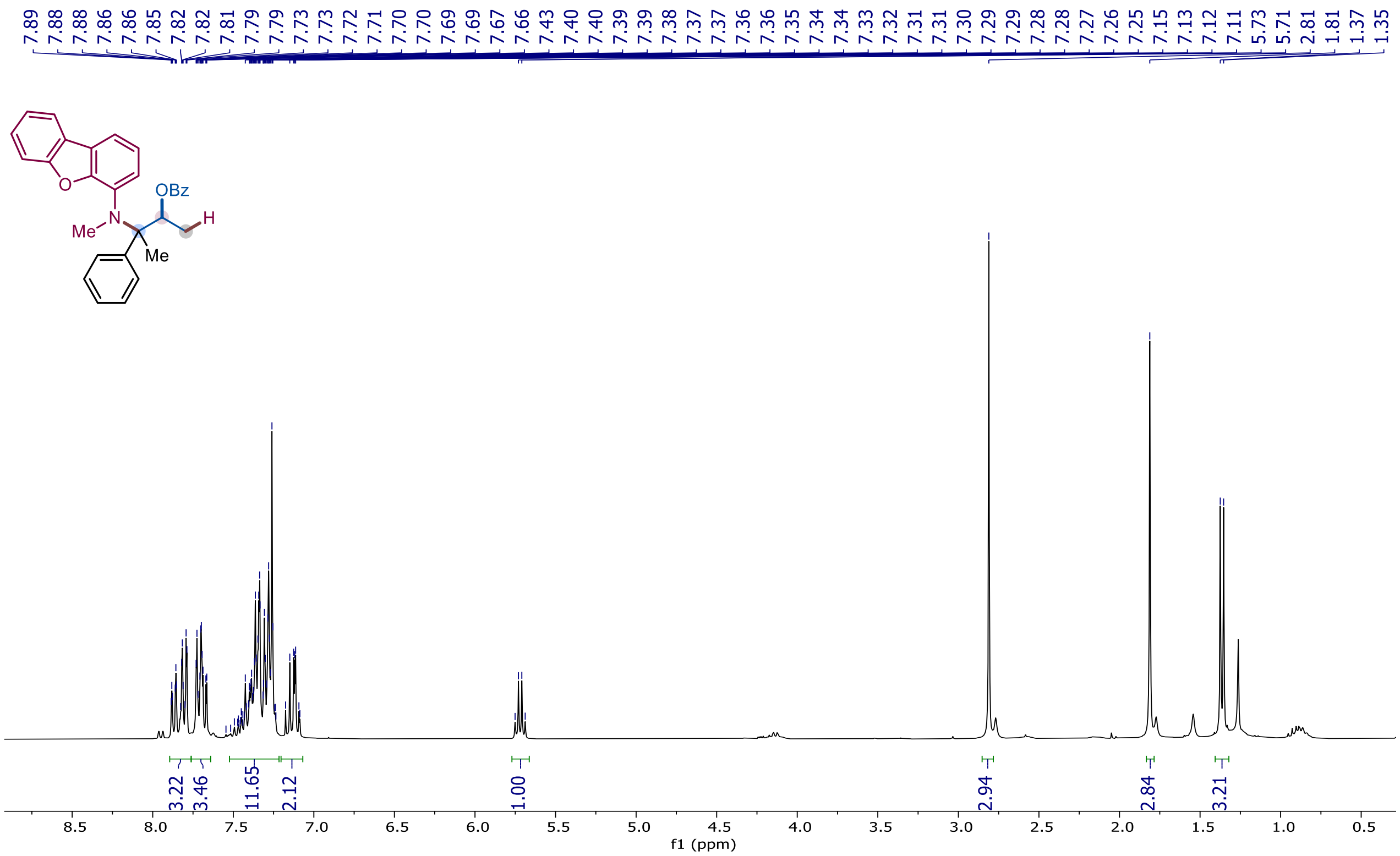

^{13}C NMR (201 MHz, CDCl$_3$, 25 ºC) of 3af (Diastereomer 2)

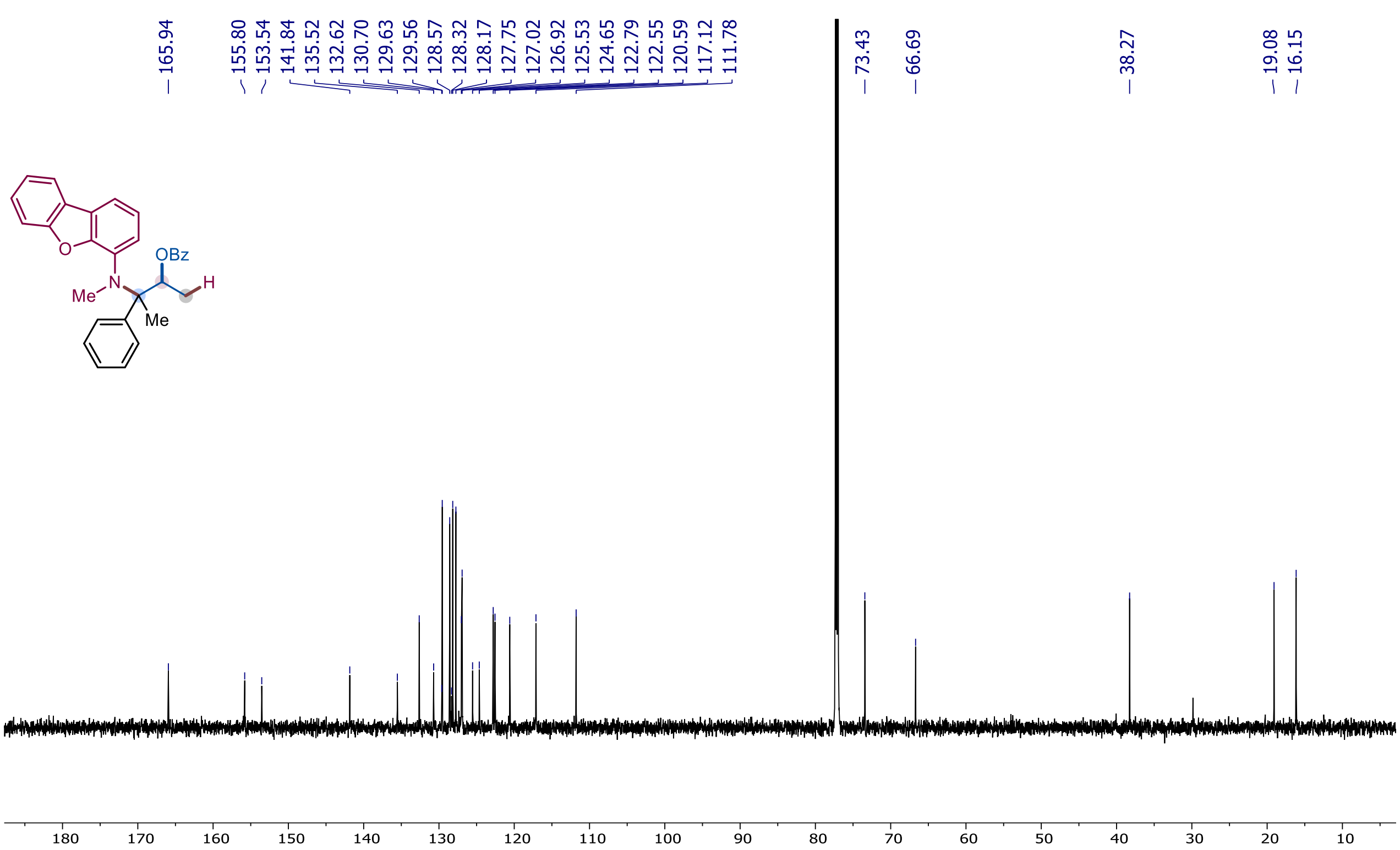

^{1}H NMR (300 MHz, $CDCl_3$, 25 ºC) of 3ag

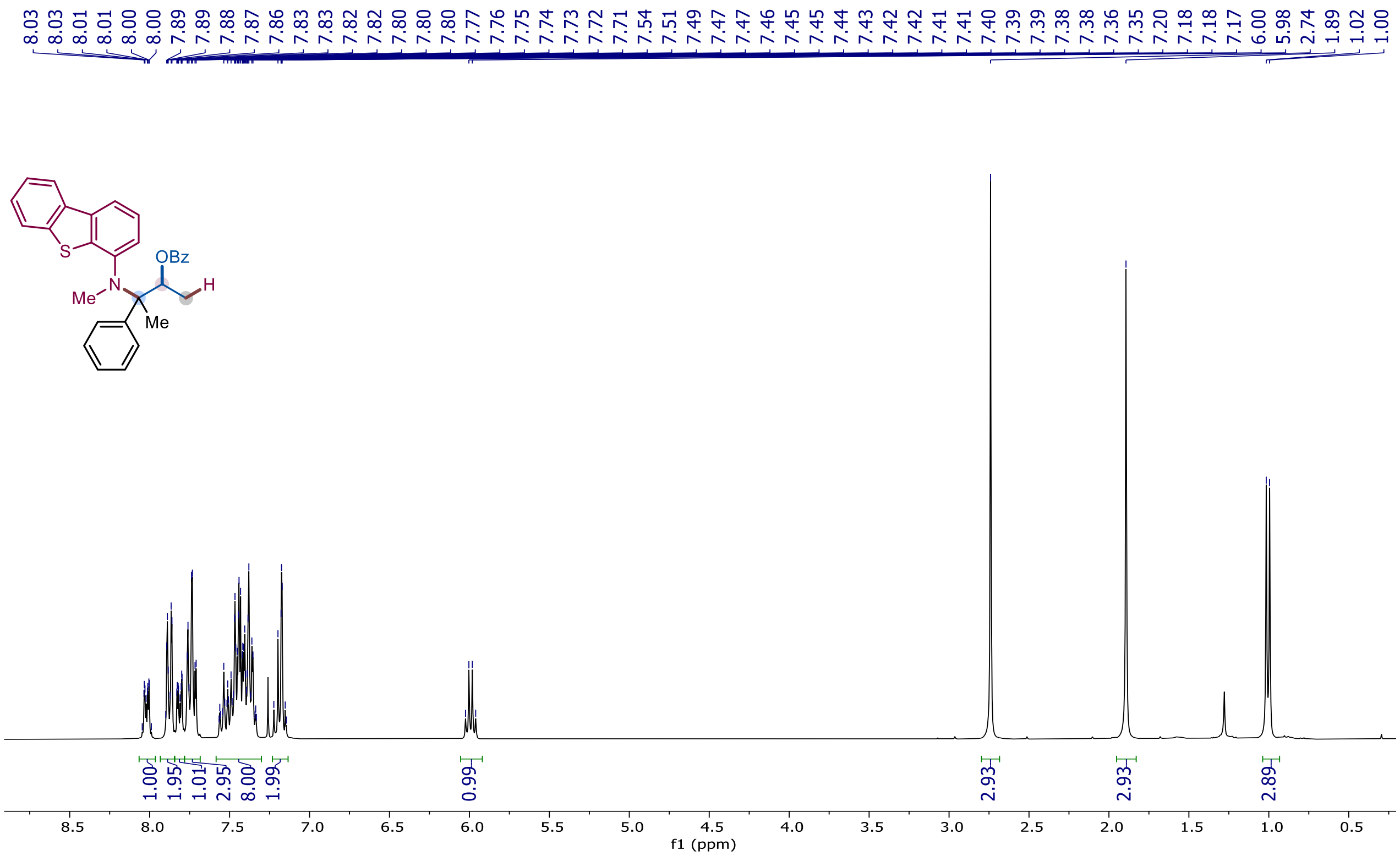

^{13}C NMR (201 MHz, $CDCl_3$, 25 ºC) of 3ag

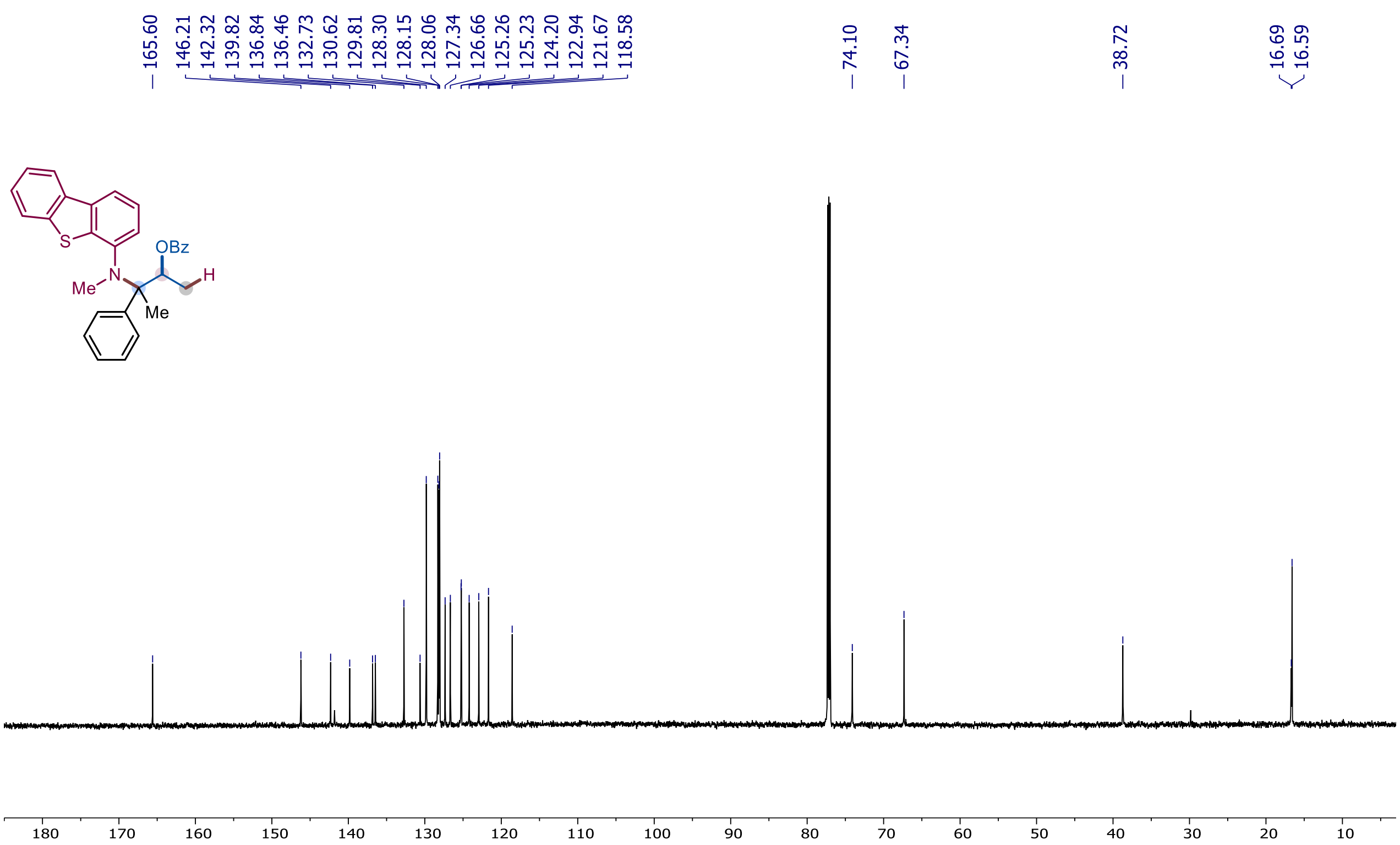

^{1}H NMR (400 MHz, $CDCl_3$, 25 ºC) of 3ah

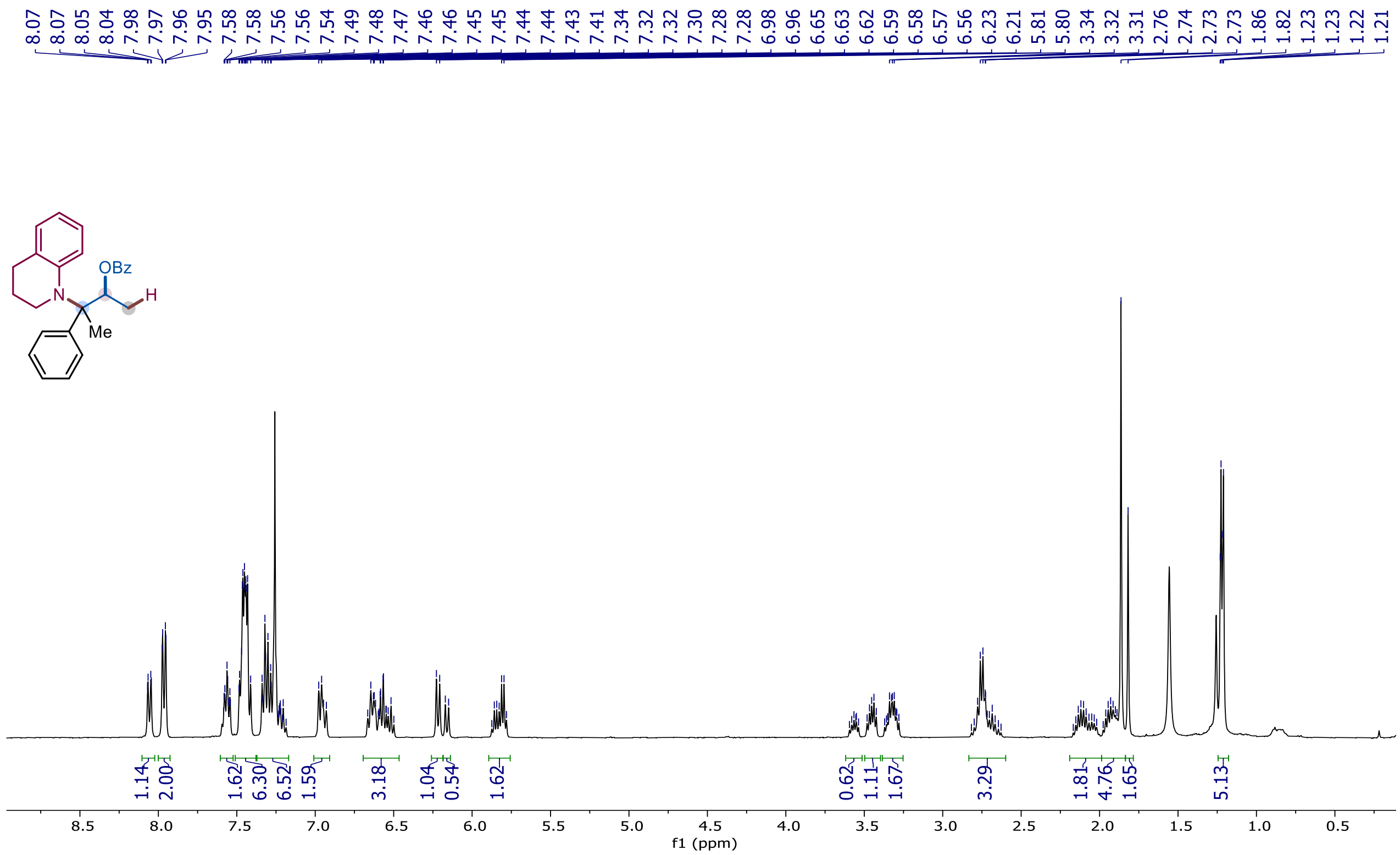

^{13}C NMR (201 MHz, $CDCl_3$, 25 ºC) of 3ah

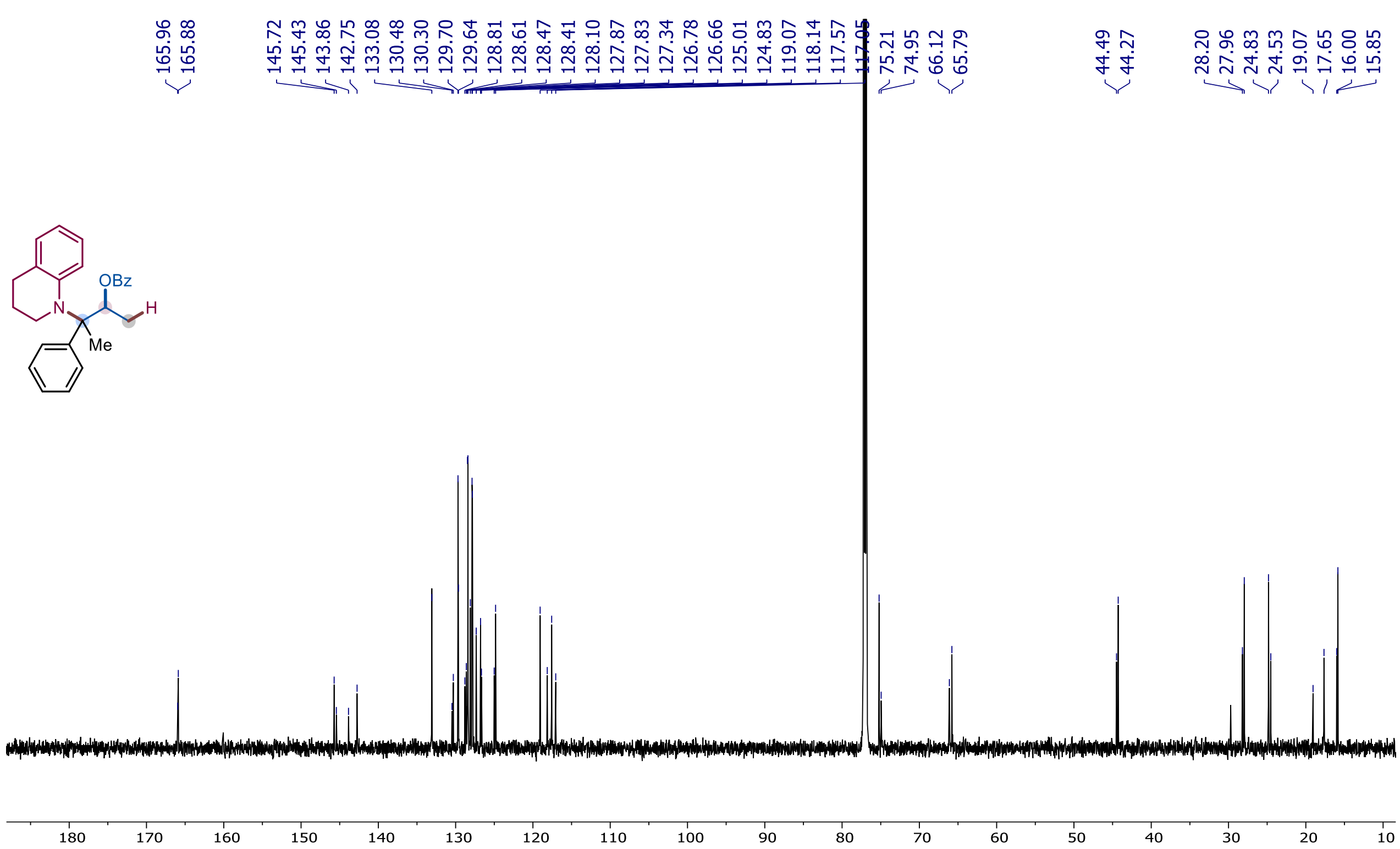

^{1}H NMR (500 MHz, $CDCl_3$, 25 ºC) of 3ai

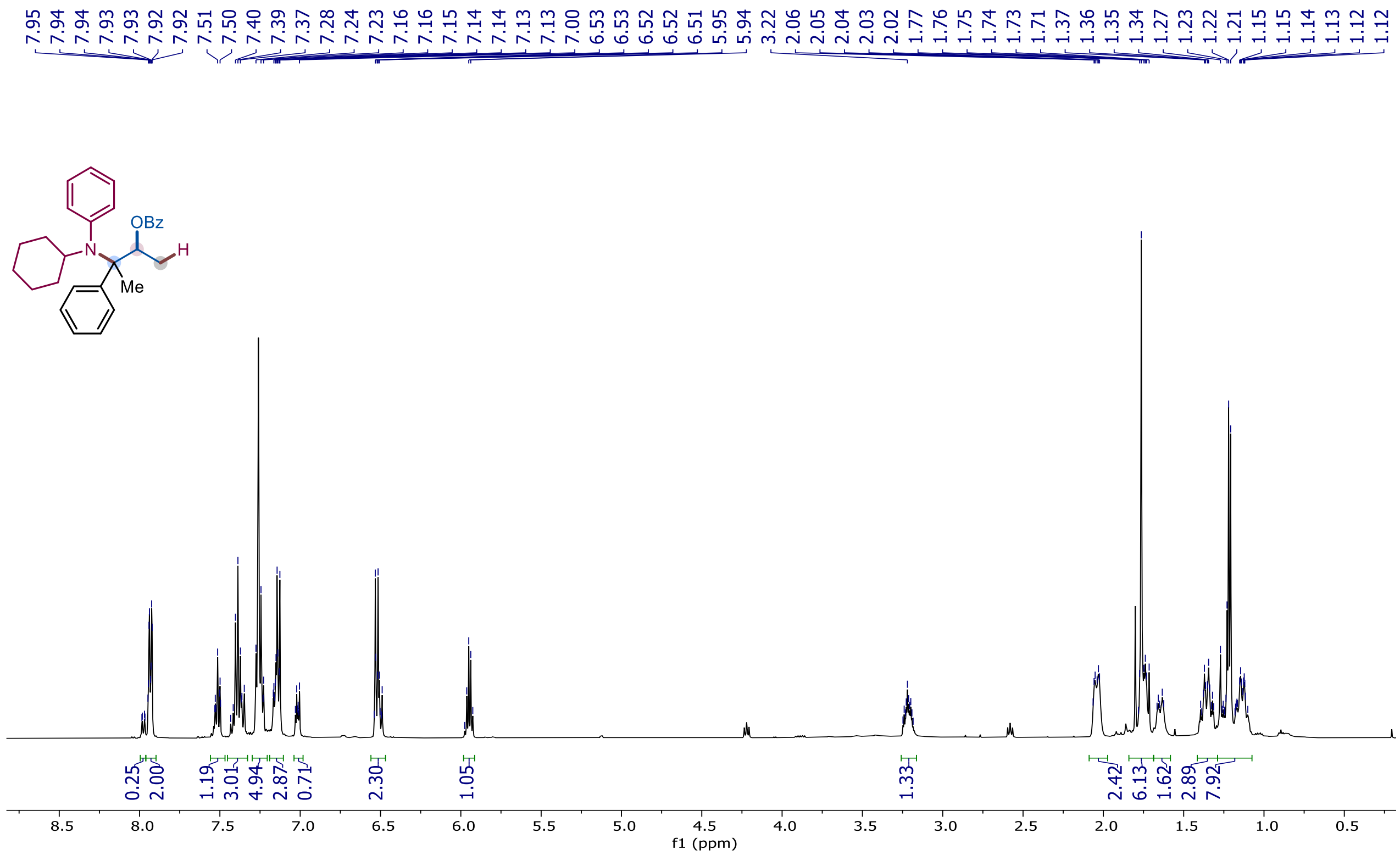

^{13}C NMR (126 MHz, $CDCl_3$, 25 ºC) of 3ai

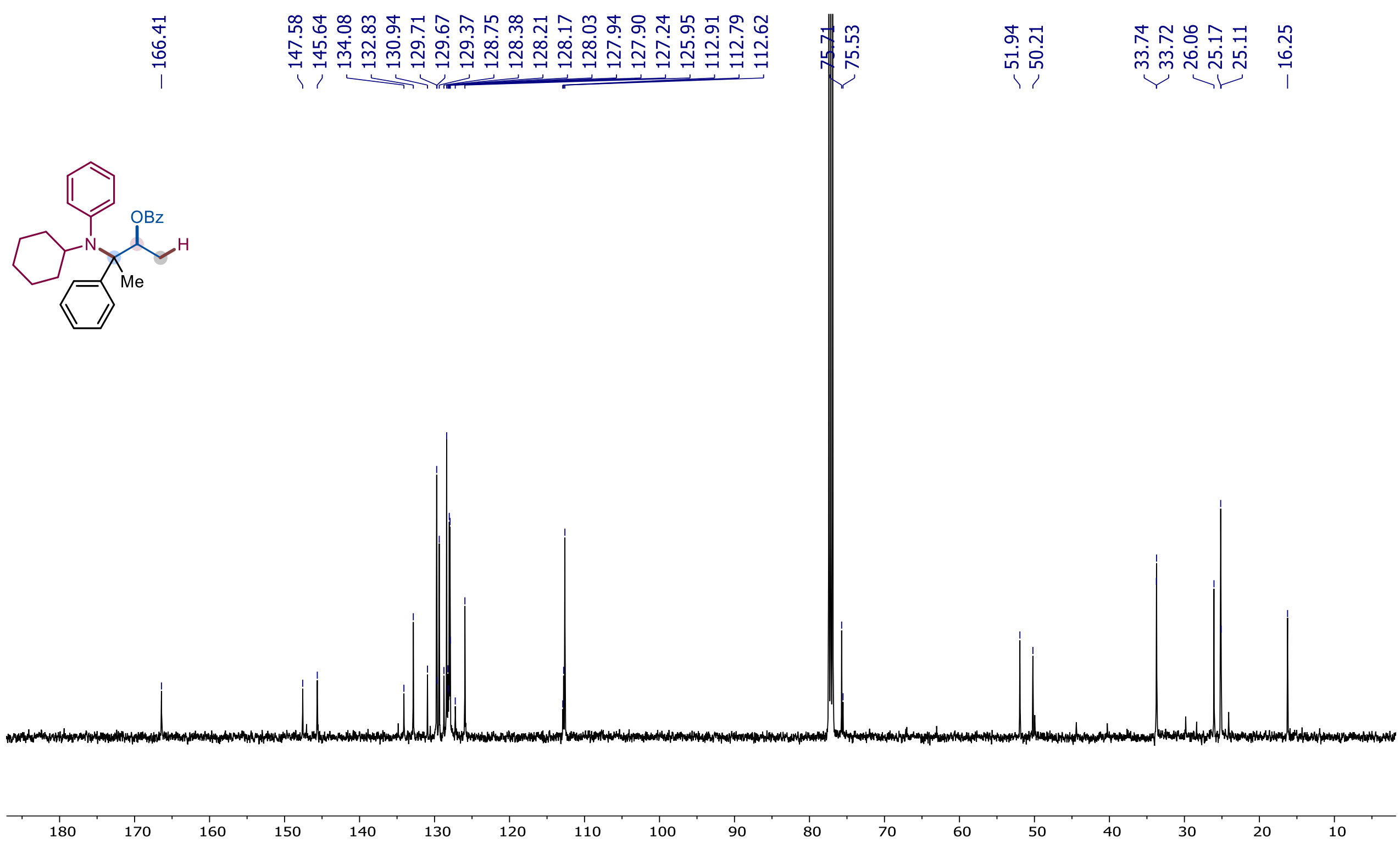

^{1}H NMR (500 MHz, $CDCl_3$, 25 °C) of 3aj

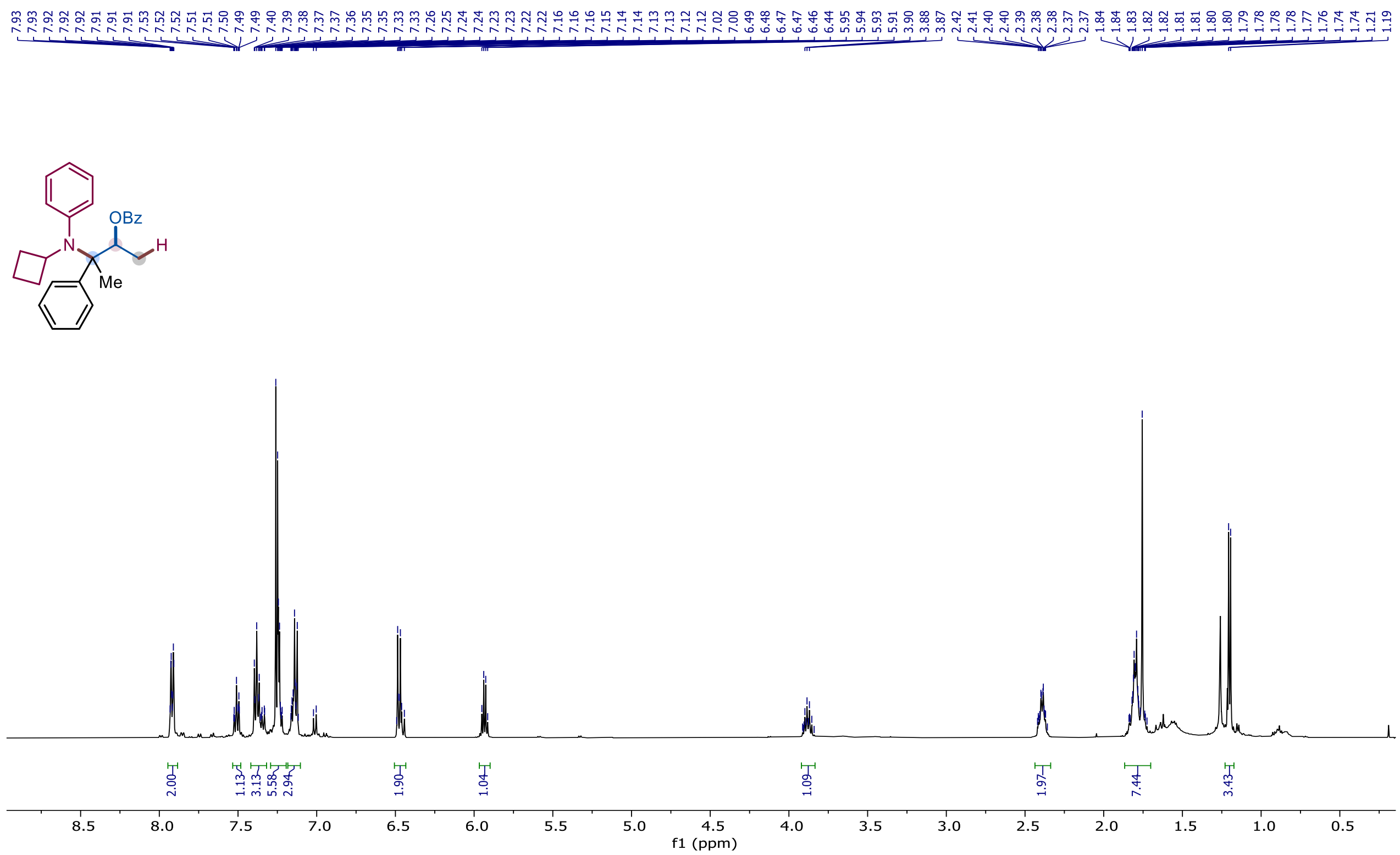

^{13}C NMR (126 MHz, $CDCl_3$, 25 °C) of 3aj

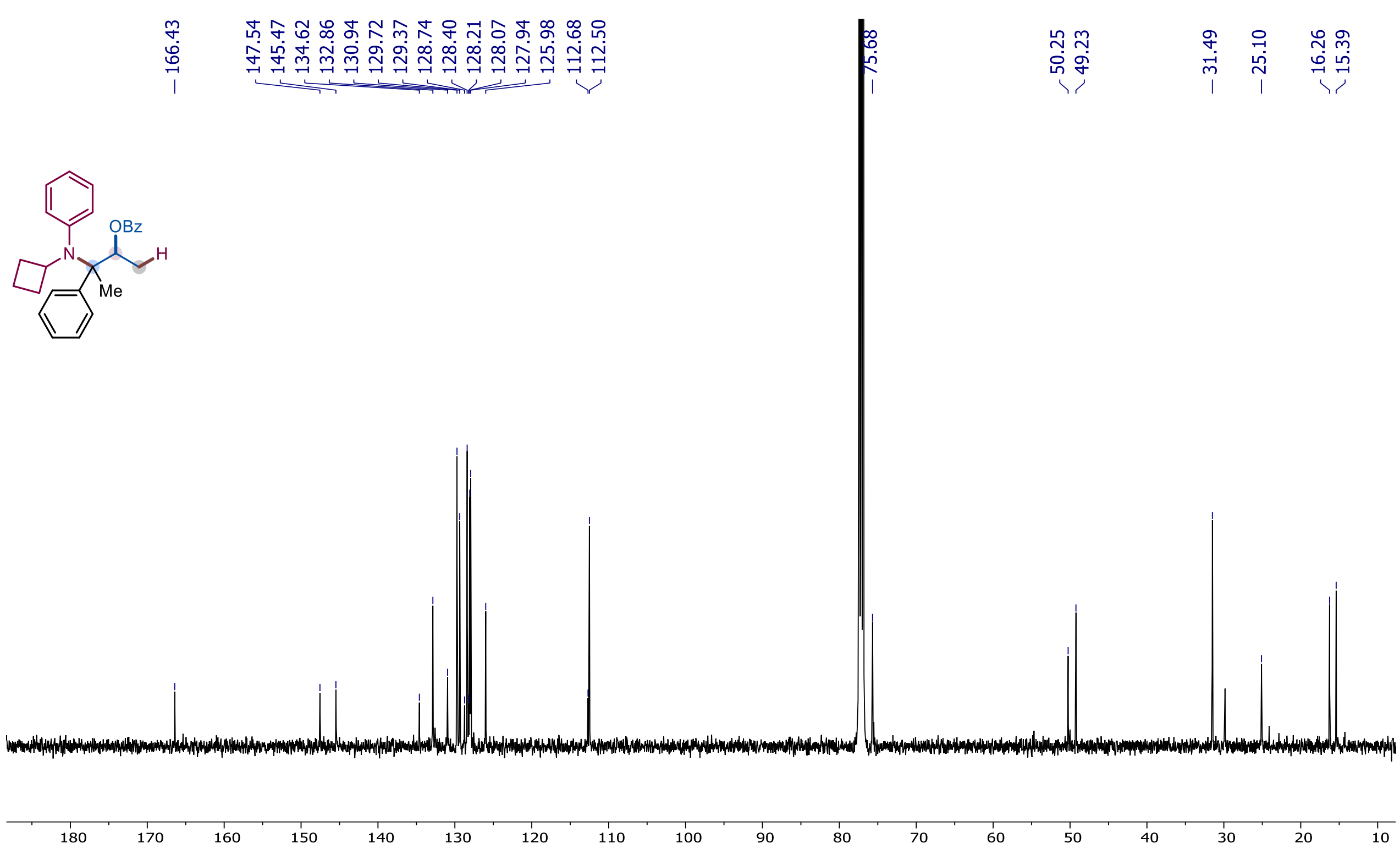

^{1}H NMR (300 MHz, $CDCl_3$, 25 ºC) of 3ak

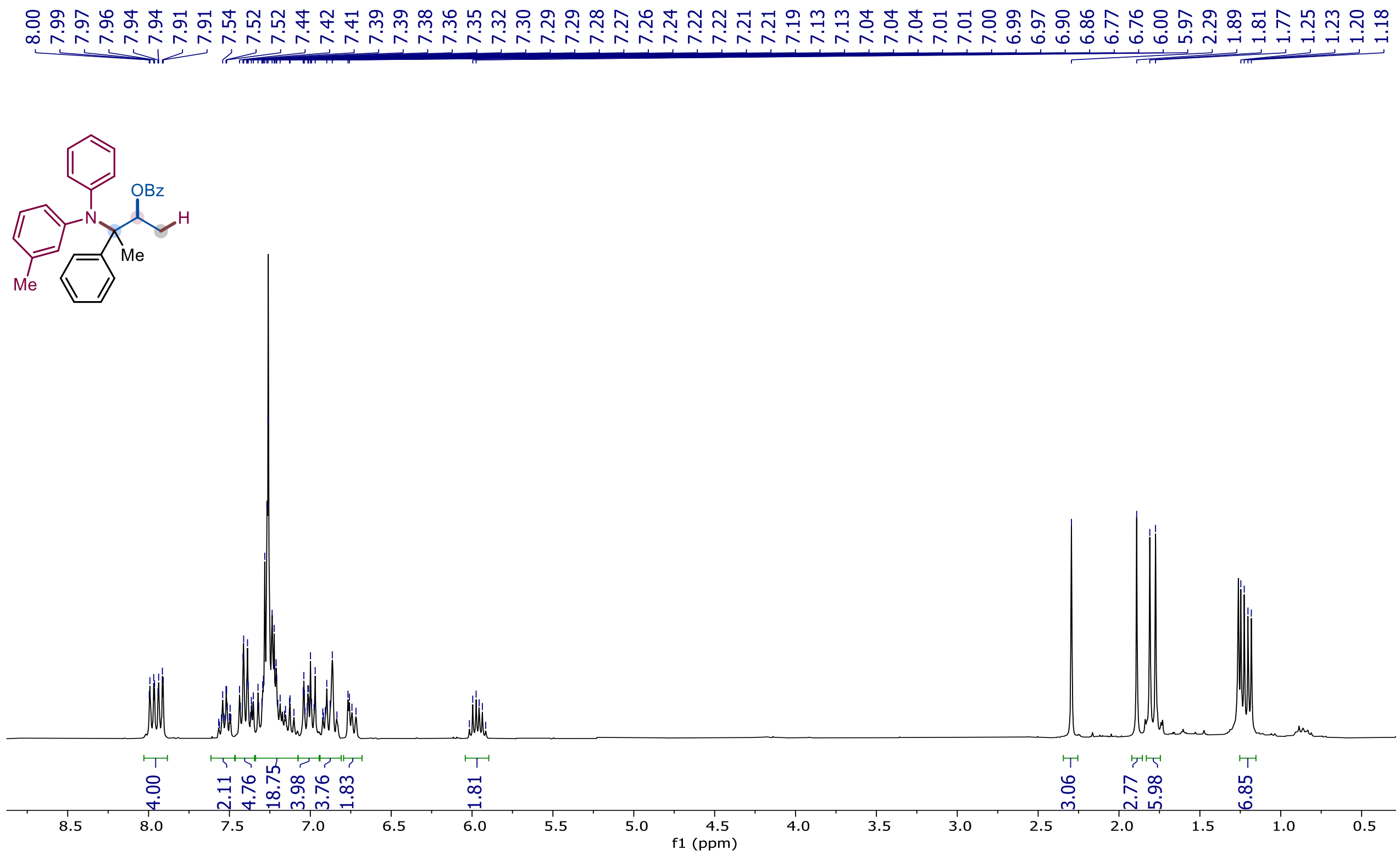

^{13}C NMR (201 MHz, $CDCl_3$, 25 ºC) of 3ak

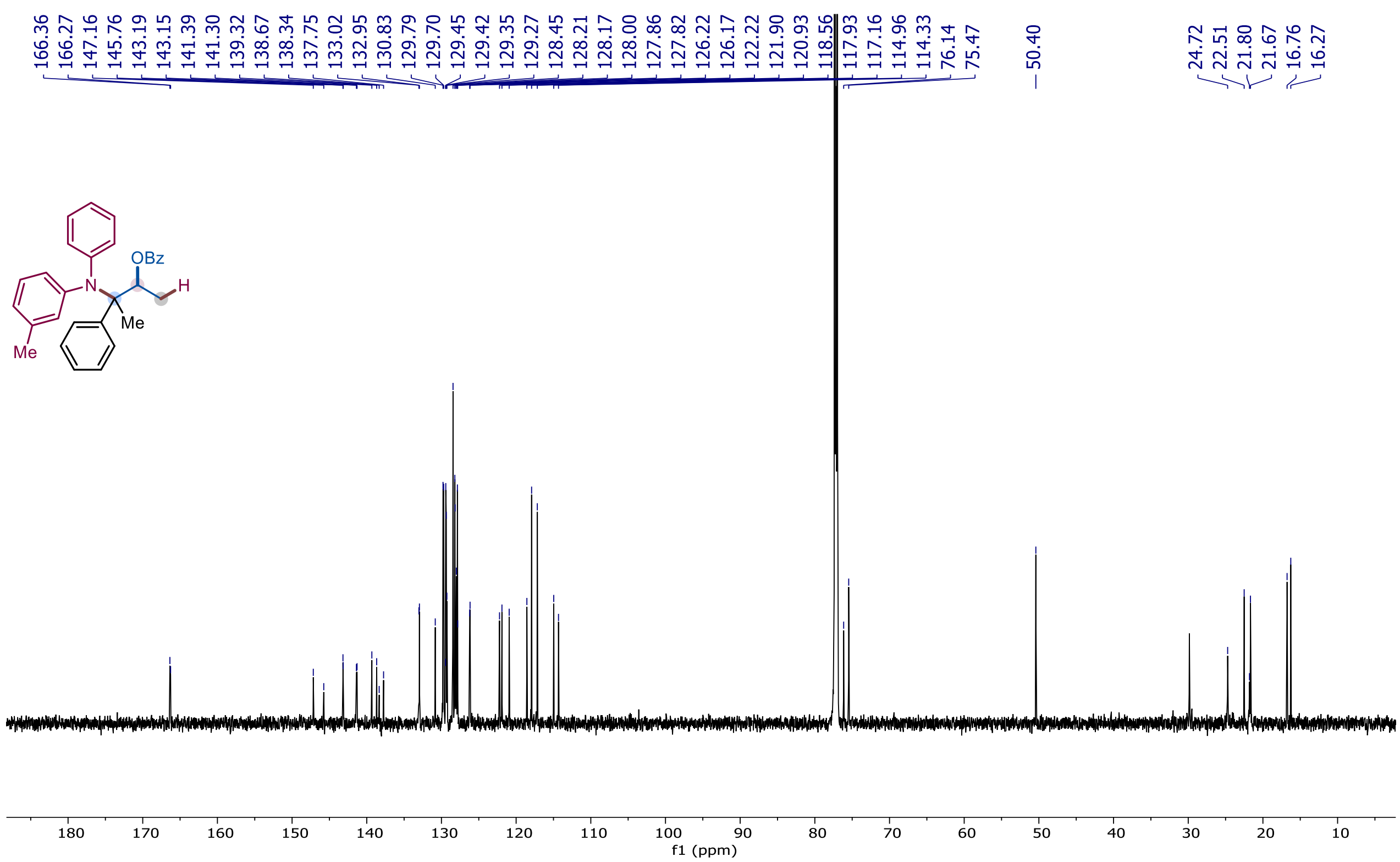

^{1}H NMR (500 MHz, CD_3CN, 25 °C) of 3al

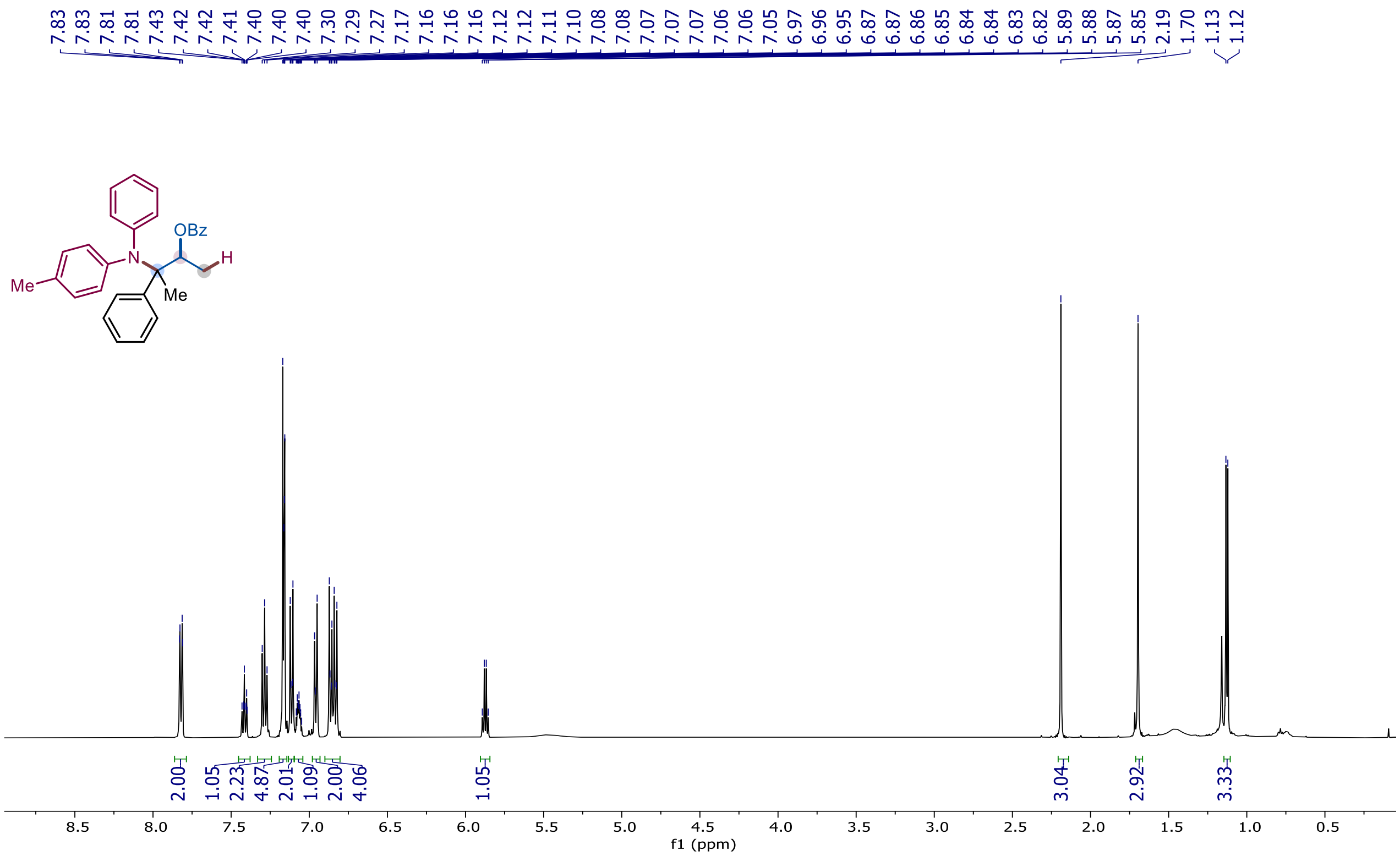

^{13}C NMR (126 MHz, $CDCl_3$, 25 °C) of 3al

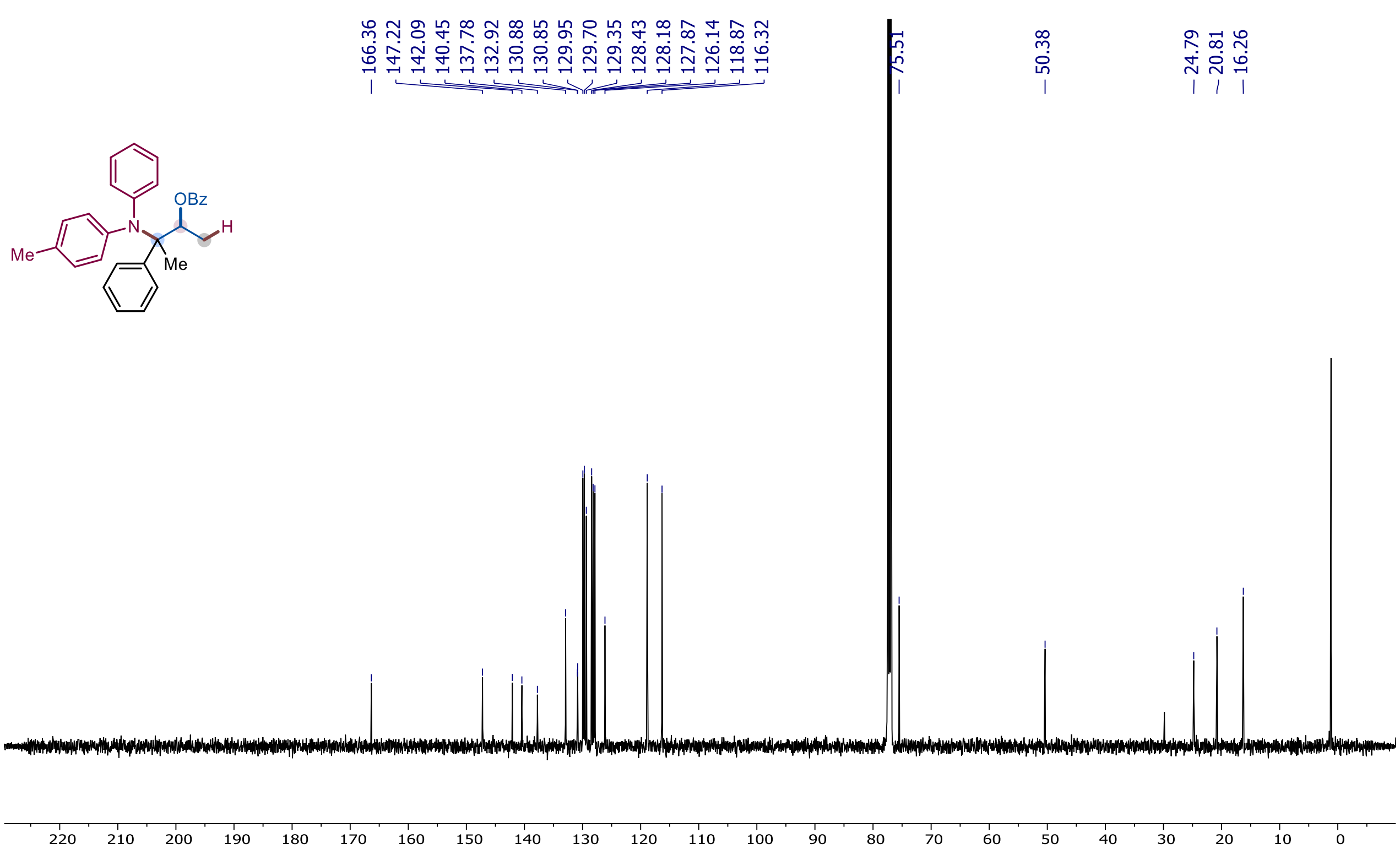

^{1}H NMR (400 MHz, CDCl$_3$, 25 ºC) of 3am

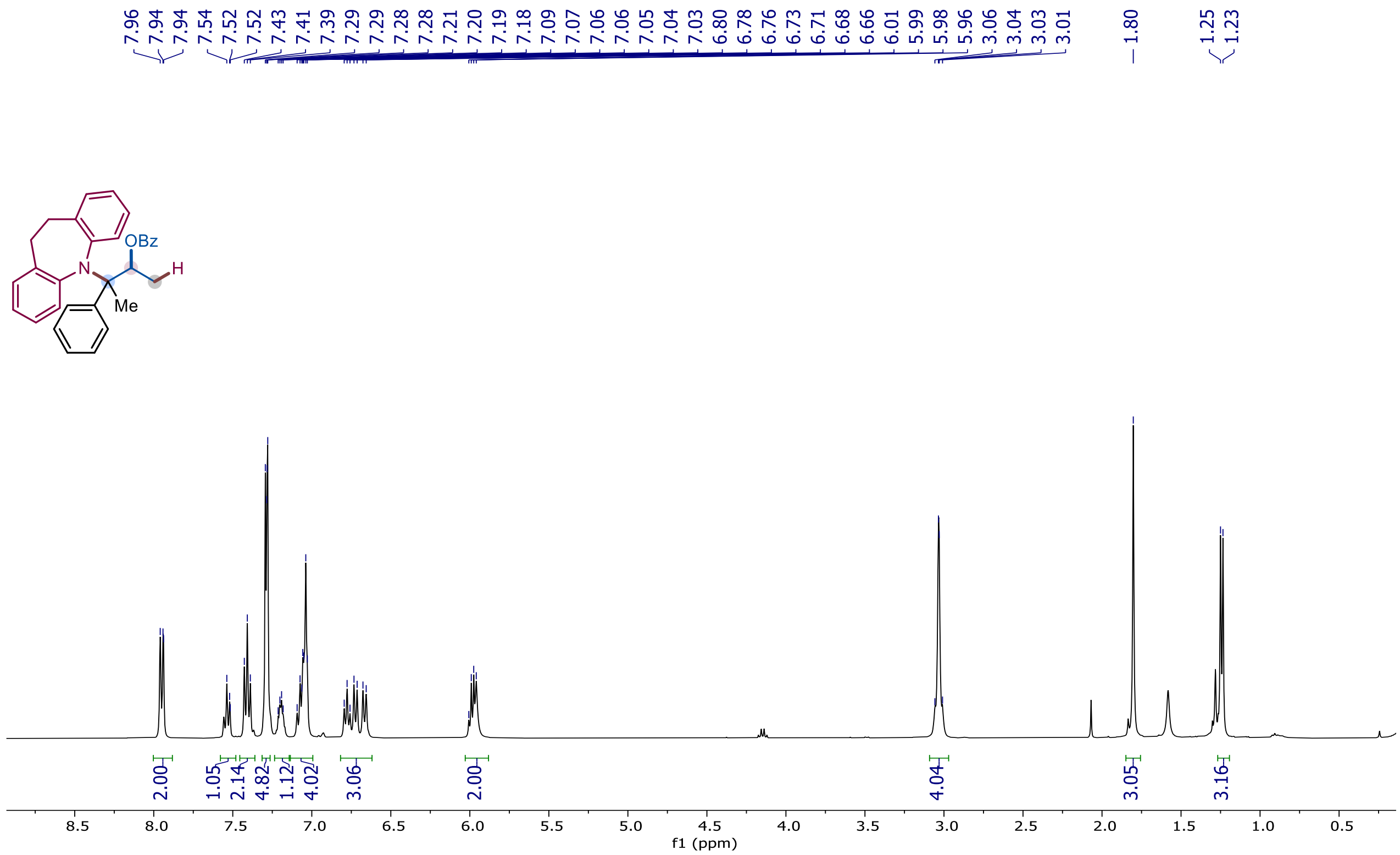

^{13}C NMR (126 MHz, CDCl$_3$, 25 ºC) of 3am

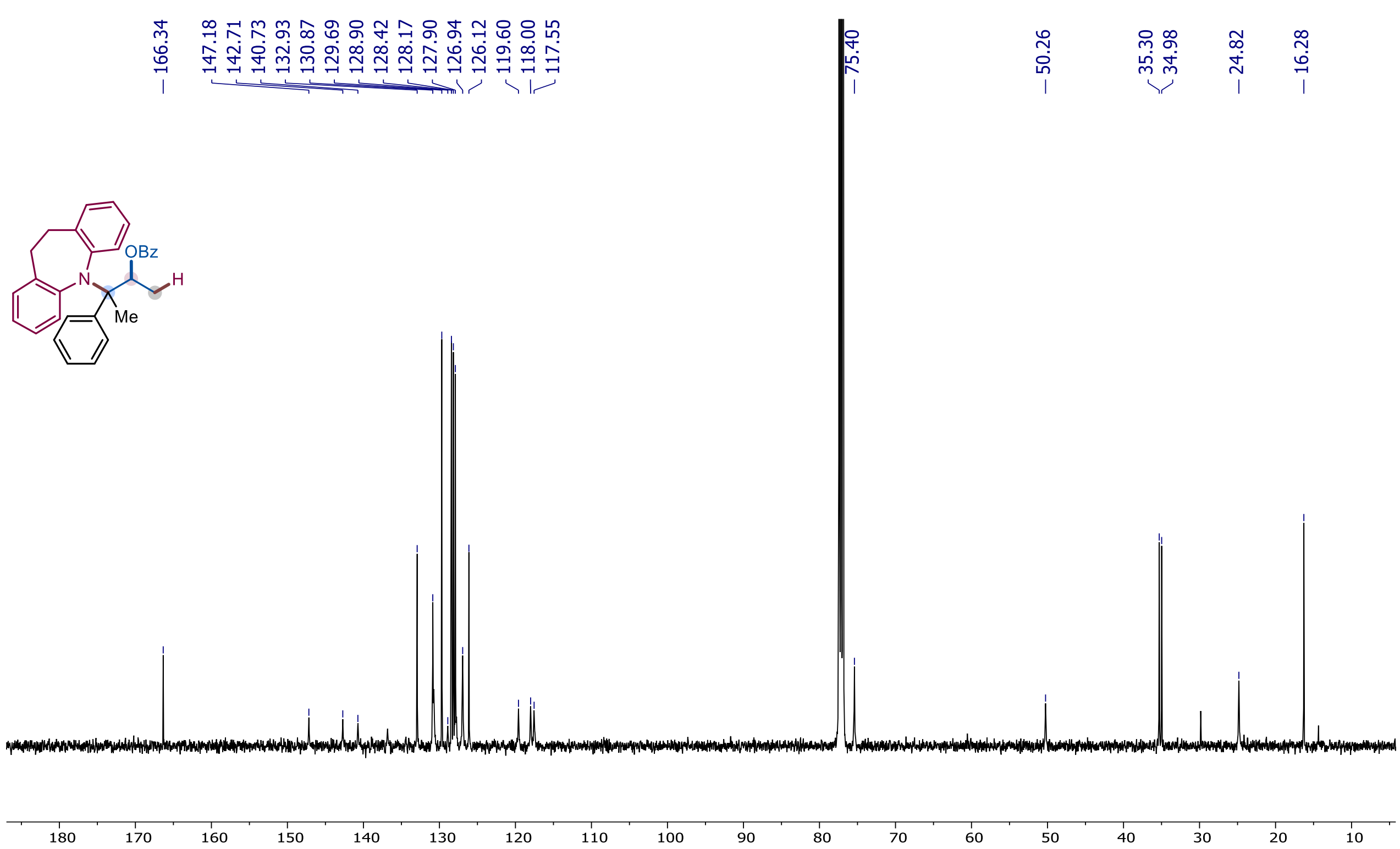

^{1}H NMR (300 MHz, $CDCl_3$, 25 ºC) of 3an

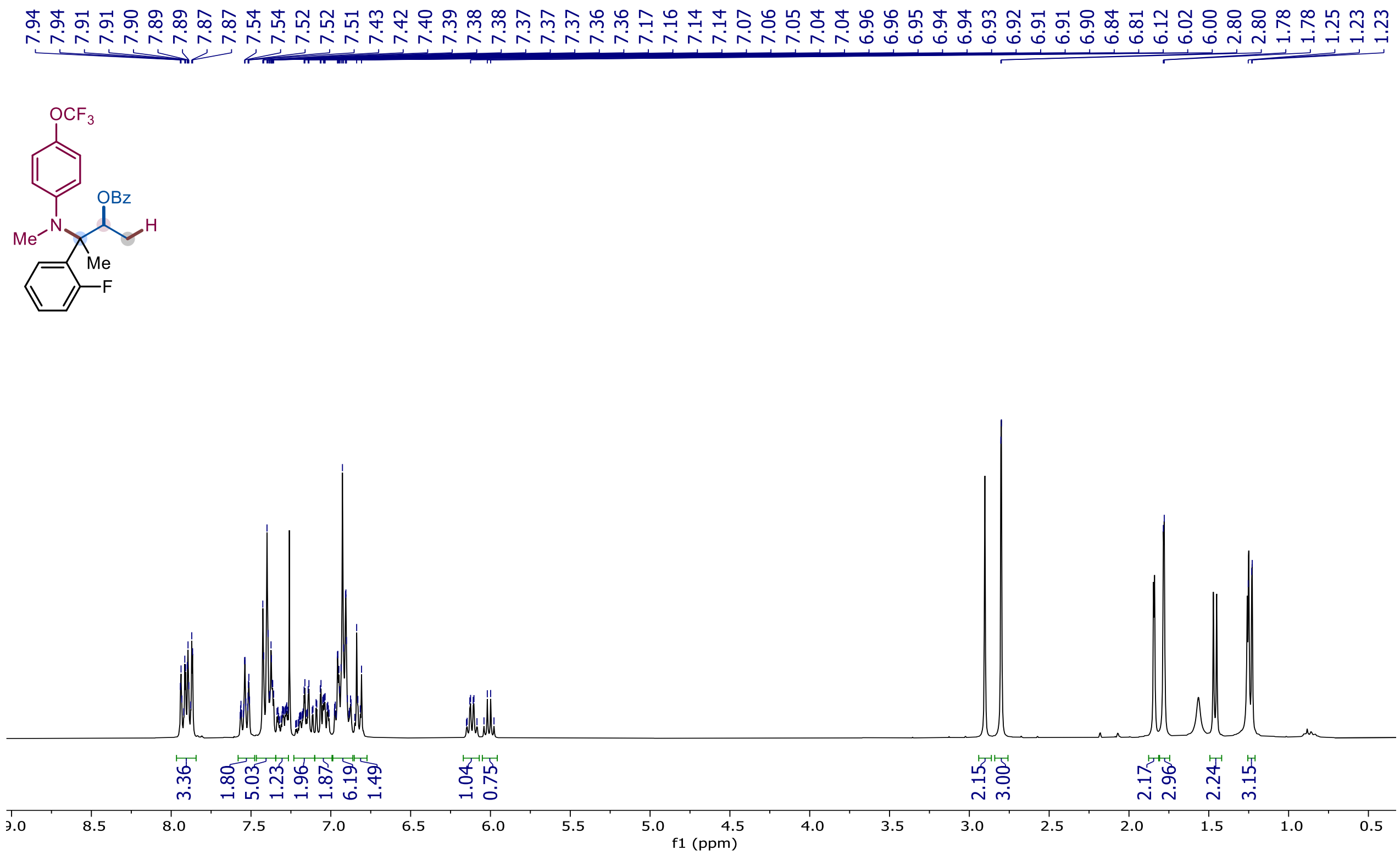

^{13}C NMR (201 MHz, $CDCl_3$, 25 ºC) of 3an

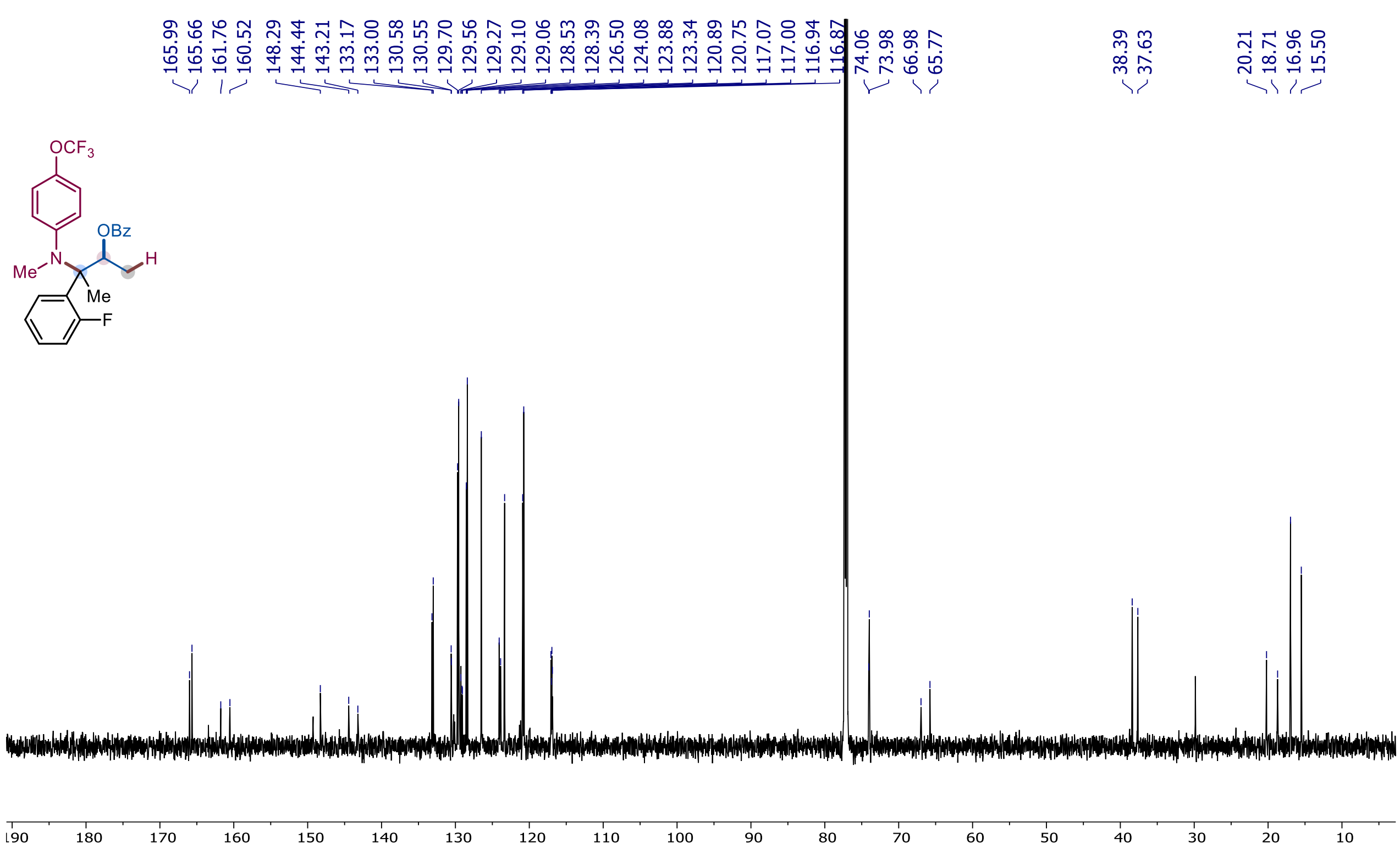

^{19}F NMR (282 MHz, $CDCl_3$, 25 ºC) of 3an

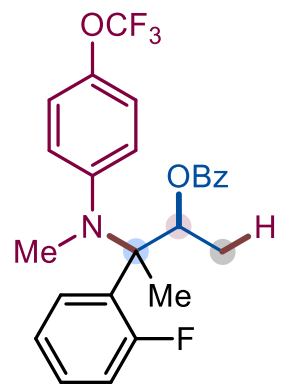

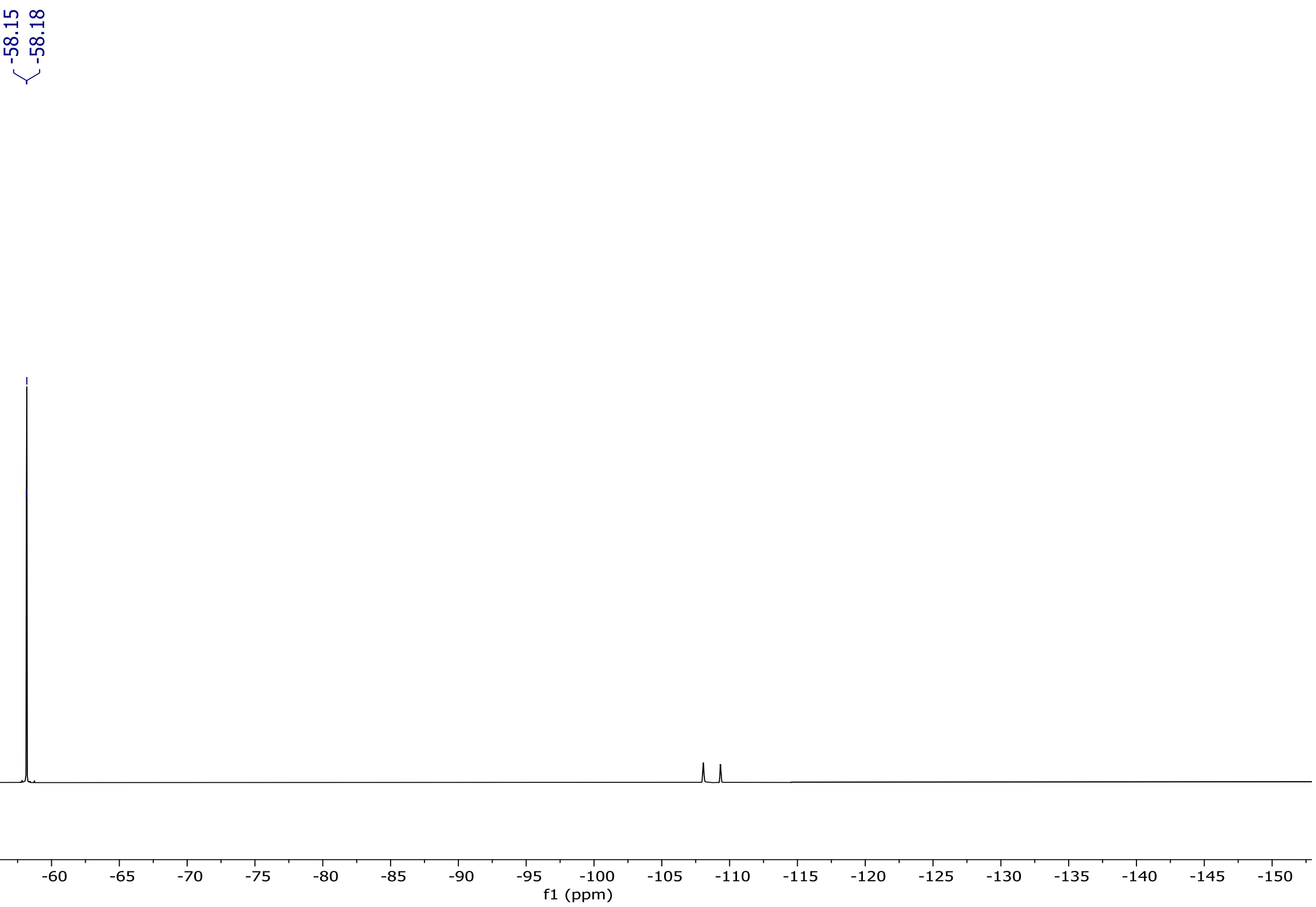

^{1}H NMR (400 MHz, CDCl$_3$, 25 °C) of 3ao

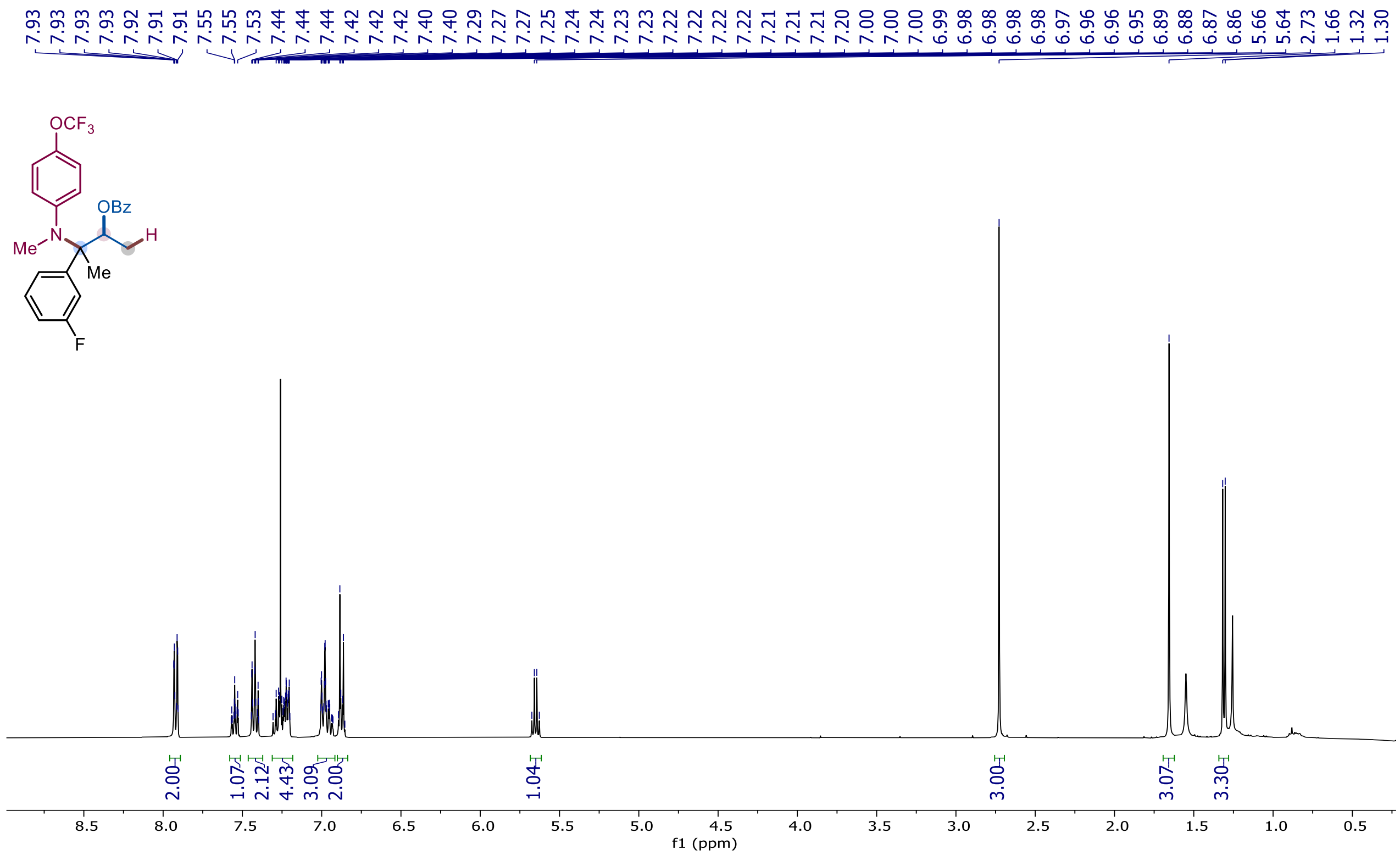

^{13}C NMR (126 MHz, CDCl$_3$, 25 °C) of 3ao

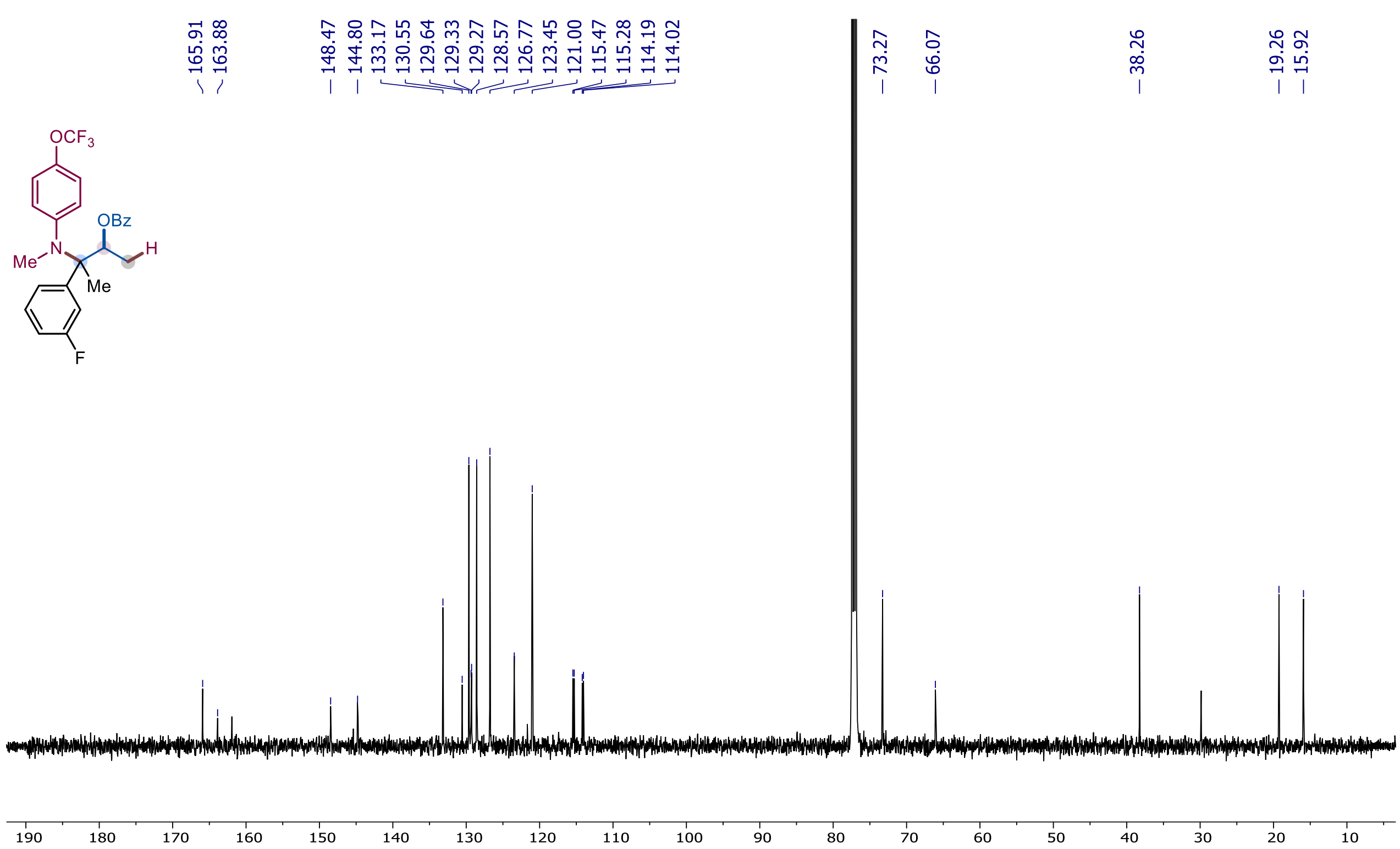

^{19}F NMR (376 MHz, $CDCl_3$, 25 °C) of 3ao

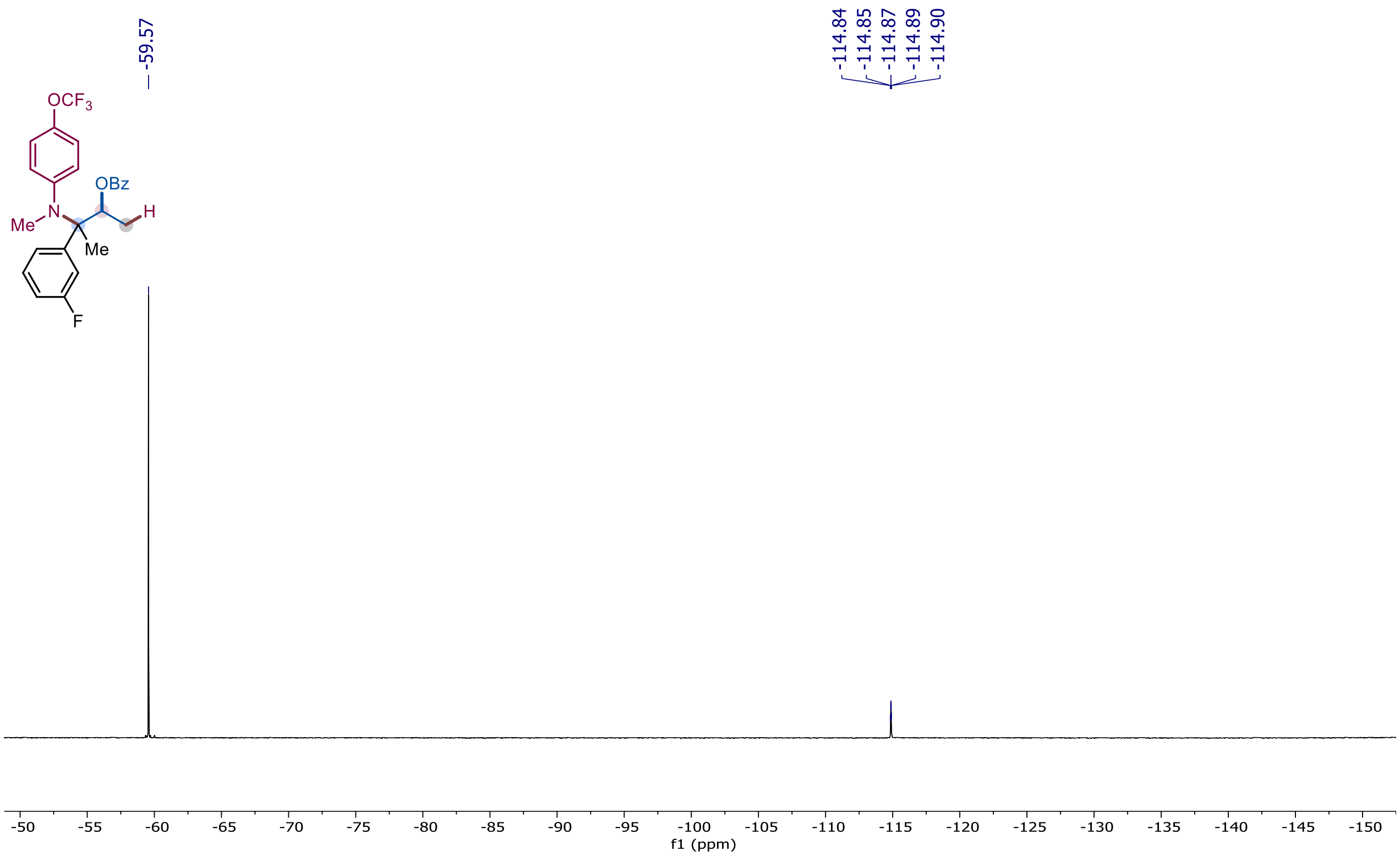

^{1}H NMR (500 MHz, CDCl$_3$, 25 ºC) of 3ap

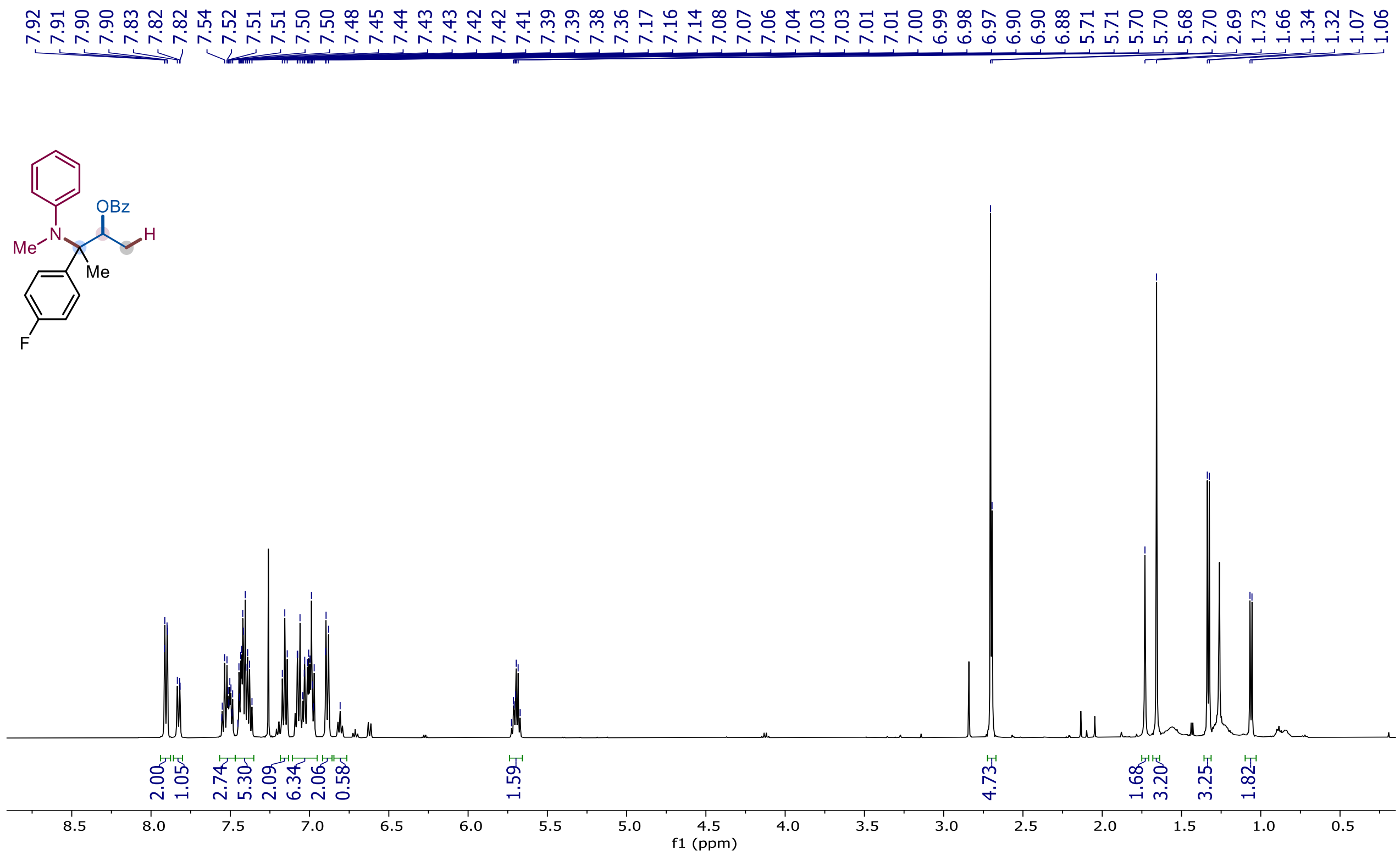

^{13}C NMR (126 MHz, CDCl$_3$, 25 ºC) of 3ap

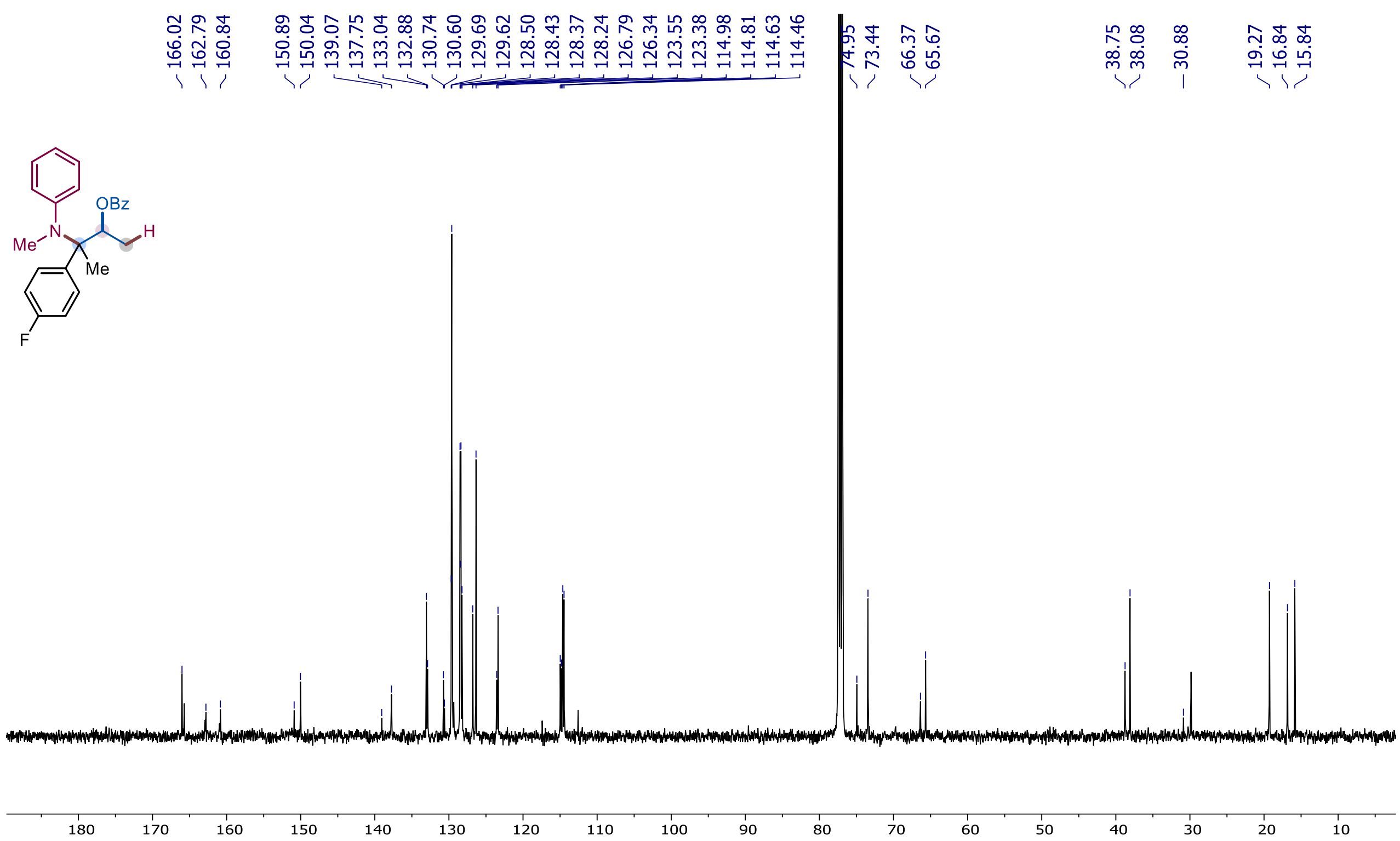

^{19}F NMR (470 MHz, CDCl₃, 25 °C) of 3ap

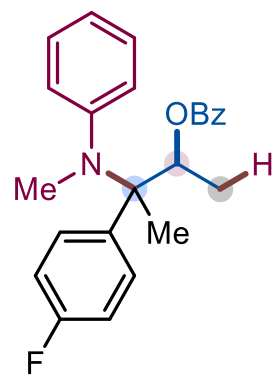

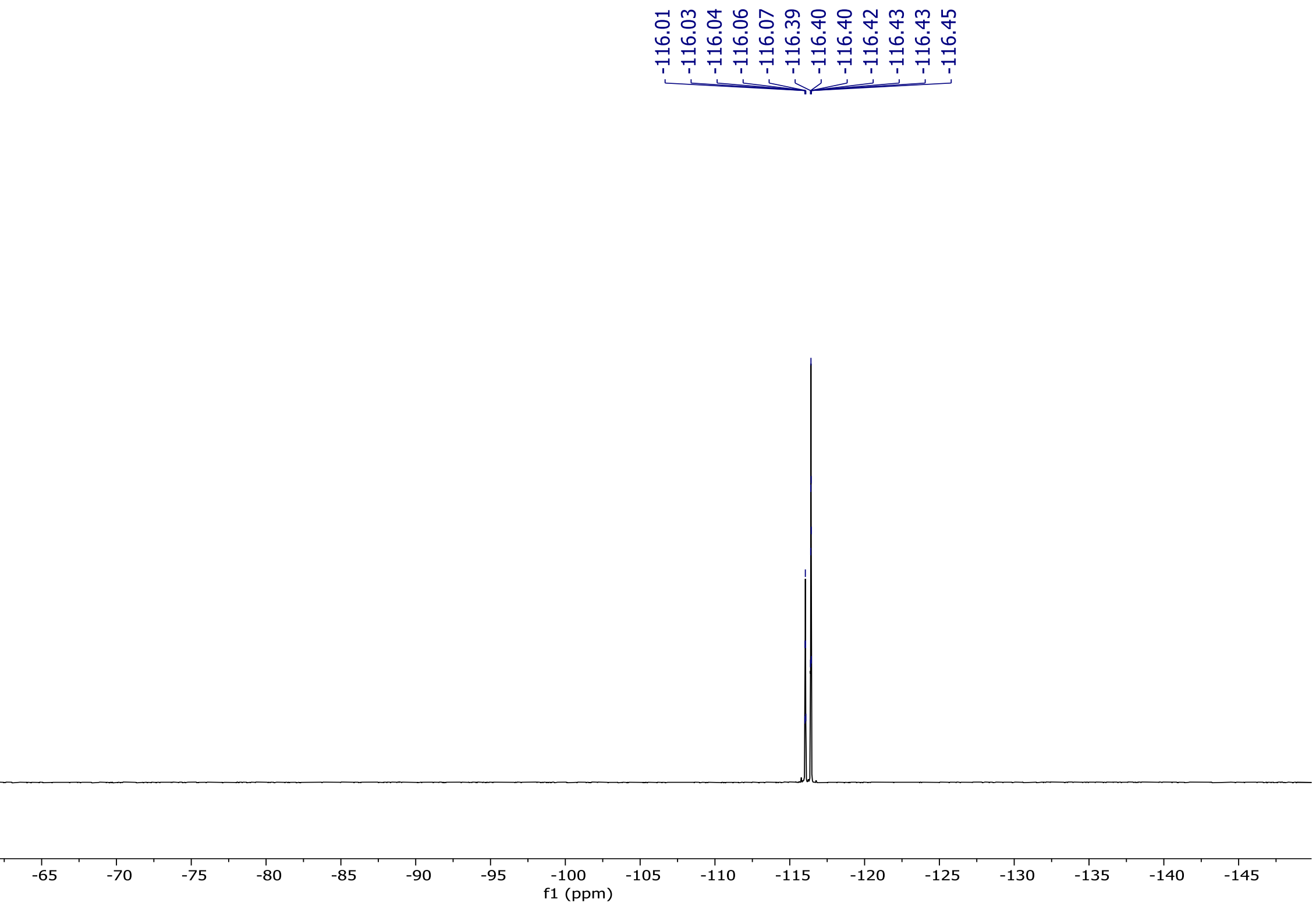

^{1}H NMR (300 MHz, $CDCl_3$, 25 °C) of 3aq

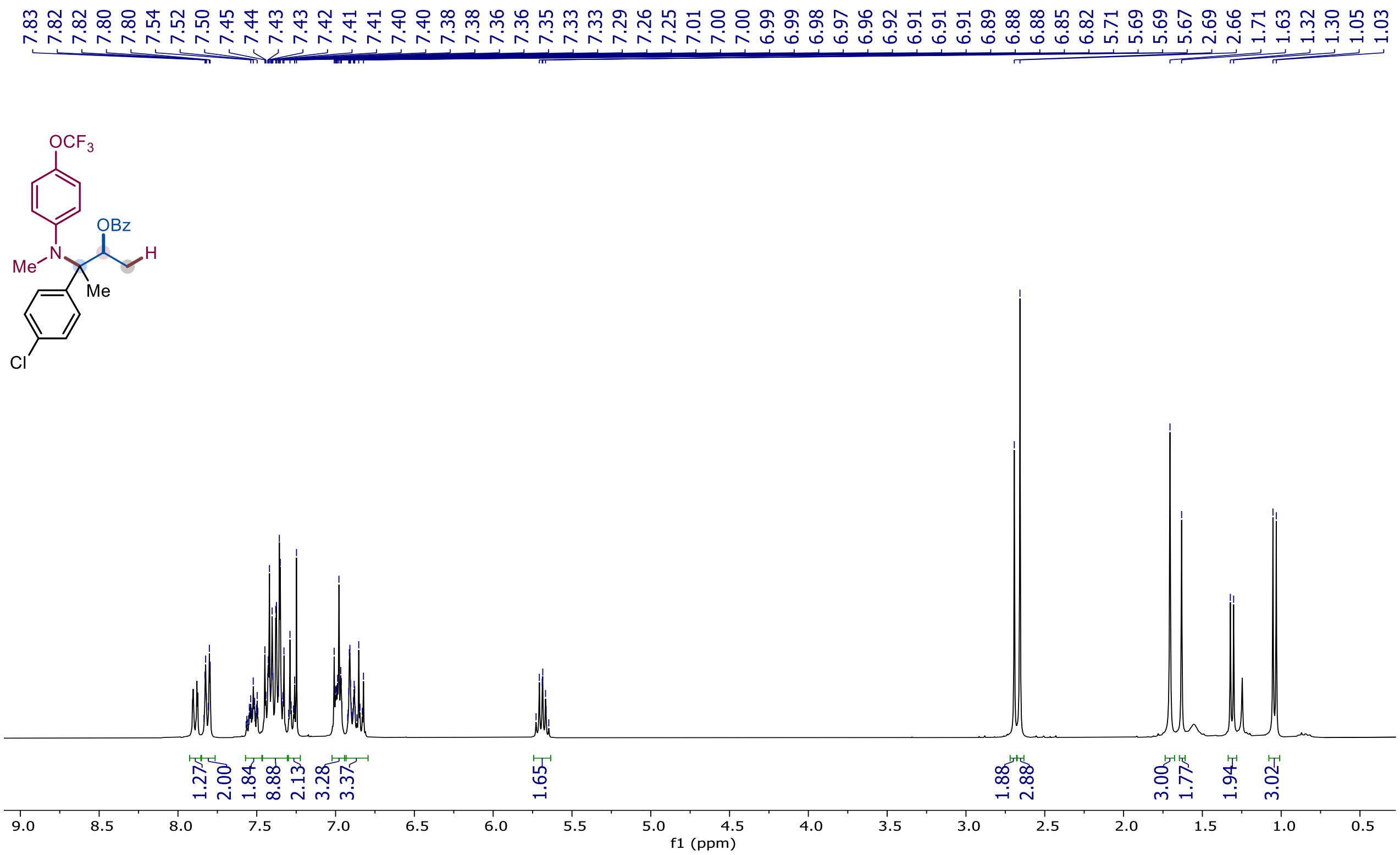

^{13}C NMR (201 MHz, $CDCl_3$, 25 °C) of 3aq

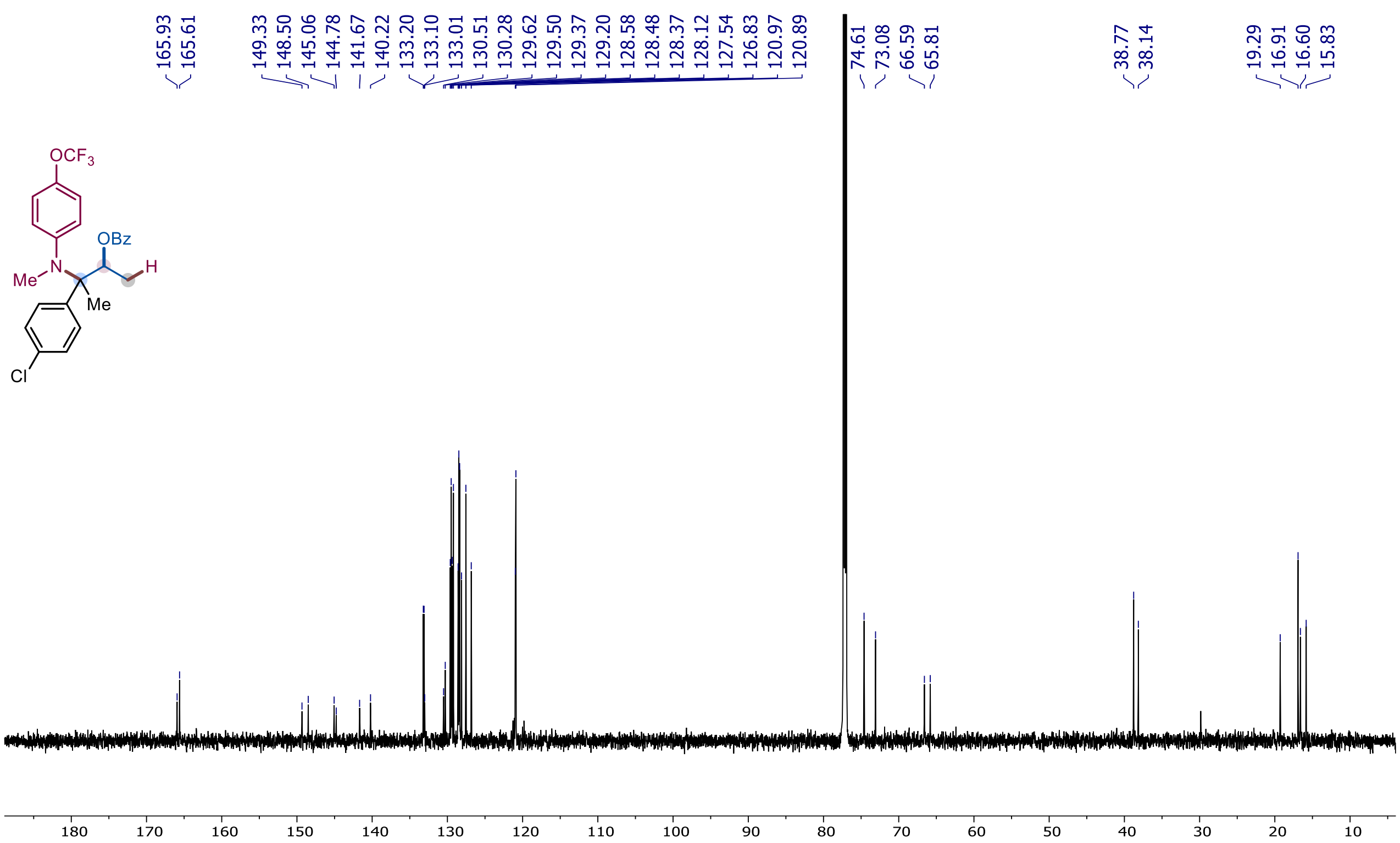

^{19}F NMR (282 MHz, $CDCl_3$, 25 ºC) of 3aq

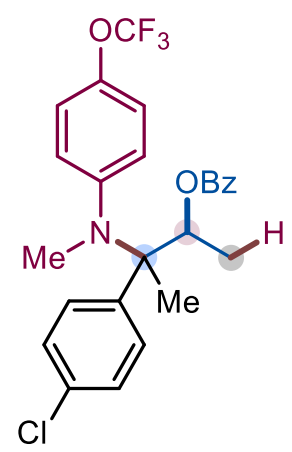

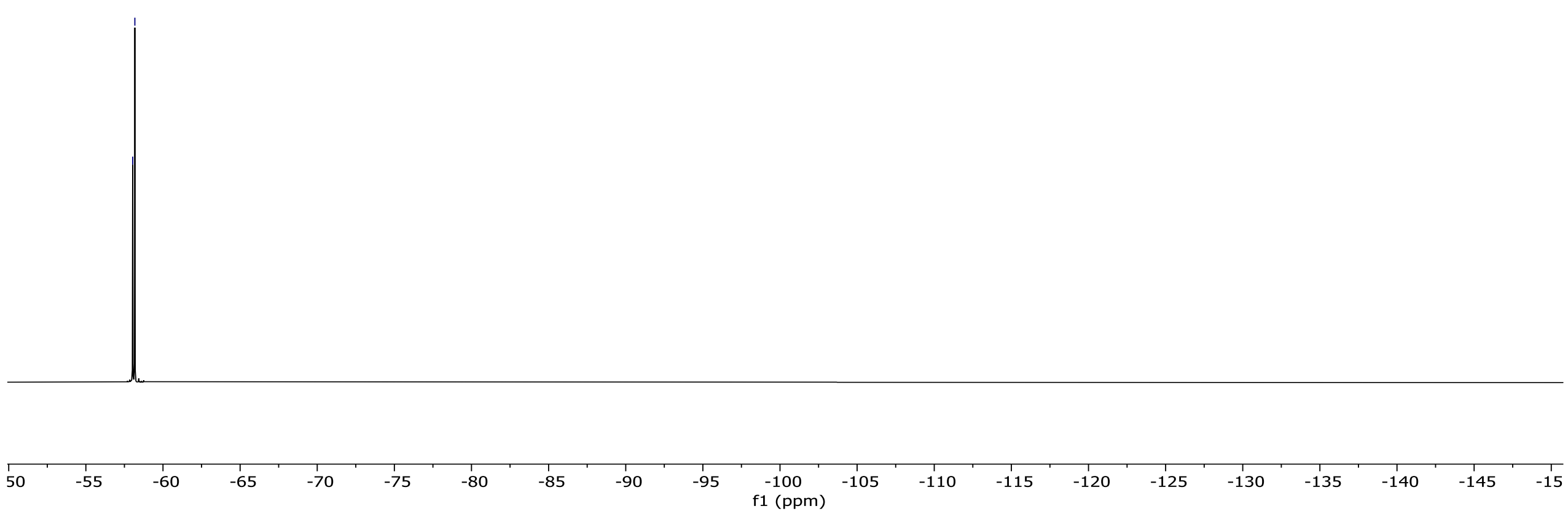

^{1}H NMR (300 MHz, $CDCl_3$, 25 ºC) of 3ar (Diastereomer 1)

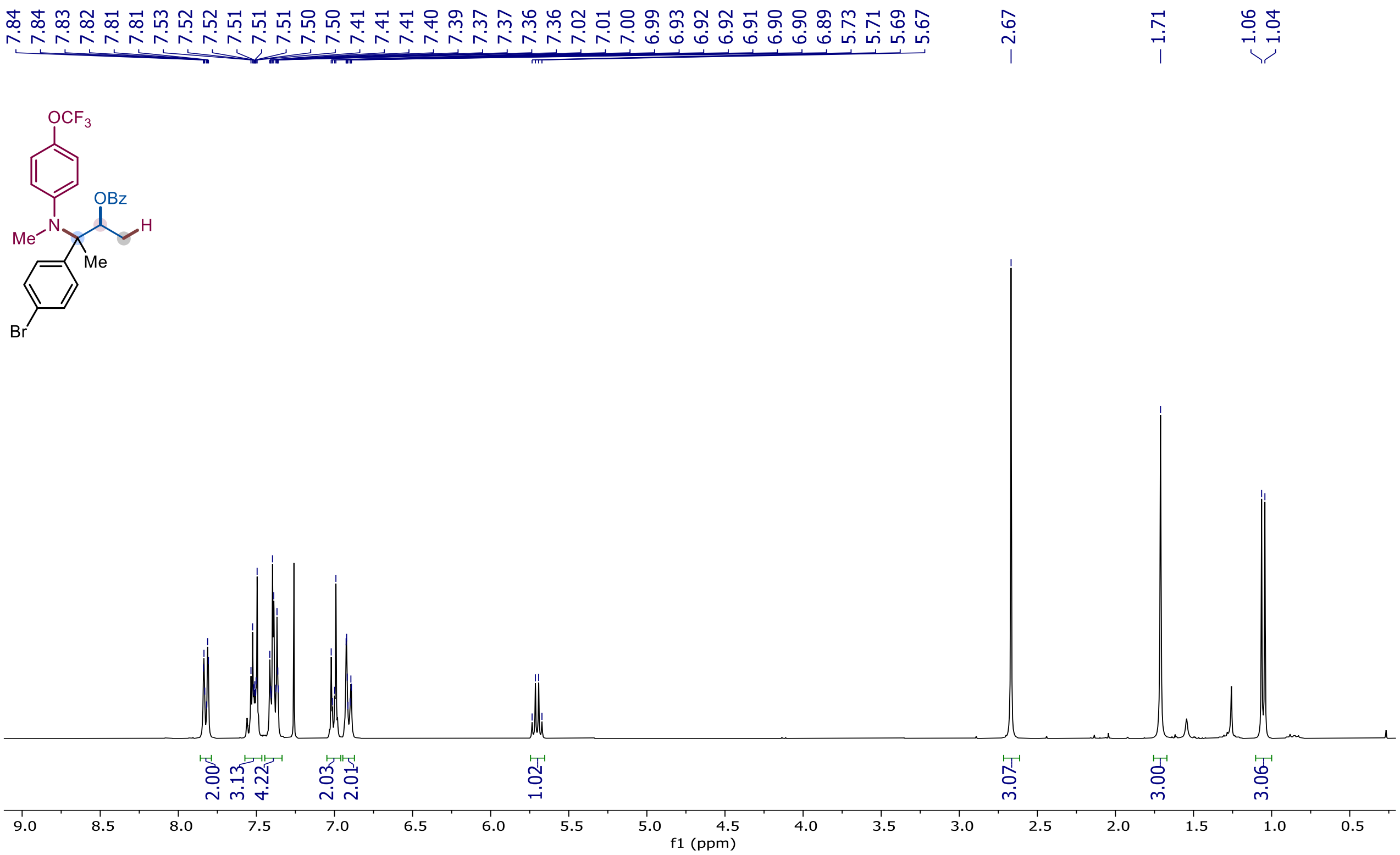

^{13}C NMR (201 MHz, $CDCl_3$, 25 ºC) of 3ar (Diastereomer 1)

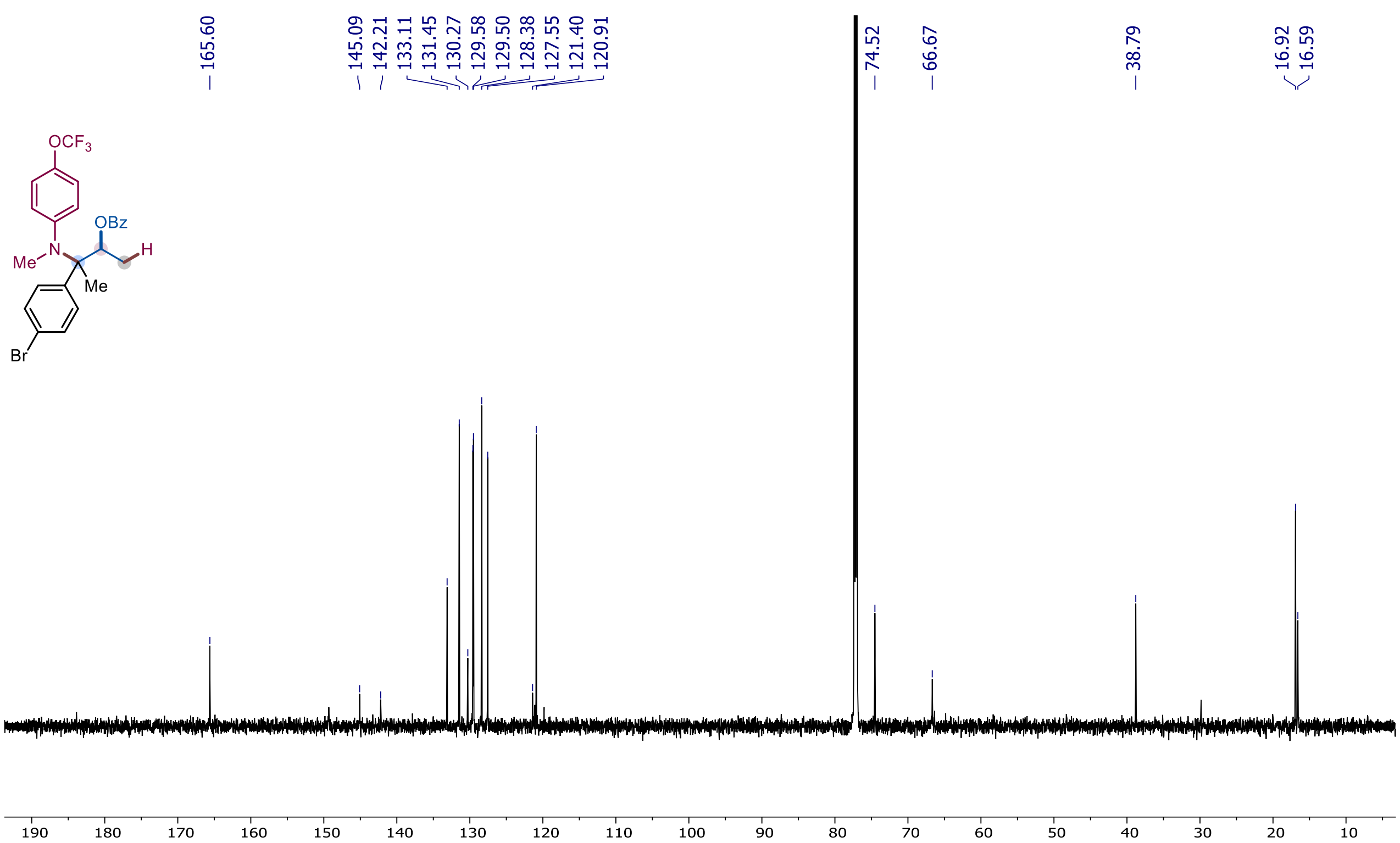

^{19}F NMR (282 MHz, $CDCl_3$, 25 ºC) of 3ar (Diastereomer 1)

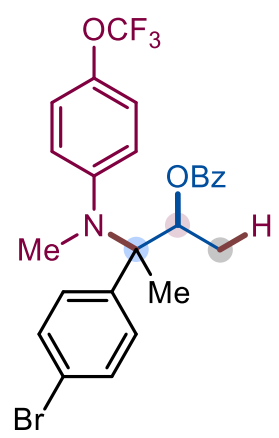

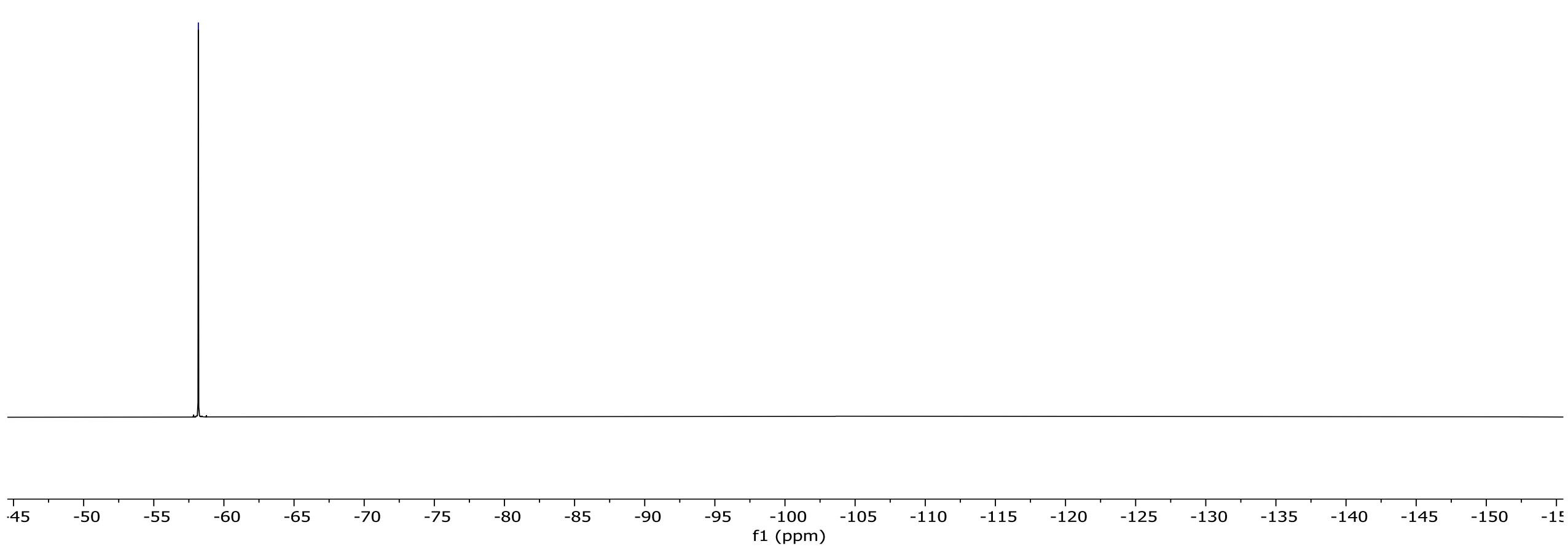

^{1}H NMR (300 MHz, CDCl$_3$, 25 ºC) of 3ar (Diastereomer 2)

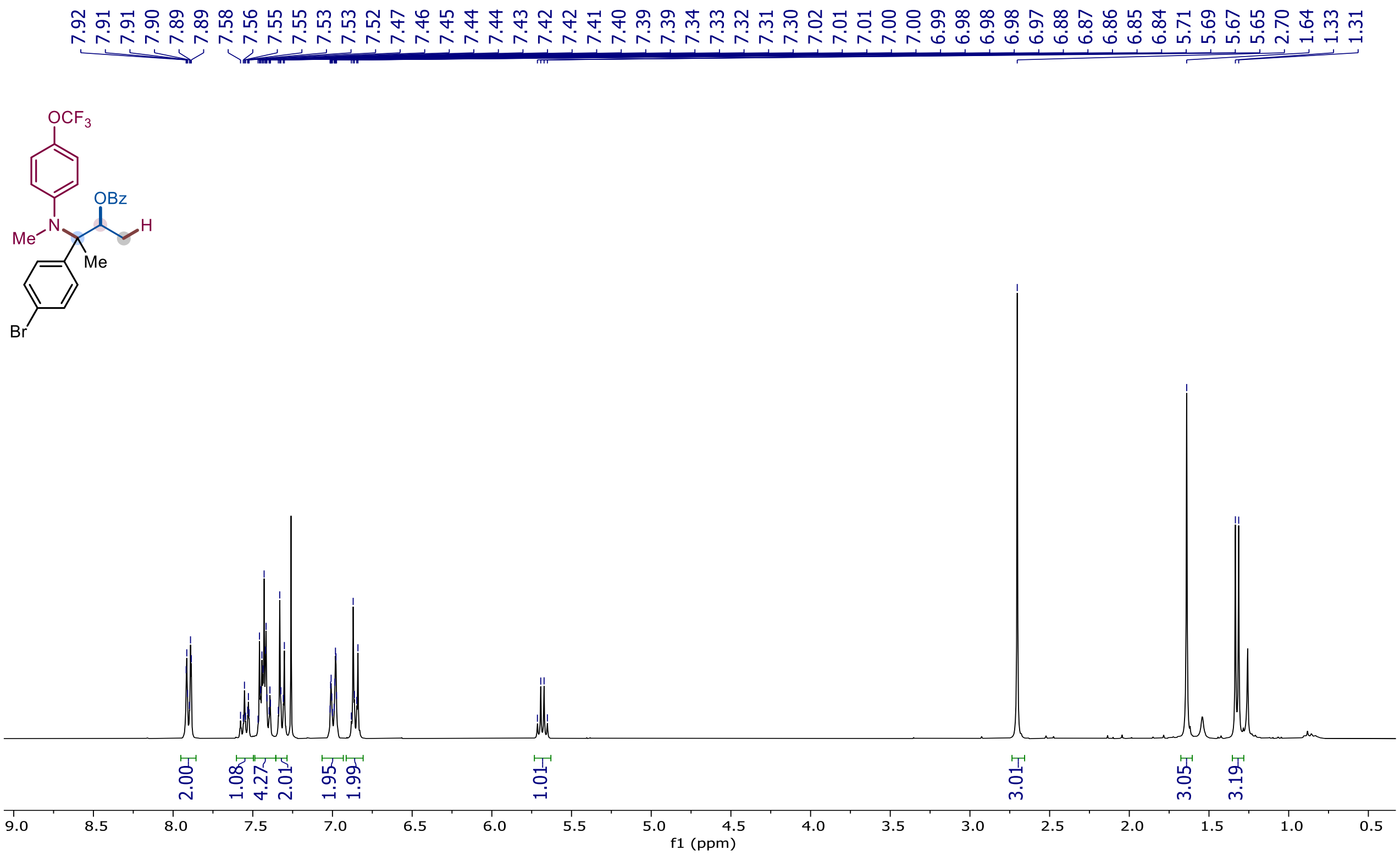

^{13}C NMR (201 MHz, CDCl$_3$, 25 ºC) of 3ar (Diastereomer 2)

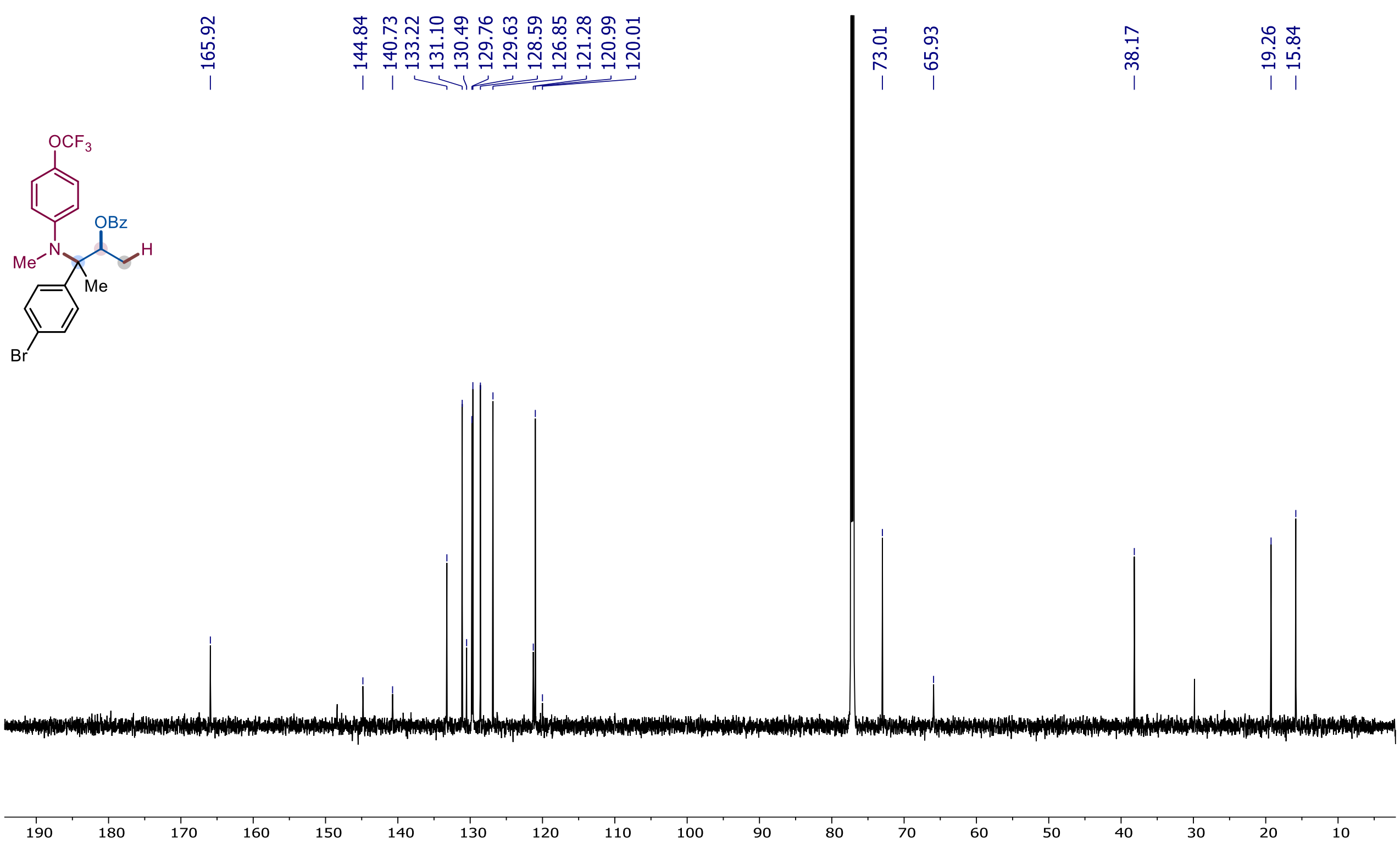

^{19}F NMR (282 MHz, CDCl$_3$, 25 ºC) of 3ar (Diastereomer 2)

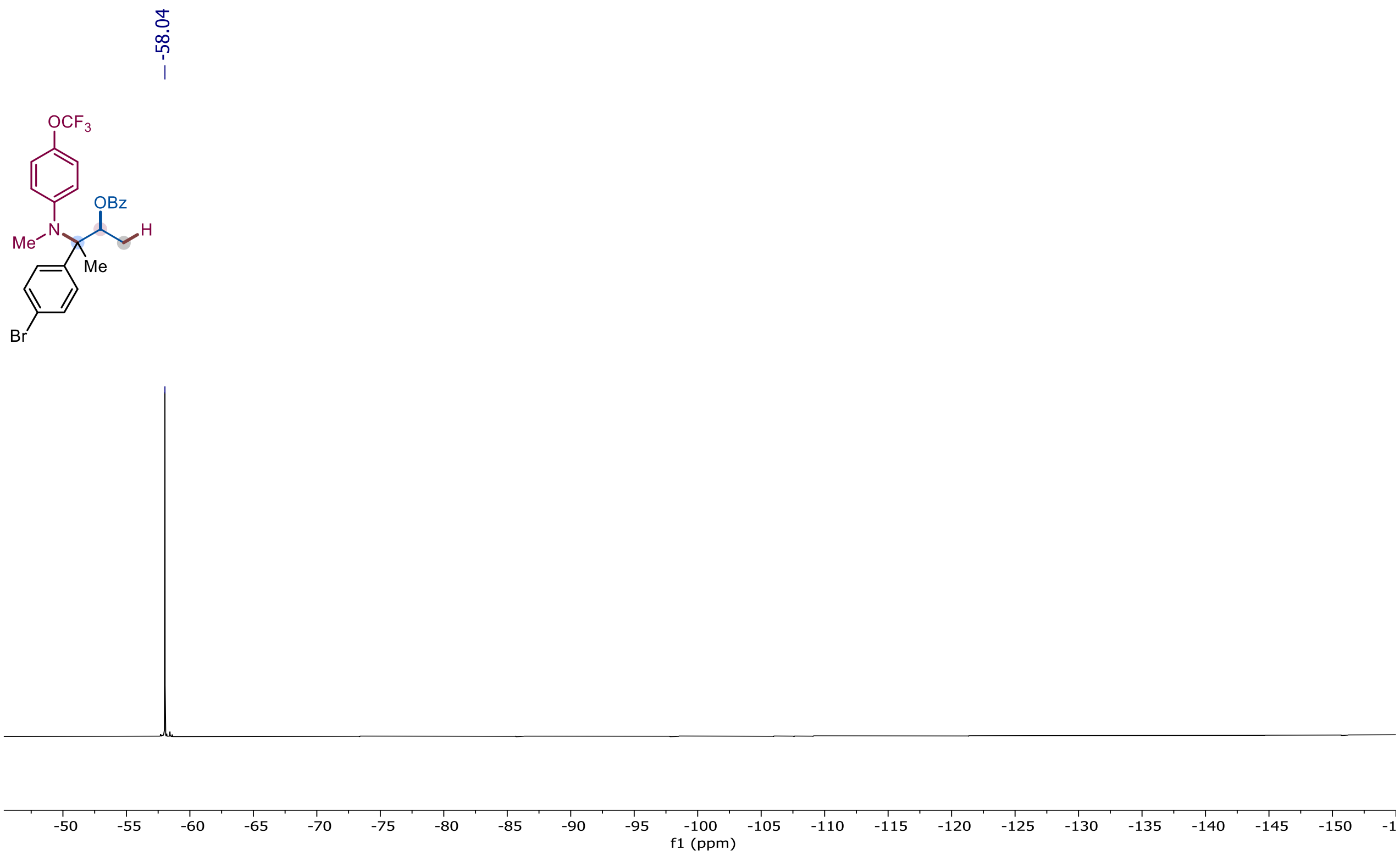

^{1}H NMR (500 MHz, $CDCl_3$, 25 °C) of 3as

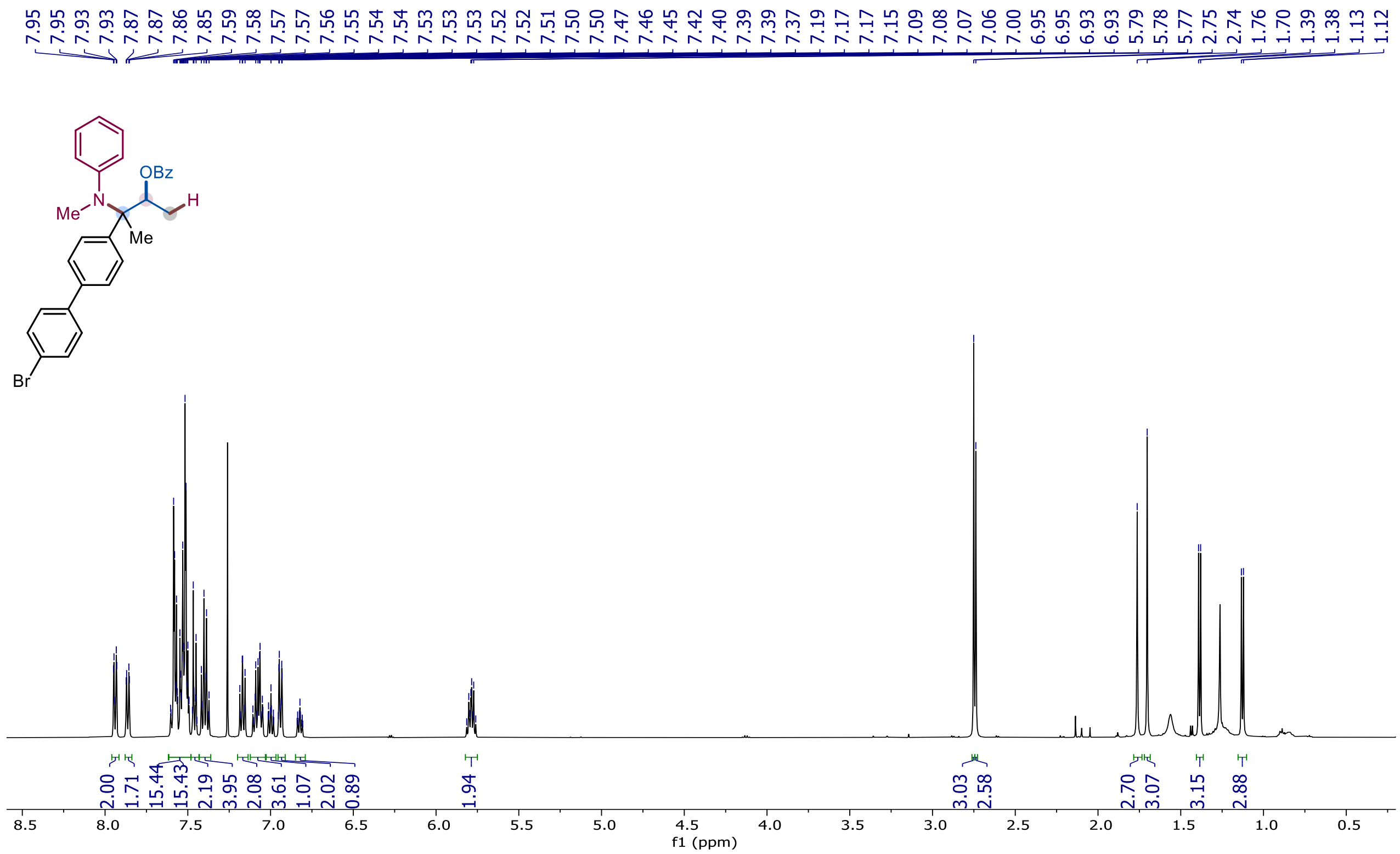

^{13}C NMR (126 MHz, $CDCl_3$, 25 °C) of 3as

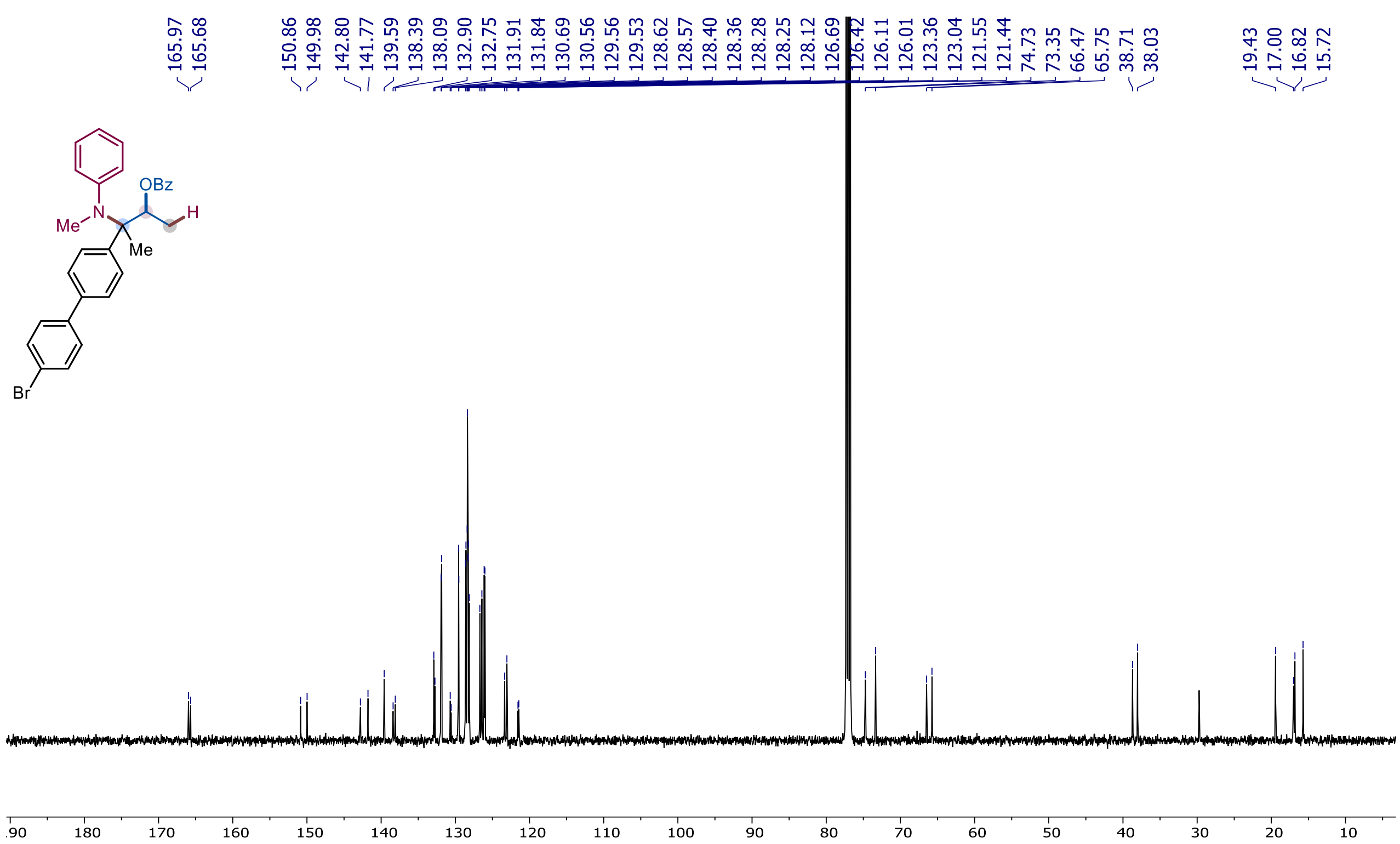

^{1}H NMR (500 MHz, $CDCl_3$, 25 ºC) of 3at

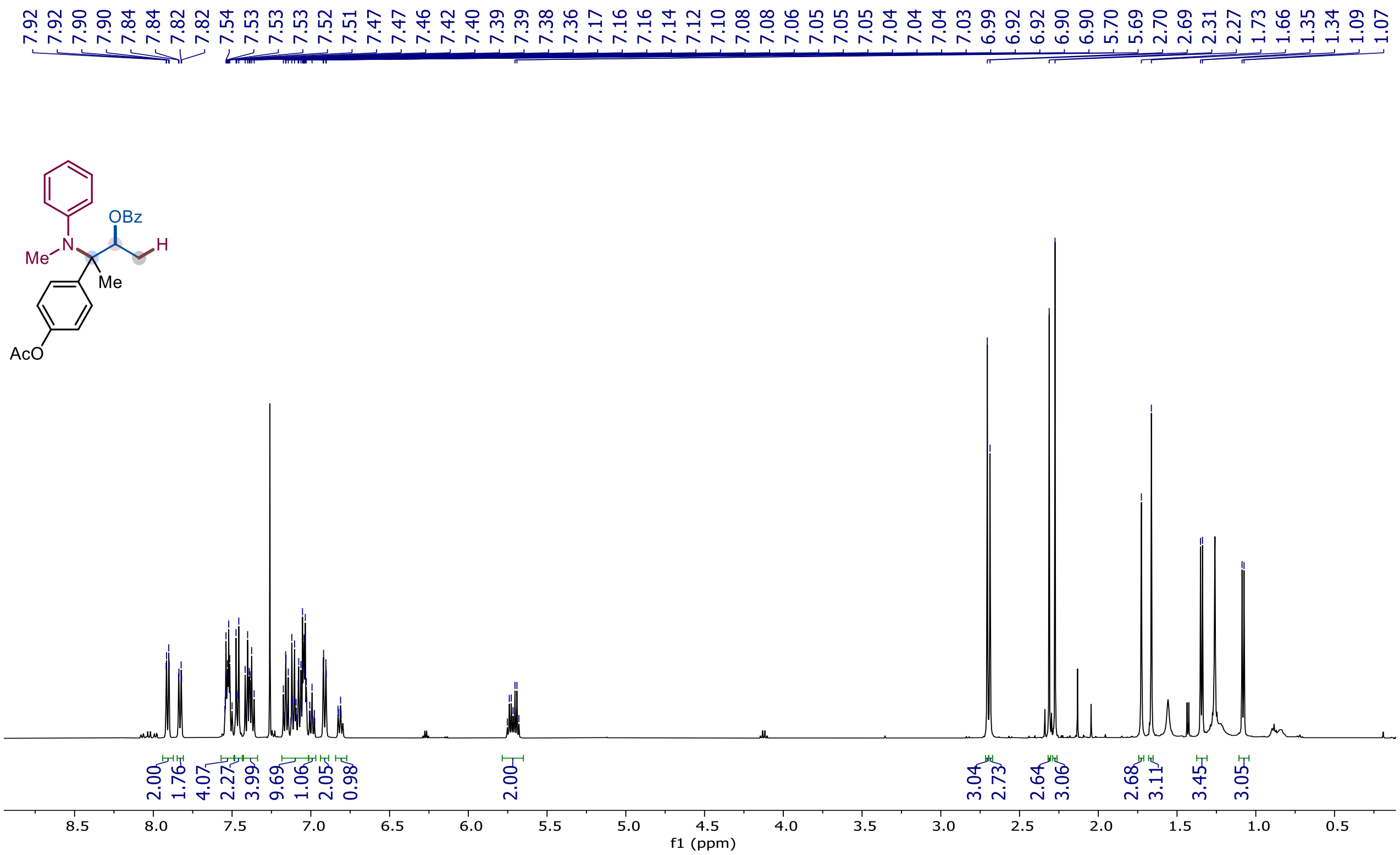

^{13}C NMR (126 MHz, $CDCl_3$, 25 ºC) of 3at

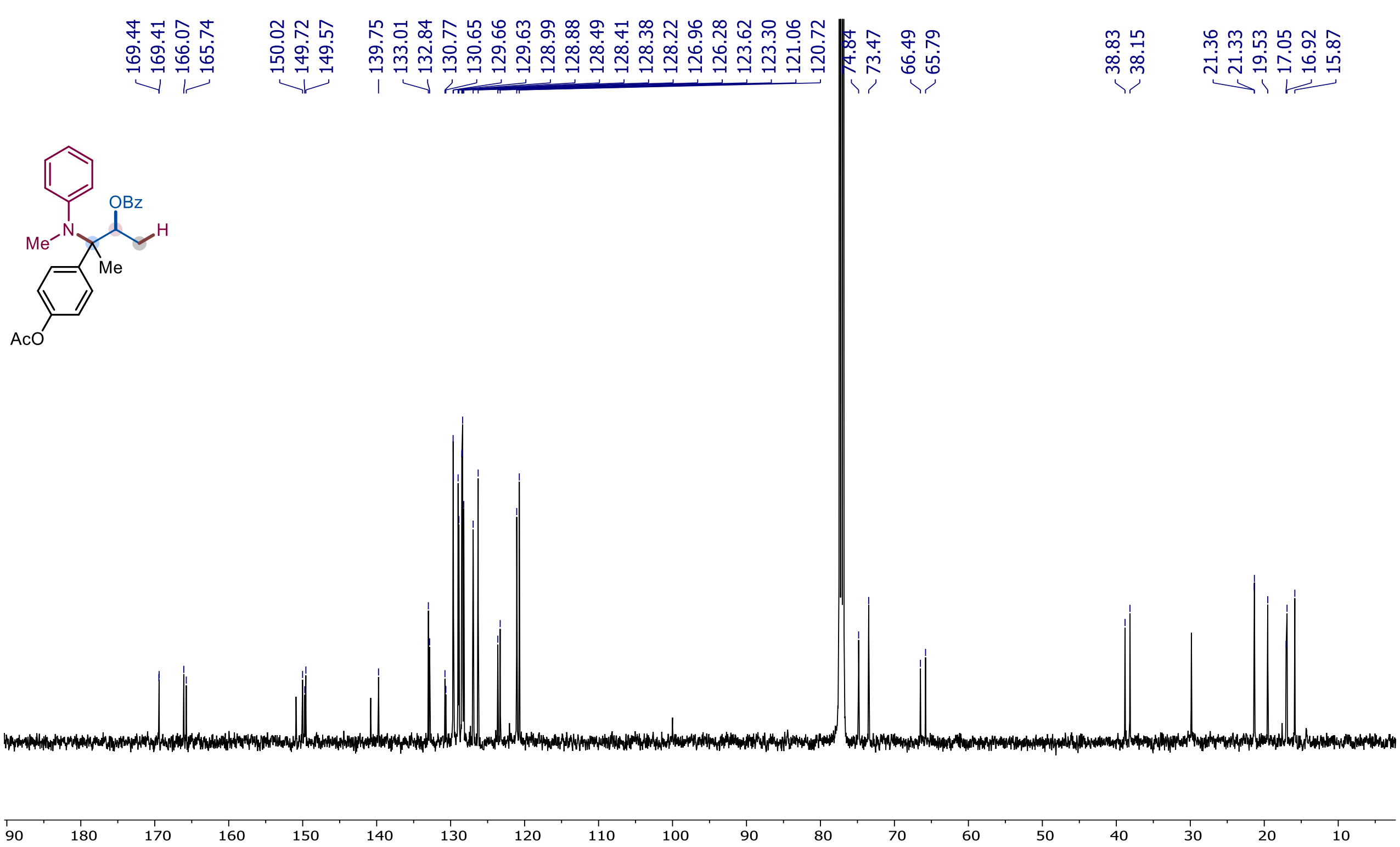

^{1}H NMR (800 MHz, $CDCl_3$, 25 °C) of 3au

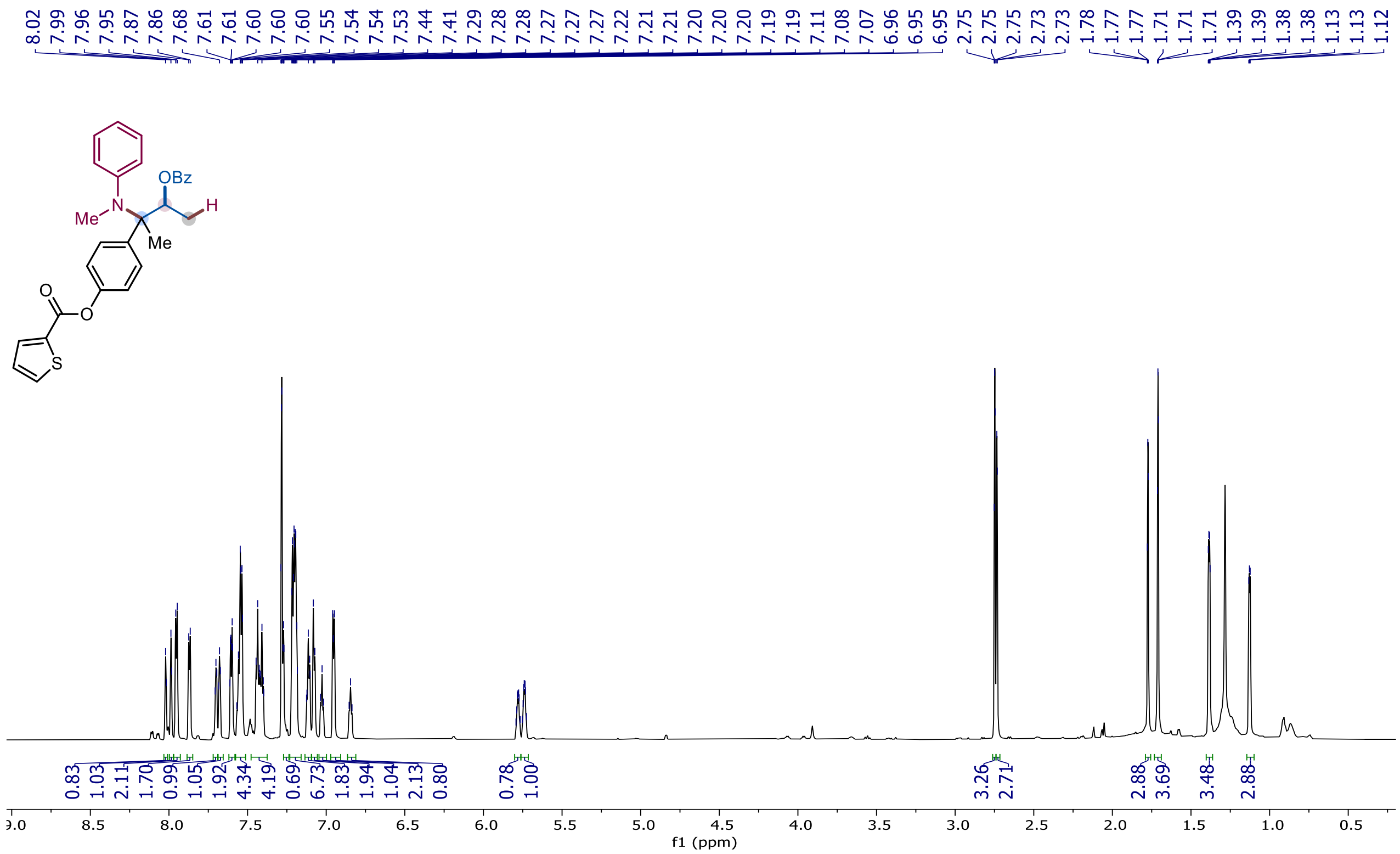

^{13}C NMR (201 MHz, CD_3CN, 25 °C) of 3au

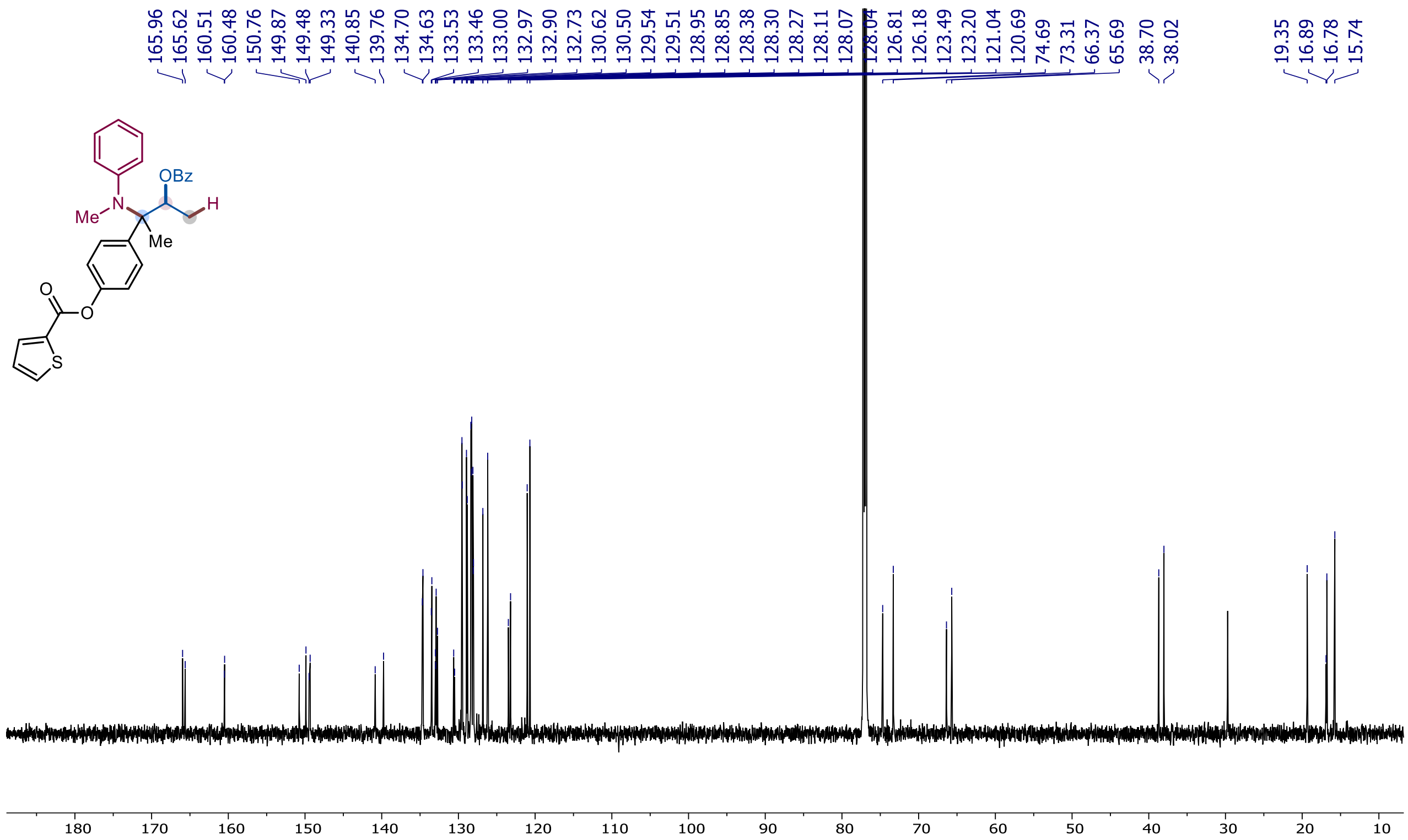

^{1}H NMR (800 MHz, $CDCl_3$, 25 ºC) of 3av

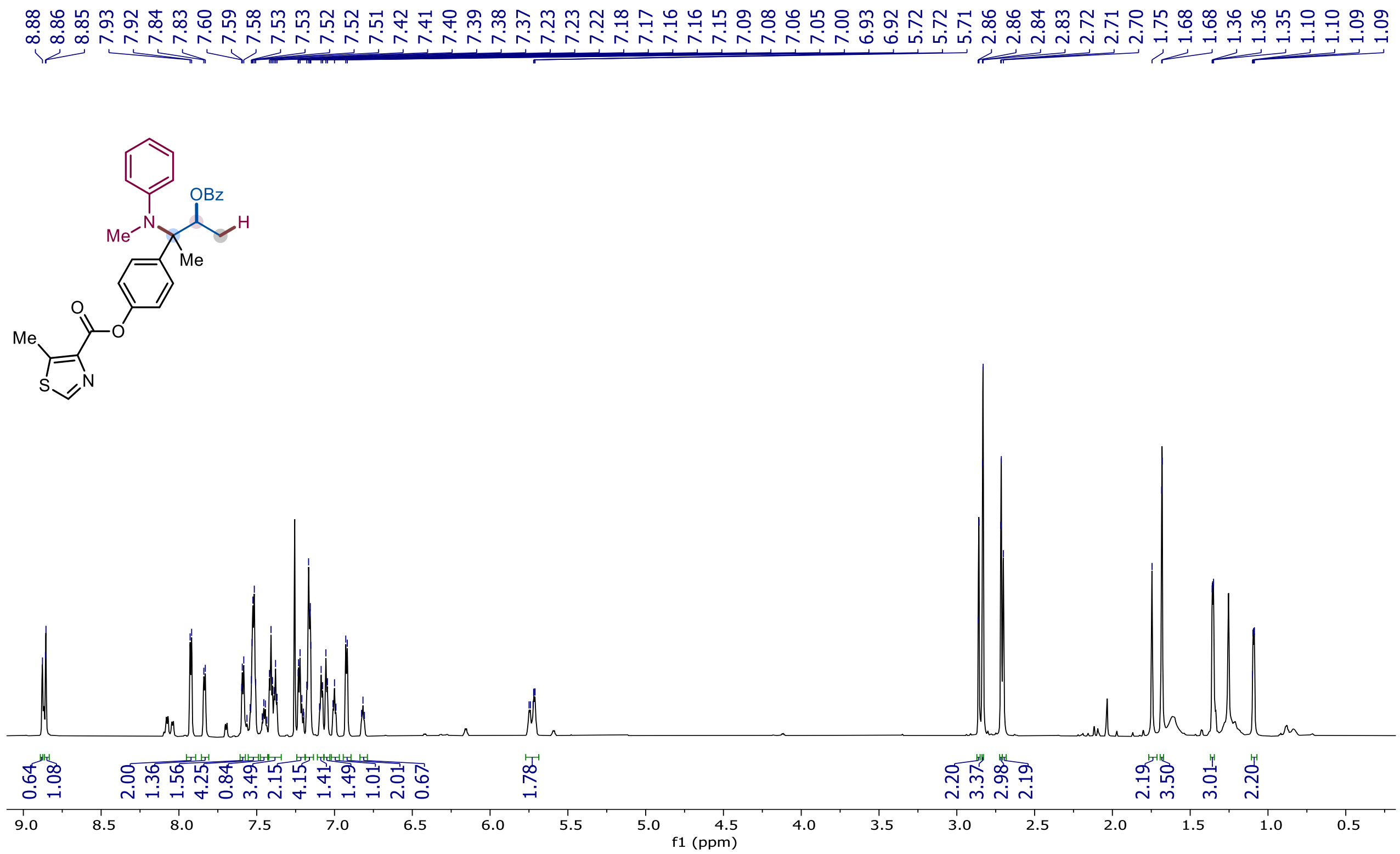

^{13}C NMR (201 MHz, $CDCl_3$, 25 ºC) of 3av

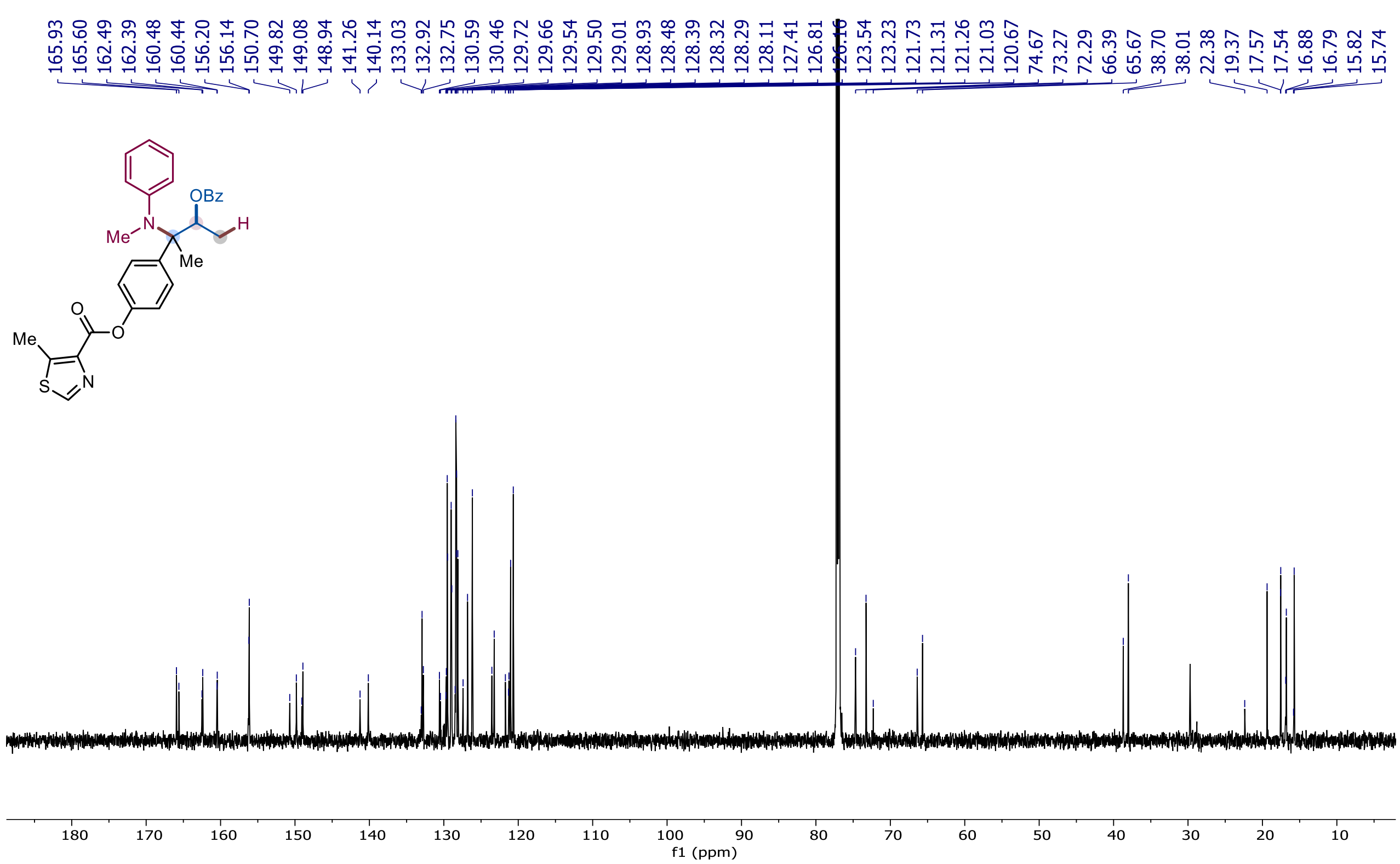

^{1}H NMR (800 MHz, CDCl₃, 25 ºC) of 3aw

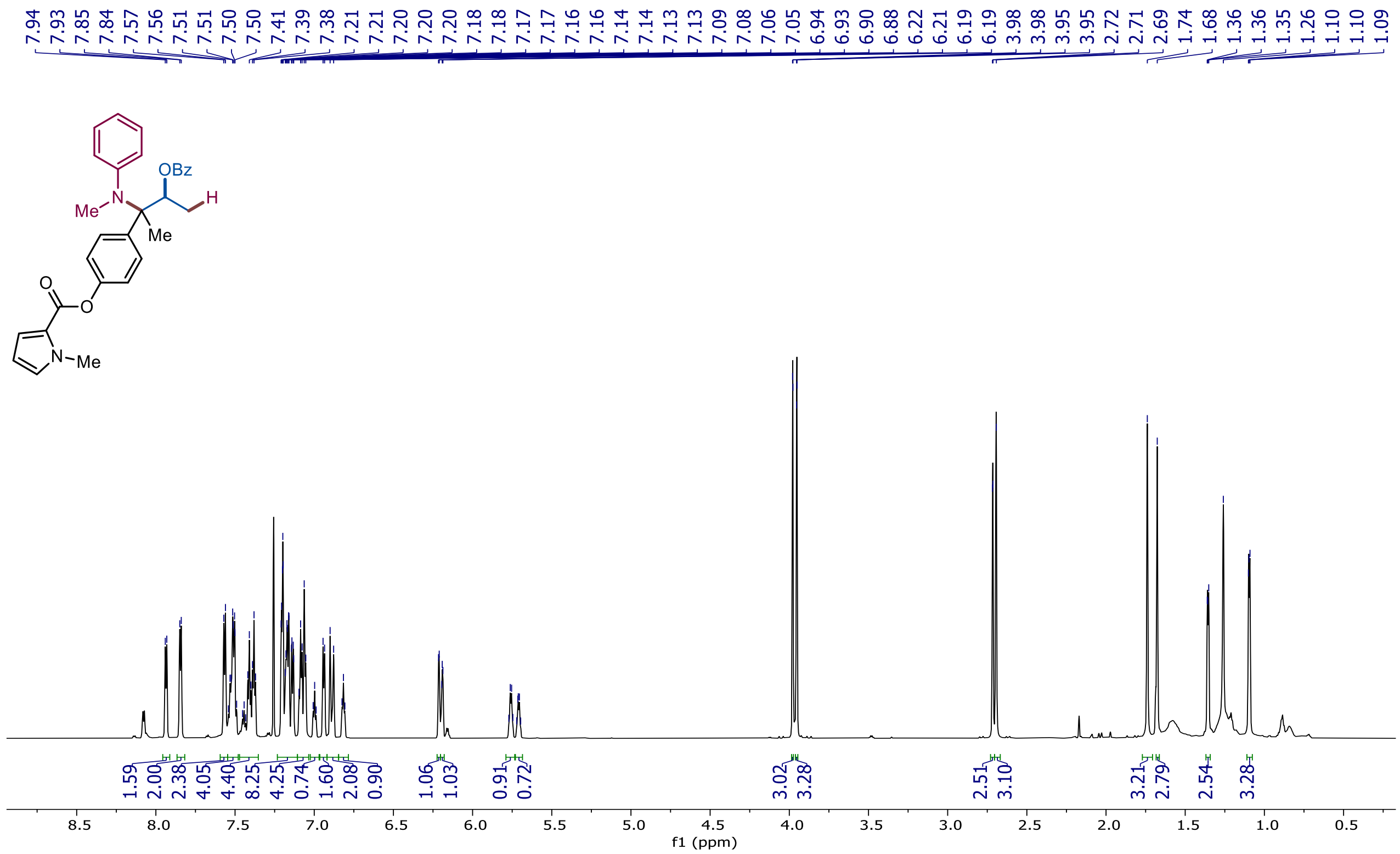

^{13}C NMR (201 MHz, CDCl₃, 25 ºC) of 3aw

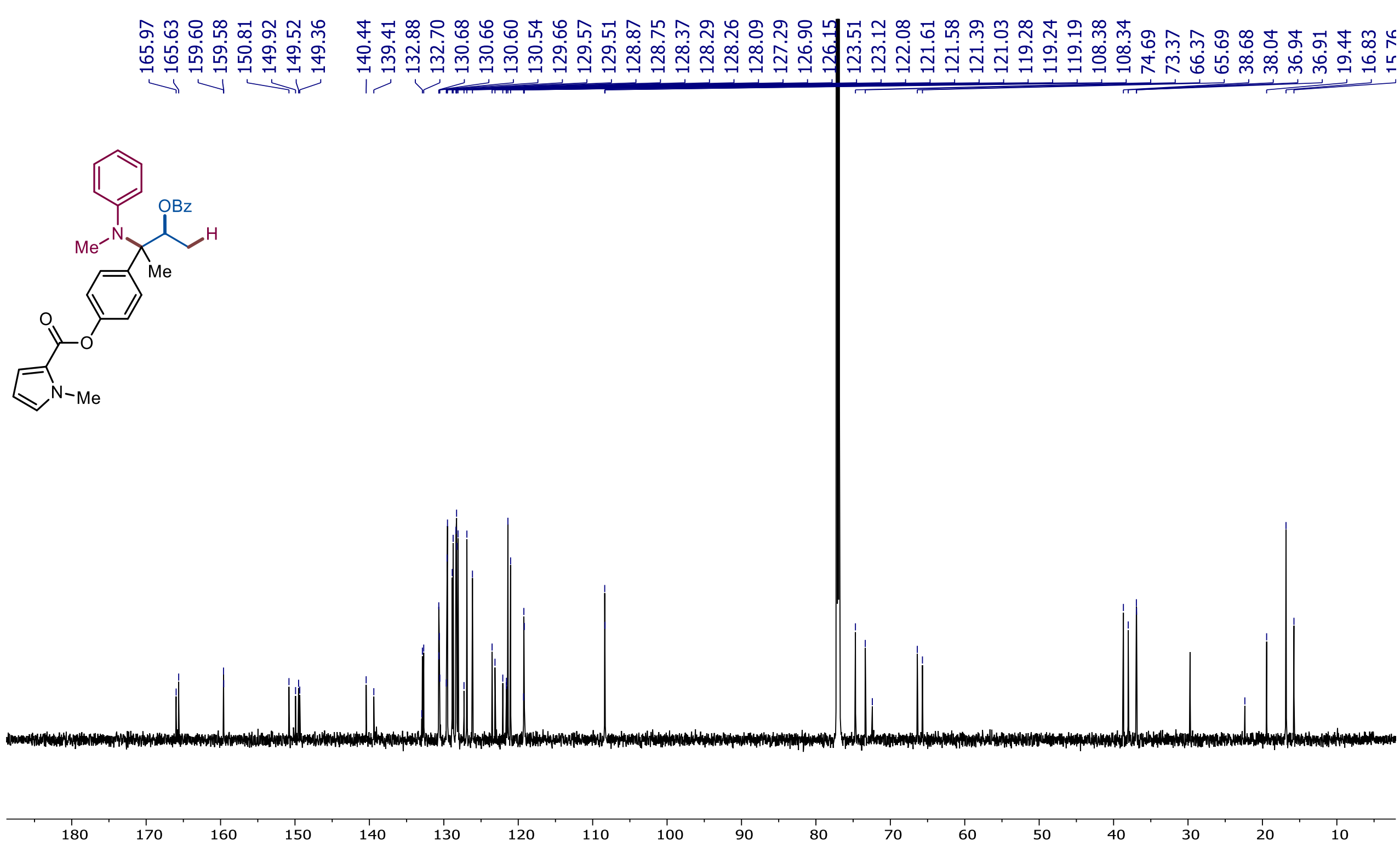

^{1}H NMR (500 MHz, $CDCl_3$, 25 ºC) of 3ax

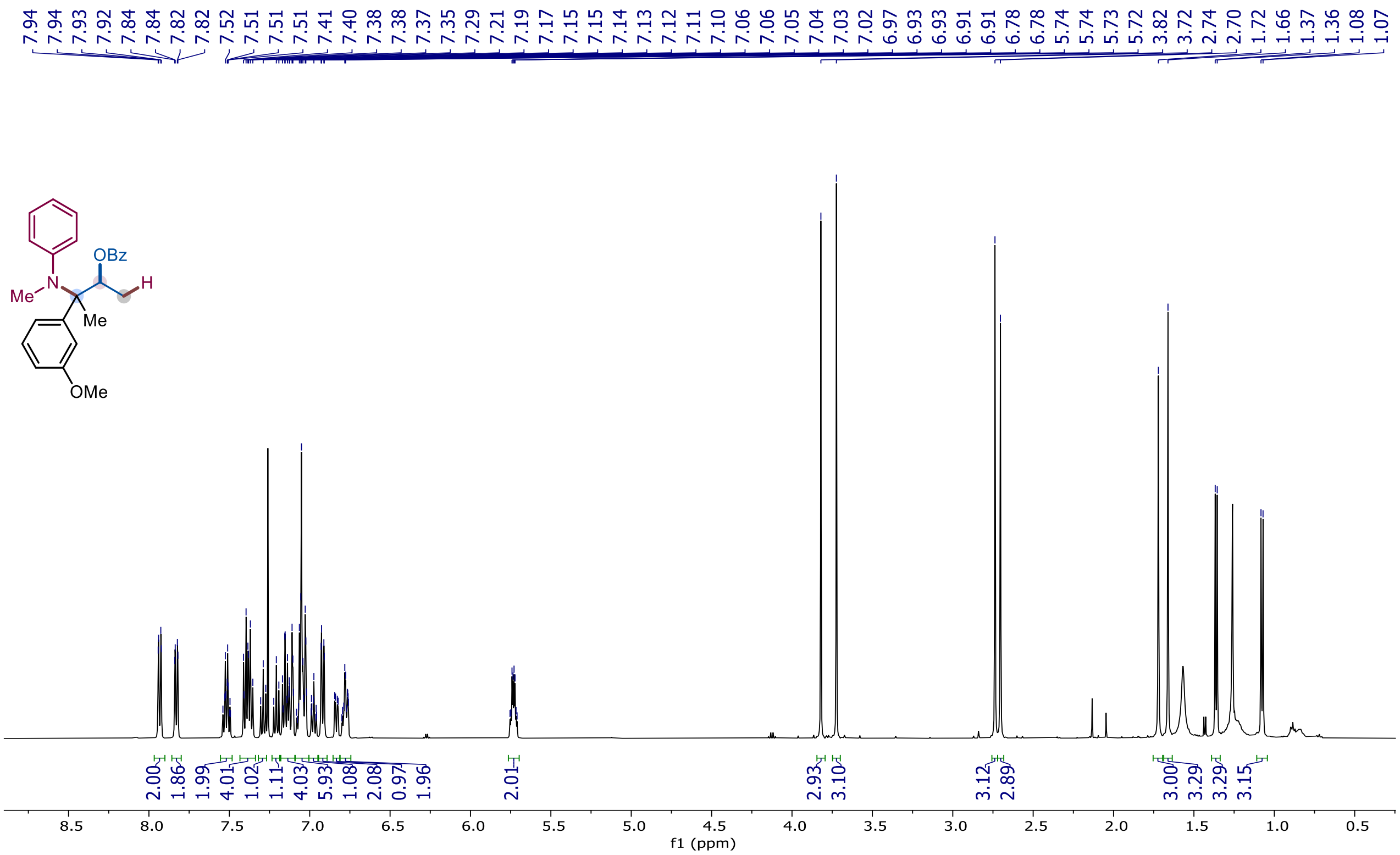

^{13}C NMR (126 MHz, $CDCl_3$, 25 ºC) of 3ax

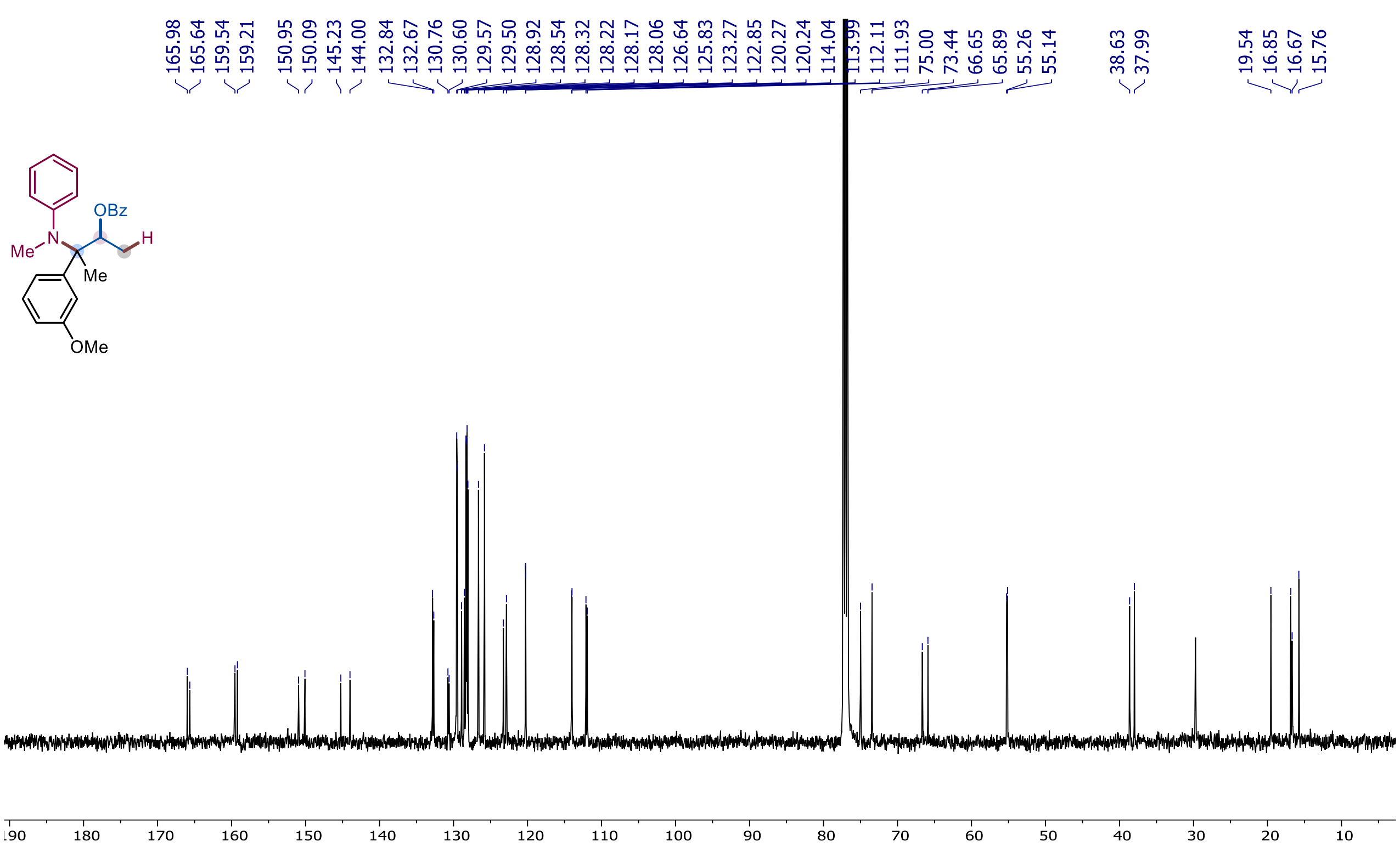

^{1}H NMR (800 MHz, $CDCl_3$, 25 ºC) of 3ay

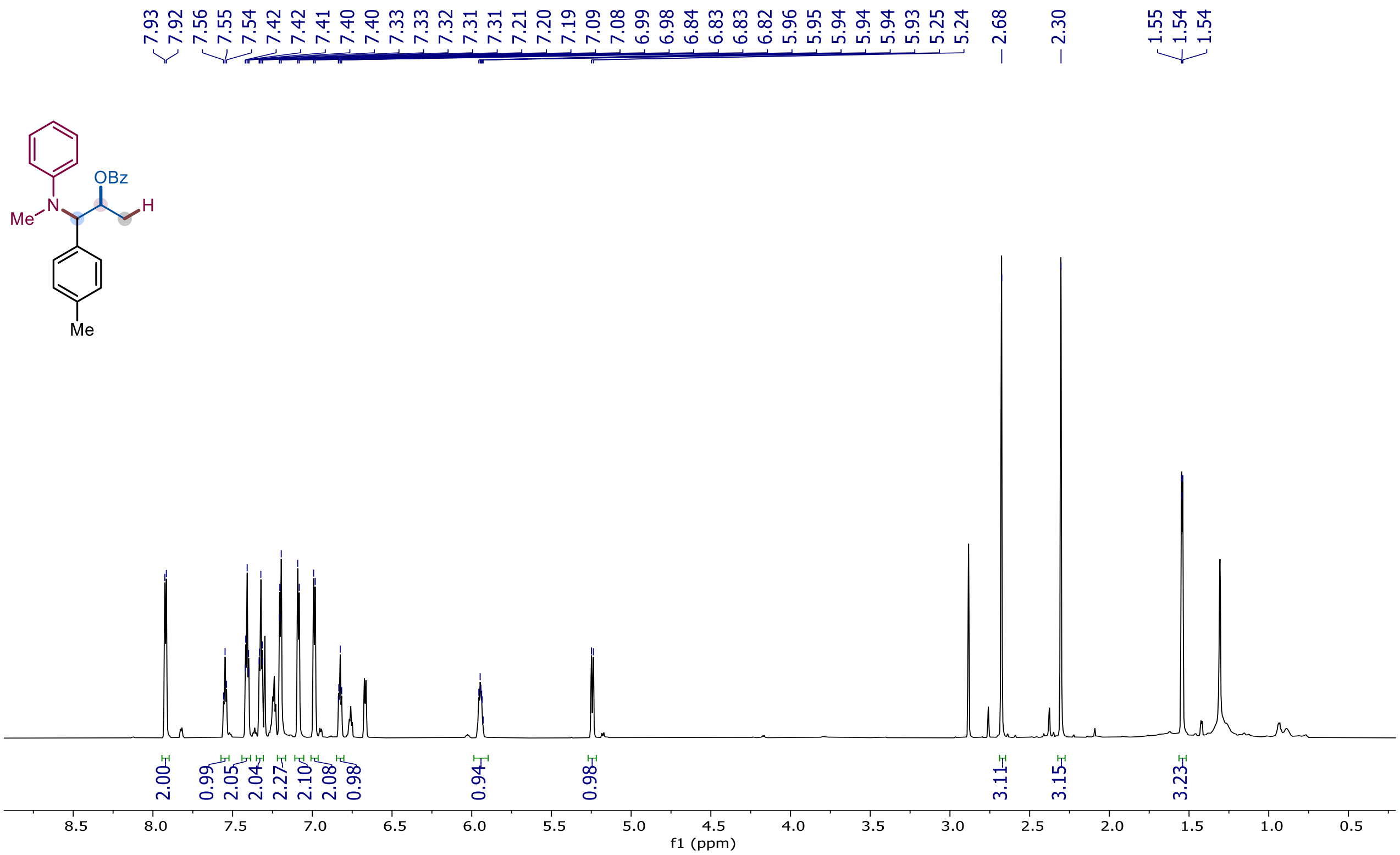

^{13}C NMR (201 MHz, $CDCl_3$, 25 ºC) of 3ay

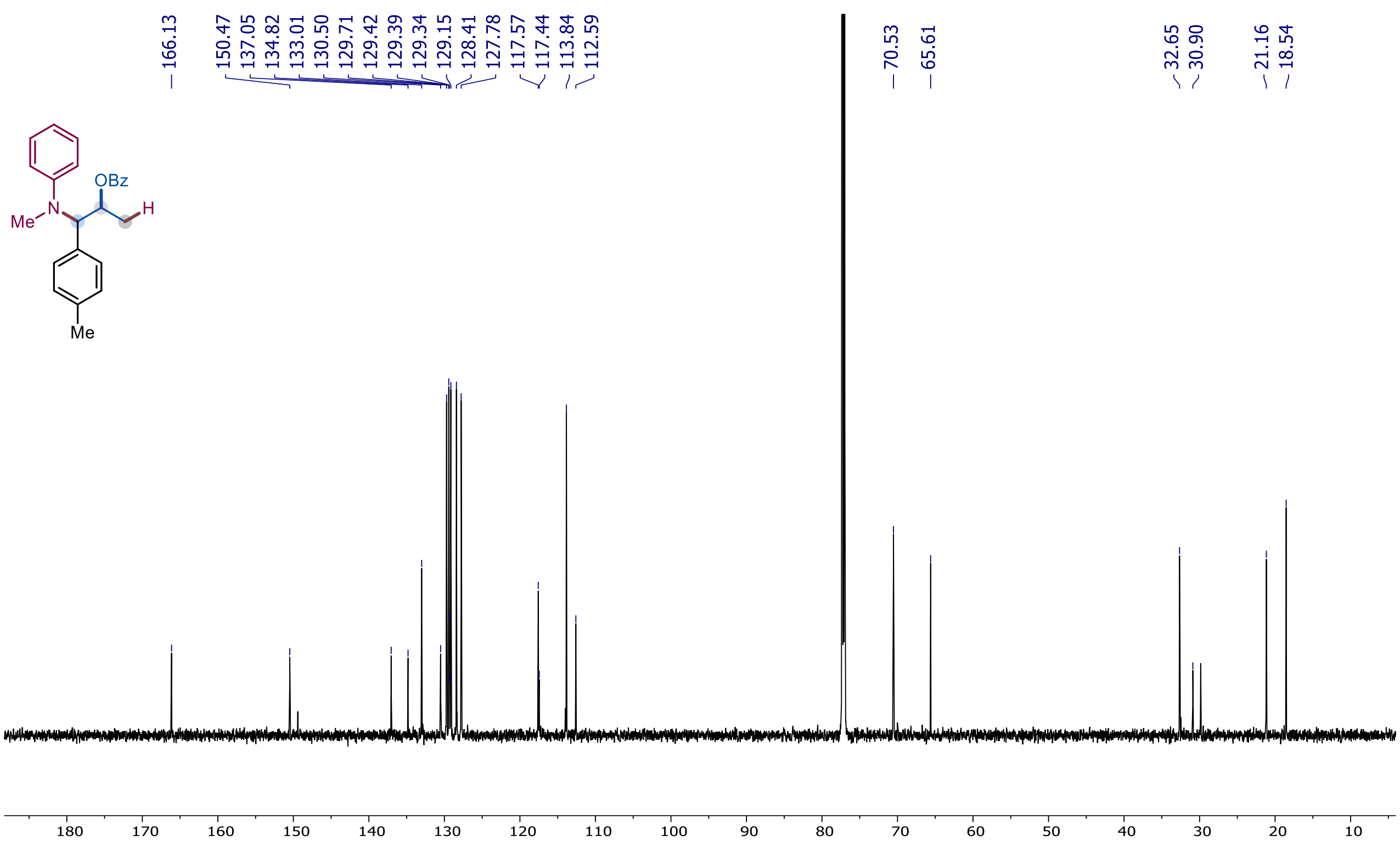

^{1}H NMR (400 MHz, CDCl$_3$, 25 ºC) of 3az

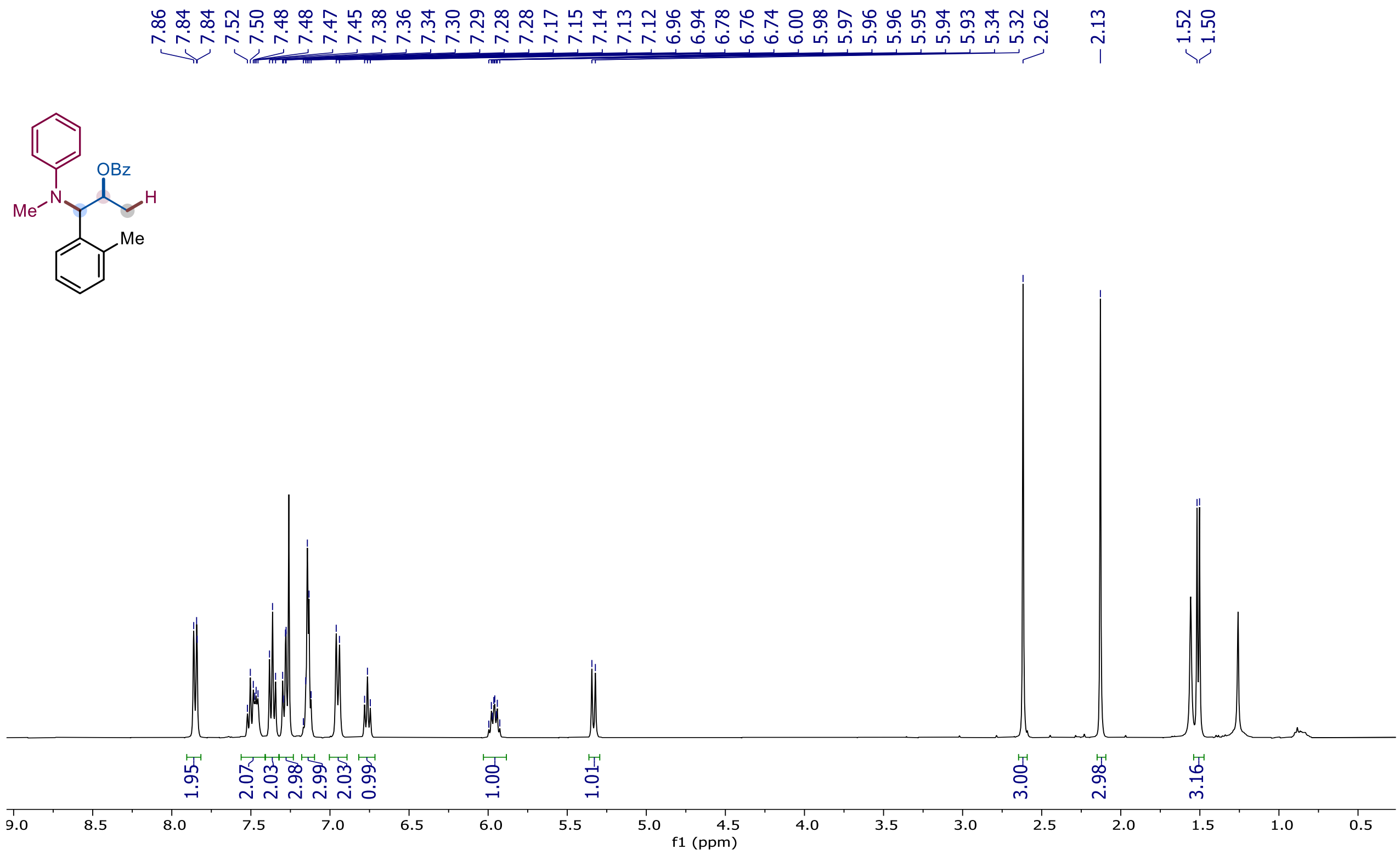

^{13}C NMR (126 MHz, CDCl$_3$, 25 ºC) of 3az

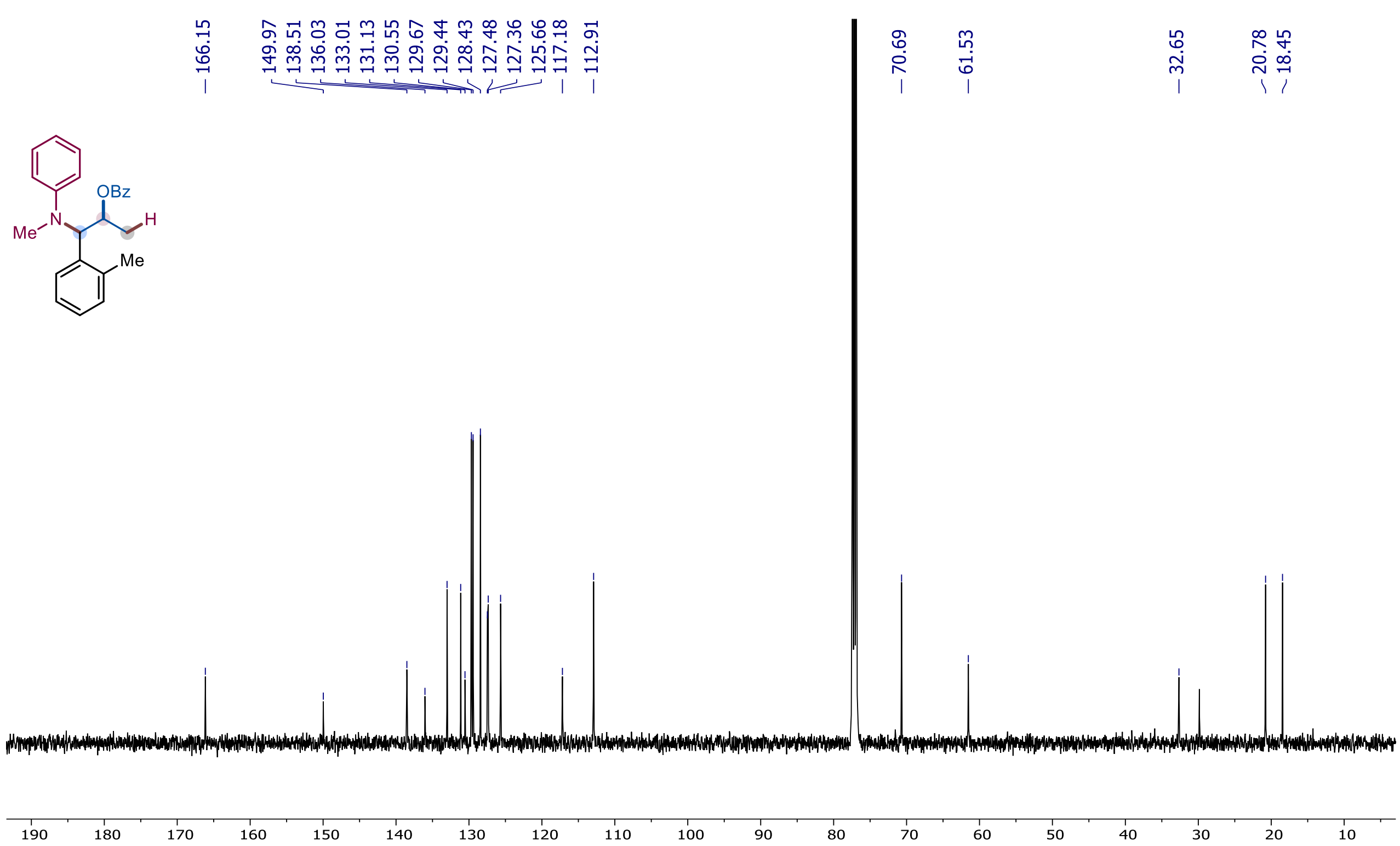

^{1}H NMR (800 MHz, $CDCl_3$, 25 ºC) of 3aaa

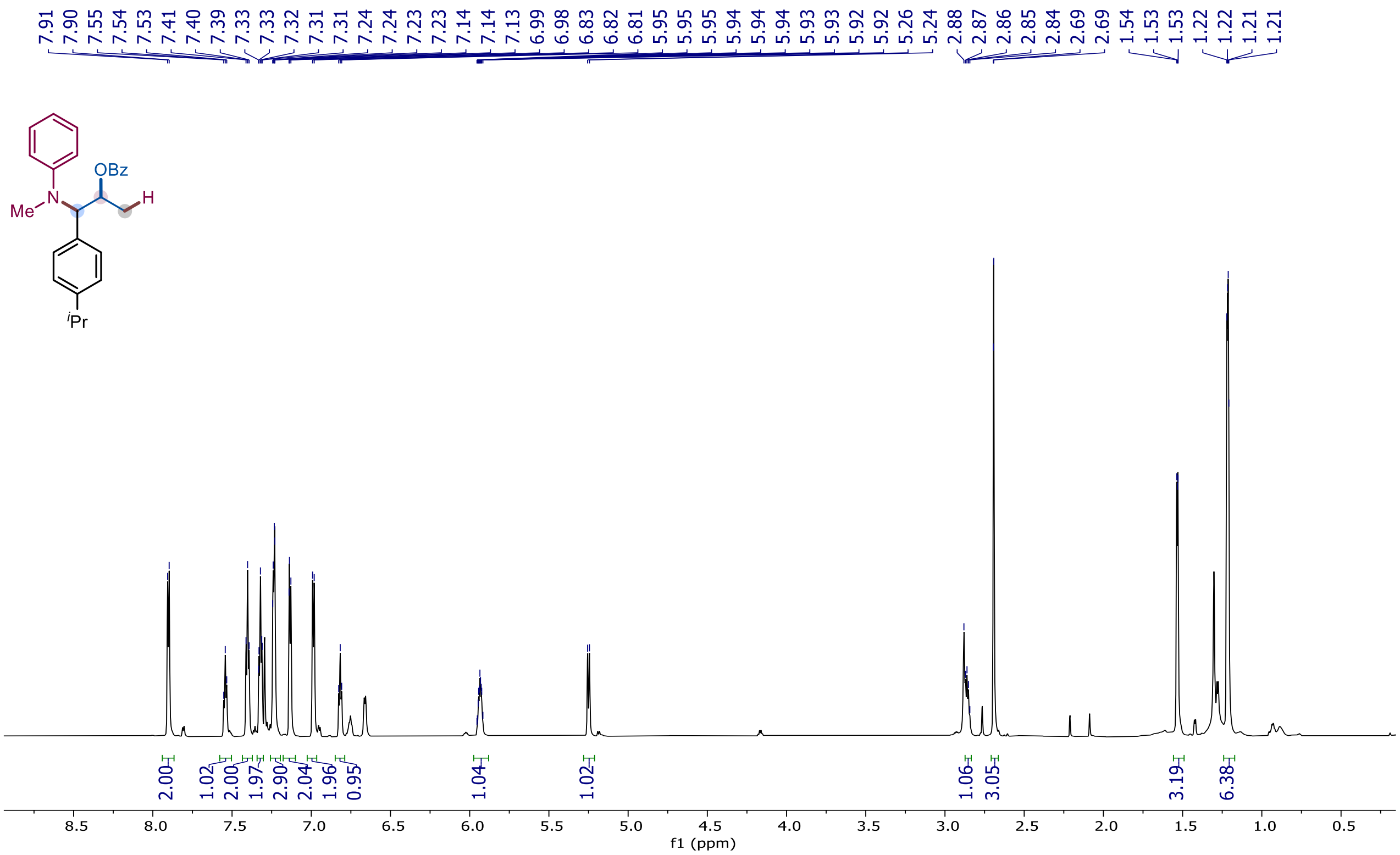

^{13}C NMR (201 MHz, $CDCl_3$, 25 ºC) of 3aaa

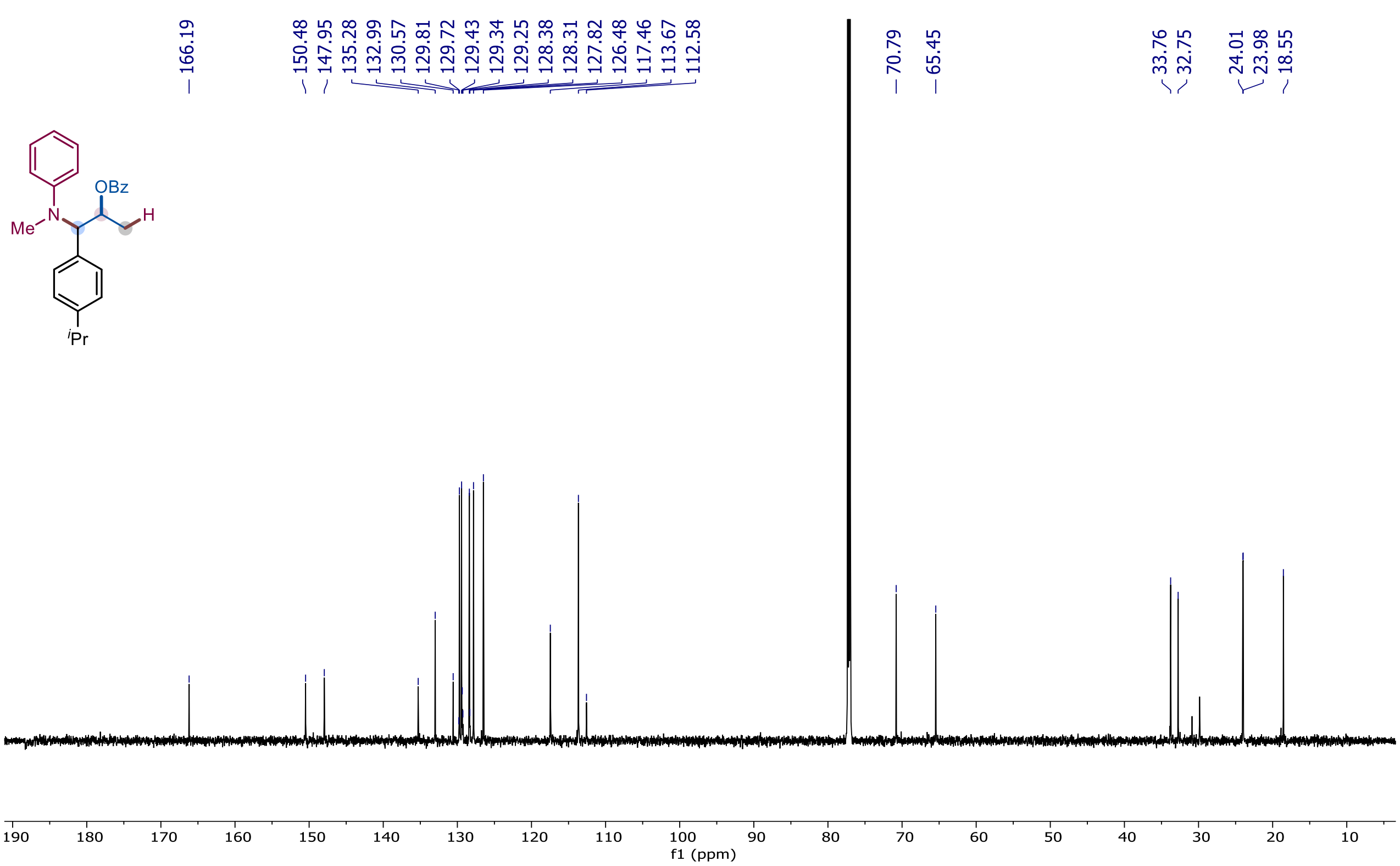

^{1}H NMR (500 MHz, $CDCl_3$, 25 ºC) of 3aab

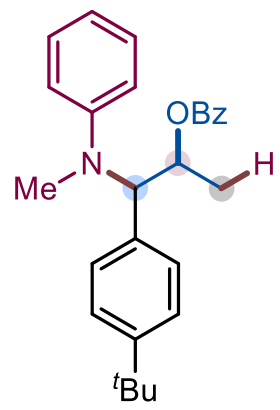

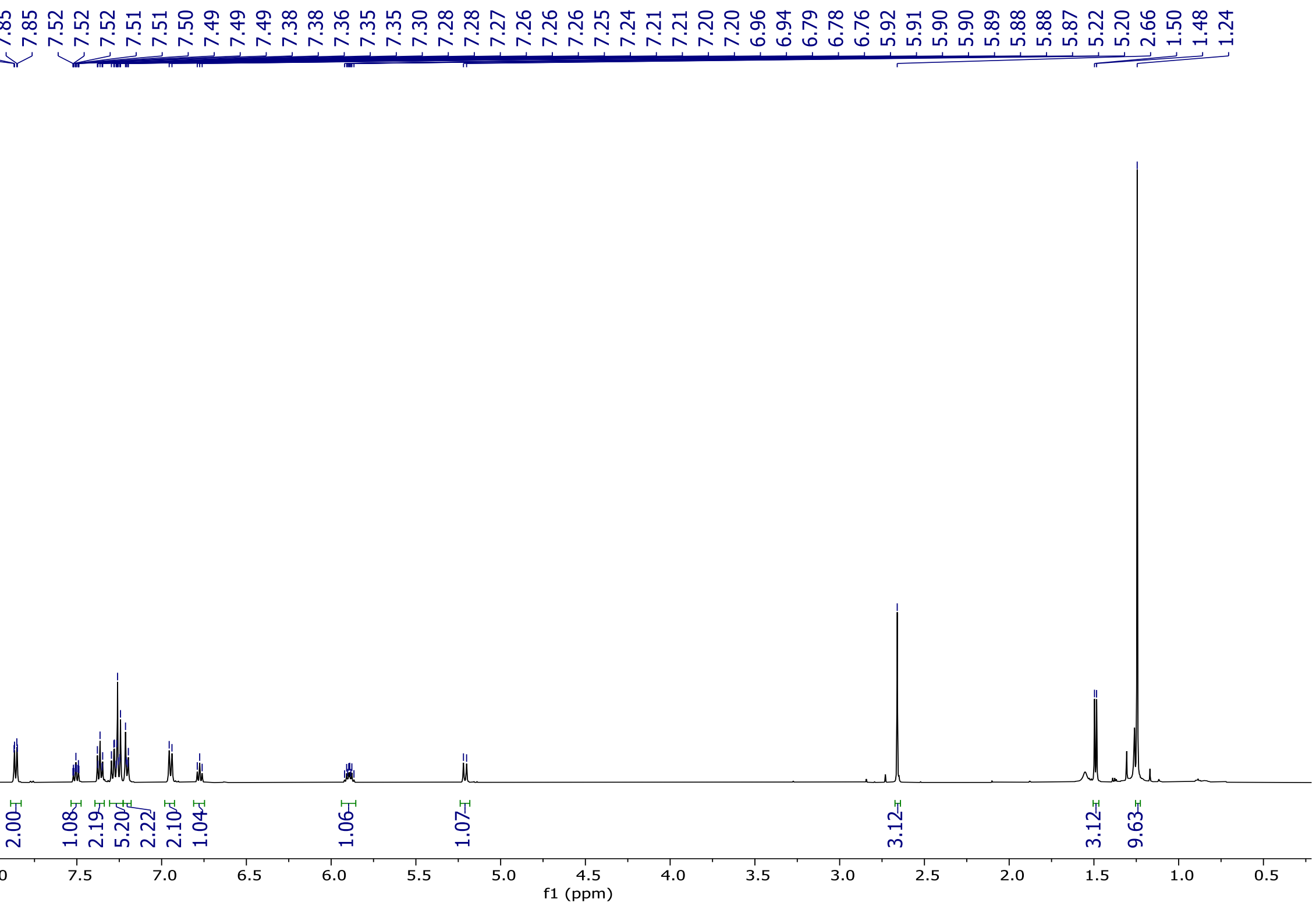

^{13}C NMR (126 MHz, $CDCl_3$, 25 ºC) of 3aab

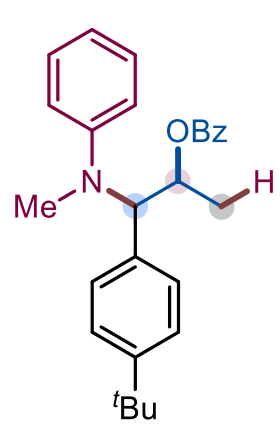

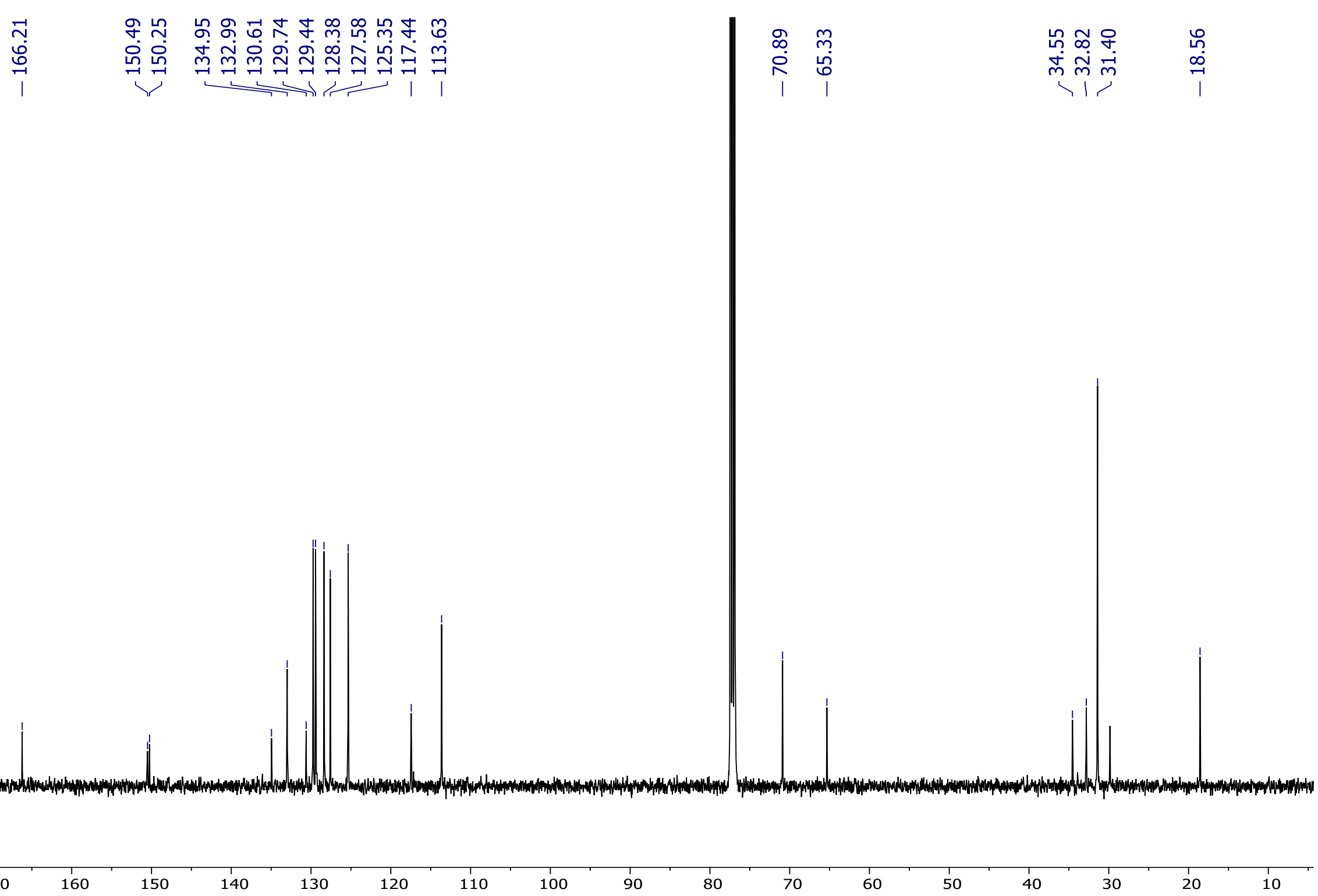

^{1}H NMR (500 MHz, $CDCl_3$, 25 °C) of 3aac

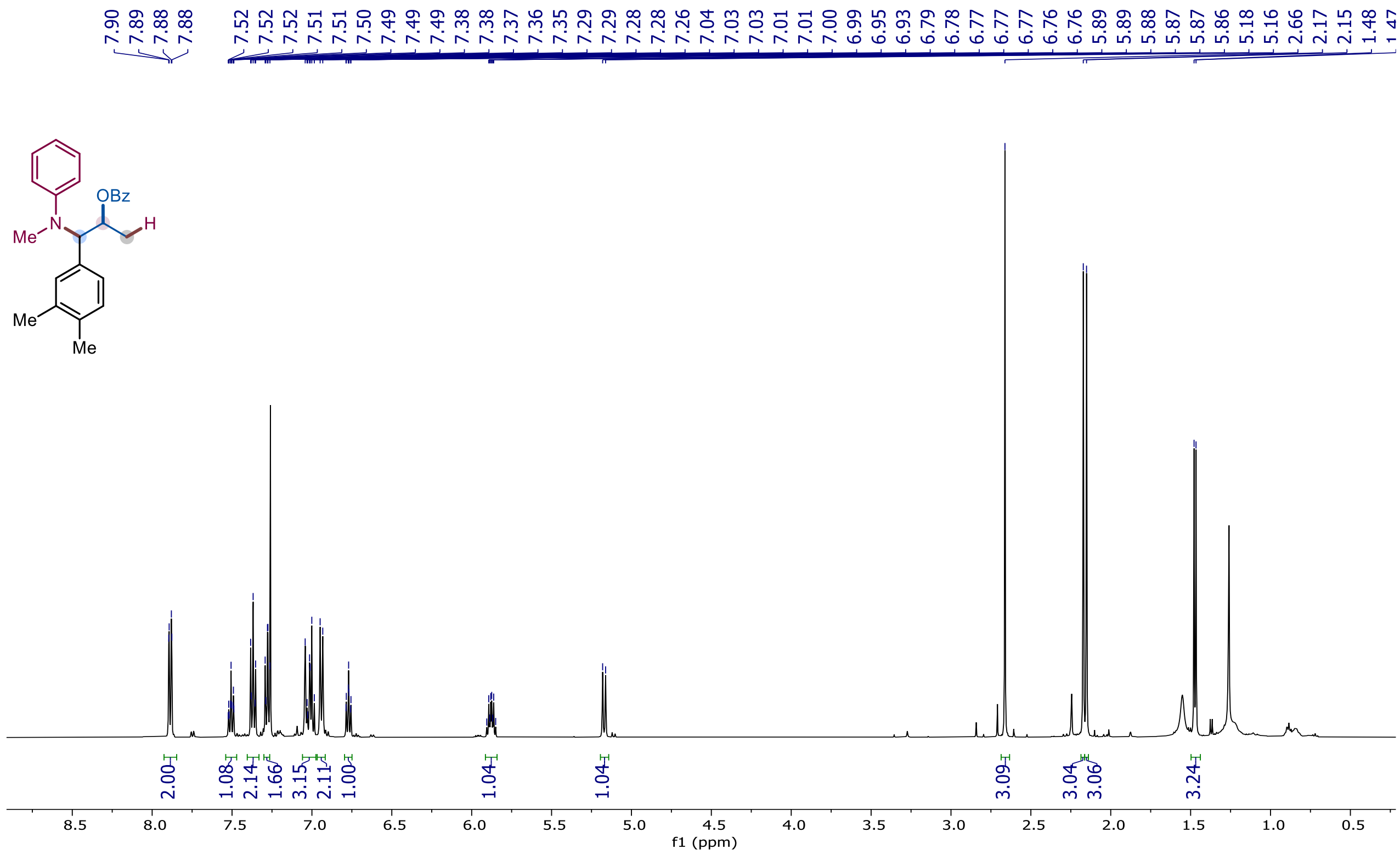

^{13}C NMR (126 MHz, $CDCl_3$, 25 °C) of 3aac

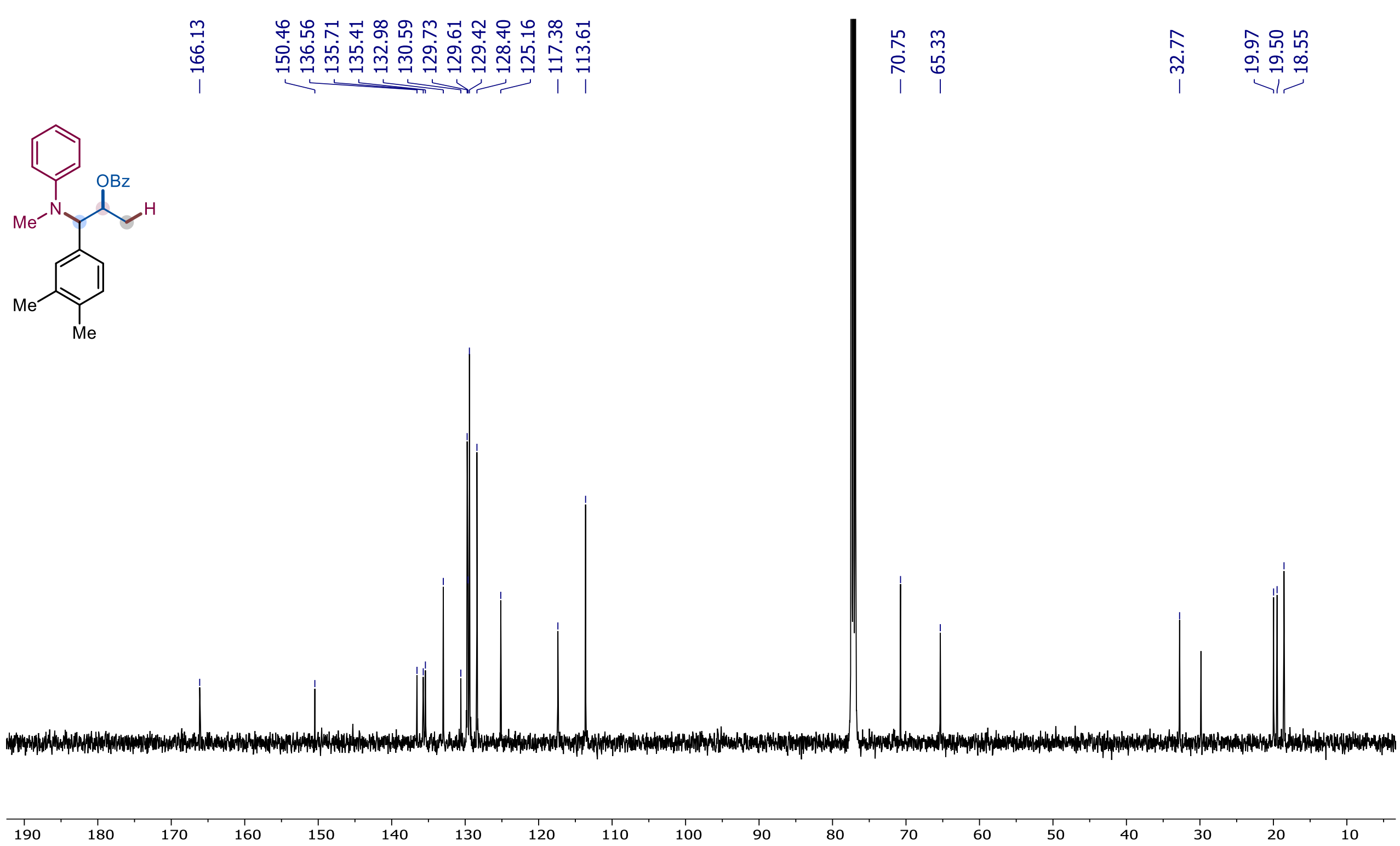

^{1}H NMR (500 MHz, $CDCl_3$, 25 ºC) of 3aad

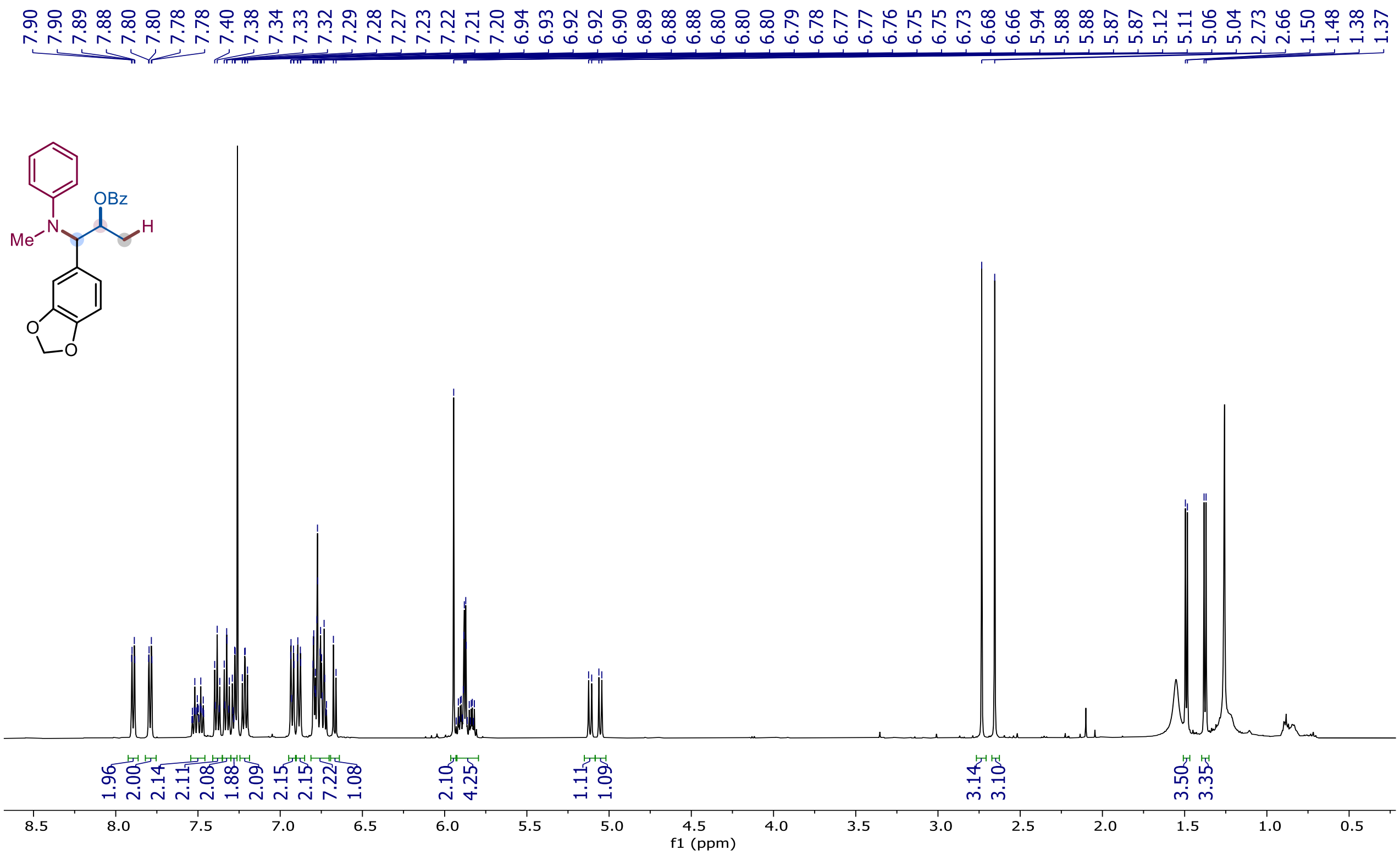

^{13}C NMR (126 MHz, $CDCl_3$, 25 ºC) of 3aad

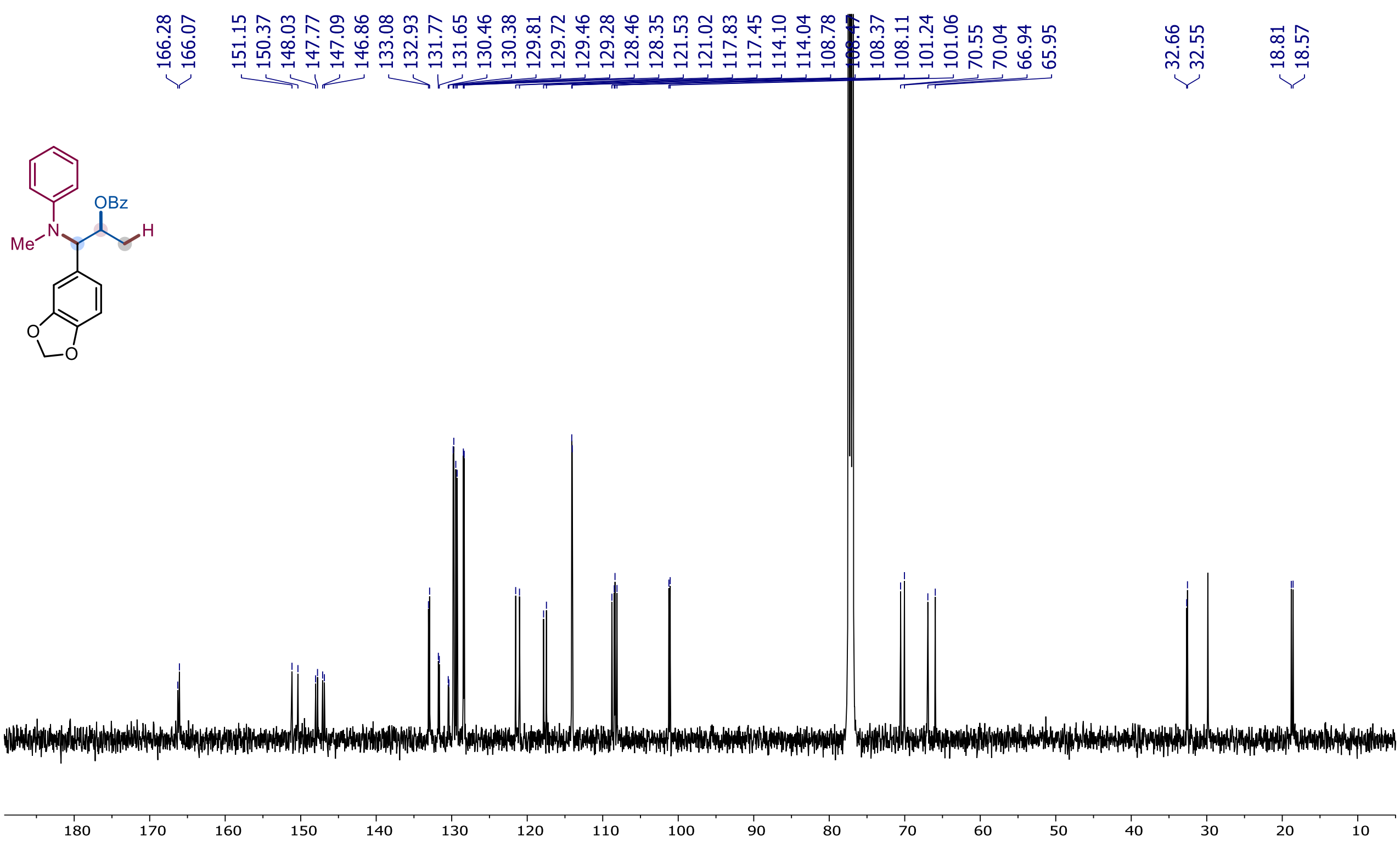

^{1}H NMR (800 MHz, $CDCl_3$, 25 °C) of 3aae

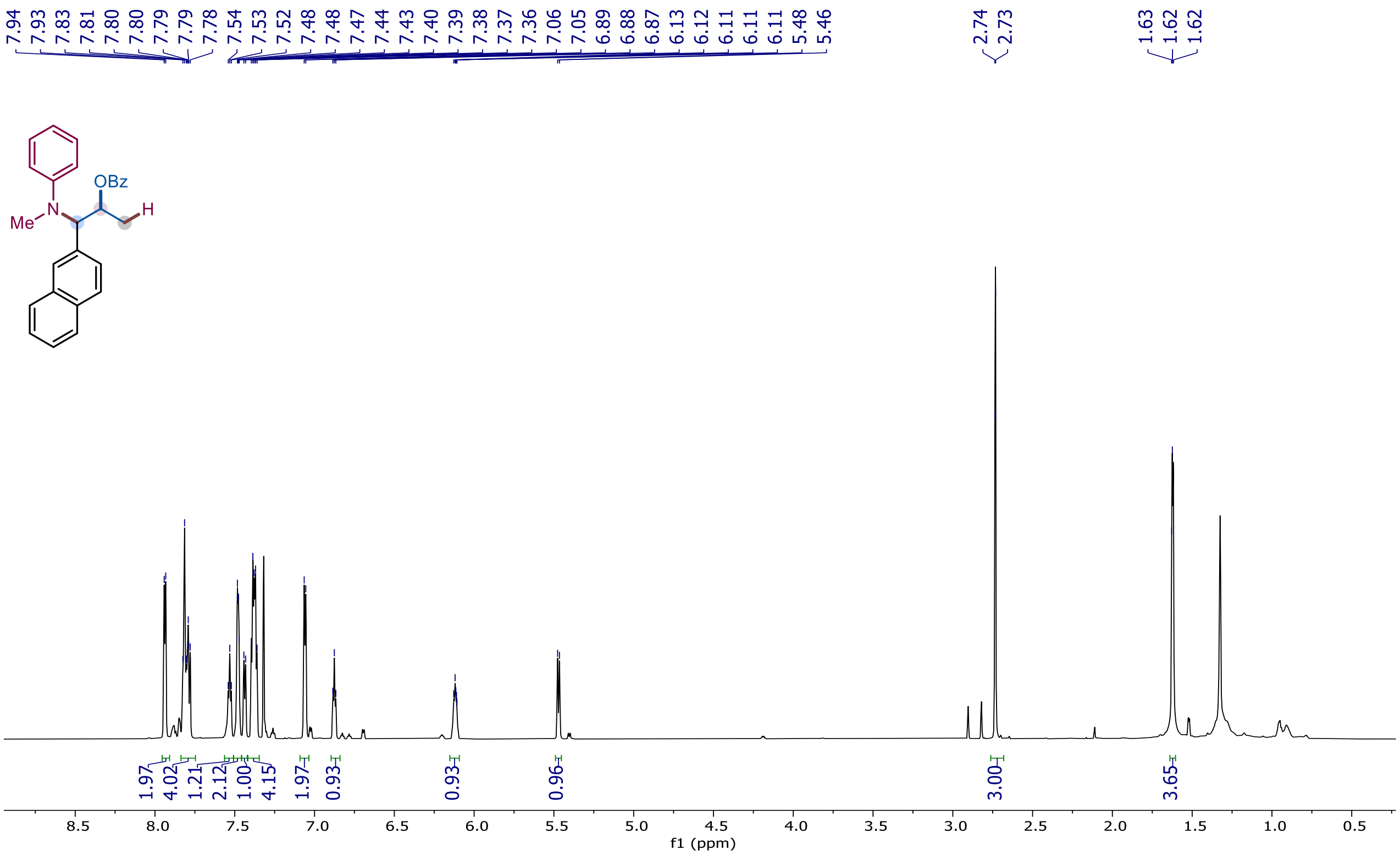

^{13}C NMR (201 MHz, $CDCl_3$, 25 °C) of 3aae

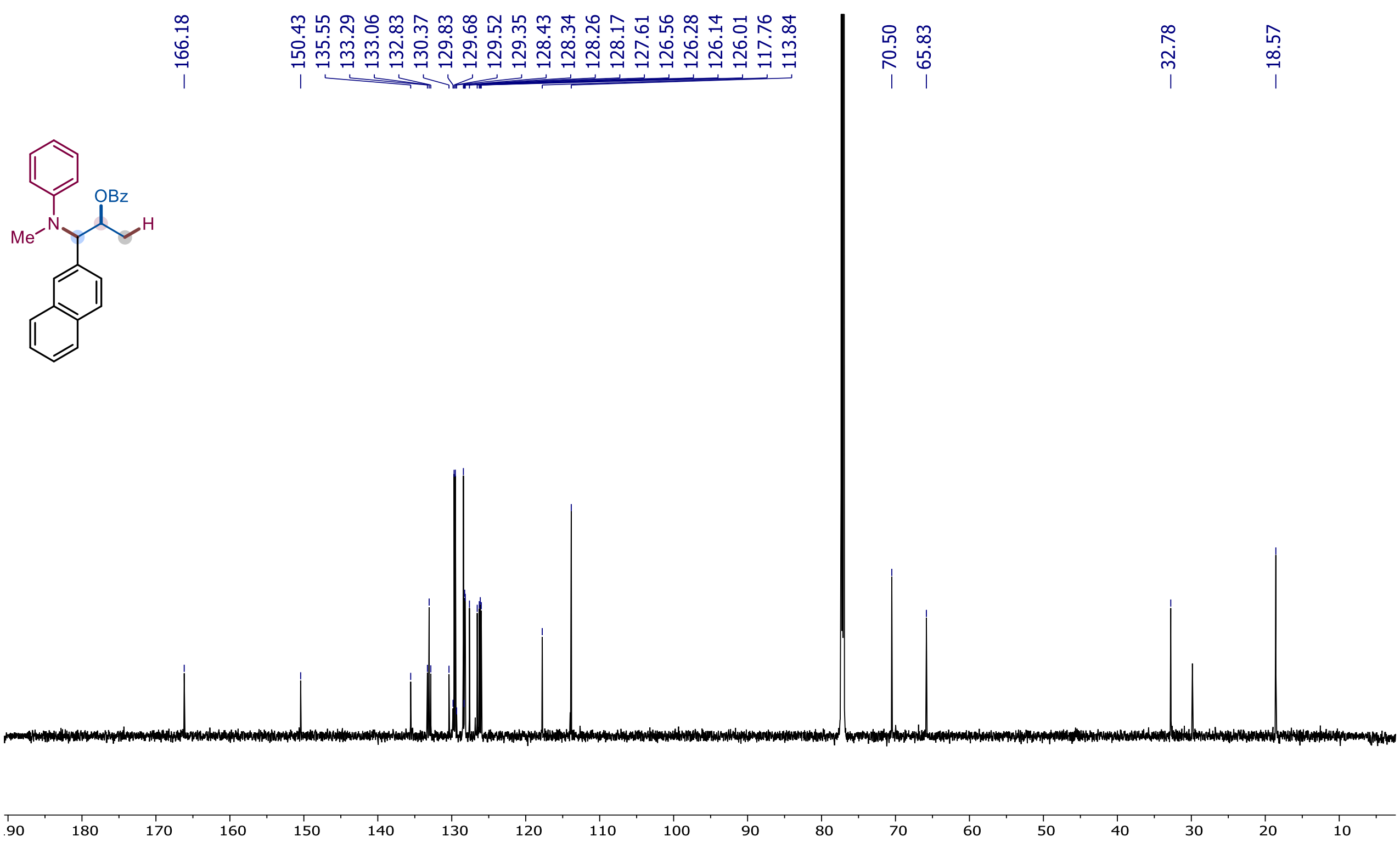

^{1}H NMR (500 MHz, $CDCl_3$, 25 ºC) of 3aaf

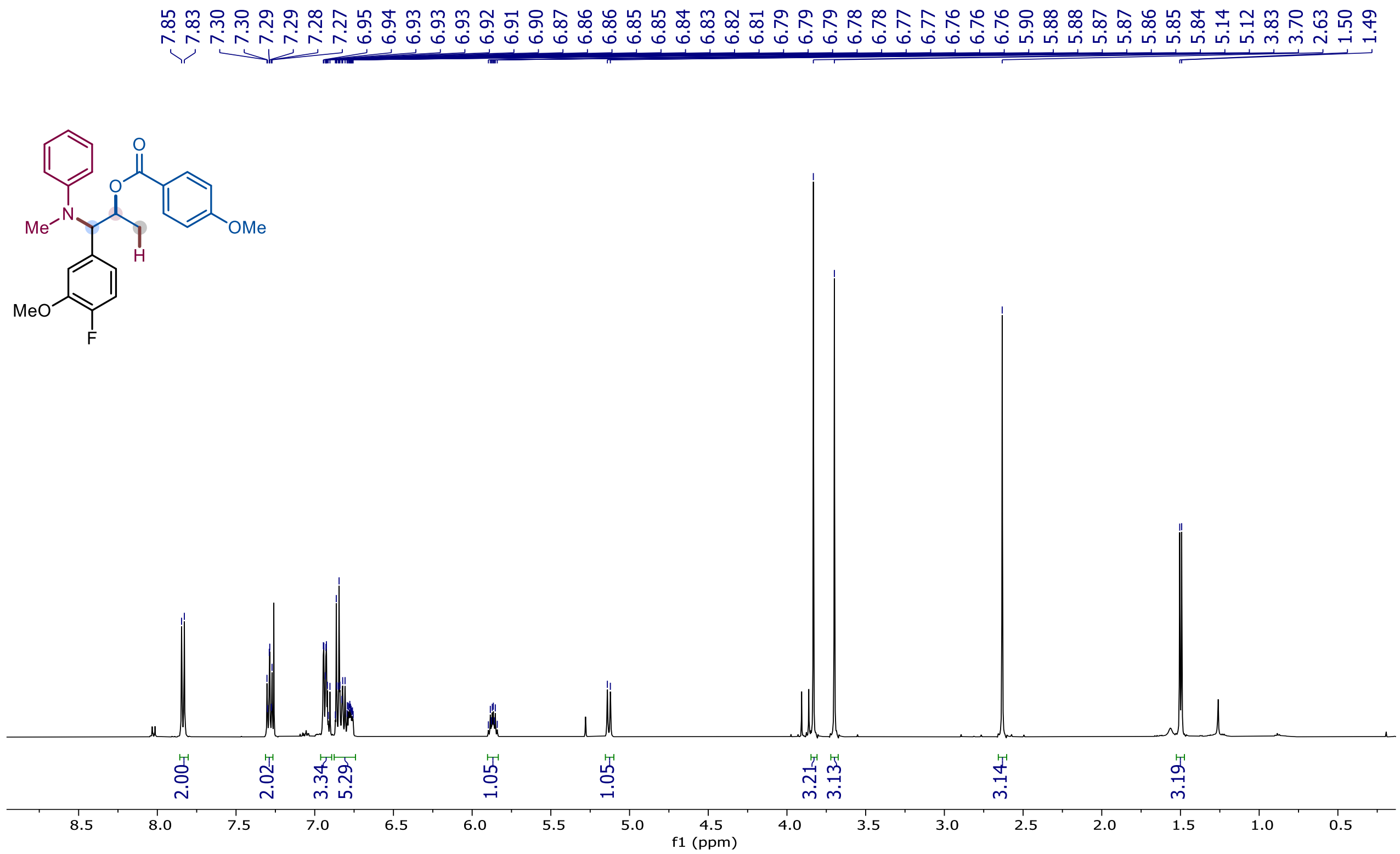

^{13}C NMR (126 MHz, $CDCl_3$, 25 ºC) of 3aaf

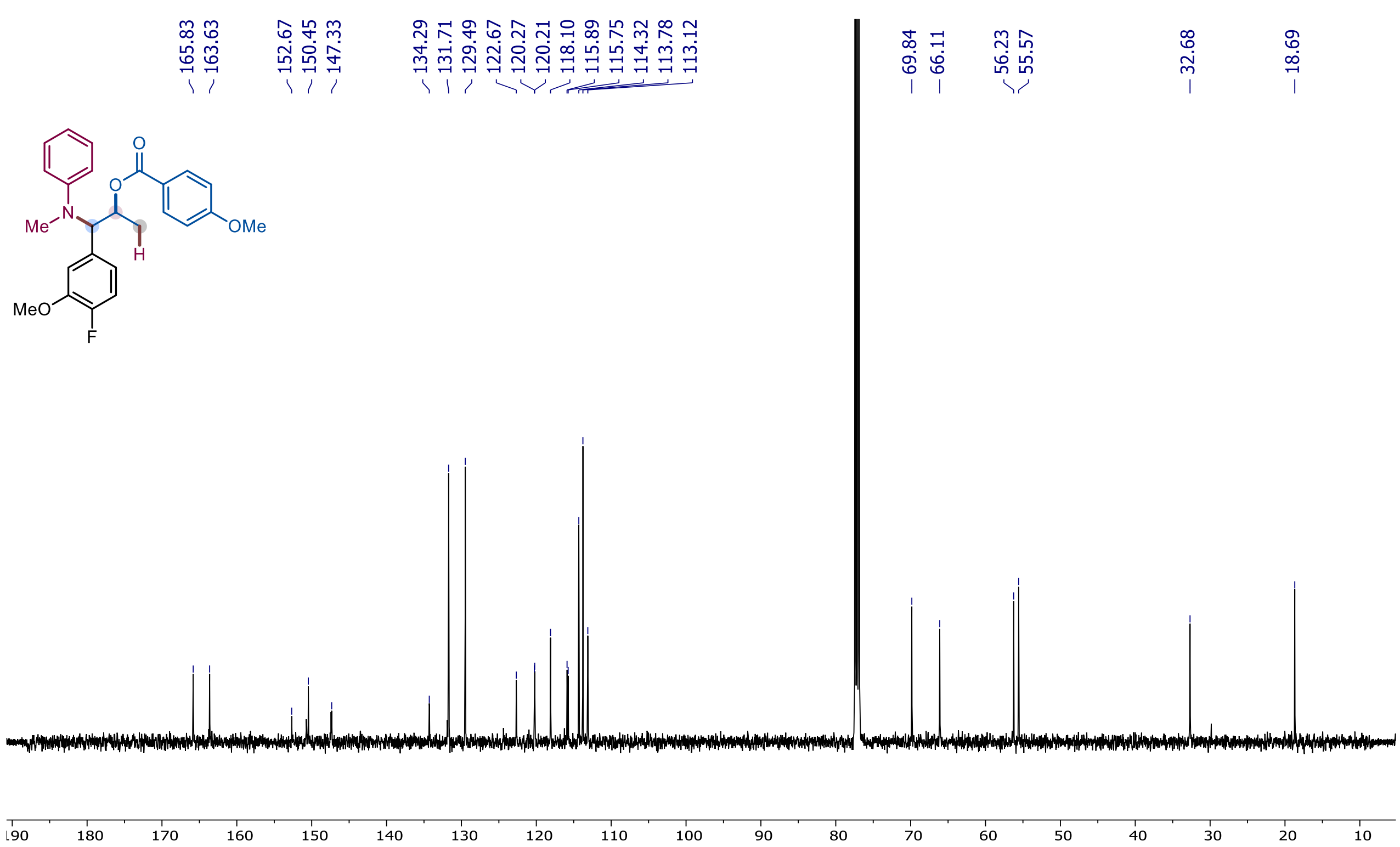

^{19}F NMR (470 MHz, CDCl$_3$, 25 °C) of 3aaf

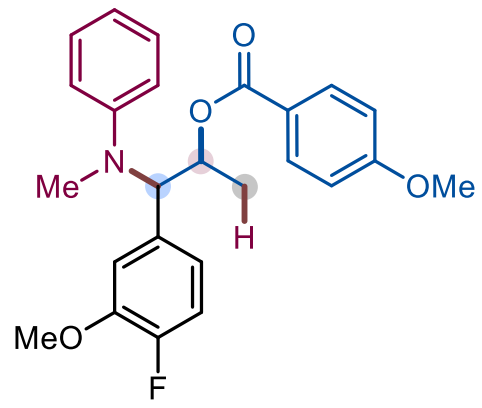

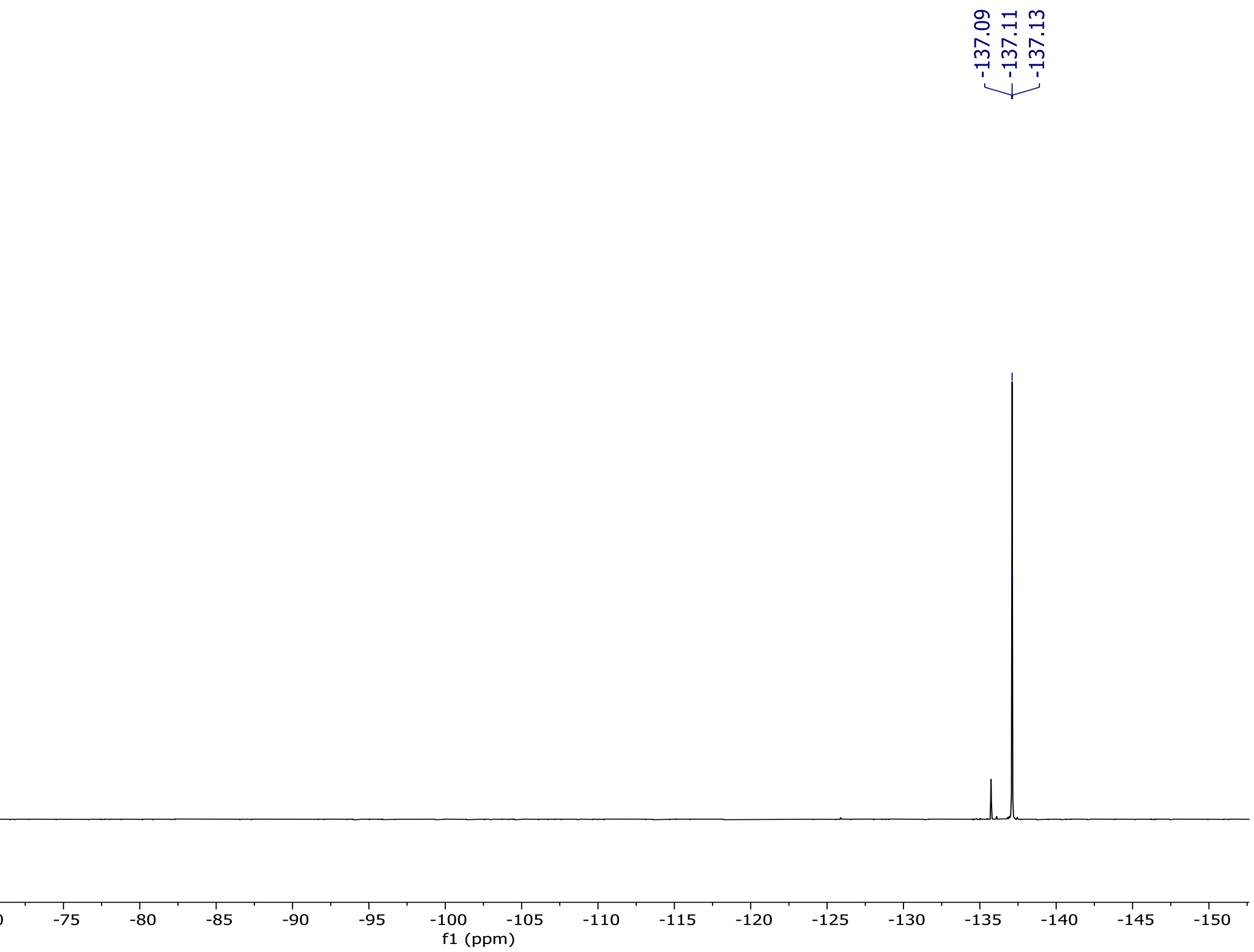

^{1}H NMR (500 MHz, CDCl$_3$, 25 ºC) of 3aag

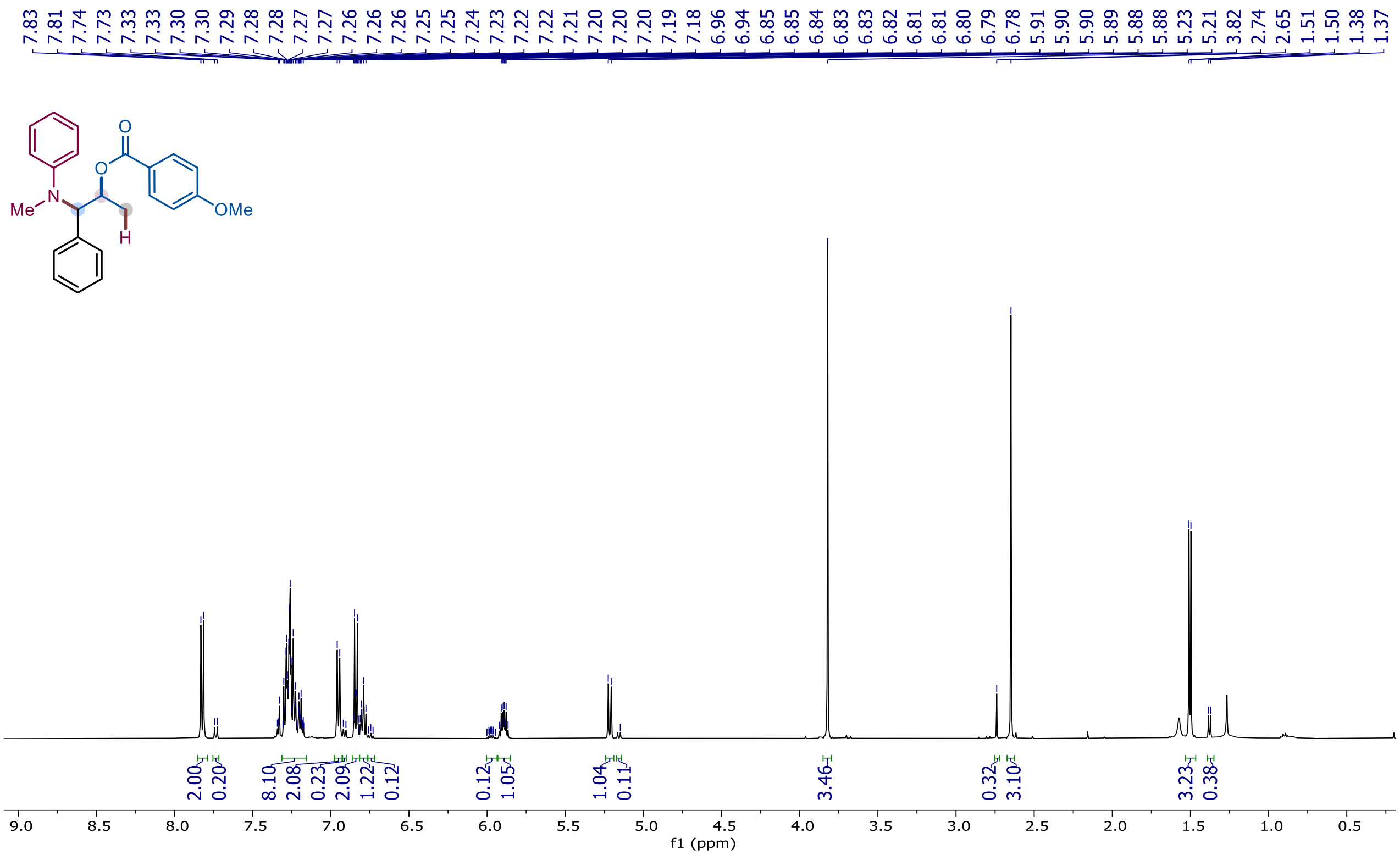

^{13}C NMR (126 MHz, CDCl$_3$, 25 ºC) of 3aag

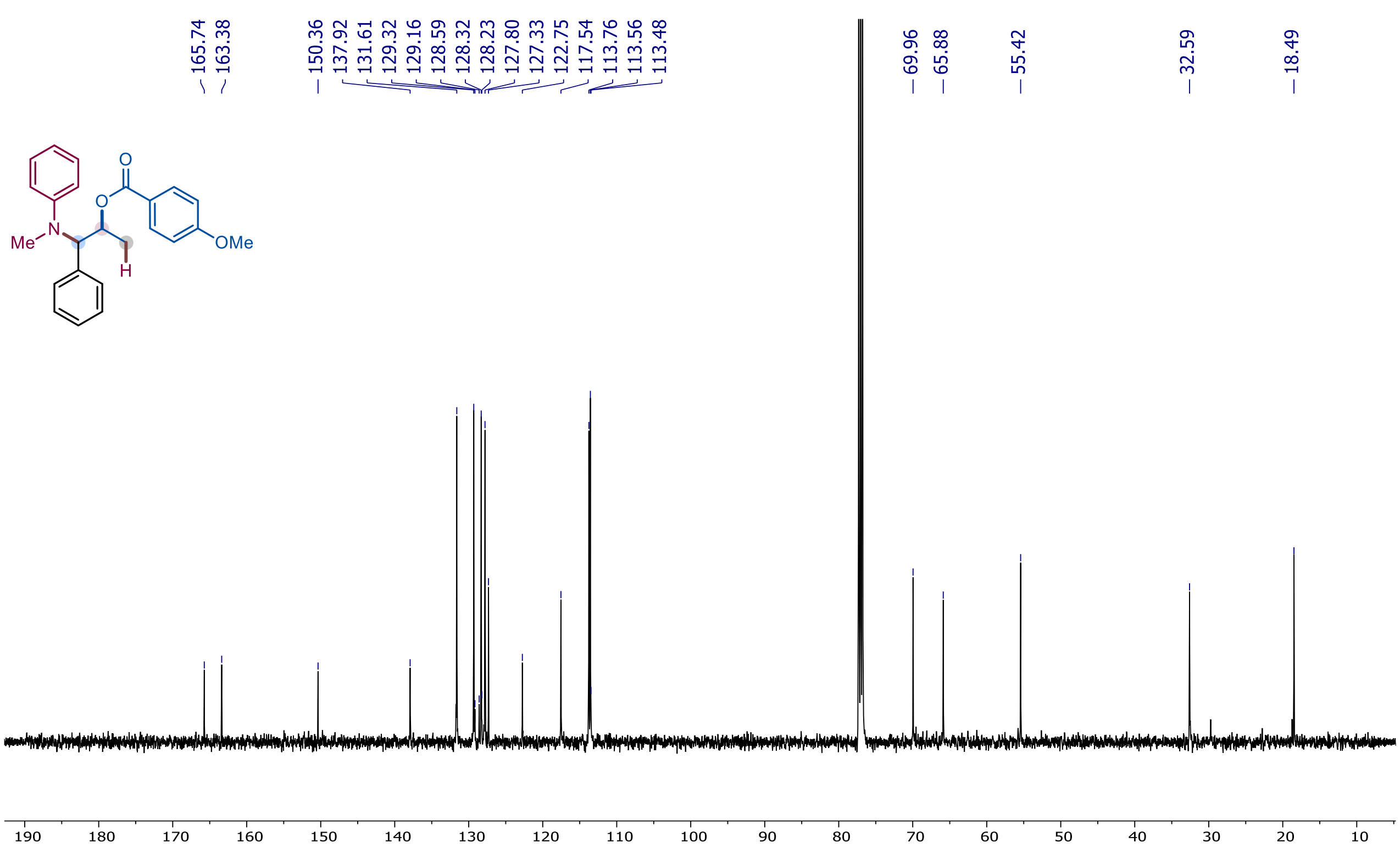

^{1}H NMR (300 MHz, $CDCl_3$, 25 ºC) of 3aah

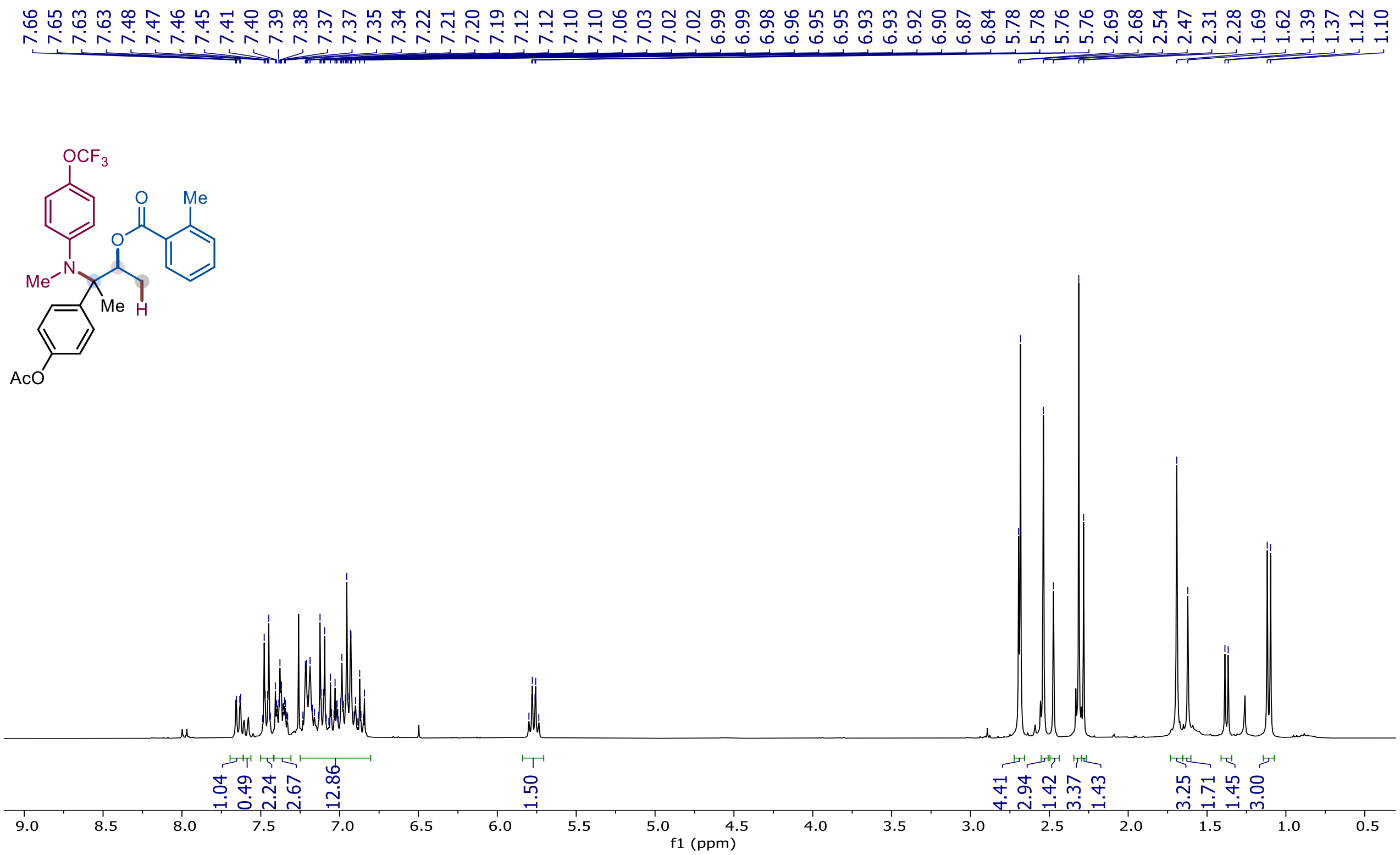

^{13}C NMR (201 MHz, $CDCl_3$, 25 ºC) of 3aah

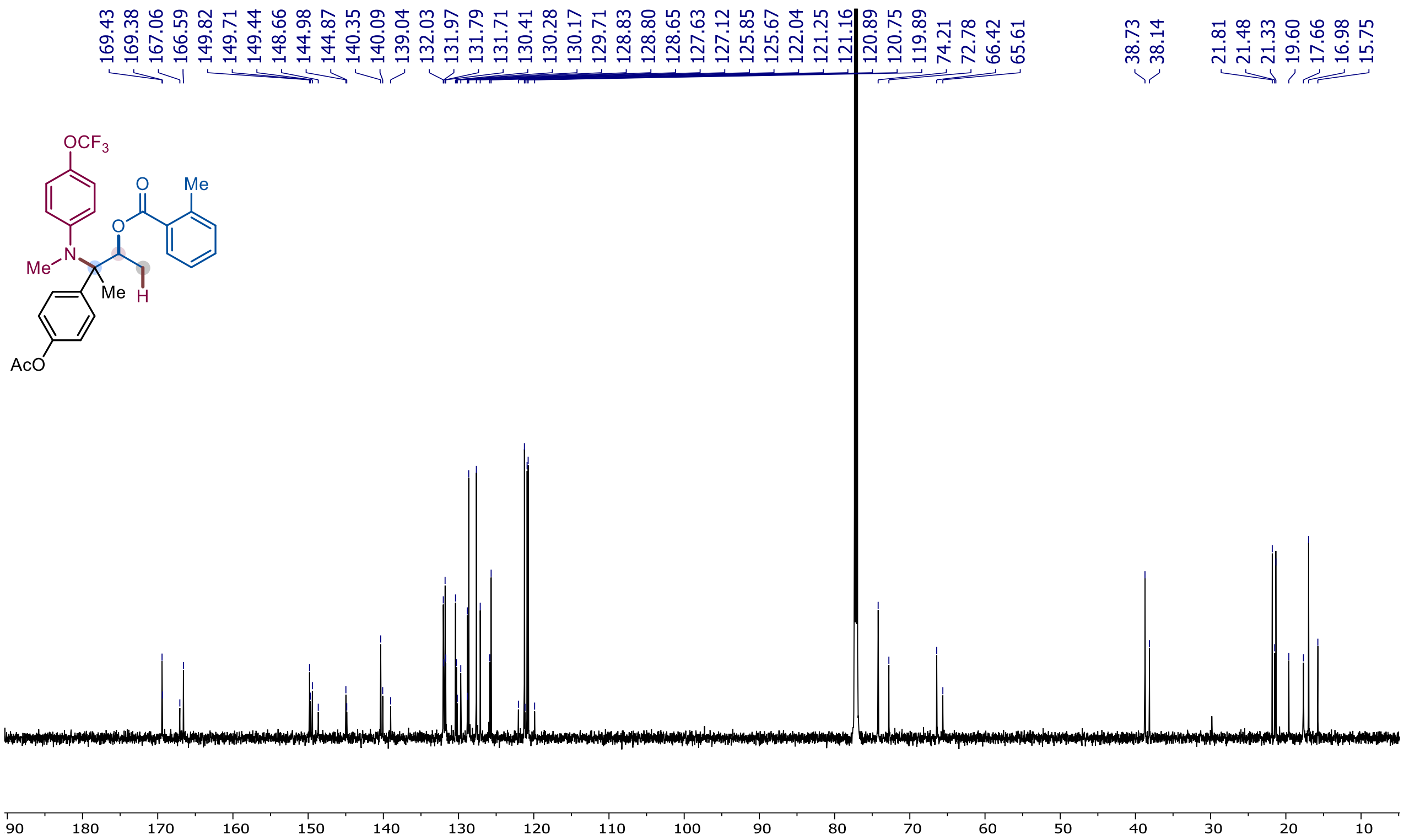

^{19}F NMR (282 MHz, $CDCl_3$, 25 °C) of 3aah

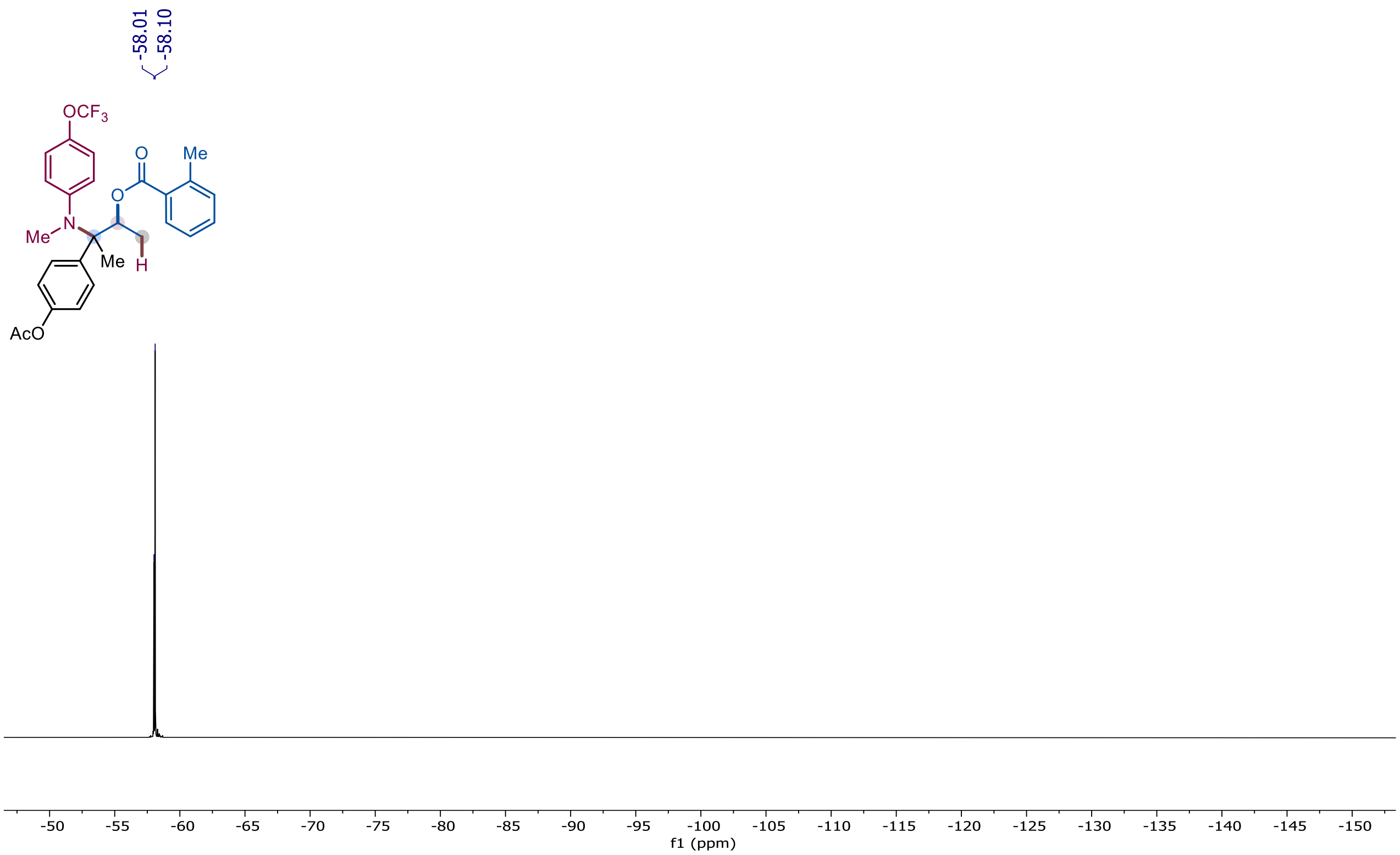

^{1}H NMR (400 MHz, $CDCl_3$, 25 ºC) of 3aai

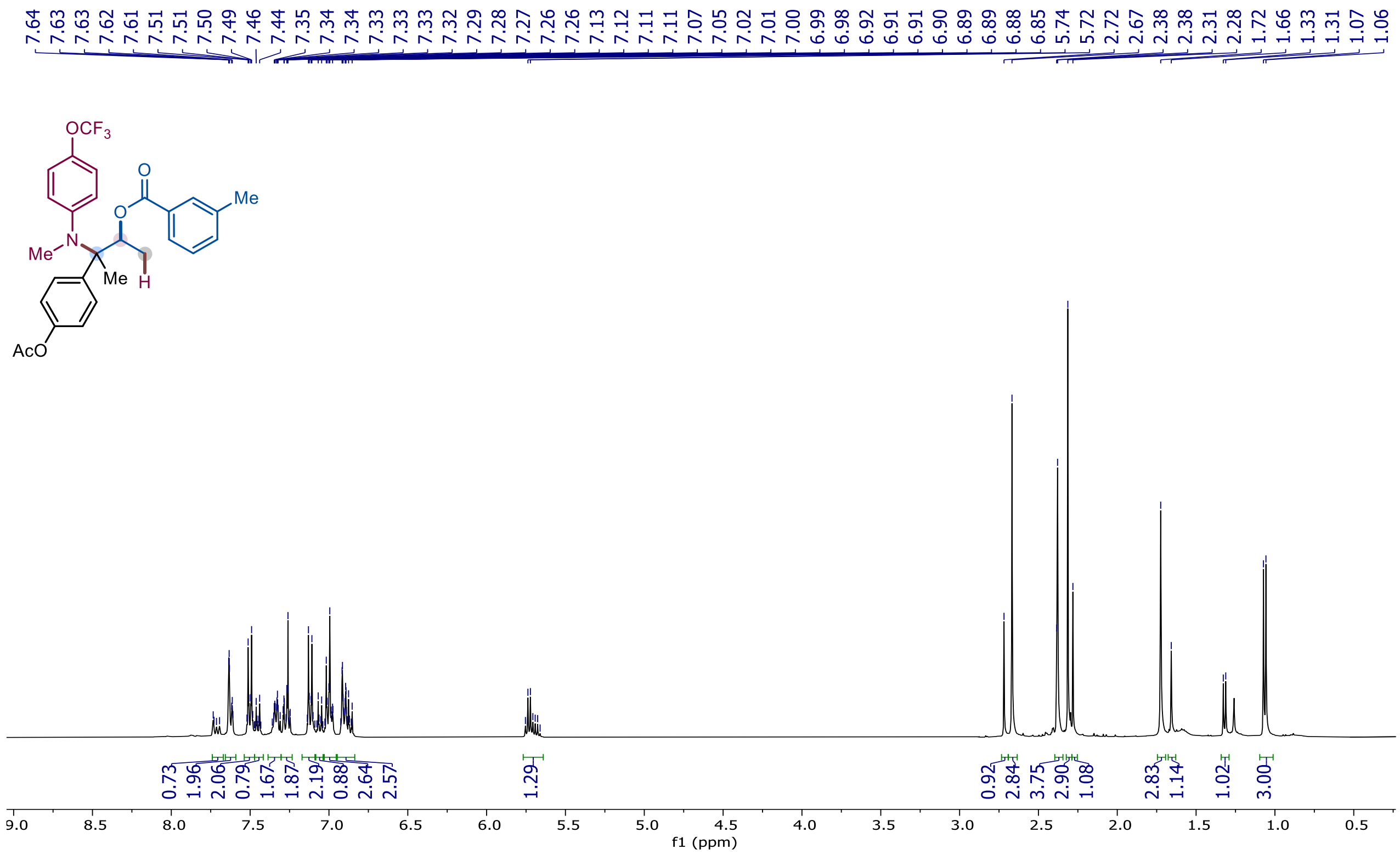

^{13}C NMR (201 MHz, $CDCl_3$, 25 ºC) of 3aai

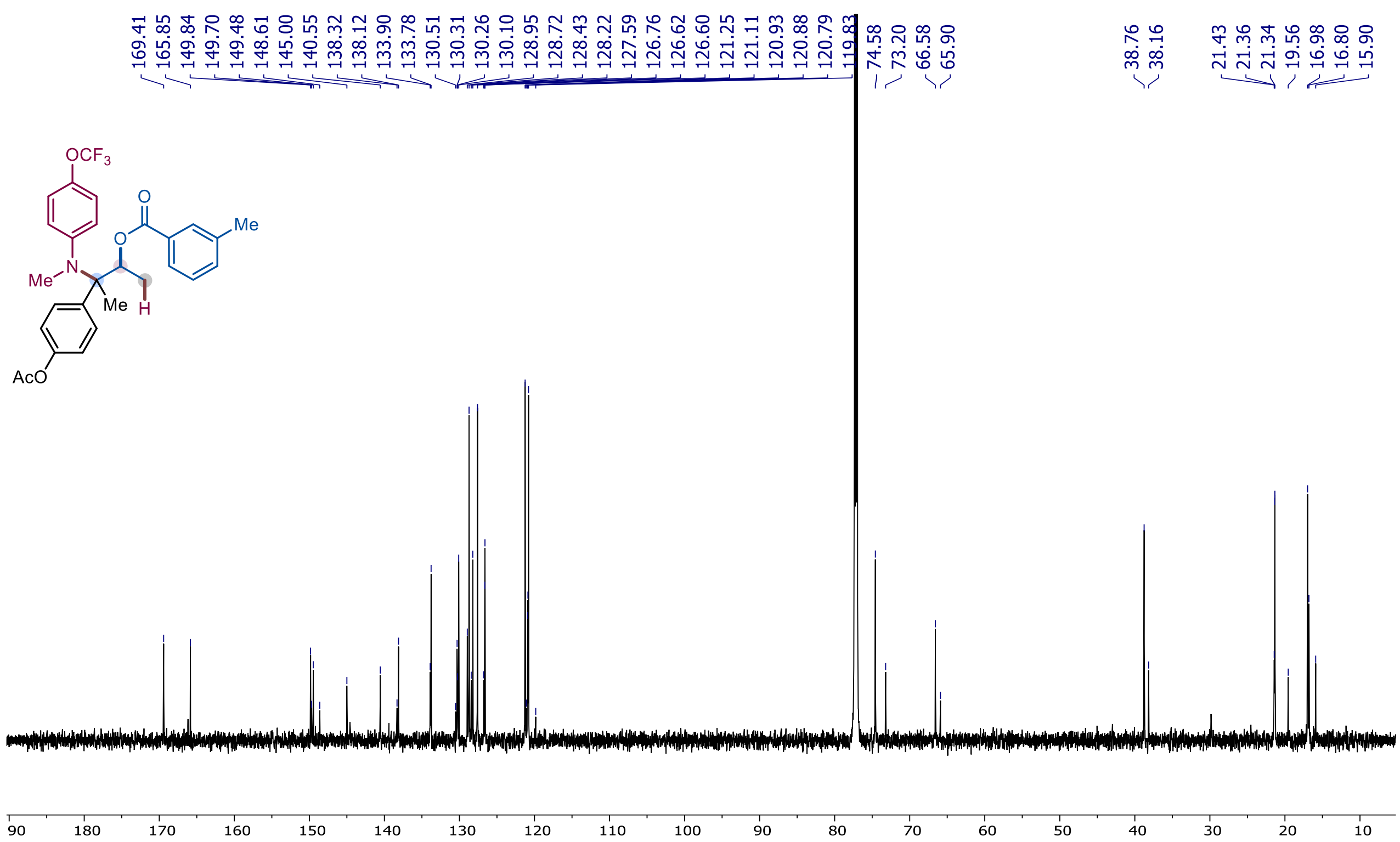

^{19}F NMR (376 MHz, $CDCl_3$, 25 °C) of 3aai

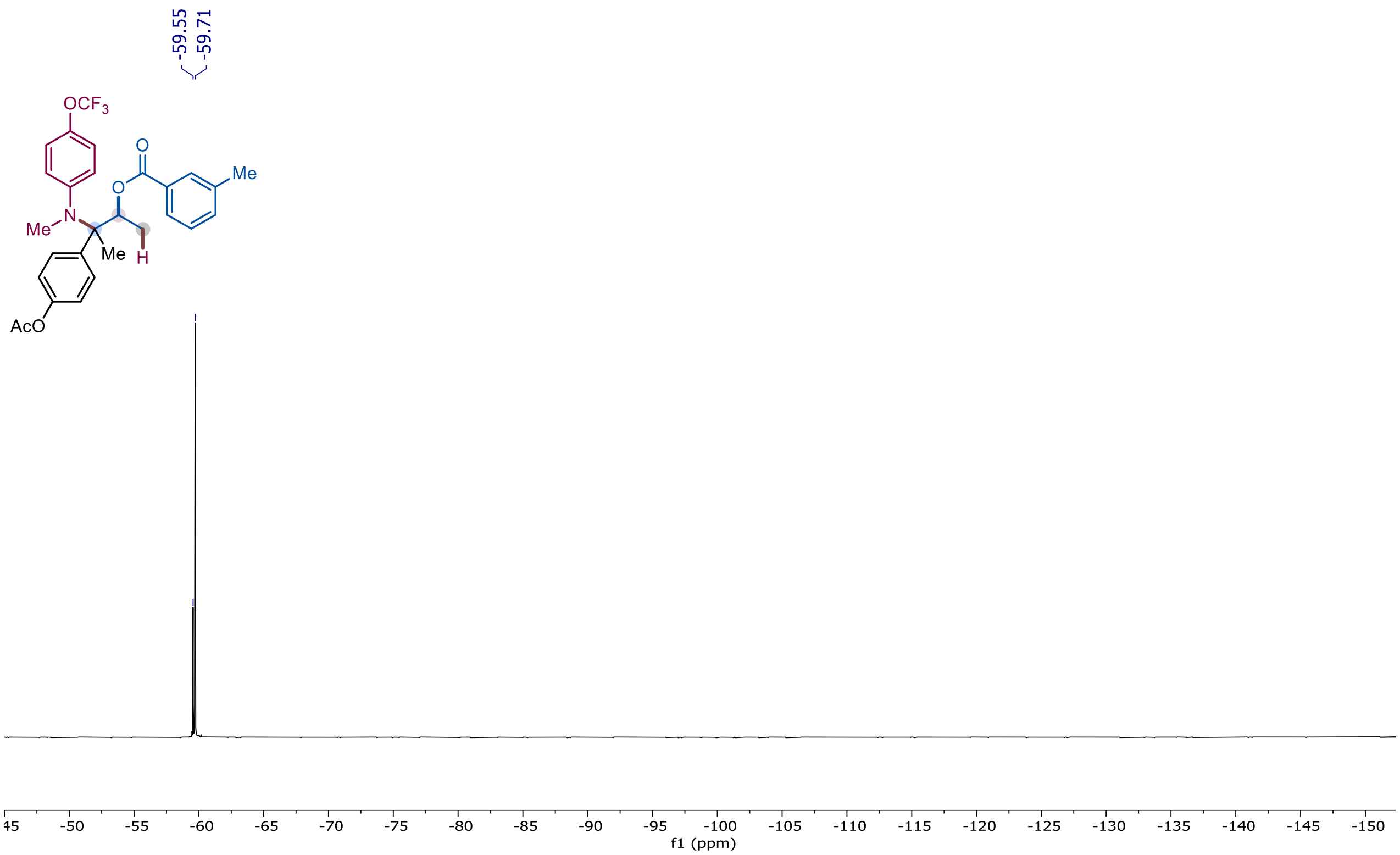

^{1}H NMR (400 MHz, $CDCl_3$, 25 ºC) of 3aaj

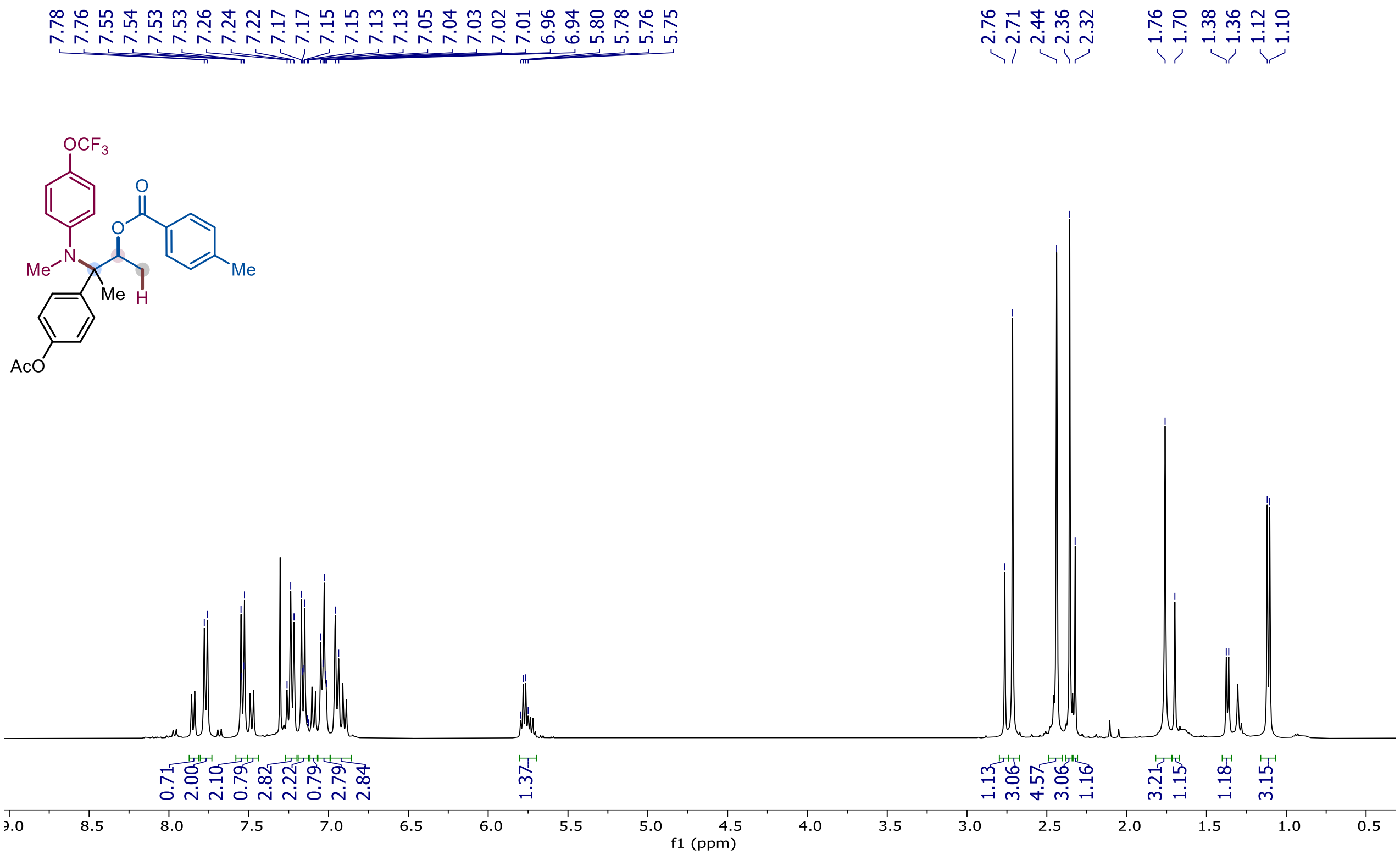

^{13}C NMR (126 MHz, $CDCl_3$, 25 ºC) of 3aaj

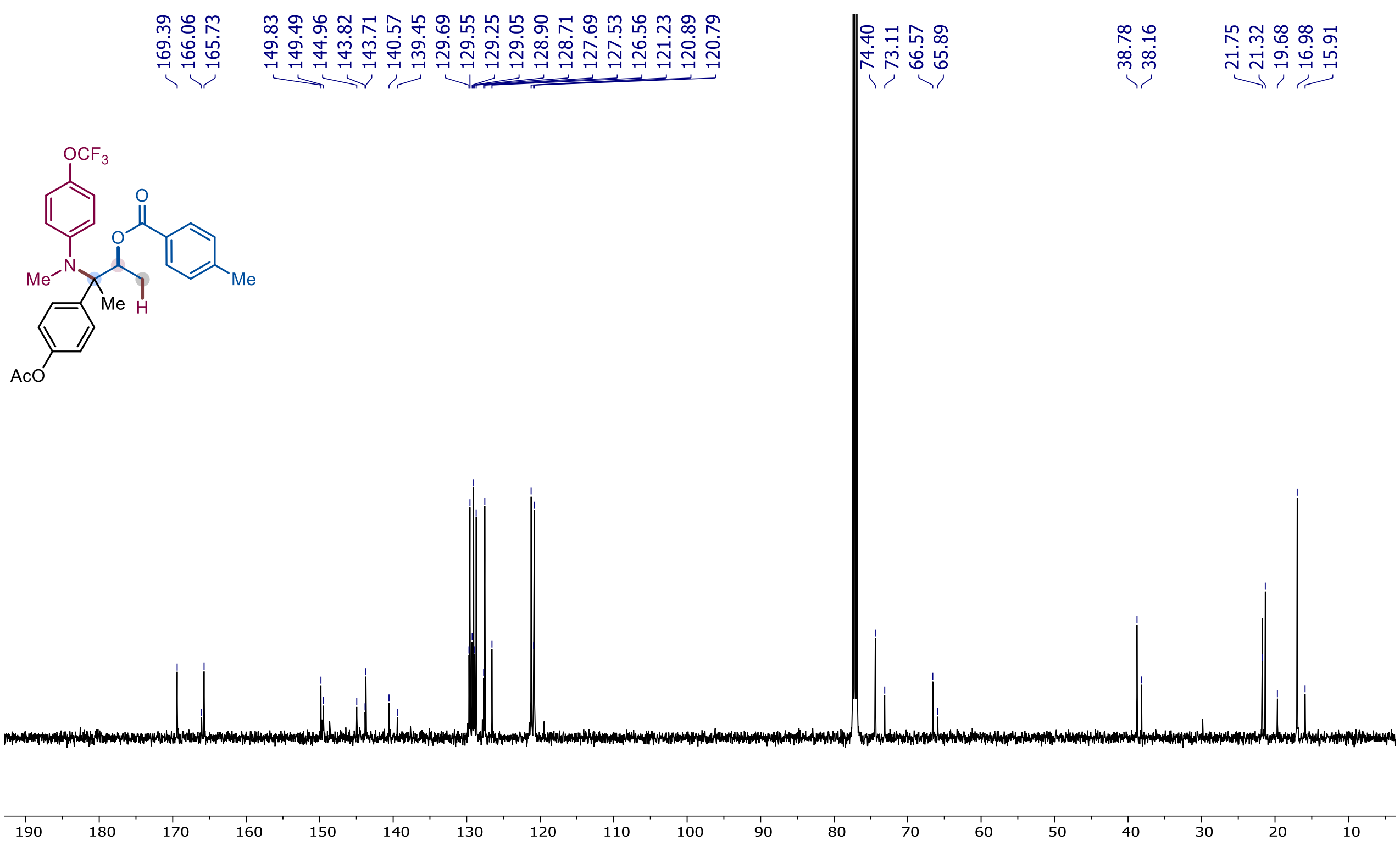

^{19}F NMR (470 MHz, $CDCl_3$, 25 °C) of 3aaj

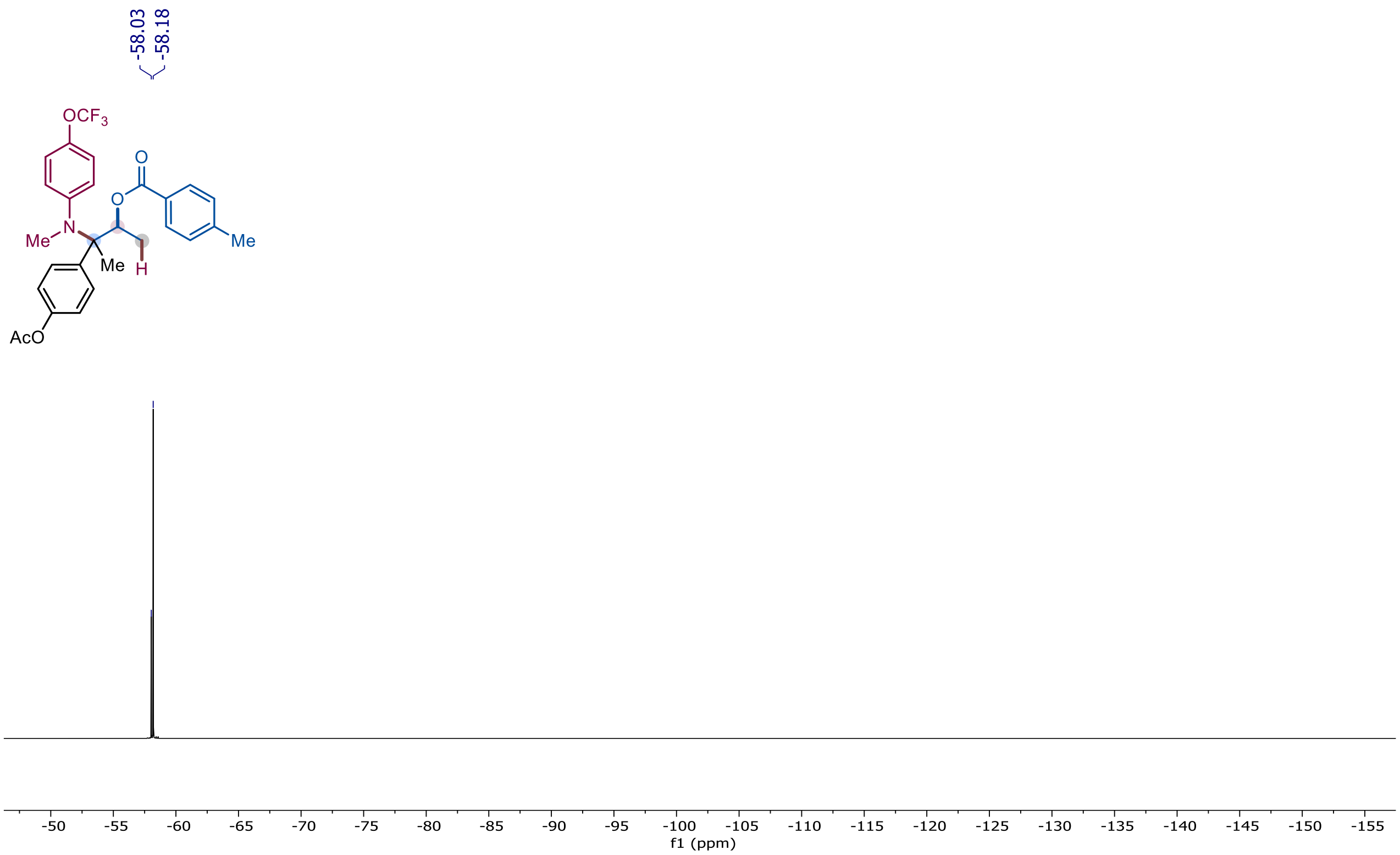

^{1}H NMR (500 MHz, $CDCl_3$, 25 ºC) of 3aak

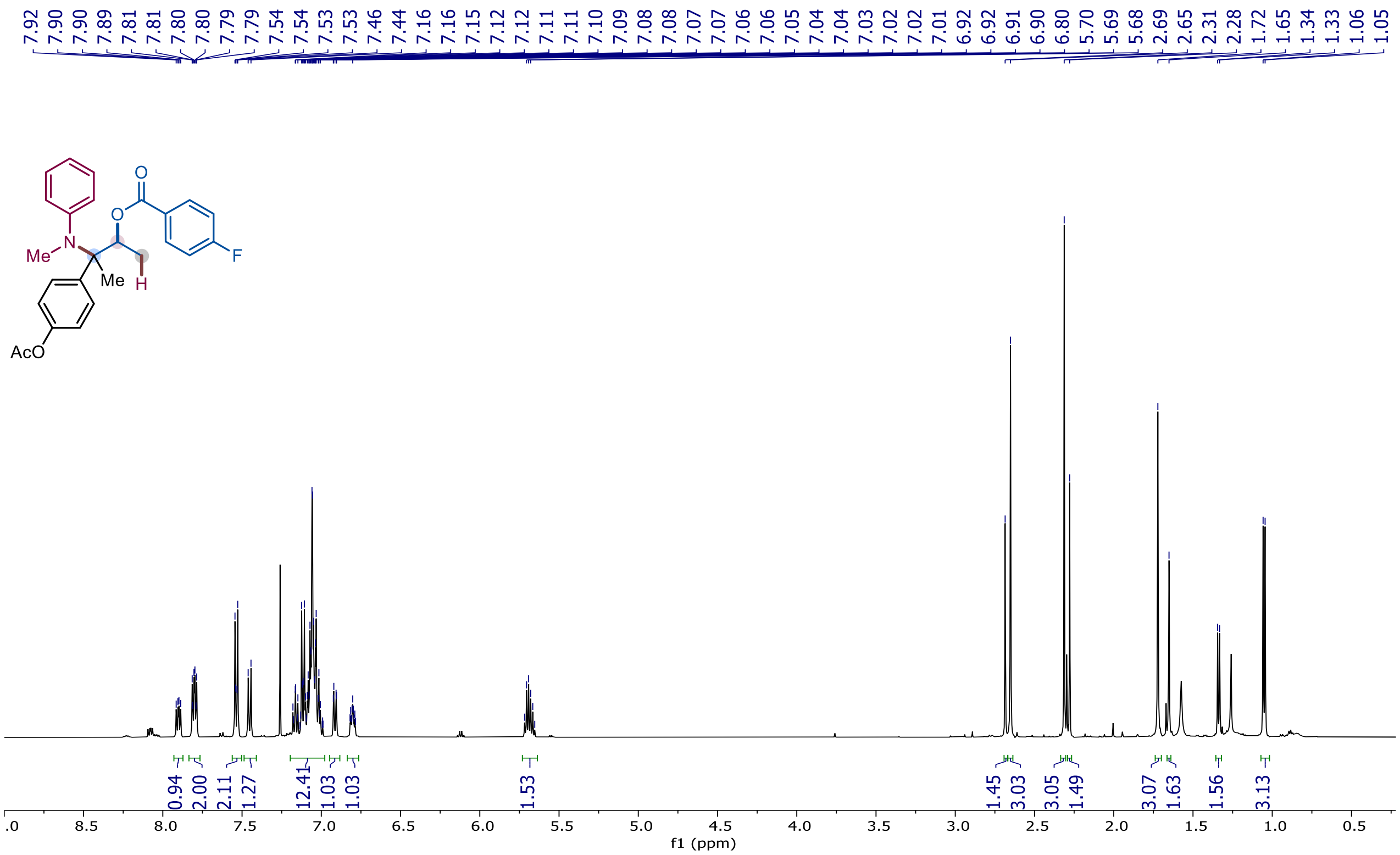

^{13}C NMR (126 MHz, CD_3CN, 25 ºC) of 3aak

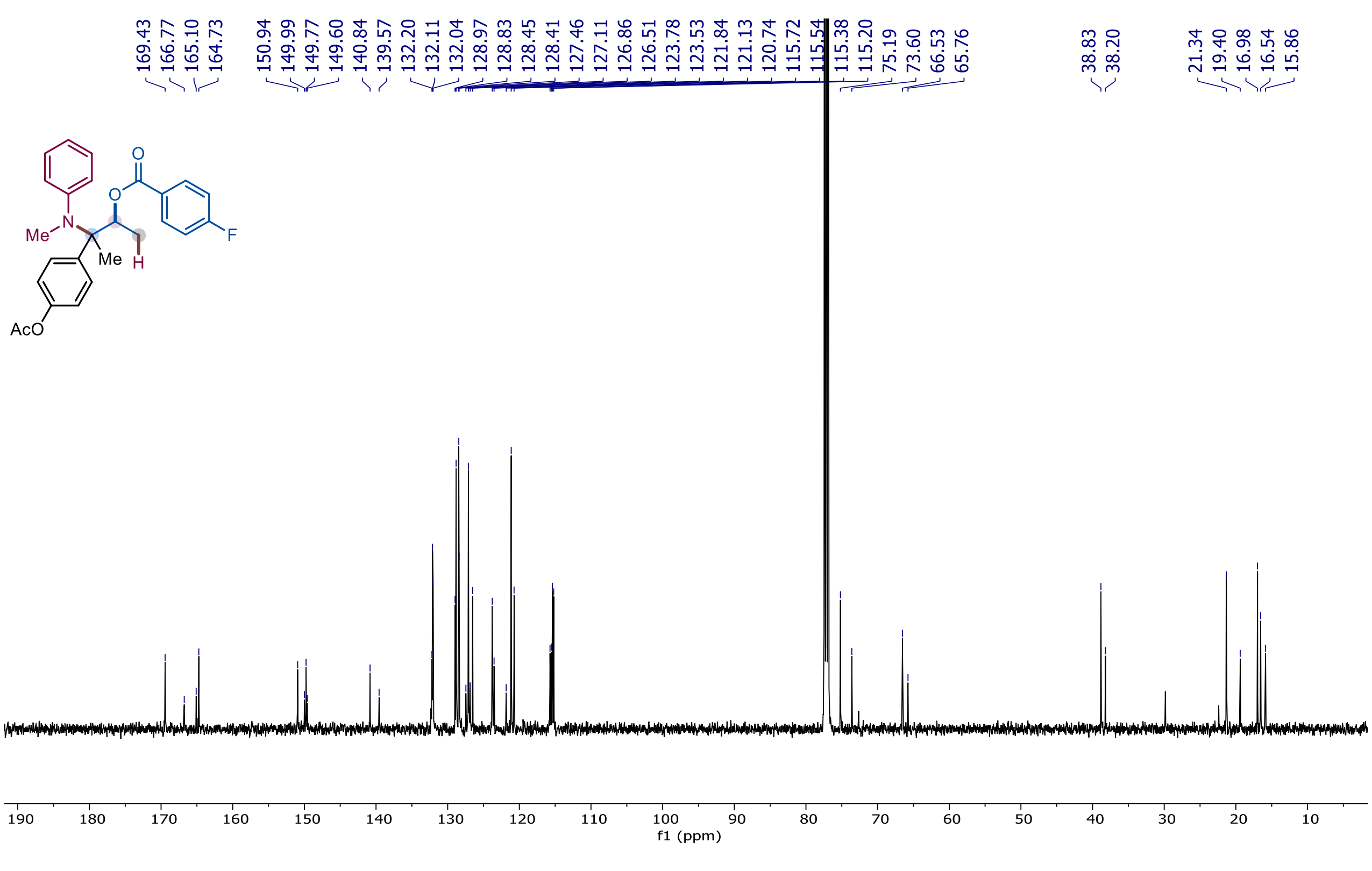

^{19}F NMR (470 MHz, $CDCl_3$, 25 ºC) of 3aak

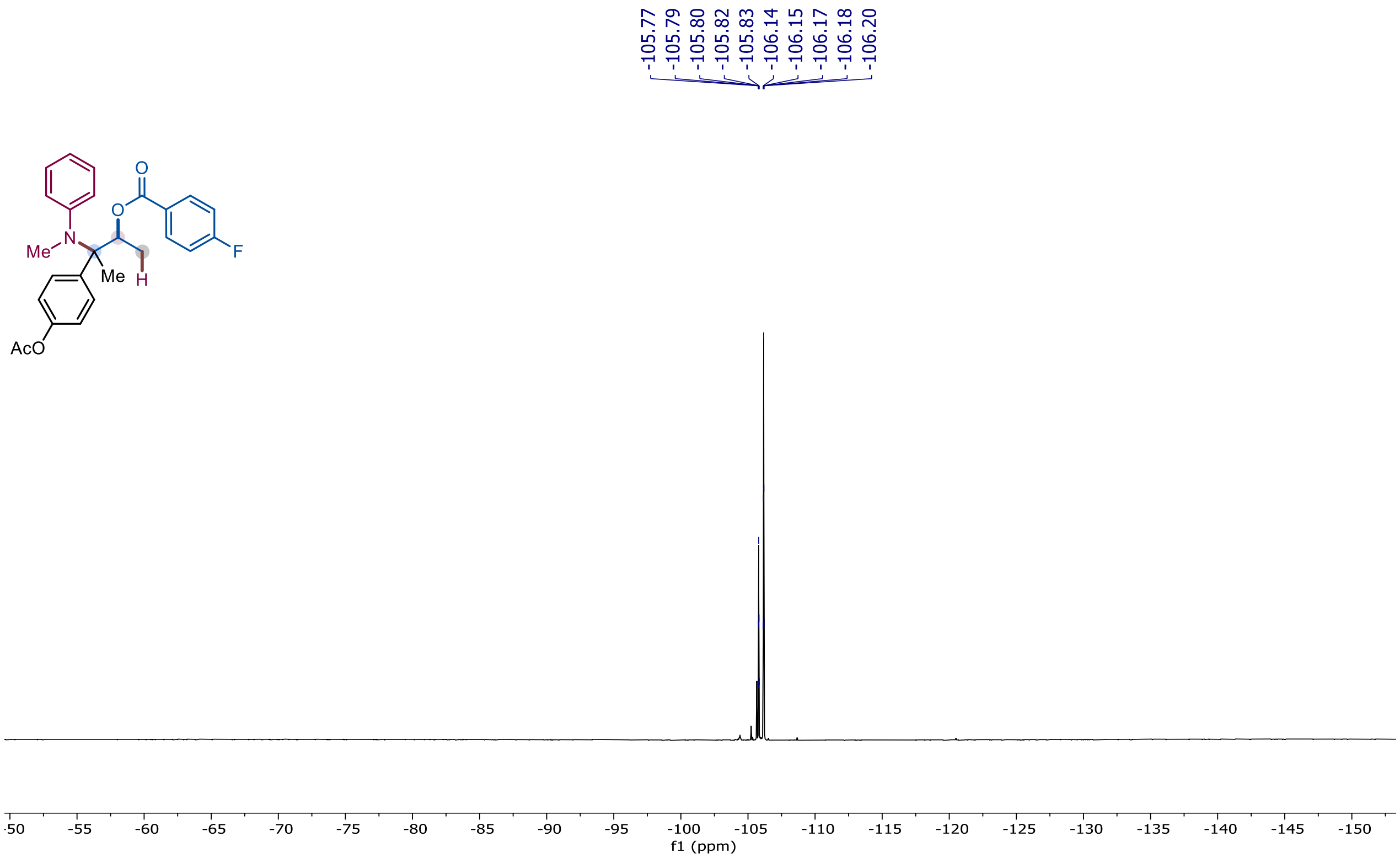

^{1}H NMR (300 MHz, CDCl$_3$, 25 ºC) of 3aal

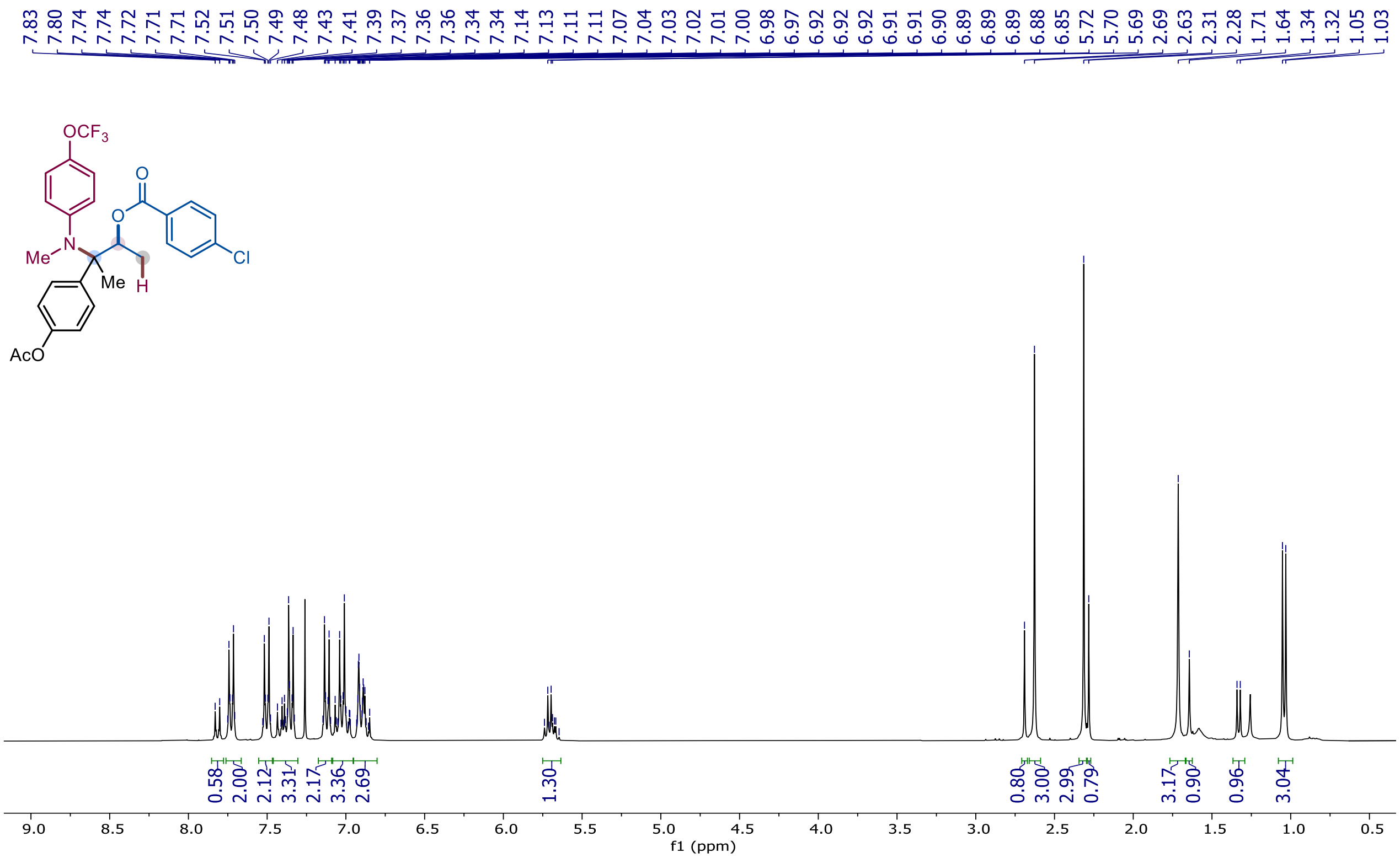

^{13}C NMR (201 MHz, CDCl$_3$, 25 ºC) of 3aal

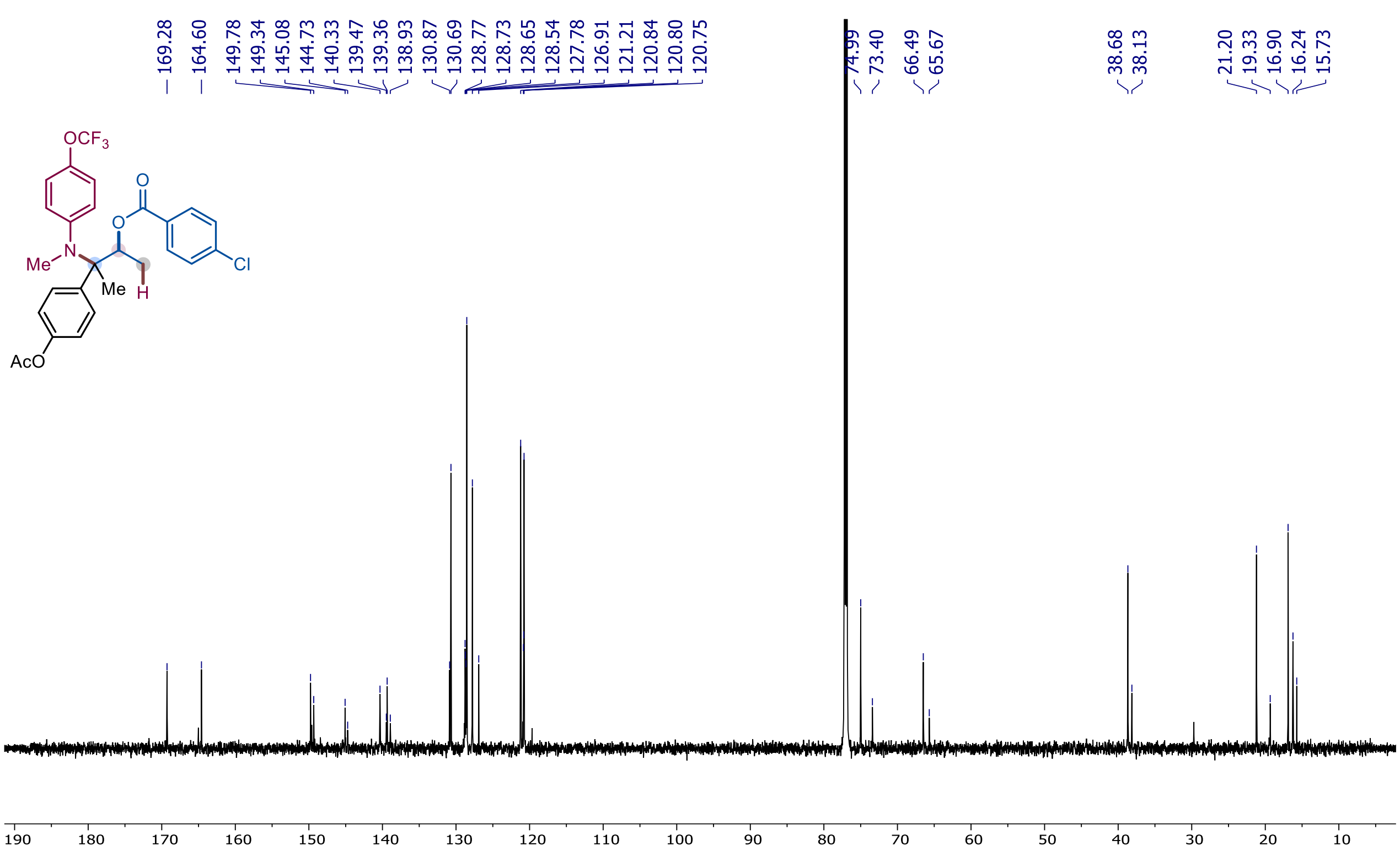

^{19}F NMR (282 MHz, CDCl$_3$, 25 °C) of 3aal

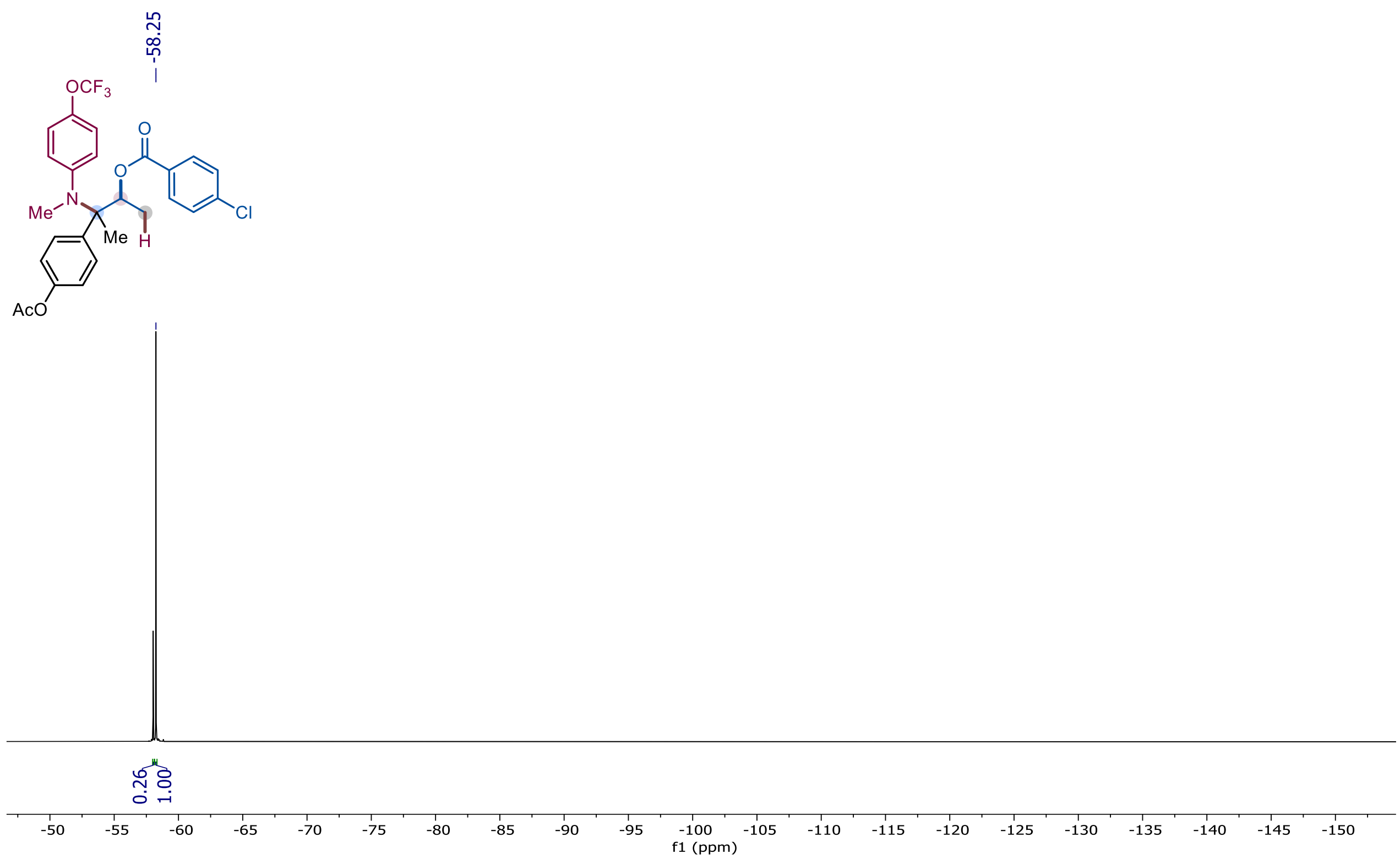

^{1}H NMR (400 MHz, CDCl$_3$, 25 °C) of 3aam

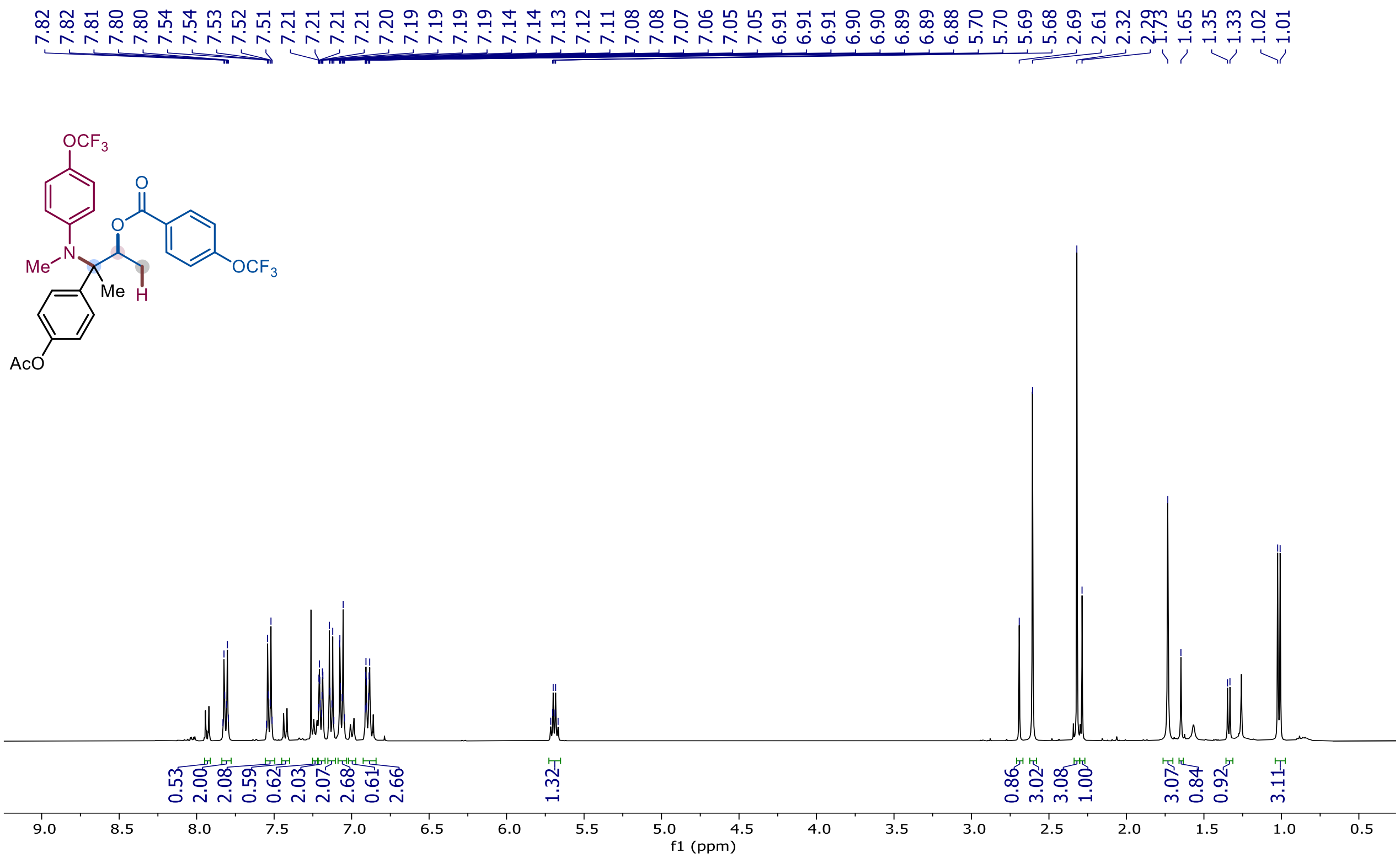

^{13}C NMR (201 MHz, CDCl$_3$, 25 °C) of 3aam

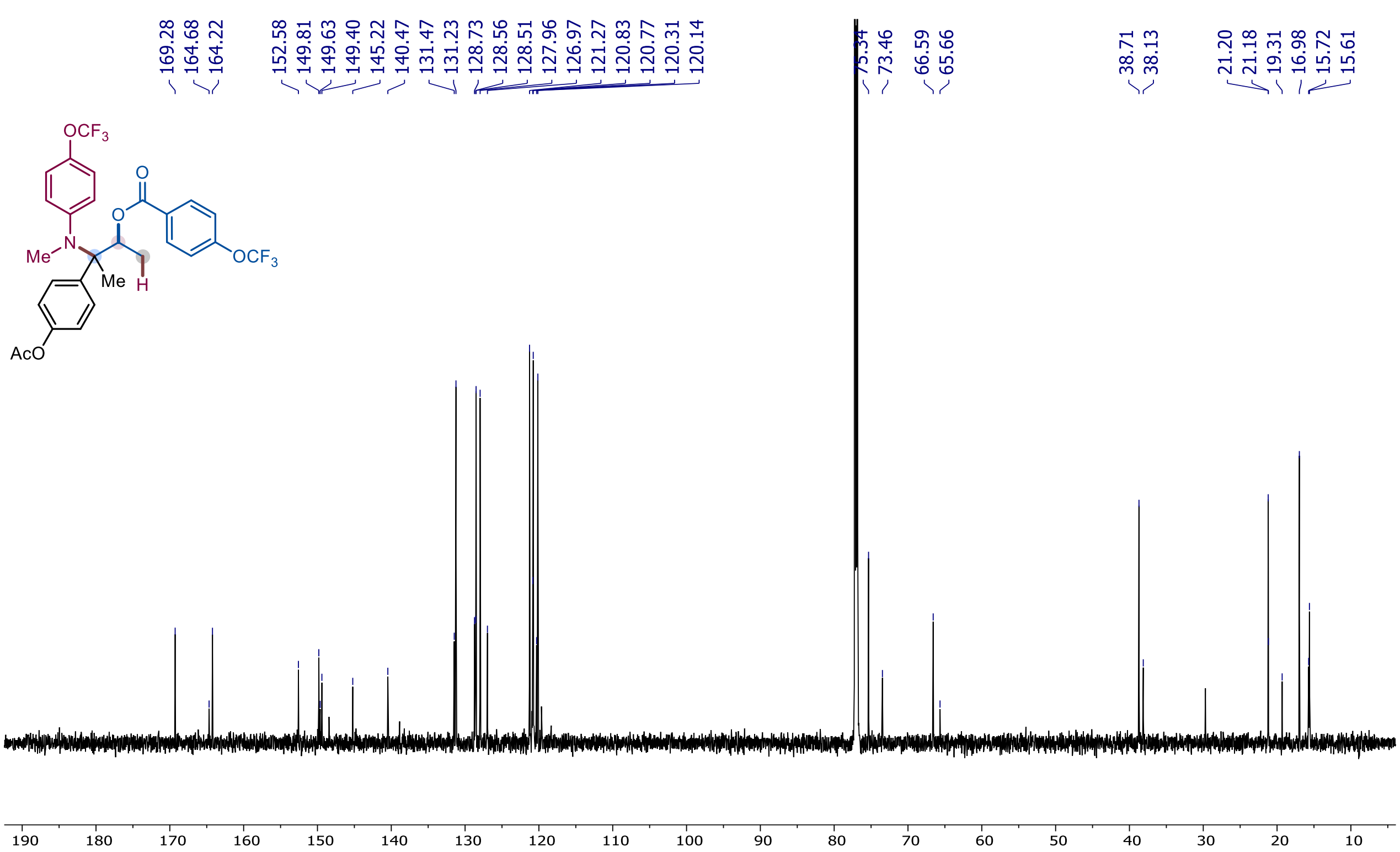

^{19}F NMR (376 MHz, CDCl$_3$, 25 °C) of 3aam

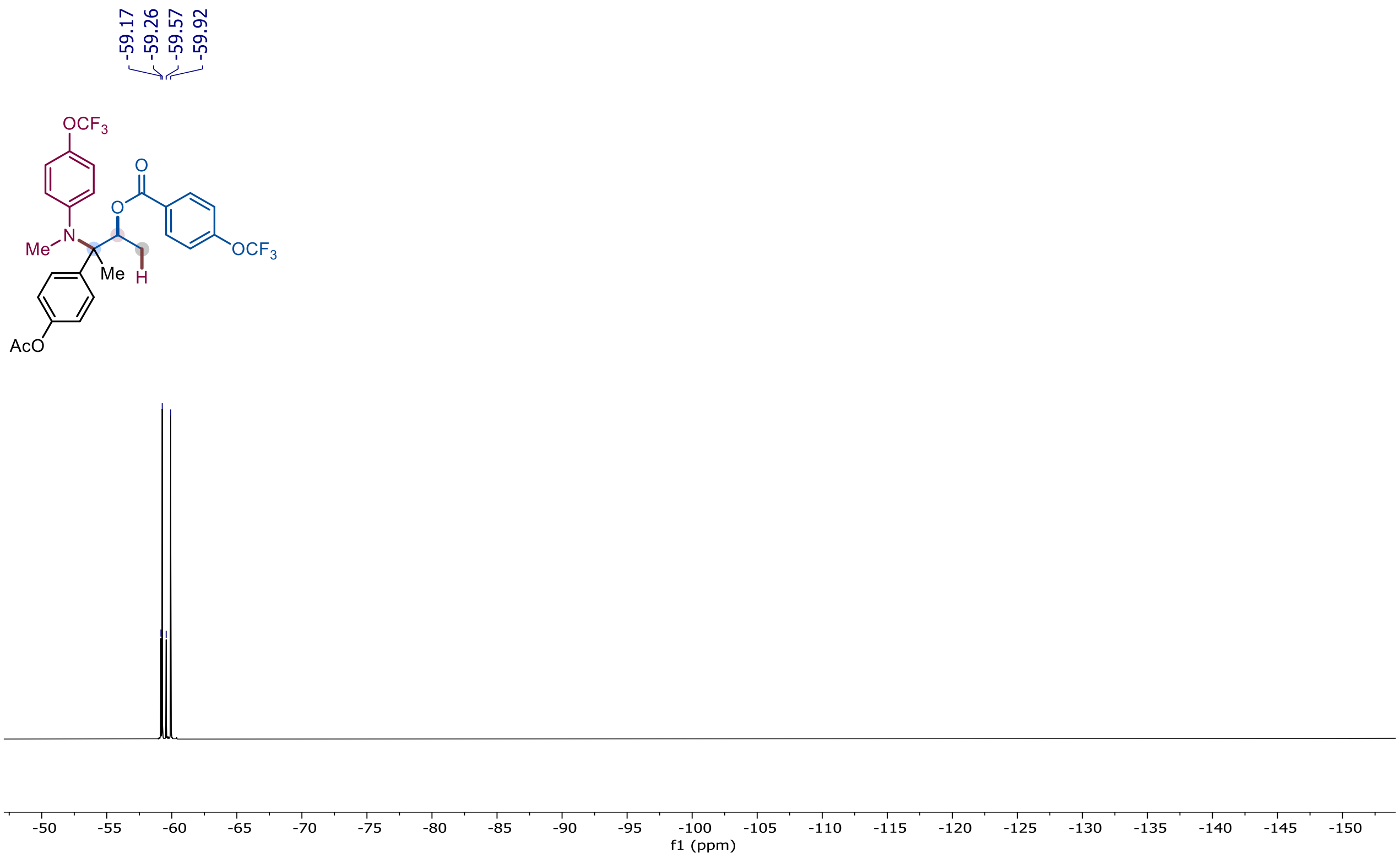

^{1}H NMR (400 MHz, CDCl$_3$, 25 ºC) of 3aan

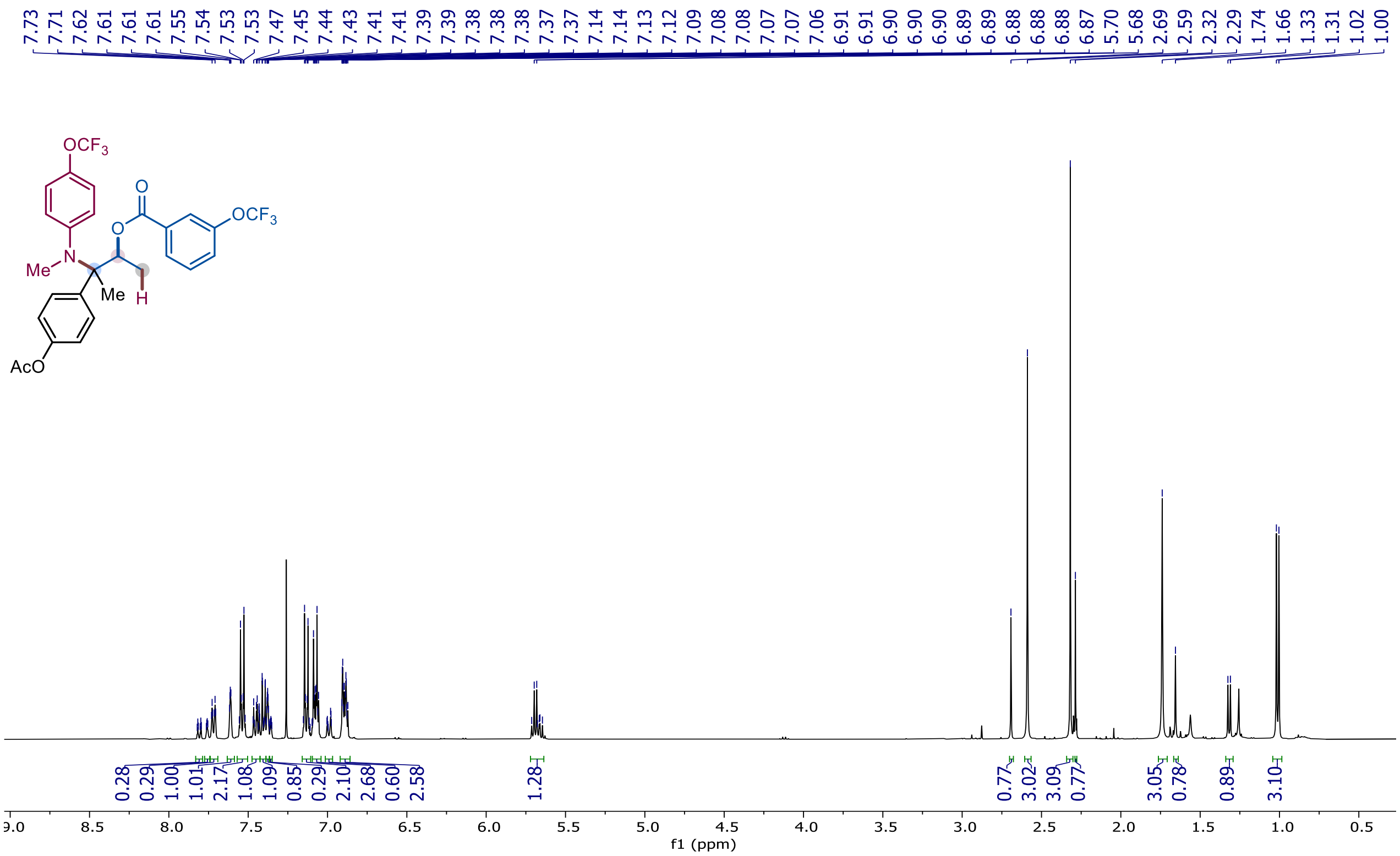

^{13}C NMR (201 MHz, CDCl$_3$, 25 ºC) of 3aan

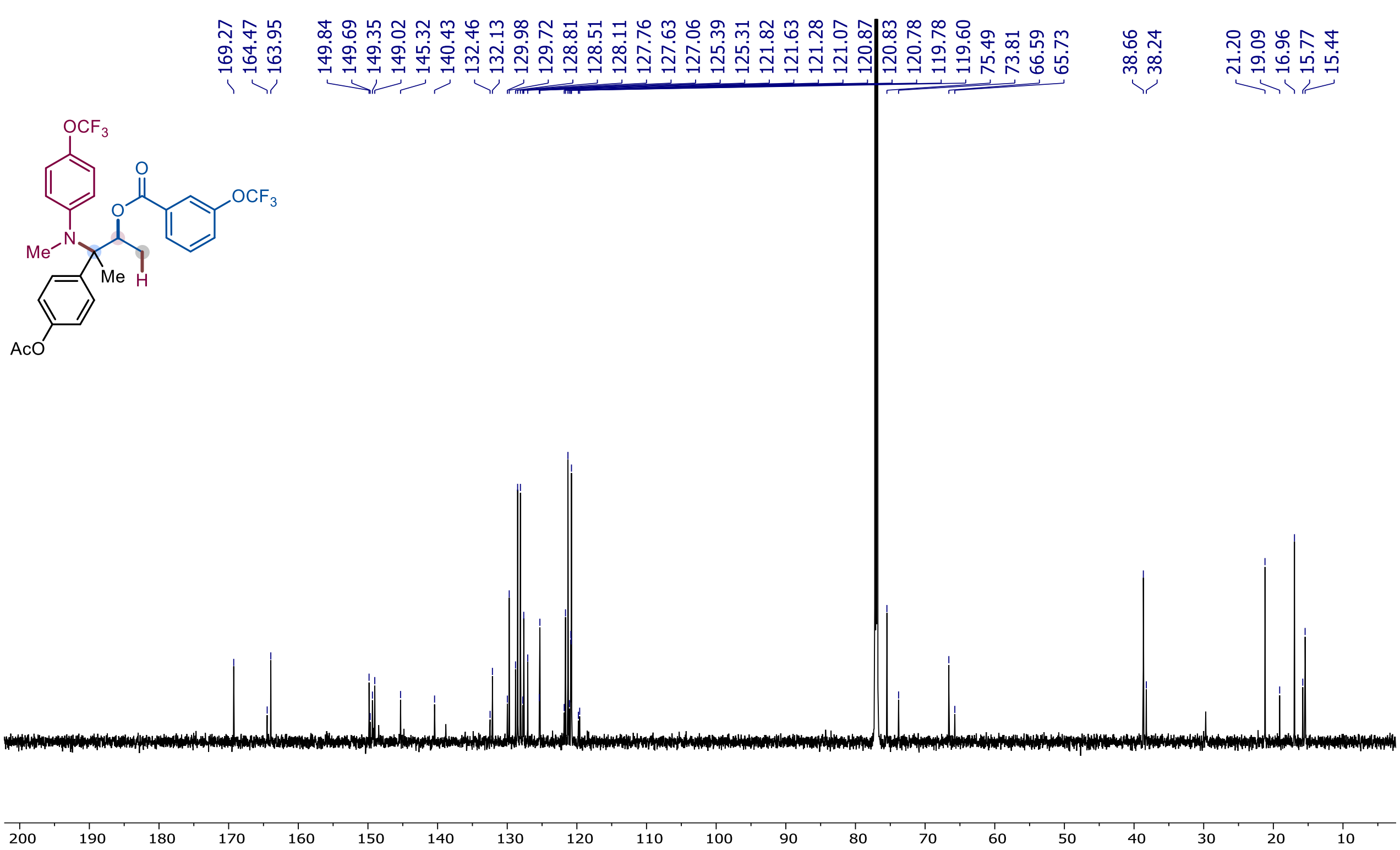

^{19}F NMR (376 MHz, $CDCl_3$, 25 °C) of 3aan

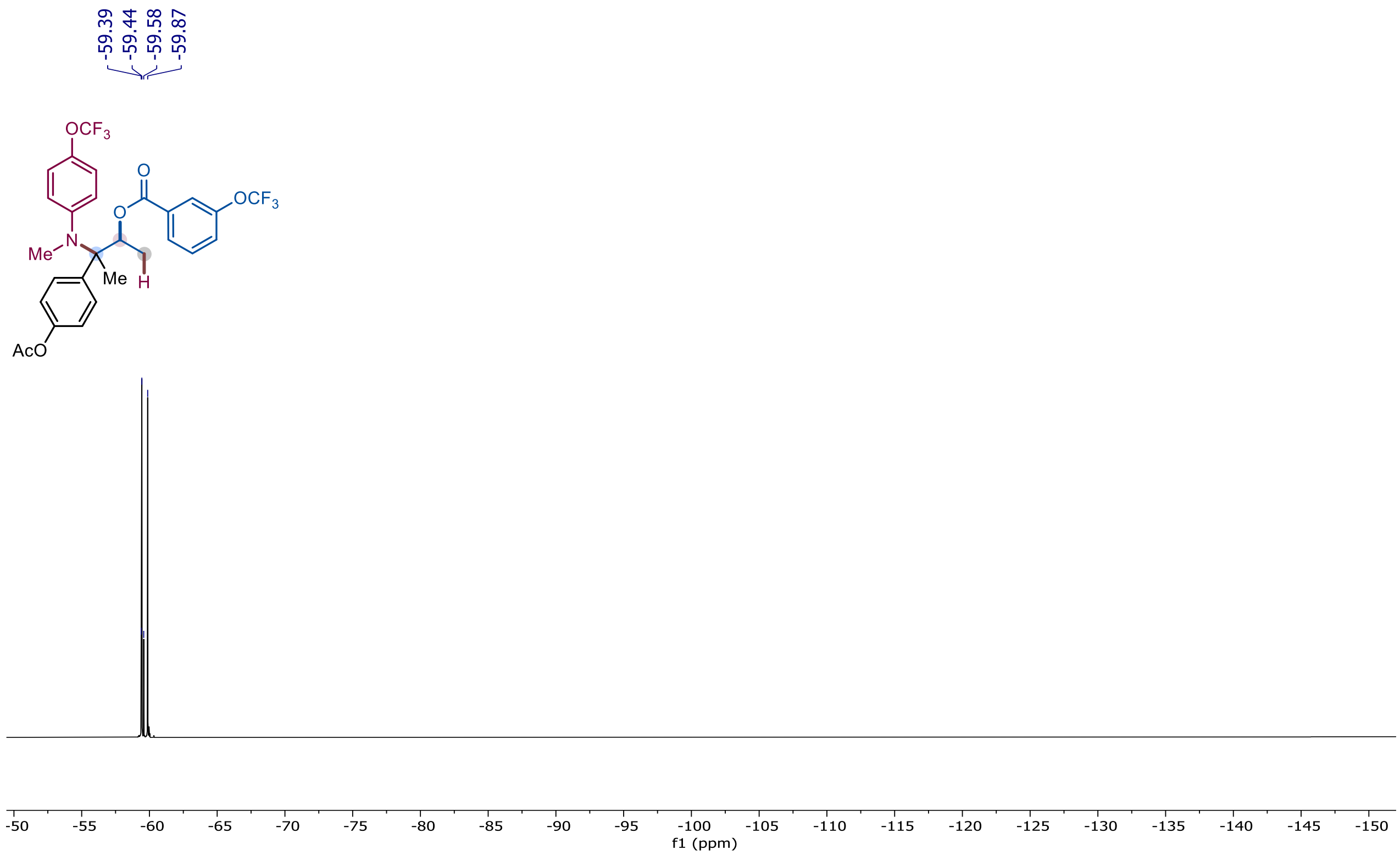

^{1}H NMR (400 MHz, $CDCl_3$, 25 ºC) of 3aao

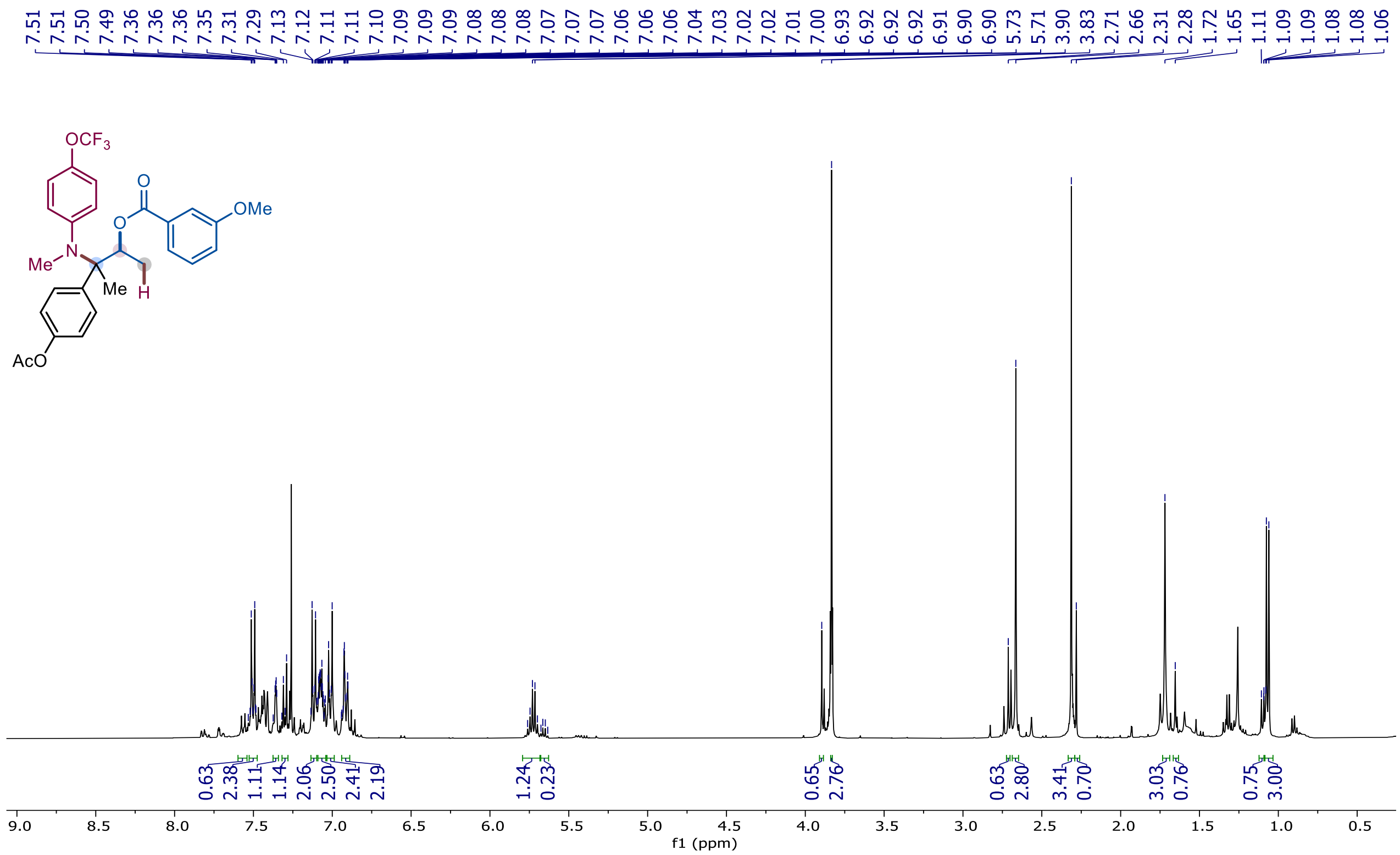

^{13}C NMR (201 MHz, $CDCl_3$, 25 ºC) of 3aao

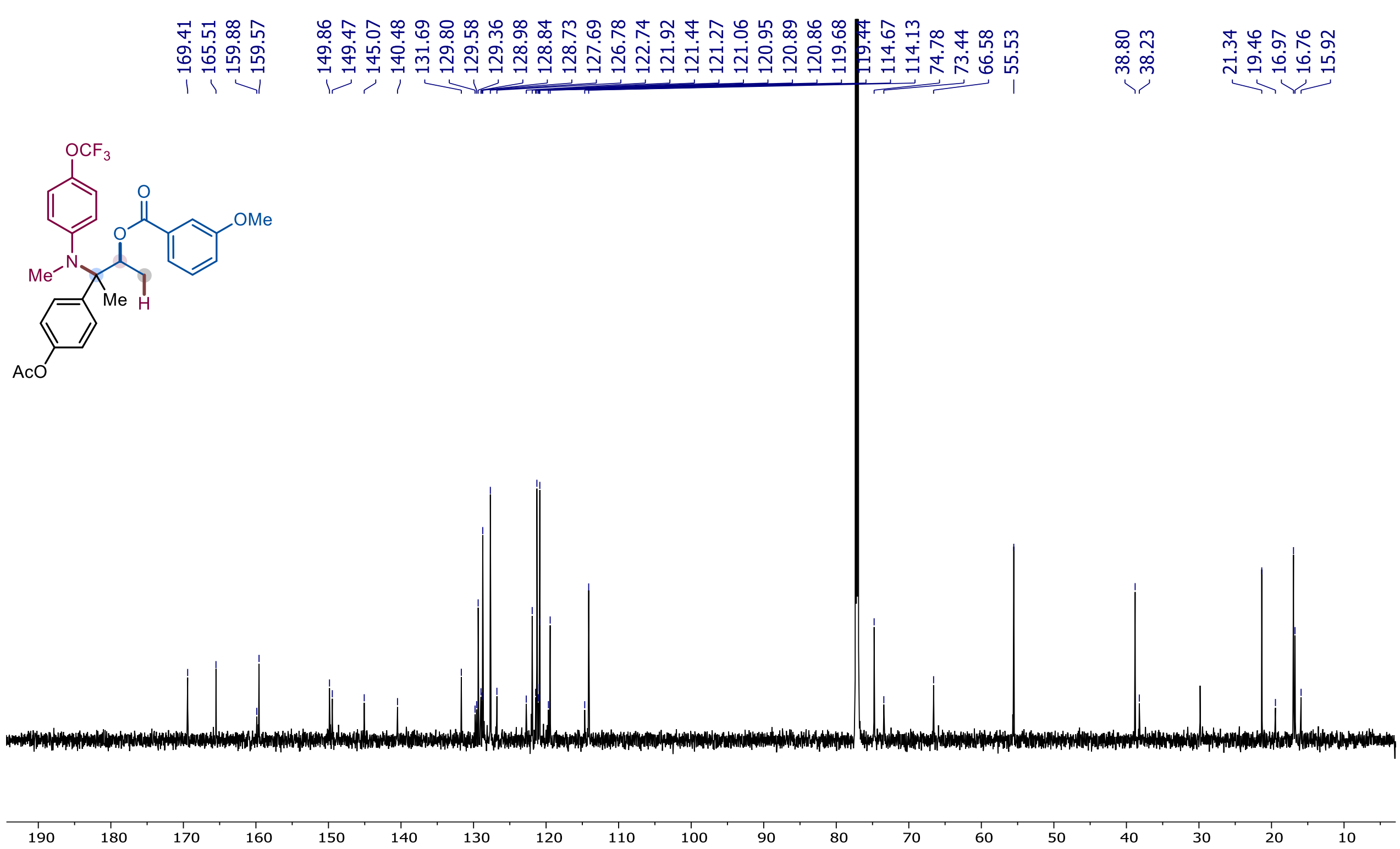

^{19}F NMR (282 MHz, CDCl3, 25 ºC) of 3aao

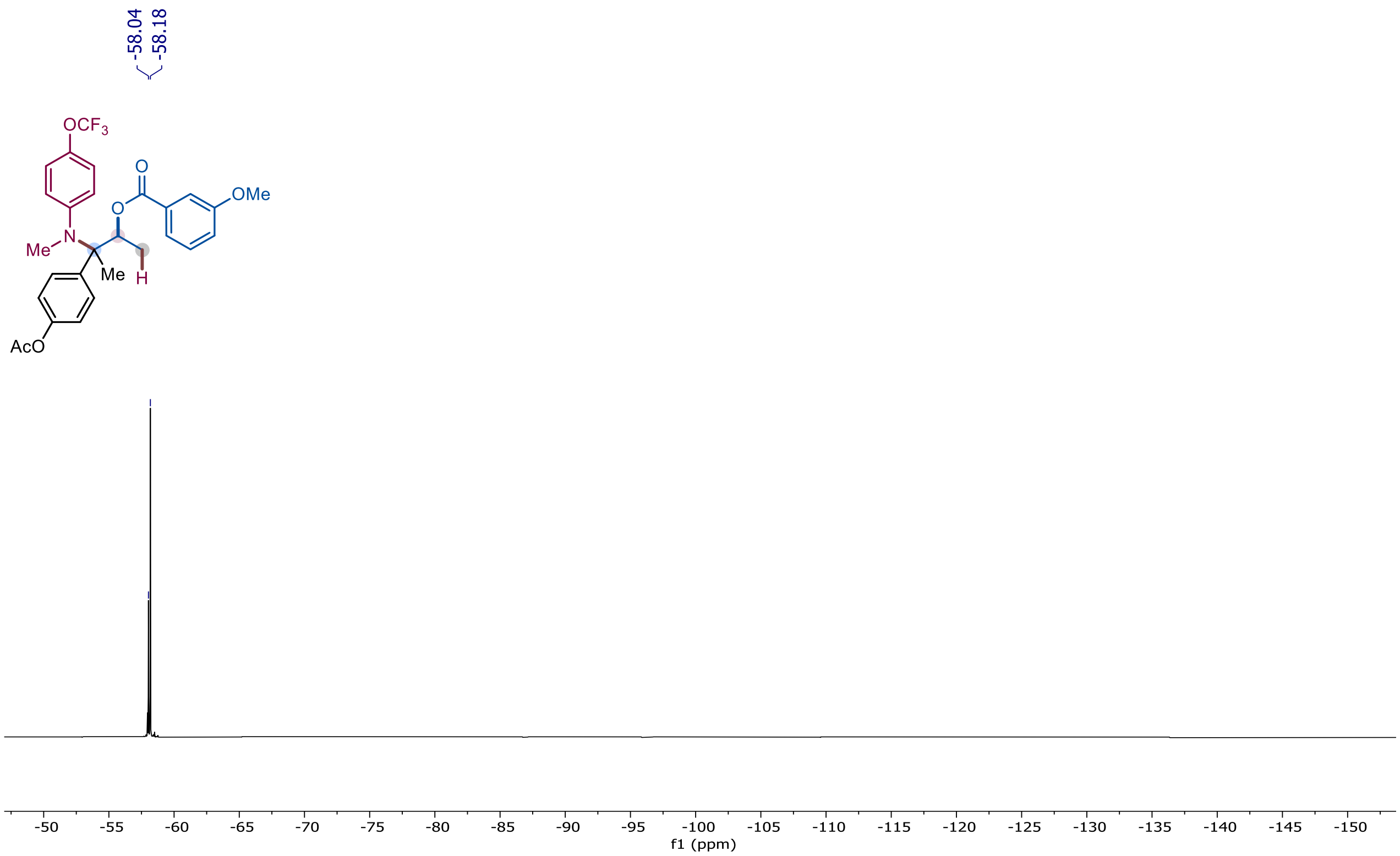

^{1}H NMR (300 MHz, CDCl$_3$, 25 °C) of 3aap

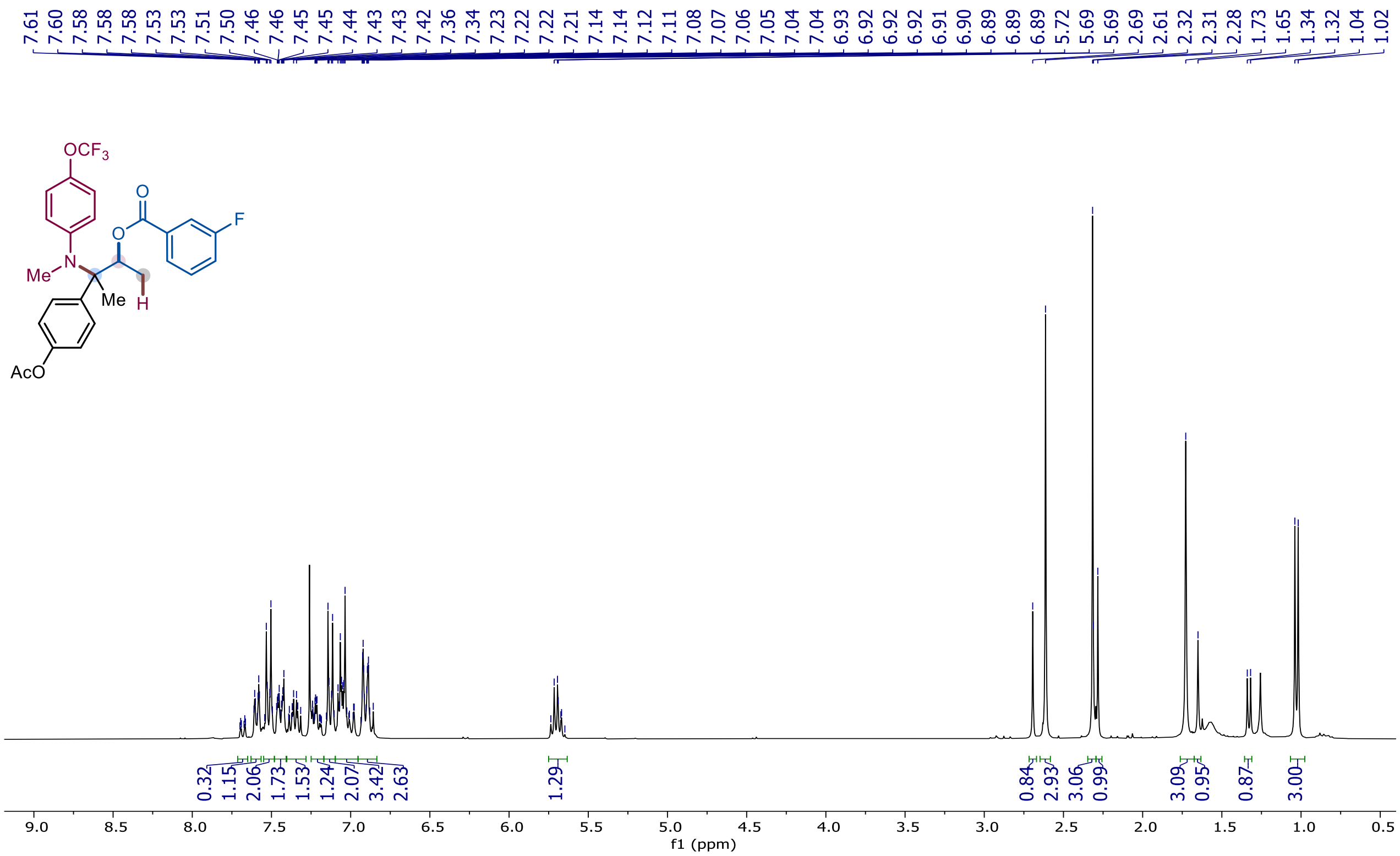

^{13}C NMR (201 MHz, CDCl$_3$, 25 °C) of 3aap

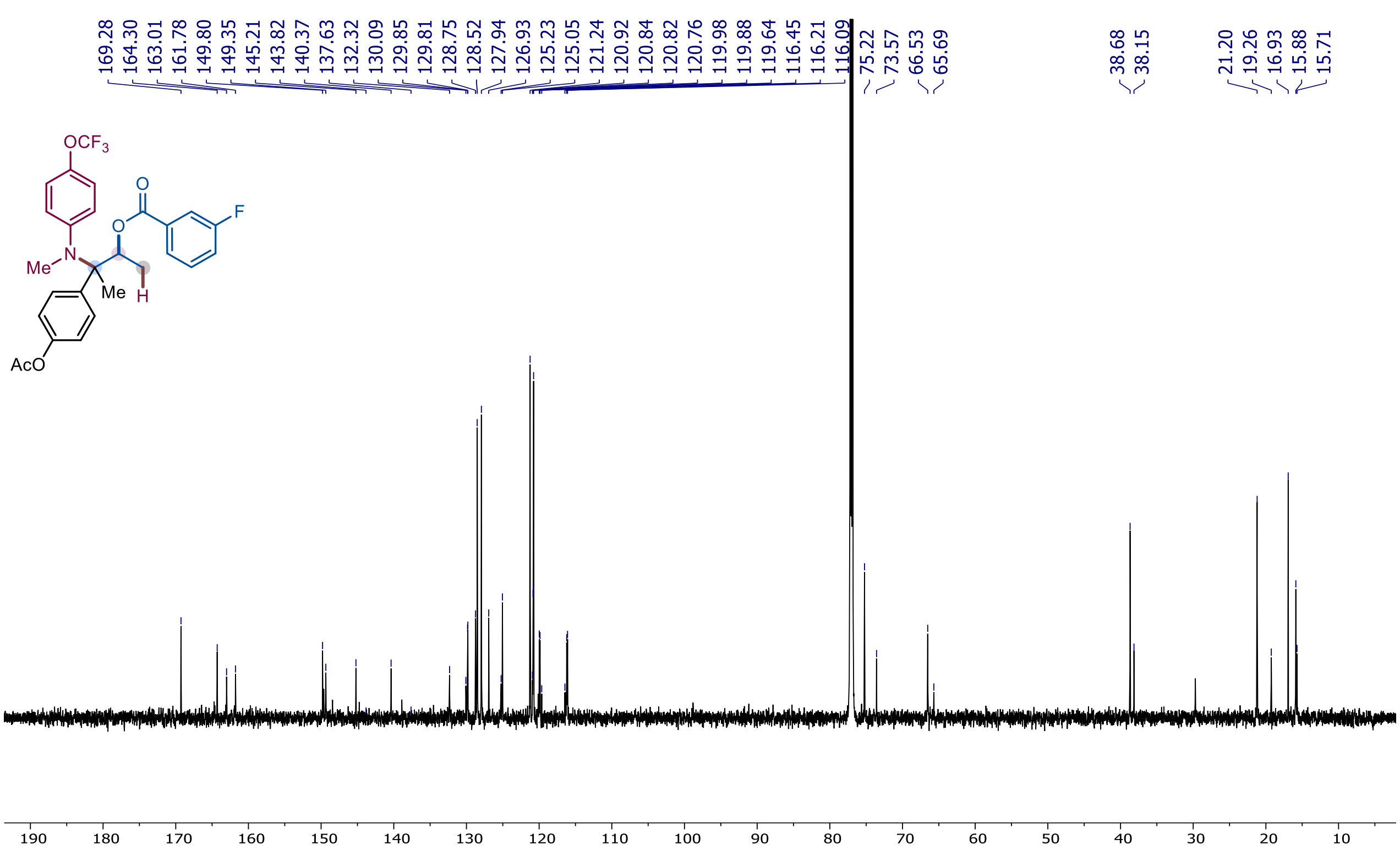

^{19}F NMR (282 MHz, CDCl$_3$, 25 ºC) of 3aap

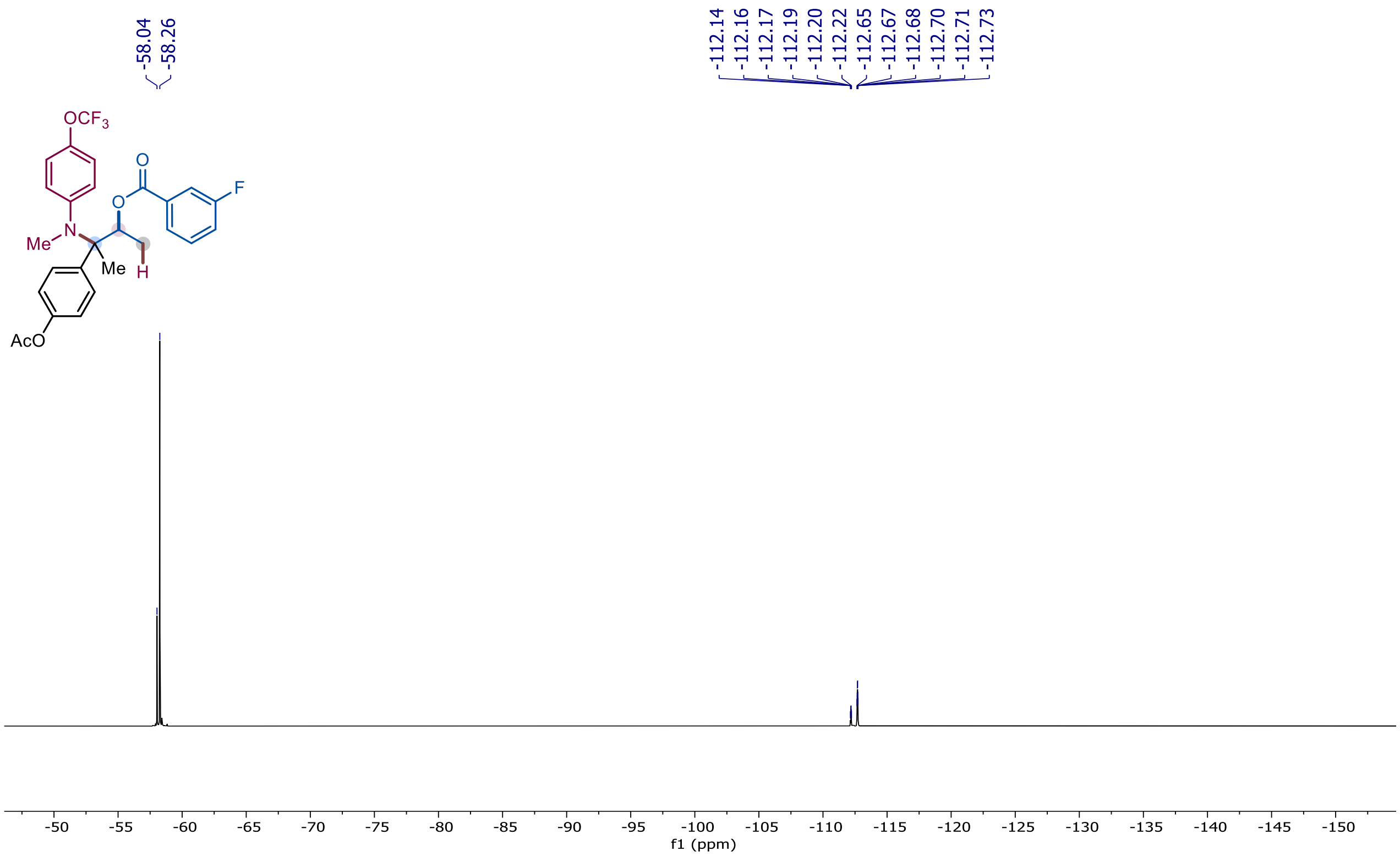

^{1}H NMR (400 MHz, $CDCl_3$, 25 ºC) of 3aaq

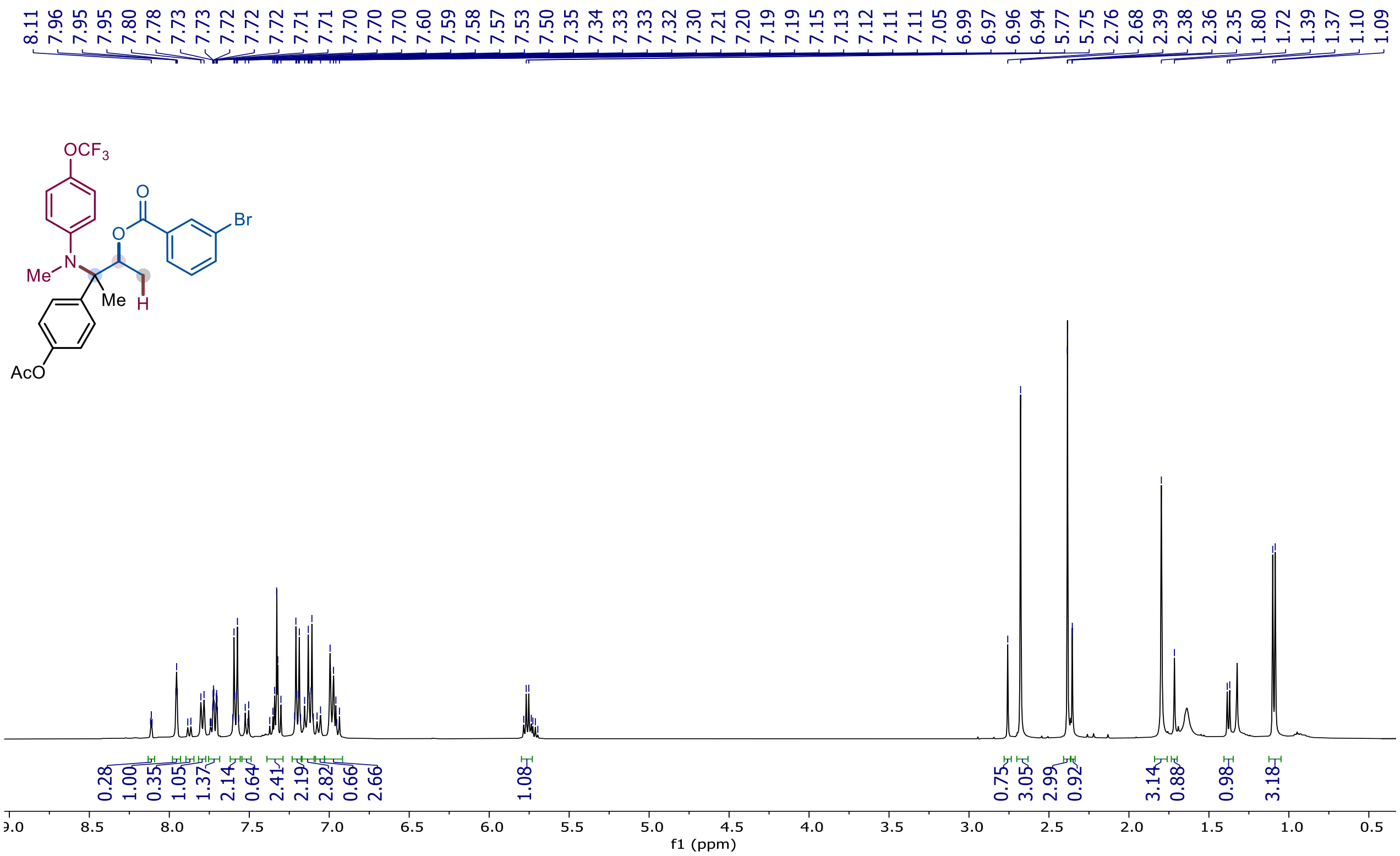

^{13}C NMR (201 MHz, $CDCl_3$, 25 ºC) of 3aaq

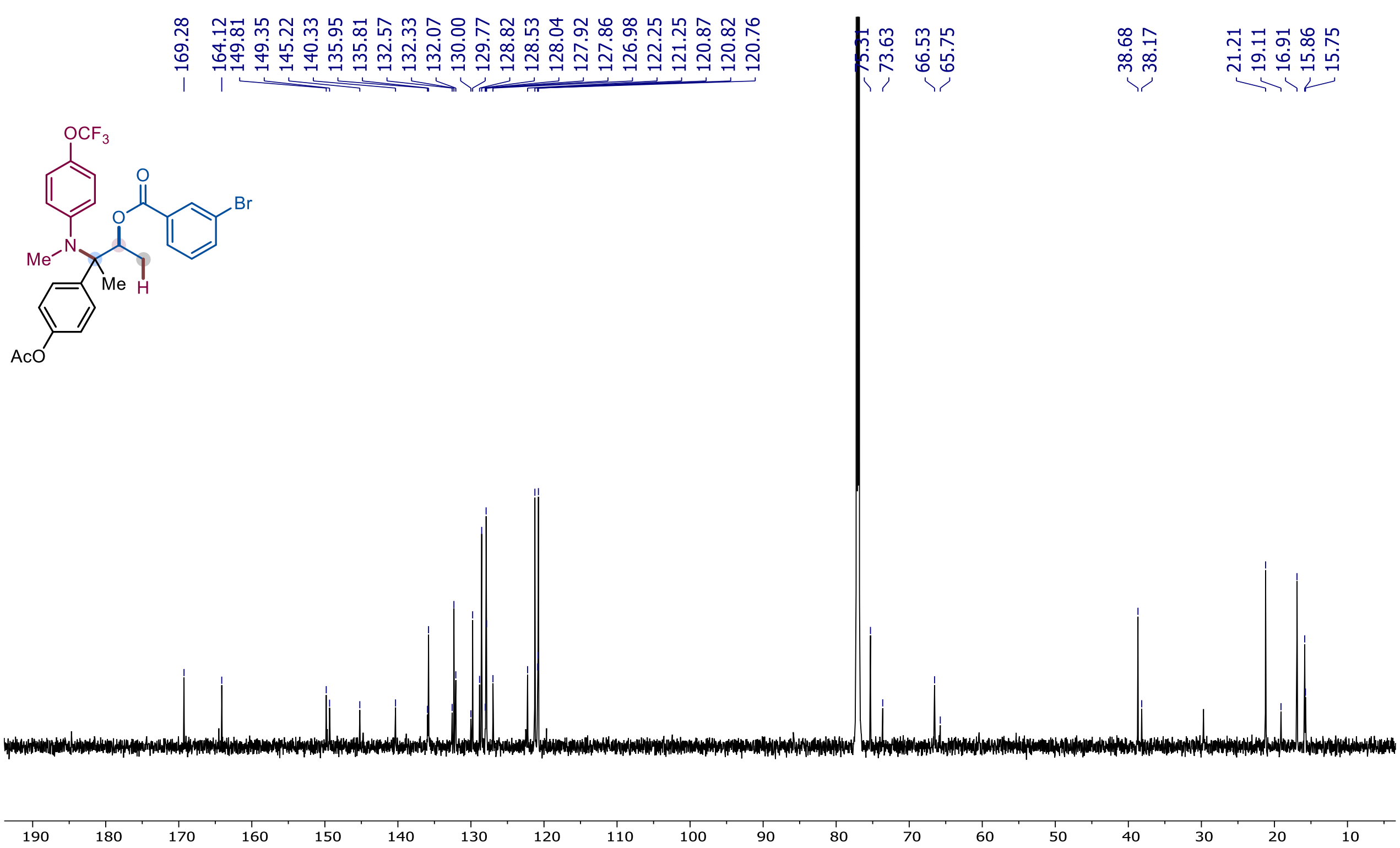

^{19}F NMR (376 MHz, CDCl₃, 25 ºC) of 3aaq

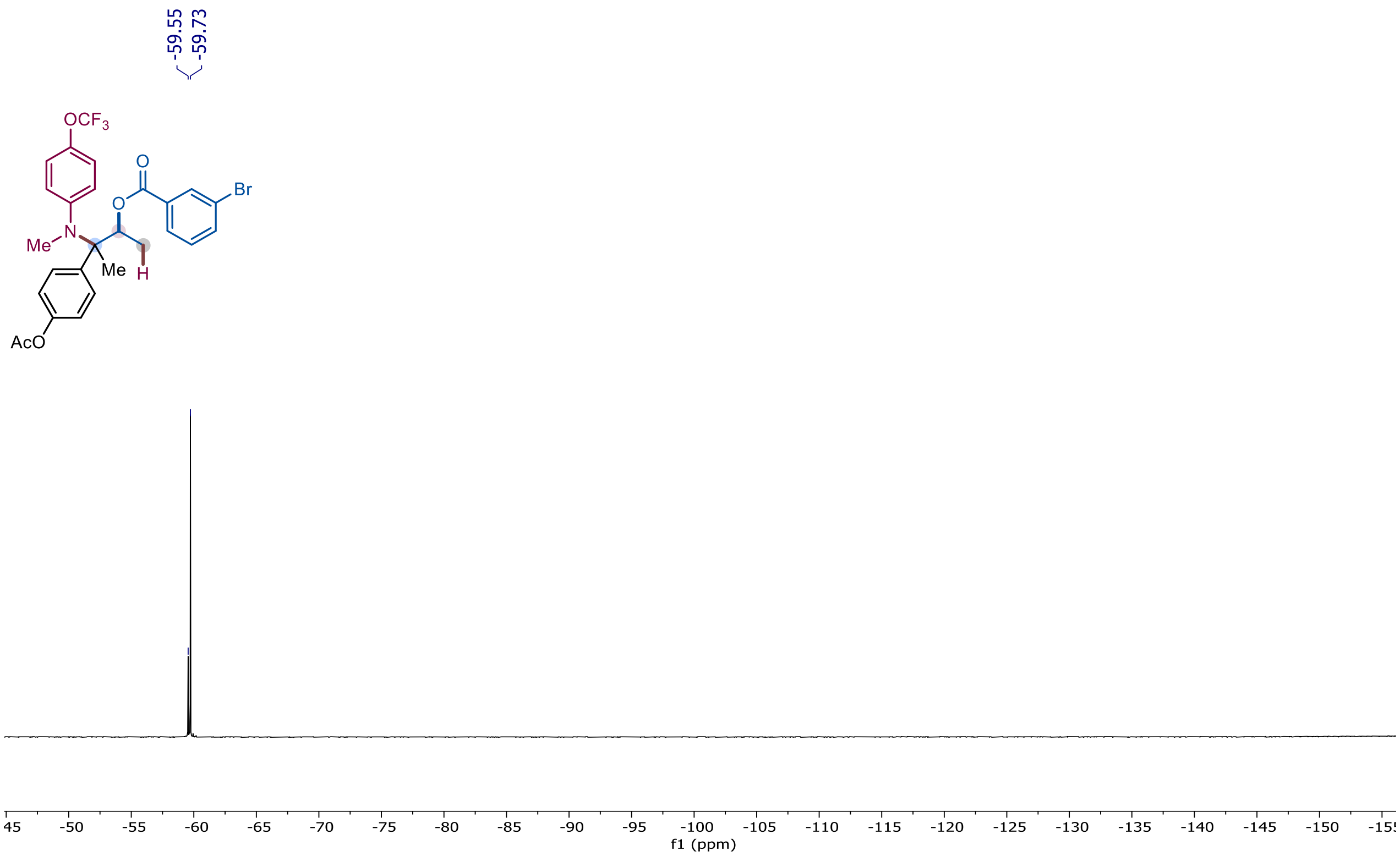

^{1}H NMR (500 MHz, $CDCl_3$, 25 ºC) of 3aar

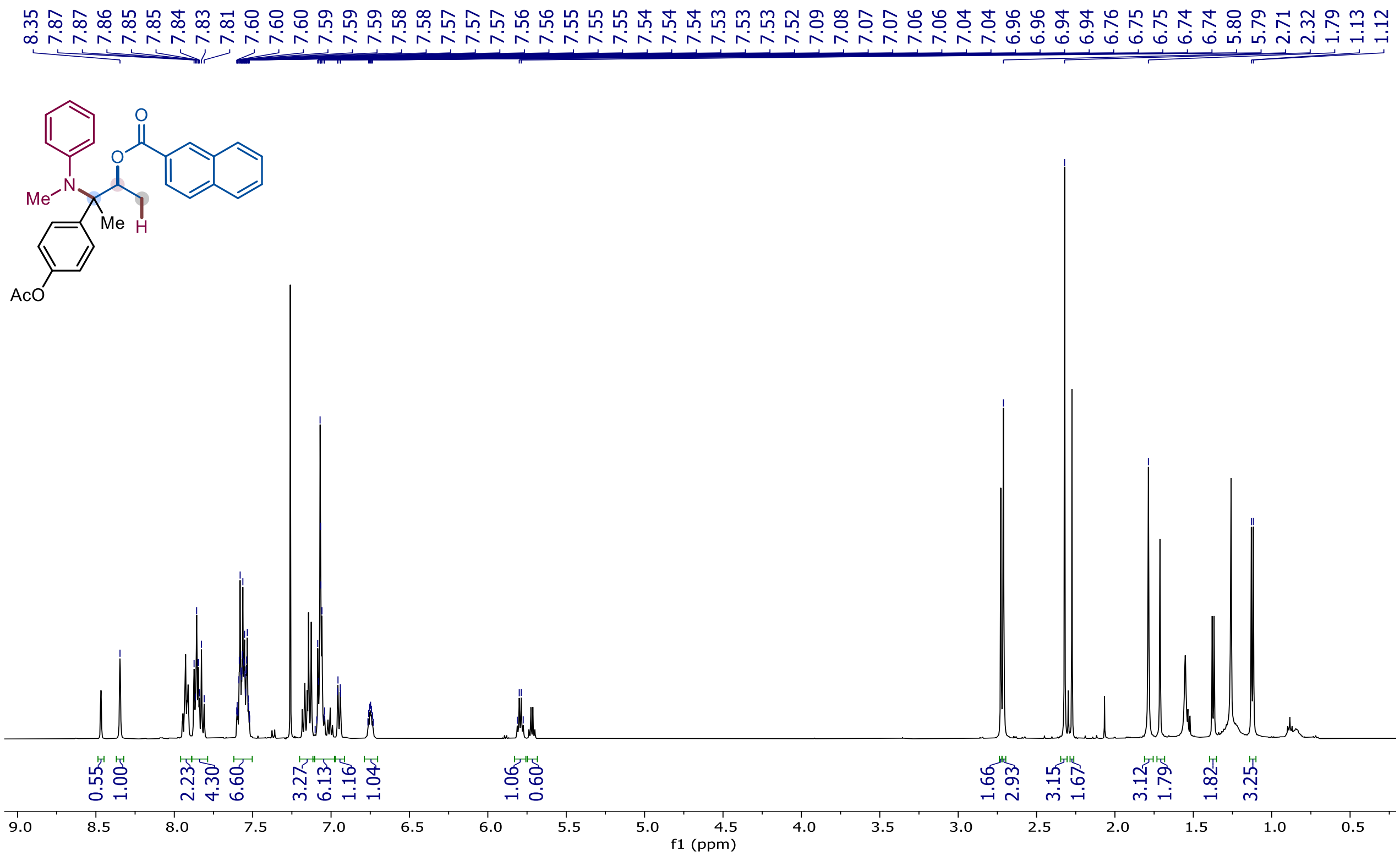

^{13}C NMR (126 MHz, $CDCl_3$, 25 ºC) of 3aar

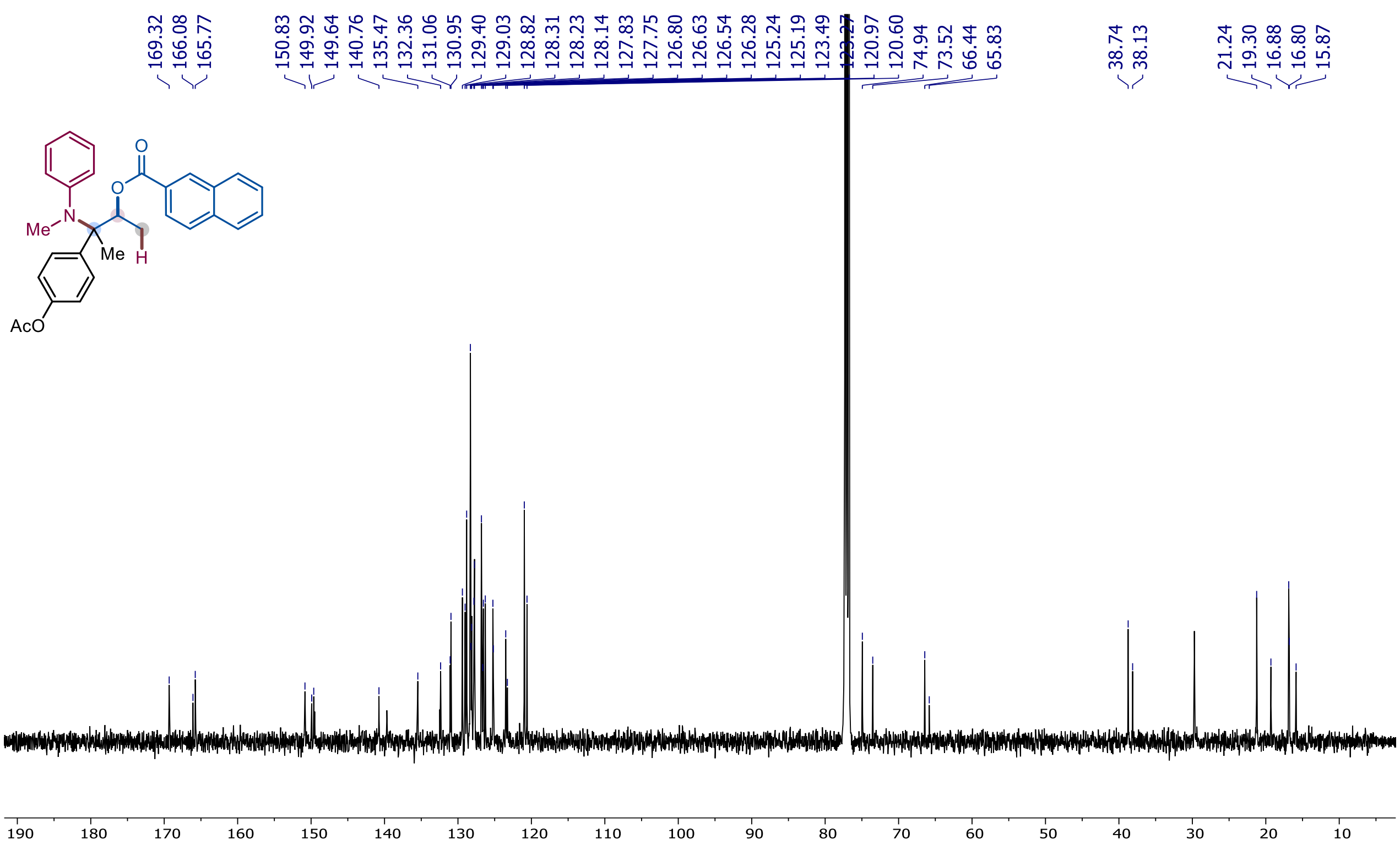

^{1}H NMR (300 MHz, $CDCl_3$, 25 ºC) of 3aas

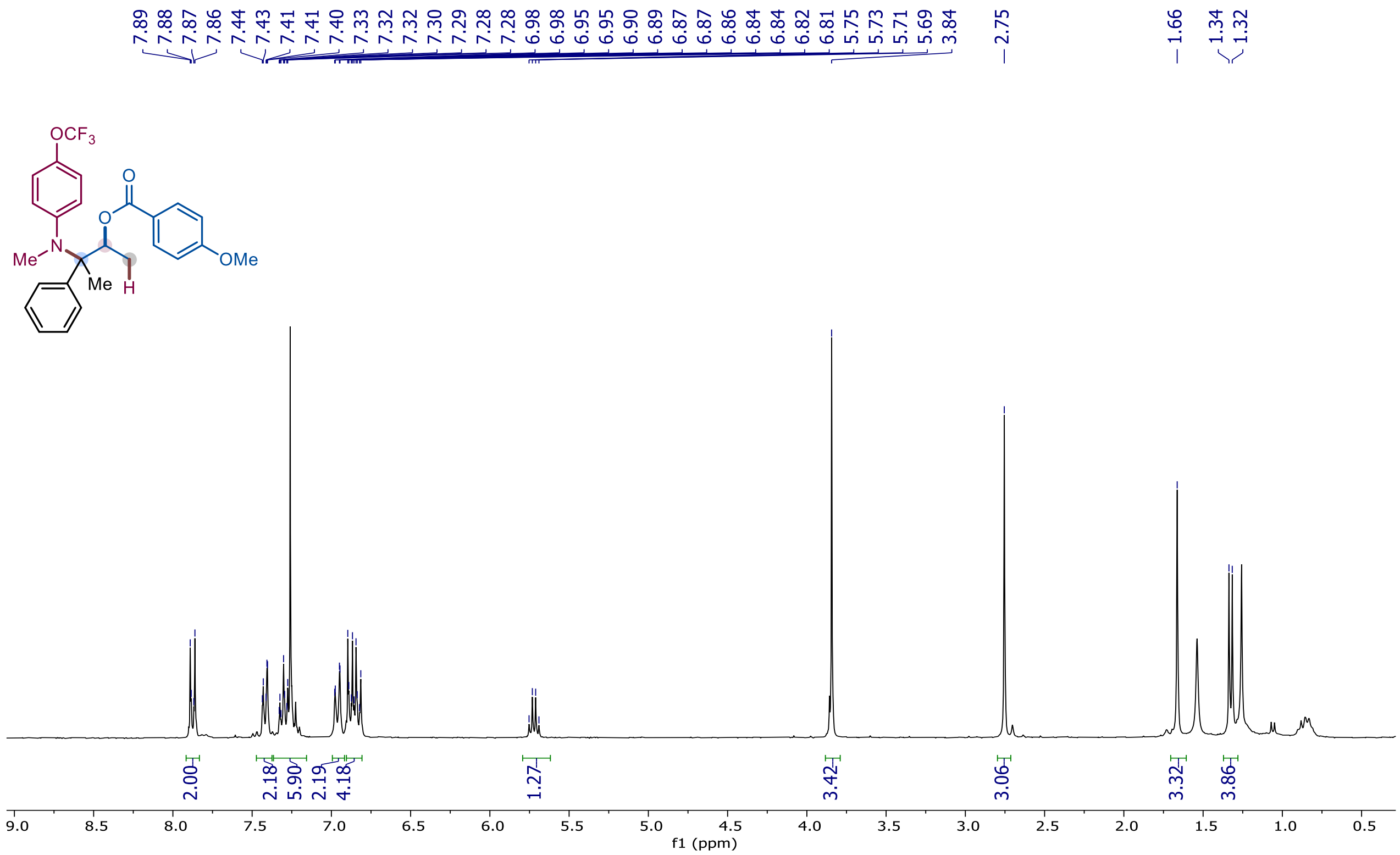

^{13}C NMR (201 MHz, $CDCl_3$, 25 ºC) of 3aas

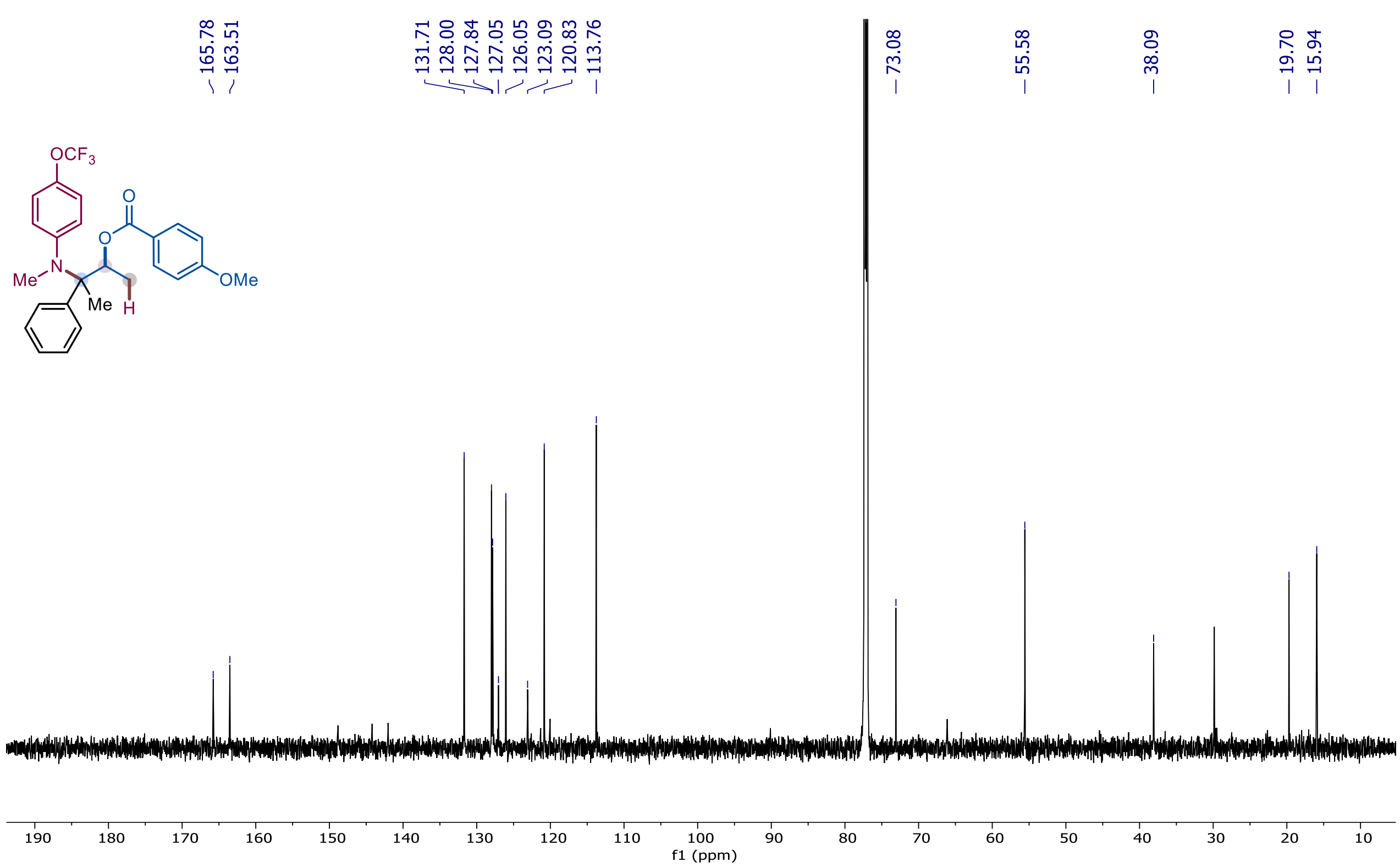

^{19}F NMR (282 MHz, CDCl$_3$, 25 °C) of 3aas

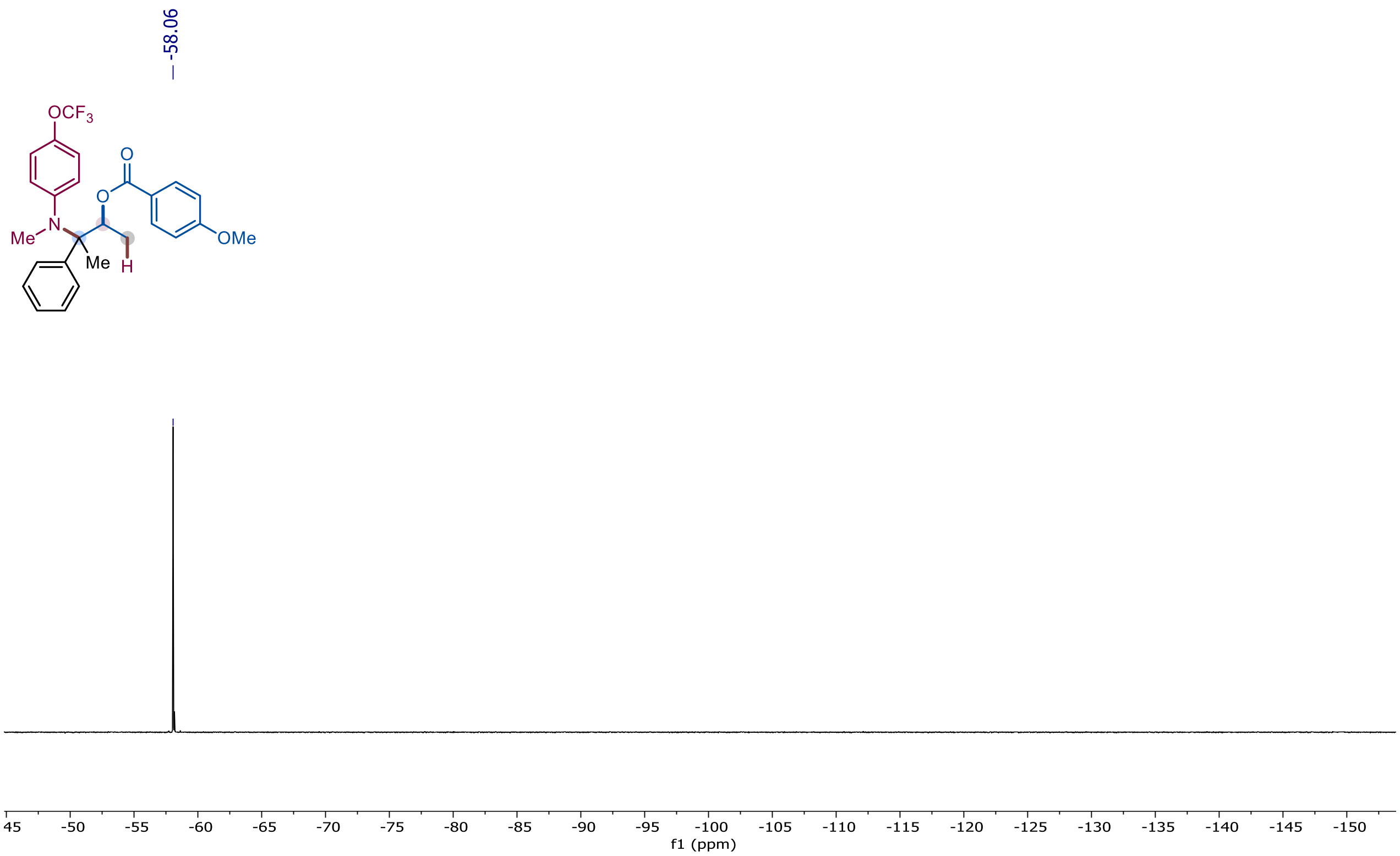

^{1}H NMR (500 MHz, $CDCl_3$, 25 ºC) of 3aat

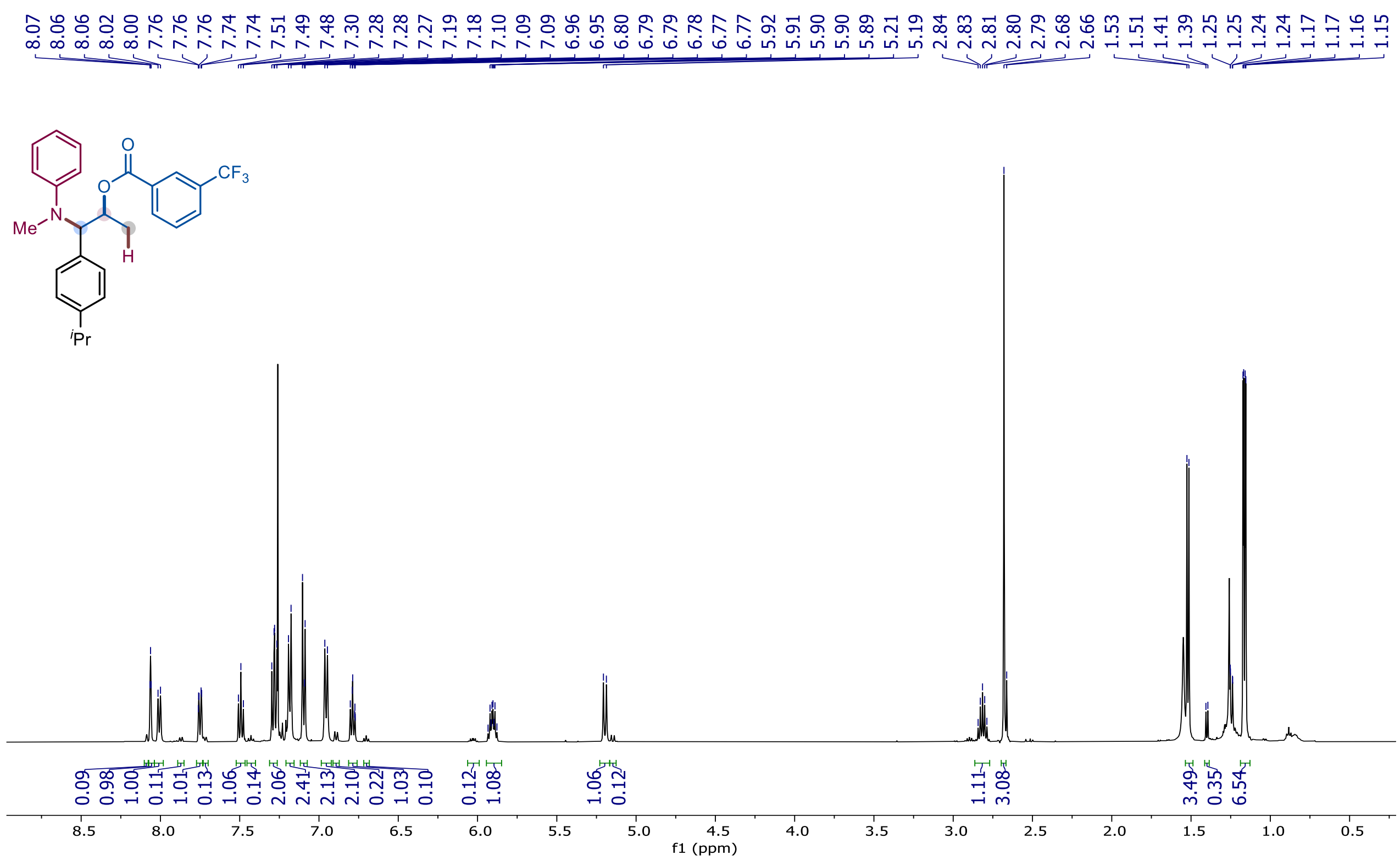

^{13}C NMR (126 MHz, $CDCl_3$, 25 ºC) of 3aat

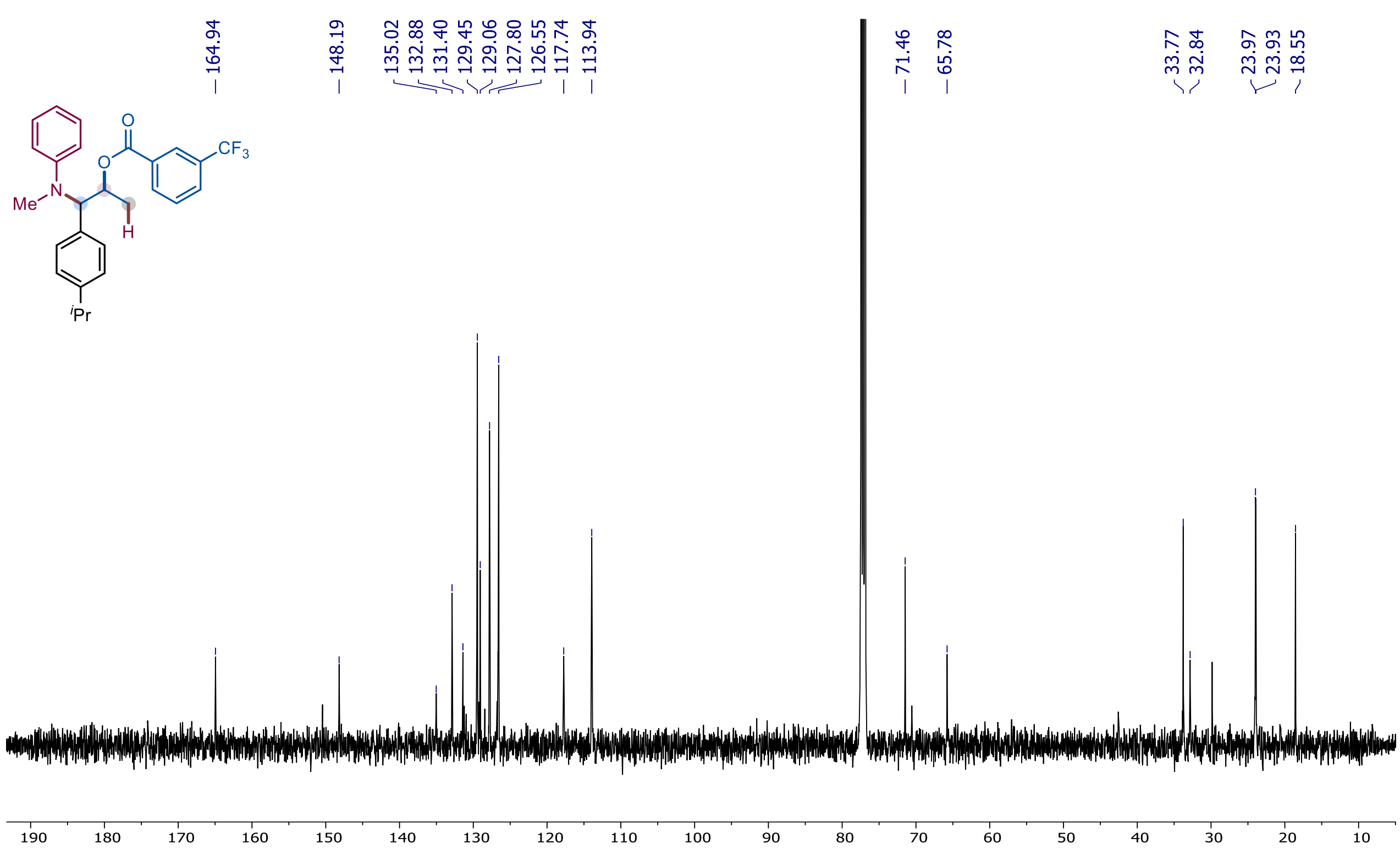

^{19}F NMR (470 MHz, CDCl$_3$, 25 ºC) of 3aat

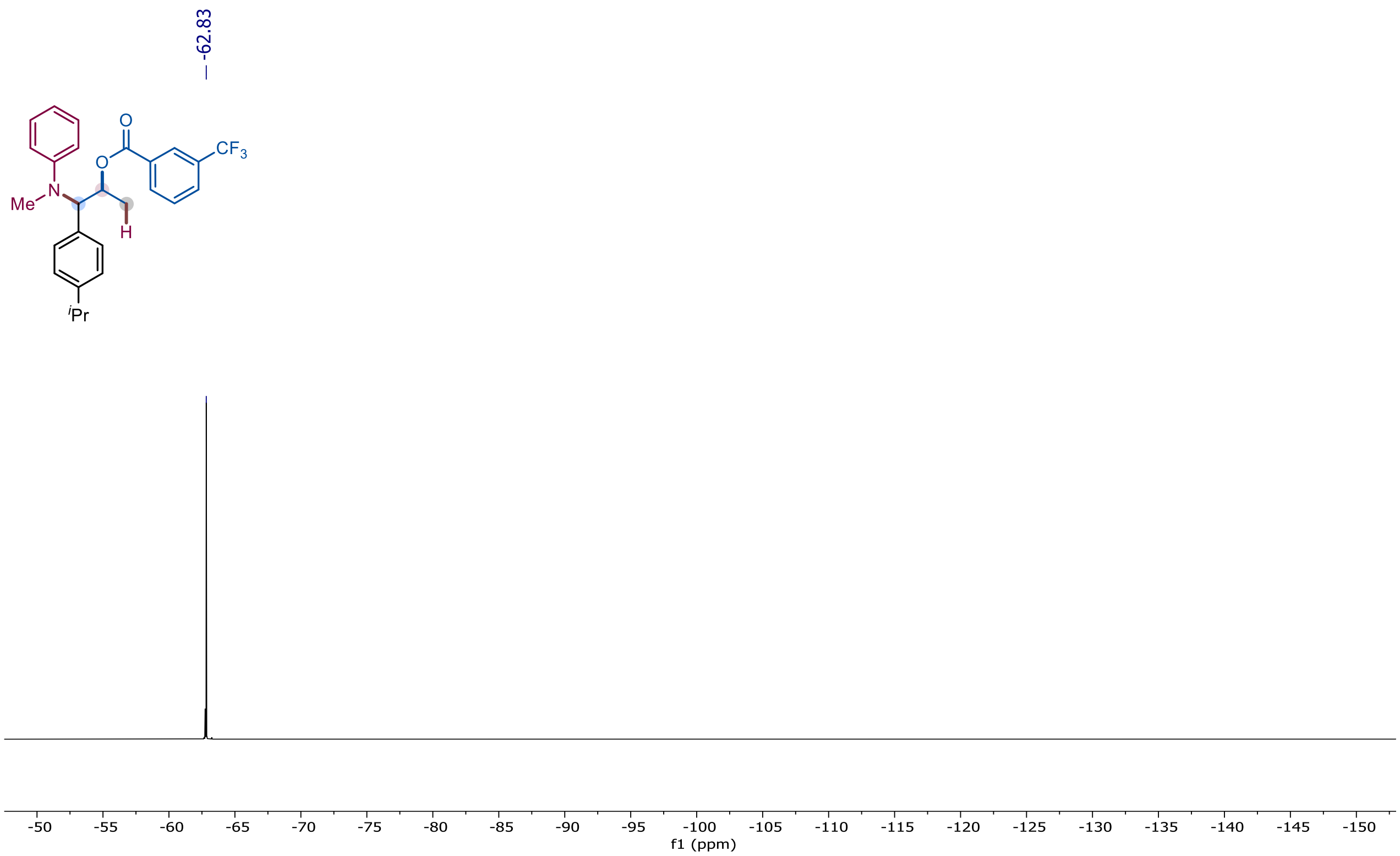

^{1}H NMR (400 MHz, $CDCl_3$, 25 ºC) of 3aau

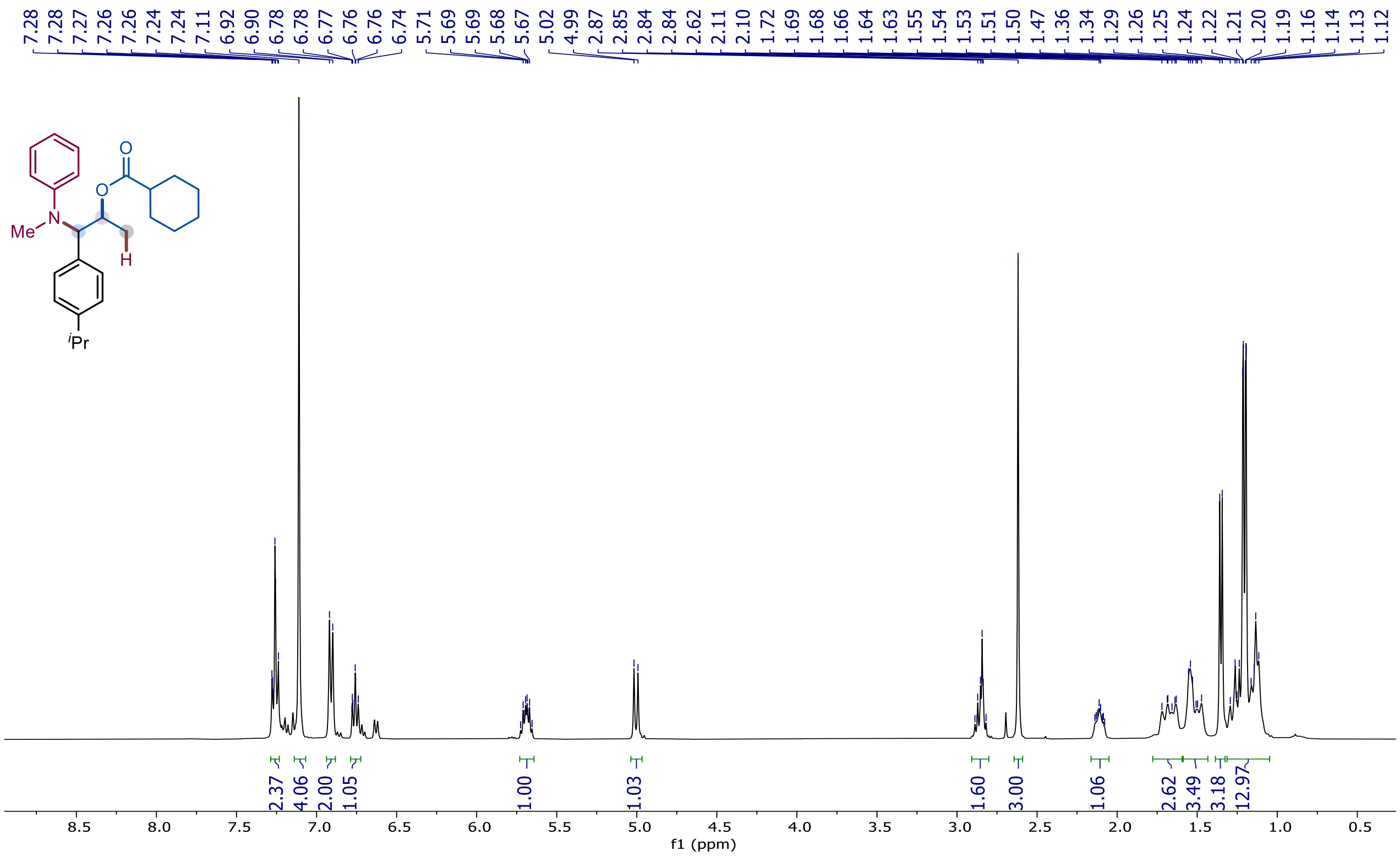

^{13}C NMR (201 MHz, $CDCl_3$, 25 ºC) of 3aau

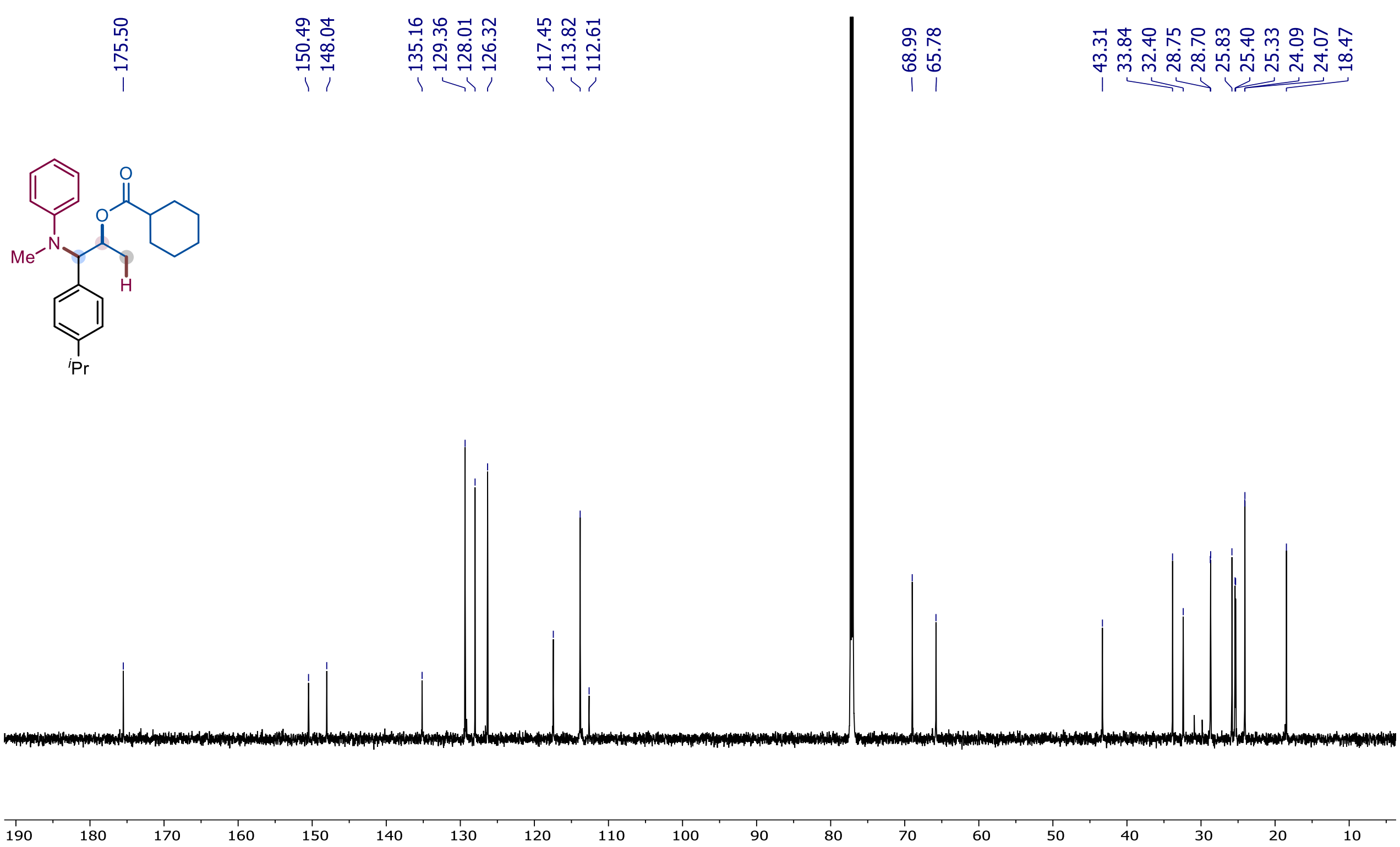

^{1}H NMR (400 MHz, CDCl$_3$, 25 °C) of 3aav

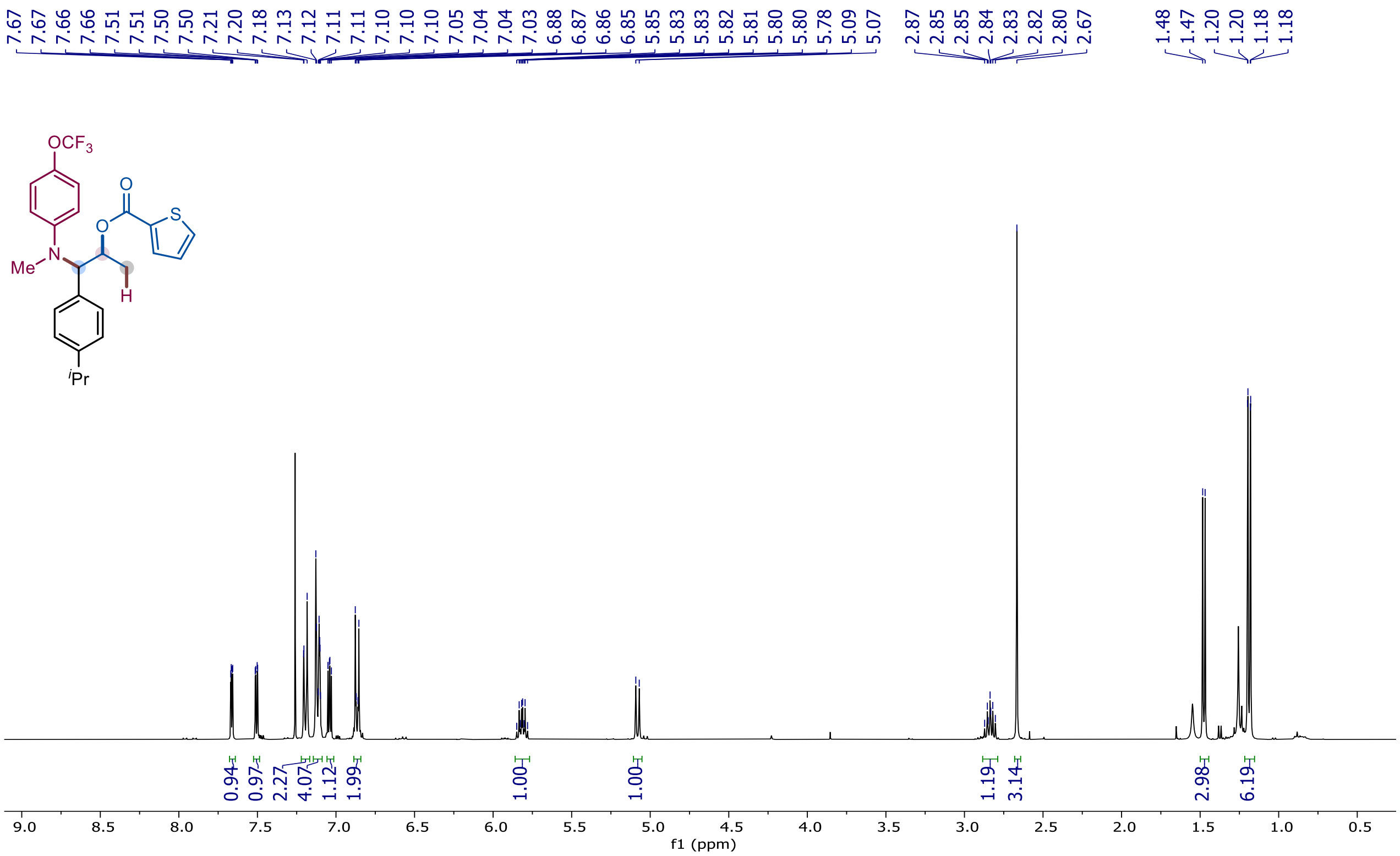

^{13}C NMR (126 MHz, CDCl$_3$, 25 °C) of 3aav

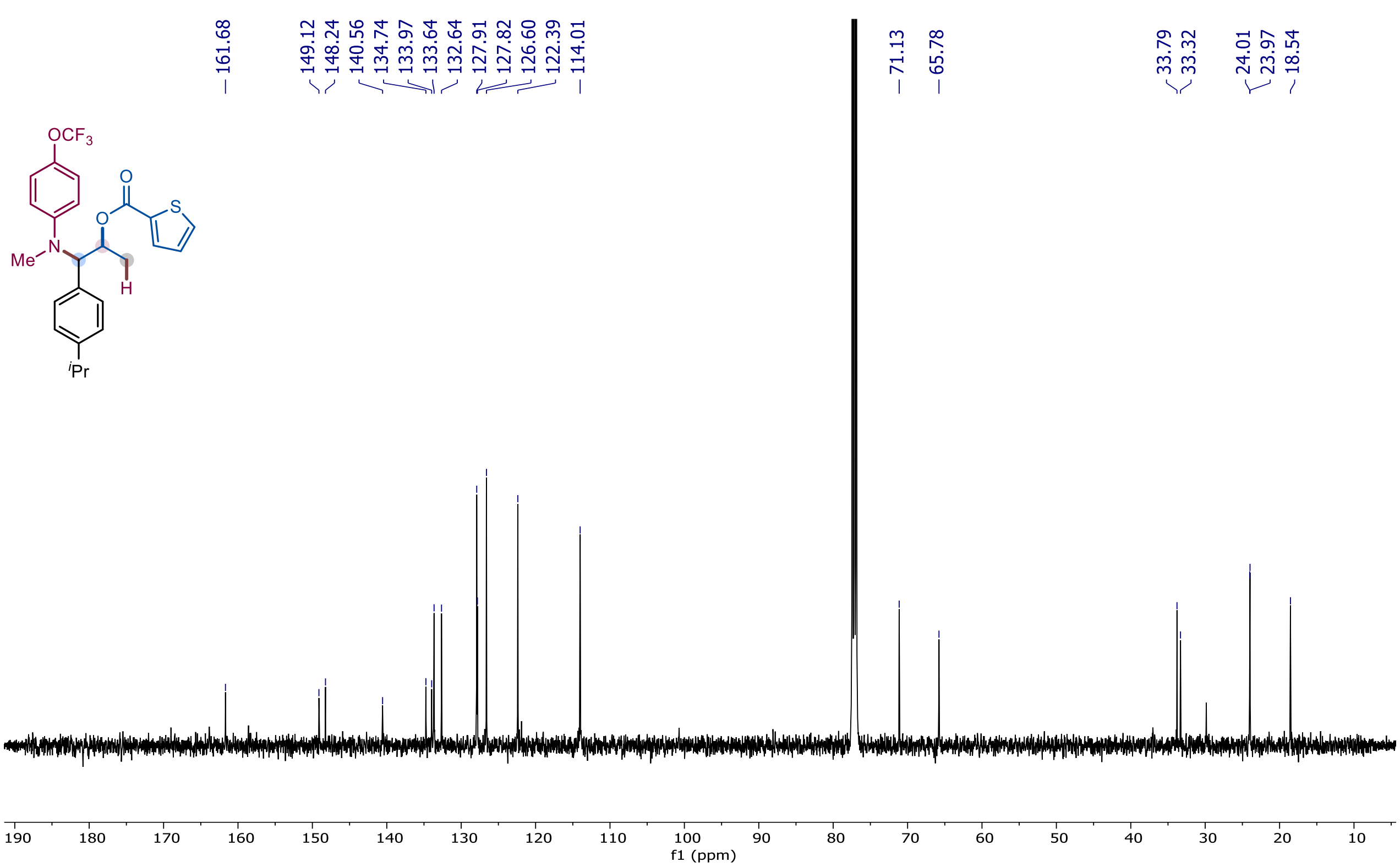

^{19}F NMR (376 MHz, $CDCl_3$, 25 ºC) of 3aav

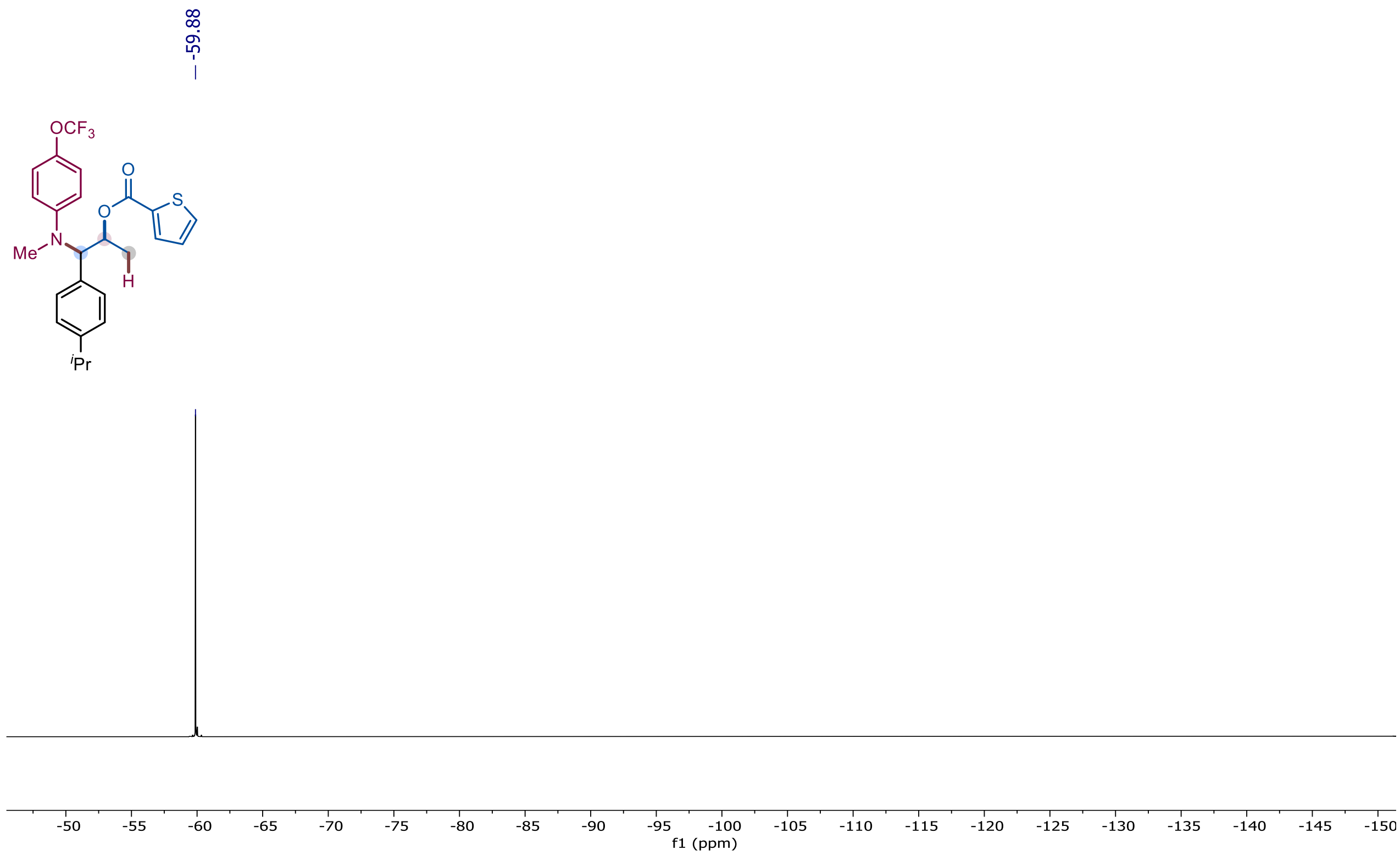

^{1}H NMR (500 MHz, $CDCl_3$, 25 ºC) of 4a

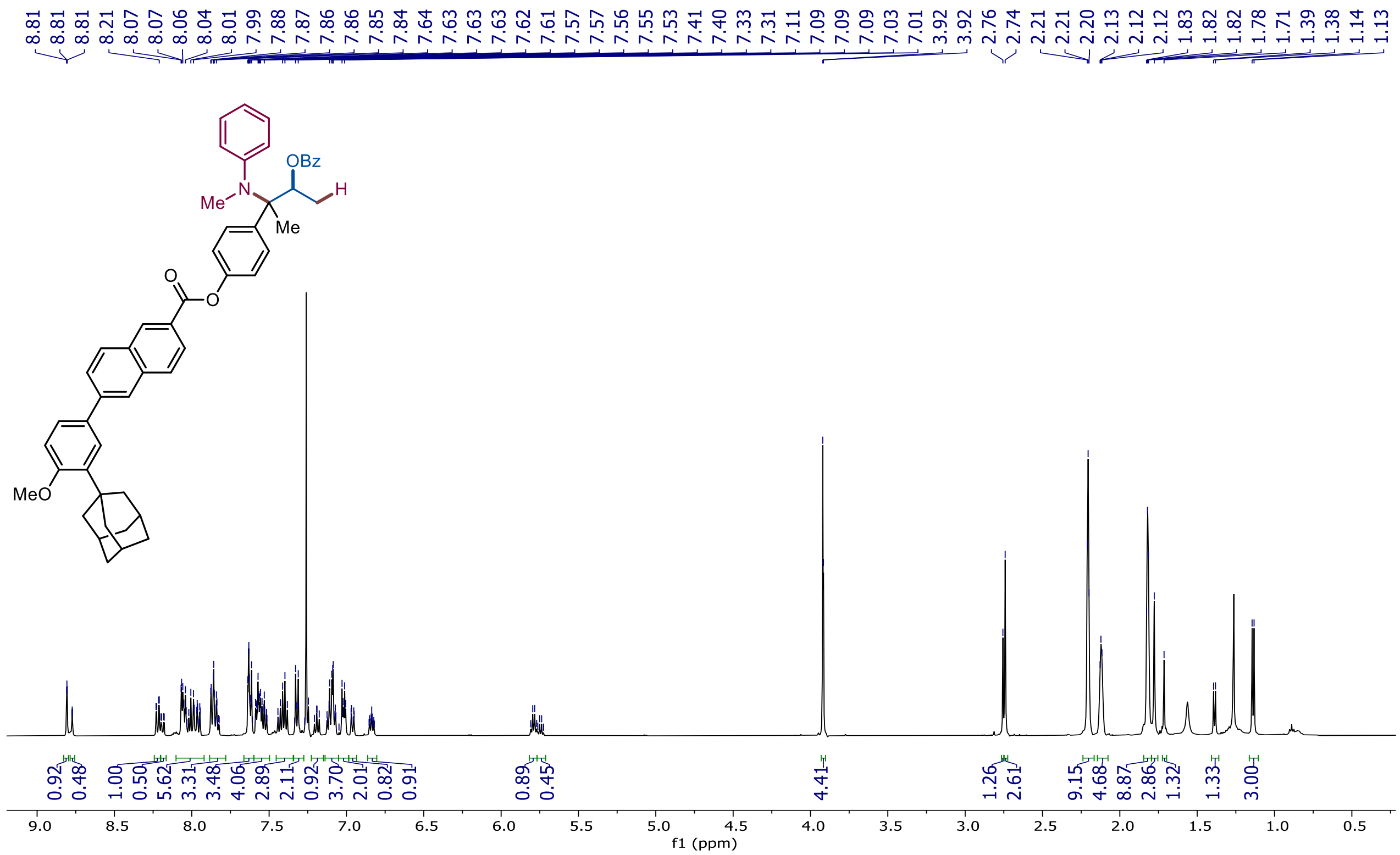

^{13}C NMR (126 MHz, $CDCl_3$, 25 ºC) of4a

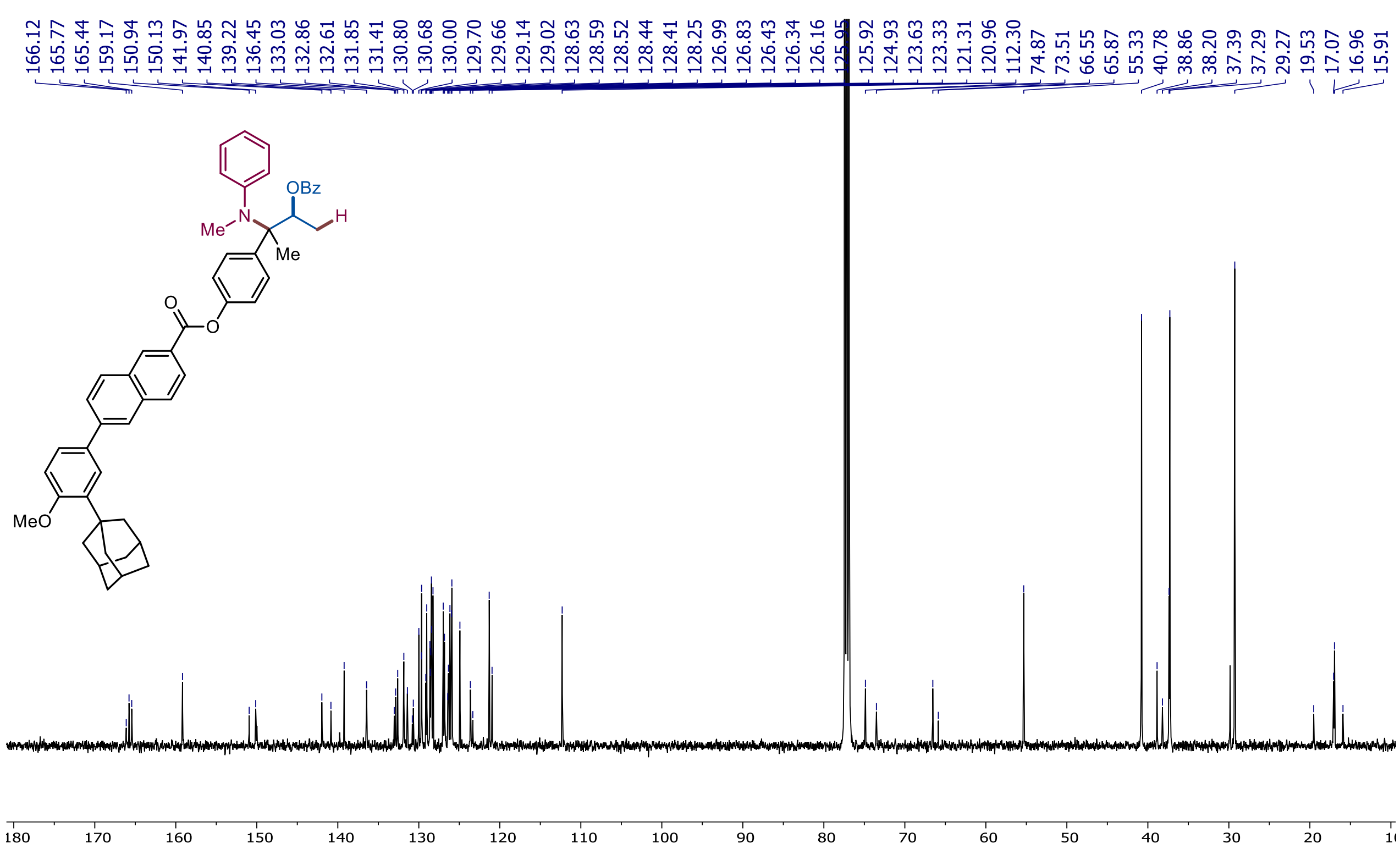

^{1}H NMR (800 MHz, $CDCl_3$, 25 ºC) of 4b

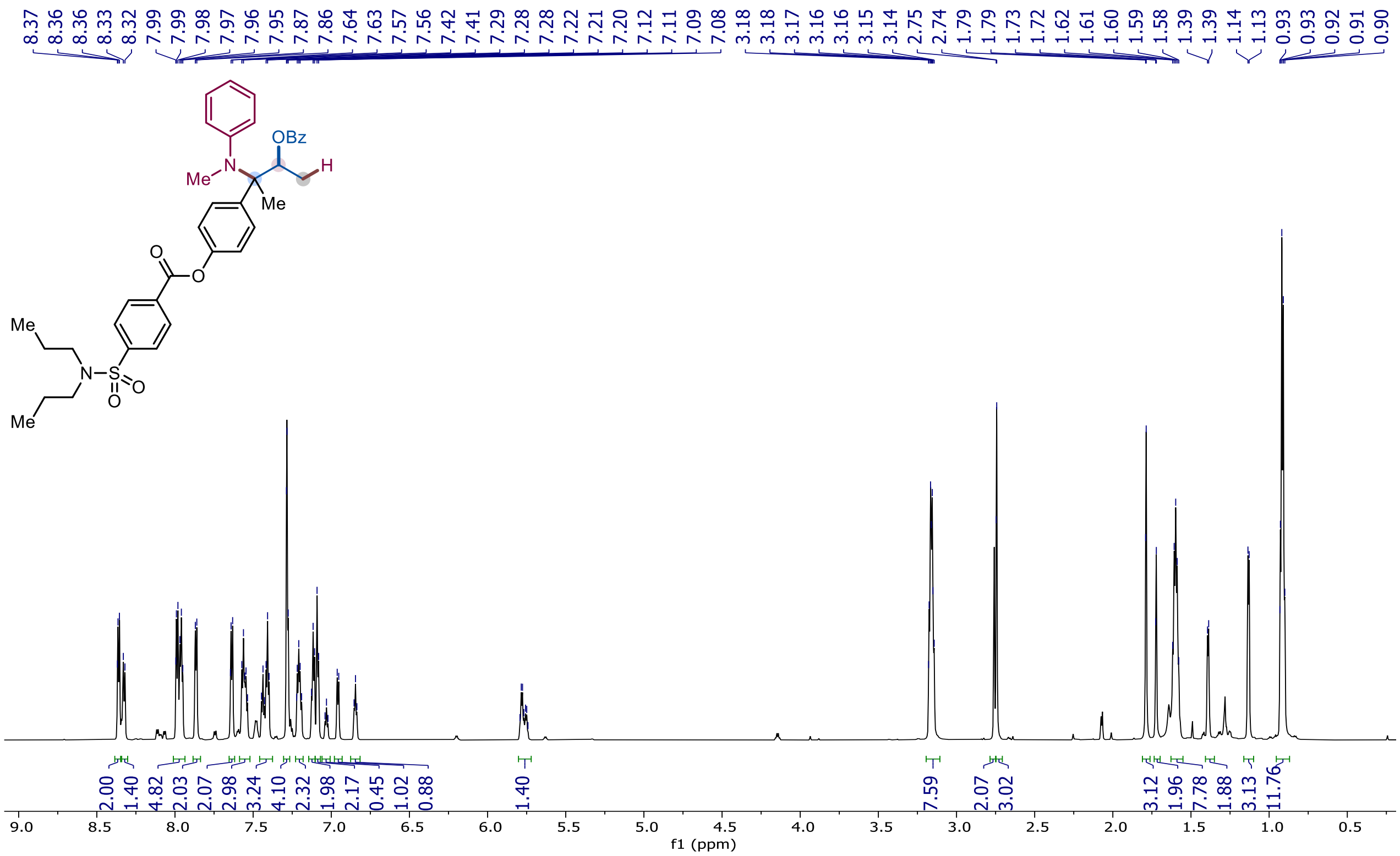

^{13}C NMR (201 MHz, $CDCl_3$, 25 ºC) of 4b

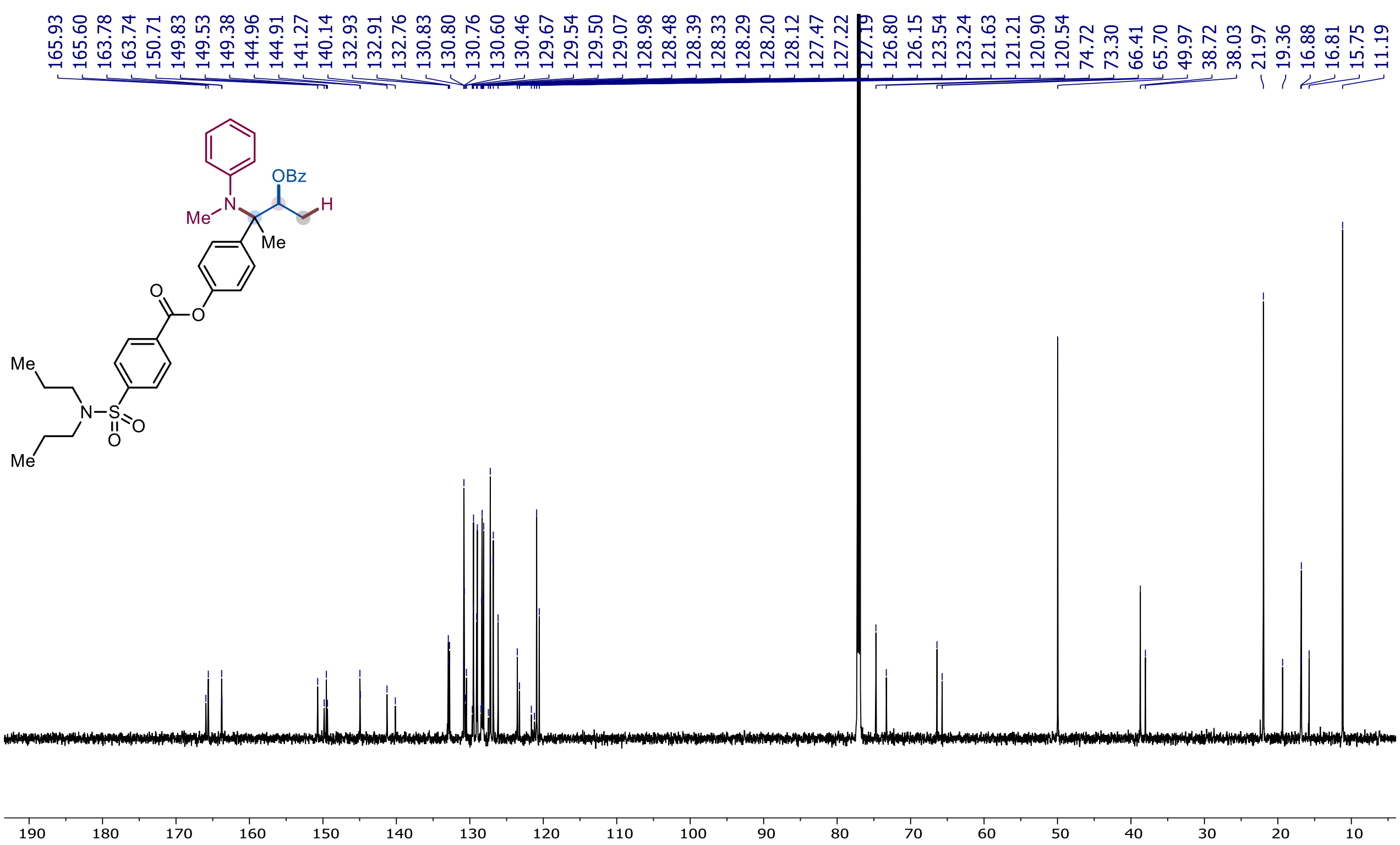

^{1}H NMR (500 MHz, $CDCl_3$, 25 ºC) of 4c

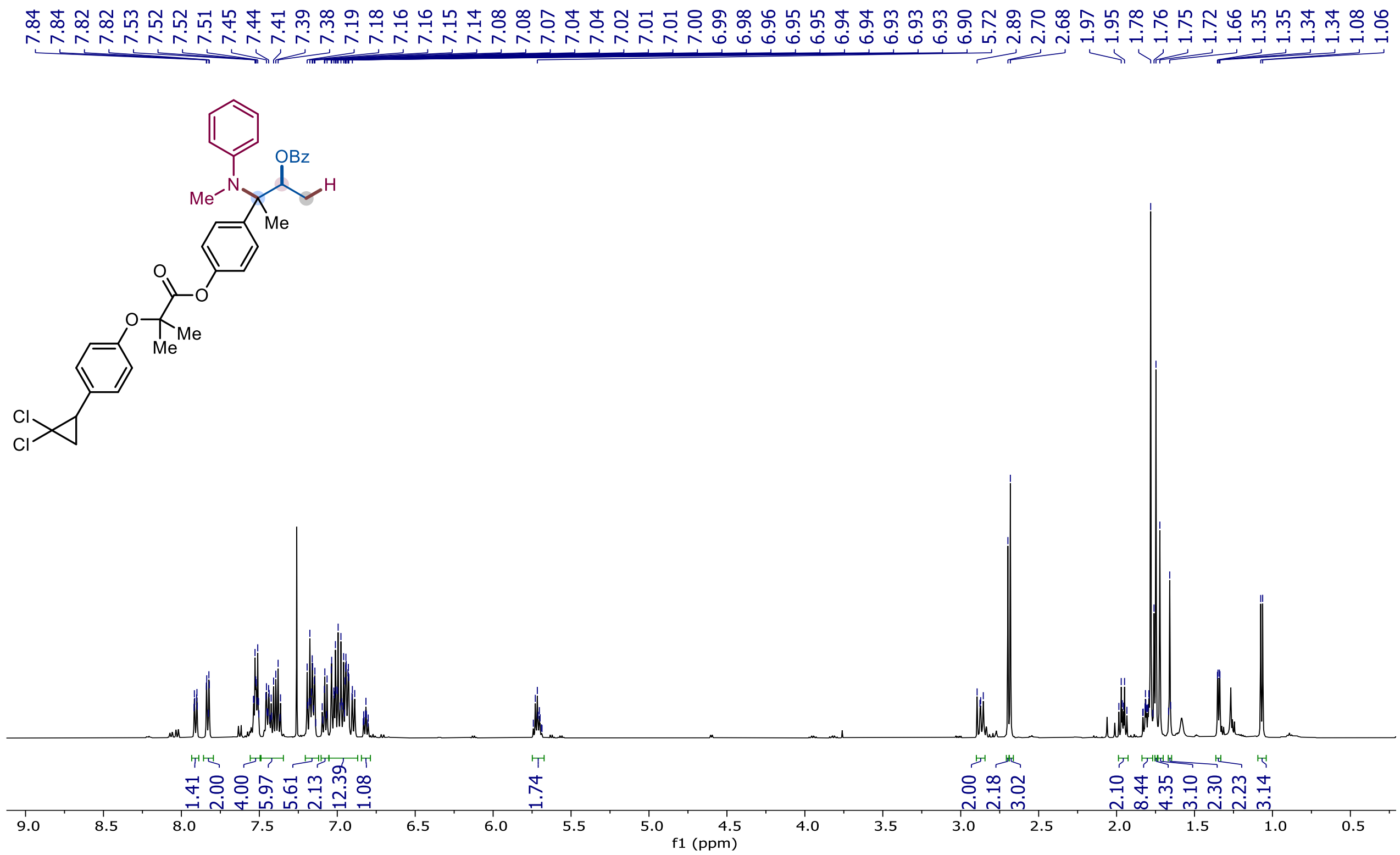

^{13}C NMR (126 MHz, $CDCl_3$, 25 ºC) of 4c

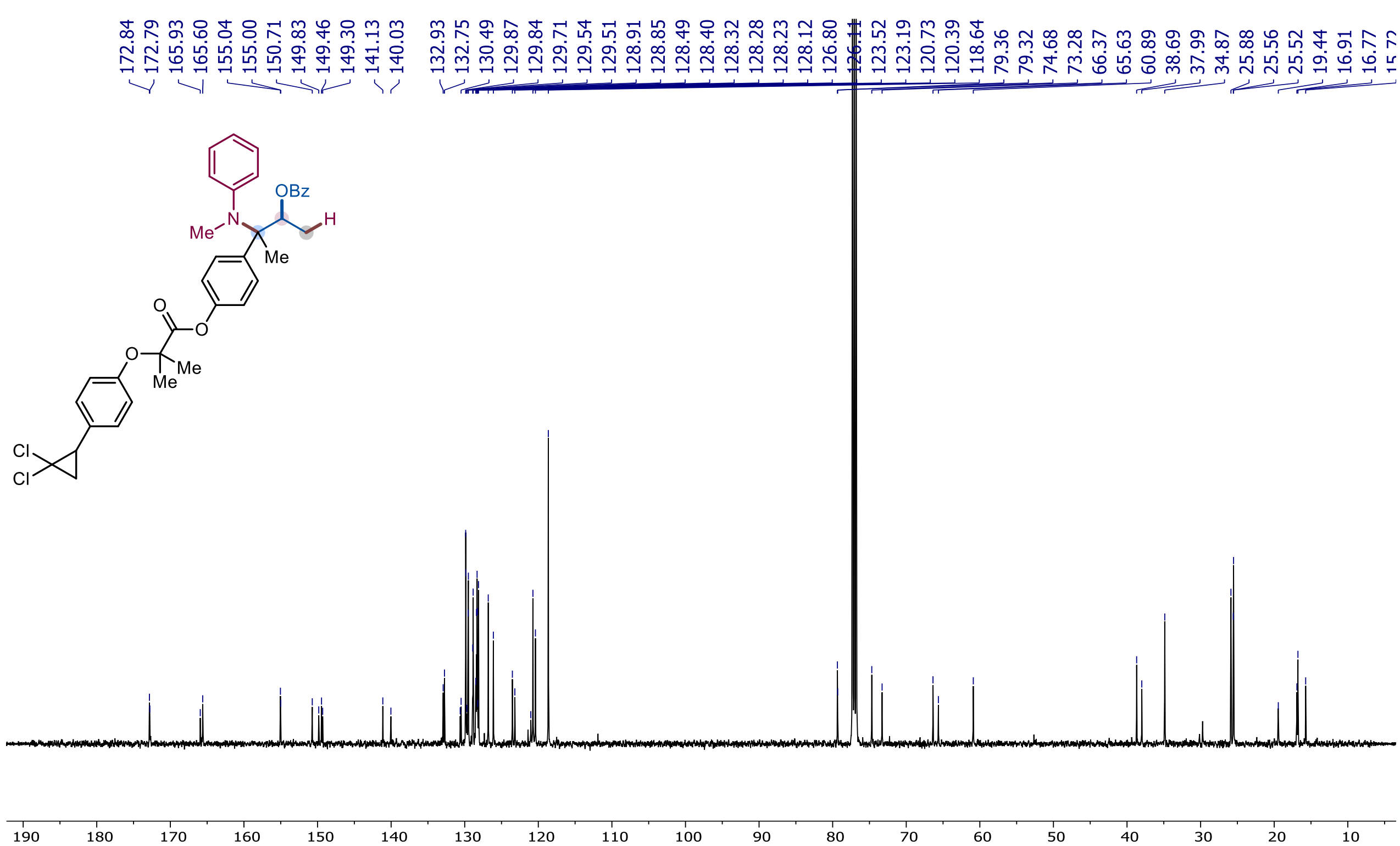

^{1}H NMR (800 MHz, $CDCl_3$, 25 ºC) of 4d

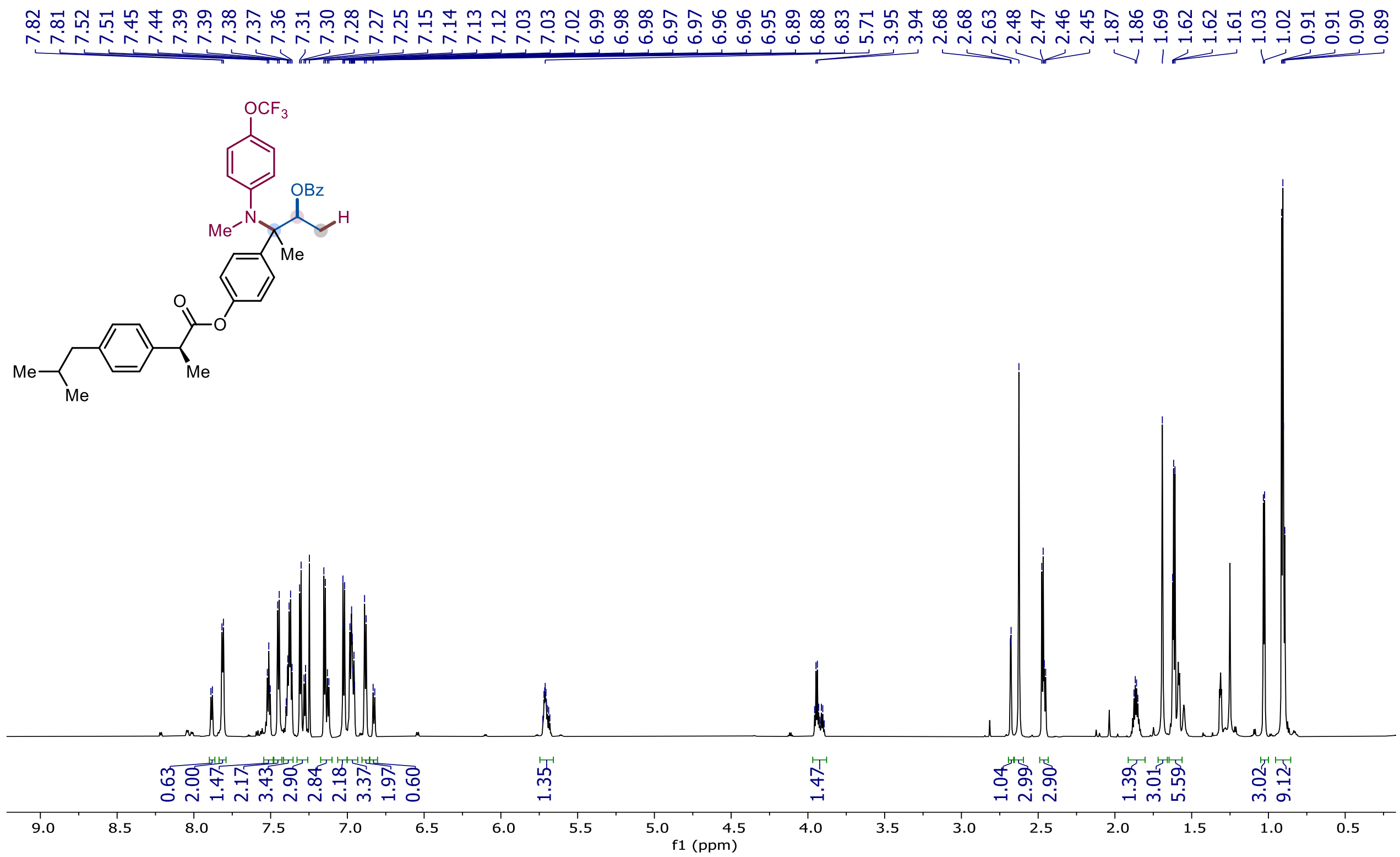

^{13}C NMR (201 MHz, $CDCl_3$, 25 ºC) of 4d

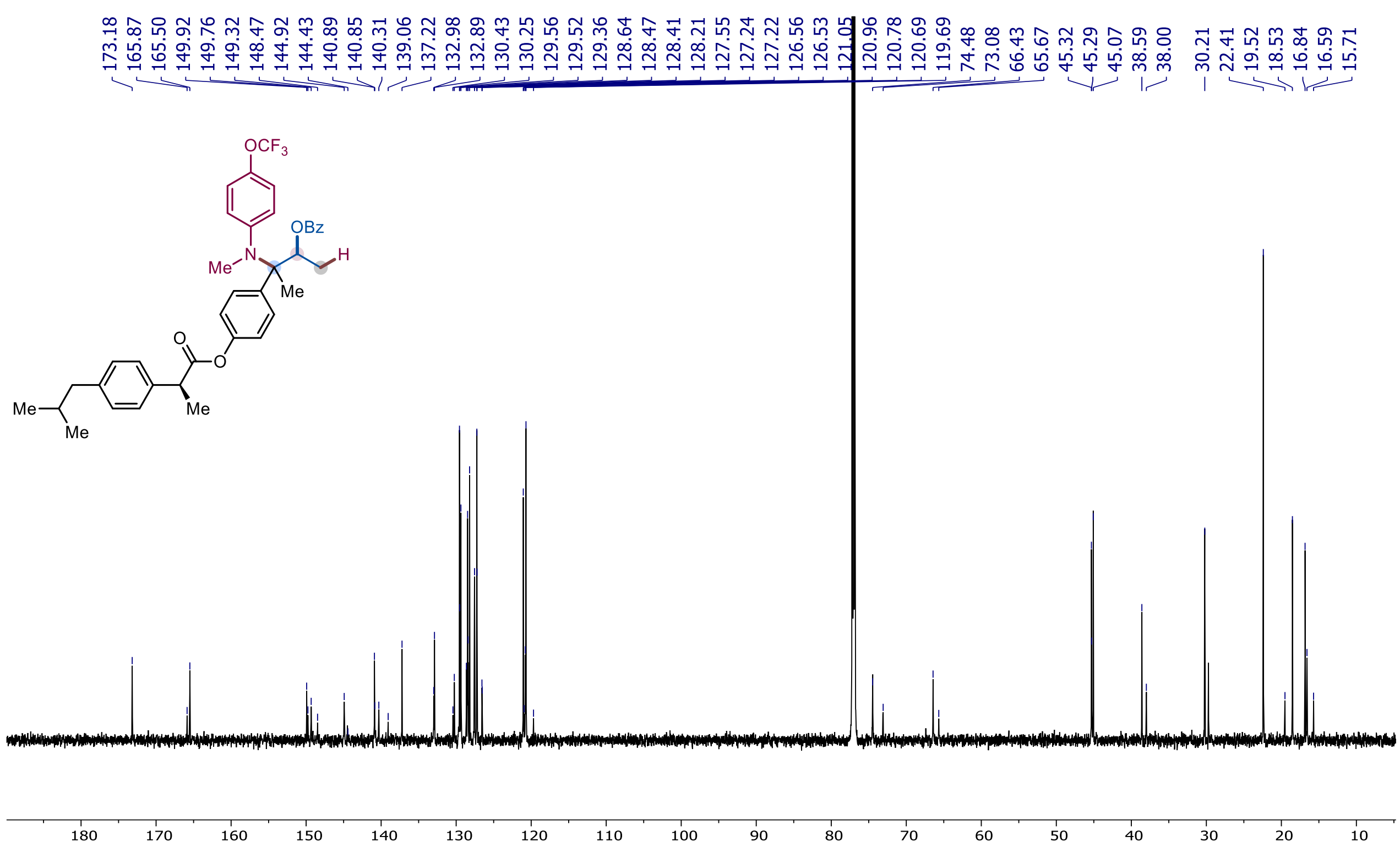

^{19}F NMR (470 MHz, $CDCl_3$, 25 °C) of 4d

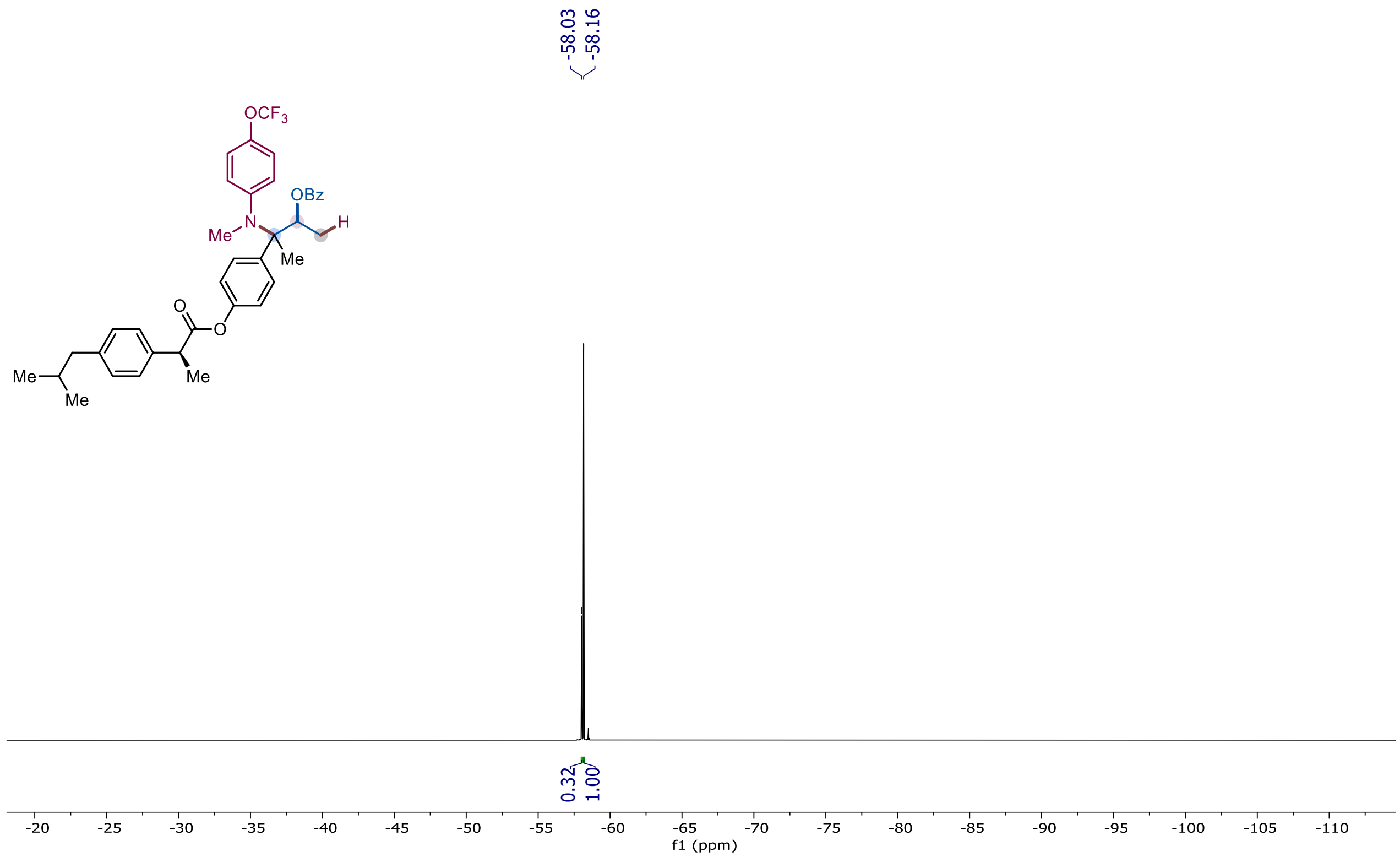

^{1}H NMR (500 MHz, $CDCl_3$, 25 ºC) of 4e

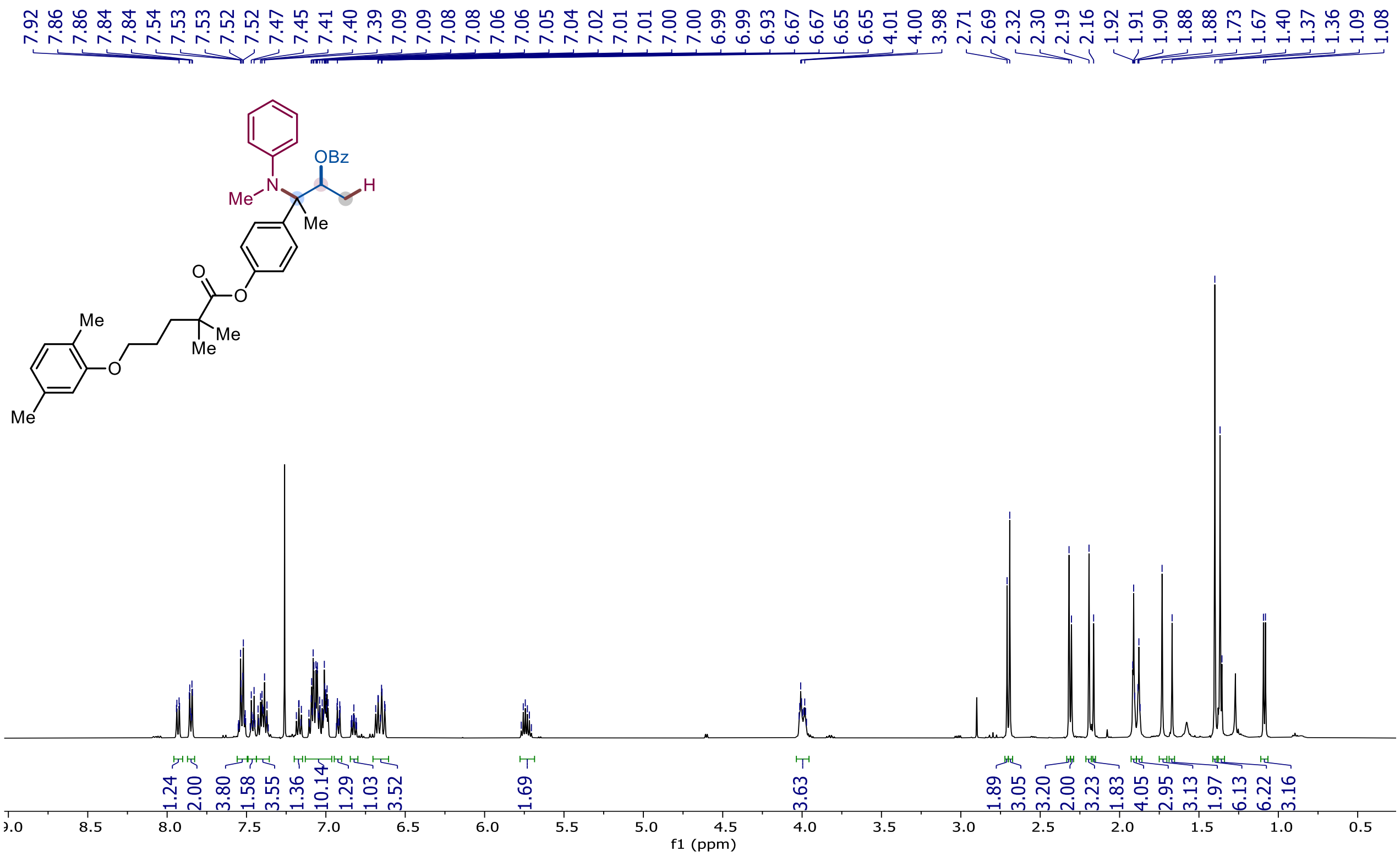

^{13}C NMR (126 MHz, $CDCl_3$, 25 ºC) of 4e

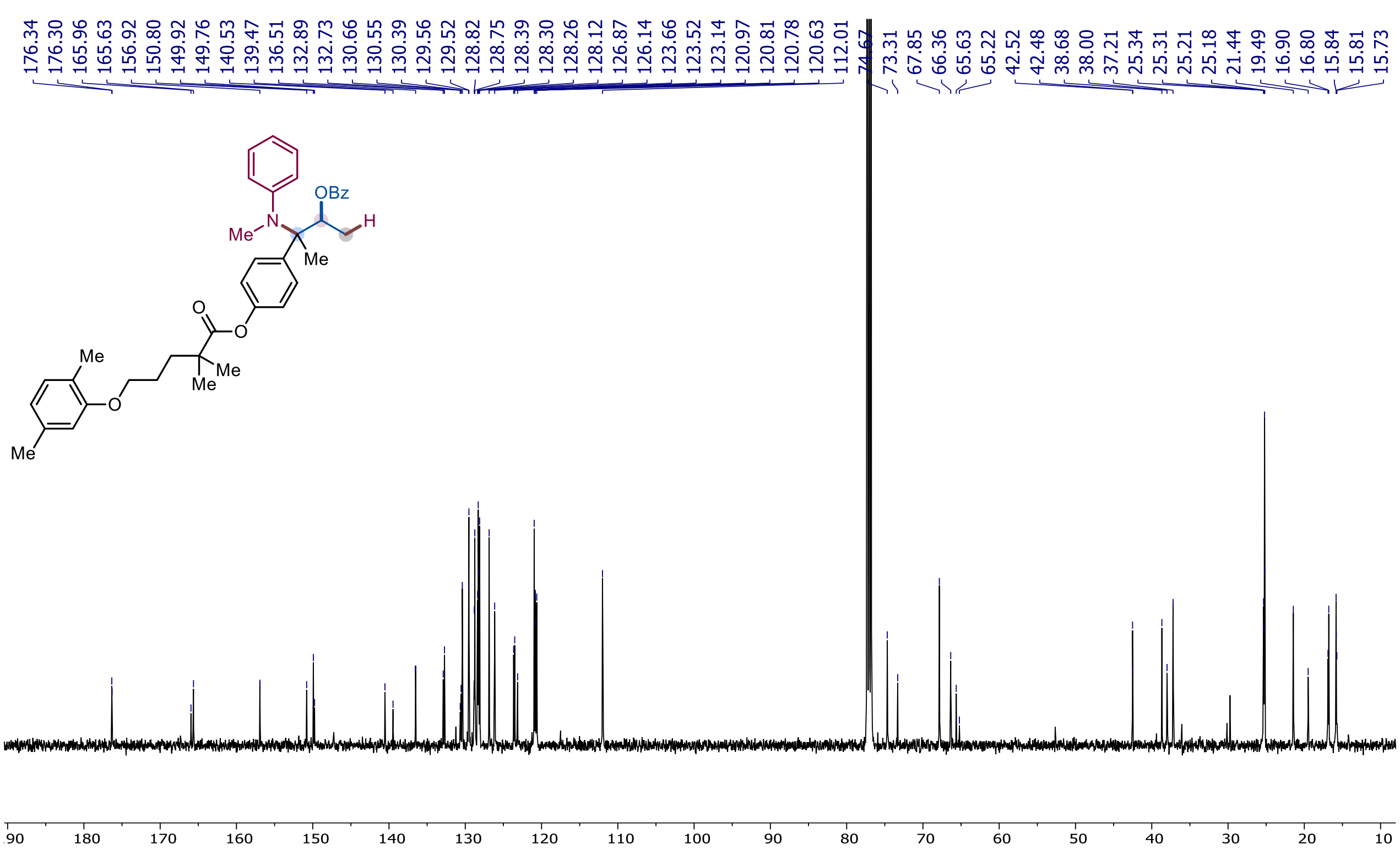

^{1}H NMR (400 MHz, $CDCl_3$, 25 ºC) of 4f

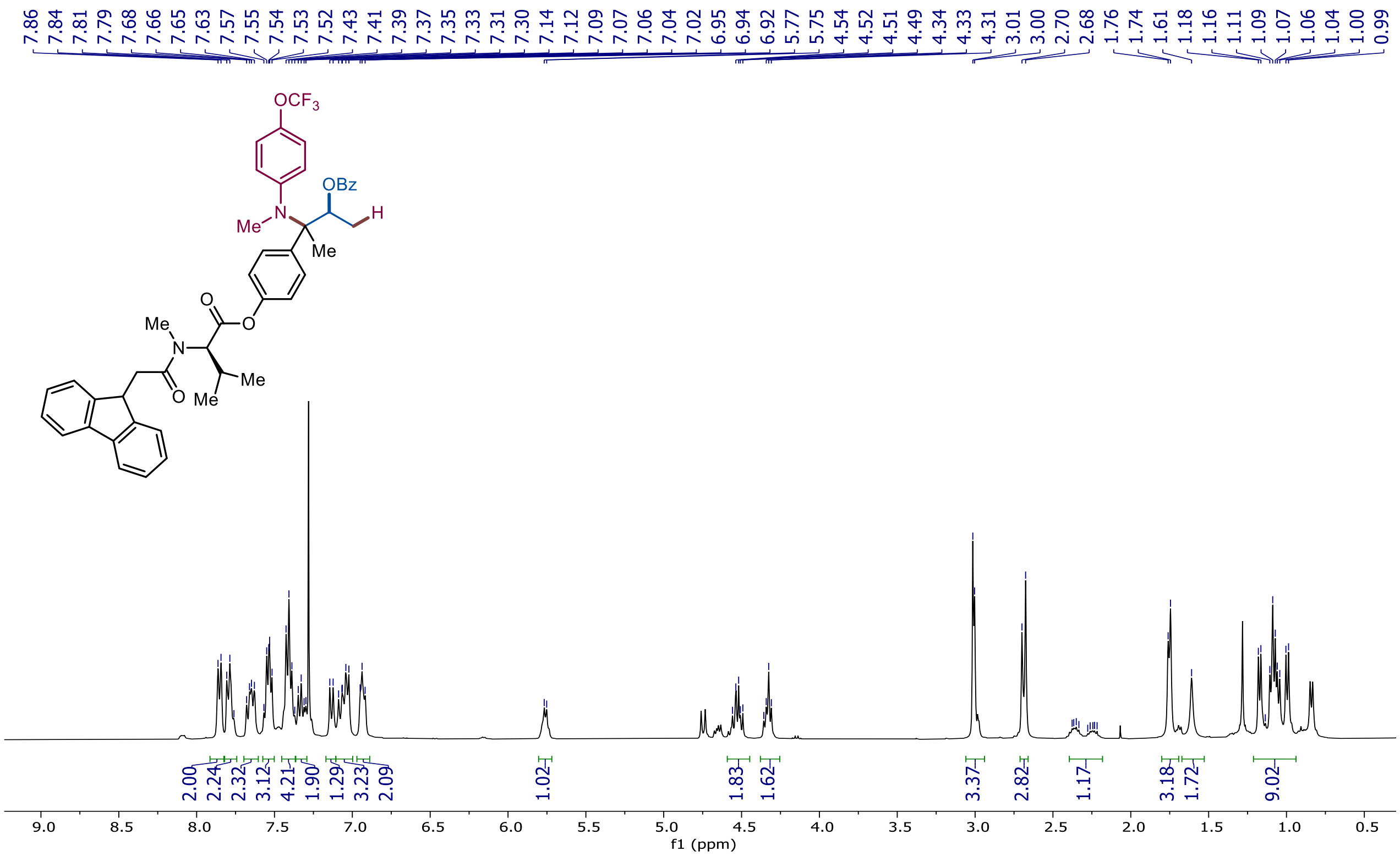

^{13}C NMR (201 MHz, $CDCl_3$, 25 ºC) of 4f

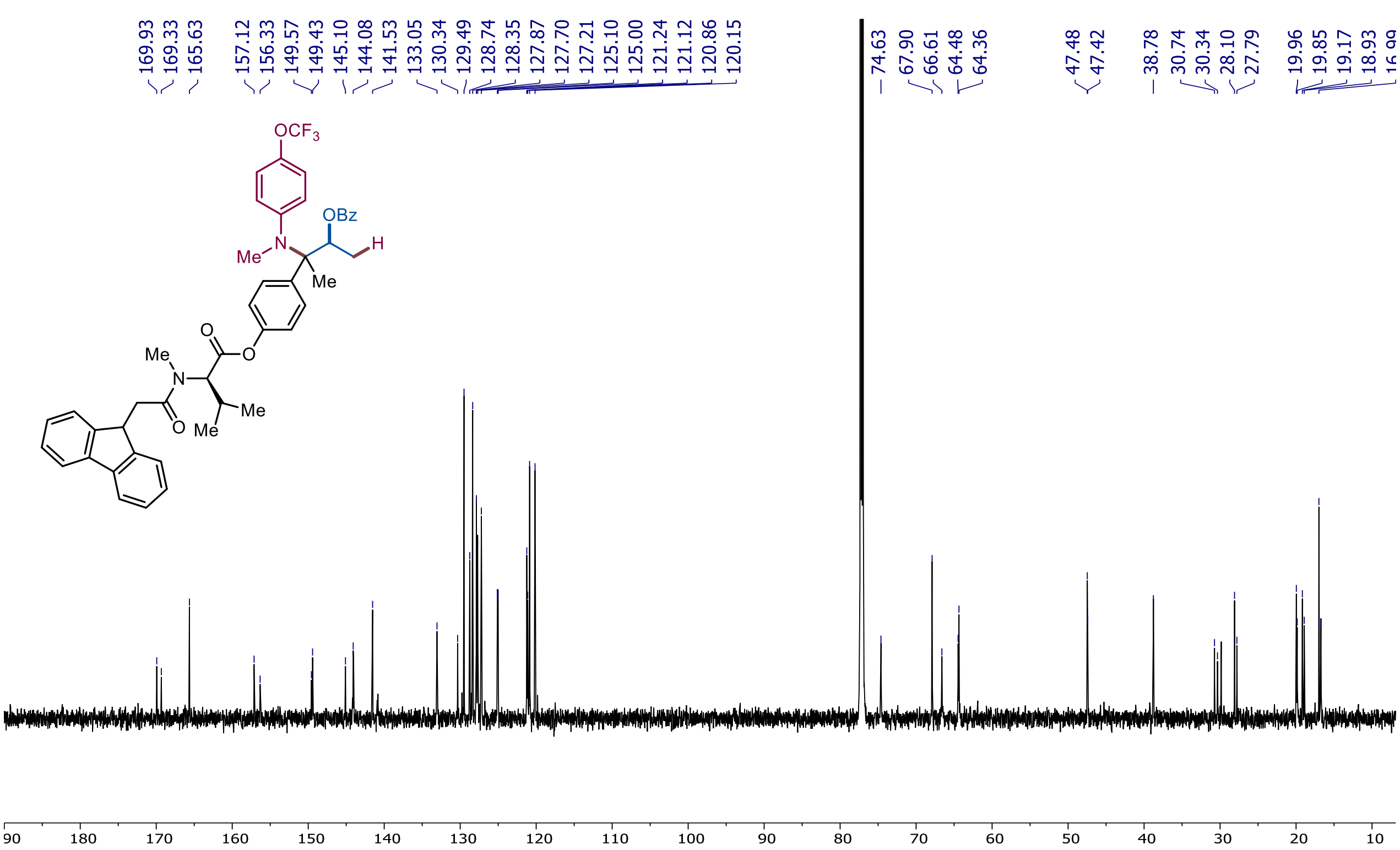

^{19}F NMR (376 MHz, CDCl$_3$, 25 °C) of 4f

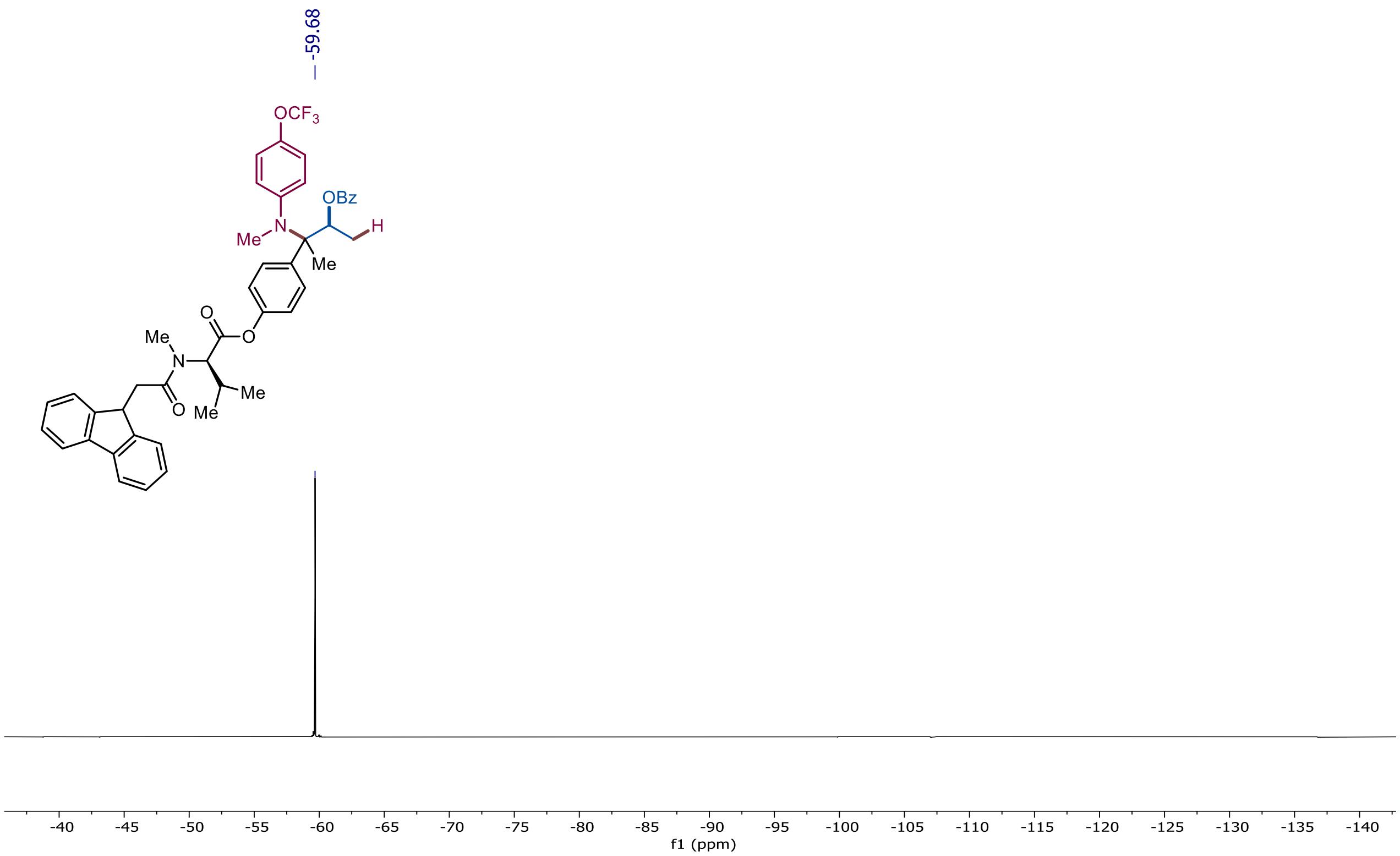

^{1}H NMR (400 MHz, $CDCl_3$, 25 ºC) of 4g

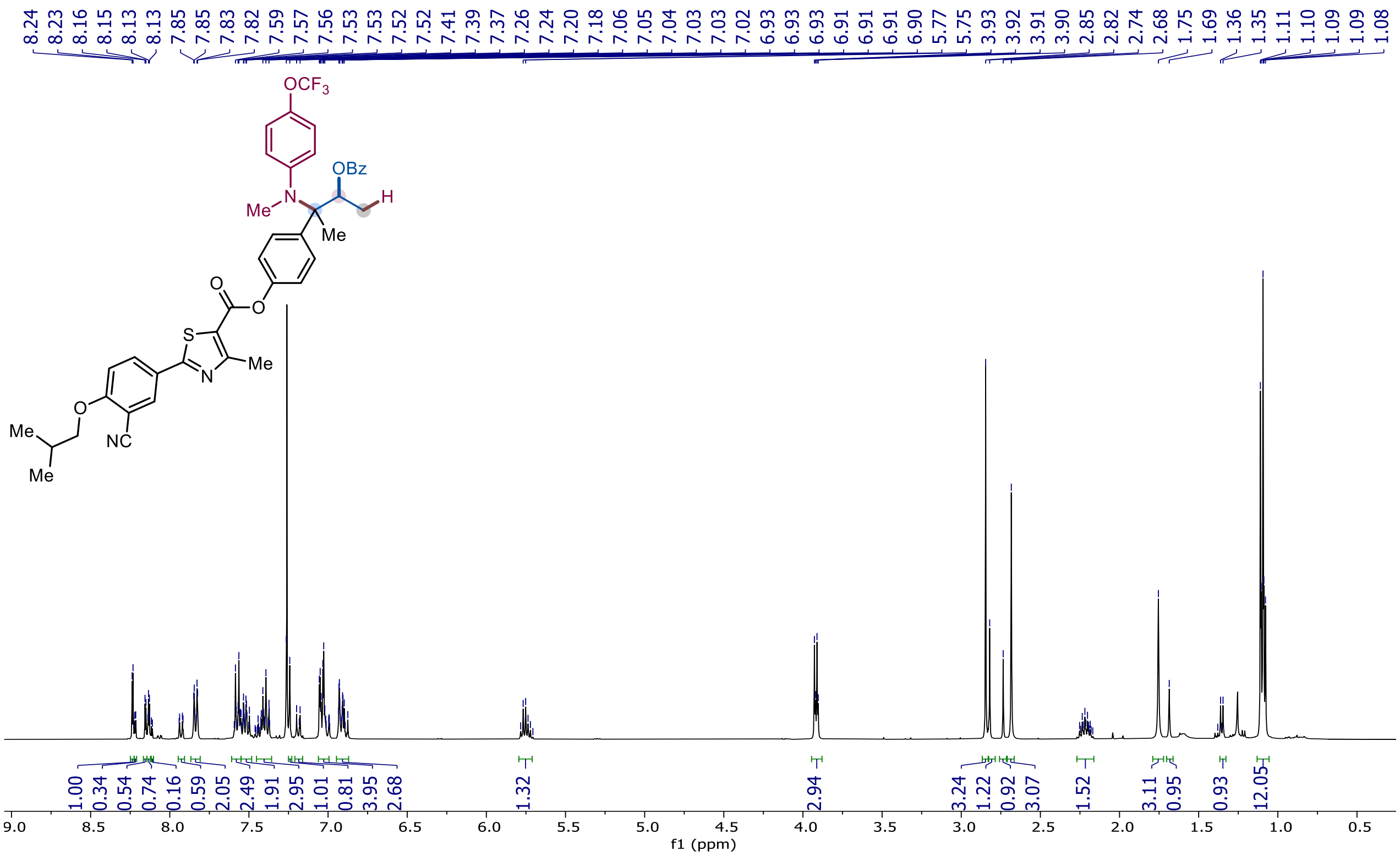

^{13}C NMR (126 MHz, $CDCl_3$, 25 ºC) of 4g

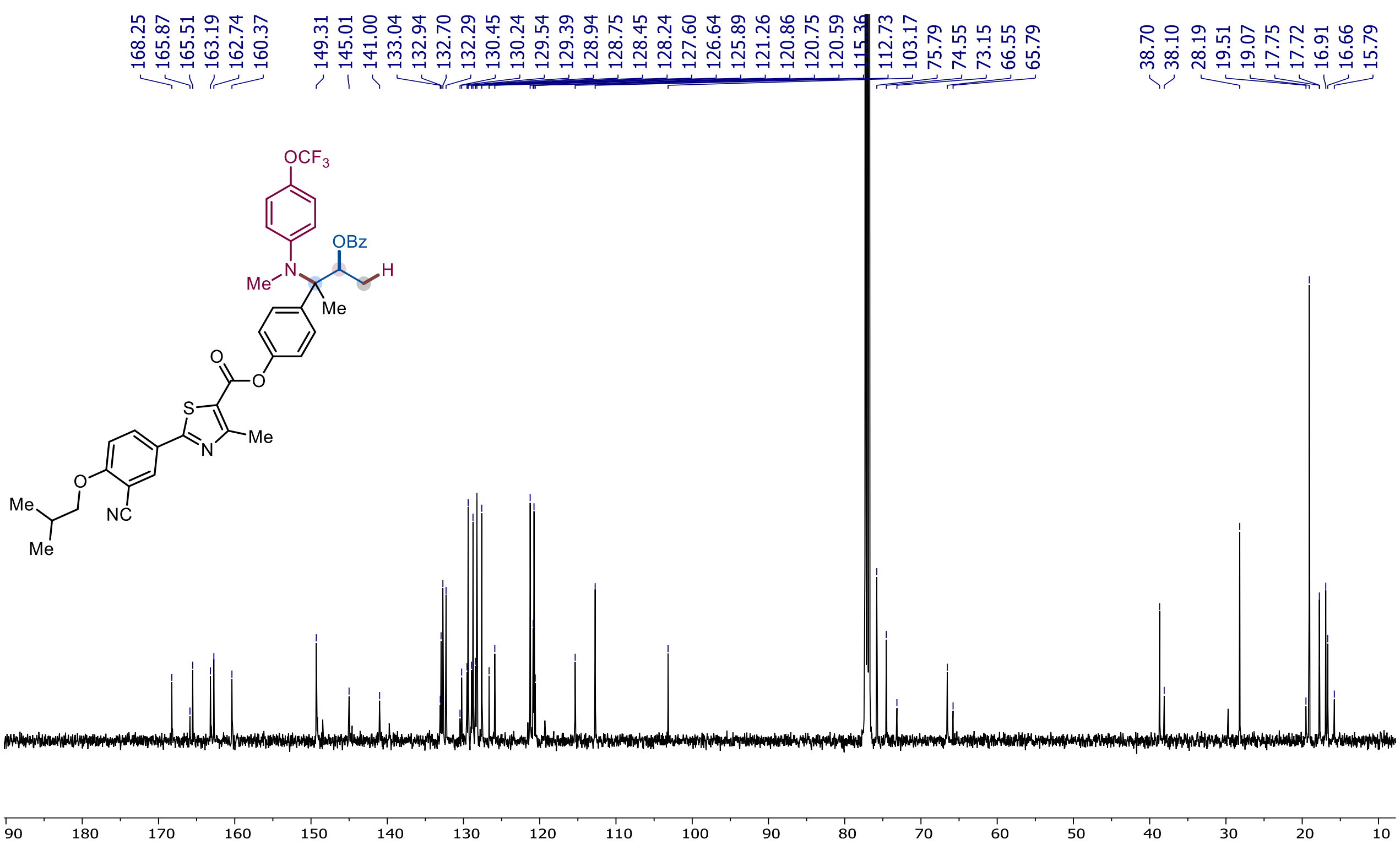

^{19}F NMR (376 MHz, CDCl$_3$, 25 °C) of 4g

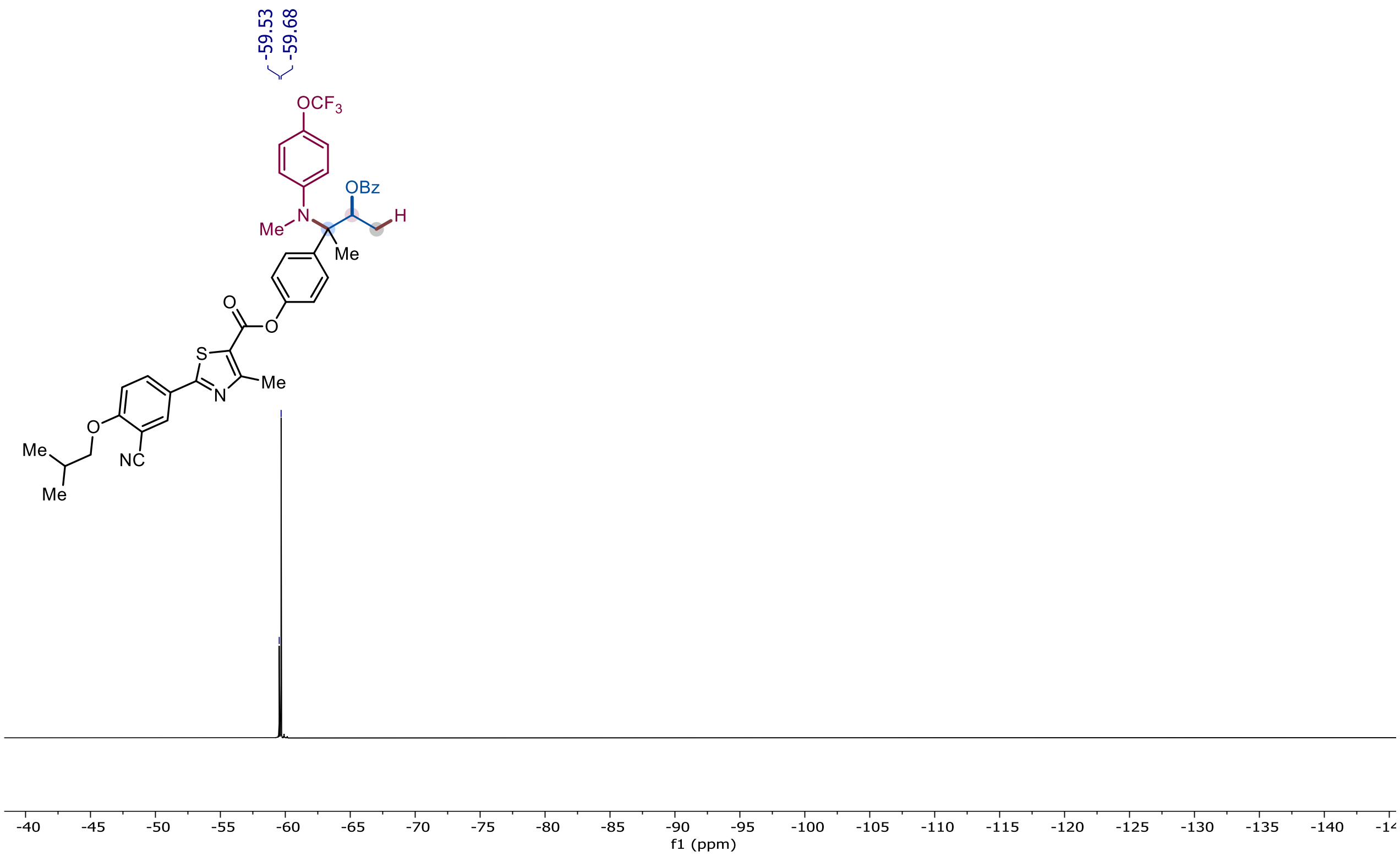

^{1}H NMR (400 MHz, $CDCl_3$, 25 ºC) of 4h

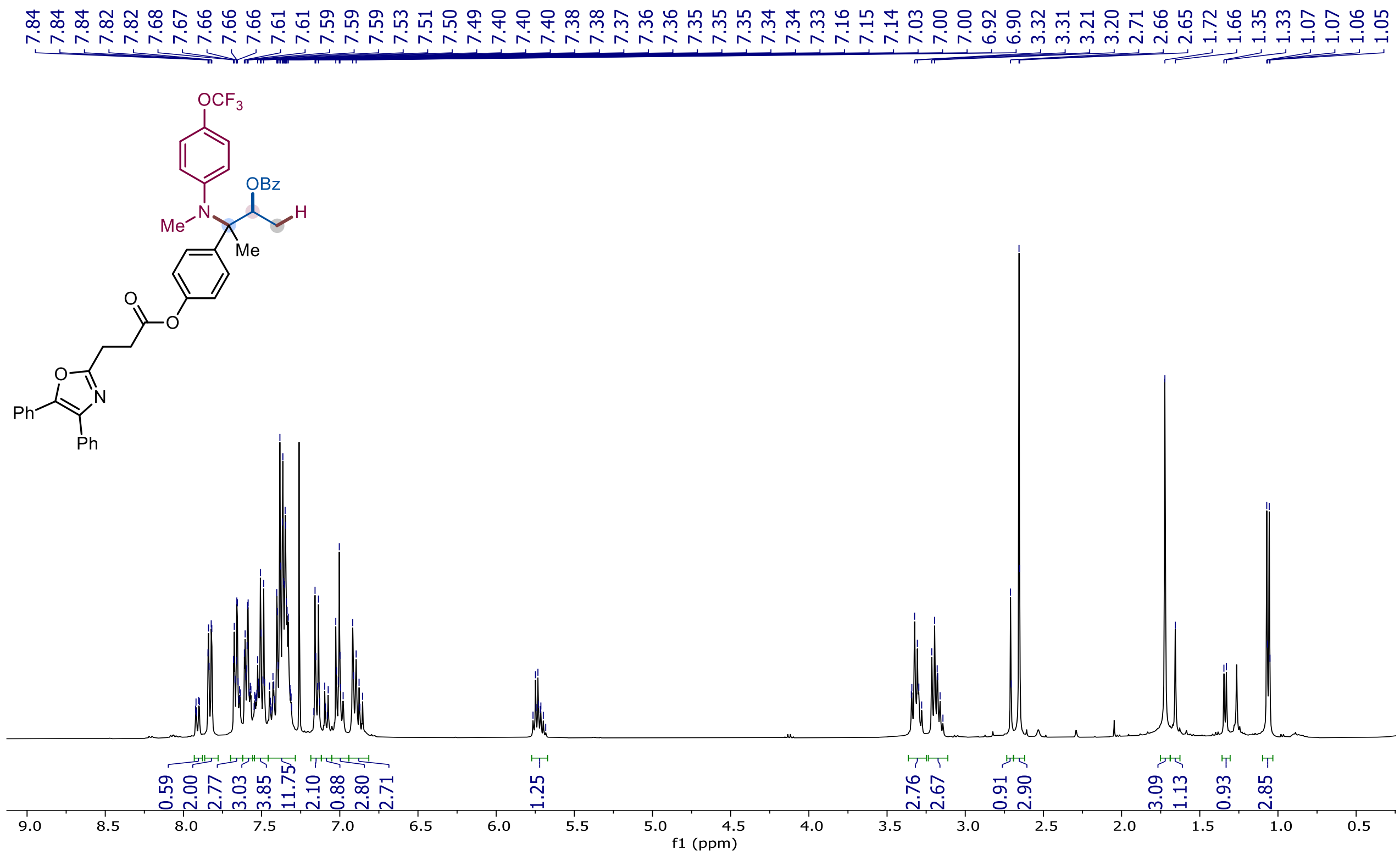

^{13}C NMR (201 MHz, $CDCl_3$, 25 ºC) of 4h

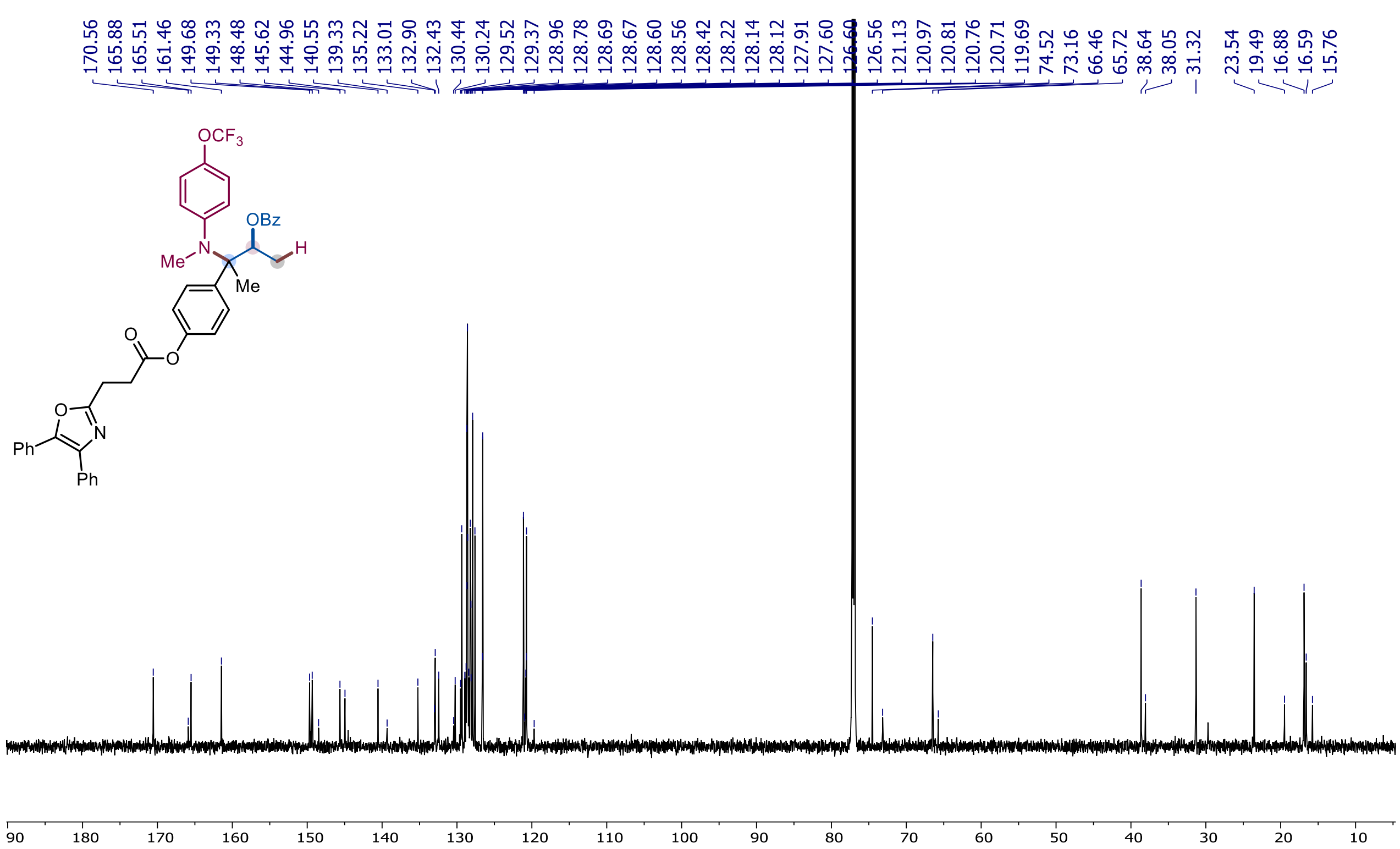

^{19}F NMR (376 MHz, $CDCl_3$, 25 ^oC) of 4h

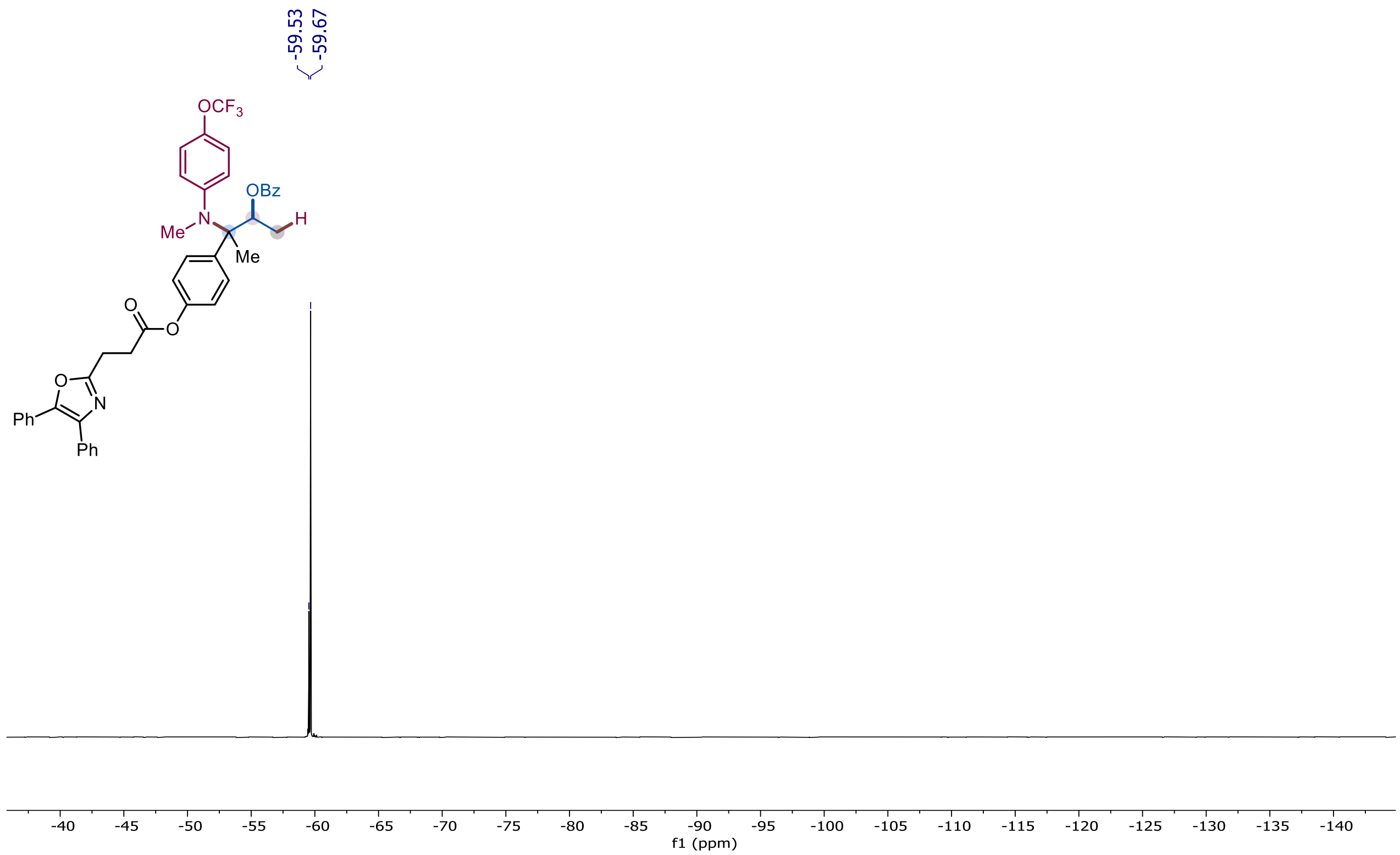

^{1}H NMR (500 MHz, $CDCl_3$, 25 ºC) of 4i

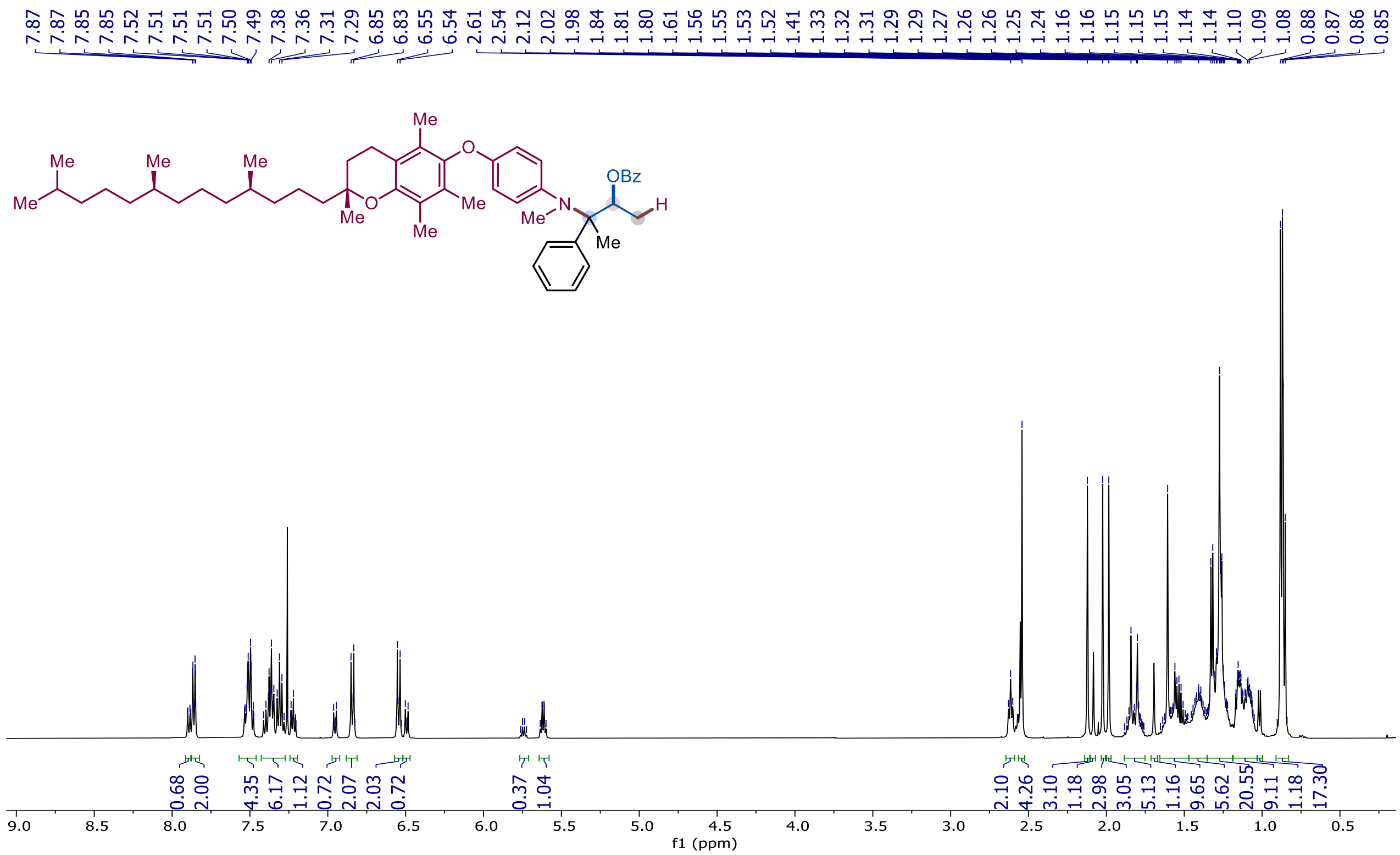

^{13}C NMR (126 MHz, $CDCl_3$, 25 ºC) of 4i

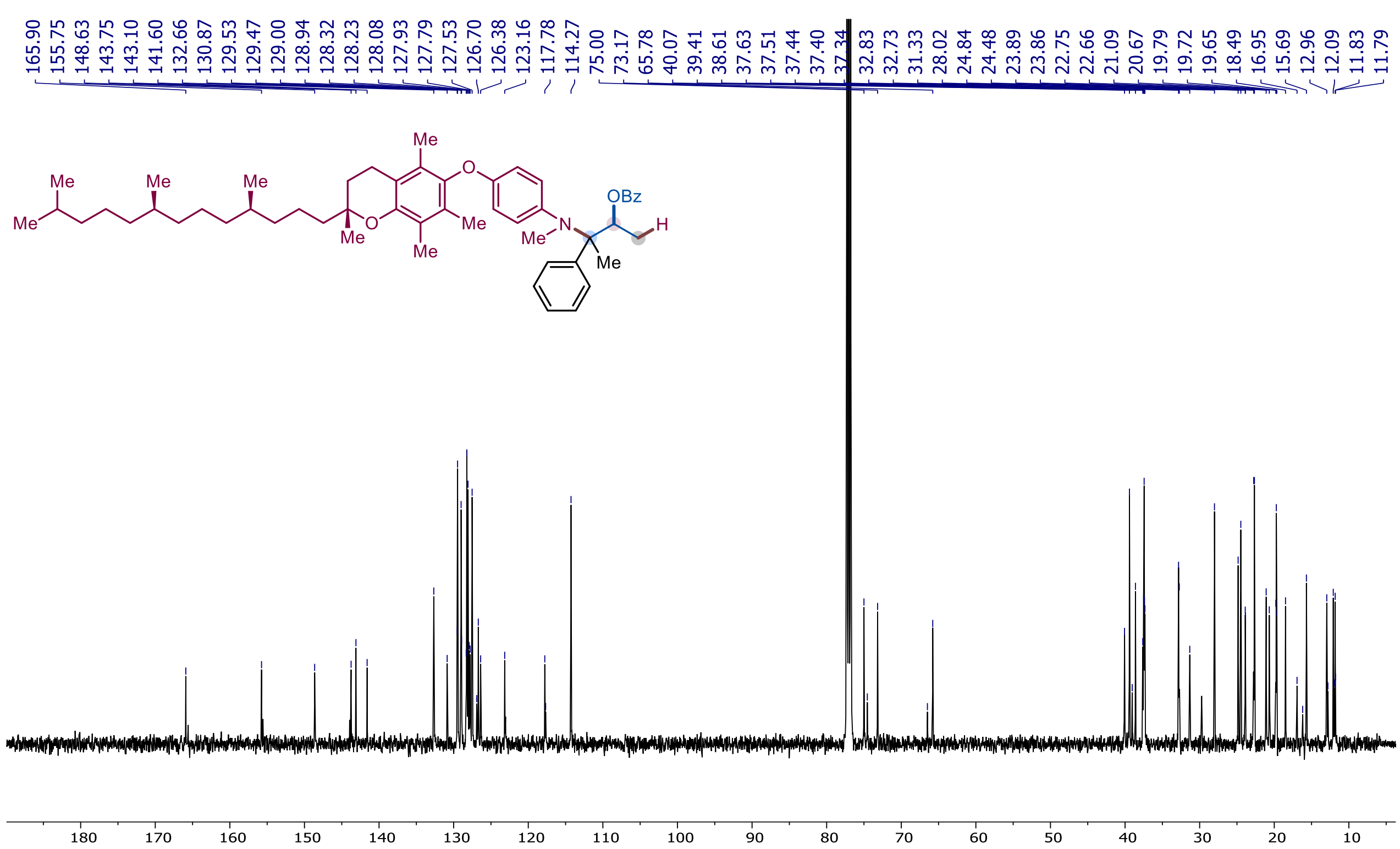

^{1}H NMR (400 MHz, CDCl$_3$, 25 °C) of 4j

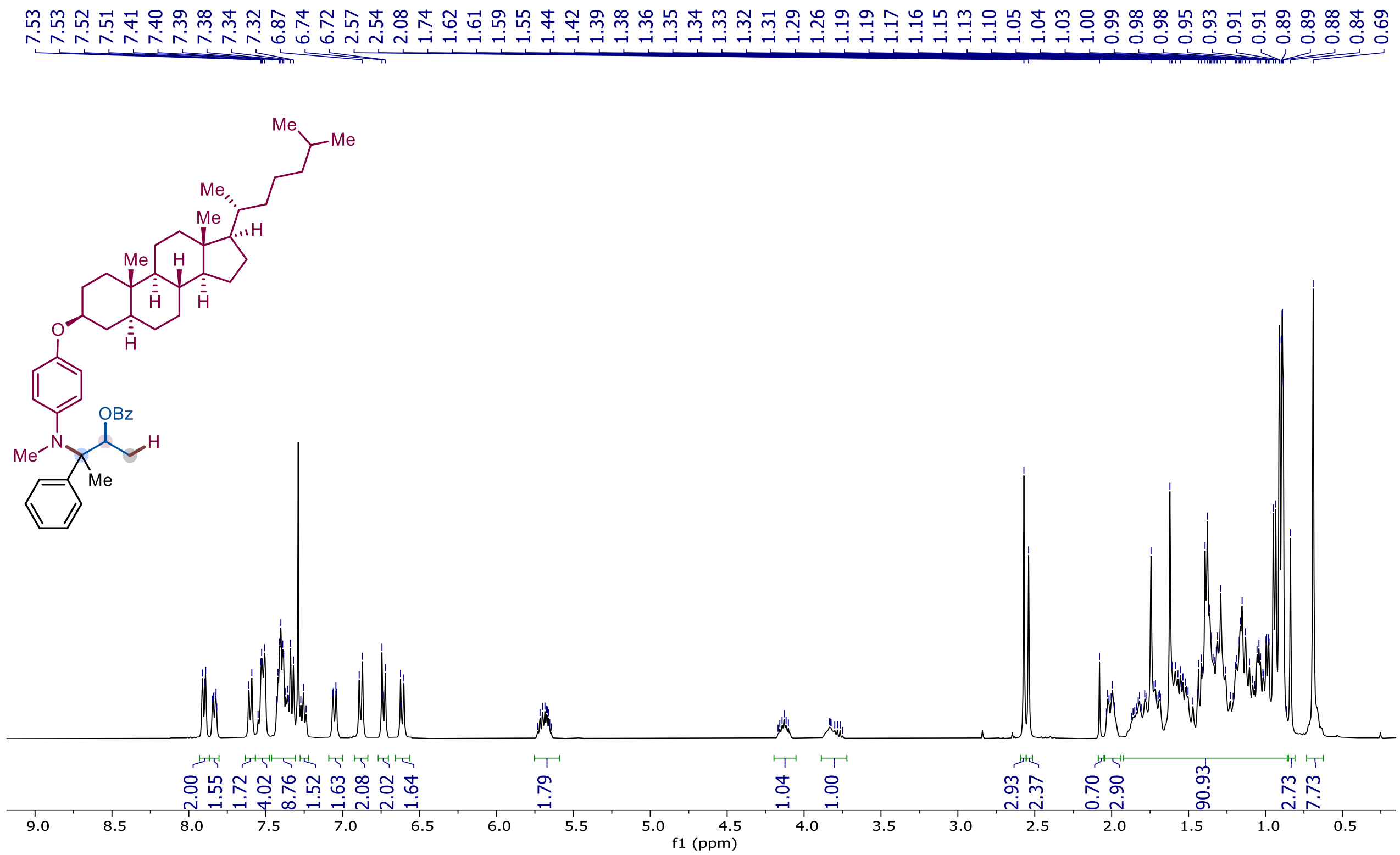

^{13}C NMR (126 MHz, CDCl$_3$, 25 °C) of 4j

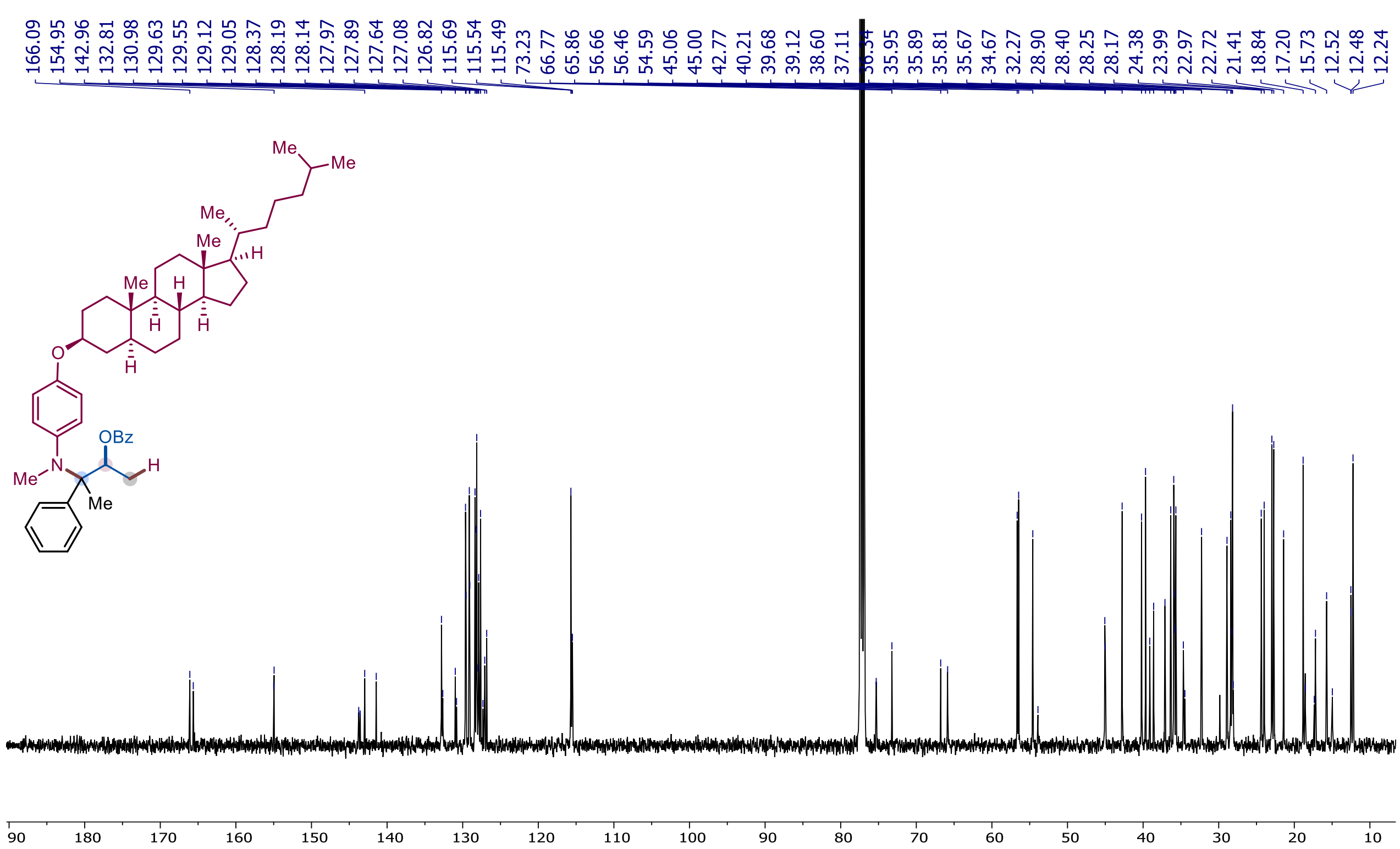

^{1}H NMR (800 MHz, $CDCl_3$, 25 °C) of 4k

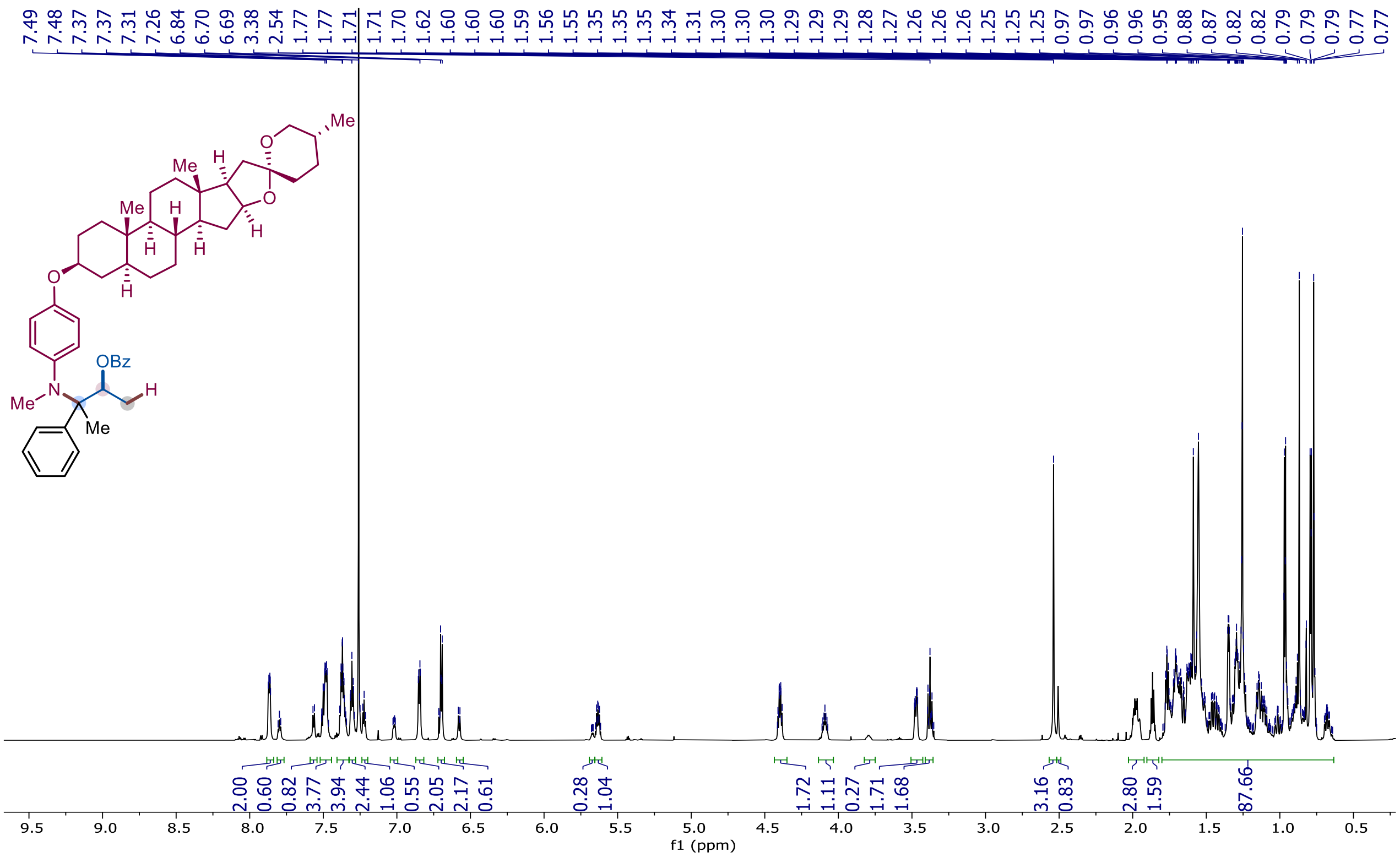

^{13}C NMR (201 MHz, $CDCl_3$, 25 °C) of 4k

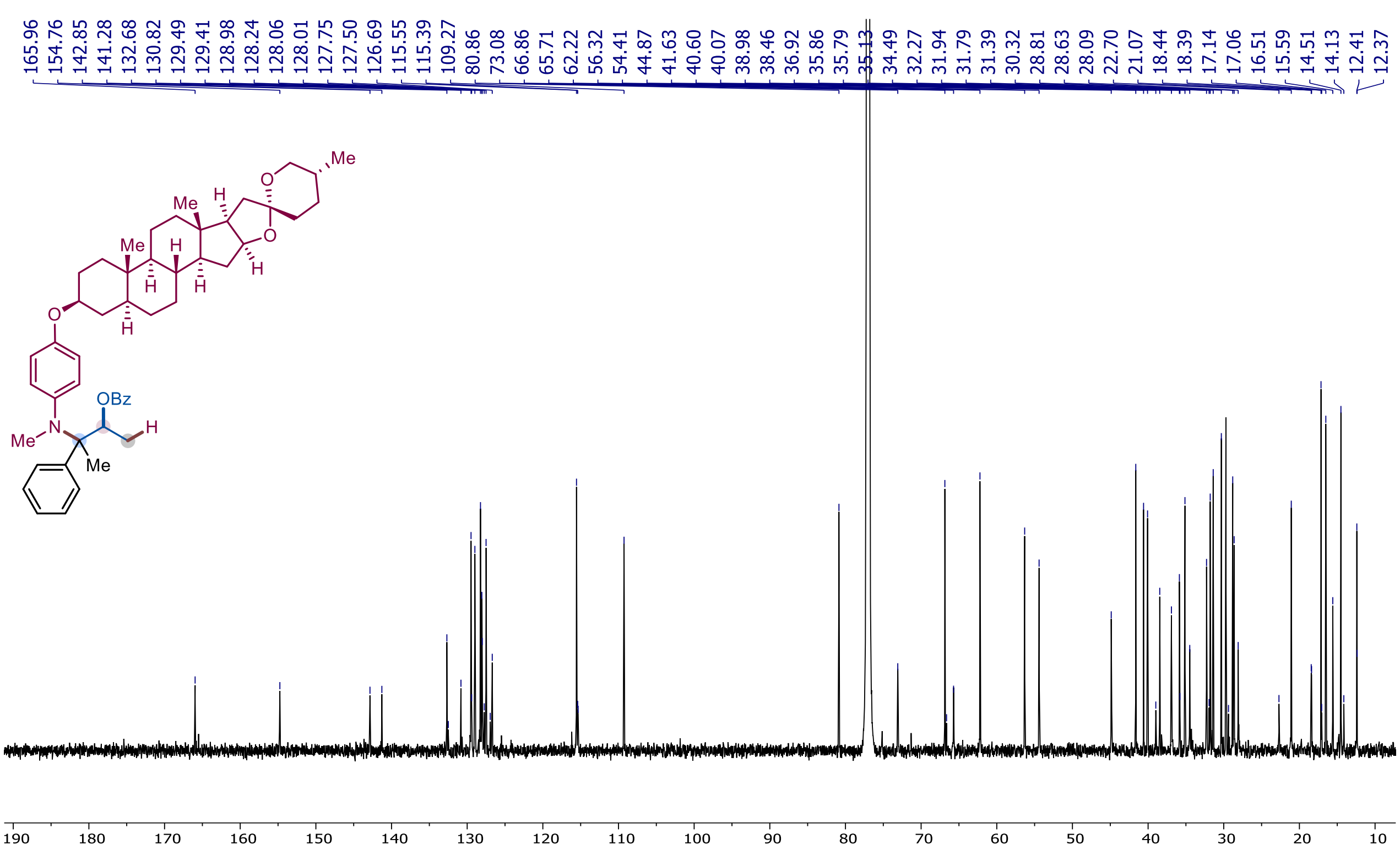

^{1}H NMR (800 MHz, CDCl$_3$, 25 ºC) of 4l

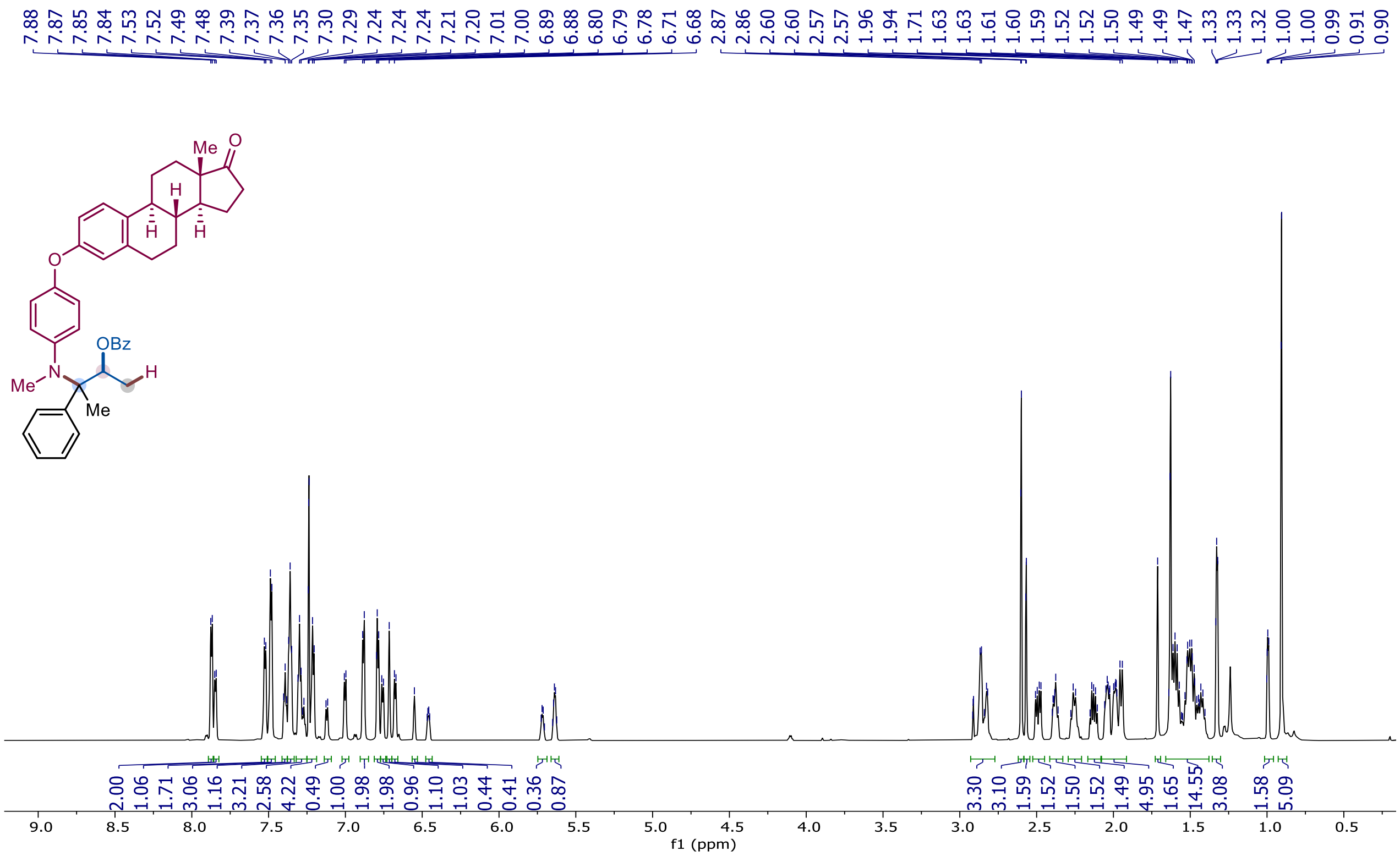

^{13}C NMR (201 MHz, CDCl$_3$, 25 ºC) of 4l

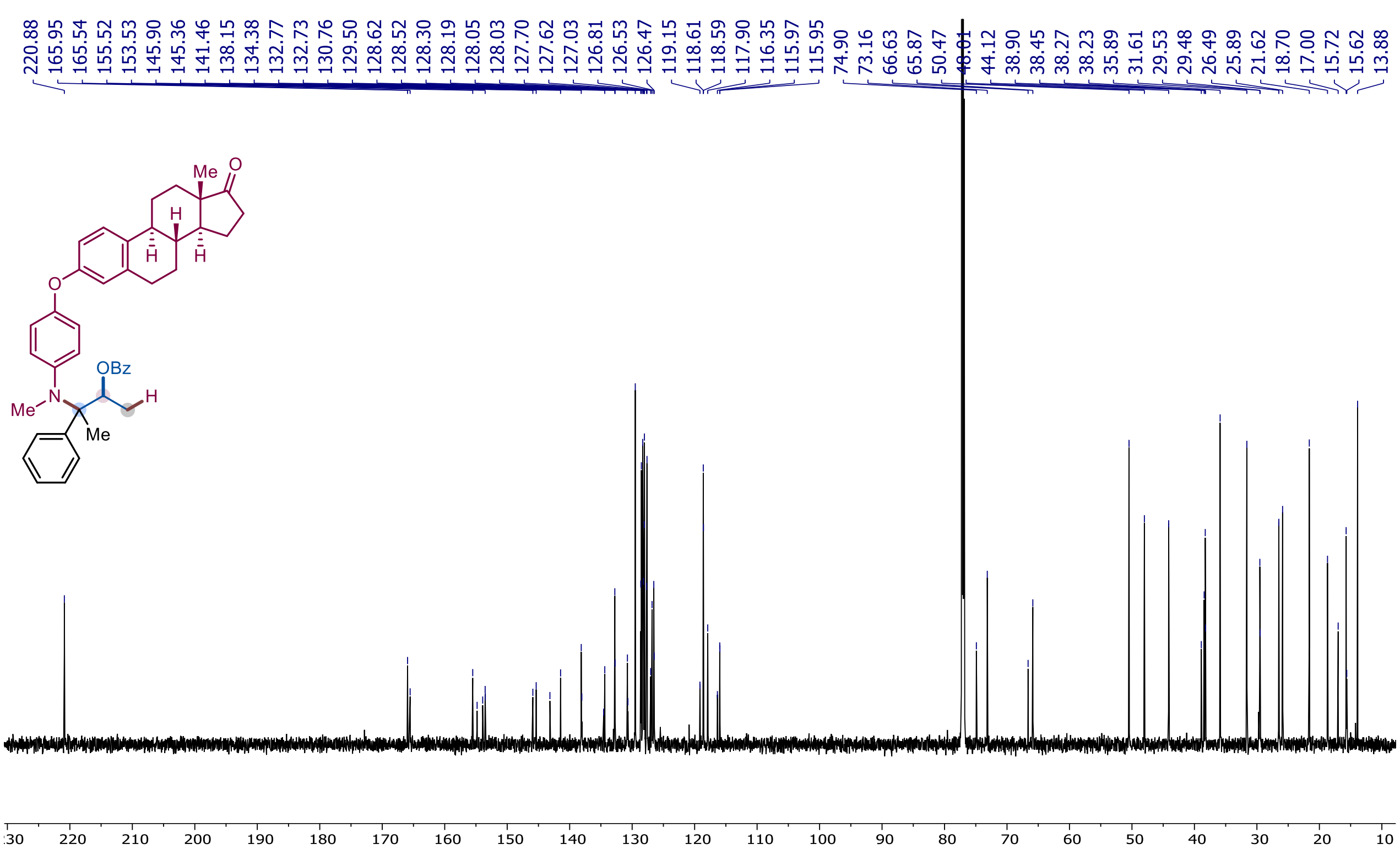

^{1}H NMR (500 MHz, $CDCl_3$, 25 ºC) of 3aaw

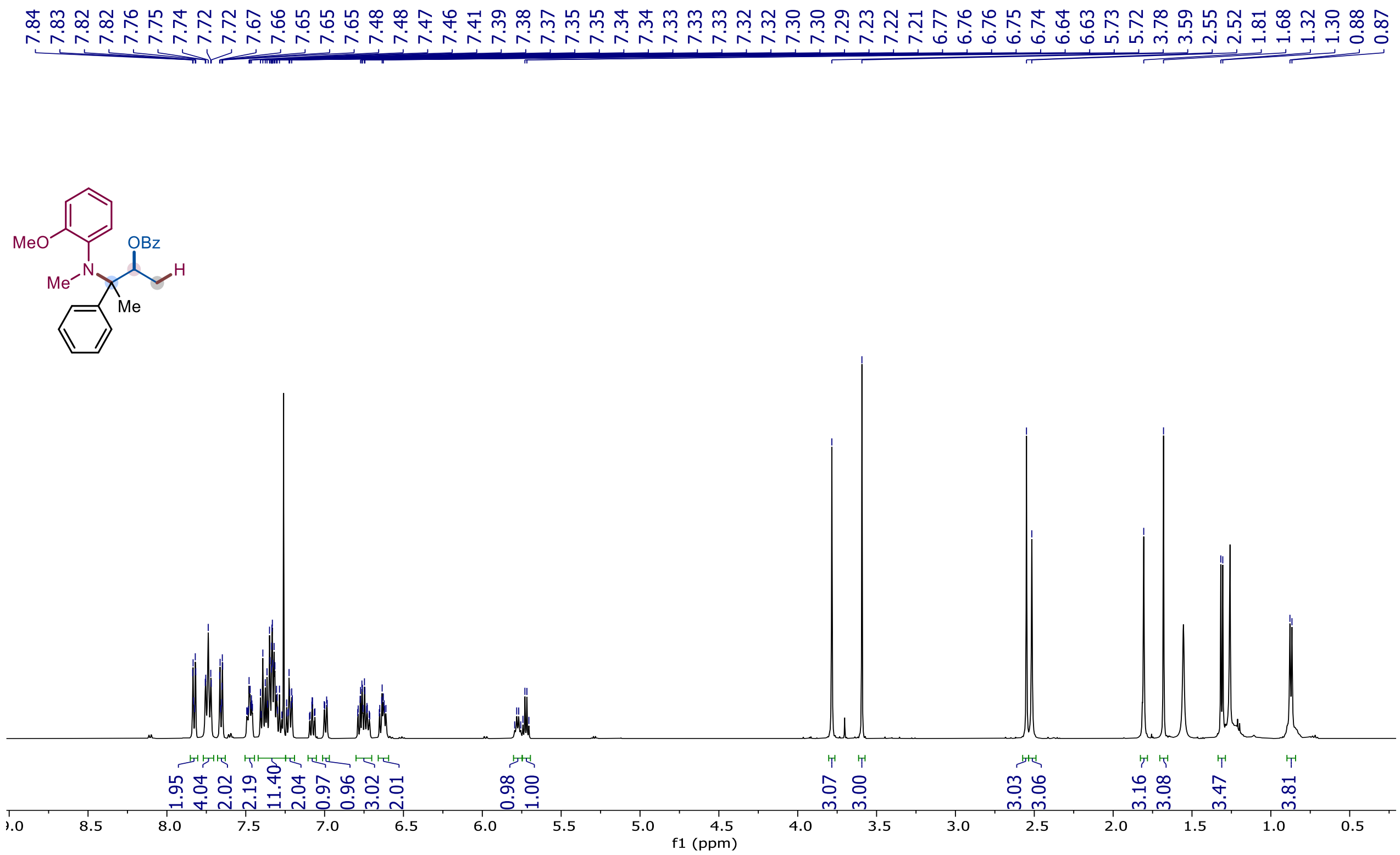

^{13}C NMR (126 MHz, $CDCl_3$, 25 ºC) of 3aaw

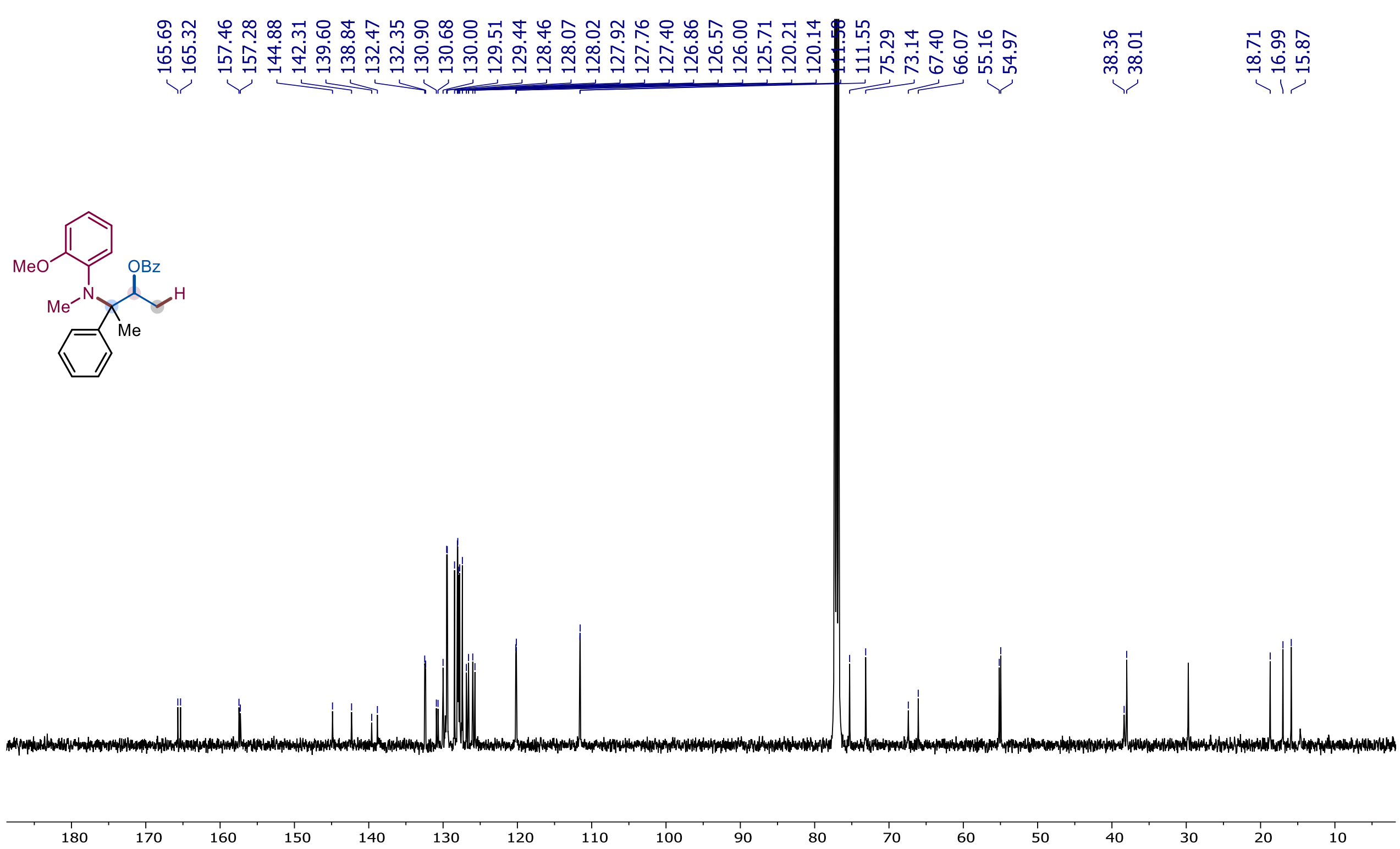

^{1}H NMR (800 MHz, CDCl$_3$, 25 ºC) of 3aax

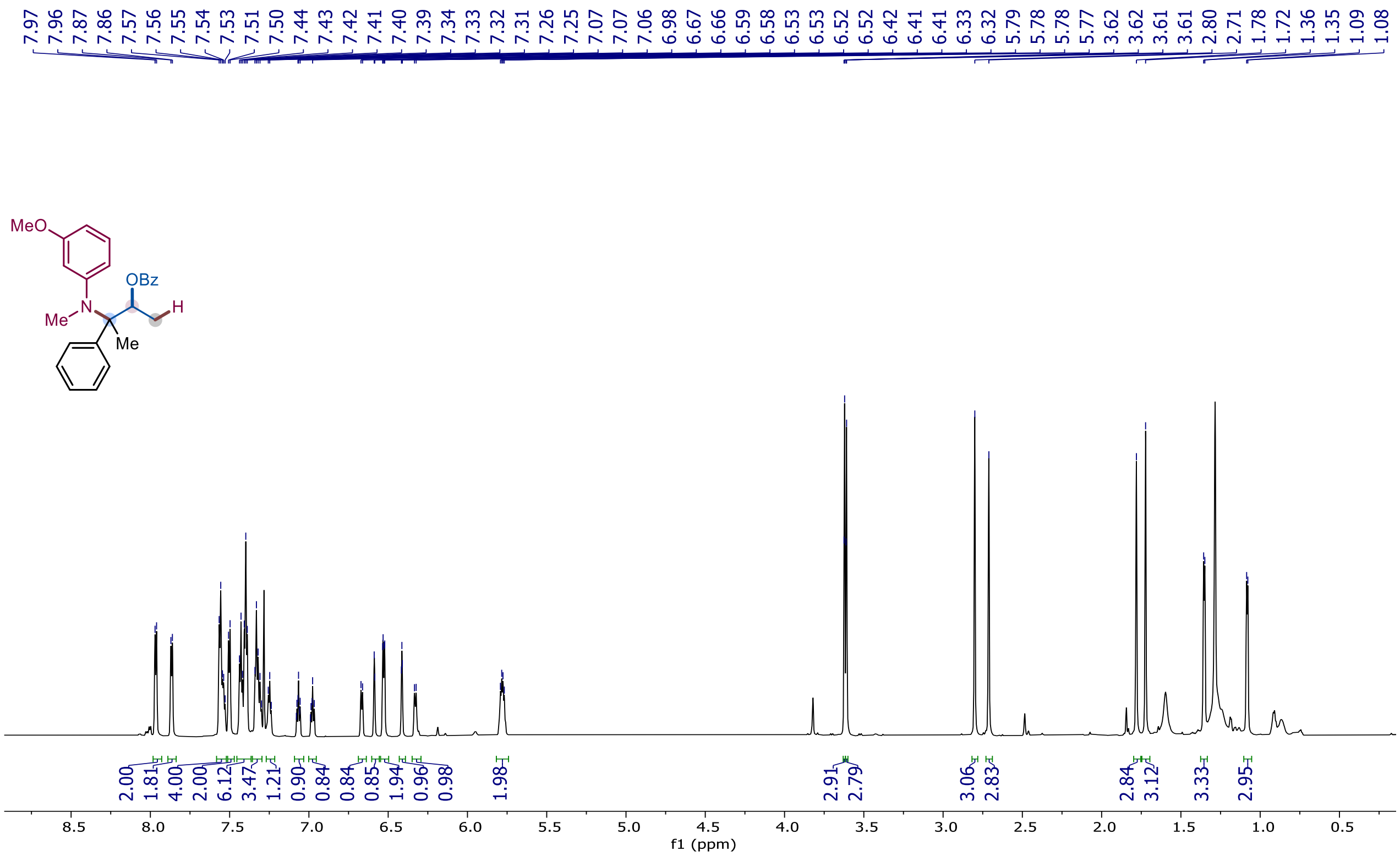

^{13}C NMR (201 MHz, CDCl$_3$, 25 ºC) of 3aax

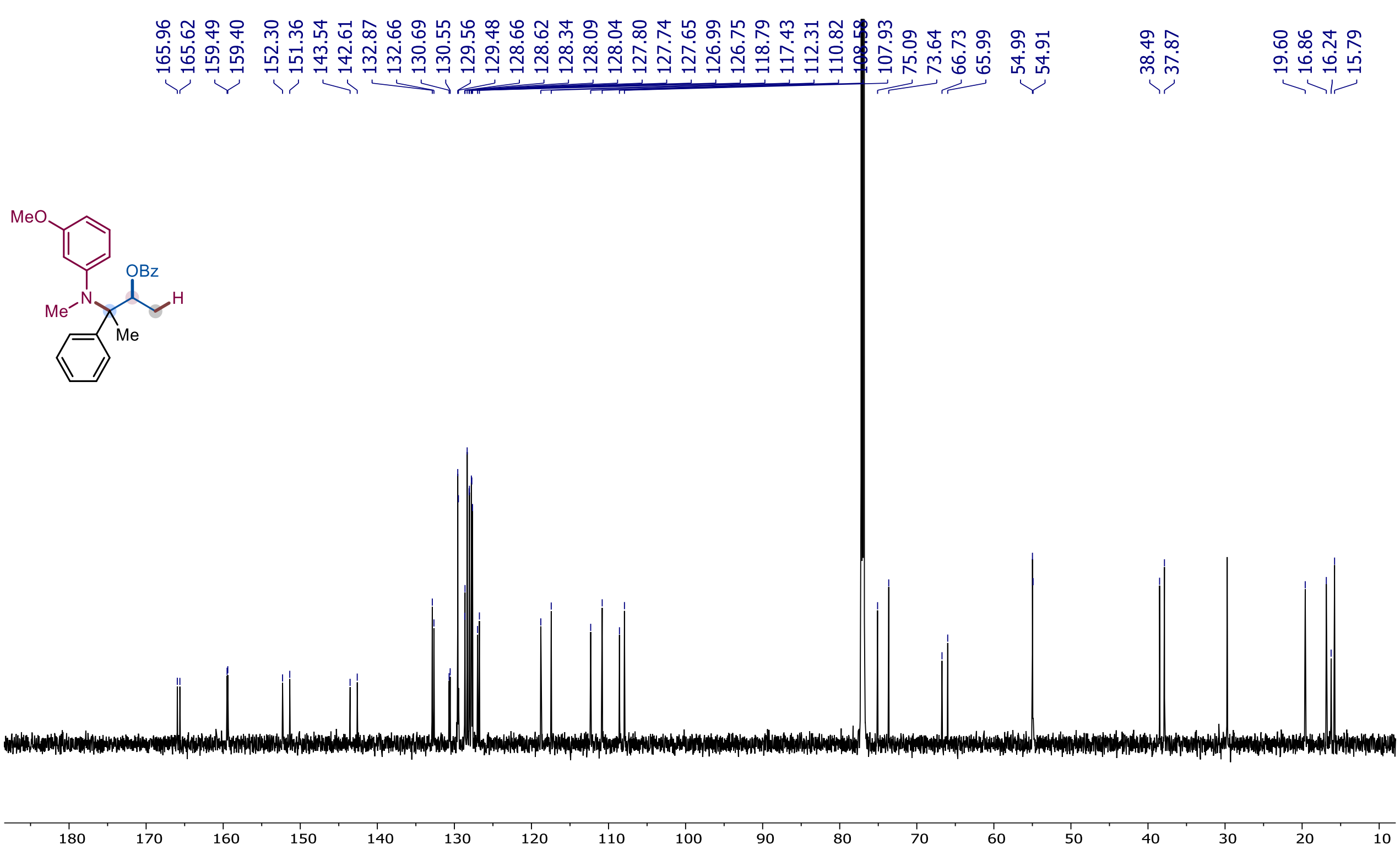

^{1}H NMR (400 MHz, $CDCl_3$, 25 ºC) of 3aay

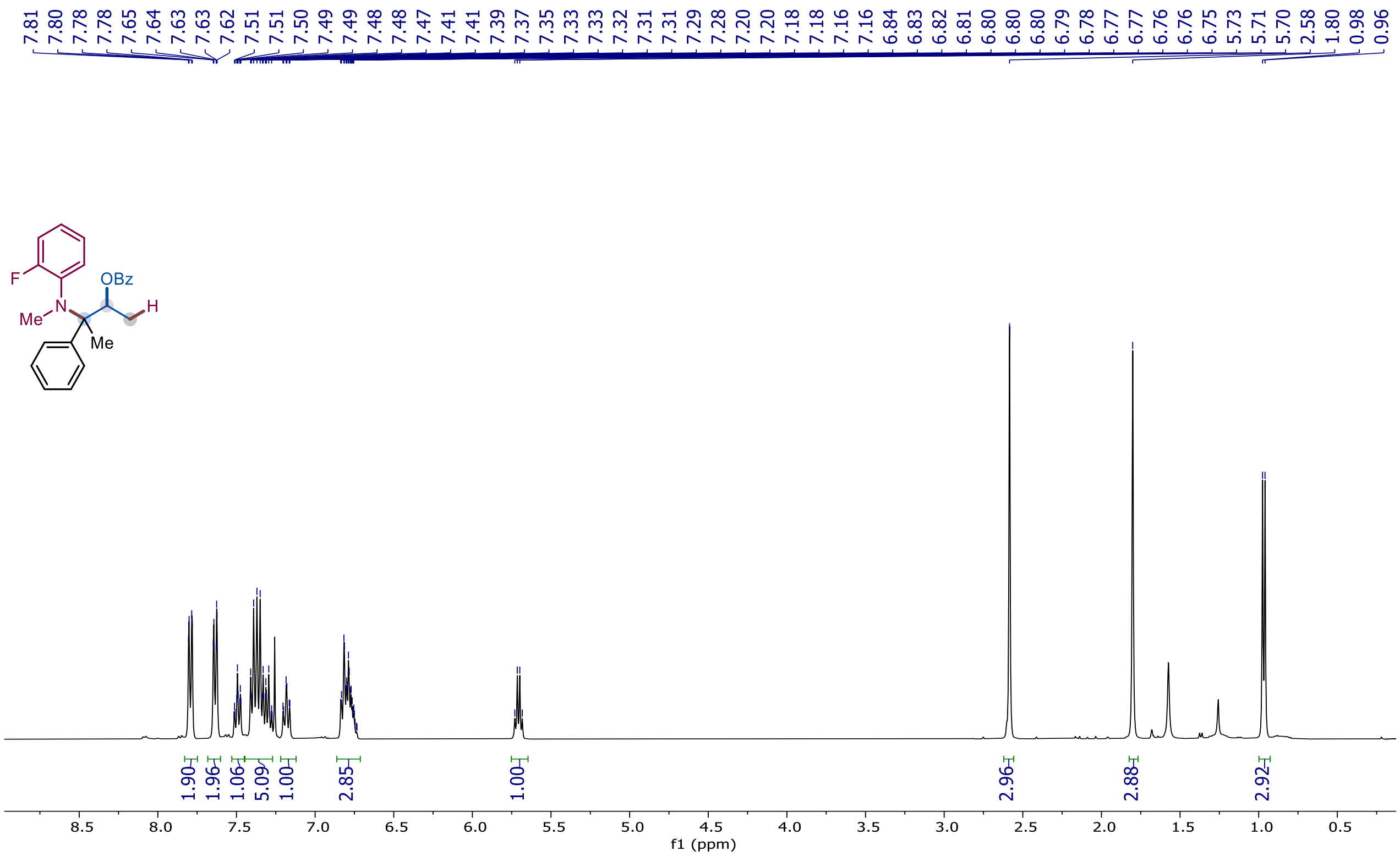

^{13}C NMR (201 MHz, $CDCl_3$, 25 ºC) of 3aay

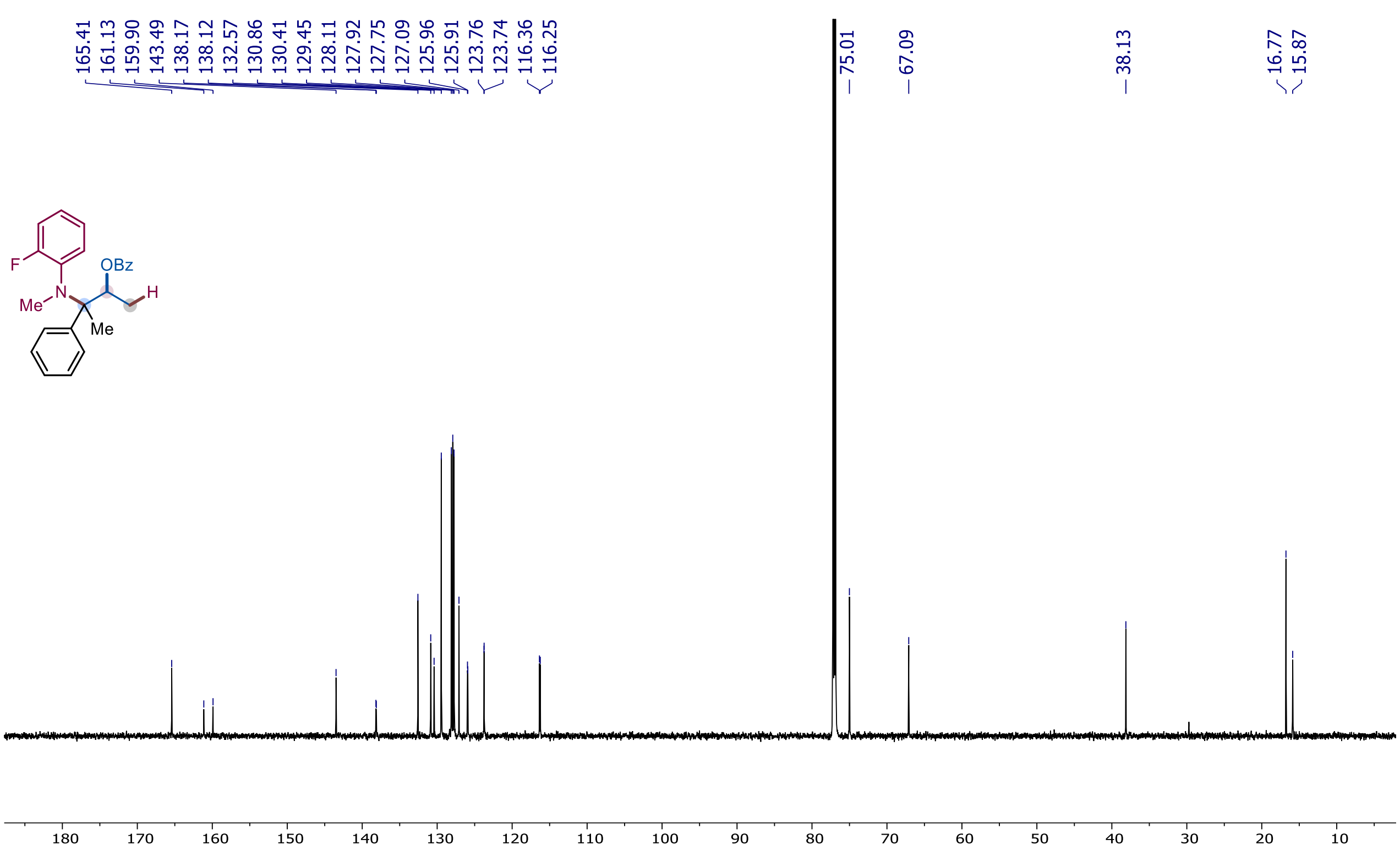

^{19}F NMR (376 MHz, $CDCl_3$, 25 °C) of 3aay

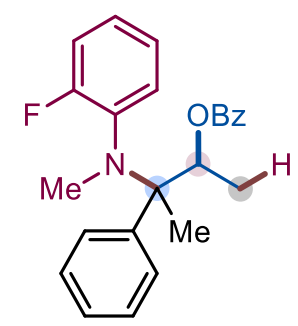

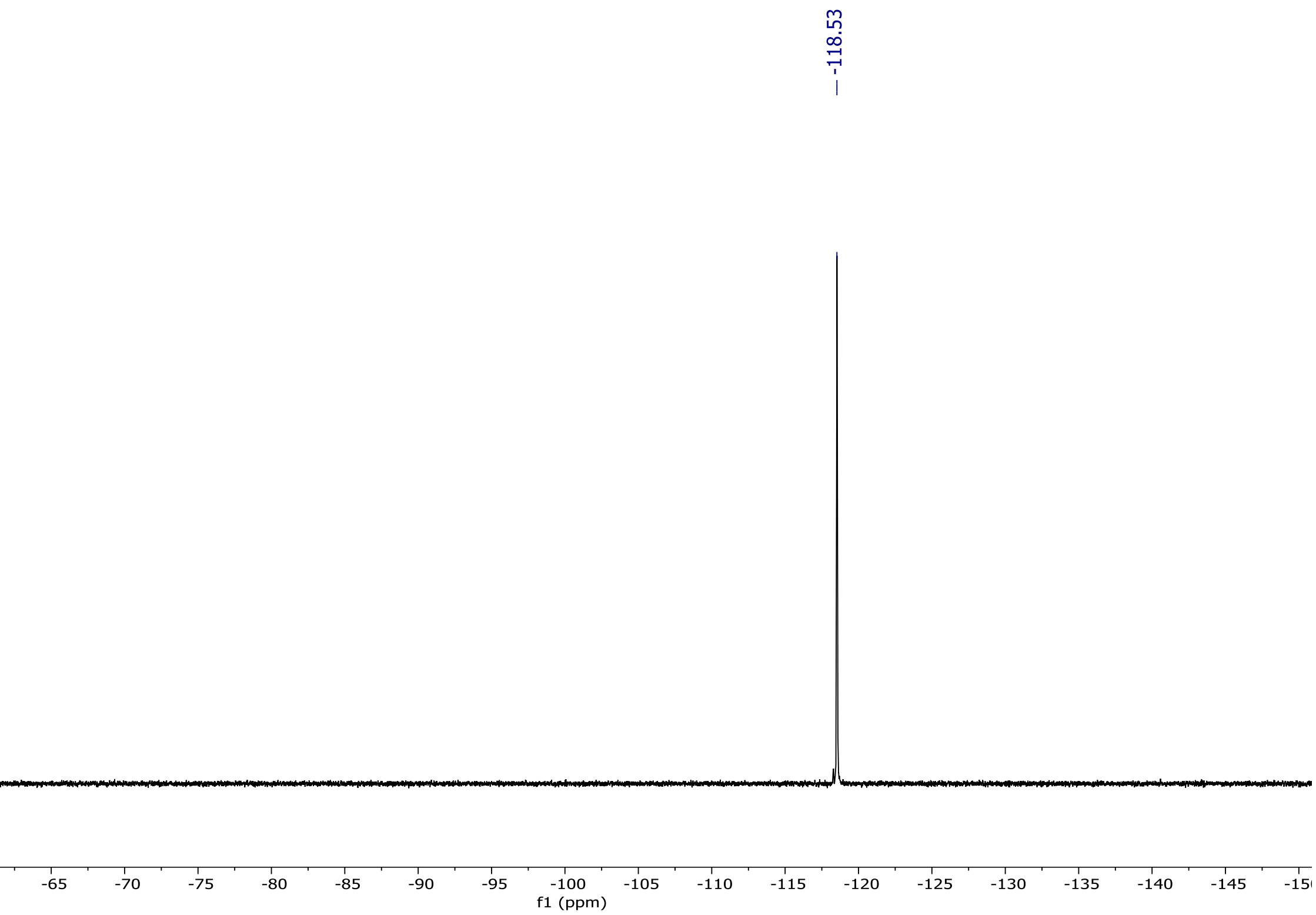

^{1}H NMR (500 MHz, $CDCl_3$, 25 ºC) of 3aaz

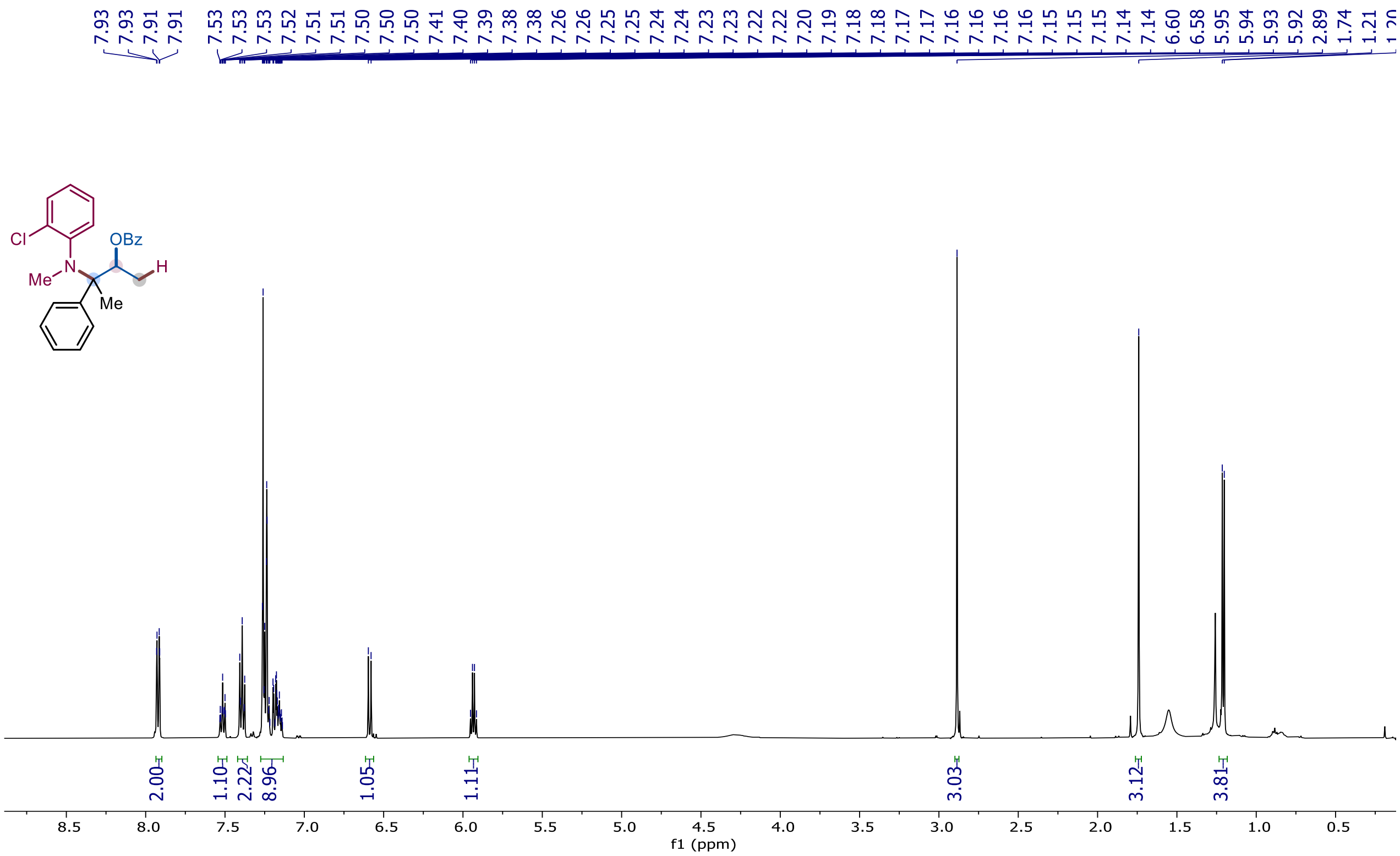

^{13}C NMR (126 MHz, $CDCl_3$, 25 ºC) of 3aaz

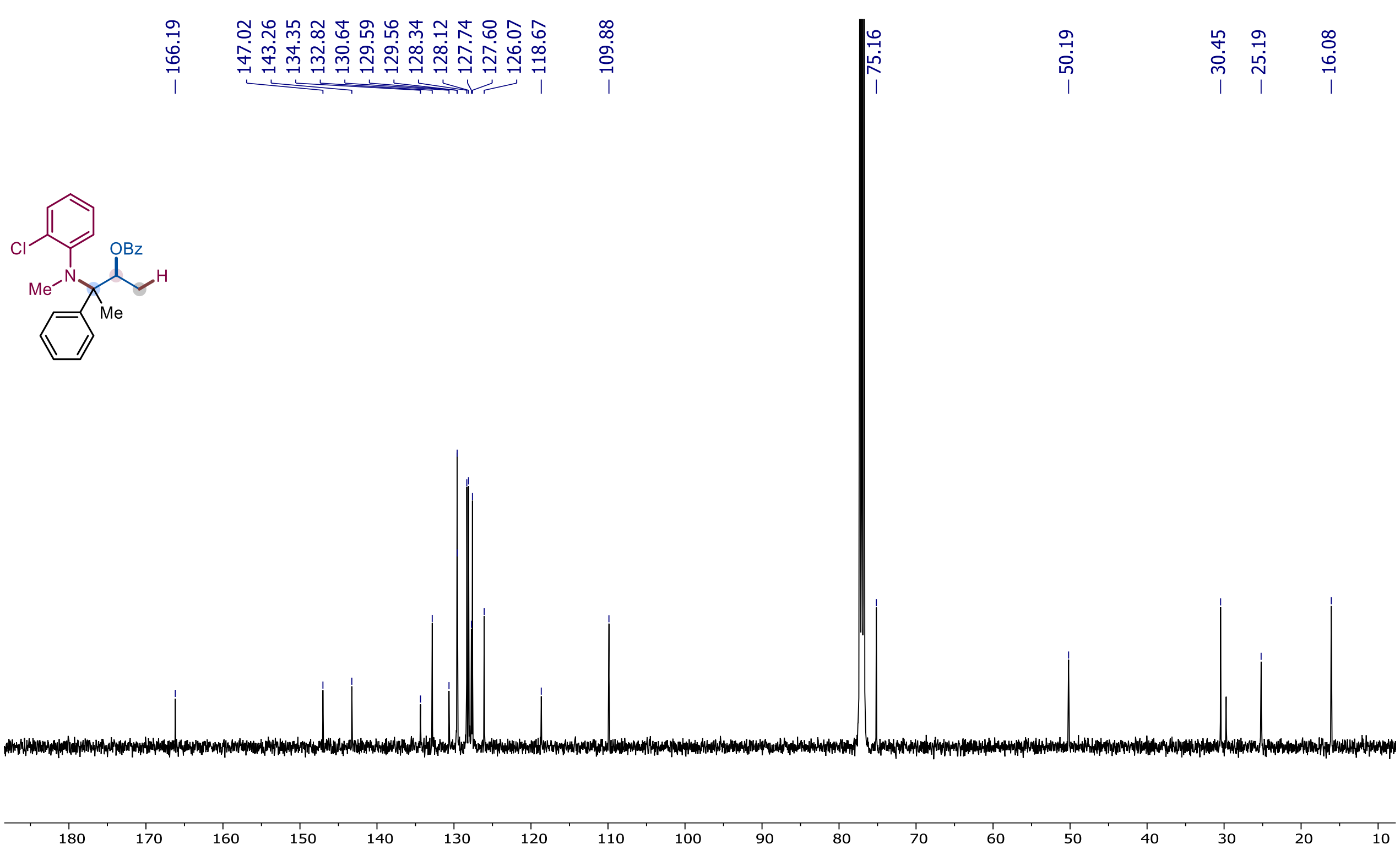

^{1}H NMR (500 MHz, $CDCl_3$, 25 ºC) of 3aaaa

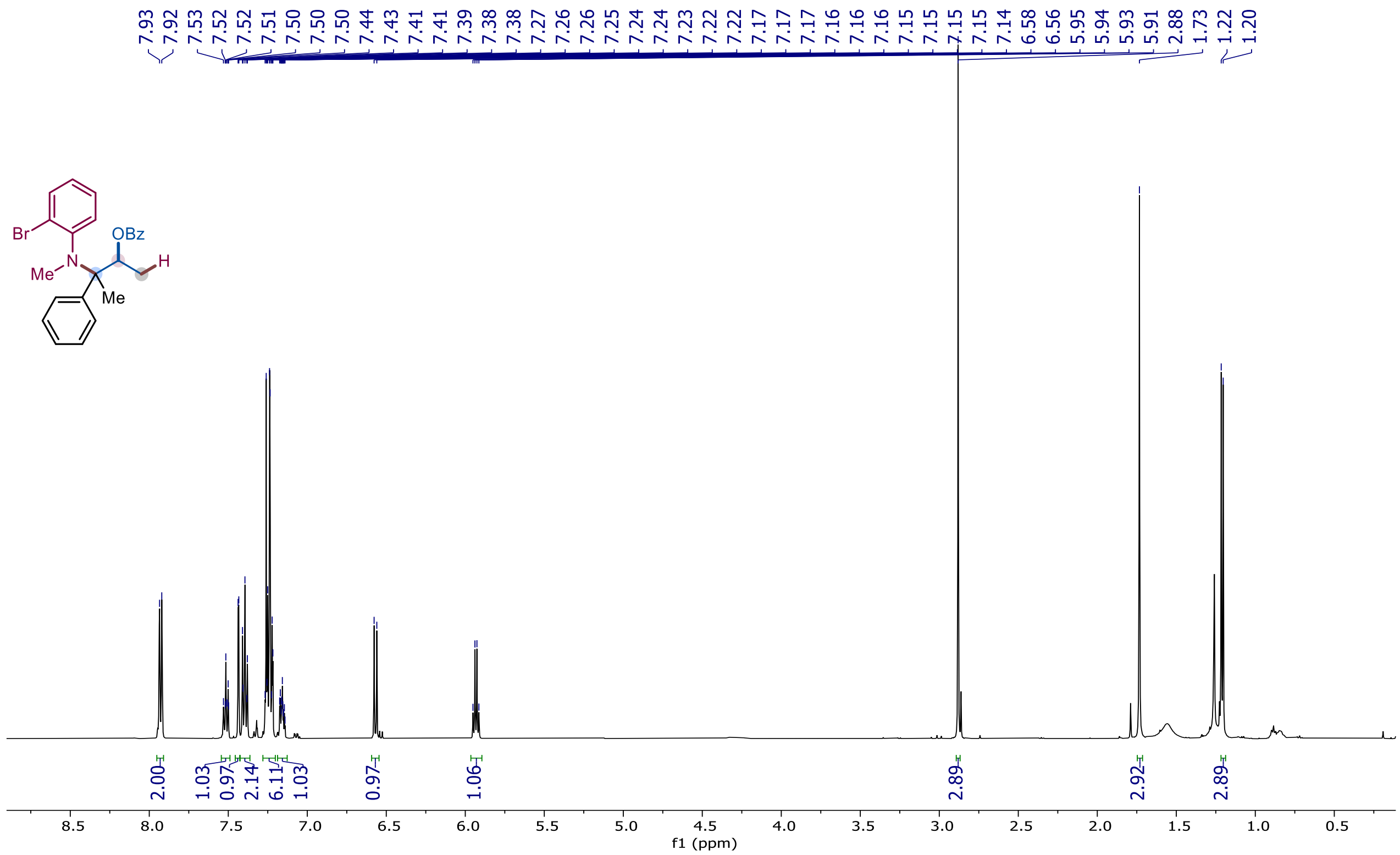

^{13}C NMR (126 MHz, $CDCl_3$, 25 ºC) of 3aaaa

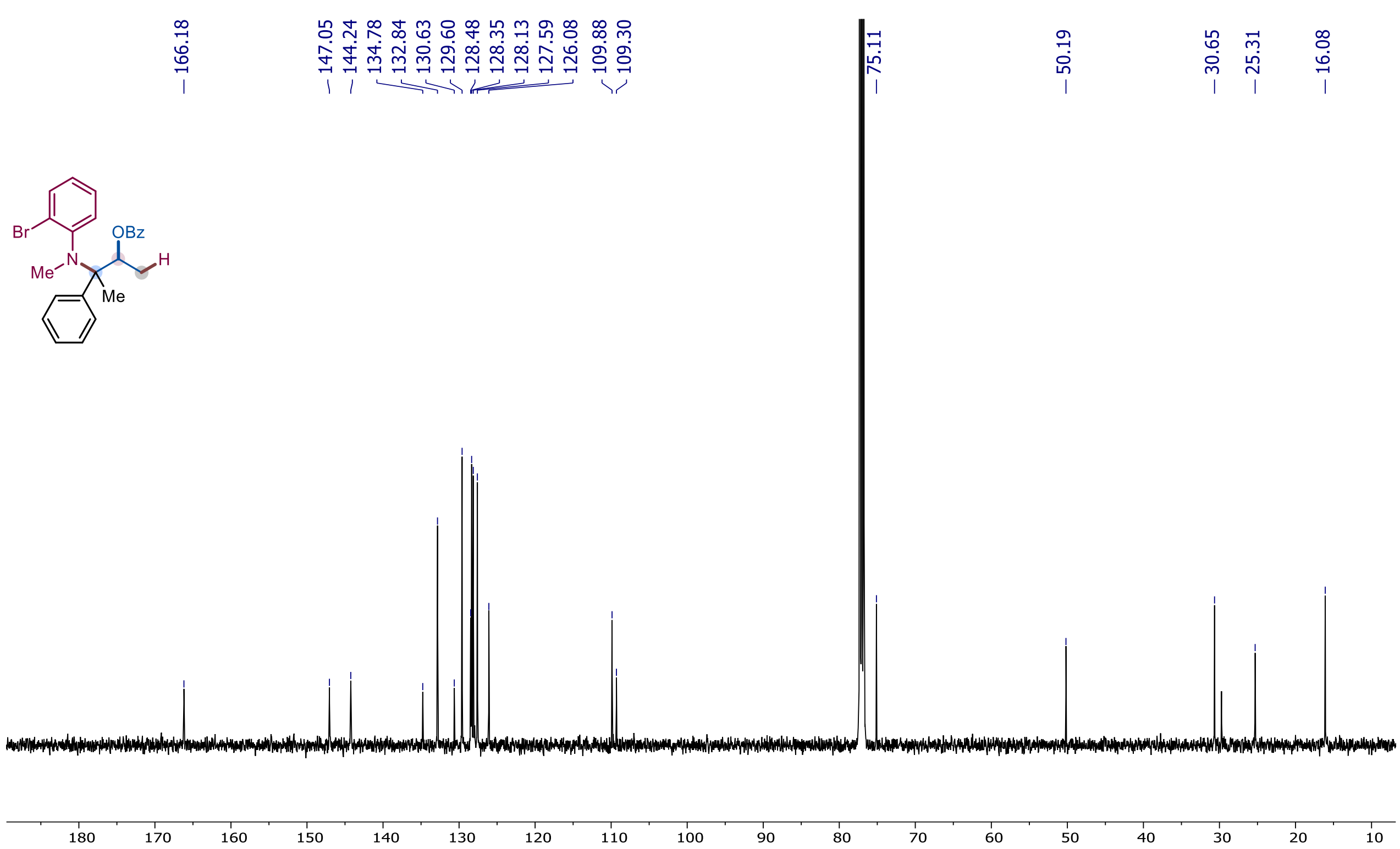

^{1}H NMR (400 MHz, CDCl$_3$, 25 ºC) of 3aaab

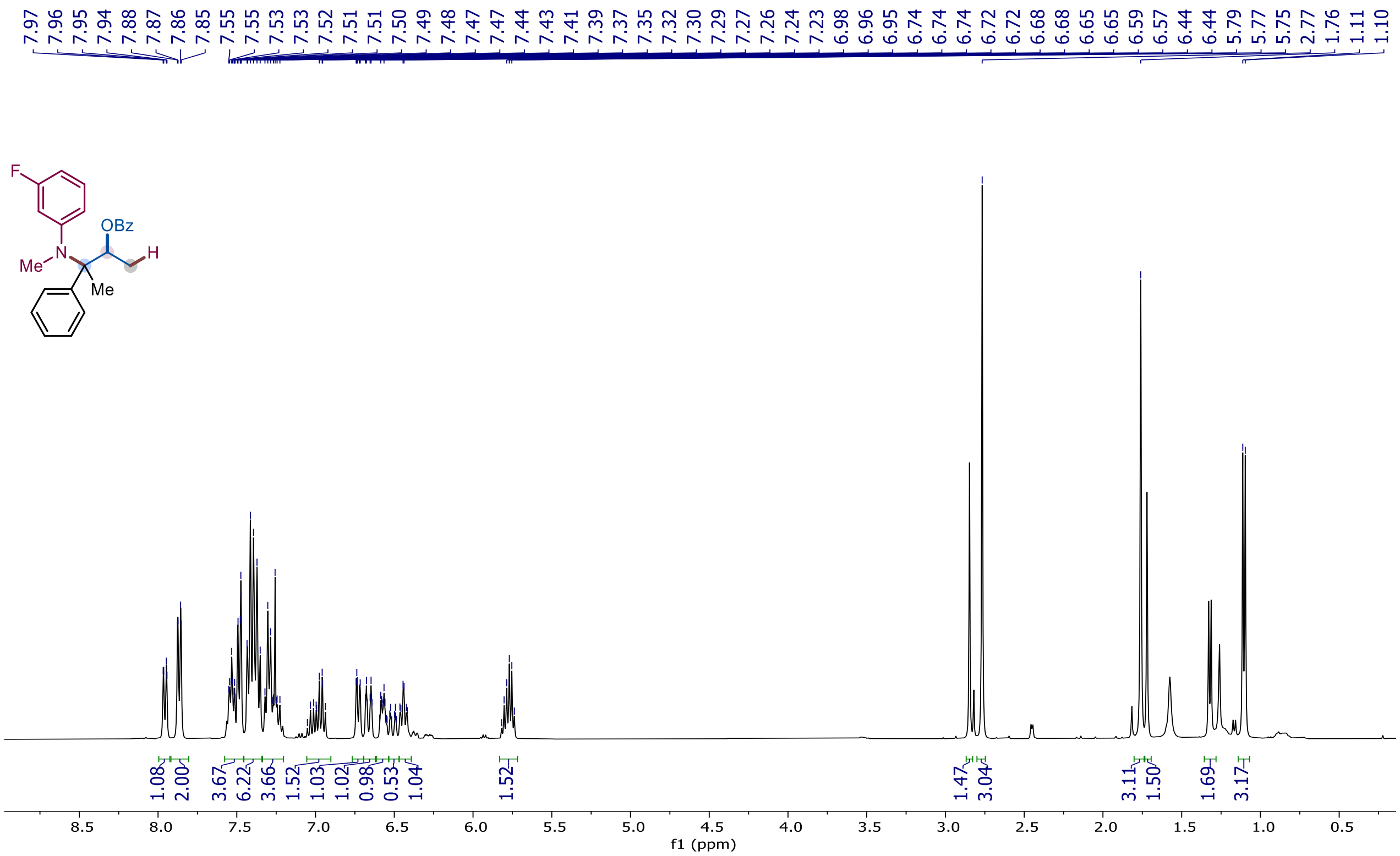

^{13}C NMR (201 MHz, CDCl$_3$, 25 ºC) of 3aaab

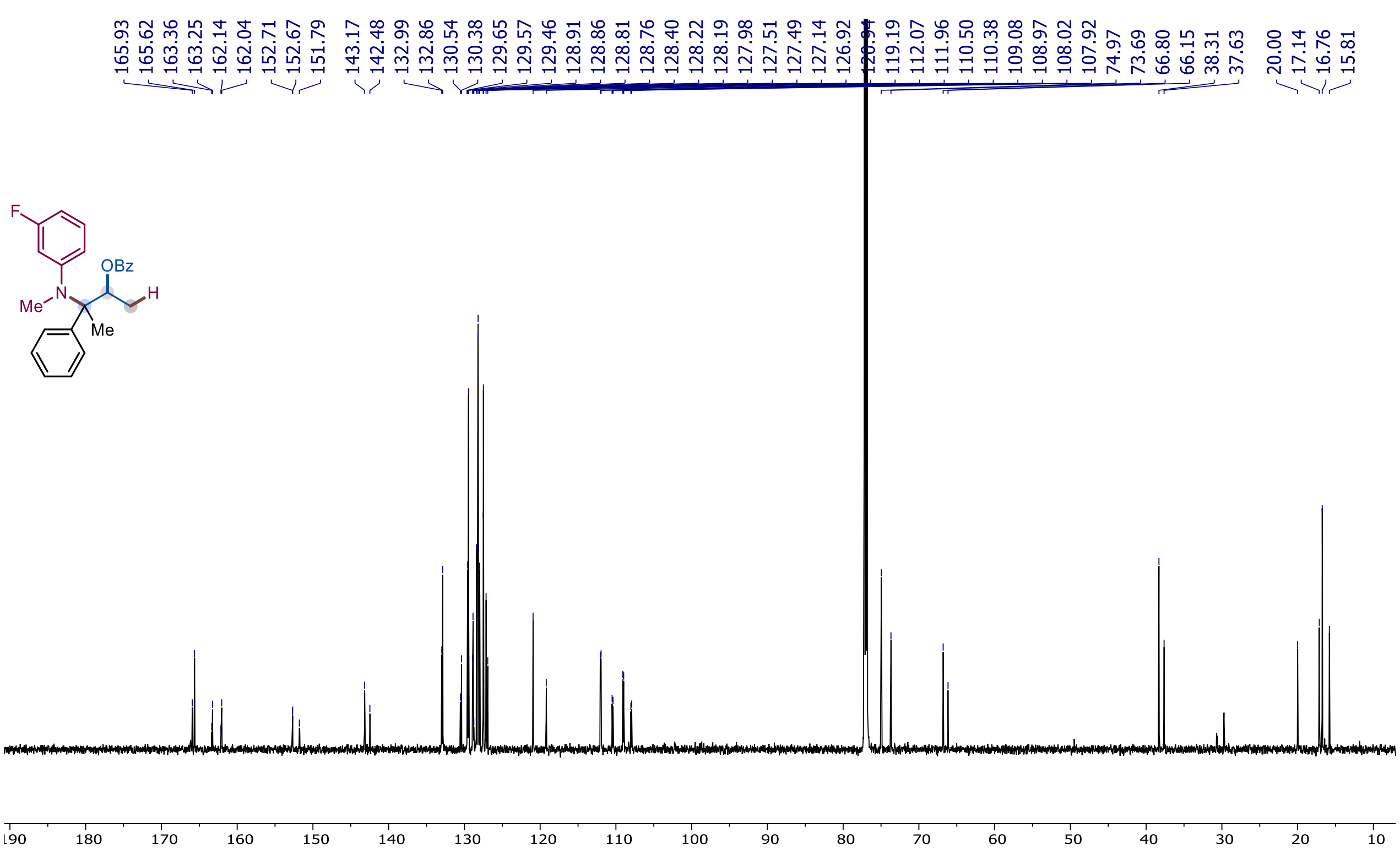

^{19}F NMR (376 MHz, $CDCl_3$, 25 ºC) of 3aaab

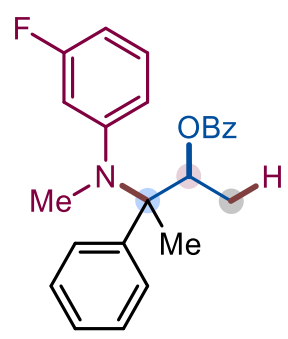

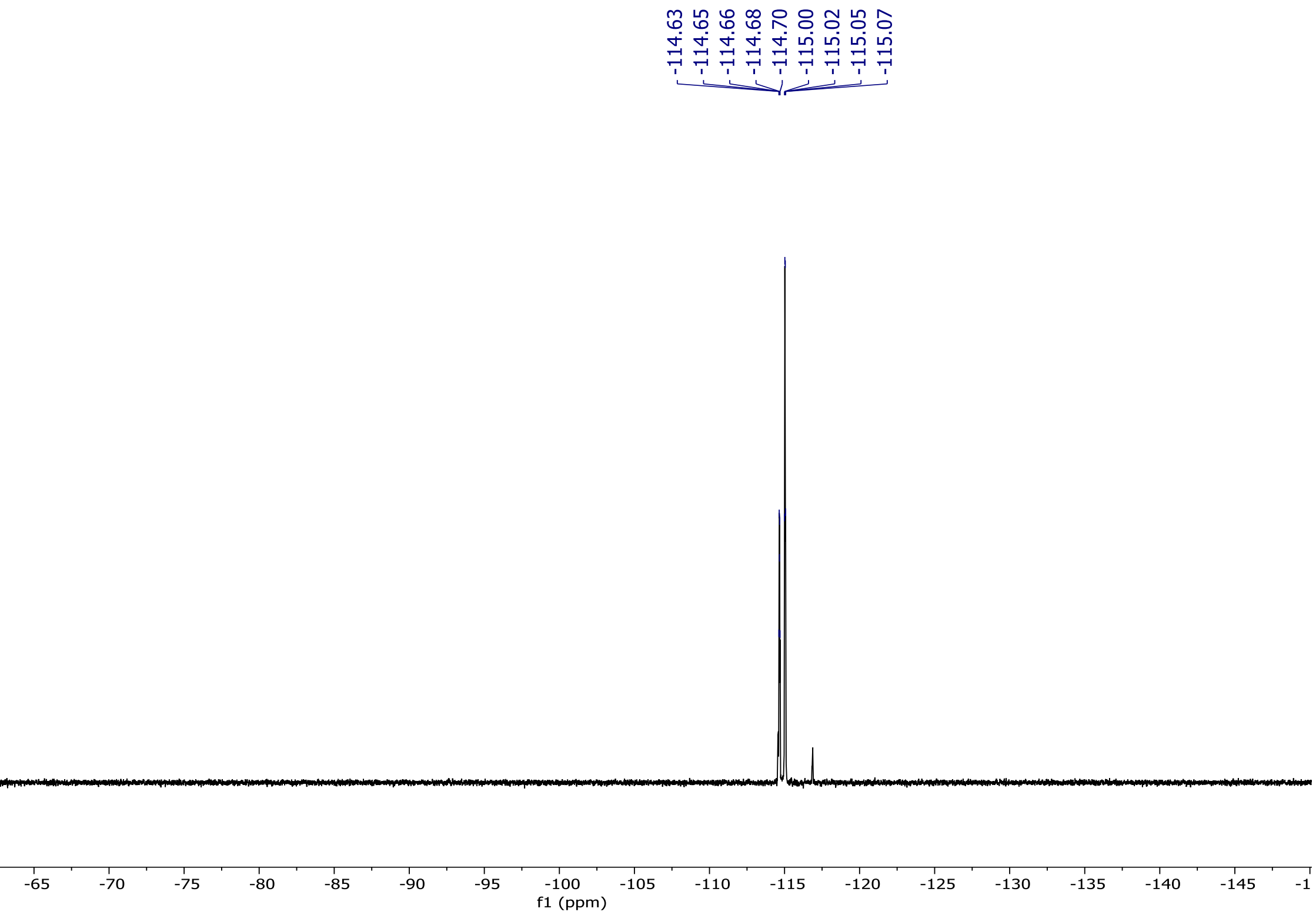

^{1}H NMR (500 MHz, $CDCl_3$, 25 ºC) of 3aaac

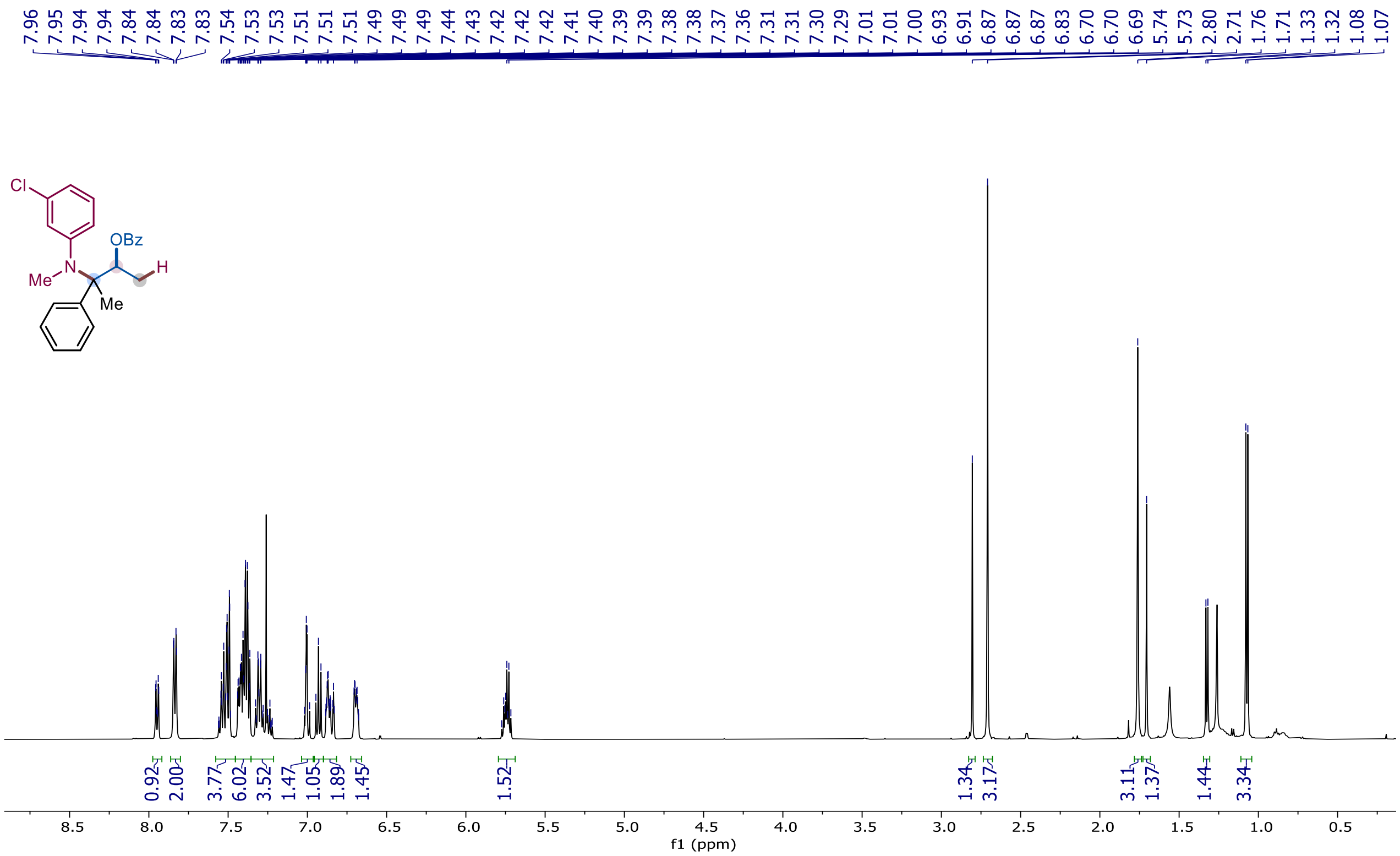

^{13}C NMR (126 MHz, $CDCl_3$, 25 ºC) of 3aaac

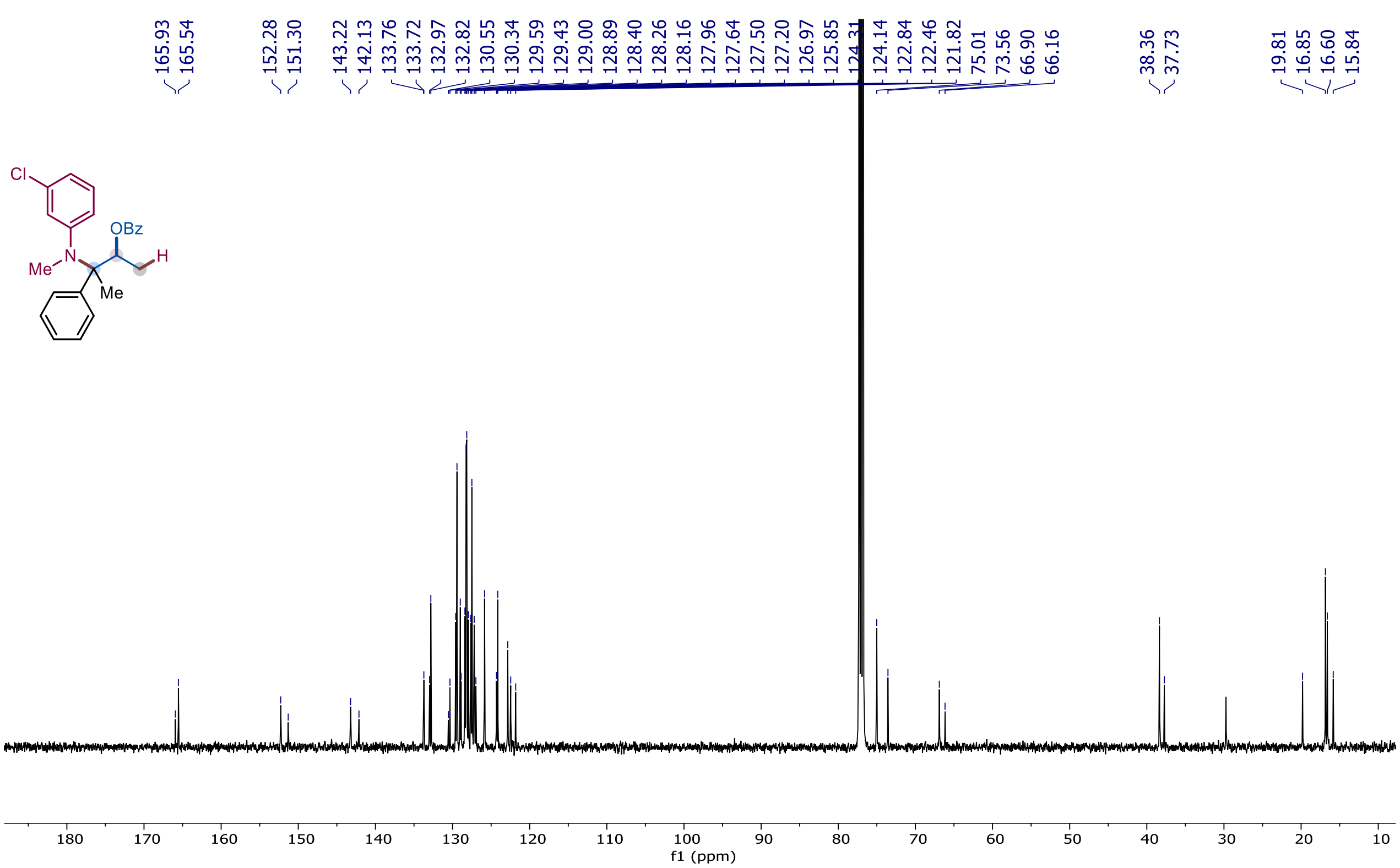

^{1}H NMR (500 MHz, $CDCl_3$, 25 ºC) of 3aaad

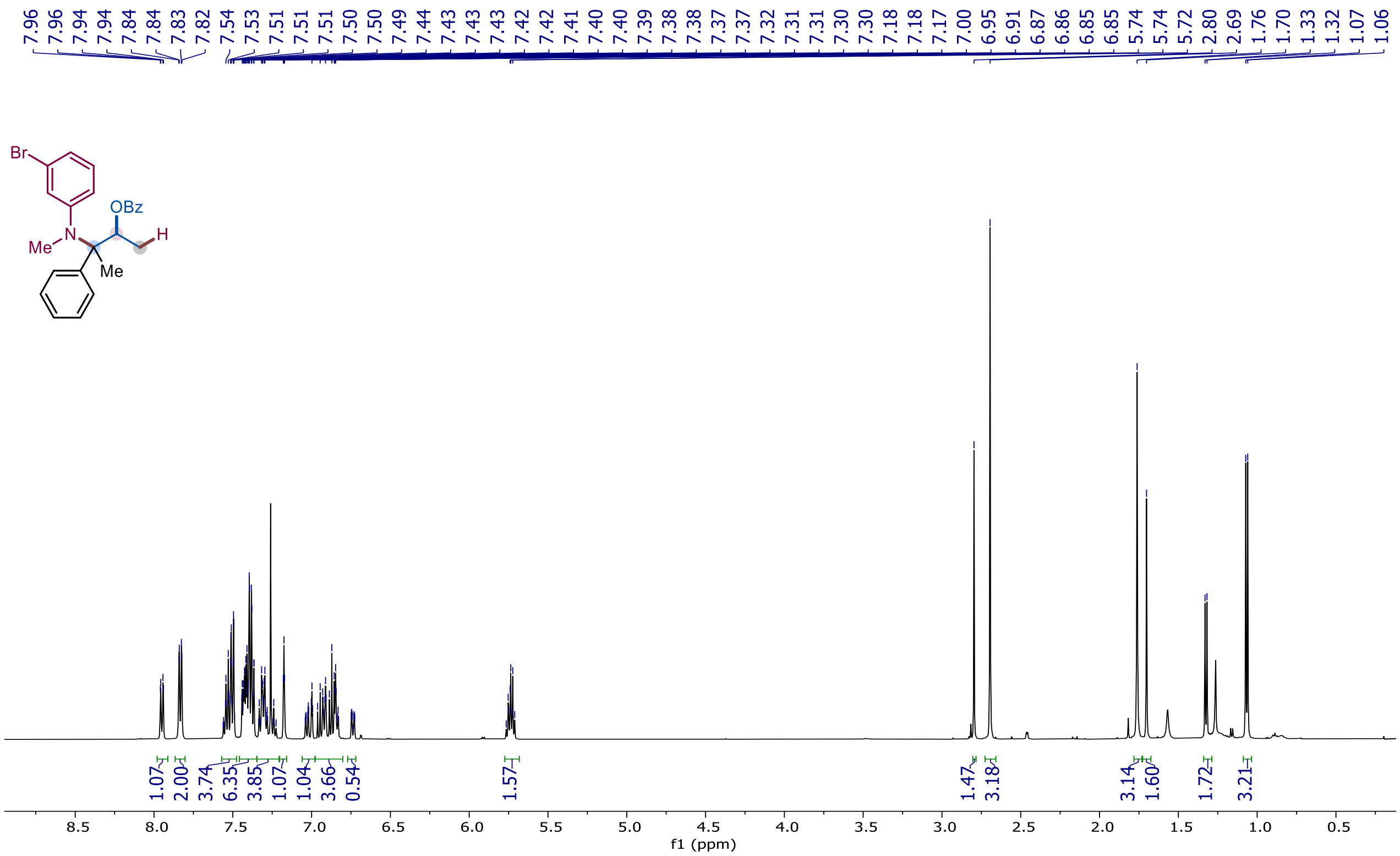

^{13}C NMR (126 MHz, $CDCl_3$, 25 ºC) of 3aaad

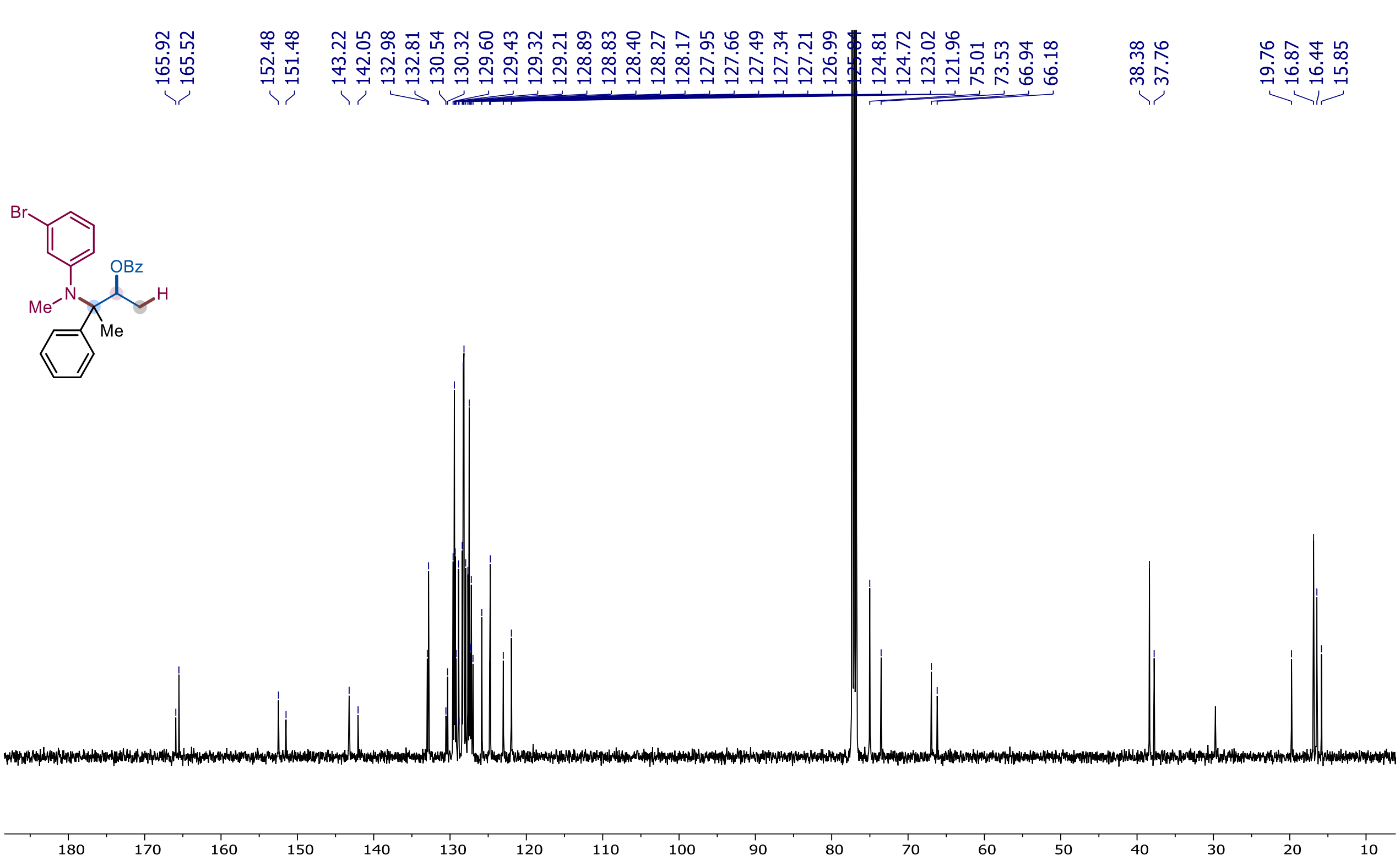

^{1}H NMR (300 MHz, $CDCl_3$, 25 ºC) of 3aaae

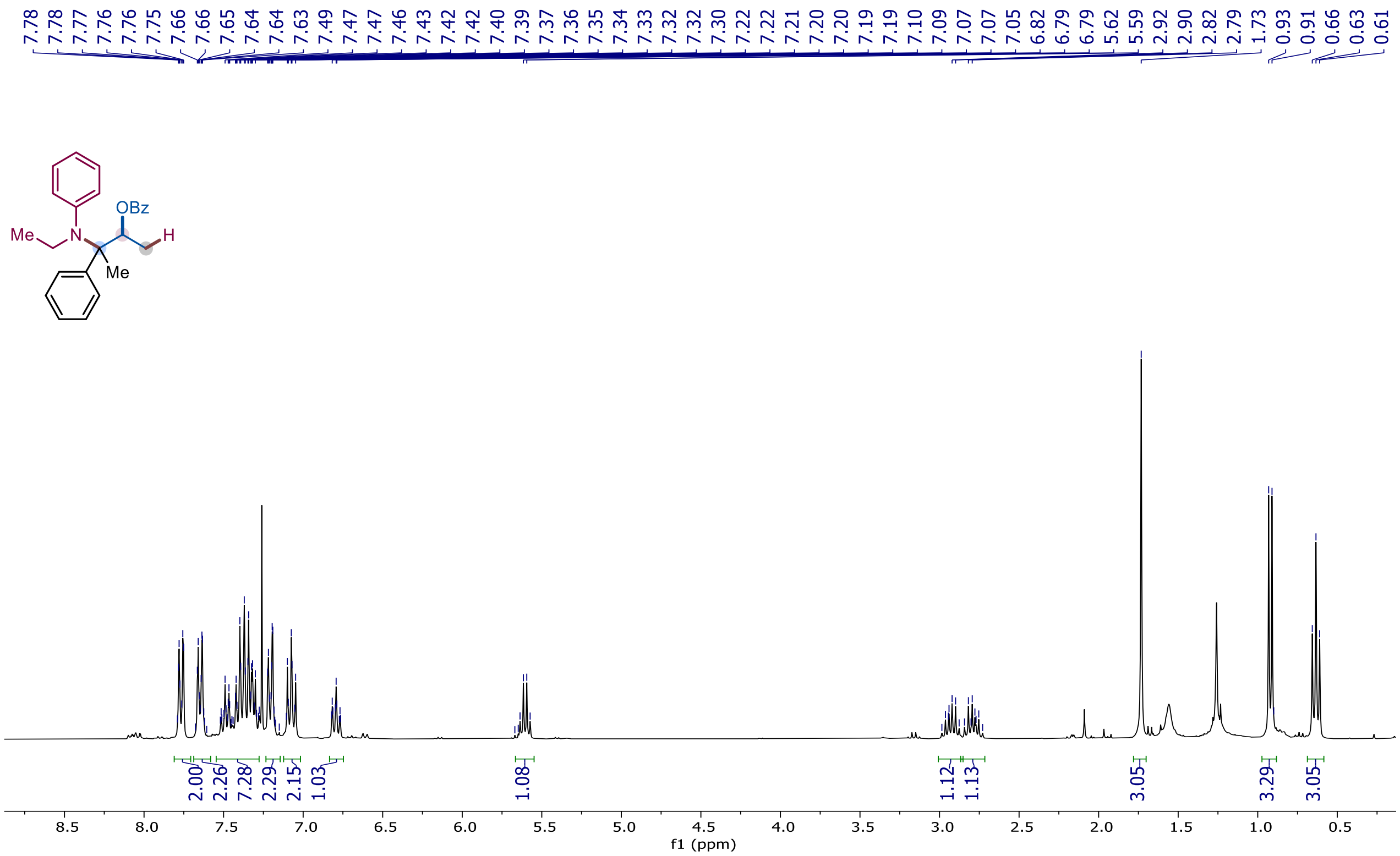

^{13}C NMR (126 MHz, $CDCl_3$, 25 ºC) of 3aaae

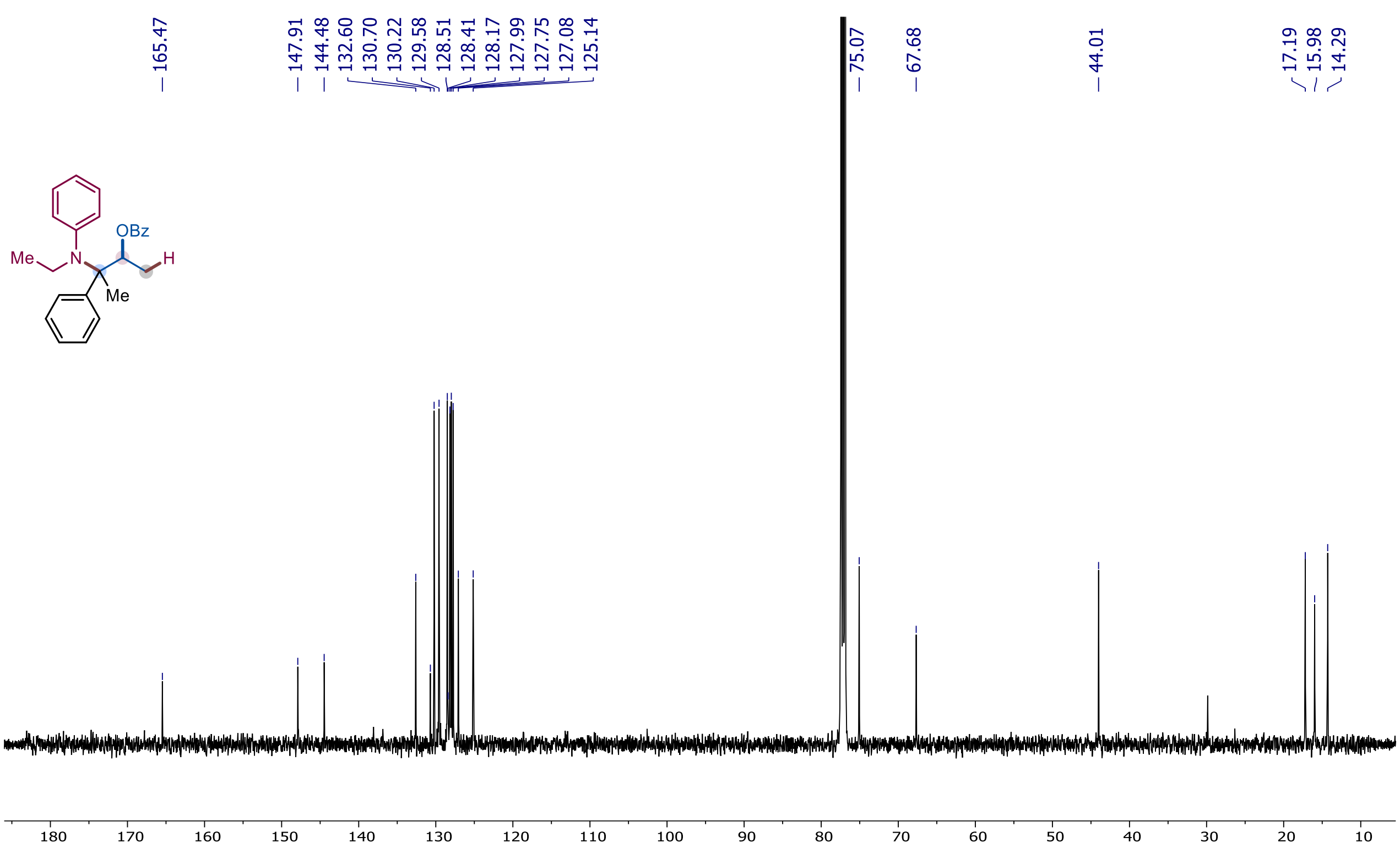

^{1}H NMR (800 MHz, $CDCl_3$, 25 ºC) of 3aaaf

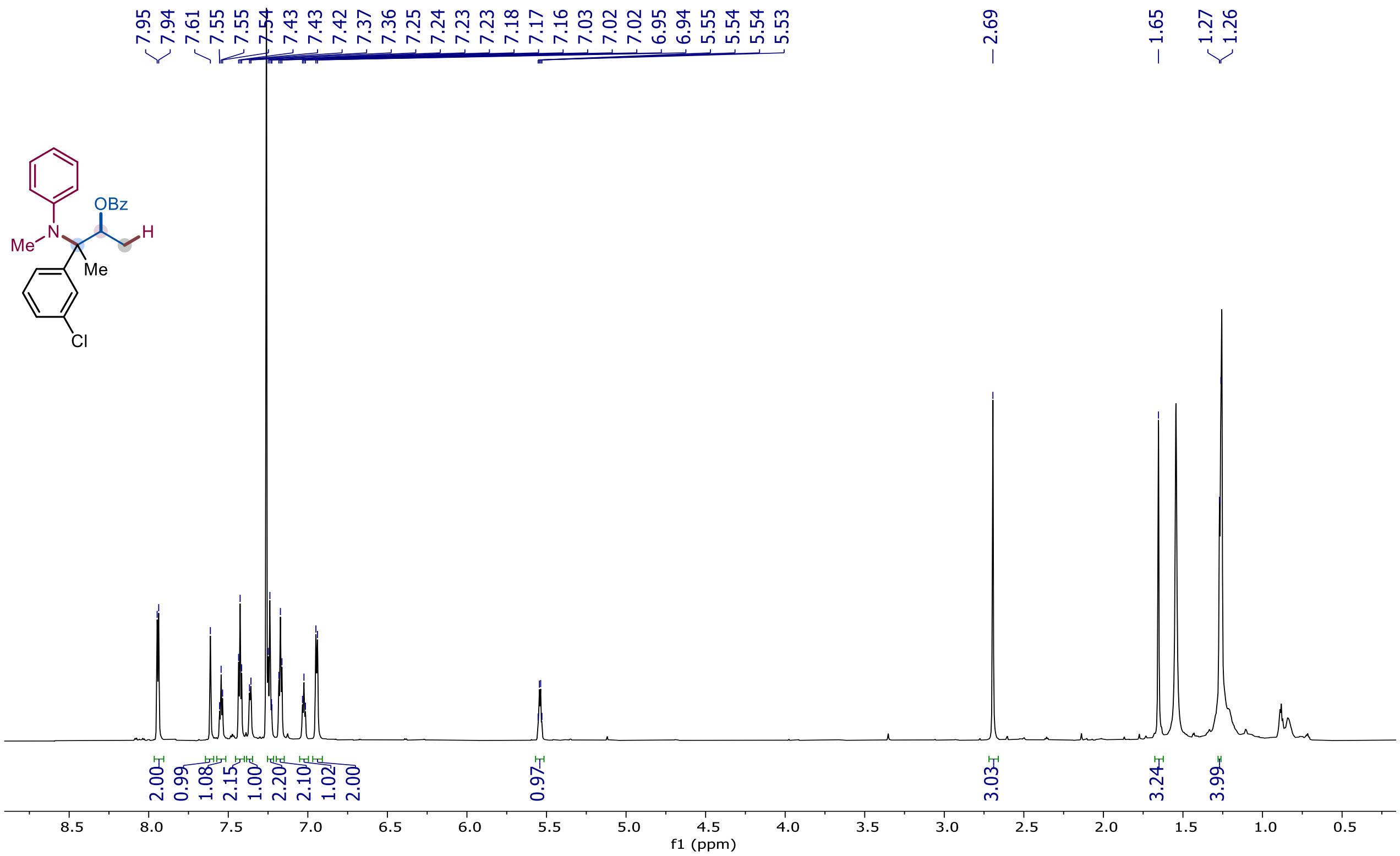

^{13}C NMR (201 MHz, $CDCl_3$, 25 ºC) of 3aaaf

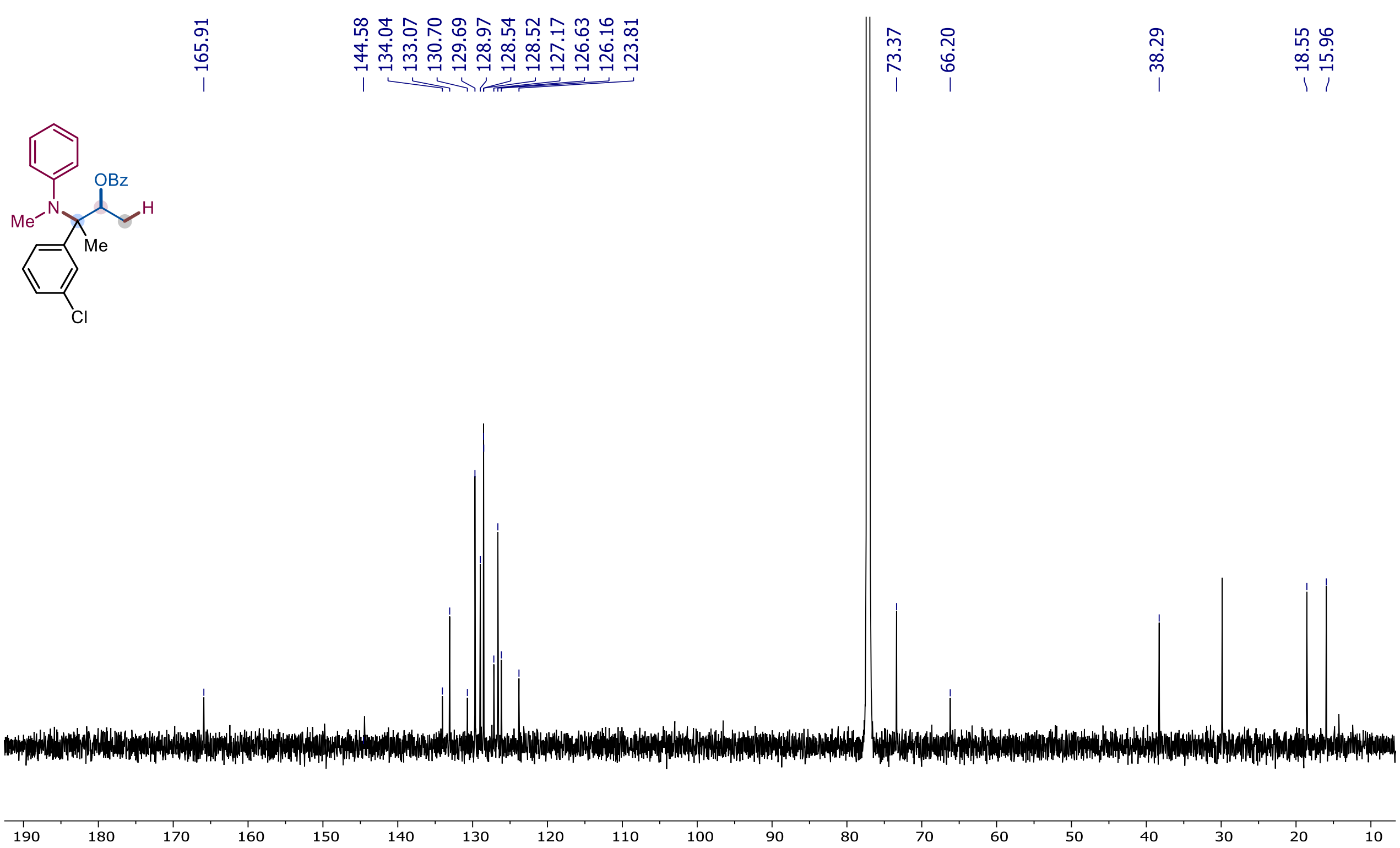

^{1}H NMR (500 MHz, CDCl$_3$, 25 ºC) of 3aaag

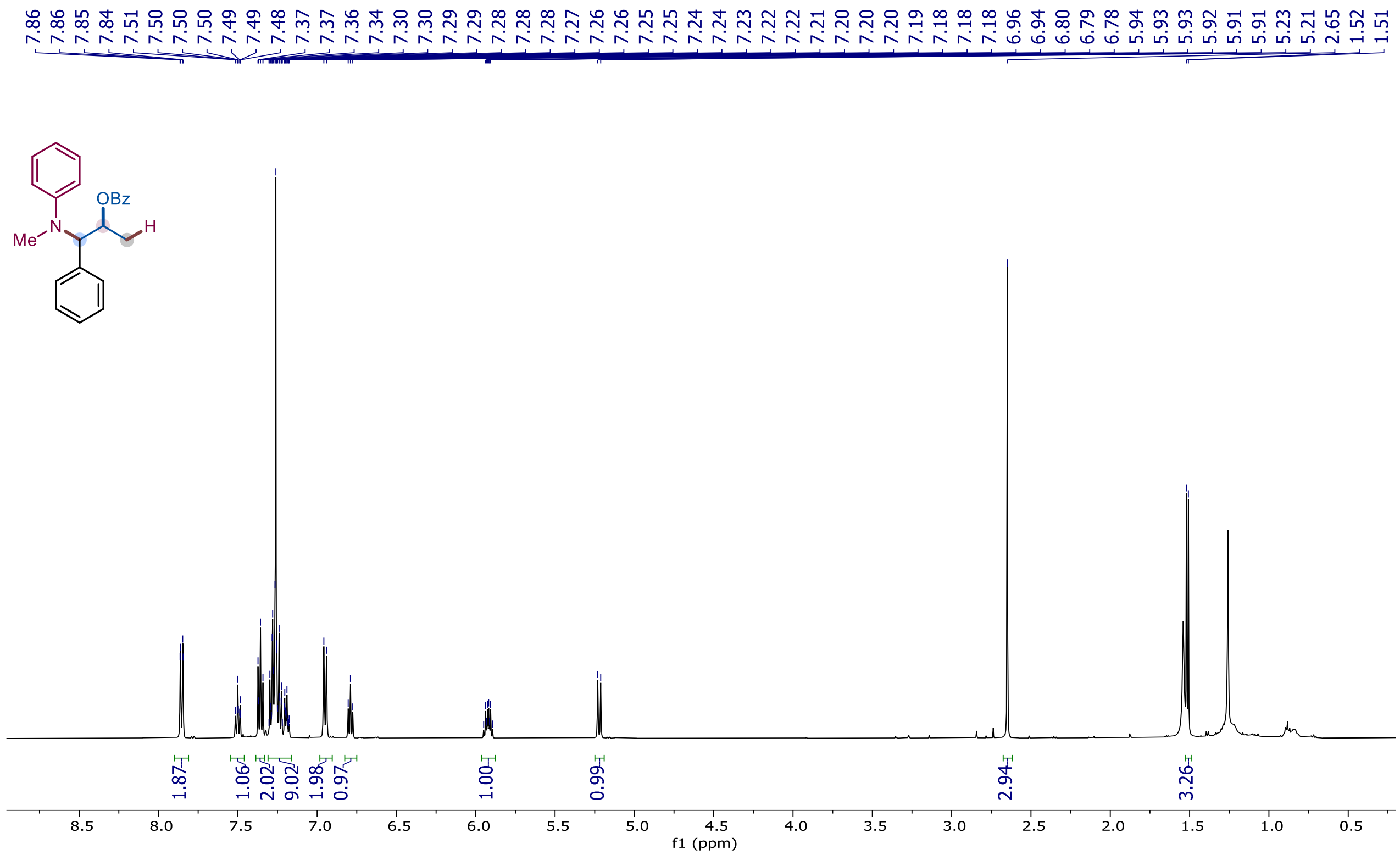

^{13}C NMR (201 MHz, CDCl$_3$, 25 ºC) of 3aaag

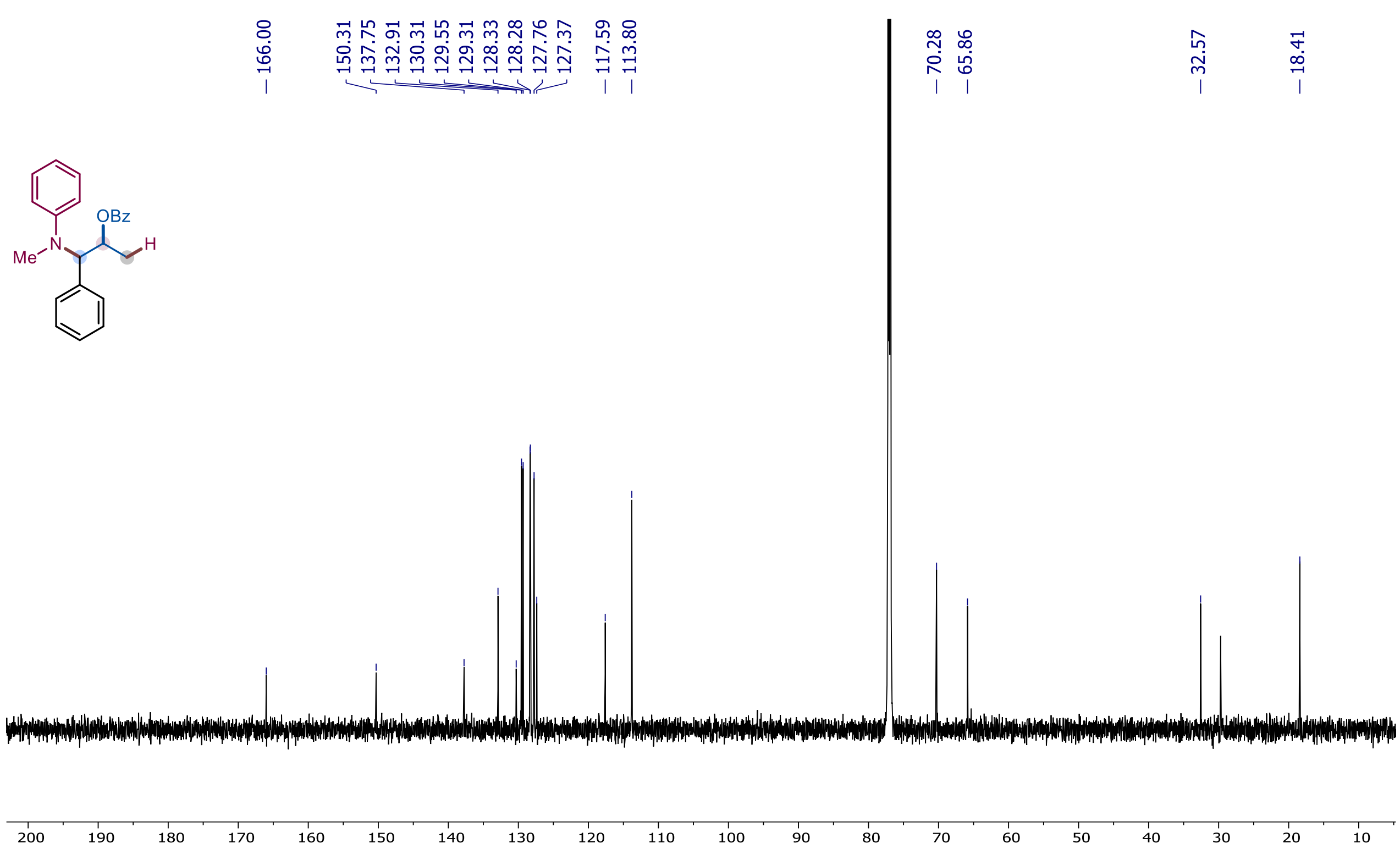

^{1}H NMR (500 MHz, $CDCl_3$, 25 °C) of 5a

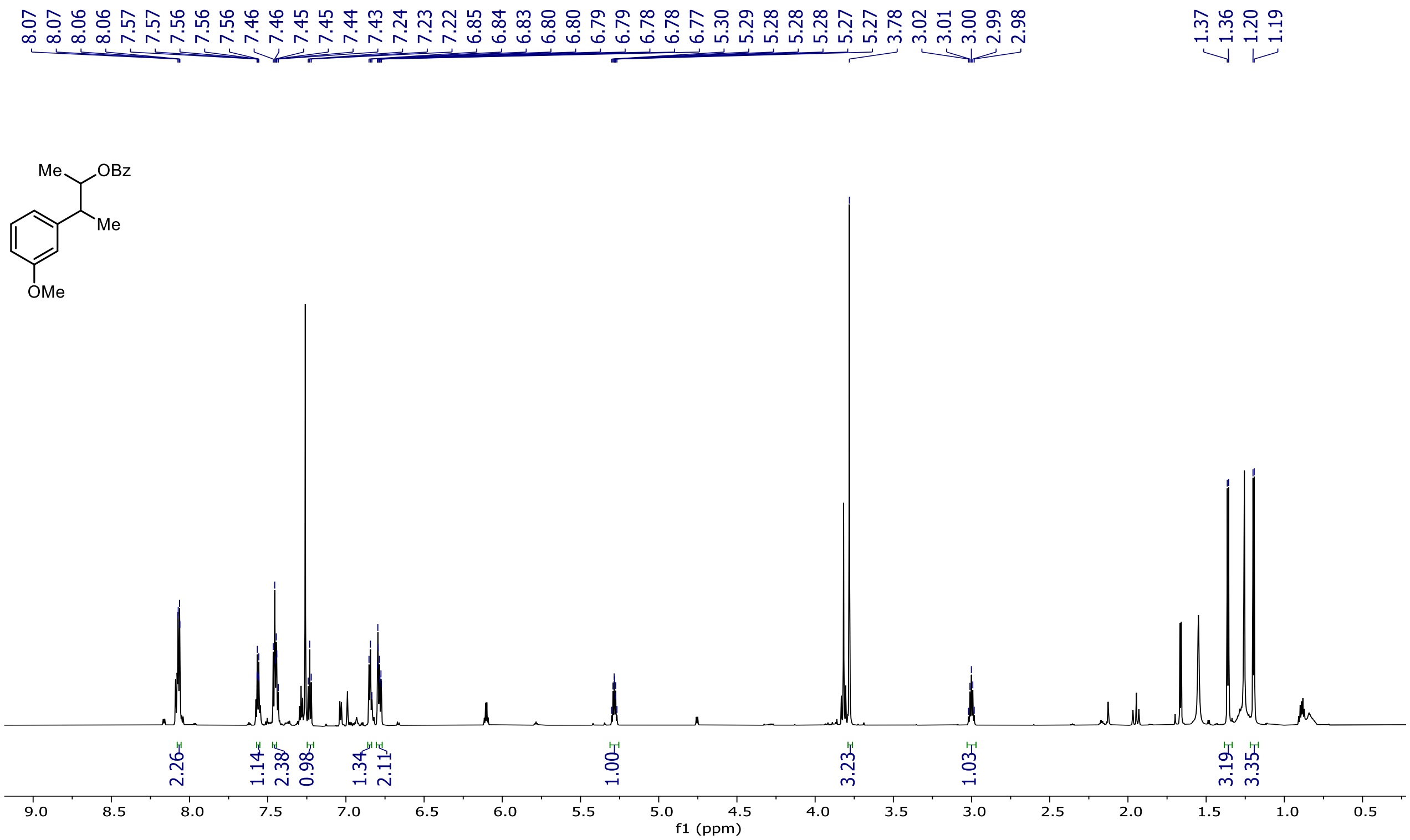

^{13}C NMR (201 MHz, $CDCl_3$, 25 °C) of 5a

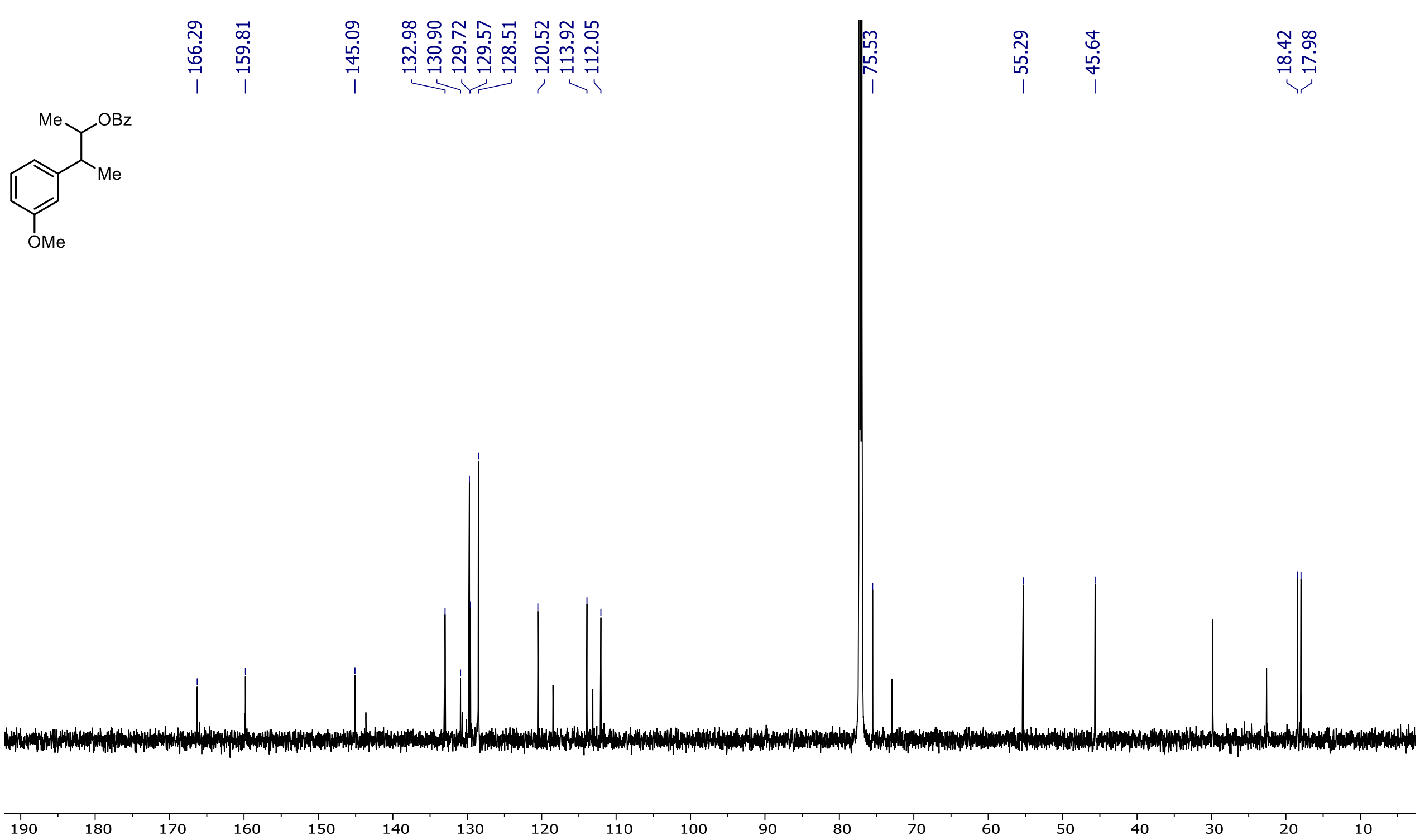

Cartesian Coordinates

1a

O	0.457614	-0.737713	1.593710
C	0.925307	-0.012011	0.738640
O	0.245332	0.980833	0.117559
C	-2.573411	2.472940	-1.291022
C	-1.518829	2.370901	-0.484494
C	-1.182912	1.192353	0.413606
C	-1.327980	1.639207	1.877750
C	2.328240	-0.112672	0.237459
C	2.828417	0.720561	-0.773379
C	4.146656	0.575893	-1.204148
C	4.969353	-0.397347	-0.630358
C	4.472307	-1.229527	0.377231
C	3.155901	-1.088401	0.810079
H	-2.754384	3.380381	-1.860504
H	-3.288464	1.664587	-1.410623
H	-0.818505	3.199931	-0.386787
H	-1.109753	0.818154	2.561154
H	-0.627565	2.458236	2.068833
H	-2.340085	2.012891	2.057452
H	2.182958	1.472134	-1.213651
H	4.532650	1.221898	-1.987938
H	5.996405	-0.507646	-0.968419
H	5.111053	-1.986969	0.823051
H	2.746361	-1.723426	1.588783
C	-1.973823	-0.059351	0.045618
C	-3.051742	-0.512670	0.811279
C	-1.649679	-0.745242	-1.133995
C	-3.785819	-1.632931	0.412340
H	-3.324696	-0.003054	1.728855
C	-2.374932	-1.866898	-1.529146
H	-0.817076	-0.395156	-1.736326

C	-3.449477	-2.315416	-0.756208
H	-4.617908	-1.973193	1.023036
H	-2.102439	-2.390525	-2.441687
H	-4.017575	-3.189535	-1.062890

III

O	0.504610	-0.689999	1.593721
C	0.960220	0.014866	0.714437
O	0.261643	0.965623	0.052472
C	-2.995794	2.605644	-0.856016
C	-1.569766	2.222140	-0.651867
C	-1.172234	1.146194	0.326250
C	-1.345565	1.655016	1.769975
C	2.370462	-0.068984	0.229903
C	2.859295	0.734666	-0.809879
C	4.185226	0.607693	-1.222246
C	5.027321	-0.318769	-0.601151
C	4.541760	-1.121691	0.435319
C	3.217686	-0.997889	0.849776
H	-3.132880	3.121302	-1.813398
H	-3.654969	1.729987	-0.838526
H	-3.357597	3.295084	-0.071878
H	-0.779240	2.901232	-0.960182
H	-1.085326	0.882244	2.495015
H	-0.691417	2.519671	1.916448
H	-2.378134	1.977983	1.934934
H	2.199045	1.449837	-1.287216
H	4.562137	1.230967	-2.028572
H	6.060363	-0.415444	-0.924855
H	5.195449	-1.842930	0.918028
H	2.816889	-1.611017	1.650260
C	-1.918459	-0.154361	0.011581
C	-2.896364	-0.691499	0.852213
C	-1.647053	-0.795455	-1.205765

C	-3.586599	-1.850979	0.487191
H	-3.121653	-0.219727	1.802439
C	-2.326393	-1.956556	-1.566806
H	-0.889192	-0.378365	-1.862582
C	-3.303415	-2.488457	-0.720224
H	-4.341648	-2.257517	1.154900
H	-2.096408	-2.445327	-2.509949
H	-3.837410	-3.392433	-1.000559

IV

C	-0.649672	2.654357	-1.547870
C	-0.605289	1.237804	-0.973997
C	-1.259828	1.974569	1.384199
C	-1.503051	1.052161	0.220086
O	0.794596	1.026617	-0.587657
C	1.256028	-0.242798	-0.622457
O	0.604432	-1.191893	-1.019373
C	2.660159	-0.346643	-0.130588
C	3.262137	-1.612560	-0.132243
C	4.571168	-1.763876	0.319173
C	5.285946	-0.651905	0.774491
C	4.688642	0.611679	0.777605
C	3.378523	0.767237	0.326803
H	0.036396	2.736071	-2.397139
H	-1.664296	2.882331	-1.890593
H	-0.360418	3.397056	-0.798367
H	-0.839298	0.523479	-1.762015
H	-0.226906	2.336327	1.377091
H	-1.427798	1.468583	2.341149
H	-1.917079	2.858827	1.367194
H	2.686688	-2.460290	-0.489312
H	5.034863	-2.746416	0.317197
H	6.307263	-0.769978	1.126934
H	5.244267	1.475635	1.131785

H	2.907521	1.743738	0.325596
C	-2.590678	0.120909	0.220686
C	-2.676557	-0.962532	-0.701946
C	-3.647829	0.242226	1.169086
C	-3.752128	-1.839802	-0.681748
H	-1.854990	-1.150083	-1.382956
C	-4.722292	-0.637355	1.173959
H	-3.631652	1.055900	1.887063
C	-4.788941	-1.684262	0.246999
H	-3.777695	-2.665238	-1.388652
H	-5.518027	-0.505961	1.903030
H	-5.629082	-2.373026	0.254804

V

C	-2.957621	-1.316964	2.060216
H	-3.648978	-1.442462	2.899383
N	-1.684530	-1.376977	2.308520
C	-3.538447	-1.134265	0.770586
C	-4.926426	-0.867659	0.688870
C	-2.746758	-1.311729	-0.409601
H	-5.461234	-0.710227	1.619954
C	-5.566564	-0.772453	-0.526795
C	-3.435549	-1.360807	-1.676662
C	-4.787430	-1.057070	-1.681631
H	-5.300426	-1.042122	-2.635304
C	2.164962	-1.475689	2.514030
H	2.679908	-1.636204	3.466762
N	0.873581	-1.292543	2.557485
C	2.981405	-1.505206	1.351079
C	2.417626	-1.443442	0.030702
C	4.381409	-1.653803	1.546195
H	4.744647	-1.674817	2.569360
C	3.314299	-1.633176	-1.086092
C	5.246950	-1.762204	0.484873

C	4.663323	-1.765251	-0.812050
H	5.339396	-1.882506	-1.649194
O	-1.453133	-1.453220	-0.362313
O	1.147495	-1.250074	-0.174499
C	-2.693005	-1.780317	-2.959937
C	-7.046595	-0.397427	-0.683673
C	2.786510	-1.681693	-2.531908
C	6.769646	-1.889084	0.628422
C	-2.129235	-3.208932	-2.758577
H	-1.580284	-3.526697	-3.653674
H	-2.942761	-3.924193	-2.585860
H	-1.445452	-3.244982	-1.908652
C	-1.532832	-0.811913	-3.282008
H	-0.782179	-0.828295	-2.495488
H	-1.900763	0.215114	-3.396635
H	-1.051481	-1.105280	-4.223239
C	-3.628234	-1.814198	-4.184917
H	-3.061341	-2.148856	-5.060804
H	-4.036777	-0.823311	-4.417830
H	-4.464361	-2.510319	-4.048142
C	1.711804	-2.788094	-2.663743
H	1.324887	-2.813600	-3.690188
H	0.875734	-2.611052	-1.987406
H	2.142290	-3.772397	-2.440192
C	3.903174	-1.998608	-3.547513
H	3.466877	-2.059403	-4.550810
H	4.389643	-2.959444	-3.340140
H	4.672368	-1.217582	-3.573095
C	2.184540	-0.311925	-2.916455
H	1.309857	-0.095399	-2.308939
H	1.875594	-0.317440	-3.969506
H	2.910992	0.493085	-2.764134
Co	-0.274226	-1.057628	1.068946
C	-7.156770	0.885273	-1.541432

H	-6.631761	1.719057	-1.060394
H	-8.207713	1.171781	-1.672718
H	-6.721529	0.747445	-2.536901
C	-7.810708	-1.547695	-1.379927
H	-7.751136	-2.467429	-0.786797
H	-7.404577	-1.764062	-2.373680
H	-8.869174	-1.286518	-1.502994
C	-7.720371	-0.123911	0.673551
H	-7.230505	0.698320	1.208436
H	-7.705957	-1.009941	1.318884
H	-8.768540	0.156408	0.519306
C	7.452346	-0.707992	-0.101015
H	7.134563	0.248674	0.328578
H	7.204964	-0.691606	-1.167682
H	8.543585	-0.781429	-0.012279
C	7.216252	-1.865768	2.101668
H	6.787627	-2.701082	2.667946
H	6.928440	-0.930509	2.595780
H	8.307254	-1.950868	2.161269
C	7.243136	-3.221983	0.002508
H	6.778792	-4.075699	0.509534
H	8.332279	-3.321486	0.090254
H	6.988613	-3.286091	-1.060716
C	-1.231172	-1.827743	3.627768
H	-1.226348	-2.925706	3.630289
H	-1.907116	-1.486507	4.422089
C	0.189224	-1.313478	3.852184
H	0.151979	-0.297433	4.255972
H	0.722581	-1.940367	4.577041
C	1.558763	1.873115	2.768451
C	1.110346	1.555260	1.344048
C	-0.311802	1.035345	0.969622
C	-0.468346	1.283861	-0.538524
O	1.293472	2.842233	0.653387

C	2.253718	2.923639	-0.290483
O	3.045288	2.033535	-0.547369
C	2.230232	4.238220	-0.995927
C	3.192311	4.466718	-1.989083
C	3.204274	5.673122	-2.685339
C	2.254821	6.657224	-2.393649
C	1.293654	6.432029	-1.404337
C	1.278728	5.226013	-0.704554
H	2.617597	2.149001	2.726006
H	1.467242	1.021645	3.440727
H	1.005100	2.720798	3.178920
H	1.825241	0.869020	0.899311
H	-1.312872	0.756179	-0.965244
H	0.421768	0.945921	-1.062209
H	-0.596224	2.354755	-0.735450
H	3.916061	3.685989	-2.198930
H	3.950683	5.847196	-3.455524
H	2.263341	7.598127	-2.937768
H	0.554816	7.196258	-1.178997
H	0.536675	5.040896	0.063886
C	-1.492171	1.552869	1.753807
C	-2.692086	1.896857	1.096770
C	-1.507175	1.666639	3.160918
C	-3.828256	2.312457	1.793480
H	-2.760961	1.822552	0.020112
C	-2.636617	2.077123	3.862699
H	-0.627163	1.410972	3.729026
C	-3.814745	2.401378	3.183145
H	-4.731152	2.550873	1.236984
H	-2.595185	2.145638	4.947304
H	-4.698898	2.720455	3.728581

VI

C	-2.957621	-1.316964	2.060216

H	-3.648978	-1.442462	2.899383
N	-1.684530	-1.376977	2.308520
C	-3.538447	-1.134265	0.770586
C	-4.926426	-0.867659	0.688870
C	-2.746758	-1.311729	-0.409601
H	-5.461234	-0.710227	1.619954
C	-5.566564	-0.772453	-0.526795
C	-3.435549	-1.360807	-1.676662
C	-4.787430	-1.057070	-1.681631
H	-5.300426	-1.042122	-2.635304
C	2.164962	-1.475689	2.514030
H	2.679908	-1.636204	3.466762
N	0.873581	-1.292543	2.557485
C	2.981405	-1.505206	1.351079
C	2.417626	-1.443442	0.030702
C	4.381409	-1.653803	1.546195
H	4.744647	-1.674817	2.569360
C	3.314299	-1.633176	-1.086092
C	5.246950	-1.762204	0.484873
C	4.663323	-1.765251	-0.812050
H	5.339396	-1.882506	-1.649194
O	-1.453133	-1.453220	-0.362313
O	1.147495	-1.250074	-0.174499
C	-2.693005	-1.780317	-2.959937
C	-7.046595	-0.397427	-0.683673
C	2.786510	-1.681693	-2.531908
C	6.769646	-1.889084	0.628422
C	-2.129235	-3.208932	-2.758577
H	-1.580284	-3.526697	-3.653674
H	-2.942761	-3.924193	-2.585860
H	-1.445452	-3.244982	-1.908652
C	-1.532832	-0.811913	-3.282008
H	-0.782179	-0.828295	-2.495488
H	-1.900763	0.215114	-3.396635

H	-1.051481	-1.105280	-4.223239
C	-3.628234	-1.814198	-4.184917
H	-3.061341	-2.148856	-5.060804
H	-4.036777	-0.823311	-4.417830
H	-4.464361	-2.510319	-4.048142
C	1.711804	-2.788094	-2.663743
H	1.324887	-2.813600	-3.690188
H	0.875734	-2.611052	-1.987406
H	2.142290	-3.772397	-2.440192
C	3.903174	-1.998608	-3.547513
H	3.466877	-2.059403	-4.550810
H	4.389643	-2.959444	-3.340140
H	4.672368	-1.217582	-3.573095
C	2.184540	-0.311925	-2.916455
H	1.309857	-0.095399	-2.308939
H	1.875594	-0.317440	-3.969506
H	2.910992	0.493085	-2.764134
Co	-0.274226	-1.057628	1.068946
C	-7.156770	0.885273	-1.541432
H	-6.631761	1.719057	-1.060394
H	-8.207713	1.171781	-1.672718
H	-6.721529	0.747445	-2.536901
C	-7.810708	-1.547695	-1.379927
H	-7.751136	-2.467429	-0.786797
H	-7.404577	-1.764062	-2.373680
H	-8.869174	-1.286518	-1.502994
C	-7.720371	-0.123911	0.673551
H	-7.230505	0.698320	1.208436
H	-7.705957	-1.009941	1.318884
H	-8.768540	0.156408	0.519306
C	7.452346	-0.707992	-0.101015
H	7.134563	0.248674	0.328578
H	7.204964	-0.691606	-1.167682
H	8.543585	-0.781429	-0.012279

C	7.216252	-1.865768	2.101668
H	6.787627	-2.701082	2.667946
H	6.928440	-0.930509	2.595780
H	8.307254	-1.950868	2.161269
C	7.243136	-3.221983	0.002508
H	6.778792	-4.075699	0.509534
H	8.332279	-3.321486	0.090254
H	6.988613	-3.286091	-1.060716
C	-1.231172	-1.827743	3.627768
H	-1.226348	-2.925706	3.630289
H	-1.907116	-1.486507	4.422089
C	0.189224	-1.313478	3.852184
H	0.151979	-0.297433	4.255972
H	0.722581	-1.940367	4.577041
C	1.558763	1.873115	2.768451
C	1.110346	1.555260	1.344048
C	-0.311802	1.035345	0.969622
C	-0.468346	1.283861	-0.538524
O	1.293472	2.842233	0.653387
C	2.253718	2.923639	-0.290483
O	3.045288	2.033535	-0.547369
C	2.230232	4.238220	-0.995927
C	3.192311	4.466718	-1.989083
C	3.204274	5.673122	-2.685339
C	2.254821	6.657224	-2.393649
C	1.293654	6.432029	-1.404337
C	1.278728	5.226013	-0.704554
H	2.617597	2.149001	2.726006
H	1.467242	1.021645	3.440727
H	1.005100	2.720798	3.178920
H	1.825241	0.869020	0.899311
H	-1.312872	0.756179	-0.965244
H	0.421768	0.945921	-1.062209
H	-0.596224	2.354755	-0.735450

H	3.916061	3.685989	-2.198930
H	3.950683	5.847196	-3.455524
H	2.263341	7.598127	-2.937768
H	0.554816	7.196258	-1.178997
H	0.536675	5.040896	0.063886
C	-1.492171	1.552869	1.753807
C	-2.692086	1.896857	1.096770
C	-1.507175	1.666639	3.160918
C	-3.828256	2.312457	1.793480
H	-2.760961	1.822552	0.020112
C	-2.636617	2.077123	3.862699
H	-0.627163	1.410972	3.729026
C	-3.814745	2.401378	3.183145
H	-4.731152	2.550873	1.236984
H	-2.595185	2.145638	4.947304
H	-4.698898	2.720455	3.728581

TS1

C	1.073354	-2.113082	-2.480245
H	1.690881	-2.211833	-3.378554
N	-0.066951	-1.505043	-2.593184
C	1.618847	-2.635668	-1.267571
C	2.969490	-3.066908	-1.294714
C	0.848100	-2.686233	-0.058387
H	3.498217	-2.996579	-2.241016
C	3.605893	-3.526792	-0.162333
C	1.495310	-3.212837	1.120657
C	2.823658	-3.592461	1.021355
H	3.314272	-3.960329	1.913930
C	-3.303502	0.569960	-2.293865
H	-3.662679	1.198466	-3.115950
N	-2.144437	-0.002240	-2.434803
C	-4.155238	0.464674	-1.152828
C	-3.756391	-0.259665	0.020838

C	-5.410453	1.124660	-1.211419
H	-5.654356	1.653259	-2.128387
C	-4.663637	-0.262569	1.144597
C	-6.292816	1.100168	-0.154729
C	-5.872699	0.397012	1.006449
H	-6.555431	0.386196	1.847082
O	-0.389698	-2.288547	0.004323
O	-2.624310	-0.896862	0.108888
C	0.725424	-3.308016	2.449677
C	5.086456	-3.928581	-0.117207
C	-4.266779	-0.954683	2.461614
C	-7.665446	1.788334	-0.167810
C	-0.510958	-4.222543	2.275415
H	-1.071753	-4.279416	3.216802
H	-0.200021	-5.238899	2.003809
H	-1.176495	-3.841762	1.500889
C	0.279347	-1.893386	2.888101
H	-0.339887	-1.428449	2.121816
H	1.155022	-1.258319	3.074077
H	-0.301697	-1.948790	3.816784
C	1.584631	-3.898785	3.584786
H	0.985248	-3.948493	4.500838
H	2.464304	-3.280792	3.801875
H	1.924309	-4.916350	3.357310
C	-3.964876	-2.453249	2.220272
H	-3.688371	-2.936192	3.166097
H	-3.145227	-2.581518	1.514281
H	-4.852303	-2.964274	1.826543
C	-5.380416	-0.872120	3.524518
H	-5.045043	-1.377935	4.436986
H	-6.301458	-1.366519	3.192965
H	-5.621145	0.163386	3.793554
C	-3.016670	-0.249152	3.040084
H	-2.187406	-0.286696	2.333801

H	-2.697393	-0.740062	3.967869
H	-3.239302	0.800444	3.269451
Co	-1.277361	-1.128264	-1.192172
H	-0.445196	0.046420	-0.645631
C	5.831823	-3.003230	0.874105
H	5.755665	-1.956139	0.558616
H	6.894779	-3.270138	0.925584
H	5.419926	-3.077273	1.886209
C	5.218840	-5.396706	0.349856
H	4.705432	-6.072502	-0.343727
H	4.787391	-5.548390	1.344914
H	6.274788	-5.690624	0.397111
C	5.764040	-3.798994	-1.493680
H	5.736851	-2.766744	-1.862046
H	5.288577	-4.444962	-2.241117
H	6.816376	-4.095555	-1.419758
C	-7.724523	2.848825	0.956915
H	-6.952462	3.612935	0.810242
H	-7.569367	2.402821	1.945077
H	-8.702568	3.346180	0.965886
C	-7.947618	2.494353	-1.506682
H	-7.943053	1.787725	-2.344804
H	-7.210318	3.278841	-1.713778
H	-8.935557	2.967900	-1.477063
C	-8.774618	0.735287	0.063491
H	-8.760464	-0.023022	-0.727735
H	-9.762995	1.211848	0.065008
H	-8.652589	0.219732	1.021983
C	-0.510172	-0.988991	-3.890383
H	-1.165099	-1.732073	-4.364383
H	0.336783	-0.786325	-4.556425
C	-1.303841	0.283114	-3.599218
H	-0.603015	1.088065	-3.341456
H	-1.896688	0.599399	-4.467109

O	4.862932	2.685997	-0.210237
C	4.056966	3.170354	0.560084
O	2.838193	2.644416	0.827941
C	-0.179154	1.308367	0.076168
C	1.023657	1.184607	0.728849
C	2.405212	1.406284	0.142649
C	3.315662	0.248683	0.581044
C	4.287369	4.433794	1.321863
C	3.329303	4.971433	2.193352
C	3.599385	6.156803	2.876106
C	4.821991	6.808857	2.693265
C	5.778318	6.274291	1.824613
C	5.512500	5.090346	1.140599
H	-0.248643	1.882568	-0.844119
H	-1.093727	1.246339	0.659773
H	1.026712	0.789209	1.741568
H	2.882297	-0.710968	0.290784
H	3.395046	0.261110	1.672432
H	4.313591	0.346986	0.152748
H	2.384017	4.458227	2.329284
H	2.856447	6.572142	3.551483
H	5.029435	7.732494	3.227265
H	6.728886	6.780855	1.682095
H	6.237930	4.655882	0.460593
C	2.364133	1.644486	-1.363789
C	1.871531	2.860575	-1.860761
C	2.751694	0.657716	-2.274809
C	1.750567	3.077191	-3.232977
H	1.590979	3.641631	-1.160499
C	2.635474	0.873776	-3.650064
H	3.146958	-0.285267	-1.917698
C	2.128501	2.079504	-4.136420
H	1.369610	4.027811	-3.596721
H	2.953935	0.097859	-4.342146

H	2.042550	2.247718	-5.206660

TS3

C	-5.017165	-1.097626	0.609879
C	-4.142287	-0.683826	1.618075
C	-2.890442	-0.164463	1.288050
C	-2.507746	-0.058935	-0.055354
C	-3.385676	-0.474658	-1.063969
C	-4.636782	-0.992229	-0.731124
H	-4.435762	-0.766277	2.661388
H	-2.202352	0.159479	2.061367
H	-3.065591	-0.384918	-2.096542
H	-5.315390	-1.314333	-1.516768
C	-1.154655	0.495885	-0.464527
O	-0.449304	0.915765	0.537700
H	-5.992600	-1.501804	0.868473
O	-0.830355	0.517313	-1.659234
C	1.927246	1.261274	0.269568
C	1.433440	1.936801	-0.828816
C	0.819930	3.293321	-0.879580
H	1.512023	1.444517	-1.791104
H	-0.122285	3.220853	-1.434627
H	0.618436	3.726409	0.101106
C	1.986087	1.881620	1.640133
H	3.002178	2.231712	1.865462
H	1.308352	2.729804	1.731532
H	1.707811	1.143664	2.397724
H	1.470565	3.980390	-1.439807
C	2.509351	-0.083769	0.097968
C	2.045662	-0.957444	-0.908793
C	3.539309	-0.532659	0.948409
C	2.611416	-2.219208	-1.066449
H	1.202900	-0.662308	-1.525756
C	4.111377	-1.791483	0.777372

H	3.912909	0.112675	1.736885
C	3.651025	-2.640106	-0.231740
H	2.229074	-2.883368	-1.836732
H	4.915532	-2.110647	1.434807
H	4.090938	-3.625444	-0.359735

TS2

C	-4.950166	-1.193377	-0.052977
C	-4.030439	-1.467447	0.963020
C	-2.766531	-0.878312	0.939353
C	-2.416577	-0.010597	-0.103061
C	-3.340421	0.263158	-1.119483
C	-4.603042	-0.327442	-1.093932
H	-4.298639	-2.140518	1.773239
H	-2.042718	-1.080680	1.721173
H	-3.049060	0.937482	-1.917617
H	-5.316833	-0.113316	-1.885122
C	-1.051190	0.641670	-0.155704
O	-0.277314	0.351034	0.830830
H	-5.934849	-1.653483	-0.033639
O	-0.778754	1.393213	-1.110682
C	1.625624	1.065729	0.549854
C	1.402346	2.075906	-0.404177
C	0.968200	3.468023	-0.082157
H	1.612804	1.846219	-1.441348
H	0.516862	3.937773	-0.958872
H	0.232739	3.483775	0.728212
C	1.759053	1.411449	2.015114
H	2.780083	1.768059	2.206529
H	1.054107	2.187681	2.317658
H	1.589472	0.530878	2.637598
H	1.823650	4.084019	0.236031
C	2.305908	-0.189371	0.108307
C	2.003846	-1.424683	0.707086

C	3.311455	-0.149730	-0.871337
C	2.673934	-2.583364	0.323091
H	1.209208	-1.473077	1.442412
C	3.988948	-1.310181	-1.248927
H	3.586312	0.797370	-1.326067
C	3.670709	-2.531814	-0.655713
H	2.413731	-3.531825	0.785332
H	4.767338	-1.255285	-2.005162
H	4.194399	-3.436749	-0.951678

Co-III-H-singlet

C	2.572140	-2.526128	-0.188422
H	3.178818	-3.433647	-0.275730
N	1.278224	-2.661860	-0.218568
C	3.277633	-1.292869	-0.079137
C	4.692650	-1.346651	0.019249
C	2.588599	-0.036492	-0.136643
H	5.155199	-2.328307	0.061268
C	5.456416	-0.202039	0.059713
C	3.384223	1.166871	-0.136941
C	4.757752	1.032919	-0.029092
H	5.354998	1.936143	-0.013272
C	-2.569270	-2.526091	0.081026
H	-3.174915	-3.437538	0.120062
N	-1.274854	-2.665265	0.092513
C	-3.277211	-1.290945	0.037108
C	-2.588111	-0.033098	0.064561
C	-4.695475	-1.346051	-0.007360
H	-5.157686	-2.328516	-0.030684
C	-3.386175	1.169319	0.092278
C	-5.462192	-0.203264	-0.020297
C	-4.762266	1.033118	0.037844
H	-5.361217	1.935277	0.048204
O	1.290717	0.049706	-0.219284

O	-1.289178	0.061007	0.059119
C	2.711632	2.545419	-0.267235
C	6.987147	-0.197380	0.180471
C	-2.712215	2.548178	0.213898
C	-6.996577	-0.201403	-0.077993
C	1.974669	2.625835	-1.626462
H	1.466434	3.593228	-1.723159
H	2.687383	2.531655	-2.455163
H	1.227806	1.836489	-1.717806
C	1.711486	2.762521	0.892720
H	0.934261	2.000072	0.889677
H	2.230490	2.732533	1.858842
H	1.232969	3.745150	0.795846
C	3.732758	3.699476	-0.223726
H	3.201526	4.652796	-0.322484
H	4.283118	3.726219	0.724546
H	4.458462	3.641880	-1.043975
C	-1.745693	2.785786	-0.970764
H	-1.279275	3.774809	-0.879757
H	-0.957288	2.035164	-0.993752
H	-2.289720	2.755054	-1.923124
C	-3.736651	3.700273	0.213520
H	-3.204720	4.653621	0.308058
H	-4.314601	3.736513	-0.717955
H	-4.437854	3.631812	1.053885
C	-1.938852	2.611139	1.553784
H	-1.198650	1.813088	1.620516
H	-1.417760	3.572163	1.644703
H	-2.631199	2.517793	2.399551
Co	0.008680	-1.289556	0.035916
H	0.161826	-1.359093	1.457247
C	7.401366	0.564848	1.461244
H	6.985237	0.081124	2.352352
H	8.493966	0.584781	1.559706

H	7.049512	1.601880	1.449294
C	7.602853	0.502931	-1.053813
H	7.330238	-0.024265	-1.975277
H	7.259794	1.538705	-1.147784
H	8.697352	0.520336	-0.979105
C	7.566797	-1.621469	0.261871
H	7.185152	-2.164020	1.134665
H	7.333136	-2.205417	-0.636092
H	8.658031	-1.574522	0.351440
C	-7.562069	0.496961	1.181184
H	-7.250268	-0.030155	2.090144
H	-7.217454	1.533373	1.261435
H	-8.658767	0.512211	1.151983
C	-7.576413	-1.626546	-0.136412
H	-7.229389	-2.168088	-1.024198
H	-7.305513	-2.210320	0.751107
H	-8.670467	-1.581638	-0.181743
C	-7.464914	0.560786	-1.339959
H	-7.085482	0.078212	-2.247953
H	-8.560682	0.578987	-1.392957
H	-7.114648	1.598410	-1.342083
C	0.667896	-3.974307	-0.446686
H	0.484691	-4.093433	-1.523122
H	1.320245	-4.792207	-0.115553
C	-0.665759	-3.980404	0.298270
H	-0.483208	-4.110688	1.372788
H	-1.317176	-4.792944	-0.047424

Co-III-triplet

C	-2.571810	-2.547801	0.112500
H	-3.178535	-3.455690	0.157531
N	-1.275821	-2.685163	0.154261
C	-3.276883	-1.309619	0.042345
C	-4.683736	-1.353567	-0.038013

C	-2.584857	-0.044296	0.112251
H	-5.161609	-2.325849	-0.088856
C	-5.441563	-0.193192	-0.049439
C	-3.365561	1.171505	0.150386
C	-4.739619	1.039956	0.054779
H	-5.336117	1.941985	0.065014
C	2.571813	-2.547807	-0.112466
H	3.178585	-3.455702	-0.156738
N	1.275870	-2.685148	-0.155416
C	3.276846	-1.309593	-0.042128
C	2.584872	-0.044277	-0.112658
C	4.683647	-1.353538	0.039001
H	5.161496	-2.325805	0.090447
C	3.365605	1.171504	-0.150675
C	5.441492	-0.193175	0.050437
C	4.739624	1.039958	-0.054450
H	5.336133	1.941979	-0.064678
O	-1.289029	0.025475	0.150255
O	1.289064	0.025488	-0.151450
C	-2.688503	2.542142	0.308077
C	-6.969858	-0.182872	-0.151838
C	2.688616	2.542136	-0.308660
C	6.969755	-0.182883	0.153308
C	-1.935590	2.579428	1.661913
H	-1.429796	3.544594	1.777434
H	-2.636766	2.463507	2.496481
H	-1.182995	1.792450	1.729201
C	-1.708095	2.792709	-0.863481
H	-0.913802	2.048429	-0.890920
H	-2.238718	2.777503	-1.822792
H	-1.246303	3.780208	-0.750910
C	-3.712732	3.694475	0.312665
H	-3.180823	4.643451	0.434615
H	-4.272763	3.752546	-0.627915

H	-4.425995	3.612802	1.140925
C	1.708015	2.792681	0.862737
H	1.245980	3.780042	0.749940
H	0.913901	2.048212	0.890188
H	2.238529	2.777755	1.822111
C	3.712842	3.694480	-0.313086
H	3.180961	4.643443	-0.435259
H	4.272642	3.752663	0.627623
H	4.426316	3.612732	-1.141161
C	1.935894	2.579410	-1.662597
H	1.183157	1.792573	-1.729912
H	1.430322	3.544672	-1.778282
H	2.637172	2.463263	-2.497048
Co	0.000004	-1.312357	-0.000661
C	-7.386793	0.611780	-1.414042
H	-6.984742	0.147178	-2.321245
H	-8.479124	0.632031	-1.496401
H	-7.038622	1.649407	-1.382154
C	-7.559792	0.499564	1.107075
H	-7.279616	-0.044127	2.016137
H	-7.219815	1.534916	1.213335
H	-8.653380	0.516022	1.042652
C	-7.556723	-1.602499	-0.255217
H	-7.193653	-2.130251	-1.144862
H	-7.323494	-2.206177	0.629656
H	-8.646994	-1.543670	-0.331924
C	7.559983	0.498487	-1.106050
H	7.279671	-0.045734	-2.014755
H	7.220329	1.533877	-1.213027
H	8.653576	0.514649	-1.041604
C	7.556387	-1.602504	0.257976
H	7.192871	-2.129533	1.147866
H	7.323441	-2.206838	-0.626531
H	8.646636	-1.543765	0.335057

C	7.386550	0.612676	1.414982
H	6.984351	0.148752	2.322464
H	8.478871	0.632896	1.497451
H	7.038495	1.650312	1.382318
C	-0.670536	-4.008858	0.365408
H	-0.504858	-4.140099	1.441791
H	-1.324220	-4.814538	0.013641
C	0.670610	-4.008826	-0.366608
H	0.504856	-4.139905	-1.443001
H	1.324326	-4.814549	-0.015013

Co-II-doublet

C	2.577703	2.533414	0.116772
H	3.184031	3.442776	0.184930
N	1.280212	2.670996	0.128736
C	3.283748	1.298935	0.047622
C	4.701159	1.348955	-0.034179
C	2.594723	0.043132	0.122345
H	5.165349	2.329232	-0.087919
C	5.463314	0.203441	-0.046297
C	3.388537	-1.161308	0.147697
C	4.762782	-1.030546	0.052221
H	5.359698	-1.934088	0.056310
C	-2.577708	2.533426	-0.116926
H	-3.184007	3.442782	-0.185348
N	-1.280211	2.670959	-0.128840
C	-3.283758	1.298951	-0.047547
C	-2.594732	0.043154	-0.122319
C	-4.701155	1.348962	0.034340
H	-5.165343	2.329236	0.088147
C	-3.388542	-1.161275	-0.147791
C	-5.463315	0.203441	0.046441
C	-4.762790	-1.030527	-0.052238
H	-5.359699	-1.934074	-0.056395

O	1.295334	-0.041815	0.186613
O	-1.295326	-0.041805	-0.186540
C	2.711671	-2.536787	0.290515
C	6.995606	0.195467	-0.146964
C	-2.711693	-2.536746	-0.290766
C	-6.995589	0.195478	0.147349
C	1.961470	-2.597066	1.643604
H	1.449654	-3.561753	1.748232
H	2.666807	-2.494064	2.477506
H	1.216182	-1.804442	1.717947
C	1.722714	-2.770693	-0.876433
H	0.941639	-2.011990	-0.890228
H	2.250877	-2.750302	-1.837871
H	1.247275	-3.754115	-0.772568
C	3.731590	-3.692712	0.273256
H	3.198318	-4.643949	0.380710
H	4.291020	-3.733614	-0.669194
H	4.449457	-3.624091	1.099484
C	-1.722761	-2.770820	0.876179
H	-1.247350	-3.754242	0.772195
H	-0.941672	-2.012134	0.890082
H	-2.250953	-2.750533	1.837602
C	-3.731633	-3.692656	-0.273659
H	-3.198382	-4.643884	-0.381299
H	-4.291023	-3.733711	0.668808
H	-4.449539	-3.623888	-1.099842
C	-1.961474	-2.596865	-1.643842
H	-1.216119	-1.804289	-1.718048
H	-1.449729	-3.561574	-1.748623
H	-2.666786	-2.493673	-2.477743
Co	-0.000008	1.292009	0.000021
C	7.425867	-0.586829	-1.410198
H	7.022240	-0.116432	-2.314123
H	8.519649	-0.609327	-1.493825

H	7.072734	-1.623234	-1.387061
C	7.593914	-0.486335	1.106085
H	7.309651	0.055361	2.015531
H	7.248326	-1.520164	1.211492
H	8.689295	-0.506011	1.046294
C	7.578184	1.617464	-0.242582
H	7.209237	2.146803	-1.128849
H	7.333007	2.215576	0.642876
H	8.670481	1.567867	-0.316511
C	-7.594139	-0.486490	-1.105502
H	-7.310007	0.055050	-2.015079
H	-7.248636	-1.520359	-1.210804
H	-8.689508	-0.506105	-1.045512
C	-7.578169	1.617484	0.242896
H	-7.209101	2.146930	1.129048
H	-7.333127	2.215487	-0.642670
H	-8.670452	1.567877	0.317008
C	-7.425588	-0.586693	1.410743
H	-7.021897	-0.116129	2.314554
H	-8.519356	-0.609325	1.494529
H	-7.072307	-1.623051	1.387686
C	0.676564	3.988344	0.354226
H	0.524029	4.123604	1.433070
H	1.320625	4.800384	-0.006918
C	-0.676492	3.988191	-0.355015
H	-0.523964	4.122835	-1.433934
H	-1.320530	4.800441	0.005688

Co-III-NR$_2$-singlet

C	-1.879239	0.043745	-2.041731
H	-2.549383	0.278733	-2.882077
N	-0.690080	0.545938	-2.026725
C	-2.428108	-0.810536	-1.017231
C	-3.828568	-0.986727	-1.000848

C	-1.593593	-1.486637	-0.059373
H	-4.409267	-0.442410	-1.739472
C	-4.453485	-1.783248	-0.060334
C	-2.230573	-2.395612	0.863396
C	-3.613097	-2.486080	0.839866
H	-4.095279	-3.139956	1.556402
C	3.064728	1.556858	-1.786908
H	3.515688	2.188191	-2.565340
N	1.769159	1.515041	-1.712666
C	3.995131	0.835137	-0.965284
C	3.589996	-0.038767	0.105318
C	5.370243	0.991859	-1.281184
H	5.621123	1.674849	-2.087779
C	4.617613	-0.794500	0.781591
C	6.359508	0.307370	-0.609044
C	5.934711	-0.586874	0.409054
H	6.705396	-1.147355	0.924184
O	-0.304482	-1.323877	-0.026560
O	2.351569	-0.164790	0.480023
C	-1.375844	-3.237781	1.829179
C	-5.976985	-1.937440	0.047004
C	4.233204	-1.812297	1.873996
C	7.858223	0.451984	-0.912273
C	-0.440592	-4.159917	1.008280
H	0.184062	-4.759851	1.682196
H	-1.027823	-4.847235	0.387213
H	0.214649	-3.575529	0.360751
C	-0.522477	-2.329145	2.747155
H	0.170319	-1.727087	2.159871
H	-1.167015	-1.663968	3.336007
H	0.058732	-2.941519	3.447867
C	-2.239446	-4.134209	2.738342
H	-1.586330	-4.720399	3.394590
H	-2.908963	-3.546777	3.378696

H	-2.846648	-4.841959	2.161724
C	5.462420	-2.559658	2.428777
H	5.135237	-3.280728	3.186454
H	5.989845	-3.119393	1.647159
H	6.176977	-1.880333	2.909224
C	3.552993	-1.090442	3.061862
H	3.296468	-1.814812	3.845677
H	4.226937	-0.342792	3.498318
H	2.639545	-0.592023	2.739608
C	3.267719	-2.873130	1.288778
H	3.749209	-3.420197	0.468823
H	2.994548	-3.600128	2.064451
H	2.353542	-2.414055	0.911179
Co	0.688026	0.347431	-0.436803
C	-6.438490	-1.502671	1.458416
H	-6.170397	-0.456507	1.643611
H	-7.526577	-1.604423	1.556636
H	-5.975008	-2.109399	2.243479
C	-6.371237	-3.414259	-0.188306
H	-6.064755	-3.742180	-1.188174
H	-5.898589	-4.080833	0.540899
H	-7.457746	-3.542139	-0.102719
C	-6.721569	-1.068324	-0.983006
H	-6.490464	-0.004589	-0.851507
H	-6.467559	-1.350570	-2.011254
H	-7.803908	-1.192906	-0.863585
C	8.596669	0.942028	0.355614
H	8.212421	1.917546	0.675225
H	8.474565	0.245163	1.191649
H	9.671760	1.043468	0.160386
C	8.127668	1.462219	-2.042614
H	7.655908	1.152822	-2.982579
H	7.760320	2.462828	-1.786397
H	9.205926	1.540412	-2.223097

C	8.436282	-0.916519	-1.343514
H	7.934593	-1.281623	-2.247025
H	9.509325	-0.831278	-1.556869
H	8.311740	-1.674902	-0.563316
C	-0.168361	1.303212	-3.158077
H	0.287773	0.600893	-3.869480
H	-0.953873	1.861093	-3.690460
C	0.927801	2.255447	-2.641200
H	0.455376	3.082363	-2.093421
H	1.496045	2.681930	-3.480874
C	0.497776	1.356971	2.361254
N	-0.236403	1.448024	1.107182
H	1.555216	1.520972	2.153872
H	0.136279	2.088494	3.095414
H	0.409067	0.353133	2.798108
C	-1.592923	1.616546	1.187428
C	-2.379046	1.157405	2.280254
C	-2.258094	2.269504	0.115662
C	-3.759314	1.301314	2.262636
H	-1.900777	0.637295	3.101919
C	-3.632739	2.445747	0.131228
H	-1.654197	2.637945	-0.704610
C	-4.396003	1.947174	1.194959
H	-4.349817	0.900843	3.081776
H	-4.119890	2.961774	-0.691768
H	-5.476170	2.061354	1.193674

Co-III-NR$_2$-triplet

C	-1.922387	-0.038069	-2.01642
H	-2.636608	0.277838	-2.7848
N	-0.694865	0.369297	-2.123704
C	-2.442067	-0.853966	-0.962312
C	-3.848811	-0.992104	-0.880462
C	-1.579706	-1.5057	-0.016241

H	-4.446406	-0.446622	-1.604466
C	-4.447619	-1.755035	0.098262
C	-2.196052	-2.399806	0.93714
C	-3.578924	-2.463953	0.969181
H	-4.041434	-3.10189	1.712519
C	3.002998	1.463086	-1.870637
H	3.43088	2.209766	-2.548762
N	1.717258	1.279954	-1.909107
C	3.926789	0.760436	-1.035843
C	3.492121	-0.227481	-0.087093
C	5.305012	1.054511	-1.202463
H	5.568196	1.819683	-1.92719
C	4.512566	-0.943151	0.644438
C	6.280305	0.397738	-0.485436
C	5.835523	-0.601124	0.421362
H	6.598078	-1.134557	0.975449
O	-0.292162	-1.331418	0.000135
O	2.237521	-0.492151	0.131446
C	-1.325021	-3.260392	1.871022
C	-5.967082	-1.864143	0.285646
C	4.124728	-2.050939	1.642763
C	7.783481	0.679008	-0.625072
C	-0.441999	-4.19793	1.011572
H	0.209164	-4.801775	1.656279
H	-1.06673	-4.880867	0.423133
H	0.187965	-3.624691	0.32984
C	-0.41922	-2.371763	2.754954
H	0.274473	-1.804133	2.137244
H	-1.023362	-1.675207	3.351054
H	0.161158	-2.993205	3.448338
C	-2.170799	-4.14263	2.810226
H	-1.504014	-4.746894	3.435697
H	-2.799454	-3.544323	3.481189
H	-2.81725	-4.833397	2.256141

C	5.358426	-2.748169	2.250273
H	5.025502	-3.535403	2.936201
H	5.982069	-3.22101	1.48213
H	5.984503	-2.056035	2.826193
C	3.314726	-1.437176	2.808331
H	3.022087	-2.218301	3.521441
H	3.914309	-0.691935	3.345777
H	2.411807	-0.956558	2.436263
C	3.279269	-3.136837	0.933605
H	3.851745	-3.597764	0.119037
H	3.00967	-3.926617	1.64631
H	2.362096	-2.715682	0.522482
Co	0.705232	0.059768	-0.866283
C	-6.342973	-1.363155	1.700456
H	-6.042364	-0.316969	1.828629
H	-7.426195	-1.433535	1.861292
H	-5.850436	-1.949895	2.483171
C	-6.414477	-3.335796	0.127484
H	-6.16703	-3.710044	-0.872487
H	-5.926495	-3.989396	0.858316
H	-7.498663	-3.427508	0.270212
C	-6.738804	-1.012633	-0.739091
H	-6.476344	0.048845	-0.65724
H	-6.540012	-1.337323	-1.767053
H	-7.817042	-1.104723	-0.565048
C	8.356492	1.123067	0.741426
H	7.864347	2.038736	1.08897
H	8.214914	0.355875	1.510078
H	9.433006	1.320594	0.662017
C	8.072757	1.791909	-1.649054
H	7.717807	1.522034	-2.650558
H	7.601407	2.738738	-1.360576
H	9.152921	1.965384	-1.715884
C	8.510879	-0.604051	-1.091554

H	8.128576	-0.934174	-2.064289
H	9.588583	-0.421921	-1.189655
H	8.37606	-1.428268	-0.383089
C	-0.256523	1.156038	-3.277548
H	0.142667	0.475124	-4.041297
H	-1.082256	1.72714	-3.723089
C	0.867179	2.076815	-2.790237
H	0.438483	2.901209	-2.205255
H	1.427537	2.505398	-3.631672
C	0.690924	1.274659	2.003739
N	-0.077725	1.459225	0.788621
H	1.74964	1.370677	1.762537
H	0.409326	1.99206	2.786579
H	0.545654	0.259529	2.393403
C	-1.413052	1.710553	0.927358
C	-2.185454	1.276404	2.041912
C	-2.085912	2.392416	-0.122895
C	-3.559443	1.470401	2.064396
H	-1.70564	0.726394	2.84317
C	-3.451869	2.62115	-0.065398
H	-1.49551	2.740883	-0.961889
C	-4.201894	2.145753	1.018896
H	-4.140668	1.08573	2.897542
H	-3.944093	3.158636	-0.871618
H	-5.277118	2.297488	1.048306

Co-III-NR_2-quintet

C	-1.879239	0.043745	-2.041731
H	-2.549383	0.278733	-2.882077
N	-0.69008	0.545938	-2.026725
C	-2.428108	-0.810536	-1.017231
C	-3.828568	-0.986727	-1.000848
C	-1.593593	-1.486637	-0.059373
H	-4.409267	-0.44241	-1.739472

C	-4.453485	-1.783248	-0.060334
C	-2.230573	-2.395612	0.863396
C	-3.613097	-2.48608	0.839866
H	-4.095279	-3.139956	1.556402
C	3.064728	1.556858	-1.786908
H	3.515688	2.188191	-2.56534
N	1.769159	1.515041	-1.712666
C	3.995131	0.835137	-0.965284
C	3.589996	-0.038767	0.105318
C	5.370243	0.991859	-1.281184
H	5.621123	1.674849	-2.087779
C	4.617613	-0.7945	0.781591
C	6.359508	0.30737	-0.609044
C	5.934711	-0.586874	0.409054
H	6.705396	-1.147355	0.924184
O	-0.304482	-1.323877	-0.02656
O	2.351569	-0.16479	0.480023
C	-1.375844	-3.237781	1.829179
C	-5.976985	-1.93744	0.047004
C	4.233204	-1.812297	1.873996
C	7.858223	0.451984	-0.912273
C	-0.440592	-4.159917	1.00828
H	0.184062	-4.759851	1.682196
H	-1.027823	-4.847235	0.387213
H	0.214649	-3.575529	0.360751
C	-0.522477	-2.329145	2.747155
H	0.170319	-1.727087	2.159871
H	-1.167015	-1.663968	3.336007
H	0.058732	-2.941519	3.447867
C	-2.239446	-4.134209	2.738342
H	-1.58633	-4.720399	3.39459
H	-2.908963	-3.546777	3.378696
H	-2.846648	-4.841959	2.161724
C	5.46242	-2.559658	2.428777

H	5.135237	-3.280728	3.186454
H	5.989845	-3.119393	1.647159
H	6.176977	-1.880333	2.909224
C	3.552993	-1.090442	3.061862
H	3.296468	-1.814812	3.845677
H	4.226937	-0.342792	3.498318
H	2.639545	-0.592023	2.739608
C	3.267719	-2.87313	1.288778
H	3.749209	-3.420197	0.468823
H	2.994548	-3.600128	2.064451
H	2.353542	-2.414055	0.911179
Co	0.688026	0.347431	-0.436803
C	-6.43849	-1.502671	1.458416
H	-6.170397	-0.456507	1.643611
H	-7.526577	-1.604423	1.556636
H	-5.975008	-2.109399	2.243479
C	-6.371237	-3.414259	-0.188306
H	-6.064755	-3.74218	-1.188174
H	-5.898589	-4.080833	0.540899
H	-7.457746	-3.542139	-0.102719
C	-6.721569	-1.068324	-0.983006
H	-6.490464	-0.004589	-0.851507
H	-6.467559	-1.35057	-2.011254
H	-7.803908	-1.192906	-0.863585
C	8.596669	0.942028	0.355614
H	8.212421	1.917546	0.675225
H	8.474565	0.245163	1.191649
H	9.67176	1.043468	0.160386
C	8.127668	1.462219	-2.042614
H	7.655908	1.152822	-2.982579
H	7.76032	2.462828	-1.786397
H	9.205926	1.540412	-2.223097
C	8.436282	-0.916519	-1.343514
H	7.934593	-1.281623	-2.247025

H	9.509325	-0.831278	-1.556869
H	8.31174	-1.674902	-0.563316
C	-0.168361	1.303212	-3.158077
H	0.287773	0.600893	-3.86948
H	-0.953873	1.861093	-3.69046
C	0.927801	2.255447	-2.6412
H	0.455376	3.082363	-2.093421
H	1.496045	2.68193	-3.480874
C	0.497776	1.356971	2.361254
N	-0.236403	1.448024	1.107182
H	1.555216	1.520972	2.153872
H	0.136279	2.088494	3.095414
H	0.409067	0.353133	2.798108
C	-1.592923	1.616546	1.187428
C	-2.379046	1.157405	2.280254
C	-2.258094	2.269504	0.115662
C	-3.759314	1.301314	2.262636
H	-1.900777	0.637295	3.101919
C	-3.632739	2.445747	0.131228
H	-1.654197	2.637945	-0.70461
C	-4.396003	1.947174	1.194959
H	-4.349817	0.900843	3.081776
H	-4.11989	2.961774	-0.691768
H	-5.47617	2.061354	1.193674

Co-III-NR_2-septet

C	-2.010681	-0.019227	-2.192276
H	-2.633839	0.241336	-3.054138
N	-0.801558	0.529918	-2.113766
C	-2.606447	-0.892861	-1.23118
C	-3.970484	-1.243788	-1.392768
C	-1.884205	-1.424847	-0.097811
H	-4.487993	-0.844436	-2.259169
C	-4.637519	-2.045329	-0.480472

C	-2.556742	-2.260677	0.846356
C	-3.904639	-2.533909	0.63023
H	-4.429495	-3.154723	1.344185
C	2.9677	1.459882	-1.957822
H	3.399969	2.147558	-2.692524
N	1.647206	1.40833	-1.849787
C	3.895888	0.708407	-1.173679
C	3.480051	-0.273538	-0.194162
C	5.281115	0.938031	-1.354956
H	5.571682	1.669183	-2.10237
C	4.460576	-0.963269	0.586279
C	6.242351	0.271919	-0.609983
C	5.801305	-0.668043	0.356613
H	6.55486	-1.18215	0.938033
O	-0.616674	-1.136953	0.072784
O	2.210927	-0.536486	-0.014446
C	-1.799338	-2.815721	2.068295
C	-6.123593	-2.410916	-0.614649
C	4.023381	-2.012522	1.626882
C	7.749623	0.509441	-0.785926
C	-0.625684	-3.709542	1.598594
H	-0.084917	-4.1066	2.467448
H	-0.998269	-4.559358	1.013948
H	0.078168	-3.146908	0.984354
C	-1.252971	-1.652849	2.933193
H	-0.536396	-1.049583	2.375378
H	-2.066746	-0.998802	3.267082
H	-0.749084	-2.053137	3.822306
C	-2.708981	-3.673814	2.970054
H	-2.126502	-4.049841	3.818796
H	-3.547981	-3.095651	3.37554
H	-3.113443	-4.542527	2.437503
C	5.226305	-2.606001	2.387307
H	4.867153	-3.341971	3.115402

H	5.9225	-3.121711	1.715872
H	5.782689	-1.838354	2.938453
C	3.079233	-1.375972	2.677968
H	2.819125	-2.116393	3.444716
H	3.571395	-0.531869	3.178156
H	2.154523	-1.025468	2.21785
C	3.294039	-3.178767	0.915648
H	3.962656	-3.669167	0.197949
H	2.976036	-3.928305	1.651371
H	2.410195	-2.825252	0.38259
Co	0.555151	0.185555	-0.615889
C	-6.894545	-1.876164	0.615905
H	-6.809272	-0.784912	0.681352
H	-7.959054	-2.132213	0.543326
H	-6.51164	-2.298238	1.550754
C	-6.273861	-3.949192	-0.684486
H	-5.738621	-4.351182	-1.552195
H	-5.875315	-4.438856	0.209875
H	-7.331634	-4.226676	-0.774777
C	-6.766333	-1.810747	-1.878712
H	-6.723042	-0.715463	-1.87445
H	-6.276445	-2.170294	-2.790759
H	-7.821994	-2.100645	-1.931861
C	8.351204	1.00847	0.549544
H	7.886386	1.952879	0.855295
H	8.204103	0.285661	1.358506
H	9.430173	1.176344	0.442759
C	8.058844	1.55775	-1.870717
H	7.681182	1.248329	-2.85191
H	7.62377	2.533682	-1.626546
H	9.143096	1.690617	-1.959169
C	8.433654	-0.818004	-1.192131
H	8.026219	-1.189977	-2.139064
H	9.513161	-0.667797	-1.318364

H	8.290676	-1.597992	-0.437369
C	-0.341178	1.361022	-3.212361
H	0.038994	0.732904	-4.032448
H	-1.155619	1.982439	-3.62126
C	0.815049	2.235743	-2.697964
H	0.401693	3.064473	-2.103509
H	1.374046	2.66959	-3.540874
C	1.609487	2.111972	1.471335
N	0.341376	1.621328	0.959372
H	2.308545	2.19286	0.638871
H	1.510547	3.088424	1.961269
H	2.033579	1.395979	2.188977
C	-0.774922	1.763231	1.720609
C	-0.761812	2.117661	3.103499
C	-2.034838	1.501195	1.1077
C	-1.945036	2.158087	3.82376
H	0.179707	2.30417	3.607252
C	-3.208688	1.554674	1.839186
H	-2.048843	1.268668	0.051999
C	-3.172792	1.875694	3.203186
H	-1.919174	2.399485	4.882773
H	-4.15219	1.329047	1.351127
H	-4.091415	1.903409	3.782148

Ir(ppy)3

N	-0.643775	1.749467	-1.082762
C	-0.161313	2.929939	-0.595432
C	-1.859526	2.900887	-2.799830
C	-0.524407	4.134264	-1.223381
C	-1.470372	1.741918	-2.141674
C	-1.369691	4.121822	-2.324713
H	-1.820806	0.762785	-2.452127
H	-2.529371	2.845959	-3.651383
C	0.710059	2.803444	0.576331

C	0.918494	1.480622	1.068954
C	1.739657	1.348155	2.205014
C	2.321226	2.454840	2.822724
N	-1.194014	-1.431309	-1.083209
C	-2.457154	-1.604602	-0.595160
C	-1.584061	-3.058085	-2.801808
C	-3.318599	-2.521135	-1.223195
C	-0.774837	-2.141871	-2.143385
C	-2.885745	-3.245667	-2.325594
H	0.247832	-1.954559	-2.454645
H	-1.202071	-3.609547	-3.654348
C	-2.782724	-0.787613	0.577371
C	-1.741095	0.054015	1.070030
C	-2.036161	0.830058	2.207171
C	-3.284966	0.779815	2.825635
H	1.916095	0.356785	2.611909
H	2.947921	2.314436	3.701195
H	-0.145887	5.074906	-0.840513
H	-1.650238	5.054262	-2.806757
C	2.105405	3.746176	2.323293
H	2.559548	4.607552	2.805551
C	1.301501	3.916075	1.203165
H	1.135625	4.920327	0.820693
H	-4.322062	-2.664633	-0.839611
H	-3.552997	-3.954800	-2.807694
H	-3.476106	1.391801	3.704900
H	-1.265424	1.477798	2.614415
C	-4.041634	-0.832282	1.204943
H	-4.828618	-1.477720	0.822343
C	-4.295756	-0.052152	2.325947
H	-5.268536	-0.089925	2.808711
Ir	-0.000146	-0.000121	0.027878
C	2.617740	-1.325185	-0.596872
C	3.438371	0.157649	-2.804121

C	3.841831	-1.612848	-1.225665
C	2.240633	0.400202	-2.145016
C	4.252085	-0.875737	-2.328367
H	1.566895	1.192220	-2.455896
H	3.724369	0.764193	-3.656858
C	2.073932	-2.015664	0.576084
C	0.824108	-1.535149	1.069110
C	0.300020	-2.179240	2.206171
C	0.968839	-3.235076	2.824576
H	4.468130	-2.410066	-0.842433
H	5.199571	-1.098933	-2.811033
H	0.534836	-3.706995	3.703839
H	-0.646638	-1.836395	2.613388
C	2.195115	-3.693416	2.324834
H	2.714967	-4.516486	2.807638
C	2.743012	-3.082968	1.203674
H	3.695708	-3.441083	0.820995
N	1.835583	-0.318117	-1.084567

[Ir(ppy)3]+

N	1.359227	-1.270547	1.073415
C	2.634821	-1.277745	0.587730
C	1.936867	-2.873007	2.755192
C	3.602427	-2.088944	1.202421
C	1.020934	-2.051497	2.114820
C	3.254970	-2.885619	2.285204
H	-0.017610	-2.001239	2.424043
H	1.625143	-3.487324	3.592547
C	2.862405	-0.407569	-0.570073
C	1.735605	0.329724	-1.046762
C	1.929649	1.219911	-2.118950
C	3.176730	1.344625	-2.728421
N	-1.778777	-0.539769	1.075189
C	-2.425755	-1.638961	0.589098

C	-3.455320	-0.234213	2.756228
C	-3.613947	-2.067609	1.202770
C	-2.284326	0.144763	2.116668
C	-4.128751	-1.366959	2.285303
H	-1.718893	1.017052	2.426644
H	-3.830026	0.343701	3.593683
C	-1.786633	-2.273669	-0.567699
C	-0.582687	-1.670205	-1.043836
C	0.091113	-2.286800	-2.114042
C	-0.426524	-3.428771	-2.722531
H	1.088169	1.792784	-2.494818
H	3.300732	2.018760	-3.571616
H	4.618913	-2.095564	0.827763
H	4.001514	-3.516181	2.758403
C	4.271369	0.603428	-2.262889
H	5.242148	0.706070	-2.738016
C	4.115778	-0.263200	-1.184866
H	4.976851	-0.820745	-0.828857
H	-4.130469	-2.942875	0.827586
H	-5.049531	-1.695355	2.757717
H	0.095314	-3.875764	-3.564375
H	1.009585	-1.847544	-2.489505
C	-2.290452	-3.430923	-1.181506
H	-3.205370	-3.895115	-0.825869
C	-1.617890	-4.002294	-2.257862
H	-2.016136	-4.893946	-2.732252
Ir	0.000491	-0.000763	-0.068882
C	-0.208549	2.919342	0.587264
C	1.520763	3.111321	2.755927
C	0.011494	4.162922	1.201502
C	1.266227	1.907412	2.115893
C	0.874649	4.259794	2.284728
H	1.740175	0.982306	2.426116
H	2.207854	3.147983	3.593948

C	-1.076457	2.681927	-0.570262
C	-1.154073	1.337298	-1.045808
C	-2.023768	1.060662	-2.116545
C	-2.754282	2.079007	-2.726230
H	-0.489751	5.046972	0.826190
H	1.048421	5.221627	2.757524
H	-3.401452	1.849540	-3.568452
H	-2.101542	0.045246	-2.491285
C	-2.656797	3.397905	-2.262298
H	-3.230081	4.187856	-2.737706
C	-1.826977	3.695983	-1.185347
H	-1.772524	4.720704	-0.830348
N	0.421702	1.810538	1.073671

X-1

C	0.700473	1.153425	0.000771
C	-0.698229	1.192820	-0.009844
C	-1.425618	-0.000675	-0.012380
C	-0.697217	-1.193485	-0.009697
C	0.701508	-1.152851	0.000834
N	1.387932	0.000563	0.006230
H	-1.214941	2.149328	-0.017933
H	-1.213075	-2.150447	-0.017607
C	-2.934419	-0.001125	0.009349
H	-3.307114	0.003741	1.042179
H	-3.343074	0.884340	-0.489097
H	-3.342520	-0.891464	-0.480744
C	1.527223	-2.416973	0.002380
H	2.180771	-2.442996	0.881837
H	0.901134	-3.314762	0.005459
H	2.177692	-2.447525	-0.879333
C	1.525127	2.418232	0.002446
H	0.898281	3.315493	0.004394
H	2.177672	2.445292	0.882632

H	2.176558	2.448842	-0.878546

2a

C	-1.594855	0.347584	0.640022
C	-0.234062	0.095685	0.510151
C	0.617067	1.032004	-0.111902
C	0.052814	2.224380	-0.601532
C	-1.314629	2.466505	-0.459350
C	-2.150828	1.537140	0.157781
H	-2.227727	-0.393215	1.122602
H	0.187504	-0.830644	0.896371
H	0.676668	2.964349	-1.091430
H	-1.726733	3.396014	-0.844801
H	-3.214257	1.731657	0.260919
N	1.982898	0.768991	-0.192805
H	2.211731	-0.213845	-0.129299
C	2.842932	1.525227	-1.083258
H	3.844524	1.087682	-1.060549
H	2.488090	1.539541	-2.127319
H	2.928597	2.563556	-0.742079

3a

C	2.225381	-2.746759	-0.309227
C	0.843018	-2.634305	-0.16325
C	0.101786	-1.752461	-0.967027
C	0.781476	-1.049382	-1.977968
C	2.159394	-1.184374	-2.14071
C	2.893186	-2.020027	-1.296544
H	2.777658	-3.414931	0.345824
H	0.324024	-3.235905	0.575683
H	0.230295	-0.377981	-2.628849
H	2.664642	-0.613253	-2.915165
H	3.969791	-2.107207	-1.410509
N	-1.30521	-1.639676	-0.798341

C	-2.080673	-1.633366	-2.034228
H	-3.131235	-1.847564	-1.816382
H	-1.703001	-2.424252	-2.68931
H	-2.057926	-0.681749	-2.587024
C	-1.95834	-0.987102	0.382211
C	-2.968853	-2.018164	0.939117
H	-2.42509	-2.918106	1.24032
H	-3.69059	-2.304556	0.169783
H	-3.532528	-1.65163	1.799898
C	-2.627843	0.334933	-0.044331
C	-1.903468	1.257588	-0.81863
C	-3.93925	0.671291	0.31393
C	-2.463005	2.472131	-1.20824
H	-0.893377	1.00925	-1.118216
C	-4.504736	1.890423	-0.073863
H	-4.540234	-0.013682	0.901516
C	-3.769699	2.797874	-0.834194
H	-1.878599	3.164553	-1.809058
H	-5.524504	2.12445	0.220766
H	-4.208873	3.744527	-1.137171
C	-0.944835	-0.694709	1.53188
H	-0.449886	-1.62488	1.813654
O	0.06845	0.206212	1.030195
C	1.318091	0.07254	1.532584
O	1.604821	-0.690636	2.435403
C	2.295431	0.949826	0.831191
C	3.65751	0.726826	1.068746
C	1.896536	1.952591	-0.062299
C	4.615649	1.490132	0.405818
H	3.94051	-0.055941	1.764512
C	2.857797	2.720501	-0.717652
H	0.840726	2.129082	-0.23084
C	4.216834	2.486916	-0.489699
H	5.671958	1.309159	0.584797

H	2.547683	3.500366	-1.407909
H	4.964183	3.082997	-1.006975
C	-1.550064	-0.058675	2.782855
H	-0.738799	0.136386	3.488822
H	-2.048958	0.883702	2.54123
H	-2.265531	-0.725202	3.271364

3a’

C	1.942369	1.839582	-1.802162
C	0.60606	1.555552	-1.51683
C	0.098818	1.718781	-0.221378
C	0.950437	2.224309	0.773788
C	2.282187	2.523965	0.484642
C	2.786496	2.323977	-0.801758
H	2.321034	1.681422	-2.808145
H	-0.057923	1.19236	-2.294537
H	0.571798	2.378052	1.779541
H	2.931695	2.900148	1.270738
H	3.828878	2.537755	-1.019893
N	-1.286365	1.416442	0.020796
C	-2.002006	2.525393	0.65573
H	-3.076073	2.322382	0.641609
H	-1.709405	2.725519	1.699556
H	-1.816649	3.43458	0.076263
C	-1.653269	0.064438	0.556647
C	-1.344083	-0.053074	2.063993
H	-1.972123	0.624303	2.649335
H	-1.504882	-1.068034	2.438647
H	-0.297051	0.198932	2.239995
C	-3.144424	-0.147089	0.234428
C	-3.607374	0.147126	-1.058168
C	-4.052374	-0.676008	1.158719
C	-4.932936	-0.088728	-1.417212
H	-2.916816	0.586159	-1.770903

C	-5.384052	-0.909979	0.802741
H	-3.73179	-0.916161	2.166452
C	-5.829606	-0.62083	-0.486309
H	-5.267962	0.147082	-2.42402
H	-6.071169	-1.319001	1.538965
H	-6.864535	-0.803128	-0.763111
C	-0.882741	-1.045369	-0.226853
H	-0.877984	-0.782722	-1.286479
C	-1.421611	-2.466417	-0.070167
H	-0.761581	-3.14298	-0.619416
H	-1.44128	-2.773626	0.980142
H	-2.429769	-2.554788	-0.479656
O	0.488446	-1.041467	0.245166
C	1.454185	-1.359312	-0.644915
O	1.229318	-1.729017	-1.78256
C	2.818876	-1.184323	-0.074139
C	3.030715	-0.791863	1.253955
C	3.913208	-1.390429	-0.924055
C	4.329513	-0.604699	1.724207
H	2.179625	-0.626394	1.903554
C	5.209651	-1.197799	-0.452867
H	3.723298	-1.690268	-1.949383
C	5.419222	-0.803581	0.872228
H	4.492347	-0.2985	2.754021
H	6.056462	-1.353512	-1.115652
H	6.430721	-0.652535	1.240379

HX-1

C	-1.882897	-0.878904	-0.521719
C	-3.172141	-1.076965	-0.044821
C	-3.984196	0.016209	0.285383
C	-3.466735	1.306378	0.111113
C	-2.175426	1.489775	-0.367529
N	-1.440475	0.391757	-0.651789

H	-3.542104	-2.090932	0.064586
H	-4.069588	2.177874	0.343465
C	-5.370053	-0.191176	0.835597
H	-5.815158	-1.118239	0.462316
H	-5.332096	-0.260302	1.930446
H	-6.031499	0.642240	0.581149
C	-1.534060	2.820408	-0.617234
H	-1.349300	2.948239	-1.690660
H	-2.179300	3.631181	-0.272601
H	-0.561080	2.883244	-0.117484
C	-0.941429	-1.970676	-0.928249
H	-1.374337	-2.951637	-0.721672
H	-0.719813	-1.884467	-1.997185
H	0.010491	-1.874244	-0.397778
H	-0.450836	0.532978	-1.013830
S	1.127634	0.496788	-3.212586
O	-0.228604	0.033978	-3.546709
C	2.213474	-1.006651	-3.471987
F	1.767960	-2.020942	-2.709382
F	2.156779	-1.375305	-4.753629
F	3.475577	-0.738075	-3.141480
O	1.753550	1.578795	-3.963649
N	1.089949	0.692765	-1.591786
S	2.258830	1.341826	-0.651218
O	1.604355	2.218910	0.328656
O	3.473021	1.759803	-1.344213
C	2.714471	-0.173697	0.350315
F	3.208428	-1.133957	-0.436061
F	1.614474	-0.653977	0.967431
F	3.617512	0.149391	1.275792

TS4

C	-2.517695	0.243918	-1.624948
H	-3.251369	0.556119	-2.378184

N	-1.306728	0.706362	-1.729294
C	-3.017945	-0.656781	-0.624414
C	-4.414732	-0.910309	-0.632014
C	-2.165798	-1.321215	0.328217
H	-5.012457	-0.374935	-1.36436
C	-5.002167	-1.793263	0.248095
C	-2.760407	-2.322225	1.183859
C	-4.131221	-2.494838	1.126906
H	-4.583431	-3.218239	1.794898
C	2.417323	1.807339	-1.89307
H	2.795553	2.450761	-2.7029
N	1.143563	1.762674	-1.680538
C	3.422472	1.063425	-1.172193
C	3.090055	-0.077388	-0.364426
C	4.773008	1.441589	-1.362634
H	4.966404	2.301789	-1.997775
C	4.173453	-0.823201	0.225881
C	5.814527	0.761087	-0.762801
C	5.467238	-0.368977	0.021347
H	6.279925	-0.917429	0.48252
O	-0.908568	-1.045157	0.446145
O	1.865765	-0.466791	-0.175692
C	-1.866642	-3.149907	2.1279
C	-6.513393	-2.054708	0.316127
C	3.873737	-2.097656	1.041059
C	7.291914	1.153326	-0.91411
C	-0.784923	-3.891621	1.303252
H	-0.15522	-4.494361	1.97039
H	-1.249766	-4.567591	0.574763
H	-0.145016	-3.187947	0.769121
C	-1.183074	-2.233654	3.170281
H	-0.563163	-1.480496	2.684772
H	-1.929188	-1.718447	3.784055
H	-0.54789	-2.833694	3.835135

C	-2.673979	-4.21227	2.899869
H	-1.994702	-4.792719	3.534528
H	-3.428173	-3.760151	3.555666
H	-3.178944	-4.914832	2.225772
C	5.158431	-2.743609	1.597046
H	4.895617	-3.646768	2.159429
H	5.847509	-3.042956	0.798319
H	5.693446	-2.073132	2.280917
C	2.954509	-1.779478	2.246161
H	2.781057	-2.688789	2.835562
H	3.423744	-1.036867	2.904576
H	1.987871	-1.400819	1.912636
C	3.178195	-3.140499	0.131134
H	3.828681	-3.412735	-0.709168
H	2.960798	-4.052294	0.702183
H	2.239806	-2.748121	-0.263759
Co	0.136881	0.449836	-0.292539
C	-7.030666	-1.685566	1.727051
H	-6.834323	-0.63018	1.947437
H	-8.112058	-1.859303	1.801452
H	-6.543154	-2.283105	2.504639
C	-6.804566	-3.548418	0.040159
H	-6.452629	-3.832985	-0.957997
H	-6.308513	-4.200131	0.766863
H	-7.882342	-3.748168	0.094706
C	-7.294097	-1.22069	-0.716202
H	-7.141262	-0.145603	-0.567769
H	-6.996991	-1.466093	-1.742271
H	-8.367927	-1.420839	-0.624435
C	7.888234	1.469156	0.477888
H	7.355177	2.304402	0.947011
H	7.820282	0.609228	1.152652
H	8.947514	1.743015	0.39159
C	7.475401	2.395764	-1.805019

H	7.098447	2.22459	-2.820072
H	6.960107	3.270114	-1.390265
H	8.540105	2.644123	-1.884056
C	8.079026	-0.015053	-1.553111
H	7.680351	-0.252923	-2.545953
H	9.139061	0.247505	-1.662052
H	8.022133	-0.923878	-0.944806
C	-0.931371	1.467038	-2.918289
H	-0.575218	0.757176	-3.677818
H	-1.782916	2.020767	-3.337895
C	0.21693	2.434348	-2.577252
H	-0.204989	3.307673	-2.068284
H	0.700783	2.790343	-3.499403
N	-0.09668	1.827066	1.402838
C	-0.603594	4.400188	0.912998
C	0.747179	4.680993	0.304574
H	0.847881	5.745153	0.043067
H	1.564797	4.43615	0.982282
H	0.89814	4.094424	-0.602312
C	-0.851271	4.770677	2.279403
C	-2.035389	4.425636	2.991704
C	0.152342	5.467474	3.016961
C	-2.212553	4.79415	4.321347
H	-2.798398	3.842267	2.505202
C	-0.037729	5.833505	4.341768
H	1.075151	5.759948	2.530482
C	-1.226771	5.506121	5.005159
H	-3.12495	4.496652	4.830222
H	0.743289	6.381635	4.861754
H	-1.372317	5.792019	6.043161
C	-1.64352	4.145908	-0.158389
H	-1.364664	3.231846	-0.686139
C	-1.743698	5.313854	-1.158294
H	-2.49444	5.063138	-1.91201

H	-2.047104	6.227209	-0.636726
H	-0.795165	5.497432	-1.668284
O	-2.968519	3.943547	0.378021
C	-3.812252	3.181368	-0.357424
O	-3.516624	2.700855	-1.437084
C	-5.116508	2.974689	0.324677
C	-5.960644	1.972599	-0.172504
C	-5.497962	3.717878	1.450818
C	-7.173176	1.707627	0.46063
H	-5.640101	1.399457	-1.035407
C	-6.713116	3.451299	2.078718
H	-4.84483	4.496909	1.827132
C	-7.548977	2.444165	1.588171
H	-7.823394	0.92634	0.079704
H	-7.007703	4.027325	2.951343
H	-8.493262	2.233425	2.082785
C	1.215945	2.056883	2.023403
C	1.327016	2.902294	3.127674
C	2.350376	1.429708	1.508365
C	2.572382	3.120953	3.716294
H	0.432581	3.397201	3.533007
C	3.596044	1.647554	2.097657
H	2.262988	0.763221	0.638143
C	3.707238	2.493122	3.201372
H	2.660104	3.787795	4.586307
H	4.490257	1.152596	1.691609
H	4.68905	2.665633	3.665681
C	-1.100237	1.54811	2.242182
H	-2.009858	1.443979	1.688426
H	-0.890199	0.634554	2.758128
H	-1.203379	2.342665	2.951367

TS4’

C	-2.270743	-0.472747	2.164701

H	-2.867011	-0.404536	3.085938
N	-1.134407	0.161307	2.128337
C	-2.841742	-1.282522	1.128552
C	-4.169158	-1.745814	1.335764
C	-2.110761	-1.673326	-0.051499
H	-4.67878	-1.411292	2.235085
C	-4.800332	-2.578741	0.439287
C	-2.7353	-2.621557	-0.946213
C	-4.035838	-3.007708	-0.681545
H	-4.515669	-3.692224	-1.370734
C	2.434247	1.721451	1.8185
H	2.863398	2.38201	2.583674
N	1.169618	1.470151	1.849786
C	3.364448	1.193191	0.849131
C	3.047972	0.054846	0.034155
C	4.633367	1.808683	0.767152
H	4.810505	2.668552	1.404948
C	4.082582	-0.466494	-0.822132
C	5.601733	1.369335	-0.114402
C	5.288432	0.217986	-0.880037
H	6.052864	-0.150034	-1.554032
O	-0.934913	-1.213462	-0.328613
O	1.877669	-0.516522	0.057797
C	-1.950941	-3.163277	-2.156906
C	-6.248207	-3.065252	0.595527
C	3.835237	-1.754598	-1.633484
C	6.958944	2.063042	-0.299677
C	-0.654206	-3.857875	-1.670736
H	-0.094222	-4.247286	-2.530732
H	-0.892563	-4.702253	-1.012086
H	-0.011884	-3.162555	-1.128512
C	-1.591533	-2.011154	-3.124247
H	-0.957525	-1.272595	-2.63518
H	-2.495912	-1.503565	-3.477357

H	-1.057207	-2.407385	-3.997657
C	-2.765138	-4.20217	-2.953415
H	-2.158445	-4.576015	-3.786007
H	-3.677918	-3.769897	-3.381394
H	-3.047148	-5.064275	-2.336922
C	5.064908	-2.153841	-2.473271
H	4.848832	-3.082658	-3.013288
H	5.94834	-2.333654	-1.849053
H	5.319006	-1.39099	-3.219437
C	2.643902	-1.580551	-2.607178
H	2.508839	-2.49356	-3.20101
H	2.830997	-0.752707	-3.303108
H	1.718001	-1.389087	-2.063751
C	3.530545	-2.920846	-0.660901
H	4.379513	-3.09313	0.012062
H	3.351651	-3.844659	-1.225918
H	2.645012	-2.705174	-0.060692
Co	0.117944	0.236667	0.493765
C	-7.078199	-2.60099	-0.624794
H	-7.084311	-1.50668	-0.694381
H	-8.116831	-2.945058	-0.539948
H	-6.672549	-2.992292	-1.56379
C	-6.277404	-4.609721	0.675764
H	-5.701086	-4.963123	1.538409
H	-5.852422	-5.07227	-0.221238
H	-7.308445	-4.970811	0.779961
C	-6.915016	-2.508273	1.866641
H	-6.948053	-1.412421	1.858526
H	-6.388457	-2.829209	2.772952
H	-7.946868	-2.871343	1.936399
C	7.088632	2.547046	-1.763295
H	6.290007	3.258017	-2.007242
H	7.020083	1.715341	-2.472332
H	8.053214	3.045519	-1.92339

C	7.114889	3.287565	0.621321
H	7.055042	3.008447	1.679673
H	6.343981	4.041679	0.421887
H	8.090898	3.758033	0.45571
C	8.105523	1.074784	0.016391
H	8.040216	0.725274	1.053142
H	9.081361	1.557175	-0.123102
H	8.073569	0.194796	-0.634587
C	-0.608834	0.786823	3.339672
H	-0.02935	0.034657	3.893014
H	-1.408261	1.152006	4.002035
C	0.332338	1.934729	2.93924
H	-0.272585	2.77569	2.583391
H	0.912321	2.2821	3.807145
C	0.997683	1.762254	-1.888121
N	-0.238229	1.680019	-1.123484
H	1.812233	2.016142	-1.213287
H	0.970505	2.500054	-2.700516
H	1.222191	0.787122	-2.335064
C	-0.493114	4.289644	-0.630783
C	-1.648361	4.092431	0.299894
H	-2.03841	5.060957	0.652242
H	-1.368804	3.4986	1.170231
H	-2.474406	3.577152	-0.19209
C	0.823765	4.581801	-0.096998
C	1.98315	4.626779	-0.914913
C	0.996869	4.886752	1.276089
C	3.233644	4.912885	-0.38258
H	1.90886	4.411977	-1.972738
C	2.247075	5.195284	1.800679
H	0.135637	4.918142	1.933033
C	3.377207	5.201252	0.977874
H	4.103541	4.909288	-1.032995
H	2.339874	5.437254	2.85623

H	4.356132	5.431997	1.388513
C	-0.80345	4.661415	-2.069858
H	-0.34621	3.946863	-2.760118
C	-1.403073	1.511743	-1.845768
C	-1.488605	1.557069	-3.262127
C	-2.599673	1.269043	-1.121371
C	-2.712498	1.384475	-3.901833
H	-0.598432	1.706214	-3.861025
C	-3.81126	1.083397	-1.769865
H	-2.53741	1.194715	-0.042208
C	-3.8796	1.149763	-3.167896
H	-2.752883	1.418969	-4.98721
H	-4.700377	0.86199	-1.186258
H	-4.826889	1.001316	-3.678037
O	-2.184645	4.797601	-2.414319
C	-2.326859	4.99304	-3.749605
O	-1.304888	5.023392	-4.433868
C	-3.802266	5.14500	-4.163951
C	-4.132744	5.353134	-5.50333
C	-4.808192	5.074675	-3.200252
C	-5.468915	5.490226	-5.878929
H	-3.339507	5.40776	-6.262957
C	-6.144757	5.212774	-3.575663
H	-4.54789	4.910816	-2.144543
C	-6.475261	5.420386	-4.914768
H	-5.729518	5.653655	-6.93471
H	-6.937646	5.157692	-2.815553
H	-7.52871	5.528565	-5.211144
C	-0.154536	6.029517	-2.350582
H	0.815509	5.883228	-2.777796
H	-0.062349	6.575183	-1.434803
H	-0.765898	6.580706	-3.034195

NTf_2^-

N	-0.351466	-0.245146	0.847222
S	0.554336	0.808103	1.670679
S	0.054452	-1.791405	0.587123
O	-0.226618	2.042395	1.813404
O	1.386775	-2.222765	1.033857
O	1.267809	0.272260	2.836633
O	-1.097759	-2.667208	0.836880
C	1.923631	1.303747	0.487793
C	0.185597	-1.764051	-1.280155
F	1.132875	-0.903673	-1.686682
F	-0.976294	-1.414274	-1.850502
F	0.517638	-2.990869	-1.725332
F	1.415222	1.732126	-0.679280
F	2.756013	0.285264	0.237606
F	2.640611	2.310151	1.024066

References

(1) Still, W. C.; Kahn, M.; Mitra, A., Rapid Chromatographic Technique for Preparative Separations with Moderate Resolution. *J. Org. Chem.* **1978,** *43*, 2923-2925.
(2) Fulmer, G. R.; Miller, A. J. M.; Sherden, N. H.; Gottlieb, H. E.; Nudelman, A.; Stoltz, B. M.; Bercaw, J. E.; Goldberg, K. I., Nmr Chemical Shifts of Trace Impurities: Common Laboratory Solvents, Organics, and Gases in Deuterated Solvents Relevant to the Organometallic Chemist. *Organometallics* **2010,** *29*, 2176-2179.
(3) Zhao, G.; Lim, S.; Musaev, D. G.; Ngai, M.-Y., Expanding Reaction Profile of Allyl Carboxylates Via 1, 2-Radical Migration (Ram): Visible-Light-Induced Phosphine-Catalyzed 1, 3-Carbobromination of Allyl Carboxylates. *J. Am. Chem. Soc.* **2023,** *145*, 8275-8284.
(4) Zhao, G.; Khosravi, A.; Sharma, S.; Musaev, D. G.; Ngai, M.-Y., Cobalt-Hydride-Catalyzed Alkene-Carboxylate Transposition (Act) of Allyl Carboxylates. *J. Am. Chem. Soc.* **2024,** *146*, 31391-31399.
(5) Cismesia, M. A.; Yoon, T. P., Characterizing Chain Processes in Visible Light Photoredox Catalysis. *Chem. Sci.* **2015,** *6*, 5426-5434.
(6) Frisch, M. J.; Trucks, G. W.; Schlegel, H. B.; Scuseria, G. E.; Robb, M. A.; Cheeseman, J. R.; Scalmani, G.; Barone, V.; Petersson, G. A.; Nakatsuji, H.; Li, X.; Caricato, M.; Marenich, A. V.; Bloino, J.; Janesko, B. G.; Gomperts, R.; Mennucci, B.; Hratchian, H. P.; Ortiz, J. V.; Izmaylov, A. F.; Sonnenberg, J. L.; Williams; Ding, F.; Lipparini, F.; Egidi, F.; Goings, J.; Peng, B.; Petrone, A.; Henderson, T.; Ranasinghe, D.; Zakrzewski, V. G.; Gao, J.; Rega, N.; Zheng, G.; Liang, W.; Hada, M.; Ehara, M.; Toyota, K.; Fukuda, R.; Hasegawa, J.; Ishida, M.; Nakajima, T.; Honda, Y.; Kitao, O.; Nakai, H.; Vreven, T.; Throssell, K.; Montgomery Jr., J. A.; Peralta, J. E.; Ogliaro, F.; Bearpark, M. J.; Heyd, J. J.; Brothers, E. N.; Kudin, K. N.; Staroverov, V. N.; Keith, T. A.; Kobayashi, R.; Normand, J.; Raghavachari, K.; Rendell, A. P.; Burant, J. C.; Iyengar, S. S.; Tomasi, J.; Cossi, M.; Millam, J. M.; Klene, M.; Adamo, C.; Cammi, R.; Ochterski, J. W.; Martin, R. L.; Morokuma, K.; Farkas, O.; Foresman, J. B.; Fox, D. J. *Gaussian 16 Rev. C.01*, Wallingford, CT, 2016.
(7) (a) Lee, C.; Yang, W.; Parr, R. G., Development of the Colle-Salvetti Correlation-Energy Formula into a Functional of the Electron Density. *Phy. Rev. B* **1988,** *37*, 785; (b) Becke, A., Thermo Chemistry Density-Functional Iii. The Role of Exact Exchange. *J. Chem. Phys* **1993,** *98*, 5648-5652.
(8) Grimme, S.; Ehrlich, S.; Goerigk, L., Effect of the Damping Function in Dispersion Corrected Density Functional Theory. *J. Comput. Chem.* **2011,** *32*, 1456-1465.
(9) Roy, L. E.; Hay, P. J.; Martin, R. L., Revised Basis Sets for the Lanl Effective Core Potentials. *J. Chem. Theory Comput.* **2008,** *4*, 1029-1031.
(10) Zhao, Y.; Truhlar, D. G., The M06 Suite of Density Functionals for Main Group Thermochemistry, Thermochemical Kinetics, Noncovalent Interactions, Excited States, and Transition Elements: Two New Functionals and Systematic Testing of Four M06-Class Functionals and 12 Other Functionals. *Theor. Chem. Acc.* **2008,** *120*, 215-241.
(11) (a) Weigend, F., Accurate Coulomb-Fitting Basis Sets for H to Rn. *Phys. Chem. Chem. Phys.* **2006,** *8*, 1057-1065; (b) Weigend, F.; Ahlrichs, R., Balanced Basis Sets of Split Valence, Triple Zeta Valence and Quadruple Zeta Valence Quality for H to Rn: Design and Assessment of Accuracy. *Phys. Chem. Chem. Phys.* **2005,** *7*, 3297-3305.

(12) Marenich, A. V.; Cramer, C. J.; Truhlar, D. G., Universal Solvation Model Based on Solute Electron Density and on a Continuum Model of the Solvent Defined by the Bulk Dielectric Constant and Atomic Surface Tensions. *J. Phys. Chem. B* **2009,** *113*, 6378-6396.
(13) Legault, C., Cylview, 1.0 B. Université de Sherbrooke Canada: 2009.
(14) Khosravi, A.; Zhang, Y.; Zhao, G.; Radefeld, K. J.; Sharma, S.; Pannilawithana, N. A.; Zhang, Y.; Liu, P.; Ngai, M.-Y., Remote-Markovnikov Hydrobromination and Hydrochlorination of Allyl Carboxylates Via Dual Photoredox/Cobalt Catalysis. *J. Am. Chem. Soc.* **2025,** *147*, 27197-27206.
